# Correction: Degradation of LMO2 in T cell leukaemia results in collateral breakdown of transcription complex partners and causes LMO2-dependent apoptosis

**DOI:** 10.7554/eLife.113186

**Published:** 2026-09-21

**Authors:** Naphannop Sereesongsaeng, Carole Bataille, Angela Russell, Nicolas Bery, Fernando J Sialana, Jyoti Choudhary, Ami Miller, Terence Rabbitts

**Keywords:** Mouse

Sereesongsaeng N, Bataille C, Russell A, Bery N, Sialana FJ, Choudhary J, Miller A, Rabbitts T. 2025. Degradation of LMO2 in T cell leukaemia results in collateral breakdown of transcription complex partners and causes LMO2-dependent apoptosis. *eLife* **14**:RP106699. doi: 10.7554/eLife.106699.

Published 12 December 2025

We were notified by eLife journal and PubPeer of the similar western blotting result in the different cell line in Figure 3.

In the original Figure 3A, the GATA3 western blot result for CCRF-CEM cells treated with Abd-VHL appears very similar to the GATA3 western blot result for KOPT-K1 cells treated with Abd-VHL shown in Figure 3C. Following a thorough review of the original raw data, we identified an error in the submitted figure, that occurred during figure assembly, which affected the CCRF-CEM GATA3 data in panel Figure 3A. Because GATA3 protein levels were not altered by either Abd-CRBN or Abd-VHL treatment in any of the cell lines examined, many of the GATA3 western blot bands displayed very similar signal intensities and patterns. This similarity increased the complexity of the figure assembly process and contributed to the inadvertent placement of two incorrect GATA3 images in Figure 3. In the original Figure 3A

1. The western blot for CCRF-CEM cells treated with Abd-VHL was incorrectly positioned in the Abd-CRBN treated panel and has now been placed in the correct location.
2. The western blot for CCRF-CEM cells treated with Abd-CRBN has now been replaced with the correct western blot data.

These are now corrected in Figure 3A.

Importantly, this does not affect the underlying interpretation of the results, as GATA3 levels remained unchanged across all cell lines and treatment conditions analysed. The GATA3 western blot result for KOPT-K1 cells shown in Figure 3C was verified against the original raw data and remains correct and unchanged.

The corrected Figure 3 (updated for panel A) is shown here:

**Figure 1:**
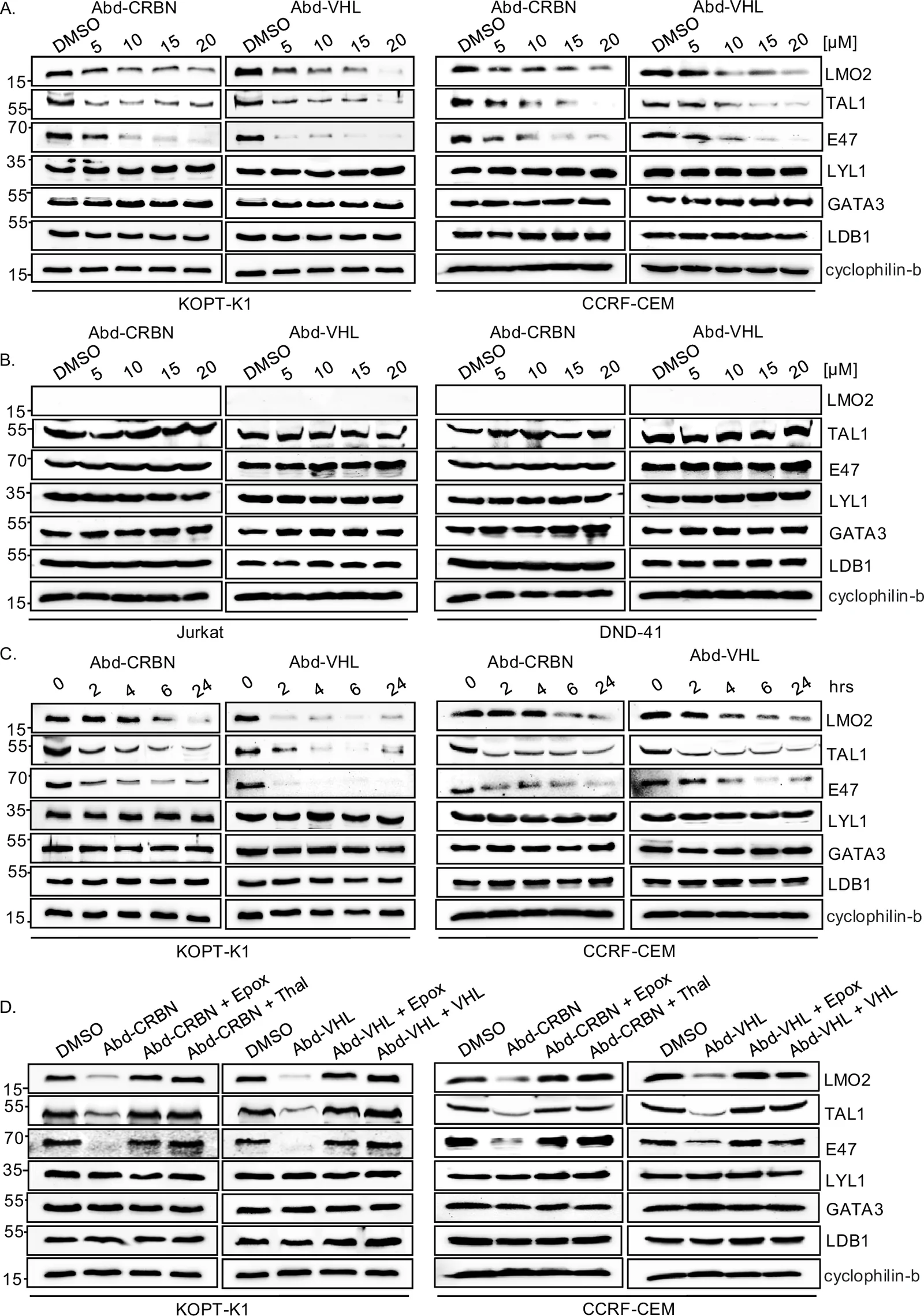


The originally published Figure 3 is shown for reference:

**Figure 2:**
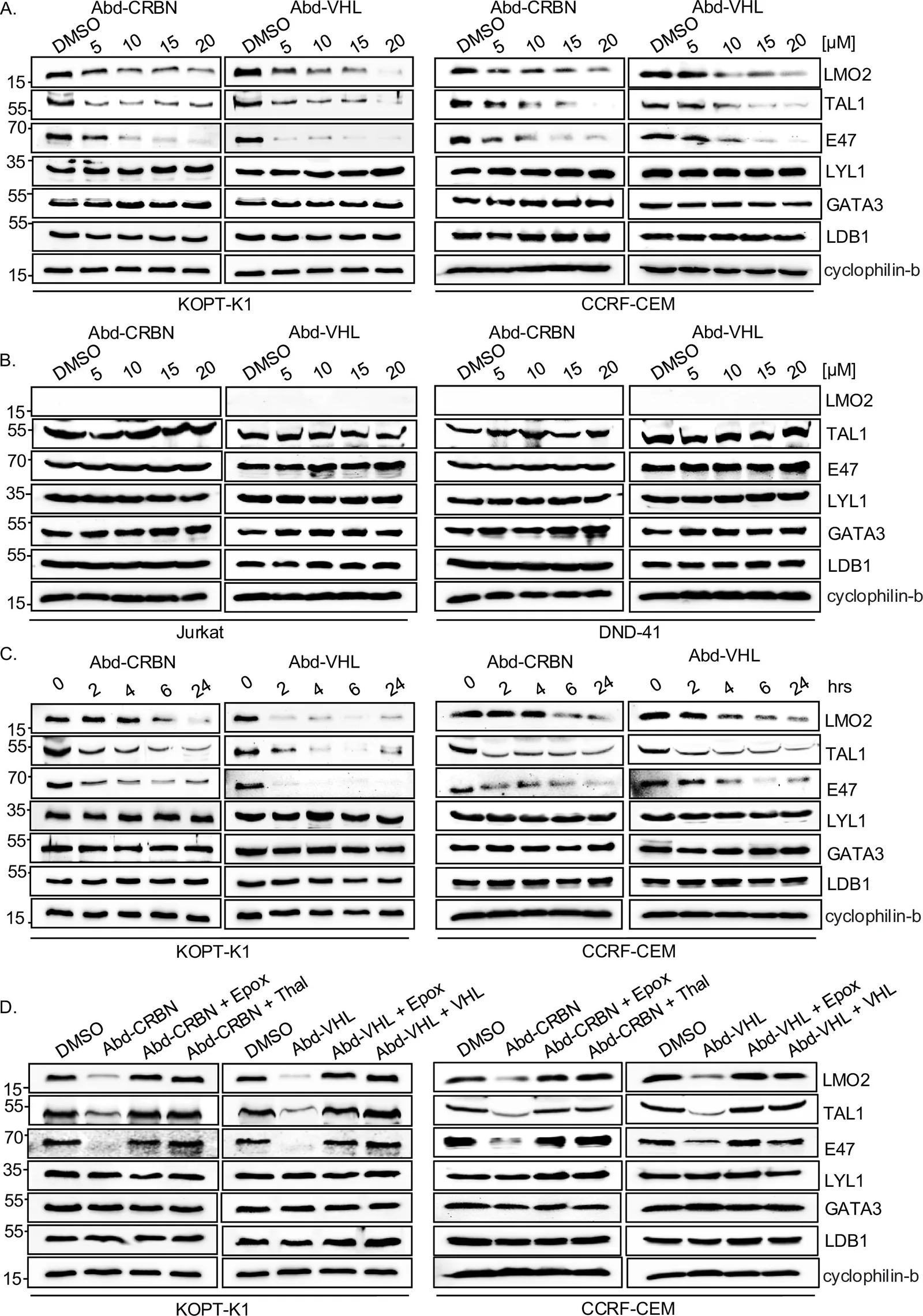


The published Source data for the gels associated with Figure 3 have also been corrected.

The article has been corrected accordingly.

