# Supplementary figures and images for: Degradation of LMO2 in T cell leukaemia results in collateral breakdown of transcription complex partners and causes LMO2-dependent apoptosis (part 1 of 5)
Source: eLife. 2025 Dec 12;14:RP106699. doi: 10.7554/eLife.106699 (PMC12700530; doi:10.7554/eLife.106699)

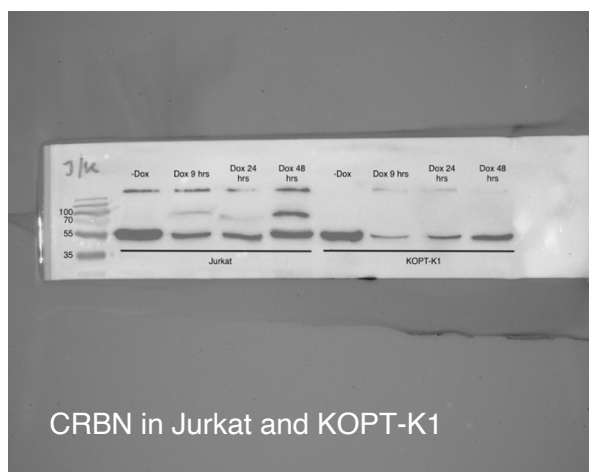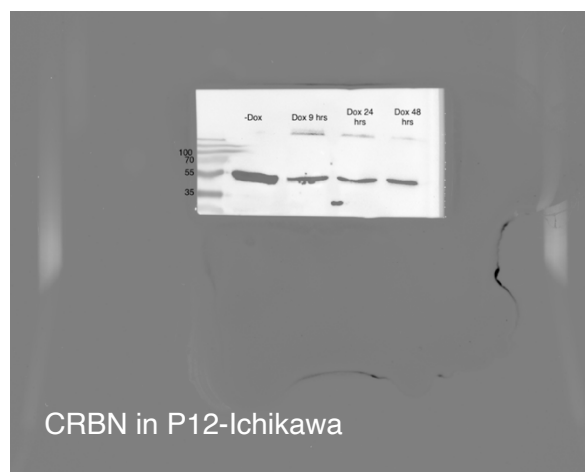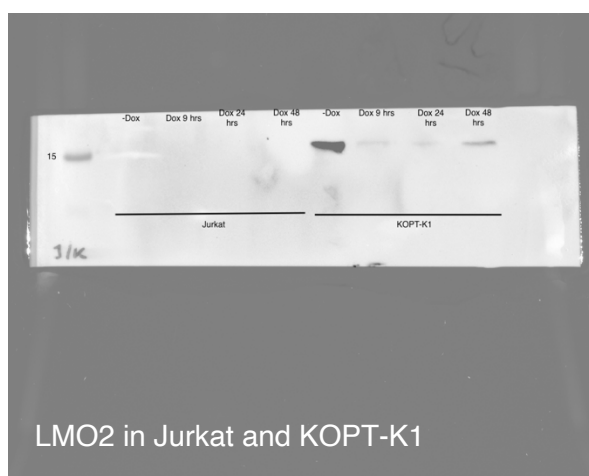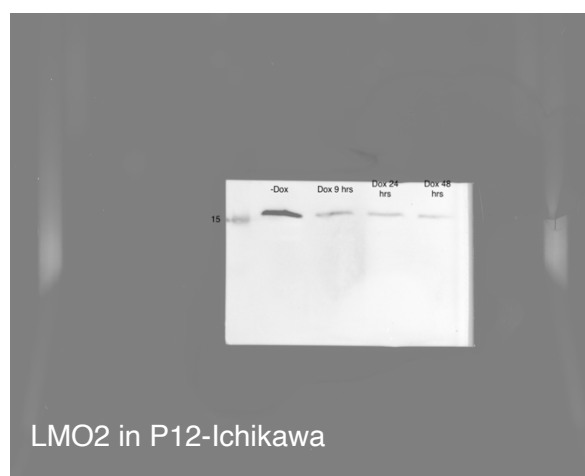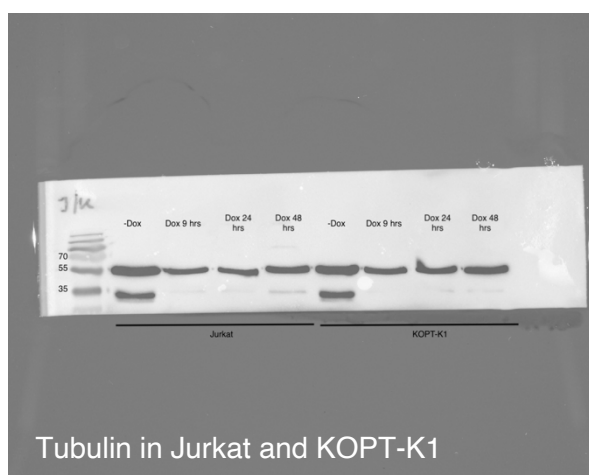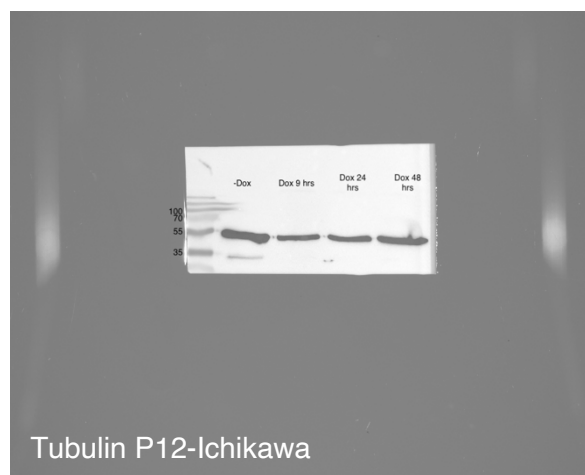

**Figure 1, Source Data 1.** Original membranes corresponding to Figure 1, panel A.

Supplement: Figure 1—source data 1. [file elife-106699-fig1-data1.zip › Figure 1ΓÇösource data 1 PDF files containing original western blots for Figure 1A, indicating the relevant bands and treatments./Figure 1ΓÇösource data 1 .pdf]

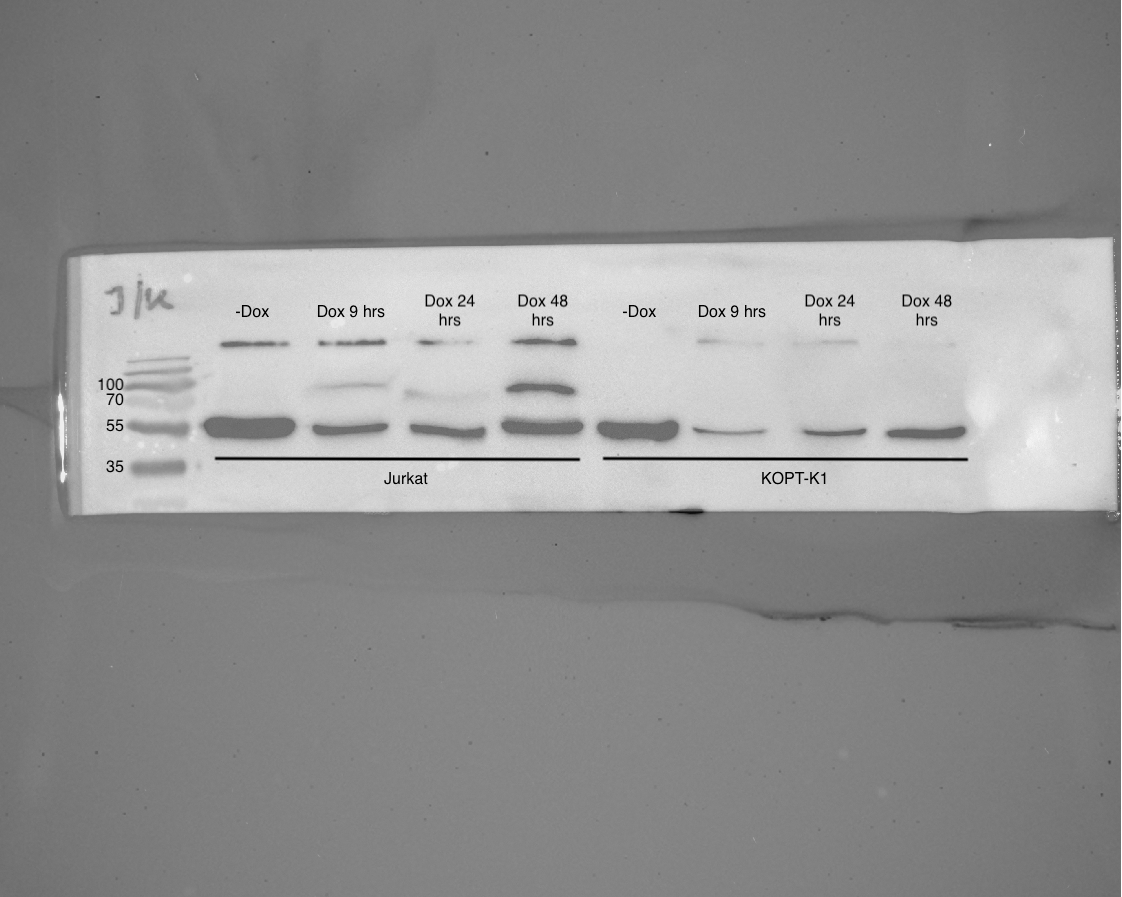

Supplement: Figure 1—source data 1. [file elife-106699-fig1-data1.zip › Figure 1ΓÇösource data 1 PDF files containing original western blots for Figure 1A, indicating the relevant bands and treatments./Raw data/CRBN Jurkat and KOPT-K1.tif]

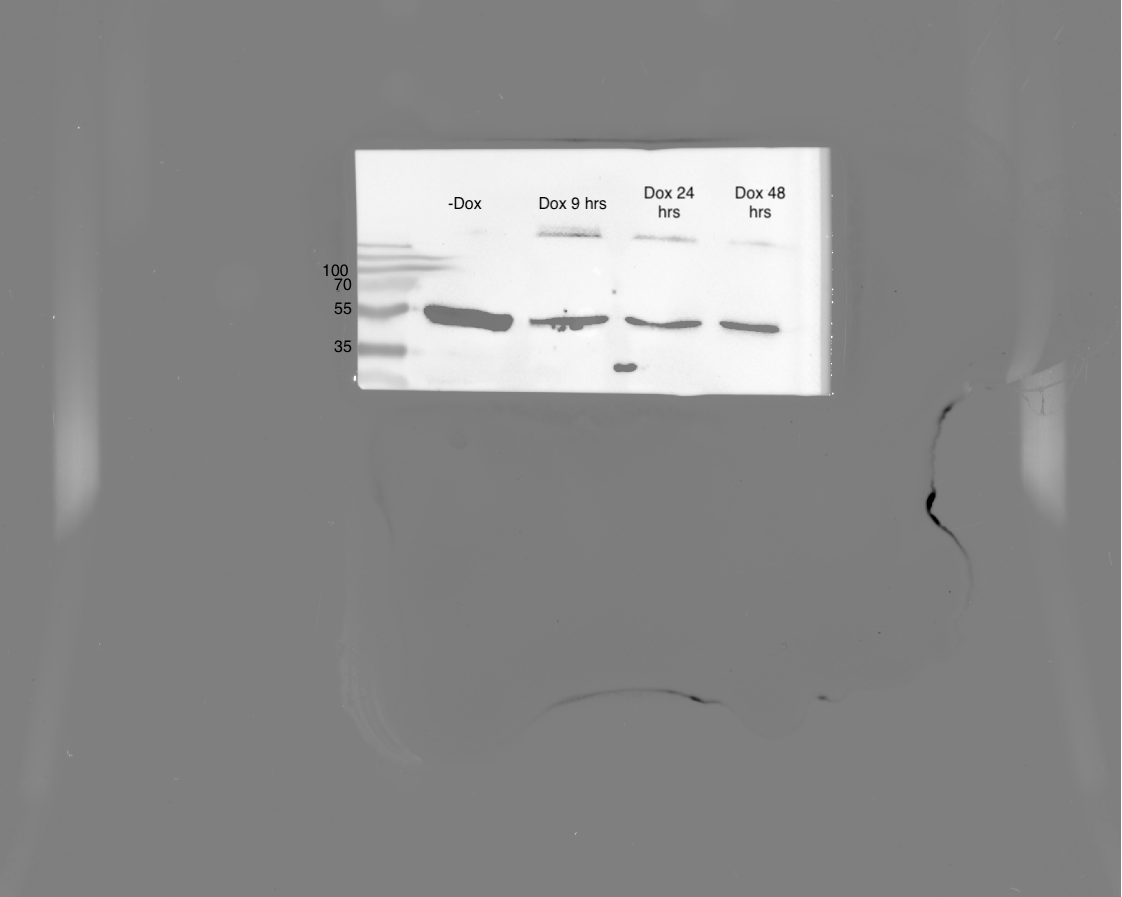

Supplement: Figure 1—source data 1. [file elife-106699-fig1-data1.zip › Figure 1ΓÇösource data 1 PDF files containing original western blots for Figure 1A, indicating the relevant bands and treatments./Raw data/CRBN P12-Ichikawa.tif]

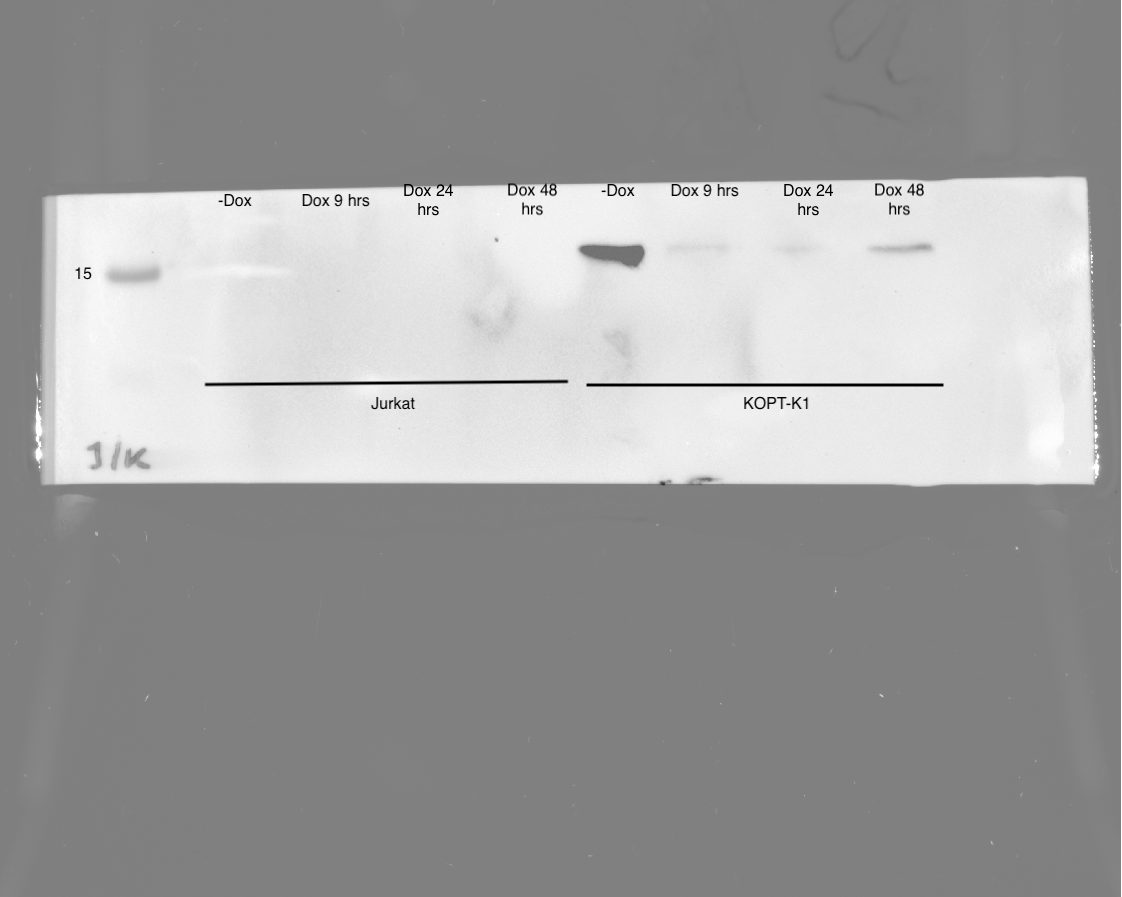

Supplement: Figure 1—source data 1. [file elife-106699-fig1-data1.zip › Figure 1ΓÇösource data 1 PDF files containing original western blots for Figure 1A, indicating the relevant bands and treatments./Raw data/LMO2 KOPT-K1 and Jurkat.tif]

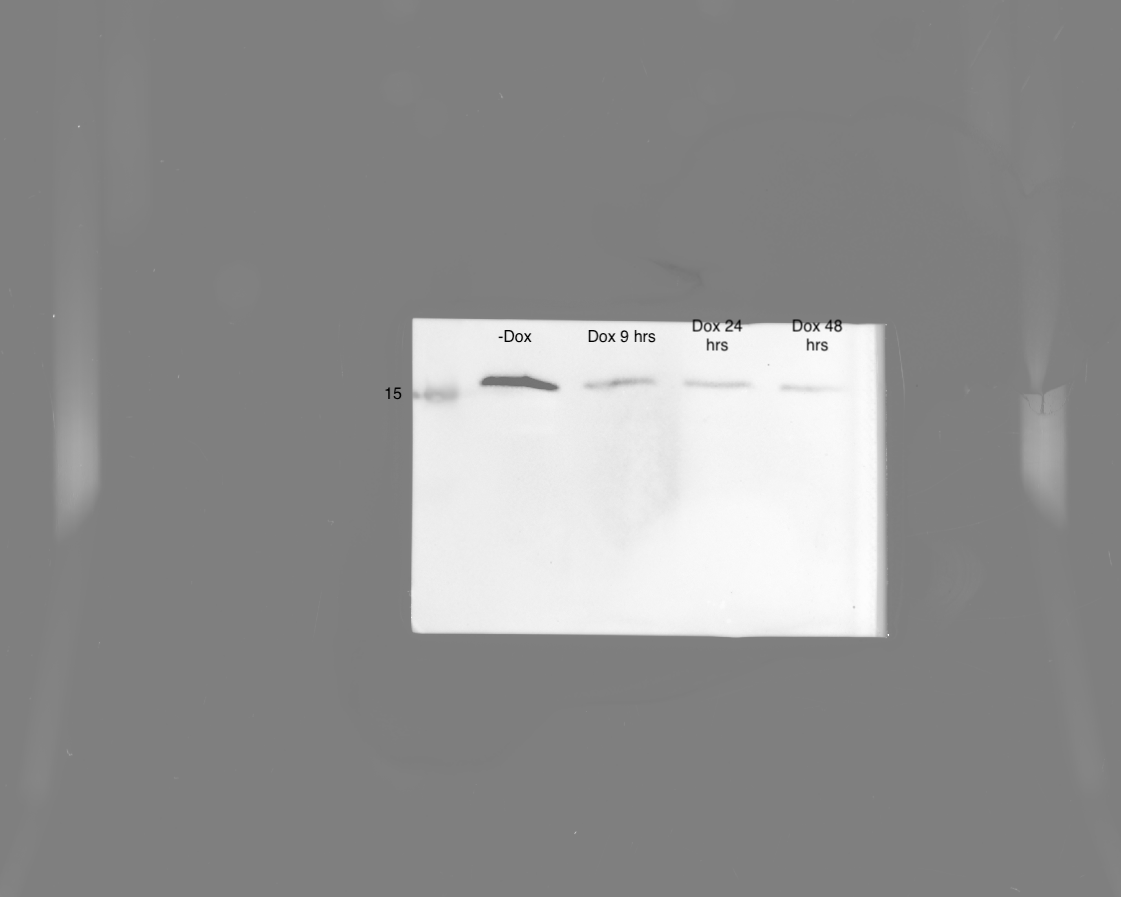

Supplement: Figure 1—source data 1. [file elife-106699-fig1-data1.zip › Figure 1ΓÇösource data 1 PDF files containing original western blots for Figure 1A, indicating the relevant bands and treatments./Raw data/LMO2 P12-Ichikawa.tif]

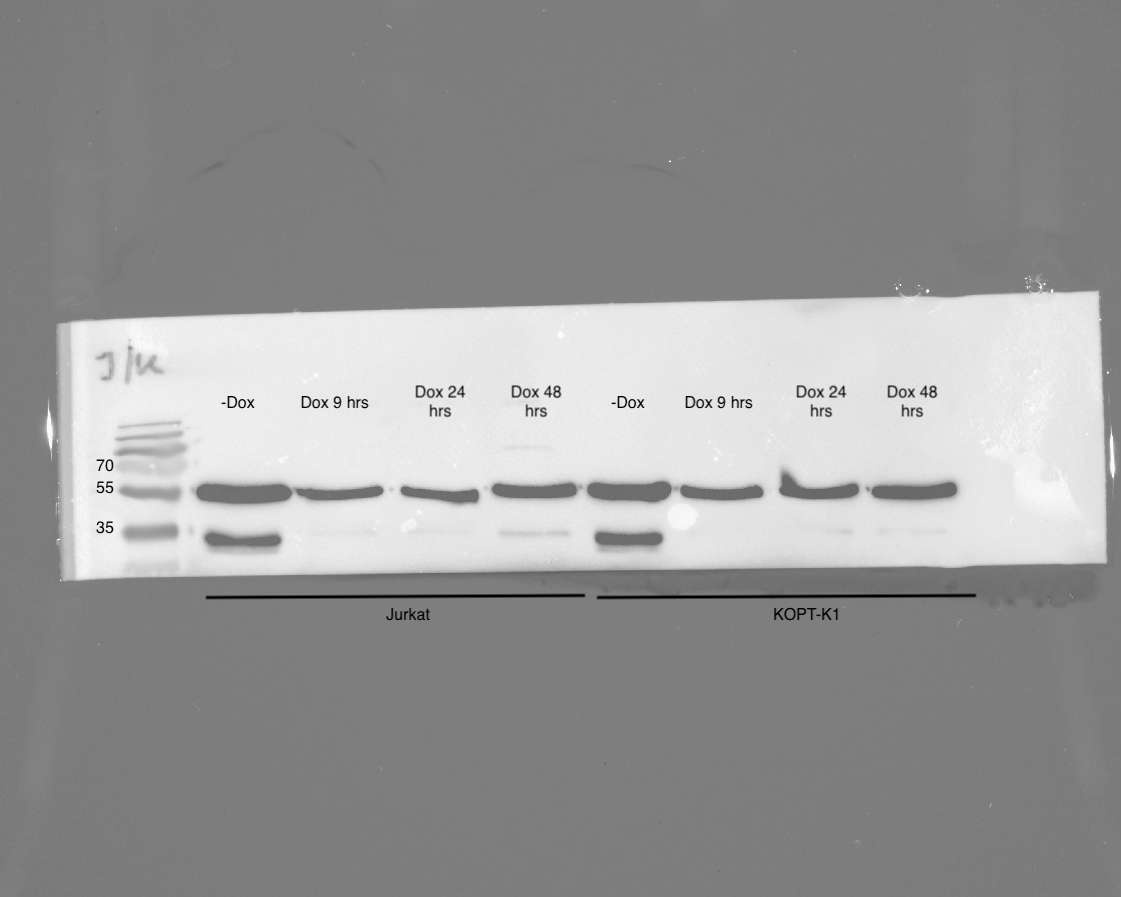

Supplement: Figure 1—source data 1. [file elife-106699-fig1-data1.zip › Figure 1ΓÇösource data 1 PDF files containing original western blots for Figure 1A, indicating the relevant bands and treatments./Raw data/Tubulin Jurkat and KOPT-K1.tif]

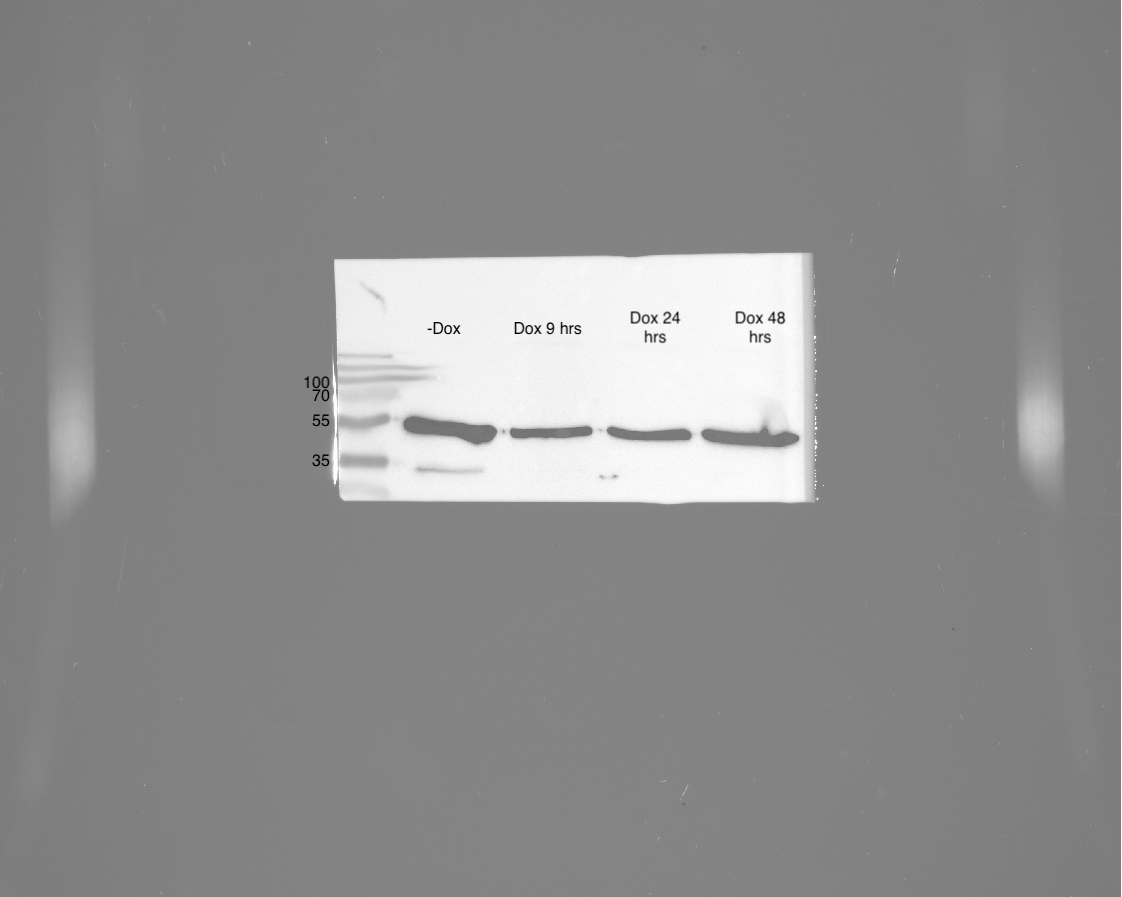

Supplement: Figure 1—source data 1. [file elife-106699-fig1-data1.zip › Figure 1ΓÇösource data 1 PDF files containing original western blots for Figure 1A, indicating the relevant bands and treatments./Raw data/Tubulin P12-Ichikawa.tif]

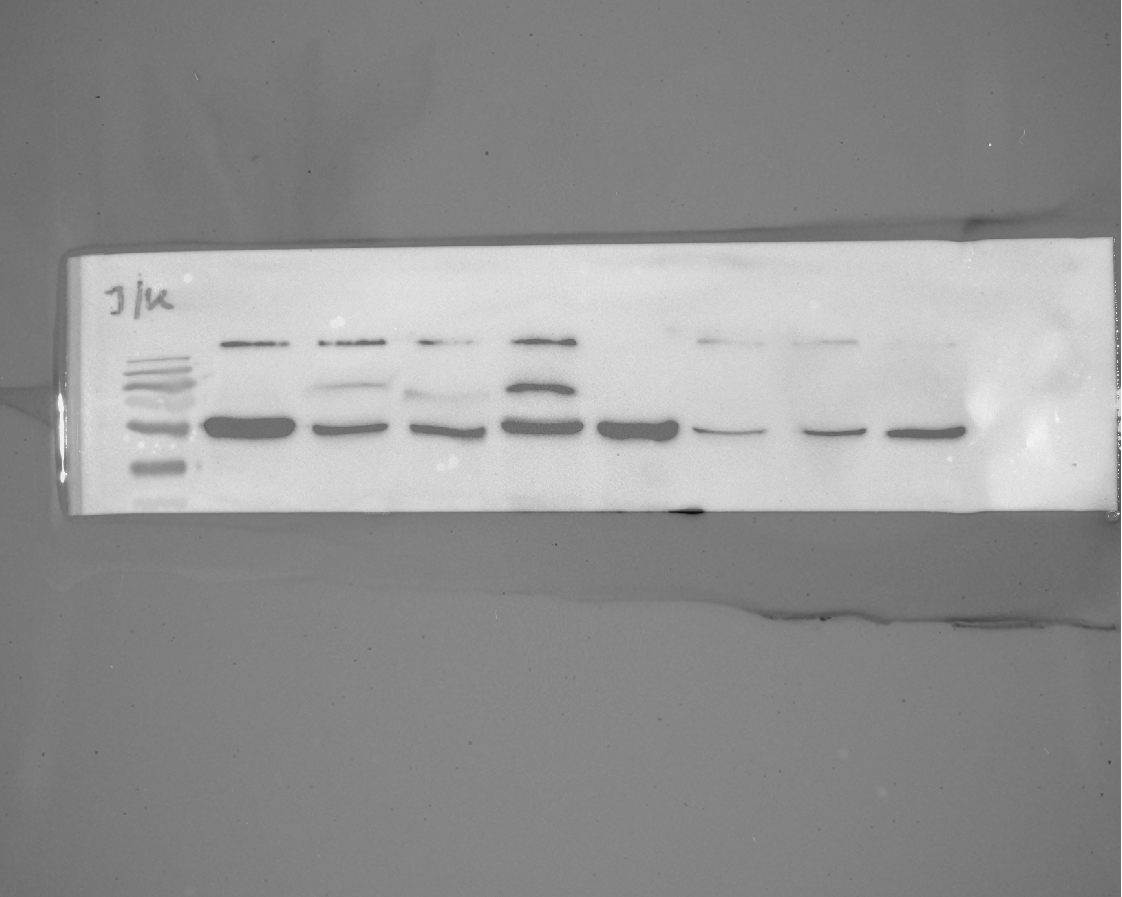

Supplement: Figure 1—source data 2. [file elife-106699-fig1-data2.zip › Figure 1ΓÇösource data 2 Original files for Western blot analysis displayed in Figure 1A./CRBN Jurkat and KOPT-K1.tif]

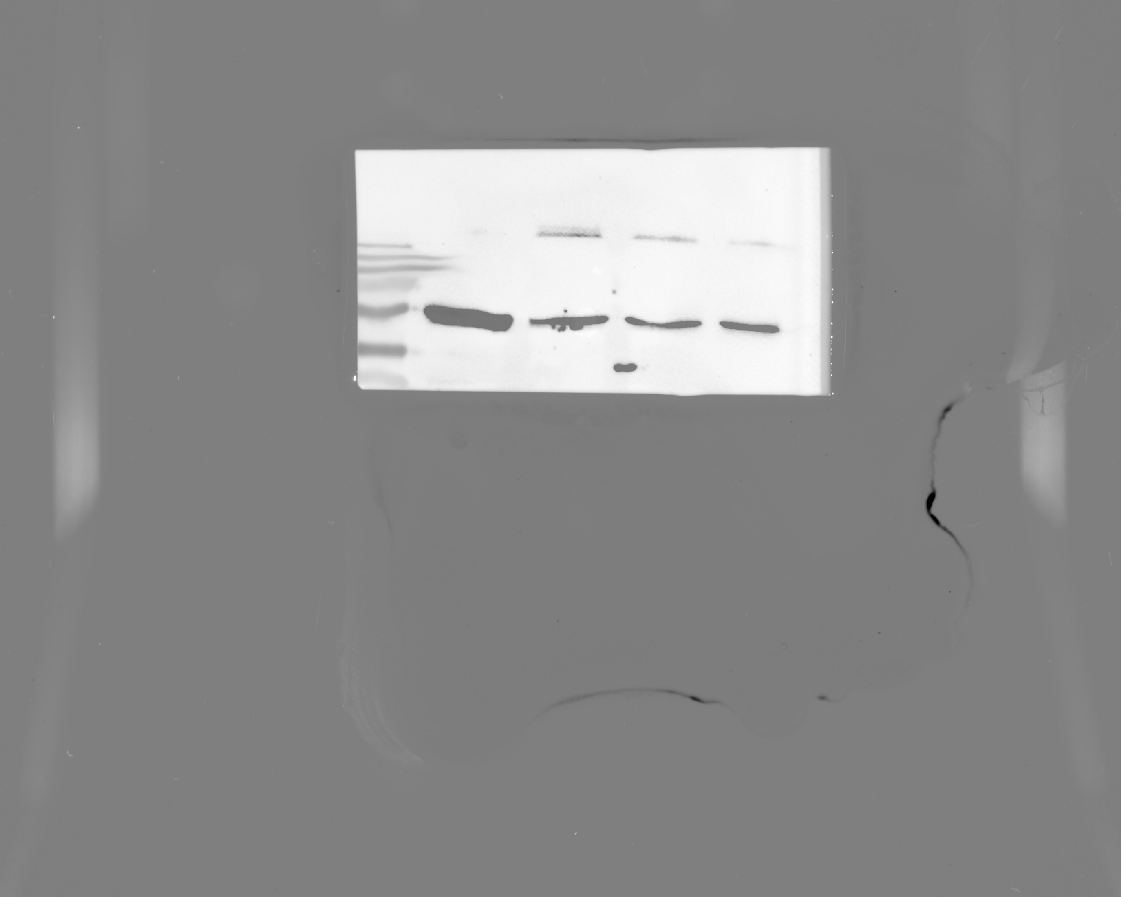

Supplement: Figure 1—source data 2. [file elife-106699-fig1-data2.zip › Figure 1ΓÇösource data 2 Original files for Western blot analysis displayed in Figure 1A./CRBN P12-Ichikawa.tif]

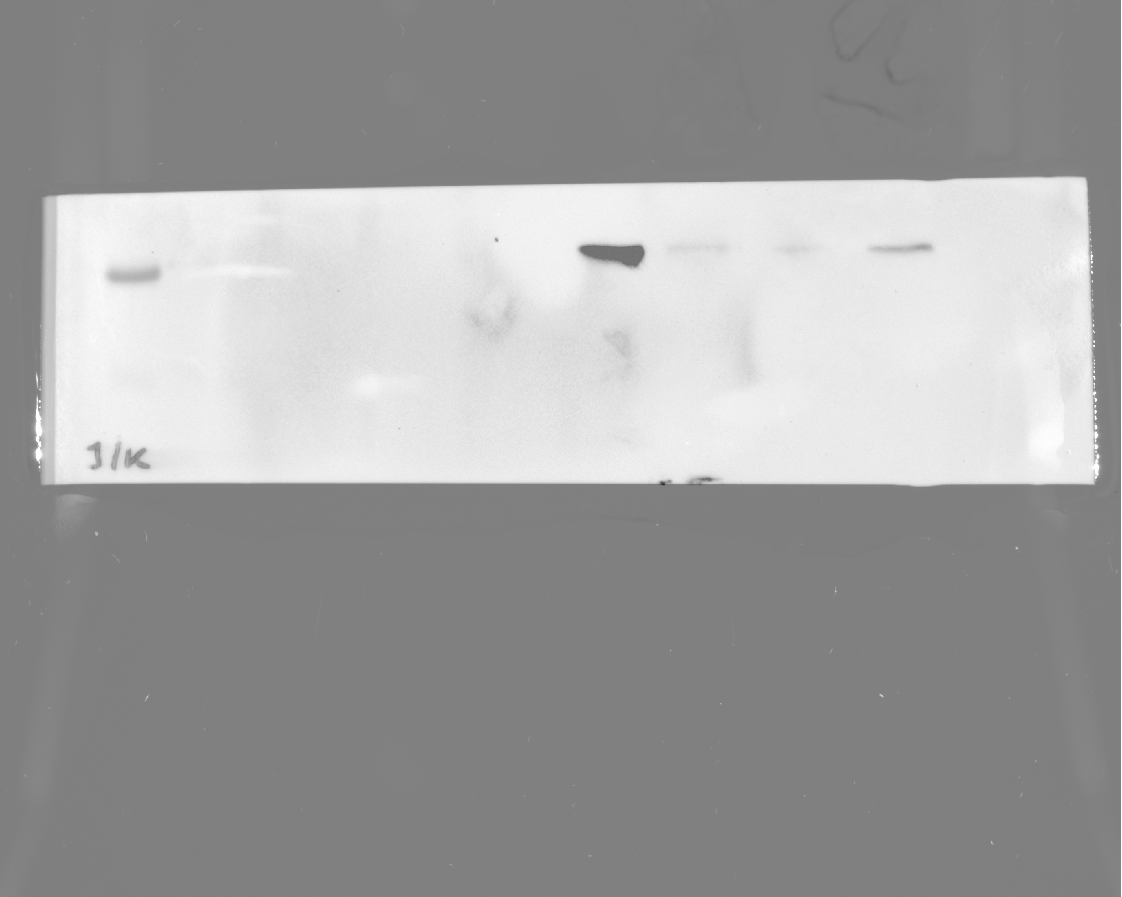

Supplement: Figure 1—source data 2. [file elife-106699-fig1-data2.zip › Figure 1ΓÇösource data 2 Original files for Western blot analysis displayed in Figure 1A./LMO2 KOPT-K1 and Jurkat.tif]

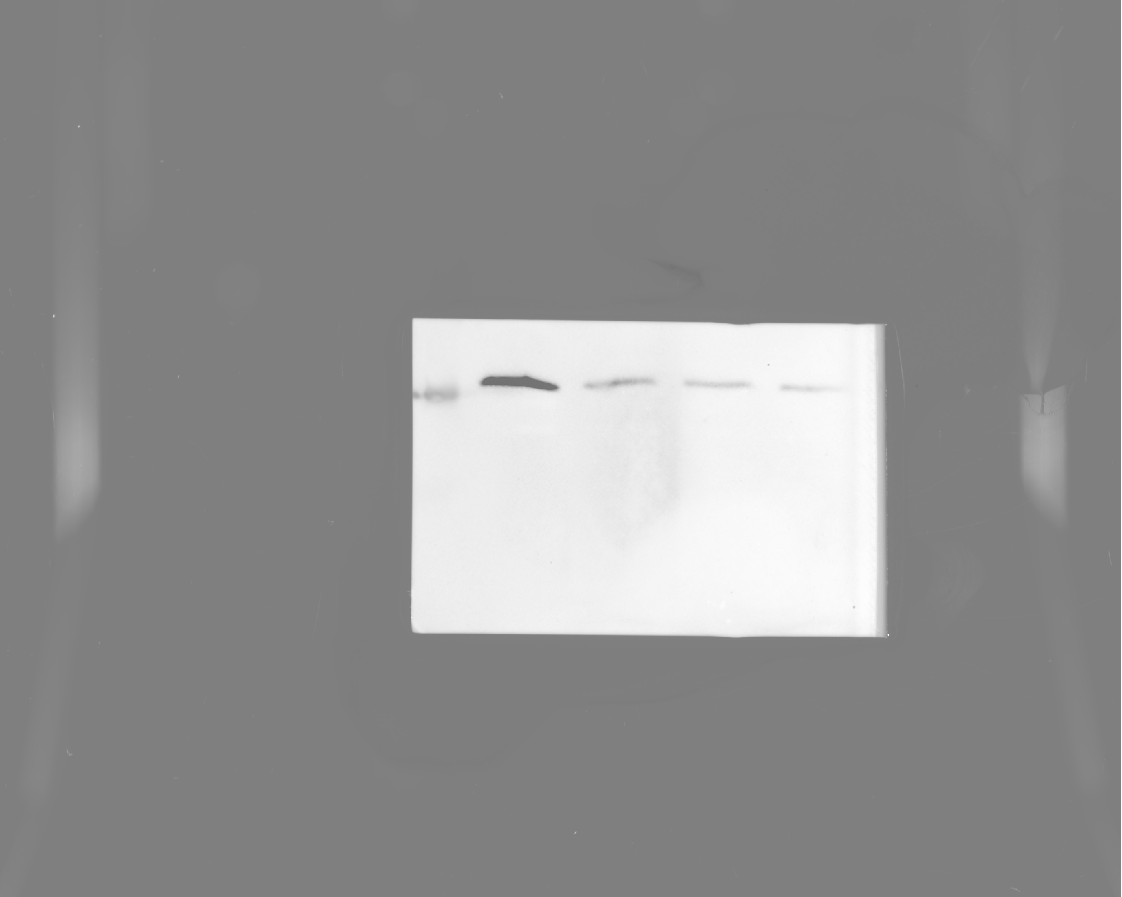

Supplement: Figure 1—source data 2. [file elife-106699-fig1-data2.zip › Figure 1ΓÇösource data 2 Original files for Western blot analysis displayed in Figure 1A./LMO2 P12-Ichikawa.tif]

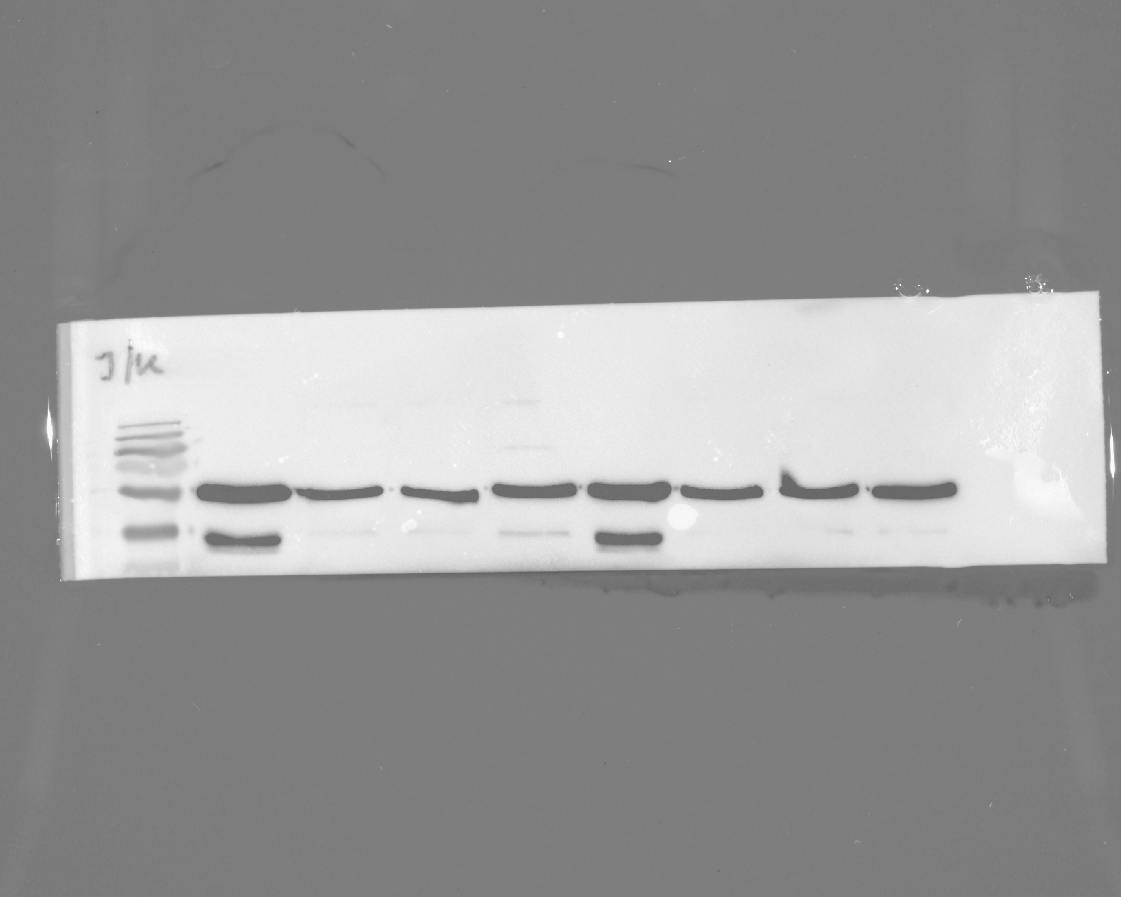

Supplement: Figure 1—source data 2. [file elife-106699-fig1-data2.zip › Figure 1ΓÇösource data 2 Original files for Western blot analysis displayed in Figure 1A./Tubulin Jurkat and KOPT-K1.tif]

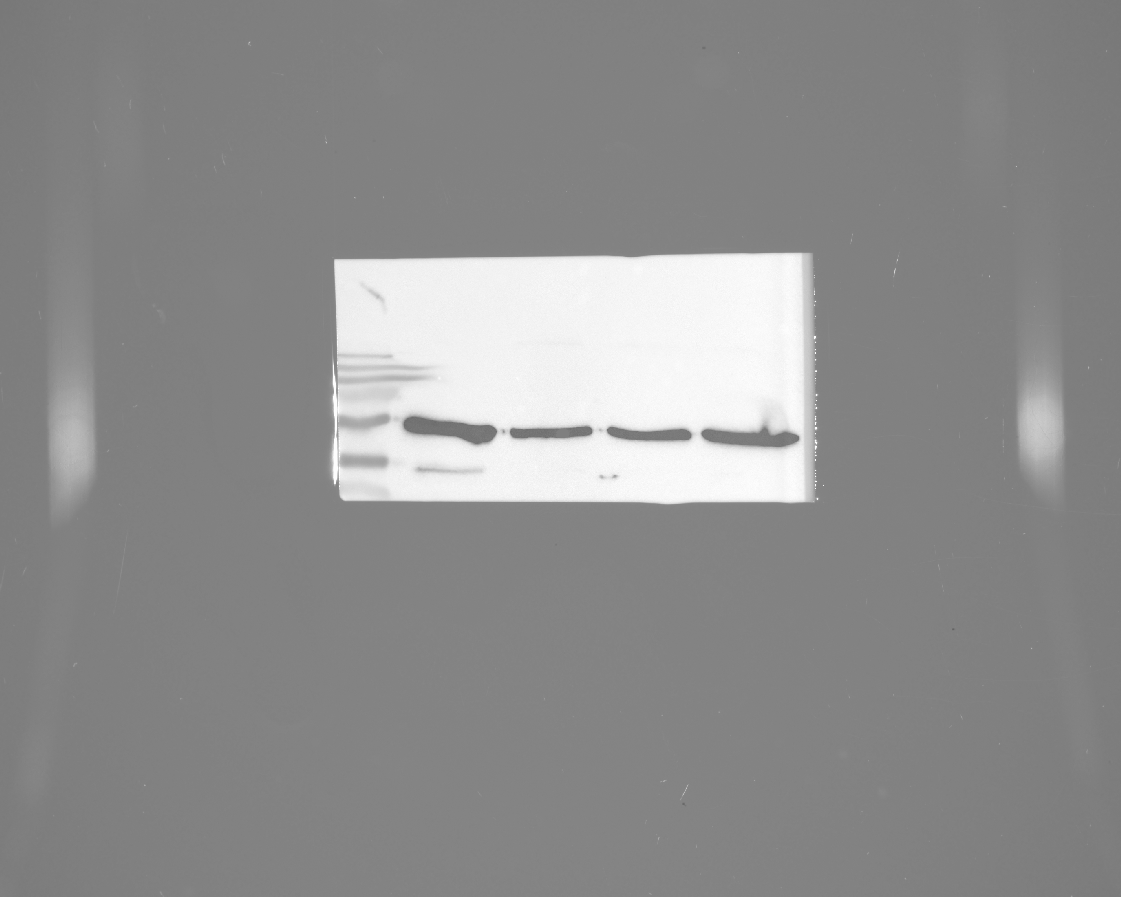

Supplement: Figure 1—source data 2. [file elife-106699-fig1-data2.zip › Figure 1ΓÇösource data 2 Original files for Western blot analysis displayed in Figure 1A./Tubulin P12-Ichikawa.tif]

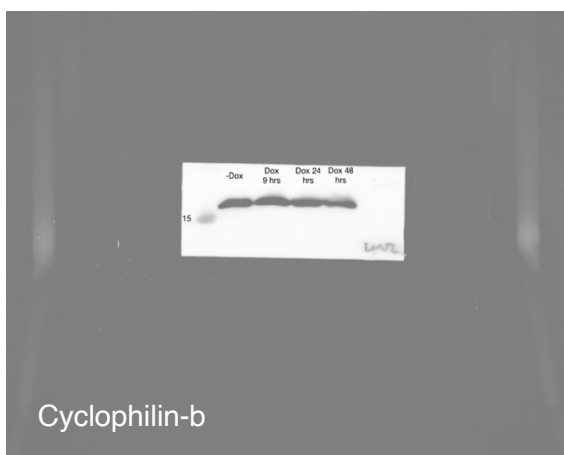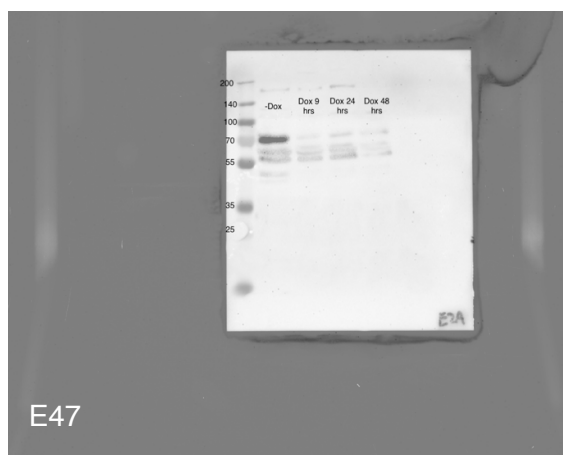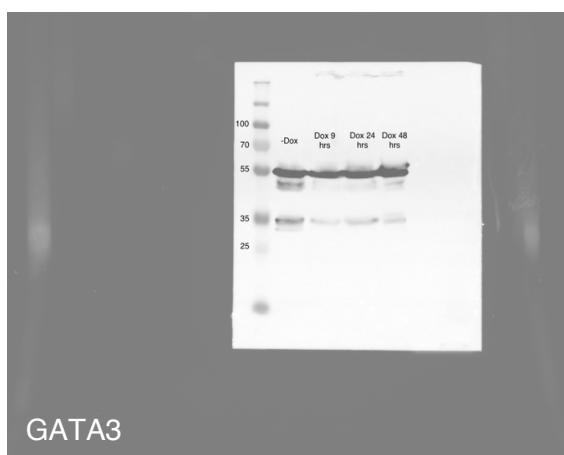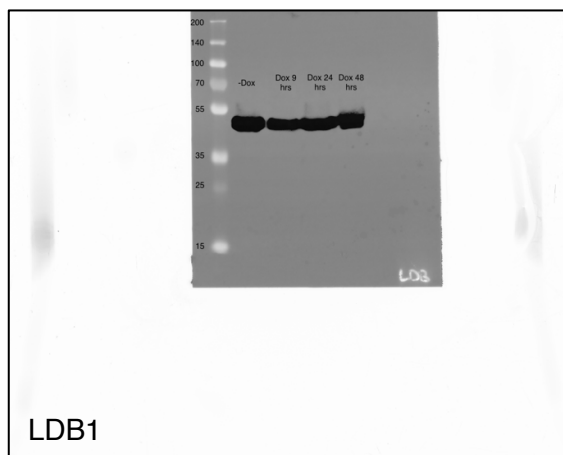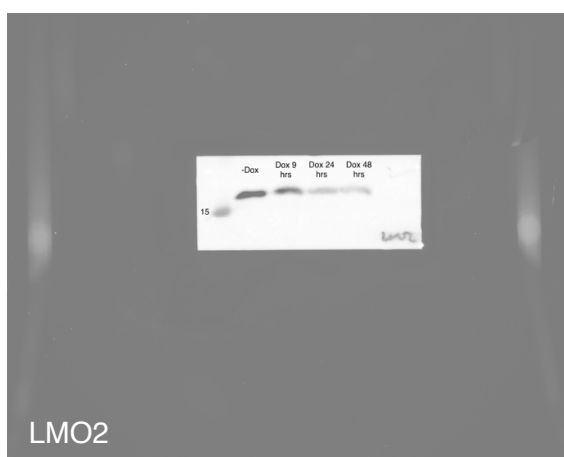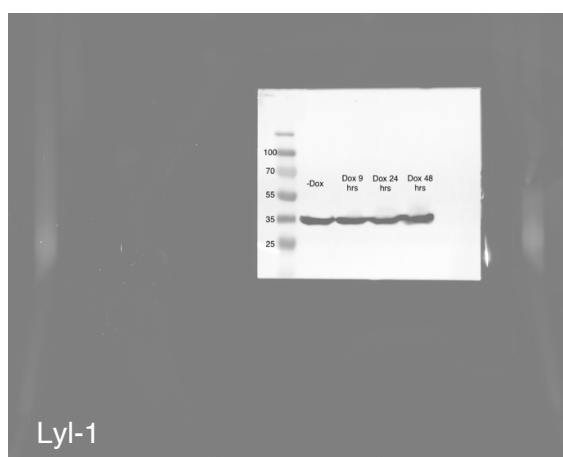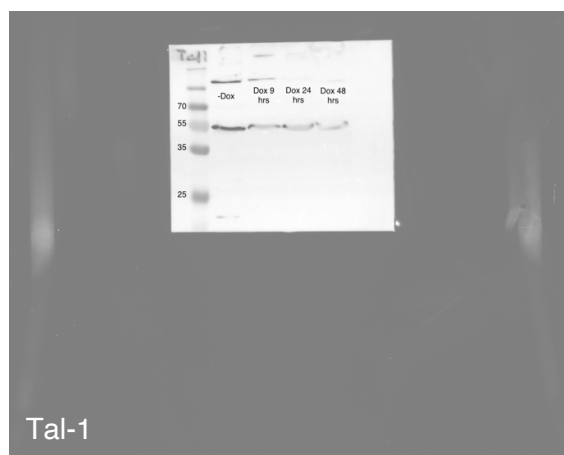

**Figure 1, Source Data 3.** Original membranes corresponding to Figure 1, panel B.

Supplement: Figure 1—source data 3. [file elife-106699-fig1-data3.zip › Figure 1ΓÇösource data 3 PDF files containing original western blots for Figure 1B, indicating the relevant bands and treatments./Figure 1ΓÇösource data 3.pdf]

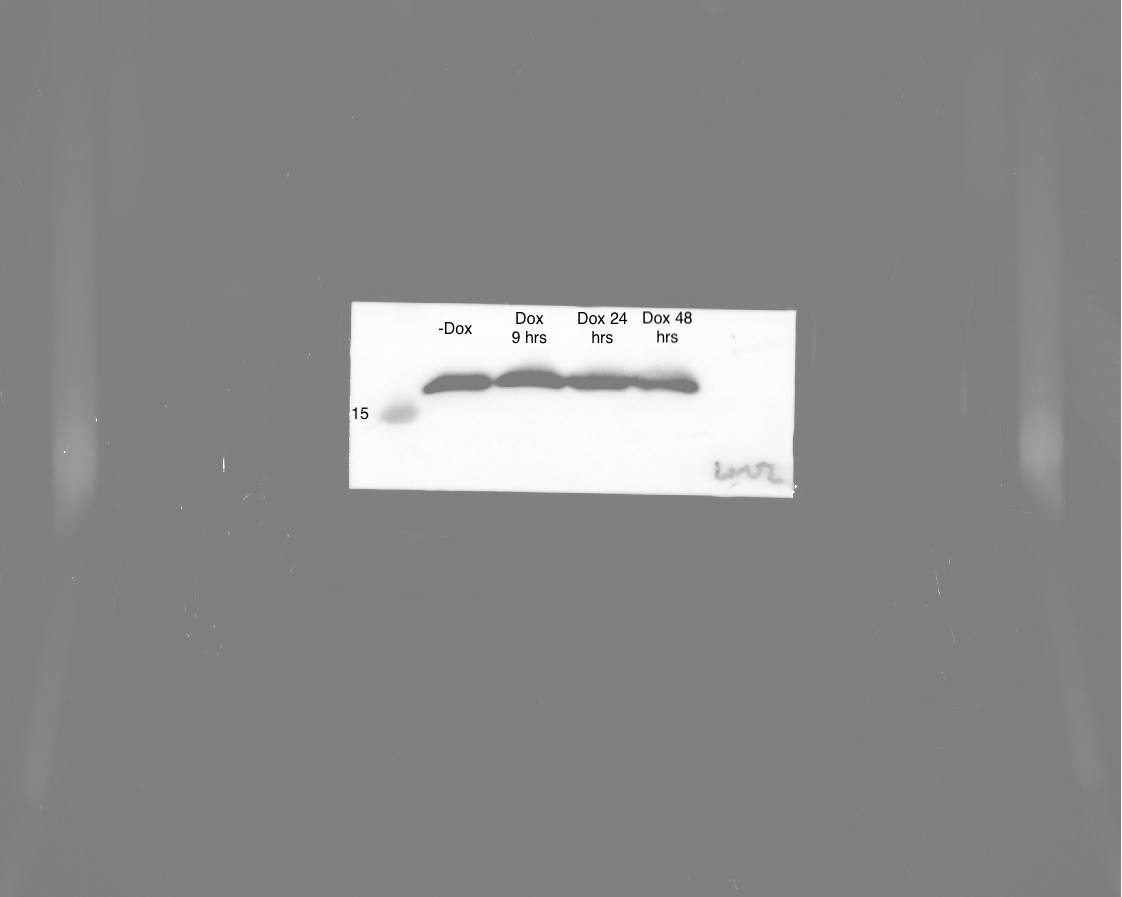

Supplement: Figure 1—source data 3. [file elife-106699-fig1-data3.zip › Figure 1ΓÇösource data 3 PDF files containing original western blots for Figure 1B, indicating the relevant bands and treatments./Raw data/Cyclophilin.tif]

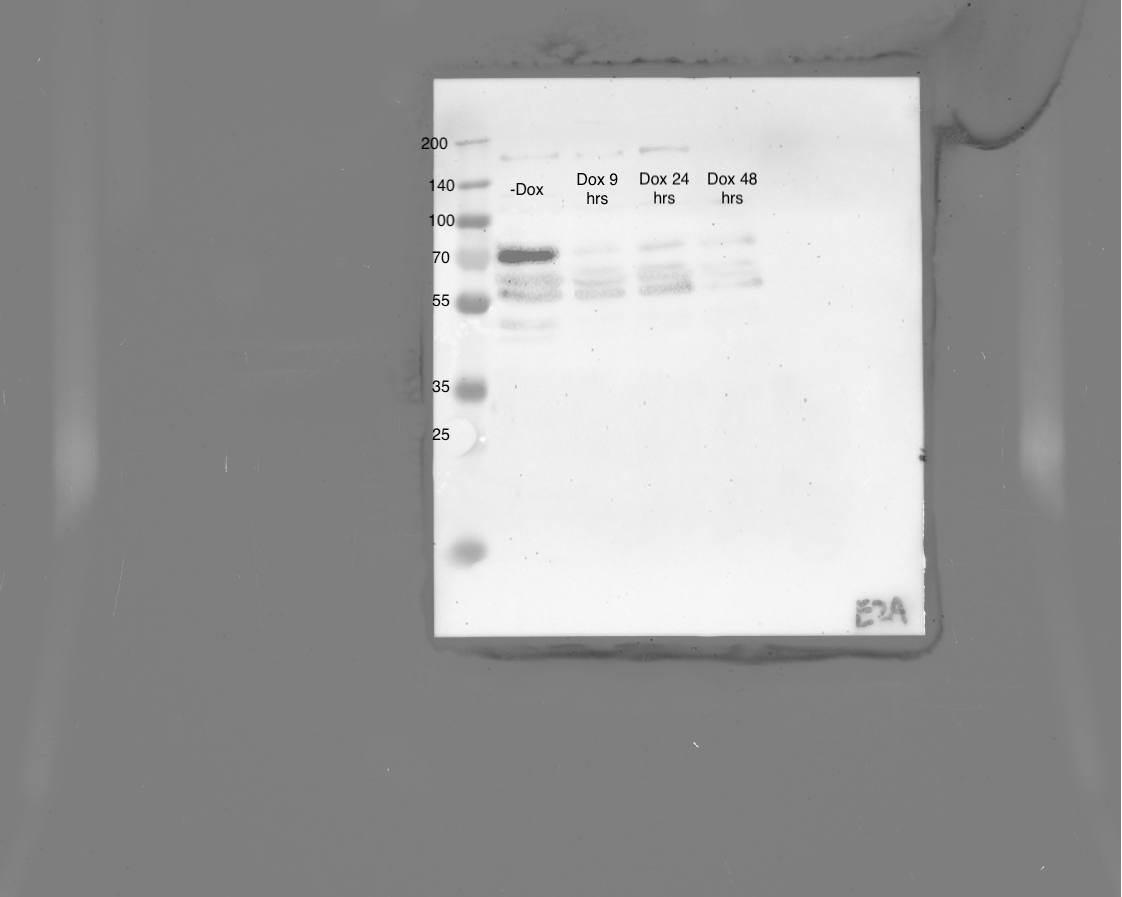

Supplement: Figure 1—source data 3. [file elife-106699-fig1-data3.zip › Figure 1ΓÇösource data 3 PDF files containing original western blots for Figure 1B, indicating the relevant bands and treatments./Raw data/E47.tif]

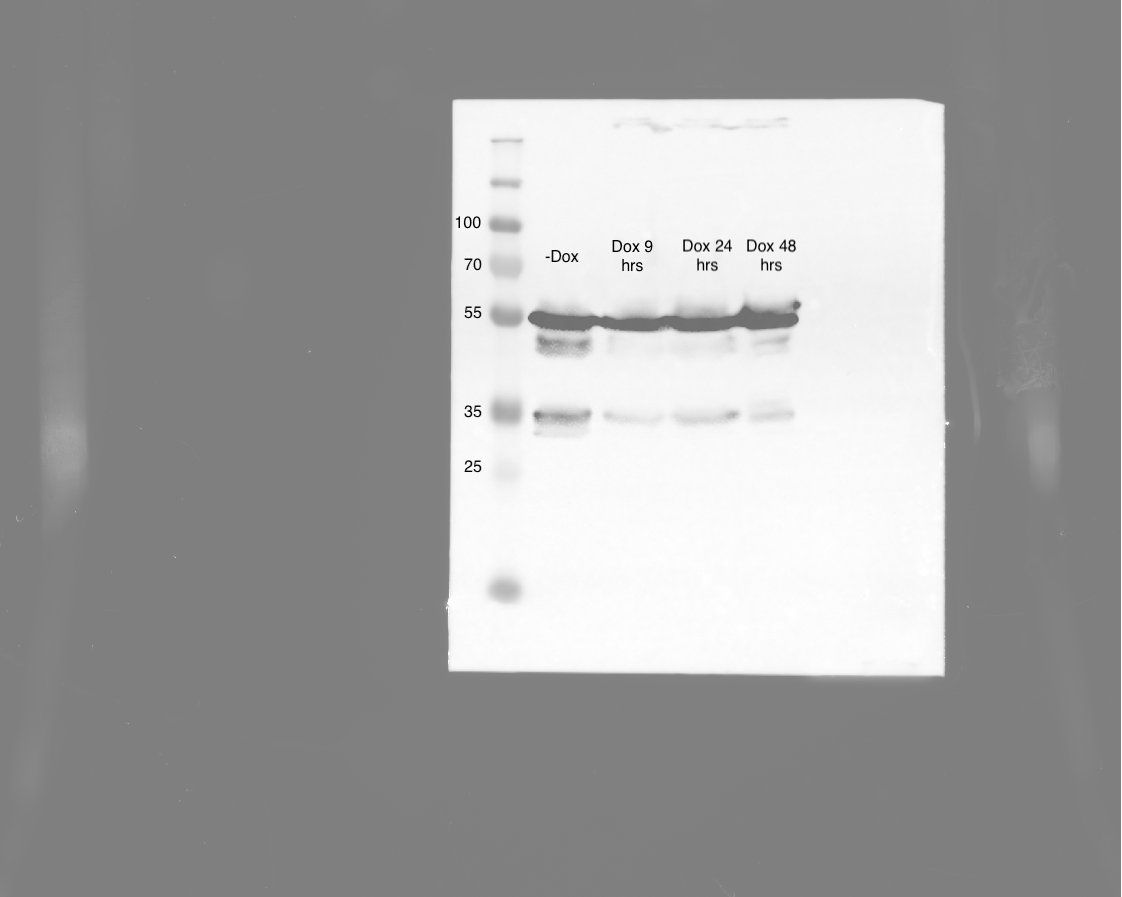

Supplement: Figure 1—source data 3. [file elife-106699-fig1-data3.zip › Figure 1ΓÇösource data 3 PDF files containing original western blots for Figure 1B, indicating the relevant bands and treatments./Raw data/GATA3.tif]

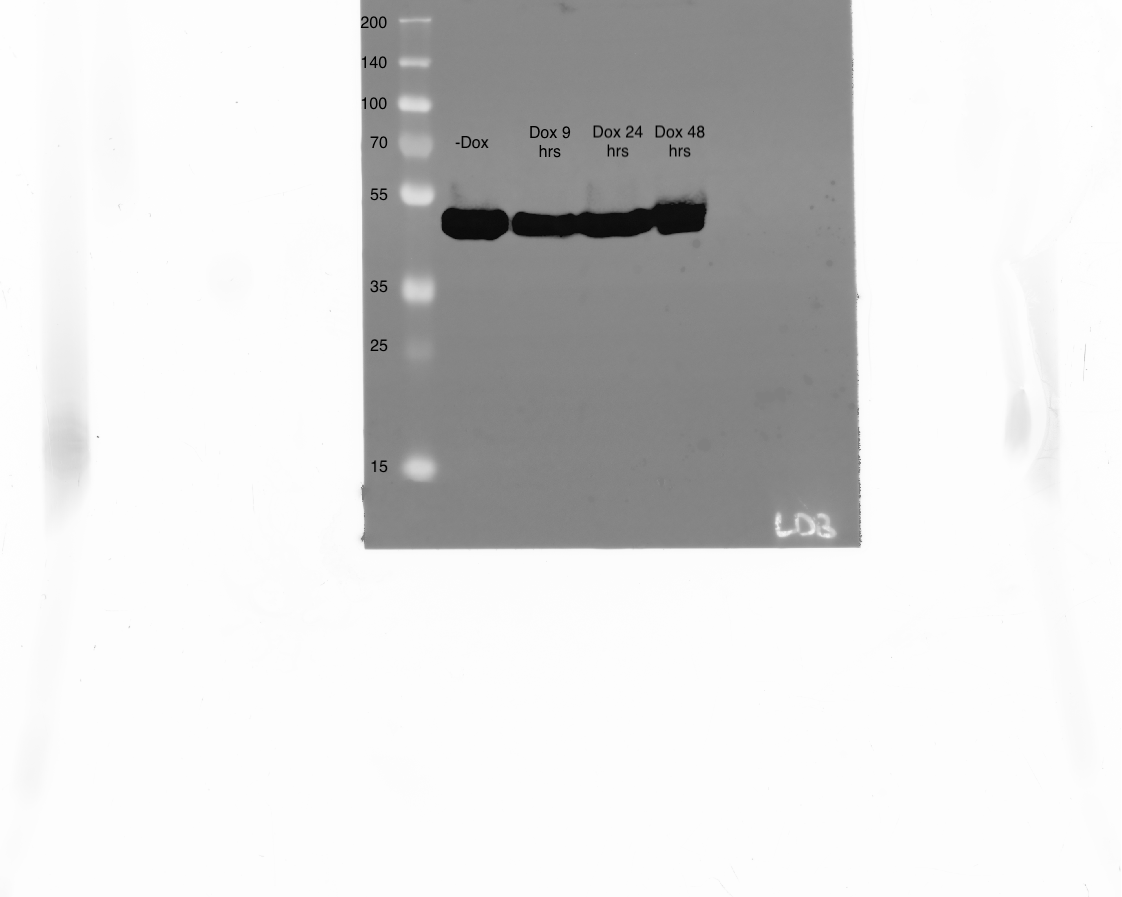

Supplement: Figure 1—source data 3. [file elife-106699-fig1-data3.zip › Figure 1ΓÇösource data 3 PDF files containing original western blots for Figure 1B, indicating the relevant bands and treatments./Raw data/LDB1.tif]

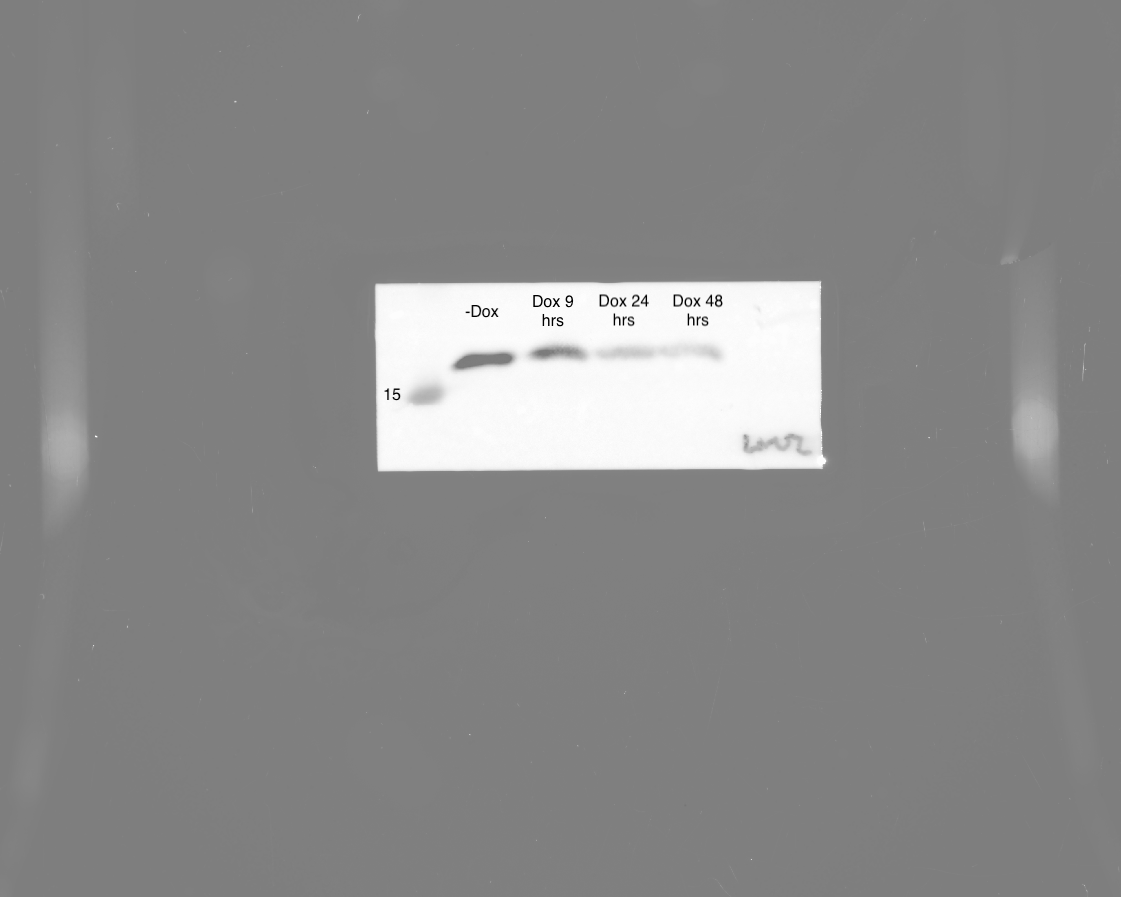

Supplement: Figure 1—source data 3. [file elife-106699-fig1-data3.zip › Figure 1ΓÇösource data 3 PDF files containing original western blots for Figure 1B, indicating the relevant bands and treatments./Raw data/LMO2.tif]

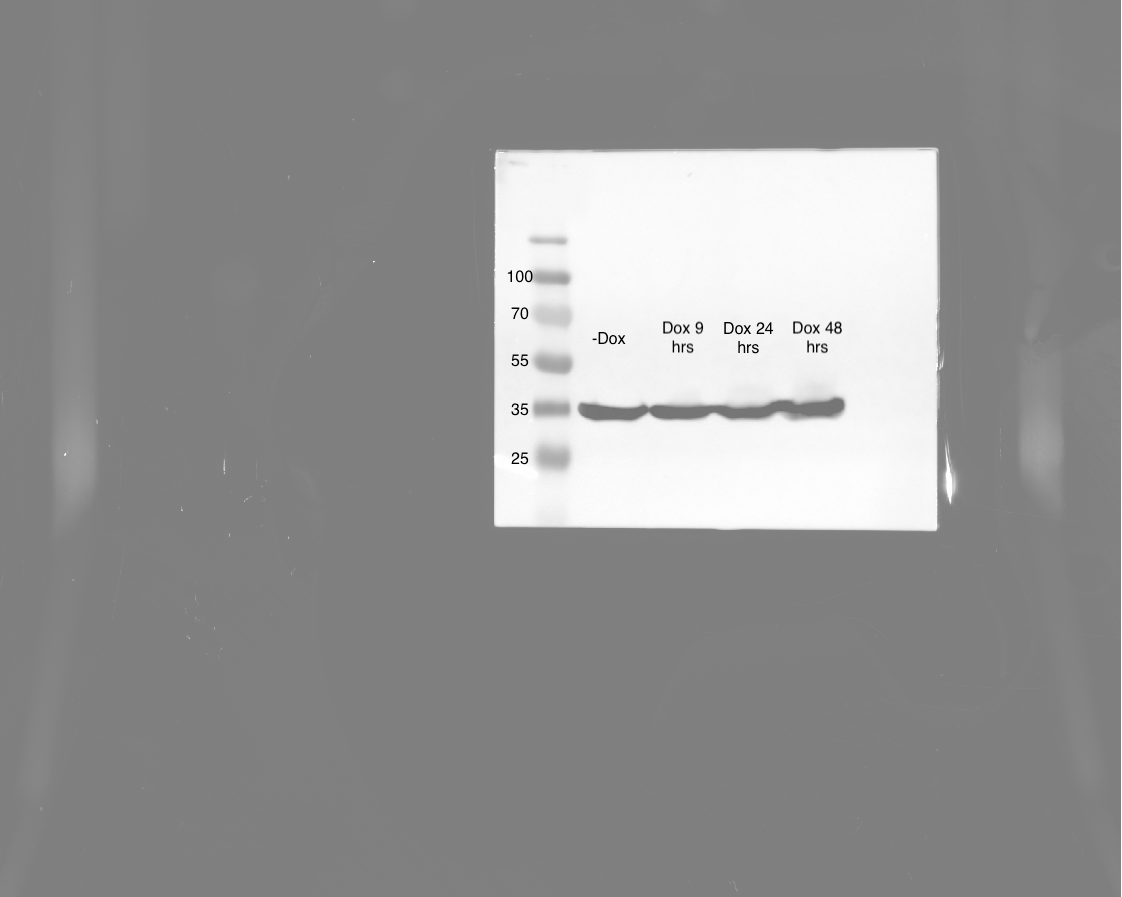

Supplement: Figure 1—source data 3. [file elife-106699-fig1-data3.zip › Figure 1ΓÇösource data 3 PDF files containing original western blots for Figure 1B, indicating the relevant bands and treatments./Raw data/Lyl-1.tif]

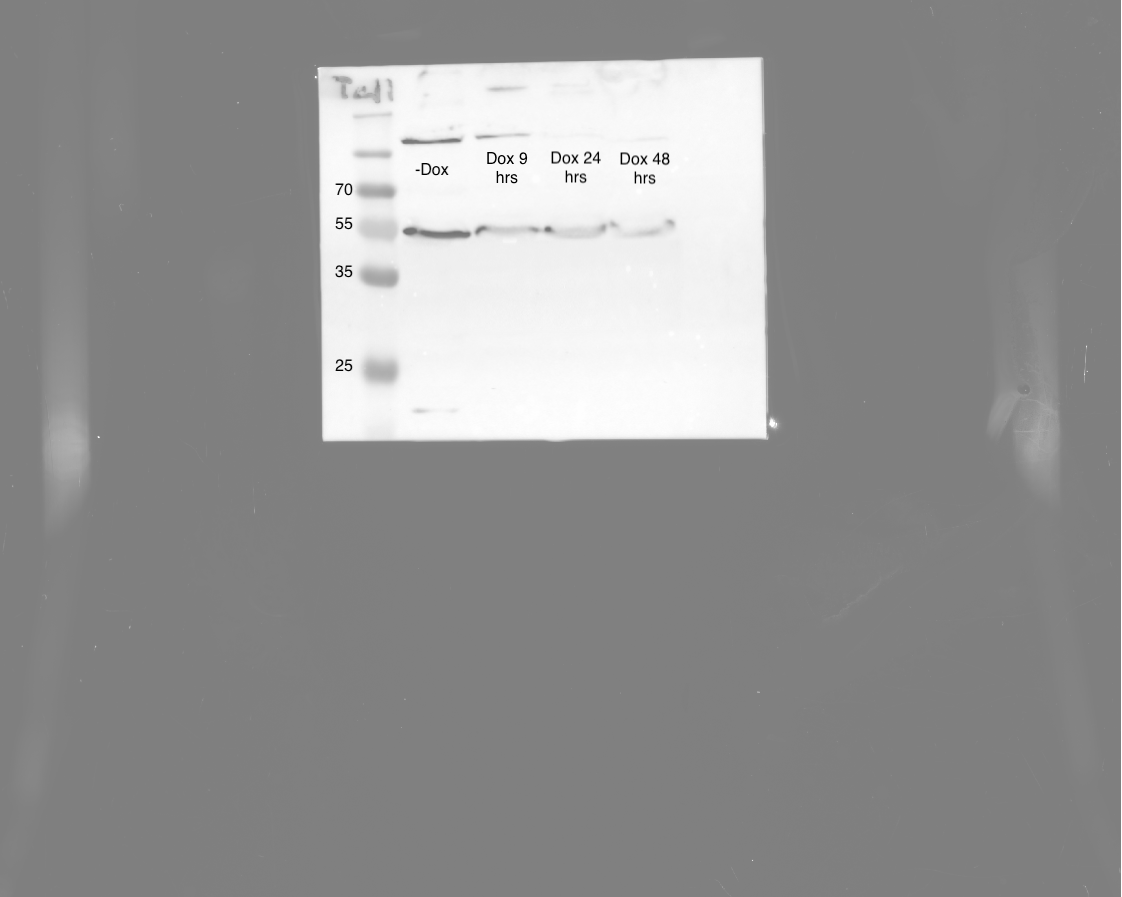

Supplement: Figure 1—source data 3. [file elife-106699-fig1-data3.zip › Figure 1ΓÇösource data 3 PDF files containing original western blots for Figure 1B, indicating the relevant bands and treatments./Raw data/Tal-1.tif]

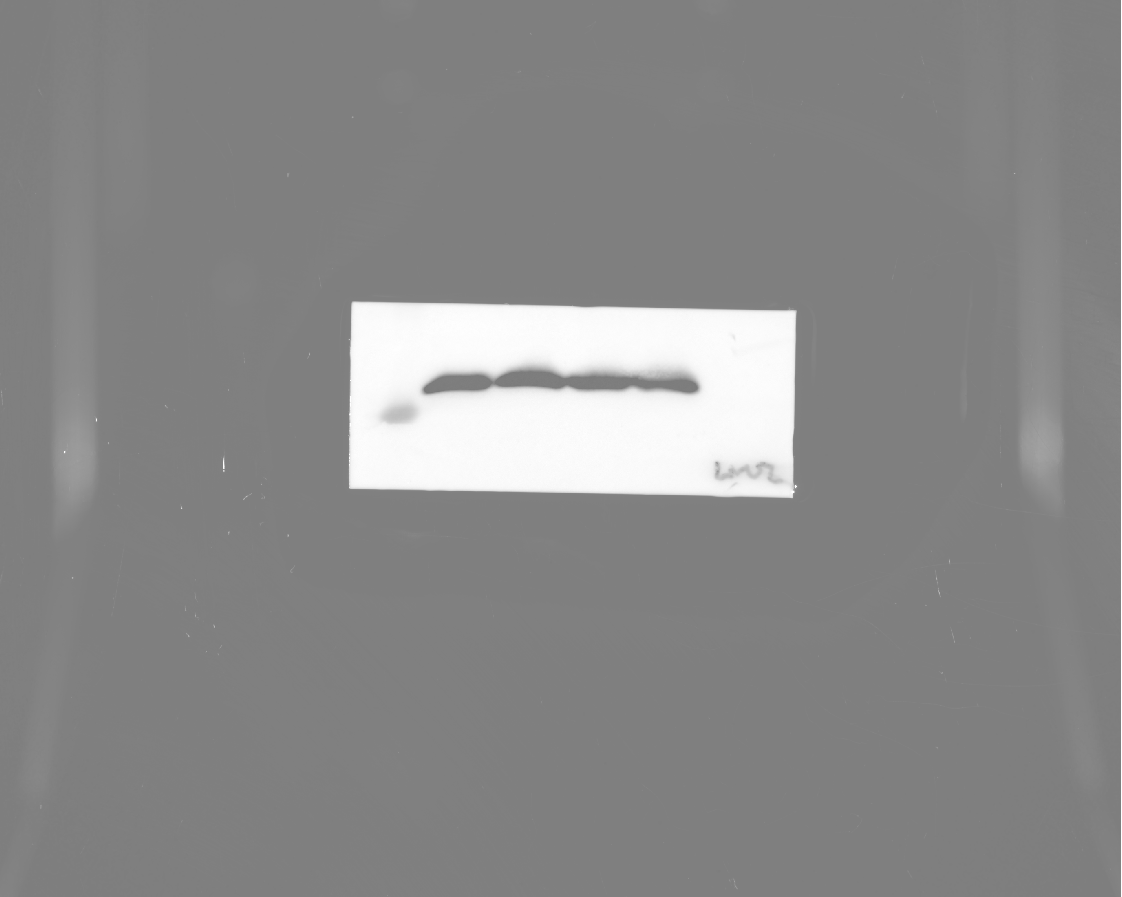

Supplement: Figure 1—source data 4. [file elife-106699-fig1-data4.zip › Figure 1ΓÇösource data 4 Original files for Western blot analysis displayed in Figure 1B./Cyclophilin.tif]

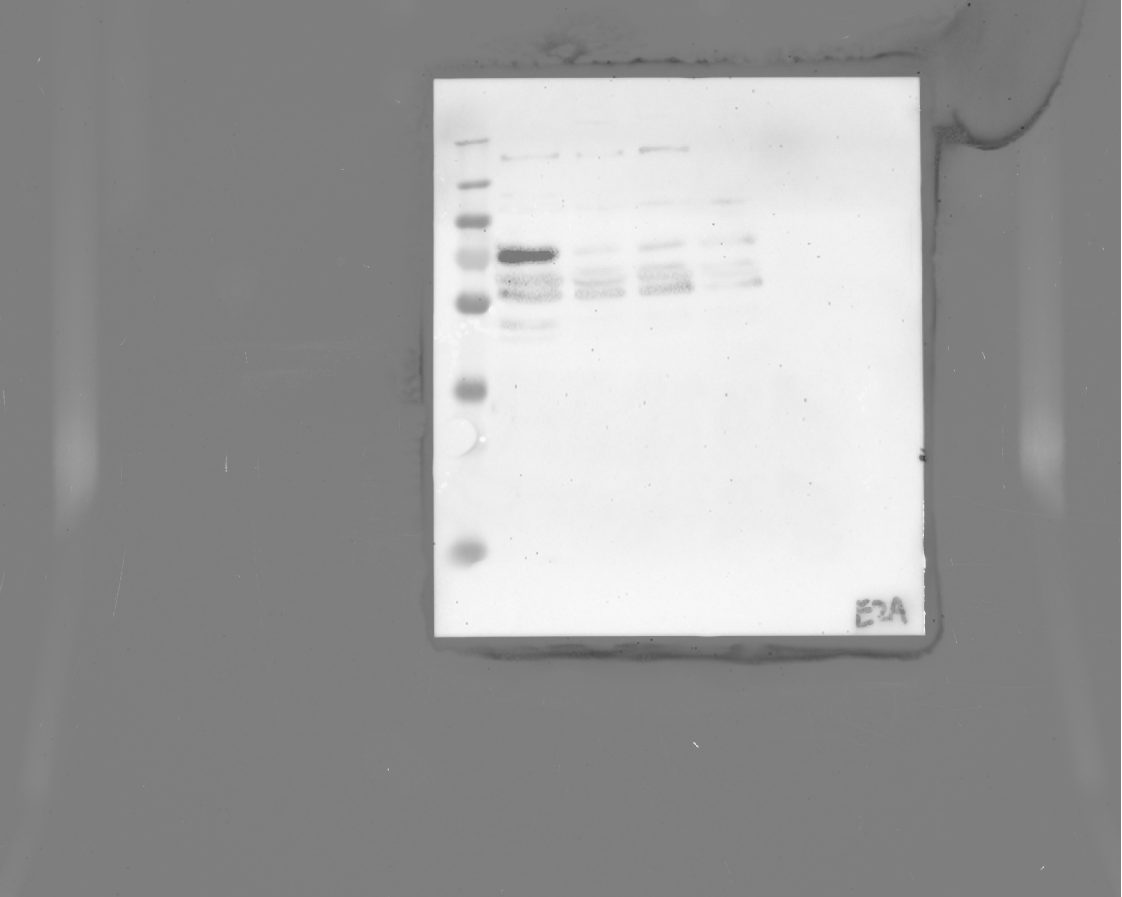

Supplement: Figure 1—source data 4. [file elife-106699-fig1-data4.zip › Figure 1ΓÇösource data 4 Original files for Western blot analysis displayed in Figure 1B./E47.tif]

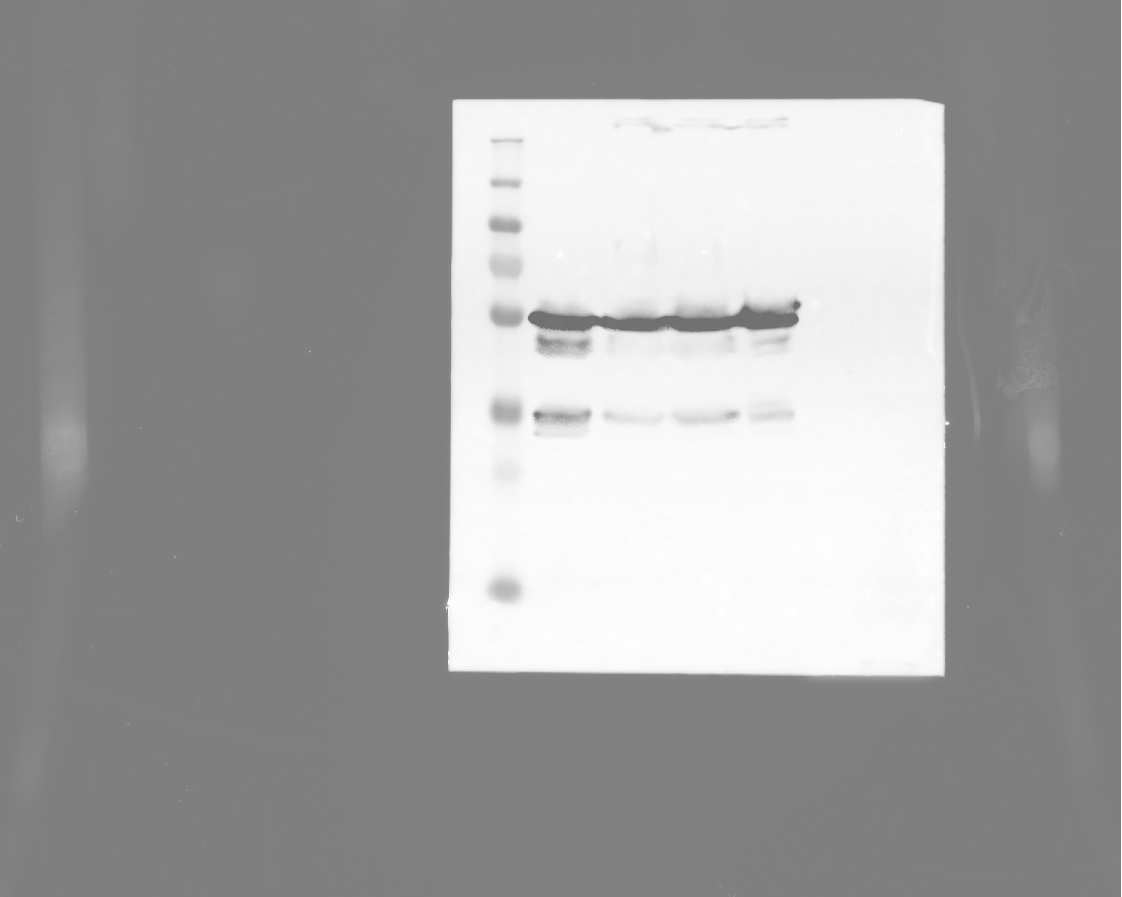

Supplement: Figure 1—source data 4. [file elife-106699-fig1-data4.zip › Figure 1ΓÇösource data 4 Original files for Western blot analysis displayed in Figure 1B./GATA3.tif]

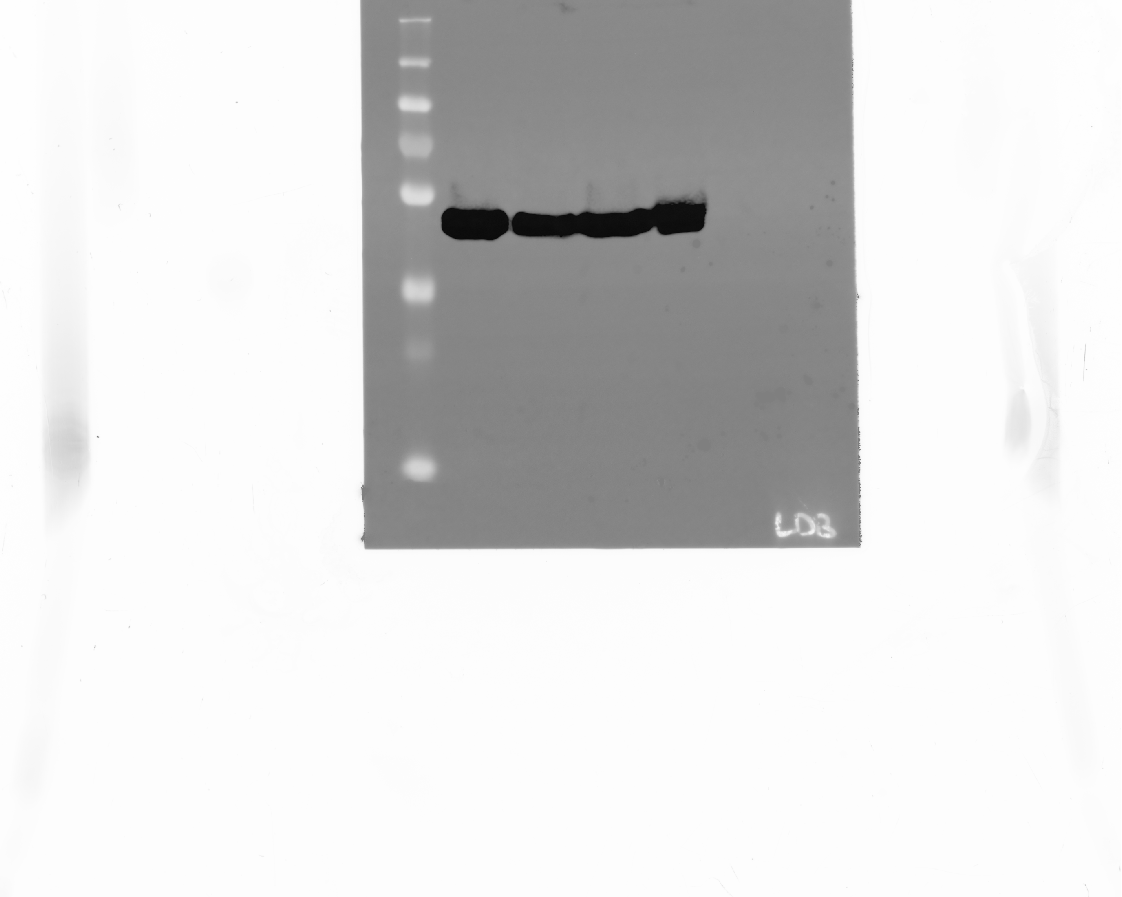

Supplement: Figure 1—source data 4. [file elife-106699-fig1-data4.zip › Figure 1ΓÇösource data 4 Original files for Western blot analysis displayed in Figure 1B./LDB1.tif]

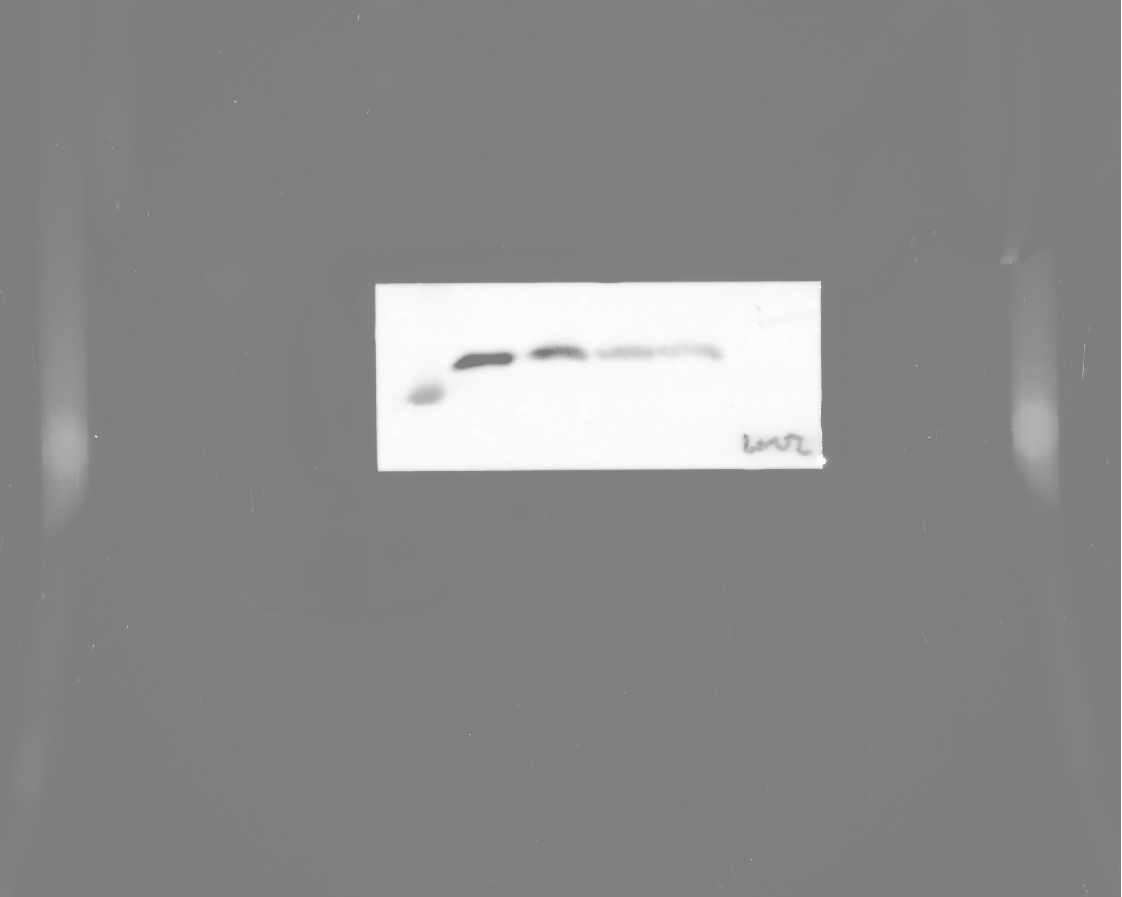

Supplement: Figure 1—source data 4. [file elife-106699-fig1-data4.zip › Figure 1ΓÇösource data 4 Original files for Western blot analysis displayed in Figure 1B./LMO2.tif]

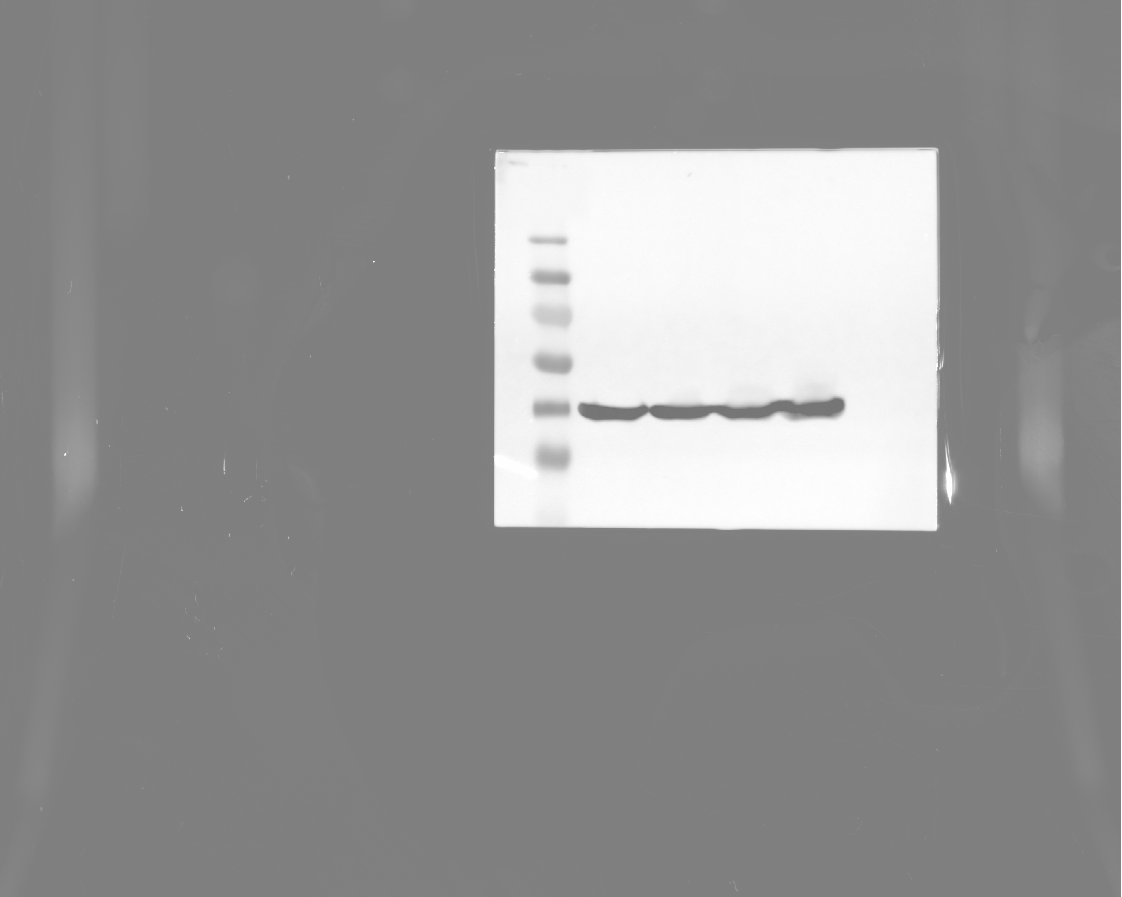

Supplement: Figure 1—source data 4. [file elife-106699-fig1-data4.zip › Figure 1ΓÇösource data 4 Original files for Western blot analysis displayed in Figure 1B./Lyl-1.tif]

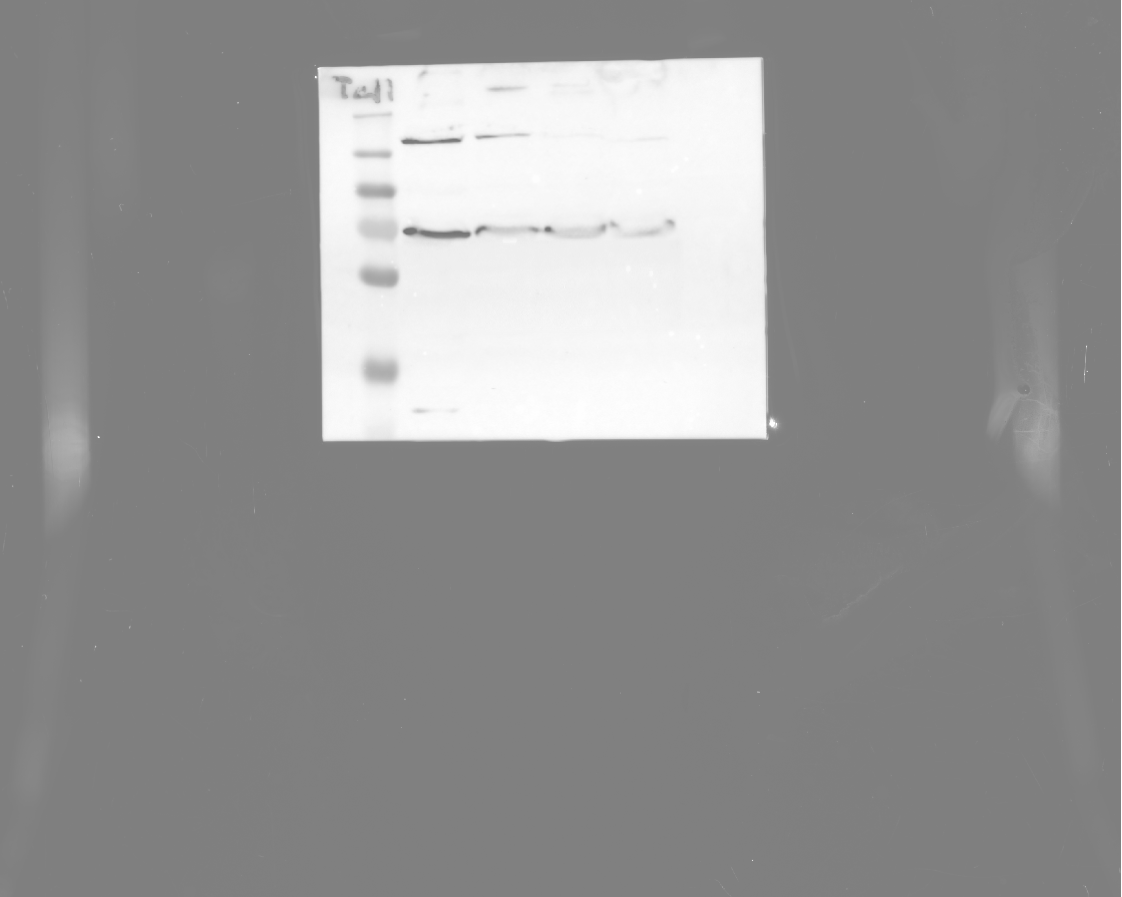

Supplement: Figure 1—source data 4. [file elife-106699-fig1-data4.zip › Figure 1ΓÇösource data 4 Original files for Western blot analysis displayed in Figure 1B./Tal-1.tif]

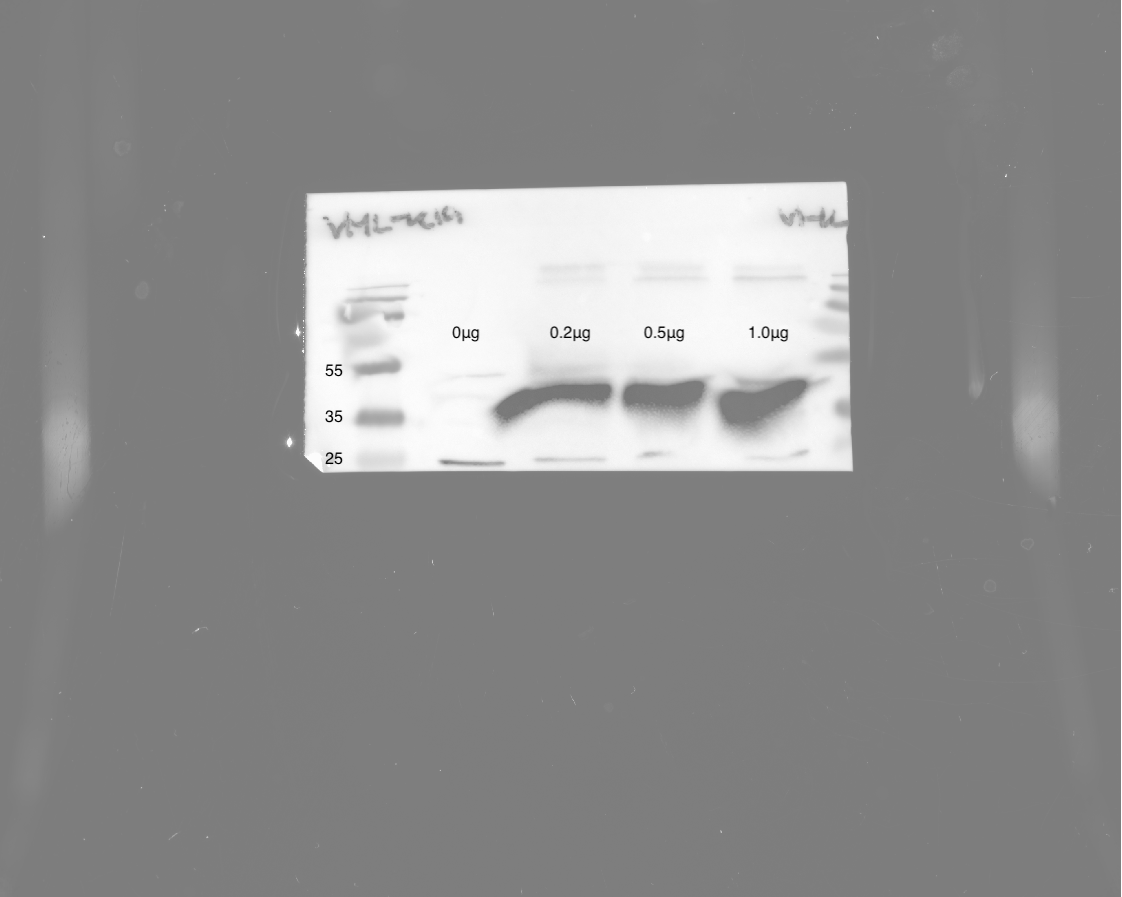

Supplement: Figure 1—figure supplement 1—source data 1. [file elife-106699-fig1-figsupp1-data1.zip › Figure 1ΓÇöfigure supplement 1-source data 1 Western blot data with label shows LMO2 protein degradation in HEK293T cells with different biodegrader contruct./Raw data/VHL VHL-iDabRas(Composite).tif]

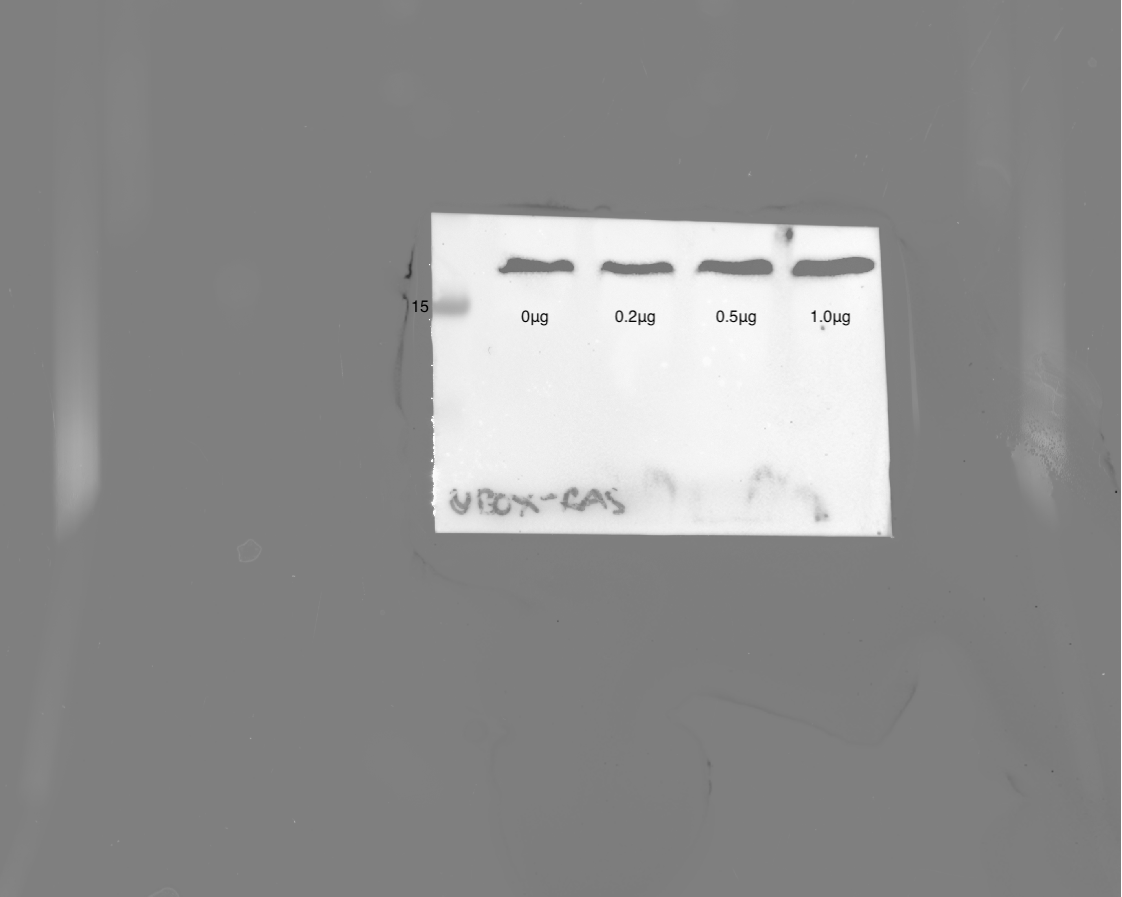

Supplement: Figure 1—figure supplement 1—source data 1. [file elife-106699-fig1-figsupp1-data1.zip › Figure 1ΓÇöfigure supplement 1-source data 1 Western blot data with label shows LMO2 protein degradation in HEK293T cells with different biodegrader contruct./Raw data/pan-RAS iDab-UBOX(Composite).tif]

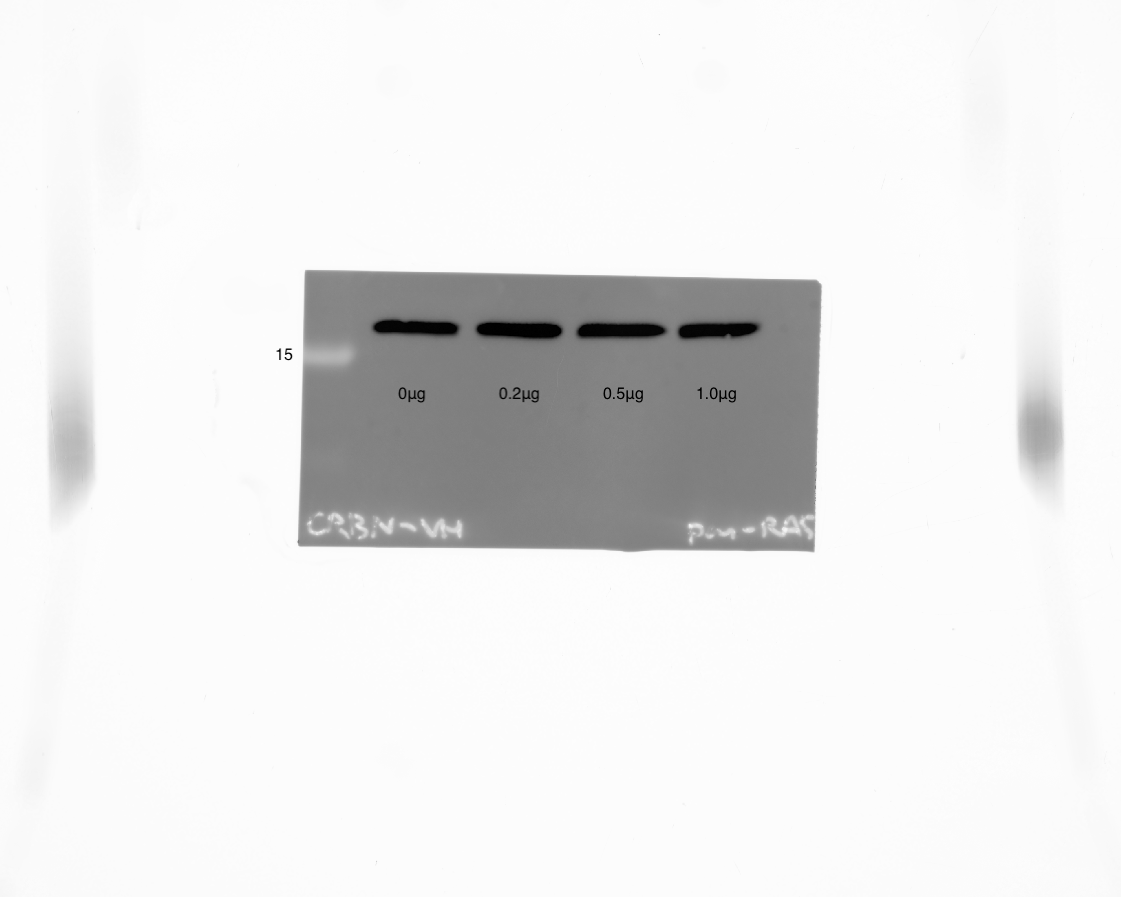

Supplement: Figure 1—figure supplement 1—source data 1. [file elife-106699-fig1-figsupp1-data1.zip › Figure 1ΓÇöfigure supplement 1-source data 1 Western blot data with label shows LMO2 protein degradation in HEK293T cells with different biodegrader contruct./Raw data/Cyclophilin CRBN-iDab(Composite).tif]

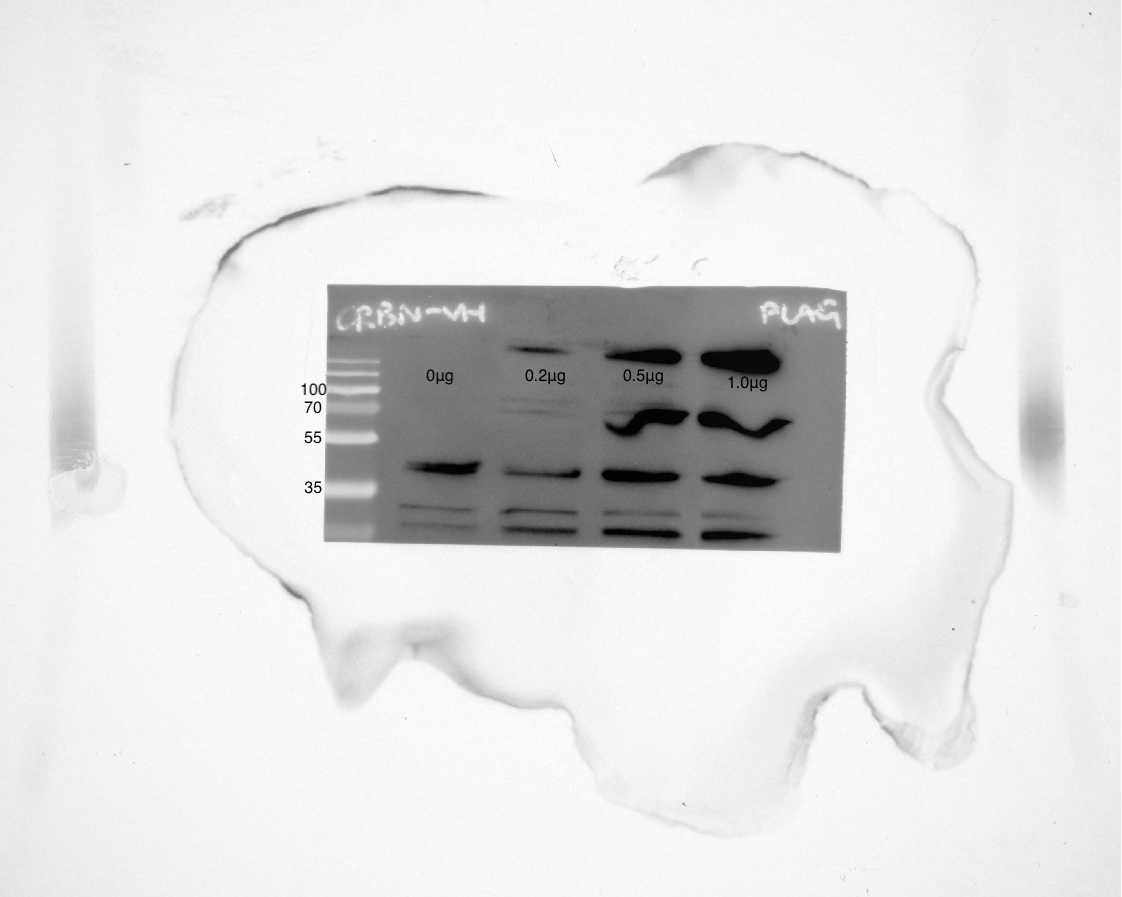

Supplement: Figure 1—figure supplement 1—source data 1. [file elife-106699-fig1-figsupp1-data1.zip › Figure 1ΓÇöfigure supplement 1-source data 1 Western blot data with label shows LMO2 protein degradation in HEK293T cells with different biodegrader contruct./Raw data/FLAG CRBN-iDab(Composite).tif]

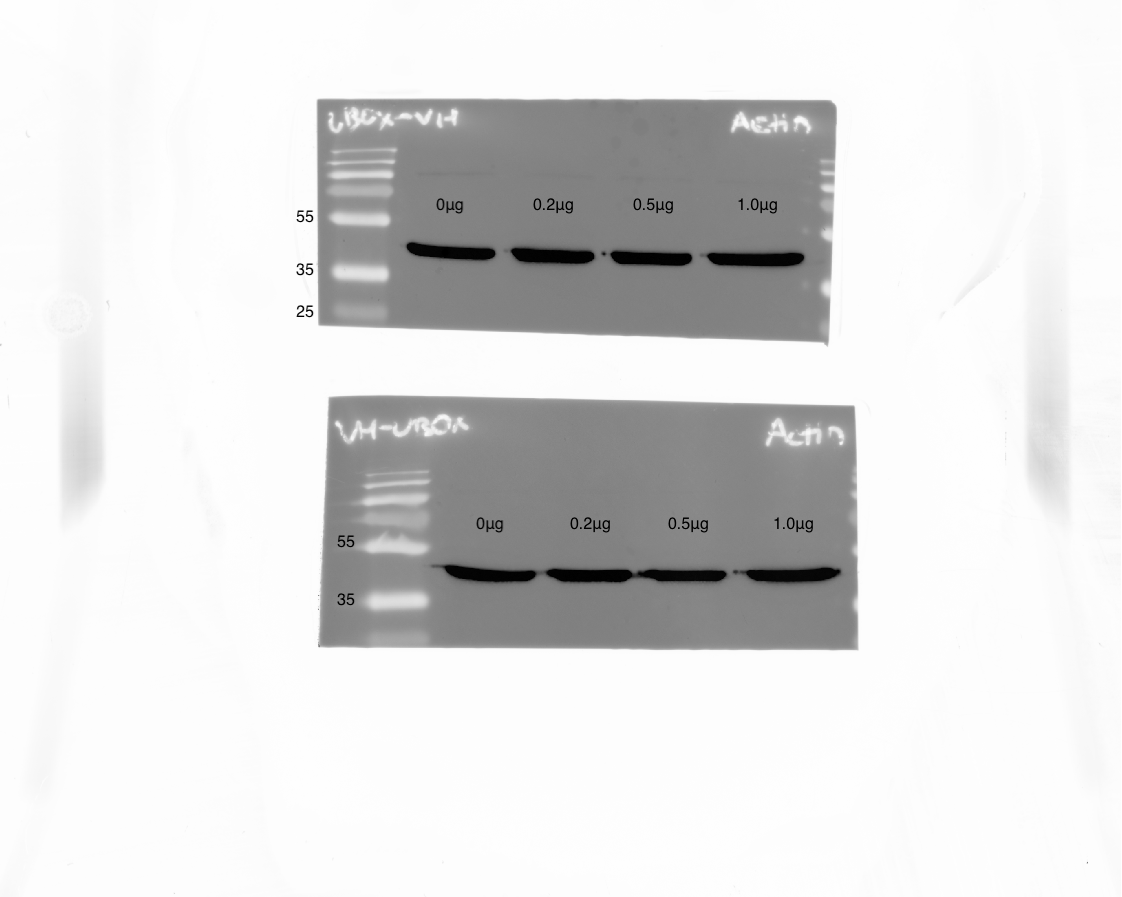

Supplement: Figure 1—figure supplement 1—source data 1. [file elife-106699-fig1-figsupp1-data1.zip › Figure 1ΓÇöfigure supplement 1-source data 1 Western blot data with label shows LMO2 protein degradation in HEK293T cells with different biodegrader contruct./Raw data/Actin UBOX-iDab AND iDab-UBOX(Composite).tif]

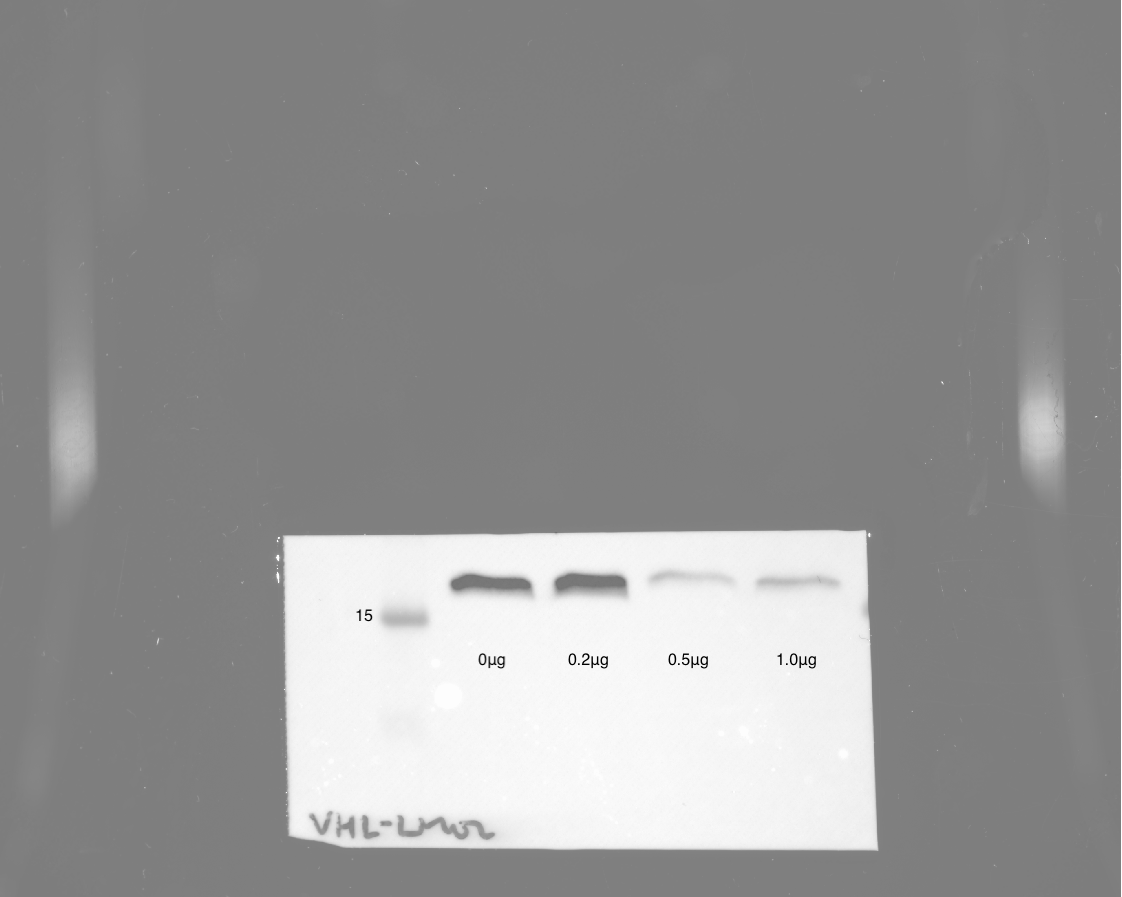

Supplement: Figure 1—figure supplement 1—source data 1. [file elife-106699-fig1-figsupp1-data1.zip › Figure 1ΓÇöfigure supplement 1-source data 1 Western blot data with label shows LMO2 protein degradation in HEK293T cells with different biodegrader contruct./Raw data/LMO2 VHL-iDab(Composite).tif]

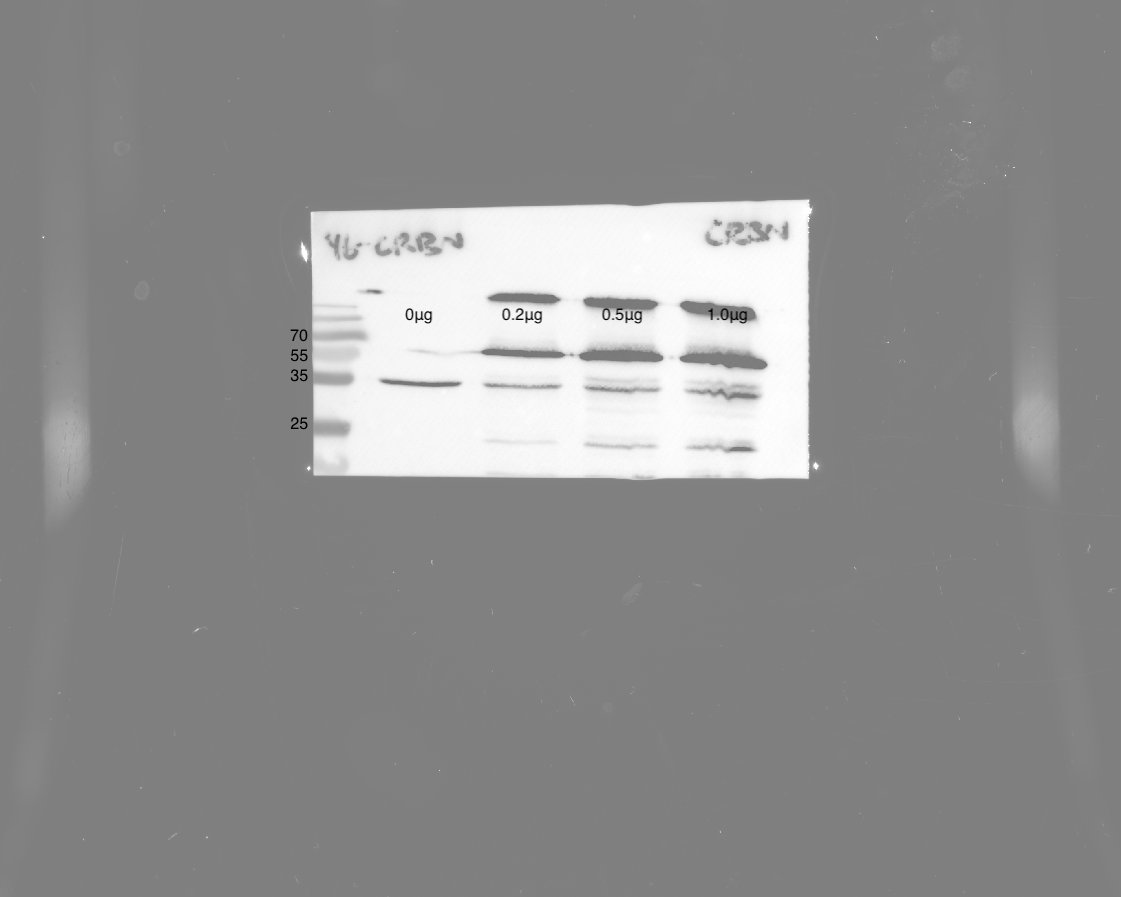

Supplement: Figure 1—figure supplement 1—source data 1. [file elife-106699-fig1-figsupp1-data1.zip › Figure 1ΓÇöfigure supplement 1-source data 1 Western blot data with label shows LMO2 protein degradation in HEK293T cells with different biodegrader contruct./Raw data/CRBN iDabRas-CRBN(Composite).tif]

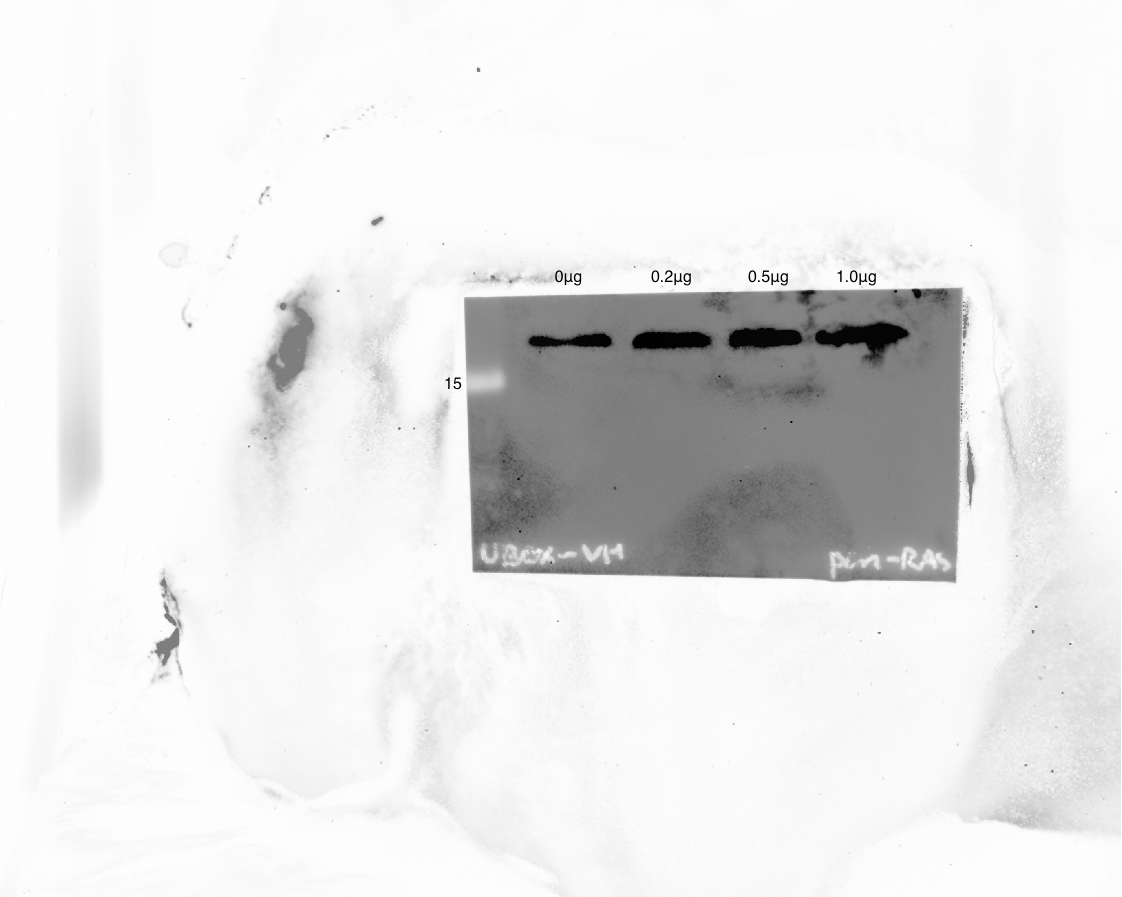

Supplement: Figure 1—figure supplement 1—source data 1. [file elife-106699-fig1-figsupp1-data1.zip › Figure 1ΓÇöfigure supplement 1-source data 1 Western blot data with label shows LMO2 protein degradation in HEK293T cells with different biodegrader contruct./Raw data/panRAS UBOX-iDab(Composite).tif]

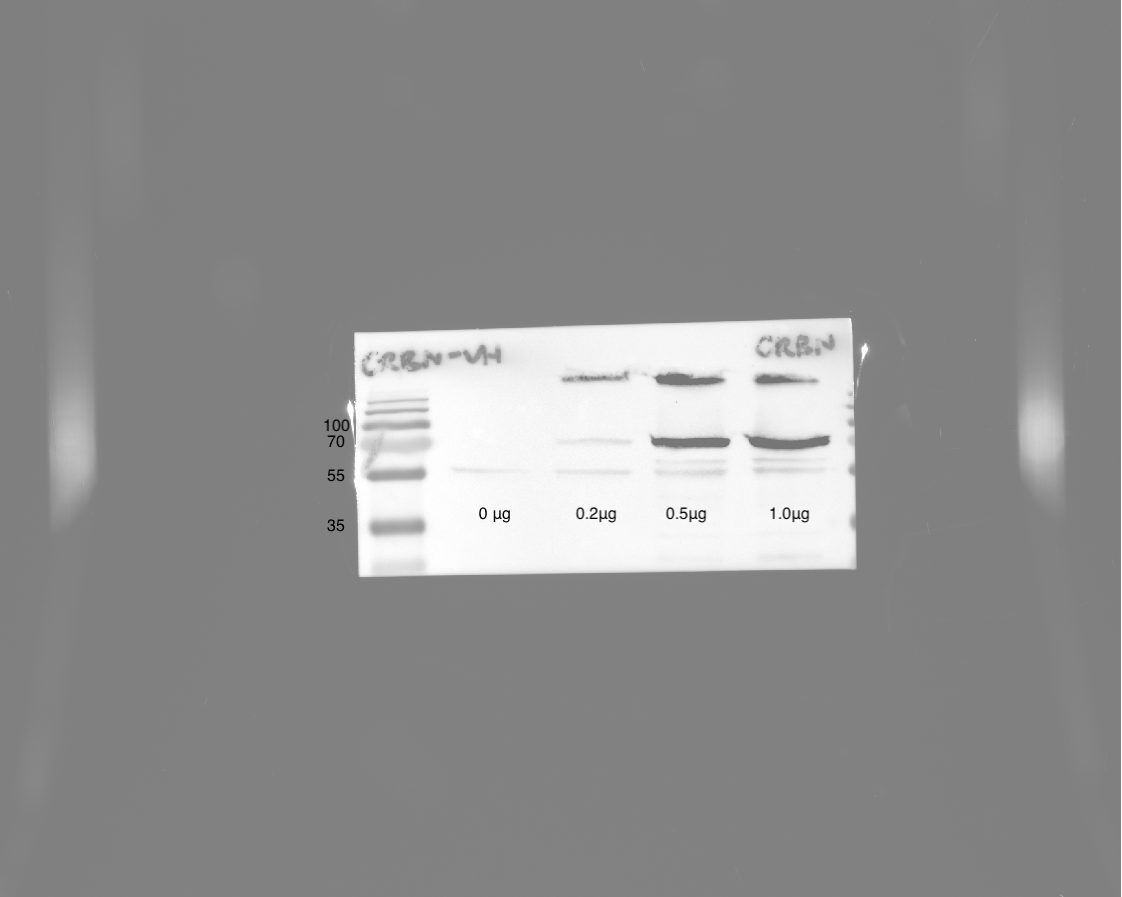

Supplement: Figure 1—figure supplement 1—source data 1. [file elife-106699-fig1-figsupp1-data1.zip › Figure 1ΓÇöfigure supplement 1-source data 1 Western blot data with label shows LMO2 protein degradation in HEK293T cells with different biodegrader contruct./Raw data/CRBN CRBN-iDab(Composite).tif]

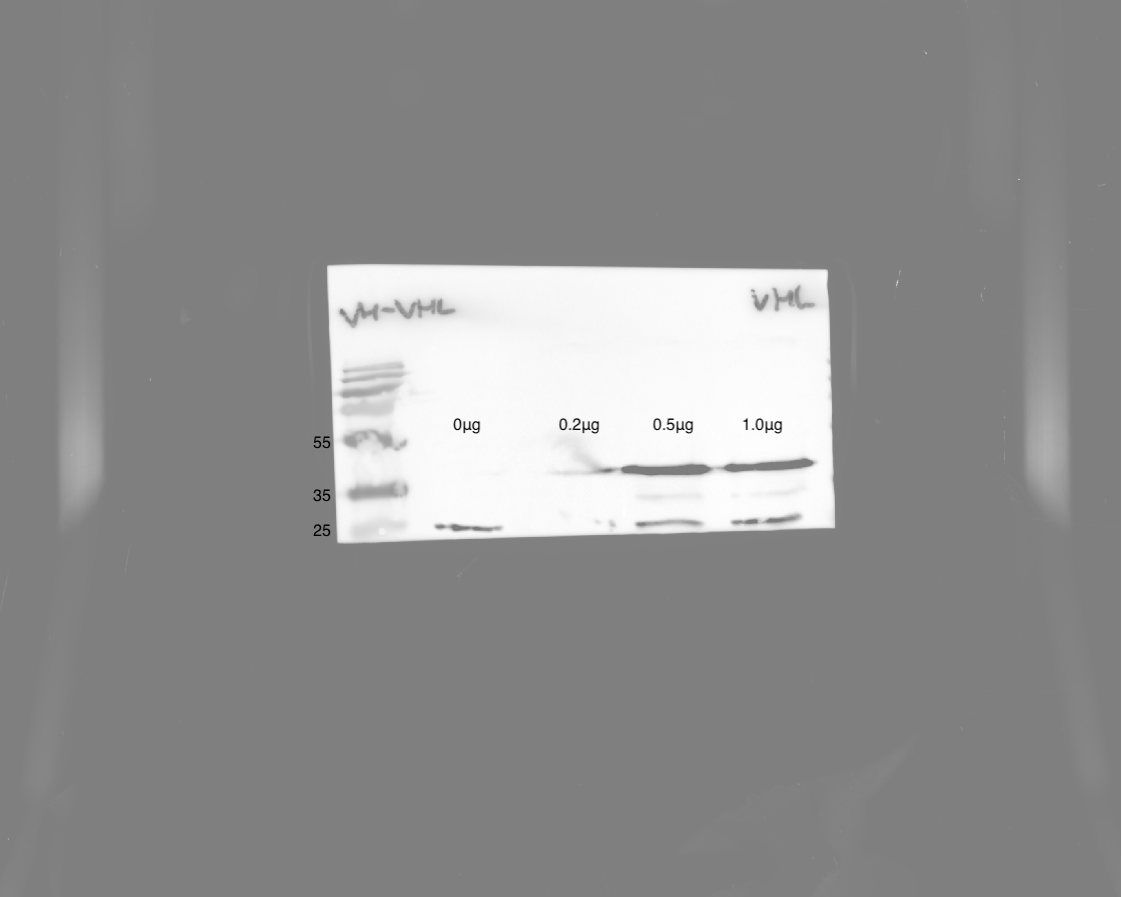

Supplement: Figure 1—figure supplement 1—source data 1. [file elife-106699-fig1-figsupp1-data1.zip › Figure 1ΓÇöfigure supplement 1-source data 1 Western blot data with label shows LMO2 protein degradation in HEK293T cells with different biodegrader contruct./Raw data/VHL iDab-VHL(Composite).tif]

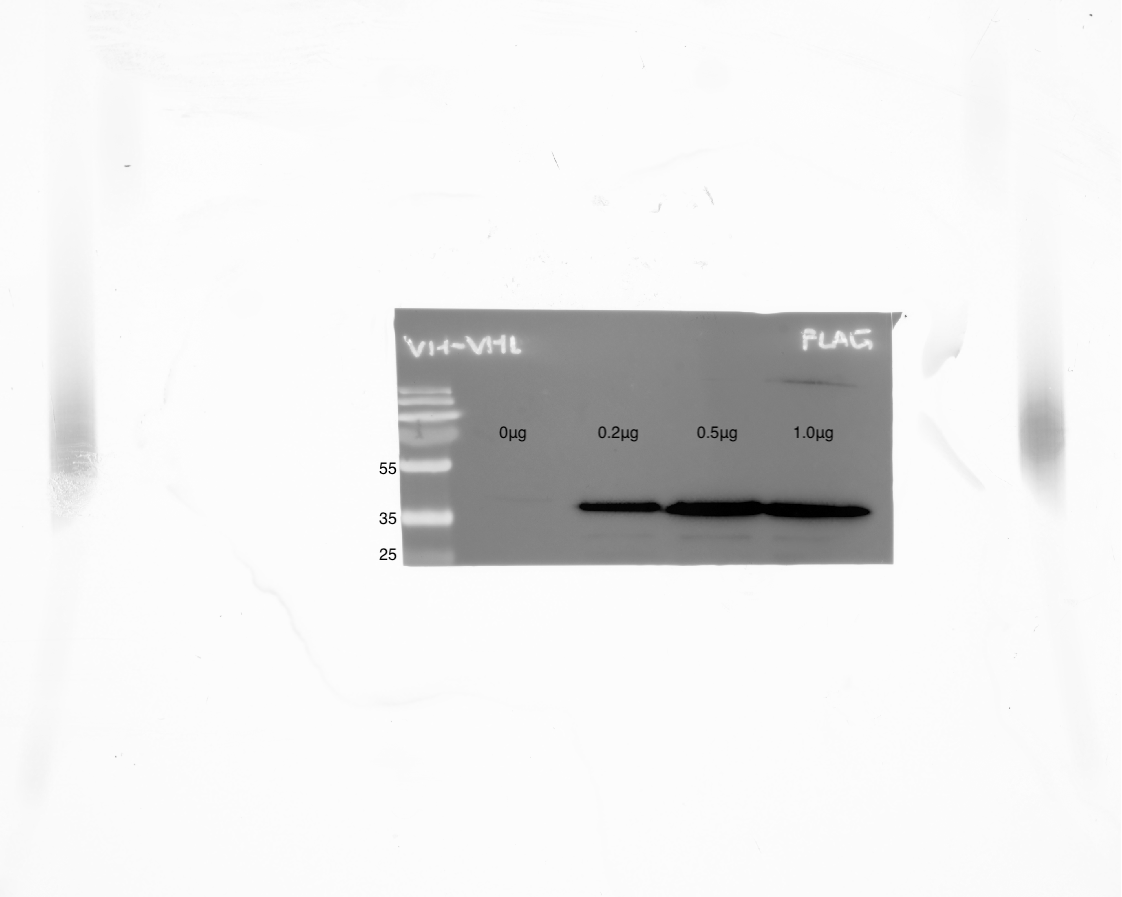

Supplement: Figure 1—figure supplement 1—source data 1. [file elife-106699-fig1-figsupp1-data1.zip › Figure 1ΓÇöfigure supplement 1-source data 1 Western blot data with label shows LMO2 protein degradation in HEK293T cells with different biodegrader contruct./Raw data/FLAG iDab-VHL(Composite).tif]

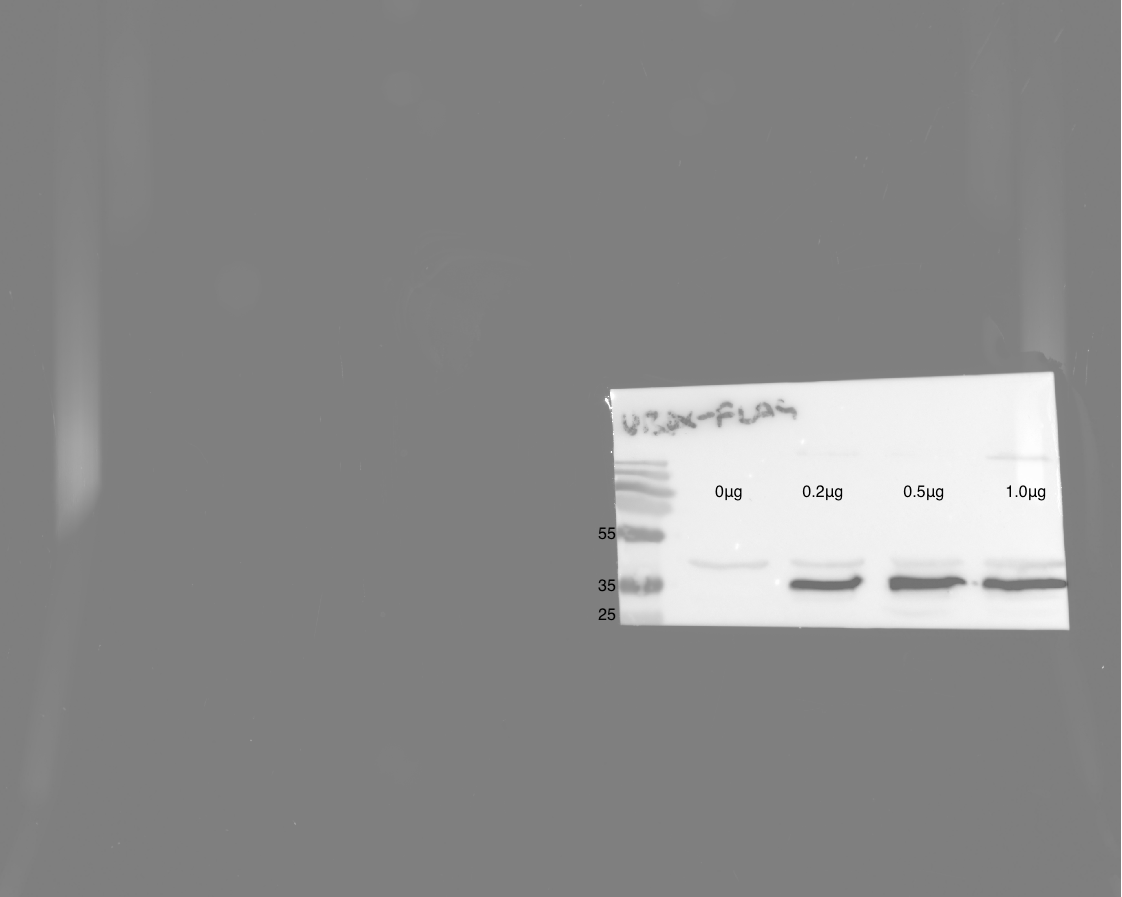

Supplement: Figure 1—figure supplement 1—source data 1. [file elife-106699-fig1-figsupp1-data1.zip › Figure 1ΓÇöfigure supplement 1-source data 1 Western blot data with label shows LMO2 protein degradation in HEK293T cells with different biodegrader contruct./Raw data/Flag iDab-UBOX(Composite).tif]

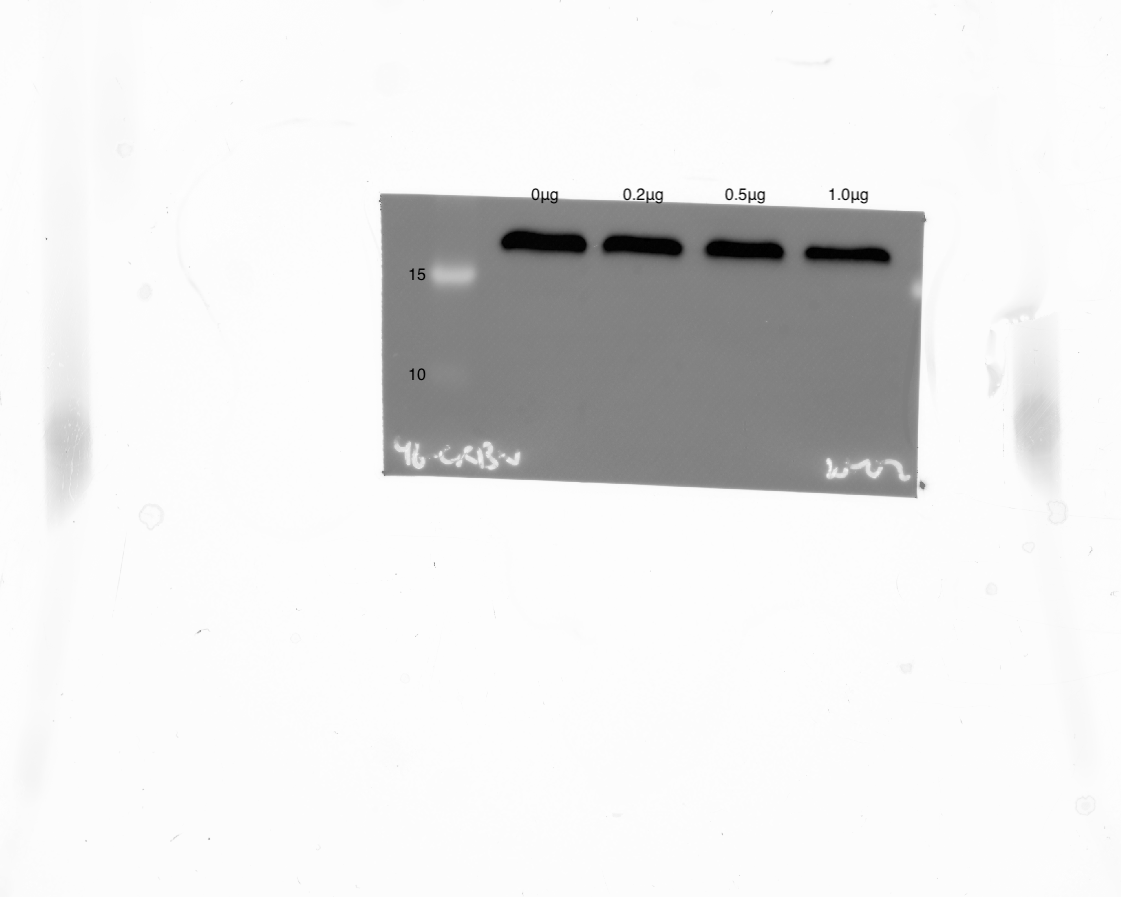

Supplement: Figure 1—figure supplement 1—source data 1. [file elife-106699-fig1-figsupp1-data1.zip › Figure 1ΓÇöfigure supplement 1-source data 1 Western blot data with label shows LMO2 protein degradation in HEK293T cells with different biodegrader contruct./Raw data/LMO2 iDabRas-CRBN(Composite).tif]

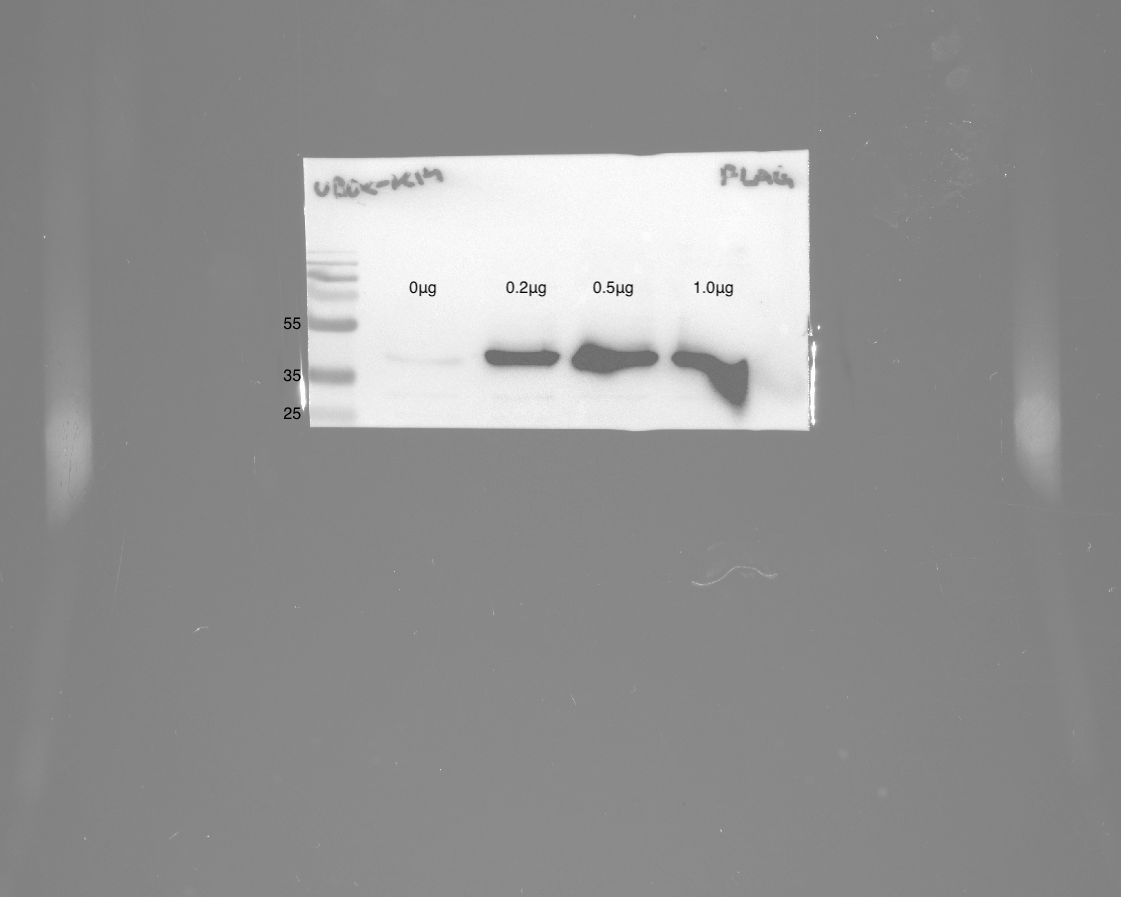

Supplement: Figure 1—figure supplement 1—source data 1. [file elife-106699-fig1-figsupp1-data1.zip › Figure 1ΓÇöfigure supplement 1-source data 1 Western blot data with label shows LMO2 protein degradation in HEK293T cells with different biodegrader contruct./Raw data/FLAG UBOX-iDabRas(Composite).tif]

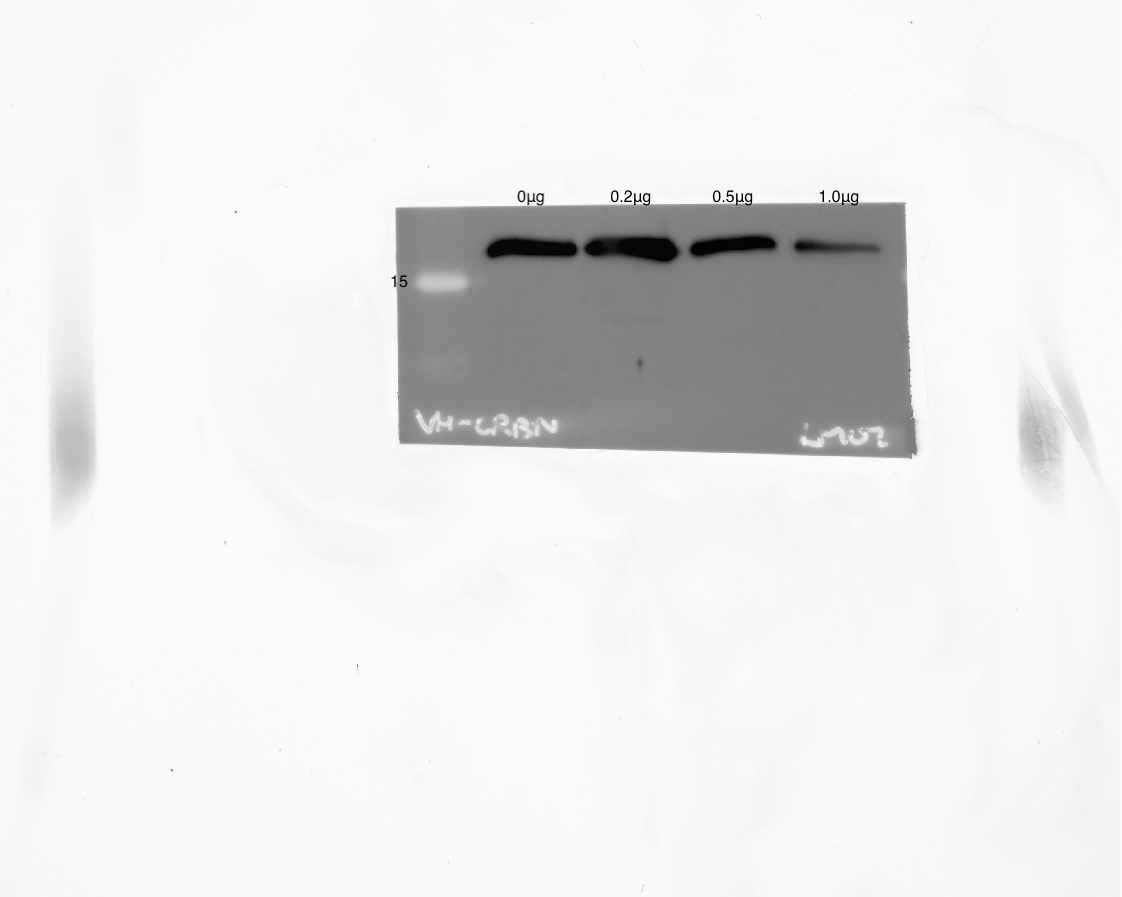

Supplement: Figure 1—figure supplement 1—source data 1. [file elife-106699-fig1-figsupp1-data1.zip › Figure 1ΓÇöfigure supplement 1-source data 1 Western blot data with label shows LMO2 protein degradation in HEK293T cells with different biodegrader contruct./Raw data/LMO2 iDab-CRBN(Composite).tif]

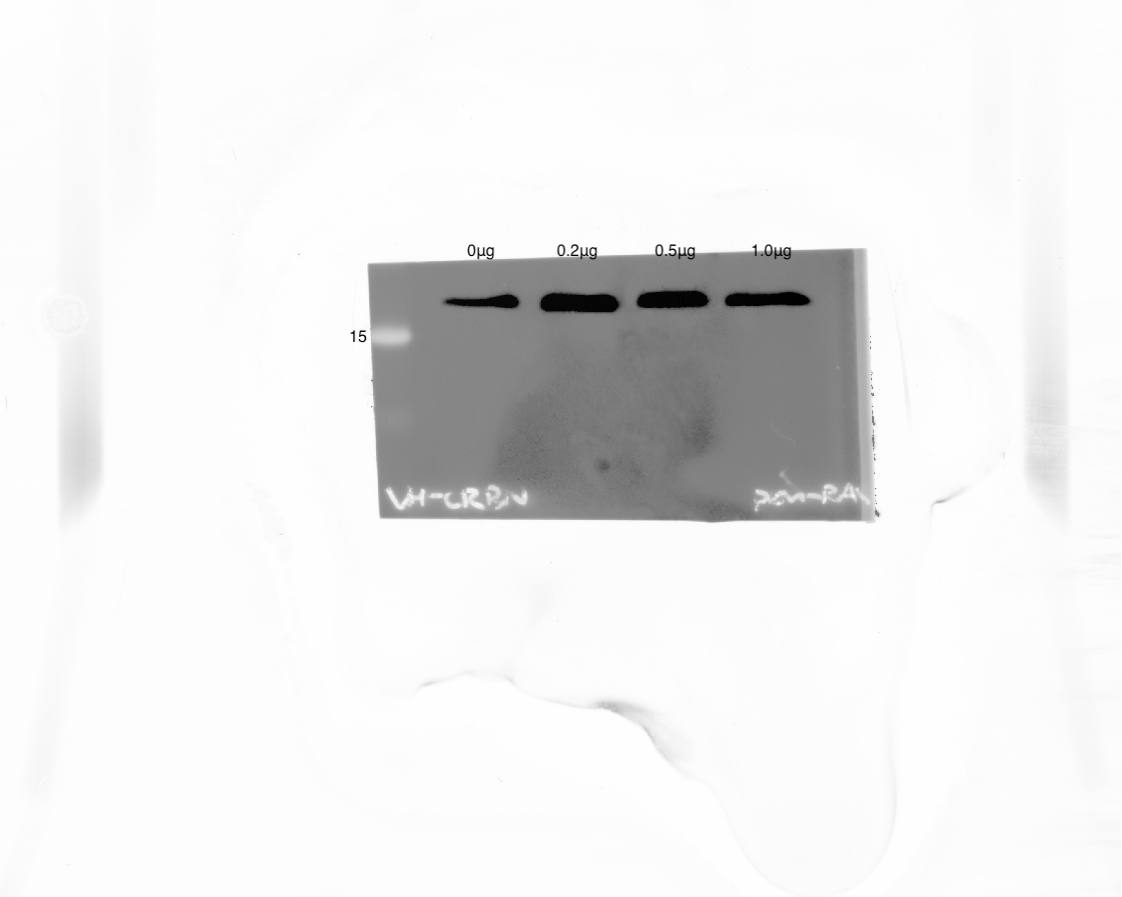

Supplement: Figure 1—figure supplement 1—source data 1. [file elife-106699-fig1-figsupp1-data1.zip › Figure 1ΓÇöfigure supplement 1-source data 1 Western blot data with label shows LMO2 protein degradation in HEK293T cells with different biodegrader contruct./Raw data/panRAS iDab-CRBN(Composite).tif]

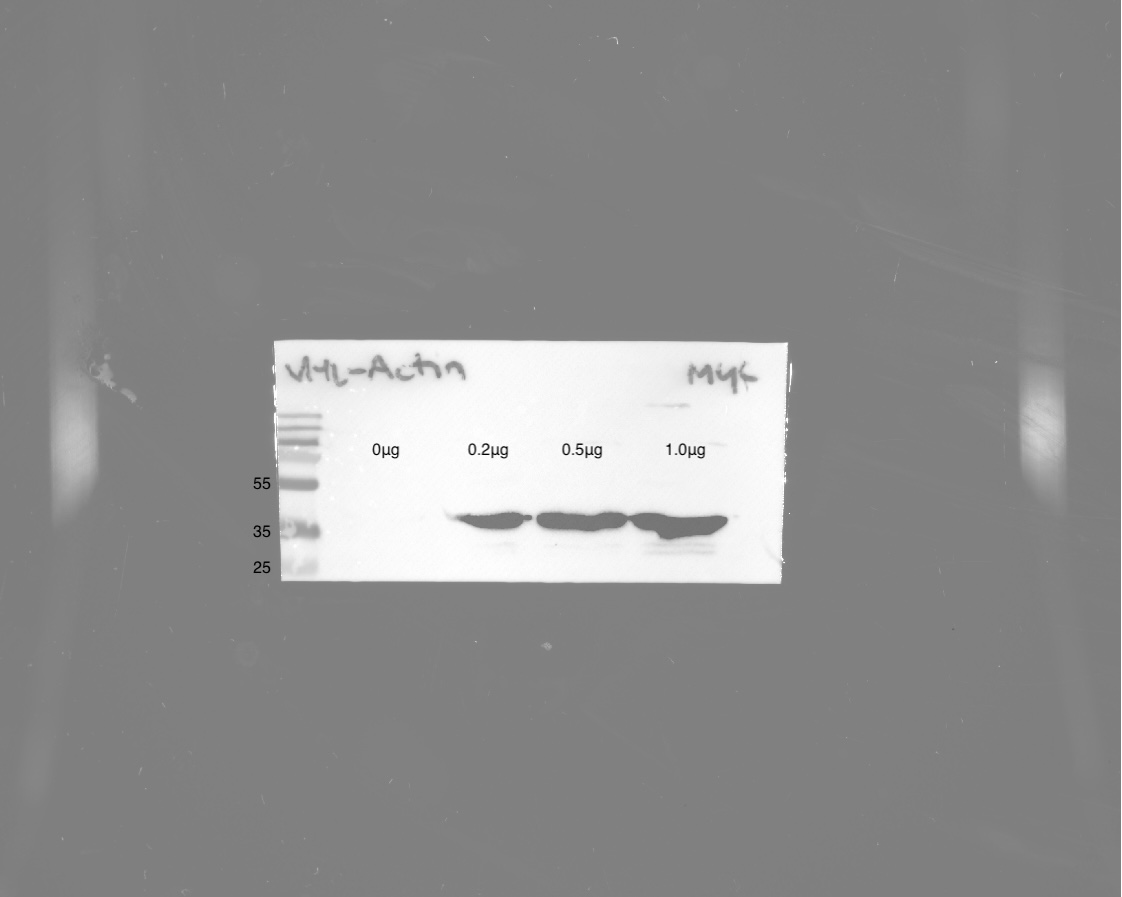

Supplement: Figure 1—figure supplement 1—source data 1. [file elife-106699-fig1-figsupp1-data1.zip › Figure 1ΓÇöfigure supplement 1-source data 1 Western blot data with label shows LMO2 protein degradation in HEK293T cells with different biodegrader contruct./Raw data/myc9E10 VHL-iDab(Composite).jpg]

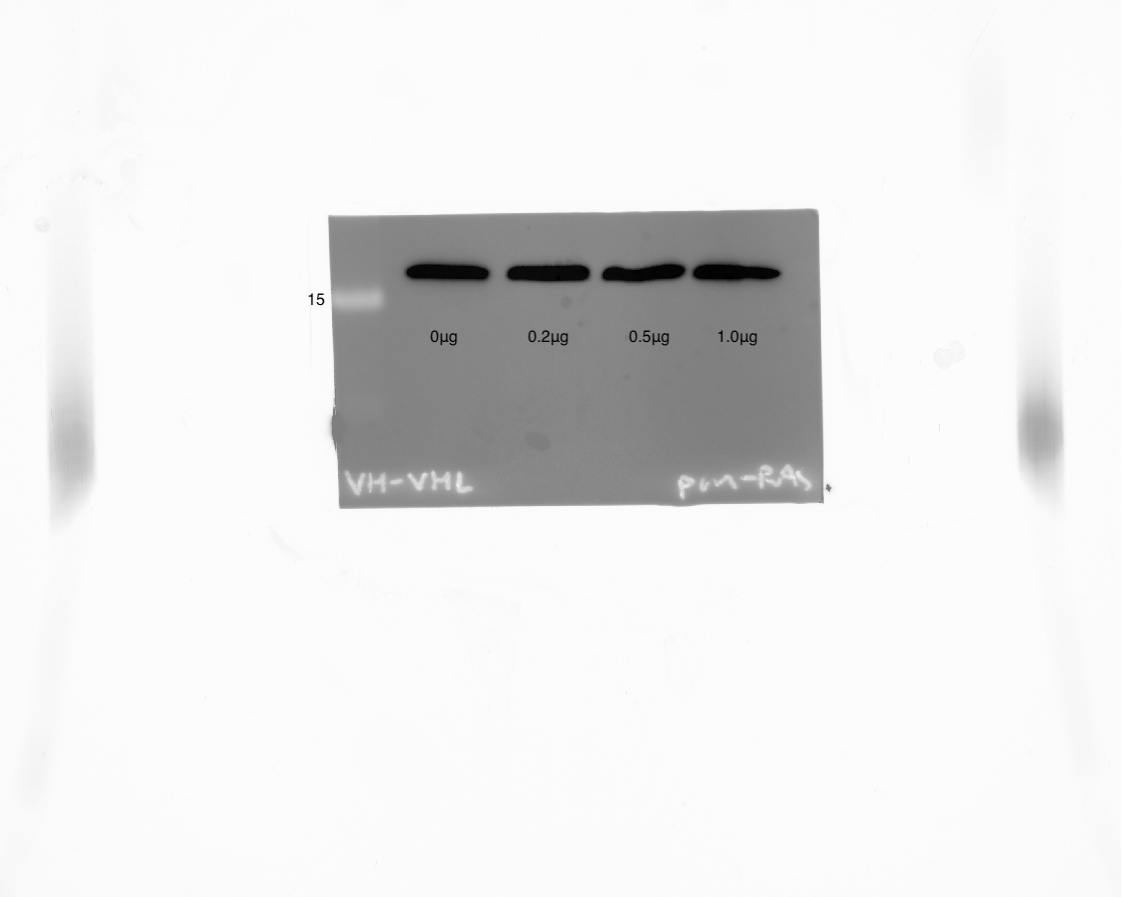

Supplement: Figure 1—figure supplement 1—source data 1. [file elife-106699-fig1-figsupp1-data1.zip › Figure 1ΓÇöfigure supplement 1-source data 1 Western blot data with label shows LMO2 protein degradation in HEK293T cells with different biodegrader contruct./Raw data/Cyclophilin iDab-VHL(Composite).tif]

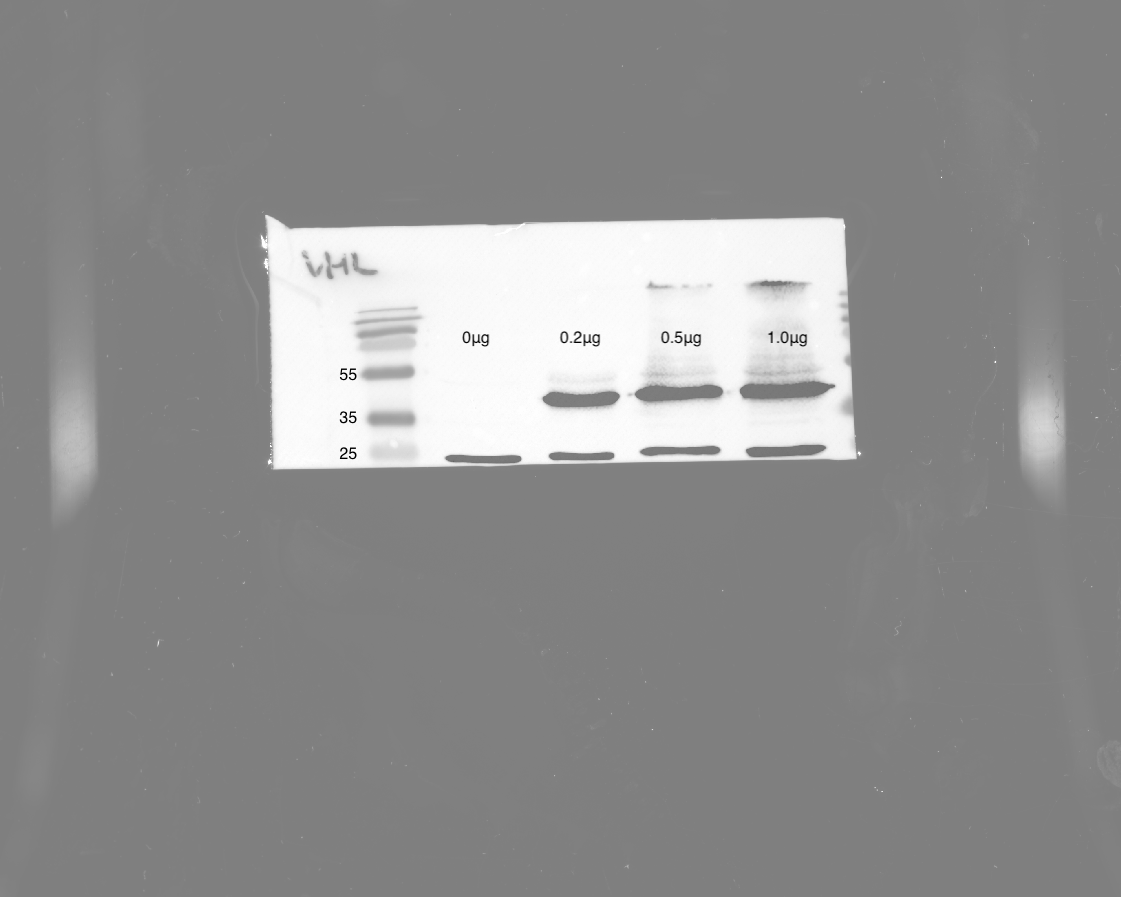

Supplement: Figure 1—figure supplement 1—source data 1. [file elife-106699-fig1-figsupp1-data1.zip › Figure 1ΓÇöfigure supplement 1-source data 1 Western blot data with label shows LMO2 protein degradation in HEK293T cells with different biodegrader contruct./Raw data/VHL VHL-iDab(Composite).tif]

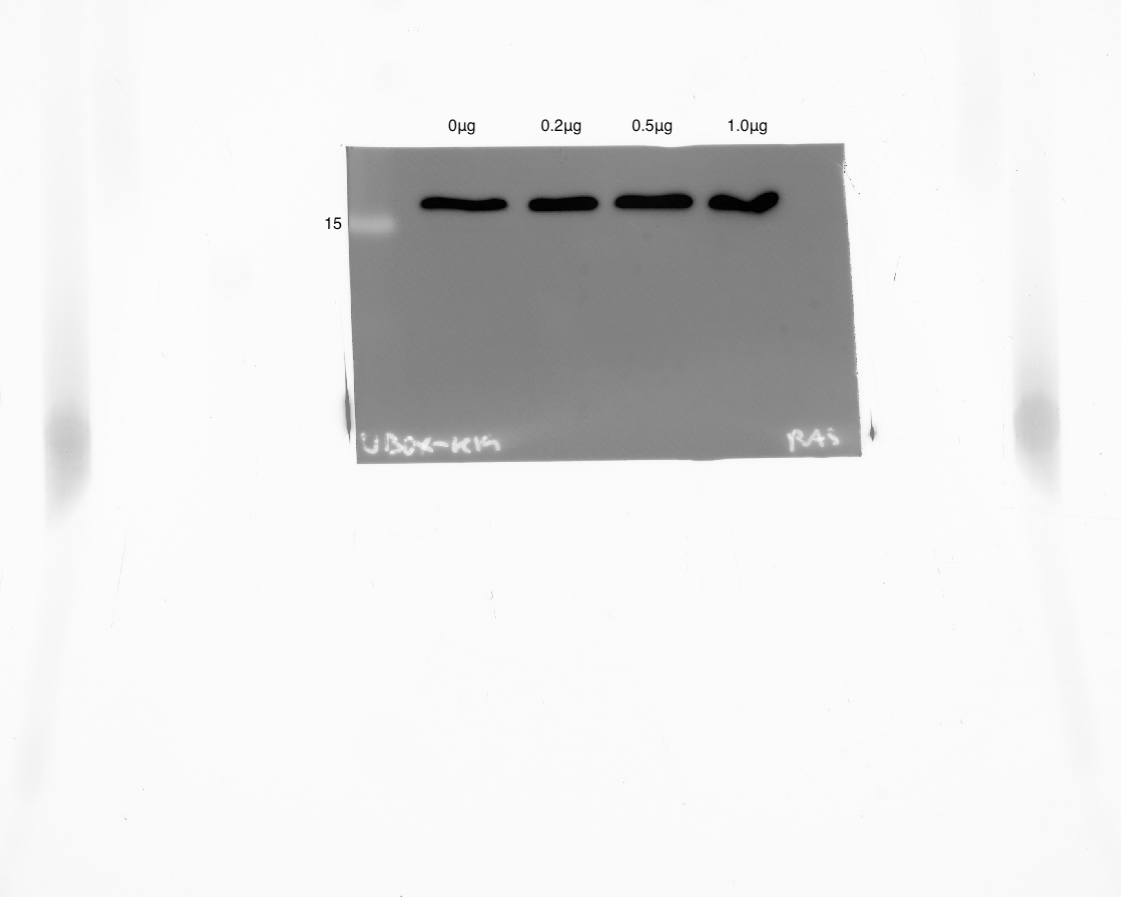

Supplement: Figure 1—figure supplement 1—source data 1. [file elife-106699-fig1-figsupp1-data1.zip › Figure 1ΓÇöfigure supplement 1-source data 1 Western blot data with label shows LMO2 protein degradation in HEK293T cells with different biodegrader contruct./Raw data/Cyclophilin VHL-iDabRas(Composite).tif]

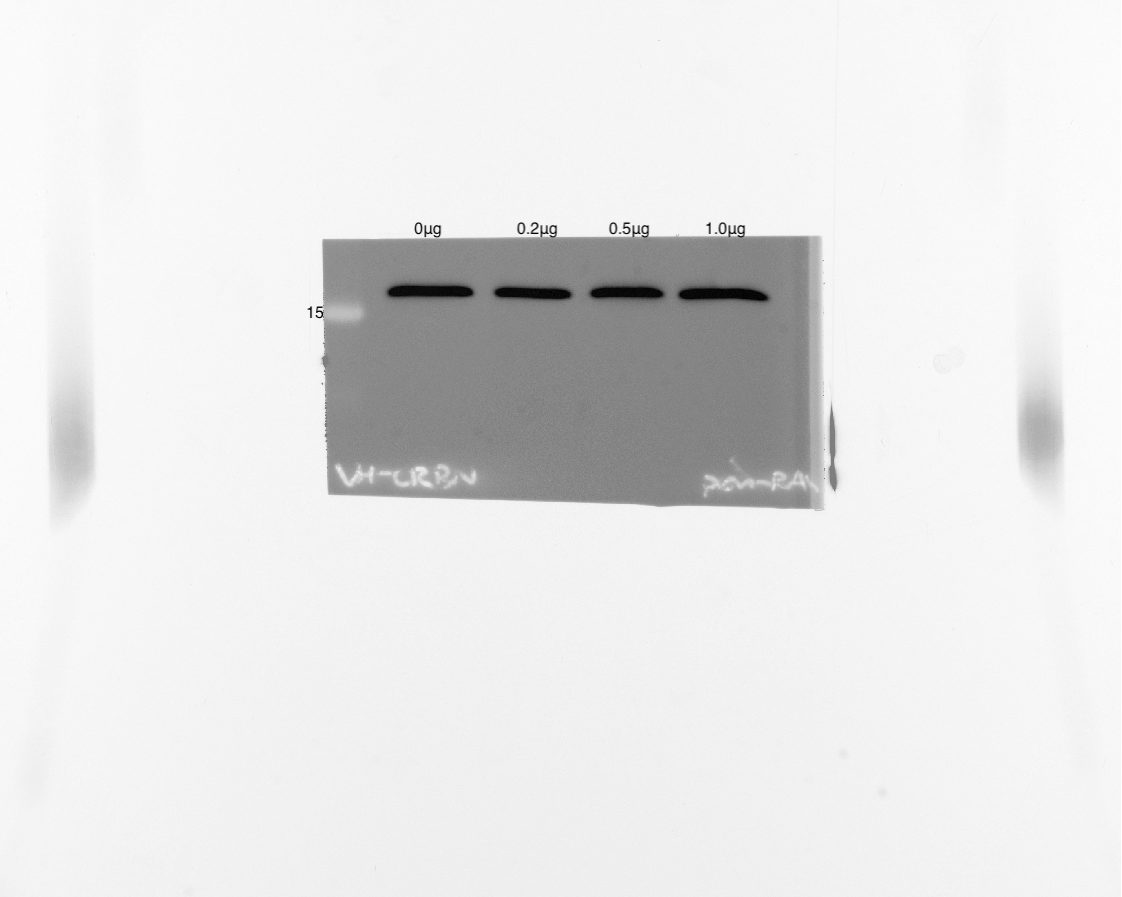

Supplement: Figure 1—figure supplement 1—source data 1. [file elife-106699-fig1-figsupp1-data1.zip › Figure 1ΓÇöfigure supplement 1-source data 1 Western blot data with label shows LMO2 protein degradation in HEK293T cells with different biodegrader contruct./Raw data/Cyclophilin iDab-CRBN(Composite).tif]

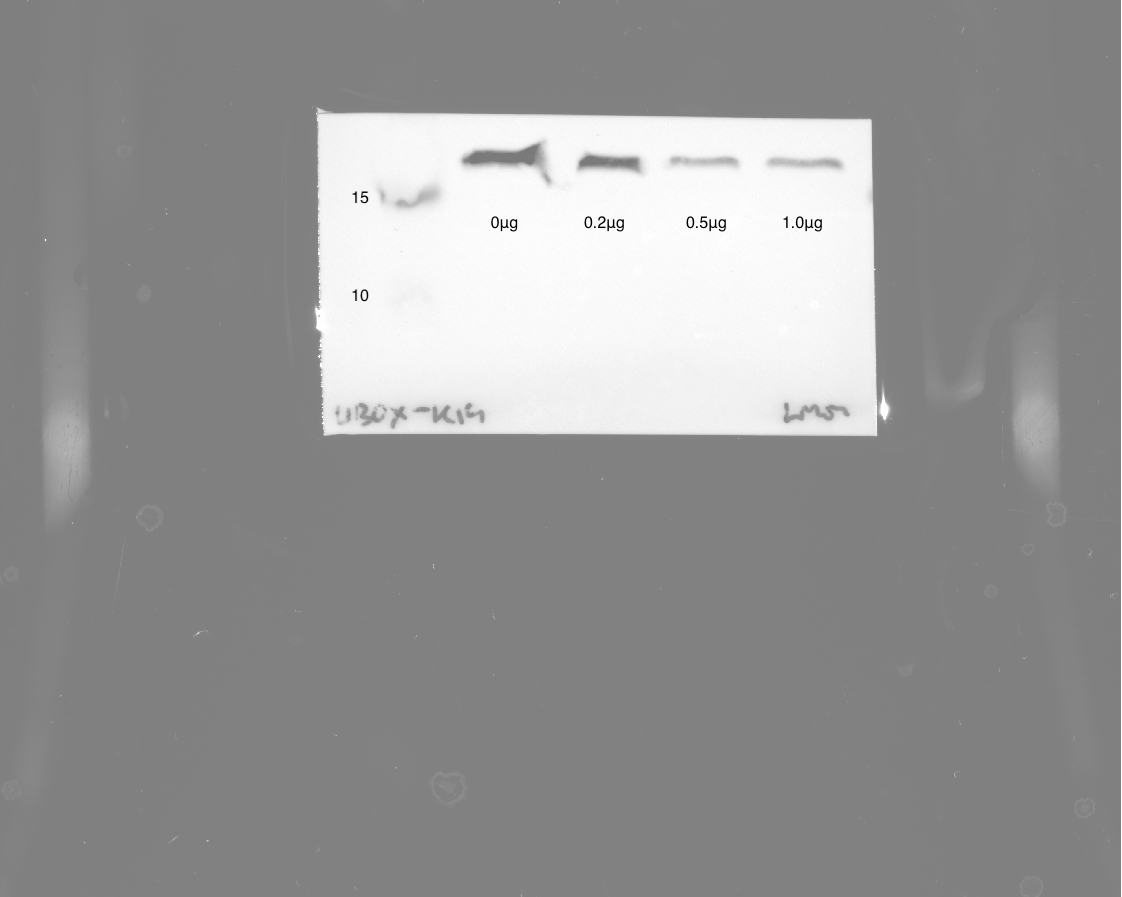

Supplement: Figure 1—figure supplement 1—source data 1. [file elife-106699-fig1-figsupp1-data1.zip › Figure 1ΓÇöfigure supplement 1-source data 1 Western blot data with label shows LMO2 protein degradation in HEK293T cells with different biodegrader contruct./Raw data/panRAS UBOX-iDabRas(Composite).tif]

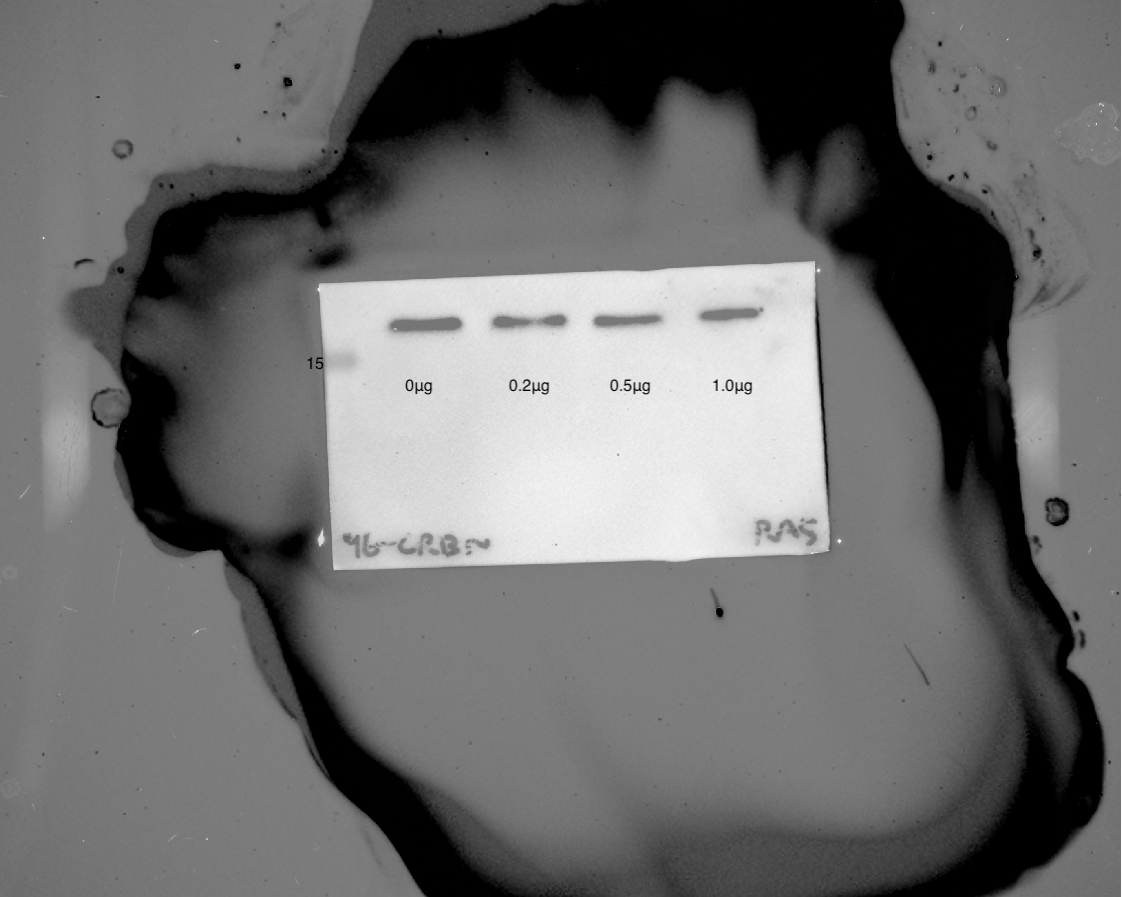

Supplement: Figure 1—figure supplement 1—source data 1. [file elife-106699-fig1-figsupp1-data1.zip › Figure 1ΓÇöfigure supplement 1-source data 1 Western blot data with label shows LMO2 protein degradation in HEK293T cells with different biodegrader contruct./Raw data/pan-RAS iDabRas-CRBN(Composite).tif]

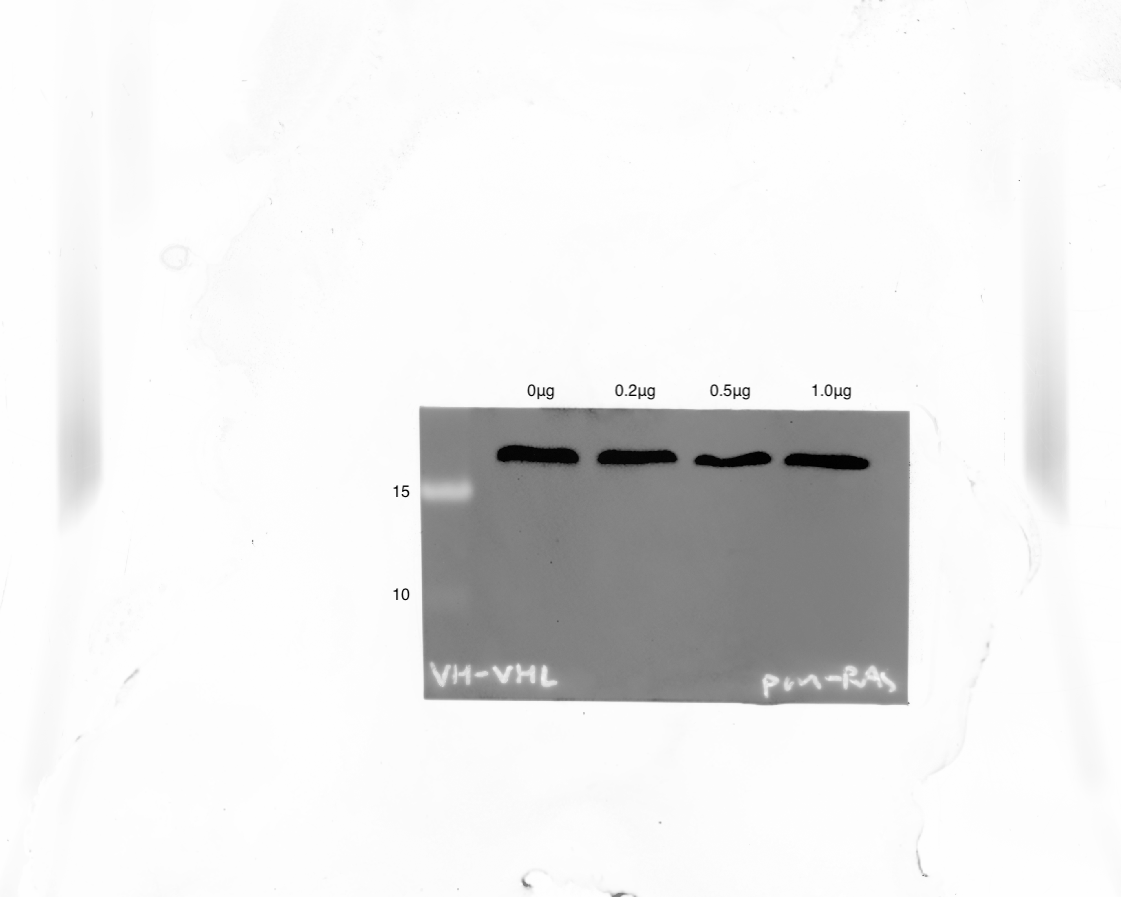

Supplement: Figure 1—figure supplement 1—source data 1. [file elife-106699-fig1-figsupp1-data1.zip › Figure 1ΓÇöfigure supplement 1-source data 1 Western blot data with label shows LMO2 protein degradation in HEK293T cells with different biodegrader contruct./Raw data/panRAS iDab-VHL(Composite).tif]

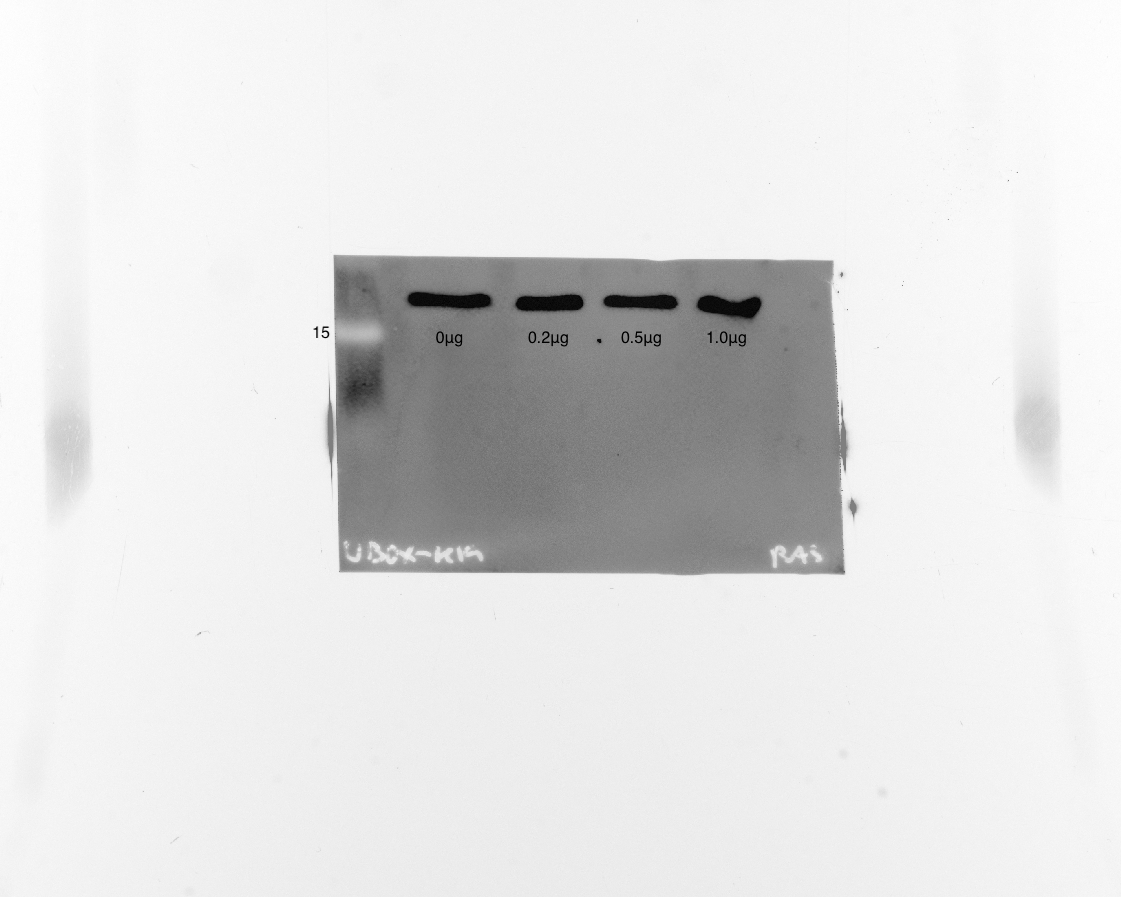

Supplement: Figure 1—figure supplement 1—source data 1. [file elife-106699-fig1-figsupp1-data1.zip › Figure 1ΓÇöfigure supplement 1-source data 1 Western blot data with label shows LMO2 protein degradation in HEK293T cells with different biodegrader contruct./Raw data/LMO2 UBOX-iDab-Ras(Composite).tif]

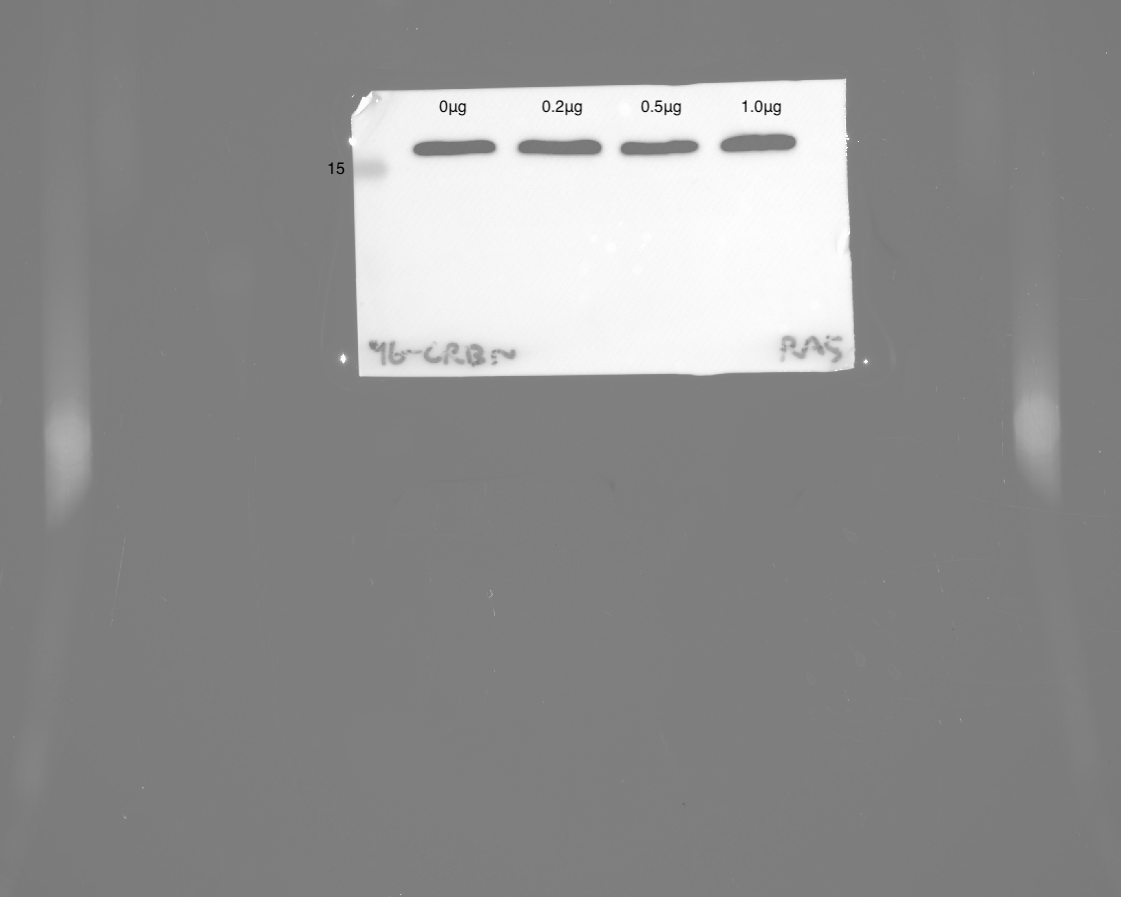

Supplement: Figure 1—figure supplement 1—source data 1. [file elife-106699-fig1-figsupp1-data1.zip › Figure 1ΓÇöfigure supplement 1-source data 1 Western blot data with label shows LMO2 protein degradation in HEK293T cells with different biodegrader contruct./Raw data/LMO2 VHL-iDabRas.tif]

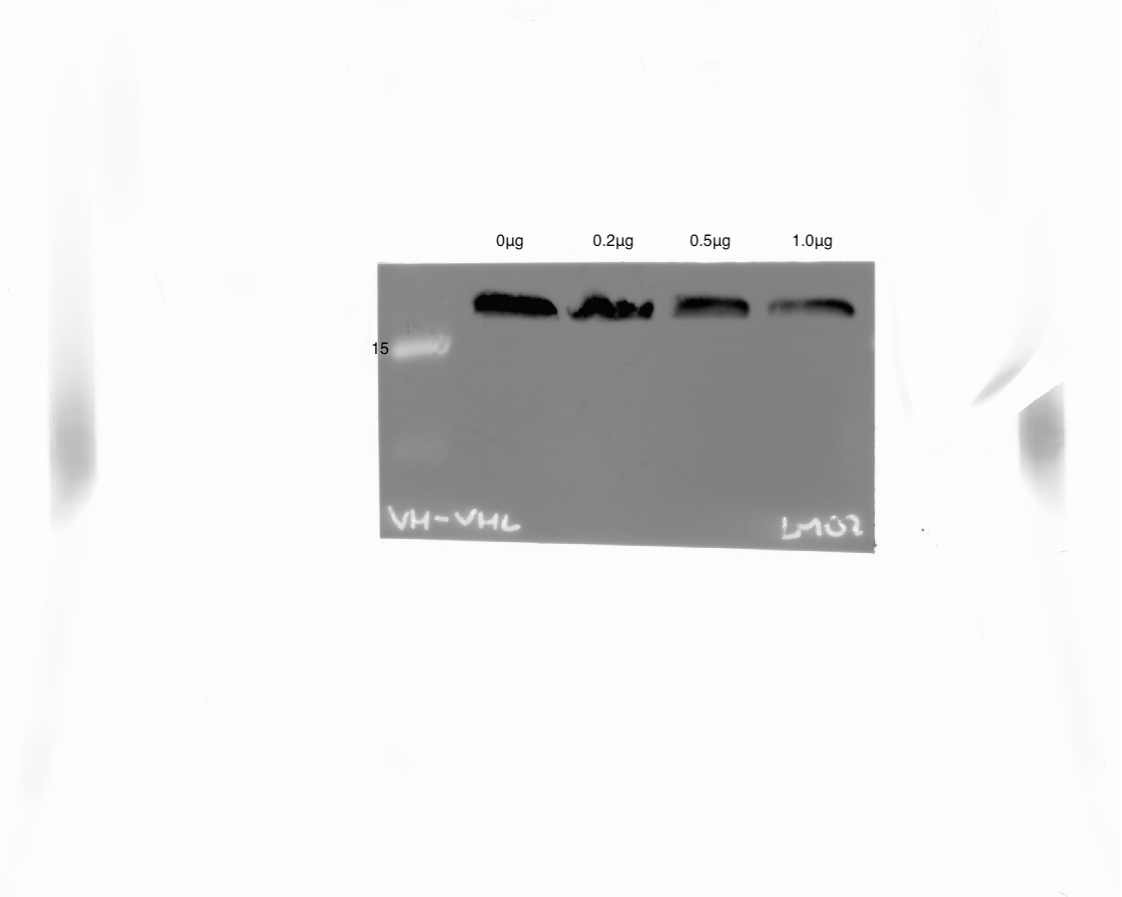

Supplement: Figure 1—figure supplement 1—source data 1. [file elife-106699-fig1-figsupp1-data1.zip › Figure 1ΓÇöfigure supplement 1-source data 1 Western blot data with label shows LMO2 protein degradation in HEK293T cells with different biodegrader contruct./Raw data/LMO2 iDab-VHL(Composite).tif]

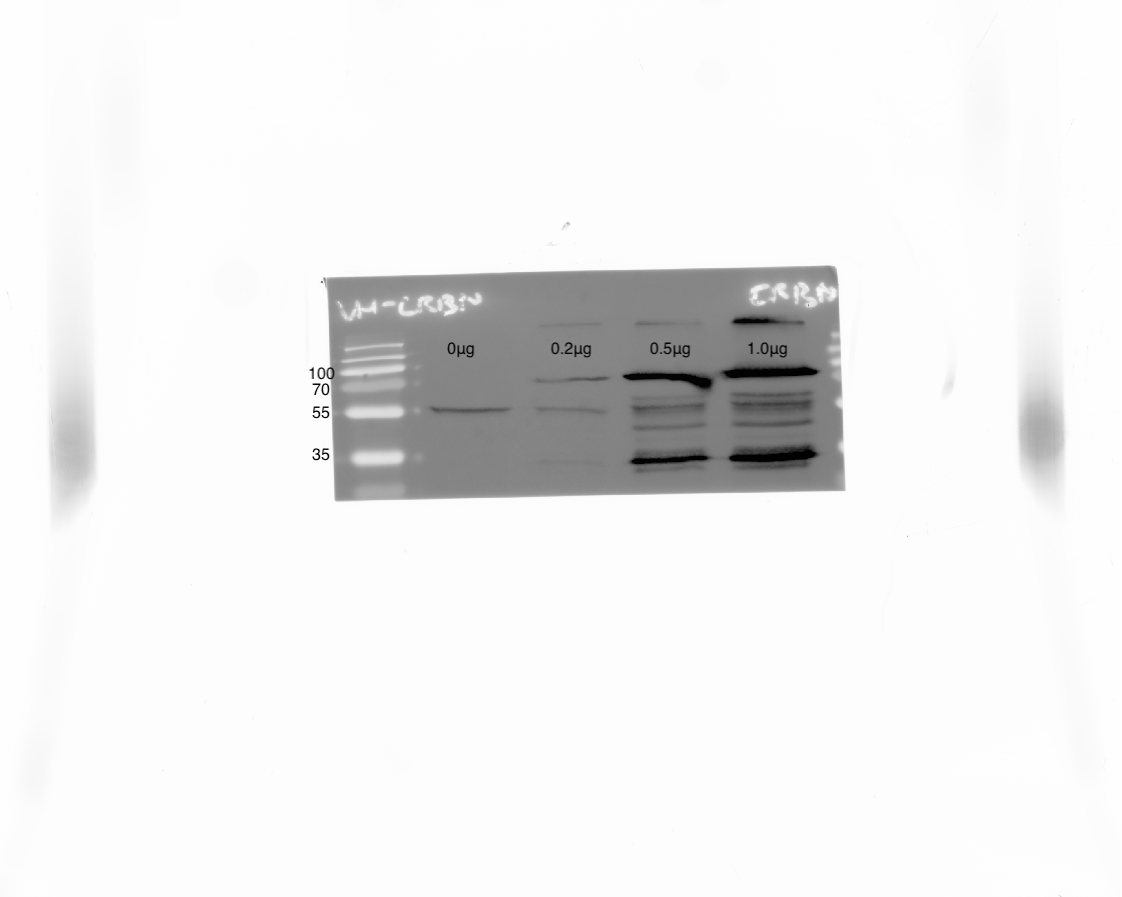

Supplement: Figure 1—figure supplement 1—source data 1. [file elife-106699-fig1-figsupp1-data1.zip › Figure 1ΓÇöfigure supplement 1-source data 1 Western blot data with label shows LMO2 protein degradation in HEK293T cells with different biodegrader contruct./Raw data/CRBN iDab-CRBN(Composite).tif]

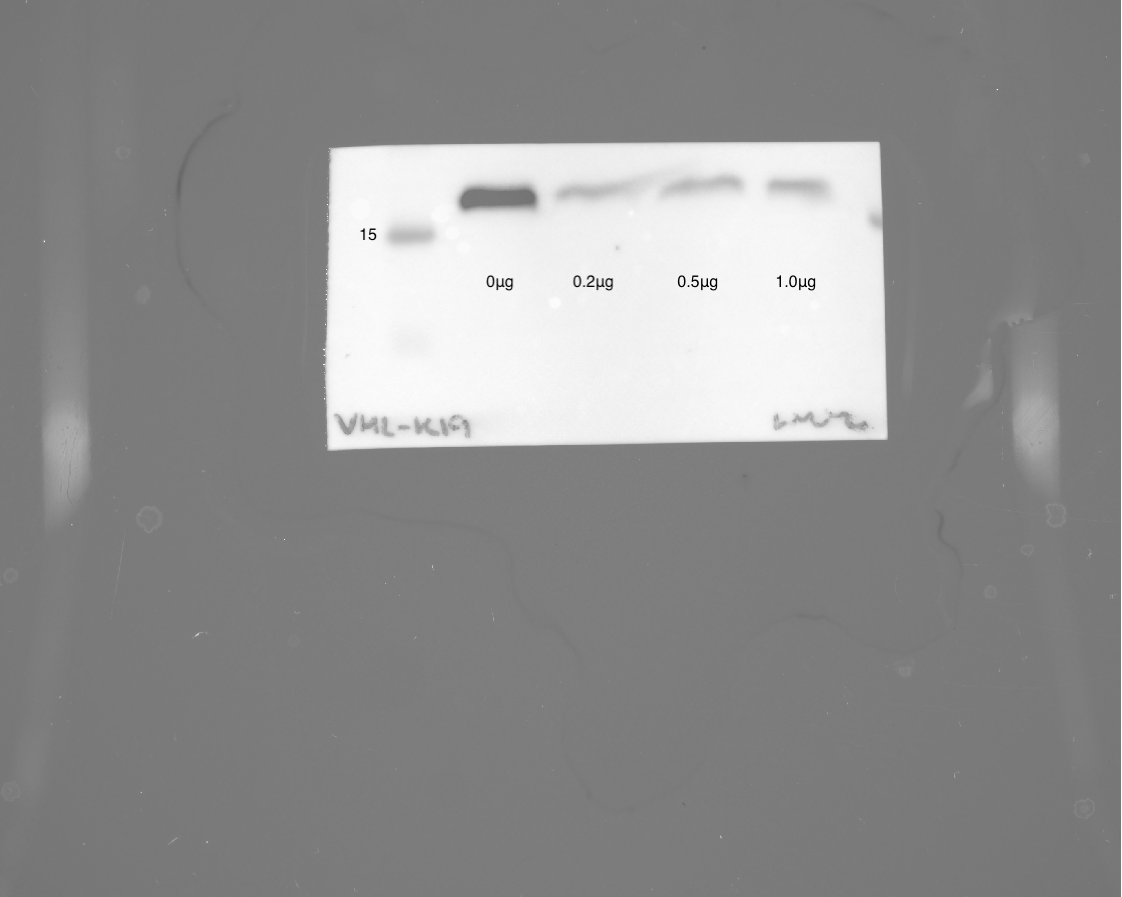

Supplement: Figure 1—figure supplement 1—source data 1. [file elife-106699-fig1-figsupp1-data1.zip › Figure 1ΓÇöfigure supplement 1-source data 1 Western blot data with label shows LMO2 protein degradation in HEK293T cells with different biodegrader contruct./Raw data/panRAS VHL-iDabRas(Composite).tif]

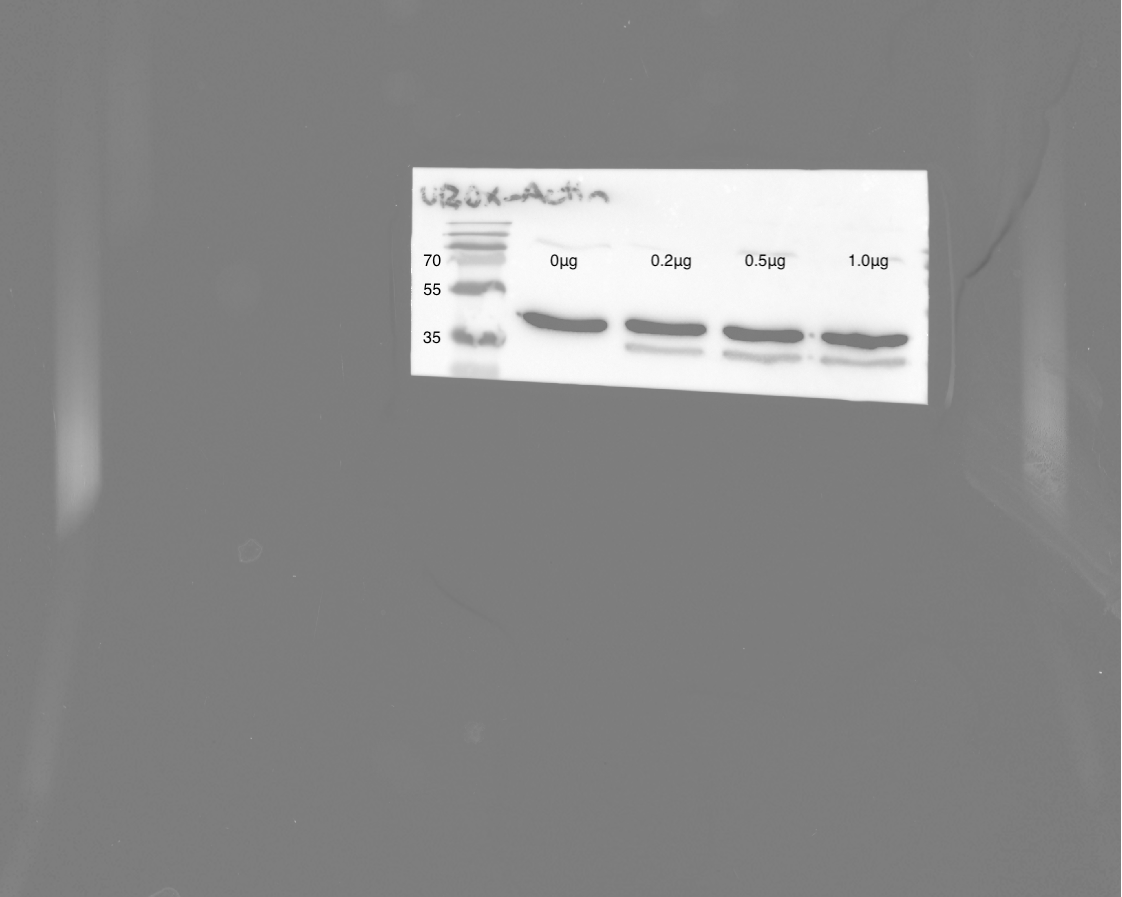

Supplement: Figure 1—figure supplement 1—source data 1. [file elife-106699-fig1-figsupp1-data1.zip › Figure 1ΓÇöfigure supplement 1-source data 1 Western blot data with label shows LMO2 protein degradation in HEK293T cells with different biodegrader contruct./Raw data/Actin iDab-UBOX(Composite).tif]

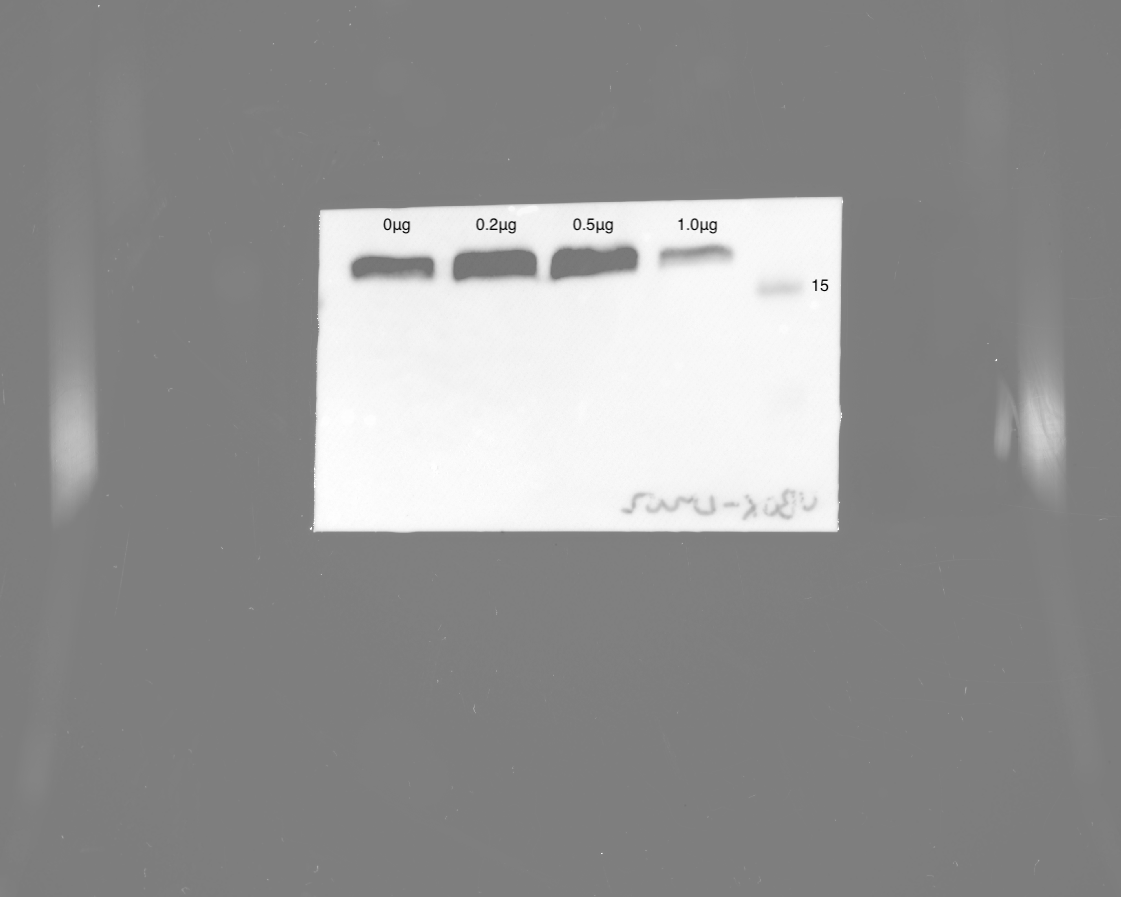

Supplement: Figure 1—figure supplement 1—source data 1. [file elife-106699-fig1-figsupp1-data1.zip › Figure 1ΓÇöfigure supplement 1-source data 1 Western blot data with label shows LMO2 protein degradation in HEK293T cells with different biodegrader contruct./Raw data/LMO2 iDab-UBOX(Composite).tif]

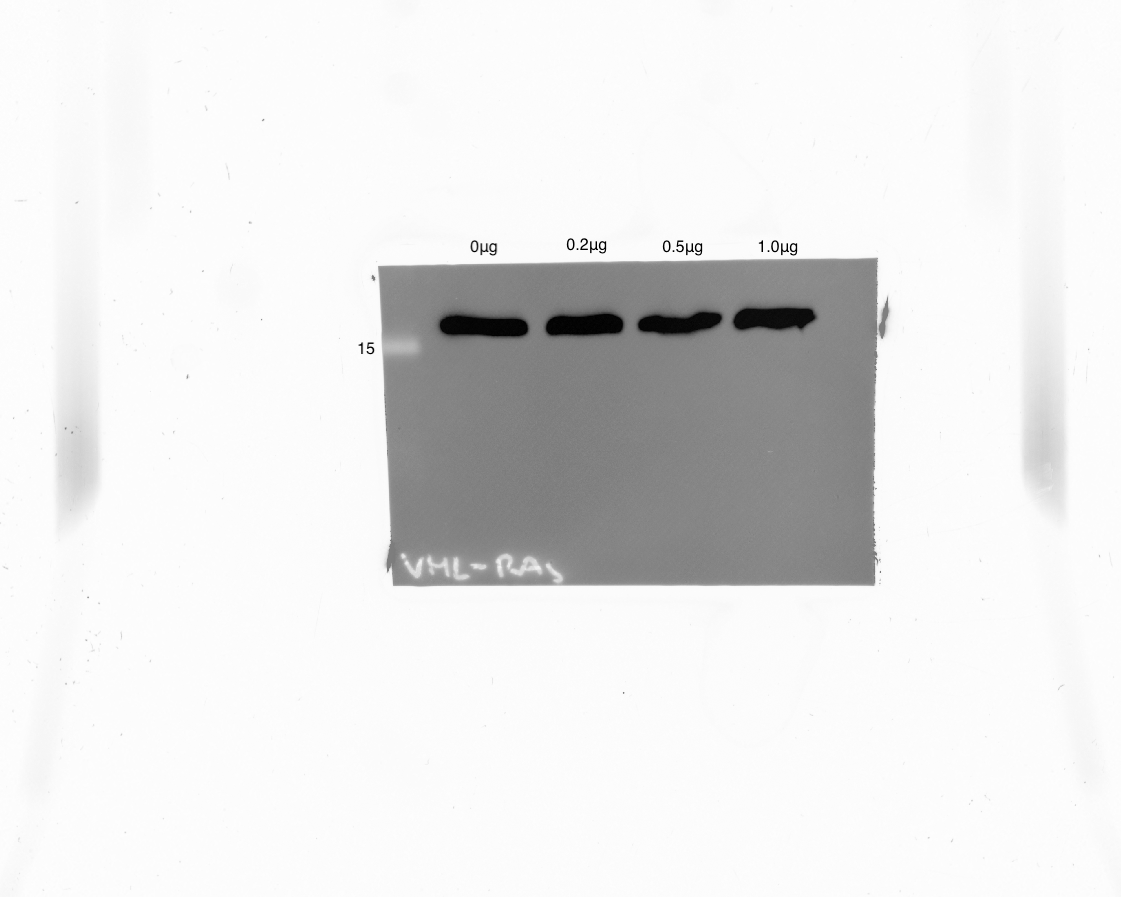

Supplement: Figure 1—figure supplement 1—source data 1. [file elife-106699-fig1-figsupp1-data1.zip › Figure 1ΓÇöfigure supplement 1-source data 1 Western blot data with label shows LMO2 protein degradation in HEK293T cells with different biodegrader contruct./Raw data/Cyclophilin VHL-iDab(Composite).tif]

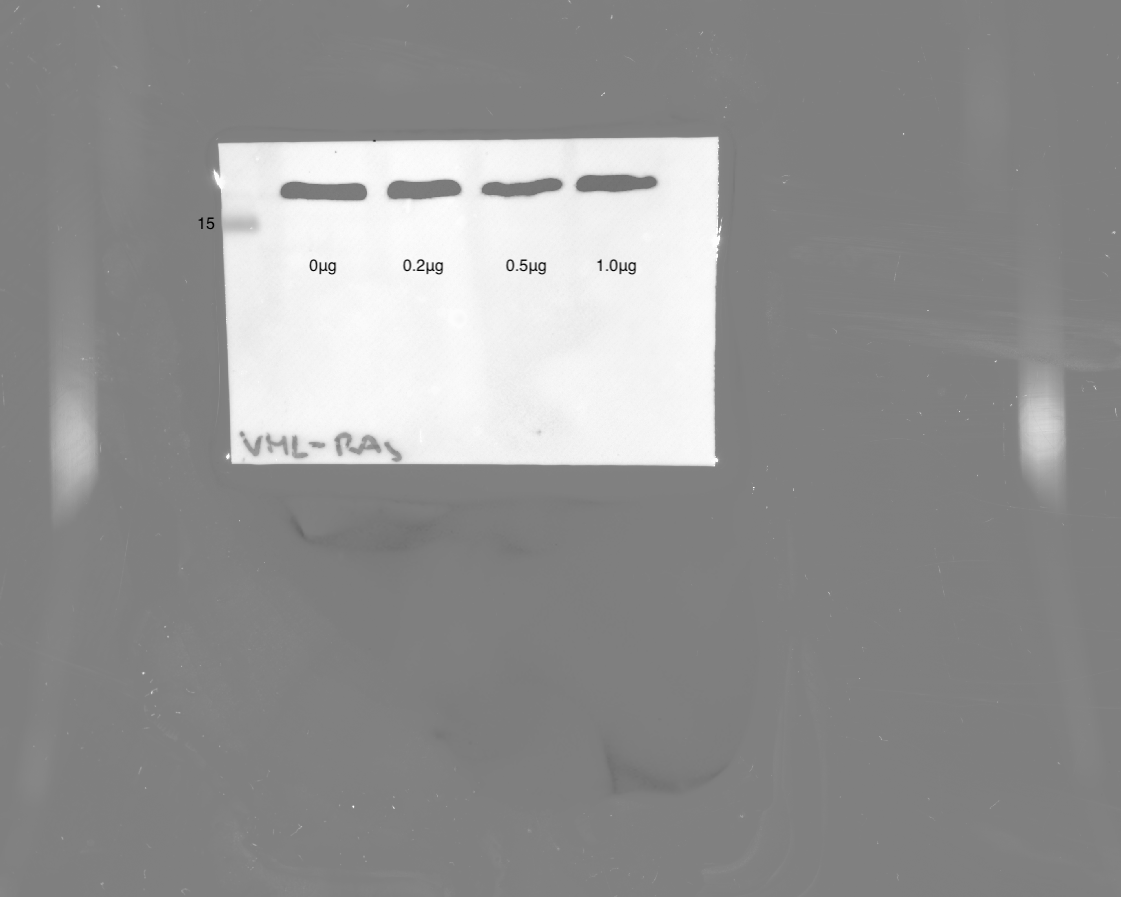

Supplement: Figure 1—figure supplement 1—source data 1. [file elife-106699-fig1-figsupp1-data1.zip › Figure 1ΓÇöfigure supplement 1-source data 1 Western blot data with label shows LMO2 protein degradation in HEK293T cells with different biodegrader contruct./Raw data/pan-RAS VHL-iDab(Composite).tif]

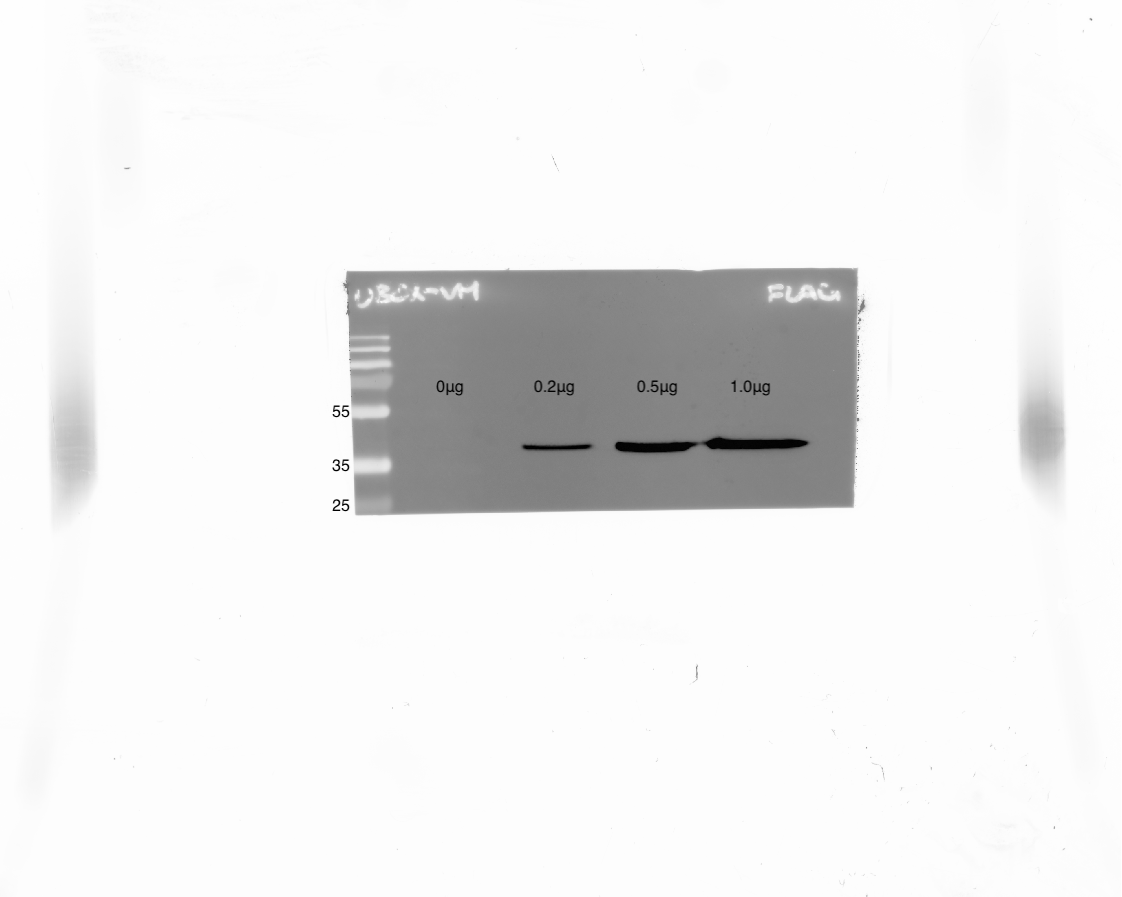

Supplement: Figure 1—figure supplement 1—source data 1. [file elife-106699-fig1-figsupp1-data1.zip › Figure 1ΓÇöfigure supplement 1-source data 1 Western blot data with label shows LMO2 protein degradation in HEK293T cells with different biodegrader contruct./Raw data/FLAG UBOX-iDab(Composite).tif]

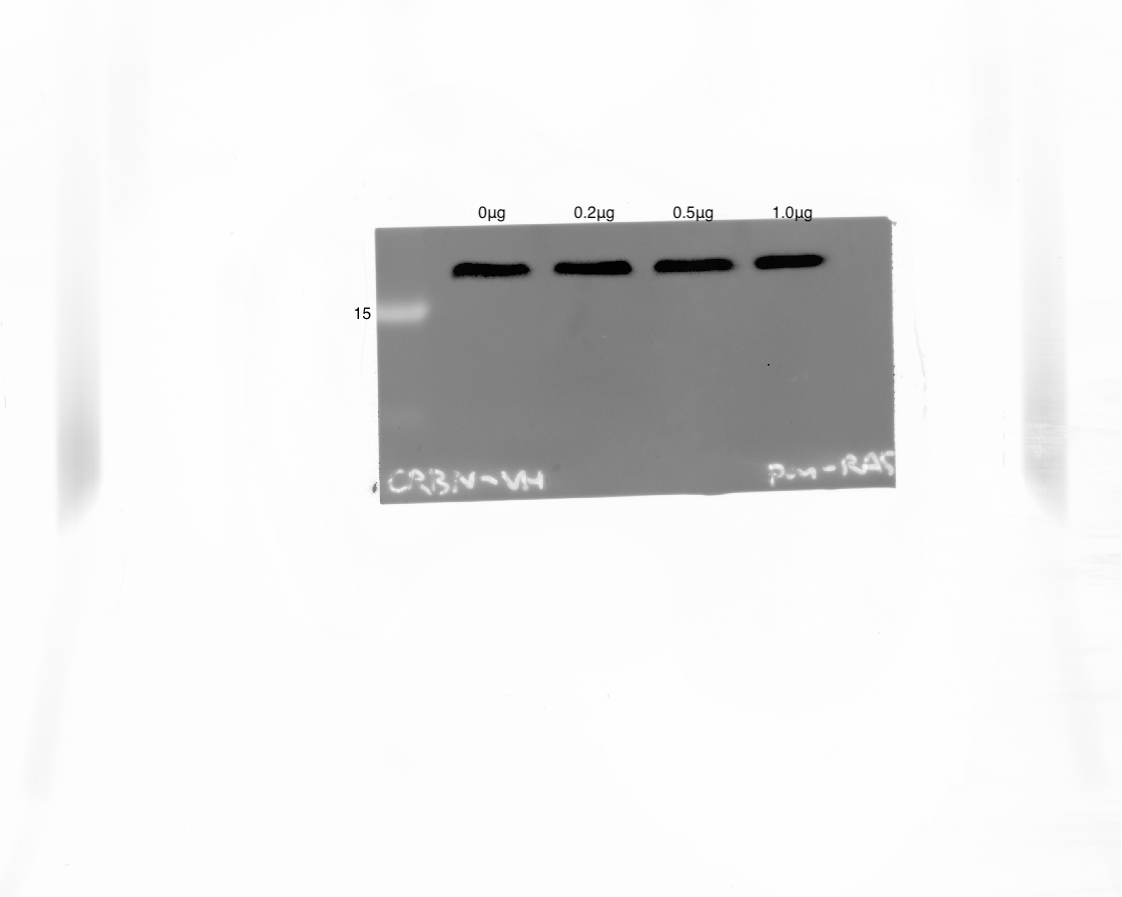

Supplement: Figure 1—figure supplement 1—source data 1. [file elife-106699-fig1-figsupp1-data1.zip › Figure 1ΓÇöfigure supplement 1-source data 1 Western blot data with label shows LMO2 protein degradation in HEK293T cells with different biodegrader contruct./Raw data/panRAS CRBN-iDab(Composite).tif]

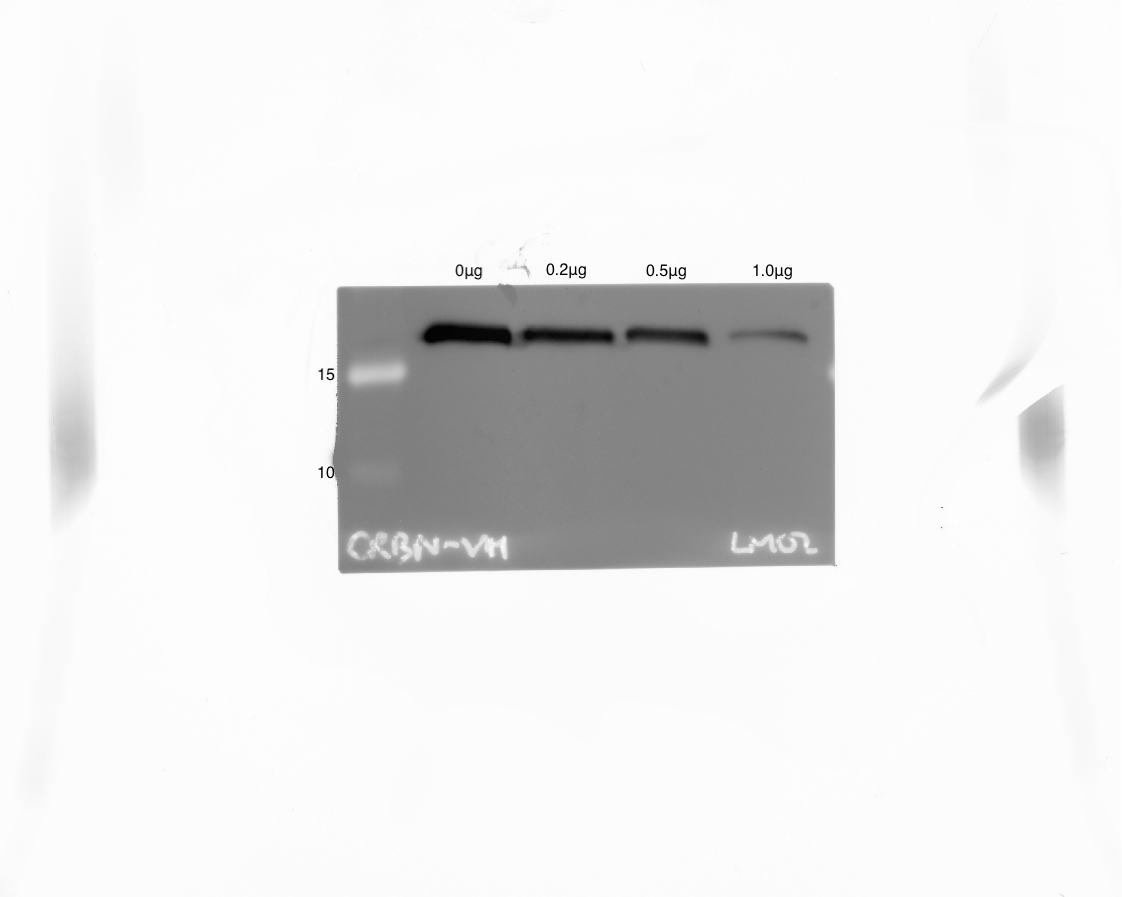

Supplement: Figure 1—figure supplement 1—source data 1. [file elife-106699-fig1-figsupp1-data1.zip › Figure 1ΓÇöfigure supplement 1-source data 1 Western blot data with label shows LMO2 protein degradation in HEK293T cells with different biodegrader contruct./Raw data/LMO2 CRBN-iDab(Composite).tif]

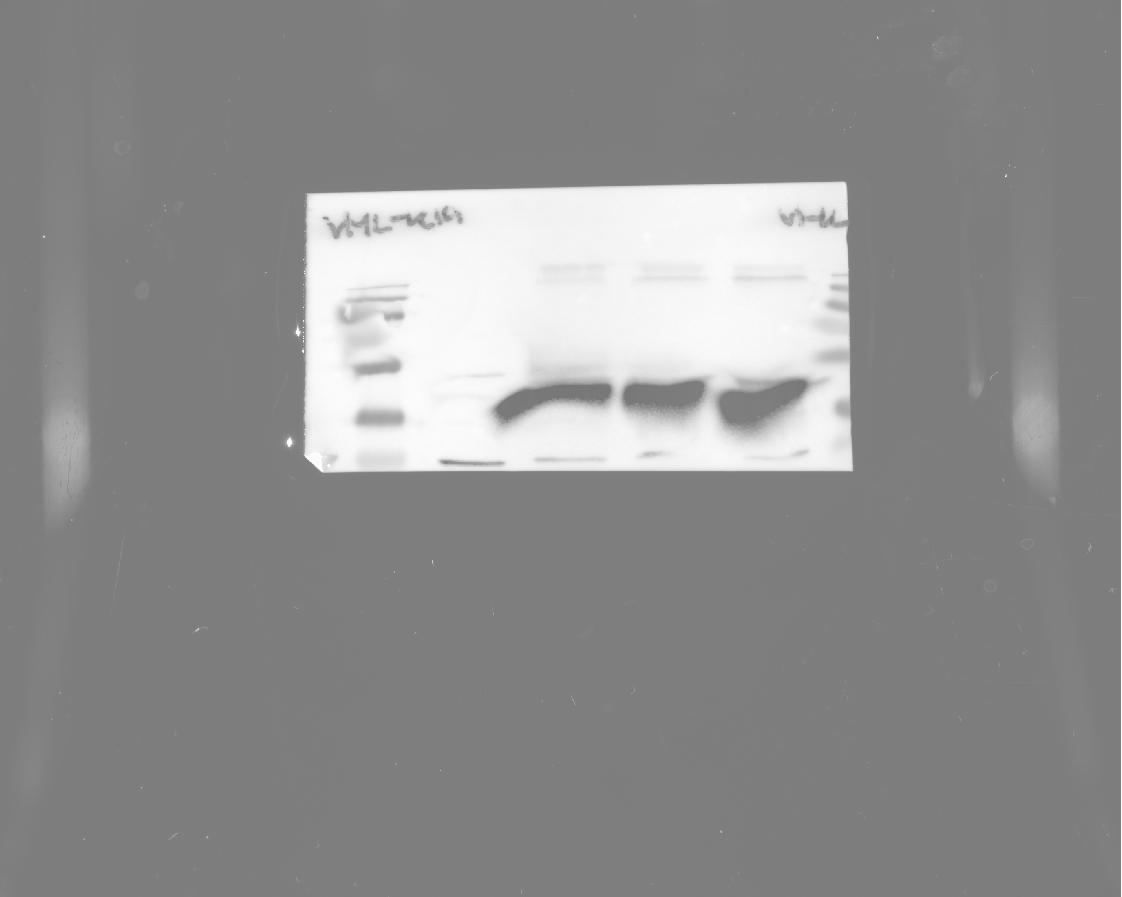

Supplement: Figure 1—figure supplement 1—source data 2. [file elife-106699-fig1-figsupp1-data2.zip › Figure 1ΓÇöfigure supplement 1-source data 2 Western blot raw data shows LMO2 protein degradation in HEK293T cells with different biodegrader contruct./VHL VHL-iDabRas(Composite).tif]

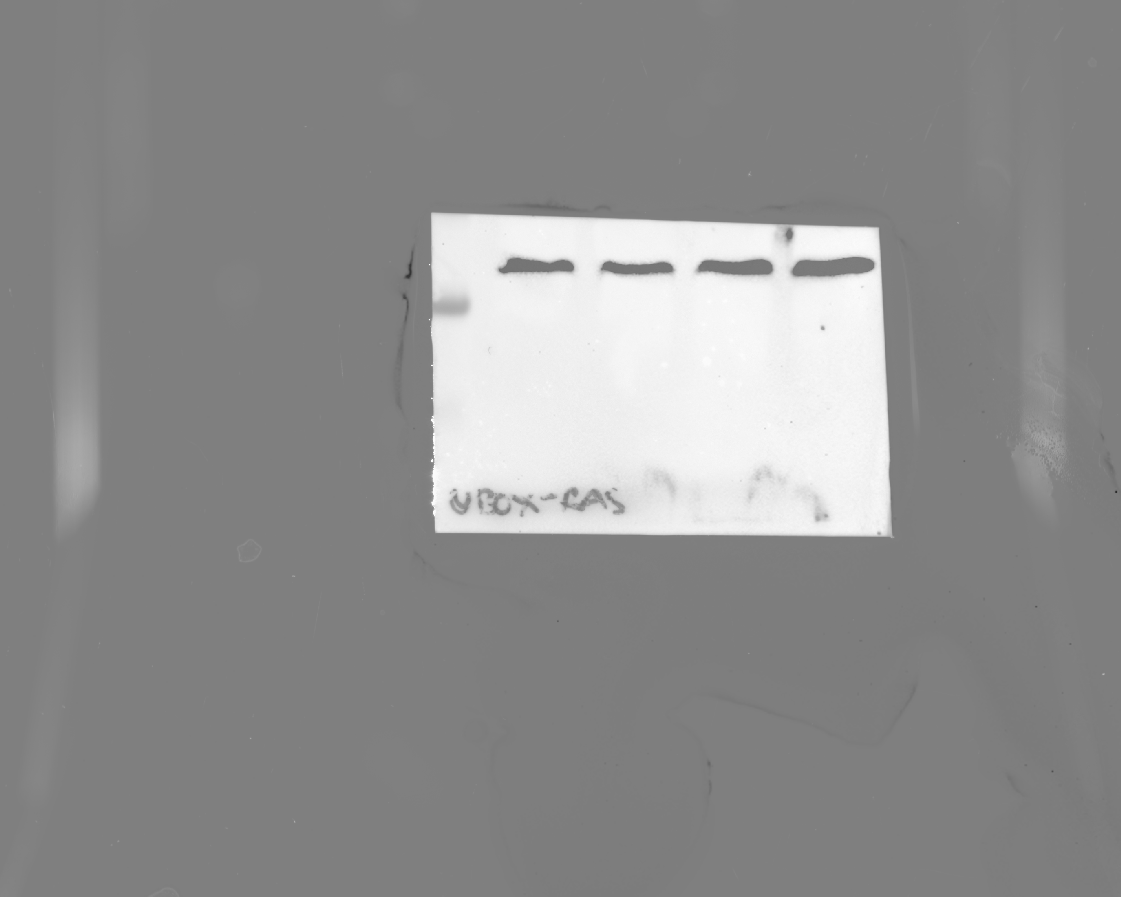

Supplement: Figure 1—figure supplement 1—source data 2. [file elife-106699-fig1-figsupp1-data2.zip › Figure 1ΓÇöfigure supplement 1-source data 2 Western blot raw data shows LMO2 protein degradation in HEK293T cells with different biodegrader contruct./pan-RAS iDab-UBOX(Composite).tif]

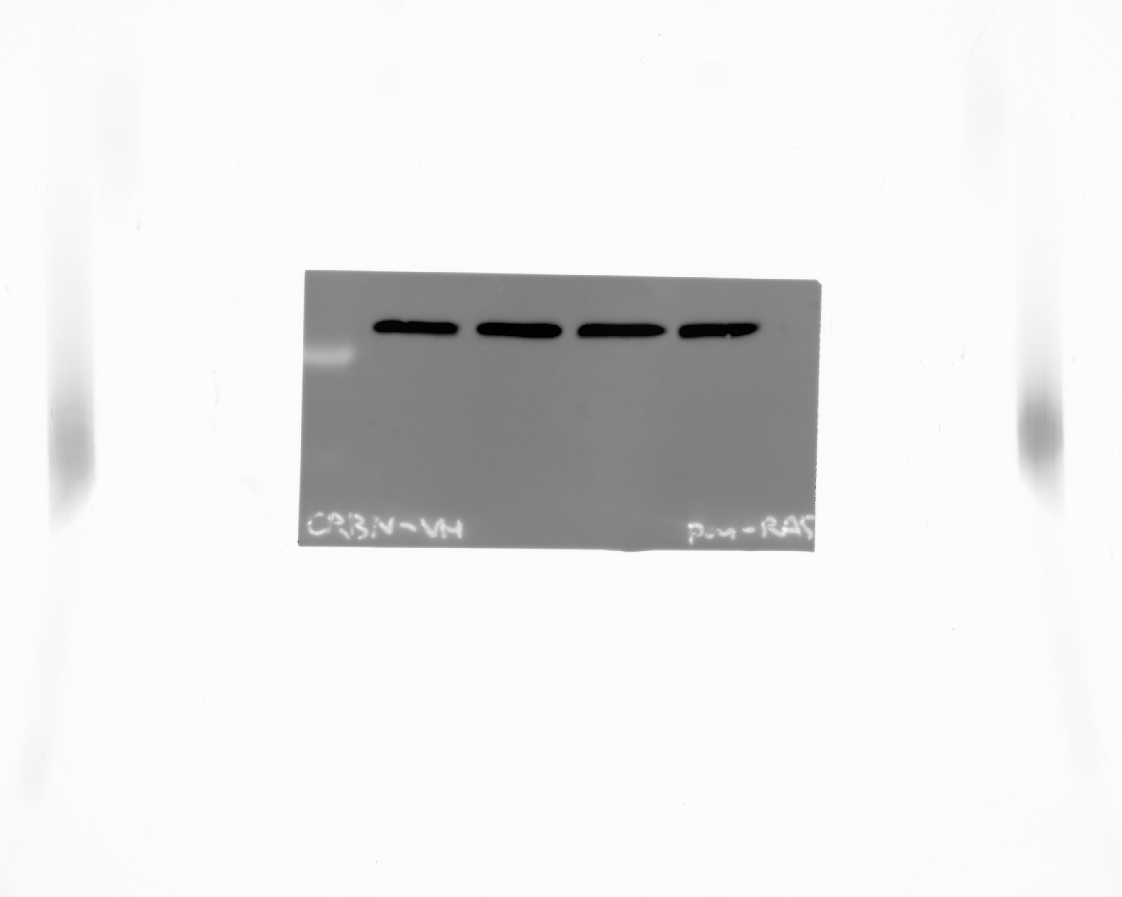

Supplement: Figure 1—figure supplement 1—source data 2. [file elife-106699-fig1-figsupp1-data2.zip › Figure 1ΓÇöfigure supplement 1-source data 2 Western blot raw data shows LMO2 protein degradation in HEK293T cells with different biodegrader contruct./Cyclophilin CRBN-iDab(Composite).tif]

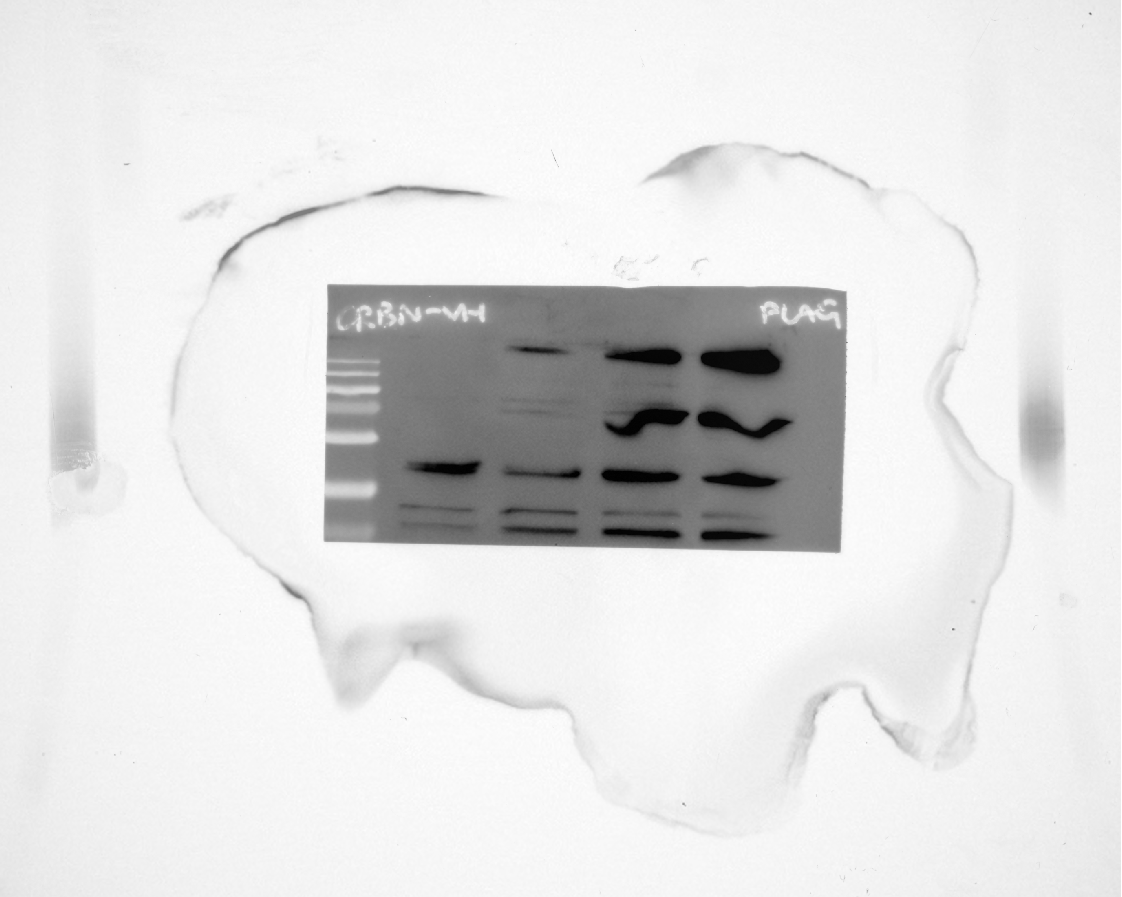

Supplement: Figure 1—figure supplement 1—source data 2. [file elife-106699-fig1-figsupp1-data2.zip › Figure 1ΓÇöfigure supplement 1-source data 2 Western blot raw data shows LMO2 protein degradation in HEK293T cells with different biodegrader contruct./FLAG CRBN-iDab(Composite).tif]

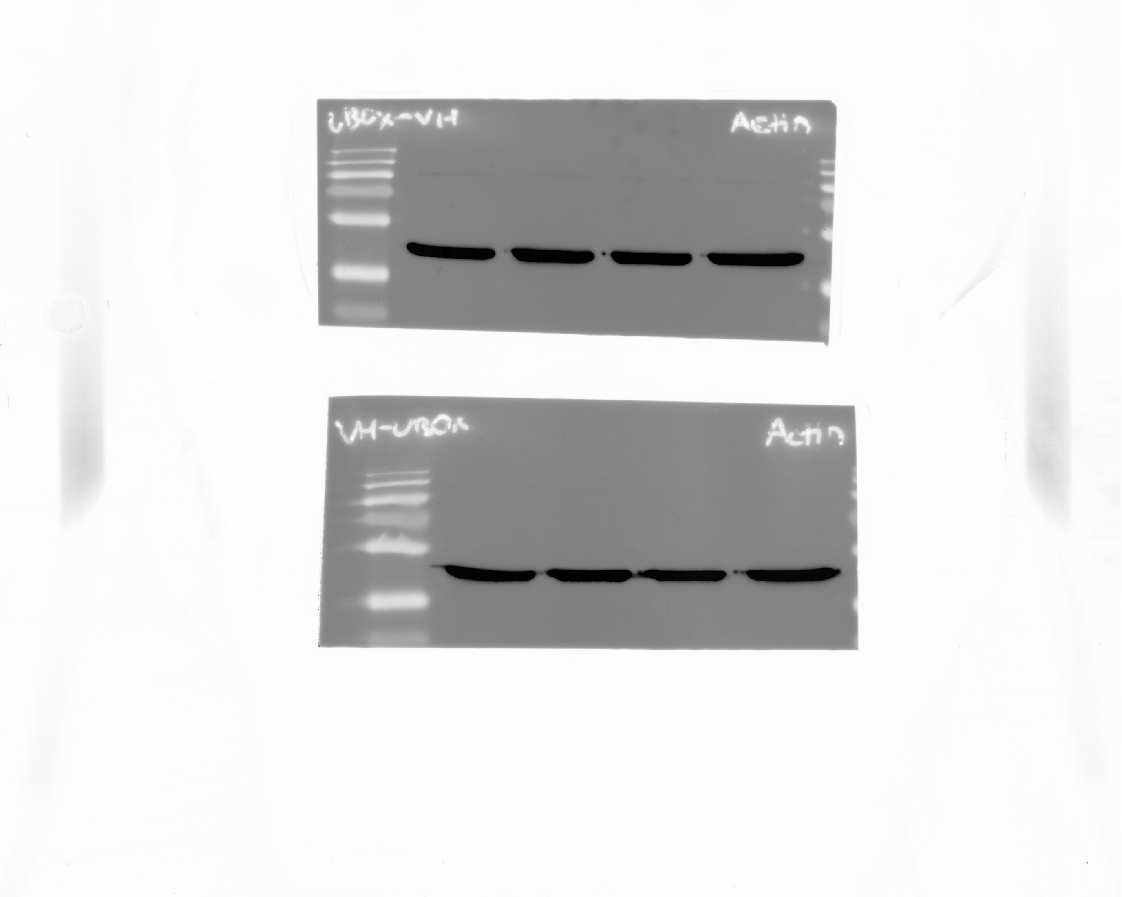

Supplement: Figure 1—figure supplement 1—source data 2. [file elife-106699-fig1-figsupp1-data2.zip › Figure 1ΓÇöfigure supplement 1-source data 2 Western blot raw data shows LMO2 protein degradation in HEK293T cells with different biodegrader contruct./Actin UBOX-iDab AND iDab-UBOX(Composite).tif]

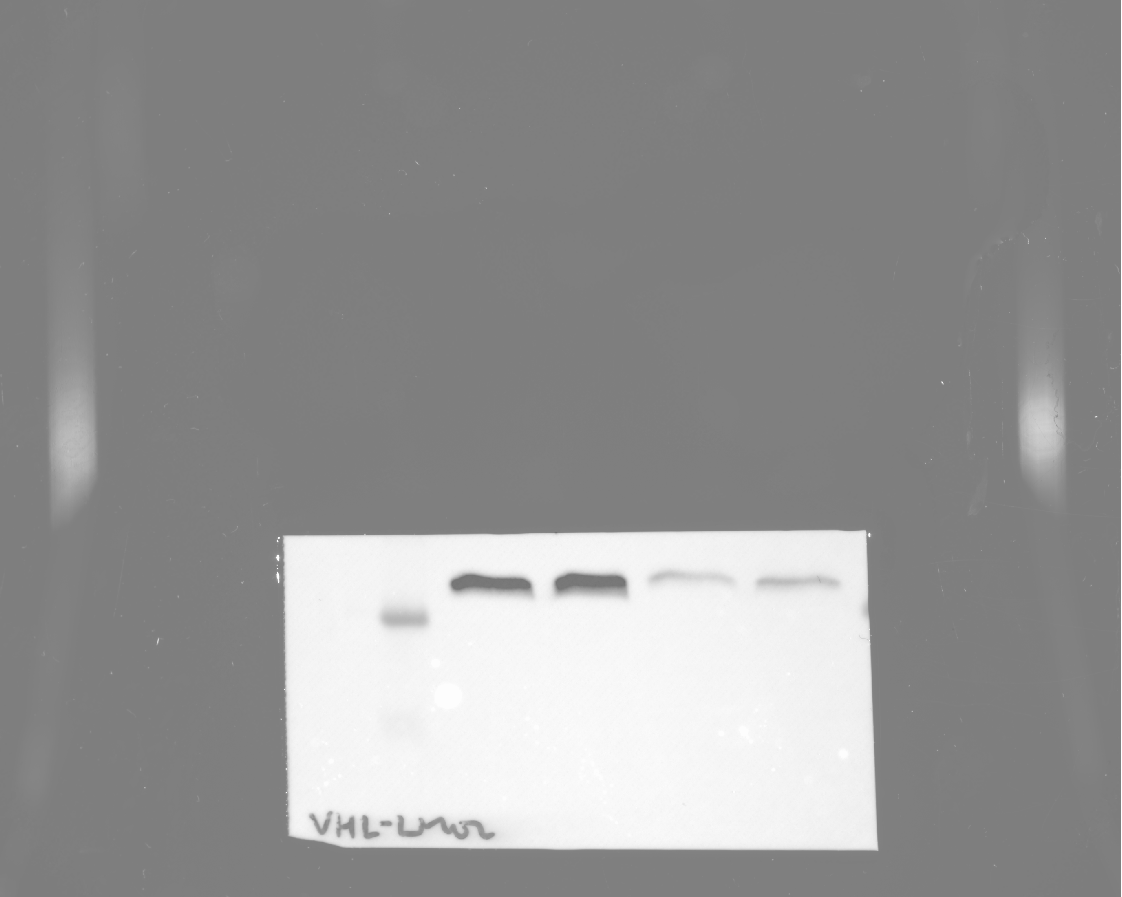

Supplement: Figure 1—figure supplement 1—source data 2. [file elife-106699-fig1-figsupp1-data2.zip › Figure 1ΓÇöfigure supplement 1-source data 2 Western blot raw data shows LMO2 protein degradation in HEK293T cells with different biodegrader contruct./LMO2 VHL-iDab(Composite).tif]

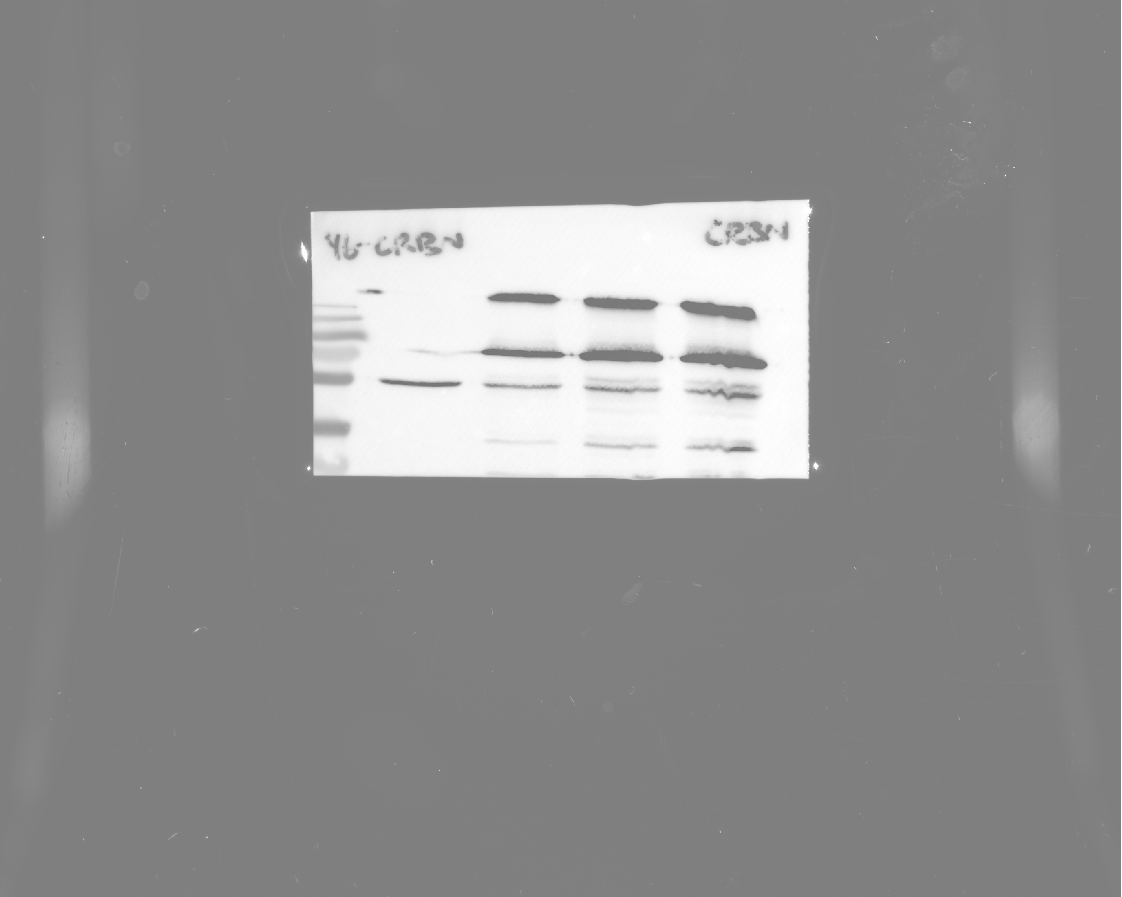

Supplement: Figure 1—figure supplement 1—source data 2. [file elife-106699-fig1-figsupp1-data2.zip › Figure 1ΓÇöfigure supplement 1-source data 2 Western blot raw data shows LMO2 protein degradation in HEK293T cells with different biodegrader contruct./CRBN iDabRas-CRBN(Composite).tif]

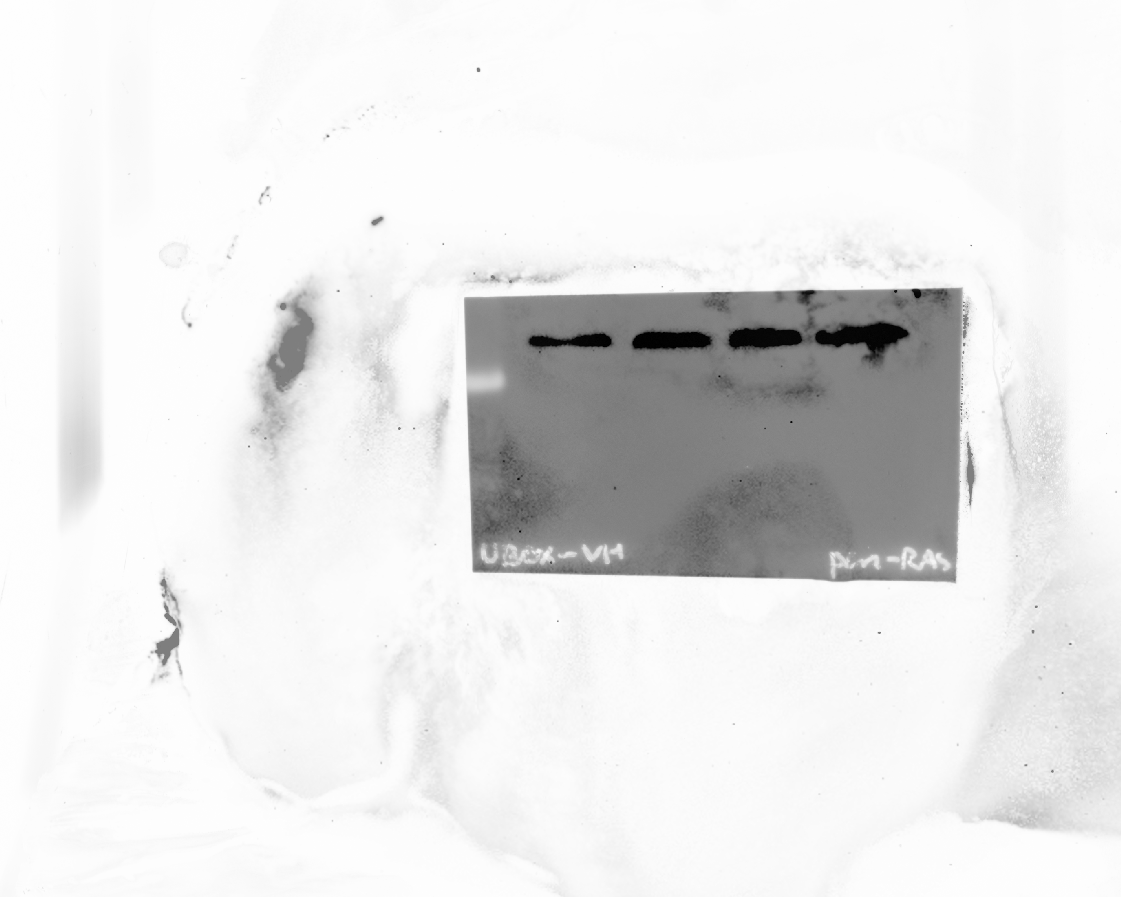

Supplement: Figure 1—figure supplement 1—source data 2. [file elife-106699-fig1-figsupp1-data2.zip › Figure 1ΓÇöfigure supplement 1-source data 2 Western blot raw data shows LMO2 protein degradation in HEK293T cells with different biodegrader contruct./panRAS UBOX-iDab(Composite).tif]

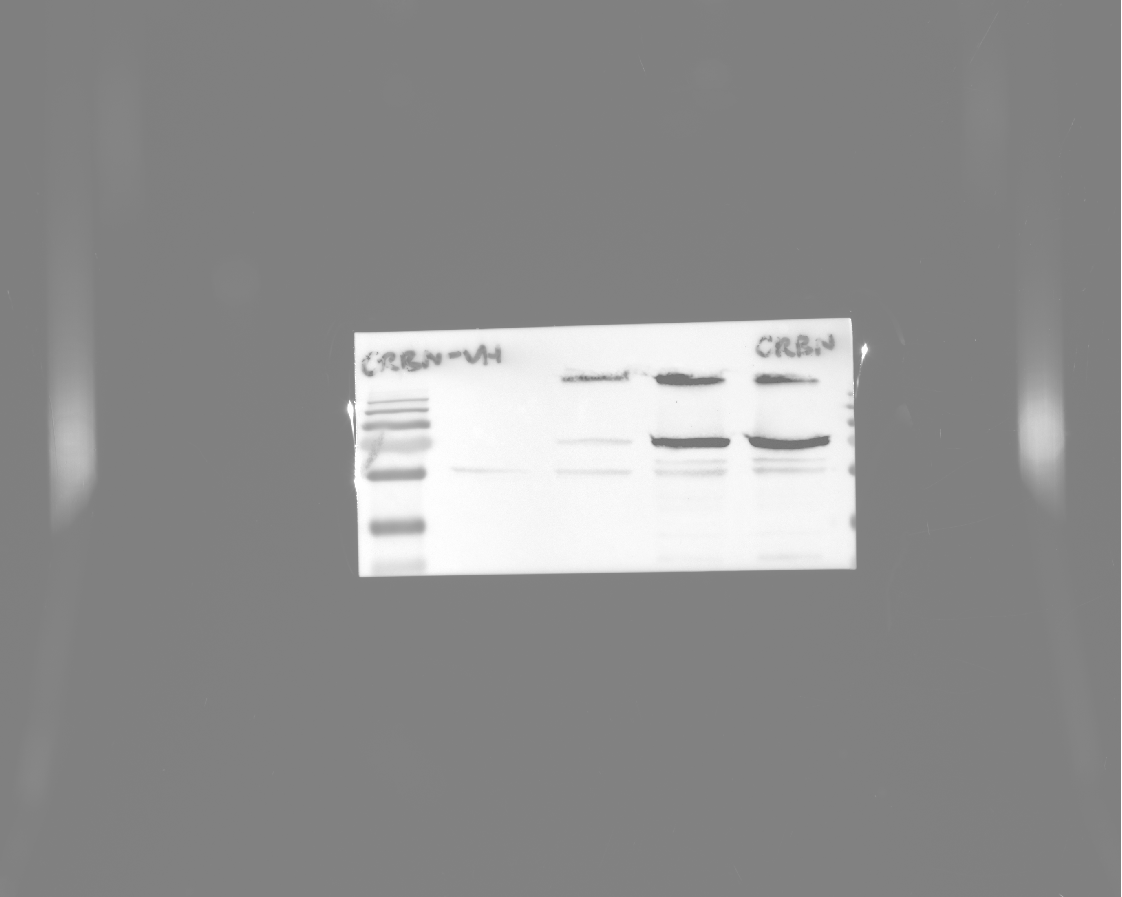

Supplement: Figure 1—figure supplement 1—source data 2. [file elife-106699-fig1-figsupp1-data2.zip › Figure 1ΓÇöfigure supplement 1-source data 2 Western blot raw data shows LMO2 protein degradation in HEK293T cells with different biodegrader contruct./CRBN CRBN-iDab(Composite).tif]

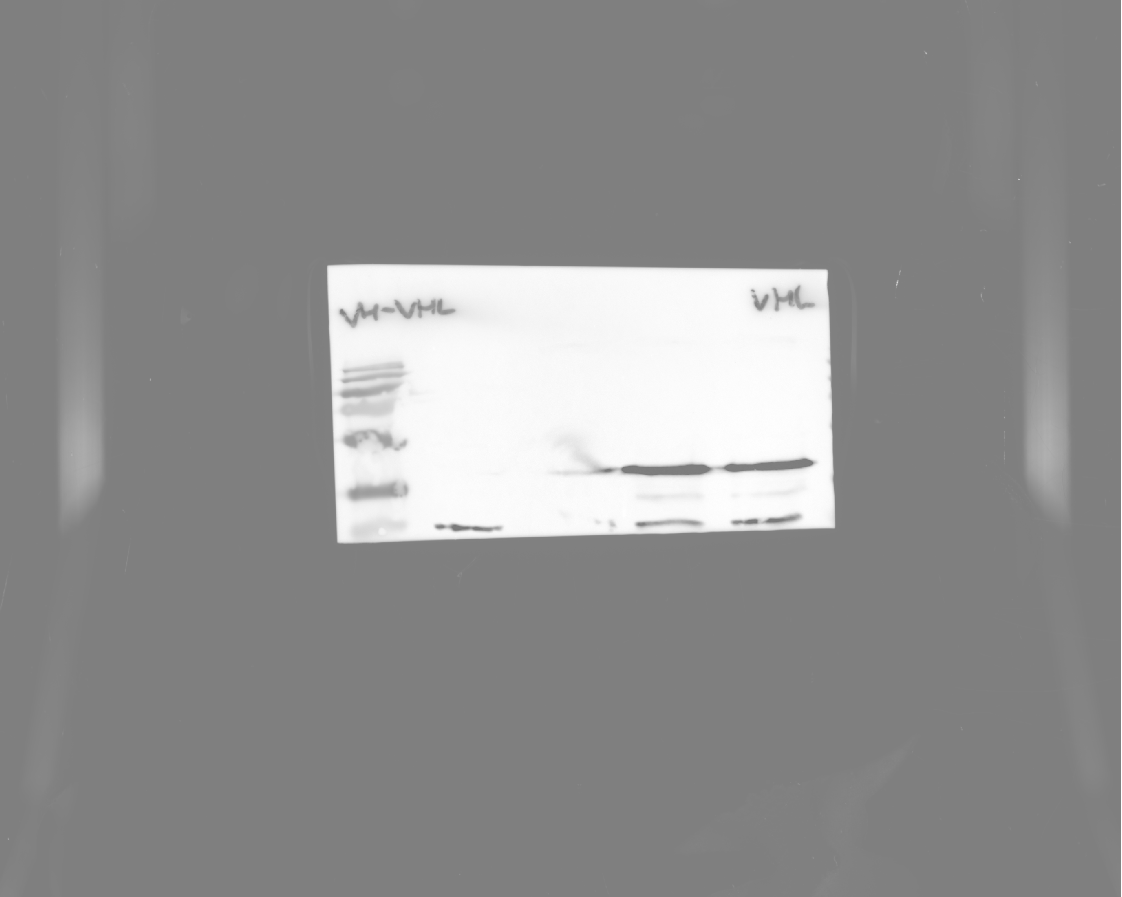

Supplement: Figure 1—figure supplement 1—source data 2. [file elife-106699-fig1-figsupp1-data2.zip › Figure 1ΓÇöfigure supplement 1-source data 2 Western blot raw data shows LMO2 protein degradation in HEK293T cells with different biodegrader contruct./VHL iDab-VHL(Composite).tif]

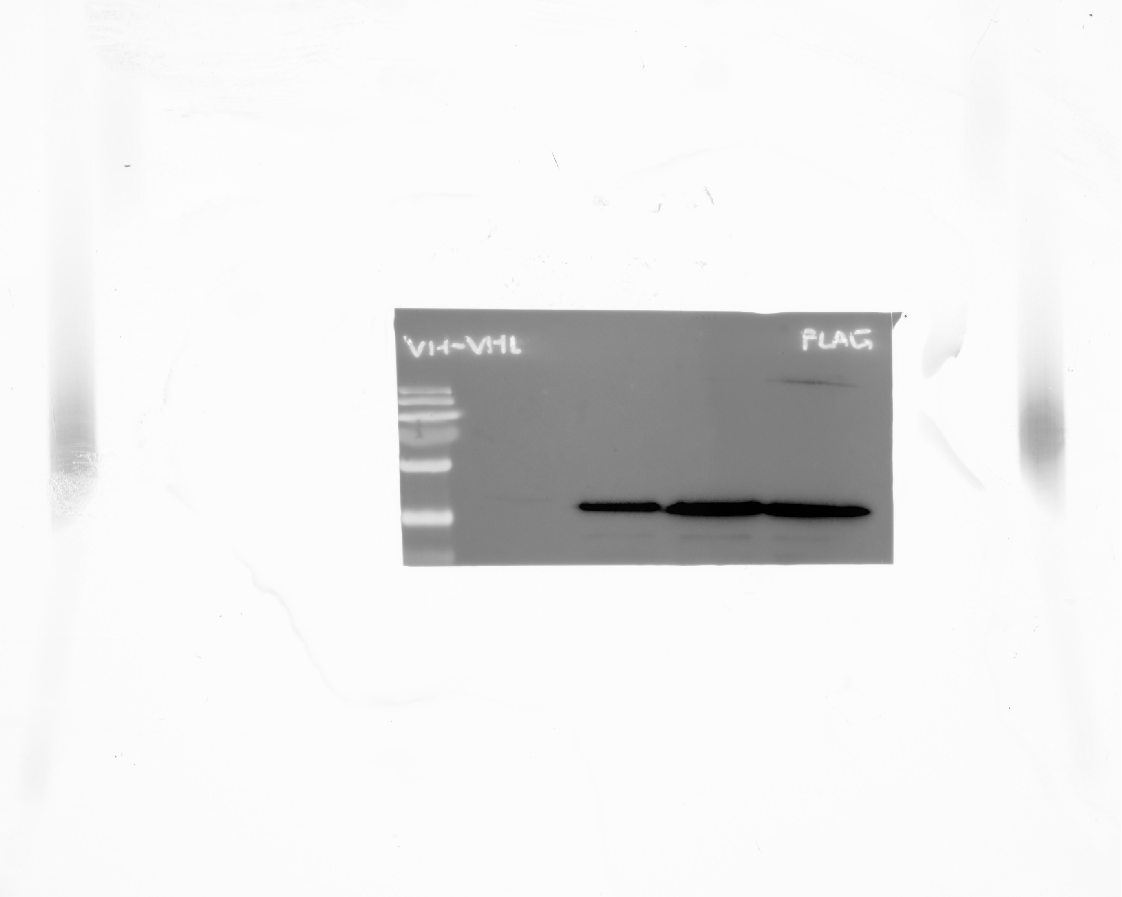

Supplement: Figure 1—figure supplement 1—source data 2. [file elife-106699-fig1-figsupp1-data2.zip › Figure 1ΓÇöfigure supplement 1-source data 2 Western blot raw data shows LMO2 protein degradation in HEK293T cells with different biodegrader contruct./FLAG iDab-VHL(Composite).tif]

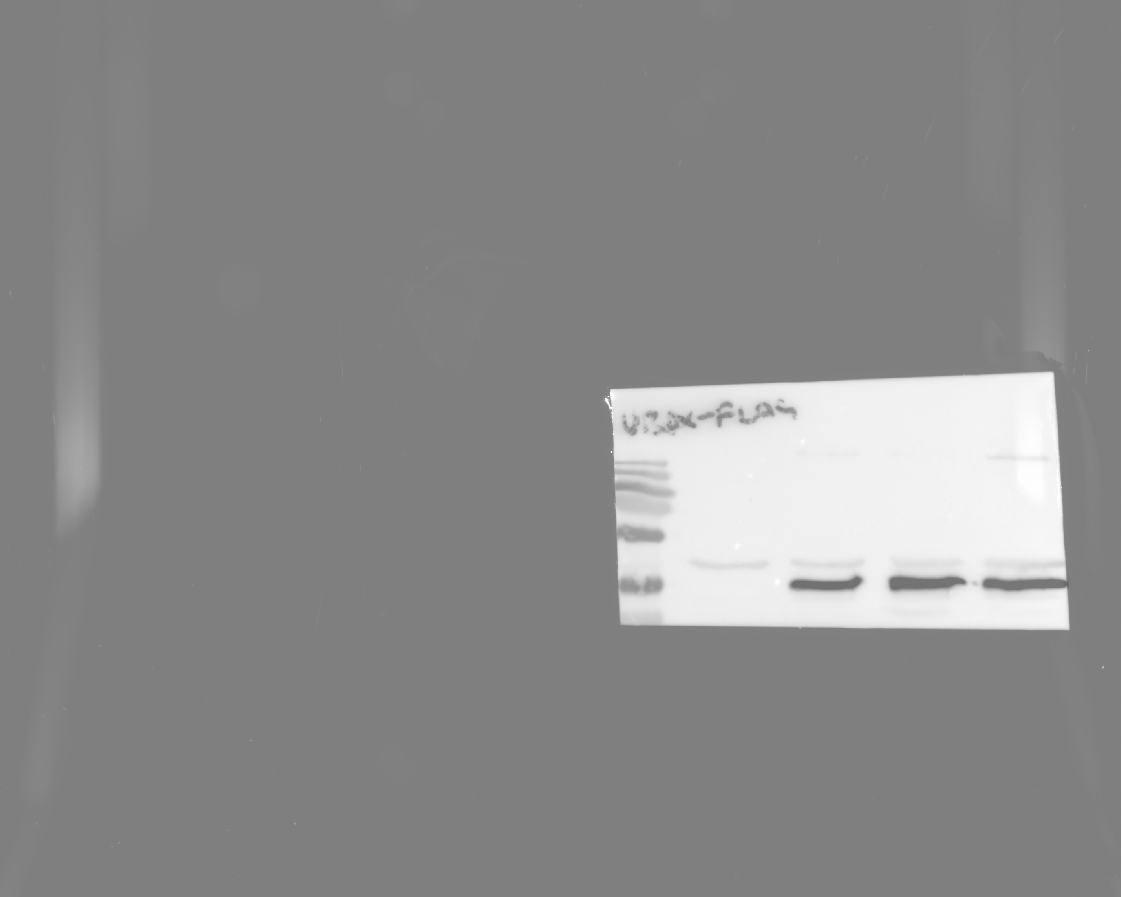

Supplement: Figure 1—figure supplement 1—source data 2. [file elife-106699-fig1-figsupp1-data2.zip › Figure 1ΓÇöfigure supplement 1-source data 2 Western blot raw data shows LMO2 protein degradation in HEK293T cells with different biodegrader contruct./Flag iDab-UBOX(Composite).tif]

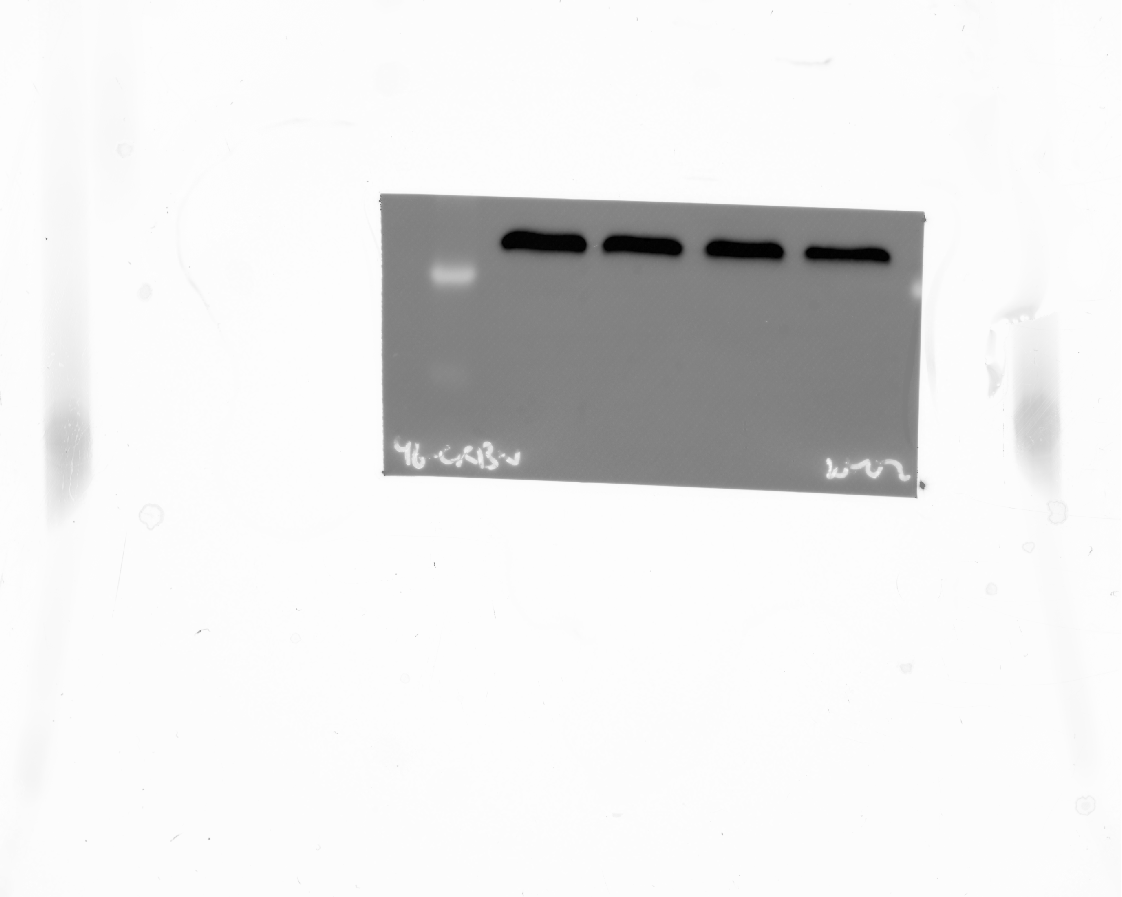

Supplement: Figure 1—figure supplement 1—source data 2. [file elife-106699-fig1-figsupp1-data2.zip › Figure 1ΓÇöfigure supplement 1-source data 2 Western blot raw data shows LMO2 protein degradation in HEK293T cells with different biodegrader contruct./LMO2 iDabRas-CRBN(Composite).tif]

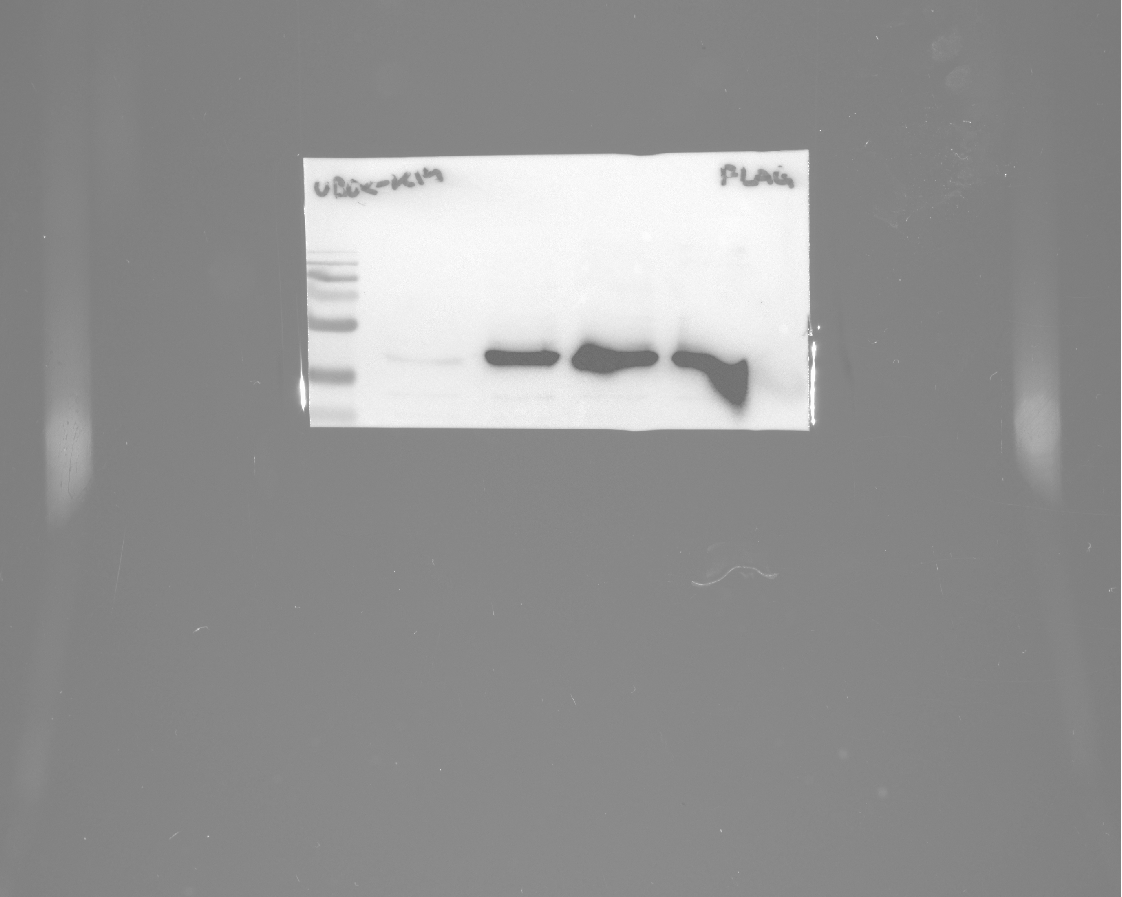

Supplement: Figure 1—figure supplement 1—source data 2. [file elife-106699-fig1-figsupp1-data2.zip › Figure 1ΓÇöfigure supplement 1-source data 2 Western blot raw data shows LMO2 protein degradation in HEK293T cells with different biodegrader contruct./FLAG UBOX-iDabRas(Composite).tif]

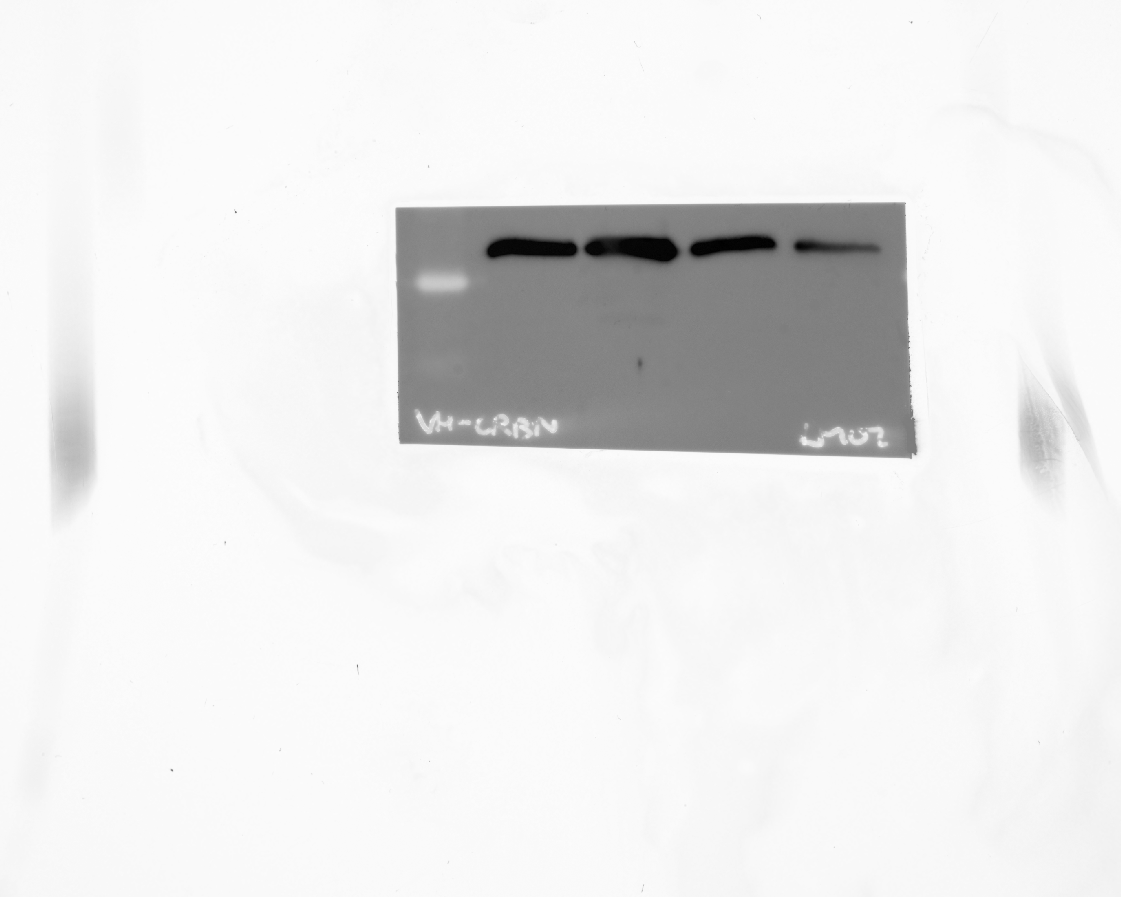

Supplement: Figure 1—figure supplement 1—source data 2. [file elife-106699-fig1-figsupp1-data2.zip › Figure 1ΓÇöfigure supplement 1-source data 2 Western blot raw data shows LMO2 protein degradation in HEK293T cells with different biodegrader contruct./LMO2 iDab-CRBN(Composite).tif]

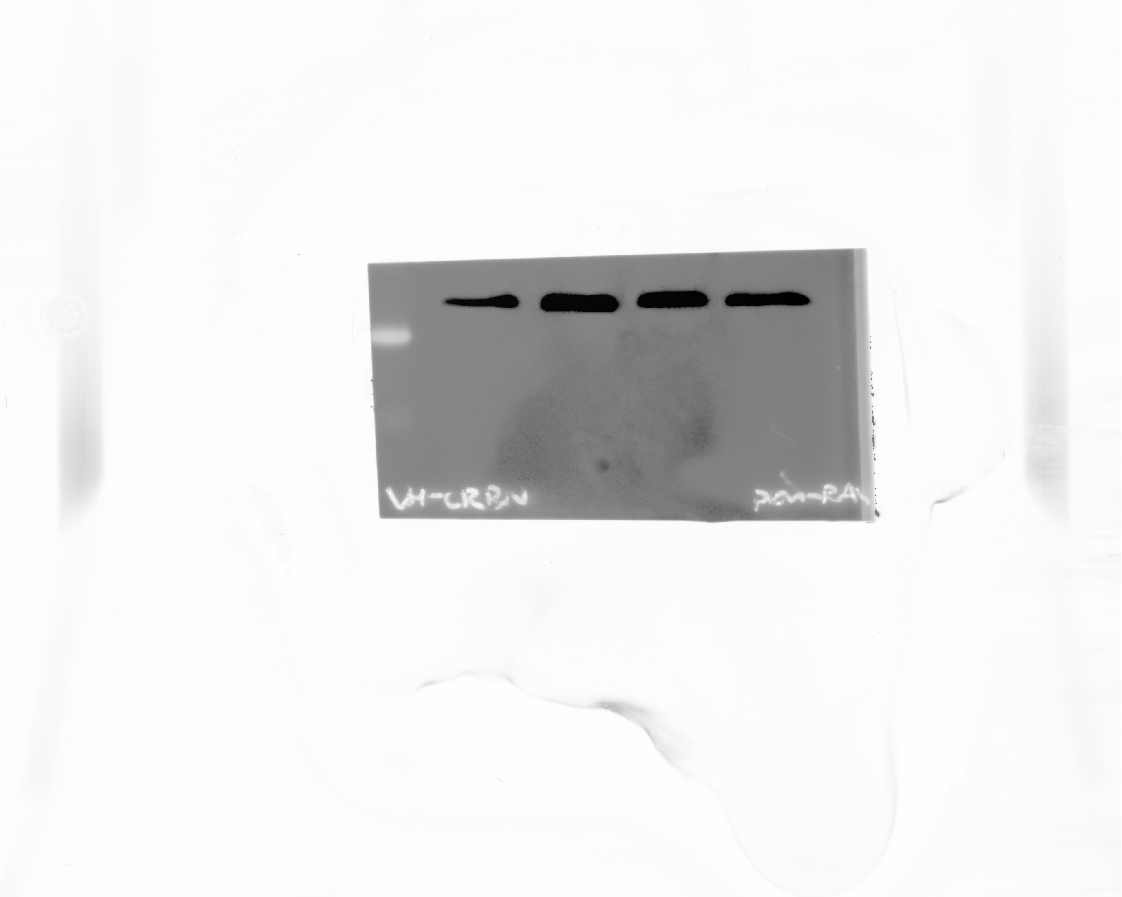

Supplement: Figure 1—figure supplement 1—source data 2. [file elife-106699-fig1-figsupp1-data2.zip › Figure 1ΓÇöfigure supplement 1-source data 2 Western blot raw data shows LMO2 protein degradation in HEK293T cells with different biodegrader contruct./panRAS iDab-CRBN(Composite).tif]

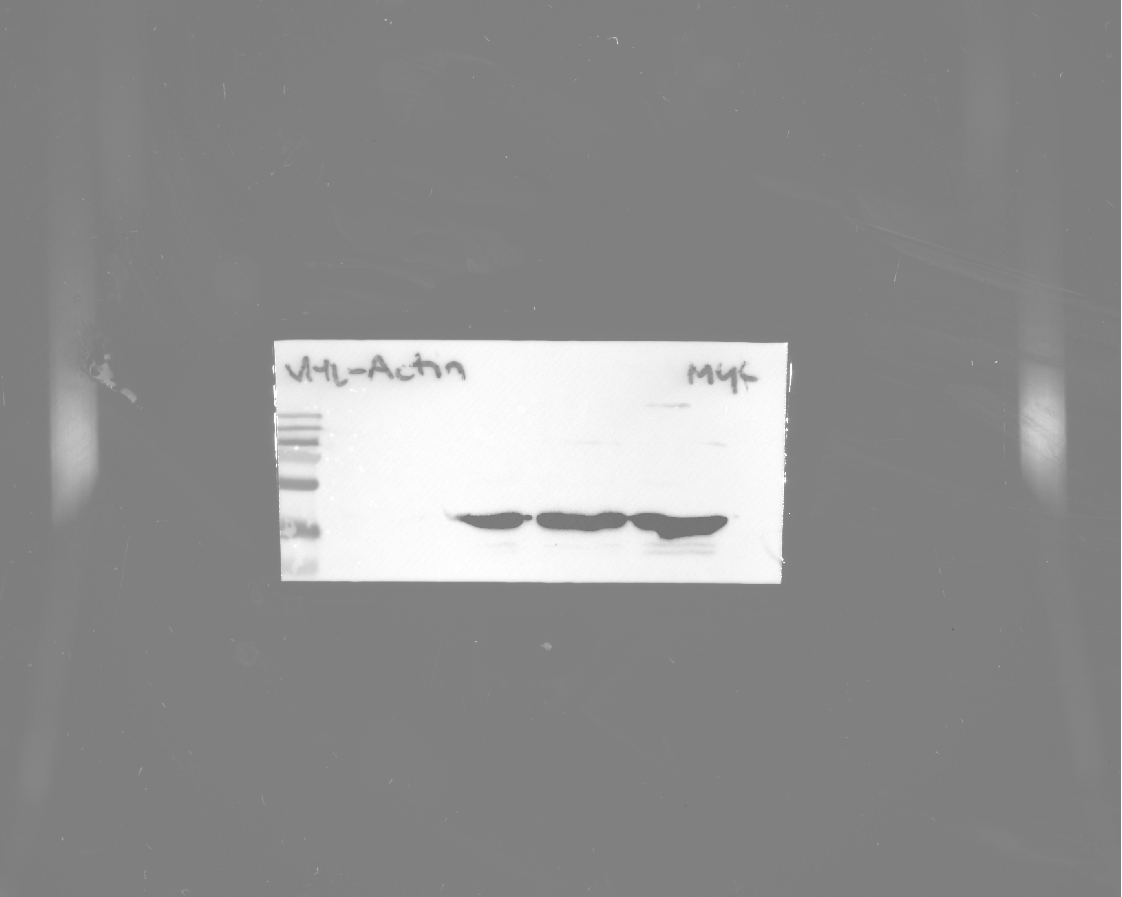

Supplement: Figure 1—figure supplement 1—source data 2. [file elife-106699-fig1-figsupp1-data2.zip › Figure 1ΓÇöfigure supplement 1-source data 2 Western blot raw data shows LMO2 protein degradation in HEK293T cells with different biodegrader contruct./myc9E10 VHL-iDab(Composite).jpg]

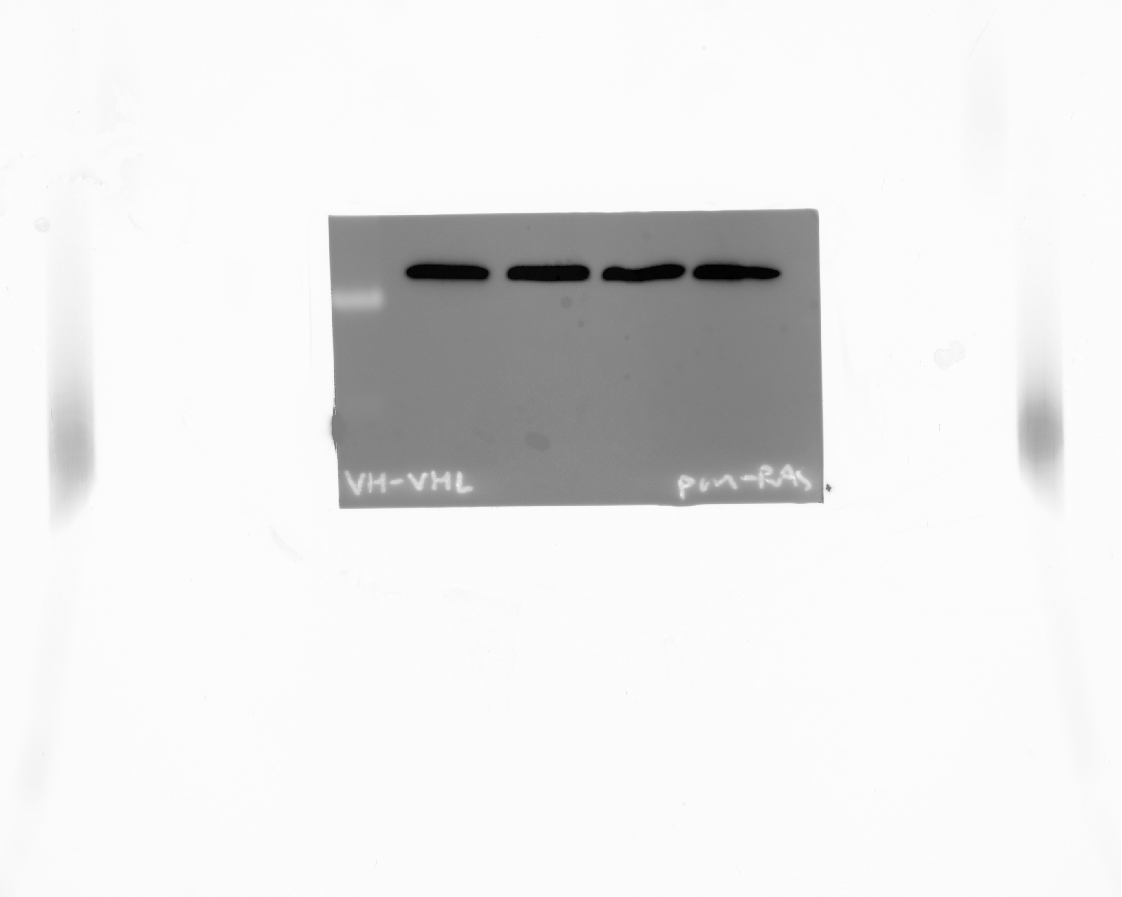

Supplement: Figure 1—figure supplement 1—source data 2. [file elife-106699-fig1-figsupp1-data2.zip › Figure 1ΓÇöfigure supplement 1-source data 2 Western blot raw data shows LMO2 protein degradation in HEK293T cells with different biodegrader contruct./Cyclophilin iDab-VHL(Composite).tif]

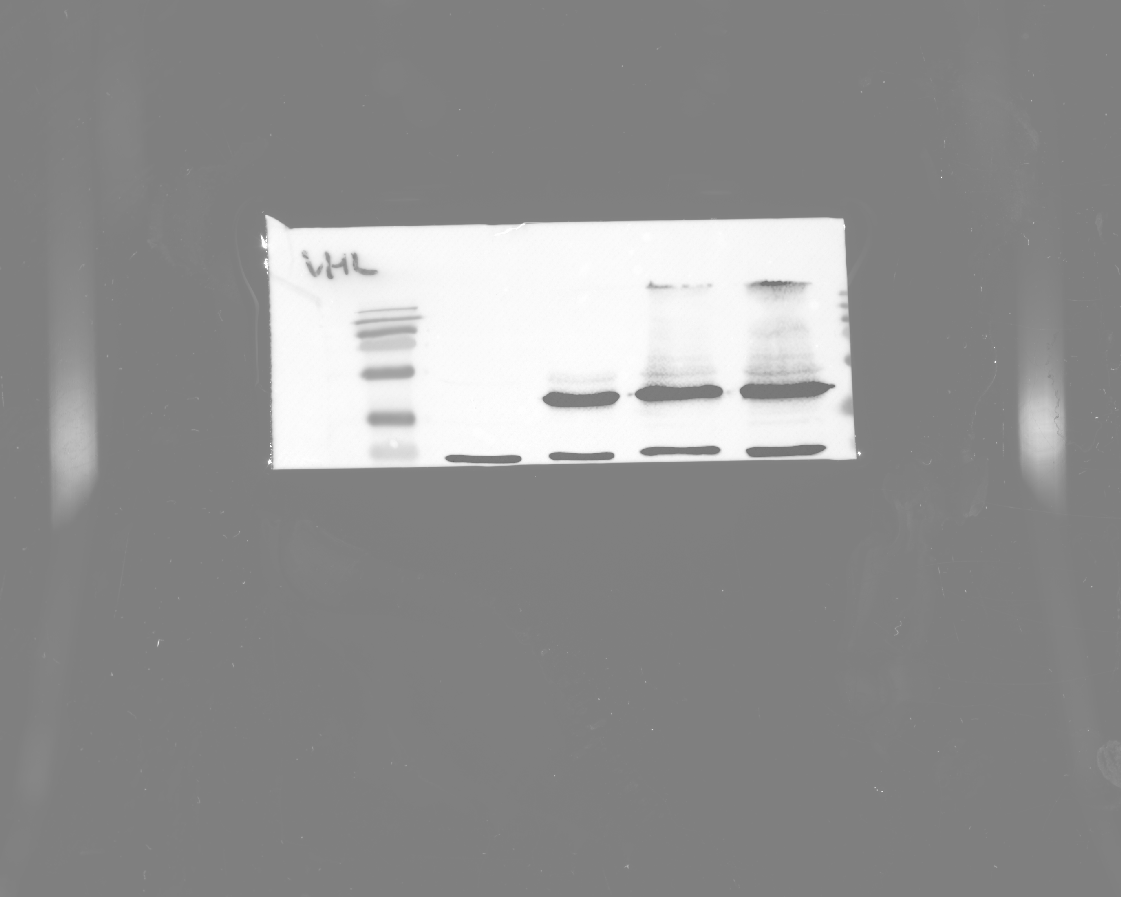

Supplement: Figure 1—figure supplement 1—source data 2. [file elife-106699-fig1-figsupp1-data2.zip › Figure 1ΓÇöfigure supplement 1-source data 2 Western blot raw data shows LMO2 protein degradation in HEK293T cells with different biodegrader contruct./VHL VHL-iDab(Composite).tif]

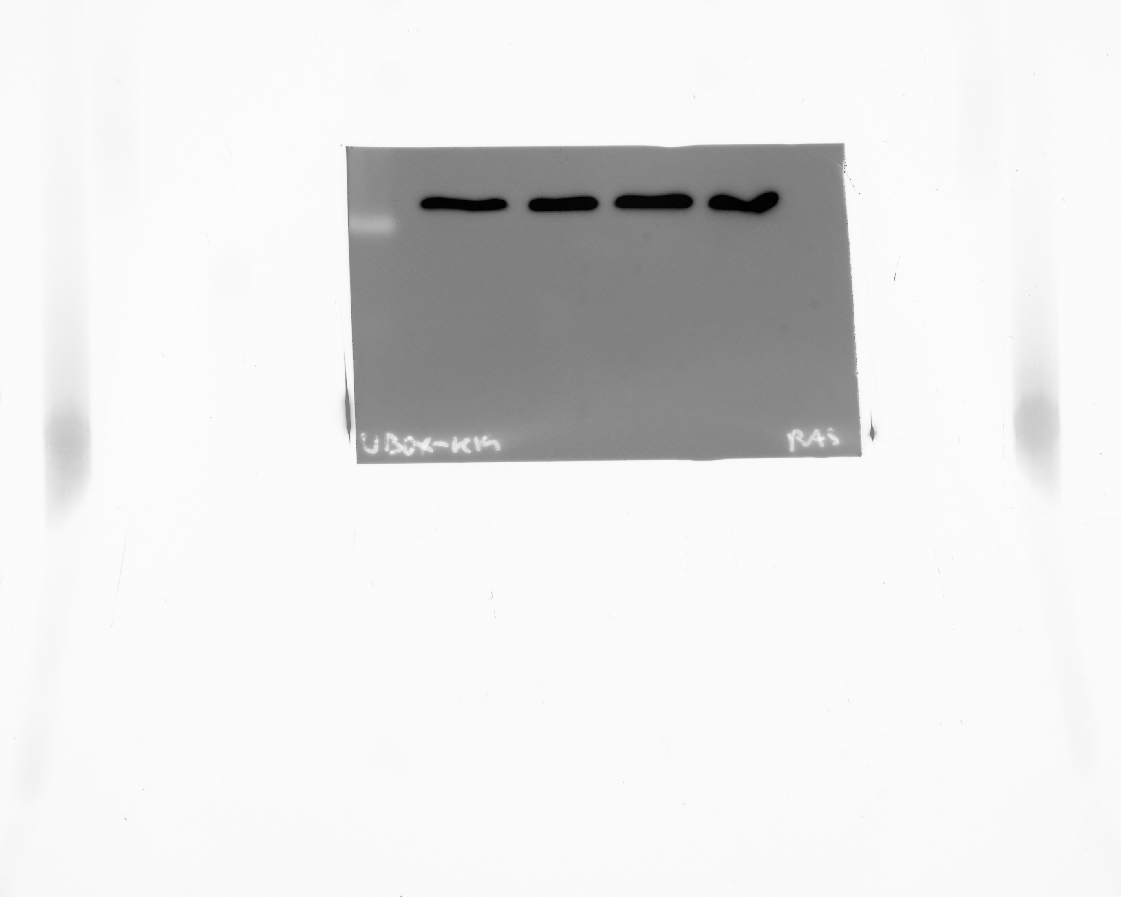

Supplement: Figure 1—figure supplement 1—source data 2. [file elife-106699-fig1-figsupp1-data2.zip › Figure 1ΓÇöfigure supplement 1-source data 2 Western blot raw data shows LMO2 protein degradation in HEK293T cells with different biodegrader contruct./Cyclophilin VHL-iDabRas(Composite).tif]

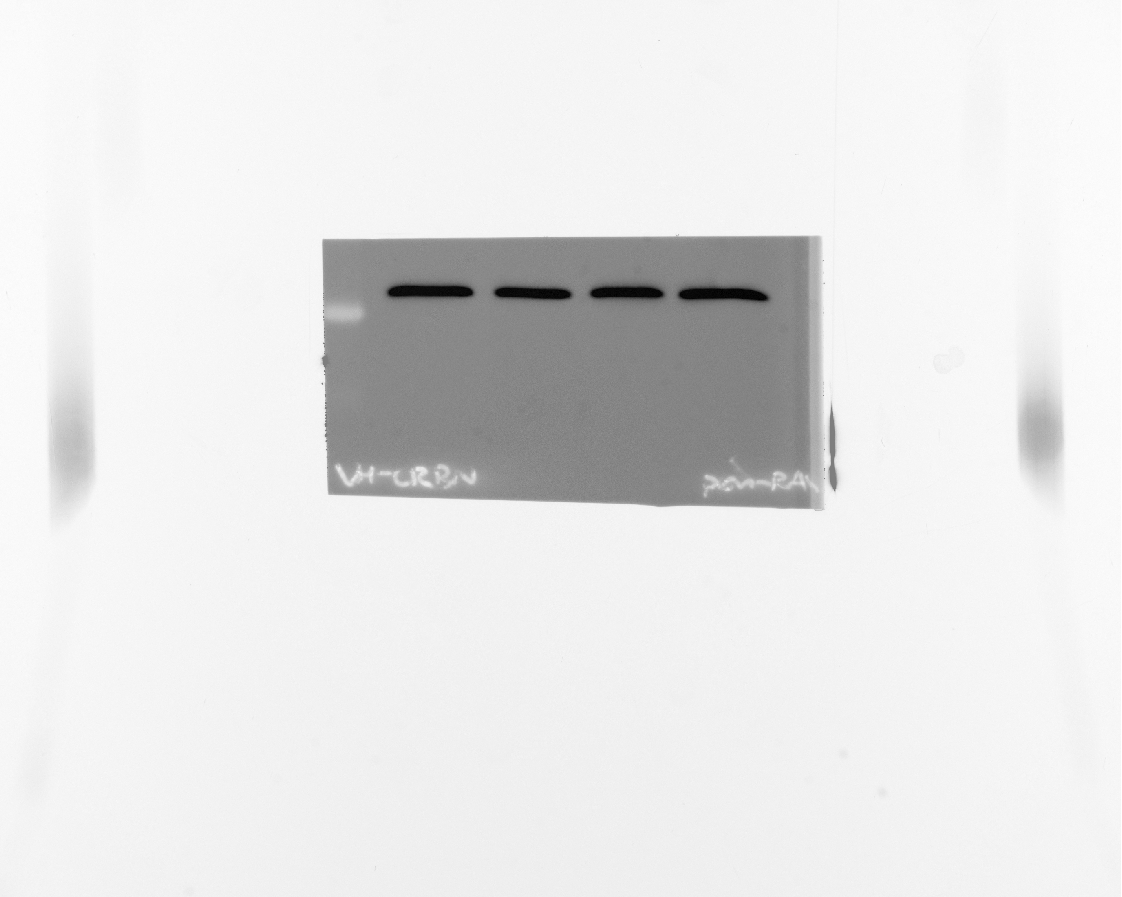

Supplement: Figure 1—figure supplement 1—source data 2. [file elife-106699-fig1-figsupp1-data2.zip › Figure 1ΓÇöfigure supplement 1-source data 2 Western blot raw data shows LMO2 protein degradation in HEK293T cells with different biodegrader contruct./Cyclophilin iDab-CRBN(Composite).tif]

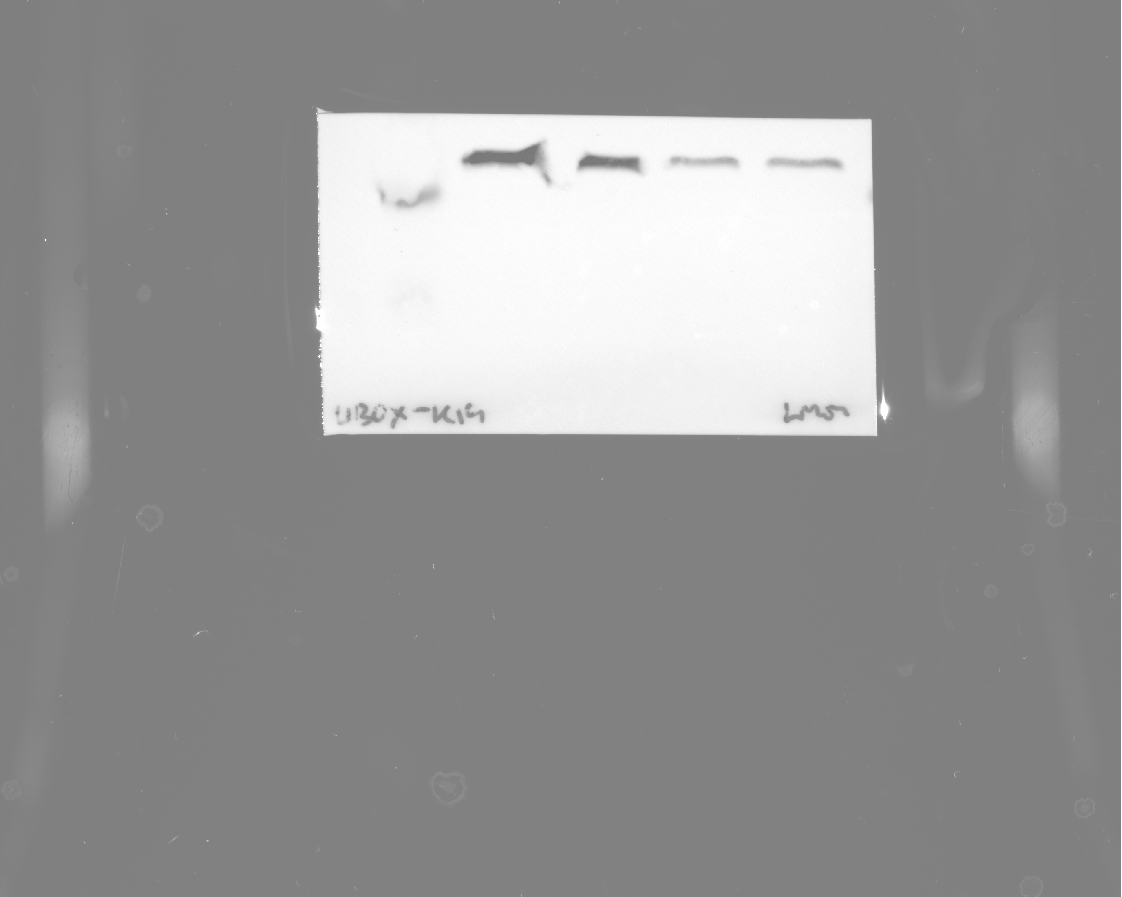

Supplement: Figure 1—figure supplement 1—source data 2. [file elife-106699-fig1-figsupp1-data2.zip › Figure 1ΓÇöfigure supplement 1-source data 2 Western blot raw data shows LMO2 protein degradation in HEK293T cells with different biodegrader contruct./panRAS UBOX-iDabRas(Composite).tif]

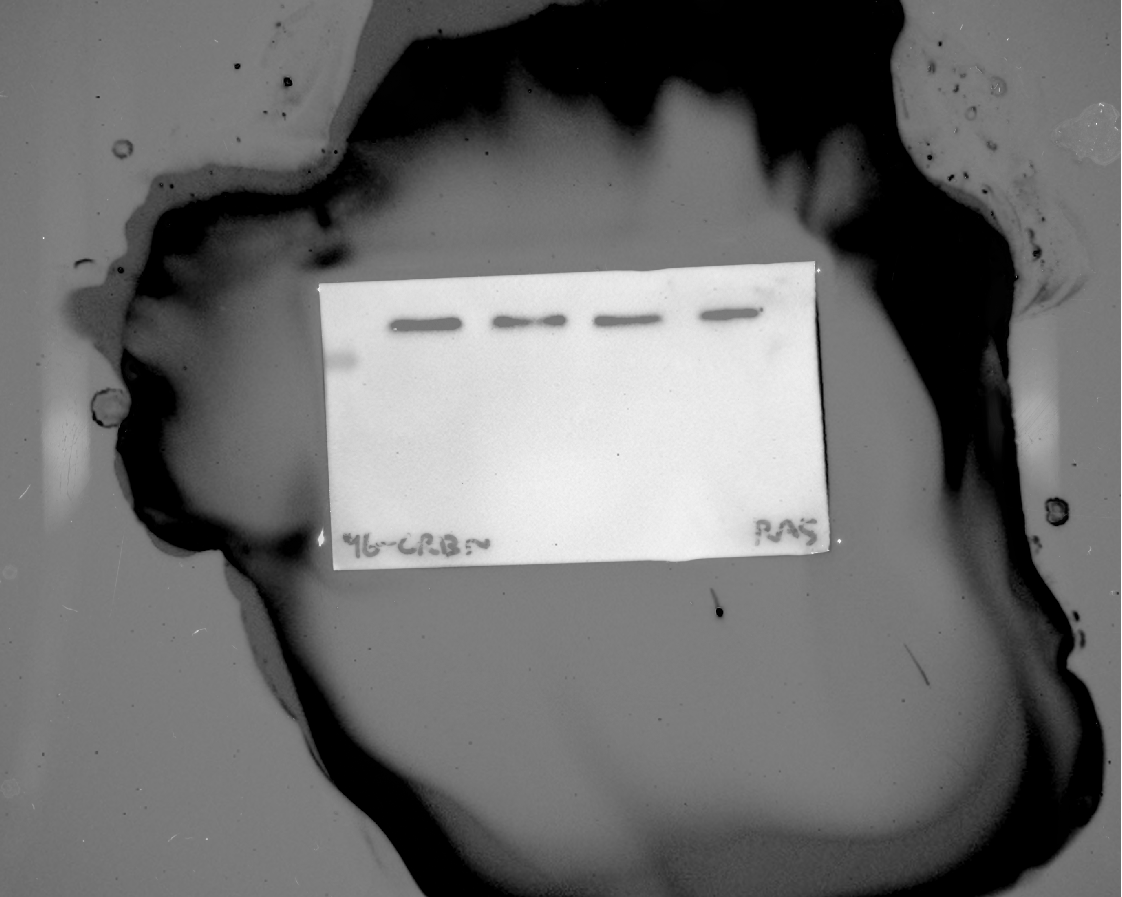

Supplement: Figure 1—figure supplement 1—source data 2. [file elife-106699-fig1-figsupp1-data2.zip › Figure 1ΓÇöfigure supplement 1-source data 2 Western blot raw data shows LMO2 protein degradation in HEK293T cells with different biodegrader contruct./pan-RAS iDabRas-CRBN(Composite).tif]

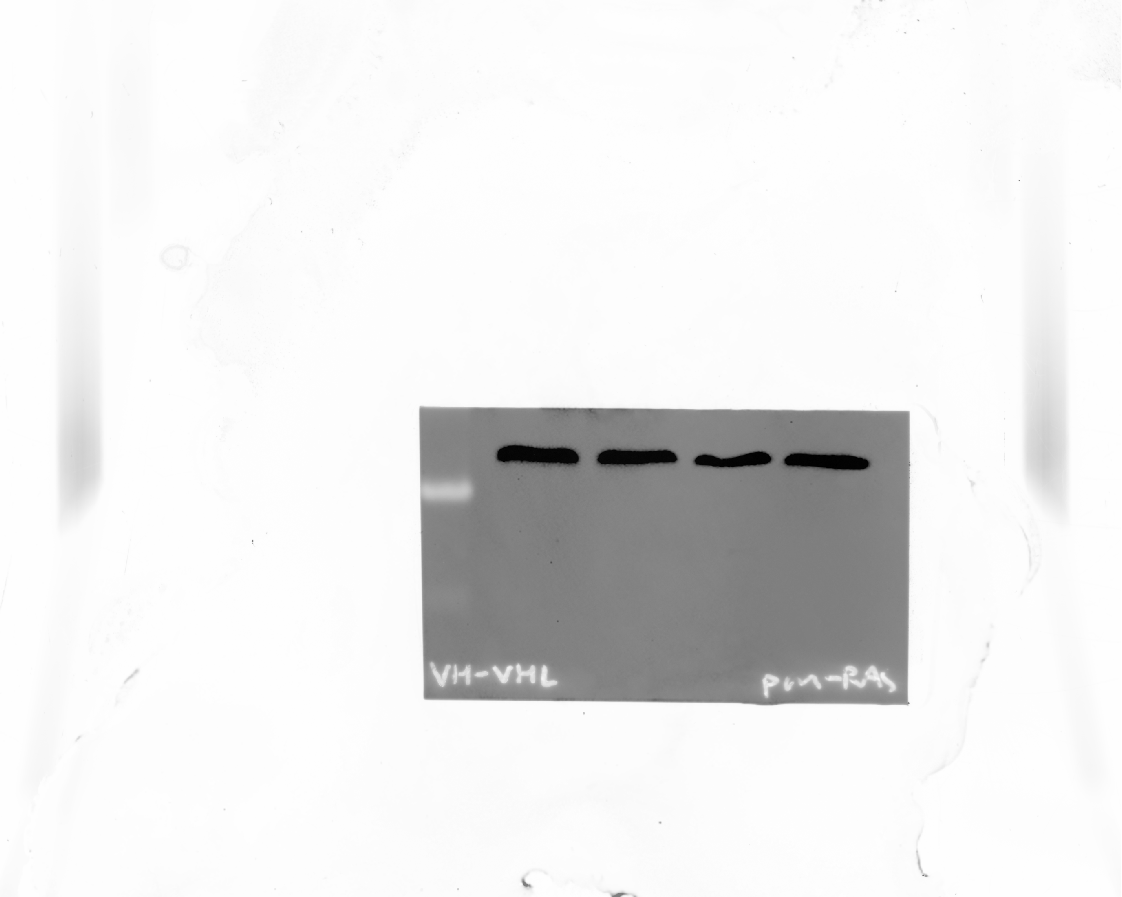

Supplement: Figure 1—figure supplement 1—source data 2. [file elife-106699-fig1-figsupp1-data2.zip › Figure 1ΓÇöfigure supplement 1-source data 2 Western blot raw data shows LMO2 protein degradation in HEK293T cells with different biodegrader contruct./panRAS iDab-VHL(Composite).tif]

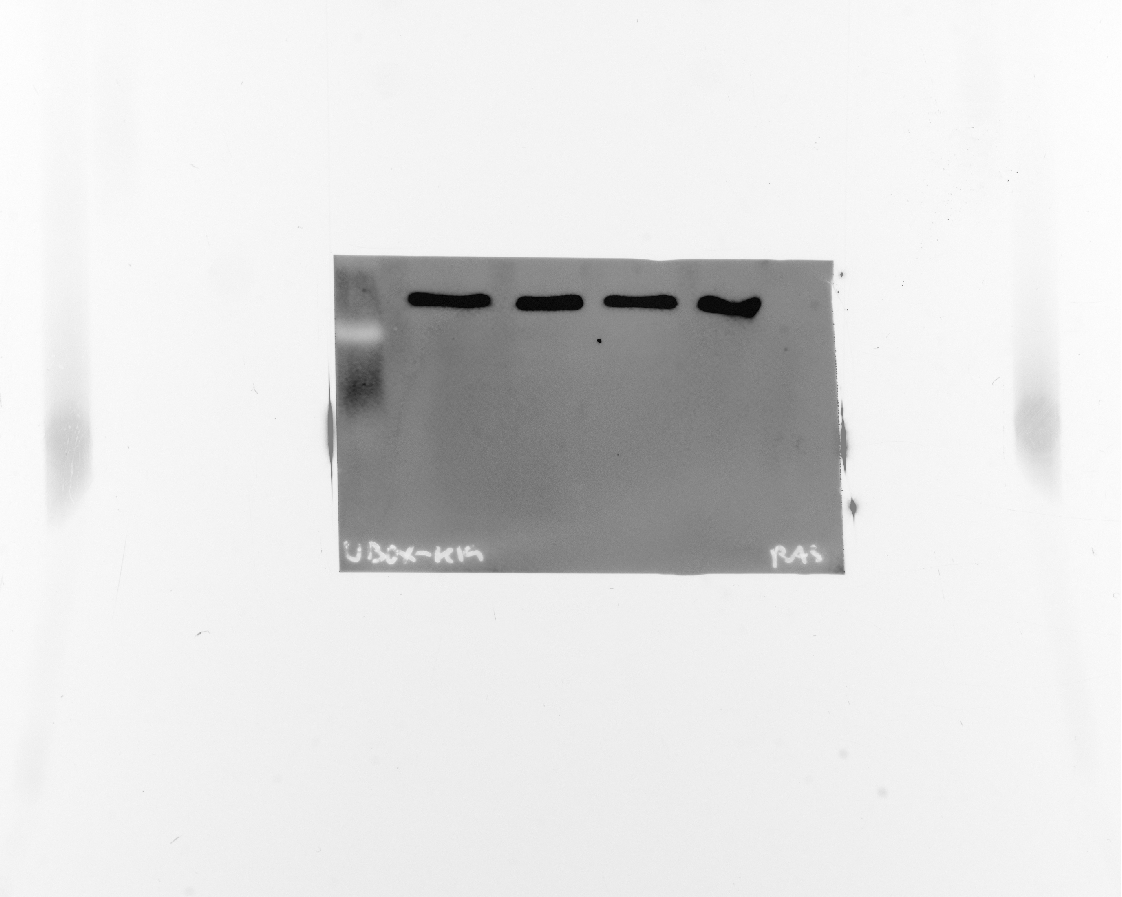

Supplement: Figure 1—figure supplement 1—source data 2. [file elife-106699-fig1-figsupp1-data2.zip › Figure 1ΓÇöfigure supplement 1-source data 2 Western blot raw data shows LMO2 protein degradation in HEK293T cells with different biodegrader contruct./LMO2 UBOX-iDab-Ras(Composite).tif]

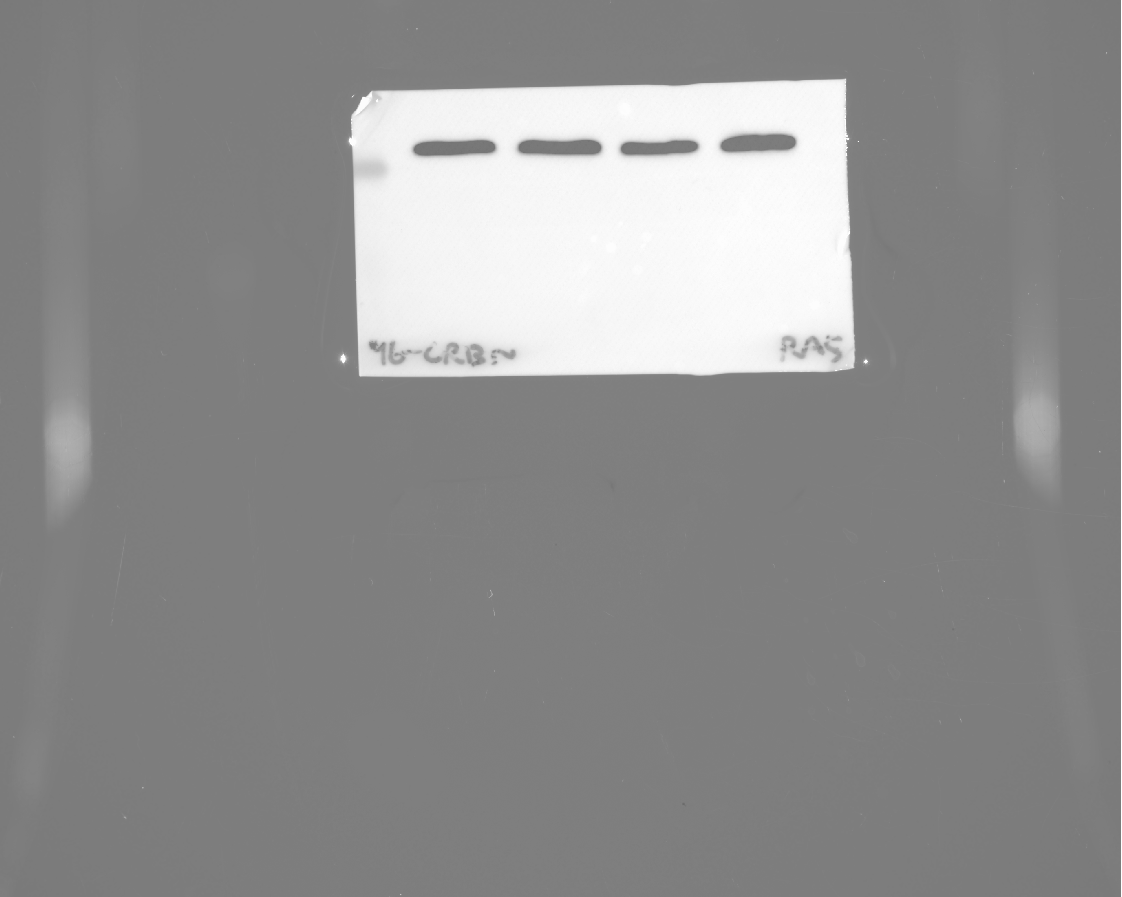

Supplement: Figure 1—figure supplement 1—source data 2. [file elife-106699-fig1-figsupp1-data2.zip › Figure 1ΓÇöfigure supplement 1-source data 2 Western blot raw data shows LMO2 protein degradation in HEK293T cells with different biodegrader contruct./LMO2 VHL-iDabRas.tif]

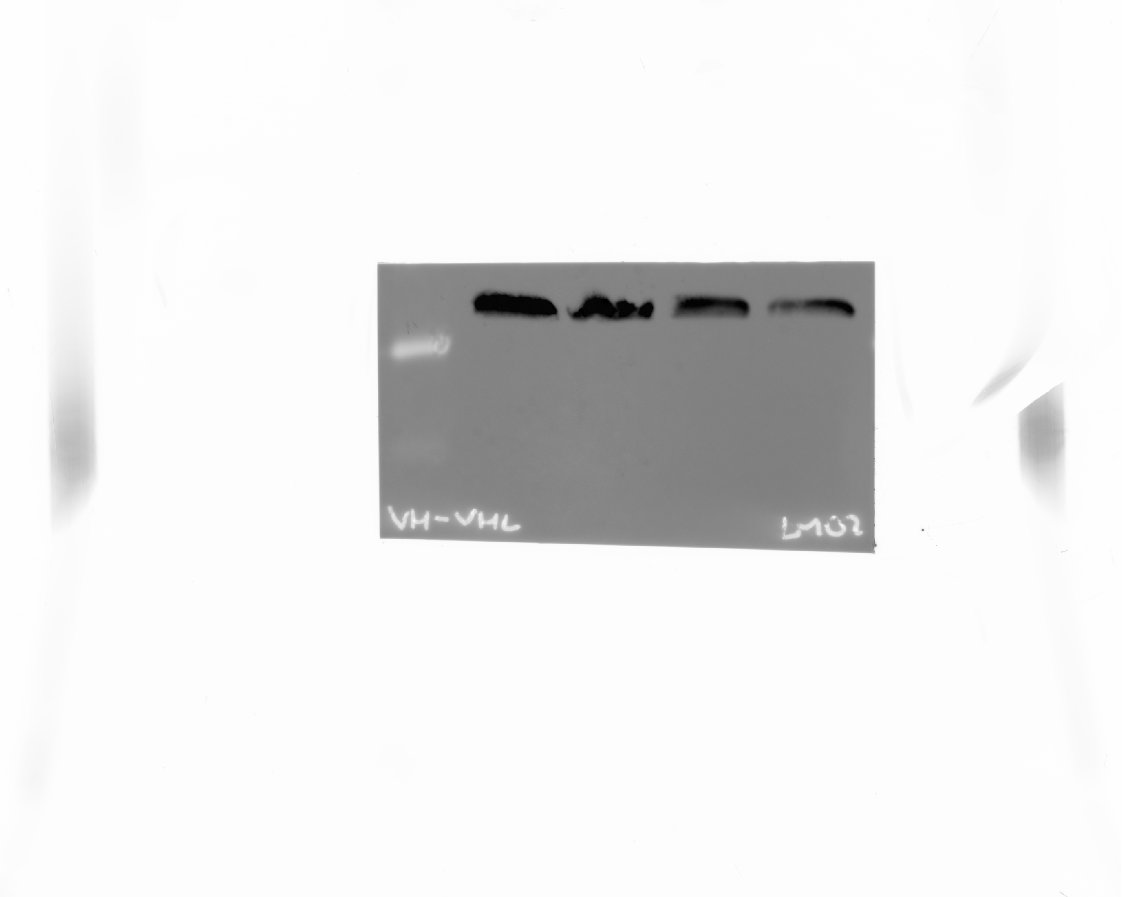

Supplement: Figure 1—figure supplement 1—source data 2. [file elife-106699-fig1-figsupp1-data2.zip › Figure 1ΓÇöfigure supplement 1-source data 2 Western blot raw data shows LMO2 protein degradation in HEK293T cells with different biodegrader contruct./LMO2 iDab-VHL(Composite).tif]

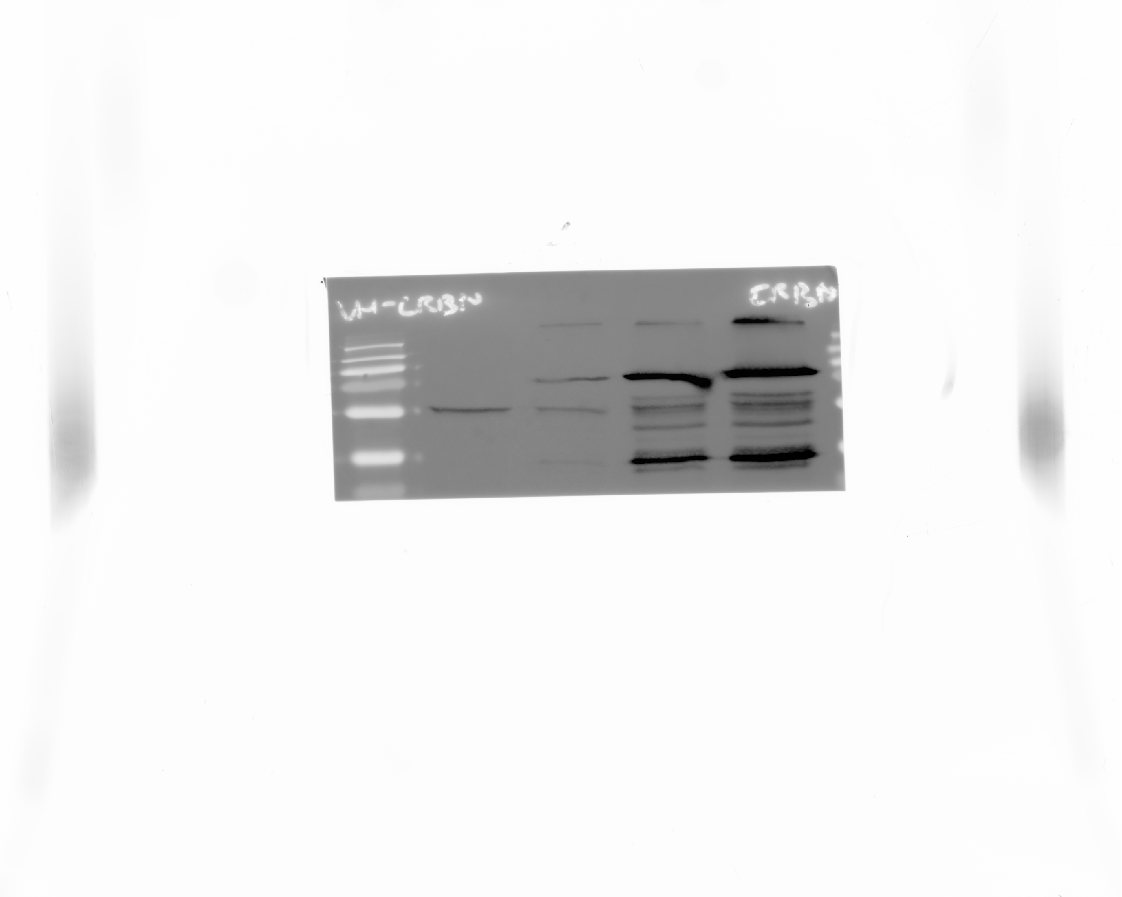

Supplement: Figure 1—figure supplement 1—source data 2. [file elife-106699-fig1-figsupp1-data2.zip › Figure 1ΓÇöfigure supplement 1-source data 2 Western blot raw data shows LMO2 protein degradation in HEK293T cells with different biodegrader contruct./CRBN iDab-CRBN(Composite).tif]

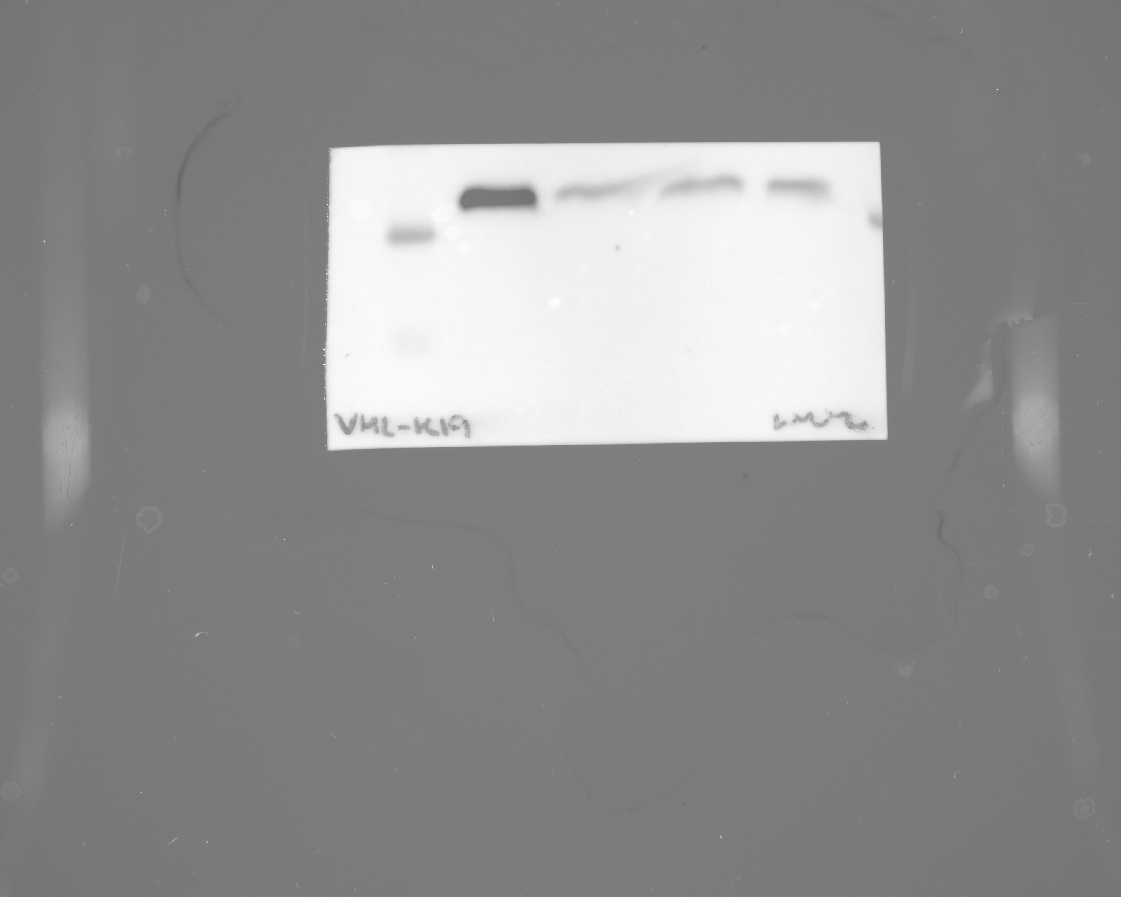

Supplement: Figure 1—figure supplement 1—source data 2. [file elife-106699-fig1-figsupp1-data2.zip › Figure 1ΓÇöfigure supplement 1-source data 2 Western blot raw data shows LMO2 protein degradation in HEK293T cells with different biodegrader contruct./panRAS VHL-iDabRas(Composite).tif]

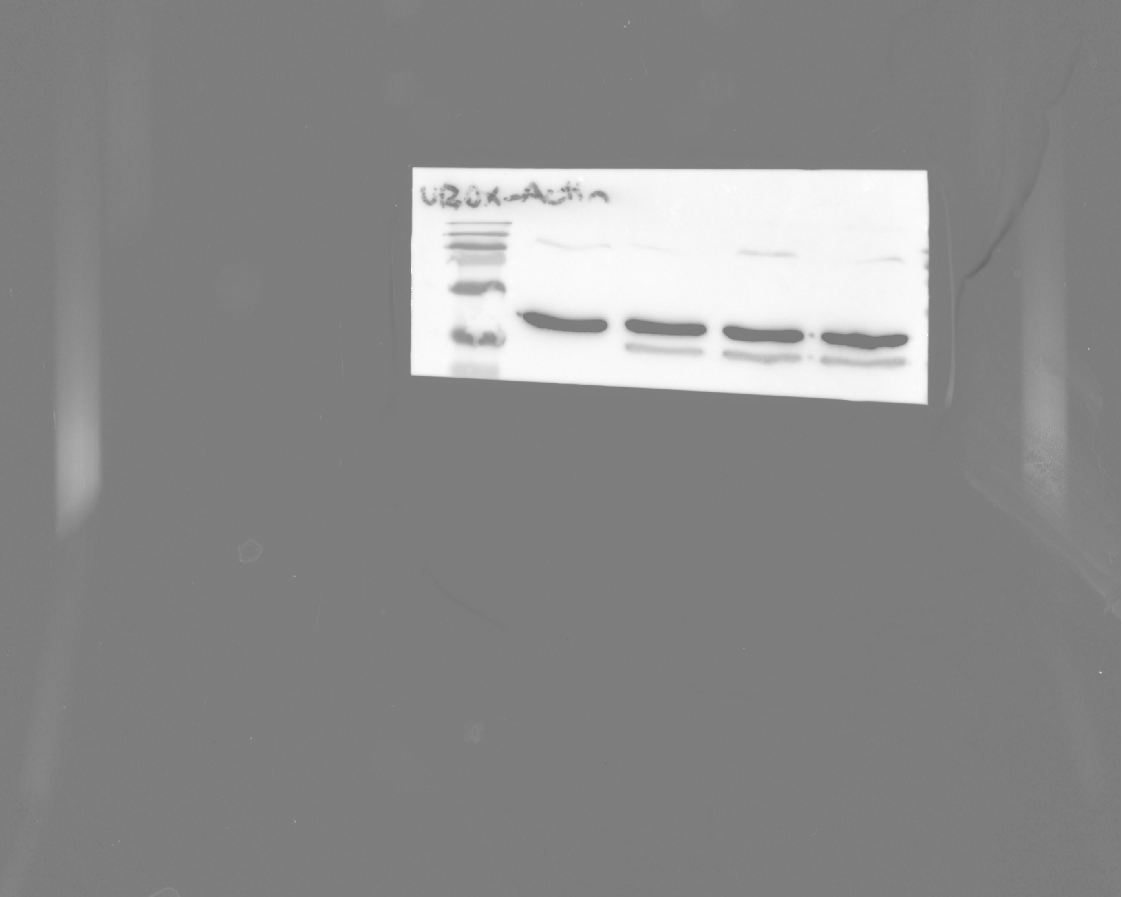

Supplement: Figure 1—figure supplement 1—source data 2. [file elife-106699-fig1-figsupp1-data2.zip › Figure 1ΓÇöfigure supplement 1-source data 2 Western blot raw data shows LMO2 protein degradation in HEK293T cells with different biodegrader contruct./Actin iDab-UBOX(Composite).tif]

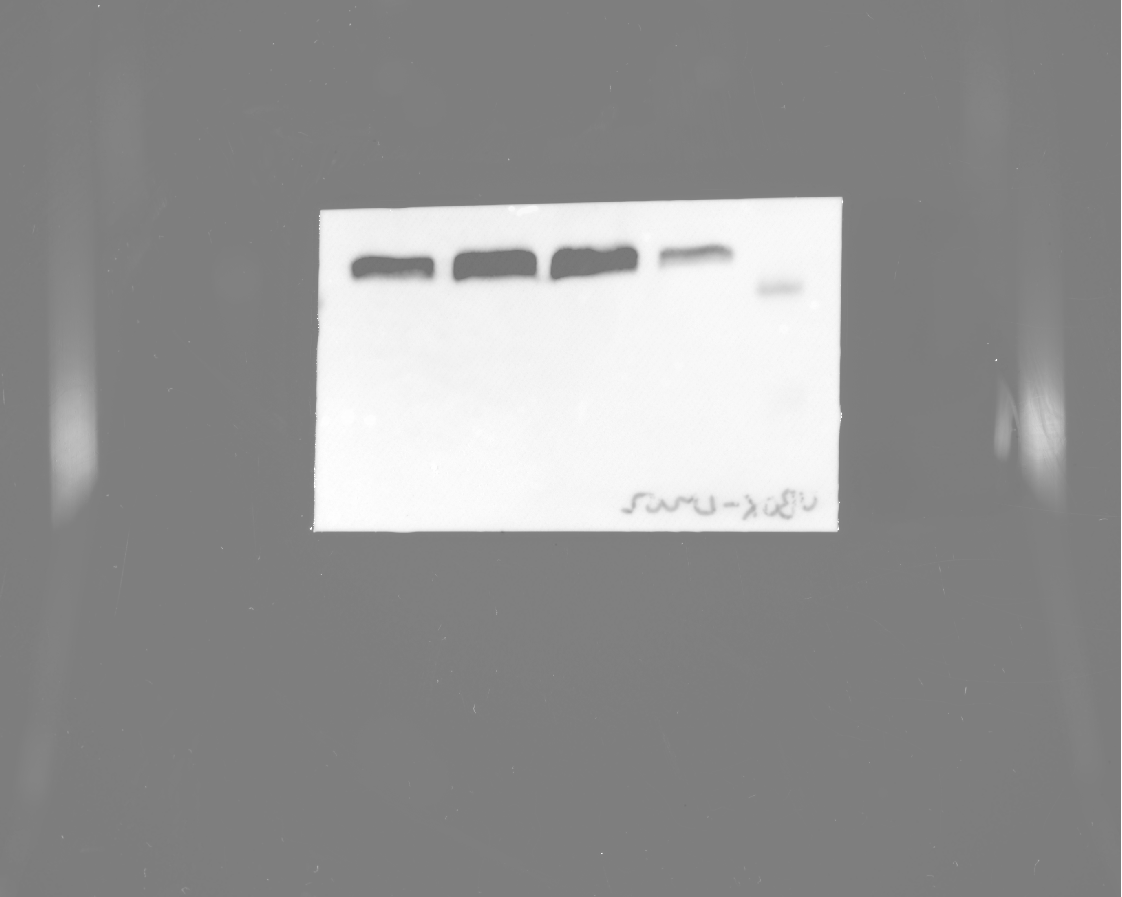

Supplement: Figure 1—figure supplement 1—source data 2. [file elife-106699-fig1-figsupp1-data2.zip › Figure 1ΓÇöfigure supplement 1-source data 2 Western blot raw data shows LMO2 protein degradation in HEK293T cells with different biodegrader contruct./LMO2 iDab-UBOX(Composite).tif]

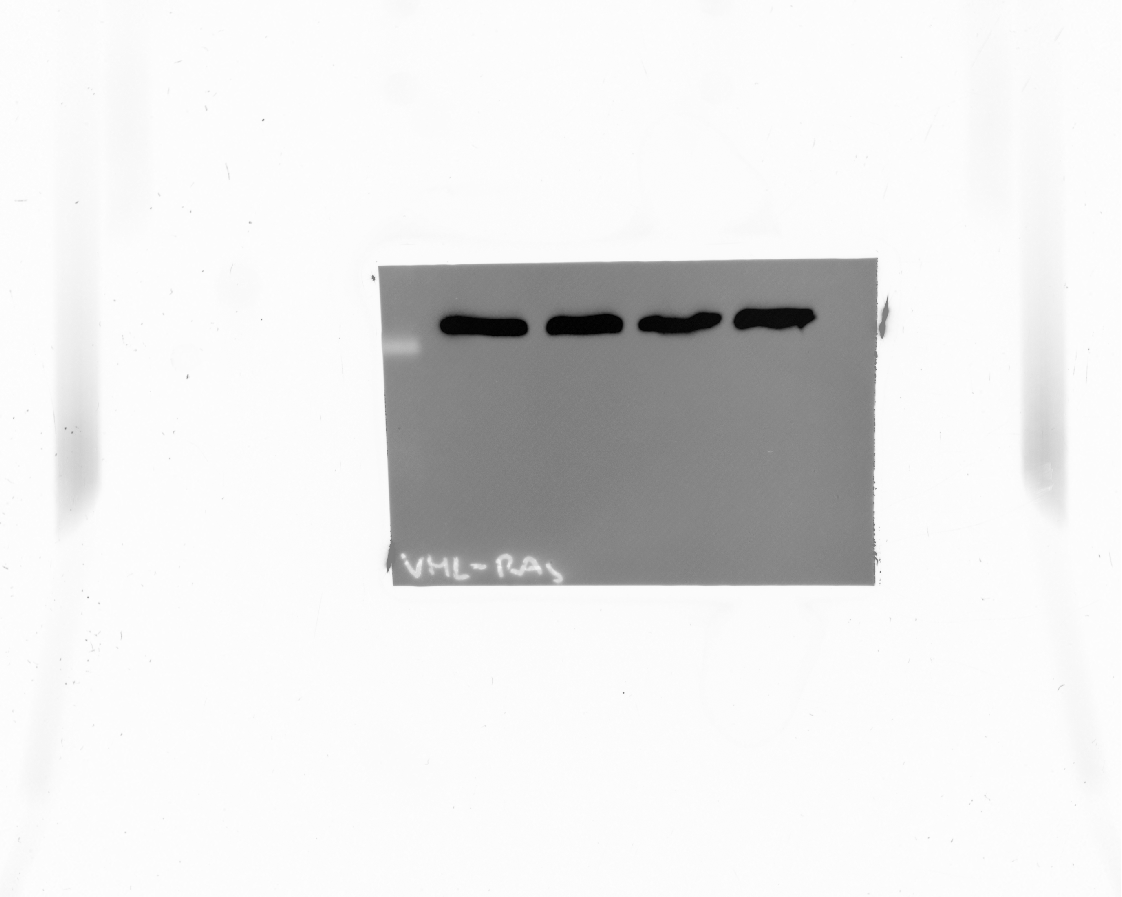

Supplement: Figure 1—figure supplement 1—source data 2. [file elife-106699-fig1-figsupp1-data2.zip › Figure 1ΓÇöfigure supplement 1-source data 2 Western blot raw data shows LMO2 protein degradation in HEK293T cells with different biodegrader contruct./Cyclophilin VHL-iDab(Composite).tif]

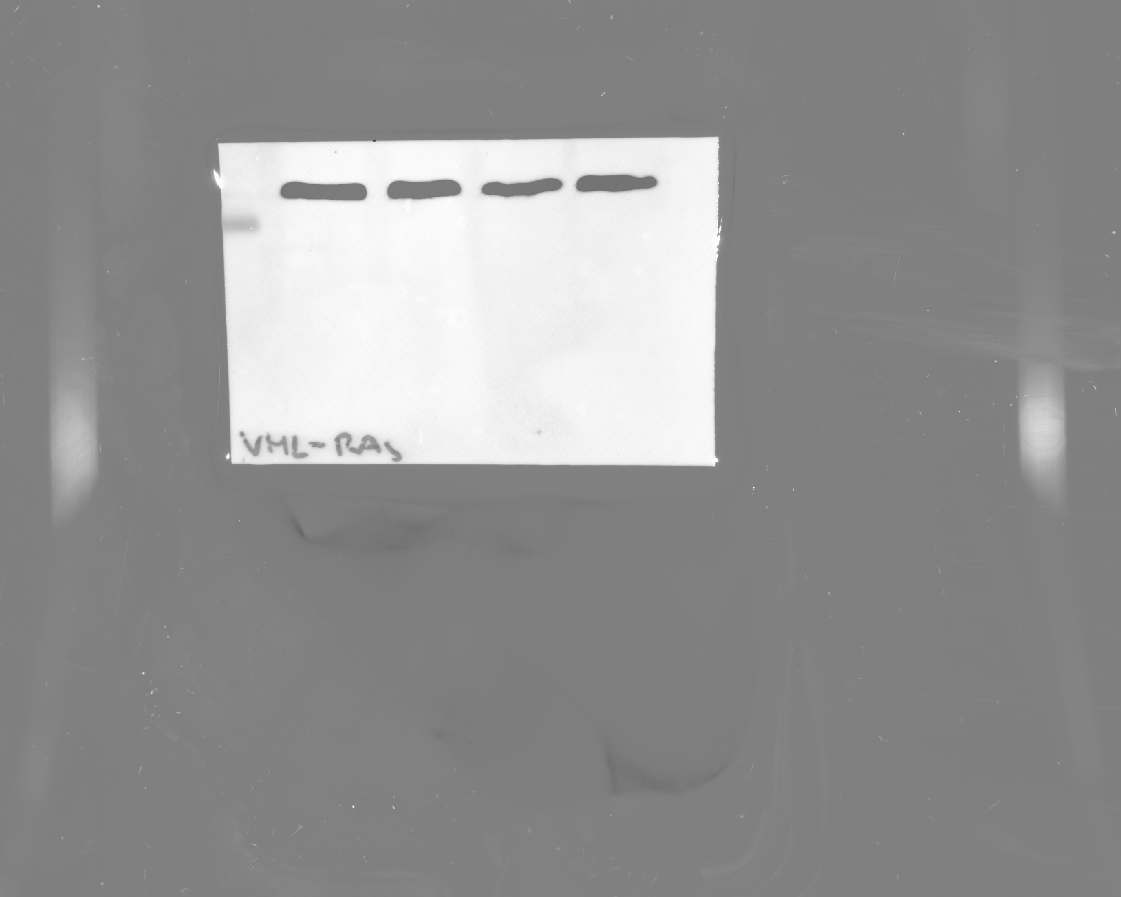

Supplement: Figure 1—figure supplement 1—source data 2. [file elife-106699-fig1-figsupp1-data2.zip › Figure 1ΓÇöfigure supplement 1-source data 2 Western blot raw data shows LMO2 protein degradation in HEK293T cells with different biodegrader contruct./pan-RAS VHL-iDab(Composite).tif]

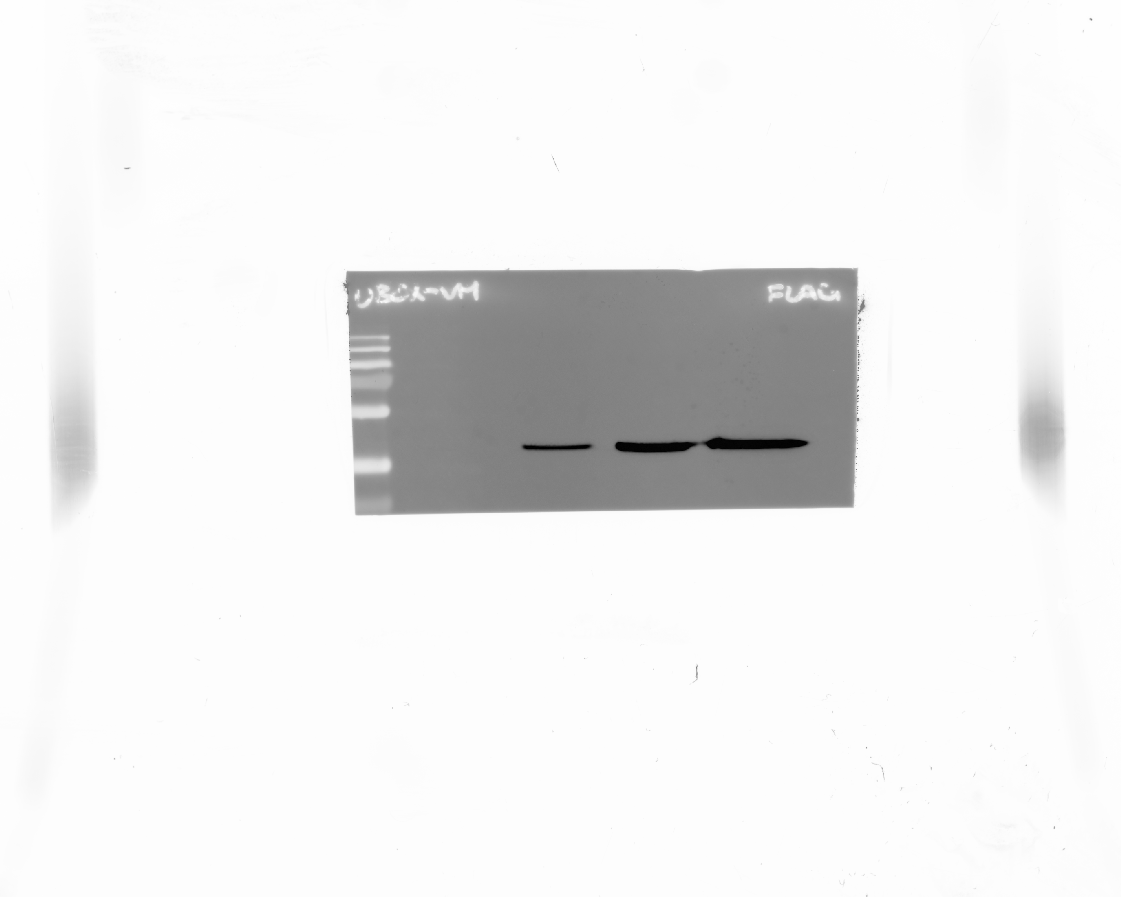

Supplement: Figure 1—figure supplement 1—source data 2. [file elife-106699-fig1-figsupp1-data2.zip › Figure 1ΓÇöfigure supplement 1-source data 2 Western blot raw data shows LMO2 protein degradation in HEK293T cells with different biodegrader contruct./FLAG UBOX-iDab(Composite).tif]

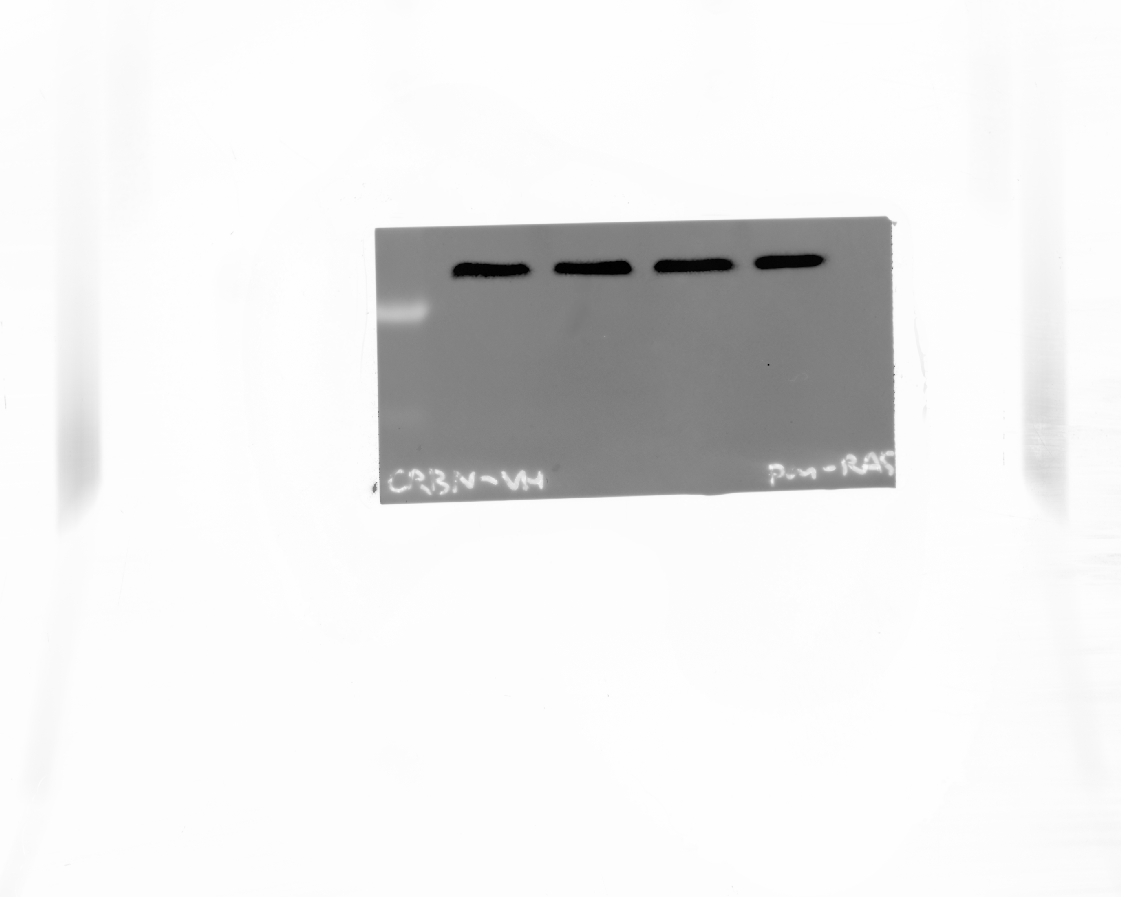

Supplement: Figure 1—figure supplement 1—source data 2. [file elife-106699-fig1-figsupp1-data2.zip › Figure 1ΓÇöfigure supplement 1-source data 2 Western blot raw data shows LMO2 protein degradation in HEK293T cells with different biodegrader contruct./panRAS CRBN-iDab(Composite).tif]

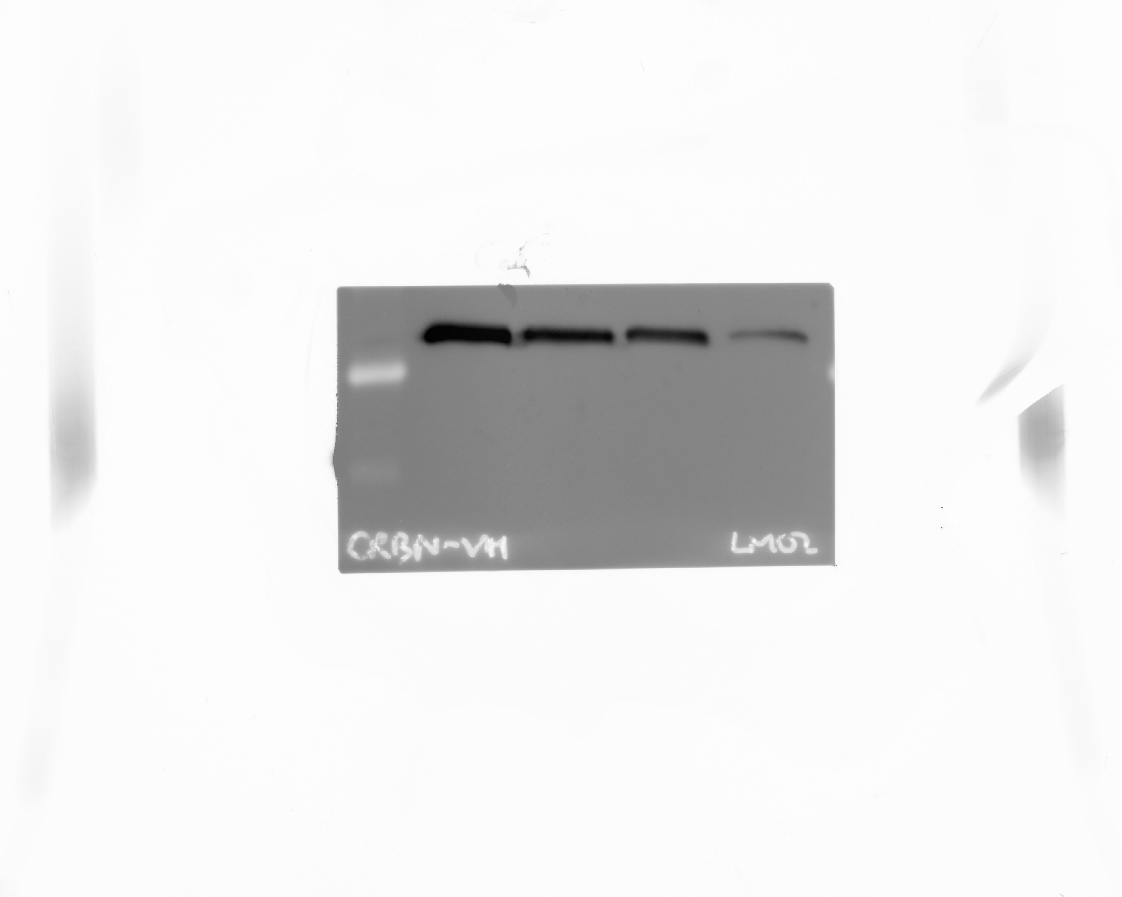

Supplement: Figure 1—figure supplement 1—source data 2. [file elife-106699-fig1-figsupp1-data2.zip › Figure 1ΓÇöfigure supplement 1-source data 2 Western blot raw data shows LMO2 protein degradation in HEK293T cells with different biodegrader contruct./LMO2 CRBN-iDab(Composite).tif]
