# Supplementary material for: Degradation of LMO2 in T cell leukaemia results in collateral breakdown of transcription complex partners and causes LMO2-dependent apoptosis
Source: eLife. 2025 Dec 12;14:RP106699. doi: 10.7554/eLife.106699 (PMC12700530; doi:10.7554/eLife.106699)
Supplement: Figure 1—figure supplement 1—source data 1. [file elife-106699-fig1-figsupp1-data1.zip › Figure 1ΓÇöfigure supplement 1-source data 1 Western blot data with label shows LMO2 protein degradation in HEK293T cells with different biodegrader contruct./Figure 1-figure supplement 1-source data 1.pdf]

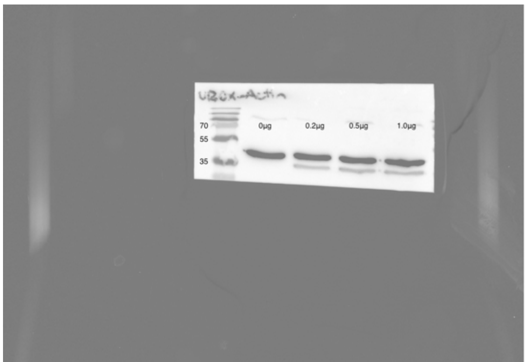

Actin iDab-UBOX

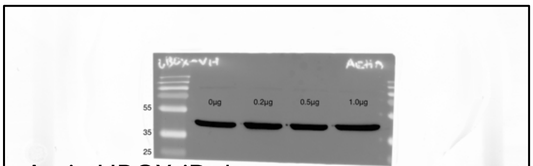

Actin UBOX-iDab

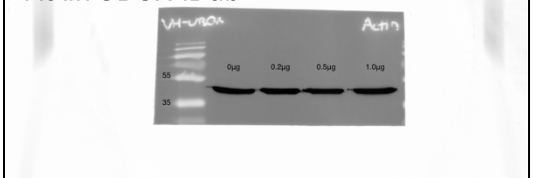

Actin iDab-UBOX

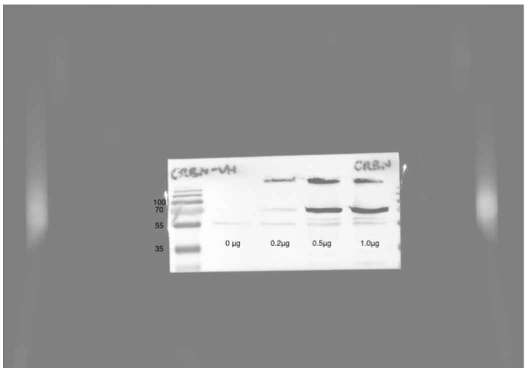

CRBN CRBN-iDab

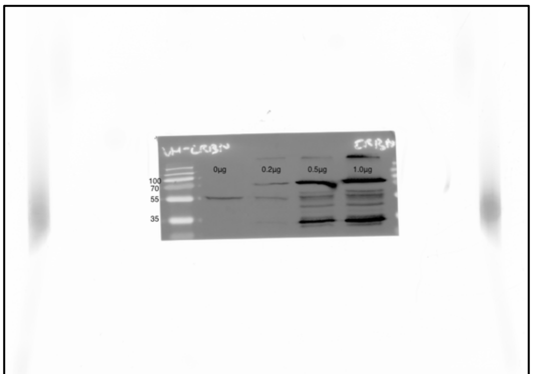

CRBN iDab-CRBN

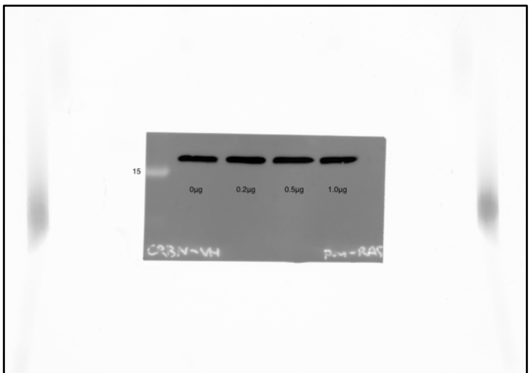

Cyclophilin CRBN-iDab

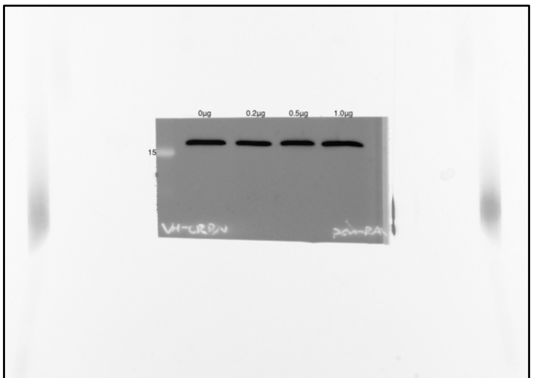

Cyclophilin iDab-CRBN

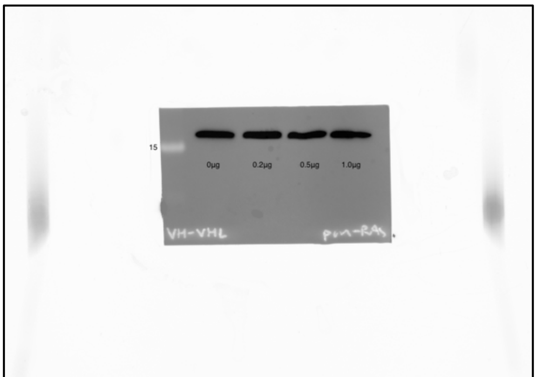

Cyclophilin iDab-VHL

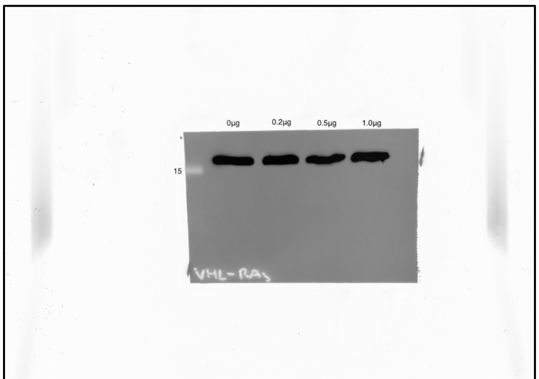

Cyclophilin VHL-iDab

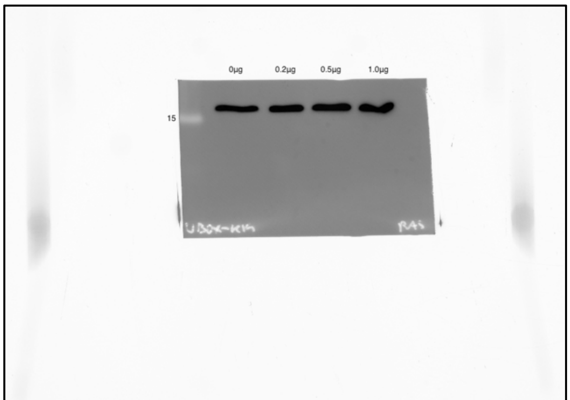

Cyclophilin VHL-iDab-Ras

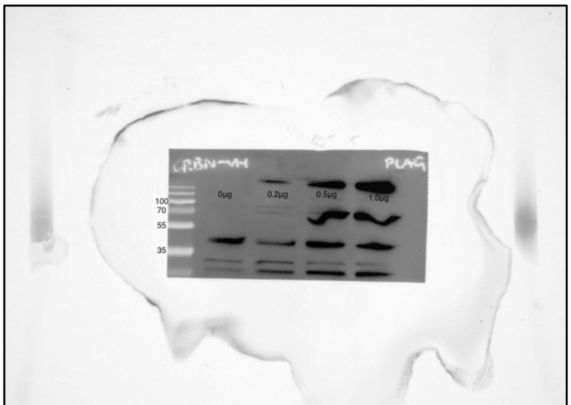

FLAG CRBN-iDab

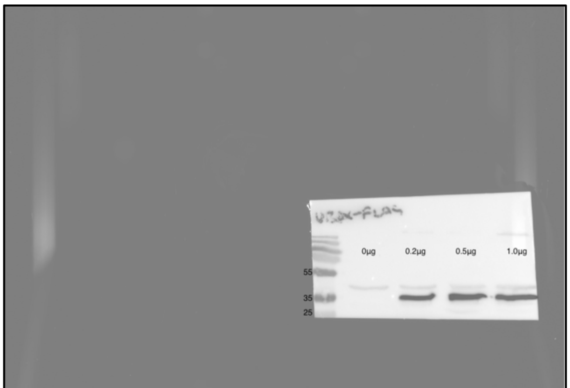

FLAG iDab-UBOX

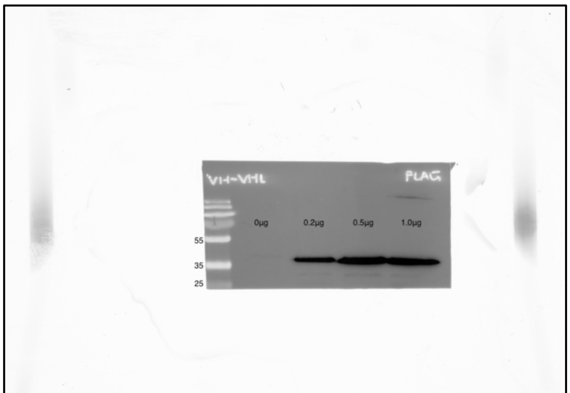

FLAG iDab-VHL

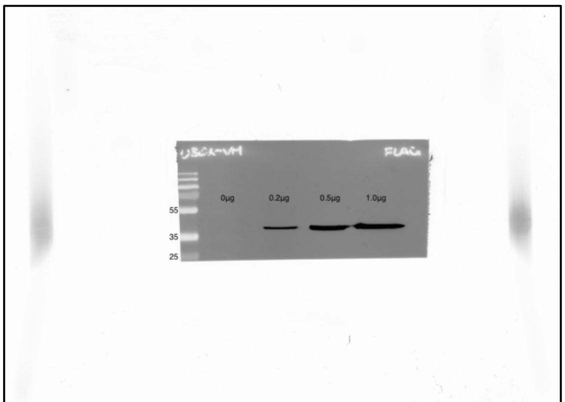

FLAG UBOX-iDab

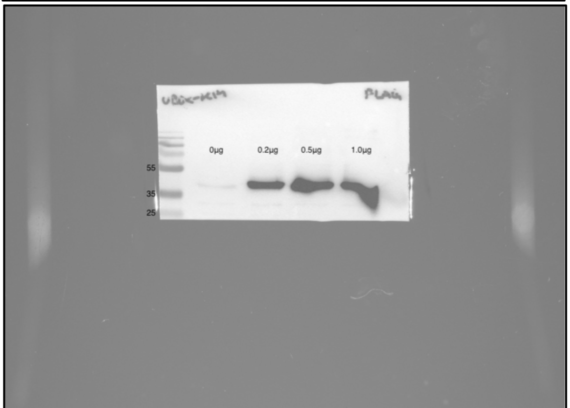

FLAG UBOX-iDabRas

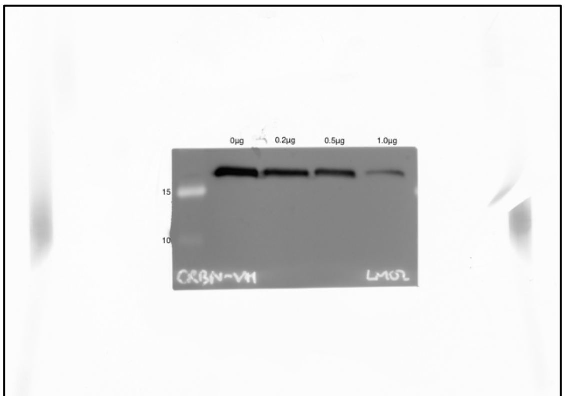

LMO2 CRBN-iDab

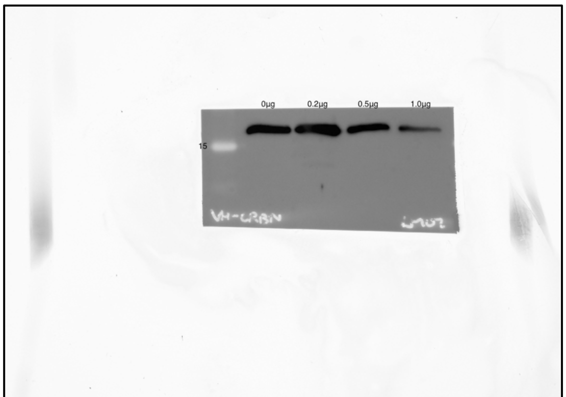

LMO2 iDab-CRBN

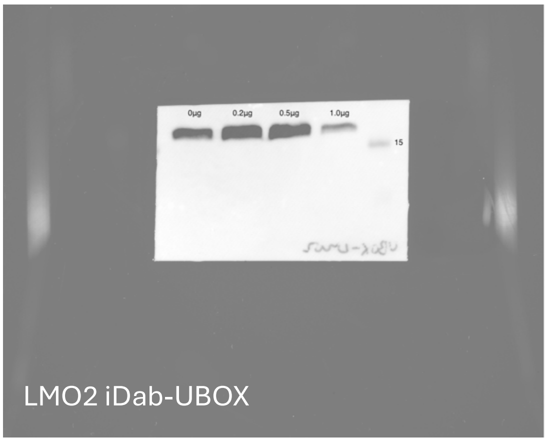
0µg
0.2µg
0.5µg
1.0µg
15
LMO2 iDab-UBOX

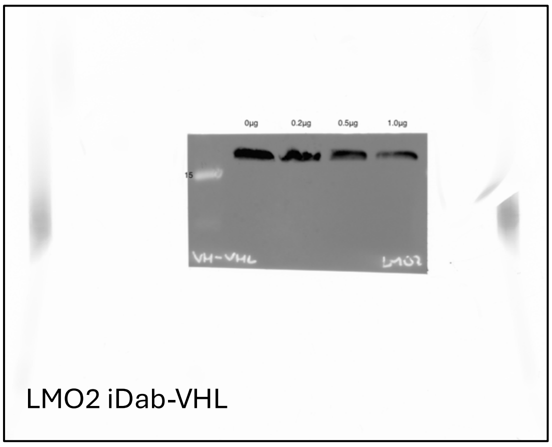
0µg
0.2µg
0.5µg
1.0µg
15
LMO2 iDab-VHL

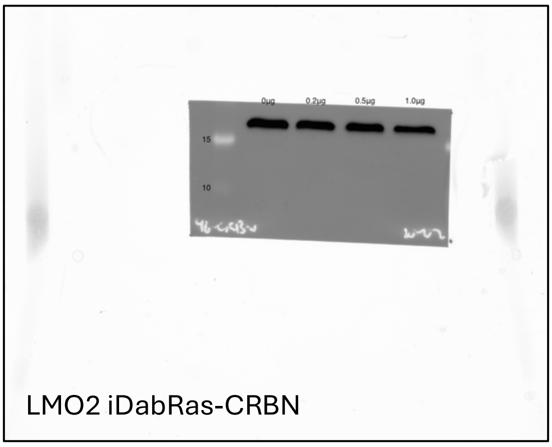
0µg
0.2µg
0.5µg
1.0µg
15
10
LMO2 iDabRas-CRBN

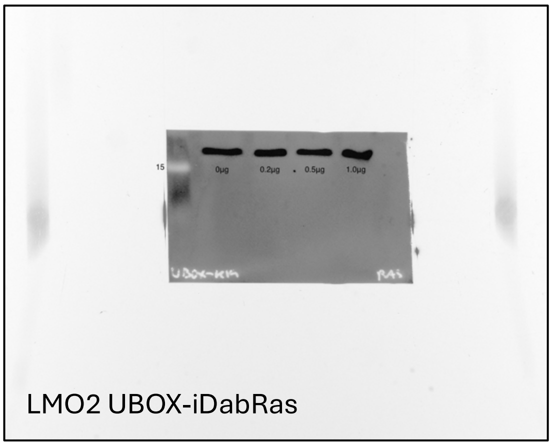
15
0µg
0.2µg
0.5µg
1.0µg
LMO2 UBOX-iDabRas

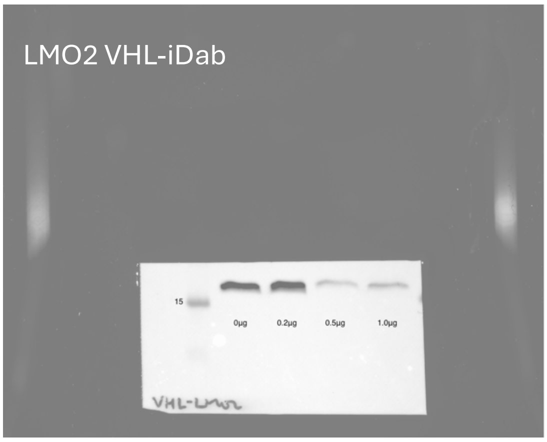
LMO2 VHL-iDab
15
0µg
0.2µg
0.5µg
1.0µg

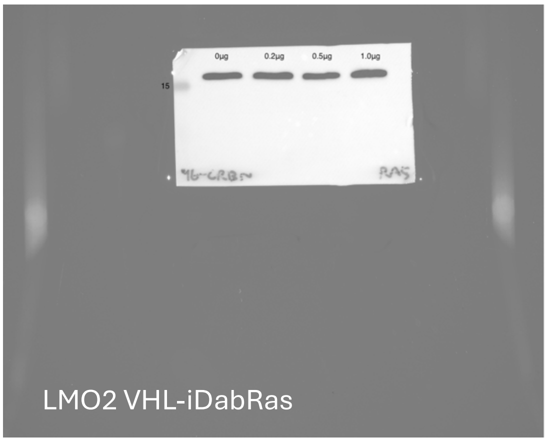
0µg
0.2µg
0.5µg
1.0µg
15
LMO2 VHL-iDabRas

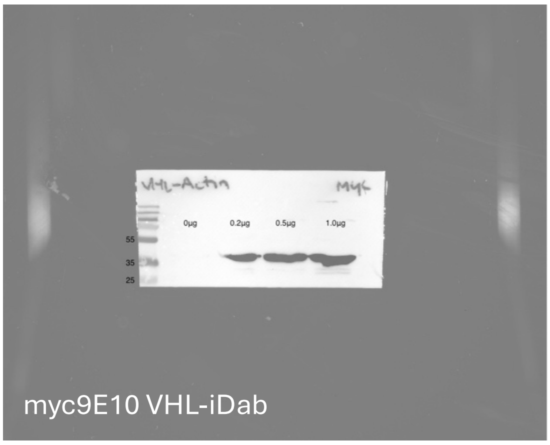
0µg
0.2µg
0.5µg
1.0µg
55
35
25
myc9E10 VHL-iDab

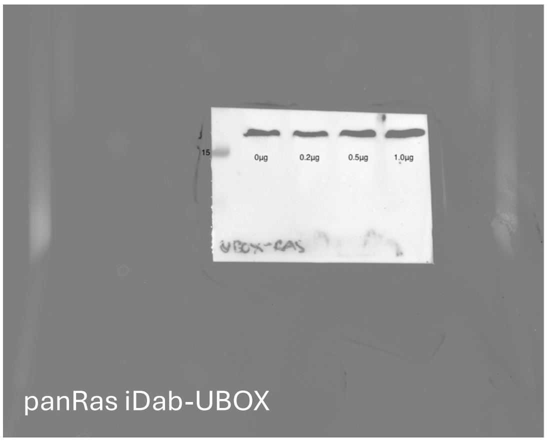
15
0µg
0.2µg
0.5µg
1.0µg
panRas iDab-UBOX

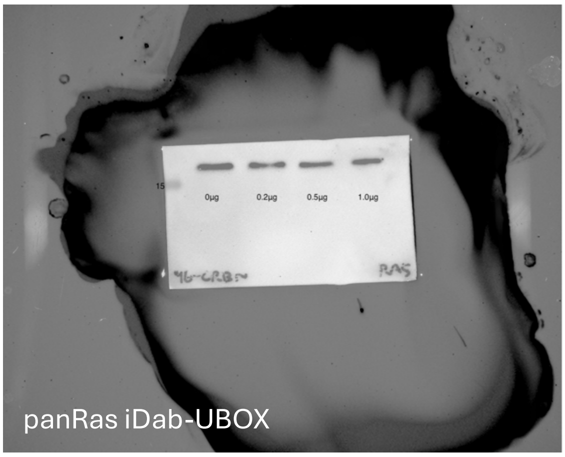
15
0μg
0.2μg
0.5μg
1.0μg
panRas iDab-UBOX

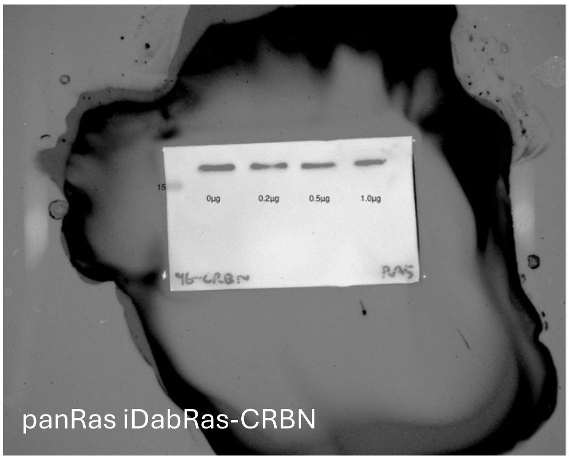
15
0μg
0.2μg
0.5μg
1.0μg
panRas iDabRas-CRBN

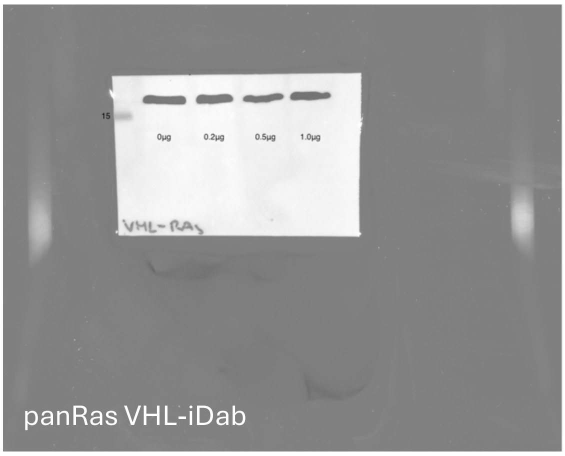
15
0μg
0.2μg
0.5μg
1.0μg
panRas VHL-iDab

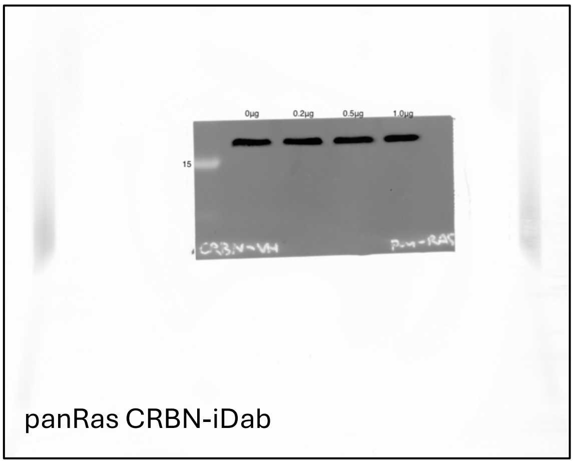
0μg
0.2μg
0.5μg
1.0μg
15
panRas CRBN-iDab

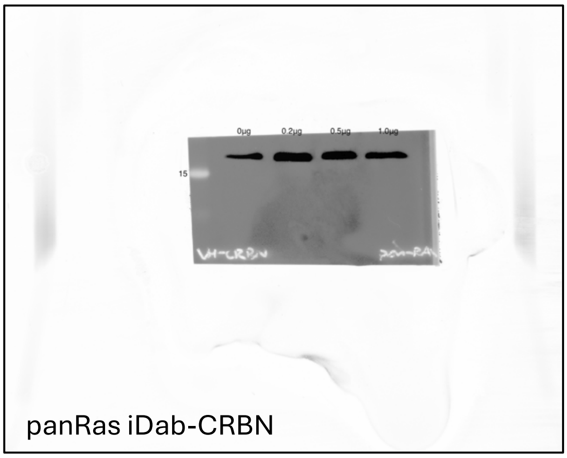
0μg
0.2μg
0.5μg
1.0μg
15
panRas iDab-CRBN

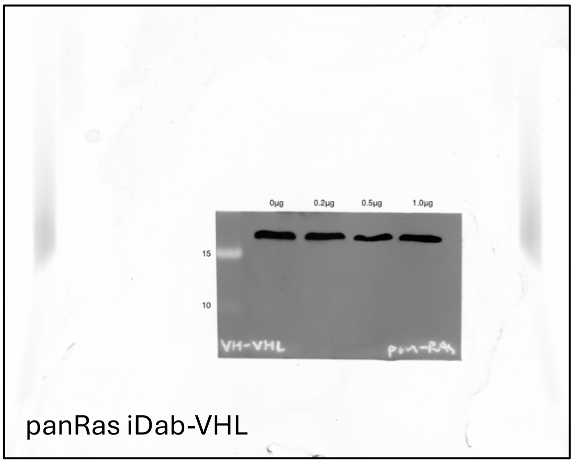
0μg
0.2μg
0.5μg
1.0μg
15
10
panRas iDab-VHL

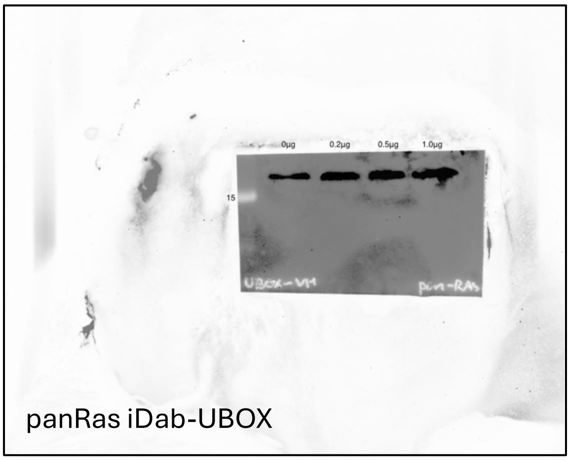
0μg
0.2μg
0.5μg
1.0μg
15
panRas iDab-UBOX

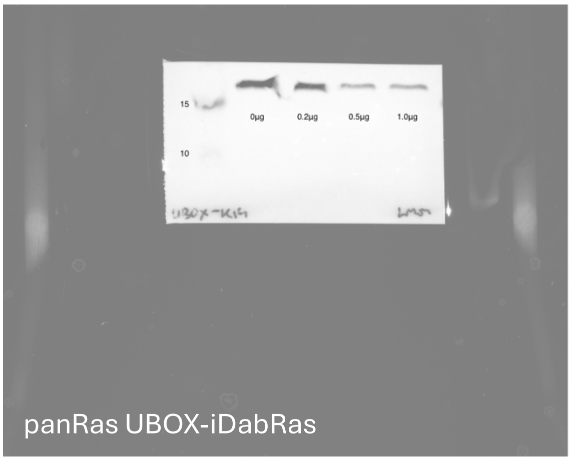
15
0μg
0.2μg
0.5μg
1.0μg
10
panRas UBOX-iDabRas

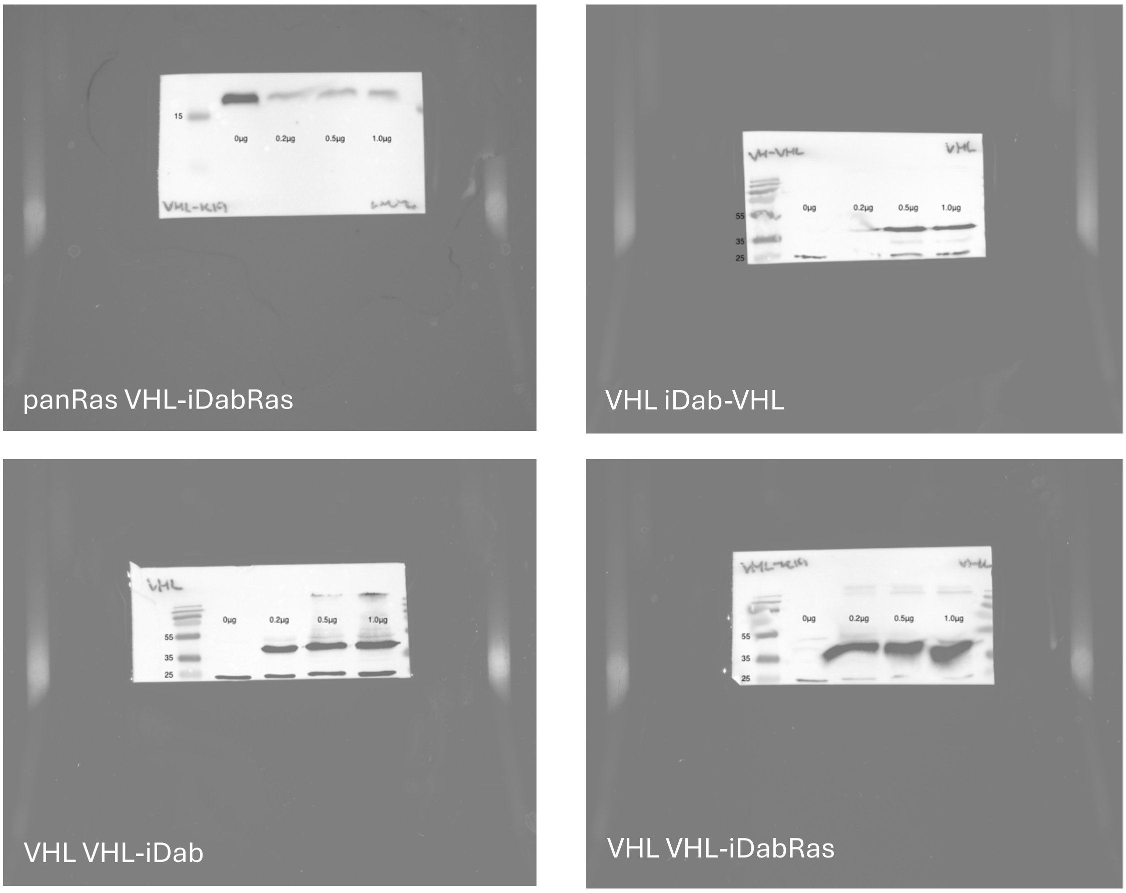

Figure 1-figure supplement 1, Source Data 1. Original membranes corresponding to Figure 1-figure supplement 1, panel A-I.
