# Supplementary material for: Degradation of LMO2 in T cell leukaemia results in collateral breakdown of transcription complex partners and causes LMO2-dependent apoptosis
Source: eLife. 2025 Dec 12;14:RP106699. doi: 10.7554/eLife.106699 (PMC12700530; doi:10.7554/eLife.106699)
Supplement: Figure 2—figure supplement 2—source data 1. [file elife-106699-fig2-figsupp2-data1.zip › Figure 2ΓÇöfigure supplement 2-source data 1 Western blot data with label shows LMO2 level in Jurkat and KOPT-K1 after the treatment from 0-20 ╬╝M at 2 and 6 hr./Figure 2-figure supplement 2-cource data 1.pdf]

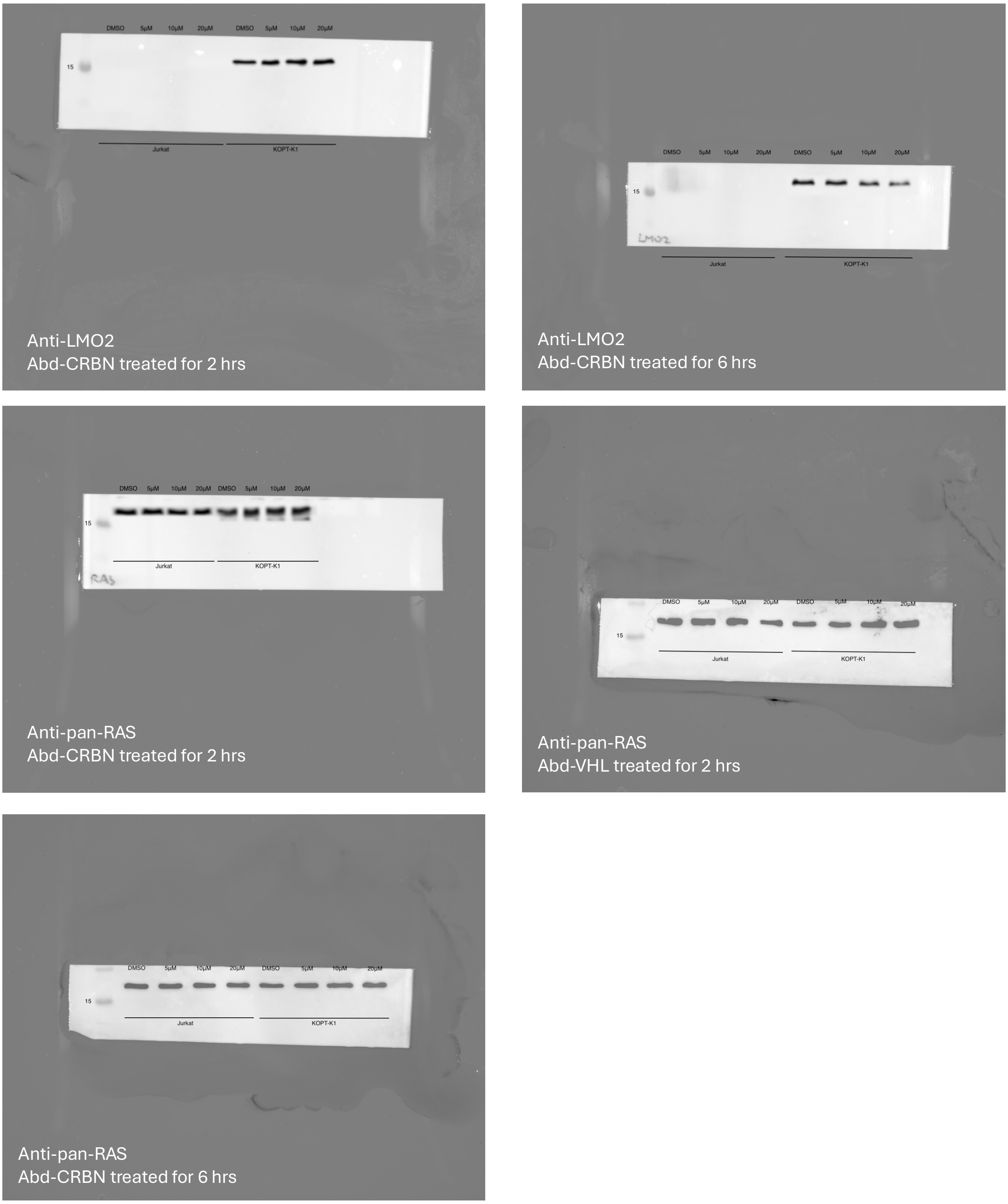

Figure 2-figure supplement 2, Source Data 1. Original membranes corresponding to Figure 2-figure supplement 2, panel A-D.
