# Supplementary figures and images for: Degradation of LMO2 in T cell leukaemia results in collateral breakdown of transcription complex partners and causes LMO2-dependent apoptosis (part 2 of 5)
Source: eLife. 2025 Dec 12;14:RP106699. doi: 10.7554/eLife.106699 (PMC12700530; doi:10.7554/eLife.106699)

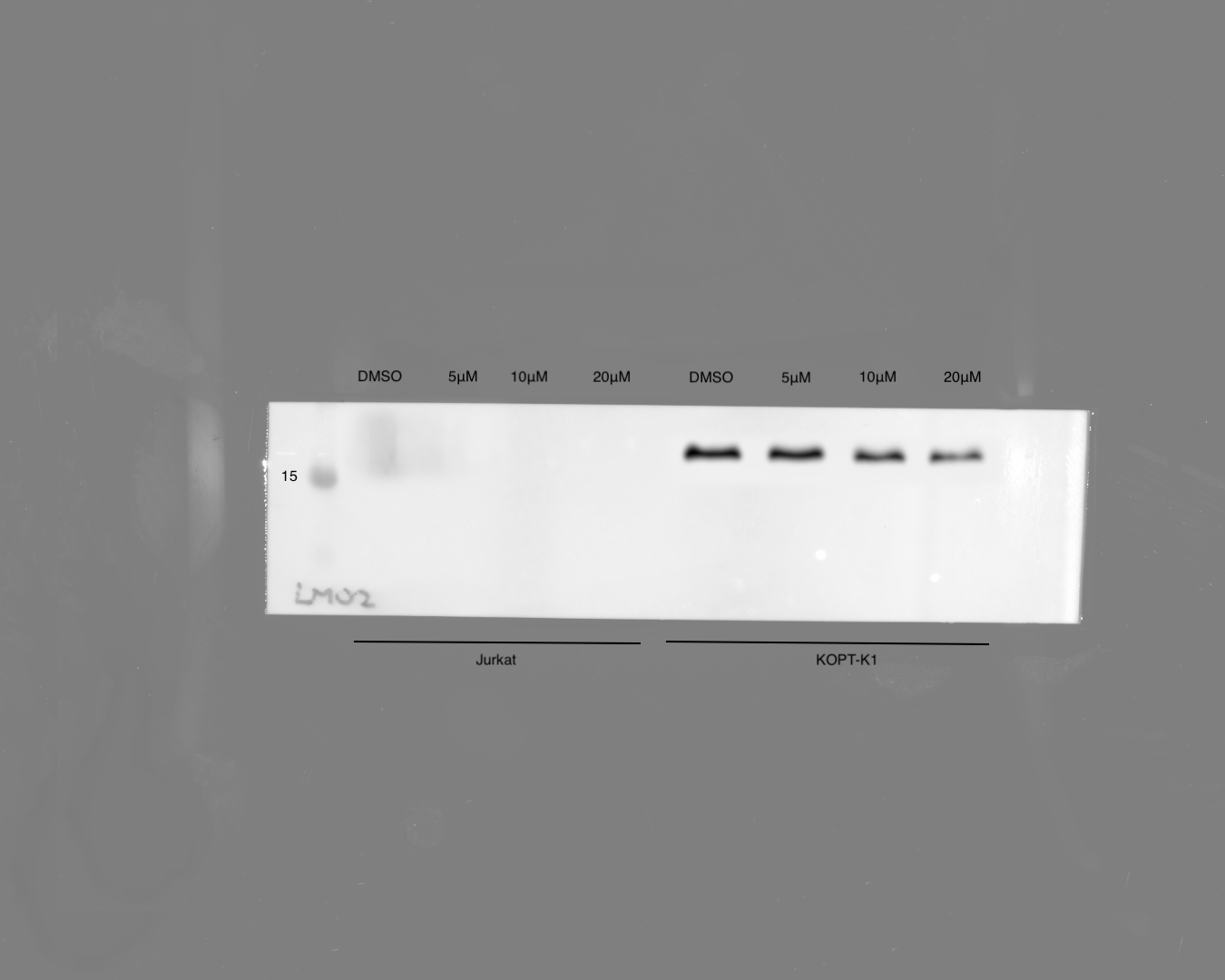

Supplement: Figure 2—figure supplement 2—source data 1. [file elife-106699-fig2-figsupp2-data1.zip › Figure 2ΓÇöfigure supplement 2-source data 1 Western blot data with label shows LMO2 level in Jurkat and KOPT-K1 after the treatment from 0-20 ╬╝M at 2 and 6 hr./Raw data /LMO2 Abd-CRBN at 6 hr(Composite).tif]

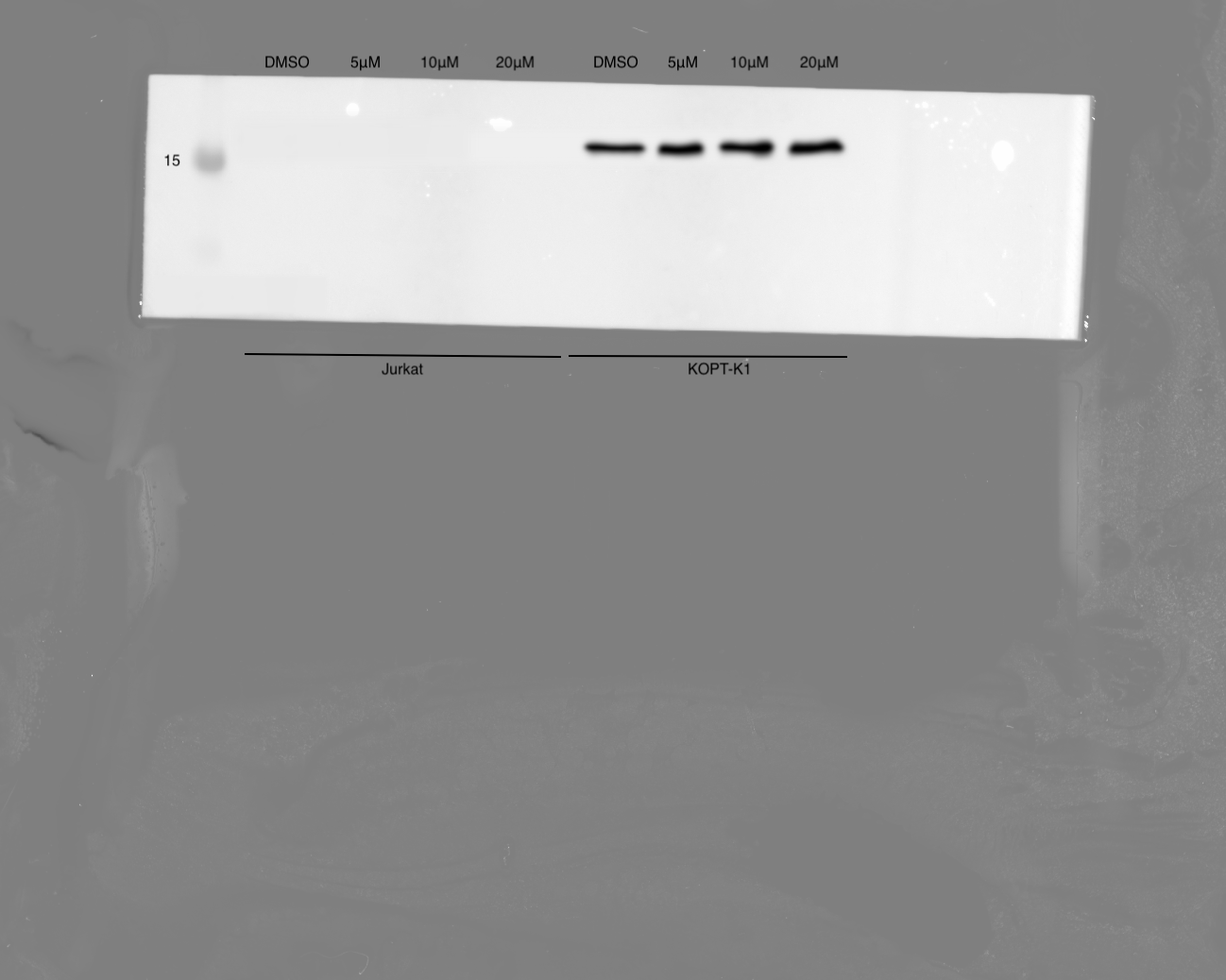

Supplement: Figure 2—figure supplement 2—source data 1. [file elife-106699-fig2-figsupp2-data1.zip › Figure 2ΓÇöfigure supplement 2-source data 1 Western blot data with label shows LMO2 level in Jurkat and KOPT-K1 after the treatment from 0-20 ╬╝M at 2 and 6 hr./Raw data /LMO2 Abd-CRBN at 2 hr (Composite).tif]

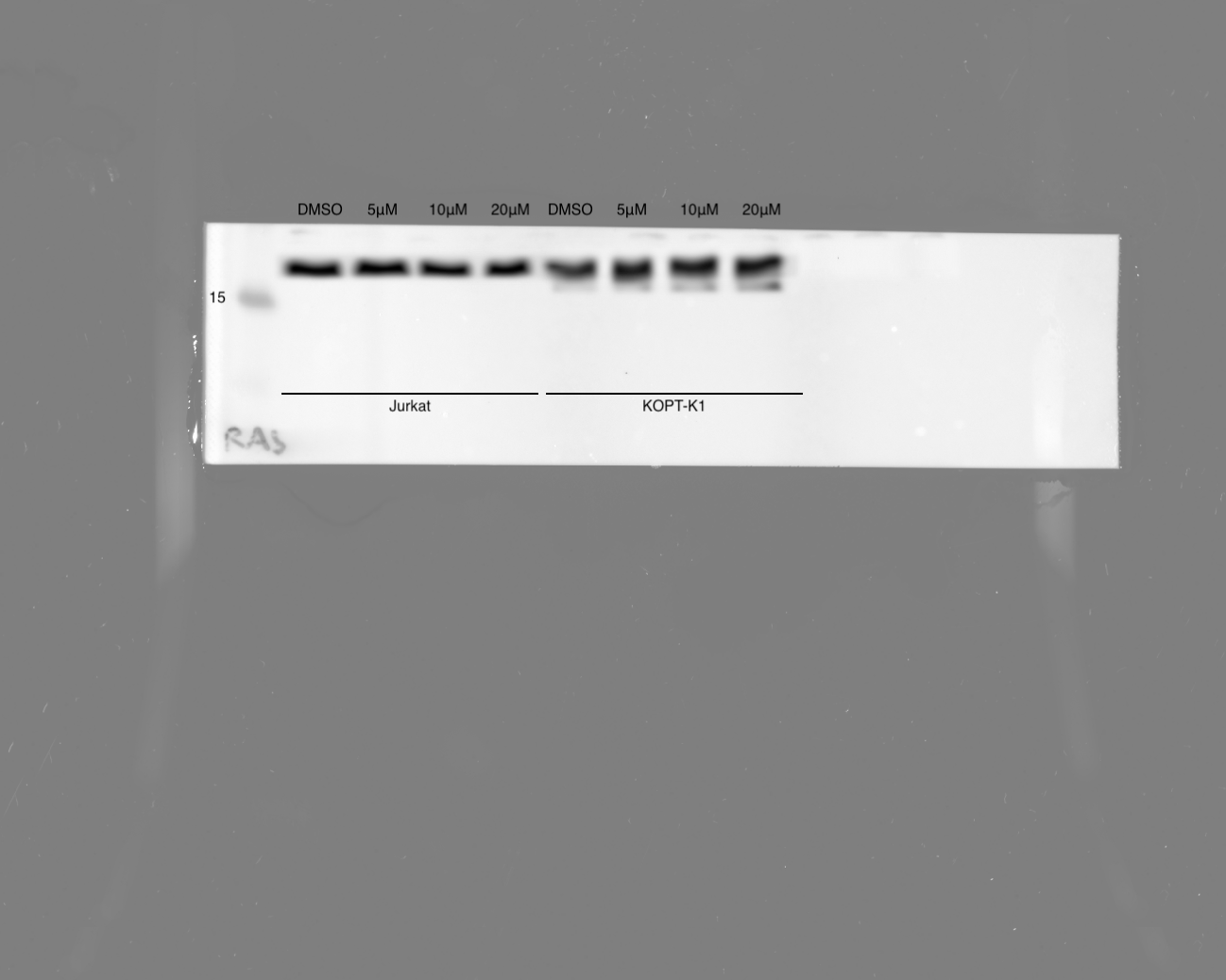

Supplement: Figure 2—figure supplement 2—source data 1. [file elife-106699-fig2-figsupp2-data1.zip › Figure 2ΓÇöfigure supplement 2-source data 1 Western blot data with label shows LMO2 level in Jurkat and KOPT-K1 after the treatment from 0-20 ╬╝M at 2 and 6 hr./Raw data /pan-RAS Abd-CRBN at 2 hr(Composite).tif]

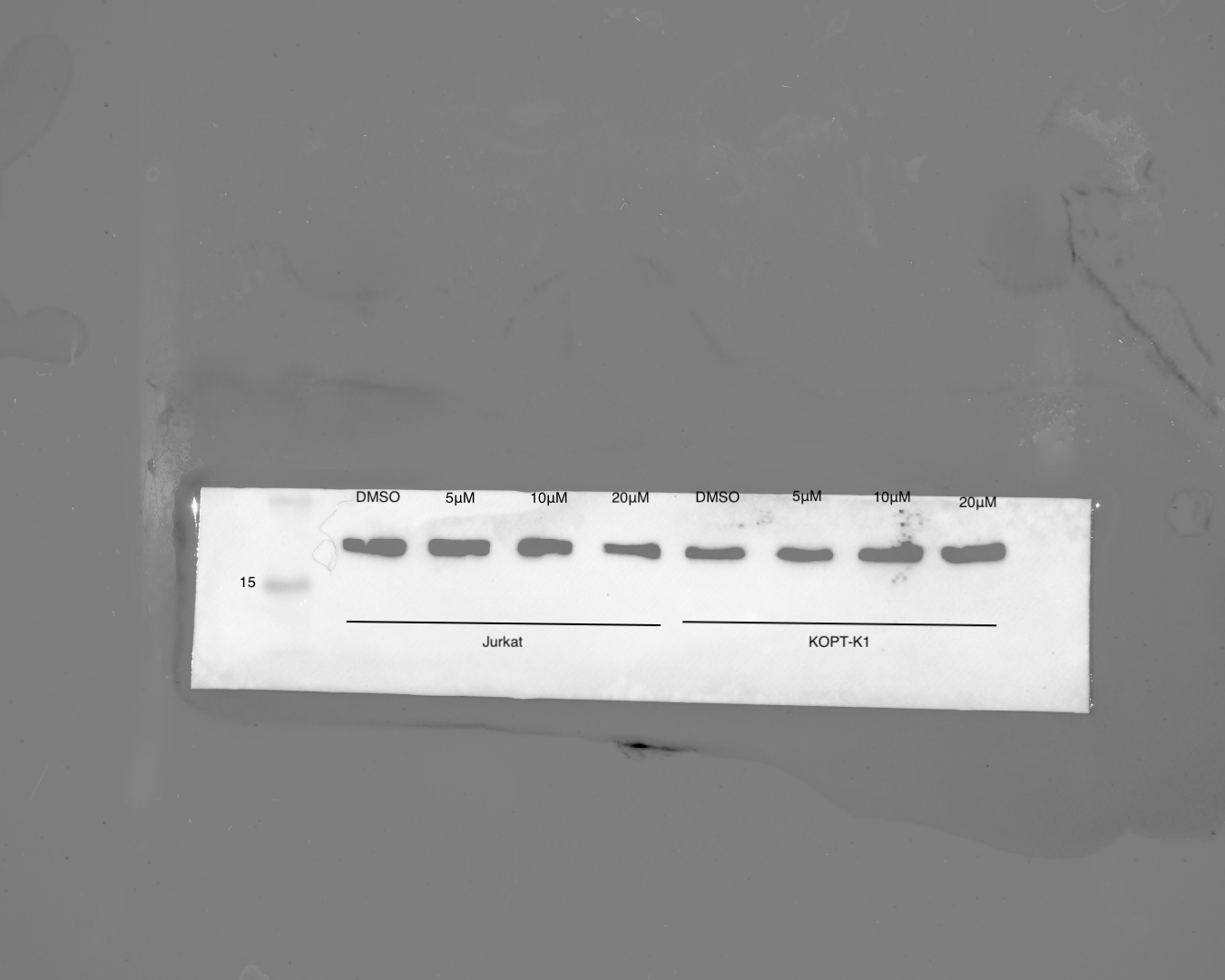

Supplement: Figure 2—figure supplement 2—source data 1. [file elife-106699-fig2-figsupp2-data1.zip › Figure 2ΓÇöfigure supplement 2-source data 1 Western blot data with label shows LMO2 level in Jurkat and KOPT-K1 after the treatment from 0-20 ╬╝M at 2 and 6 hr./Raw data /pan-RAS Abd-VHL at 2 hr (Composite).tif]

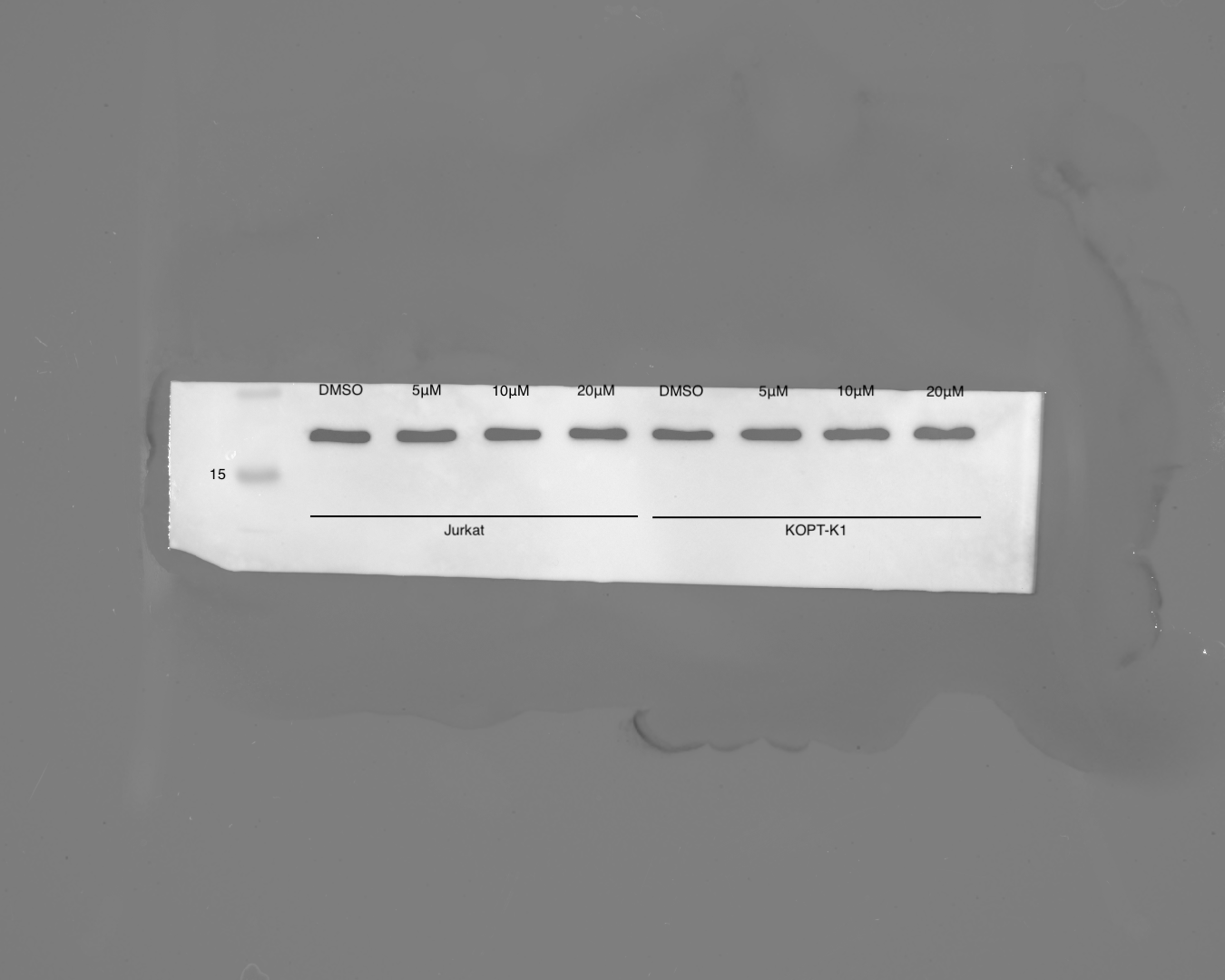

Supplement: Figure 2—figure supplement 2—source data 1. [file elife-106699-fig2-figsupp2-data1.zip › Figure 2ΓÇöfigure supplement 2-source data 1 Western blot data with label shows LMO2 level in Jurkat and KOPT-K1 after the treatment from 0-20 ╬╝M at 2 and 6 hr./Raw data /pan-RAS Abd-VHL at 6 hr(Composite).tif]

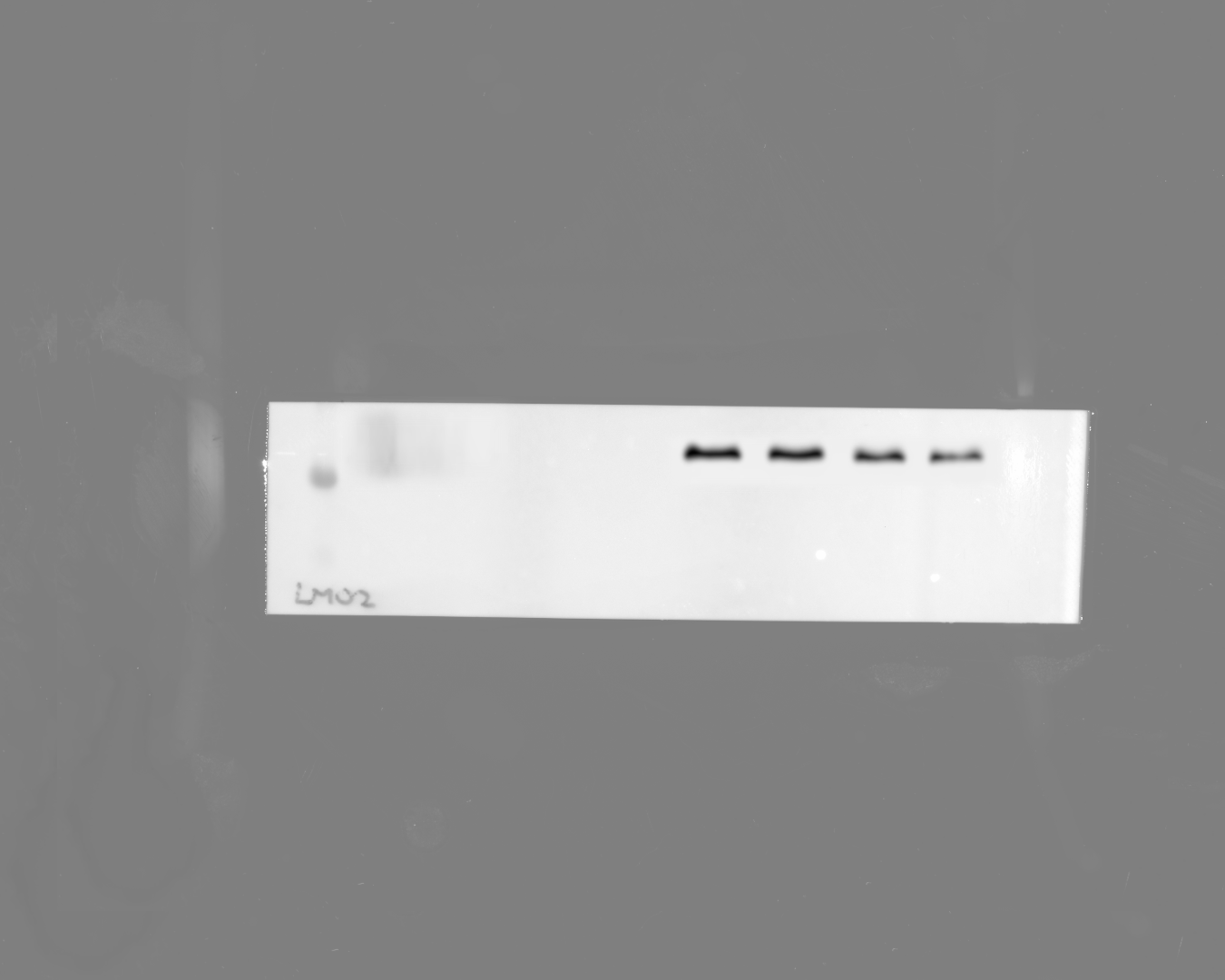

Supplement: Figure 2—figure supplement 2—source data 2. [file elife-106699-fig2-figsupp2-data2.zip › Figure 2ΓÇöfigure supplement 2-source data 2 Western blot raw data shows LMO2 level in Jurkat and KOPT-K1 after the treatment from 0-20 ╬╝M at 2 and 6 hr./LMO2 Abd-CRBN at 6 hr(Composite).tif]

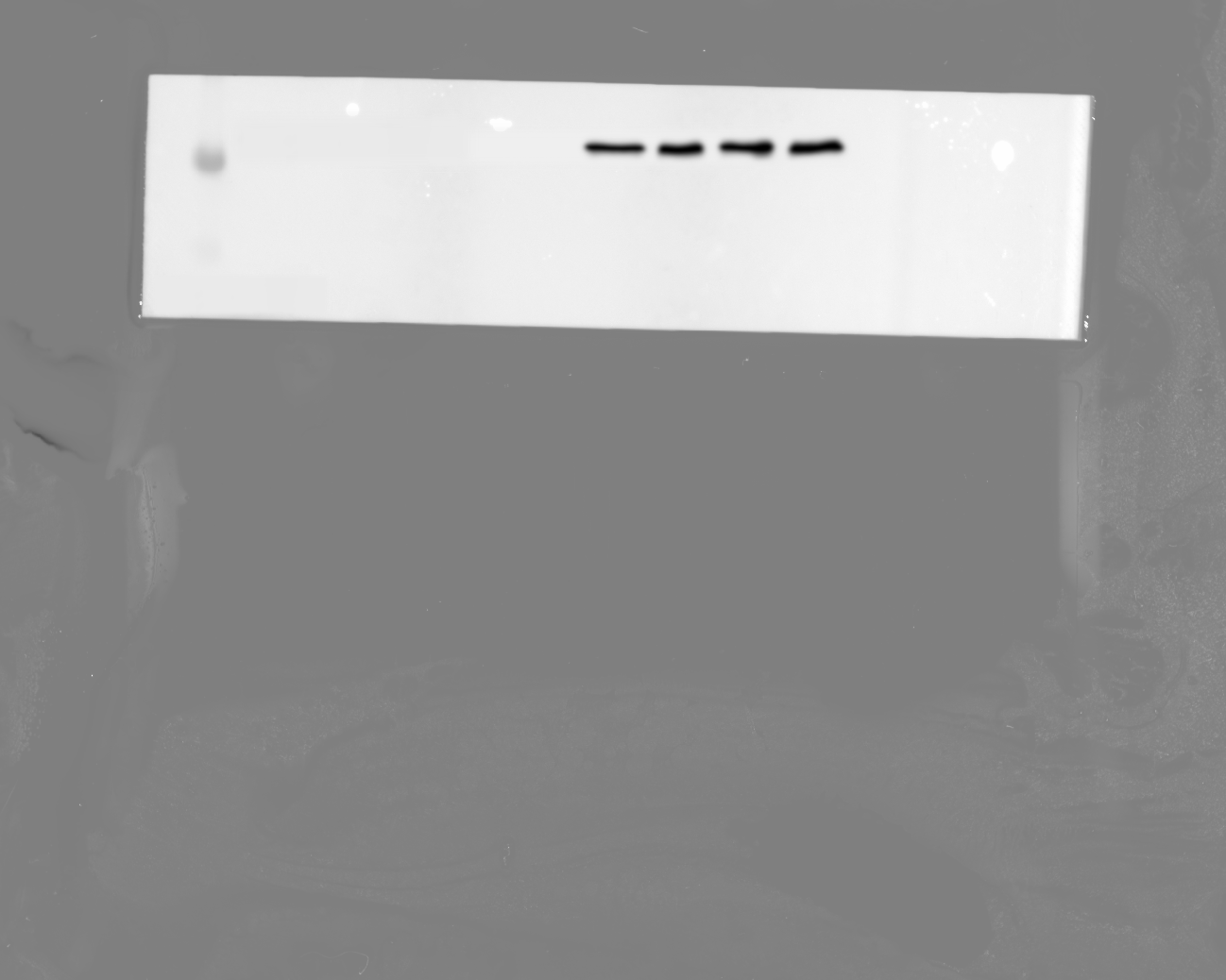

Supplement: Figure 2—figure supplement 2—source data 2. [file elife-106699-fig2-figsupp2-data2.zip › Figure 2ΓÇöfigure supplement 2-source data 2 Western blot raw data shows LMO2 level in Jurkat and KOPT-K1 after the treatment from 0-20 ╬╝M at 2 and 6 hr./LMO2 Abd-CRBN at 2 hr (Composite).tif]

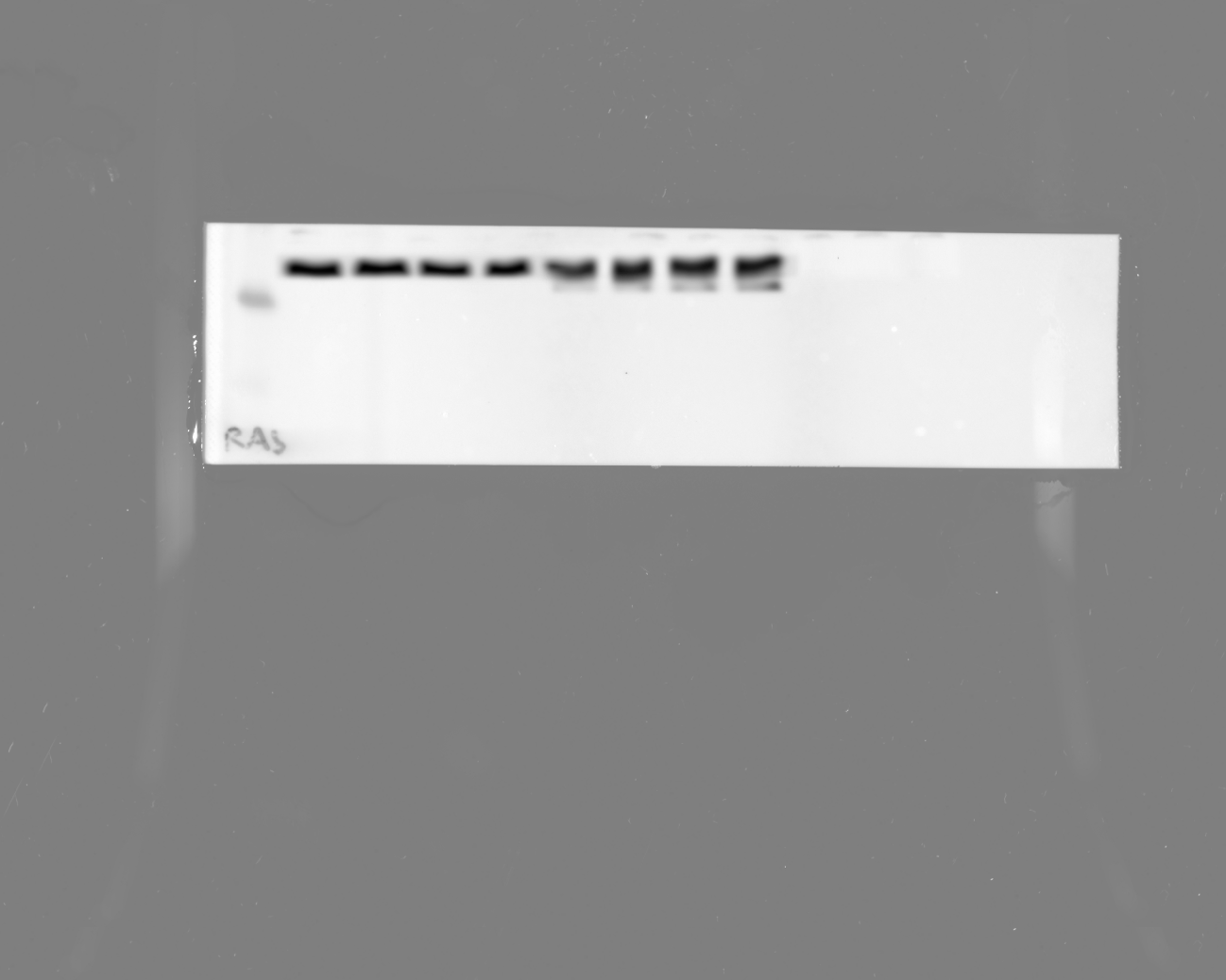

Supplement: Figure 2—figure supplement 2—source data 2. [file elife-106699-fig2-figsupp2-data2.zip › Figure 2ΓÇöfigure supplement 2-source data 2 Western blot raw data shows LMO2 level in Jurkat and KOPT-K1 after the treatment from 0-20 ╬╝M at 2 and 6 hr./pan-RAS Abd-CRBN at 2 hr(Composite).tif]

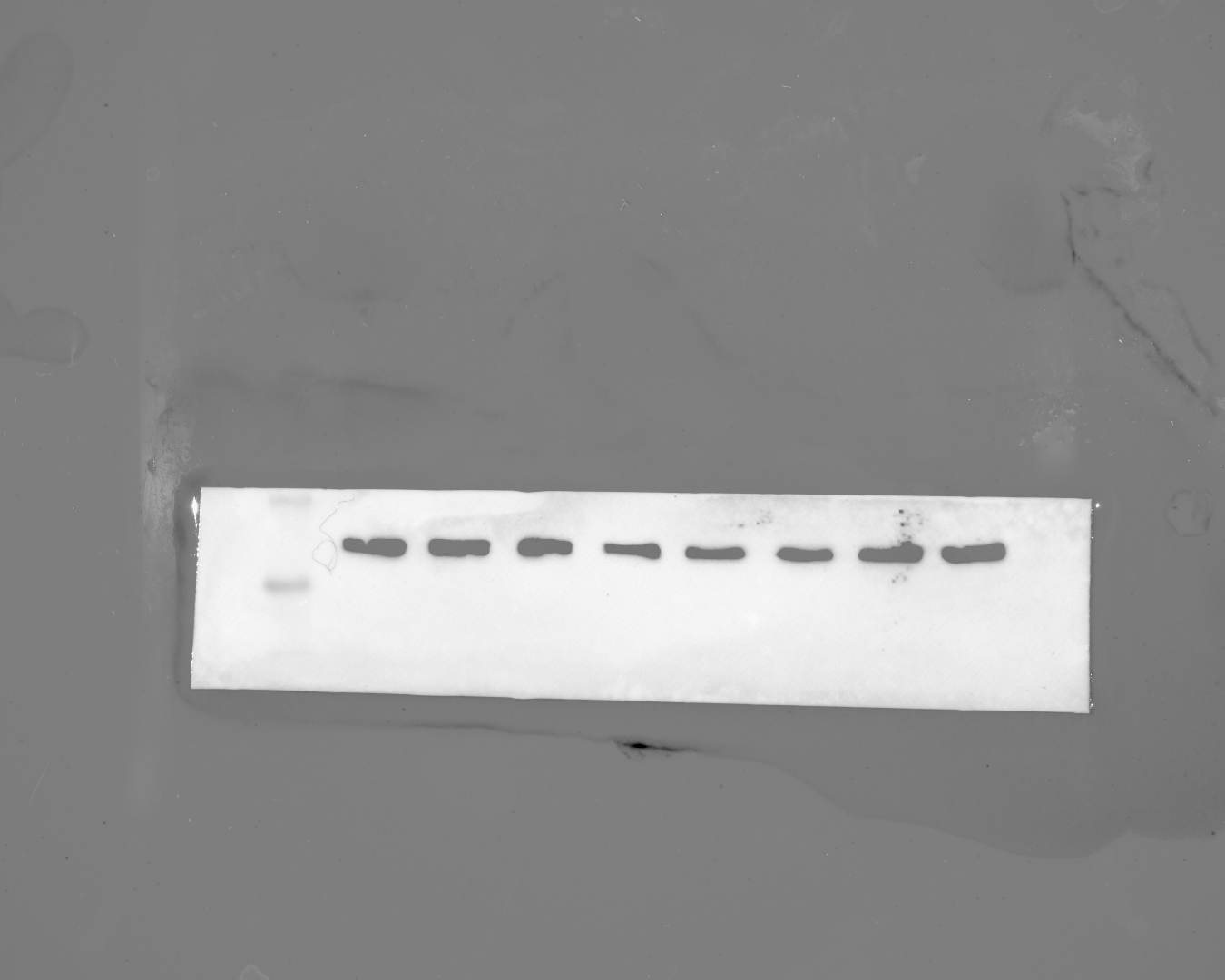

Supplement: Figure 2—figure supplement 2—source data 2. [file elife-106699-fig2-figsupp2-data2.zip › Figure 2ΓÇöfigure supplement 2-source data 2 Western blot raw data shows LMO2 level in Jurkat and KOPT-K1 after the treatment from 0-20 ╬╝M at 2 and 6 hr./pan-RAS Abd-VHL at 2 hr (Composite).tif]

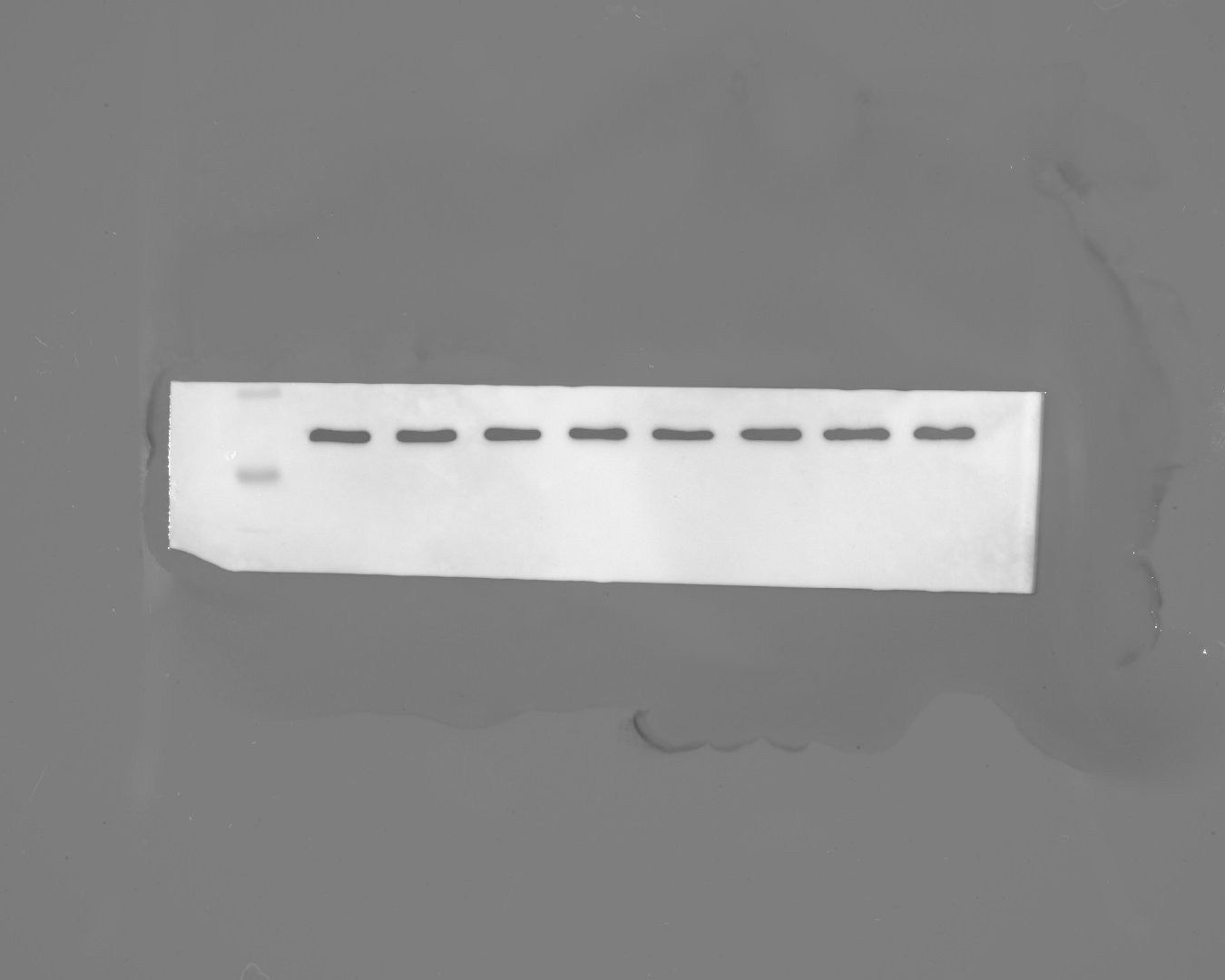

Supplement: Figure 2—figure supplement 2—source data 2. [file elife-106699-fig2-figsupp2-data2.zip › Figure 2ΓÇöfigure supplement 2-source data 2 Western blot raw data shows LMO2 level in Jurkat and KOPT-K1 after the treatment from 0-20 ╬╝M at 2 and 6 hr./pan-RAS Abd-VHL at 6 hr(Composite).tif]

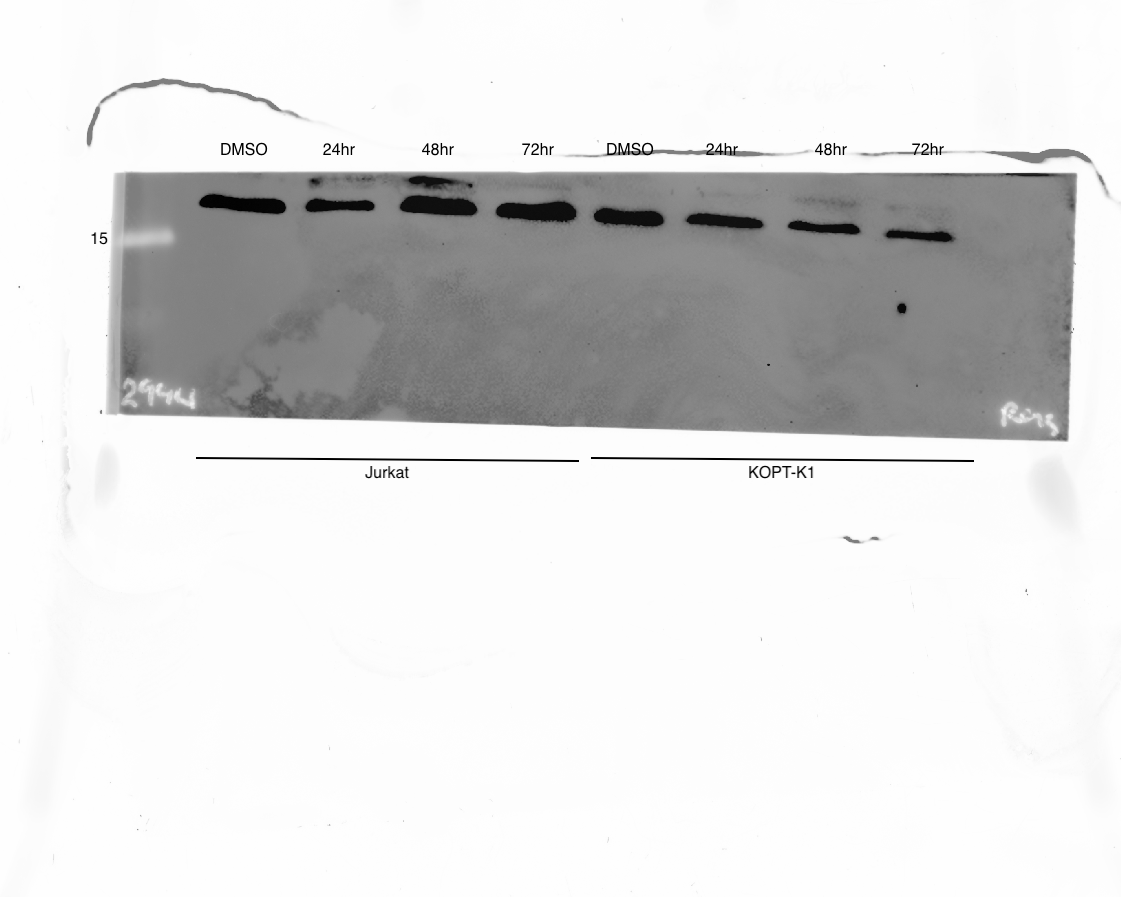

Supplement: Figure 2—figure supplement 5—source data 1. [file elife-106699-fig2-figsupp5-data1.zip › Figure 2ΓÇöfigure supplement 5-source data 1 Western blot data with label shows the longevity of responses of KOPT-K1 cells to single dose treatment./Raw data/pan-RAS Jurkat&KOP Abd-VHL (Composite).tif]

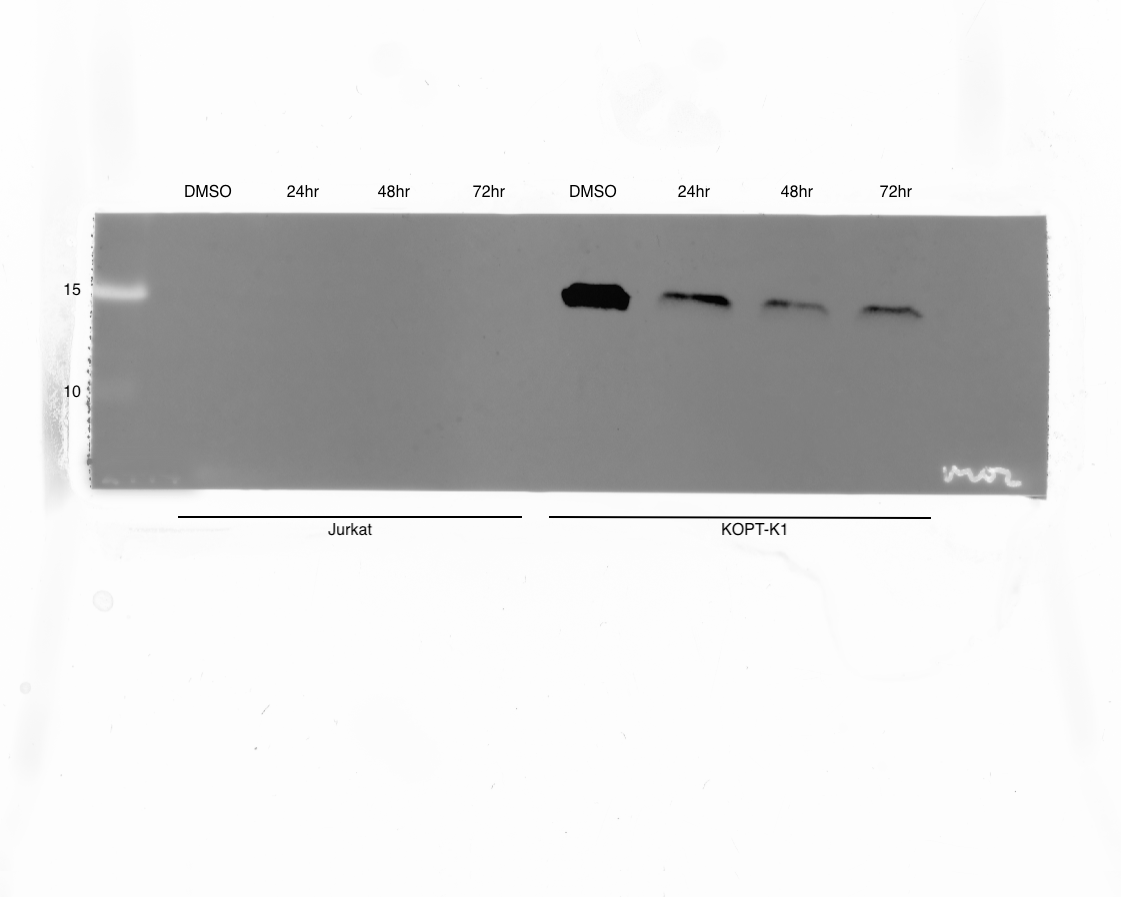

Supplement: Figure 2—figure supplement 5—source data 1. [file elife-106699-fig2-figsupp5-data1.zip › Figure 2ΓÇöfigure supplement 5-source data 1 Western blot data with label shows the longevity of responses of KOPT-K1 cells to single dose treatment./Raw data/LMO2 Jurkat&KOP Abd-CRBN (Composite).tif]

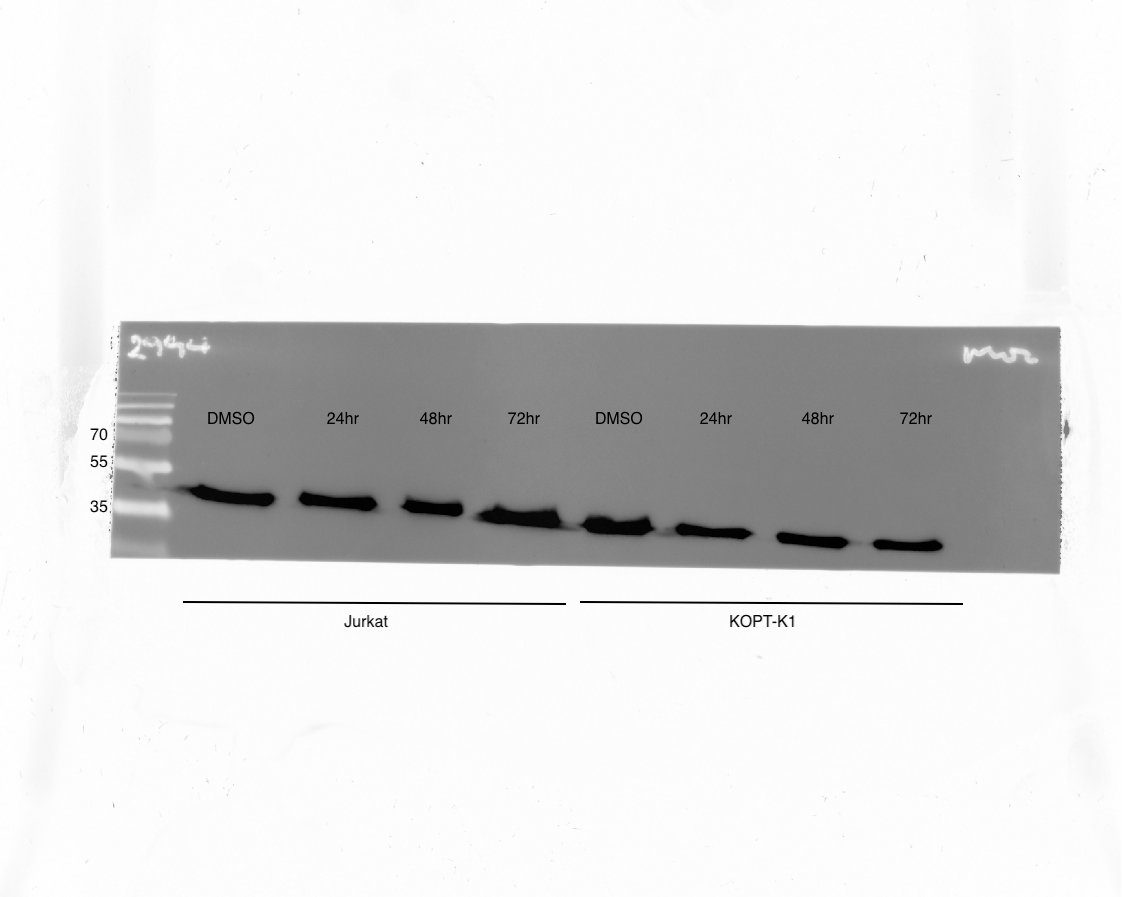

Supplement: Figure 2—figure supplement 5—source data 1. [file elife-106699-fig2-figsupp5-data1.zip › Figure 2ΓÇöfigure supplement 5-source data 1 Western blot data with label shows the longevity of responses of KOPT-K1 cells to single dose treatment./Raw data/Actin Jurkat&KOP Abd-VHL (Composite).tif]

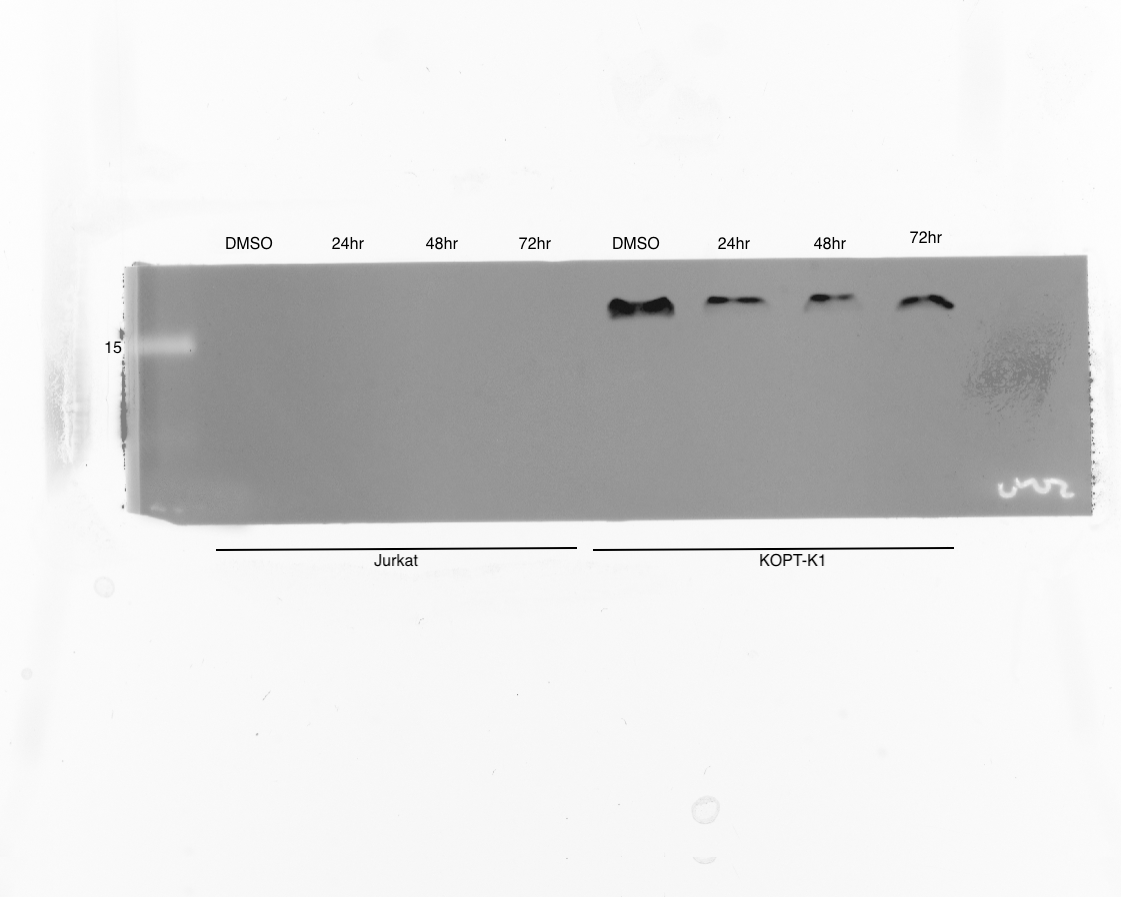

Supplement: Figure 2—figure supplement 5—source data 1. [file elife-106699-fig2-figsupp5-data1.zip › Figure 2ΓÇöfigure supplement 5-source data 1 Western blot data with label shows the longevity of responses of KOPT-K1 cells to single dose treatment./Raw data/LMO2 Jurkat&KOP Abd-VHL (Composite).tif]

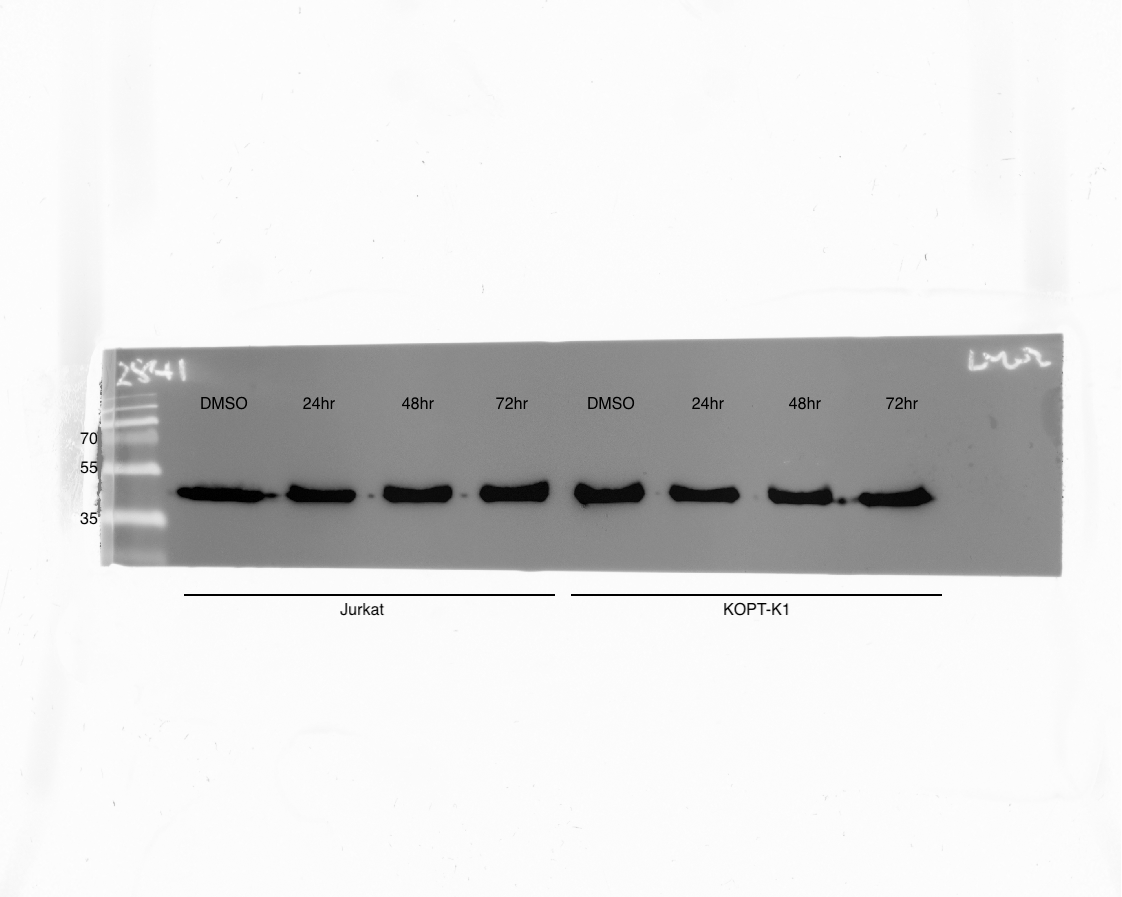

Supplement: Figure 2—figure supplement 5—source data 1. [file elife-106699-fig2-figsupp5-data1.zip › Figure 2ΓÇöfigure supplement 5-source data 1 Western blot data with label shows the longevity of responses of KOPT-K1 cells to single dose treatment./Raw data/Actin Jurkat&KOP Abd-CRBN (Composite).tif]

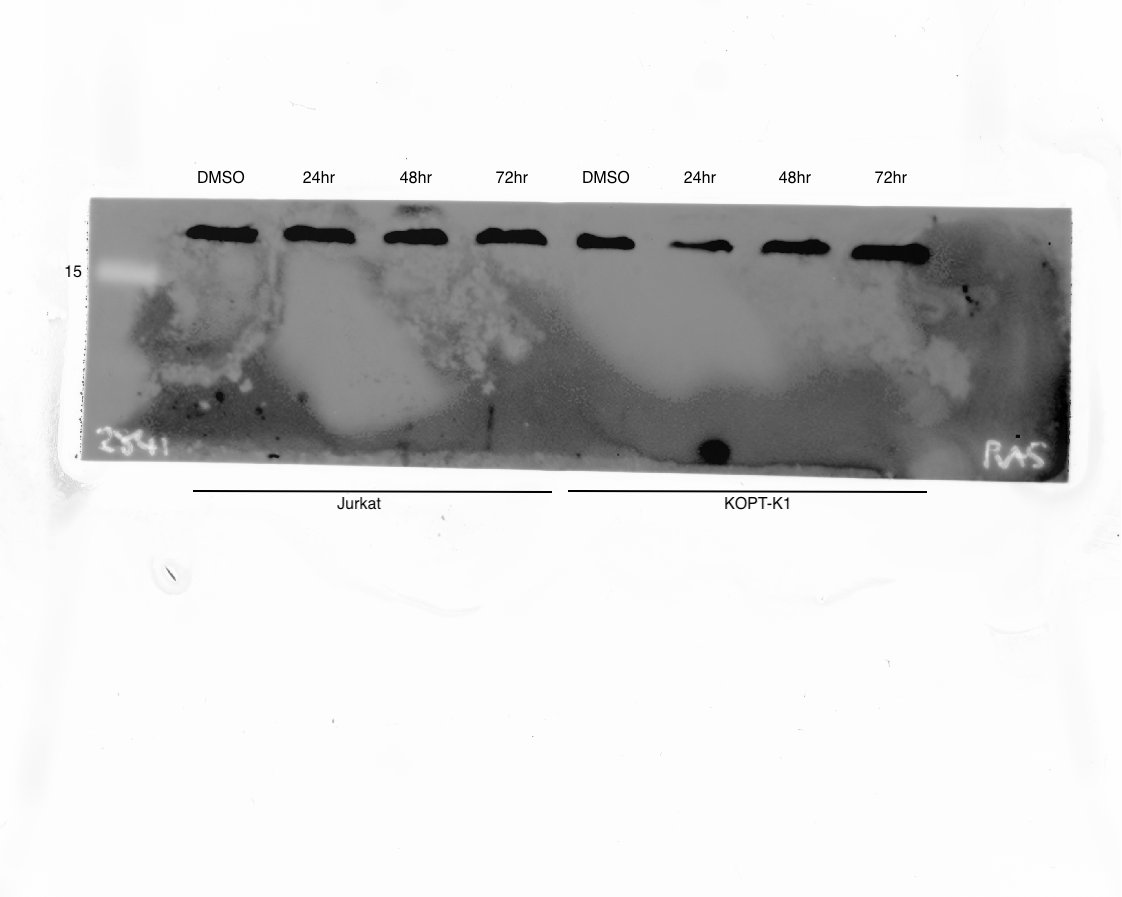

Supplement: Figure 2—figure supplement 5—source data 1. [file elife-106699-fig2-figsupp5-data1.zip › Figure 2ΓÇöfigure supplement 5-source data 1 Western blot data with label shows the longevity of responses of KOPT-K1 cells to single dose treatment./Raw data/pan-RAS Jurkat&KOP Abd-CRBN (Composite).tif]

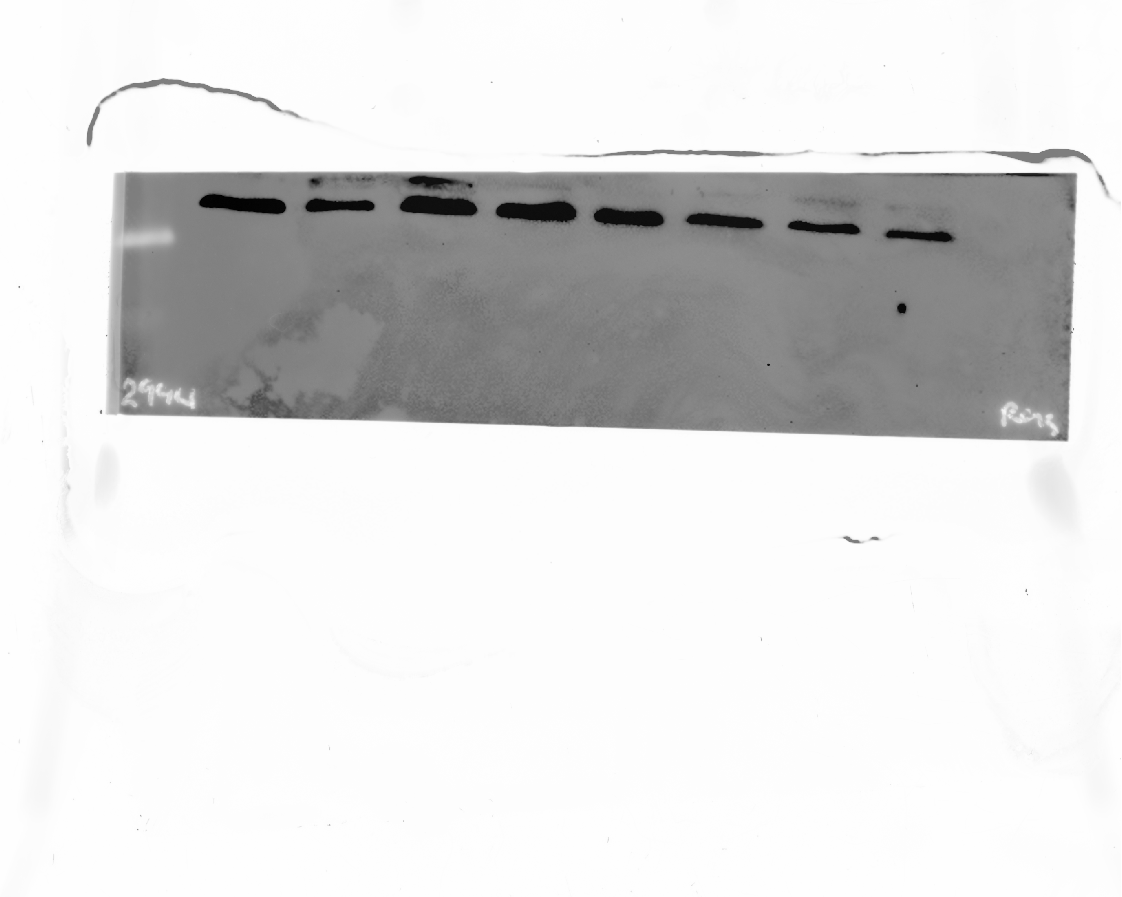

Supplement: Figure 2—figure supplement 5—source data 2. [file elife-106699-fig2-figsupp5-data2.zip › Figure 2ΓÇöfigure supplement 5-source data 2 Western blot raw data shows the longevity of responses of KOPT-K1 cells to single dose treatment./pan-RAS Jurkat&KOP Abd-VHL (Composite).tif]

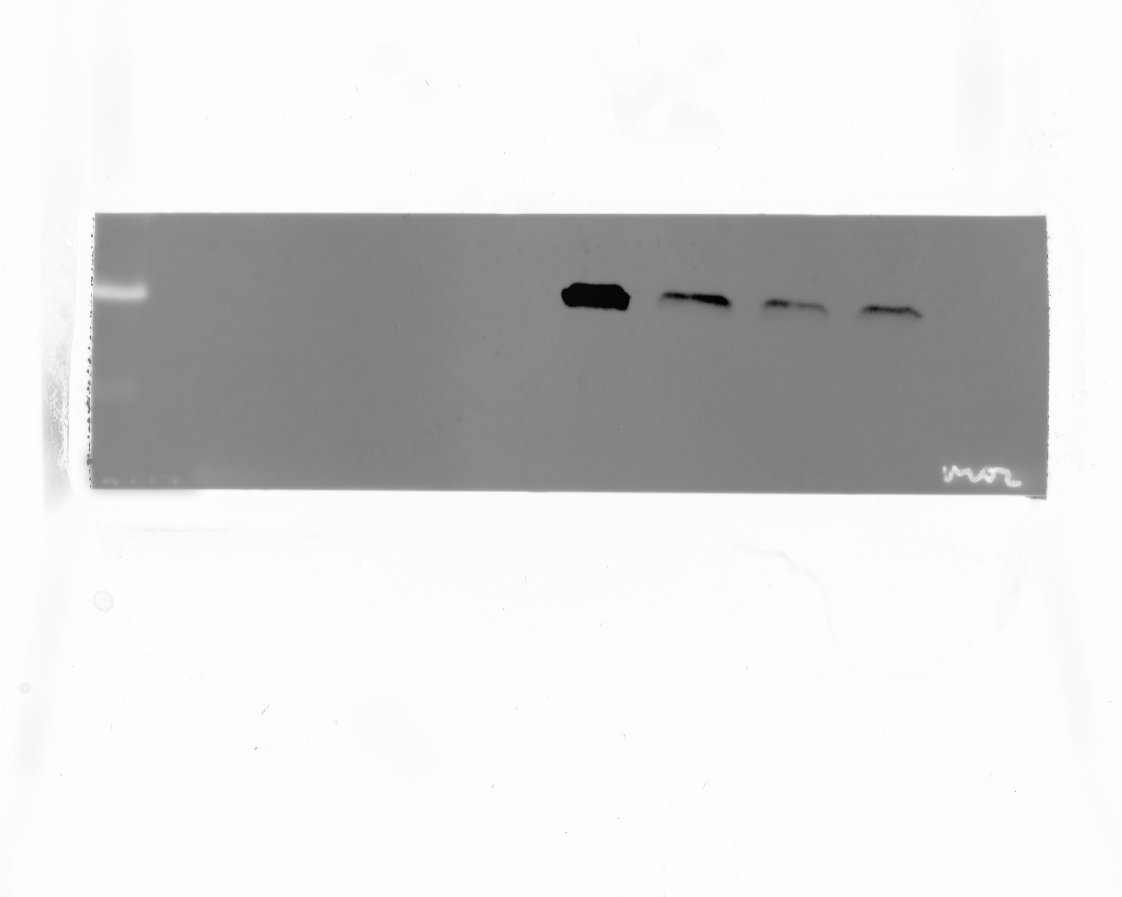

Supplement: Figure 2—figure supplement 5—source data 2. [file elife-106699-fig2-figsupp5-data2.zip › Figure 2ΓÇöfigure supplement 5-source data 2 Western blot raw data shows the longevity of responses of KOPT-K1 cells to single dose treatment./LMO2 Jurkat&KOP Abd-CRBN (Composite).tif]

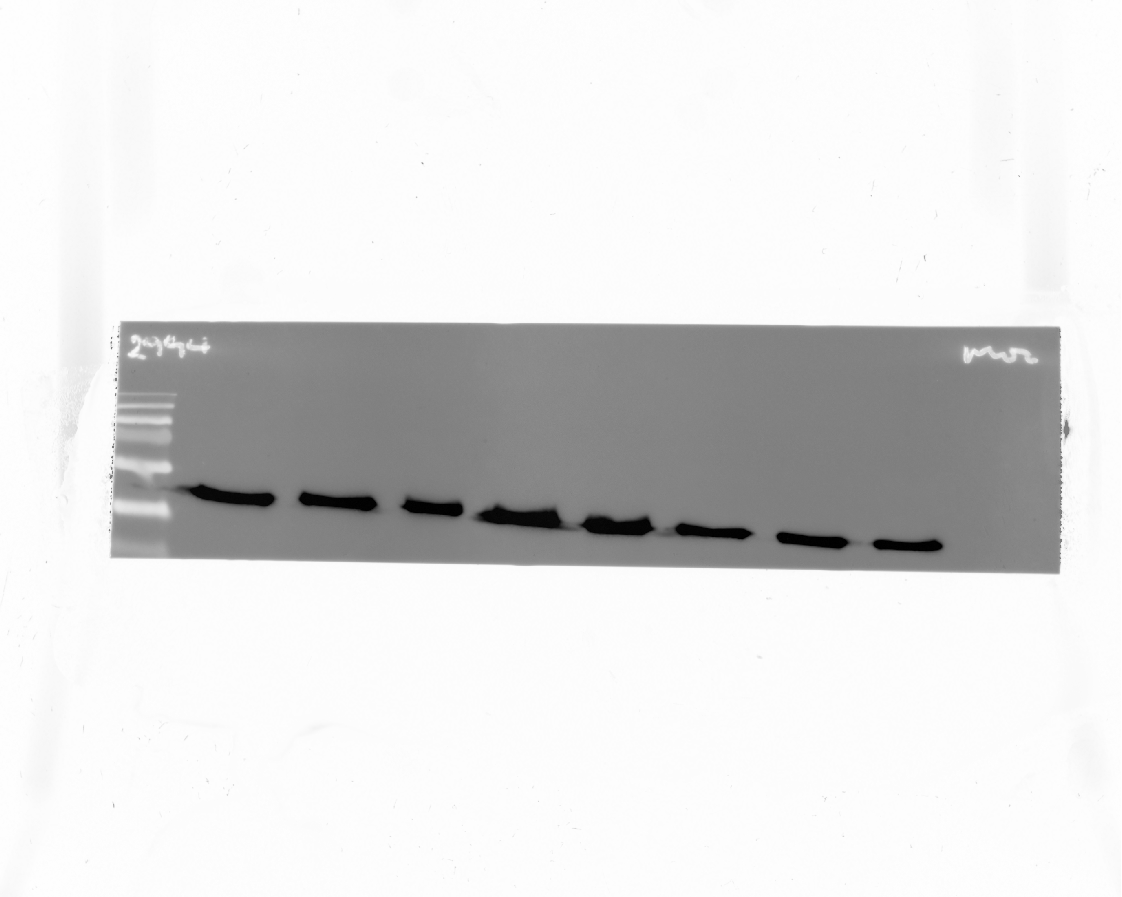

Supplement: Figure 2—figure supplement 5—source data 2. [file elife-106699-fig2-figsupp5-data2.zip › Figure 2ΓÇöfigure supplement 5-source data 2 Western blot raw data shows the longevity of responses of KOPT-K1 cells to single dose treatment./Actin Jurkat&KOP Abd-VHL (Composite).tif]

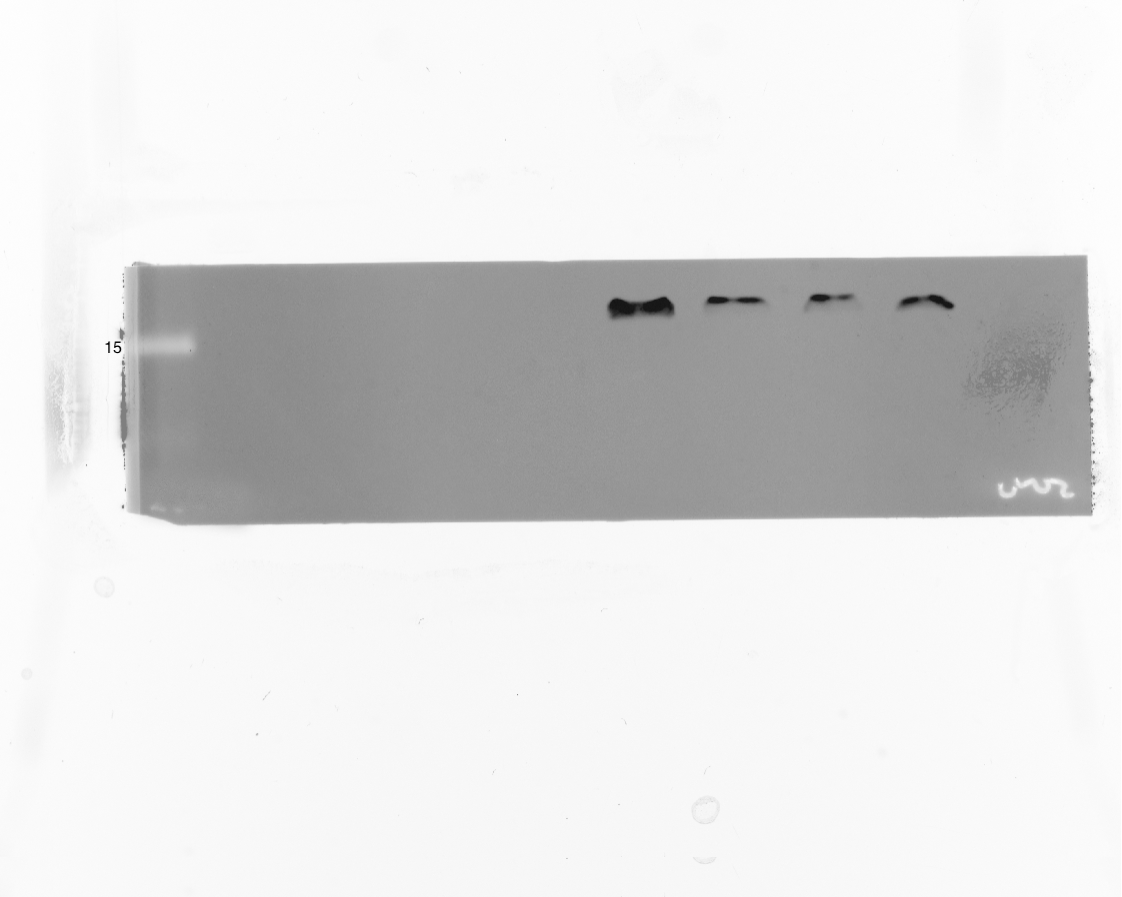

Supplement: Figure 2—figure supplement 5—source data 2. [file elife-106699-fig2-figsupp5-data2.zip › Figure 2ΓÇöfigure supplement 5-source data 2 Western blot raw data shows the longevity of responses of KOPT-K1 cells to single dose treatment./LMO2 Jurkat&KOP Abd-VHL (Composite).tif]

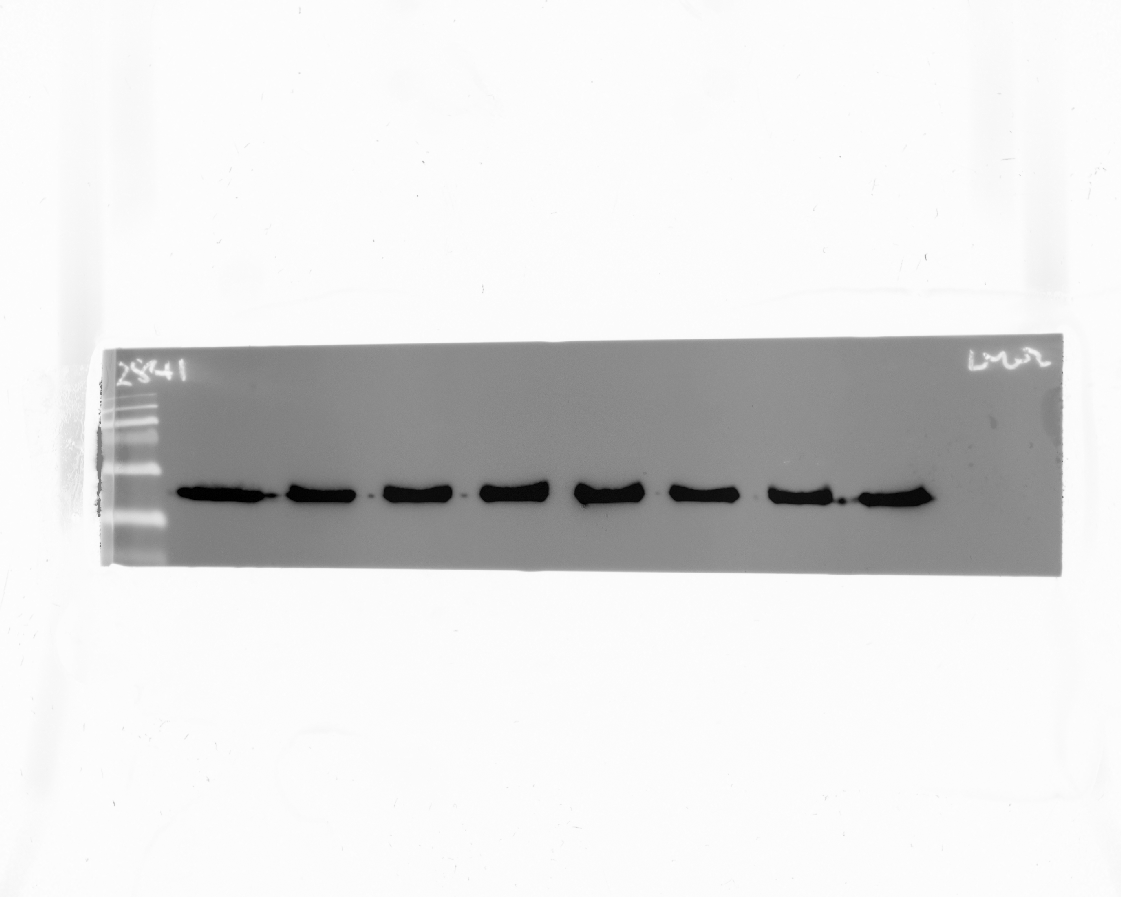

Supplement: Figure 2—figure supplement 5—source data 2. [file elife-106699-fig2-figsupp5-data2.zip › Figure 2ΓÇöfigure supplement 5-source data 2 Western blot raw data shows the longevity of responses of KOPT-K1 cells to single dose treatment./Actin Jurkat&KOP Abd-CRBN (Composite).tif]

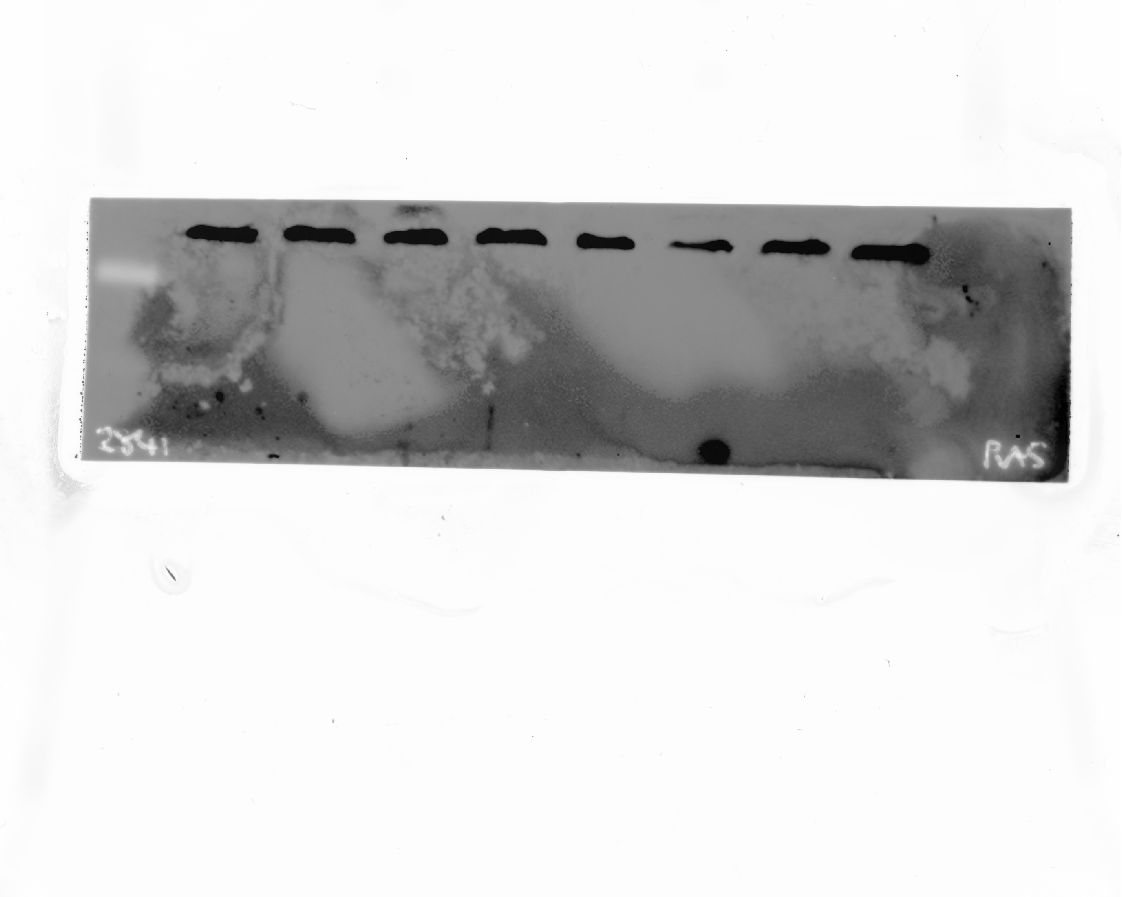

Supplement: Figure 2—figure supplement 5—source data 2. [file elife-106699-fig2-figsupp5-data2.zip › Figure 2ΓÇöfigure supplement 5-source data 2 Western blot raw data shows the longevity of responses of KOPT-K1 cells to single dose treatment./pan-RAS Jurkat&KOP Abd-CRBN (Composite).tif]

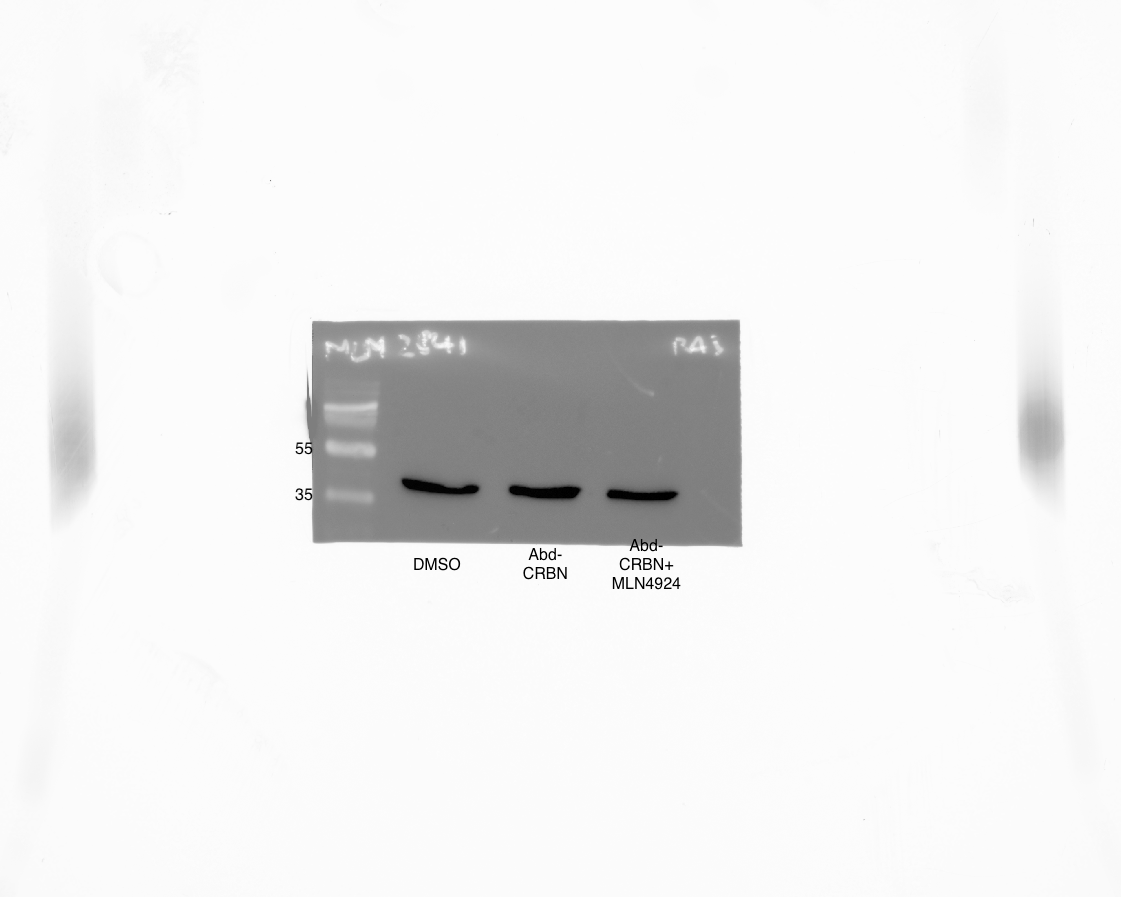

Supplement: Figure 2—figure supplement 6—source data 1. [file elife-106699-fig2-figsupp6-data1.zip › Figure 2ΓÇöfigure supplement 6-source data 1 Western blot data with label shows LMO2 level of KOPT-K1 treated with Abd compounds and inhibitors./Raw data/Actin Abd-CRBN-MLN(Composite).tif]

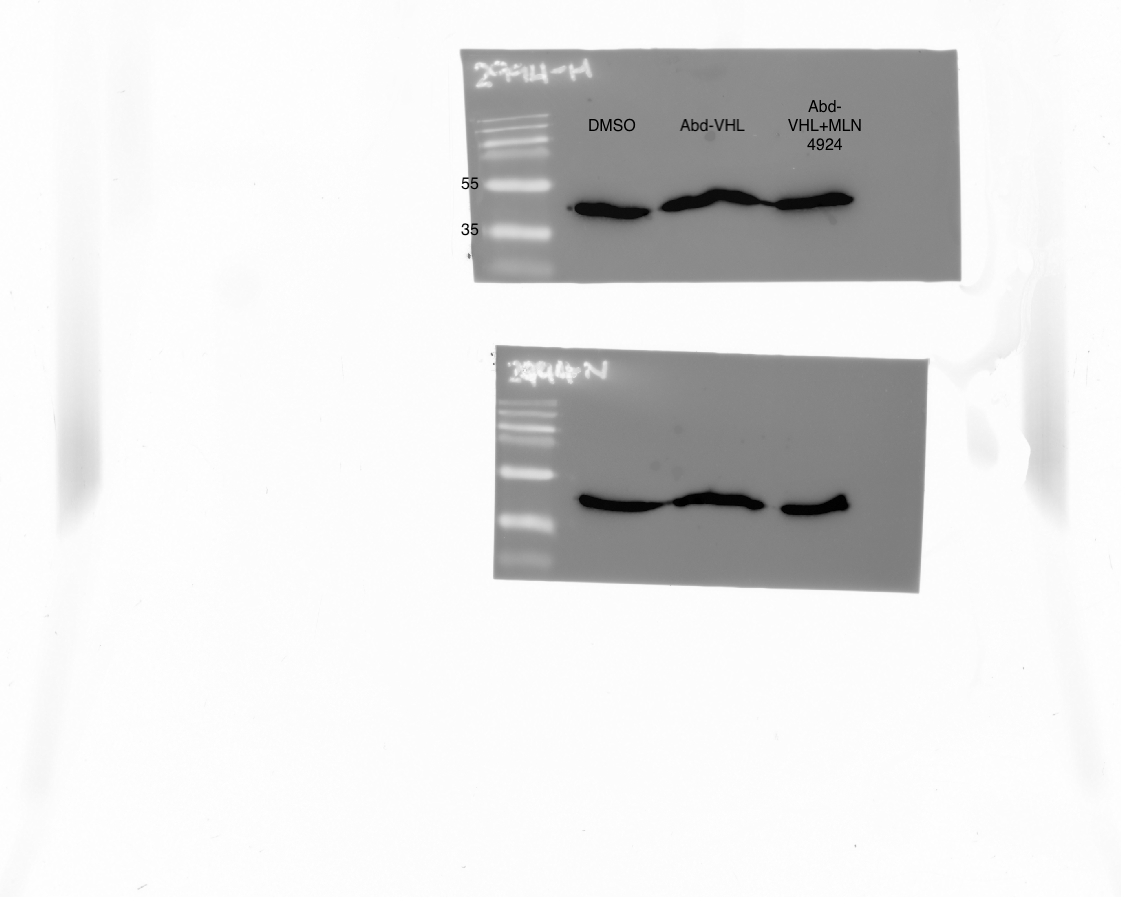

Supplement: Figure 2—figure supplement 6—source data 1. [file elife-106699-fig2-figsupp6-data1.zip › Figure 2ΓÇöfigure supplement 6-source data 1 Western blot data with label shows LMO2 level of KOPT-K1 treated with Abd compounds and inhibitors./Raw data/Actin Abd-VHL-MLN(Composite).tif]

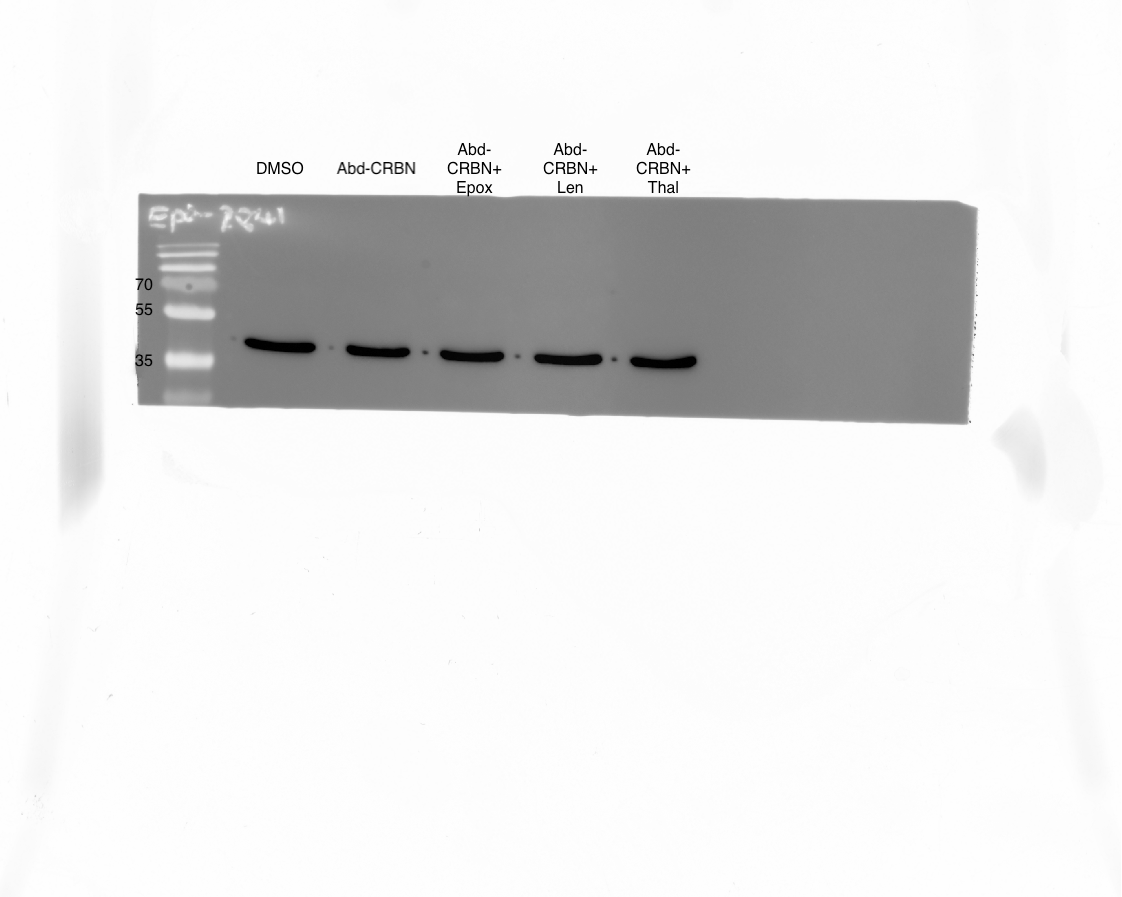

Supplement: Figure 2—figure supplement 6—source data 1. [file elife-106699-fig2-figsupp6-data1.zip › Figure 2ΓÇöfigure supplement 6-source data 1 Western blot data with label shows LMO2 level of KOPT-K1 treated with Abd compounds and inhibitors./Raw data/Actin Abd-CRBN-Epox(Composite).tif]

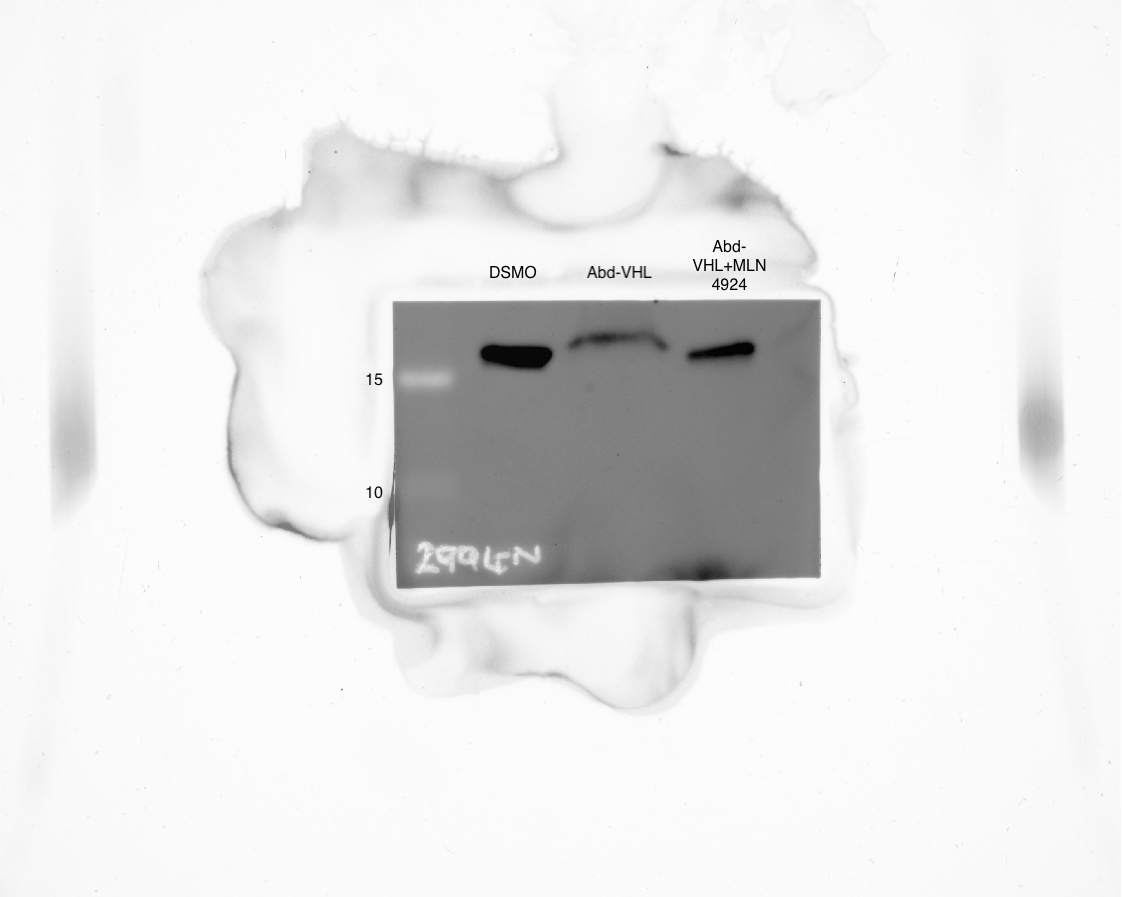

Supplement: Figure 2—figure supplement 6—source data 1. [file elife-106699-fig2-figsupp6-data1.zip › Figure 2ΓÇöfigure supplement 6-source data 1 Western blot data with label shows LMO2 level of KOPT-K1 treated with Abd compounds and inhibitors./Raw data/LMO2 Abd-VHL+MLN(Composite).tif]

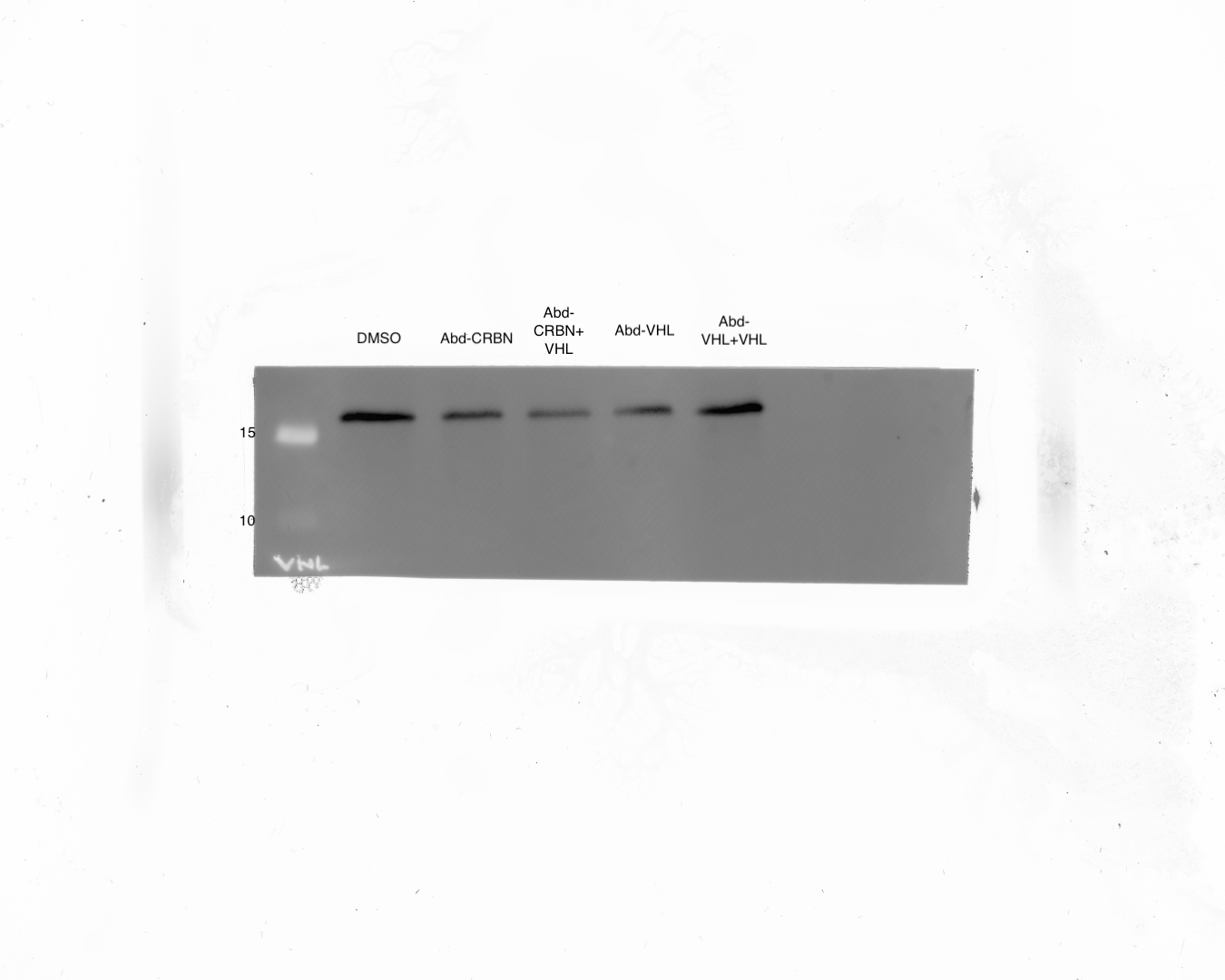

Supplement: Figure 2—figure supplement 6—source data 1. [file elife-106699-fig2-figsupp6-data1.zip › Figure 2ΓÇöfigure supplement 6-source data 1 Western blot data with label shows LMO2 level of KOPT-K1 treated with Abd compounds and inhibitors./Raw data/LMO2 Abd-CRBN and VHL-VHL(Composite).tif]

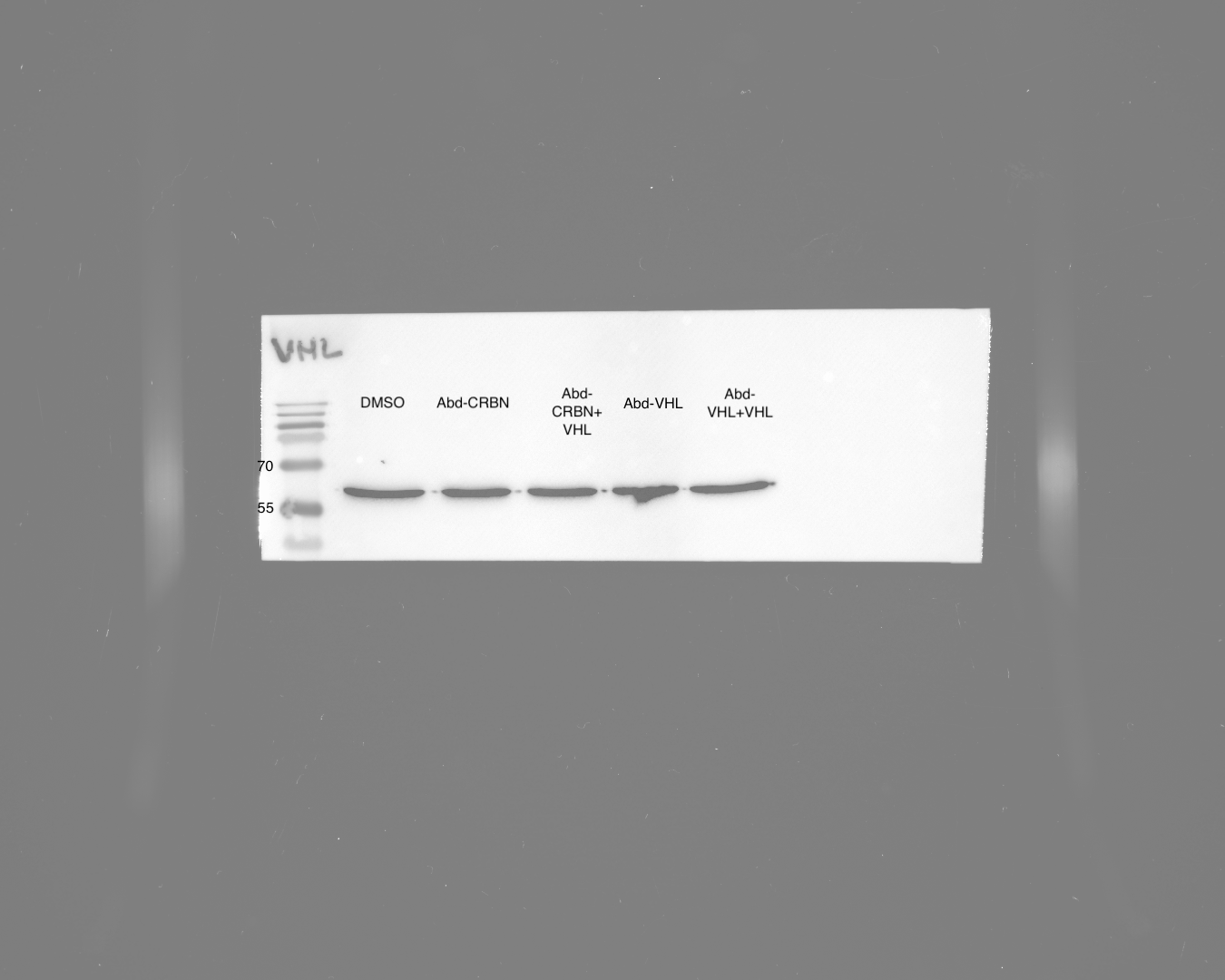

Supplement: Figure 2—figure supplement 6—source data 1. [file elife-106699-fig2-figsupp6-data1.zip › Figure 2ΓÇöfigure supplement 6-source data 1 Western blot data with label shows LMO2 level of KOPT-K1 treated with Abd compounds and inhibitors./Raw data/Actin Abd-VHL-VHL(Composite).tif]

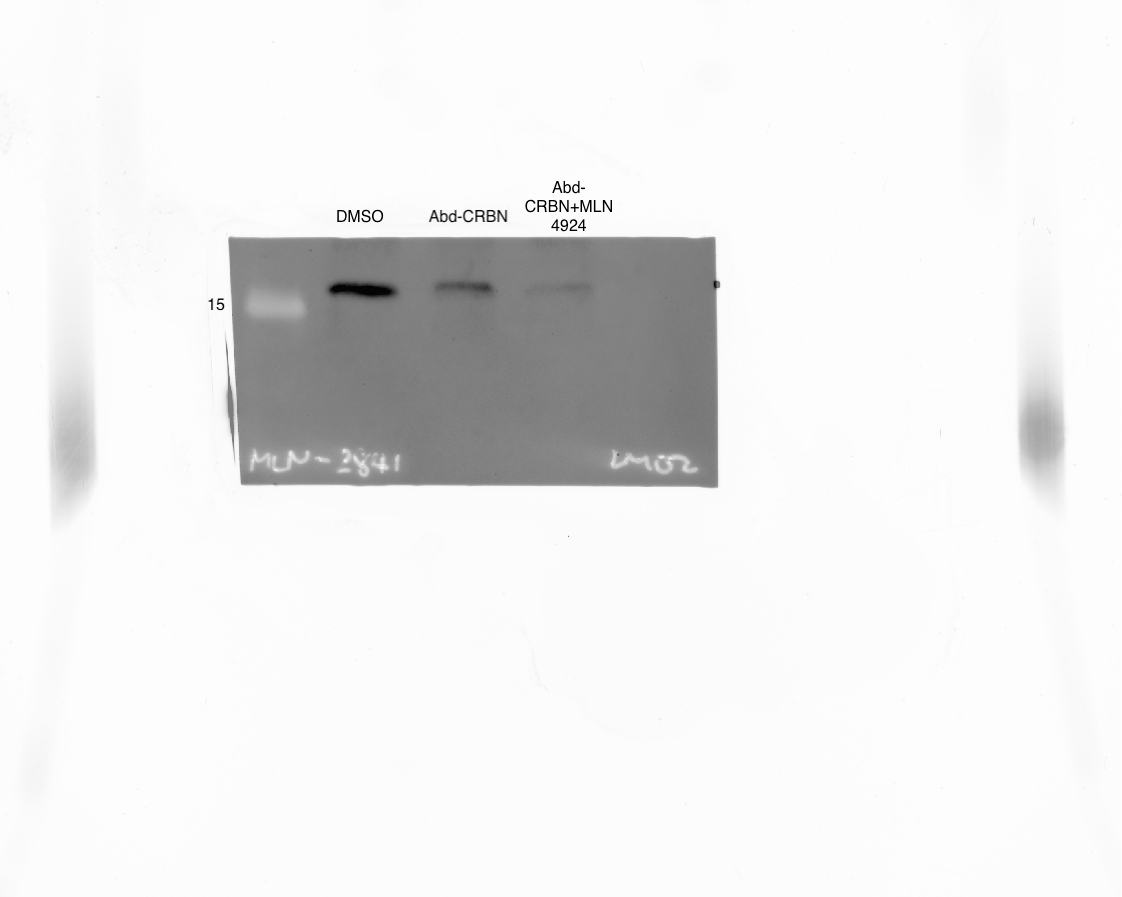

Supplement: Figure 2—figure supplement 6—source data 1. [file elife-106699-fig2-figsupp6-data1.zip › Figure 2ΓÇöfigure supplement 6-source data 1 Western blot data with label shows LMO2 level of KOPT-K1 treated with Abd compounds and inhibitors./Raw data/LMO2 Abd-CRBN+MLN(Composite).tif]

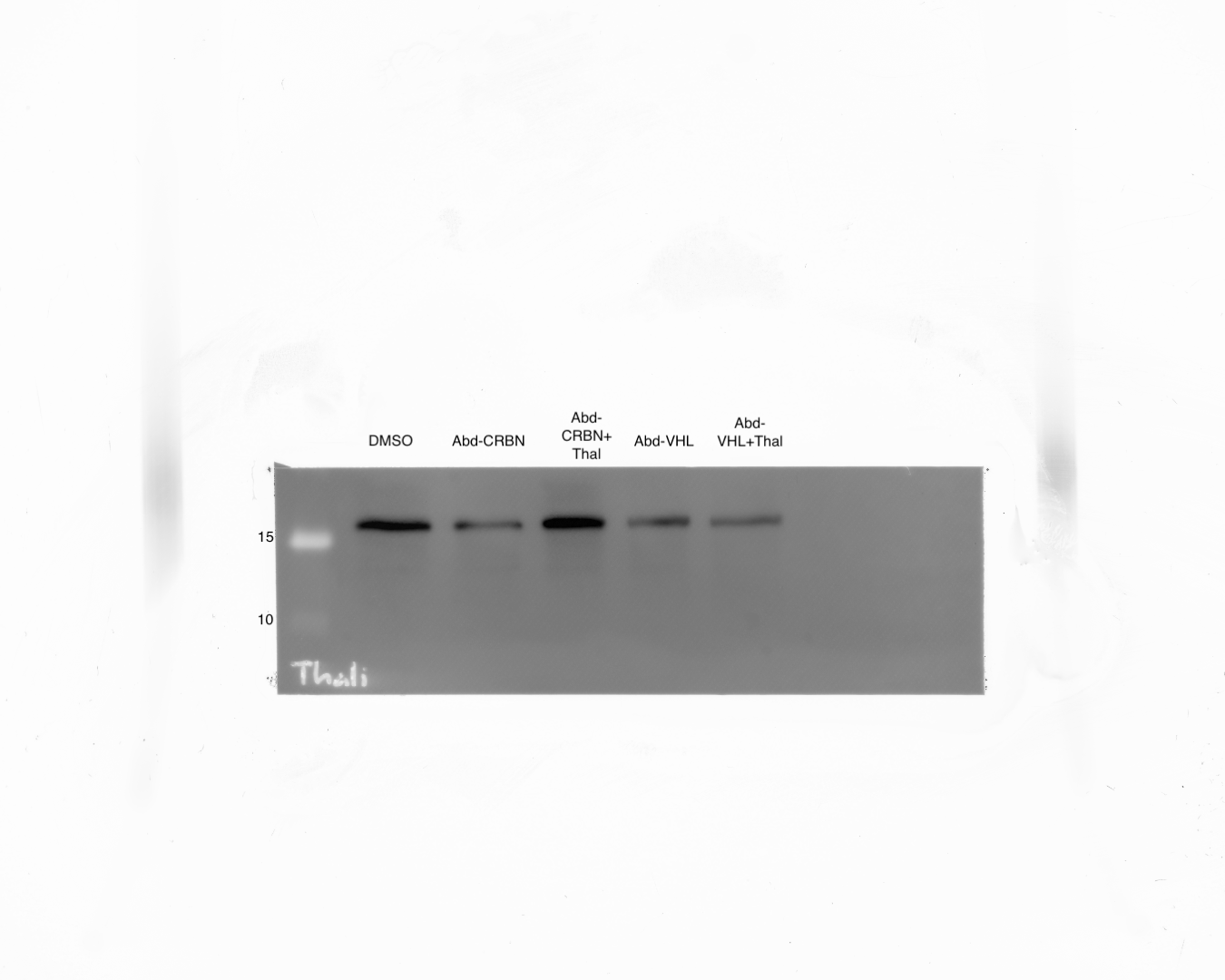

Supplement: Figure 2—figure supplement 6—source data 1. [file elife-106699-fig2-figsupp6-data1.zip › Figure 2ΓÇöfigure supplement 6-source data 1 Western blot data with label shows LMO2 level of KOPT-K1 treated with Abd compounds and inhibitors./Raw data/LMO2 Abd-CRBN and VHL-Thalidomide(Composite).tif]

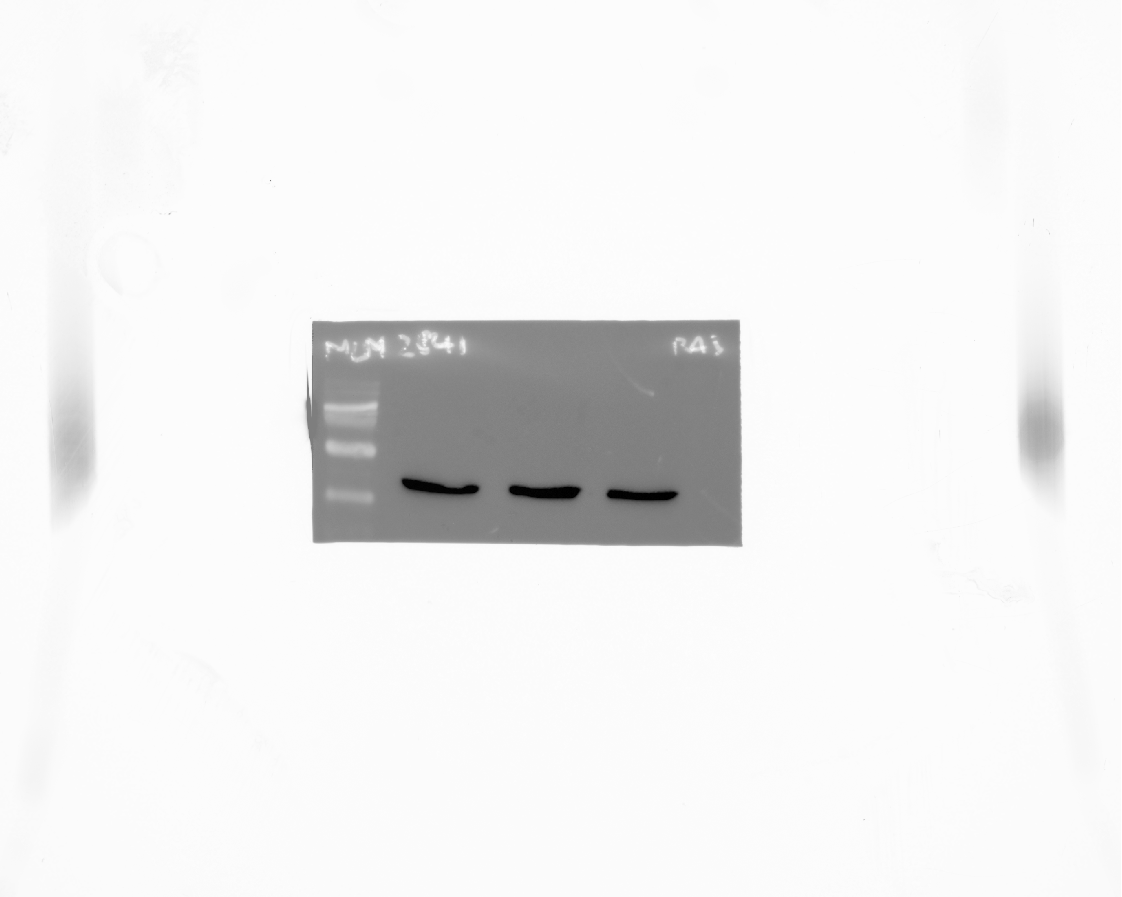

Supplement: Figure 2—figure supplement 6—source data 2. [file elife-106699-fig2-figsupp6-data2.zip › Figure 2ΓÇöfigure supplement 6-source data 2 Western blot raw datashows LMO2 level of KOPT-K1 treated with Abd compounds and inhibitors./Actin Abd-CRBN-MLN(Composite).tif]

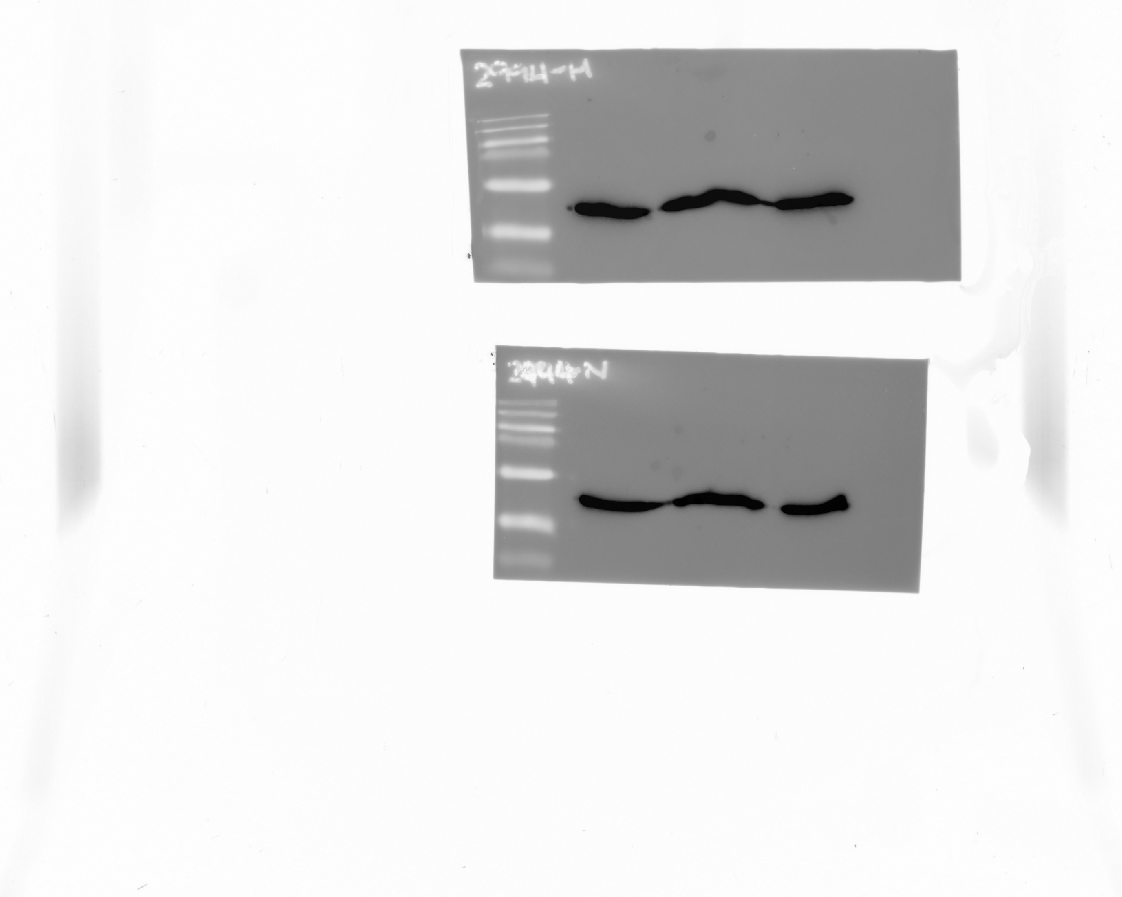

Supplement: Figure 2—figure supplement 6—source data 2. [file elife-106699-fig2-figsupp6-data2.zip › Figure 2ΓÇöfigure supplement 6-source data 2 Western blot raw datashows LMO2 level of KOPT-K1 treated with Abd compounds and inhibitors./Actin Abd-VHL-MLN(Composite).tif]

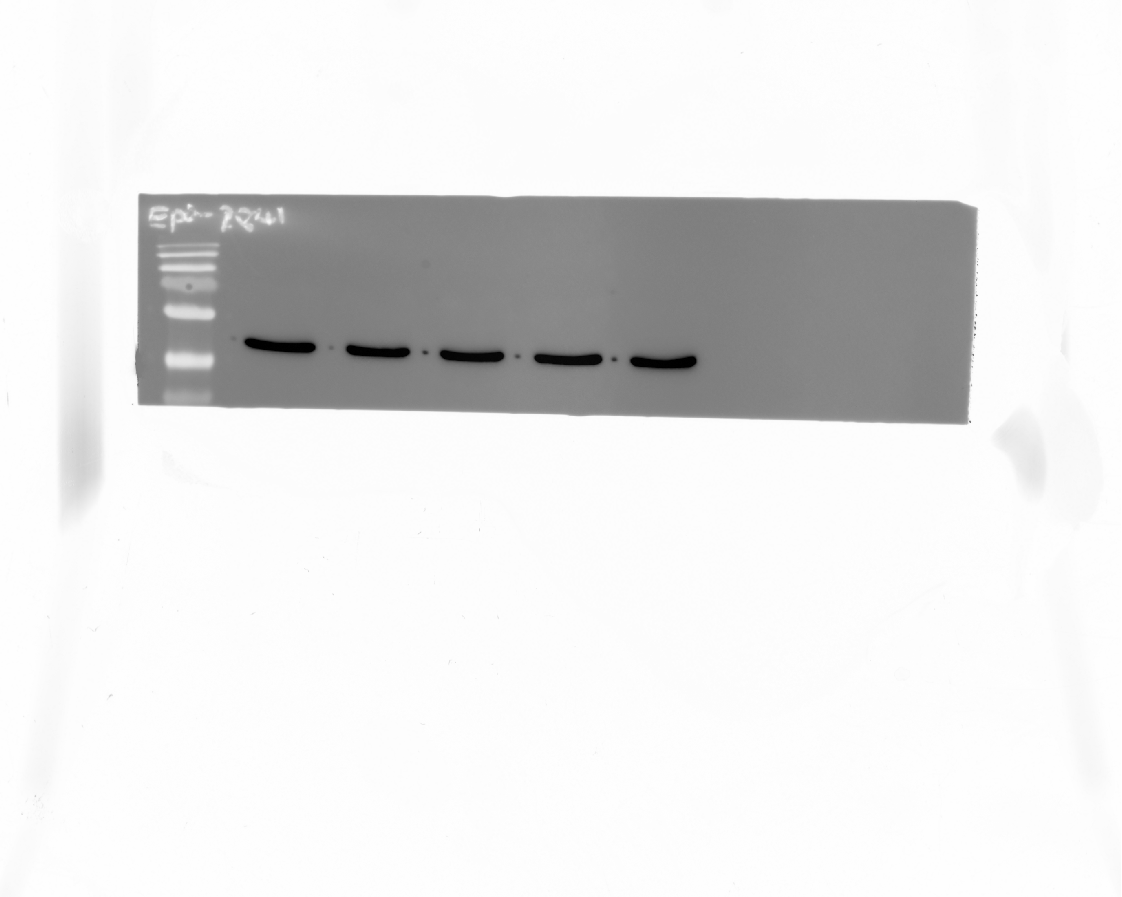

Supplement: Figure 2—figure supplement 6—source data 2. [file elife-106699-fig2-figsupp6-data2.zip › Figure 2ΓÇöfigure supplement 6-source data 2 Western blot raw datashows LMO2 level of KOPT-K1 treated with Abd compounds and inhibitors./Actin Abd-CRBN-Epox(Composite).tif]

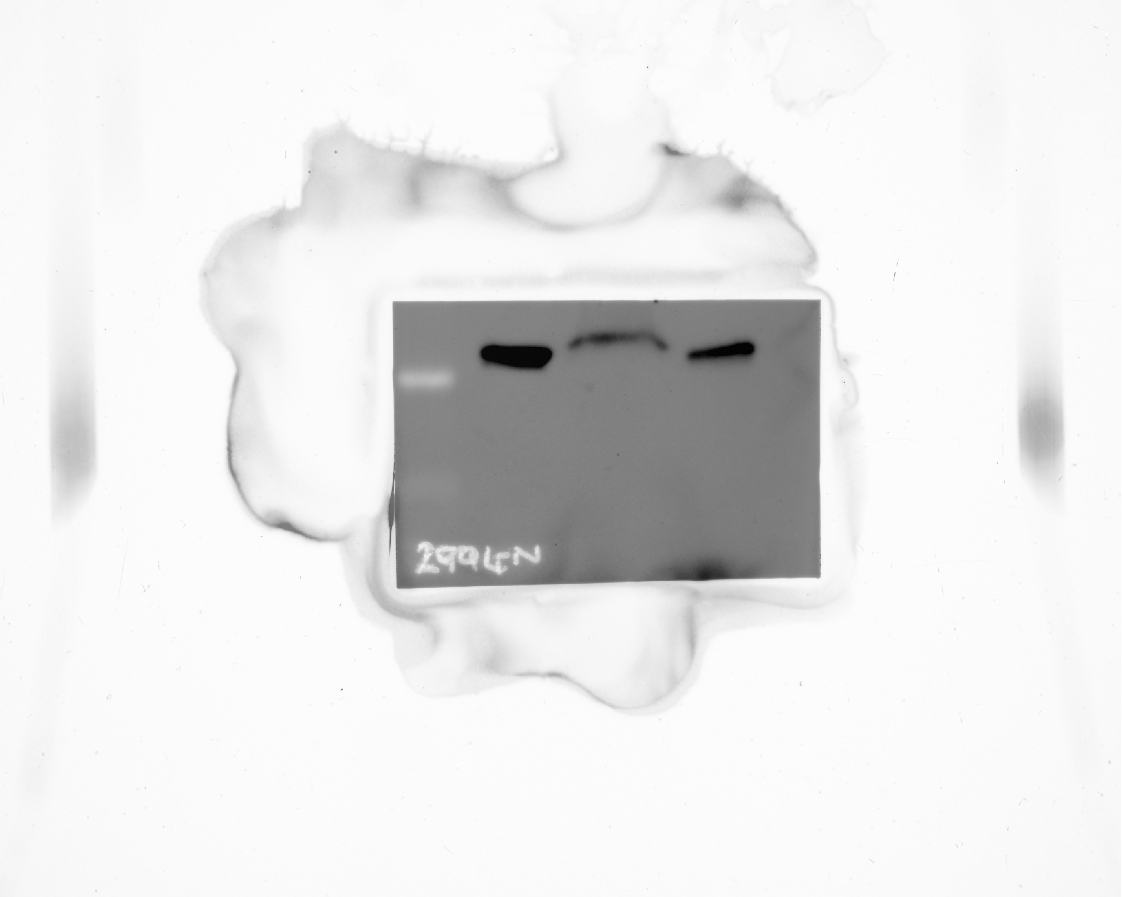

Supplement: Figure 2—figure supplement 6—source data 2. [file elife-106699-fig2-figsupp6-data2.zip › Figure 2ΓÇöfigure supplement 6-source data 2 Western blot raw datashows LMO2 level of KOPT-K1 treated with Abd compounds and inhibitors./LMO2 Abd-VHL+MLN(Composite).tif]

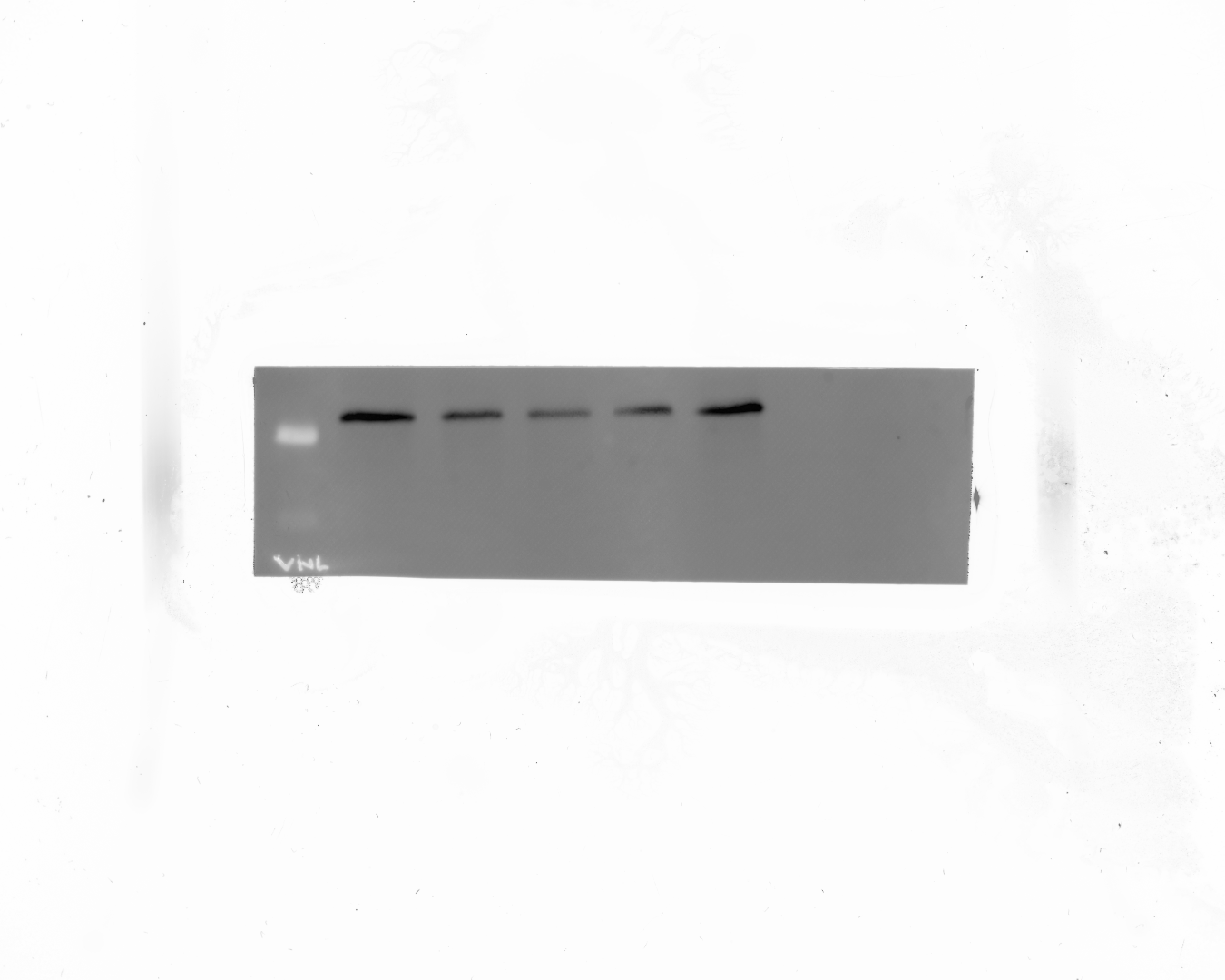

Supplement: Figure 2—figure supplement 6—source data 2. [file elife-106699-fig2-figsupp6-data2.zip › Figure 2ΓÇöfigure supplement 6-source data 2 Western blot raw datashows LMO2 level of KOPT-K1 treated with Abd compounds and inhibitors./LMO2 Abd-CRBN and VHL-VHL(Composite).tif]

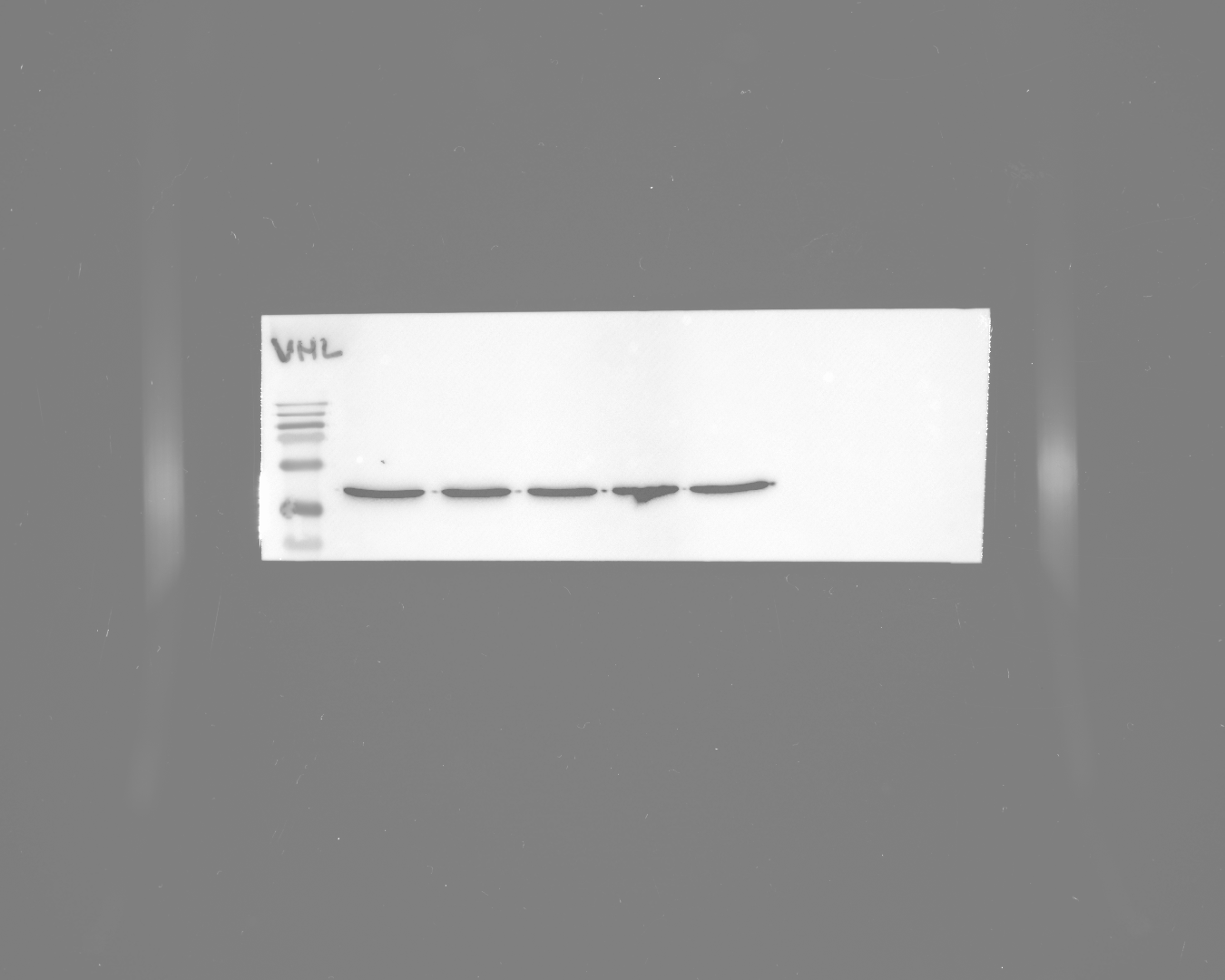

Supplement: Figure 2—figure supplement 6—source data 2. [file elife-106699-fig2-figsupp6-data2.zip › Figure 2ΓÇöfigure supplement 6-source data 2 Western blot raw datashows LMO2 level of KOPT-K1 treated with Abd compounds and inhibitors./Actin Abd-VHL-VHL(Composite).tif]

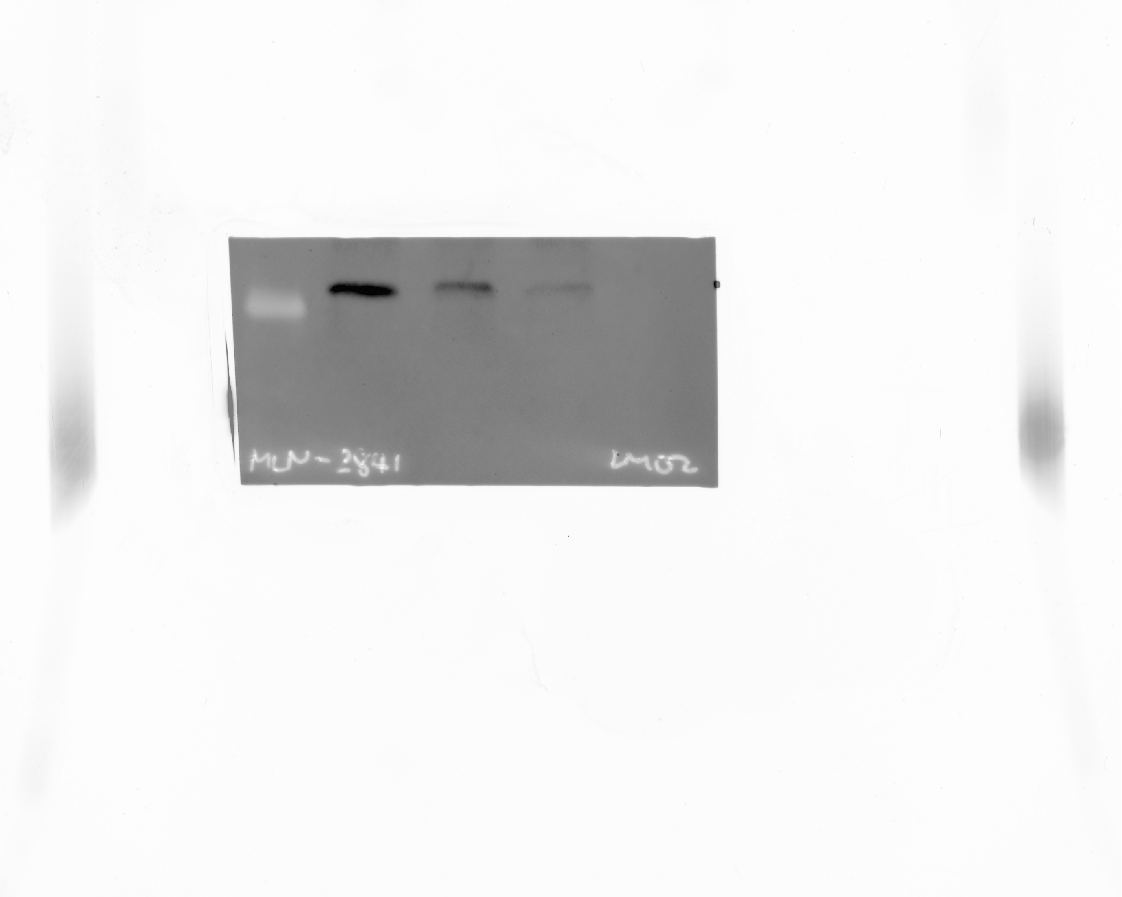

Supplement: Figure 2—figure supplement 6—source data 2. [file elife-106699-fig2-figsupp6-data2.zip › Figure 2ΓÇöfigure supplement 6-source data 2 Western blot raw datashows LMO2 level of KOPT-K1 treated with Abd compounds and inhibitors./LMO2 MLN 2841(Composite).tif]

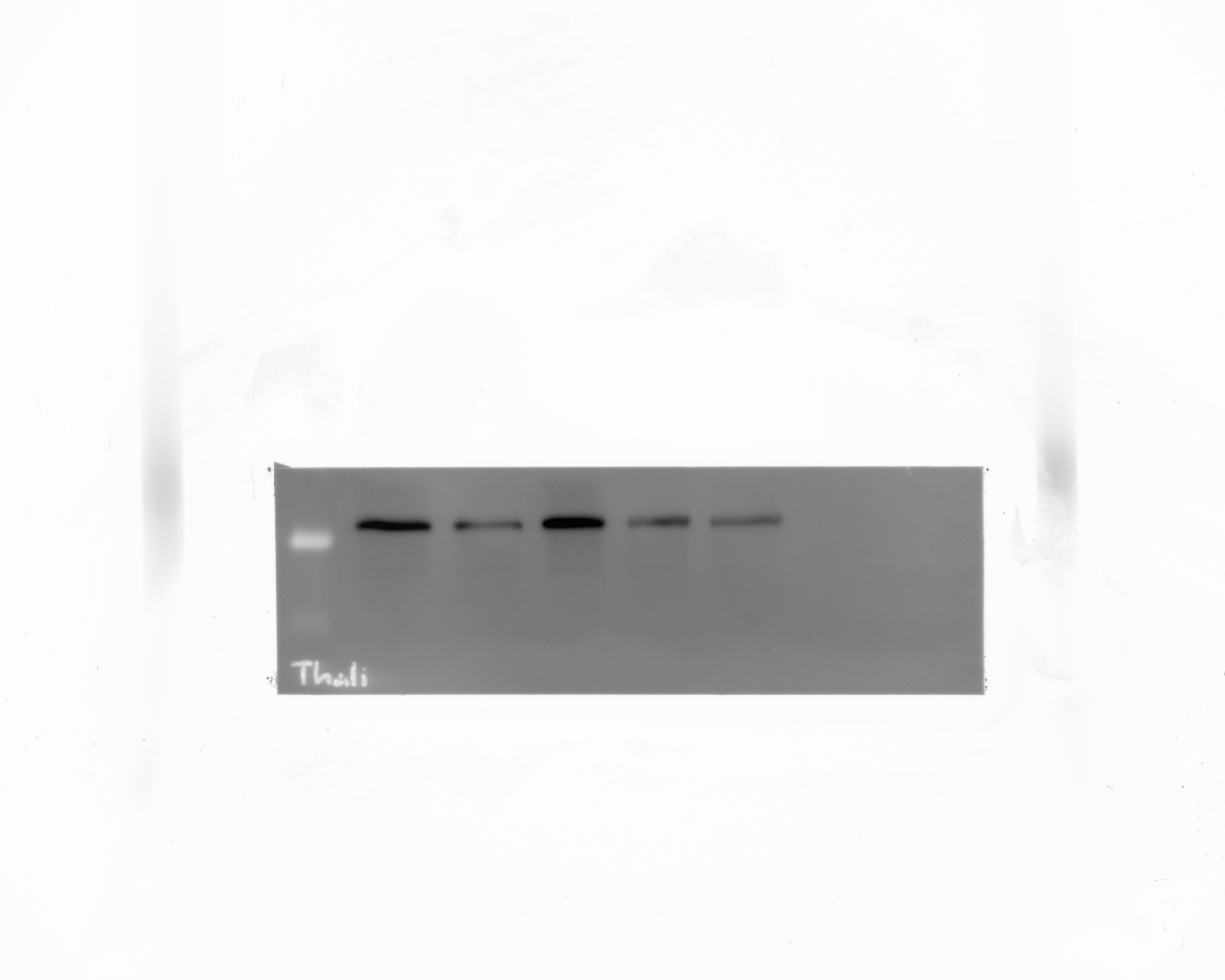

Supplement: Figure 2—figure supplement 6—source data 2. [file elife-106699-fig2-figsupp6-data2.zip › Figure 2ΓÇöfigure supplement 6-source data 2 Western blot raw datashows LMO2 level of KOPT-K1 treated with Abd compounds and inhibitors./LMO2 Abd-CRBN and VHL-Thalidomide(Composite).tif]

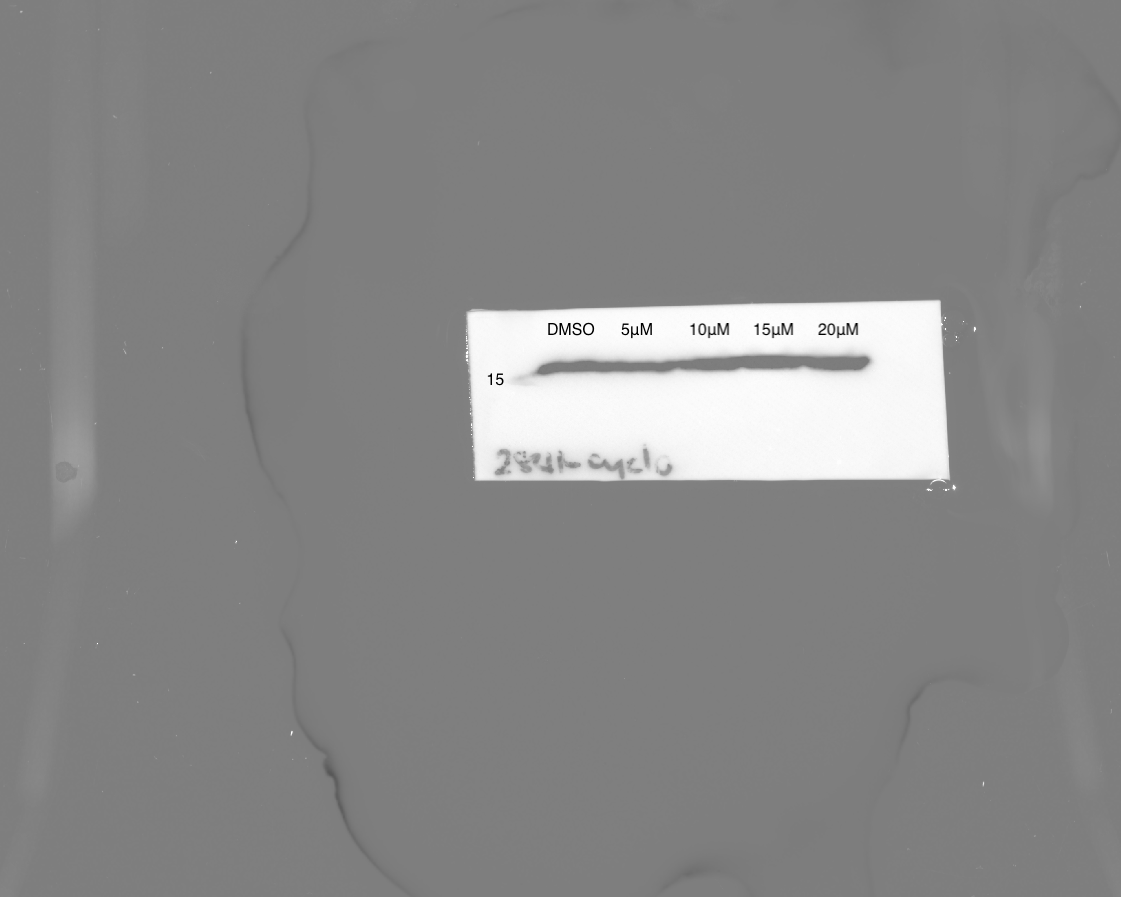

Supplement: Figure 3—source data 1. [file elife-106699-fig3-data1.zip › Figure 3ΓÇösource data 1 PDF files containing original western blots for Figure 3A, indicating the relevant bands and treatments./Raw data/Cyclophilin CCRF-CEM Abd-CRBN.tif]

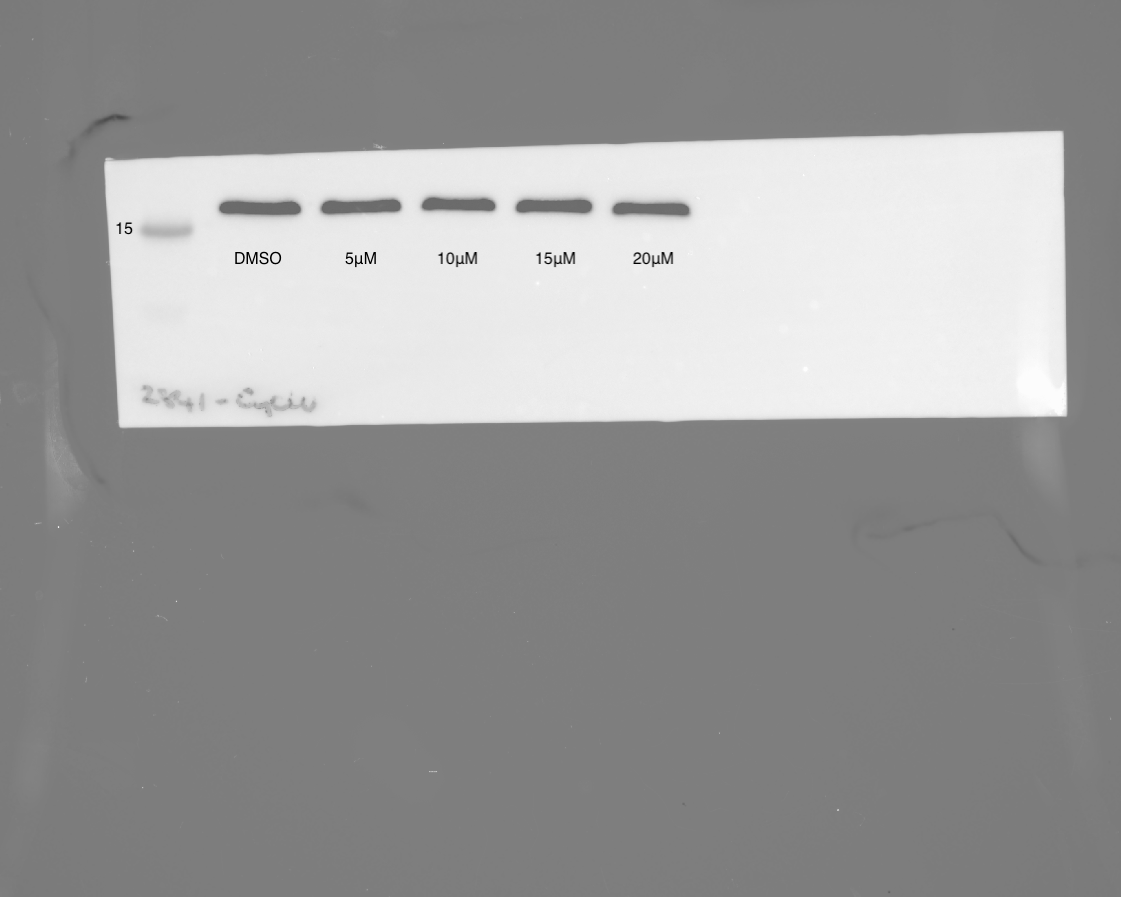

Supplement: Figure 3—source data 1. [file elife-106699-fig3-data1.zip › Figure 3ΓÇösource data 1 PDF files containing original western blots for Figure 3A, indicating the relevant bands and treatments./Raw data/Cyclophilin KOPT-K1 Abd-CRBN.tif]

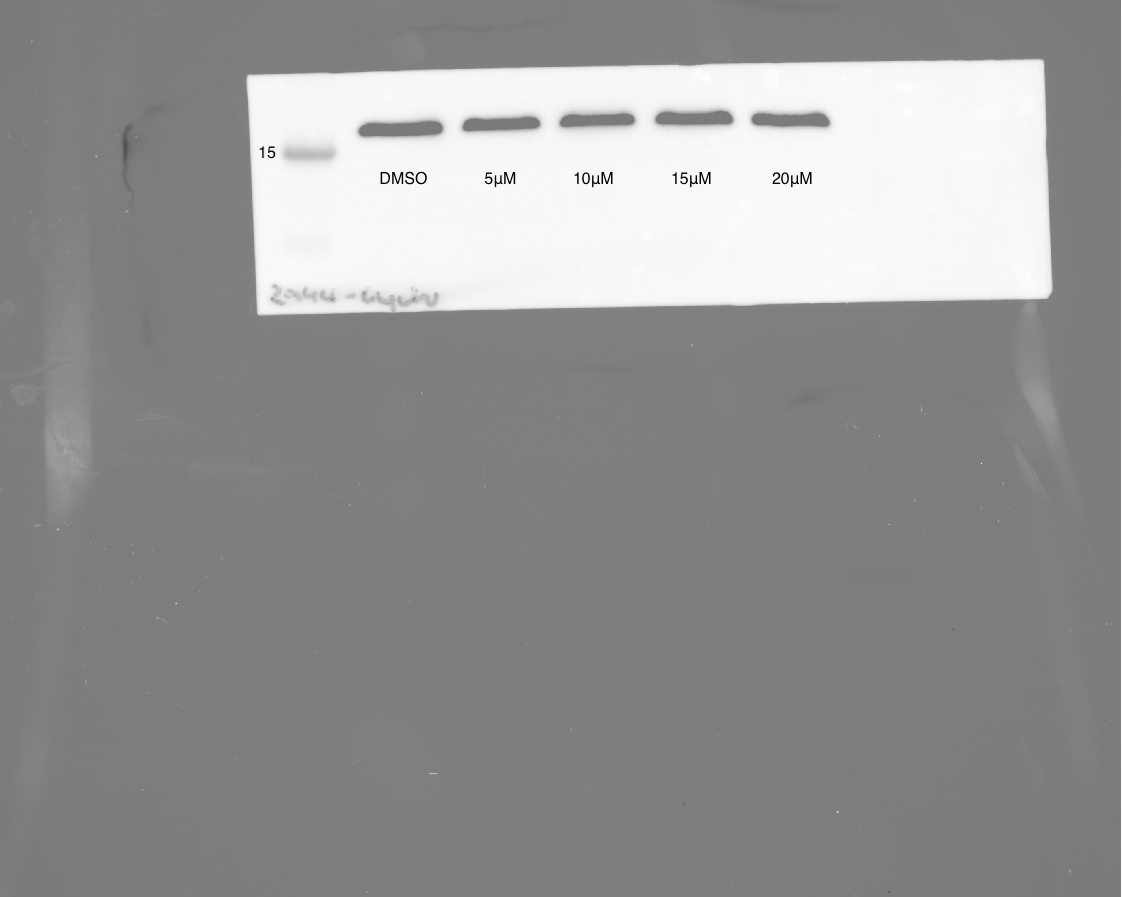

Supplement: Figure 3—source data 1. [file elife-106699-fig3-data1.zip › Figure 3ΓÇösource data 1 PDF files containing original western blots for Figure 3A, indicating the relevant bands and treatments./Raw data/Cyclophilin KOPT-K1 Abd-VHL.tif]

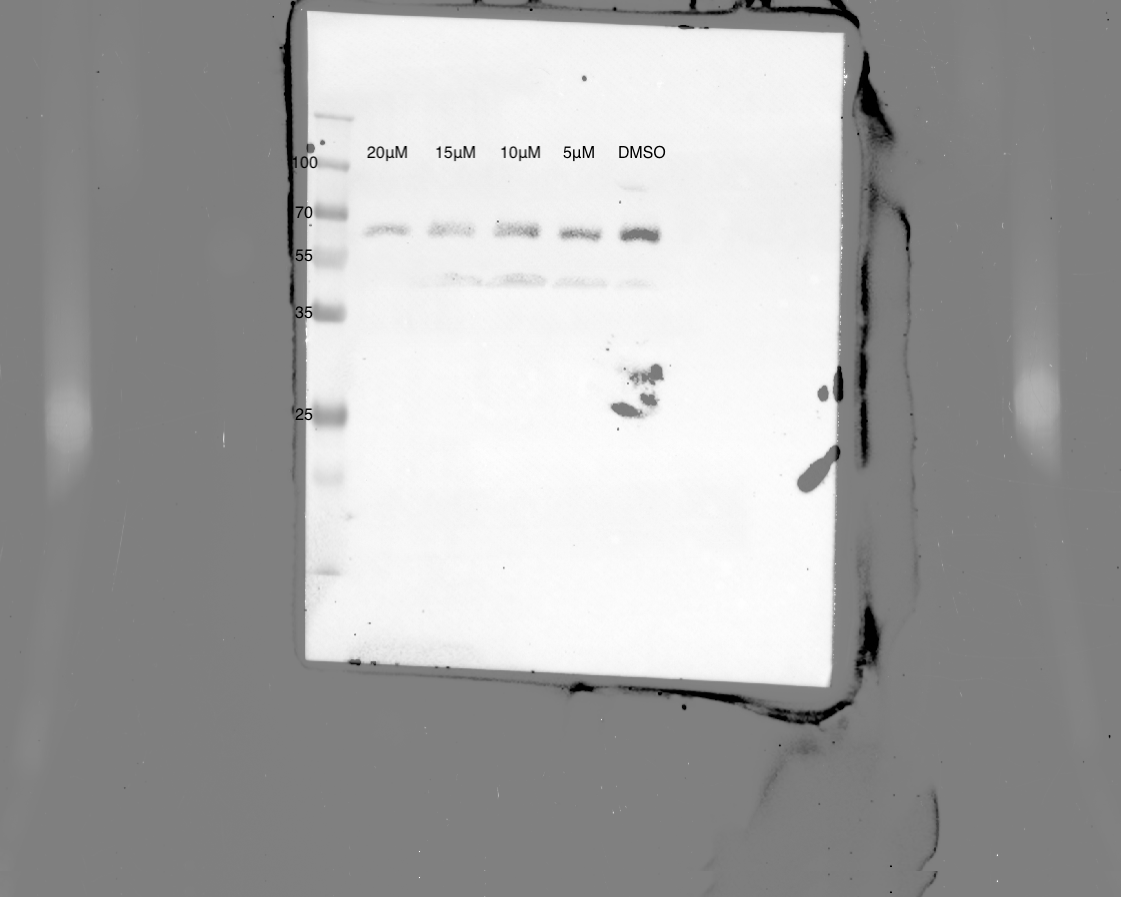

Supplement: Figure 3—source data 1. [file elife-106699-fig3-data1.zip › Figure 3ΓÇösource data 1 PDF files containing original western blots for Figure 3A, indicating the relevant bands and treatments./Raw data/E2A CCRF-CEM Abd-CRBN.tif]

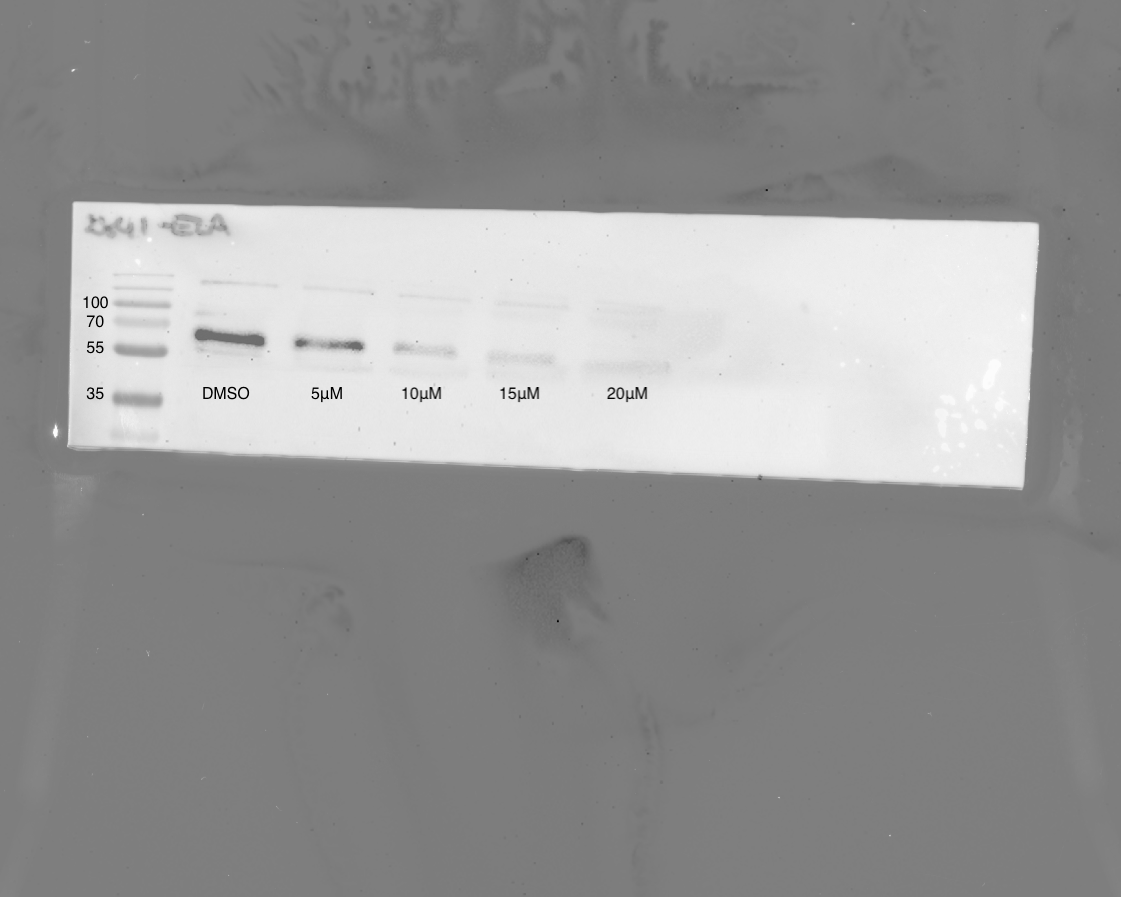

Supplement: Figure 3—source data 1. [file elife-106699-fig3-data1.zip › Figure 3ΓÇösource data 1 PDF files containing original western blots for Figure 3A, indicating the relevant bands and treatments./Raw data/E2A KOPT-K1 Abd-CRBN.tif]

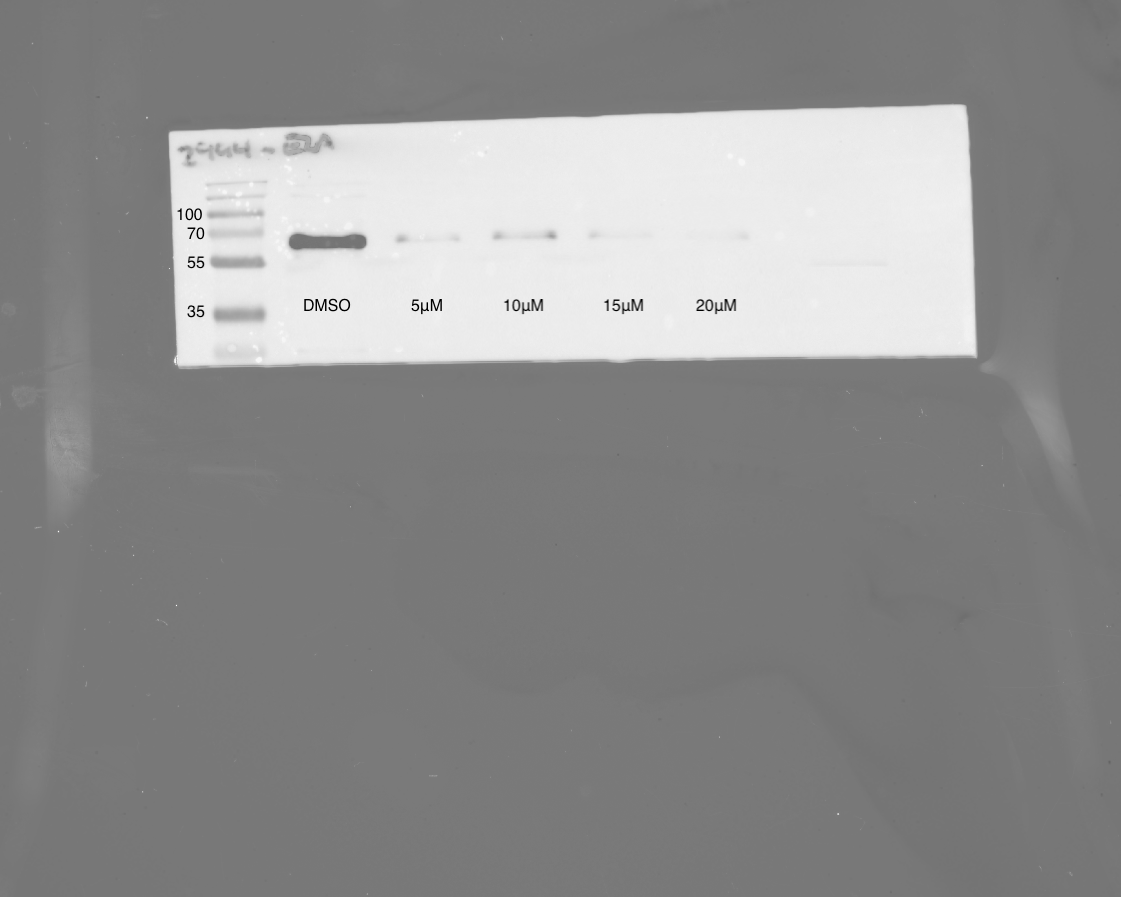

Supplement: Figure 3—source data 1. [file elife-106699-fig3-data1.zip › Figure 3ΓÇösource data 1 PDF files containing original western blots for Figure 3A, indicating the relevant bands and treatments./Raw data/E2A KOPT-K1 Abd-VHL.tif]

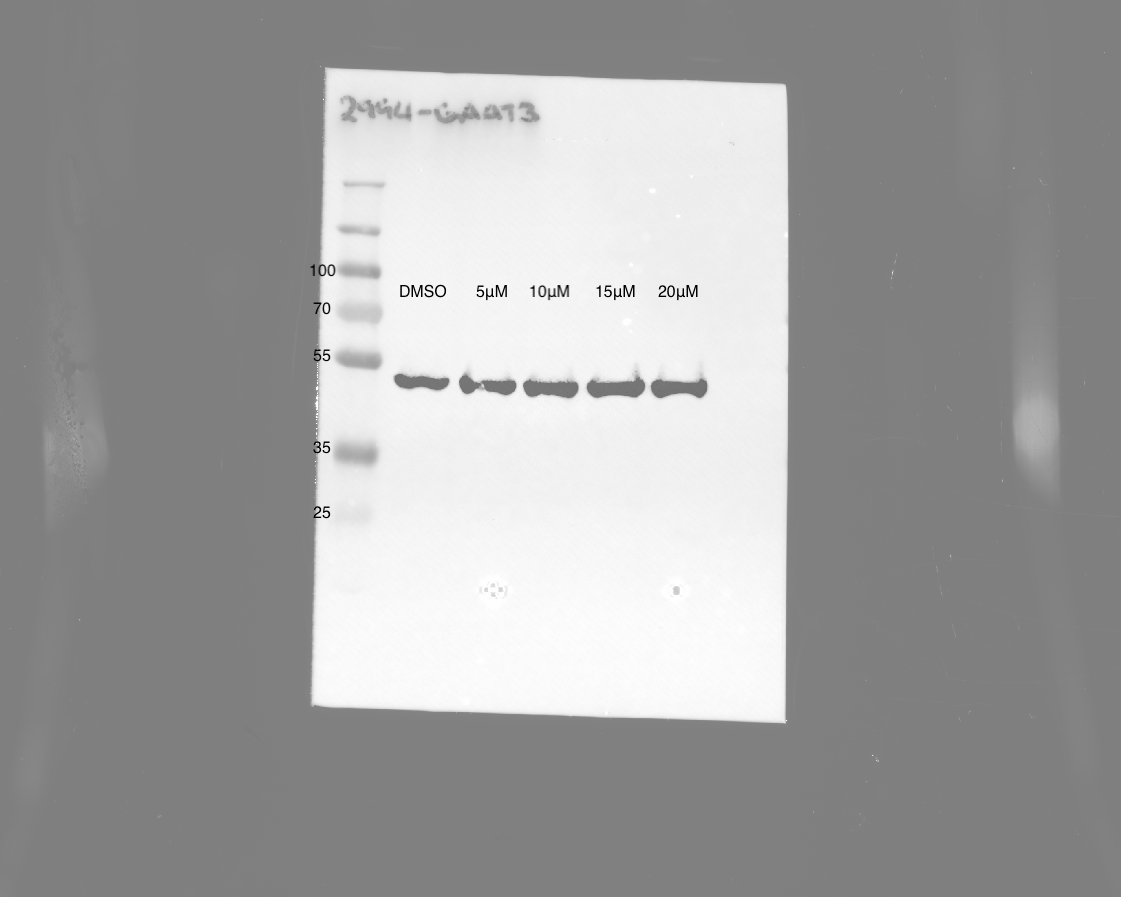

Supplement: Figure 3—source data 1. [file elife-106699-fig3-data1.zip › Figure 3ΓÇösource data 1 PDF files containing original western blots for Figure 3A, indicating the relevant bands and treatments./Raw data/GATA3 CCRF-CEM Abd-CRBN.tif]

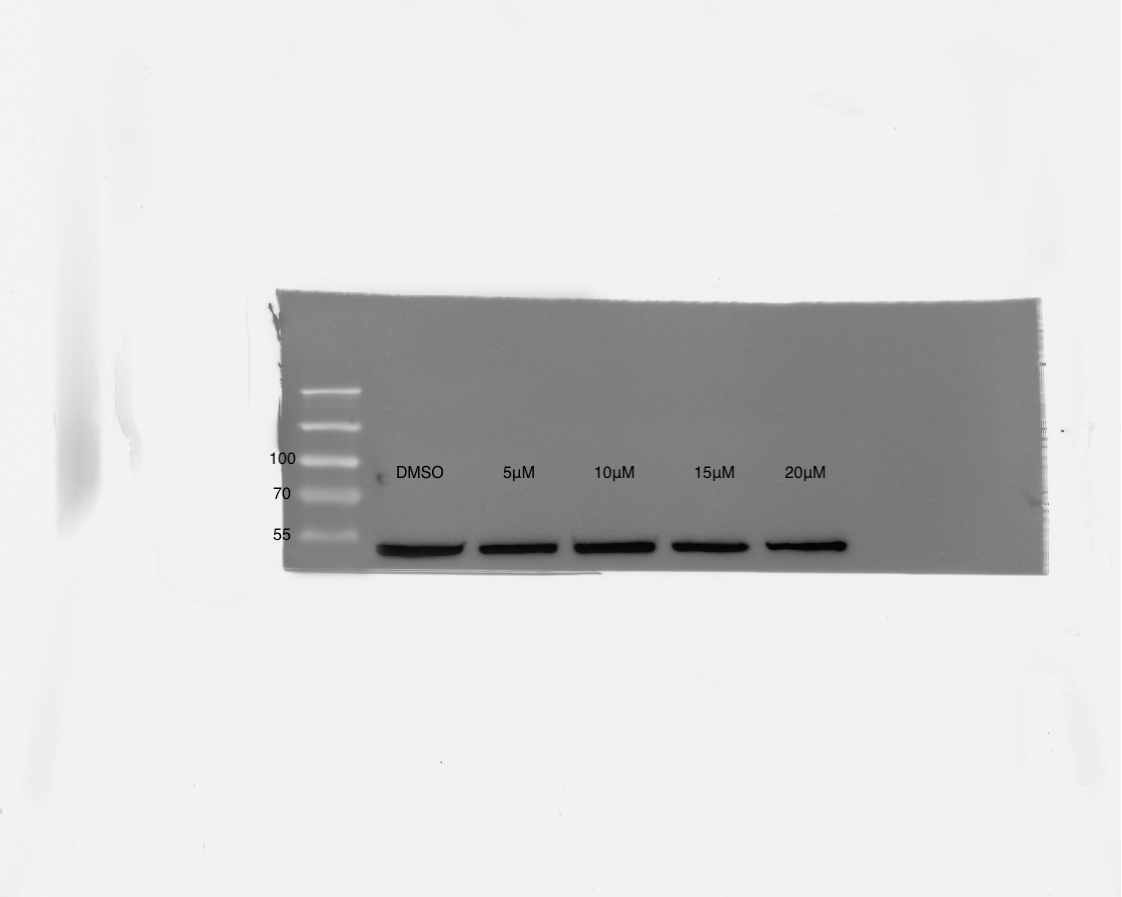

Supplement: Figure 3—source data 1. [file elife-106699-fig3-data1.zip › Figure 3ΓÇösource data 1 PDF files containing original western blots for Figure 3A, indicating the relevant bands and treatments./Raw data/GATA3 CCRF-CEM Abd-VHL.tif]

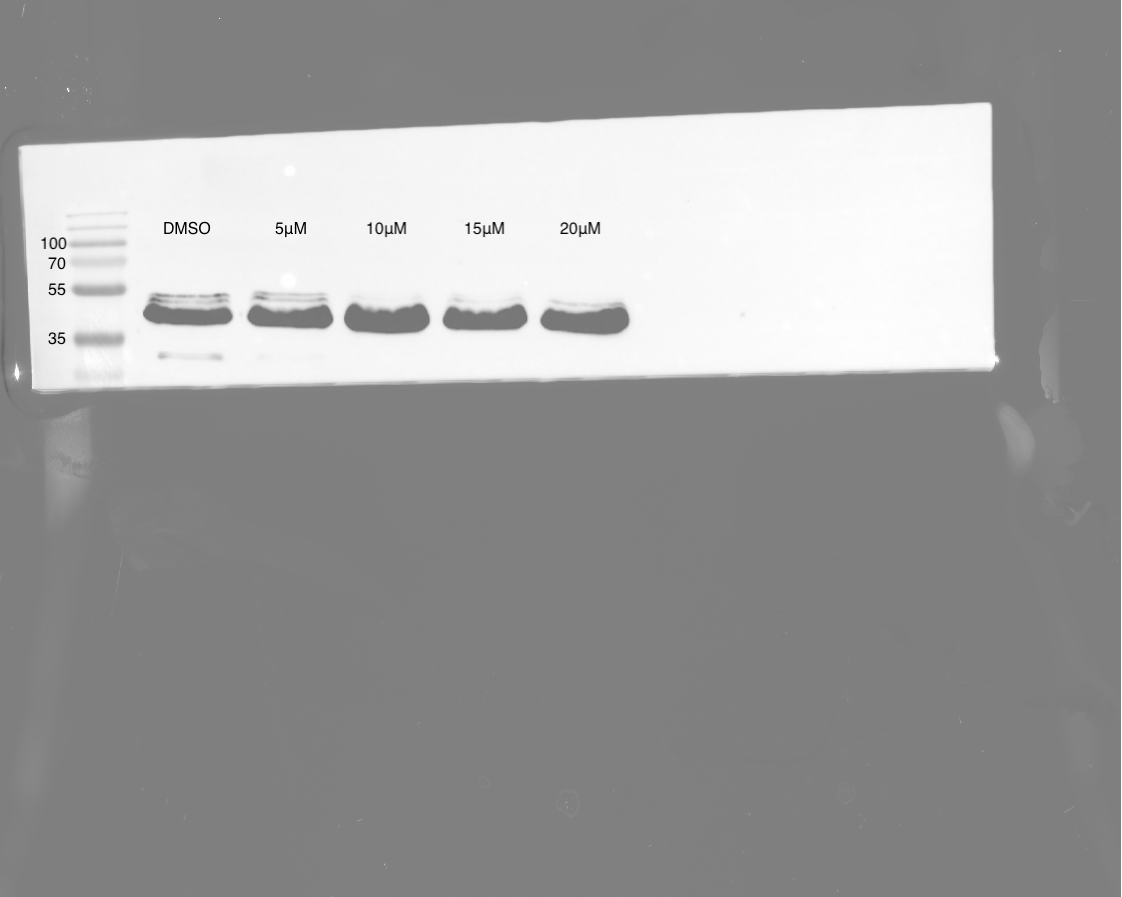

Supplement: Figure 3—source data 1. [file elife-106699-fig3-data1.zip › Figure 3ΓÇösource data 1 PDF files containing original western blots for Figure 3A, indicating the relevant bands and treatments./Raw data/GATA3 KOPT-K1 Abd-CRBN.tif]

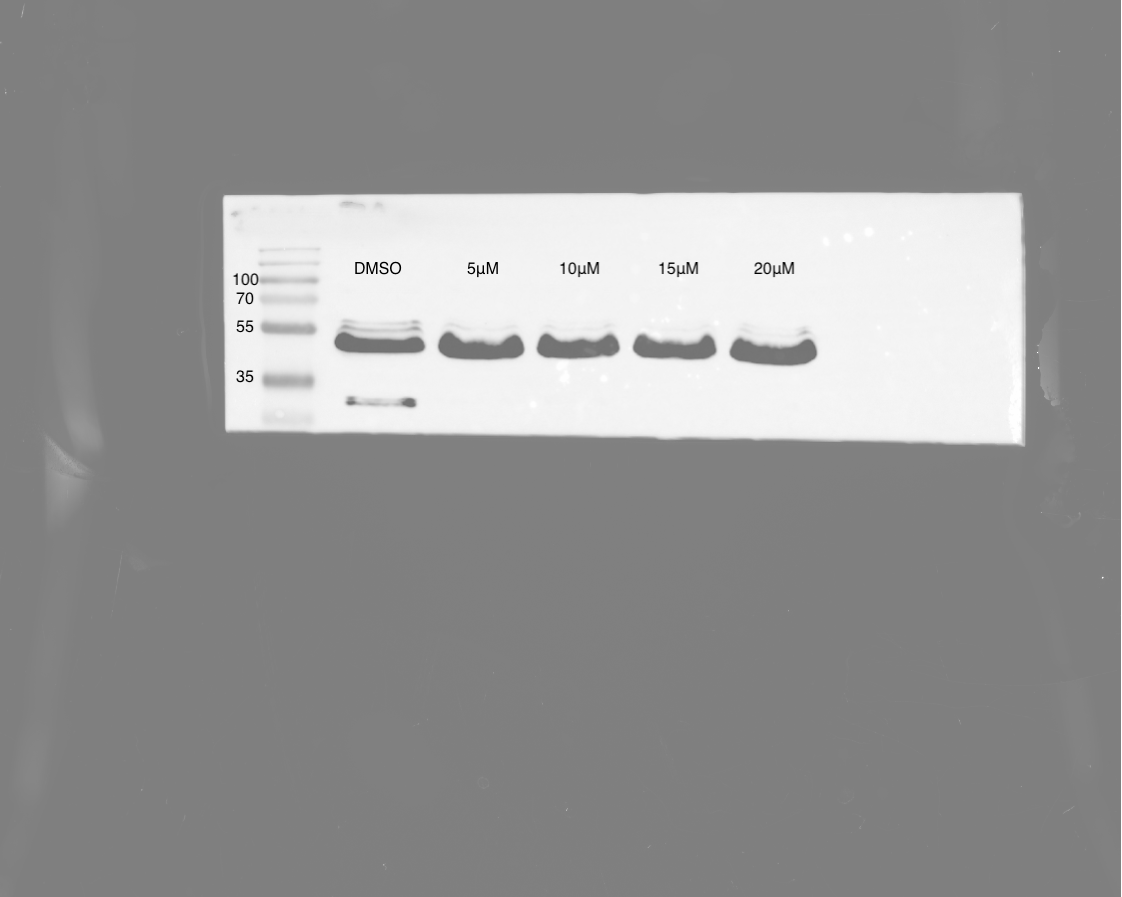

Supplement: Figure 3—source data 1. [file elife-106699-fig3-data1.zip › Figure 3ΓÇösource data 1 PDF files containing original western blots for Figure 3A, indicating the relevant bands and treatments./Raw data/GATA3 KOPT-K1 Abd-VHL.tif]

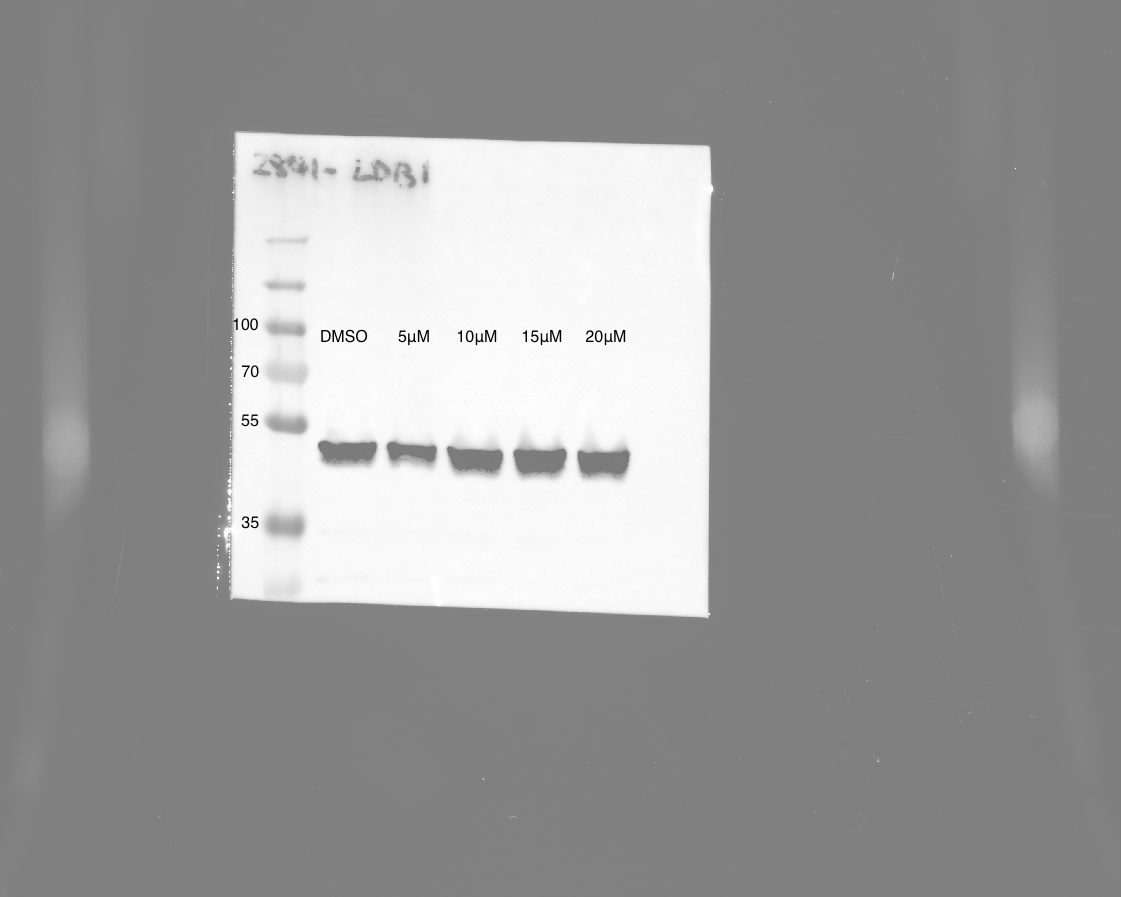

Supplement: Figure 3—source data 1. [file elife-106699-fig3-data1.zip › Figure 3ΓÇösource data 1 PDF files containing original western blots for Figure 3A, indicating the relevant bands and treatments./Raw data/LDB1 CCRF-CEM Abd-CRBN.tif]

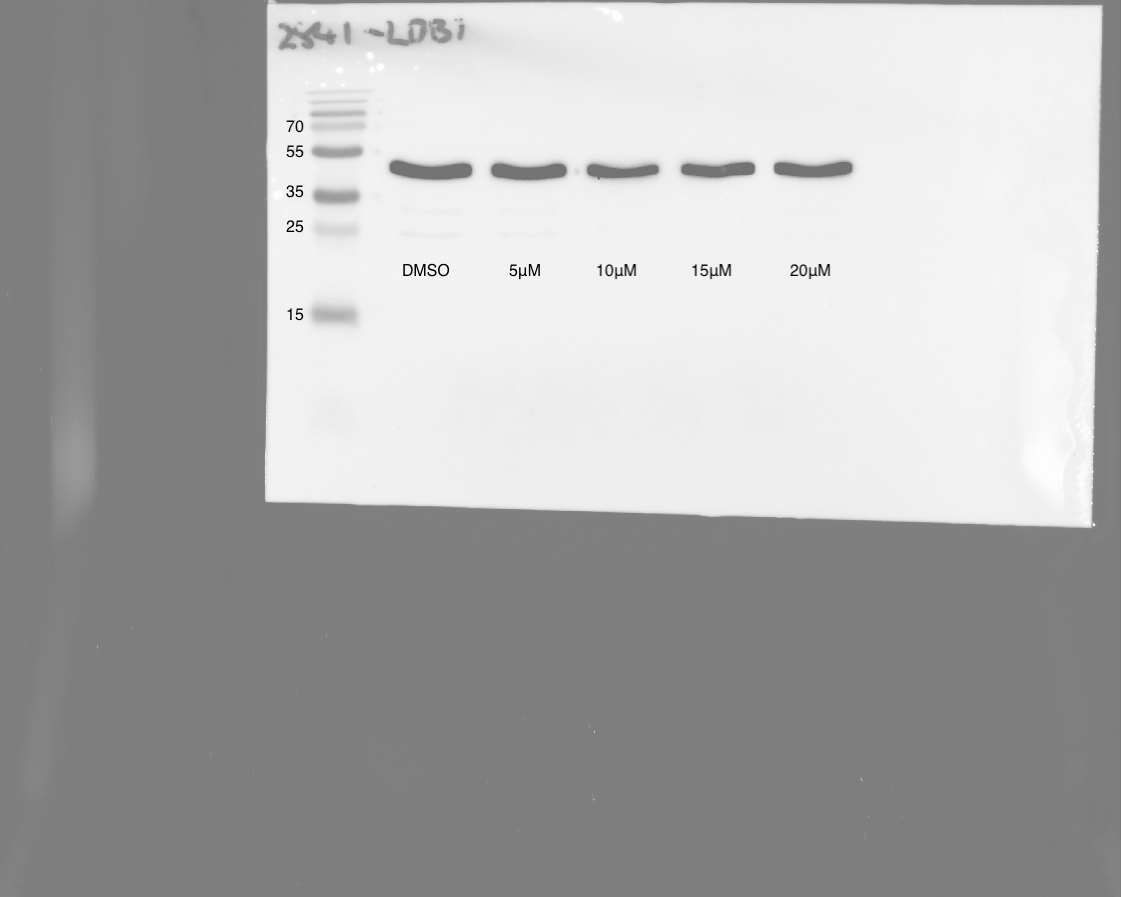

Supplement: Figure 3—source data 1. [file elife-106699-fig3-data1.zip › Figure 3ΓÇösource data 1 PDF files containing original western blots for Figure 3A, indicating the relevant bands and treatments./Raw data/LDB1 KOPT-K1 Abd-CRBN.tif]

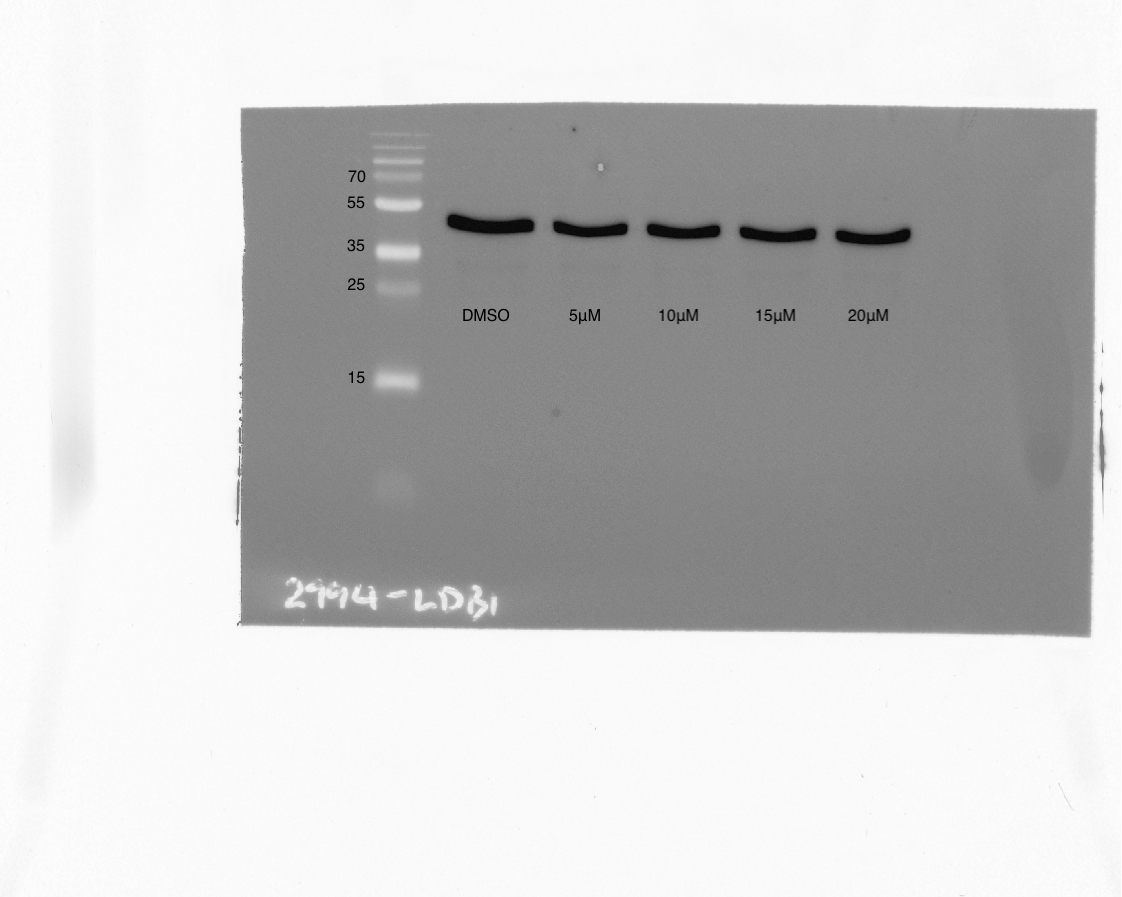

Supplement: Figure 3—source data 1. [file elife-106699-fig3-data1.zip › Figure 3ΓÇösource data 1 PDF files containing original western blots for Figure 3A, indicating the relevant bands and treatments./Raw data/LDB1 KOPT-K1 Abd-VHL.tif]

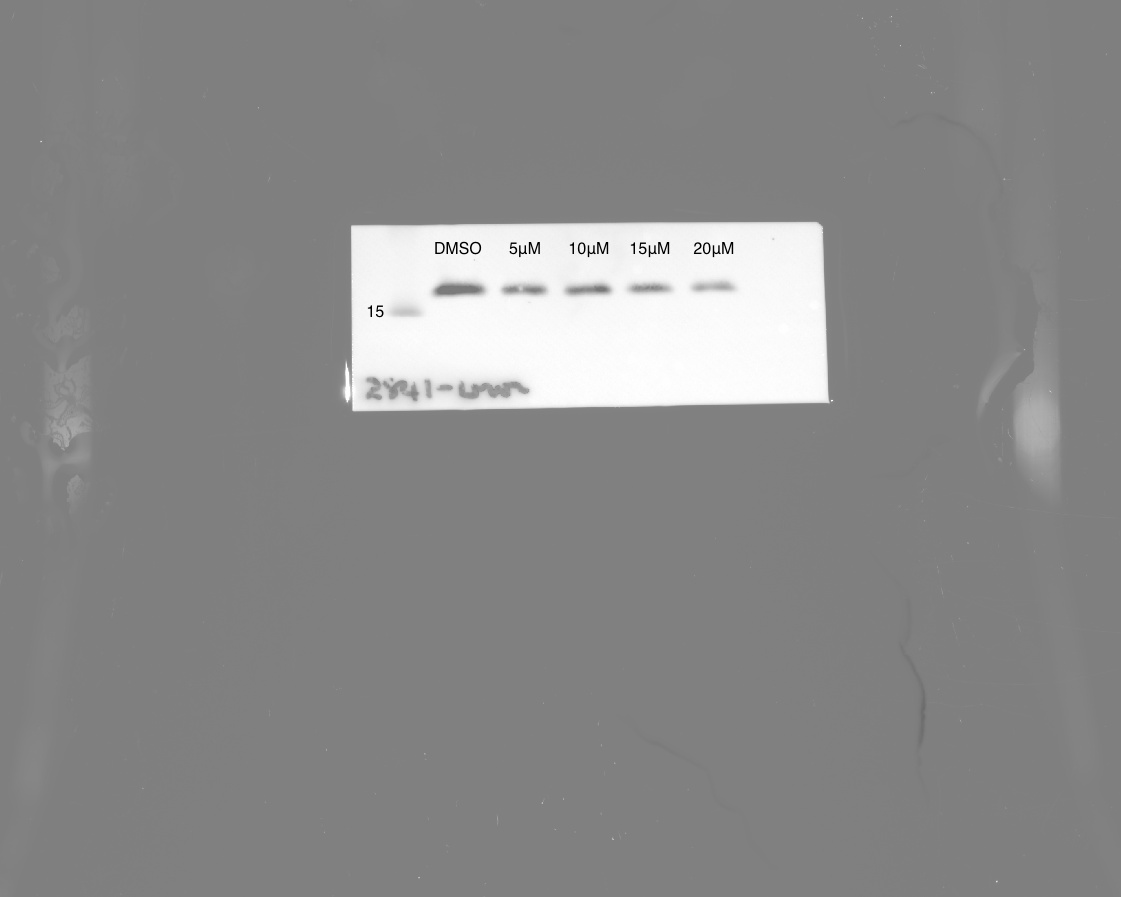

Supplement: Figure 3—source data 1. [file elife-106699-fig3-data1.zip › Figure 3ΓÇösource data 1 PDF files containing original western blots for Figure 3A, indicating the relevant bands and treatments./Raw data/LMO2 CCRF-CEM Abd-CRBN.tif]

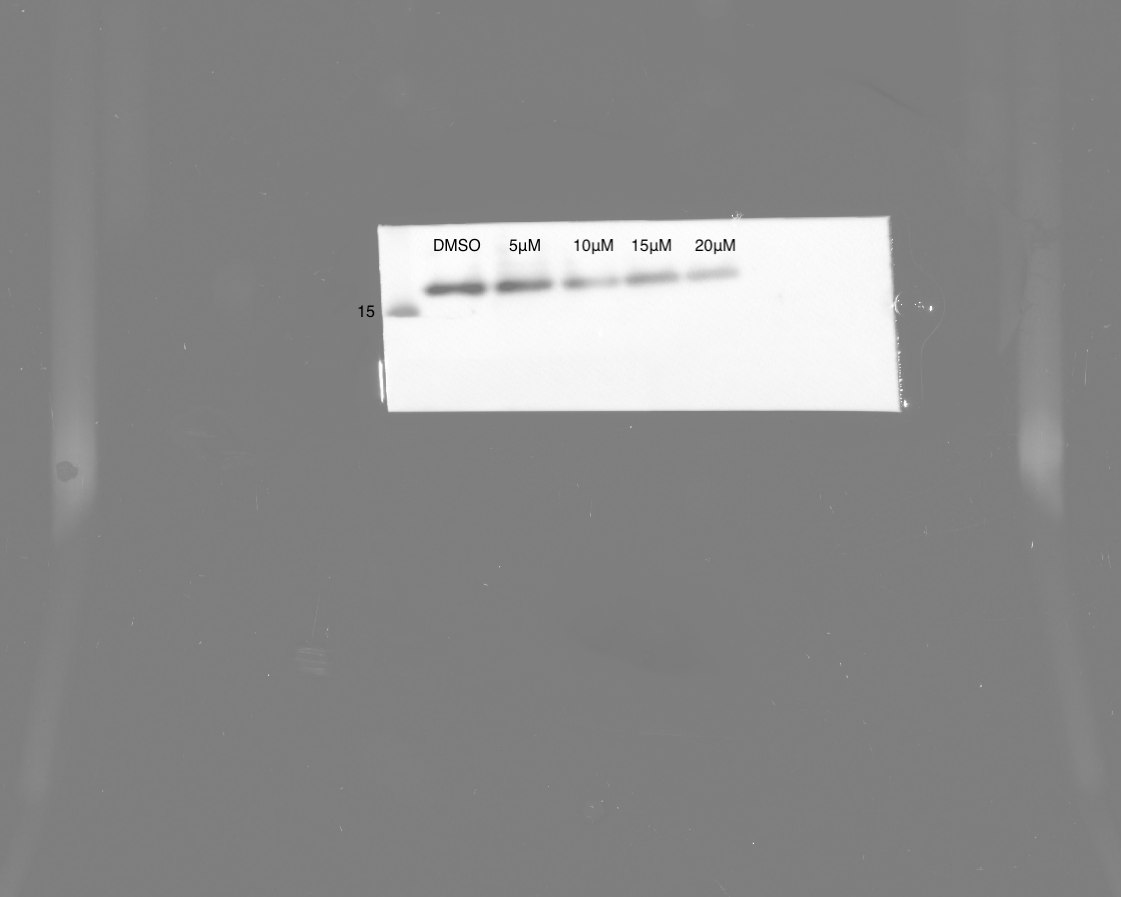

Supplement: Figure 3—source data 1. [file elife-106699-fig3-data1.zip › Figure 3ΓÇösource data 1 PDF files containing original western blots for Figure 3A, indicating the relevant bands and treatments./Raw data/LMO2 CCRF-CEM Abd-VHL.tif]

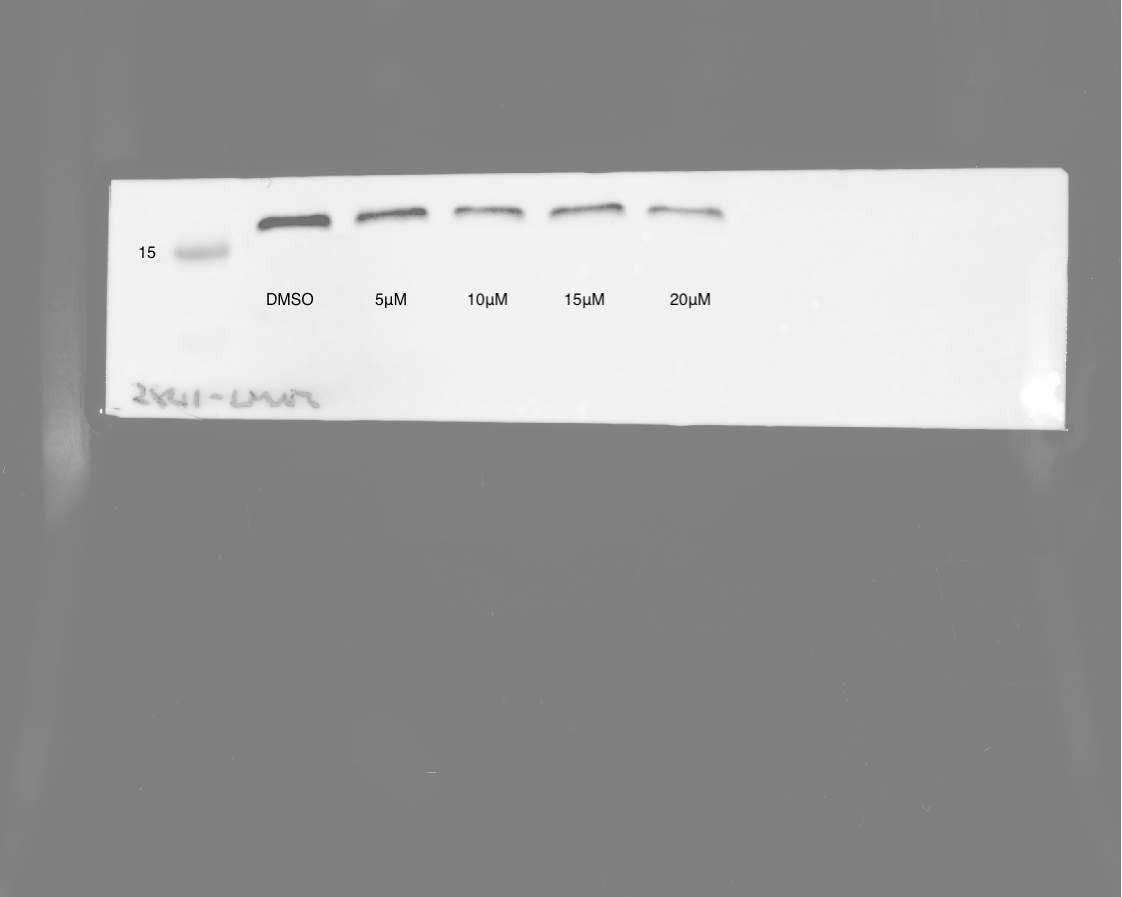

Supplement: Figure 3—source data 1. [file elife-106699-fig3-data1.zip › Figure 3ΓÇösource data 1 PDF files containing original western blots for Figure 3A, indicating the relevant bands and treatments./Raw data/LMO2 KOPT-K1 Abd-CRBN.tif]

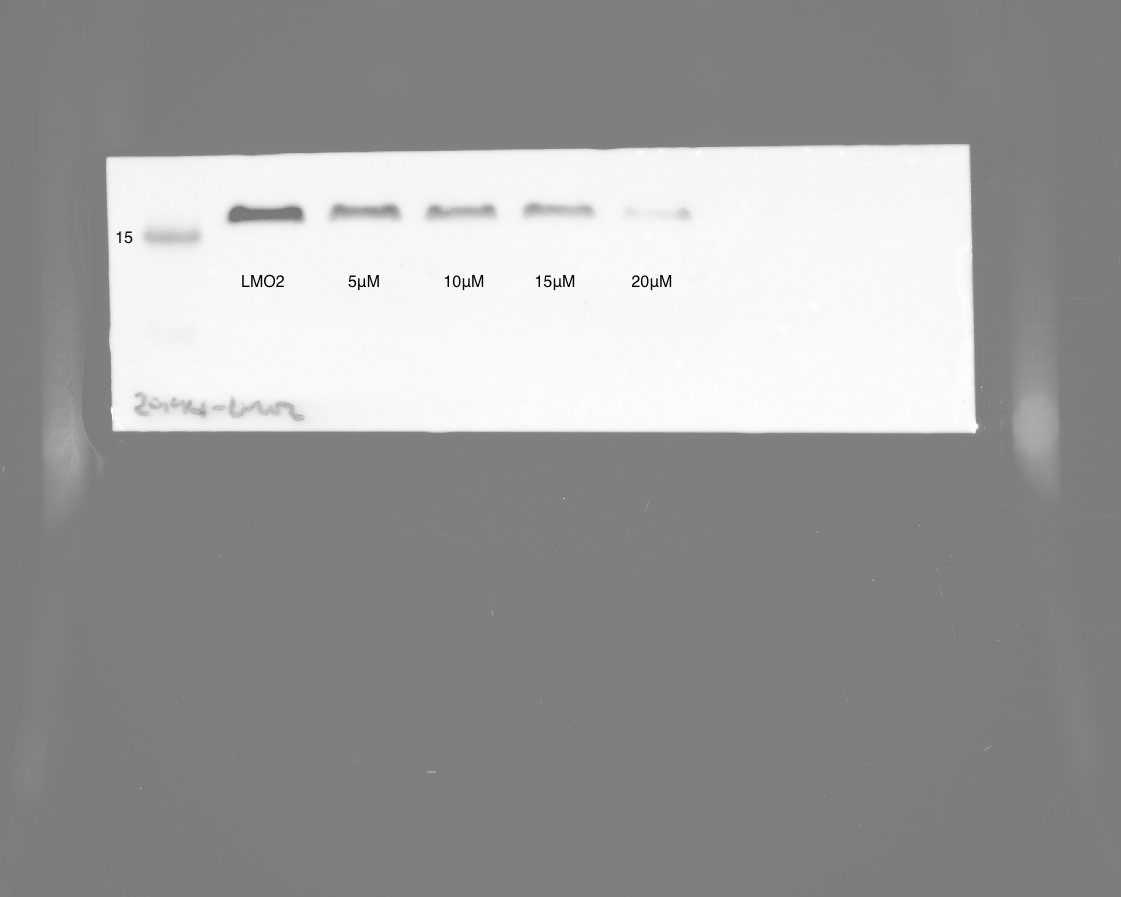

Supplement: Figure 3—source data 1. [file elife-106699-fig3-data1.zip › Figure 3ΓÇösource data 1 PDF files containing original western blots for Figure 3A, indicating the relevant bands and treatments./Raw data/LMO2 KOPT-K1 Abd-VHL.tif]

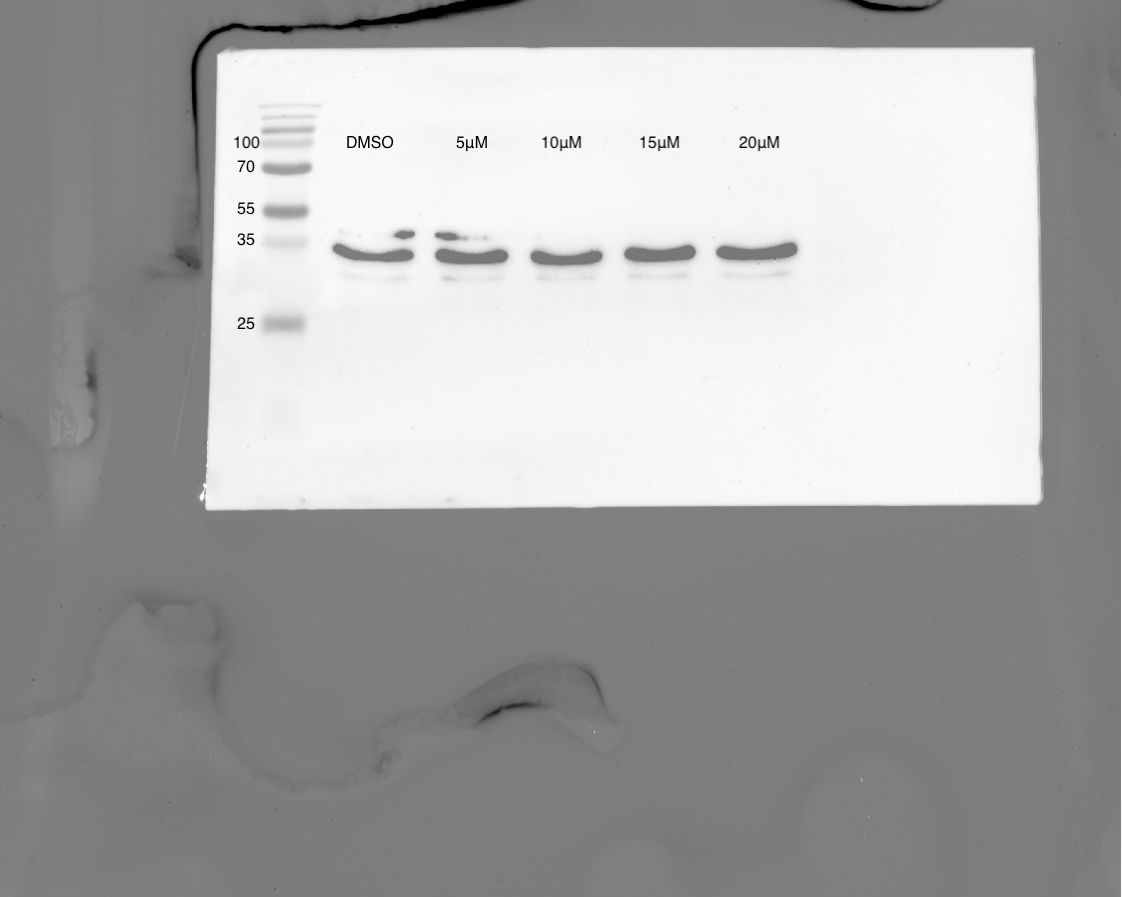

Supplement: Figure 3—source data 1. [file elife-106699-fig3-data1.zip › Figure 3ΓÇösource data 1 PDF files containing original western blots for Figure 3A, indicating the relevant bands and treatments./Raw data/Lyl-1 KOPT-K1 Abd-CRBN.tif]

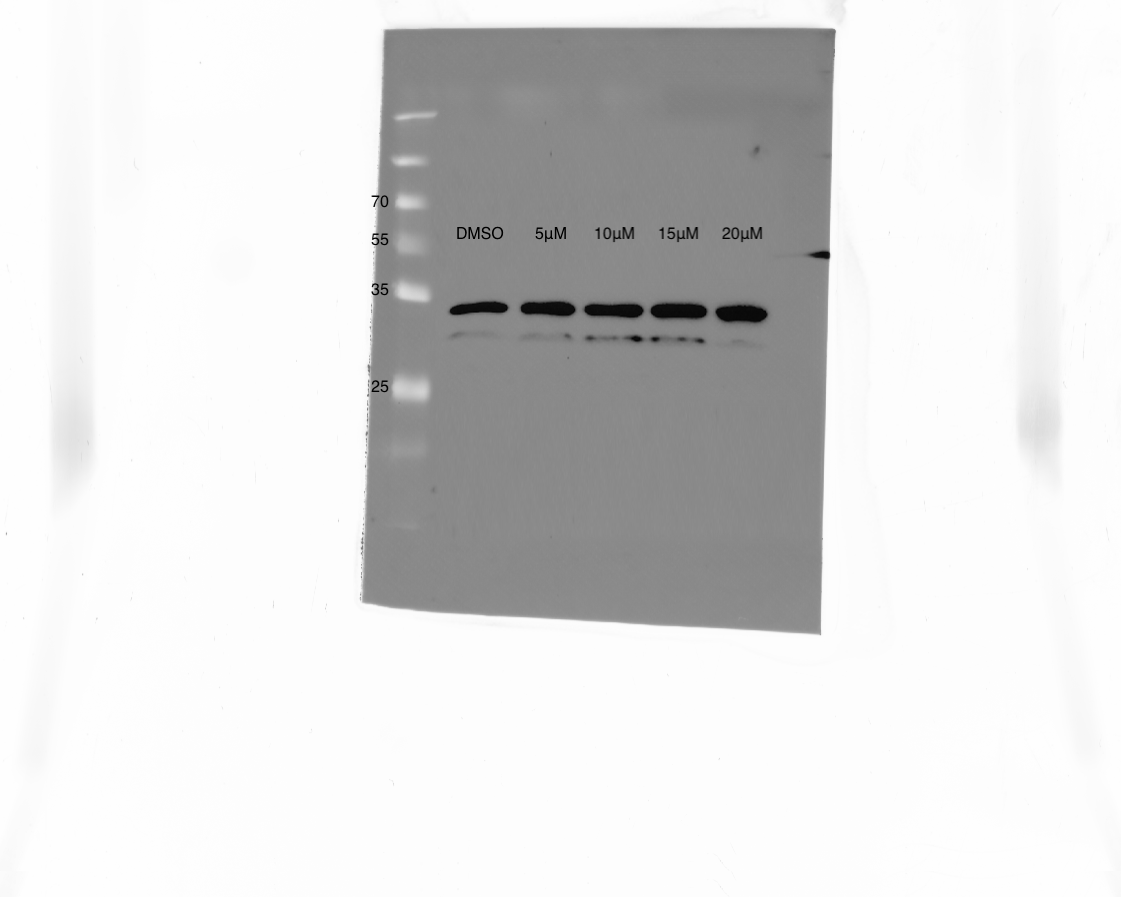

Supplement: Figure 3—source data 1. [file elife-106699-fig3-data1.zip › Figure 3ΓÇösource data 1 PDF files containing original western blots for Figure 3A, indicating the relevant bands and treatments./Raw data/Lyl1 CCRF-CEM Abd-CRBN.tif]

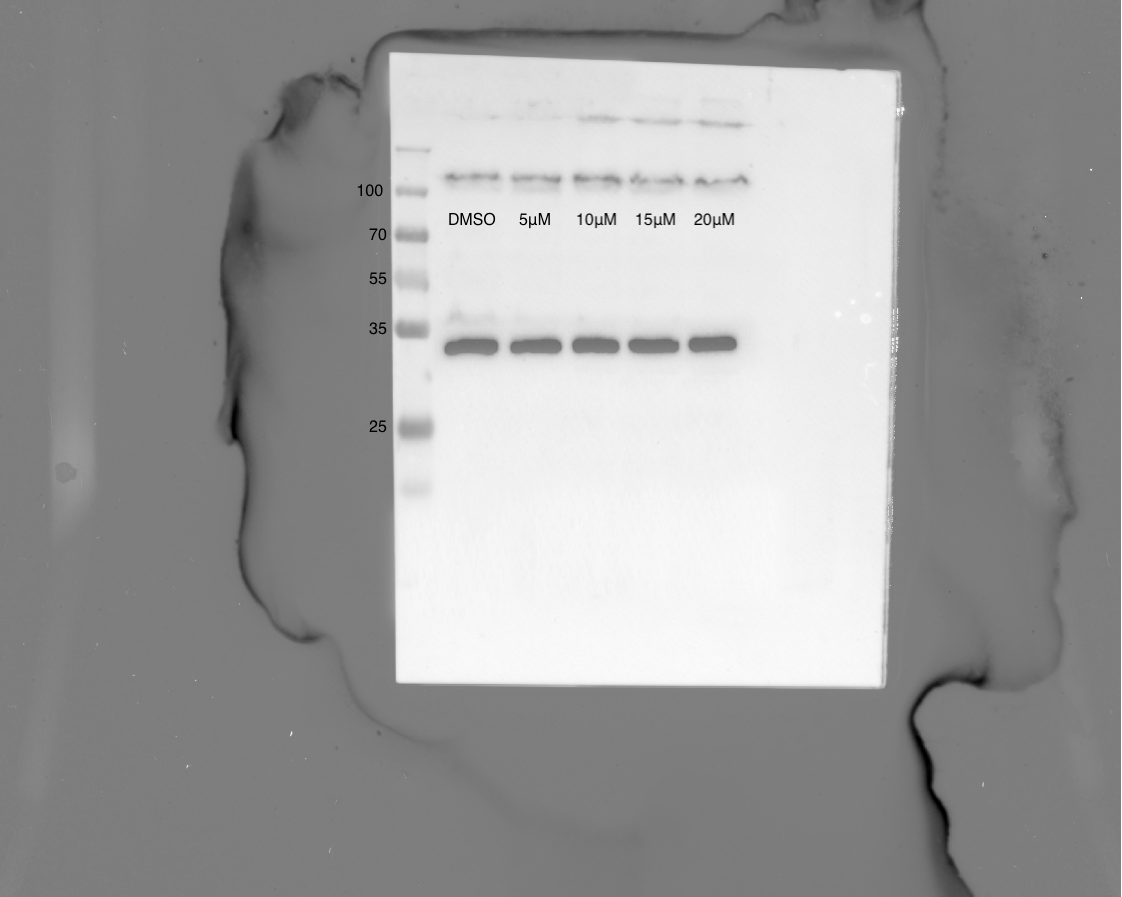

Supplement: Figure 3—source data 1. [file elife-106699-fig3-data1.zip › Figure 3ΓÇösource data 1 PDF files containing original western blots for Figure 3A, indicating the relevant bands and treatments./Raw data/Lyl1- CCRF-CEM Abd-VHL.tif]

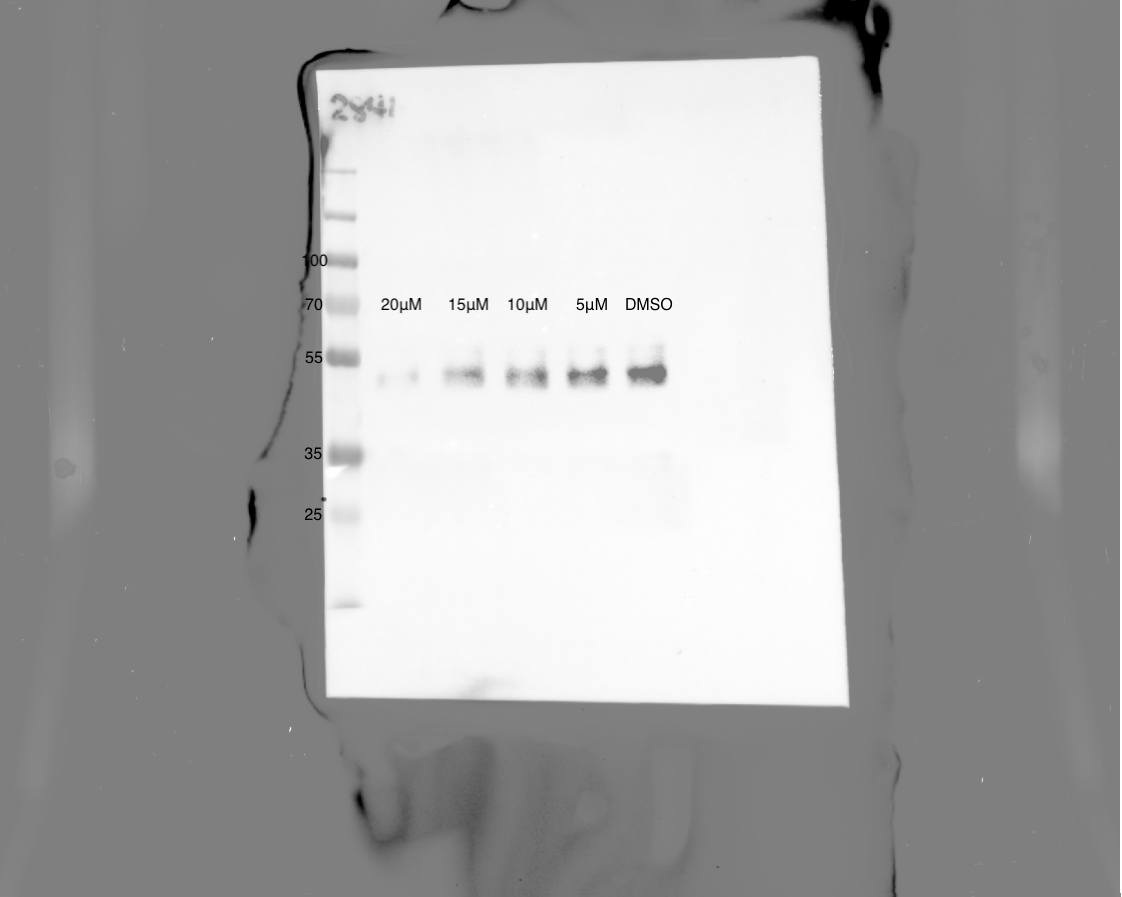

Supplement: Figure 3—source data 1. [file elife-106699-fig3-data1.zip › Figure 3ΓÇösource data 1 PDF files containing original western blots for Figure 3A, indicating the relevant bands and treatments./Raw data/Tal-1 CCRF-CEM Abd-CRBN.tif]

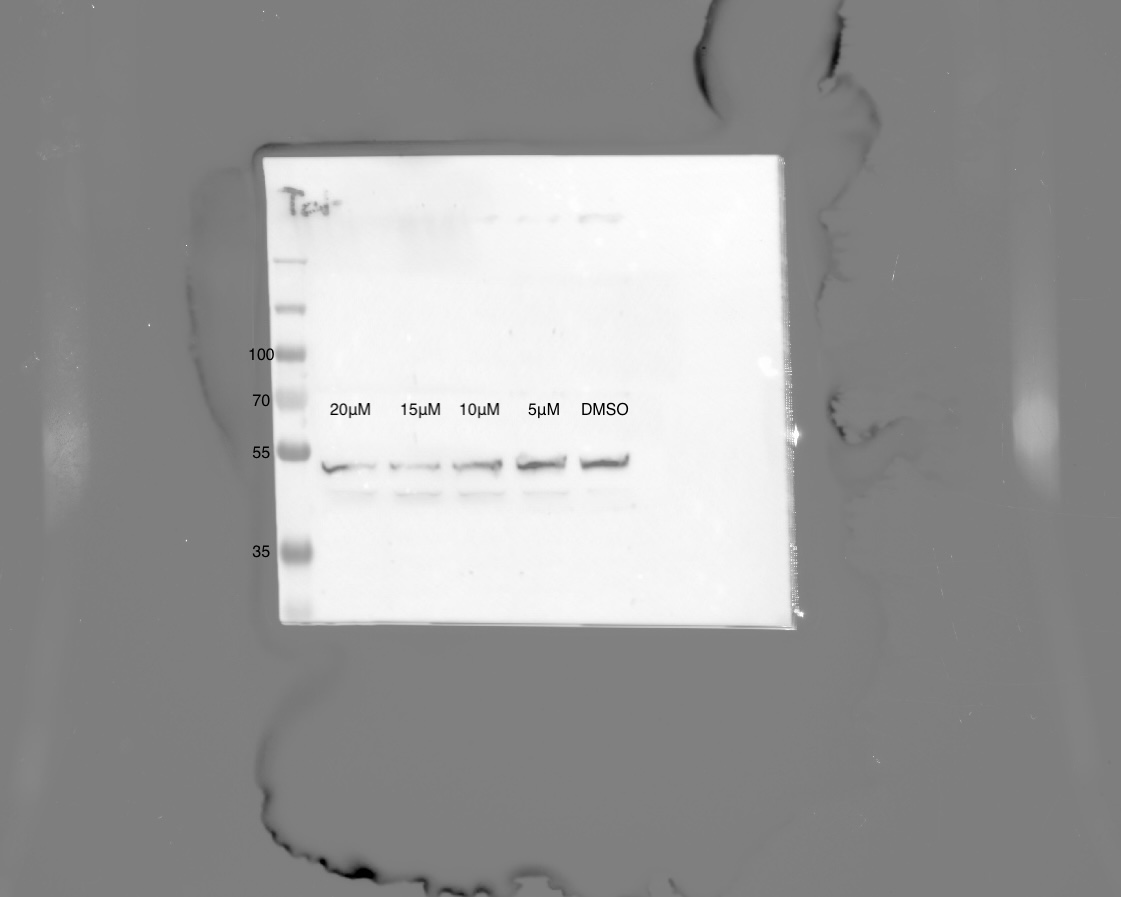

Supplement: Figure 3—source data 1. [file elife-106699-fig3-data1.zip › Figure 3ΓÇösource data 1 PDF files containing original western blots for Figure 3A, indicating the relevant bands and treatments./Raw data/Tal-1 CCRF-CEM Abd-VHL.jpg]

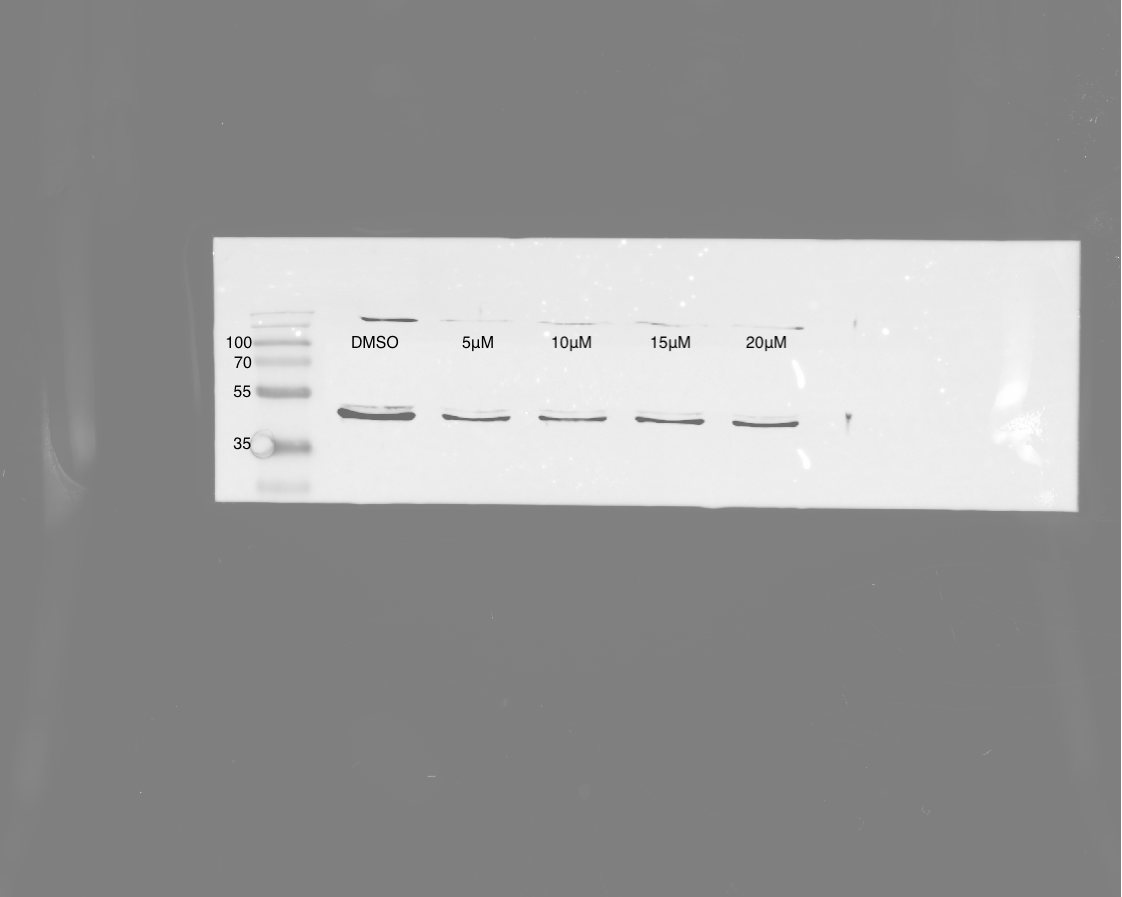

Supplement: Figure 3—source data 1. [file elife-106699-fig3-data1.zip › Figure 3ΓÇösource data 1 PDF files containing original western blots for Figure 3A, indicating the relevant bands and treatments./Raw data/Tal-1 KOPT-K1 Abd-CRBN.tif]

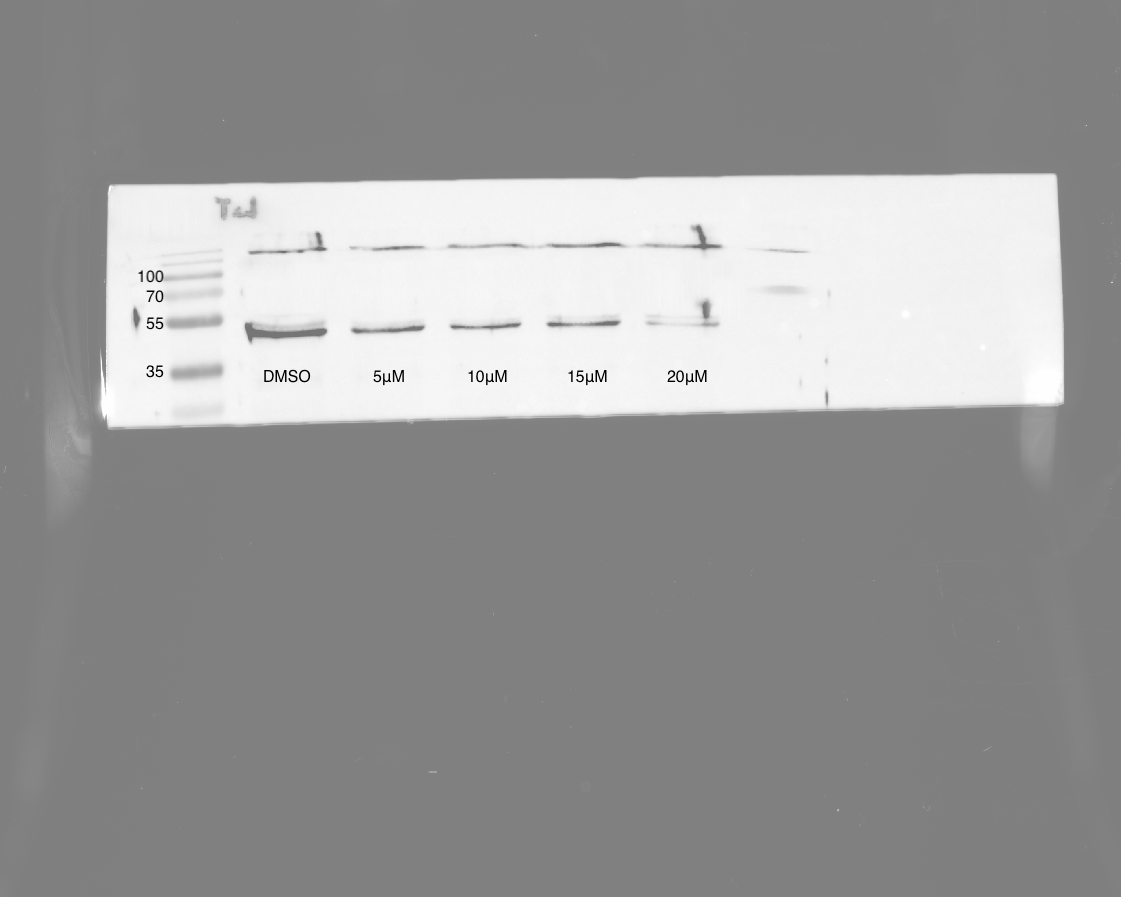

Supplement: Figure 3—source data 1. [file elife-106699-fig3-data1.zip › Figure 3ΓÇösource data 1 PDF files containing original western blots for Figure 3A, indicating the relevant bands and treatments./Raw data/Tal1 KOPT-K1 Abd-VHL.tif]

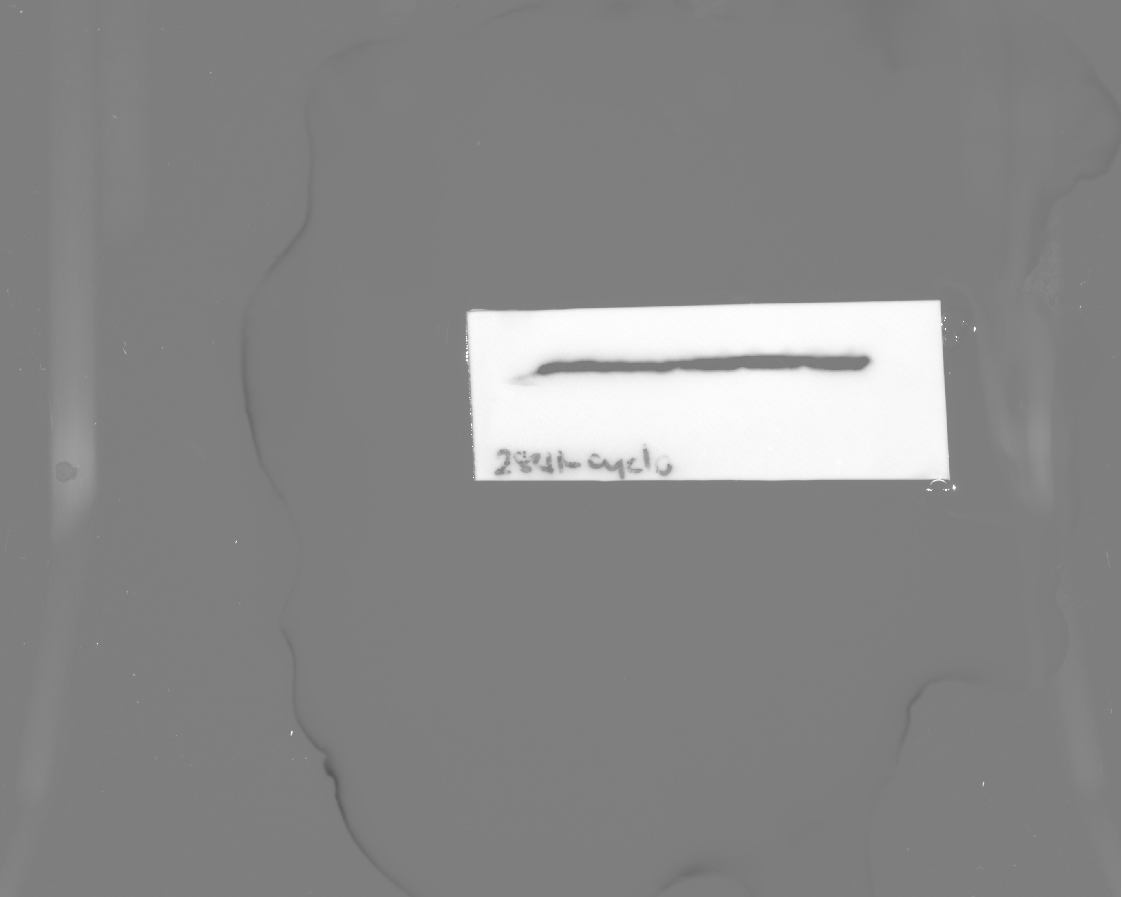

Supplement: Figure 3—source data 2. [file elife-106699-fig3-data2.zip › Figure 3ΓÇösource data 2 Original files for Western blot analysis displayed in Figure 3A./Cyclophilin CCRF-CEM Abd-CRBN.tif]

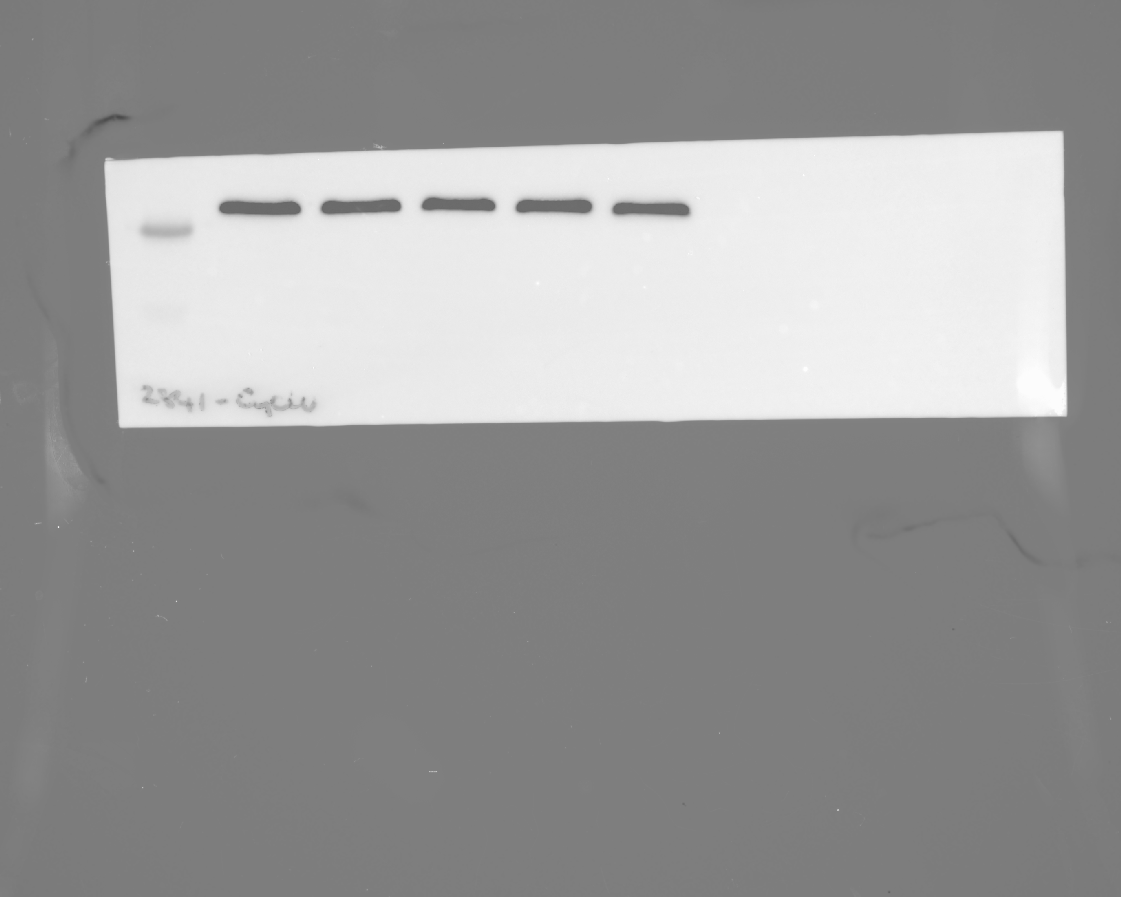

Supplement: Figure 3—source data 2. [file elife-106699-fig3-data2.zip › Figure 3ΓÇösource data 2 Original files for Western blot analysis displayed in Figure 3A./Cyclophilin KOPT-K1 Abd-CRBN.tif]

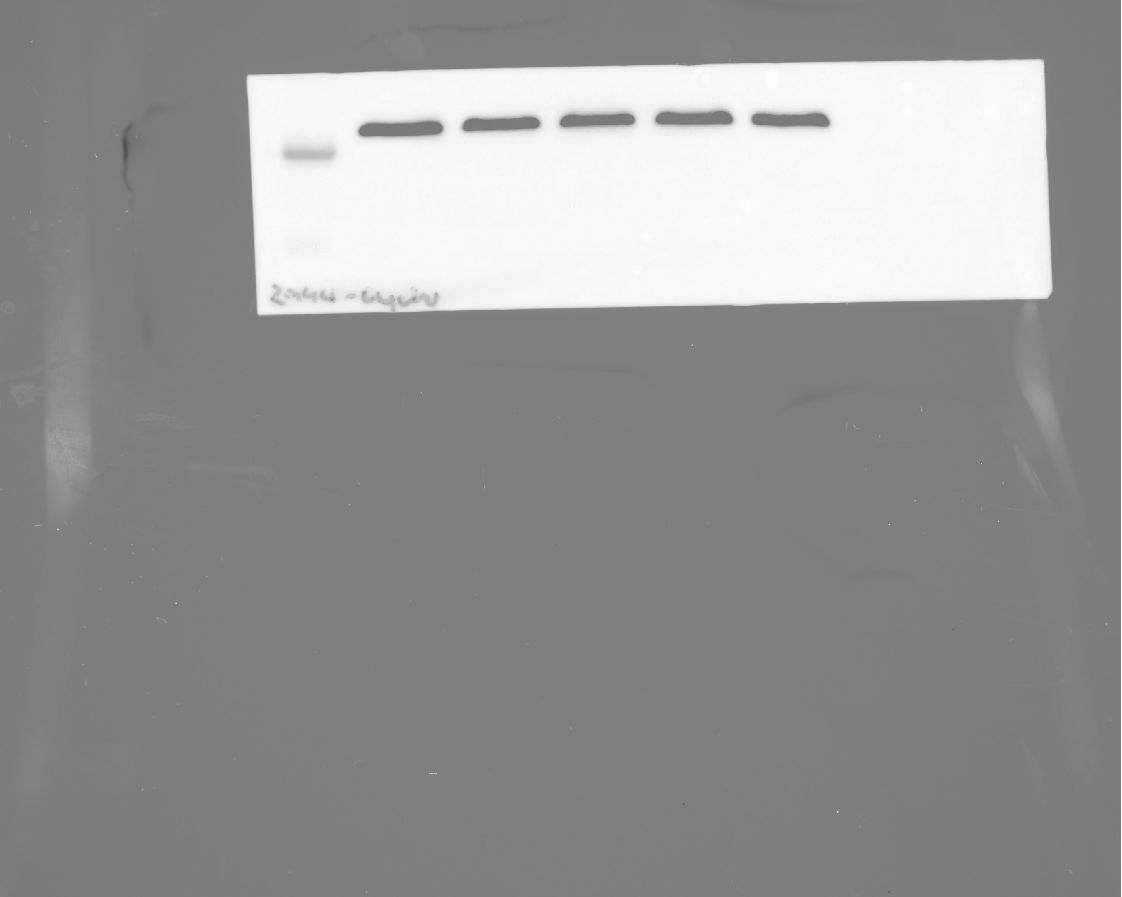

Supplement: Figure 3—source data 2. [file elife-106699-fig3-data2.zip › Figure 3ΓÇösource data 2 Original files for Western blot analysis displayed in Figure 3A./Cyclophilin KOPT-K1 Abd-VHL.tif]

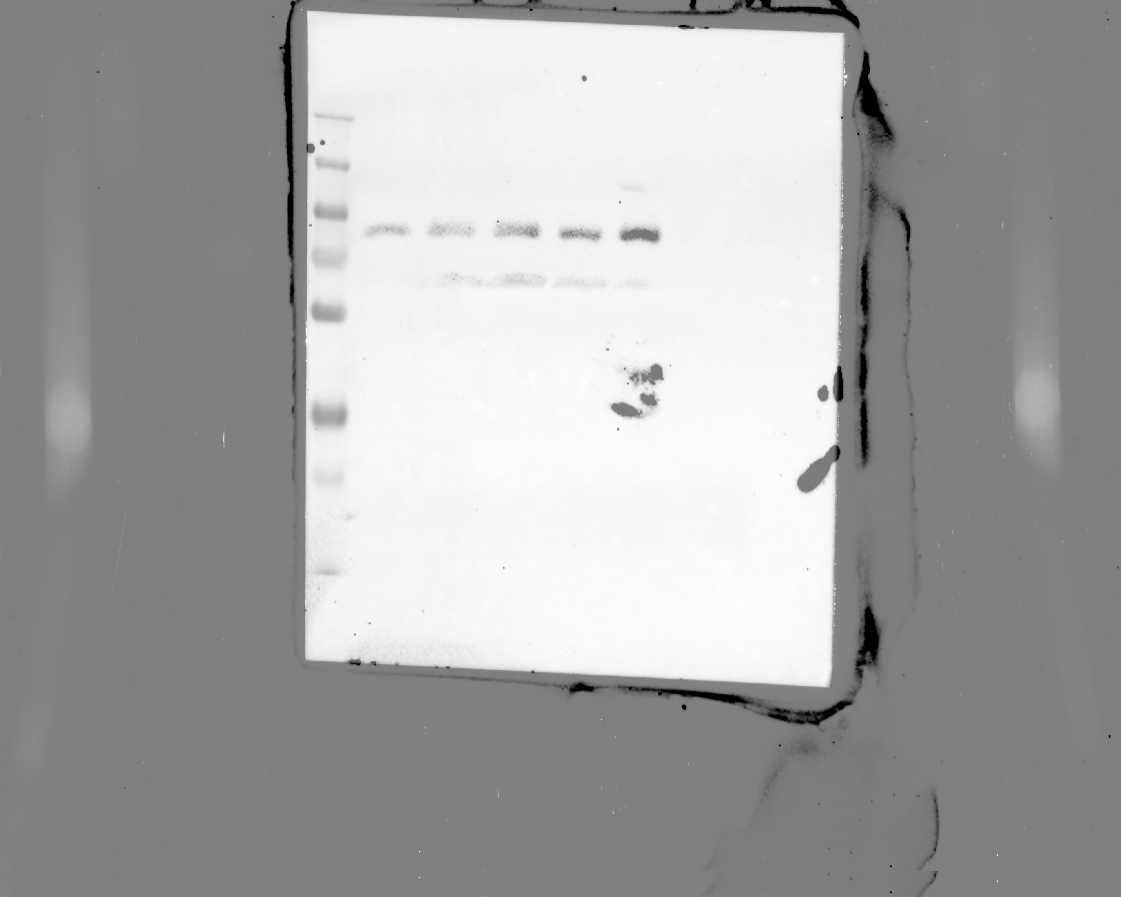

Supplement: Figure 3—source data 2. [file elife-106699-fig3-data2.zip › Figure 3ΓÇösource data 2 Original files for Western blot analysis displayed in Figure 3A./E2A CCRF-CEM Abd-CRBN.tif]

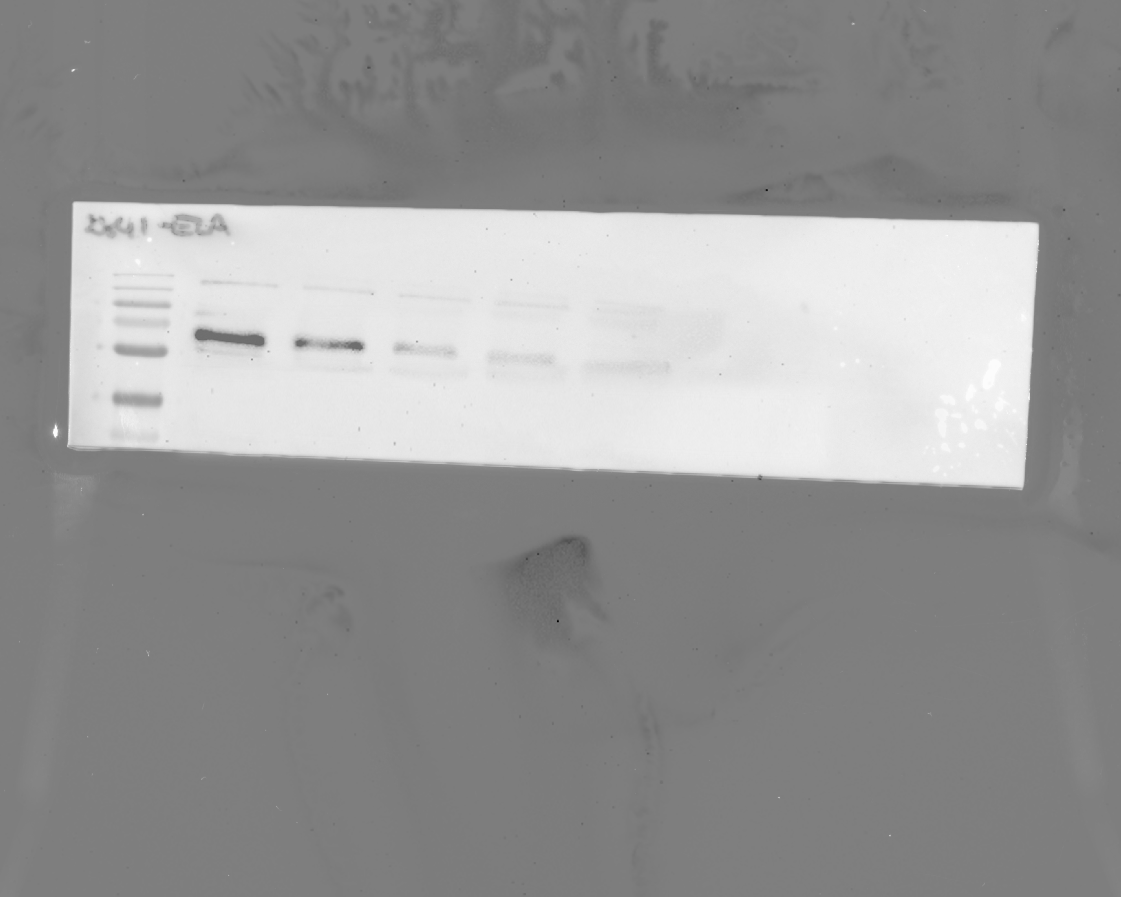

Supplement: Figure 3—source data 2. [file elife-106699-fig3-data2.zip › Figure 3ΓÇösource data 2 Original files for Western blot analysis displayed in Figure 3A./E2A KOPT-K1 Abd-CRBN.tif]

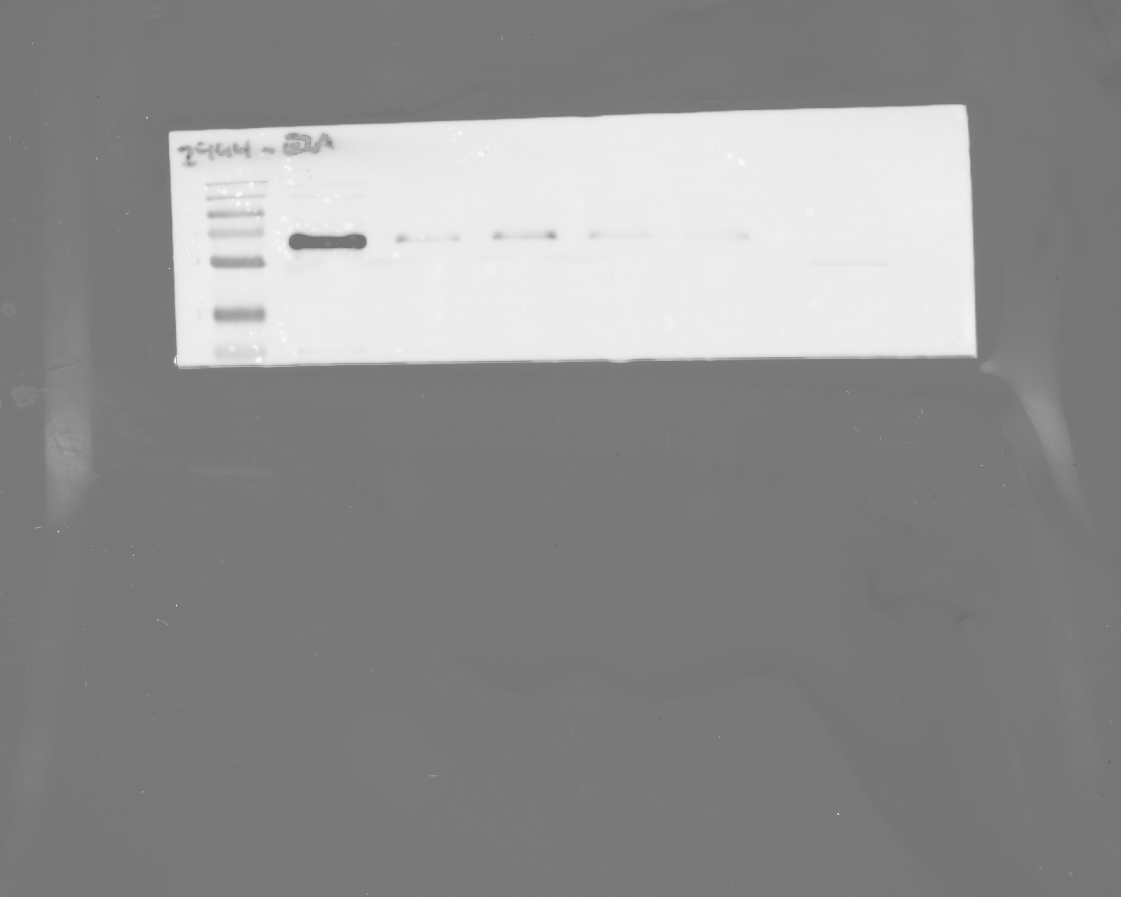

Supplement: Figure 3—source data 2. [file elife-106699-fig3-data2.zip › Figure 3ΓÇösource data 2 Original files for Western blot analysis displayed in Figure 3A./E2A KOPT-K1 Abd-VHL.tif]

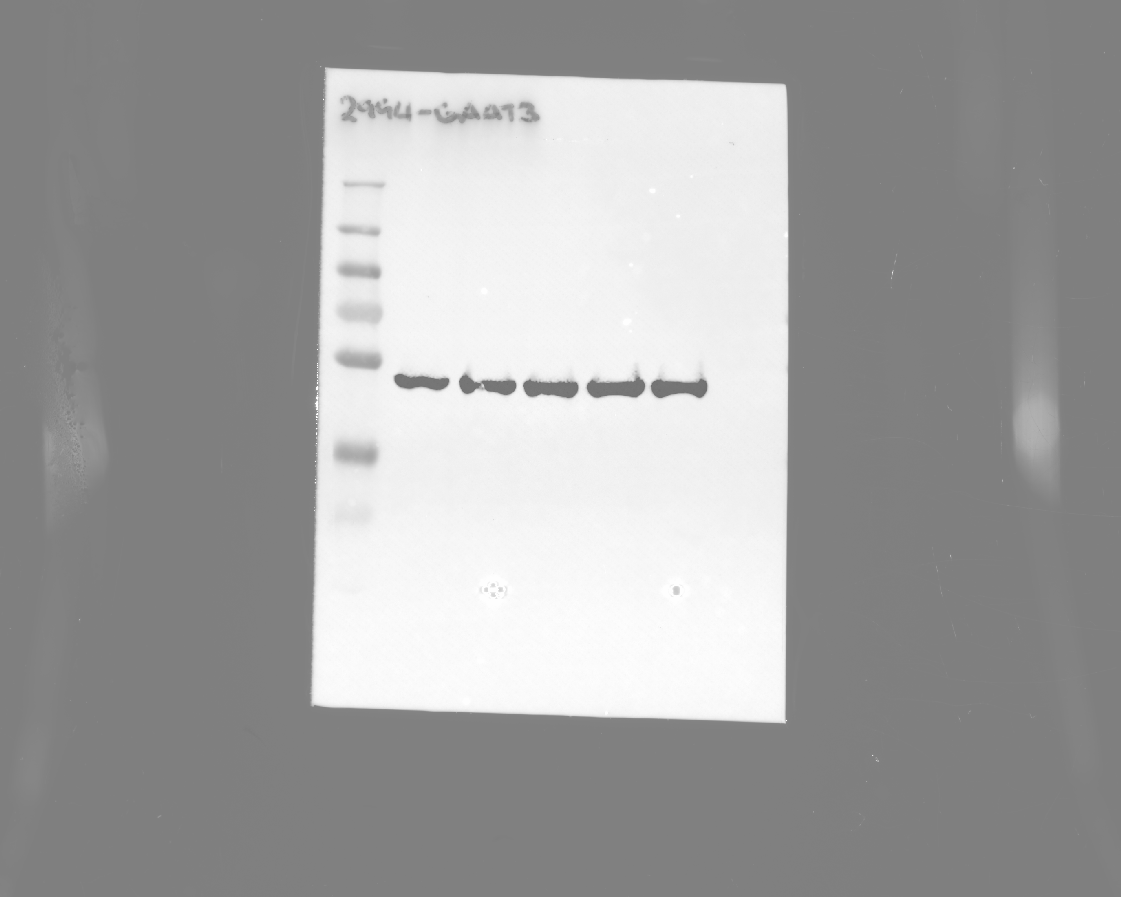

Supplement: Figure 3—source data 2. [file elife-106699-fig3-data2.zip › Figure 3ΓÇösource data 2 Original files for Western blot analysis displayed in Figure 3A./GATA3 CCRF-CEM Abd-CRBN.tif]

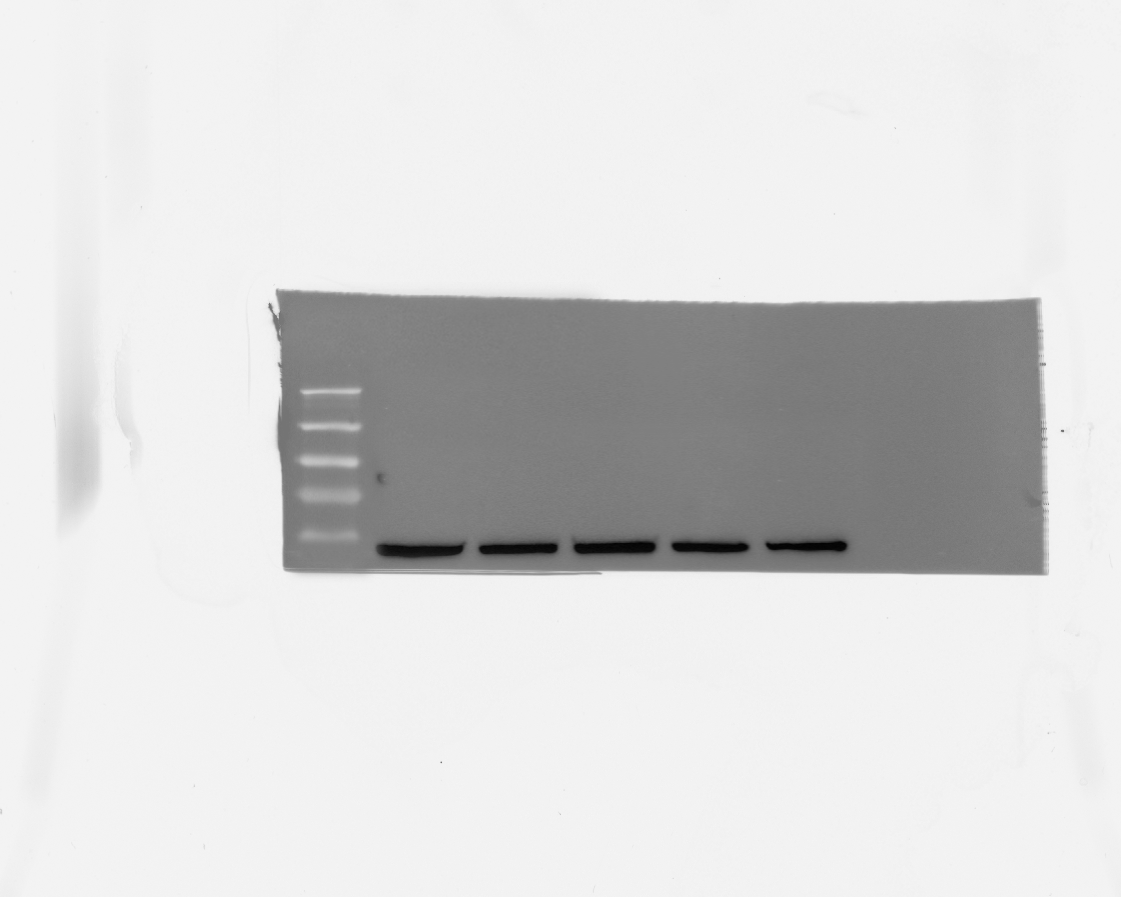

Supplement: Figure 3—source data 2. [file elife-106699-fig3-data2.zip › Figure 3ΓÇösource data 2 Original files for Western blot analysis displayed in Figure 3A./GATA3 CCRF-CEM Abd-VHL.tif]

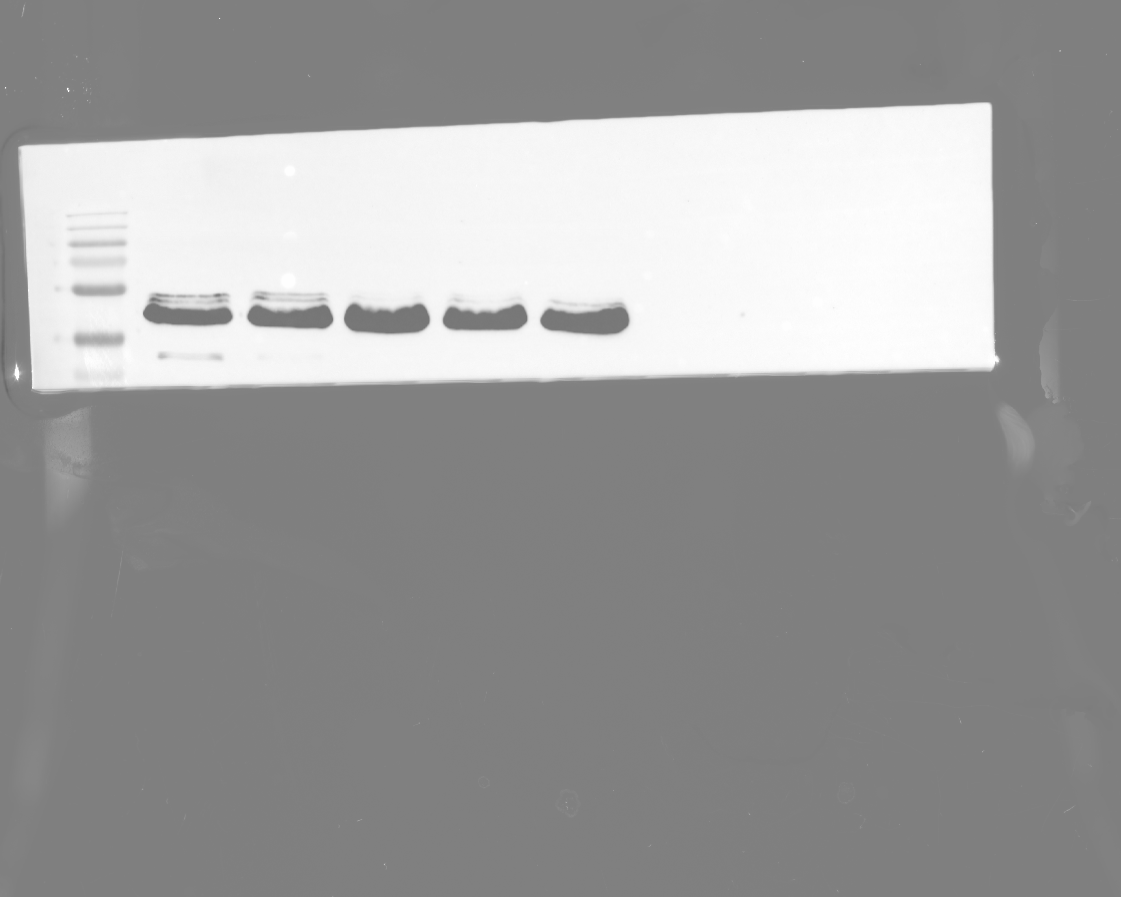

Supplement: Figure 3—source data 2. [file elife-106699-fig3-data2.zip › Figure 3ΓÇösource data 2 Original files for Western blot analysis displayed in Figure 3A./GATA3 KOPT-K1 Abd-CRBN.tif]

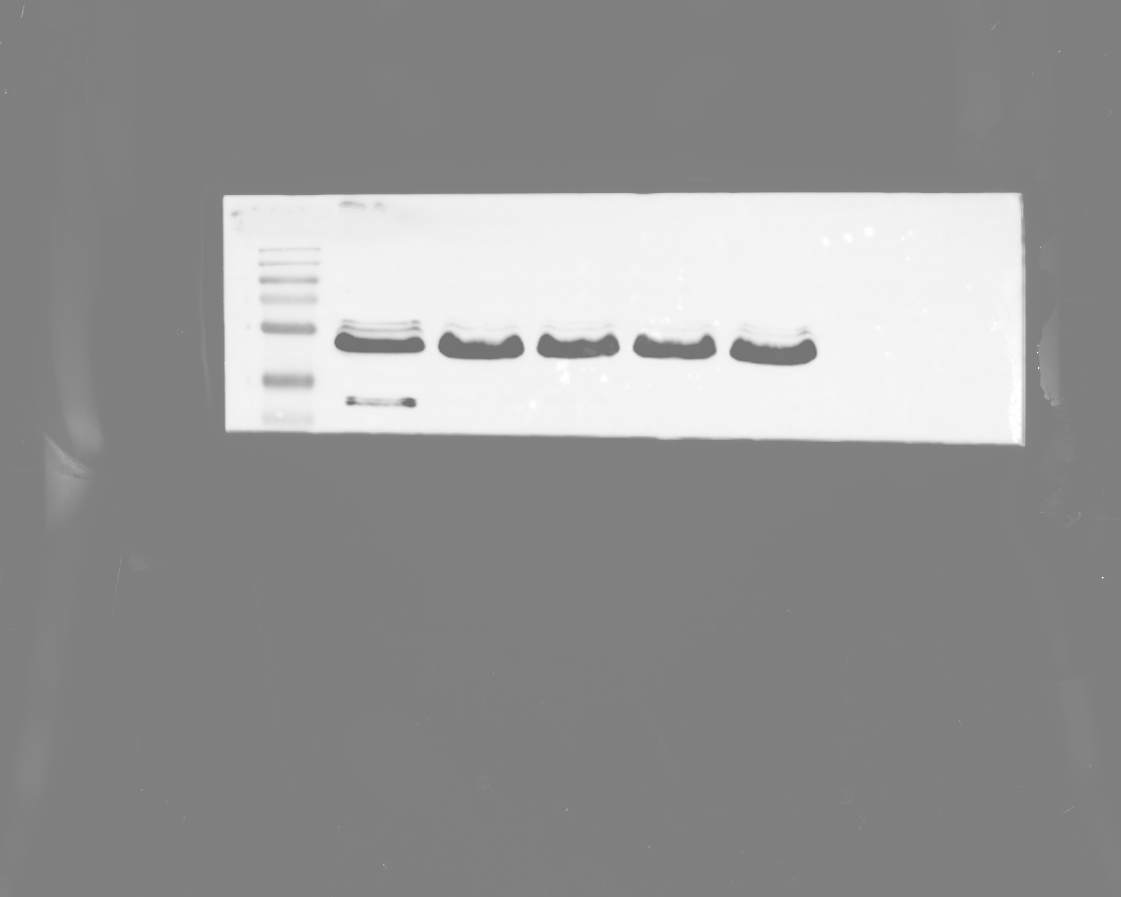

Supplement: Figure 3—source data 2. [file elife-106699-fig3-data2.zip › Figure 3ΓÇösource data 2 Original files for Western blot analysis displayed in Figure 3A./GATA3 KOPT-K1 Abd-VHL.tif]

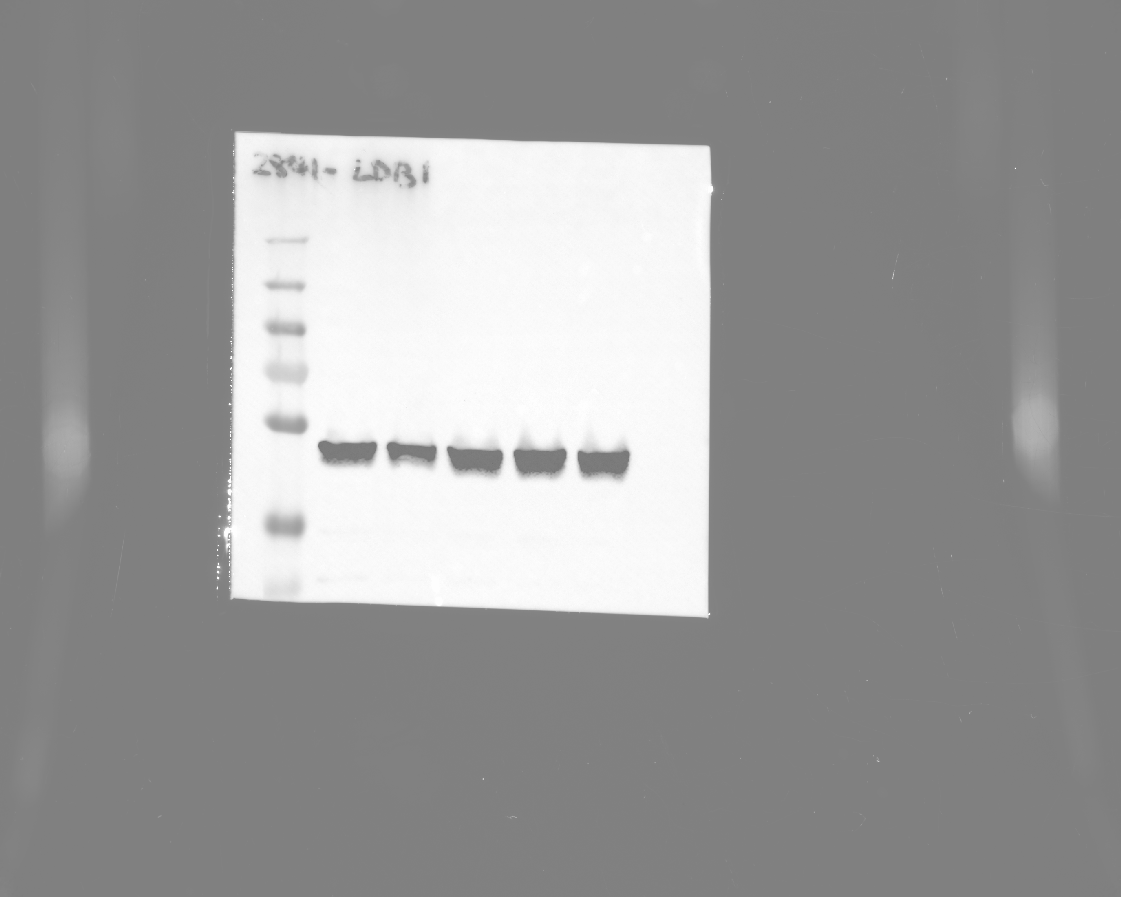

Supplement: Figure 3—source data 2. [file elife-106699-fig3-data2.zip › Figure 3ΓÇösource data 2 Original files for Western blot analysis displayed in Figure 3A./LDB1 CCRF-CEM Abd-CRBN.tif]

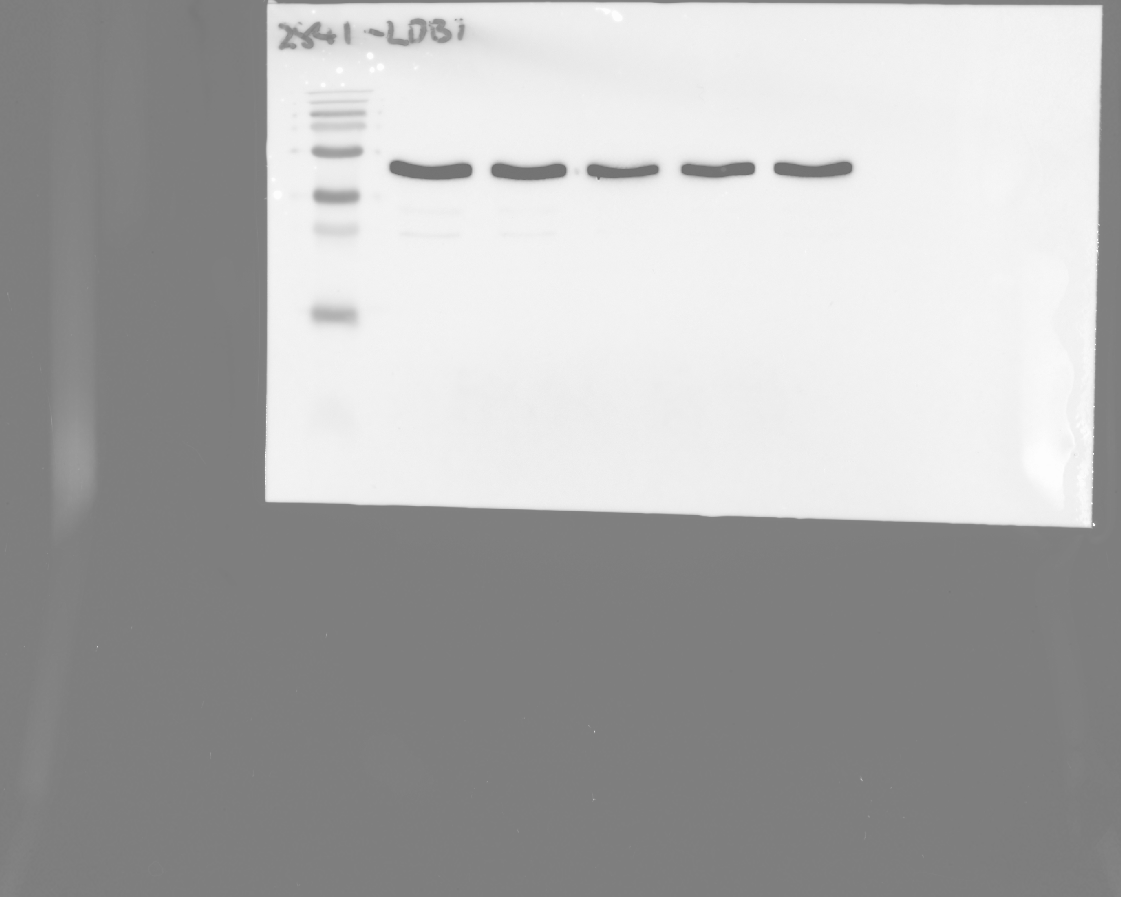

Supplement: Figure 3—source data 2. [file elife-106699-fig3-data2.zip › Figure 3ΓÇösource data 2 Original files for Western blot analysis displayed in Figure 3A./LDB1 KOPT-K1 Abd-CRBN.tif]

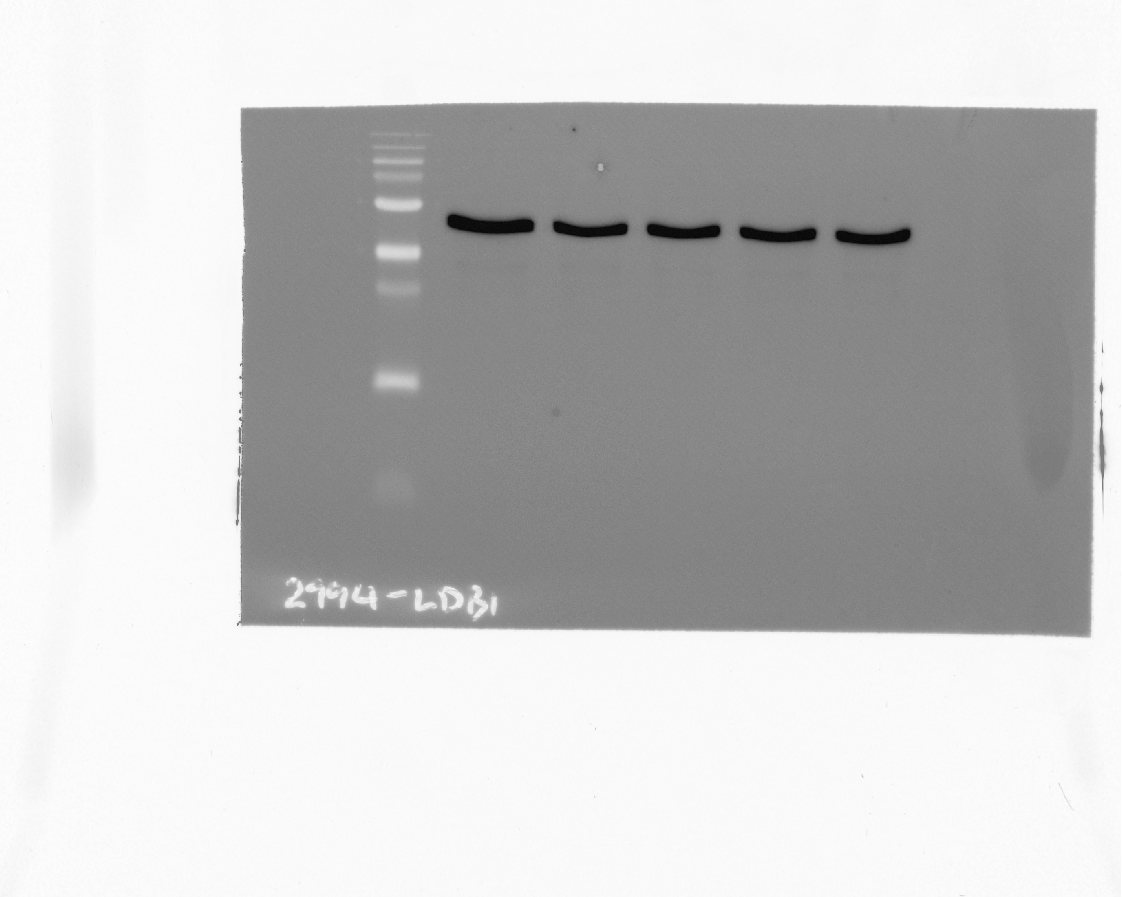

Supplement: Figure 3—source data 2. [file elife-106699-fig3-data2.zip › Figure 3ΓÇösource data 2 Original files for Western blot analysis displayed in Figure 3A./LDB1 KOPT-K1 Abd-VHL.tif]

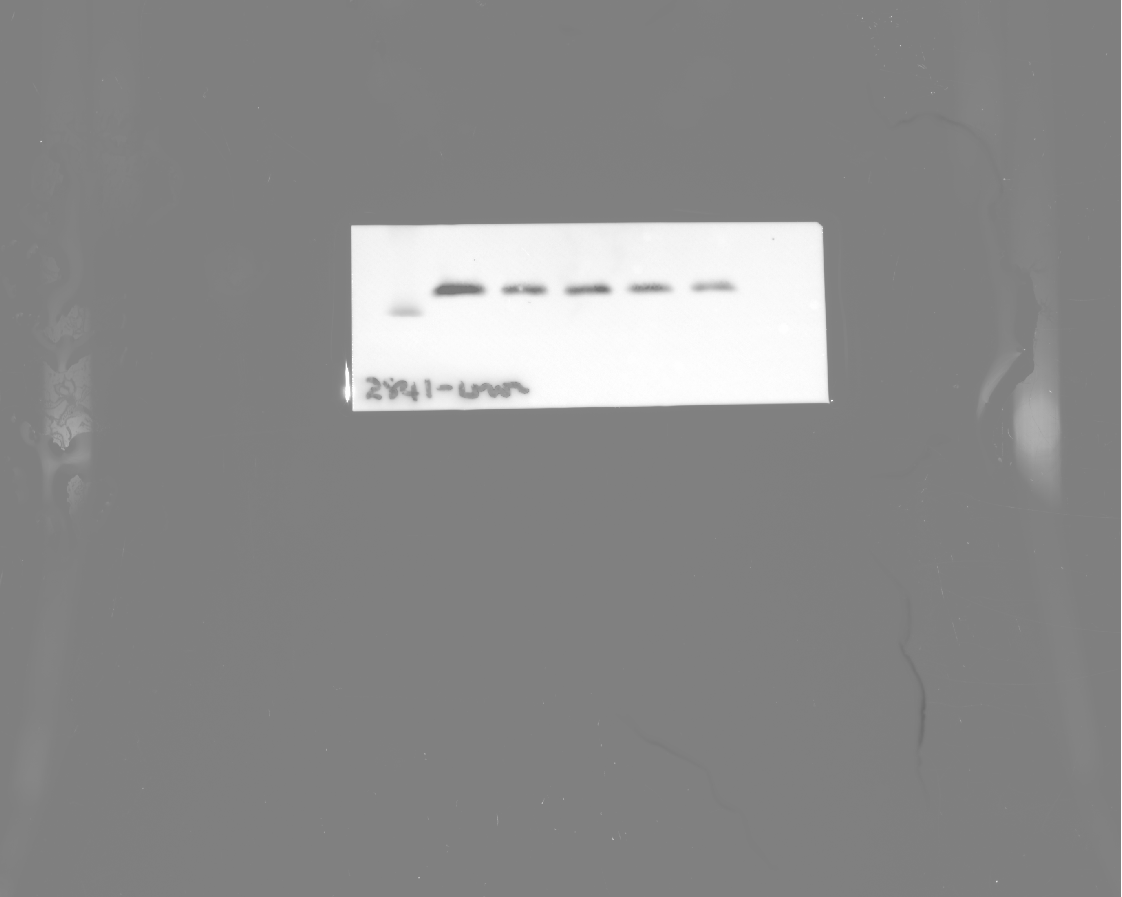

Supplement: Figure 3—source data 2. [file elife-106699-fig3-data2.zip › Figure 3ΓÇösource data 2 Original files for Western blot analysis displayed in Figure 3A./LMO2 CCRF-CEM Abd-CRBN.tif]

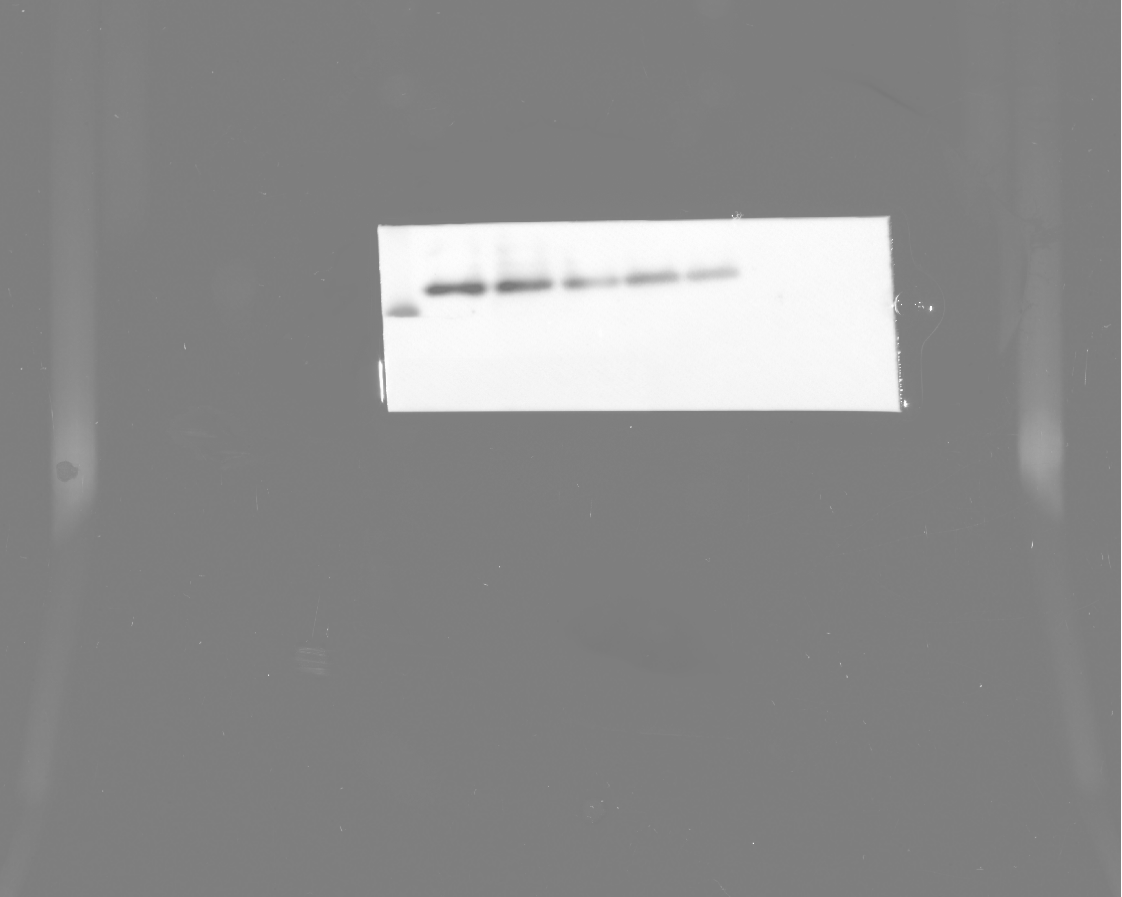

Supplement: Figure 3—source data 2. [file elife-106699-fig3-data2.zip › Figure 3ΓÇösource data 2 Original files for Western blot analysis displayed in Figure 3A./LMO2 CCRF-CEM Abd-VHL.tif]

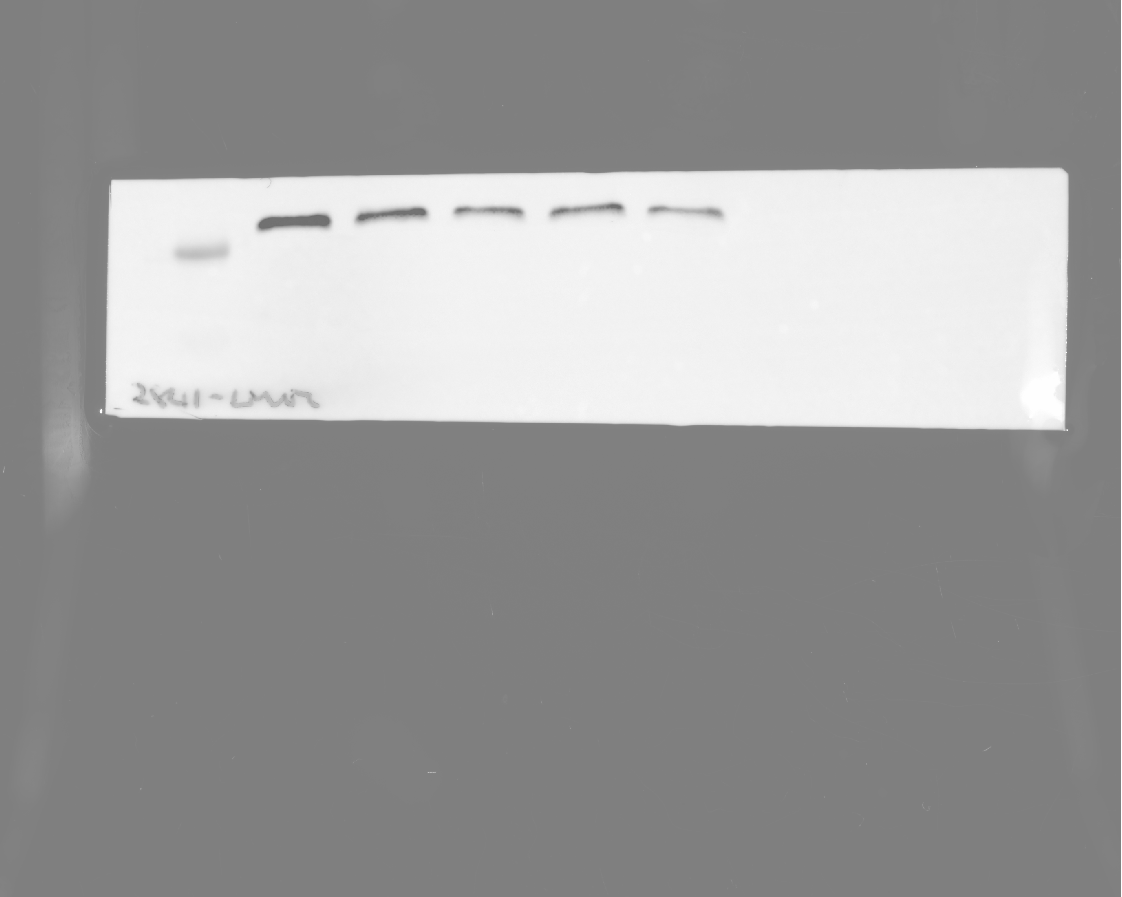

Supplement: Figure 3—source data 2. [file elife-106699-fig3-data2.zip › Figure 3ΓÇösource data 2 Original files for Western blot analysis displayed in Figure 3A./LMO2 KOPT-K1 Abd-CRBN.tif]

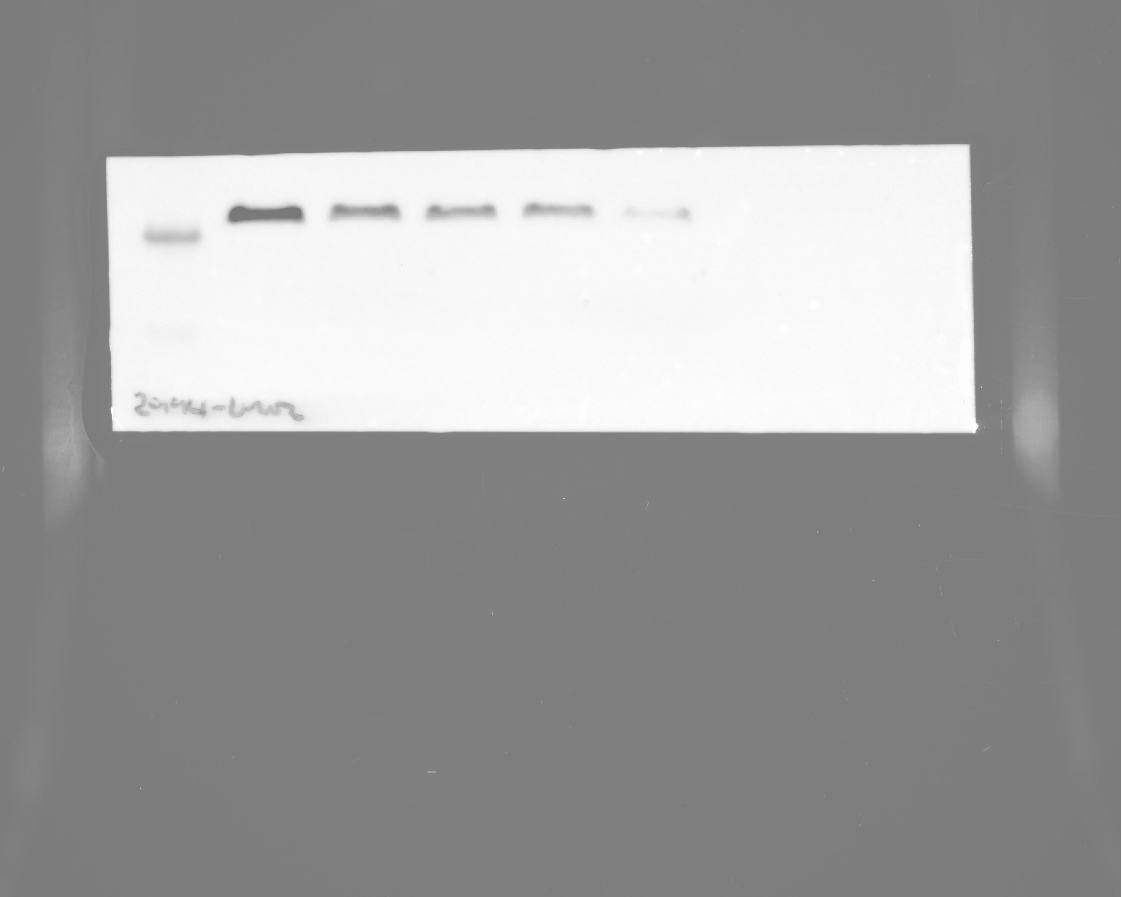

Supplement: Figure 3—source data 2. [file elife-106699-fig3-data2.zip › Figure 3ΓÇösource data 2 Original files for Western blot analysis displayed in Figure 3A./LMO2 KOPT-K1 Abd-VHL.tif]

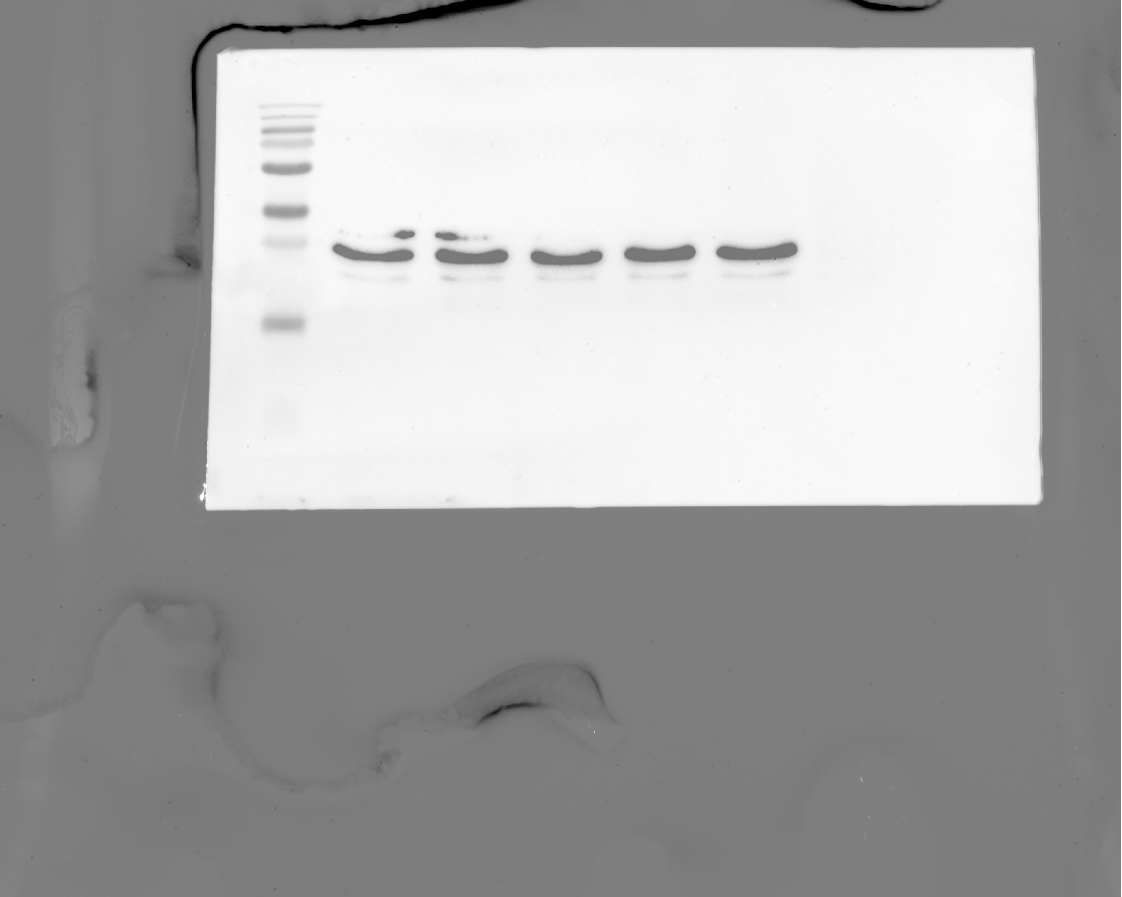

Supplement: Figure 3—source data 2. [file elife-106699-fig3-data2.zip › Figure 3ΓÇösource data 2 Original files for Western blot analysis displayed in Figure 3A./Lyl-1 KOPT-K1 Abd-CRBN.tif]

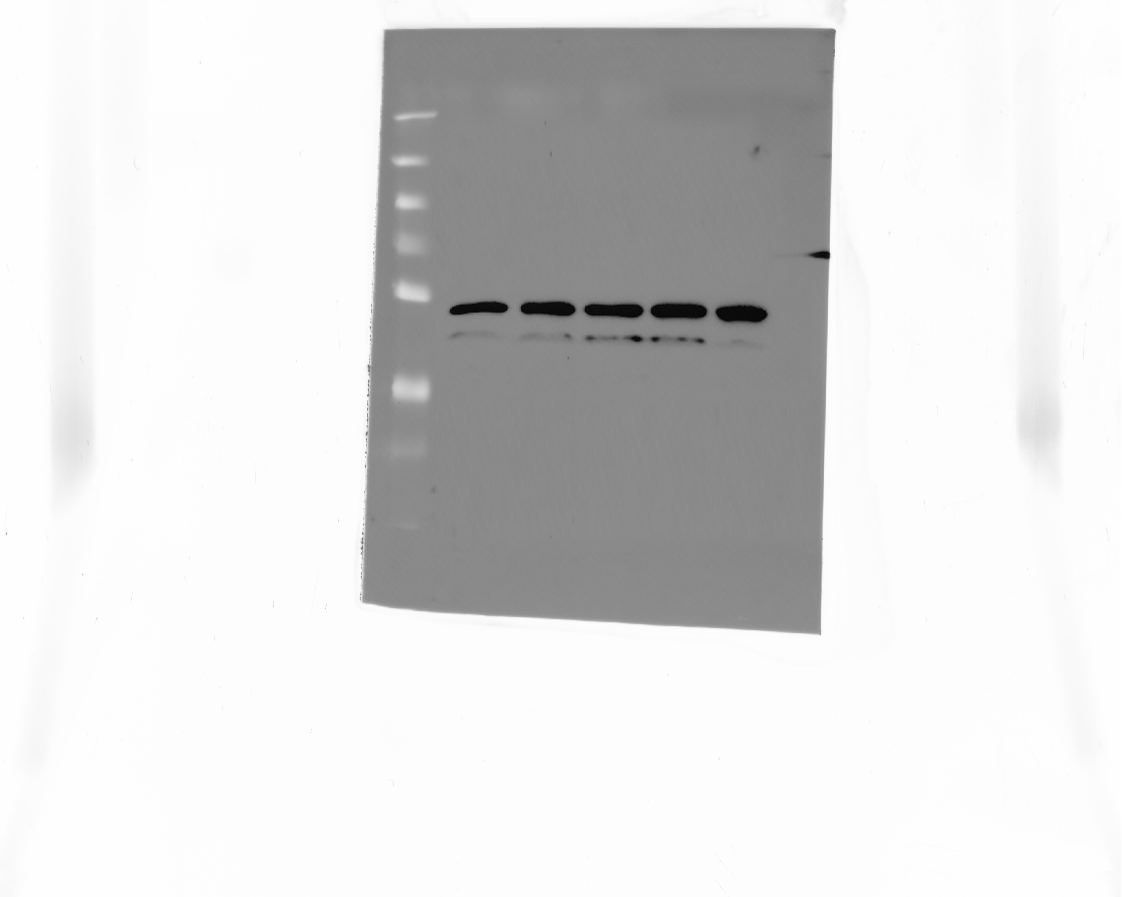

Supplement: Figure 3—source data 2. [file elife-106699-fig3-data2.zip › Figure 3ΓÇösource data 2 Original files for Western blot analysis displayed in Figure 3A./Lyl1 CCRF-CEM Abd-CRBN.tif]

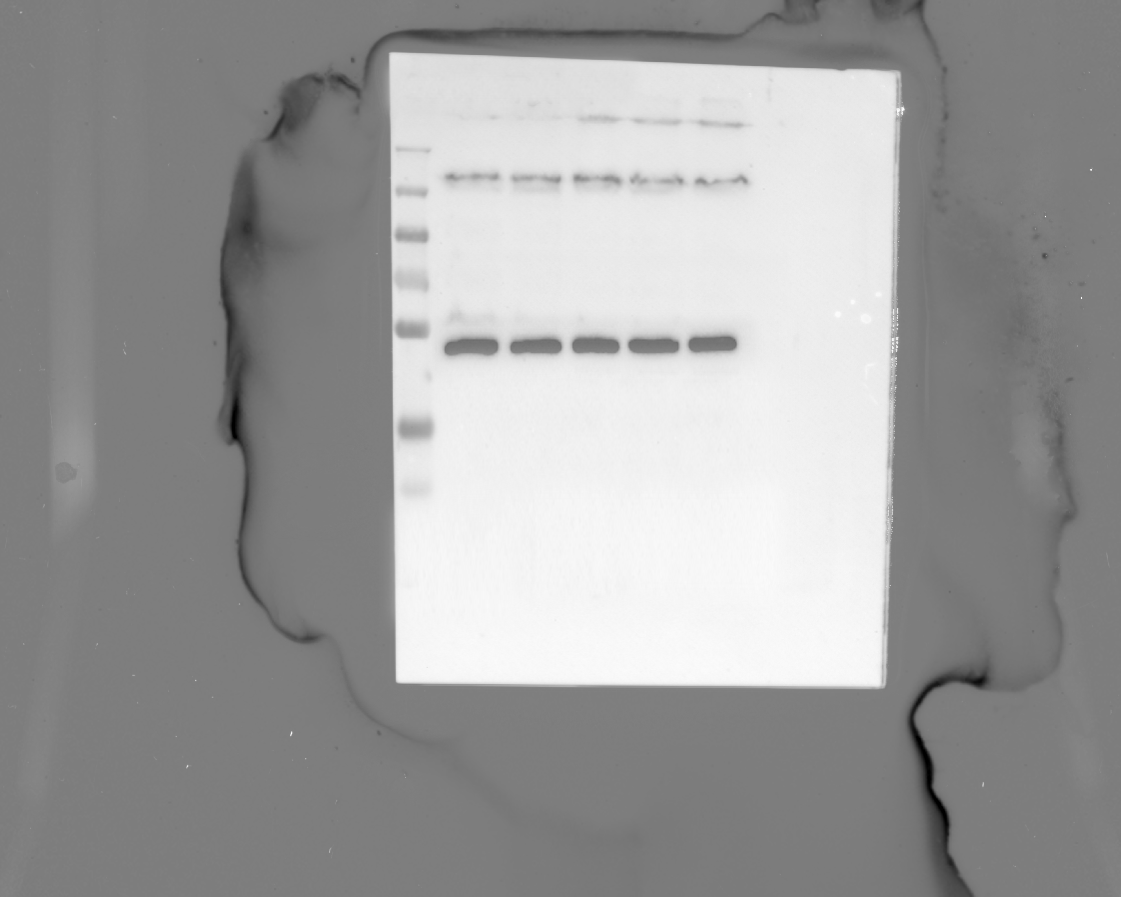

Supplement: Figure 3—source data 2. [file elife-106699-fig3-data2.zip › Figure 3ΓÇösource data 2 Original files for Western blot analysis displayed in Figure 3A./Lyl1- CCRF-CEM Abd-VHL.tif]

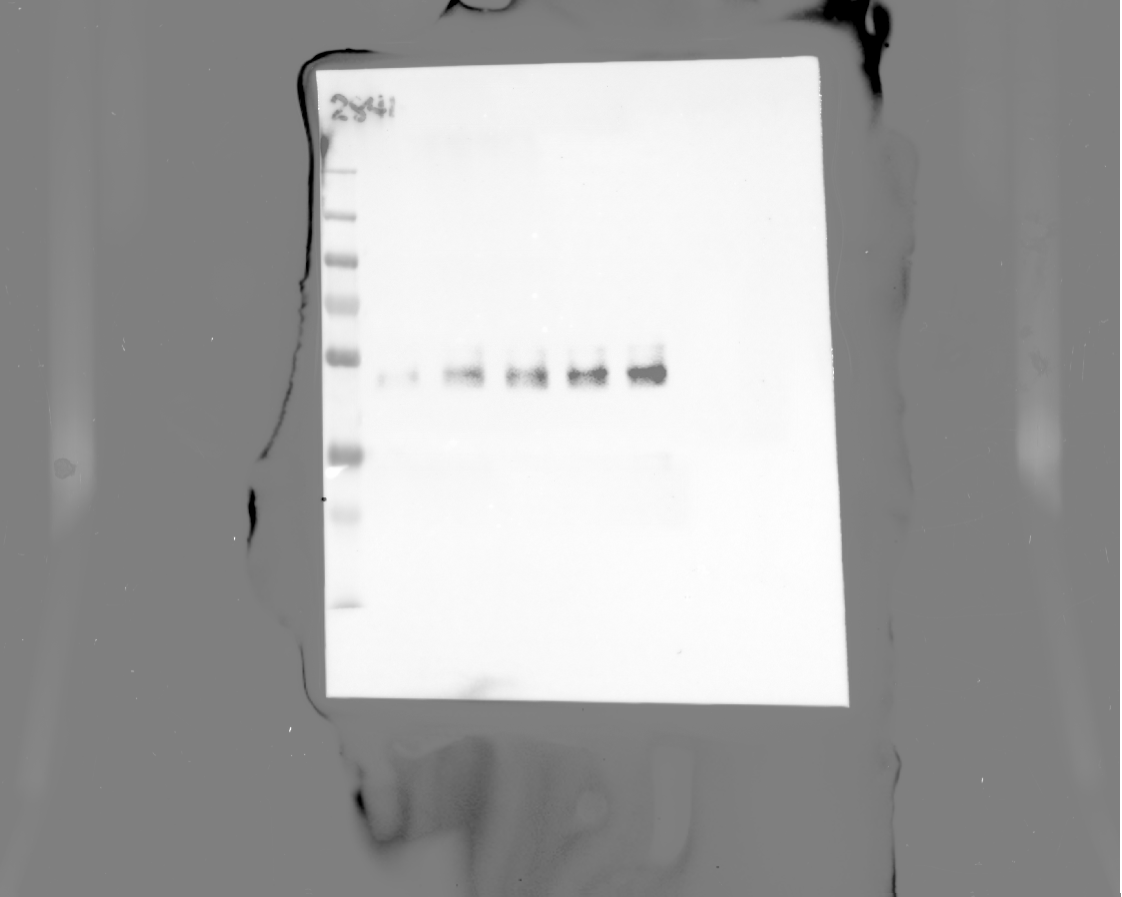

Supplement: Figure 3—source data 2. [file elife-106699-fig3-data2.zip › Figure 3ΓÇösource data 2 Original files for Western blot analysis displayed in Figure 3A./Tal-1 CCRF-CEM Abd-CRBN.tif]

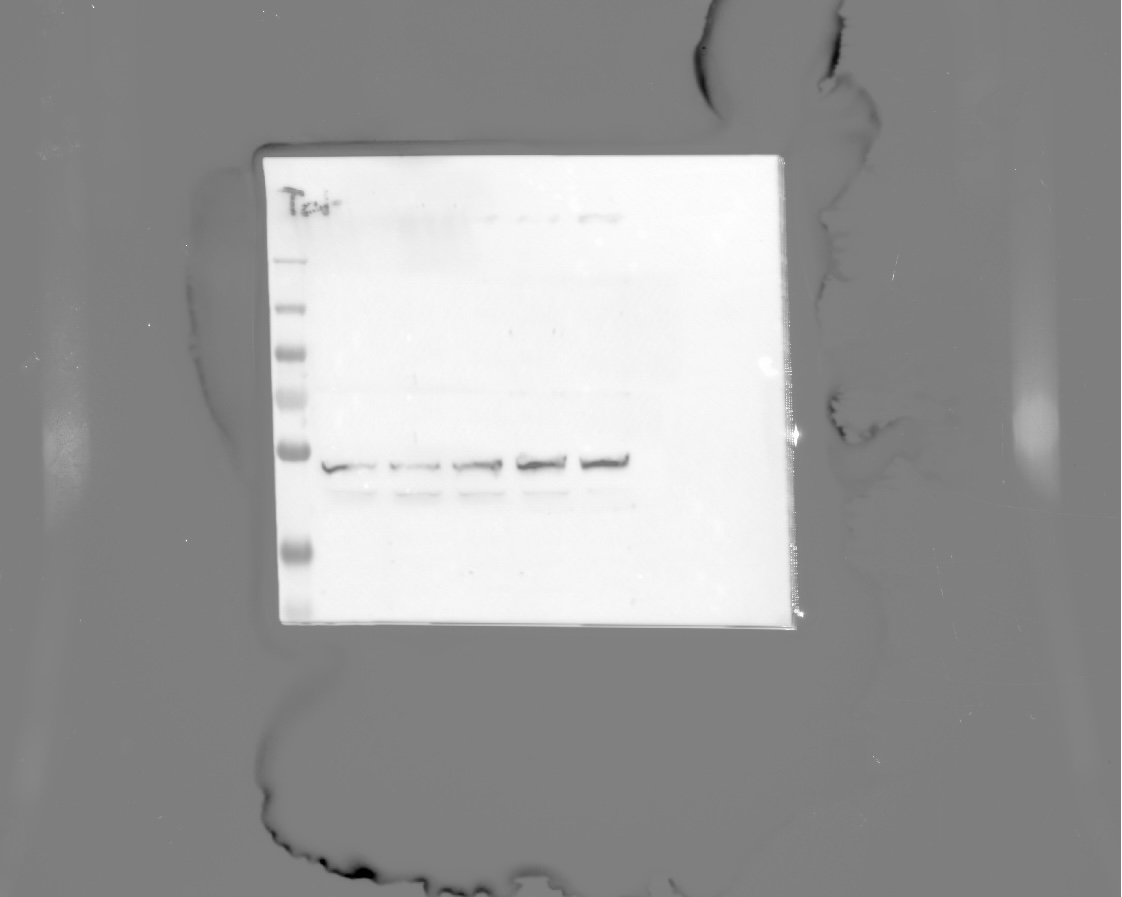

Supplement: Figure 3—source data 2. [file elife-106699-fig3-data2.zip › Figure 3ΓÇösource data 2 Original files for Western blot analysis displayed in Figure 3A./Tal-1 CCRF-CEM Abd-VHL.jpg]

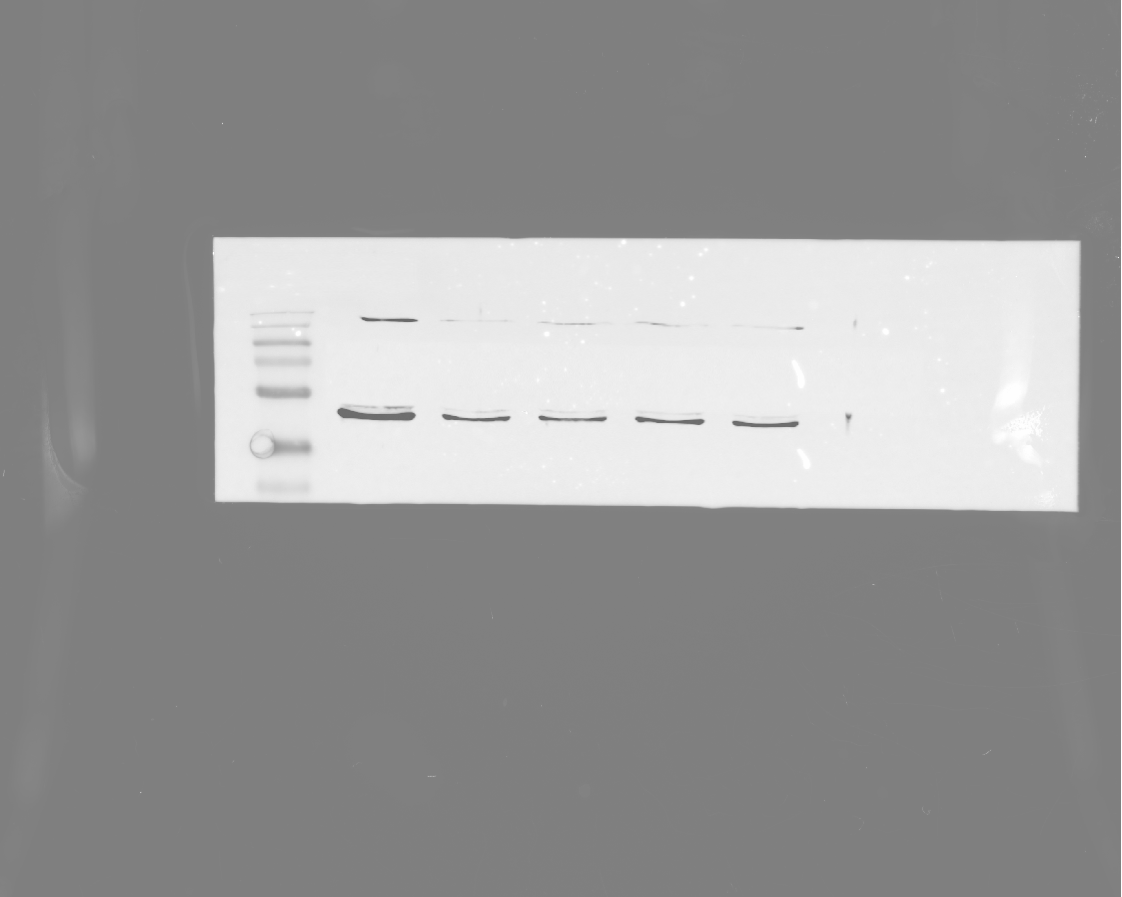

Supplement: Figure 3—source data 2. [file elife-106699-fig3-data2.zip › Figure 3ΓÇösource data 2 Original files for Western blot analysis displayed in Figure 3A./Tal-1 KOPT-K1 Abd-CRBN.tif]

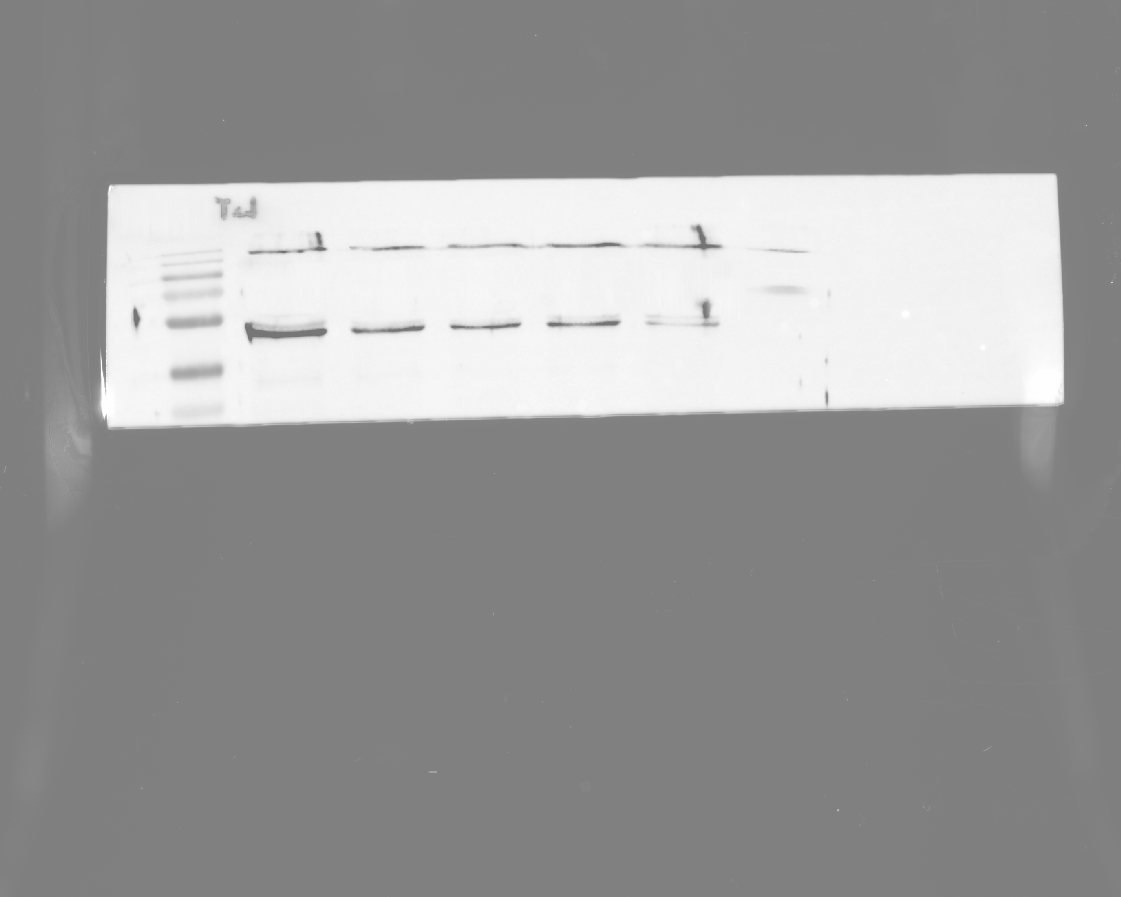

Supplement: Figure 3—source data 2. [file elife-106699-fig3-data2.zip › Figure 3ΓÇösource data 2 Original files for Western blot analysis displayed in Figure 3A./Tal1 KOPT-K1 Abd-VHL.tif]

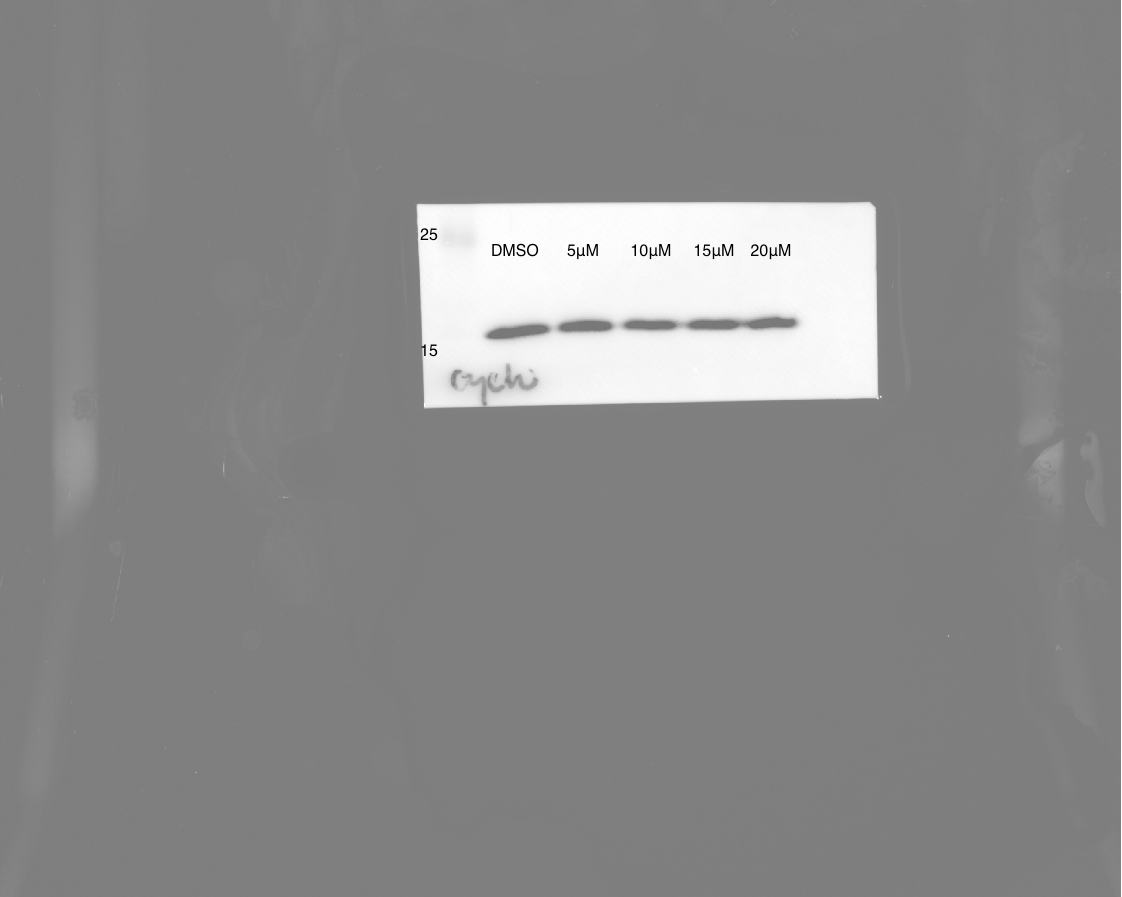

Supplement: Figure 3—source data 3. [file elife-106699-fig3-data3.zip › Figure 3ΓÇösource data 3 PDF files containing original western blots for Figure 3B, indicating the relevant bands and treatments./Raw data/Cyclophilin DND-41 Abd-CRBN.tif]

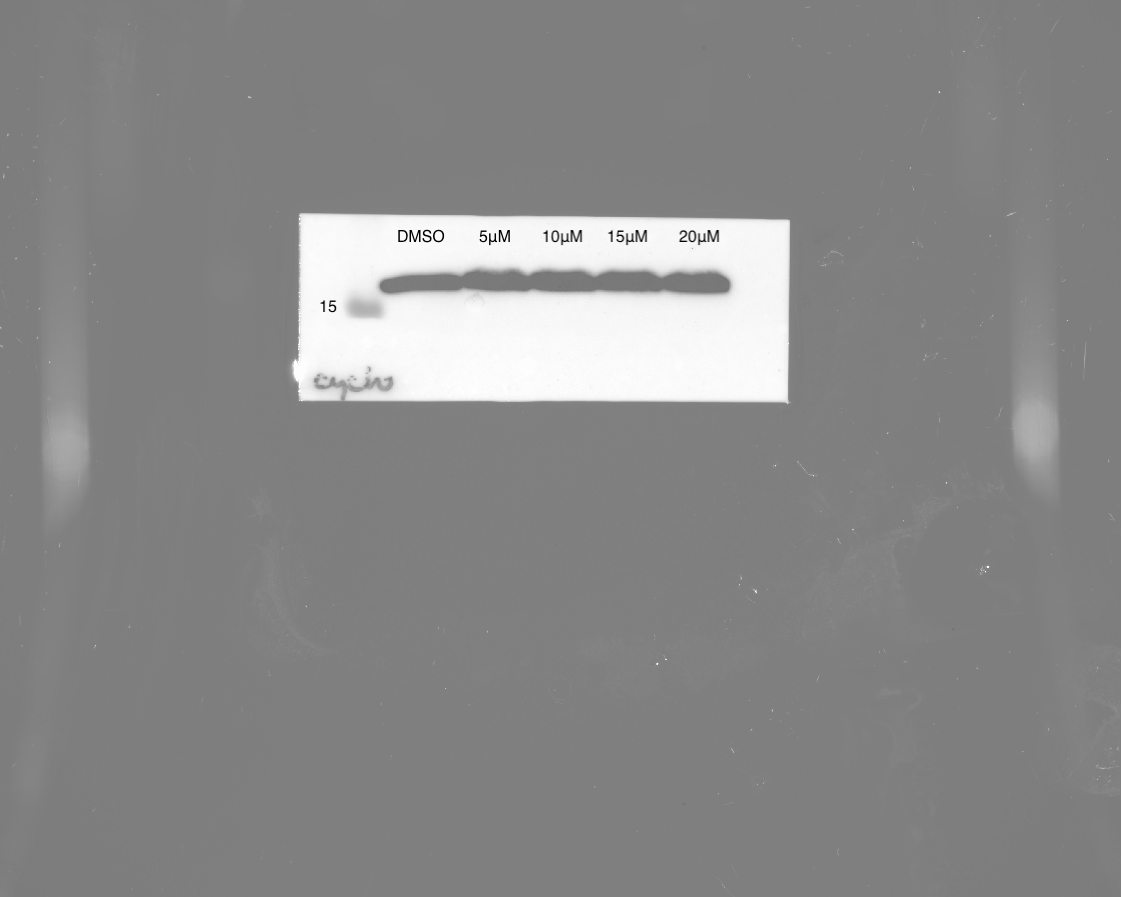

Supplement: Figure 3—source data 3. [file elife-106699-fig3-data3.zip › Figure 3ΓÇösource data 3 PDF files containing original western blots for Figure 3B, indicating the relevant bands and treatments./Raw data/Cyclophilin DND-41 Abd-VHL.tif]

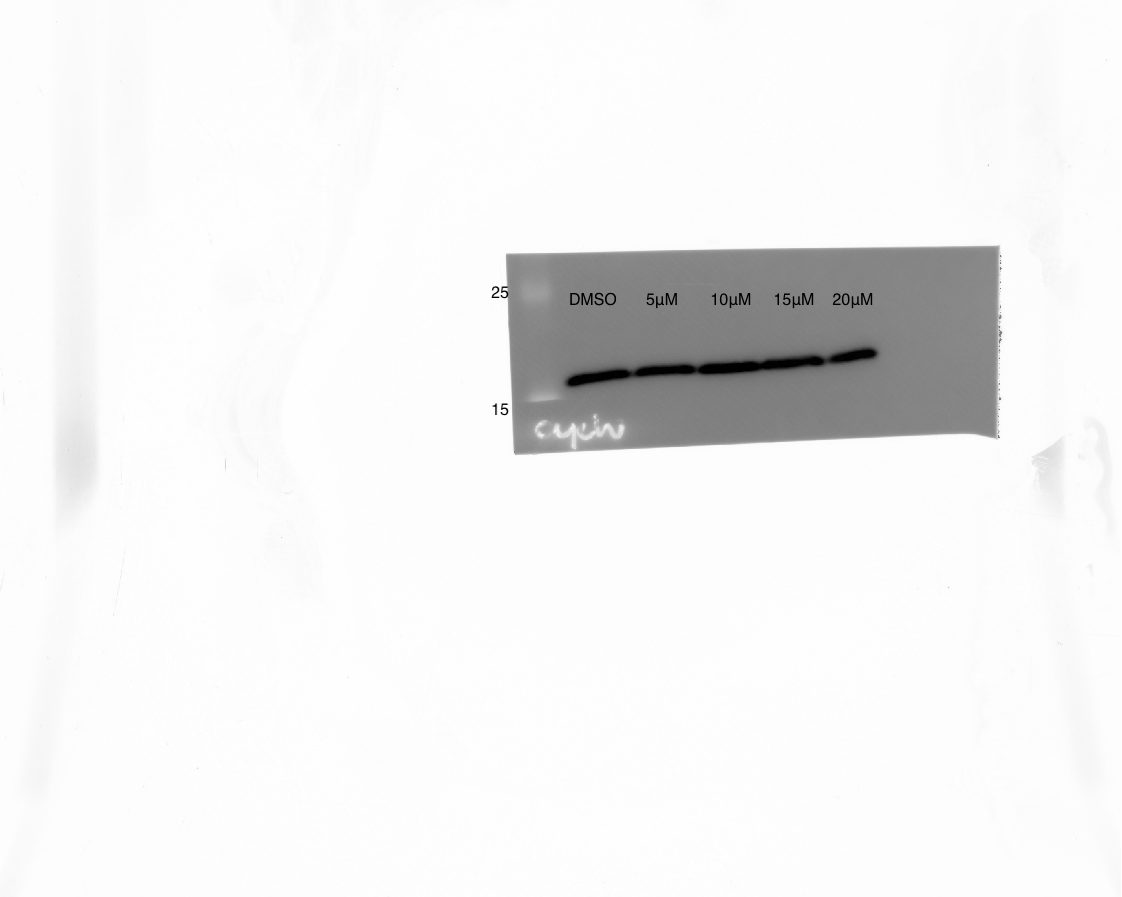

Supplement: Figure 3—source data 3. [file elife-106699-fig3-data3.zip › Figure 3ΓÇösource data 3 PDF files containing original western blots for Figure 3B, indicating the relevant bands and treatments./Raw data/Cyclophilin Jurkat Abd-CRBN.tif]

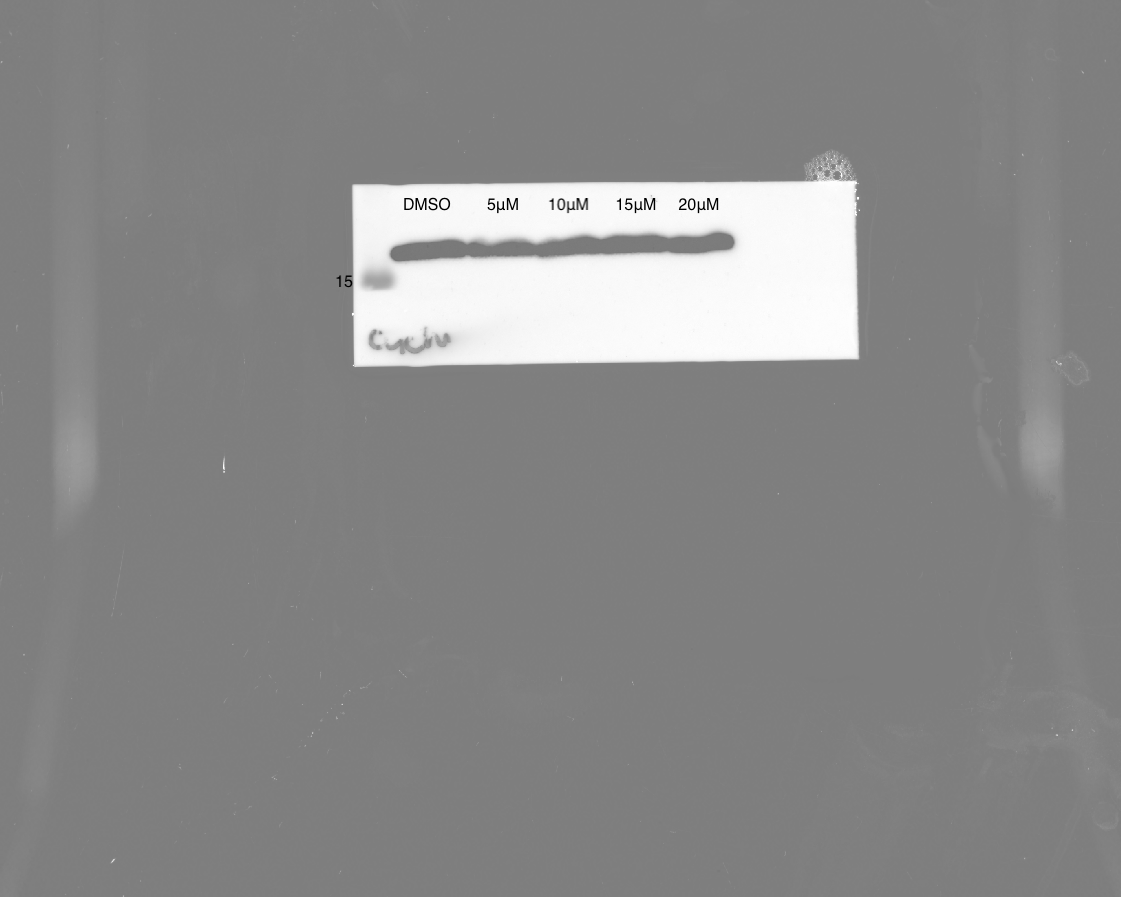

Supplement: Figure 3—source data 3. [file elife-106699-fig3-data3.zip › Figure 3ΓÇösource data 3 PDF files containing original western blots for Figure 3B, indicating the relevant bands and treatments./Raw data/Cyclophilin Jurkat Abd-VHL.tif]

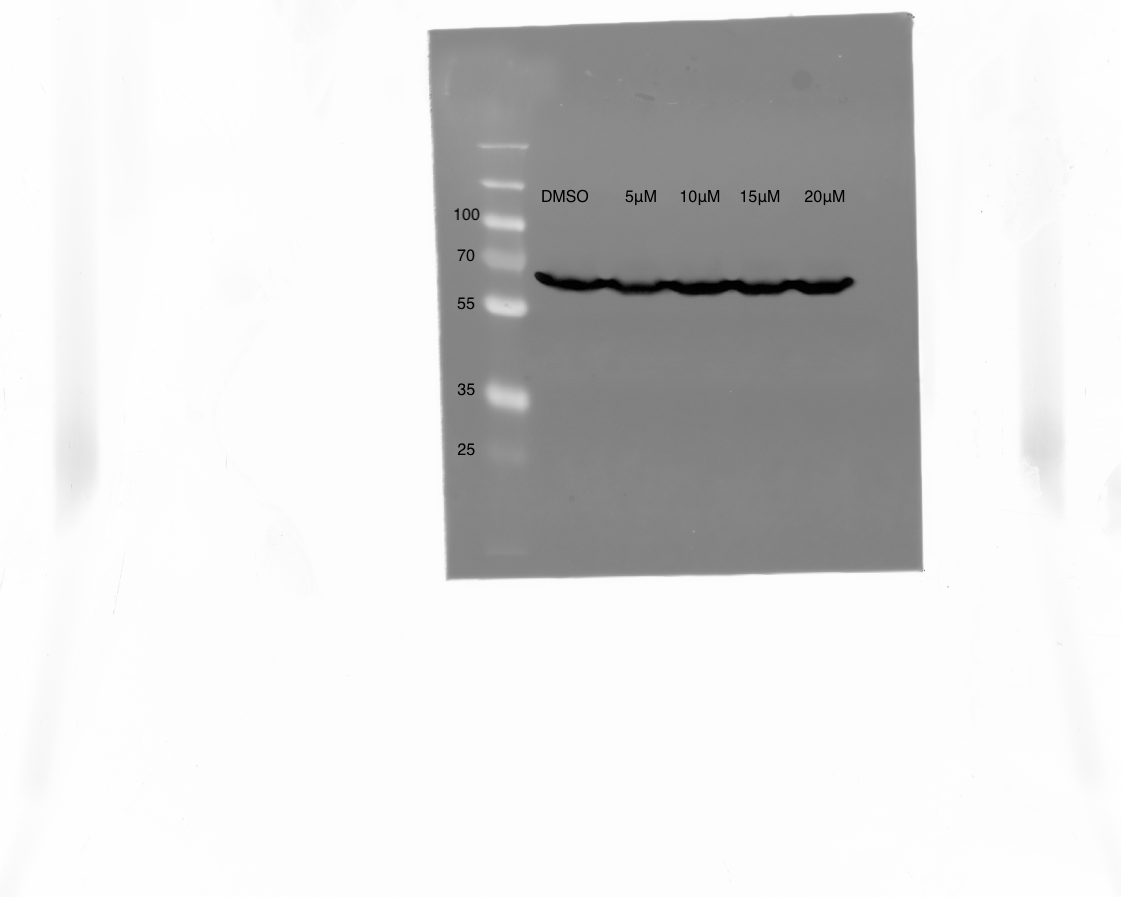

Supplement: Figure 3—source data 3. [file elife-106699-fig3-data3.zip › Figure 3ΓÇösource data 3 PDF files containing original western blots for Figure 3B, indicating the relevant bands and treatments./Raw data/E2A DND-41 Abd-CRBN.tif]

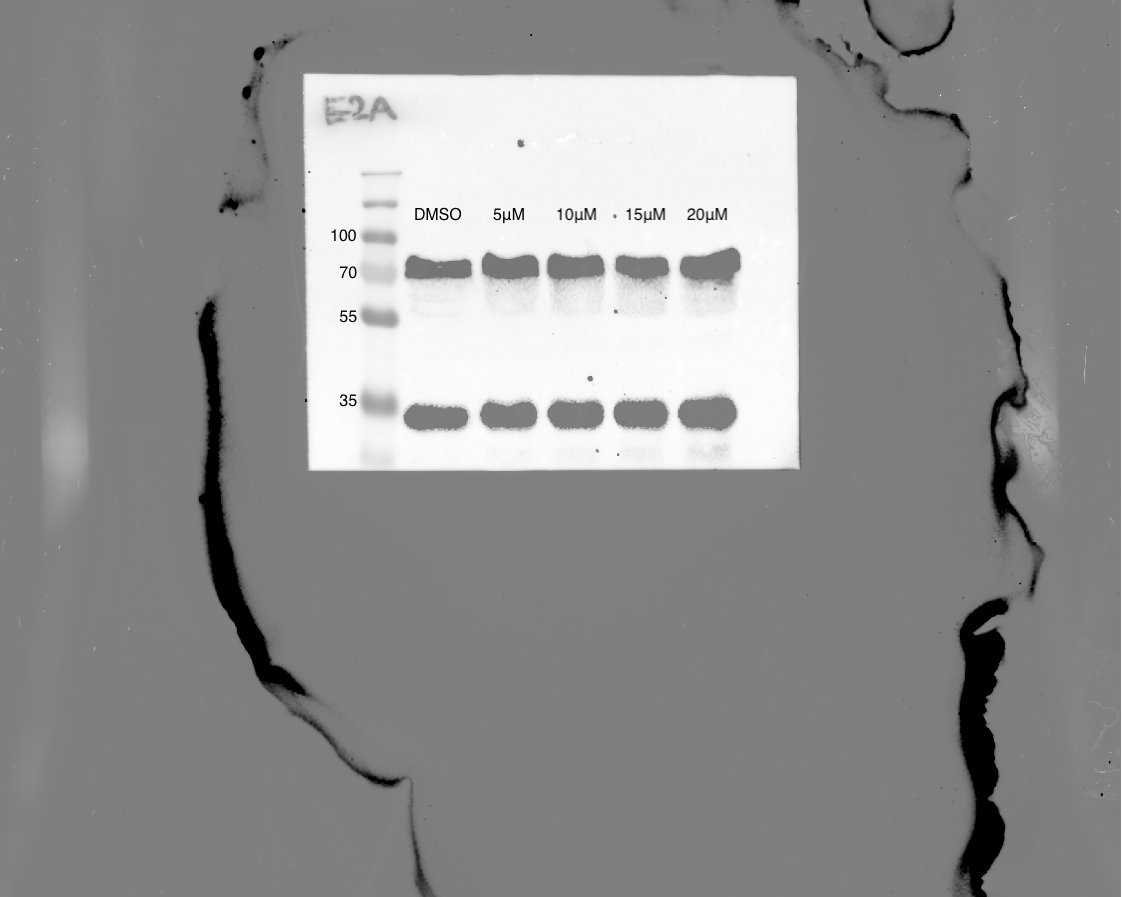

Supplement: Figure 3—source data 3. [file elife-106699-fig3-data3.zip › Figure 3ΓÇösource data 3 PDF files containing original western blots for Figure 3B, indicating the relevant bands and treatments./Raw data/E2A DND-41 Abd-VHL.tif]

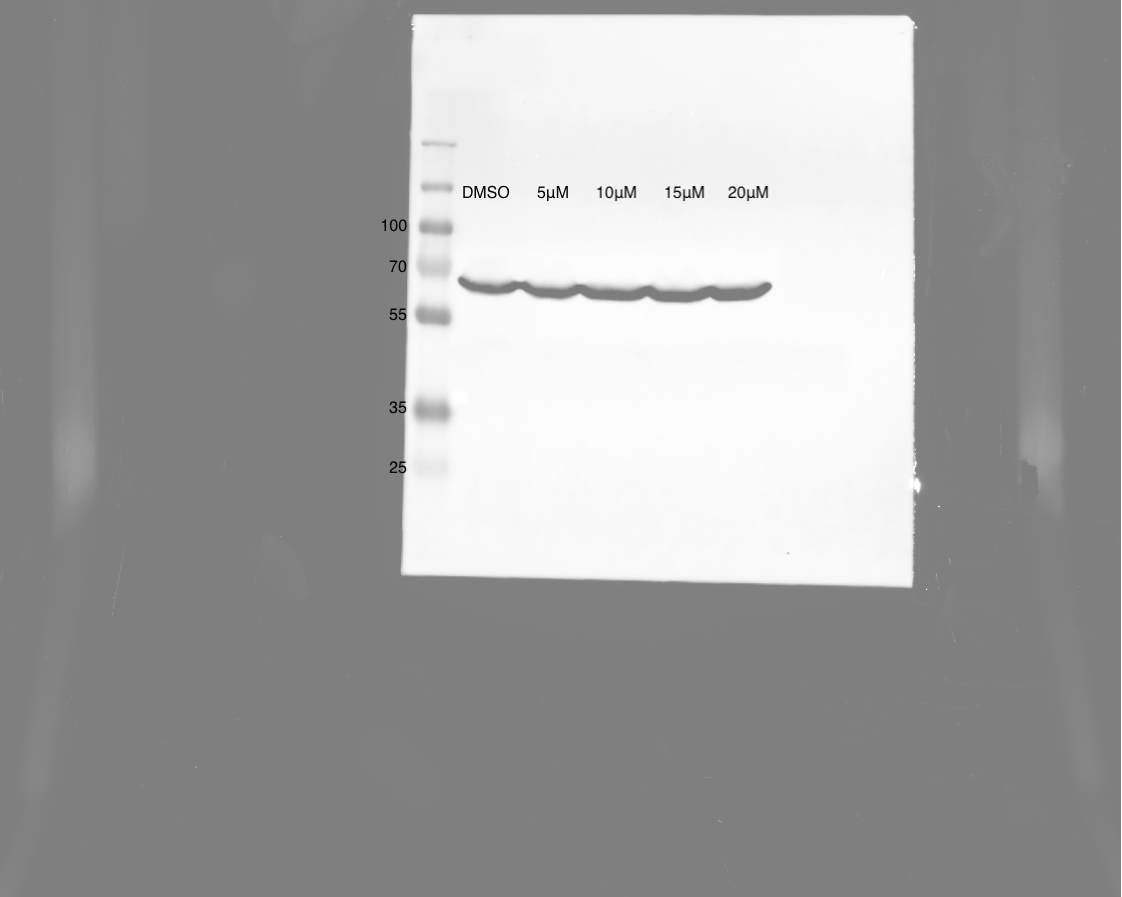

Supplement: Figure 3—source data 3. [file elife-106699-fig3-data3.zip › Figure 3ΓÇösource data 3 PDF files containing original western blots for Figure 3B, indicating the relevant bands and treatments./Raw data/E2A Jurkat Abd-CRBN.tif]

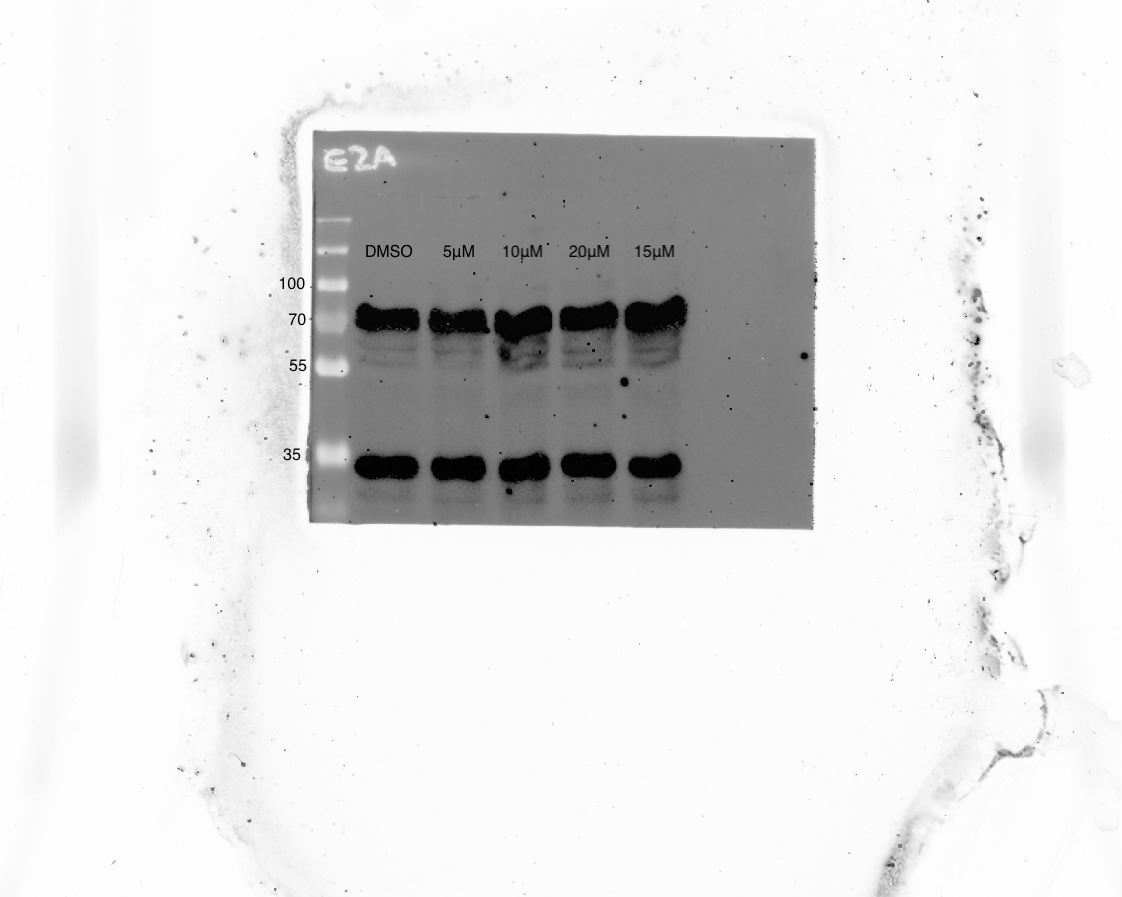

Supplement: Figure 3—source data 3. [file elife-106699-fig3-data3.zip › Figure 3ΓÇösource data 3 PDF files containing original western blots for Figure 3B, indicating the relevant bands and treatments./Raw data/E2A Jurkat Abd-VHL.tif]

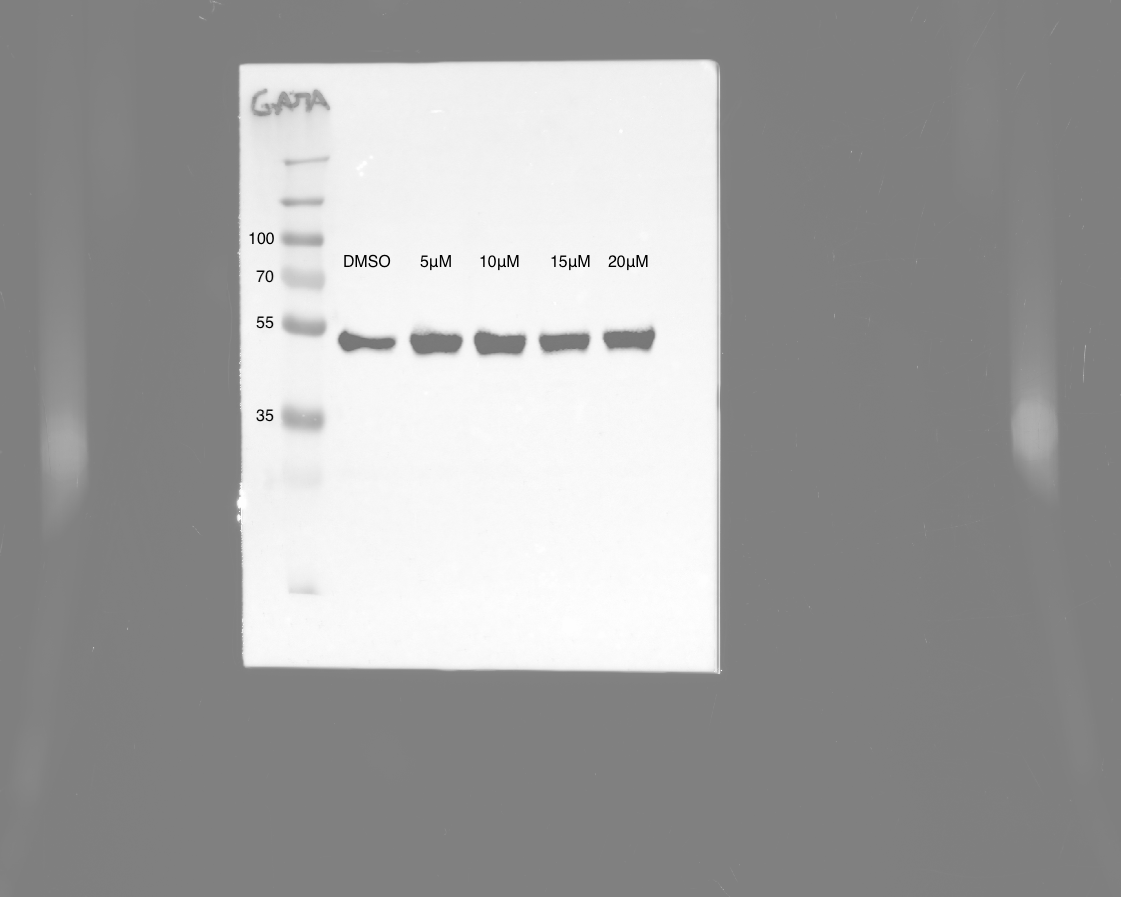

Supplement: Figure 3—source data 3. [file elife-106699-fig3-data3.zip › Figure 3ΓÇösource data 3 PDF files containing original western blots for Figure 3B, indicating the relevant bands and treatments./Raw data/GATA3 DND-41 Abd-VHL.tif]

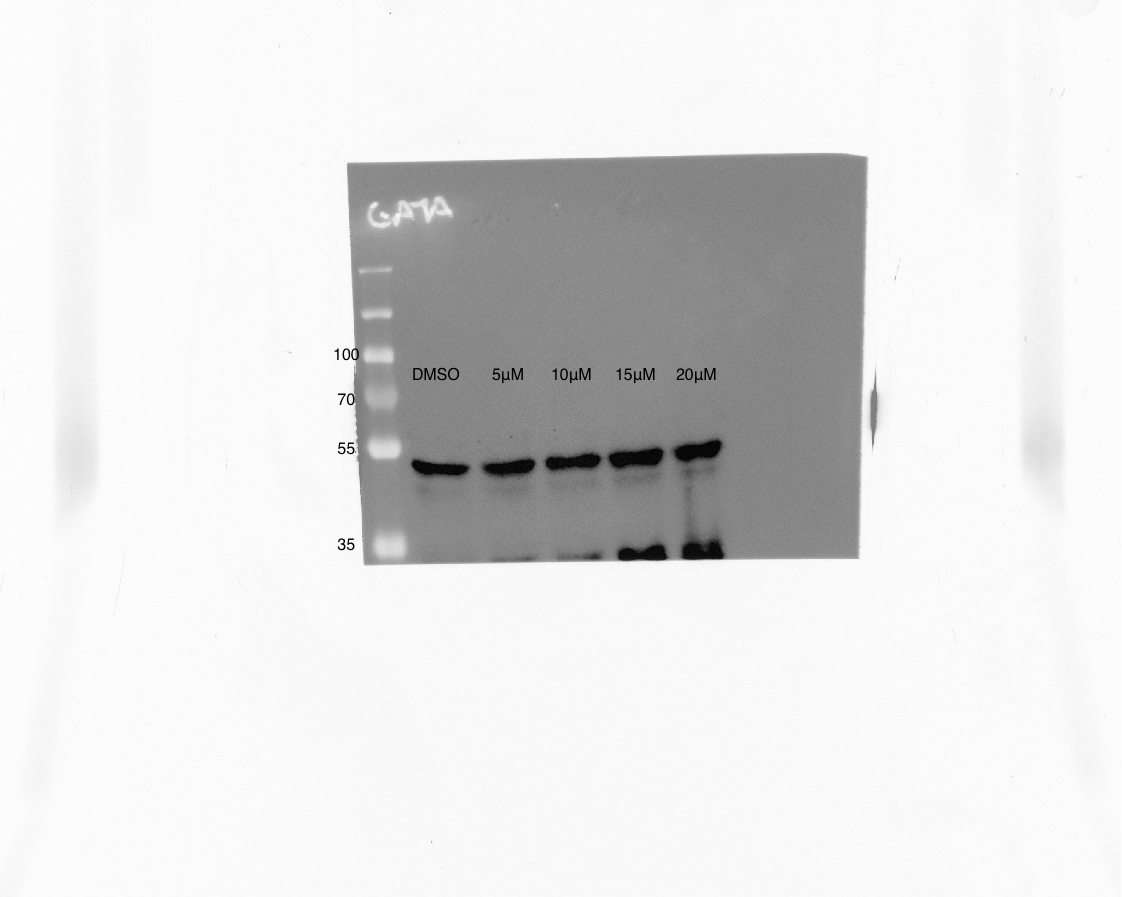

Supplement: Figure 3—source data 3. [file elife-106699-fig3-data3.zip › Figure 3ΓÇösource data 3 PDF files containing original western blots for Figure 3B, indicating the relevant bands and treatments./Raw data/GATA3 Jurkat Abd-CRBN.tif]

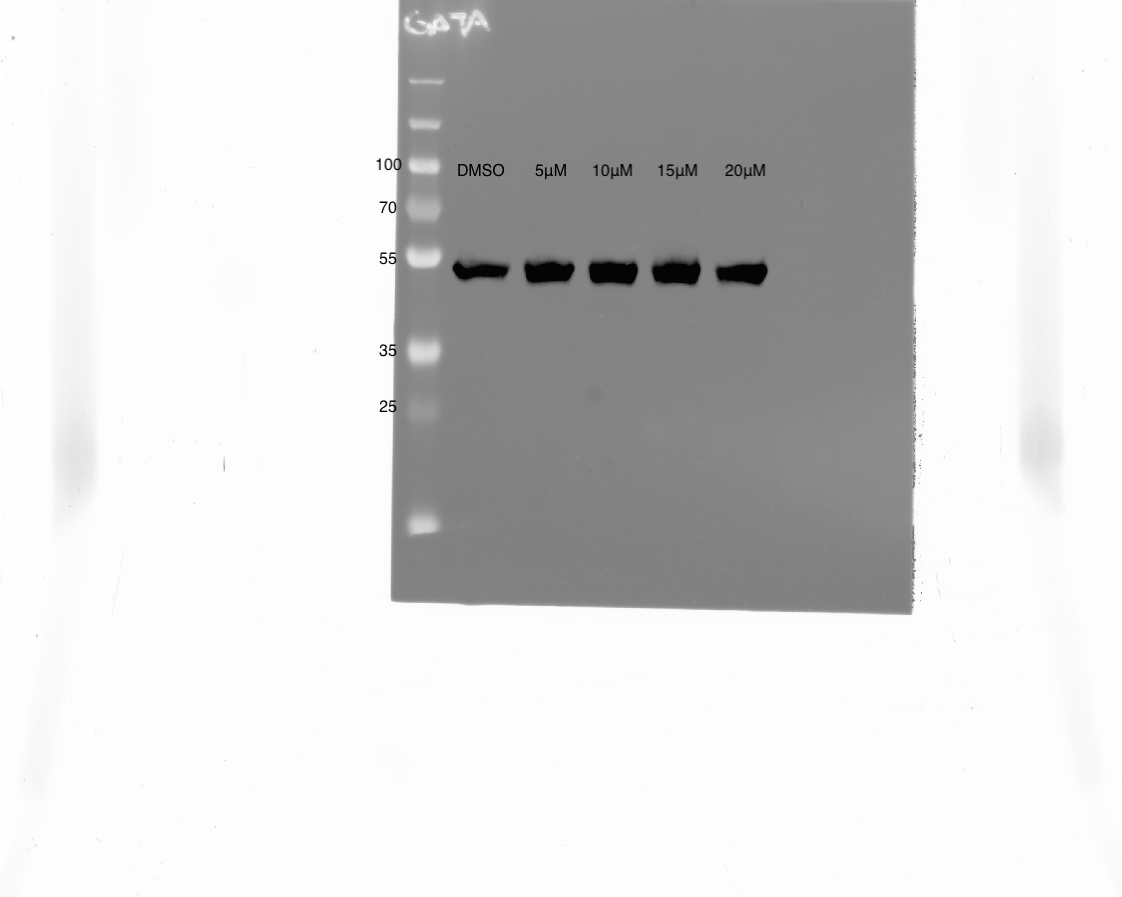

Supplement: Figure 3—source data 3. [file elife-106699-fig3-data3.zip › Figure 3ΓÇösource data 3 PDF files containing original western blots for Figure 3B, indicating the relevant bands and treatments./Raw data/GATA3 Jurkat Abd-VHL.tif]

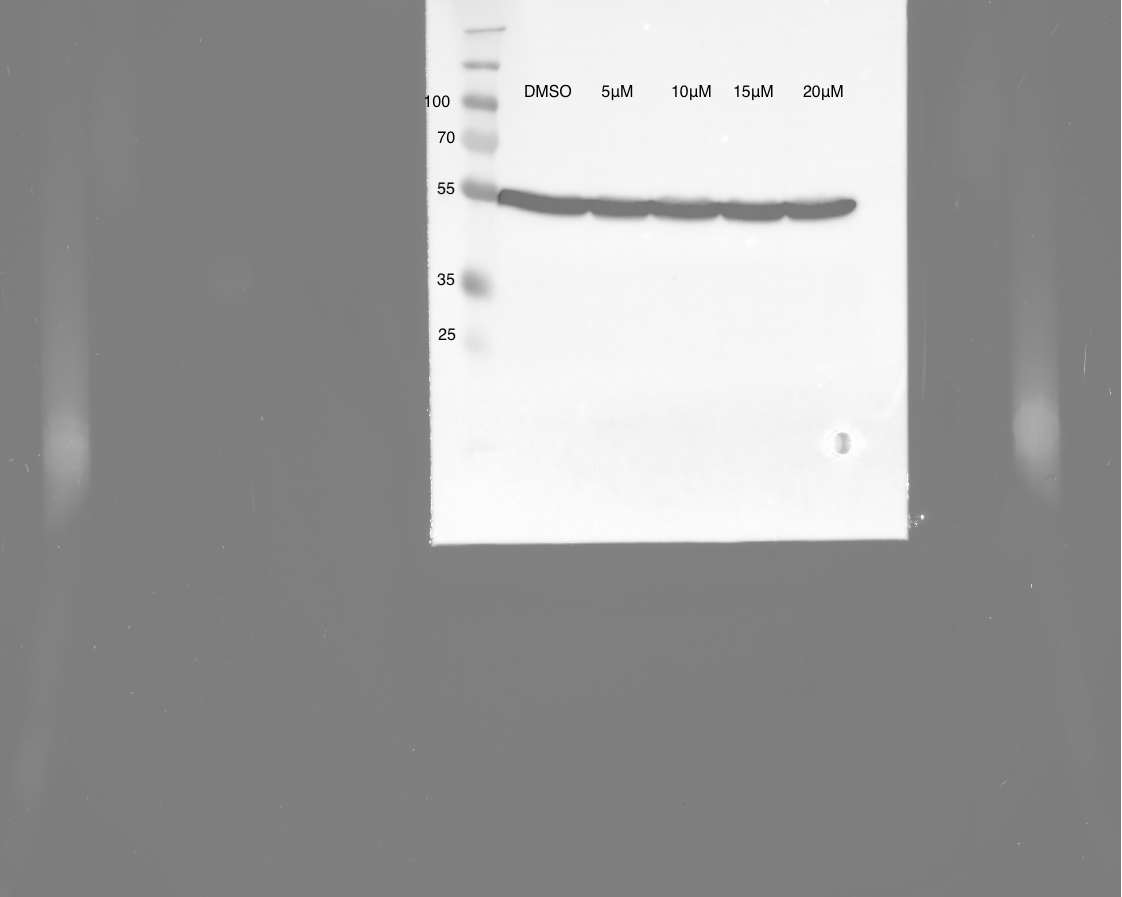

Supplement: Figure 3—source data 3. [file elife-106699-fig3-data3.zip › Figure 3ΓÇösource data 3 PDF files containing original western blots for Figure 3B, indicating the relevant bands and treatments./Raw data/LDB1 DND-41 Abd-CRBN.tif]

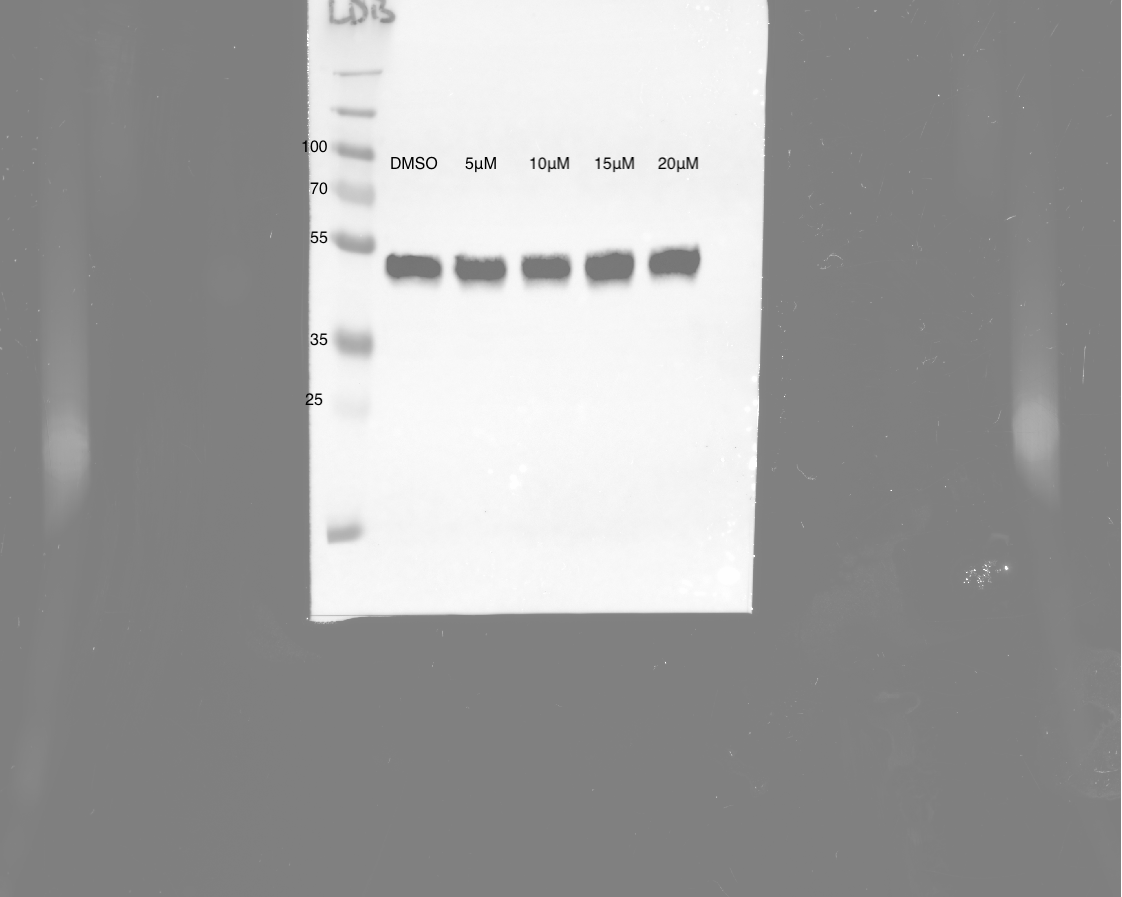

Supplement: Figure 3—source data 3. [file elife-106699-fig3-data3.zip › Figure 3ΓÇösource data 3 PDF files containing original western blots for Figure 3B, indicating the relevant bands and treatments./Raw data/LDB1 DND-41 Abd-VHL.tif]

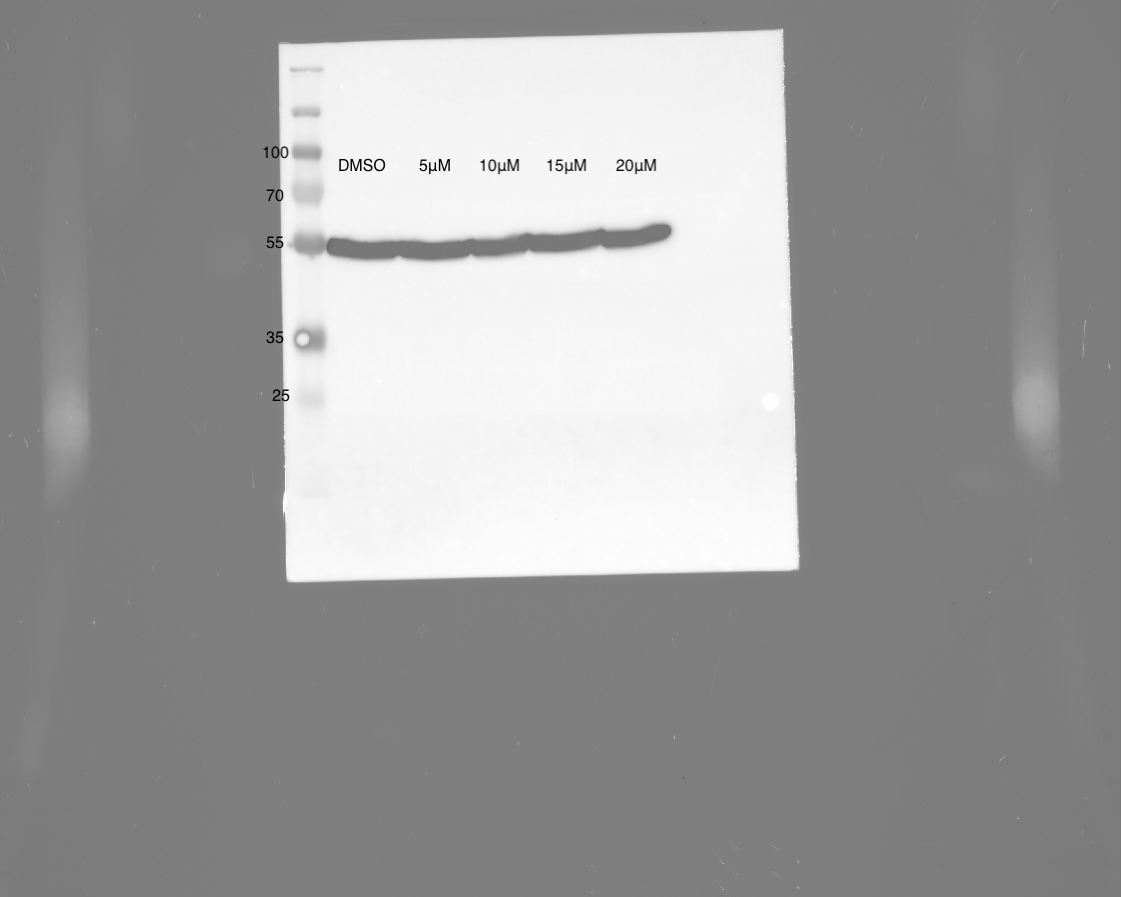

Supplement: Figure 3—source data 3. [file elife-106699-fig3-data3.zip › Figure 3ΓÇösource data 3 PDF files containing original western blots for Figure 3B, indicating the relevant bands and treatments./Raw data/LDB1 Jurkat Abd-CRBN.tif]
