# Supplementary material for: Degradation of LMO2 in T cell leukaemia results in collateral breakdown of transcription complex partners and causes LMO2-dependent apoptosis
Source: eLife. 2025 Dec 12;14:RP106699. doi: 10.7554/eLife.106699 (PMC12700530; doi:10.7554/eLife.106699)
Supplement: Figure 2—figure supplement 5—source data 1. [file elife-106699-fig2-figsupp5-data1.zip › Figure 2ΓÇöfigure supplement 5-source data 1 Western blot data with label shows the longevity of responses of KOPT-K1 cells to single dose treatment./Figure 1-figure supplement 5-source data 1.pdf]

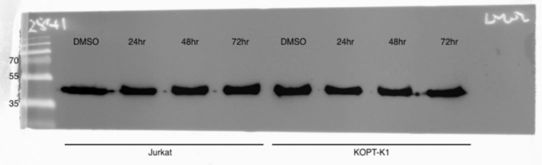

Anti-Actin
Jurkat and KOPT-K1 treated with Abd-CRBN

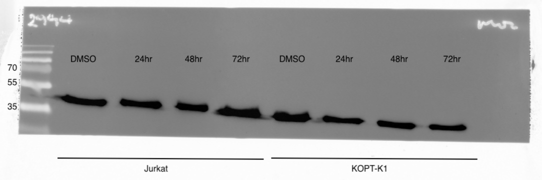

Anti-Actin
Jurkat and KOPT-K1 treated with Abd-VHL

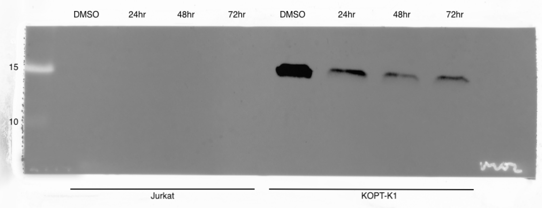

Anti-LMO2
Jurkat and KOPT-K1 treated with Abd-CRBN

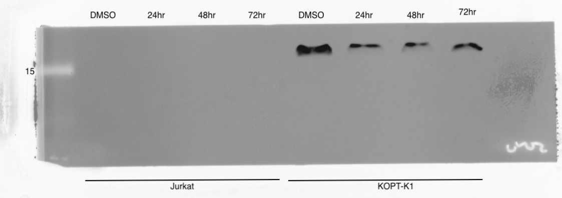

Anti-LMO2
Jurkat and KOPT-K1 treated with Abd-VHL

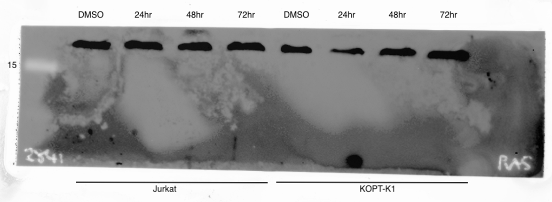

Anti-panRas
Jurkat and KOPT-K1 treated with Abd-CRBN

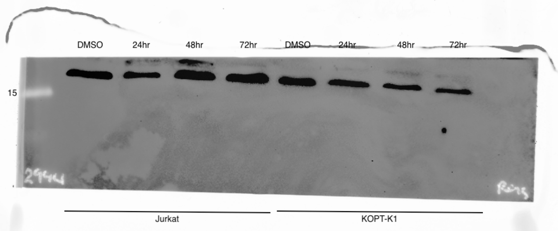

Anti-panRas
Jurkat and KOPT-K1 treated with Abd-VHL

Figure 2-figure supplement 5, Source Data 1. Original membranes corresponding to Figure 2-figure supplement 5, panel A-B.
