# Supplementary material for: Degradation of LMO2 in T cell leukaemia results in collateral breakdown of transcription complex partners and causes LMO2-dependent apoptosis
Source: eLife. 2025 Dec 12;14:RP106699. doi: 10.7554/eLife.106699 (PMC12700530; doi:10.7554/eLife.106699)
Supplement: Figure 3—source data 3. [file elife-106699-fig3-data3.zip › Figure 3ΓÇösource data 3 PDF files containing original western blots for Figure 3B, indicating the relevant bands and treatments./Figure 3-source data 3.pdf]

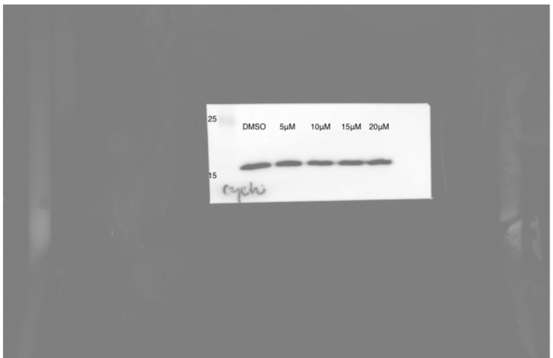

Cyclophilin-b in DND-41
treated with Abd-CRBN

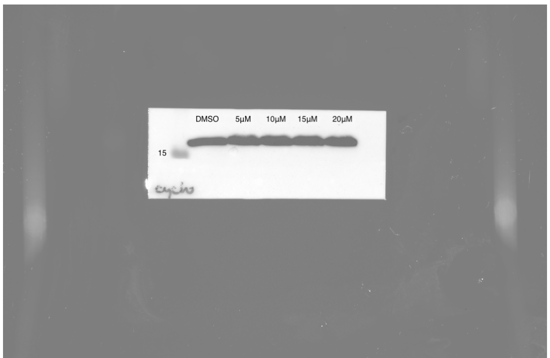

Cyclophilin-b in DND-41
treated with Abd-VHL

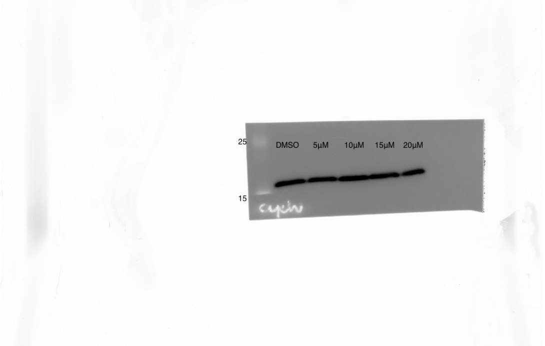

Cyclophilin-b in Jurkat
treated with Abd-CRBN

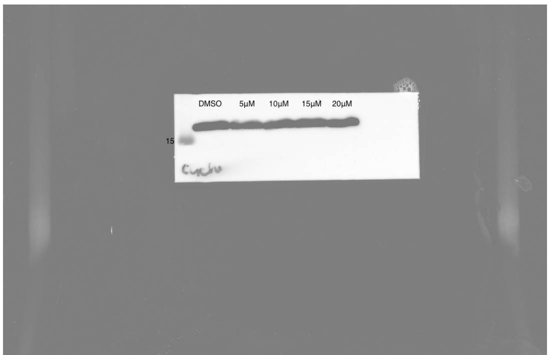

Cyclophilin-b in Jurkat
treated with Abd-VHL

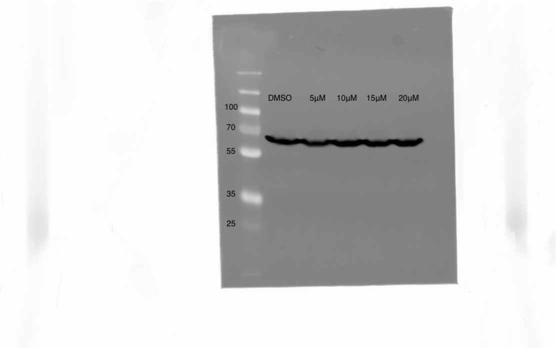

E47 in DND-41
treated with Abd-CRBN

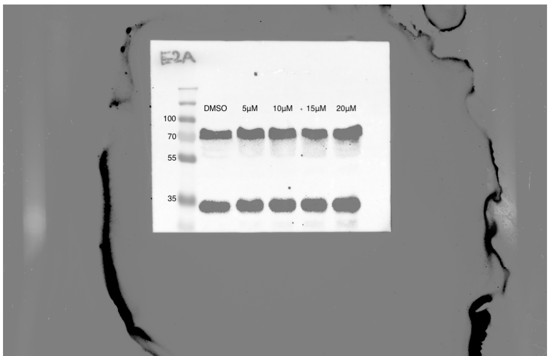

E47 in DND-41
treated with Abd-VHL

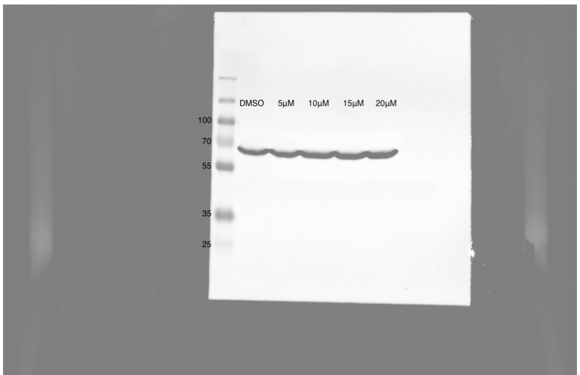

E47 in Jurkat
treated with Abd-CRBN

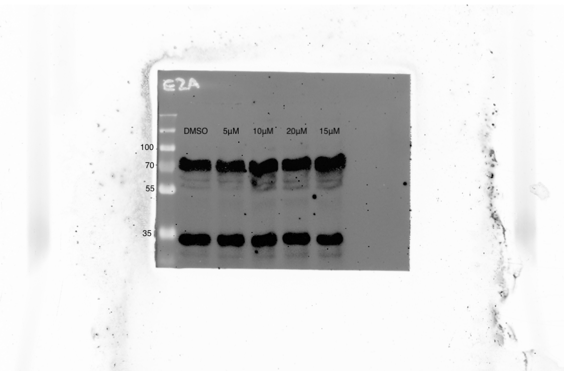

E47 in Jurkat
treated with Abd-VHL

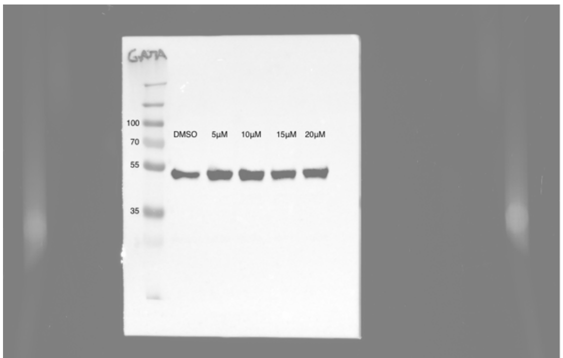

GATA3 in DND-41
treated with Abd-VHL

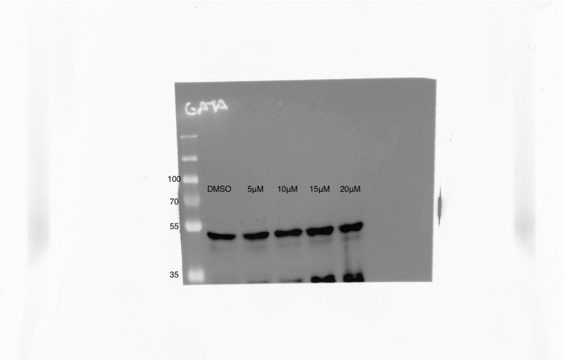

GATA3 in Jurkat
treated with Abd-CRBN

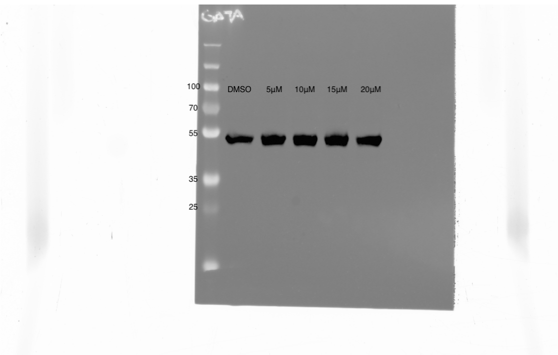

GATA3 in Jurkat
treated with Abd-VHL

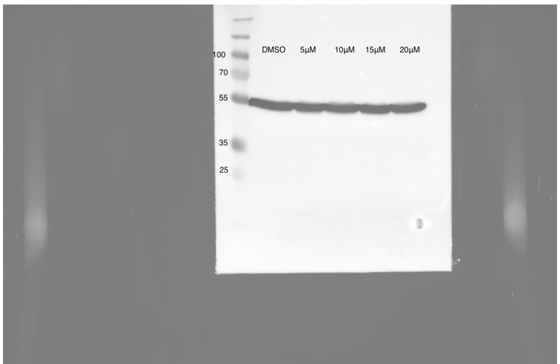

LDB1 in DND-41
treated with Abd-CRBN

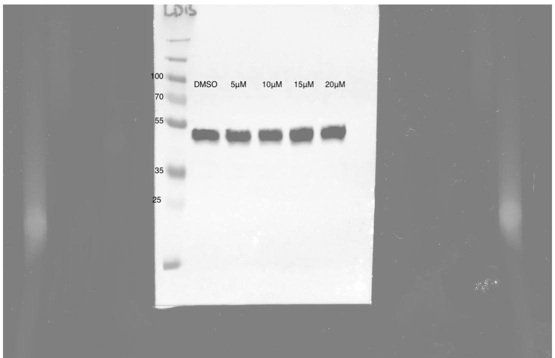

LDB1 in DND-41
treated with Abd-VHL

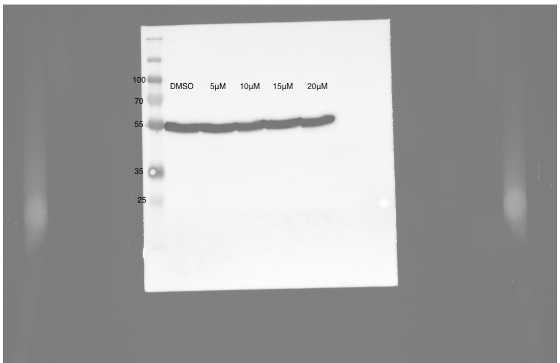

LDB1 in Jurkat
treated with Abd-CRBN

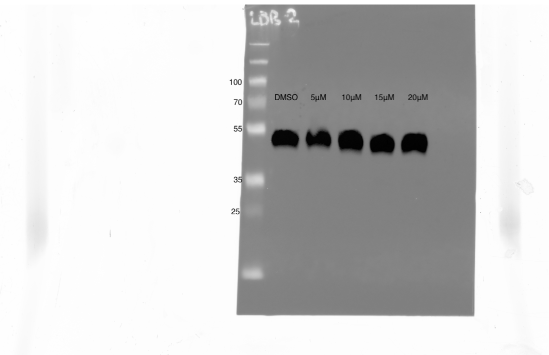

LDB1 in Jurkat
treated with Abd-VHL

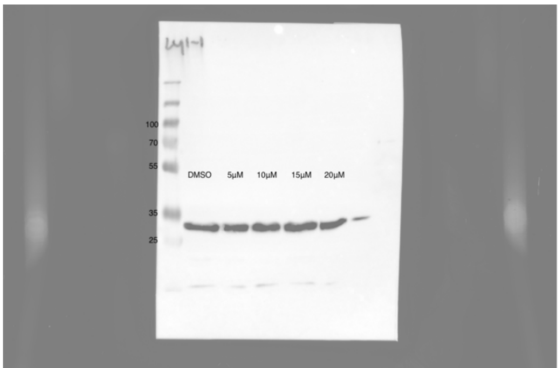

Lyl-1 in DND-41
treated with Abd-CRBN

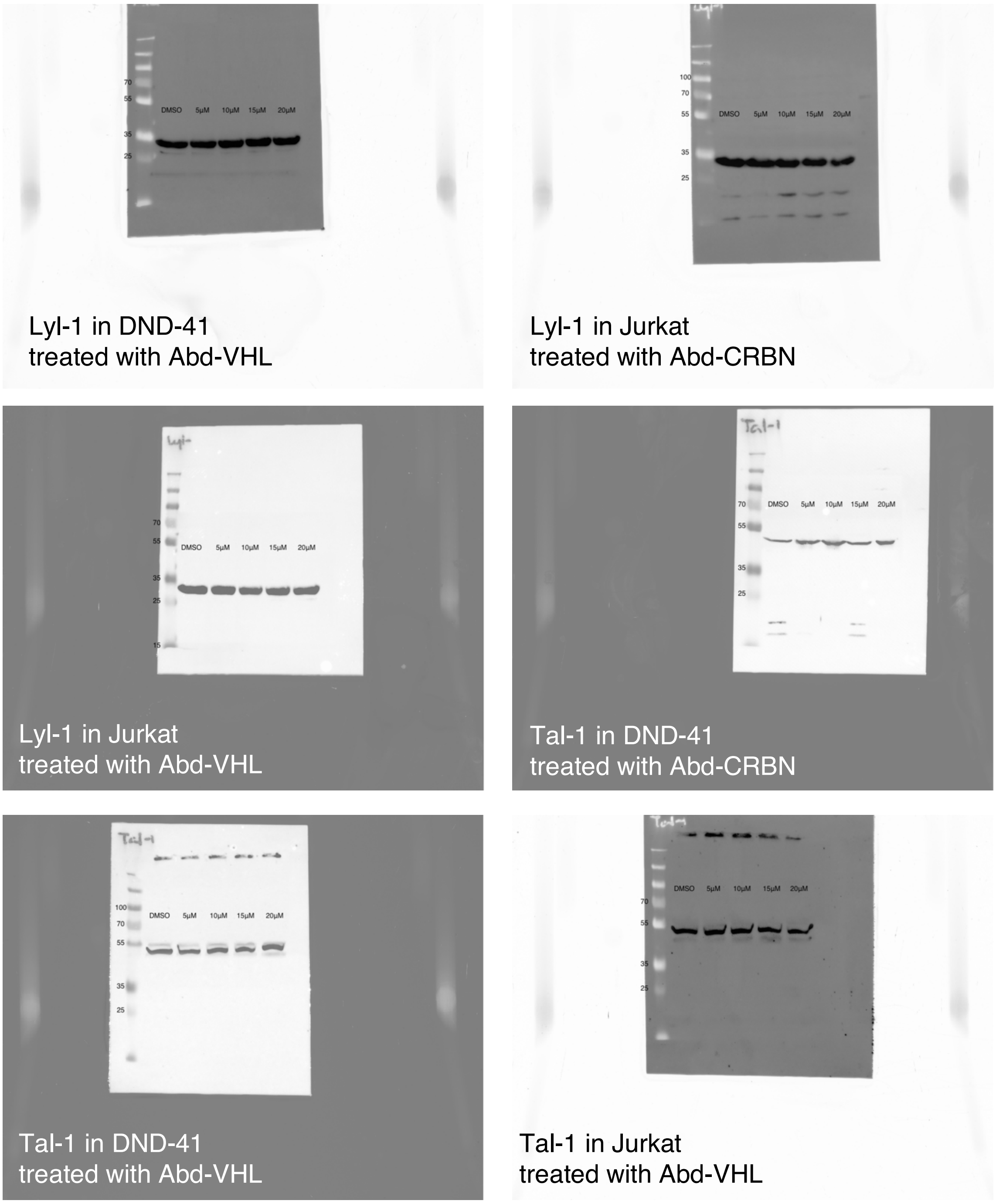

Figure 3, Source Data 3. Original membranes corresponding to Figure 3, panel B.
