# Supplementary figures and images for: Degradation of LMO2 in T cell leukaemia results in collateral breakdown of transcription complex partners and causes LMO2-dependent apoptosis (part 3 of 5)
Source: eLife. 2025 Dec 12;14:RP106699. doi: 10.7554/eLife.106699 (PMC12700530; doi:10.7554/eLife.106699)

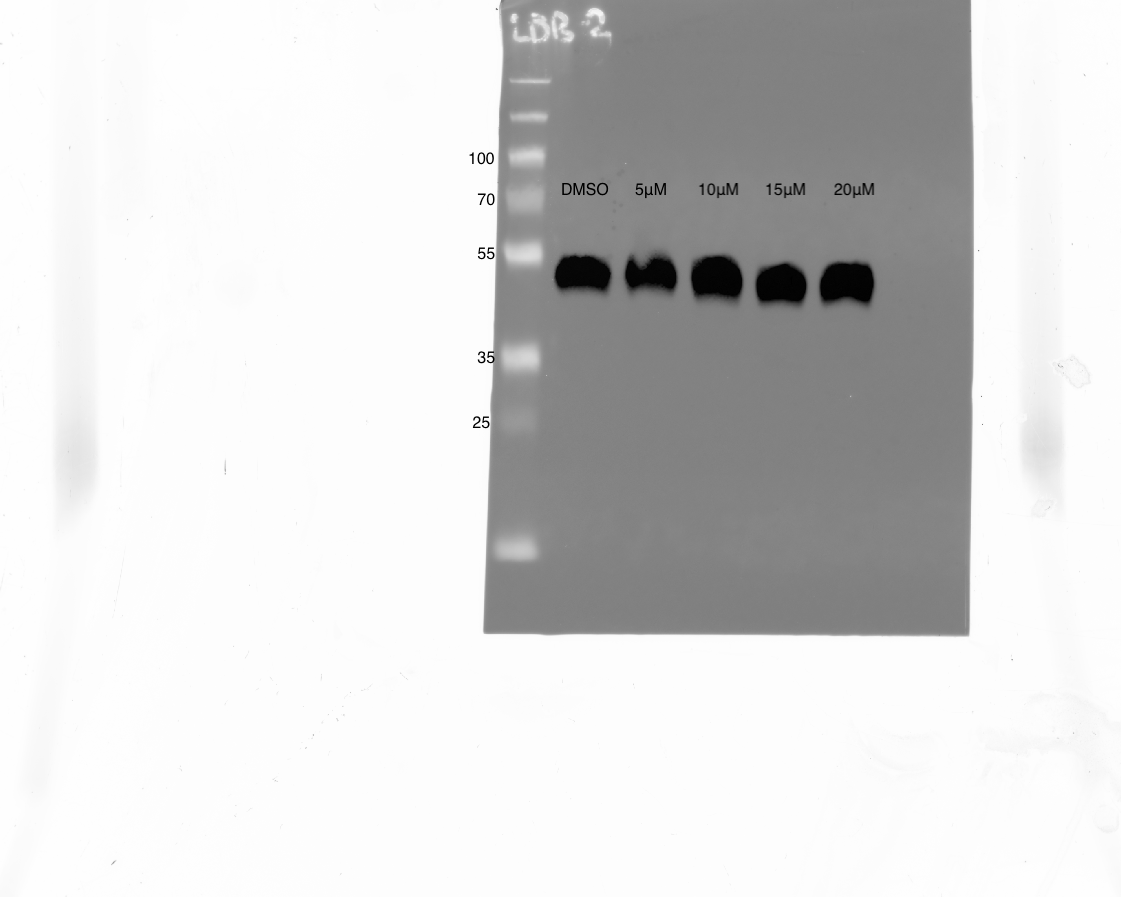

Supplement: Figure 3—source data 3. [file elife-106699-fig3-data3.zip › Figure 3ΓÇösource data 3 PDF files containing original western blots for Figure 3B, indicating the relevant bands and treatments./Raw data/LDB1 Jurkat Abd-VHL.tif]

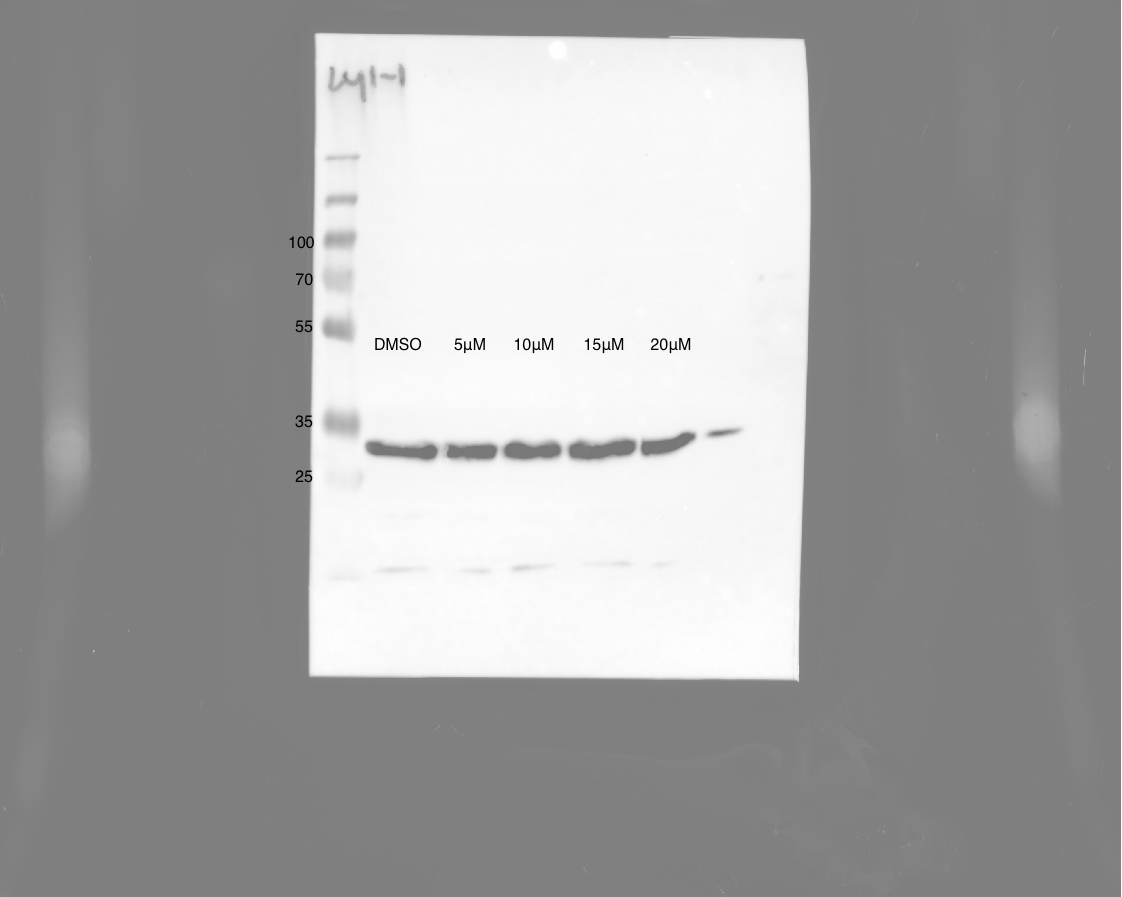

Supplement: Figure 3—source data 3. [file elife-106699-fig3-data3.zip › Figure 3ΓÇösource data 3 PDF files containing original western blots for Figure 3B, indicating the relevant bands and treatments./Raw data/Lyl1 DND-41 Abd-CRBN.tif]

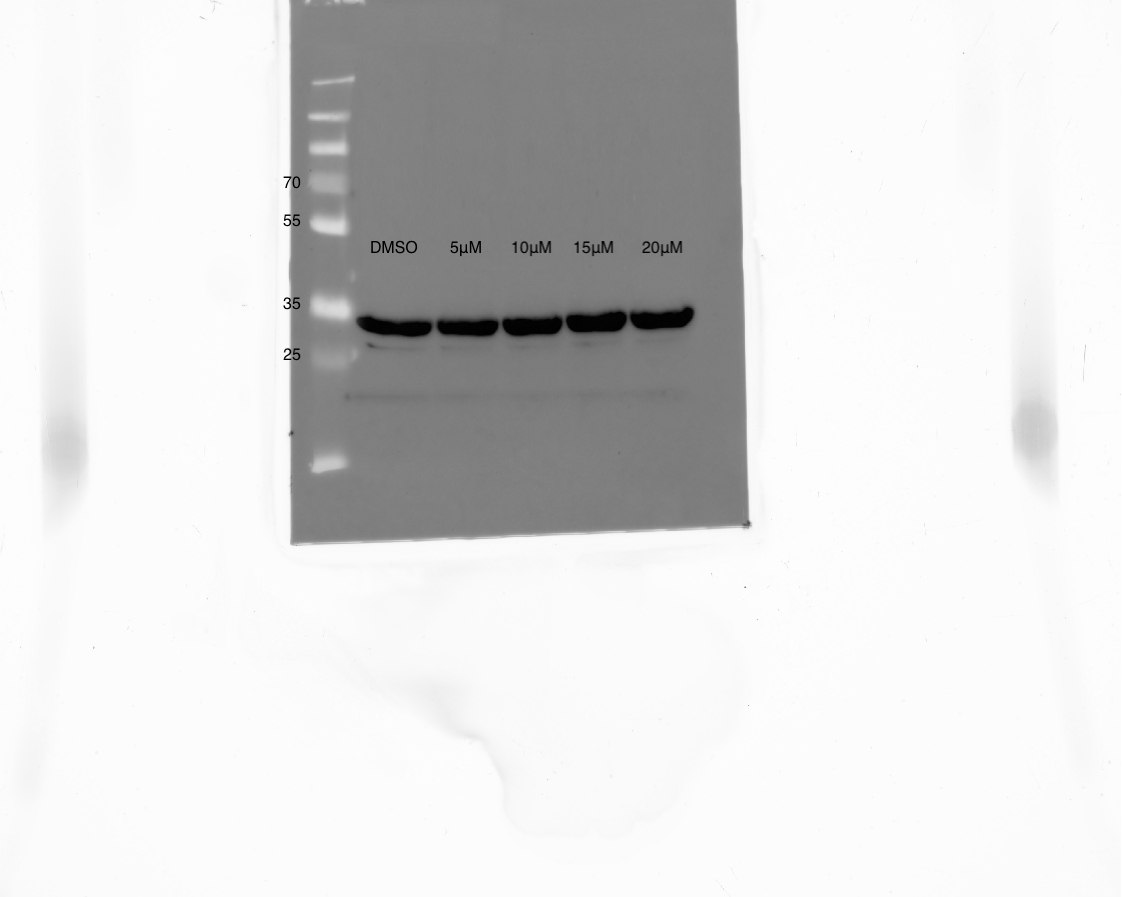

Supplement: Figure 3—source data 3. [file elife-106699-fig3-data3.zip › Figure 3ΓÇösource data 3 PDF files containing original western blots for Figure 3B, indicating the relevant bands and treatments./Raw data/Lyl1 DND-41 Abd-VHL.tif]

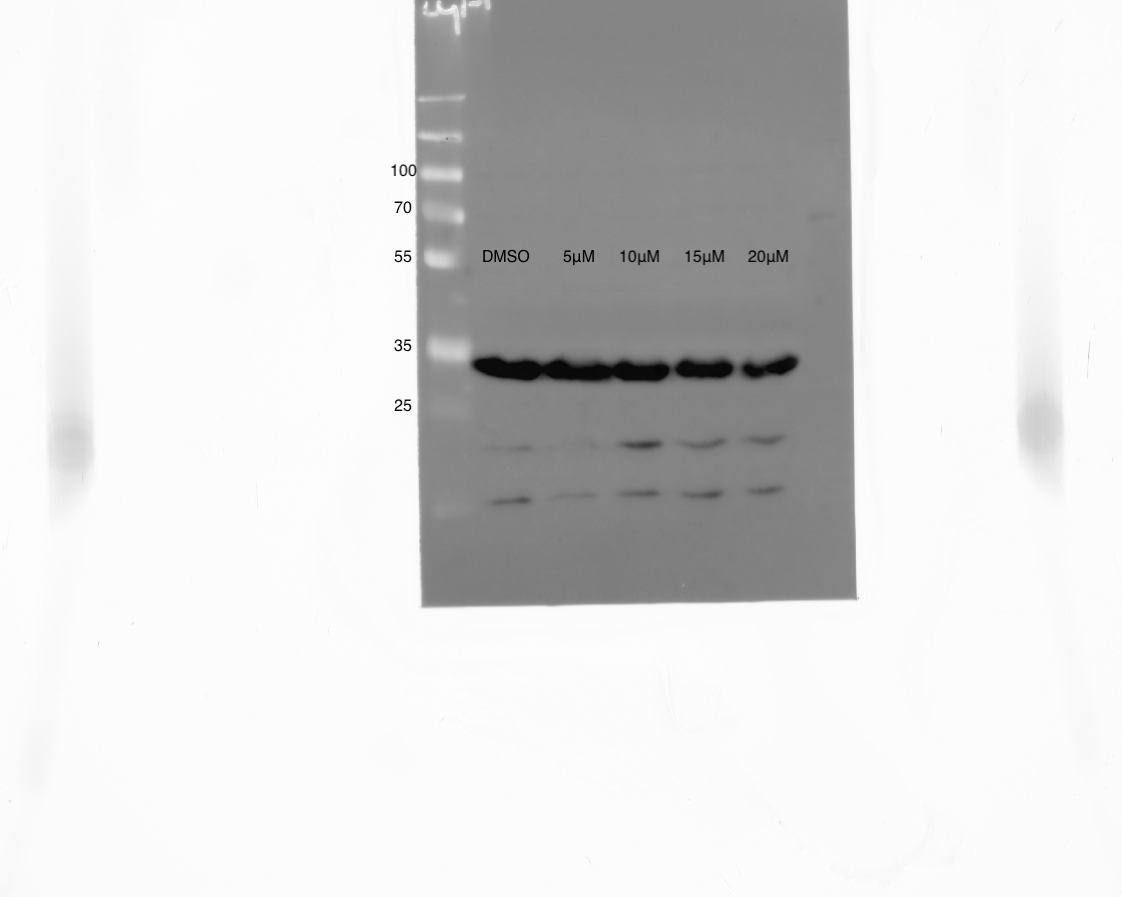

Supplement: Figure 3—source data 3. [file elife-106699-fig3-data3.zip › Figure 3ΓÇösource data 3 PDF files containing original western blots for Figure 3B, indicating the relevant bands and treatments./Raw data/Lyl1 Jurkat Abd-CRBN.tif]

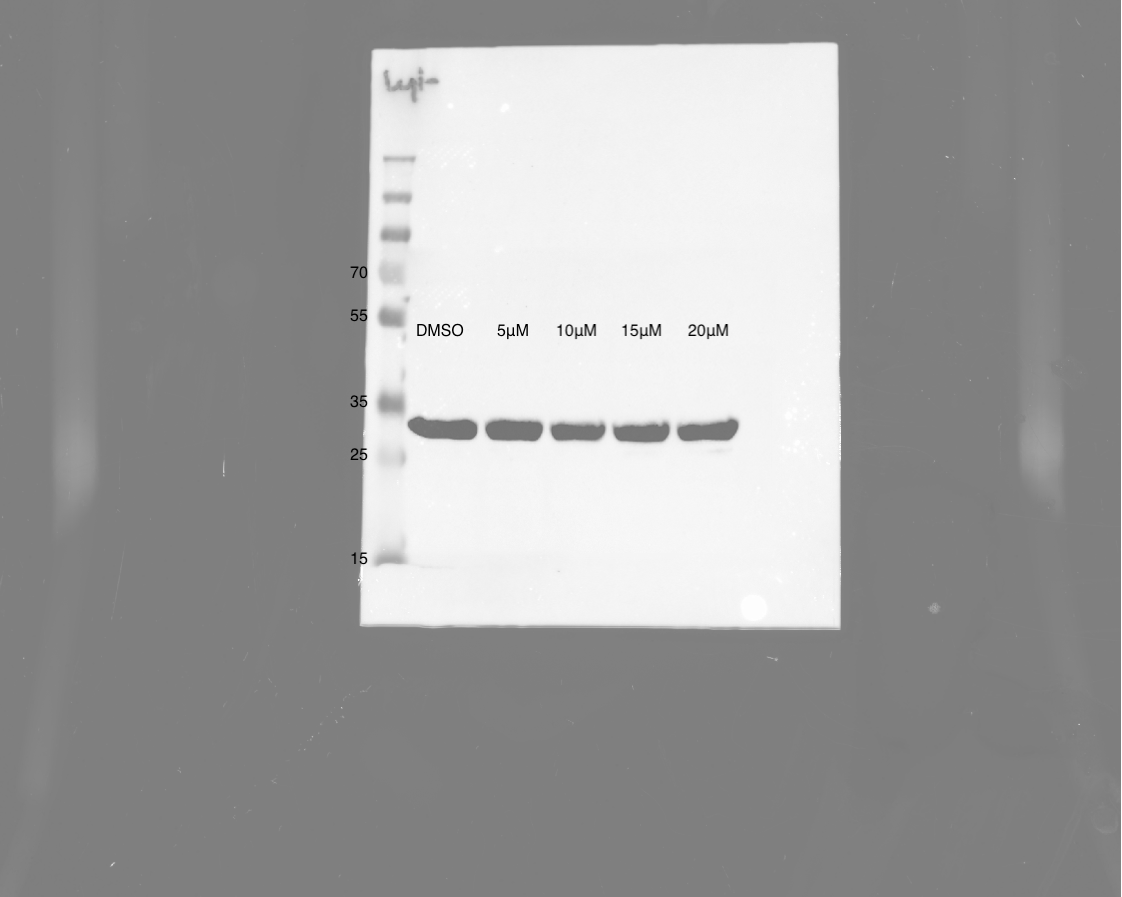

Supplement: Figure 3—source data 3. [file elife-106699-fig3-data3.zip › Figure 3ΓÇösource data 3 PDF files containing original western blots for Figure 3B, indicating the relevant bands and treatments./Raw data/Lyl1 Jurkat Abd-VHL.tif]

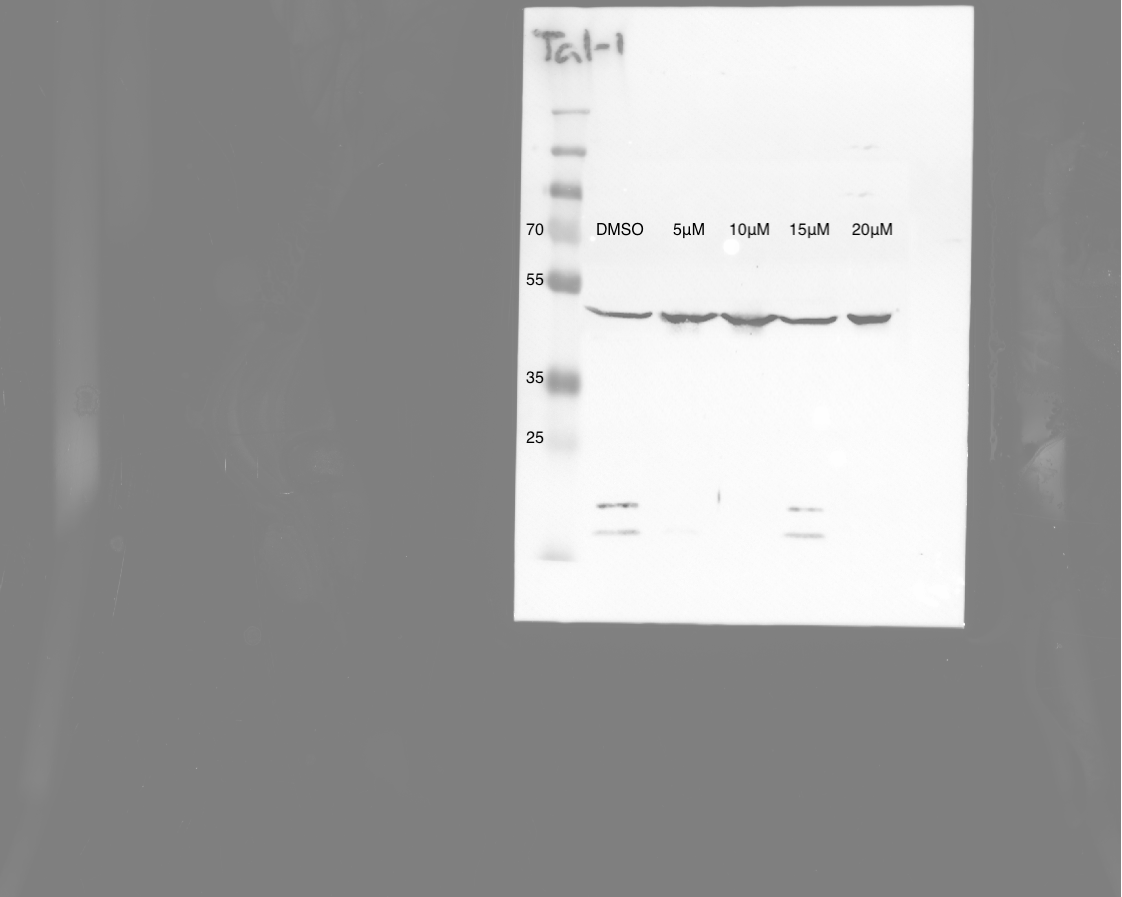

Supplement: Figure 3—source data 3. [file elife-106699-fig3-data3.zip › Figure 3ΓÇösource data 3 PDF files containing original western blots for Figure 3B, indicating the relevant bands and treatments./Raw data/Tal-1 DND-41 Abd-CRBN.tif]

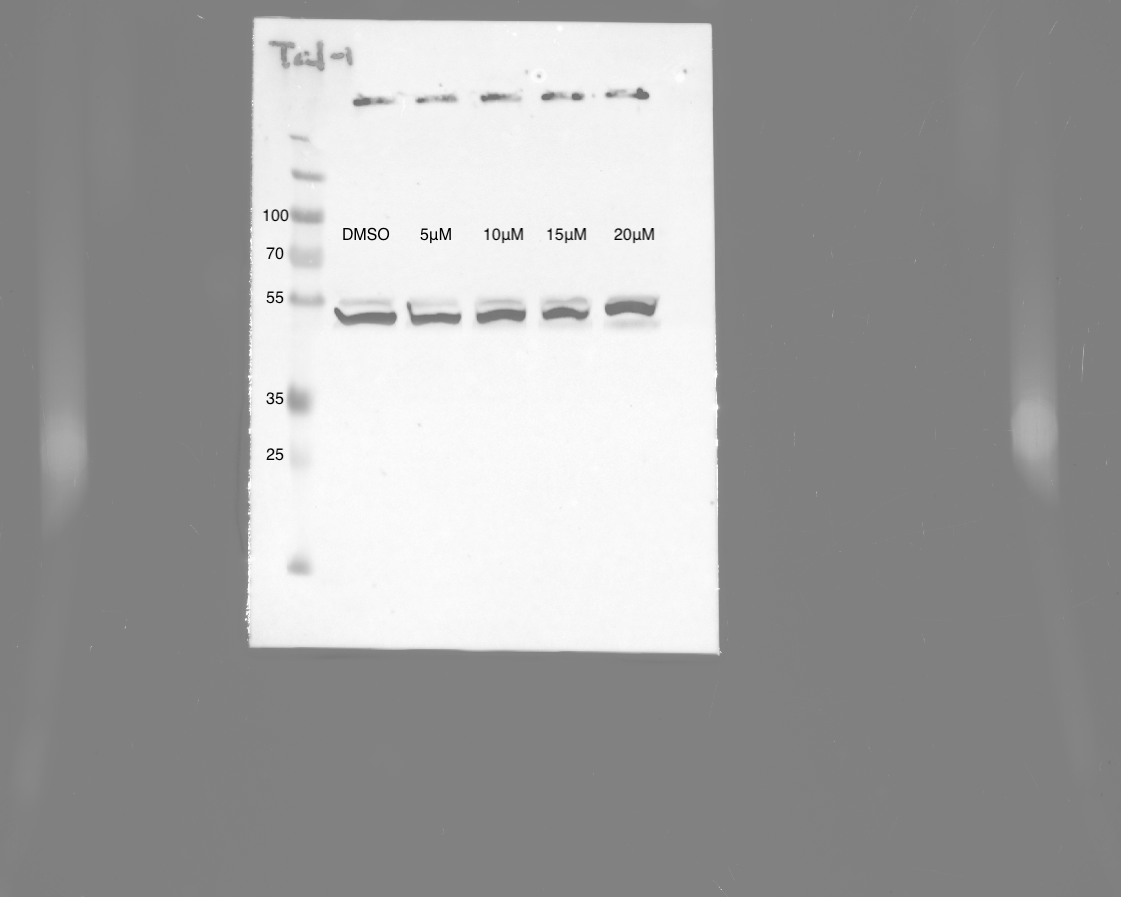

Supplement: Figure 3—source data 3. [file elife-106699-fig3-data3.zip › Figure 3ΓÇösource data 3 PDF files containing original western blots for Figure 3B, indicating the relevant bands and treatments./Raw data/Tal-1 DND-41 Abd-VHL.tif]

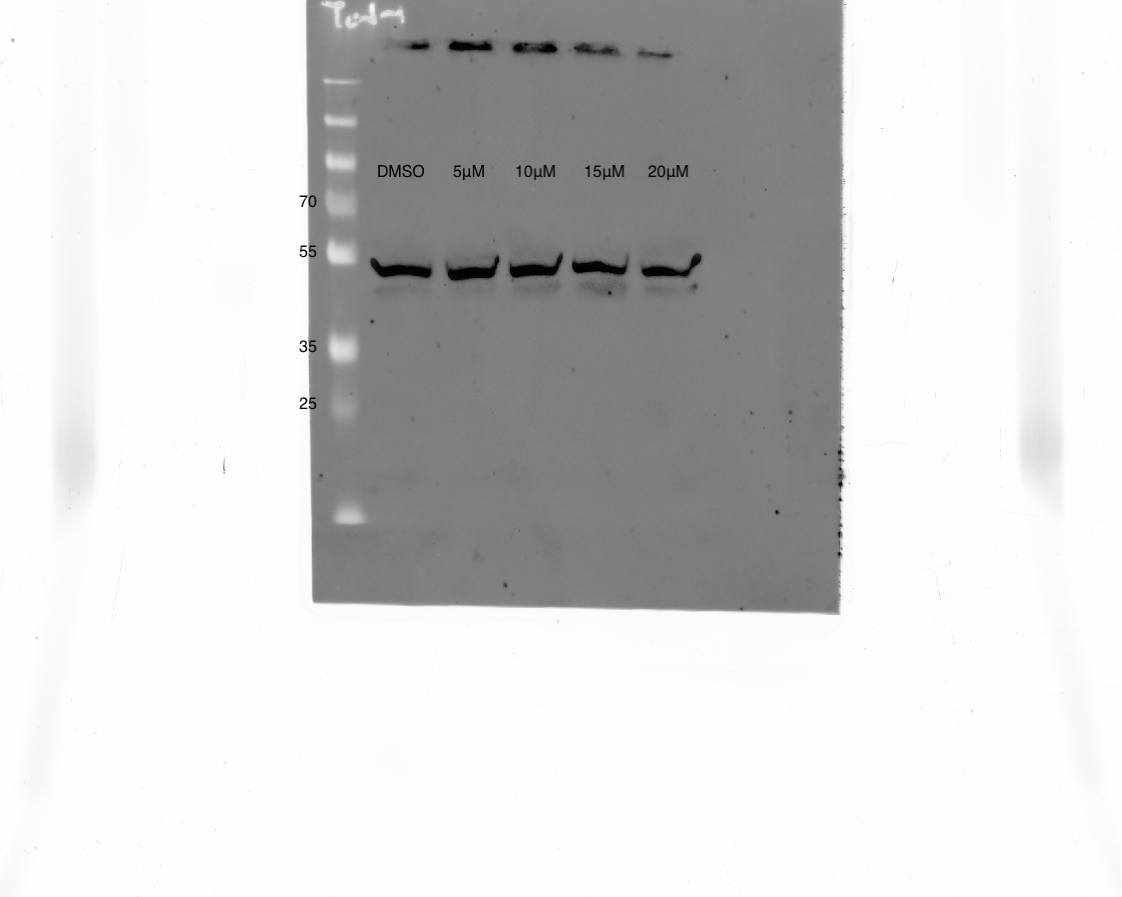

Supplement: Figure 3—source data 3. [file elife-106699-fig3-data3.zip › Figure 3ΓÇösource data 3 PDF files containing original western blots for Figure 3B, indicating the relevant bands and treatments./Raw data/Tal-1 Jukat Abd-VHL.tif]

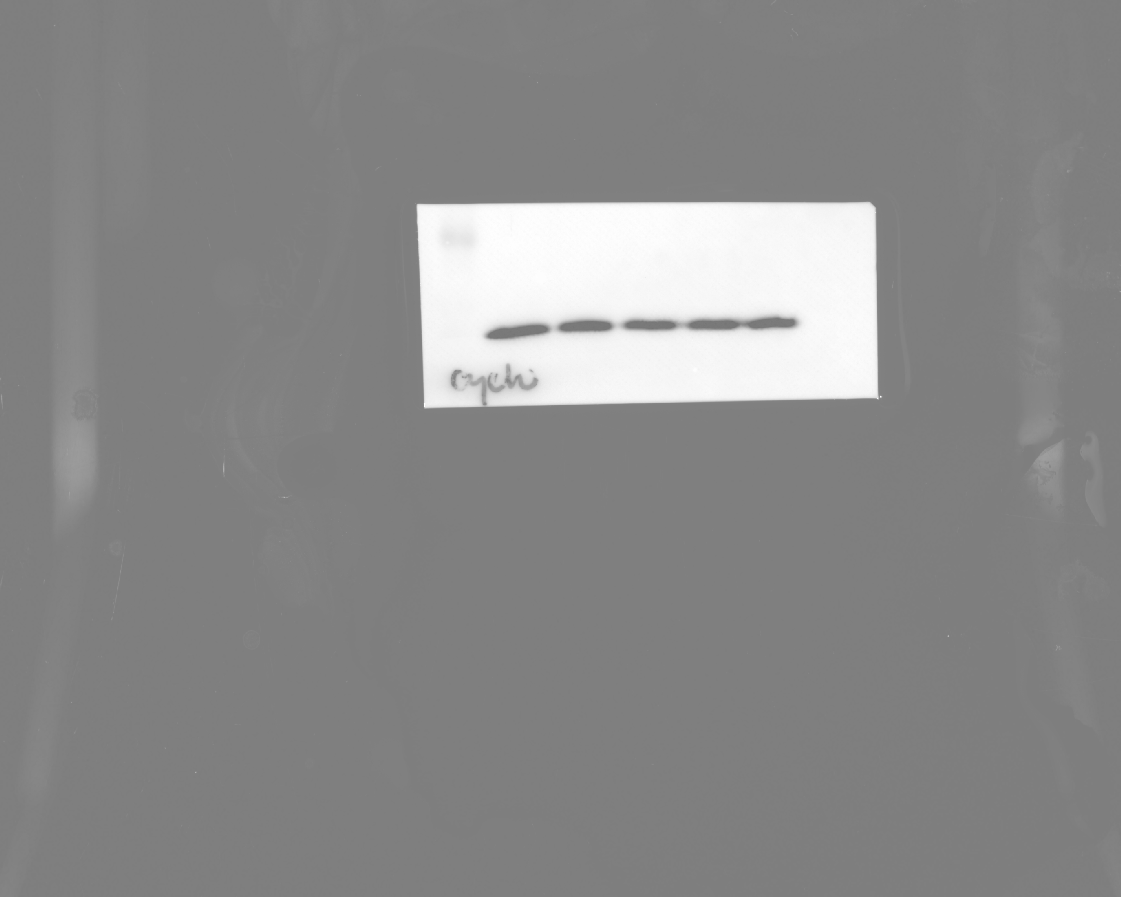

Supplement: Figure 3—source data 4. [file elife-106699-fig3-data4.zip › Figure 3ΓÇösource data 4 Original files for Western blot analysis displayed in Figure 3B./Cyclophilin DND-41 Abd-CRBN.tif]

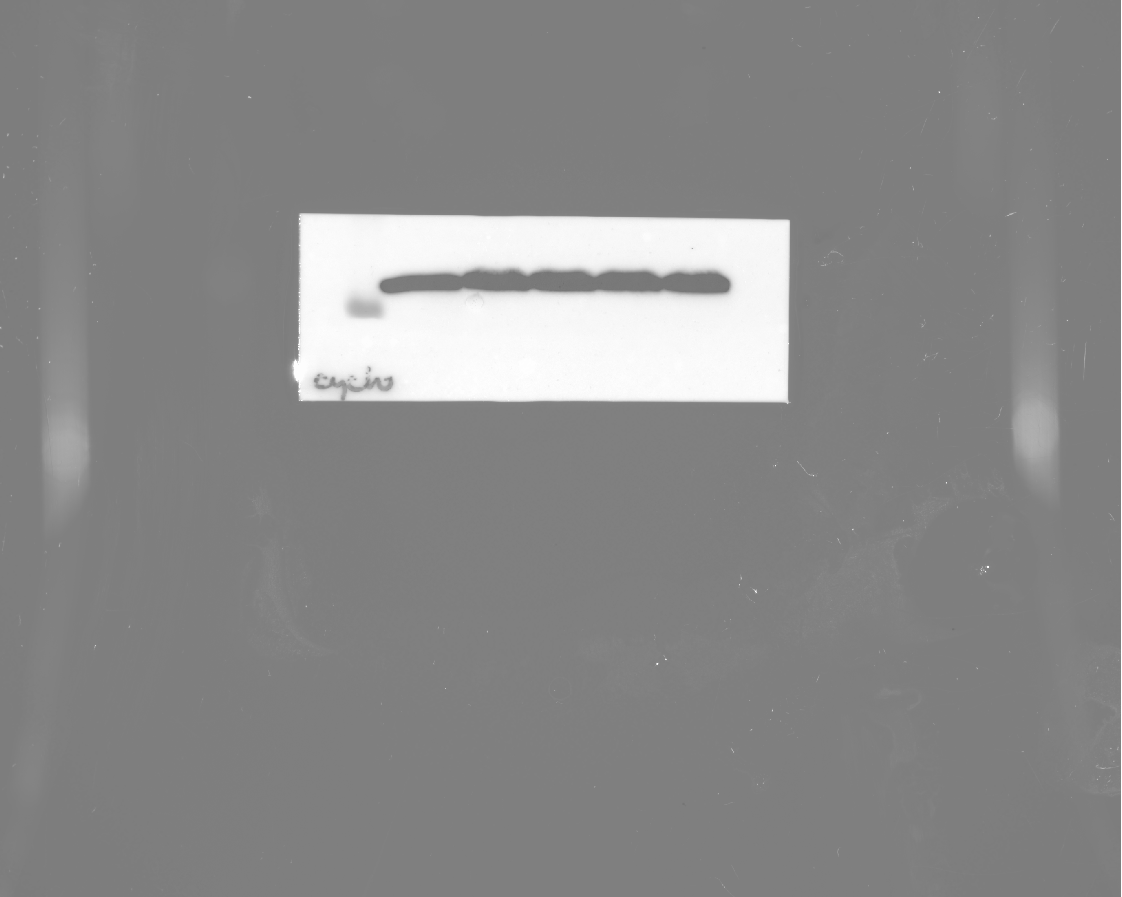

Supplement: Figure 3—source data 4. [file elife-106699-fig3-data4.zip › Figure 3ΓÇösource data 4 Original files for Western blot analysis displayed in Figure 3B./Cyclophilin DND-41 Abd-VHL.tif]

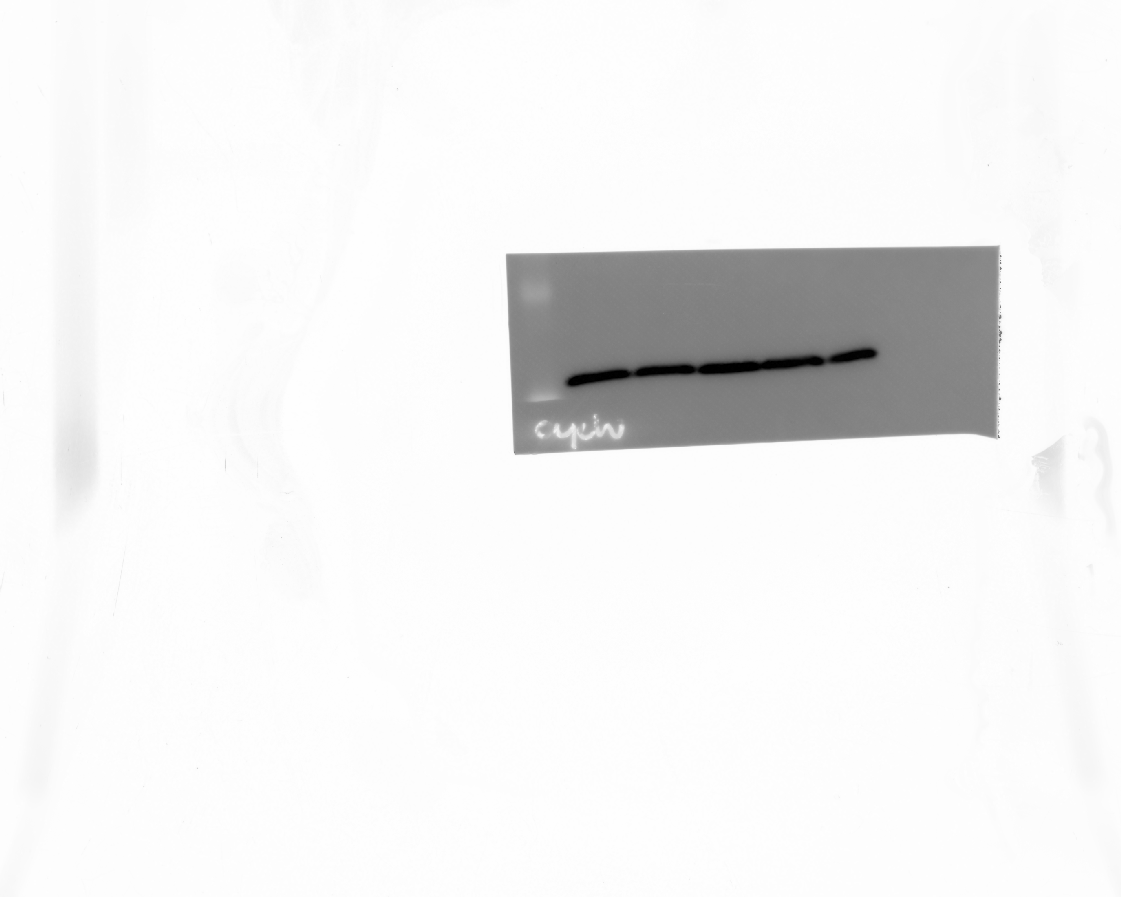

Supplement: Figure 3—source data 4. [file elife-106699-fig3-data4.zip › Figure 3ΓÇösource data 4 Original files for Western blot analysis displayed in Figure 3B./Cyclophilin Jurkat Abd-CRBN.tif]

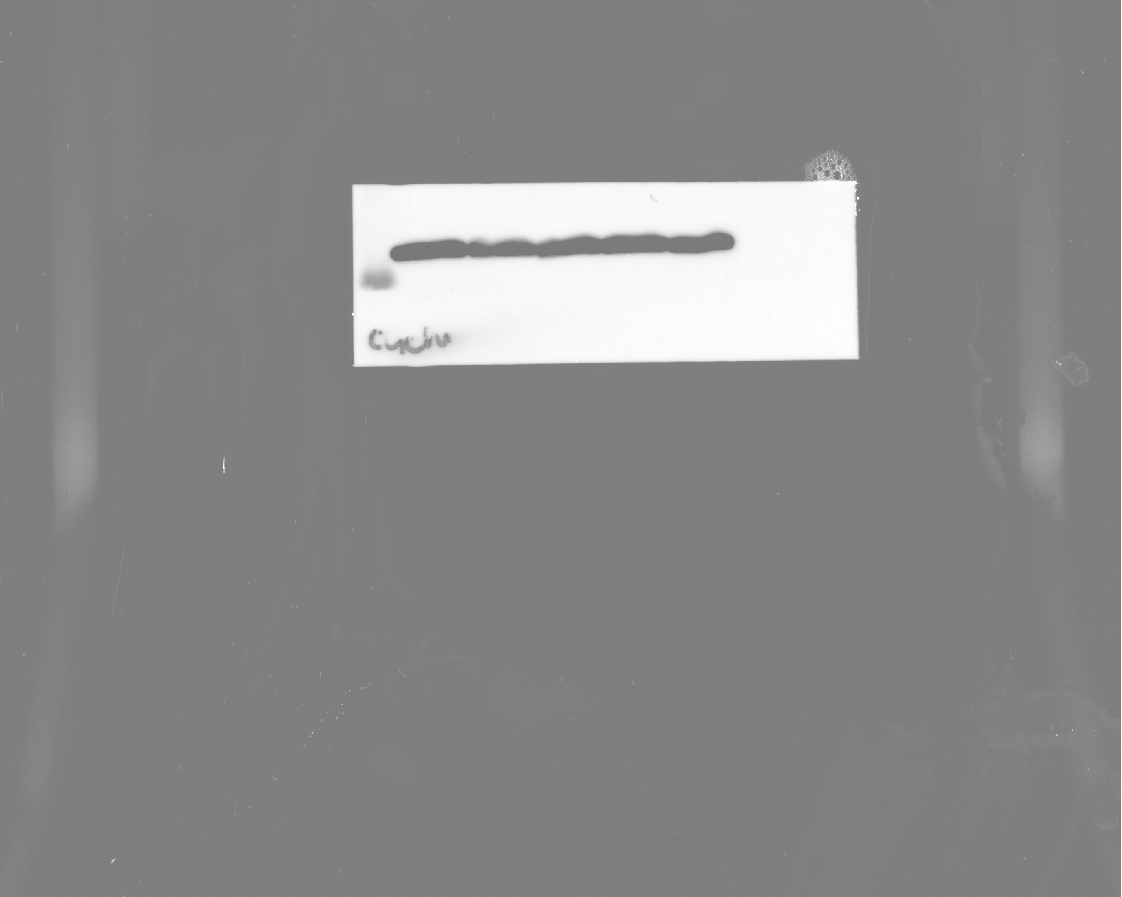

Supplement: Figure 3—source data 4. [file elife-106699-fig3-data4.zip › Figure 3ΓÇösource data 4 Original files for Western blot analysis displayed in Figure 3B./Cyclophilin Jurkat Abd-VHL.tif]

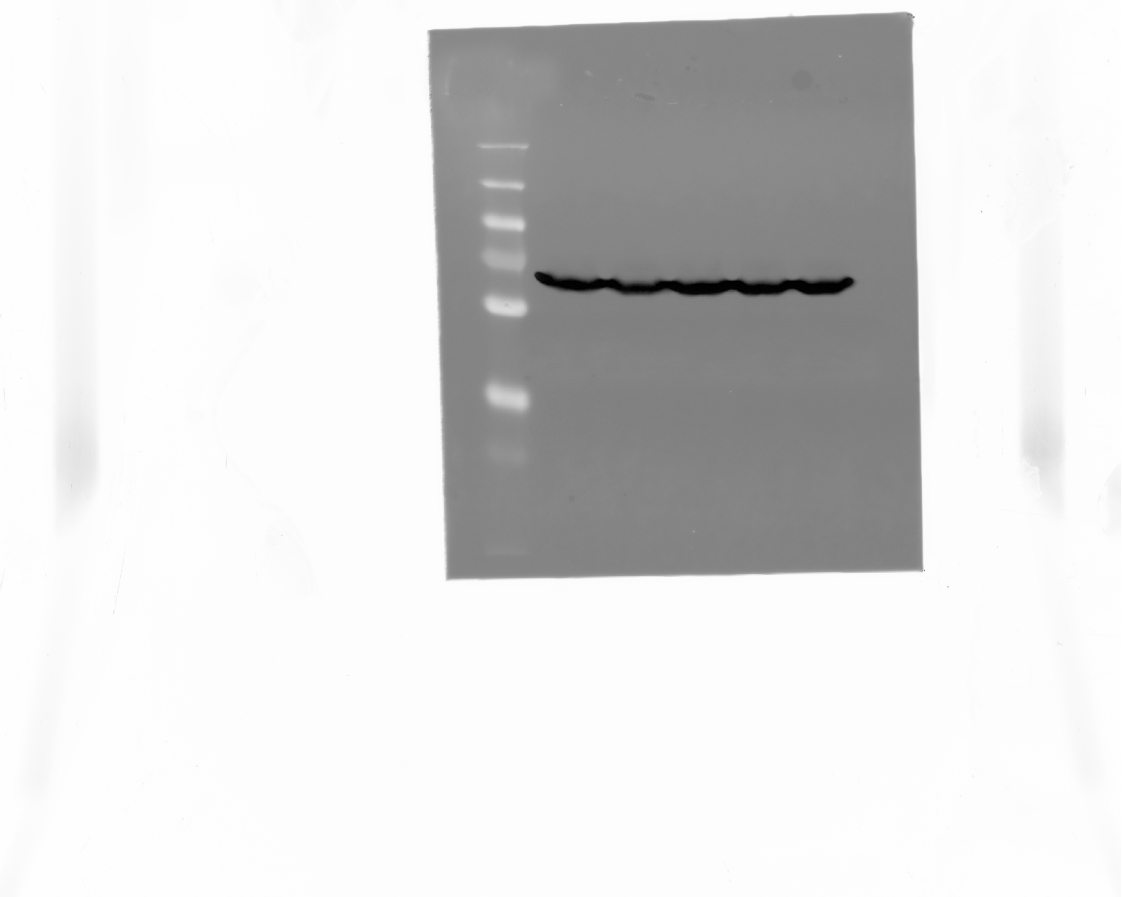

Supplement: Figure 3—source data 4. [file elife-106699-fig3-data4.zip › Figure 3ΓÇösource data 4 Original files for Western blot analysis displayed in Figure 3B./E2A DND-41 Abd-CRBN.tif]

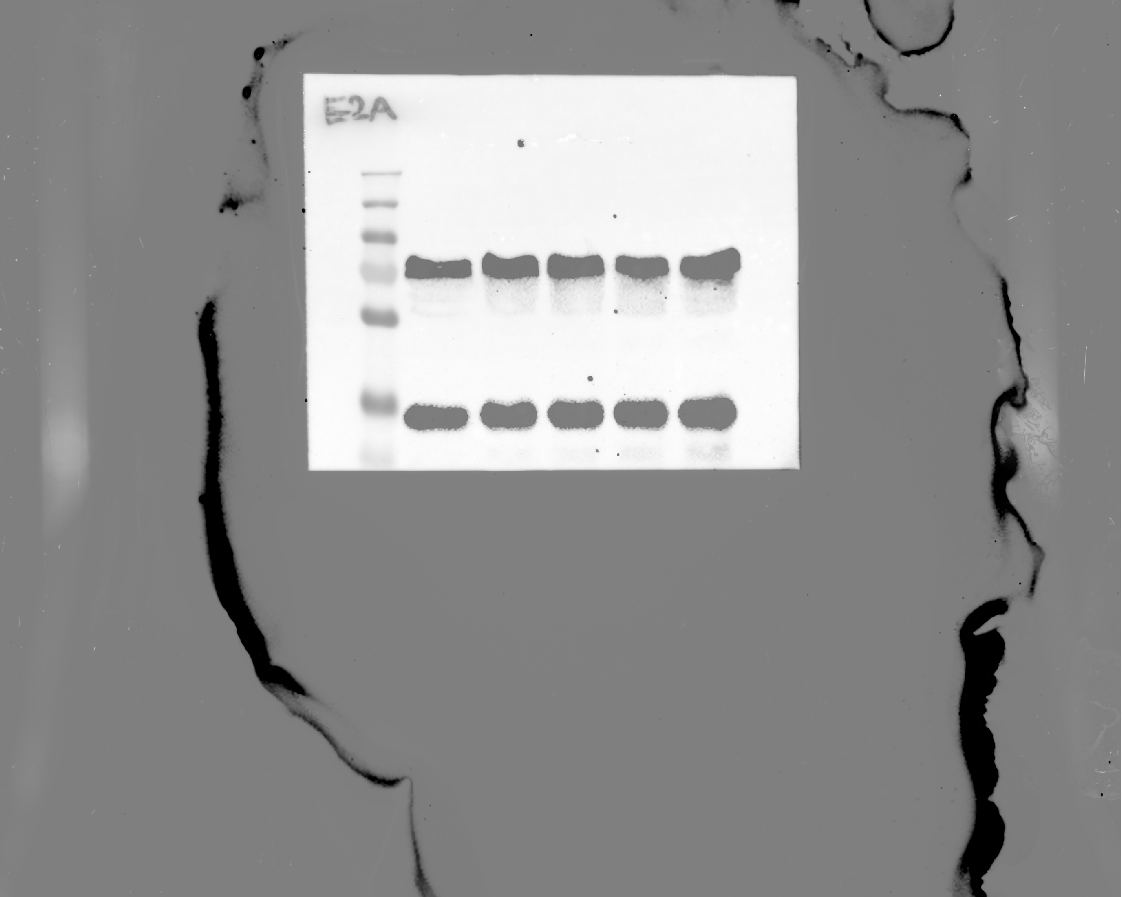

Supplement: Figure 3—source data 4. [file elife-106699-fig3-data4.zip › Figure 3ΓÇösource data 4 Original files for Western blot analysis displayed in Figure 3B./E2A DND-41 Abd-VHL.tif]

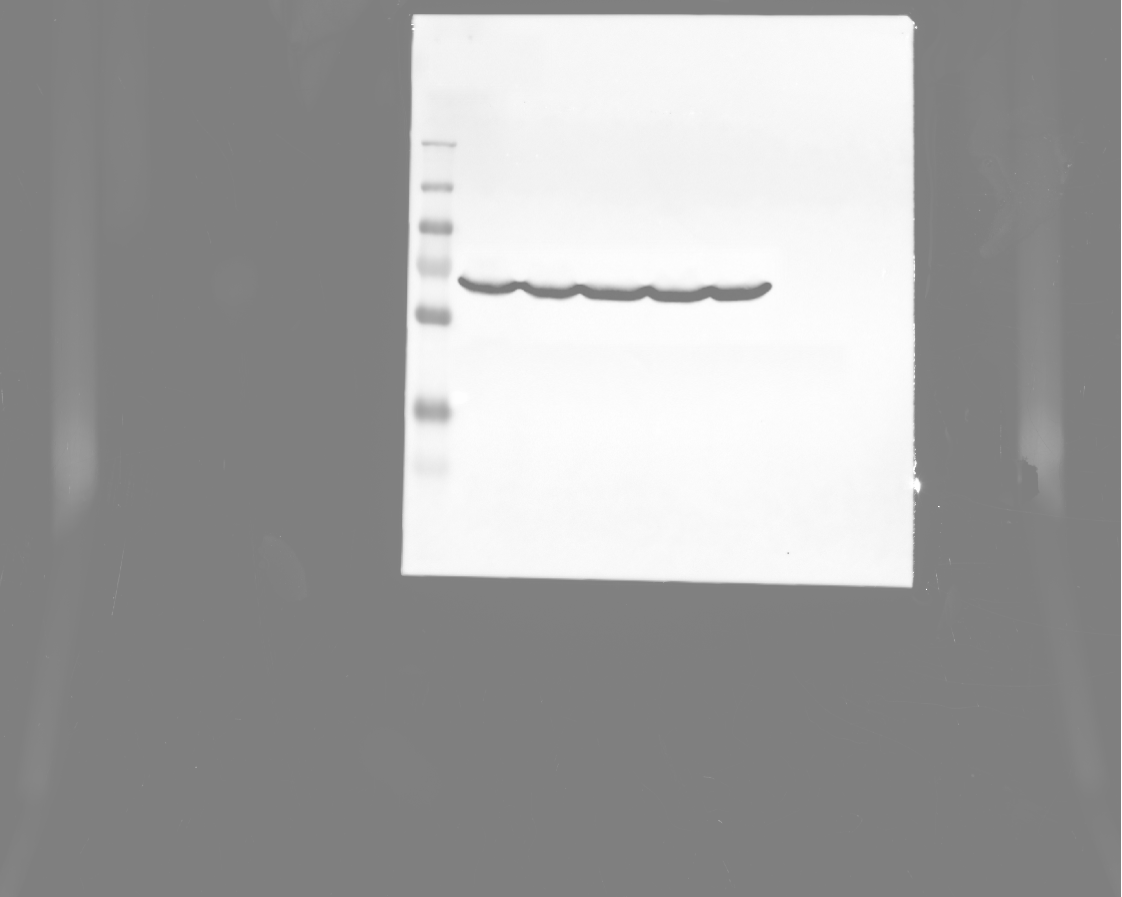

Supplement: Figure 3—source data 4. [file elife-106699-fig3-data4.zip › Figure 3ΓÇösource data 4 Original files for Western blot analysis displayed in Figure 3B./E2A Jurkat Abd-CRBN.tif]

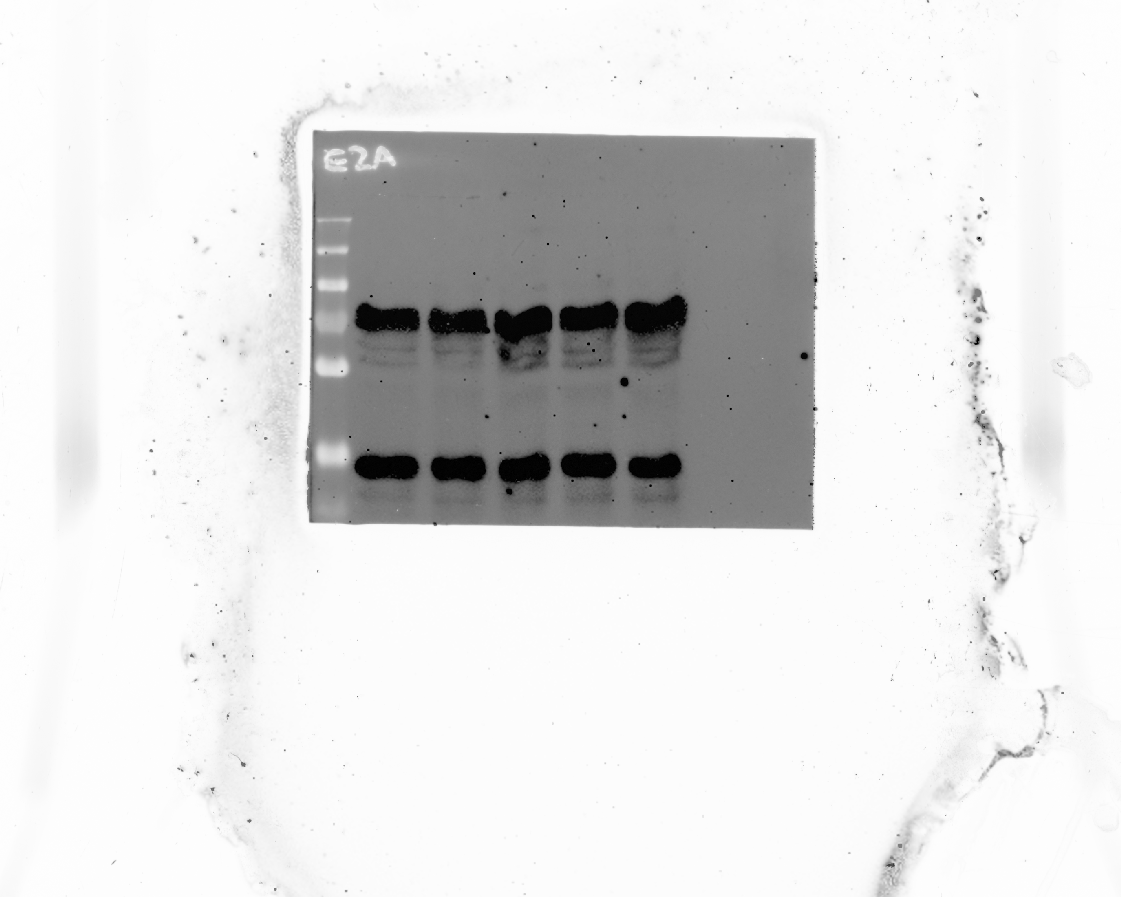

Supplement: Figure 3—source data 4. [file elife-106699-fig3-data4.zip › Figure 3ΓÇösource data 4 Original files for Western blot analysis displayed in Figure 3B./E2A Jurkat Abd-VHL.tif]

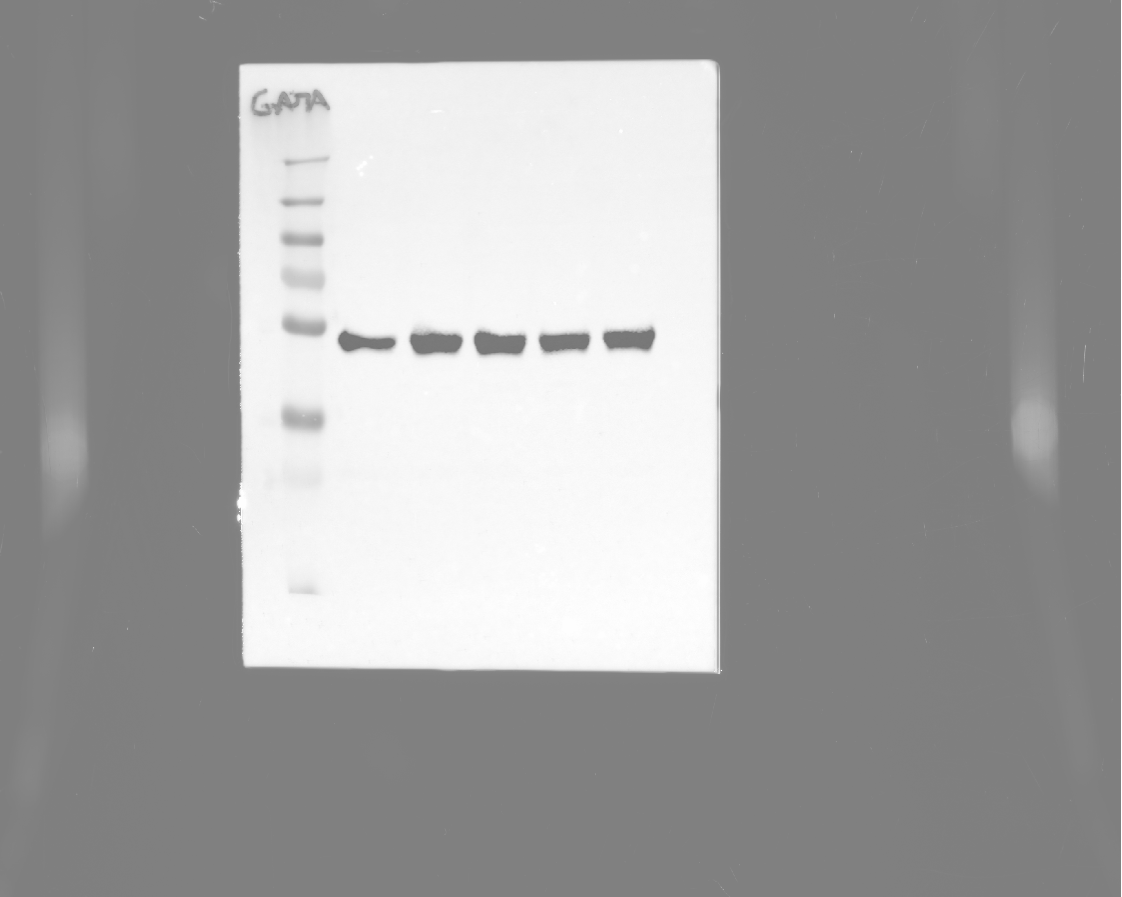

Supplement: Figure 3—source data 4. [file elife-106699-fig3-data4.zip › Figure 3ΓÇösource data 4 Original files for Western blot analysis displayed in Figure 3B./GATA3 DND-41 Abd-VHL.tif]

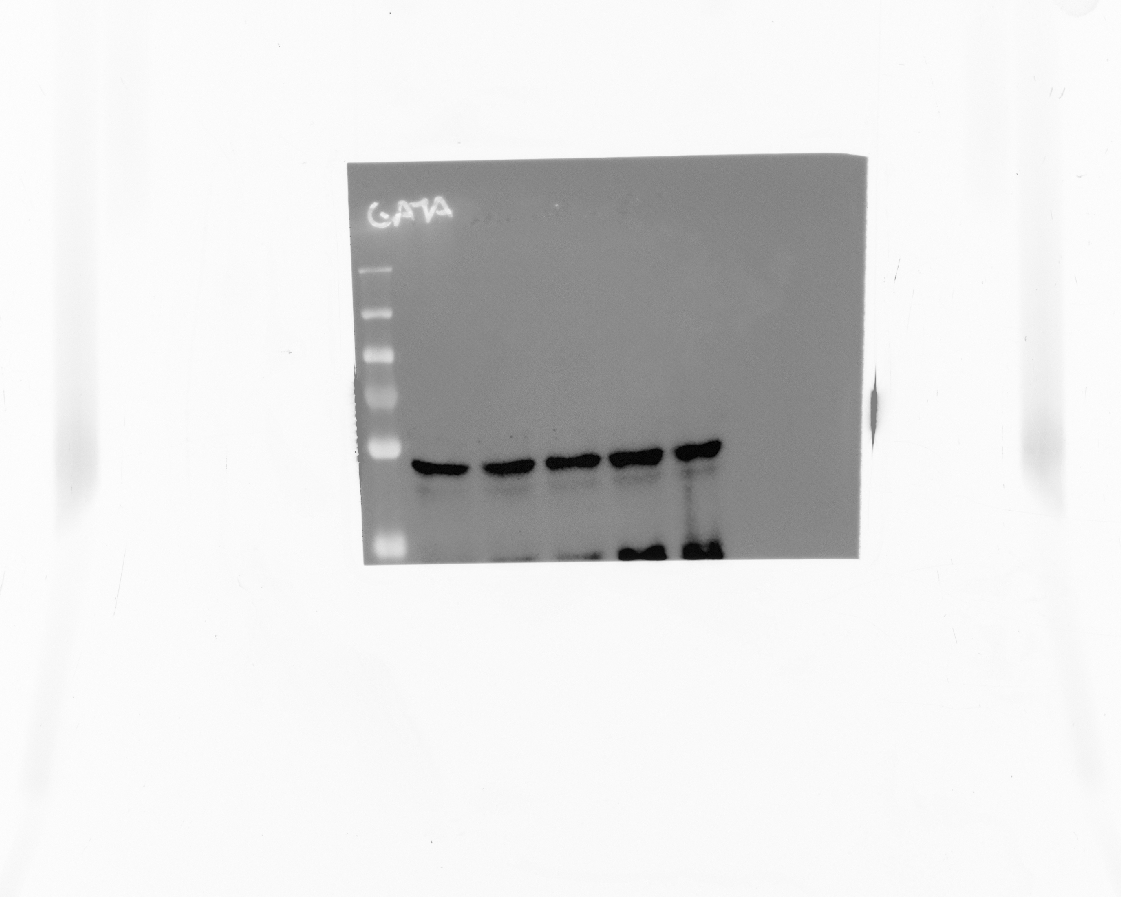

Supplement: Figure 3—source data 4. [file elife-106699-fig3-data4.zip › Figure 3ΓÇösource data 4 Original files for Western blot analysis displayed in Figure 3B./GATA3 Jurkat Abd-CRBN.tif]

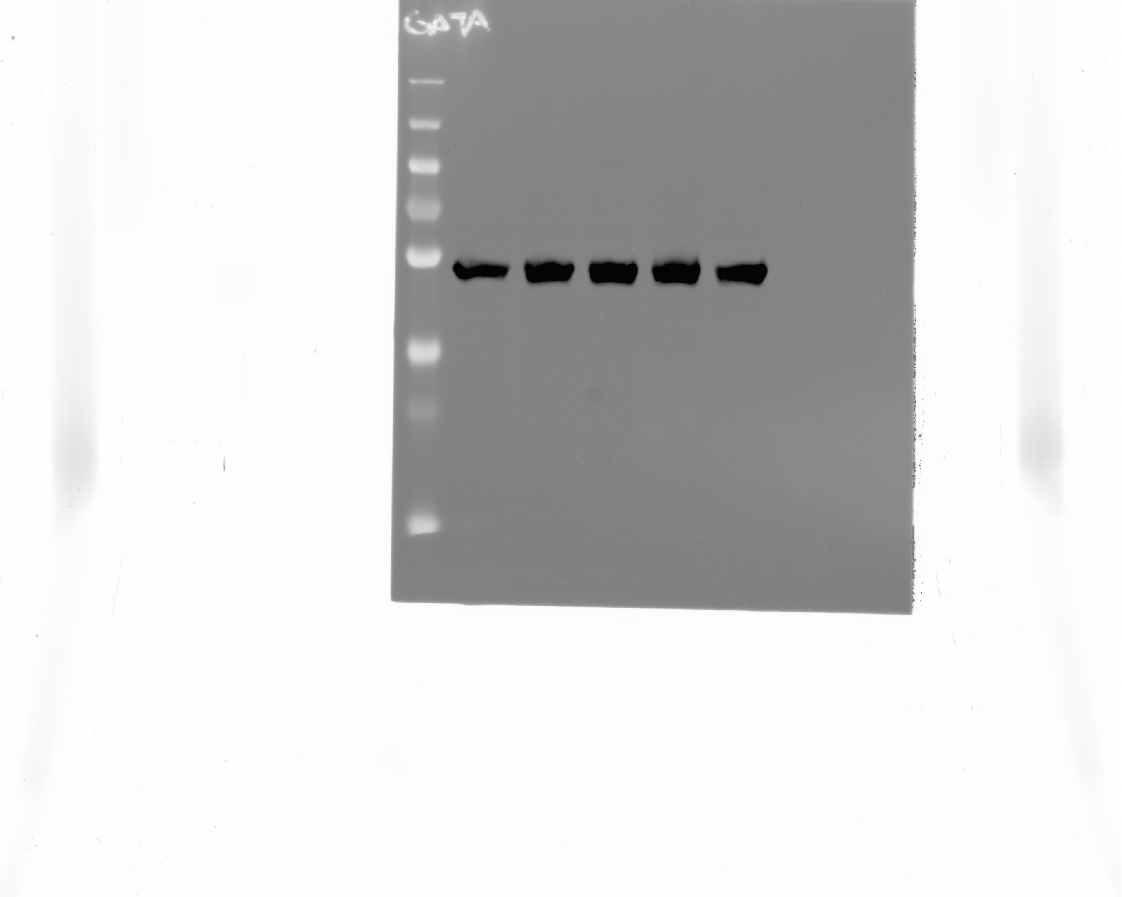

Supplement: Figure 3—source data 4. [file elife-106699-fig3-data4.zip › Figure 3ΓÇösource data 4 Original files for Western blot analysis displayed in Figure 3B./GATA3 Jurkat Abd-VHL.tif]

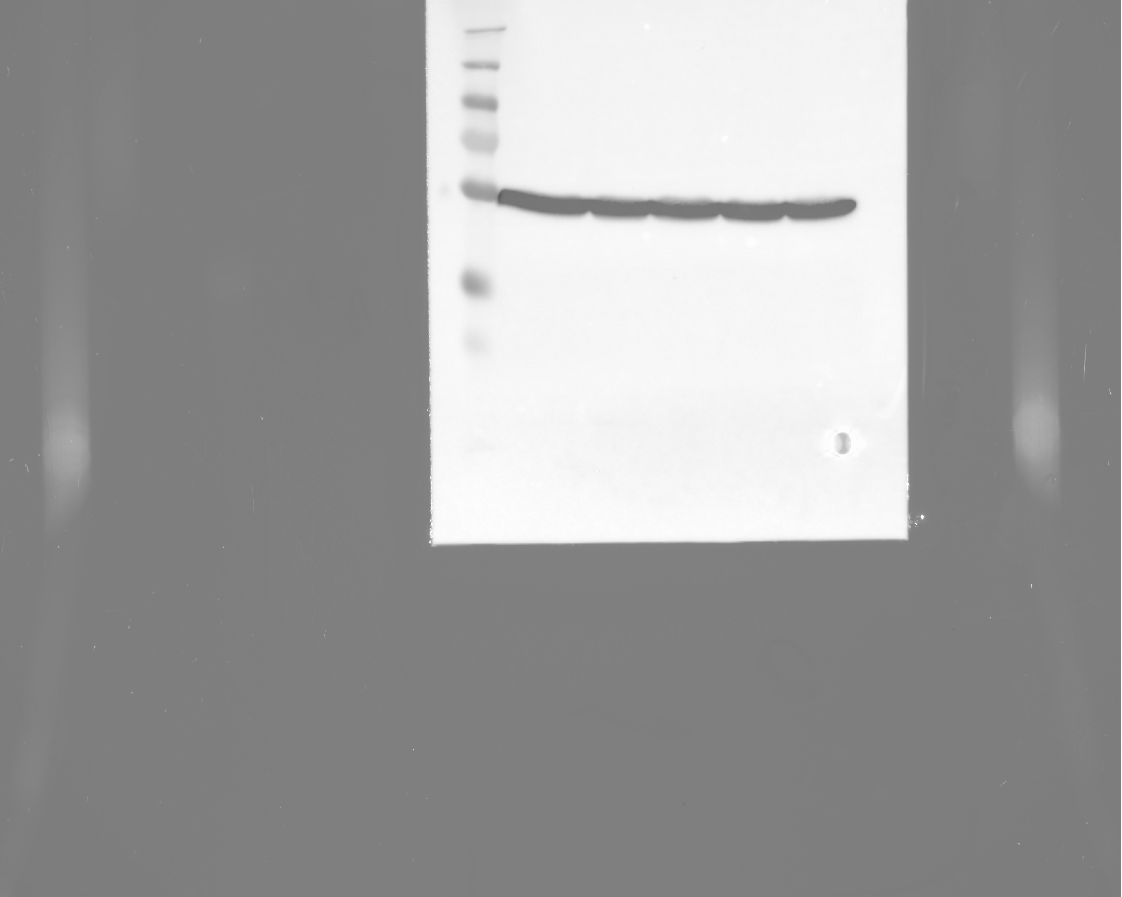

Supplement: Figure 3—source data 4. [file elife-106699-fig3-data4.zip › Figure 3ΓÇösource data 4 Original files for Western blot analysis displayed in Figure 3B./LDB1 DND-41 Abd-CRBN.tif]

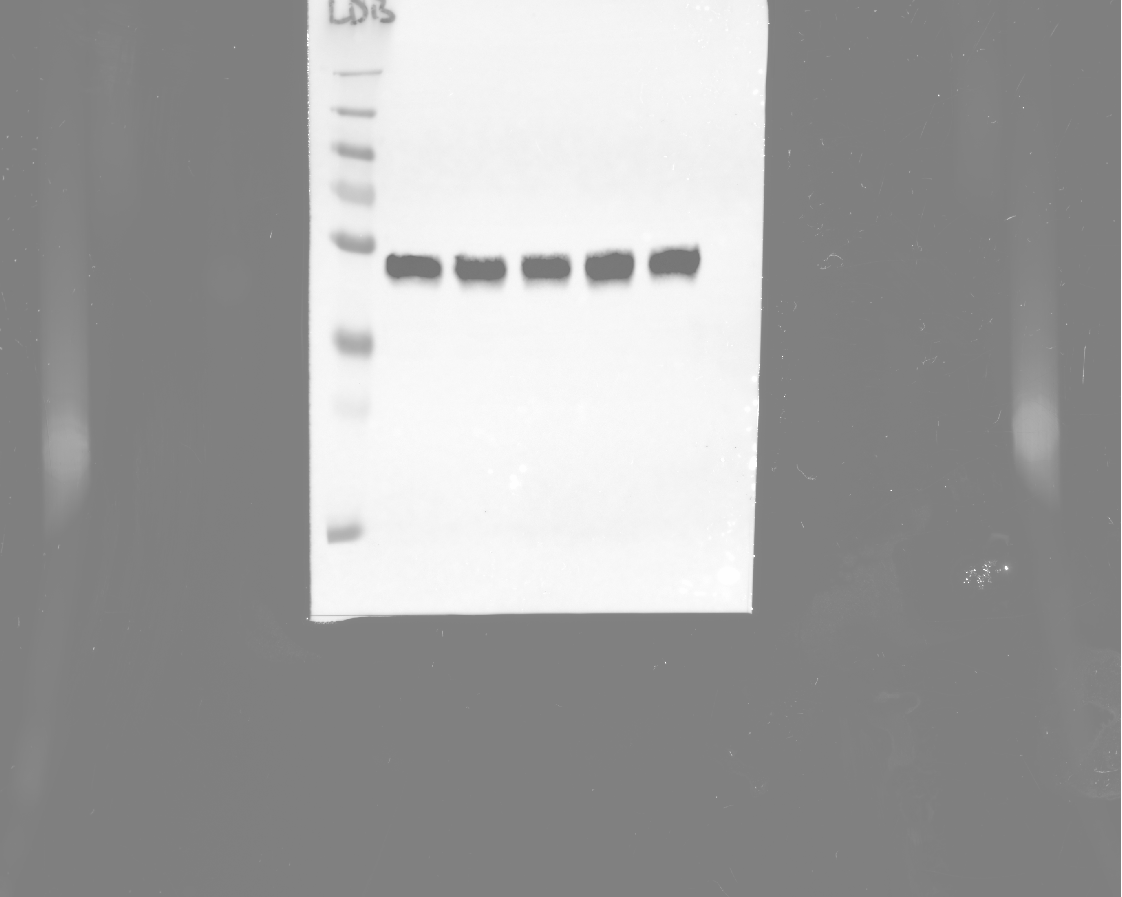

Supplement: Figure 3—source data 4. [file elife-106699-fig3-data4.zip › Figure 3ΓÇösource data 4 Original files for Western blot analysis displayed in Figure 3B./LDB1 DND-41 Abd-VHL.tif]

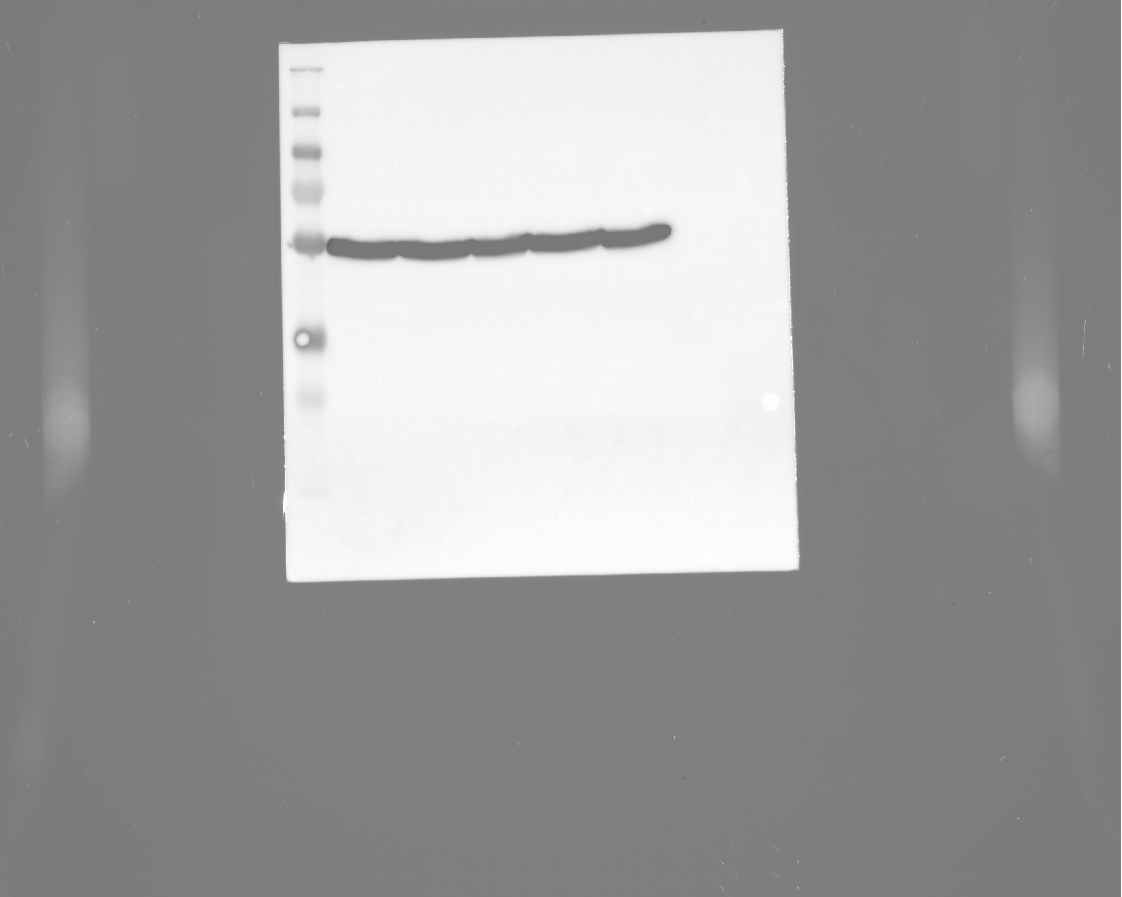

Supplement: Figure 3—source data 4. [file elife-106699-fig3-data4.zip › Figure 3ΓÇösource data 4 Original files for Western blot analysis displayed in Figure 3B./LDB1 Jurkat Abd-CRBN.tif]

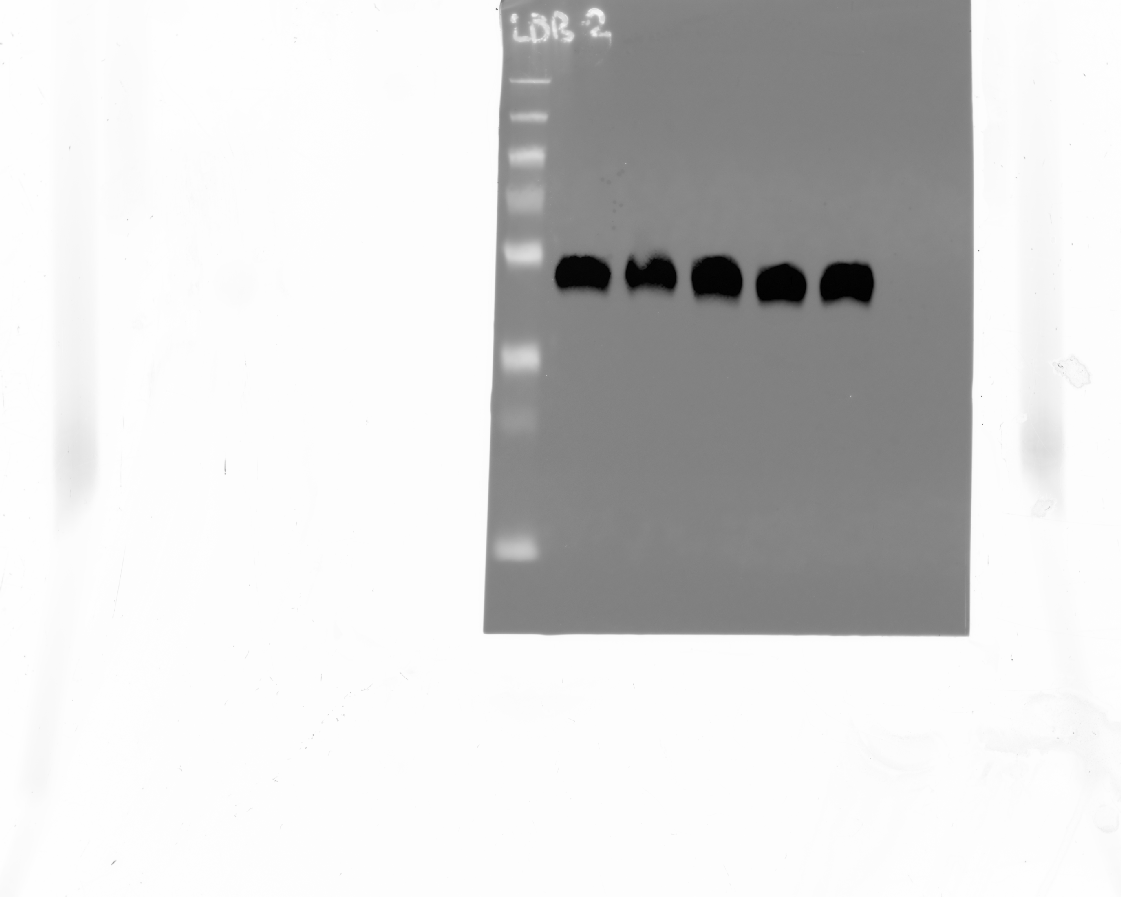

Supplement: Figure 3—source data 4. [file elife-106699-fig3-data4.zip › Figure 3ΓÇösource data 4 Original files for Western blot analysis displayed in Figure 3B./LDB1 Jurkat Abd-VHL.tif]

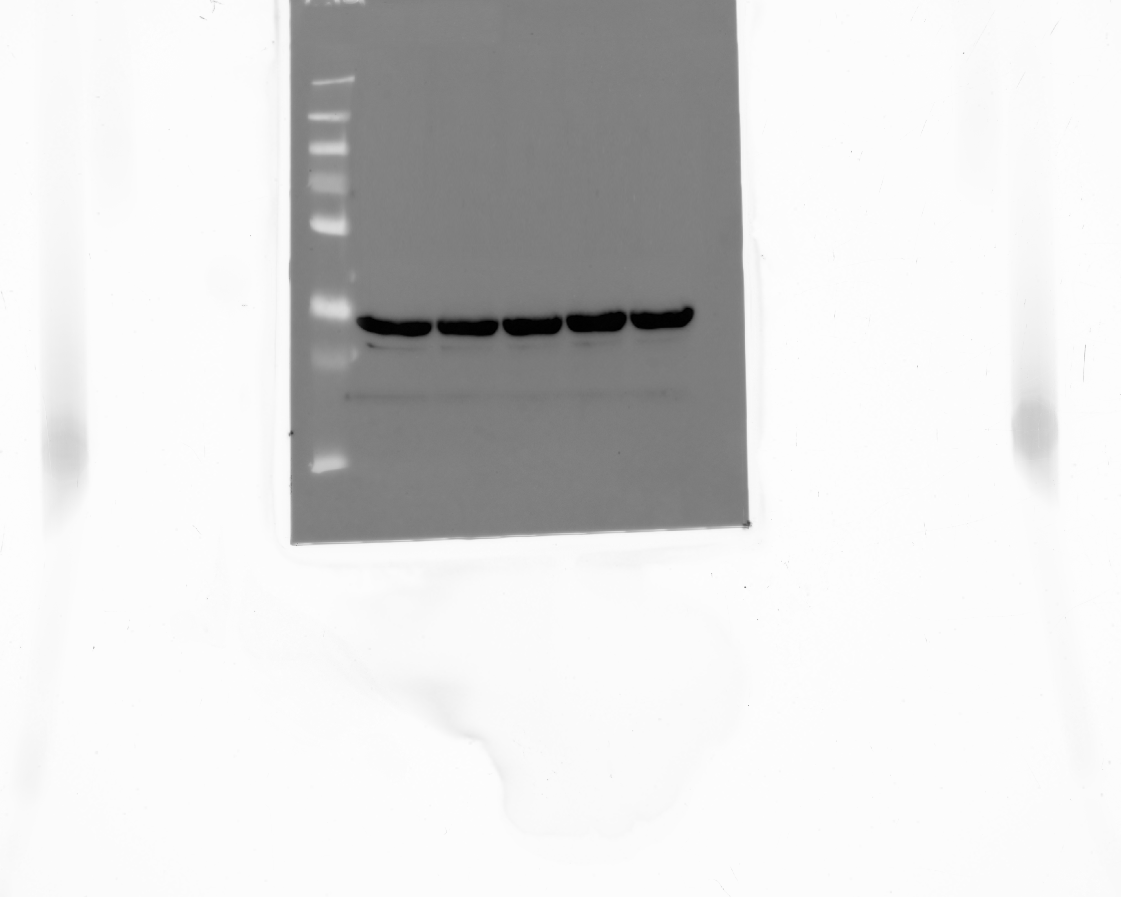

Supplement: Figure 3—source data 4. [file elife-106699-fig3-data4.zip › Figure 3ΓÇösource data 4 Original files for Western blot analysis displayed in Figure 3B./Lyl-1 DND-41 Abd-VHL.tif]

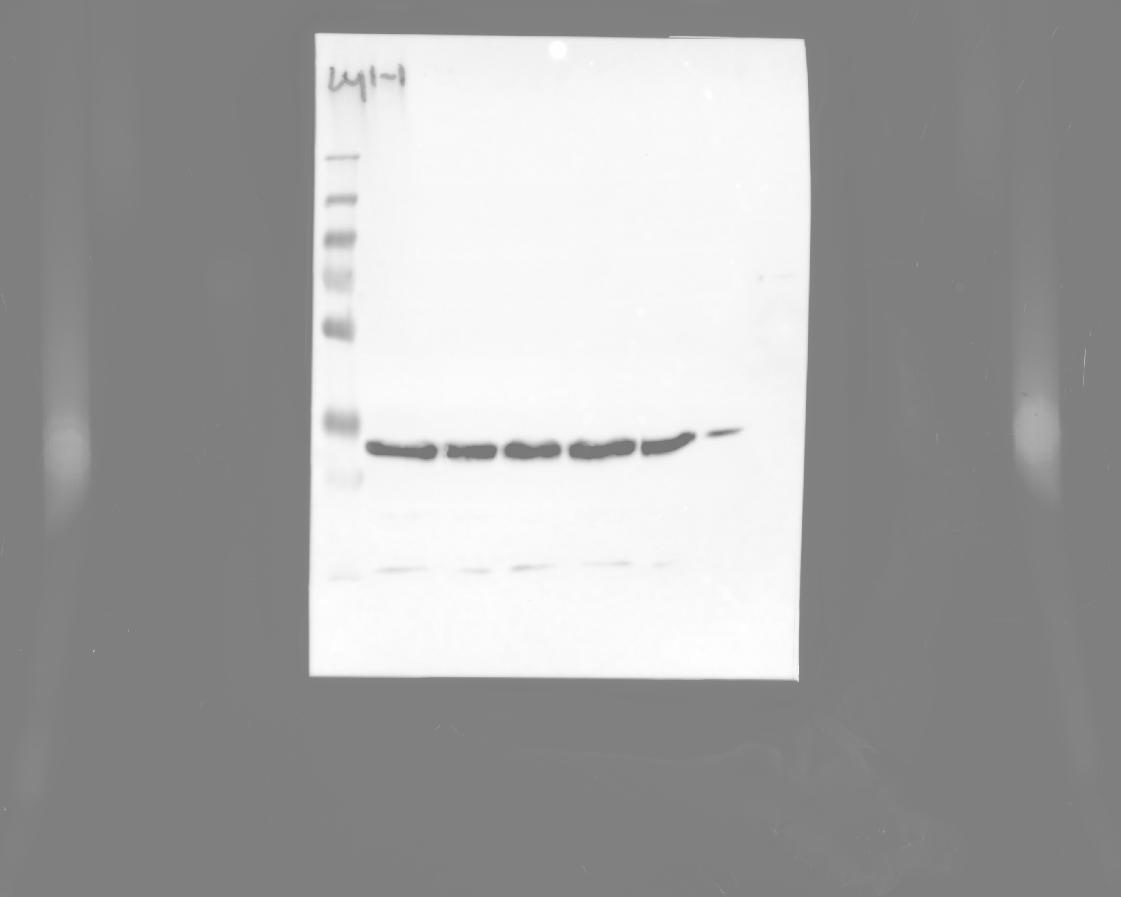

Supplement: Figure 3—source data 4. [file elife-106699-fig3-data4.zip › Figure 3ΓÇösource data 4 Original files for Western blot analysis displayed in Figure 3B./Lyl1 DND-41 Abd-CRBN.tif]

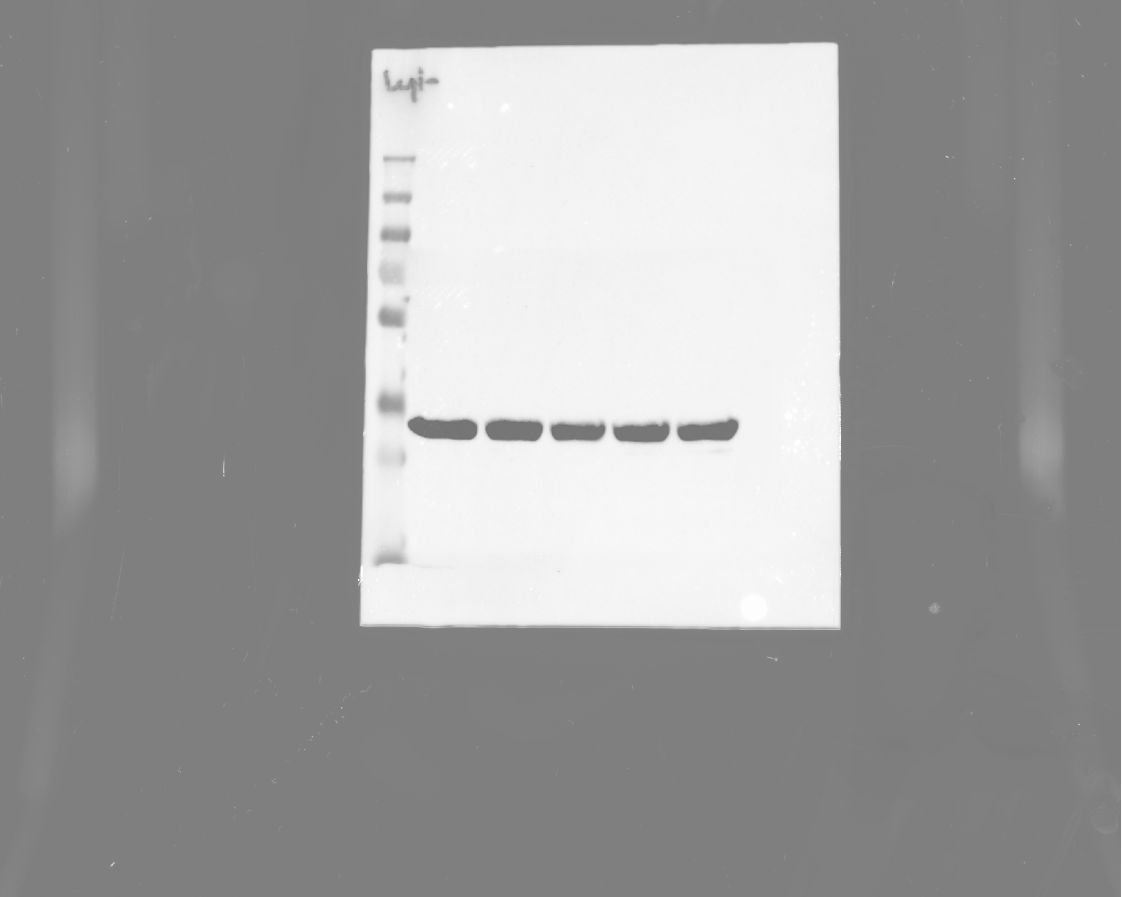

Supplement: Figure 3—source data 4. [file elife-106699-fig3-data4.zip › Figure 3ΓÇösource data 4 Original files for Western blot analysis displayed in Figure 3B./Lyl1 Jurkat Abd-VHL.tif]

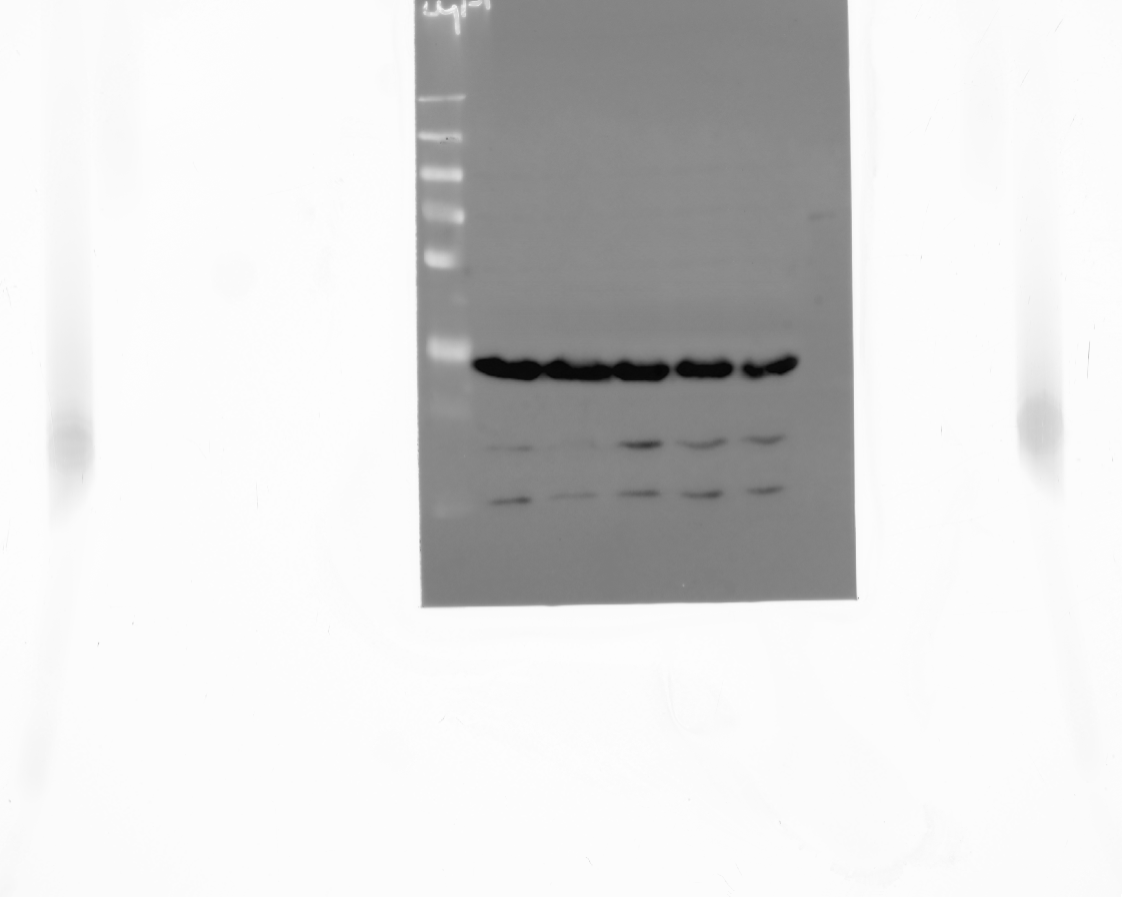

Supplement: Figure 3—source data 4. [file elife-106699-fig3-data4.zip › Figure 3ΓÇösource data 4 Original files for Western blot analysis displayed in Figure 3B./Lyl1-1 Jurkat Abd-CRBN.tif]

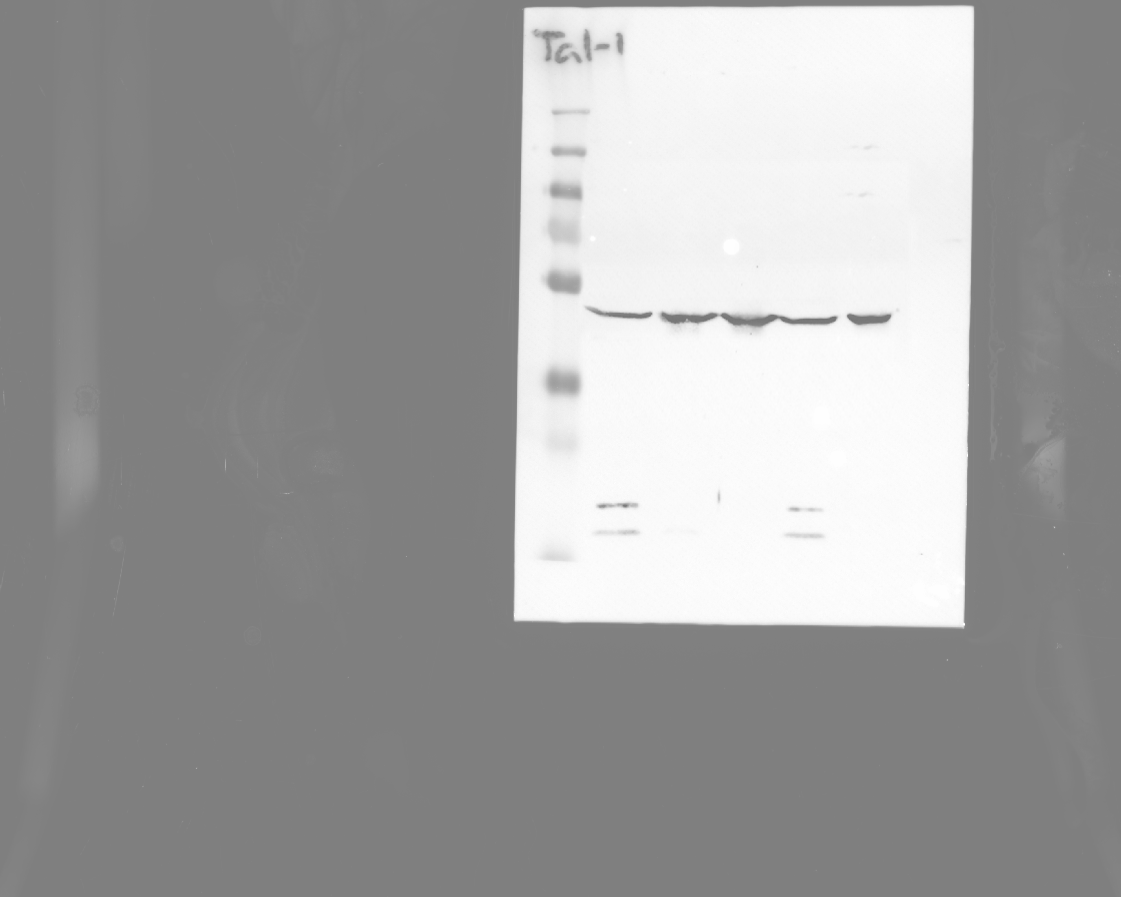

Supplement: Figure 3—source data 4. [file elife-106699-fig3-data4.zip › Figure 3ΓÇösource data 4 Original files for Western blot analysis displayed in Figure 3B./Tal-1 DND-41 Abd-CRBN.tif]

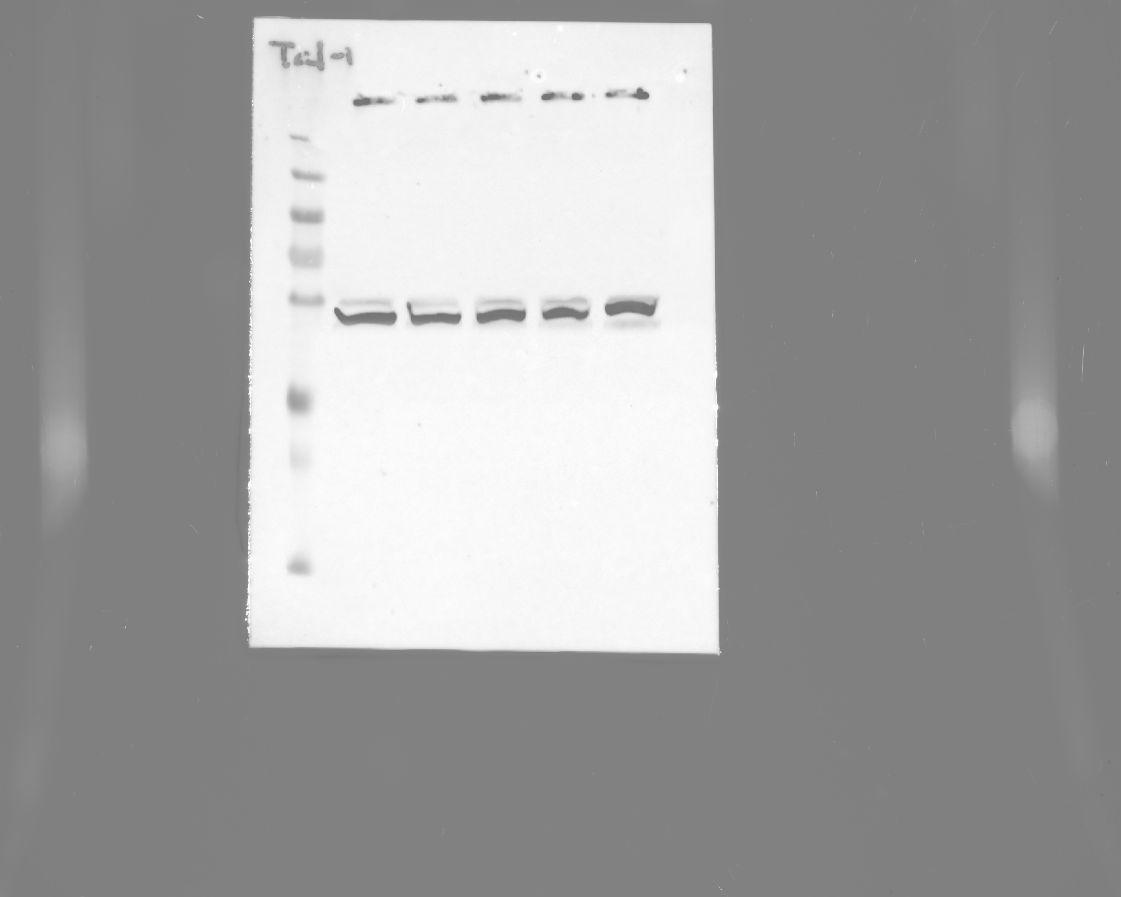

Supplement: Figure 3—source data 4. [file elife-106699-fig3-data4.zip › Figure 3ΓÇösource data 4 Original files for Western blot analysis displayed in Figure 3B./Tal-1 DND-41 Abd-VHL.tif]

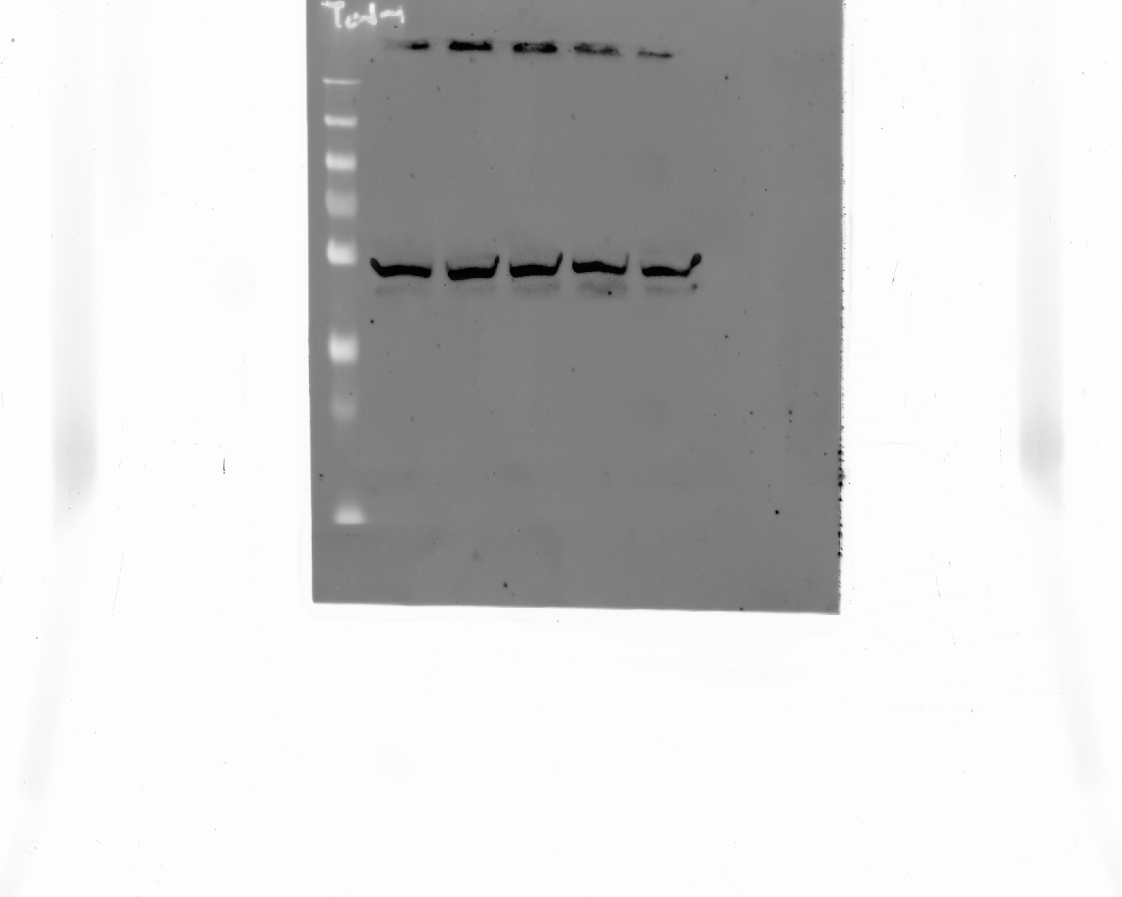

Supplement: Figure 3—source data 4. [file elife-106699-fig3-data4.zip › Figure 3ΓÇösource data 4 Original files for Western blot analysis displayed in Figure 3B./Tal-1 Jukat Abd-VHL.tif]

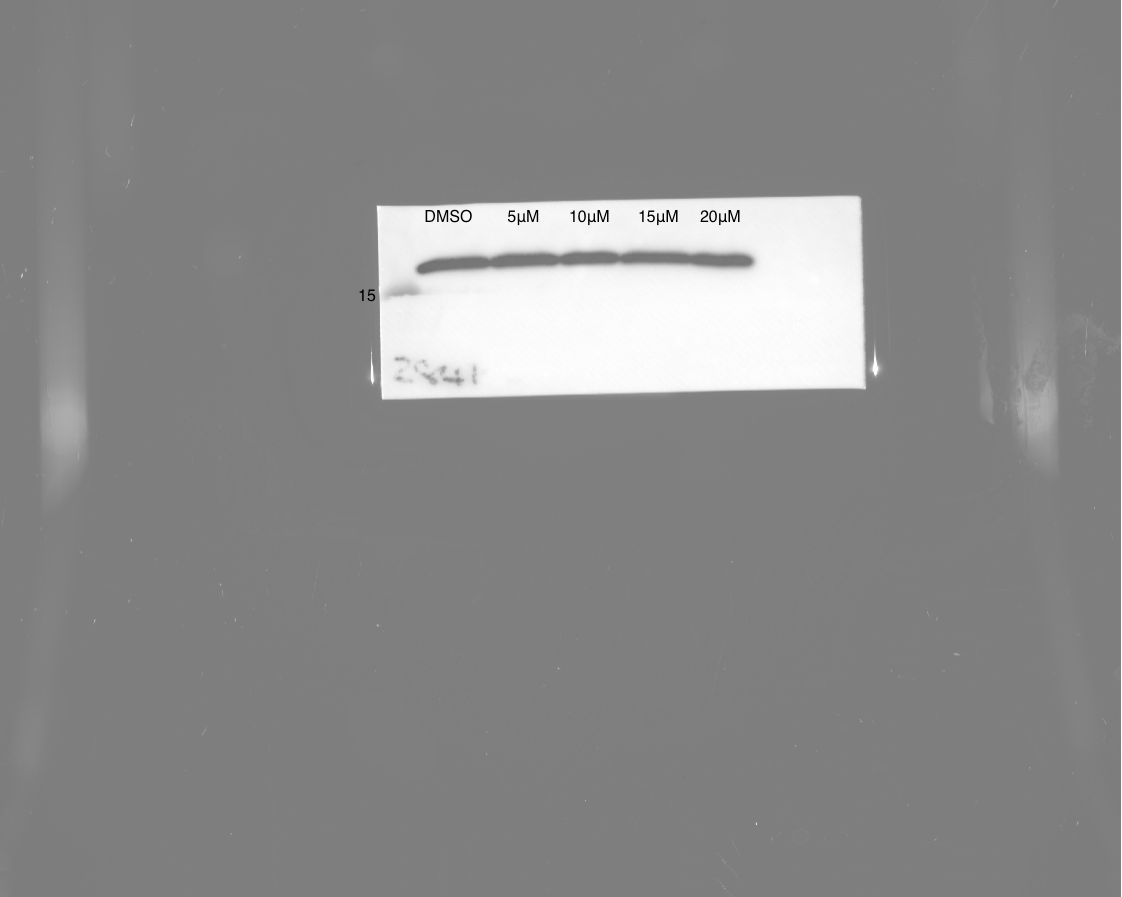

Supplement: Figure 3—source data 5. [file elife-106699-fig3-data5.zip › Figure 3ΓÇösource data 5 PDF files containing original western blots for Figure 3C, indicating the relevant bands and treatments./Raw data/Cyclophilin CCRF-CEM Abd-CRBN.tif]

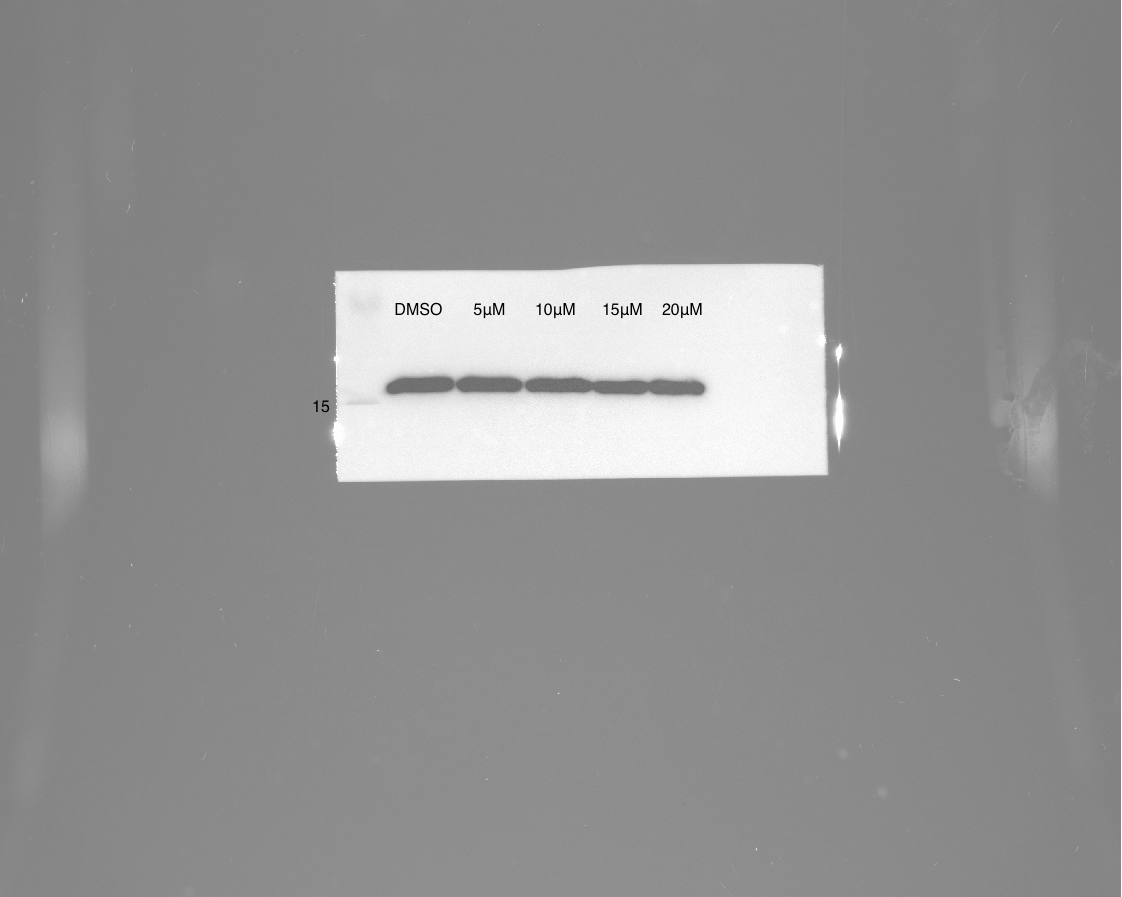

Supplement: Figure 3—source data 5. [file elife-106699-fig3-data5.zip › Figure 3ΓÇösource data 5 PDF files containing original western blots for Figure 3C, indicating the relevant bands and treatments./Raw data/Cyclophilin CCRF-CEM Abd-VHL.tif]

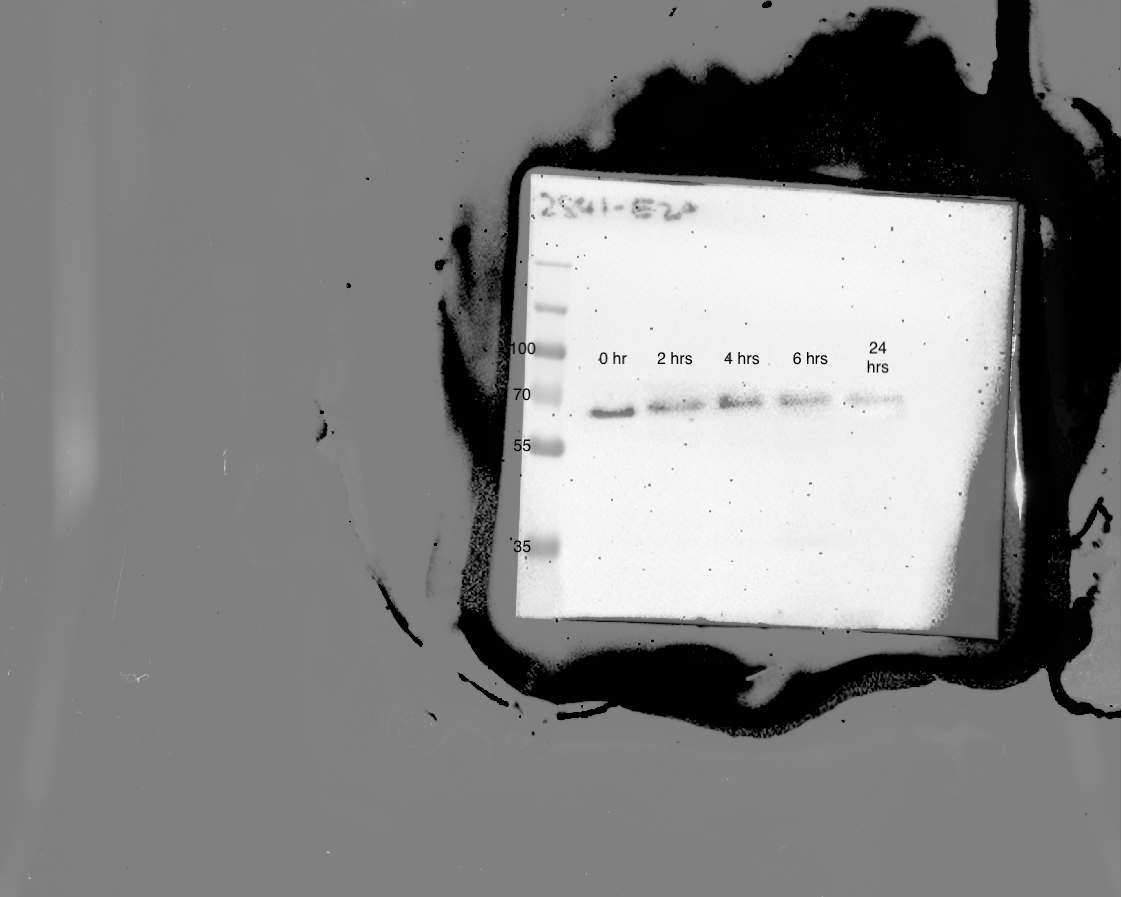

Supplement: Figure 3—source data 5. [file elife-106699-fig3-data5.zip › Figure 3ΓÇösource data 5 PDF files containing original western blots for Figure 3C, indicating the relevant bands and treatments./Raw data/E2A CCRF-CEM Abd-CRBN.tif]

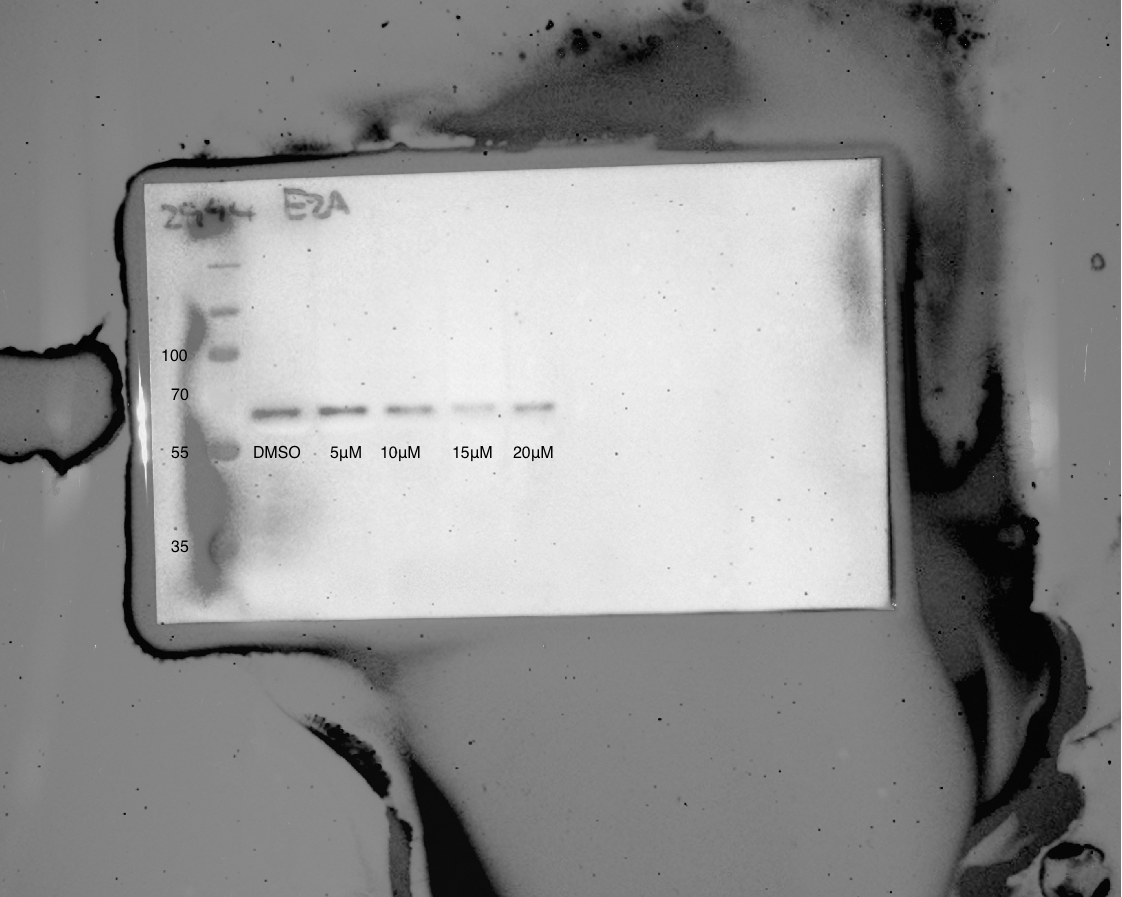

Supplement: Figure 3—source data 5. [file elife-106699-fig3-data5.zip › Figure 3ΓÇösource data 5 PDF files containing original western blots for Figure 3C, indicating the relevant bands and treatments./Raw data/E2A CCRF-CEM Abd-VHL.tif]

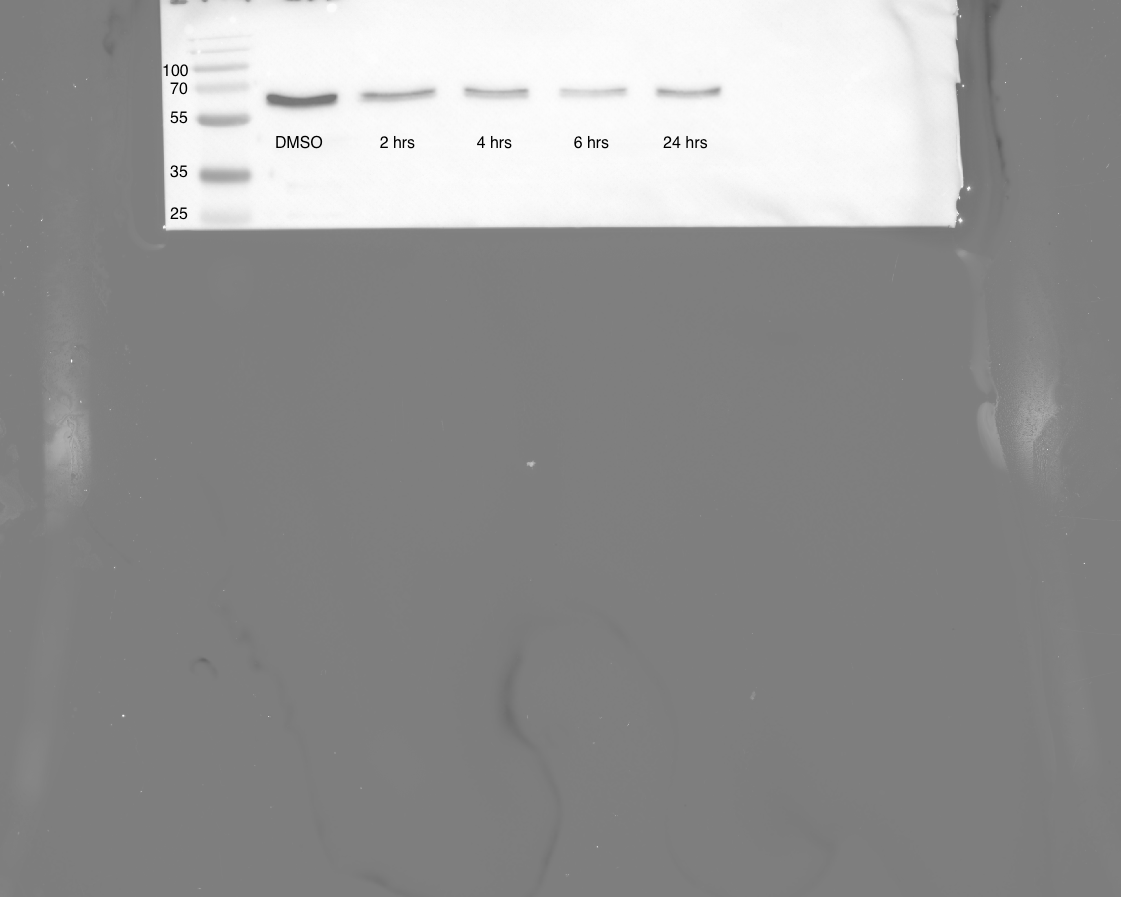

Supplement: Figure 3—source data 5. [file elife-106699-fig3-data5.zip › Figure 3ΓÇösource data 5 PDF files containing original western blots for Figure 3C, indicating the relevant bands and treatments./Raw data/E2A KOPT-K1 Abd-CRBN.tif]

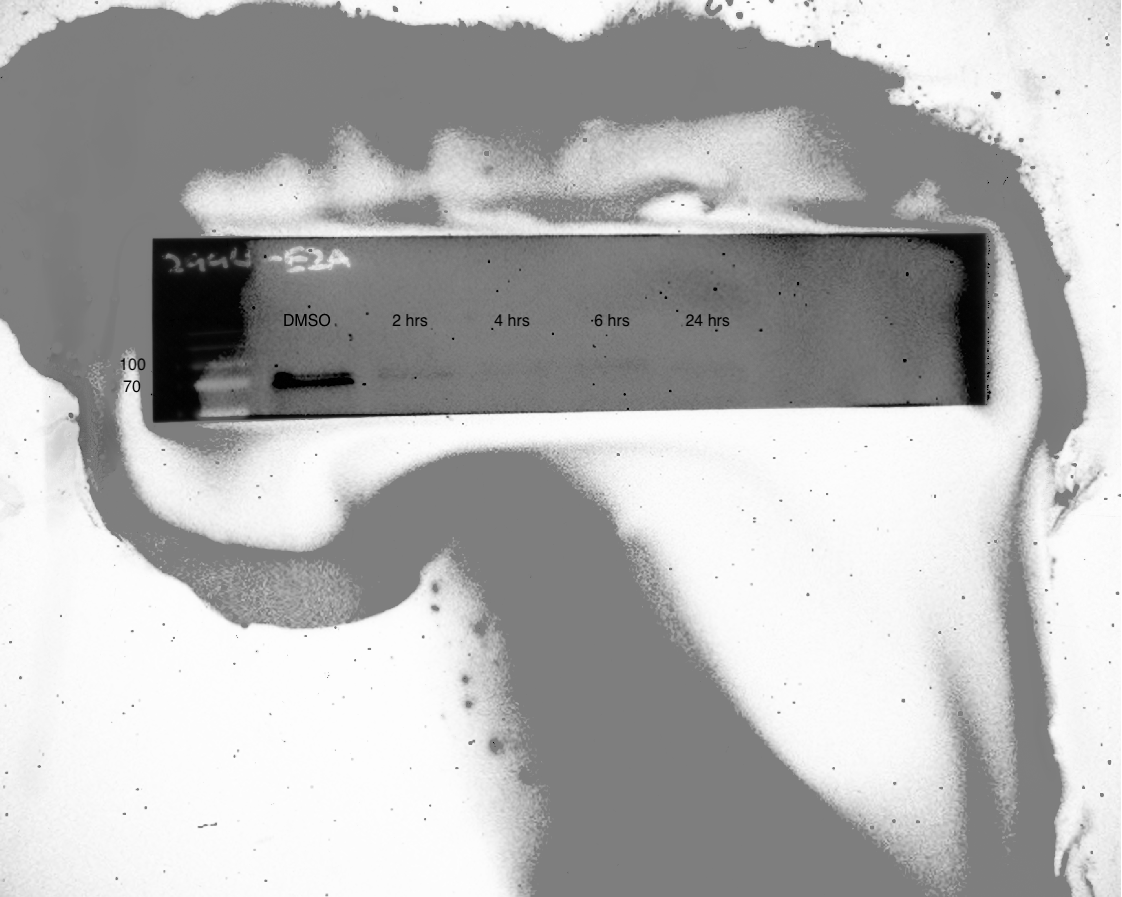

Supplement: Figure 3—source data 5. [file elife-106699-fig3-data5.zip › Figure 3ΓÇösource data 5 PDF files containing original western blots for Figure 3C, indicating the relevant bands and treatments./Raw data/E2A KOPT-K1 Abd-VHL.tif]

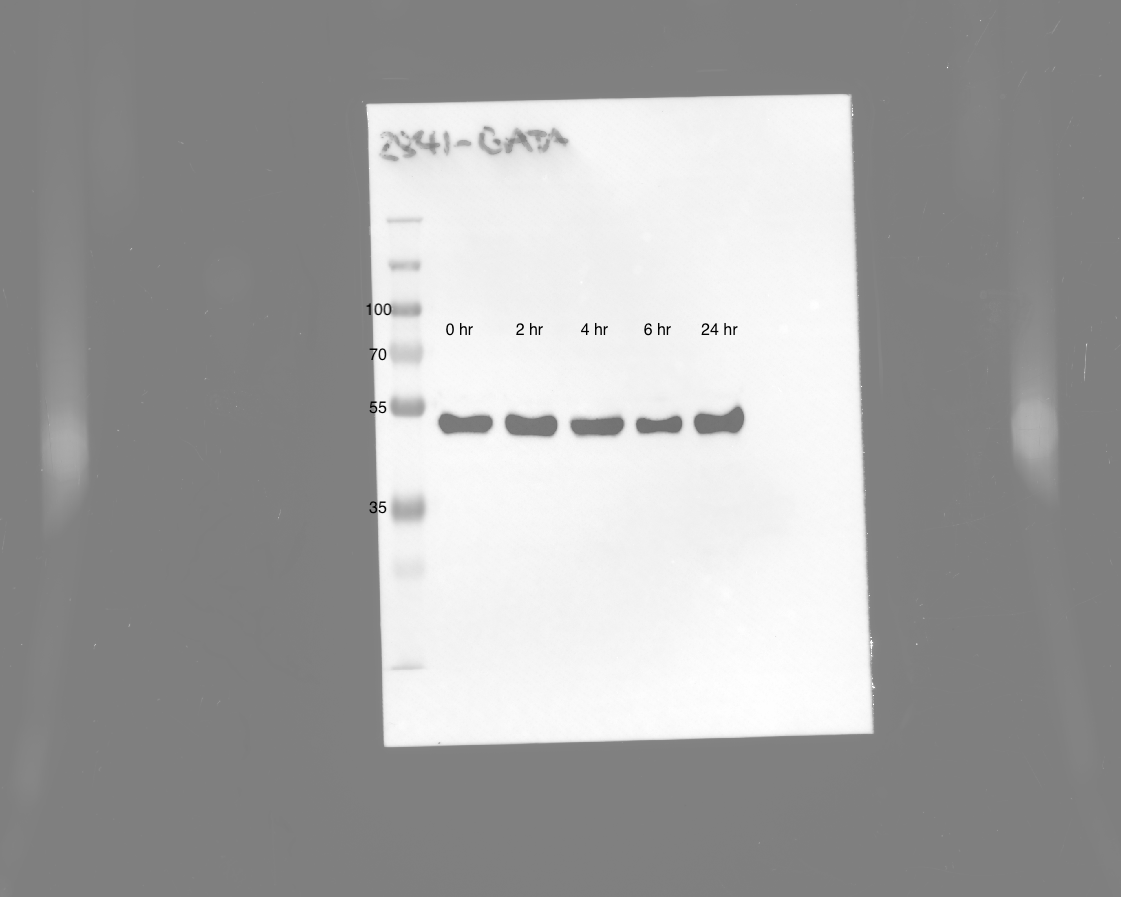

Supplement: Figure 3—source data 5. [file elife-106699-fig3-data5.zip › Figure 3ΓÇösource data 5 PDF files containing original western blots for Figure 3C, indicating the relevant bands and treatments./Raw data/GATA3 CCRF-CEM Abd-CRBN.tif]

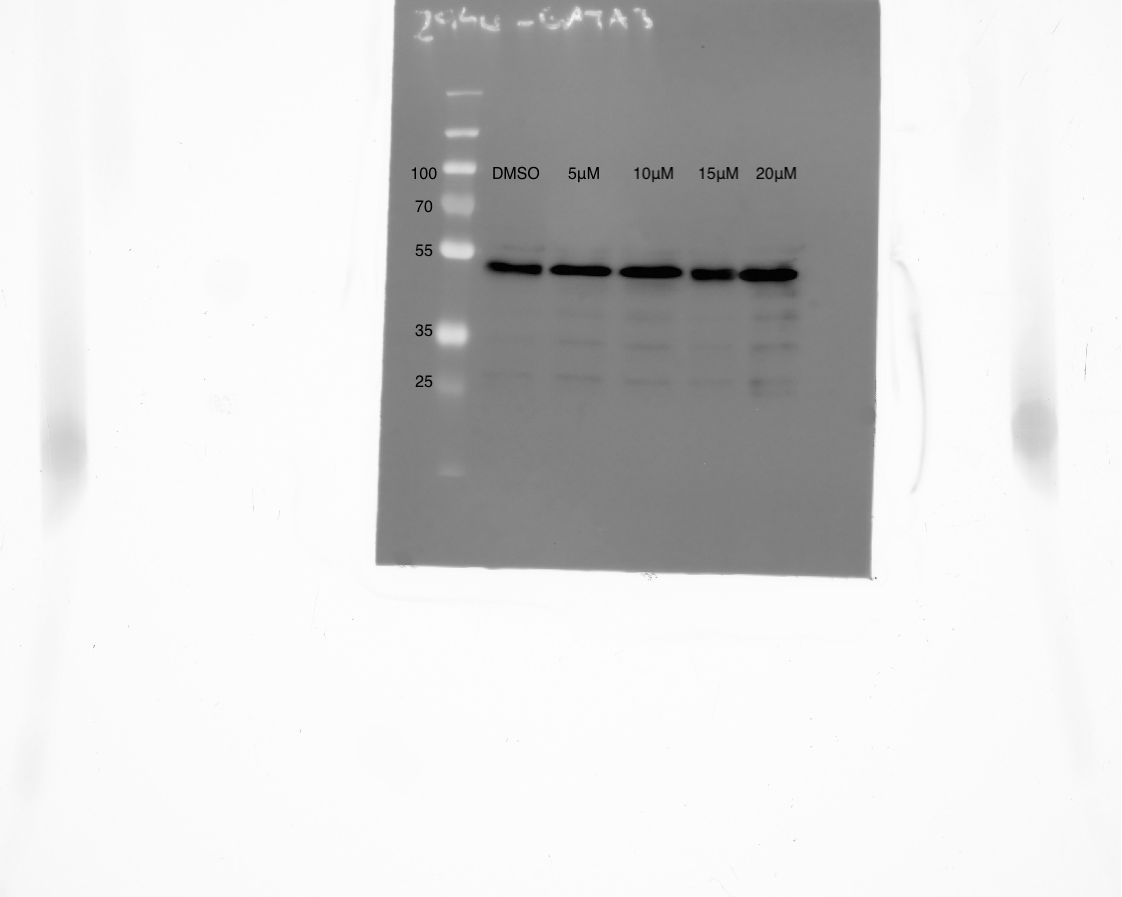

Supplement: Figure 3—source data 5. [file elife-106699-fig3-data5.zip › Figure 3ΓÇösource data 5 PDF files containing original western blots for Figure 3C, indicating the relevant bands and treatments./Raw data/GATA3 CCRF-CEM Abd-VHL.tif]

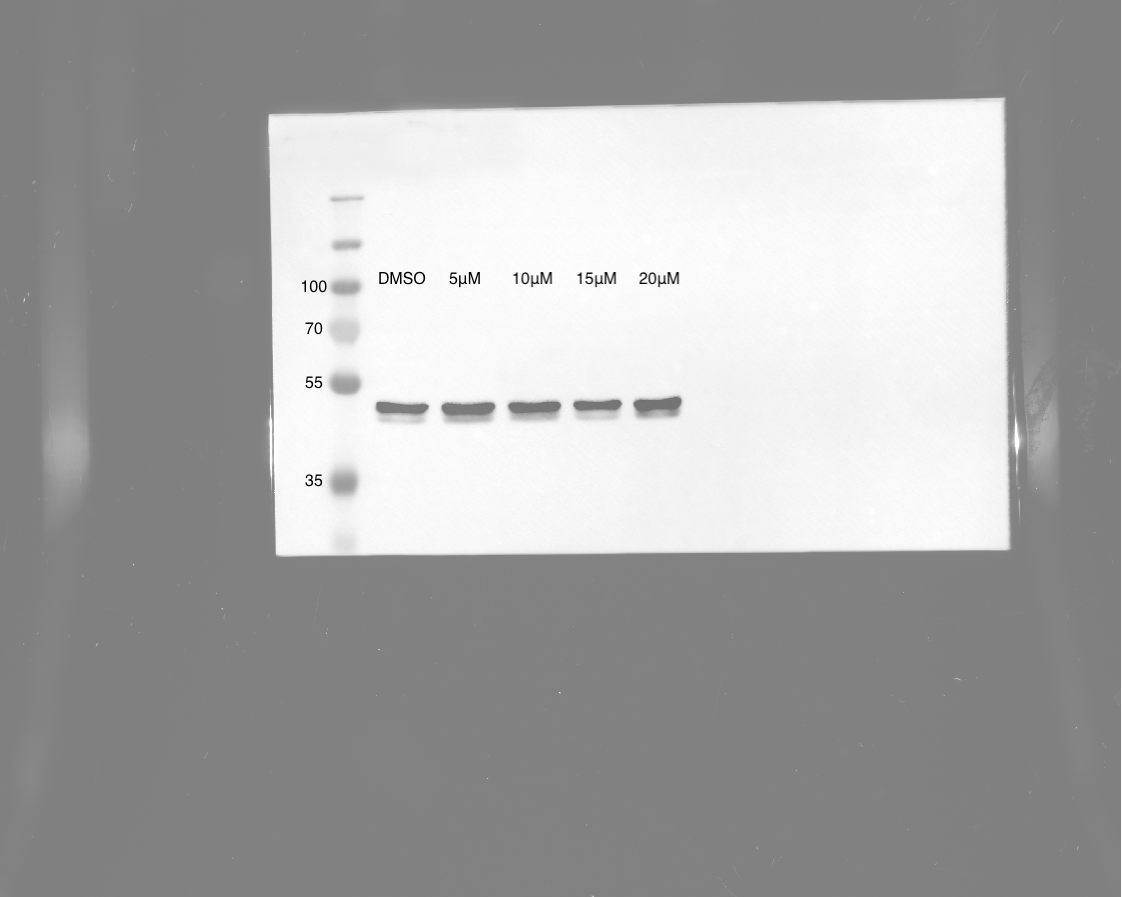

Supplement: Figure 3—source data 5. [file elife-106699-fig3-data5.zip › Figure 3ΓÇösource data 5 PDF files containing original western blots for Figure 3C, indicating the relevant bands and treatments./Raw data/LDB1 CCRF-CEM Abd-CRBN.tif]

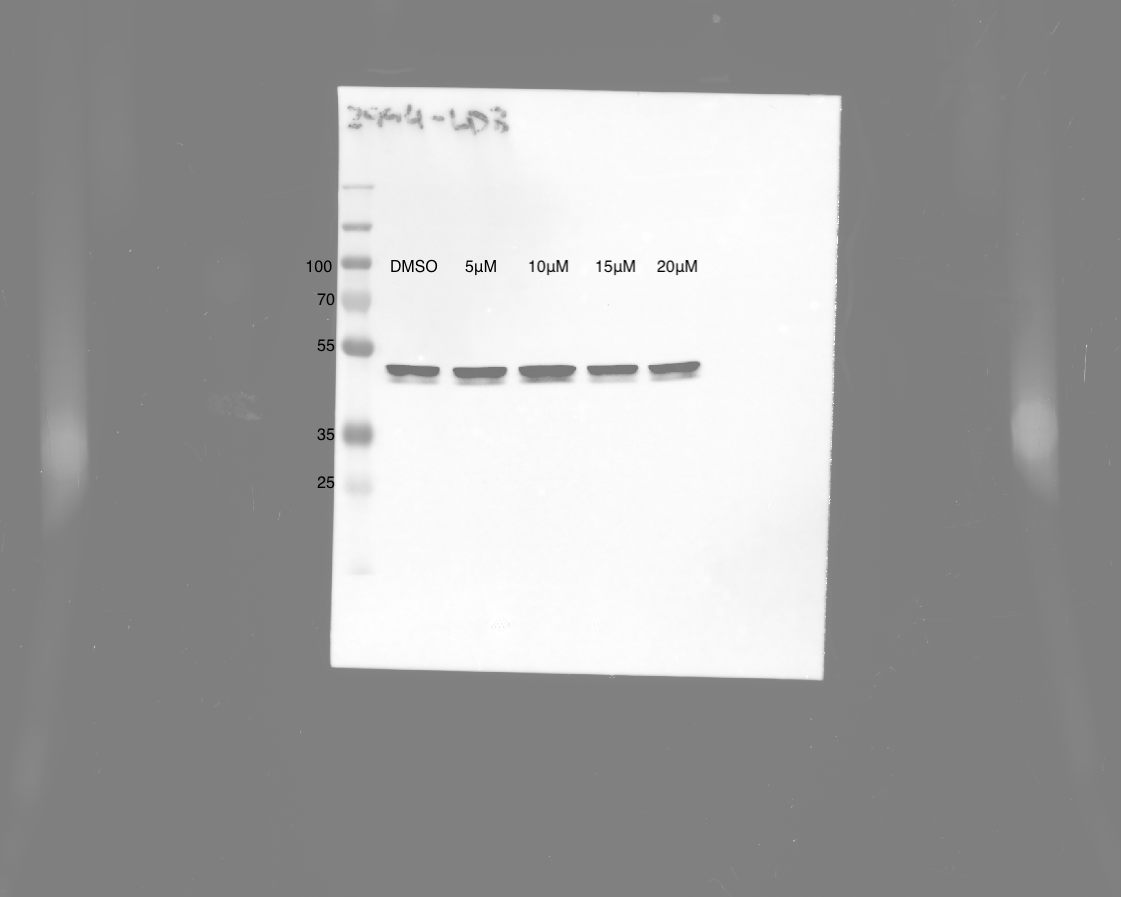

Supplement: Figure 3—source data 5. [file elife-106699-fig3-data5.zip › Figure 3ΓÇösource data 5 PDF files containing original western blots for Figure 3C, indicating the relevant bands and treatments./Raw data/LDB1 CCRF-CEM Abd-VHL.tif]

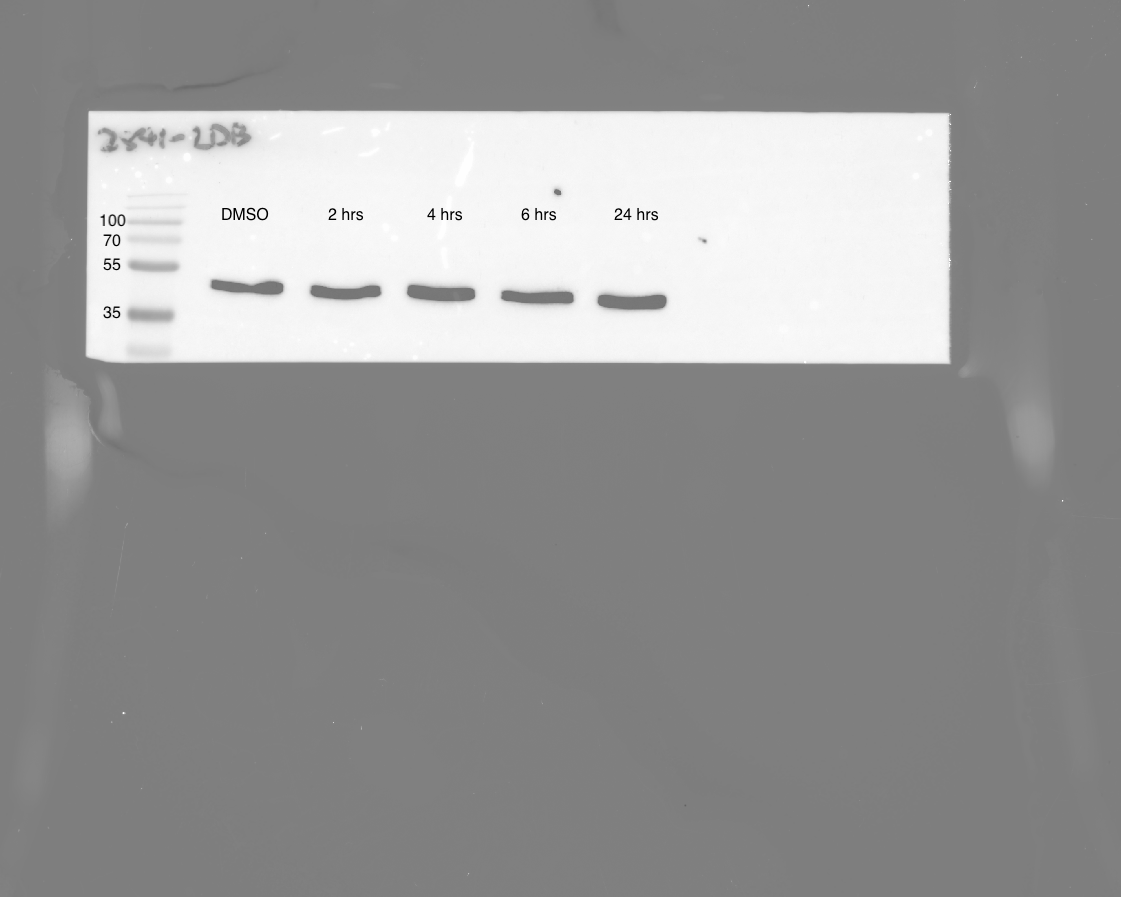

Supplement: Figure 3—source data 5. [file elife-106699-fig3-data5.zip › Figure 3ΓÇösource data 5 PDF files containing original western blots for Figure 3C, indicating the relevant bands and treatments./Raw data/LDB1 KOPT-K1 Abd-CRBN.tif]

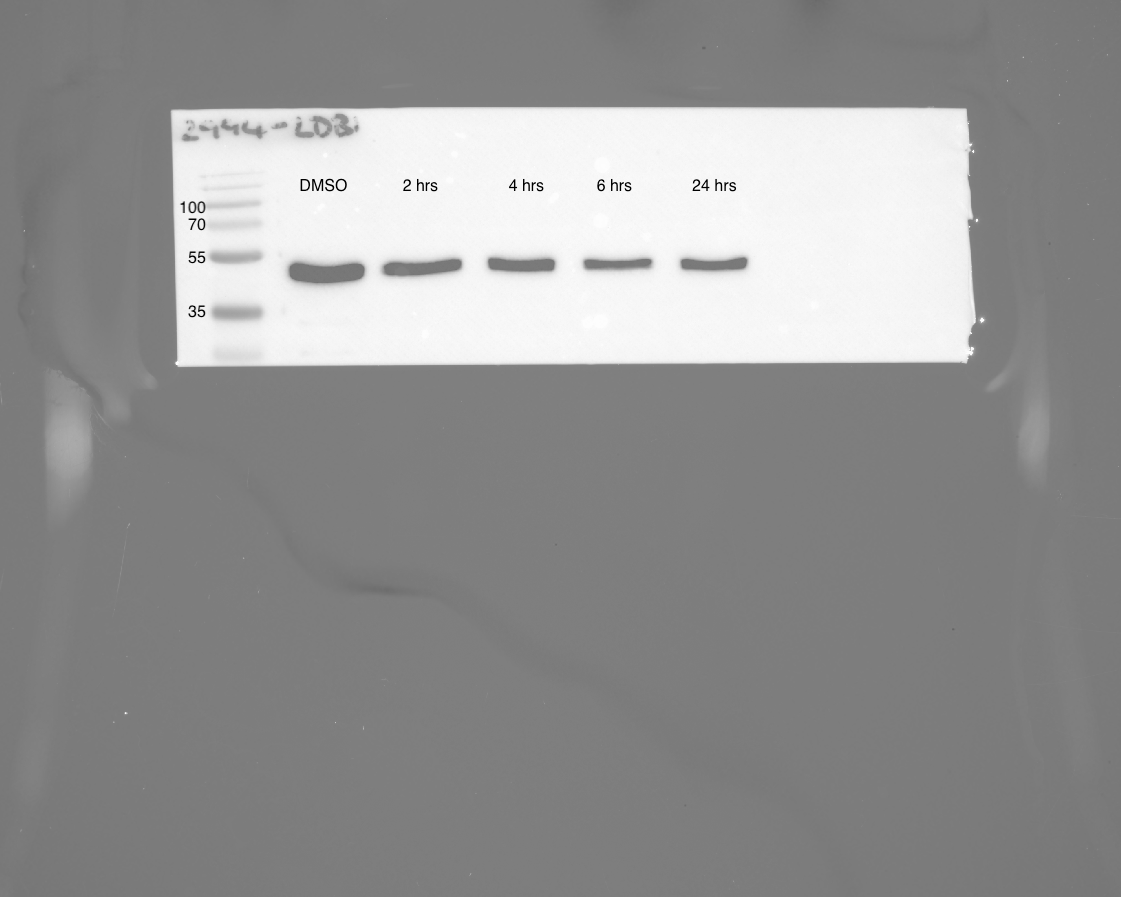

Supplement: Figure 3—source data 5. [file elife-106699-fig3-data5.zip › Figure 3ΓÇösource data 5 PDF files containing original western blots for Figure 3C, indicating the relevant bands and treatments./Raw data/LDB1 KOPT-K1 Abd-VHL.tif]

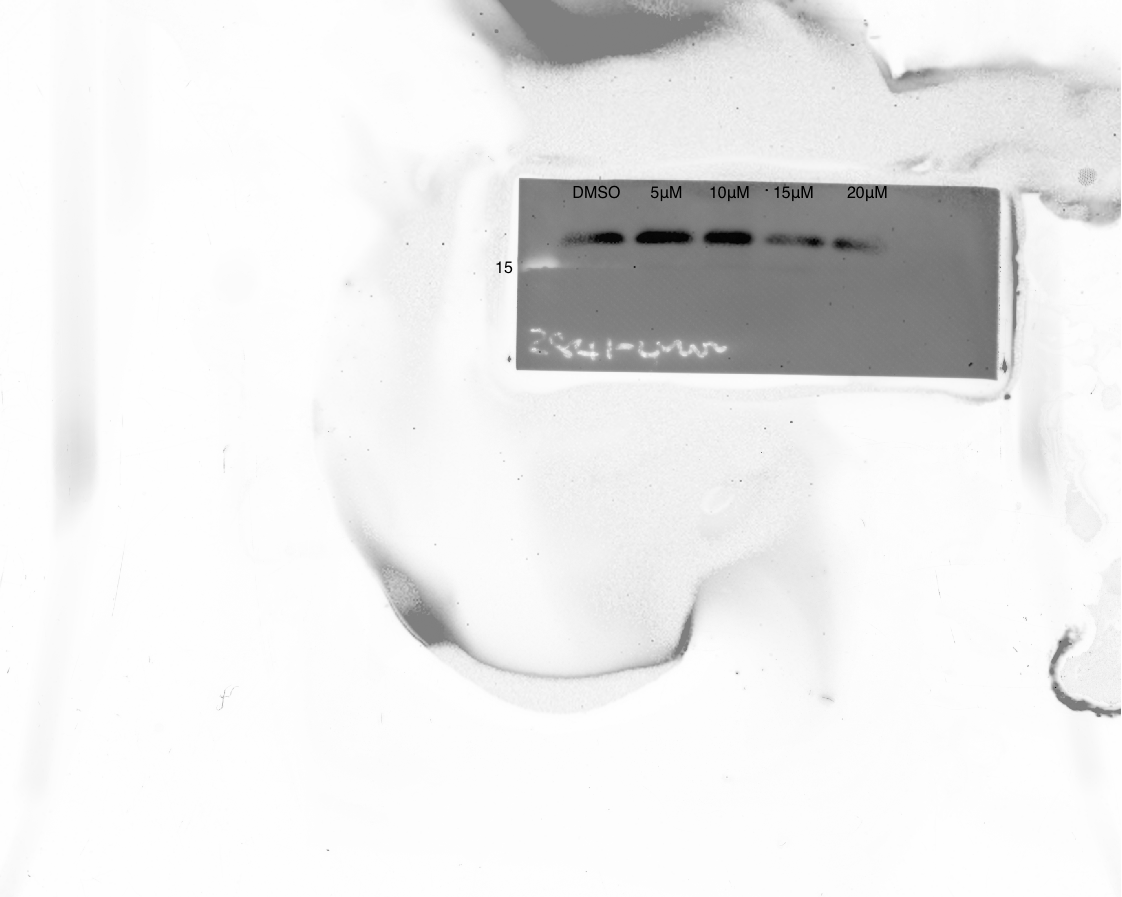

Supplement: Figure 3—source data 5. [file elife-106699-fig3-data5.zip › Figure 3ΓÇösource data 5 PDF files containing original western blots for Figure 3C, indicating the relevant bands and treatments./Raw data/LMO2 CCRF-CEM Abd-CRBN.tif]

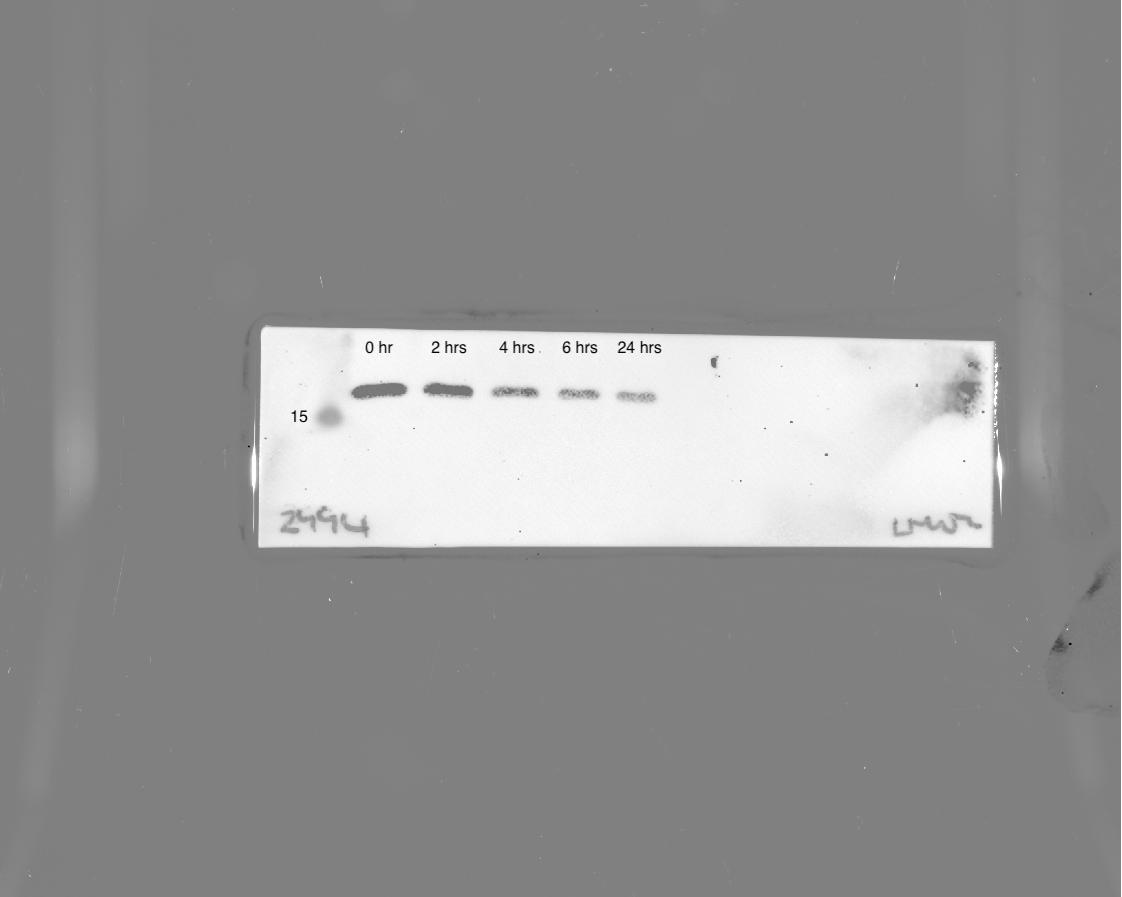

Supplement: Figure 3—source data 5. [file elife-106699-fig3-data5.zip › Figure 3ΓÇösource data 5 PDF files containing original western blots for Figure 3C, indicating the relevant bands and treatments./Raw data/LMO2 CCRF-CEM Abd-VHL.tif]

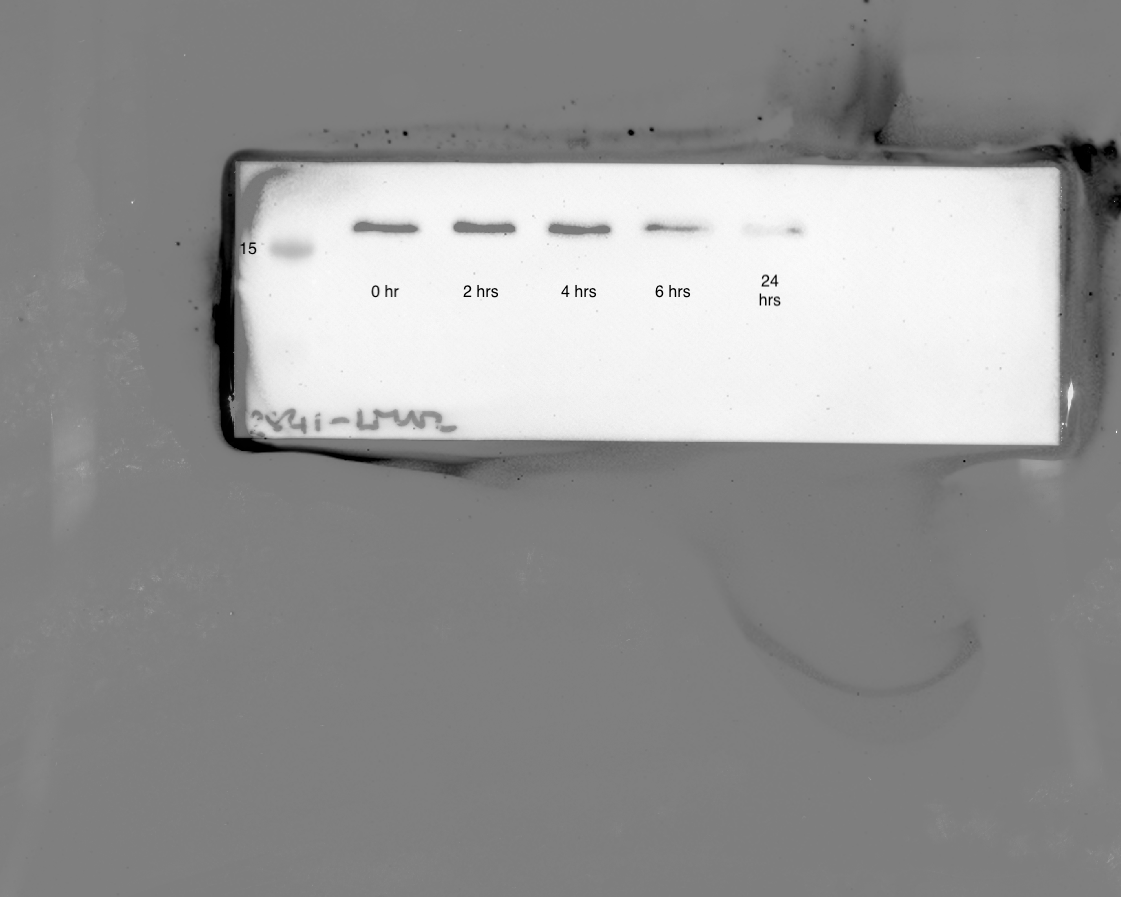

Supplement: Figure 3—source data 5. [file elife-106699-fig3-data5.zip › Figure 3ΓÇösource data 5 PDF files containing original western blots for Figure 3C, indicating the relevant bands and treatments./Raw data/LMO2 KOPT-K1 Abd-CRBN.tif]

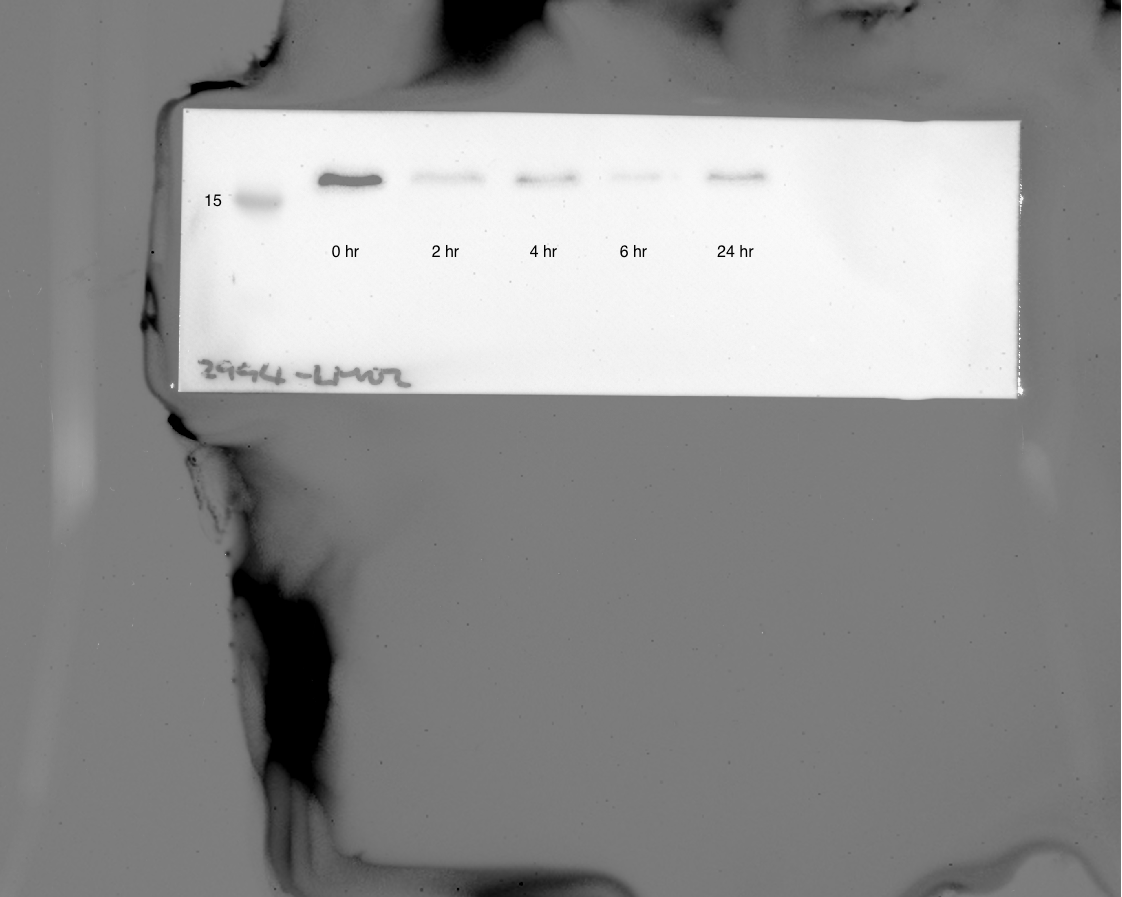

Supplement: Figure 3—source data 5. [file elife-106699-fig3-data5.zip › Figure 3ΓÇösource data 5 PDF files containing original western blots for Figure 3C, indicating the relevant bands and treatments./Raw data/LMO2 KOPT-K1 Abd-VHL.tif]

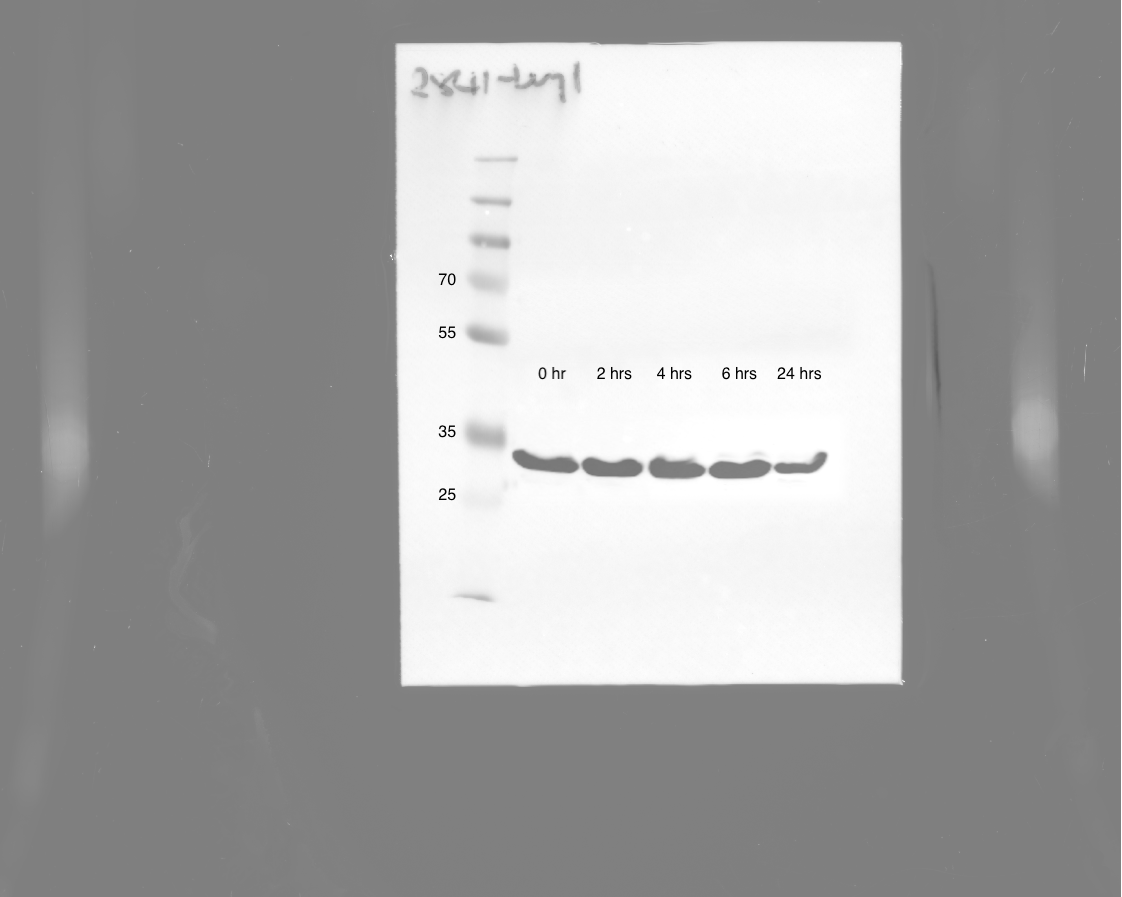

Supplement: Figure 3—source data 5. [file elife-106699-fig3-data5.zip › Figure 3ΓÇösource data 5 PDF files containing original western blots for Figure 3C, indicating the relevant bands and treatments./Raw data/Lyl1 CCRF-CEM Abd-CRBN.tif]

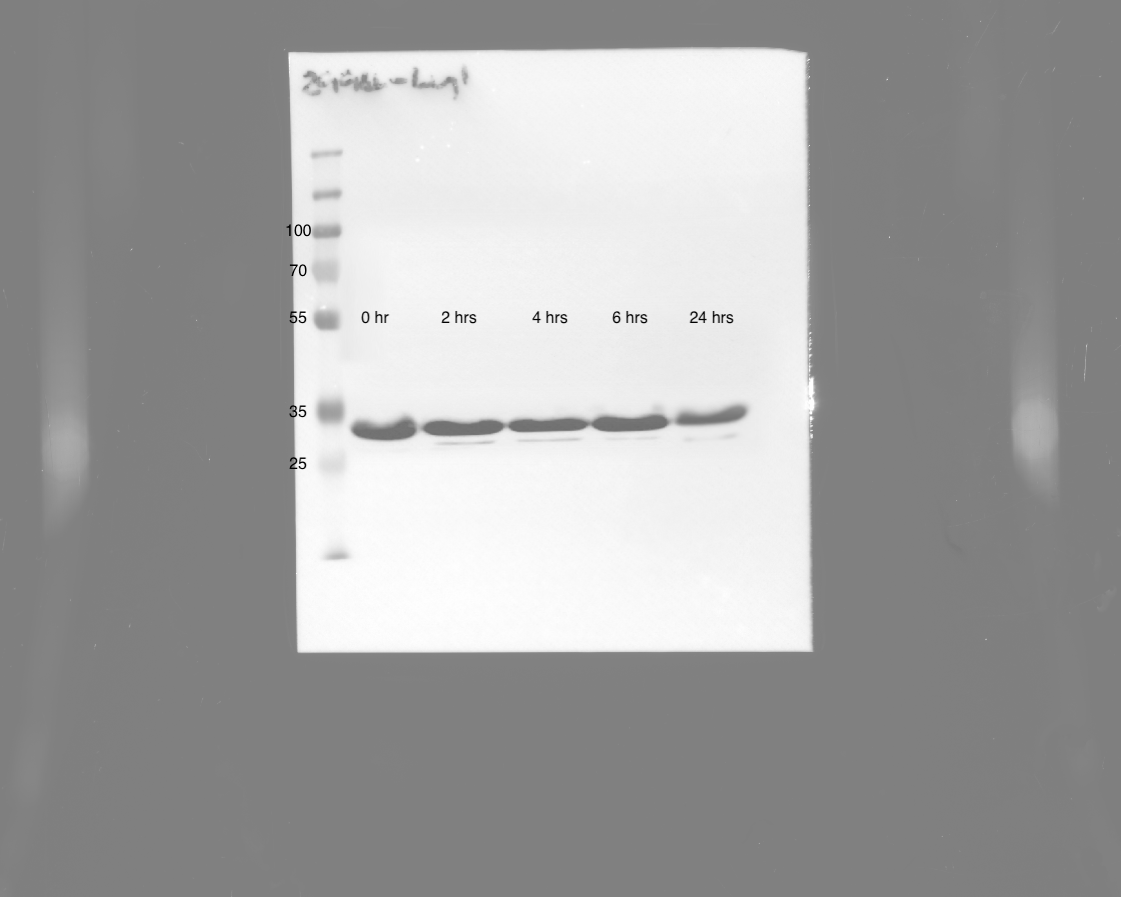

Supplement: Figure 3—source data 5. [file elife-106699-fig3-data5.zip › Figure 3ΓÇösource data 5 PDF files containing original western blots for Figure 3C, indicating the relevant bands and treatments./Raw data/Lyl1 CCRF-CEM Abd-VHL.tif]

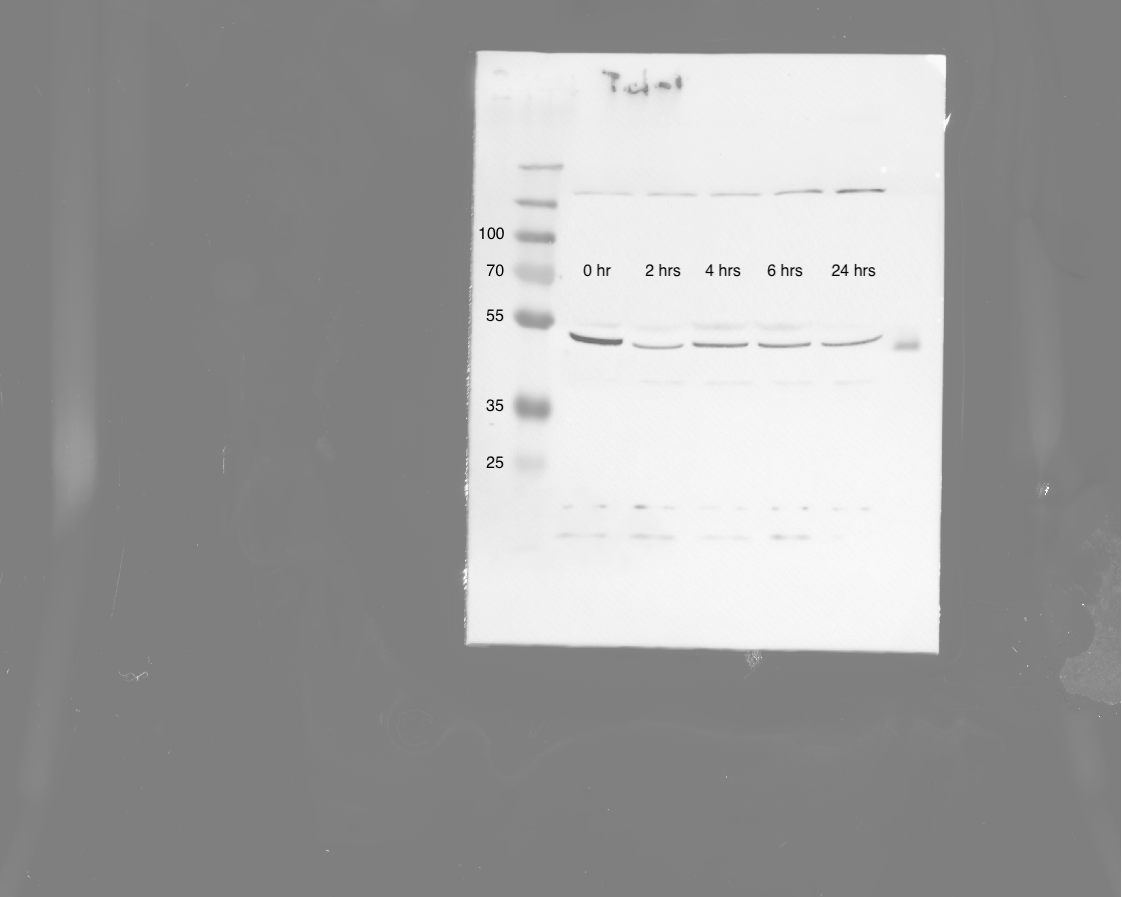

Supplement: Figure 3—source data 5. [file elife-106699-fig3-data5.zip › Figure 3ΓÇösource data 5 PDF files containing original western blots for Figure 3C, indicating the relevant bands and treatments./Raw data/Tal-1 CCRF-CEM Abd-CRBN.tif]

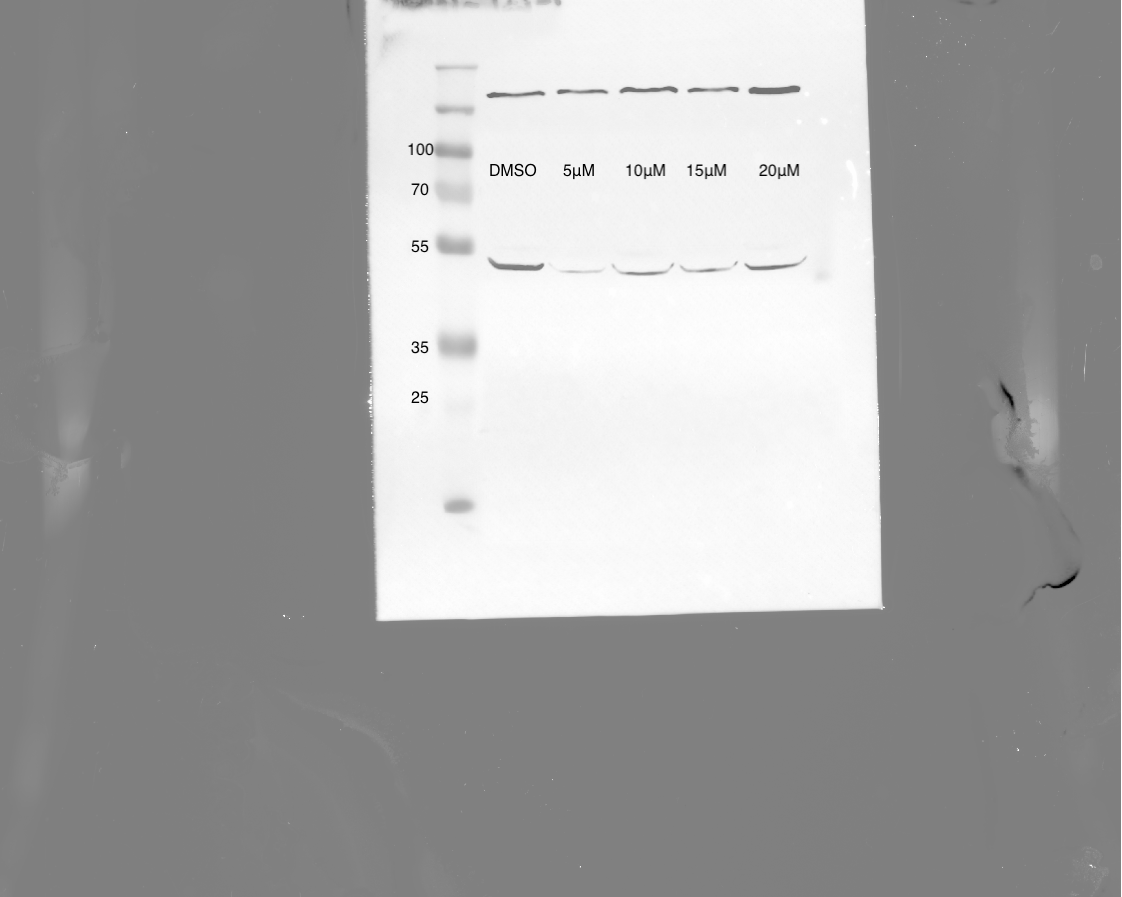

Supplement: Figure 3—source data 5. [file elife-106699-fig3-data5.zip › Figure 3ΓÇösource data 5 PDF files containing original western blots for Figure 3C, indicating the relevant bands and treatments./Raw data/Tal-1 CCRF-CEM Abd-VHL.tif]

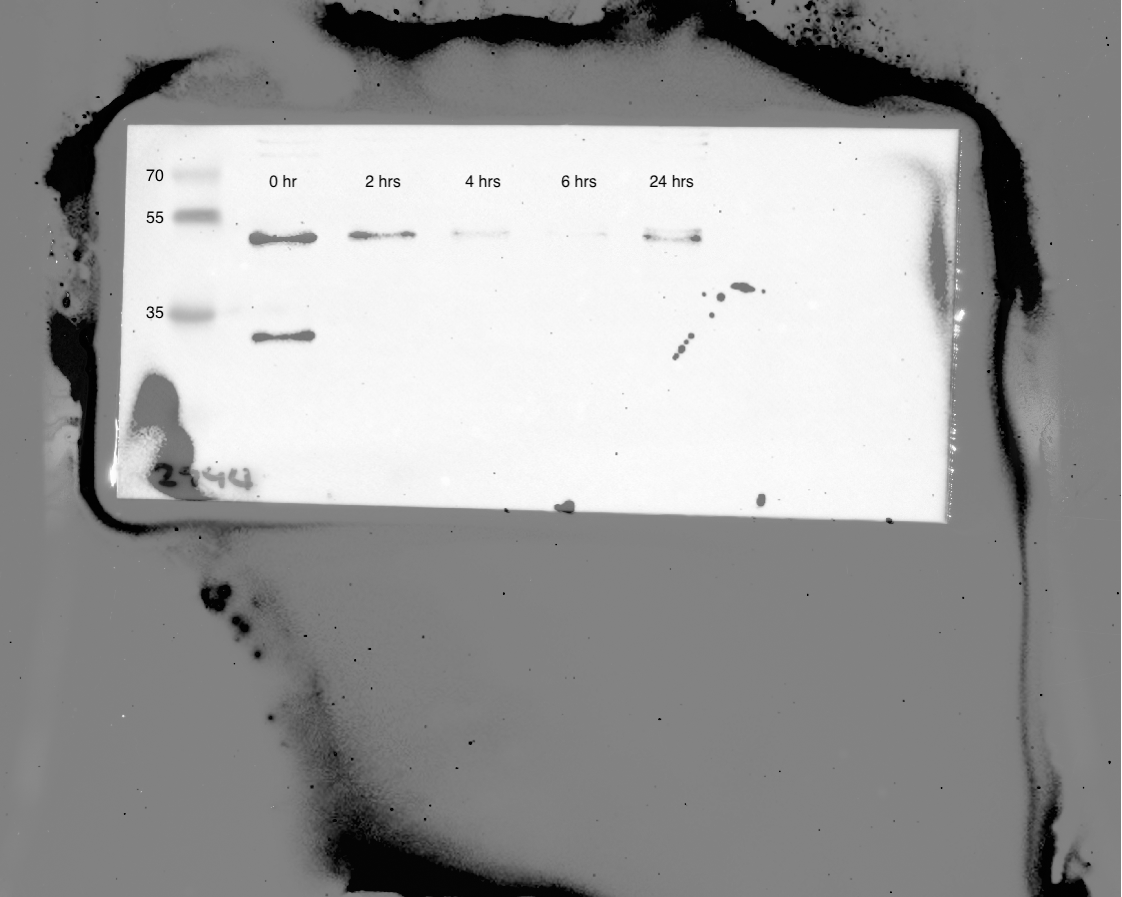

Supplement: Figure 3—source data 5. [file elife-106699-fig3-data5.zip › Figure 3ΓÇösource data 5 PDF files containing original western blots for Figure 3C, indicating the relevant bands and treatments./Raw data/Tal-1 KOPT-K1 Abd-VHL.tif]

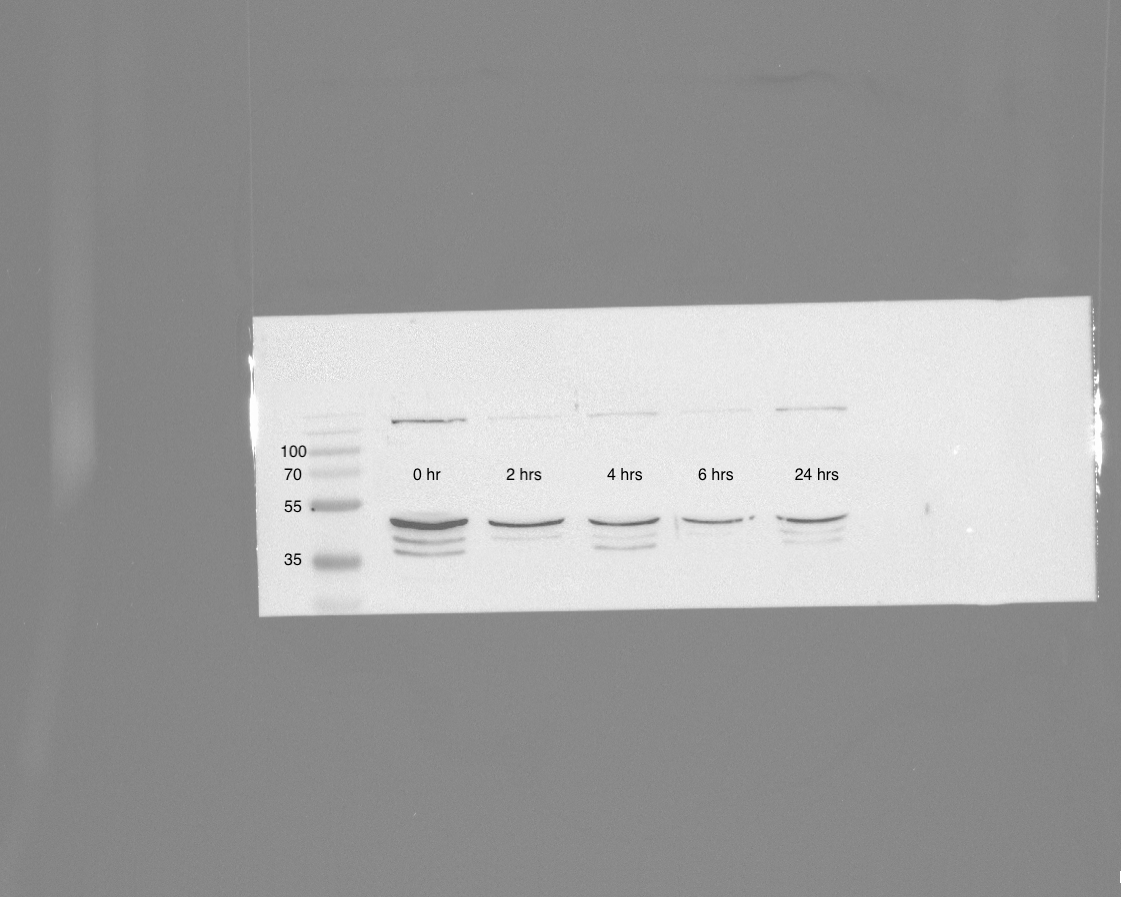

Supplement: Figure 3—source data 5. [file elife-106699-fig3-data5.zip › Figure 3ΓÇösource data 5 PDF files containing original western blots for Figure 3C, indicating the relevant bands and treatments./Raw data/Tal1 KOPT-K1 Abd-CRBN.tif]

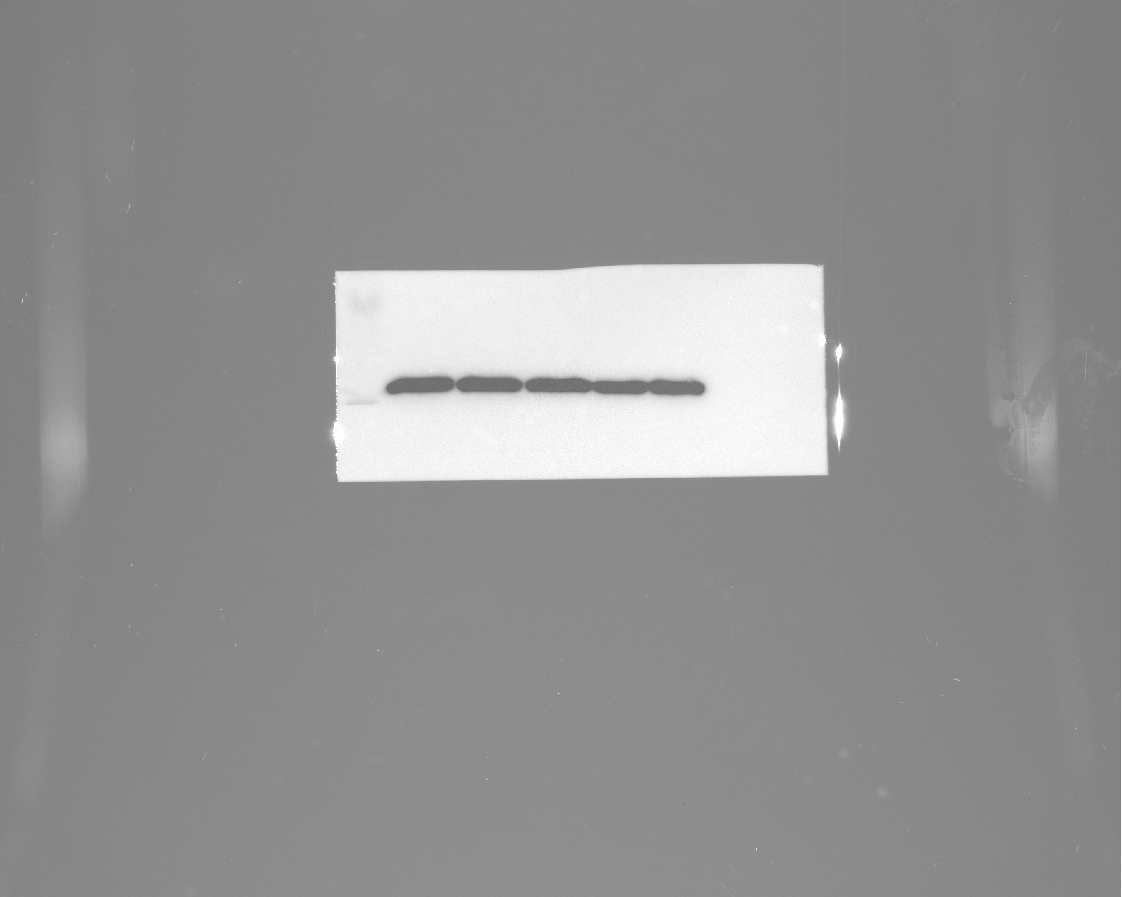

Supplement: Figure 3—source data 6. [file elife-106699-fig3-data6.zip › Figure 3ΓÇösource data 6 Original files for Western blot analysis displayed in Figure 3C./Cyclophilin CCRF Abd-VHL.tif]

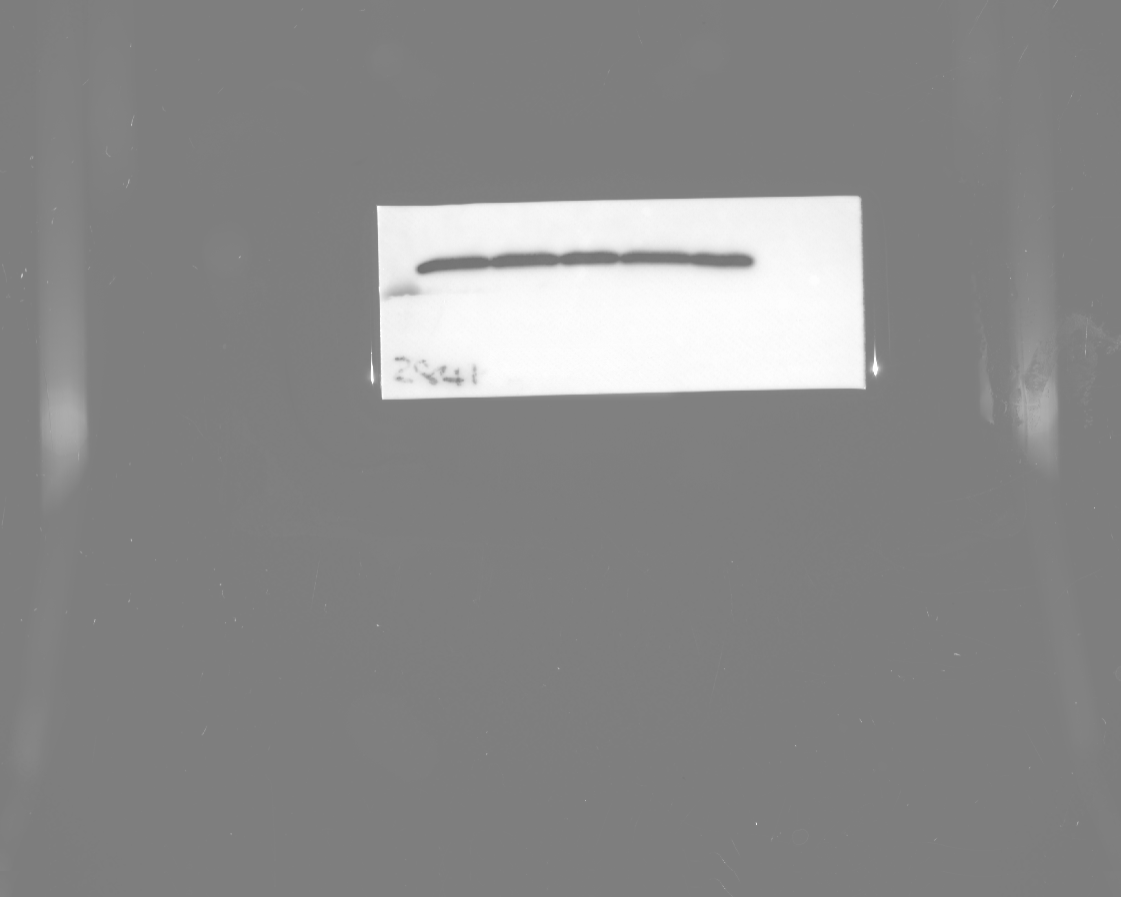

Supplement: Figure 3—source data 6. [file elife-106699-fig3-data6.zip › Figure 3ΓÇösource data 6 Original files for Western blot analysis displayed in Figure 3C./Cyclophilin CCRF-CEM Abd-CRBN.tif]

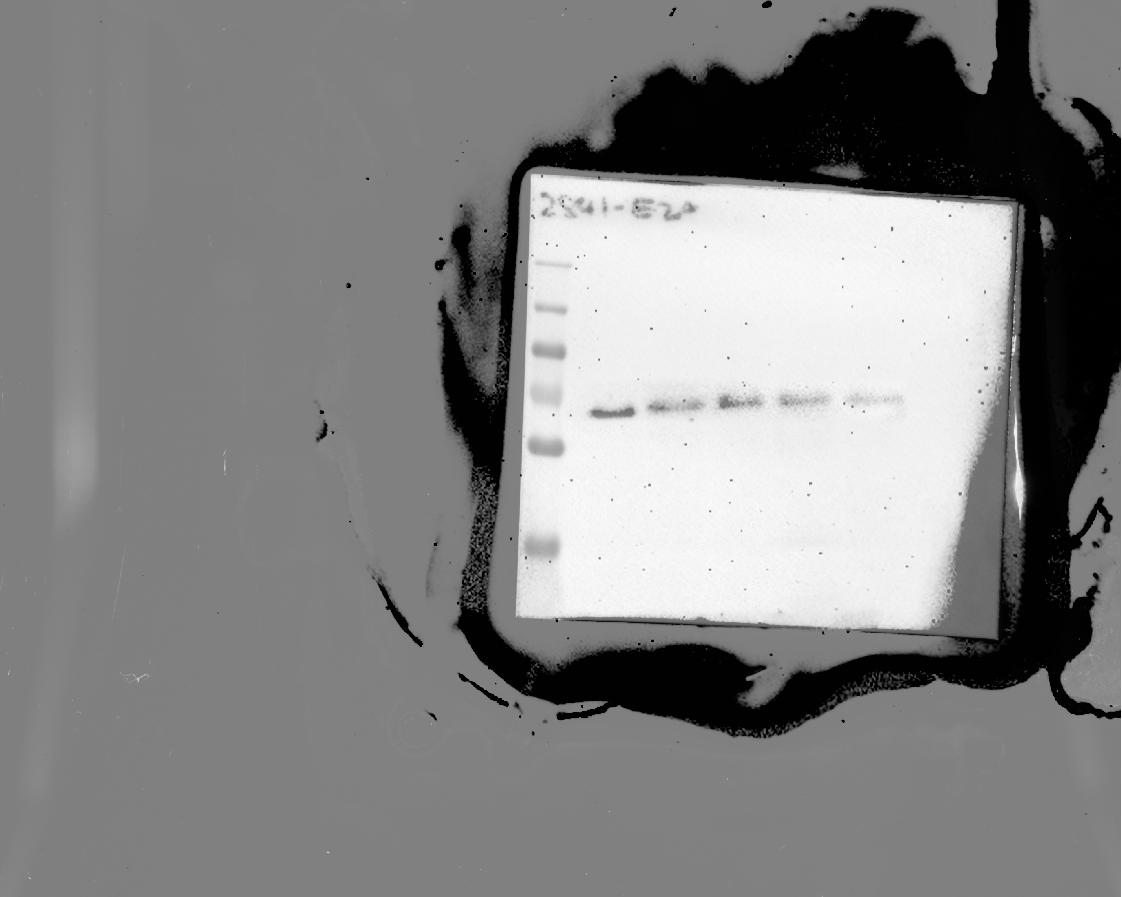

Supplement: Figure 3—source data 6. [file elife-106699-fig3-data6.zip › Figure 3ΓÇösource data 6 Original files for Western blot analysis displayed in Figure 3C./E2A CCRF-CEM Abd-CRBN.tif]

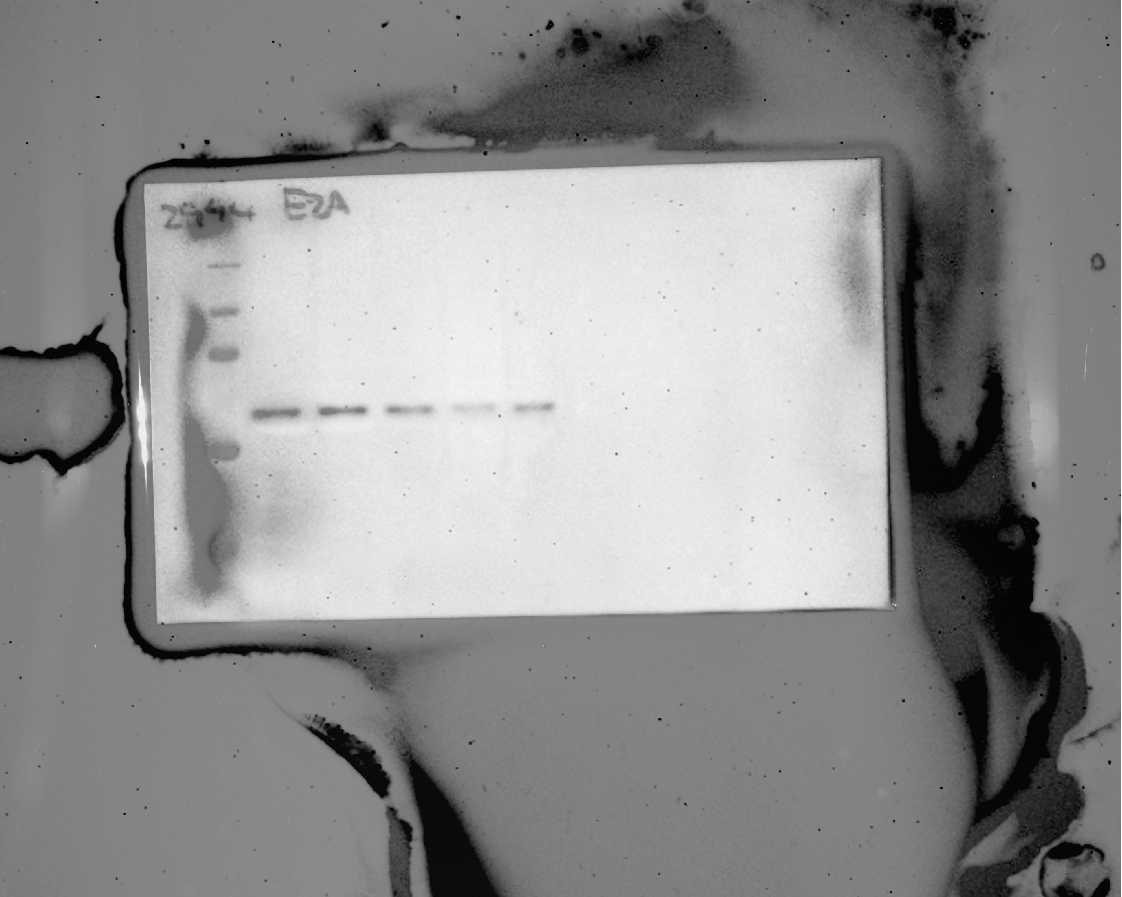

Supplement: Figure 3—source data 6. [file elife-106699-fig3-data6.zip › Figure 3ΓÇösource data 6 Original files for Western blot analysis displayed in Figure 3C./E2A CCRF-CEM Abd-VHL.tif]

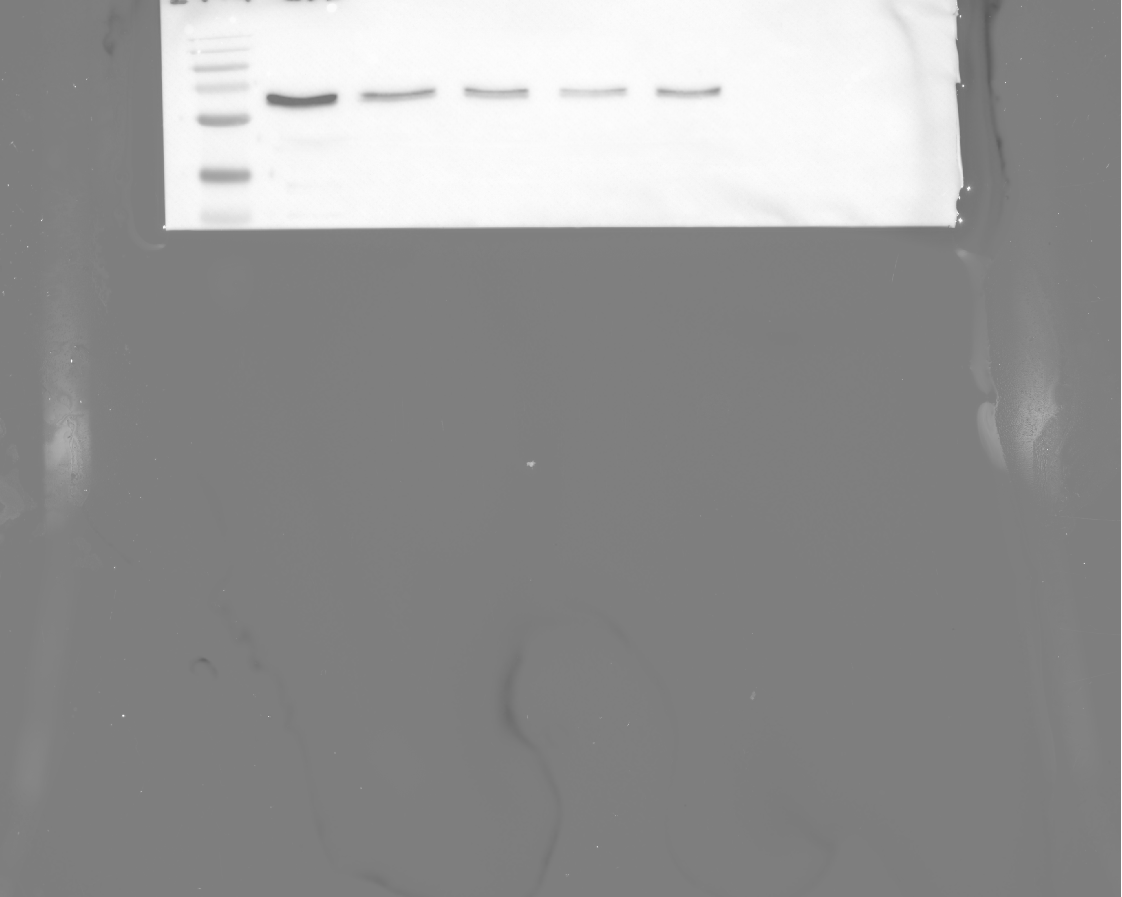

Supplement: Figure 3—source data 6. [file elife-106699-fig3-data6.zip › Figure 3ΓÇösource data 6 Original files for Western blot analysis displayed in Figure 3C./E2A KOPT-K1 Abd-CRBN.tif]

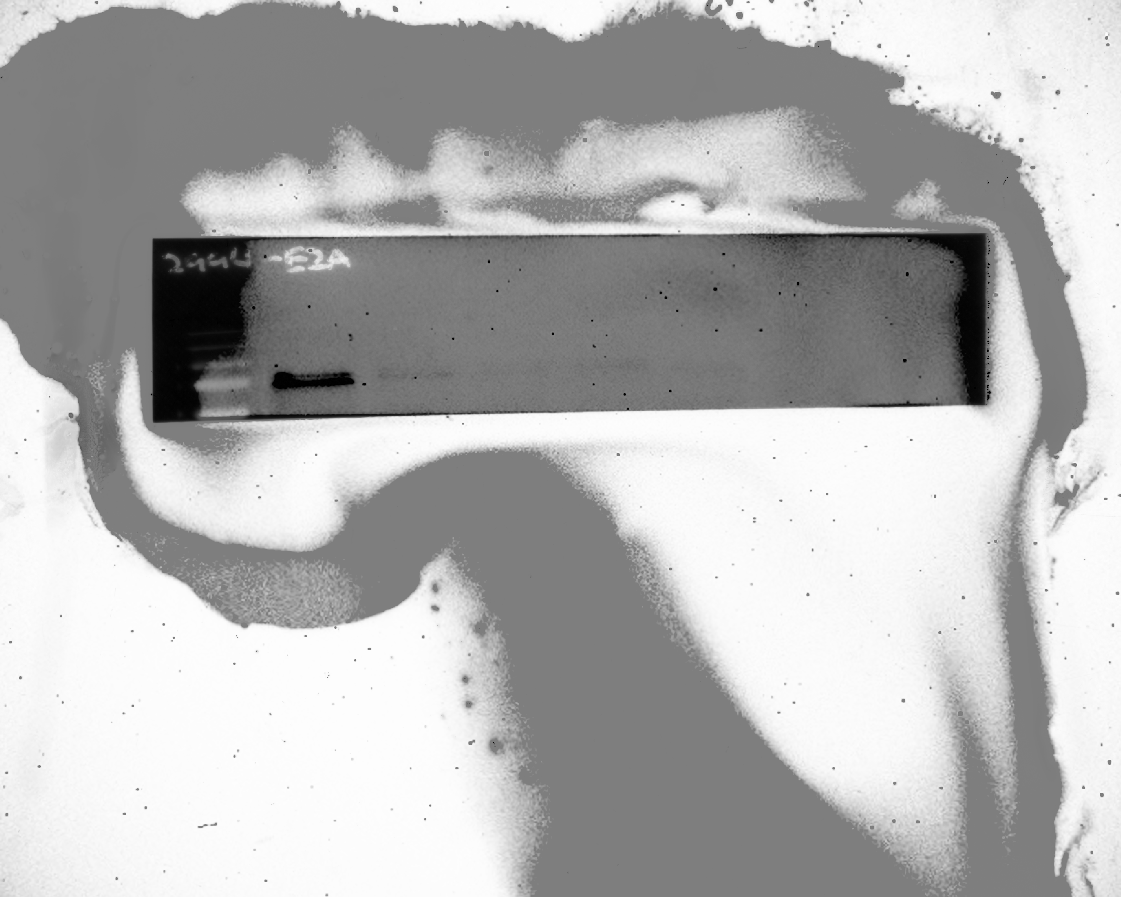

Supplement: Figure 3—source data 6. [file elife-106699-fig3-data6.zip › Figure 3ΓÇösource data 6 Original files for Western blot analysis displayed in Figure 3C./E2A KOPT-K1 Abd-VHL.tif]

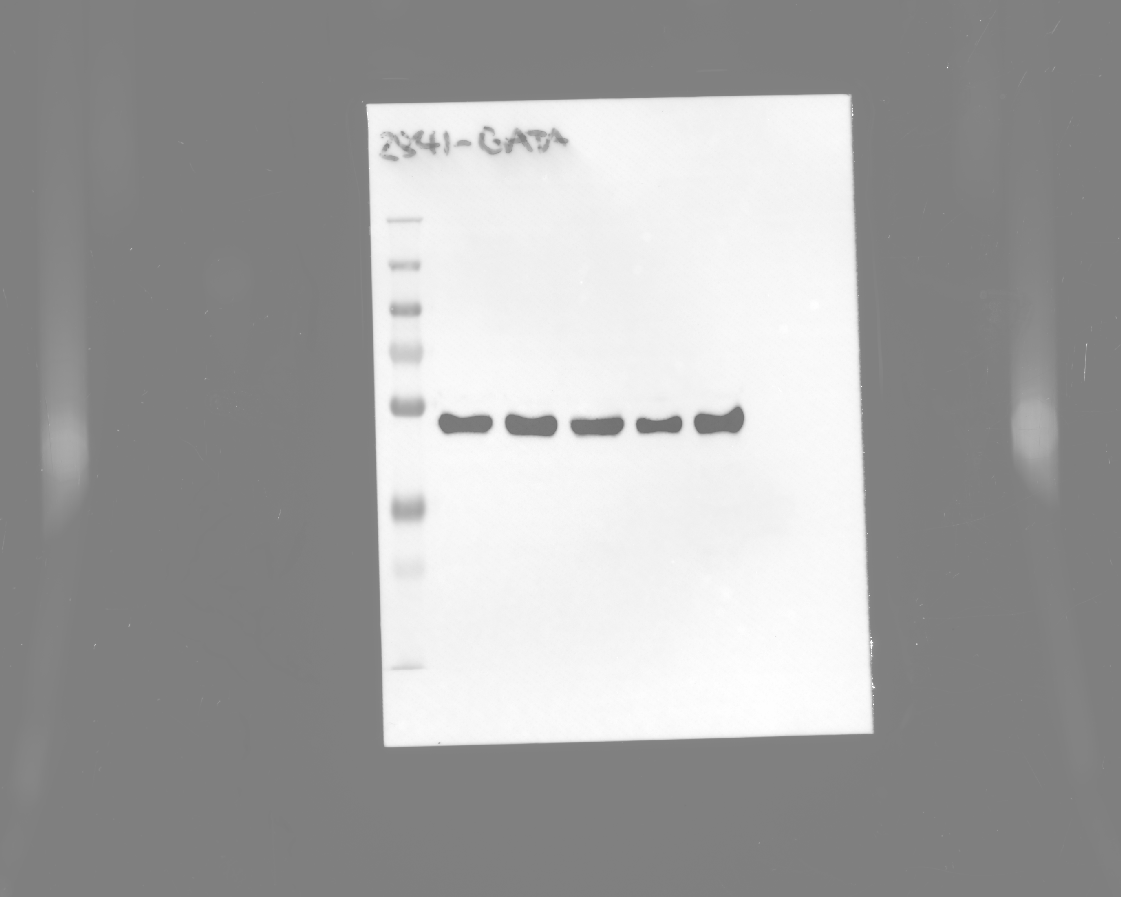

Supplement: Figure 3—source data 6. [file elife-106699-fig3-data6.zip › Figure 3ΓÇösource data 6 Original files for Western blot analysis displayed in Figure 3C./GATA3 CCRF-CEM Abd-CRBN.tif]

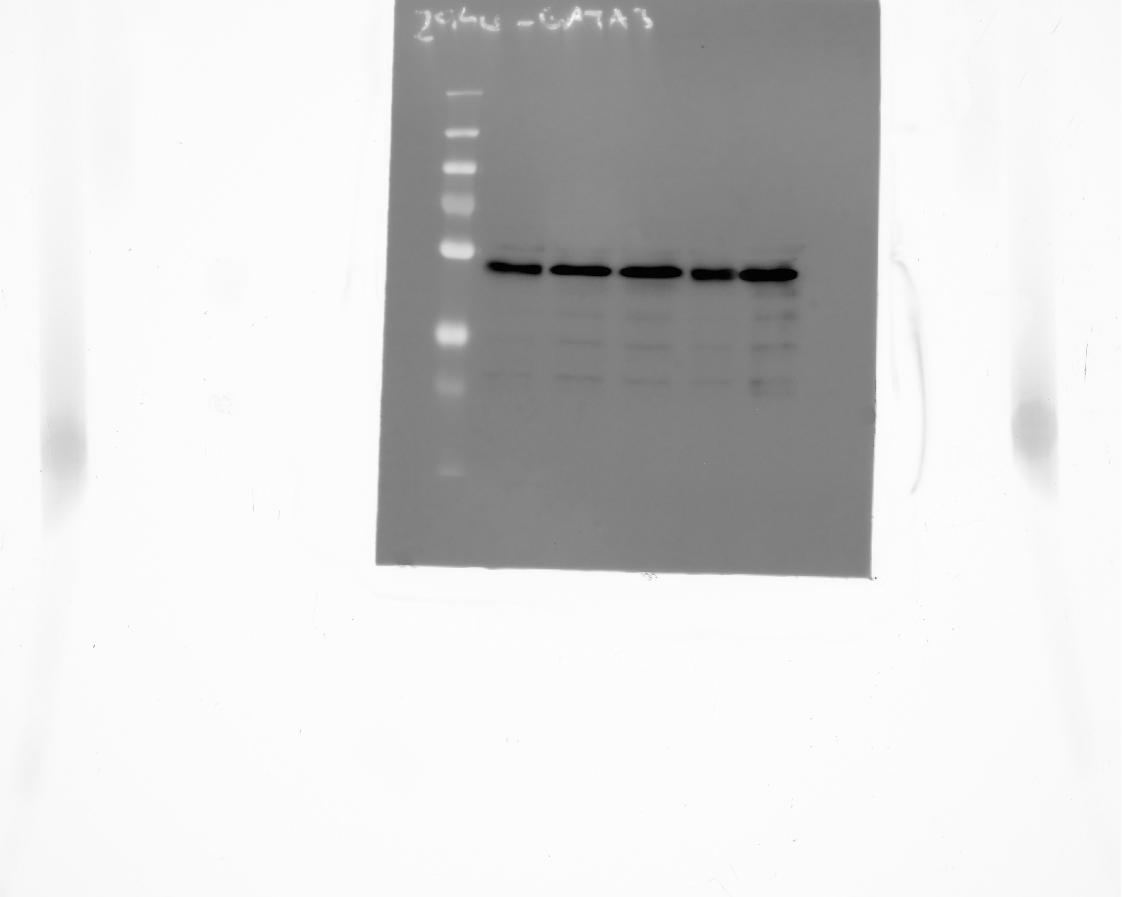

Supplement: Figure 3—source data 6. [file elife-106699-fig3-data6.zip › Figure 3ΓÇösource data 6 Original files for Western blot analysis displayed in Figure 3C./GATA3 CCRF-CEM Abd-VHL.tif]

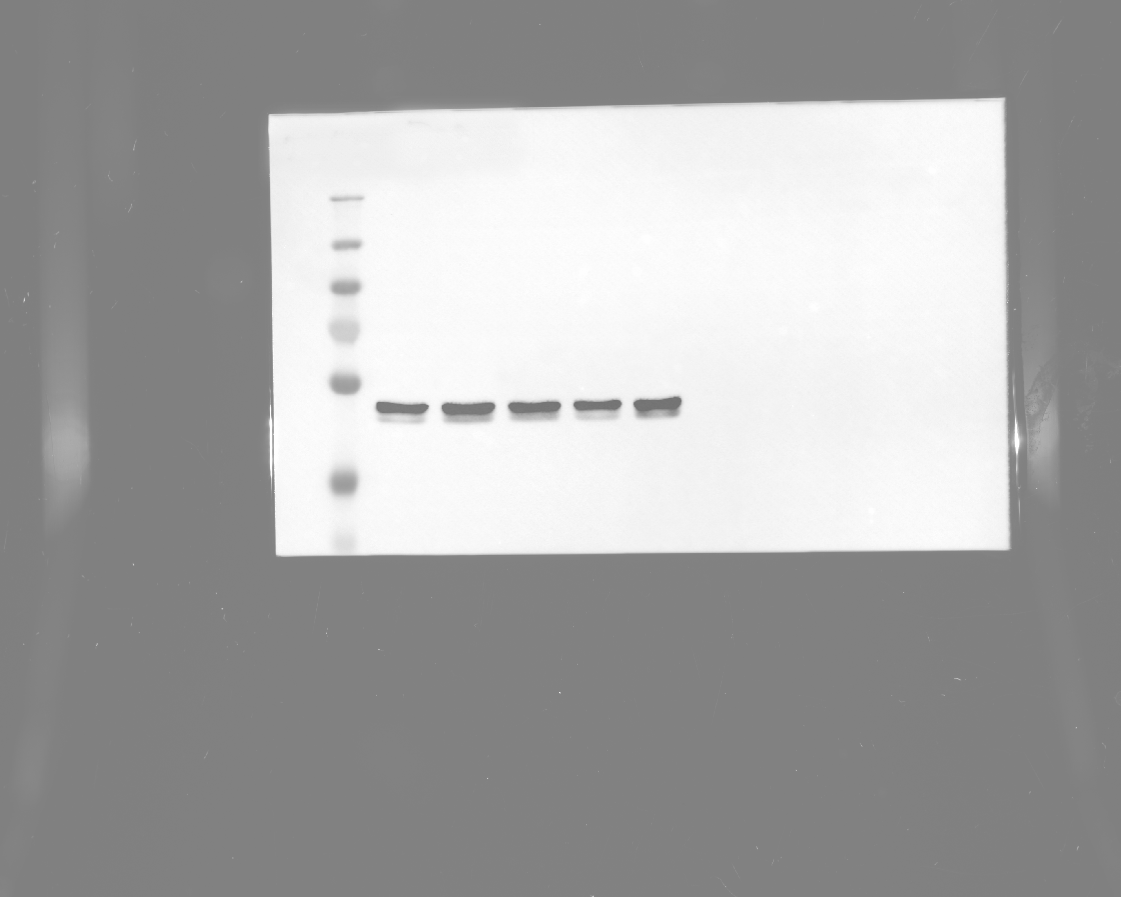

Supplement: Figure 3—source data 6. [file elife-106699-fig3-data6.zip › Figure 3ΓÇösource data 6 Original files for Western blot analysis displayed in Figure 3C./LDB1 CCRF-CEM Abd-CRBN.tif]

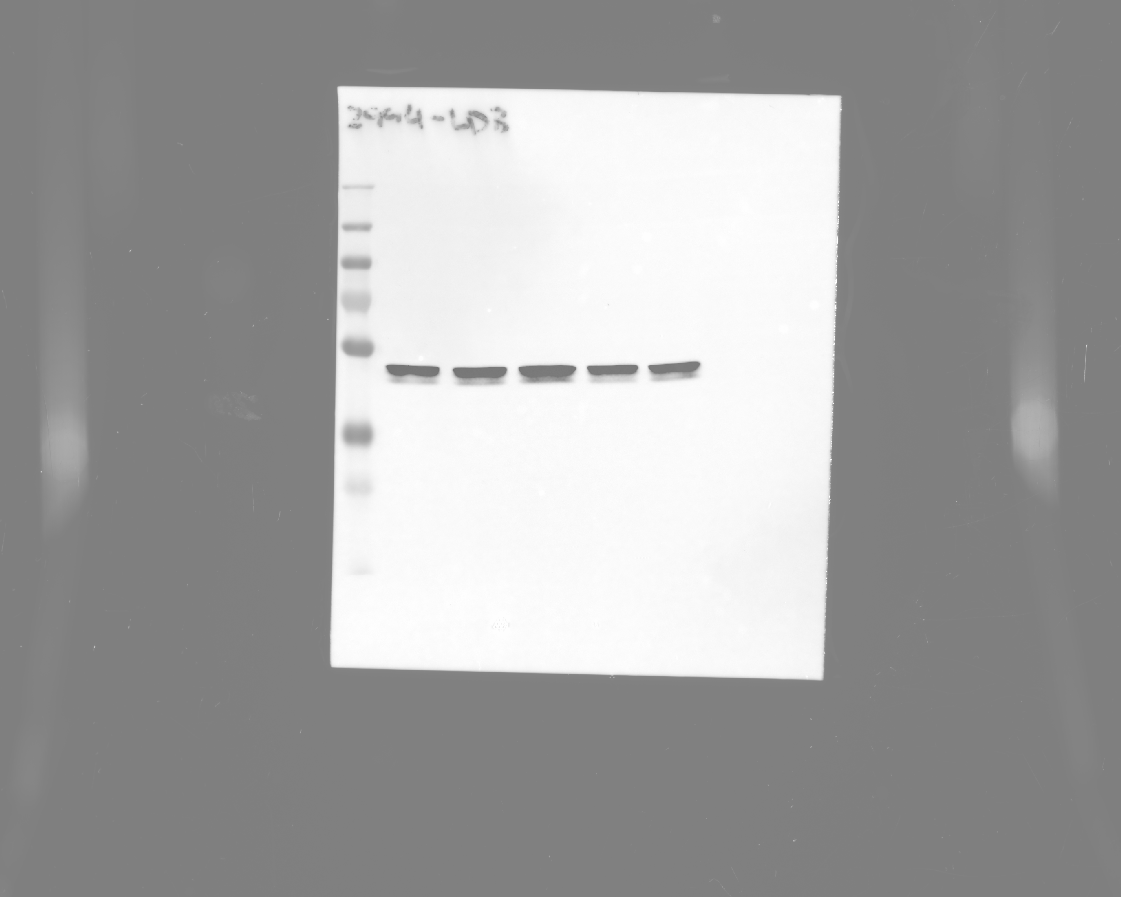

Supplement: Figure 3—source data 6. [file elife-106699-fig3-data6.zip › Figure 3ΓÇösource data 6 Original files for Western blot analysis displayed in Figure 3C./LDB1 CCRF-CEM Abd-VHL.tif]

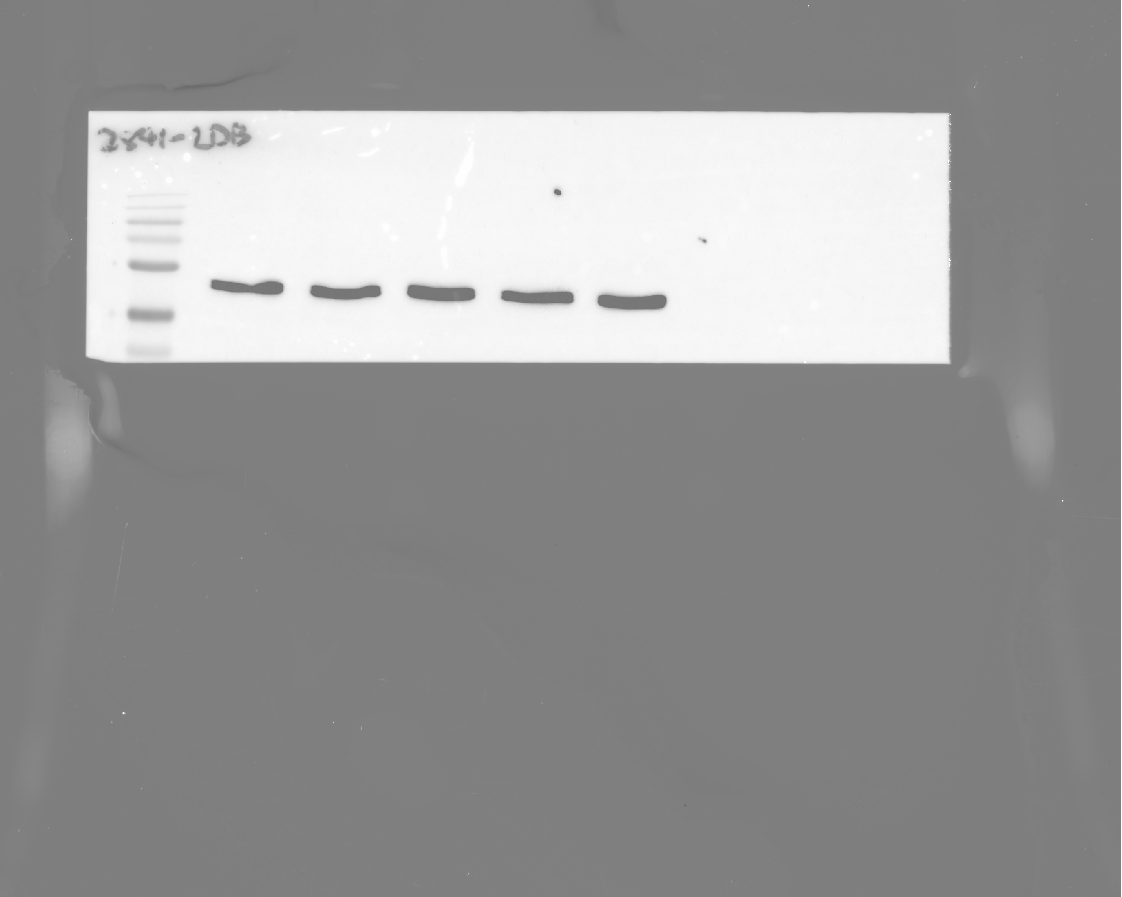

Supplement: Figure 3—source data 6. [file elife-106699-fig3-data6.zip › Figure 3ΓÇösource data 6 Original files for Western blot analysis displayed in Figure 3C./LDB1 KOPT-K1 Abd-CRBN.tif]

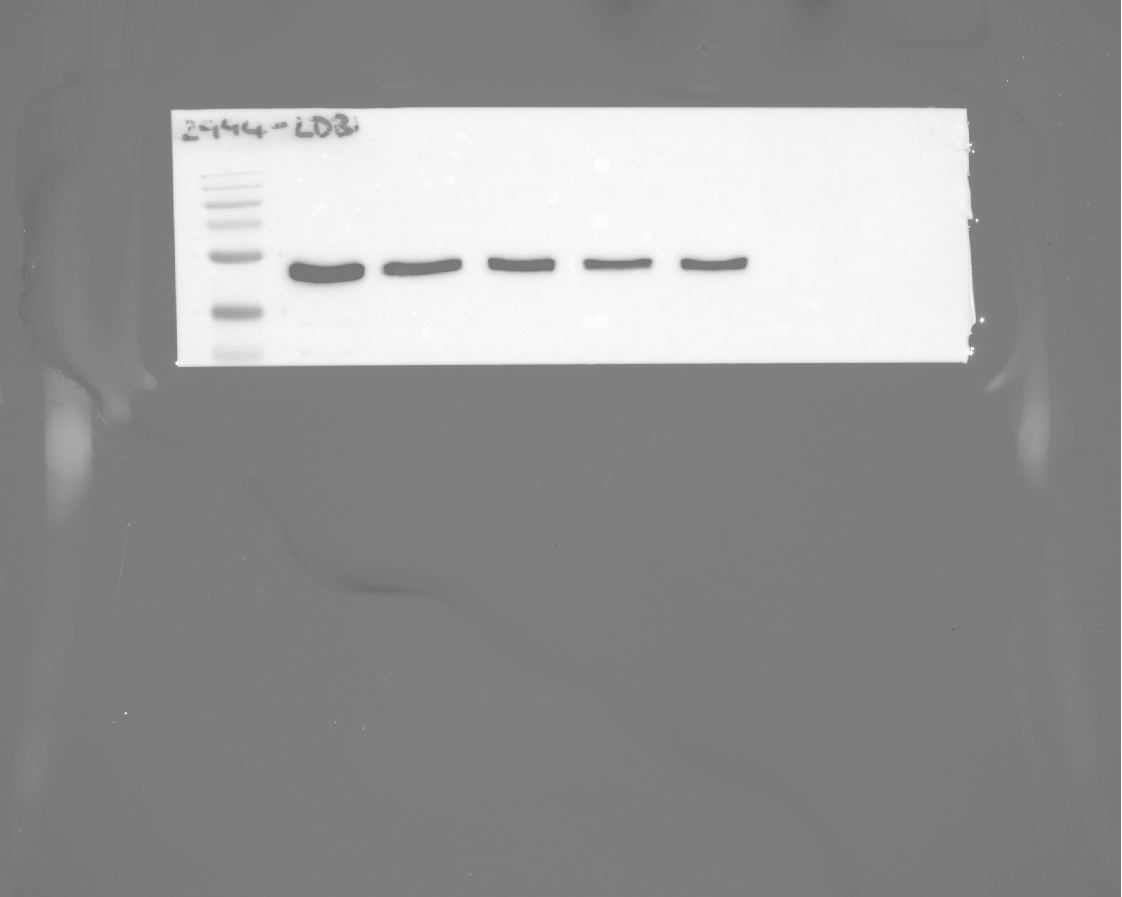

Supplement: Figure 3—source data 6. [file elife-106699-fig3-data6.zip › Figure 3ΓÇösource data 6 Original files for Western blot analysis displayed in Figure 3C./LDB1 KOPT-K1 Abd-VHL.tif]

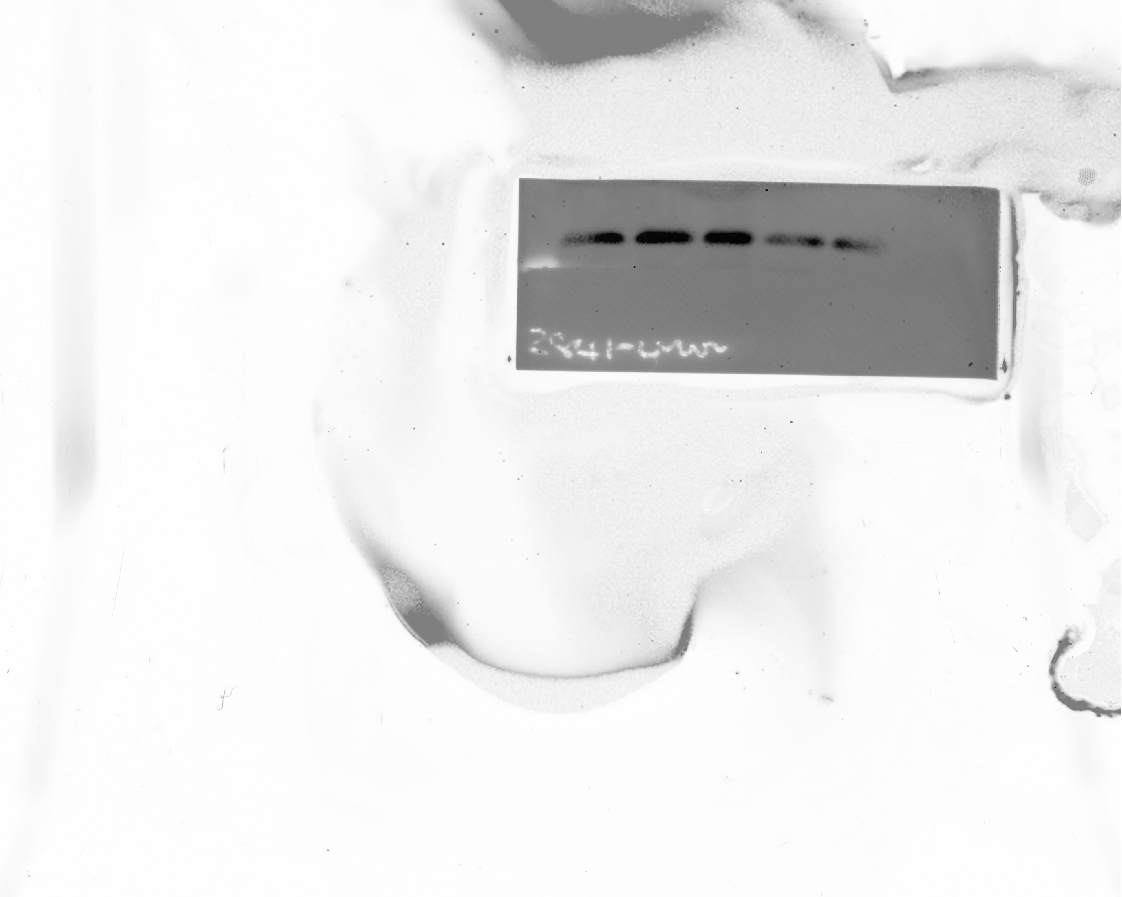

Supplement: Figure 3—source data 6. [file elife-106699-fig3-data6.zip › Figure 3ΓÇösource data 6 Original files for Western blot analysis displayed in Figure 3C./LMO2 CCRF-CEM Abd-CRBN.tif]

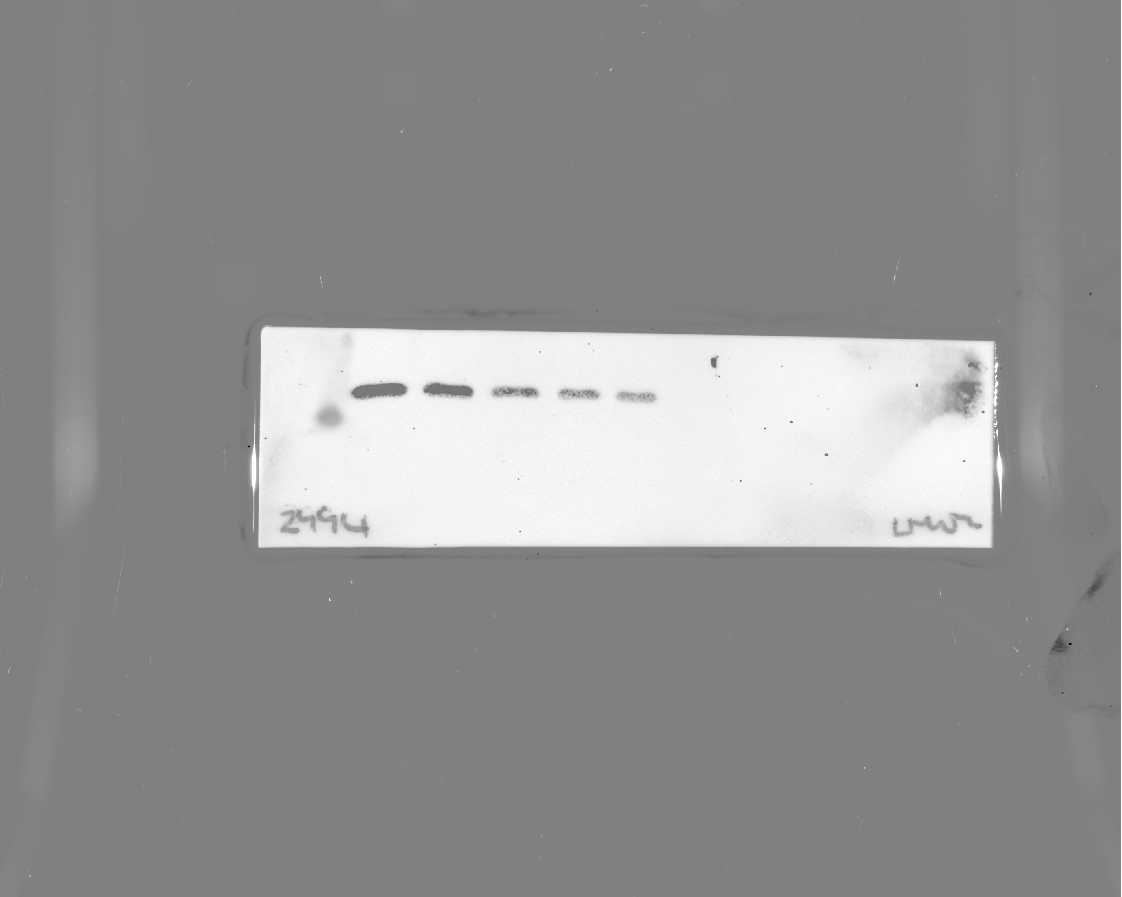

Supplement: Figure 3—source data 6. [file elife-106699-fig3-data6.zip › Figure 3ΓÇösource data 6 Original files for Western blot analysis displayed in Figure 3C./LMO2 CCRF-CEM Abd-VHL.tif]

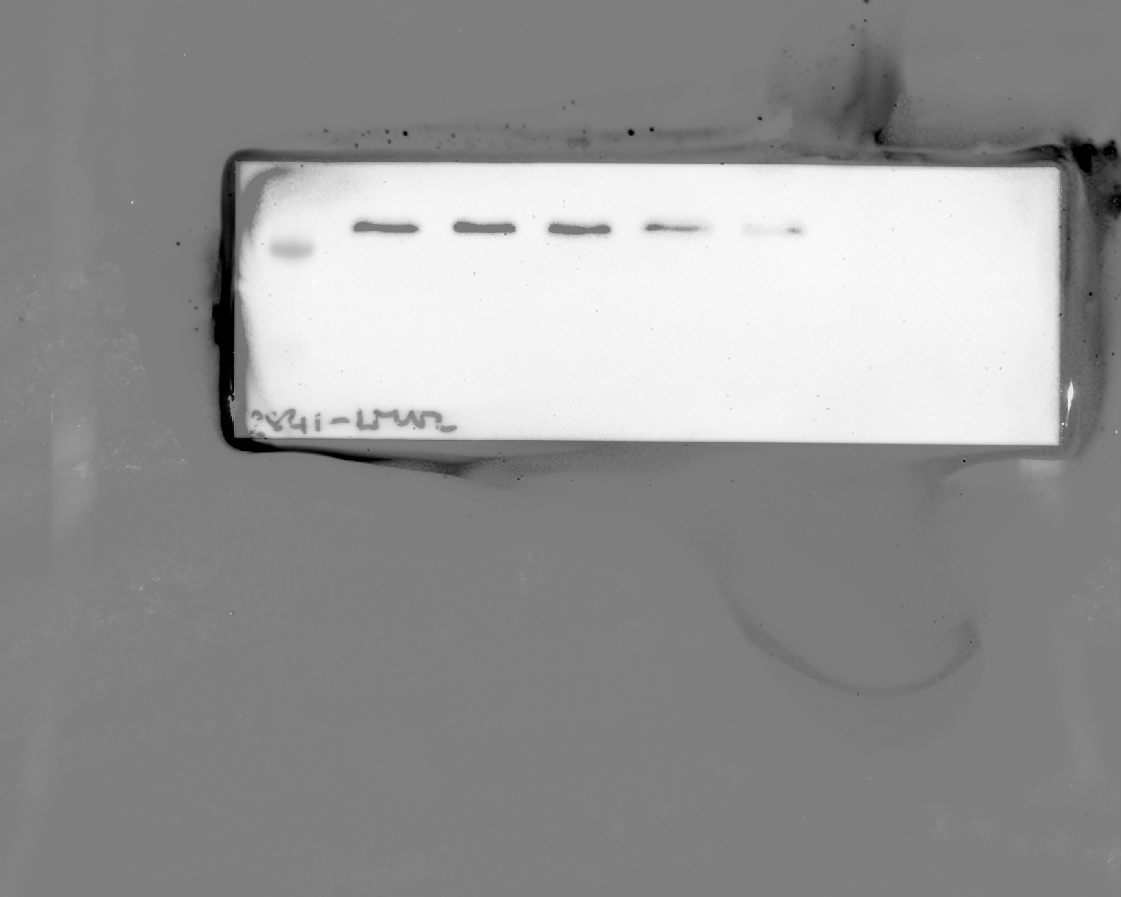

Supplement: Figure 3—source data 6. [file elife-106699-fig3-data6.zip › Figure 3ΓÇösource data 6 Original files for Western blot analysis displayed in Figure 3C./LMO2 KOPT-K1 Abd-CRBN.tif]

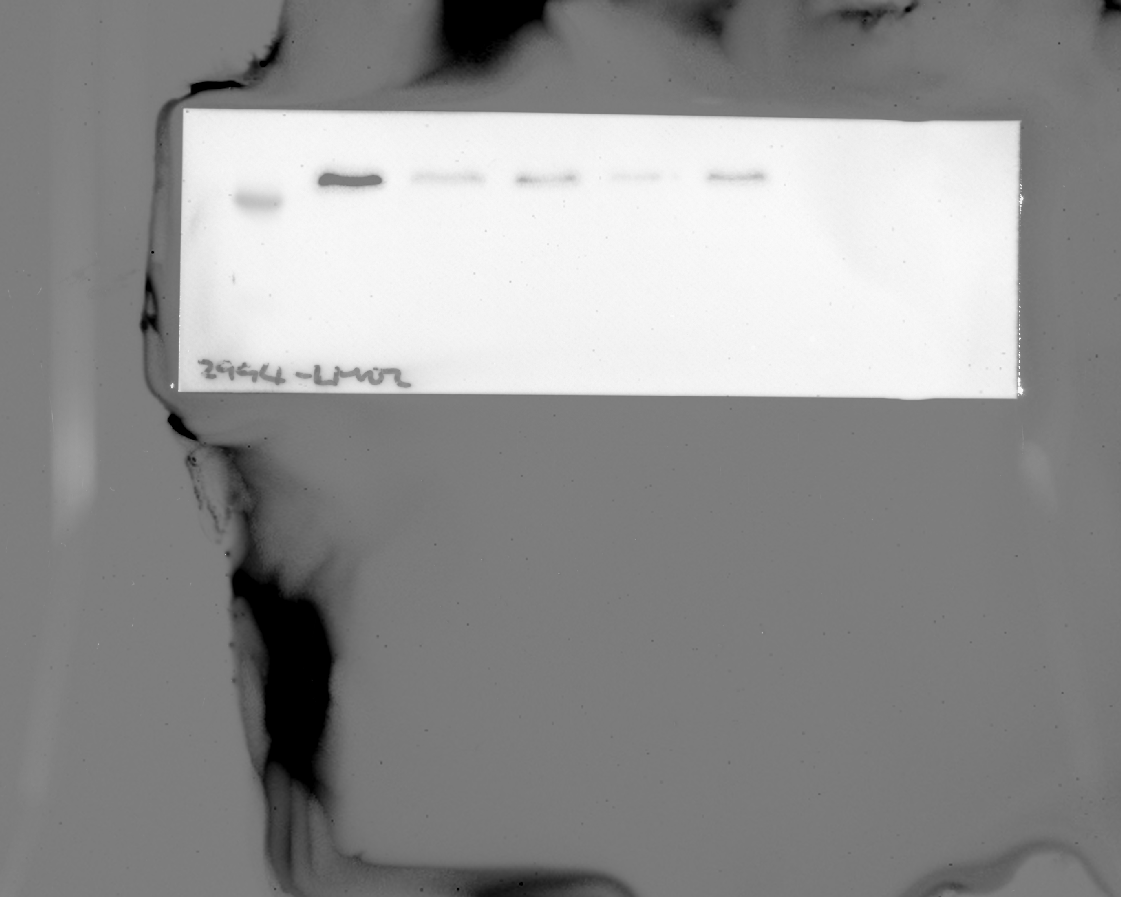

Supplement: Figure 3—source data 6. [file elife-106699-fig3-data6.zip › Figure 3ΓÇösource data 6 Original files for Western blot analysis displayed in Figure 3C./LMO2 KOPT-K1 Abd-VHL.tif]

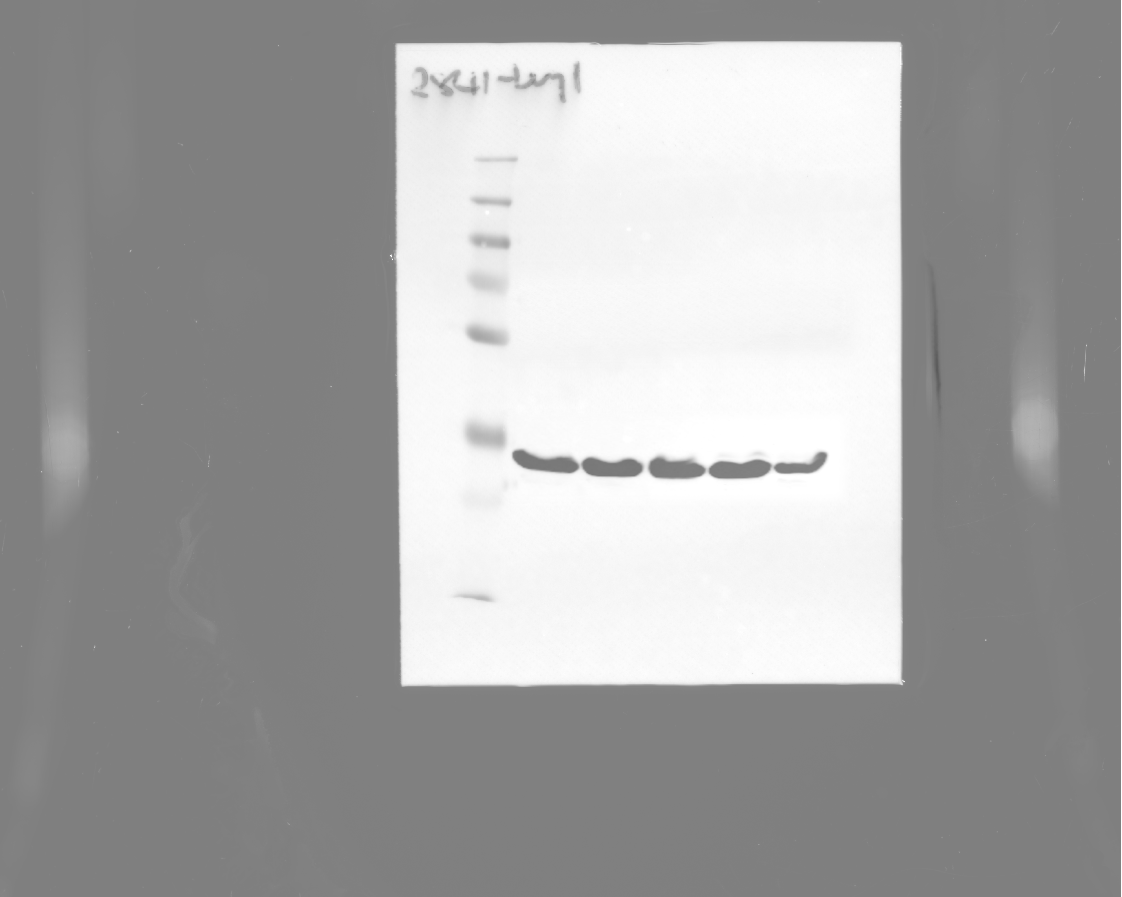

Supplement: Figure 3—source data 6. [file elife-106699-fig3-data6.zip › Figure 3ΓÇösource data 6 Original files for Western blot analysis displayed in Figure 3C./Lyl1 CCRF-CEM Abd-CRBN.tif]

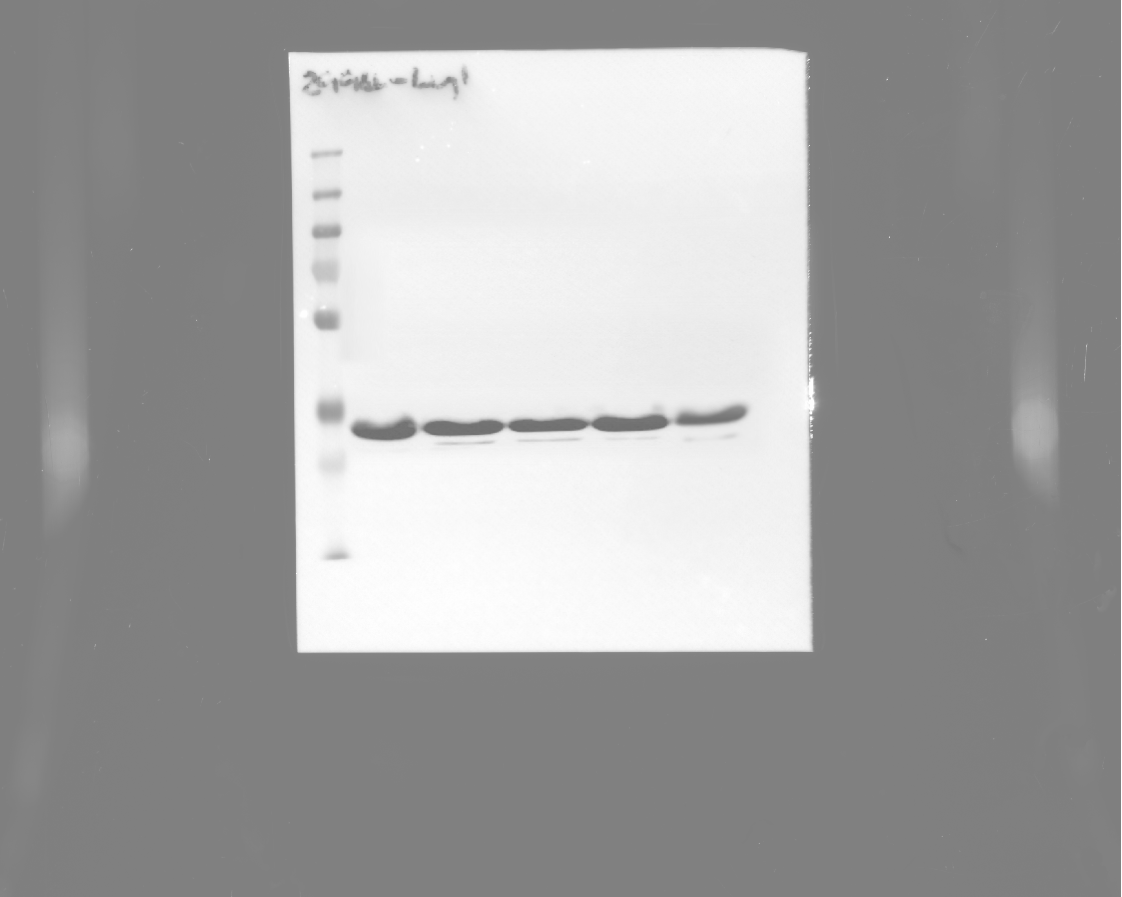

Supplement: Figure 3—source data 6. [file elife-106699-fig3-data6.zip › Figure 3ΓÇösource data 6 Original files for Western blot analysis displayed in Figure 3C./Lyl1 CCRF-CEM Abd-VHL.tif]

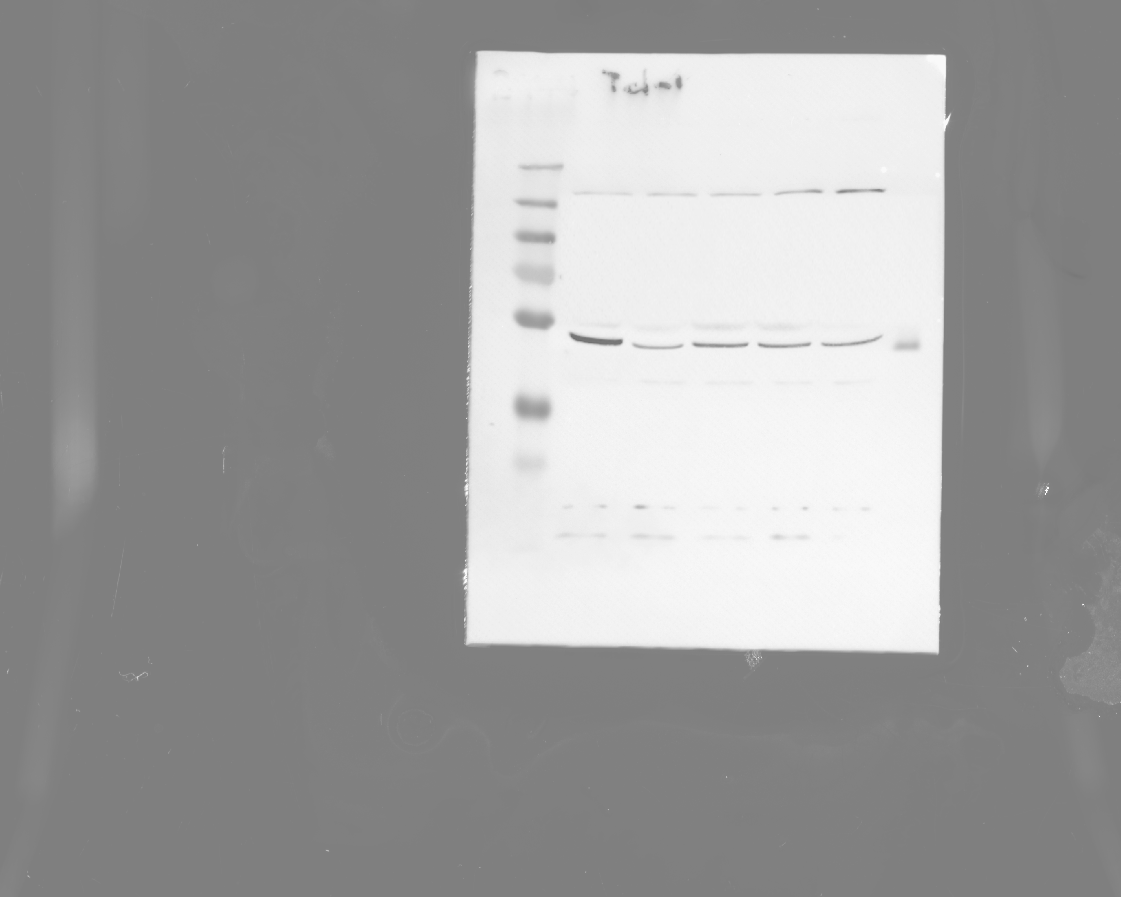

Supplement: Figure 3—source data 6. [file elife-106699-fig3-data6.zip › Figure 3ΓÇösource data 6 Original files for Western blot analysis displayed in Figure 3C./Tal-1 CCRF-CEM Abd-CRBN.tif]

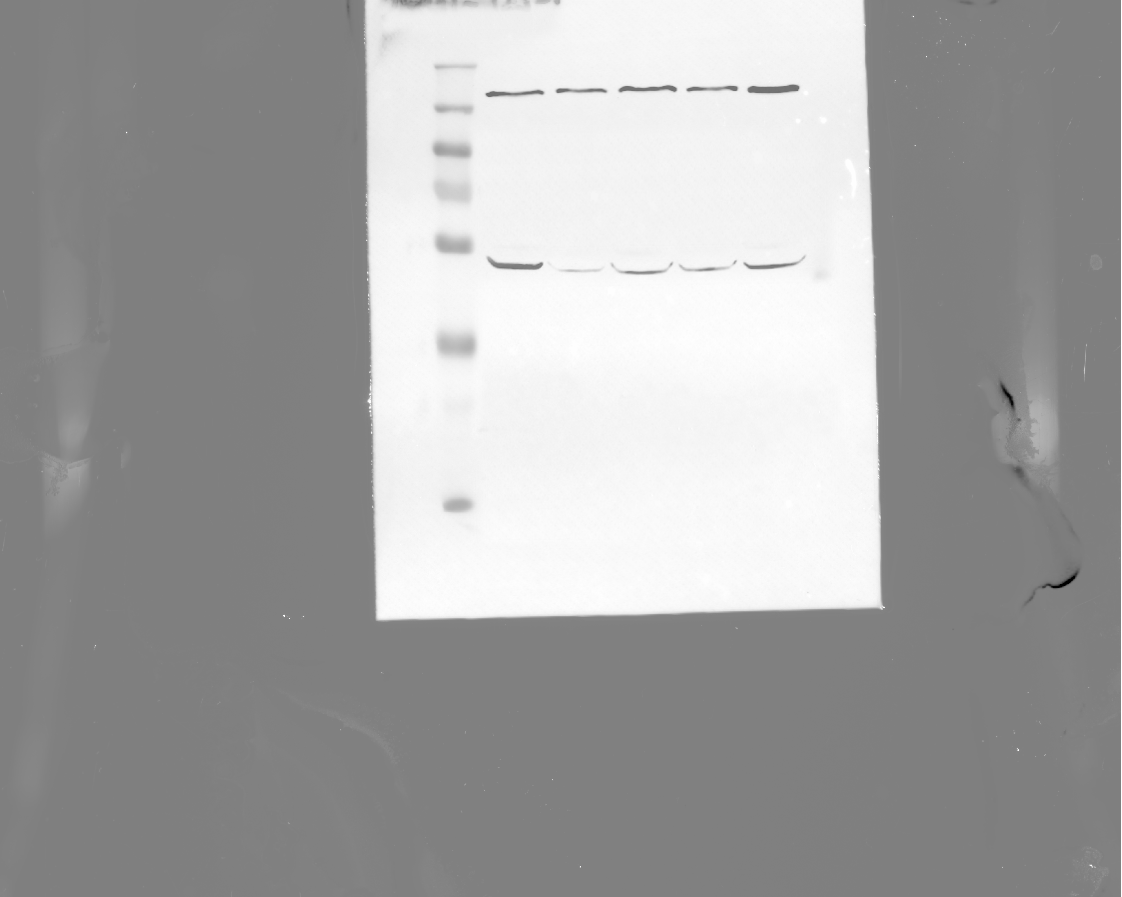

Supplement: Figure 3—source data 6. [file elife-106699-fig3-data6.zip › Figure 3ΓÇösource data 6 Original files for Western blot analysis displayed in Figure 3C./Tal-1 CCRF-CEM Abd-VHL.tif]

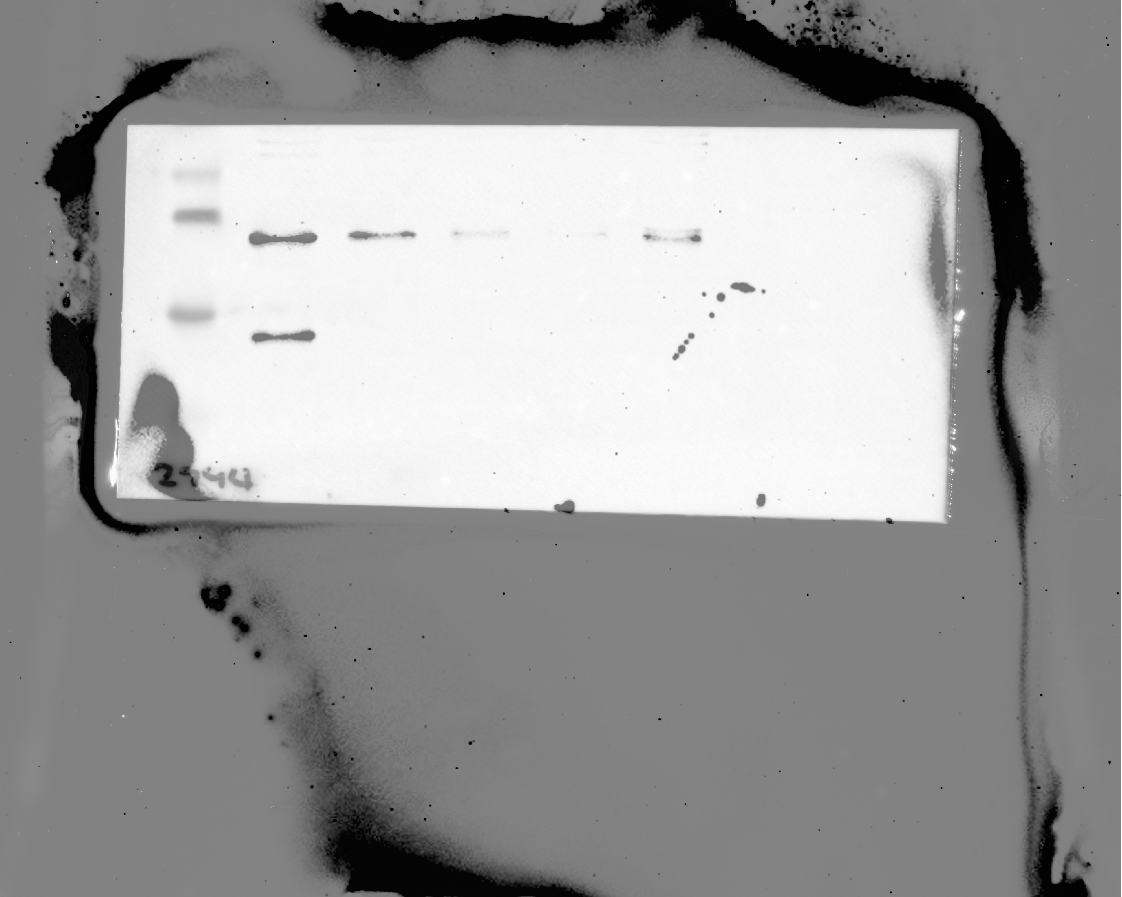

Supplement: Figure 3—source data 6. [file elife-106699-fig3-data6.zip › Figure 3ΓÇösource data 6 Original files for Western blot analysis displayed in Figure 3C./Tal-1 KOPT-K1 Abd-VHL.tif]

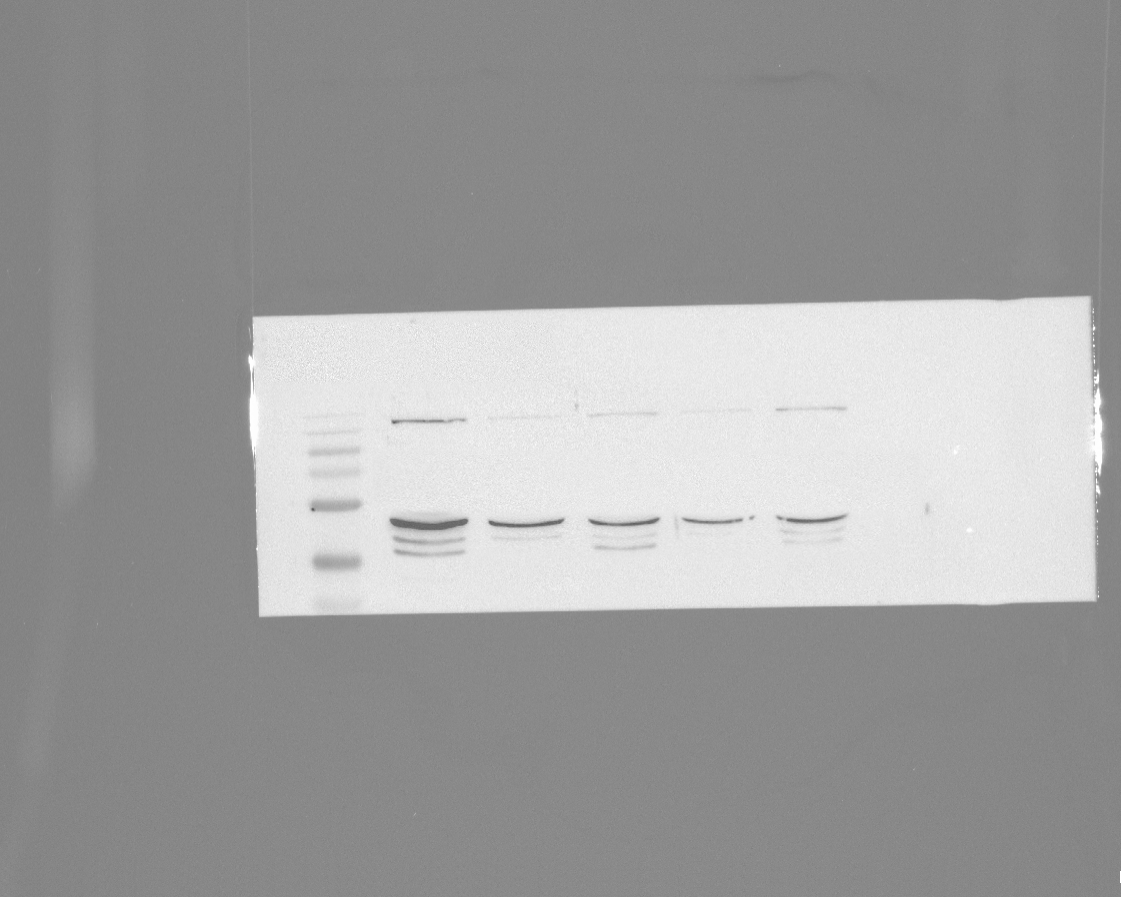

Supplement: Figure 3—source data 6. [file elife-106699-fig3-data6.zip › Figure 3ΓÇösource data 6 Original files for Western blot analysis displayed in Figure 3C./Tal1 KOPT-K1 Abd-CRBN.tif]

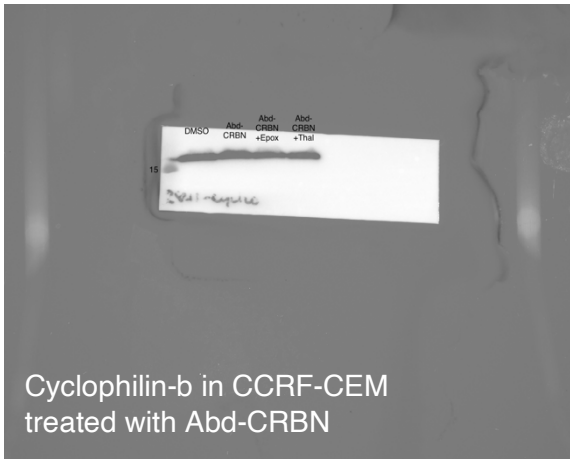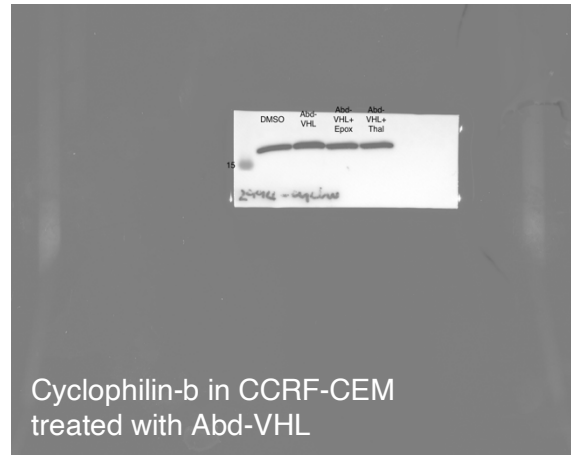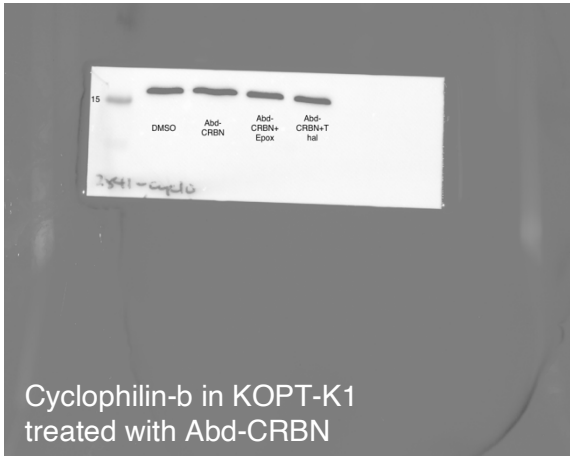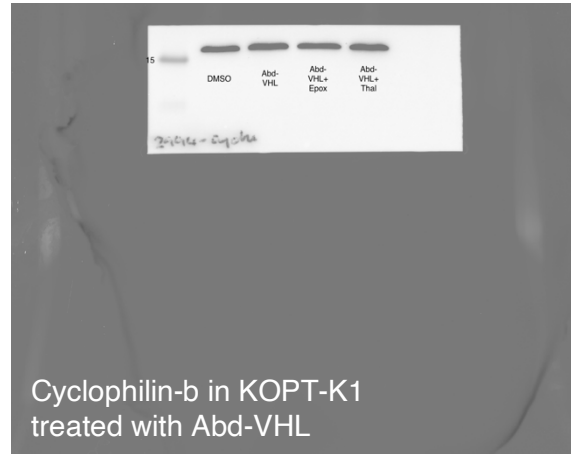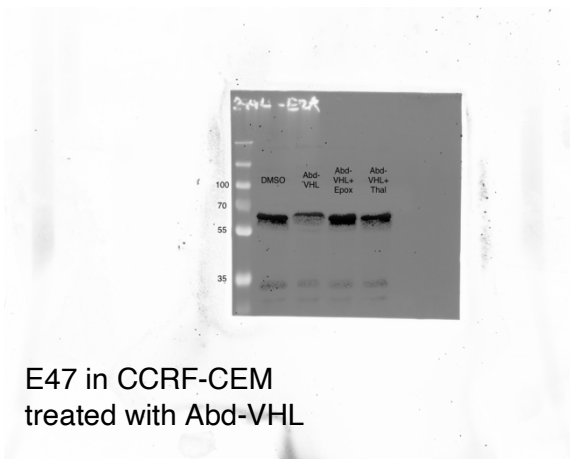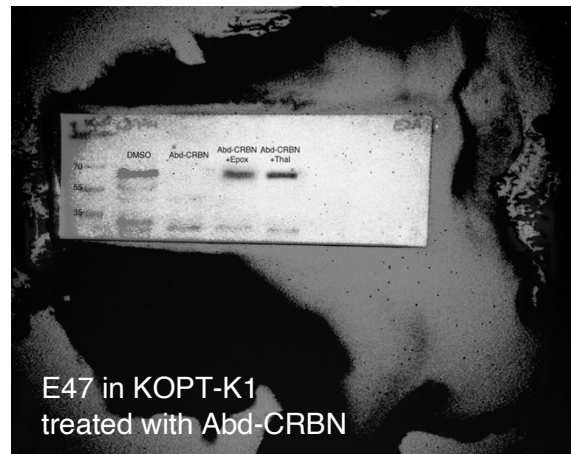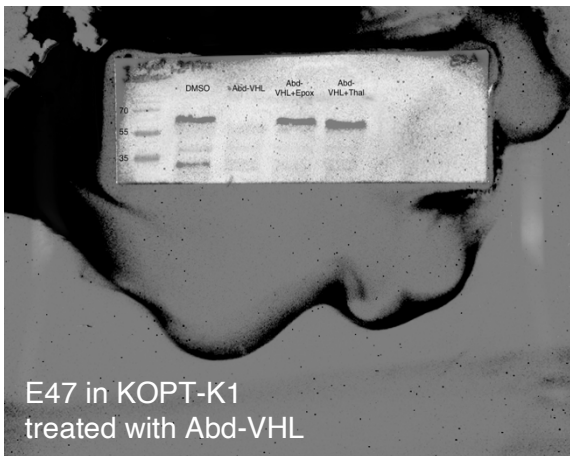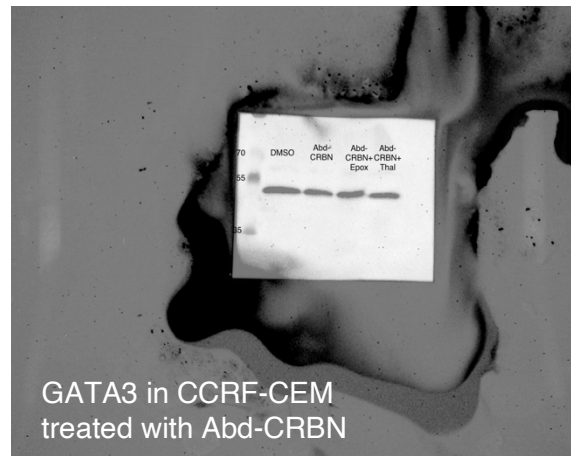

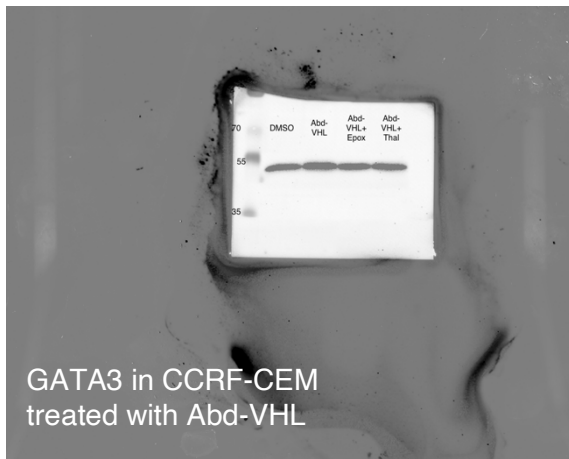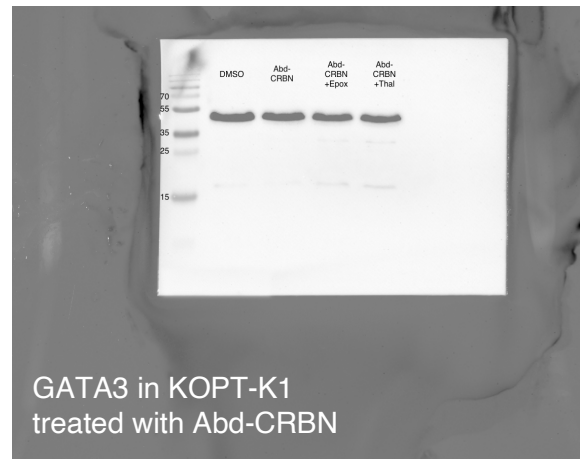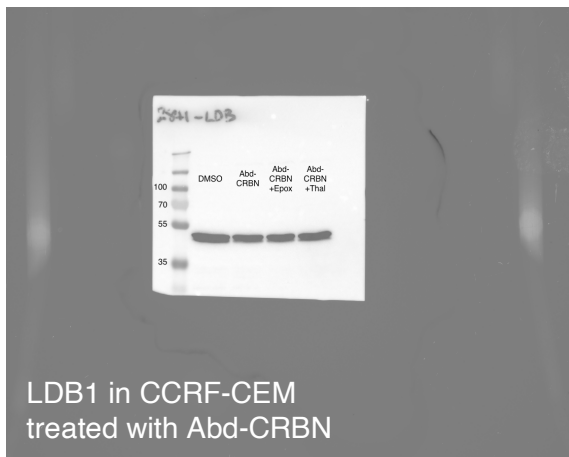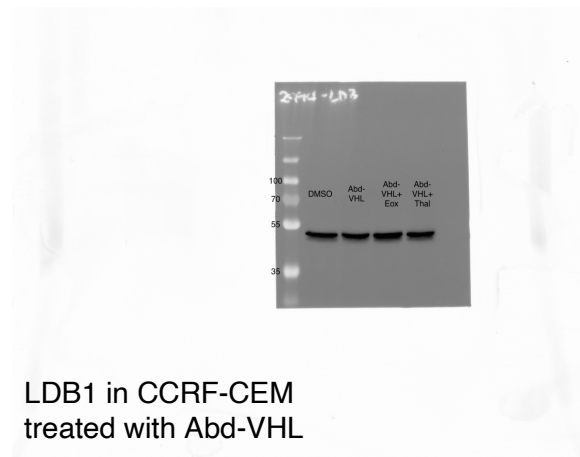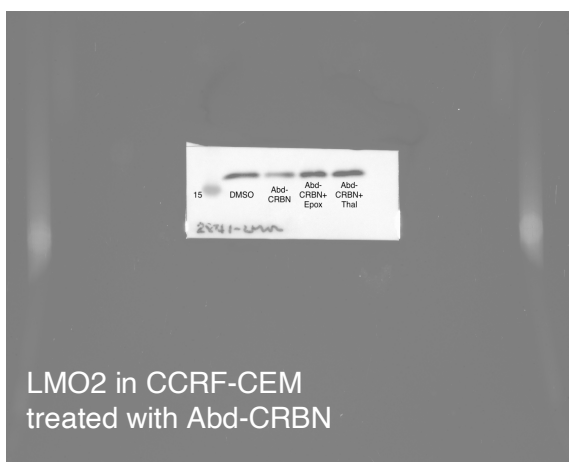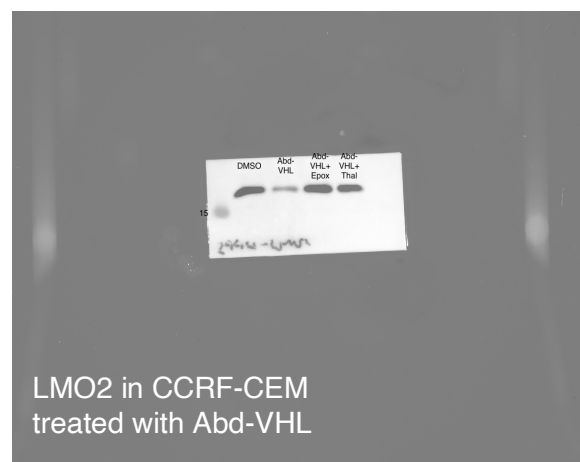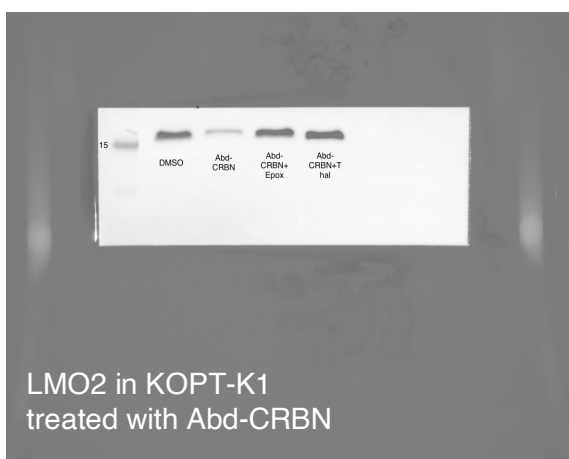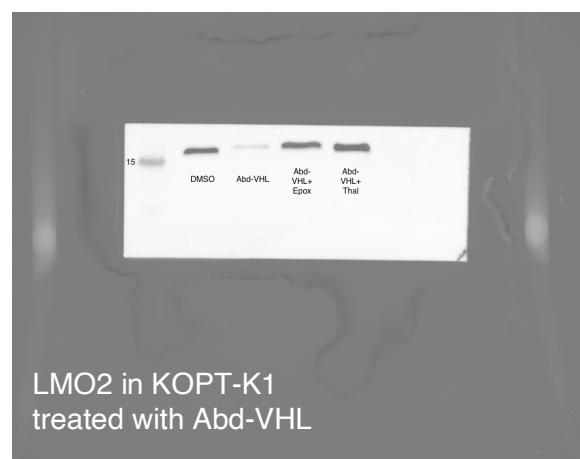

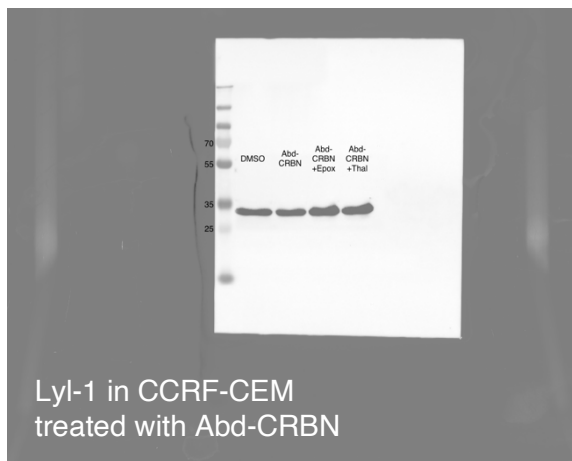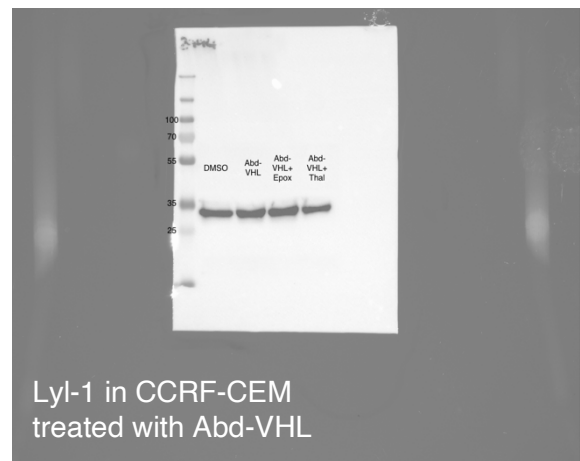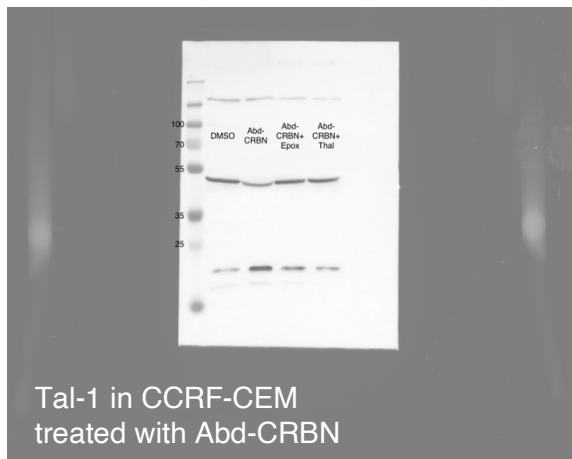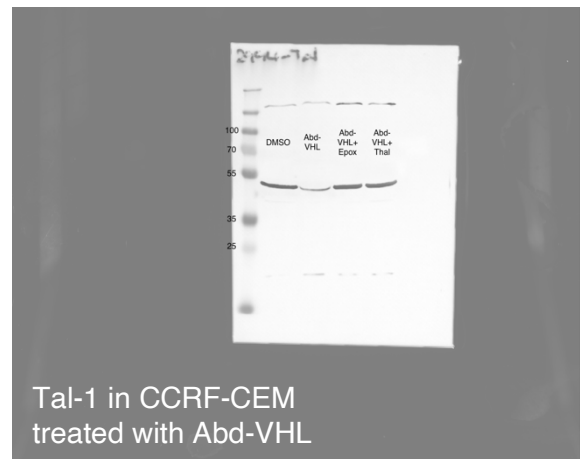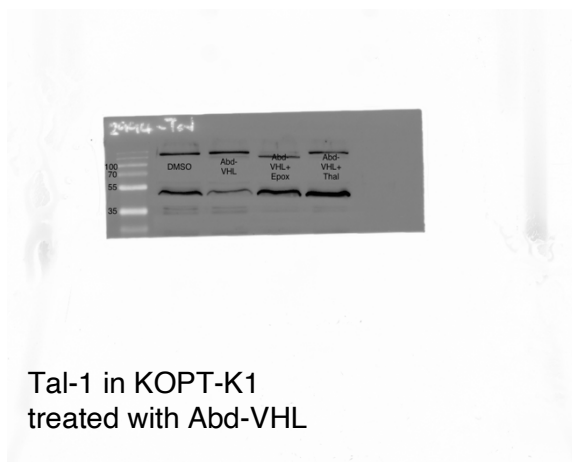

**Figure 3, Source Data 7.** Original membranes corresponding to Figure 3, panel D.

Supplement: Figure 3—source data 7. [file elife-106699-fig3-data7.zip › Figure 3ΓÇösource data 7 PDF files containing original western blots for Figure 3D, indicating the relevant bands and treatments./Figure 3-source data 7.pdf]

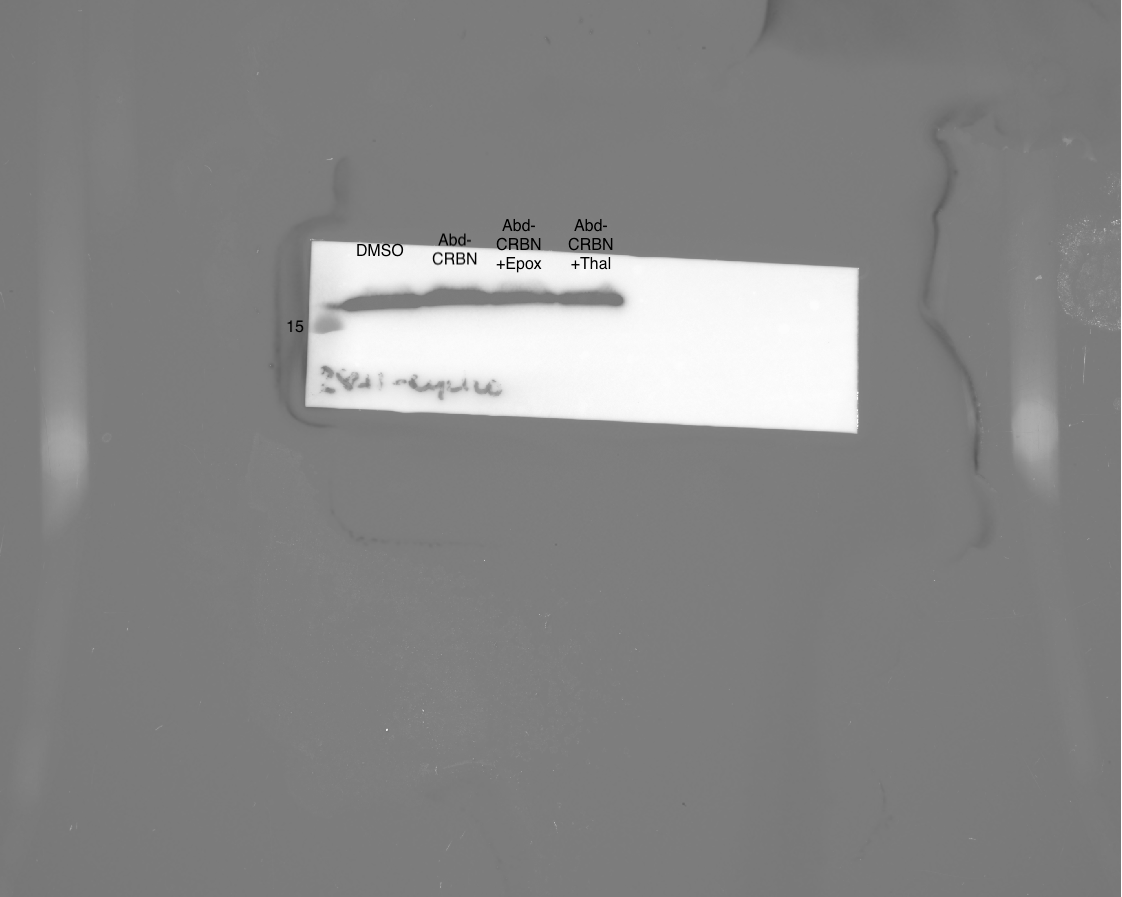

Supplement: Figure 3—source data 7. [file elife-106699-fig3-data7.zip › Figure 3ΓÇösource data 7 PDF files containing original western blots for Figure 3D, indicating the relevant bands and treatments./Raw data/Cyclophilin CCRF-CEM Abd-CRBN.tif]

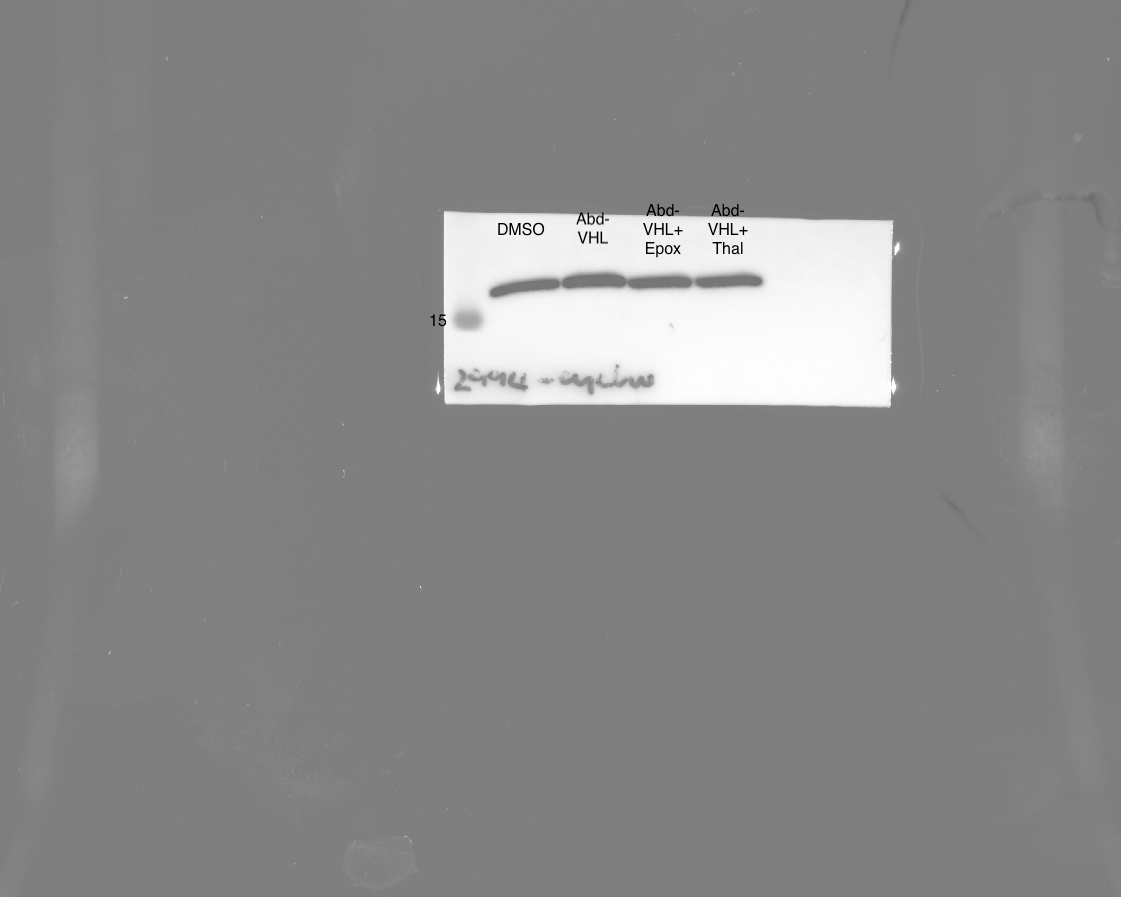

Supplement: Figure 3—source data 7. [file elife-106699-fig3-data7.zip › Figure 3ΓÇösource data 7 PDF files containing original western blots for Figure 3D, indicating the relevant bands and treatments./Raw data/Cyclophilin CCRF-CEM Abd-VHL.tif]

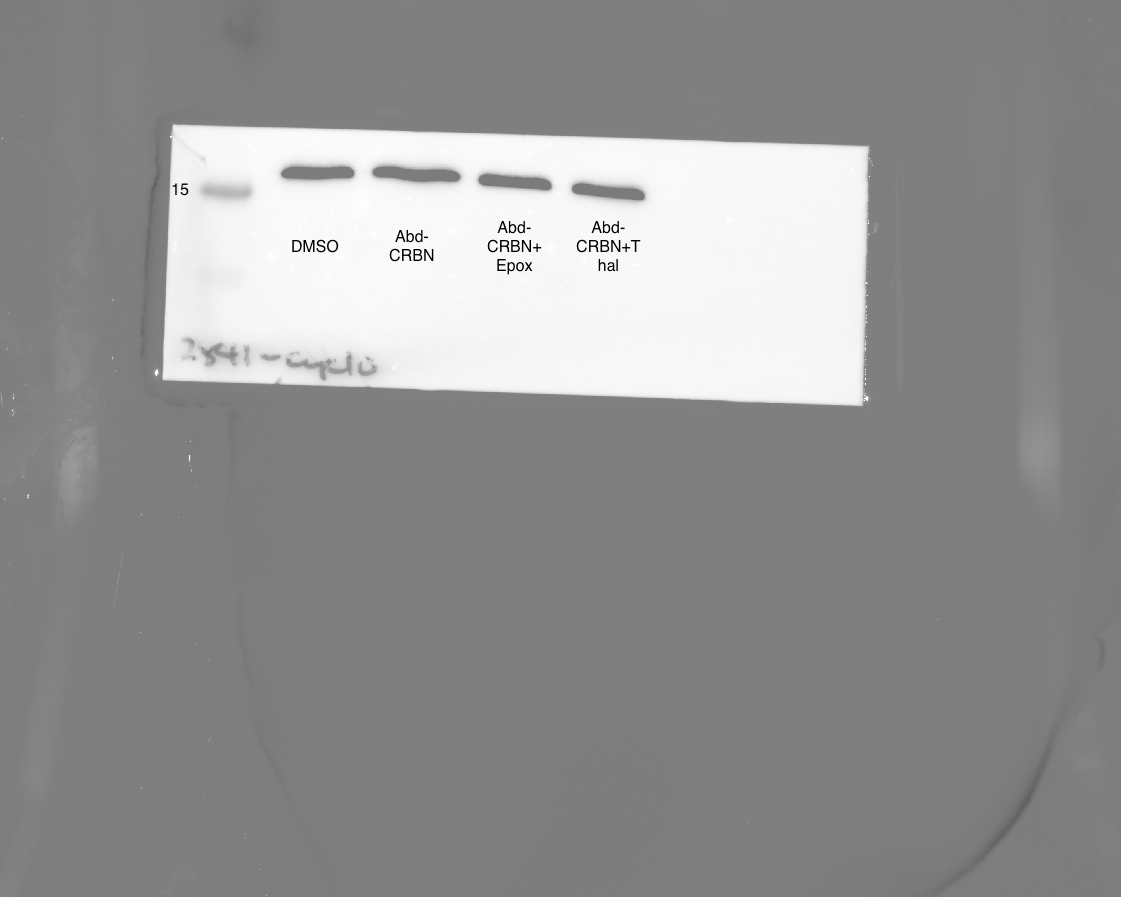

Supplement: Figure 3—source data 7. [file elife-106699-fig3-data7.zip › Figure 3ΓÇösource data 7 PDF files containing original western blots for Figure 3D, indicating the relevant bands and treatments./Raw data/Cyclophilin KOPT-K1 Abd-CRBN.tif]

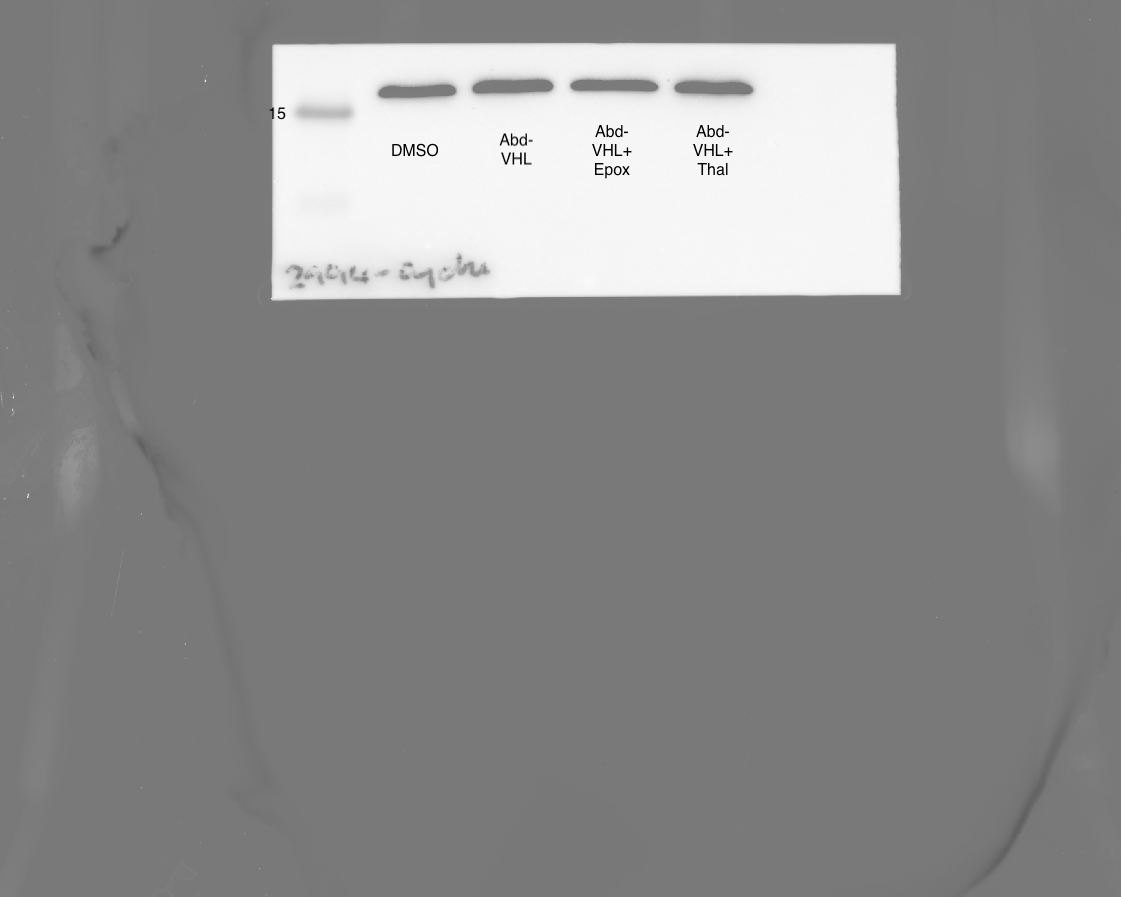

Supplement: Figure 3—source data 7. [file elife-106699-fig3-data7.zip › Figure 3ΓÇösource data 7 PDF files containing original western blots for Figure 3D, indicating the relevant bands and treatments./Raw data/Cyclophilin KOPT-K1 Abd-VHL.tif]

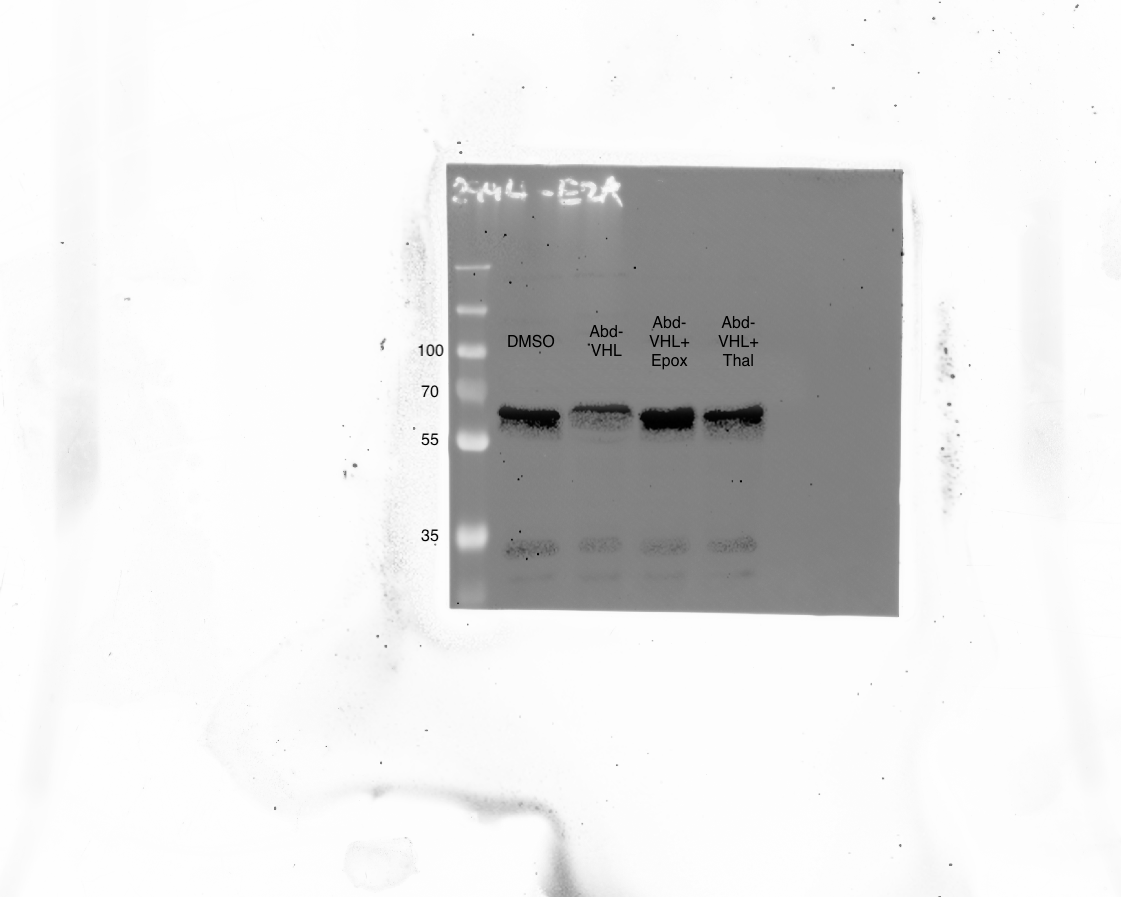

Supplement: Figure 3—source data 7. [file elife-106699-fig3-data7.zip › Figure 3ΓÇösource data 7 PDF files containing original western blots for Figure 3D, indicating the relevant bands and treatments./Raw data/E2A CCRF-CEM Abd-VHL.tif]

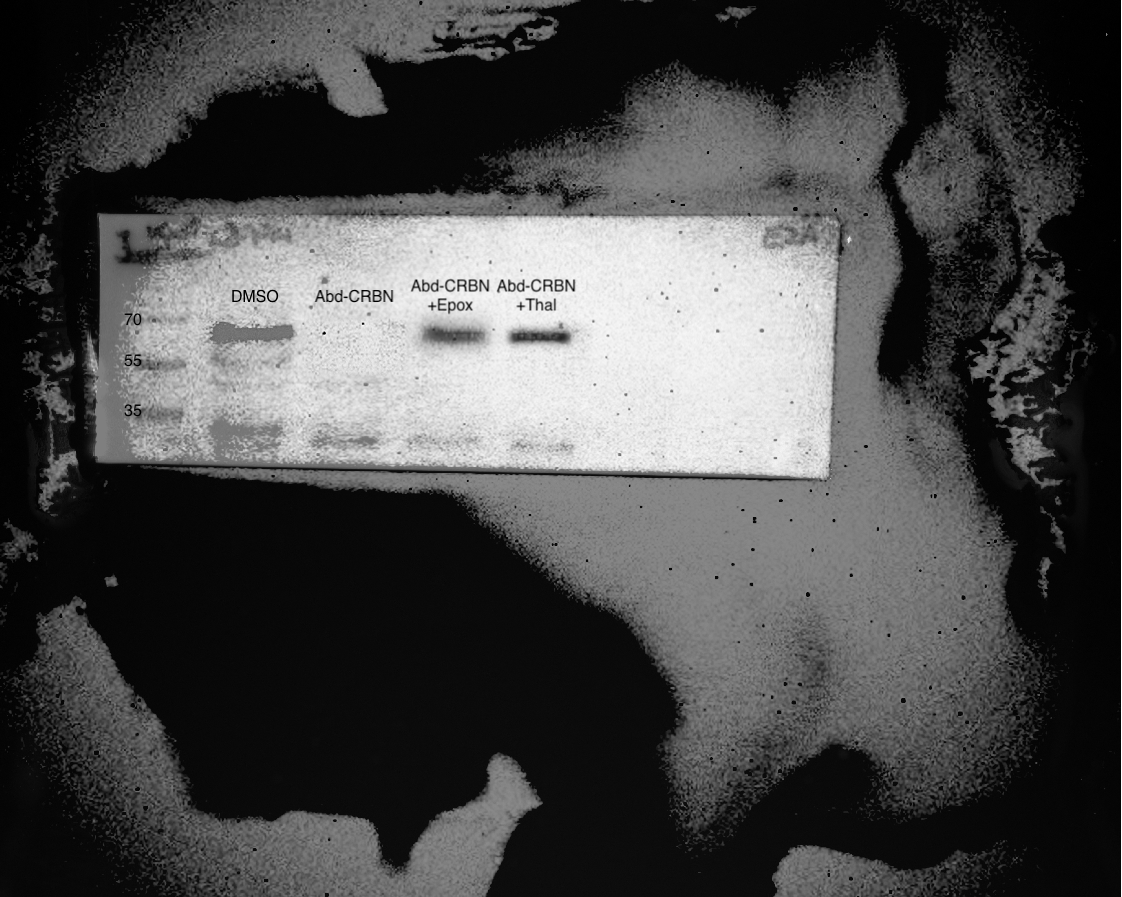

Supplement: Figure 3—source data 7. [file elife-106699-fig3-data7.zip › Figure 3ΓÇösource data 7 PDF files containing original western blots for Figure 3D, indicating the relevant bands and treatments./Raw data/E2A KOPT-K1 Abd-CRBN.tif]

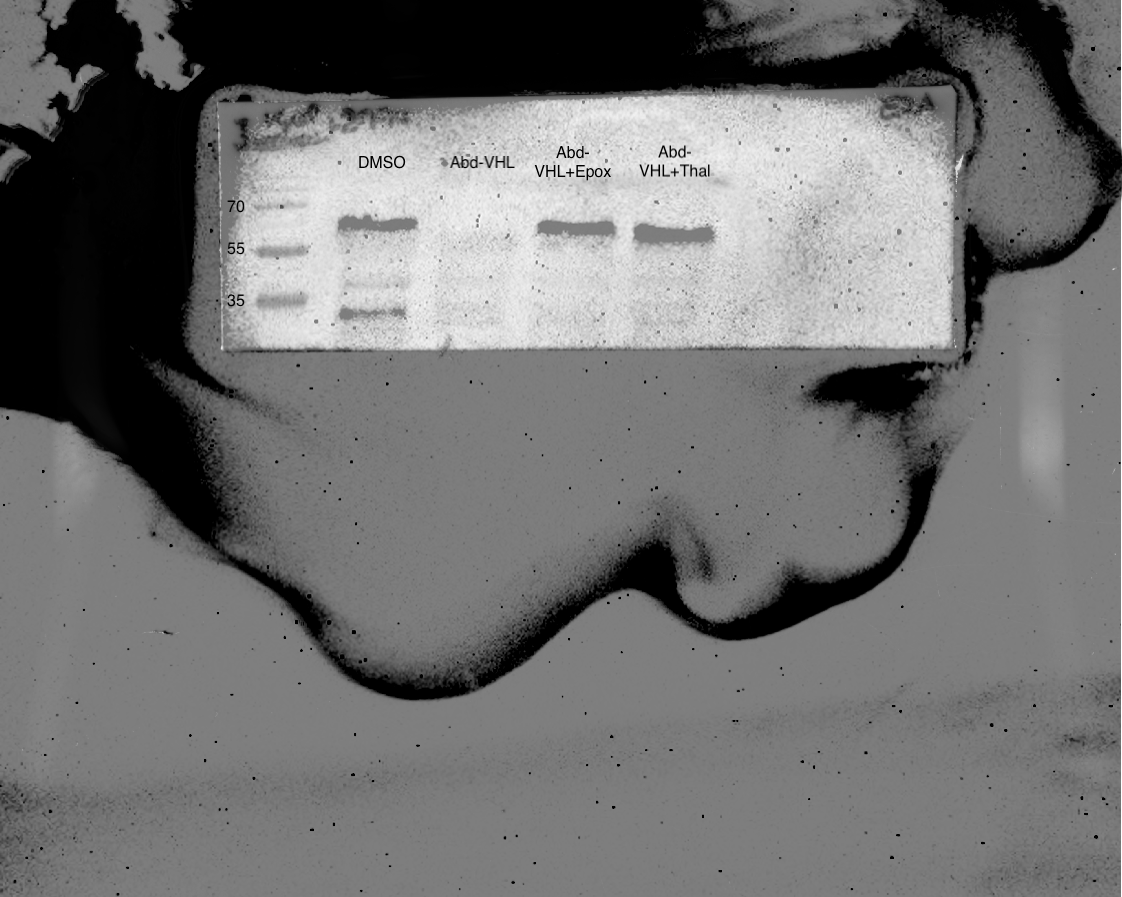

Supplement: Figure 3—source data 7. [file elife-106699-fig3-data7.zip › Figure 3ΓÇösource data 7 PDF files containing original western blots for Figure 3D, indicating the relevant bands and treatments./Raw data/E2A KOPT-K1 Abd-VHL.tif]

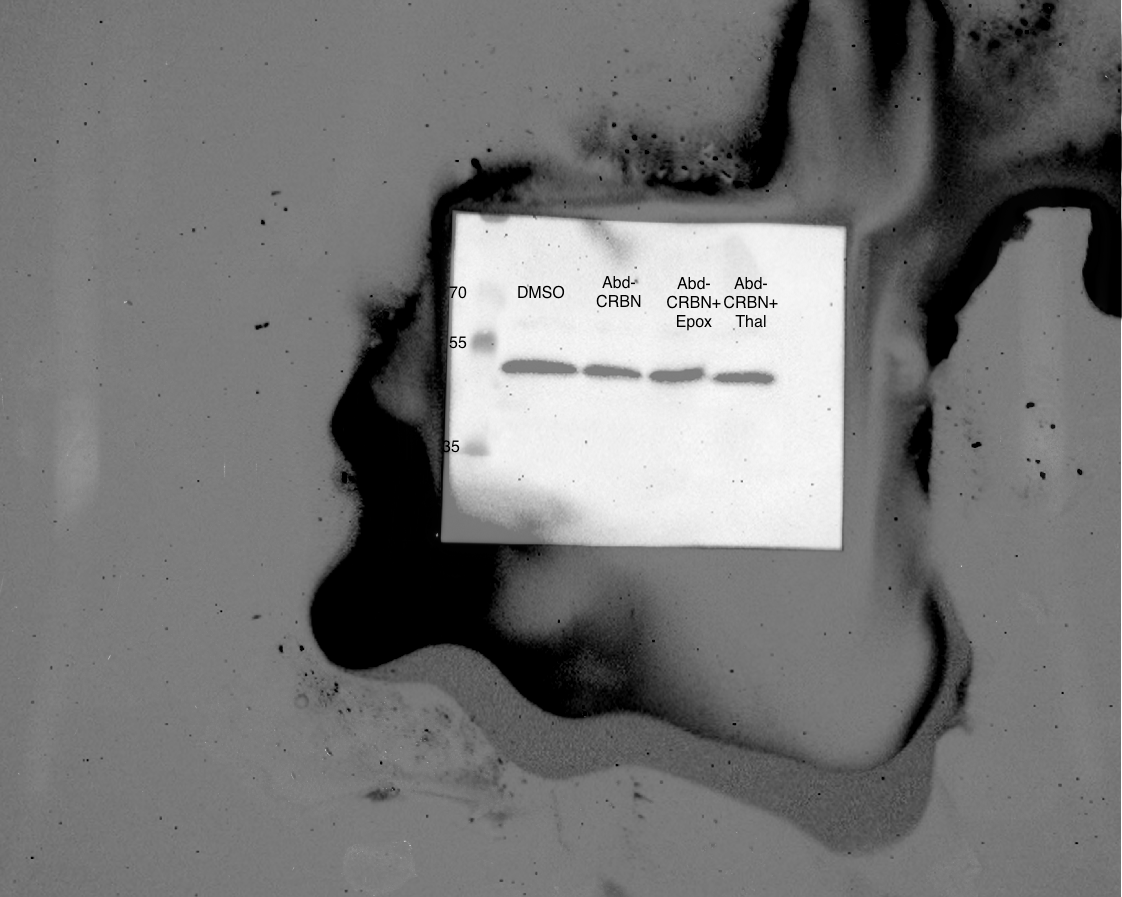

Supplement: Figure 3—source data 7. [file elife-106699-fig3-data7.zip › Figure 3ΓÇösource data 7 PDF files containing original western blots for Figure 3D, indicating the relevant bands and treatments./Raw data/GATA3 CCRF-CEM Abd-CRBN.tif]

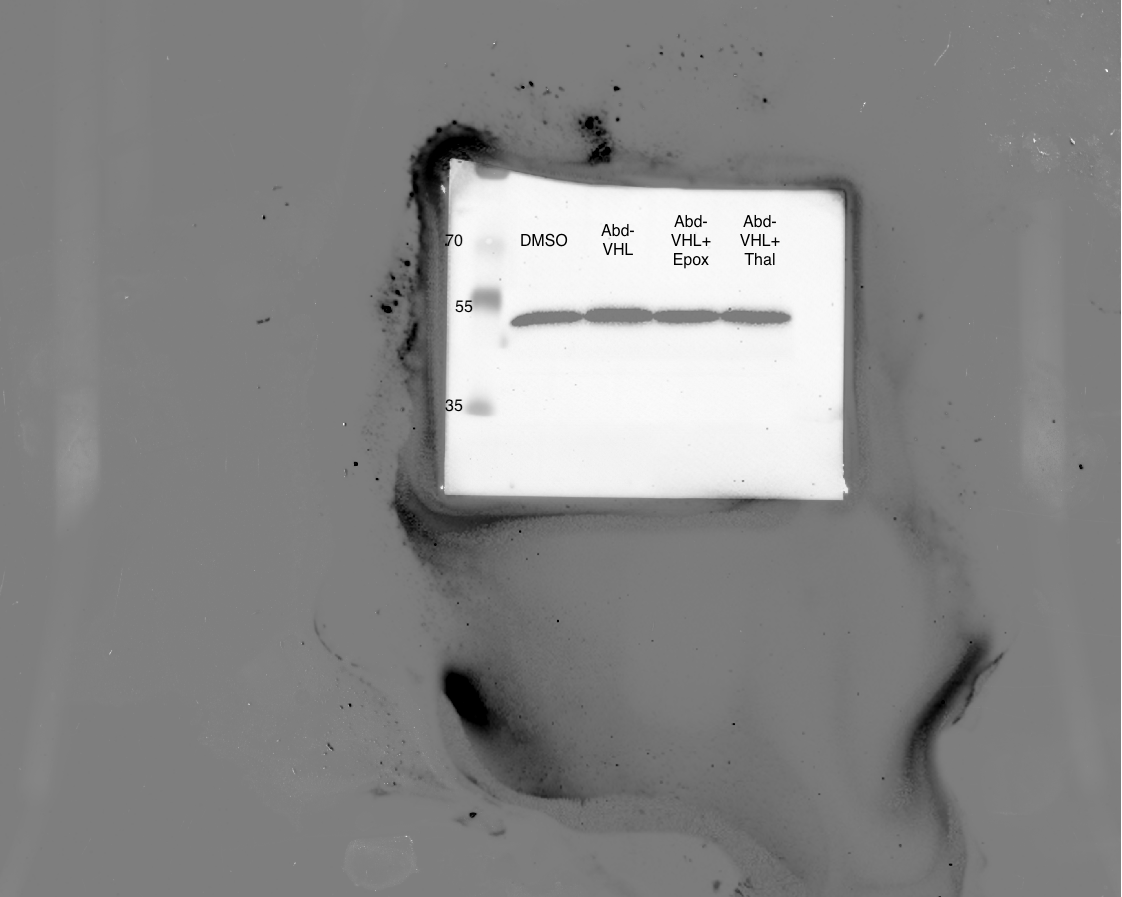

Supplement: Figure 3—source data 7. [file elife-106699-fig3-data7.zip › Figure 3ΓÇösource data 7 PDF files containing original western blots for Figure 3D, indicating the relevant bands and treatments./Raw data/GATA3 CCRF-CEM Abd-VHL.tif]

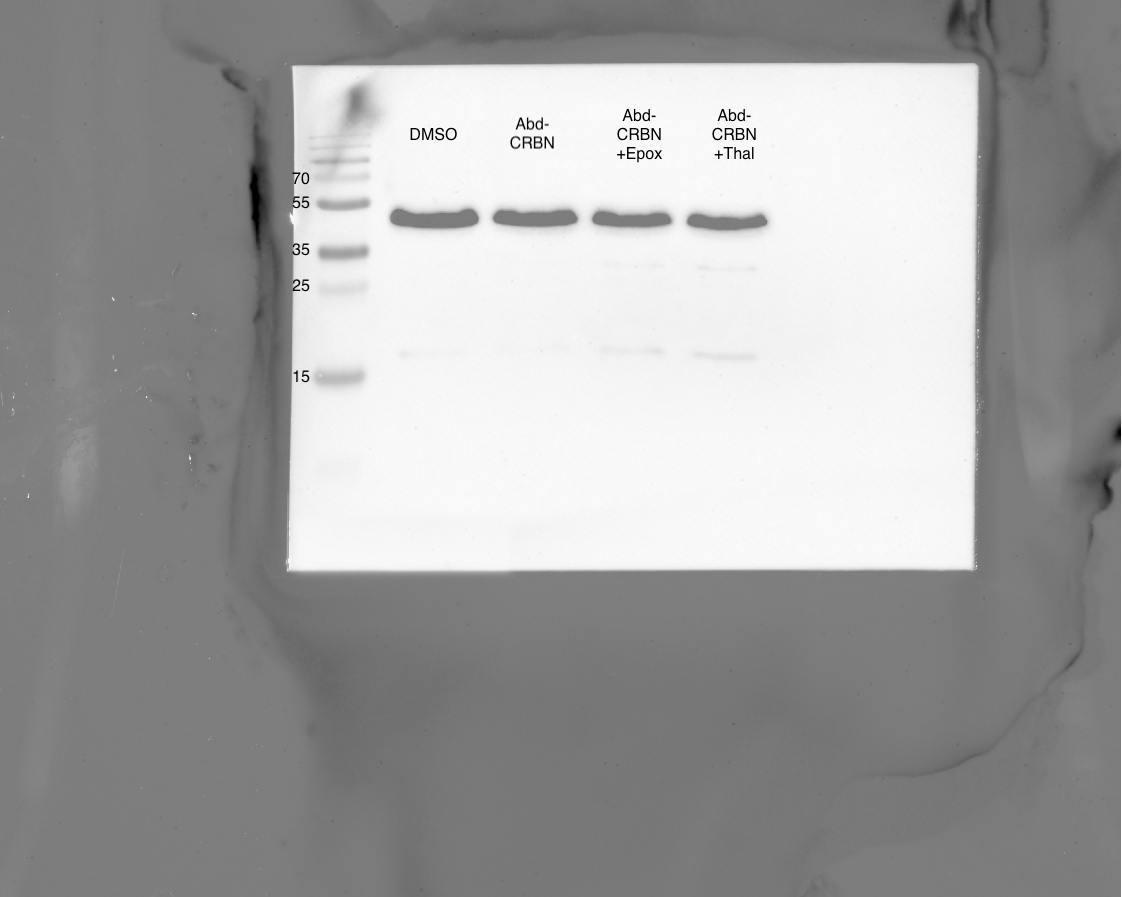

Supplement: Figure 3—source data 7. [file elife-106699-fig3-data7.zip › Figure 3ΓÇösource data 7 PDF files containing original western blots for Figure 3D, indicating the relevant bands and treatments./Raw data/GATA3 KOPT-K1 Abd-CRBN.tif]

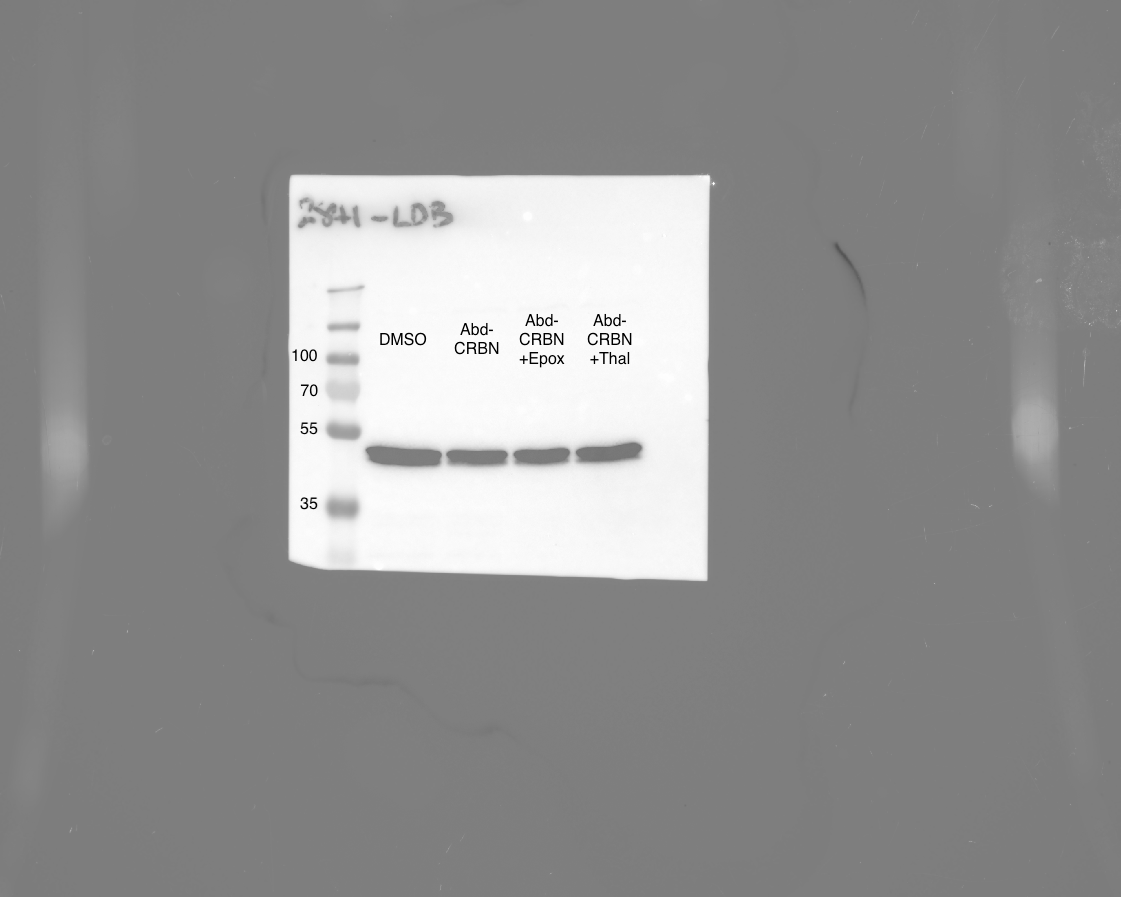

Supplement: Figure 3—source data 7. [file elife-106699-fig3-data7.zip › Figure 3ΓÇösource data 7 PDF files containing original western blots for Figure 3D, indicating the relevant bands and treatments./Raw data/LDB1 CCRF-CEM Abd-CRBN.tif]

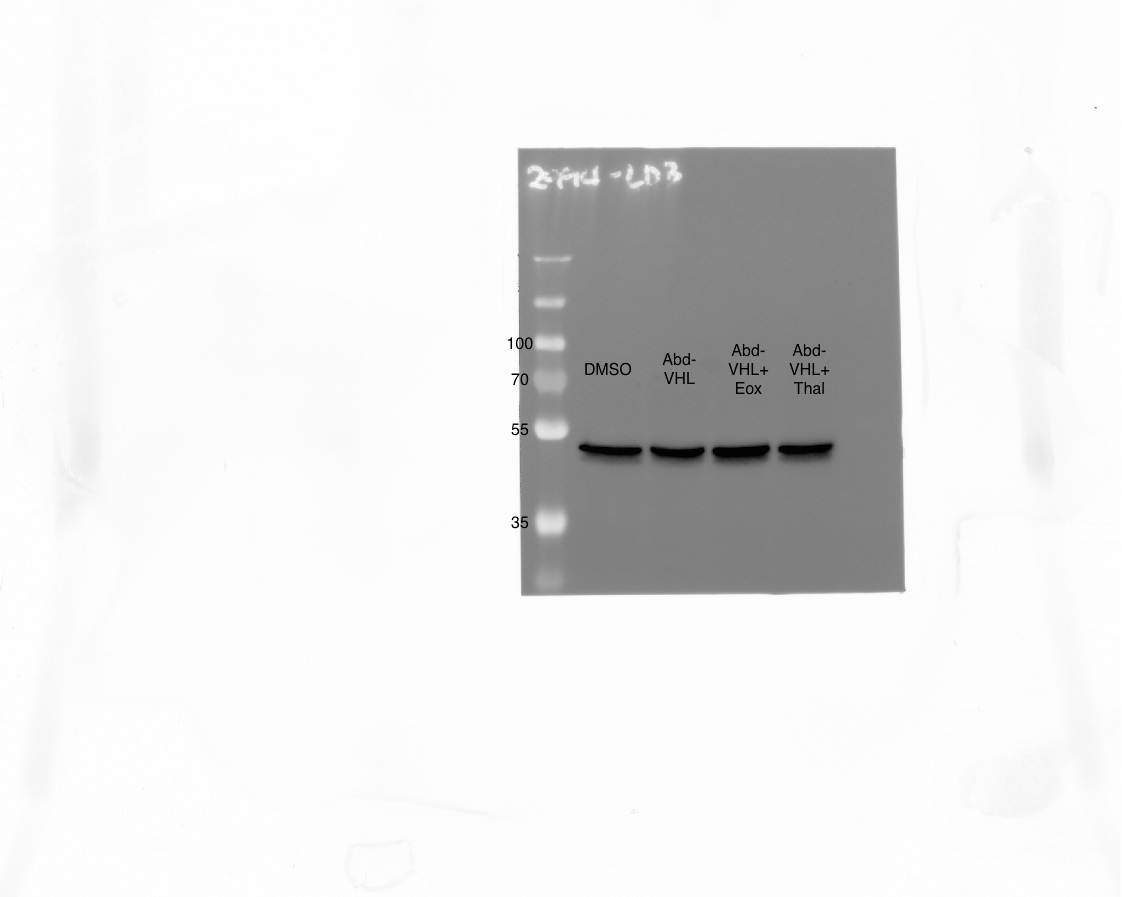

Supplement: Figure 3—source data 7. [file elife-106699-fig3-data7.zip › Figure 3ΓÇösource data 7 PDF files containing original western blots for Figure 3D, indicating the relevant bands and treatments./Raw data/LDB1 CCRF-CEM Abd-VHL.tif]

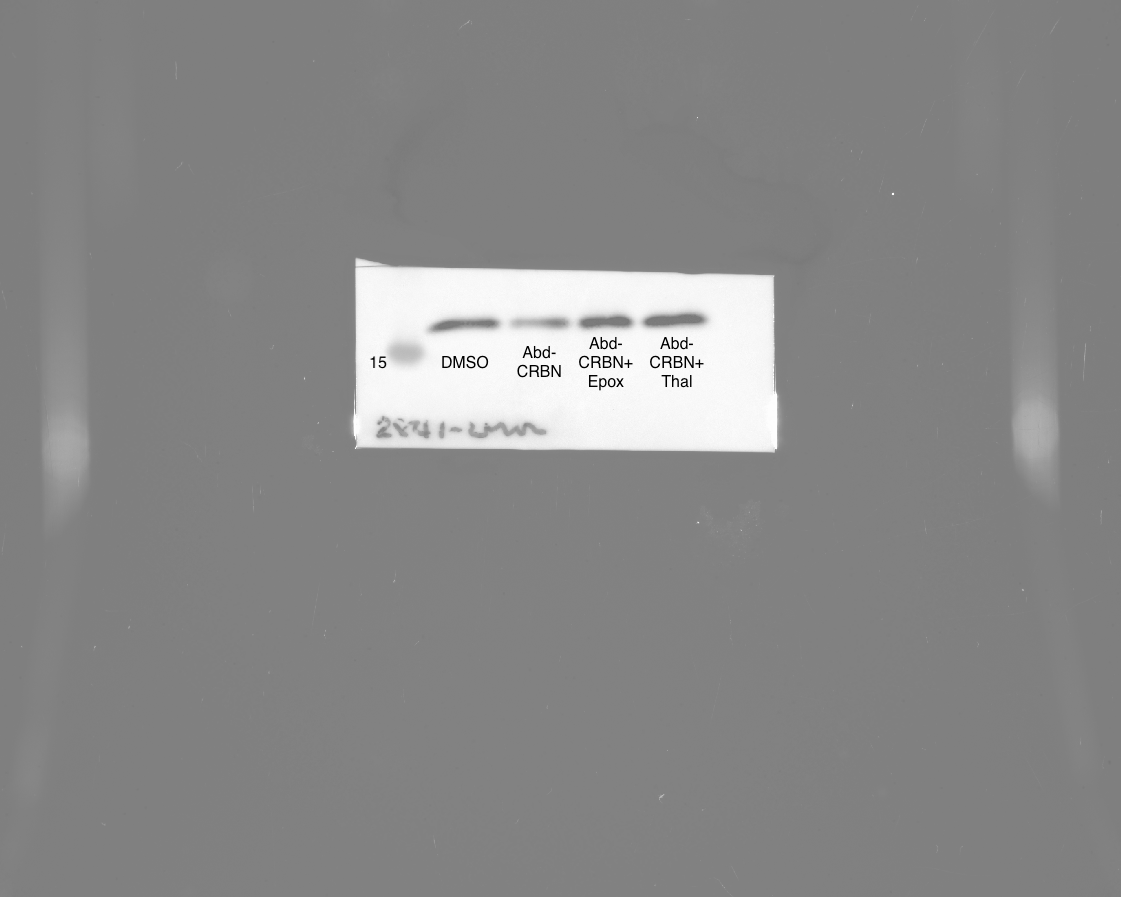

Supplement: Figure 3—source data 7. [file elife-106699-fig3-data7.zip › Figure 3ΓÇösource data 7 PDF files containing original western blots for Figure 3D, indicating the relevant bands and treatments./Raw data/LMO2 CCRF-CEM Abd-CRBN.tif]

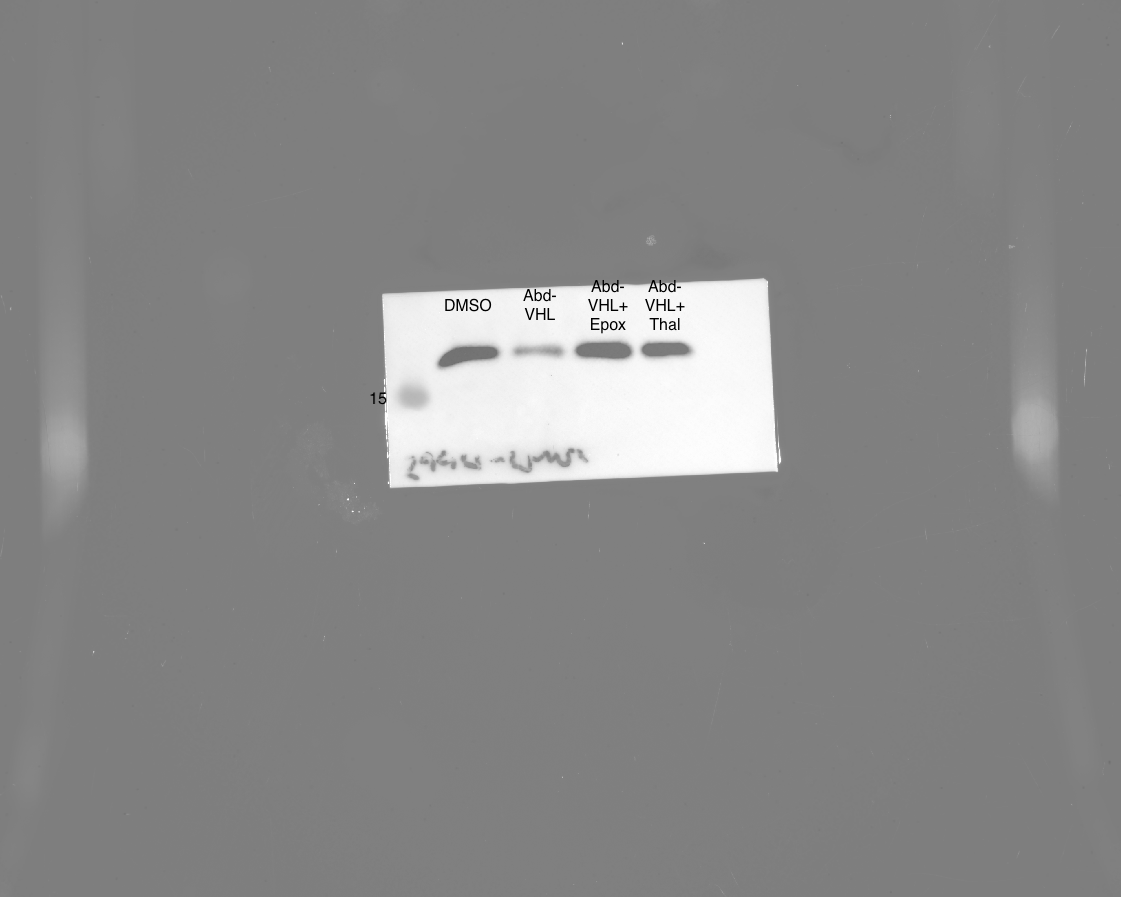

Supplement: Figure 3—source data 7. [file elife-106699-fig3-data7.zip › Figure 3ΓÇösource data 7 PDF files containing original western blots for Figure 3D, indicating the relevant bands and treatments./Raw data/LMO2 CCRF-CEM Abd-VHL.tif]

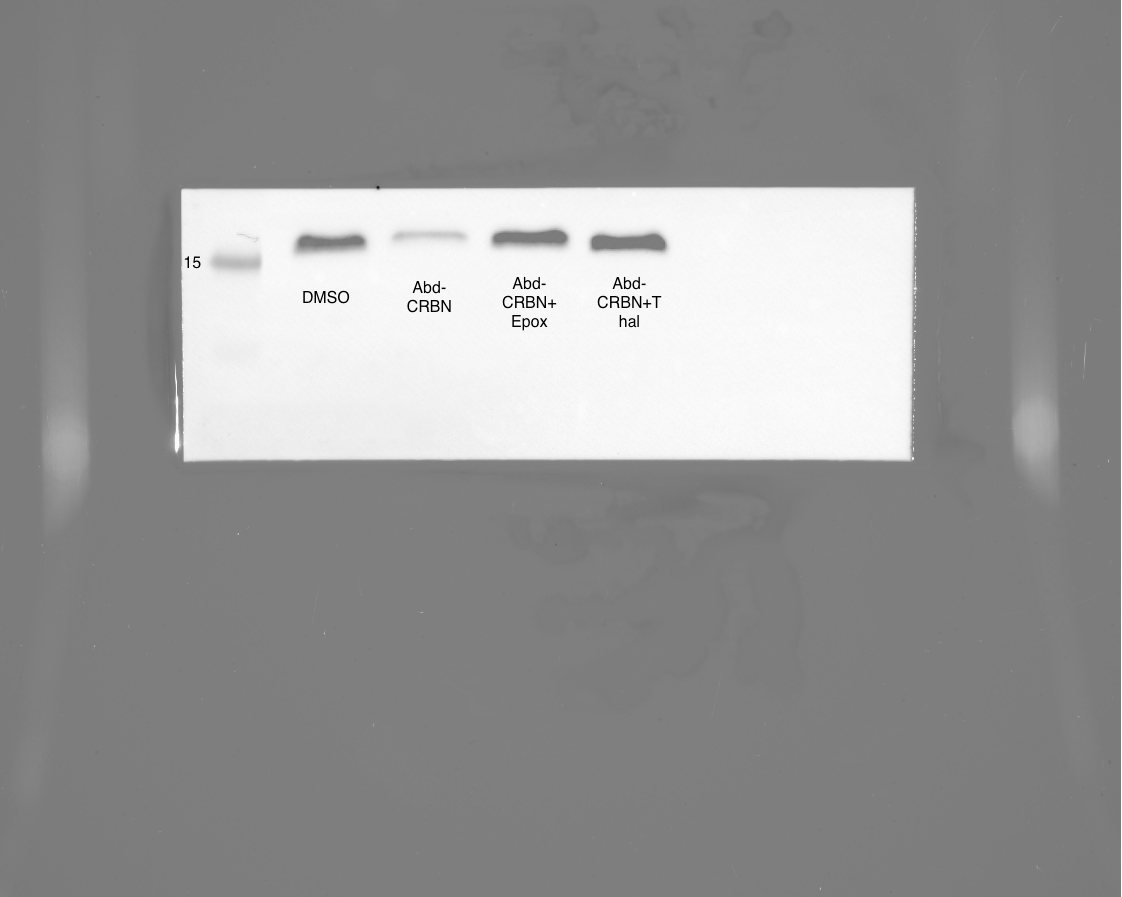

Supplement: Figure 3—source data 7. [file elife-106699-fig3-data7.zip › Figure 3ΓÇösource data 7 PDF files containing original western blots for Figure 3D, indicating the relevant bands and treatments./Raw data/LMO2 KOPT-K1 Abd-CRBN.tif]

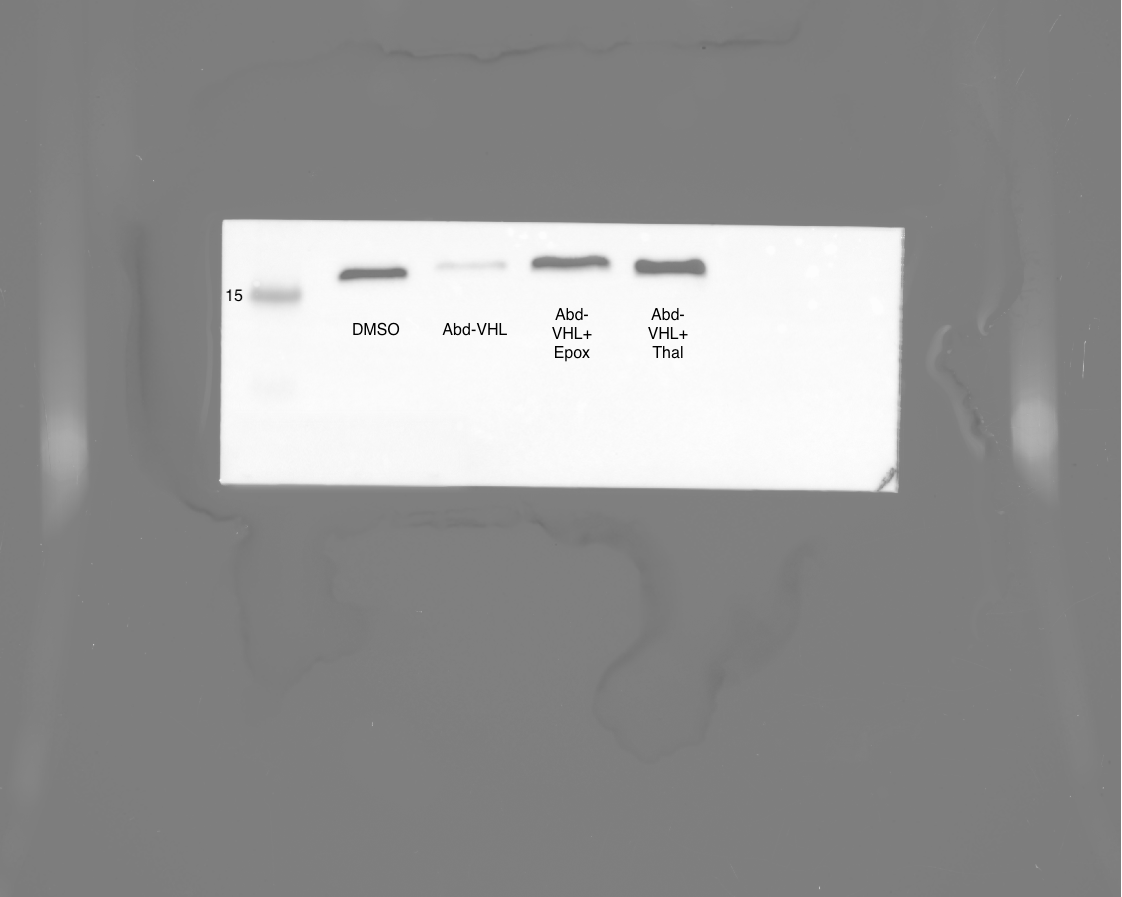

Supplement: Figure 3—source data 7. [file elife-106699-fig3-data7.zip › Figure 3ΓÇösource data 7 PDF files containing original western blots for Figure 3D, indicating the relevant bands and treatments./Raw data/LMO2 KOPT-K1 Abd-VHL.tif]

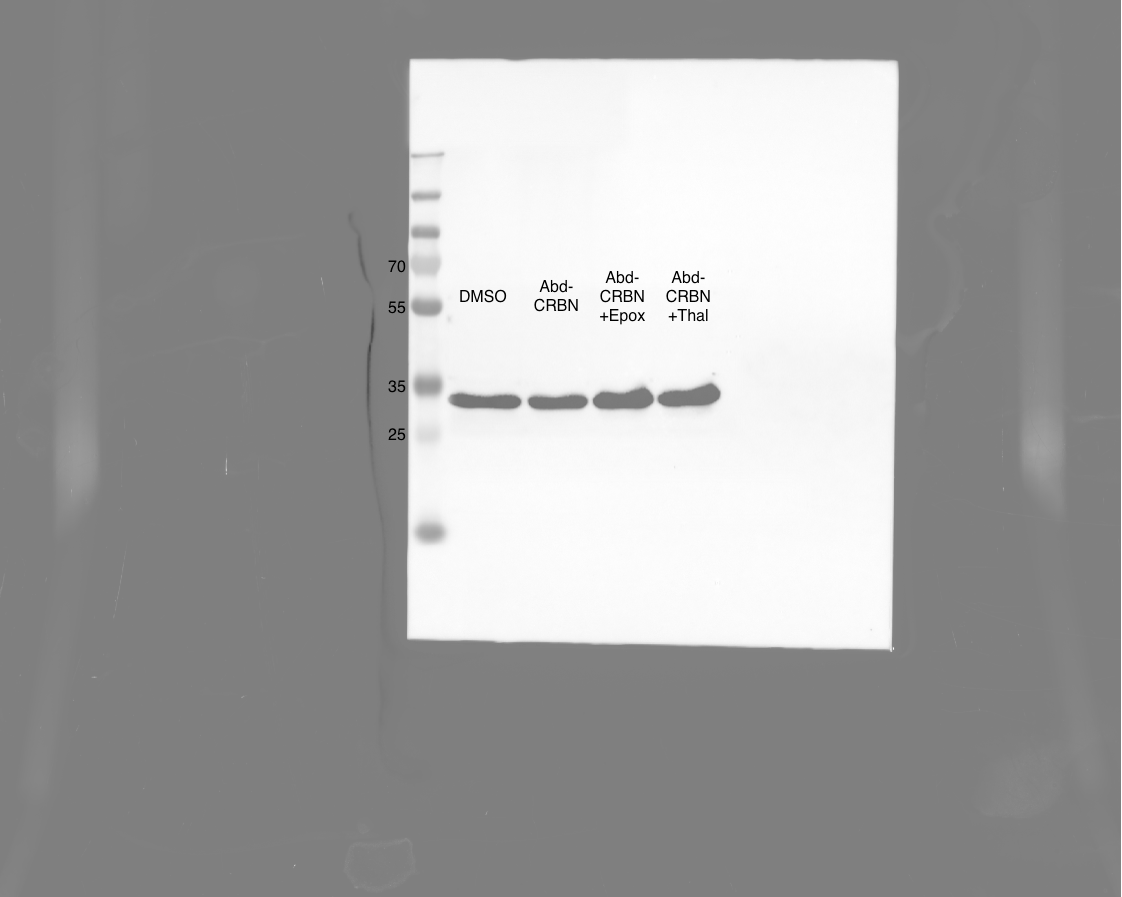

Supplement: Figure 3—source data 7. [file elife-106699-fig3-data7.zip › Figure 3ΓÇösource data 7 PDF files containing original western blots for Figure 3D, indicating the relevant bands and treatments./Raw data/Lyl1 CCRF-CEM Abd-CRBN.tif]

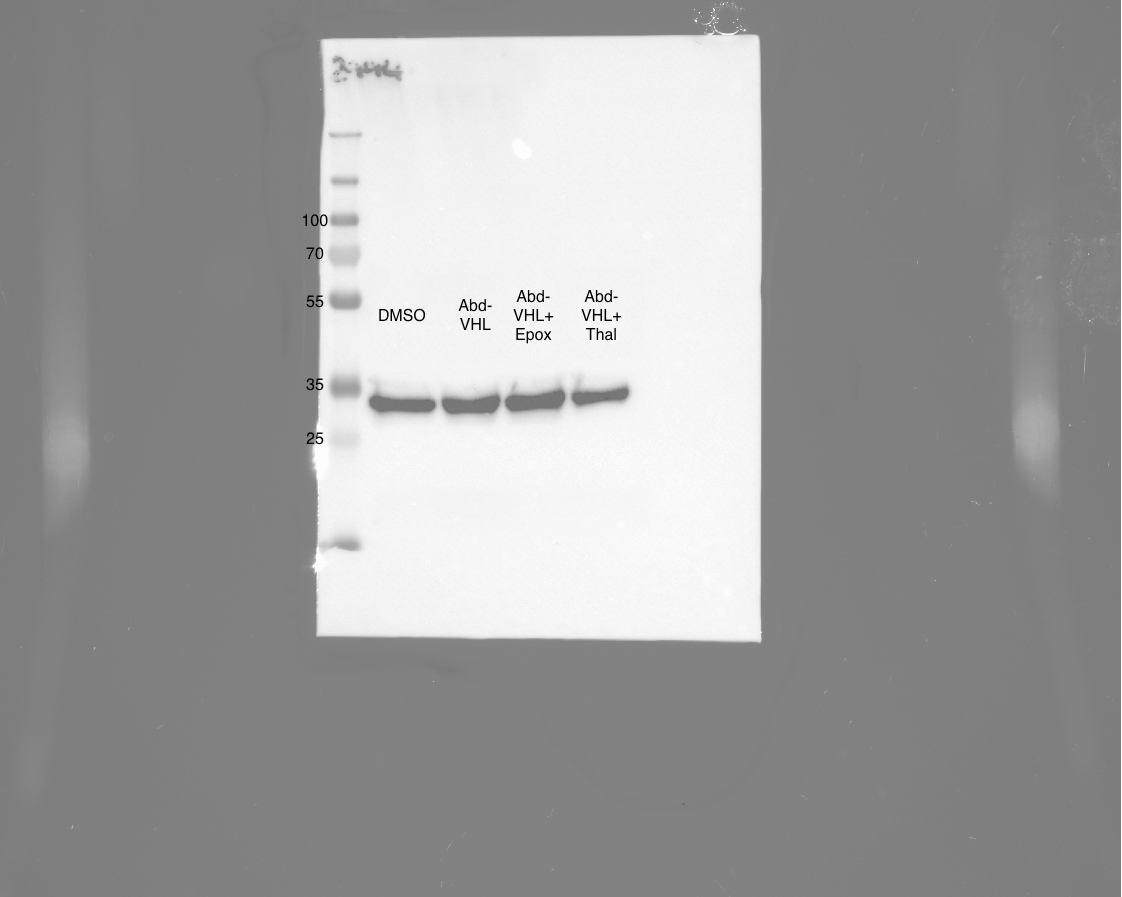

Supplement: Figure 3—source data 7. [file elife-106699-fig3-data7.zip › Figure 3ΓÇösource data 7 PDF files containing original western blots for Figure 3D, indicating the relevant bands and treatments./Raw data/Lyl1 CCRF-CEM Abd-VHL.tif]

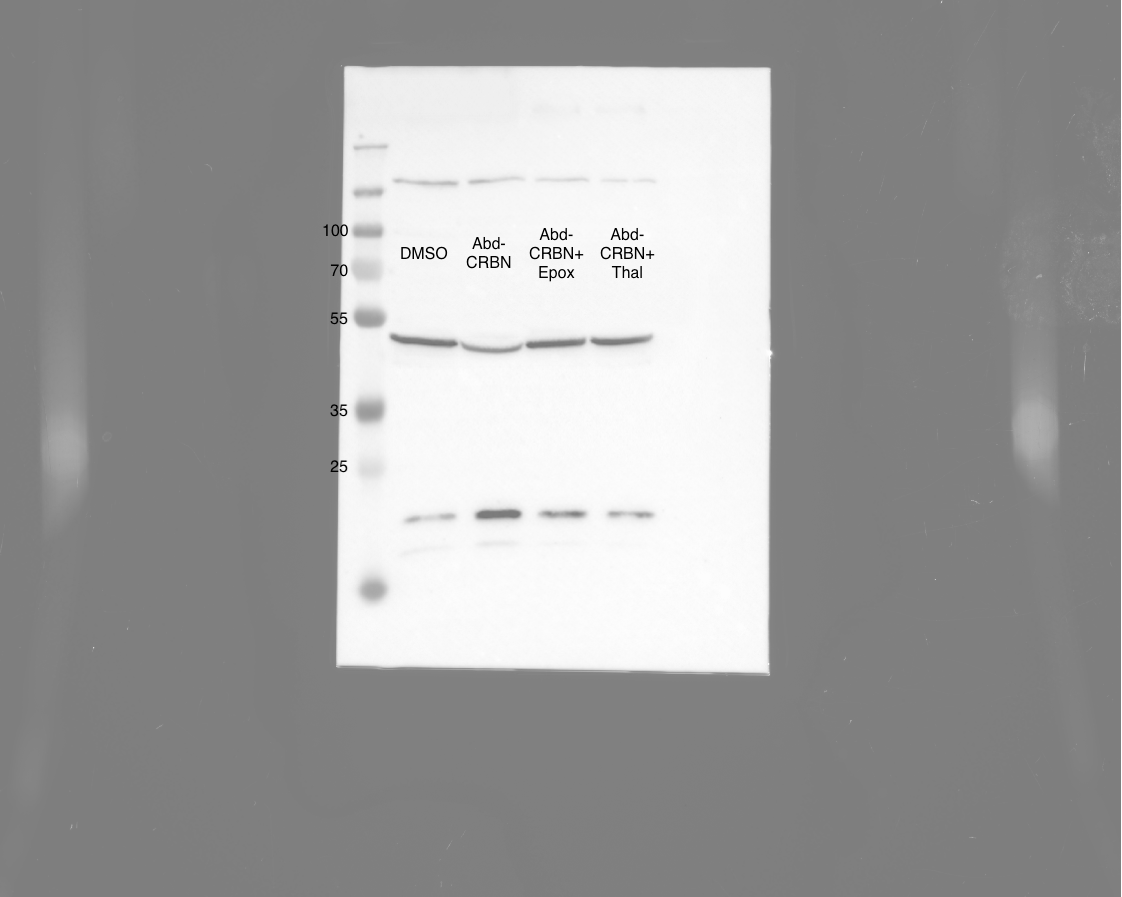

Supplement: Figure 3—source data 7. [file elife-106699-fig3-data7.zip › Figure 3ΓÇösource data 7 PDF files containing original western blots for Figure 3D, indicating the relevant bands and treatments./Raw data/Tal-1 CCRF-CEM Abd-CRBN.tif]

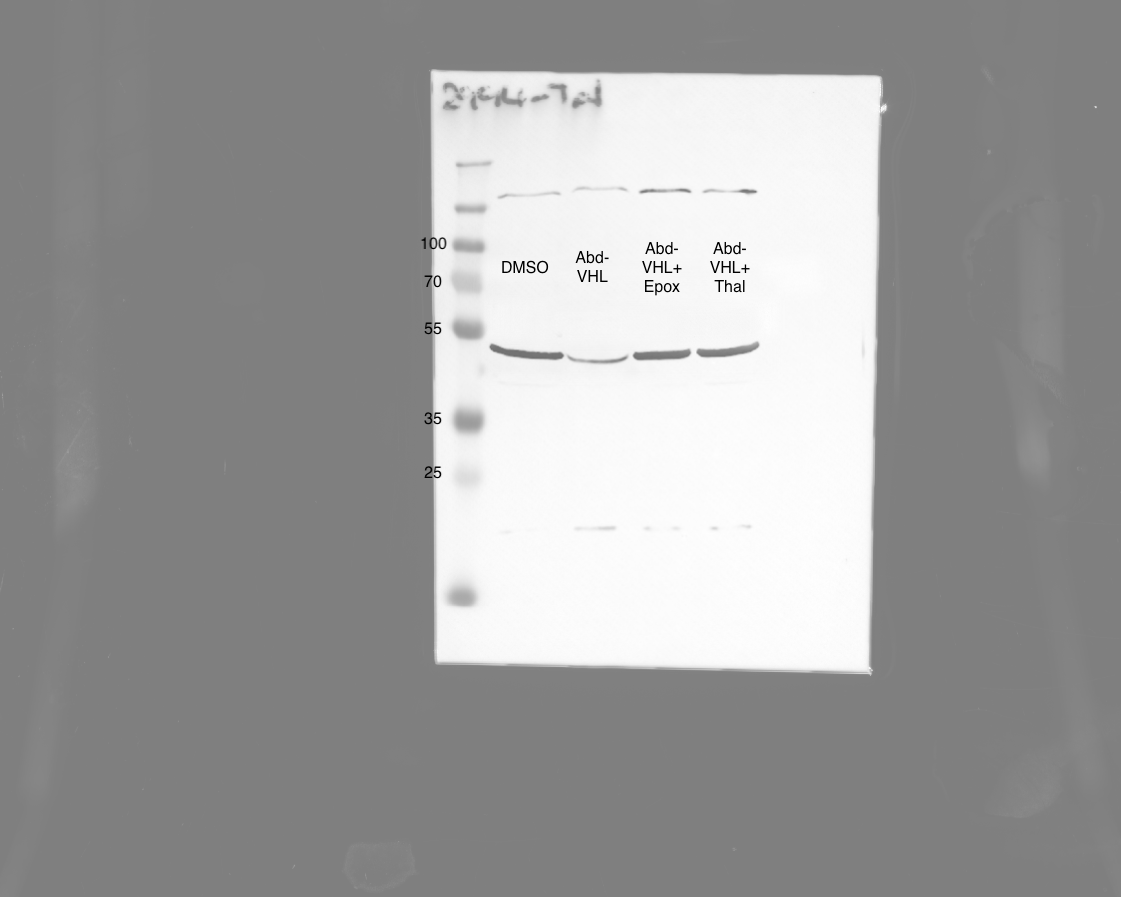

Supplement: Figure 3—source data 7. [file elife-106699-fig3-data7.zip › Figure 3ΓÇösource data 7 PDF files containing original western blots for Figure 3D, indicating the relevant bands and treatments./Raw data/Tal1 CCRF-CEM Abd-VHL.tif]

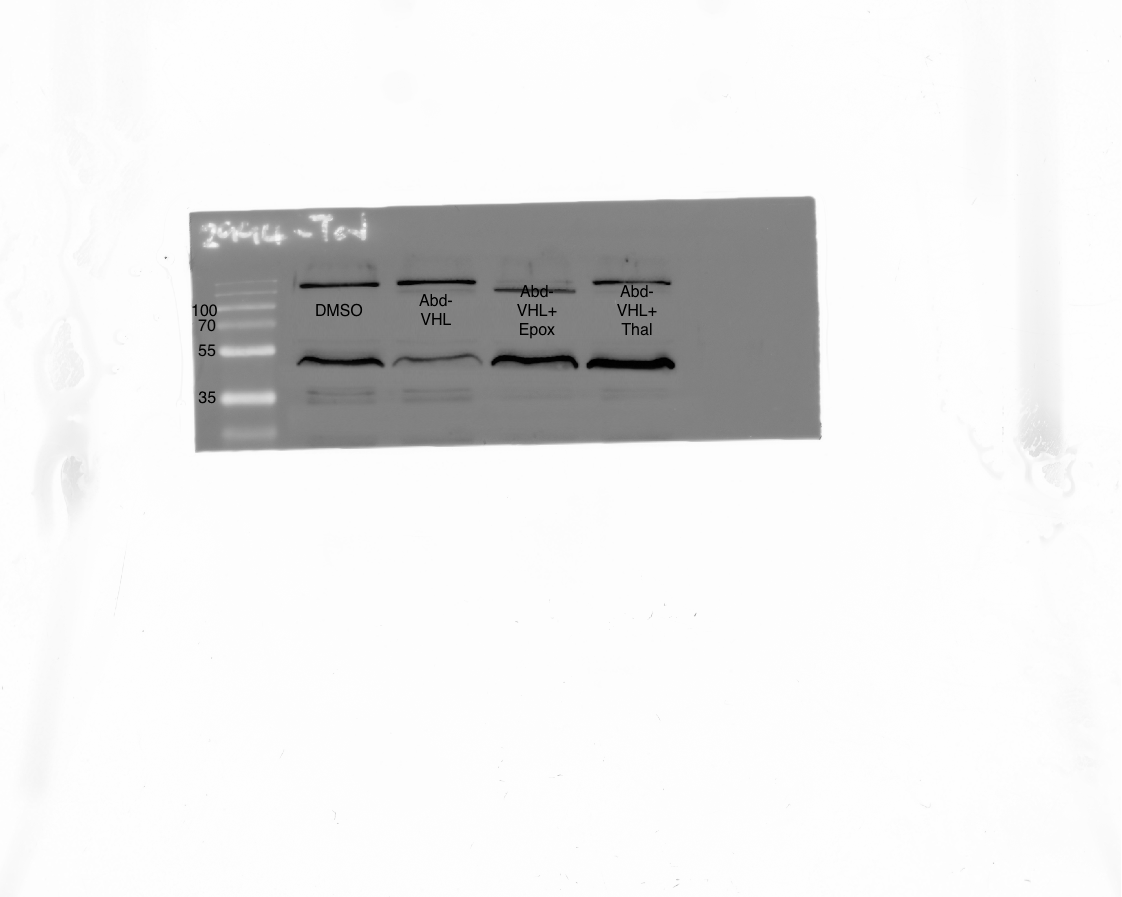

Supplement: Figure 3—source data 7. [file elife-106699-fig3-data7.zip › Figure 3ΓÇösource data 7 PDF files containing original western blots for Figure 3D, indicating the relevant bands and treatments./Raw data/Tal1 KOPT-K1 Abd-VHL.tif]

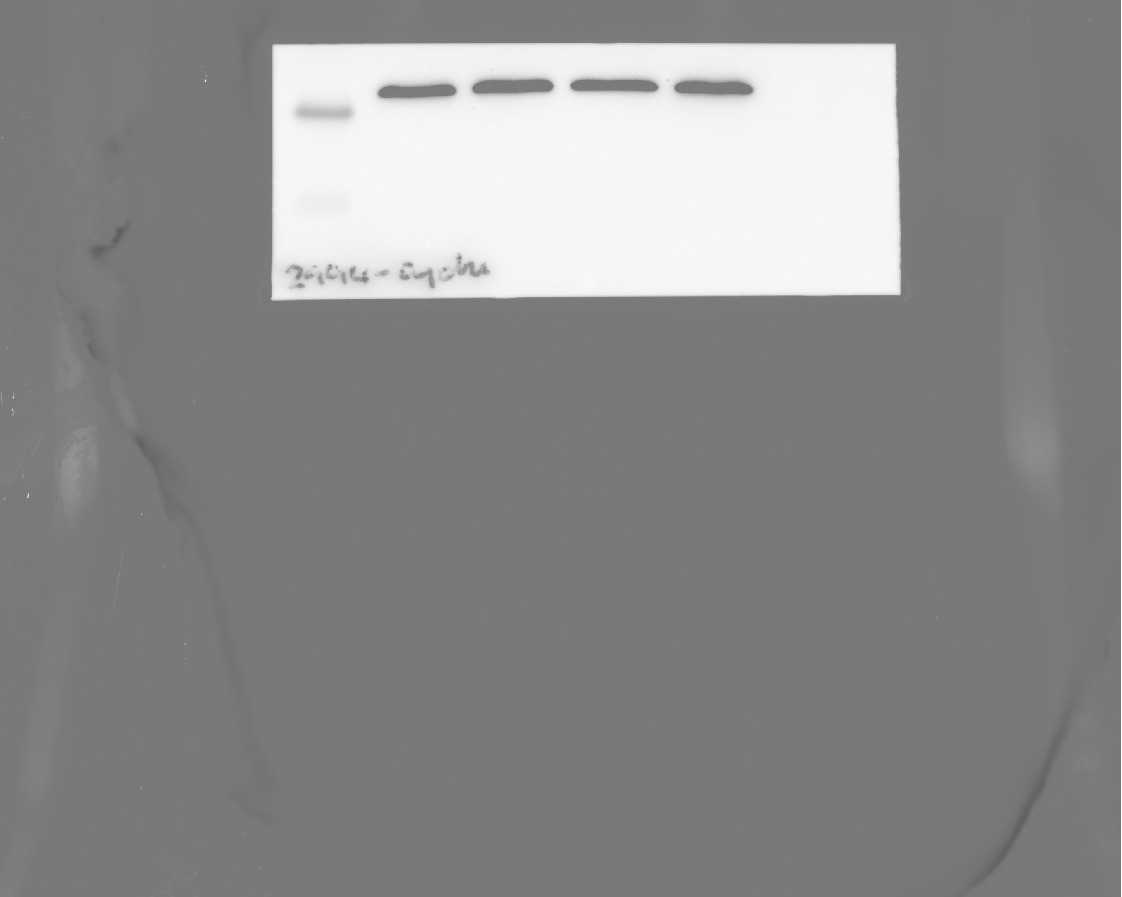

Supplement: Figure 3—source data 8. [file elife-106699-fig3-data8.zip › Figure 3ΓÇösource data 8 Original files for Western blot analysis displayed in Figure 3D./Cyclophilin KOPT-K1 Abd-VHL.tif]

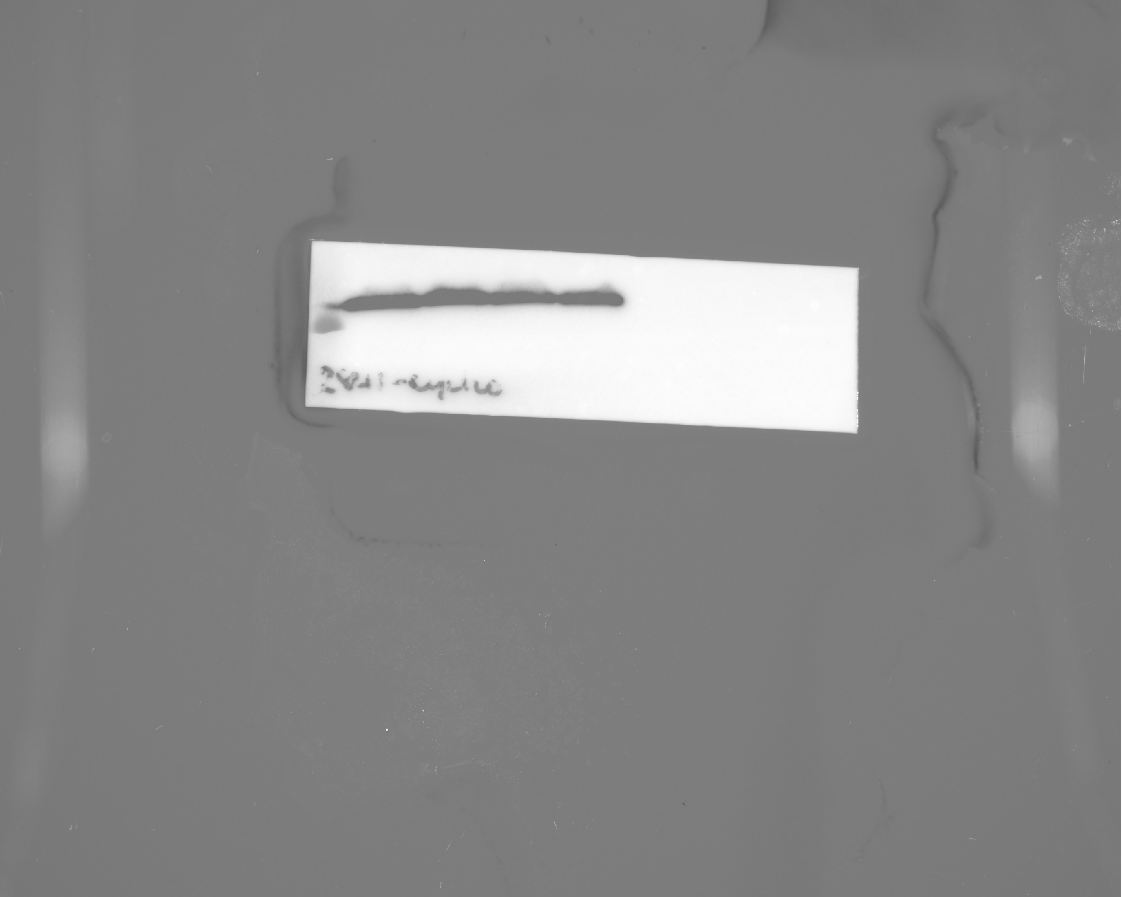

Supplement: Figure 3—source data 8. [file elife-106699-fig3-data8.zip › Figure 3ΓÇösource data 8 Original files for Western blot analysis displayed in Figure 3D./Cyclophilin CCRF-CEM Abd-CRBN.tif]

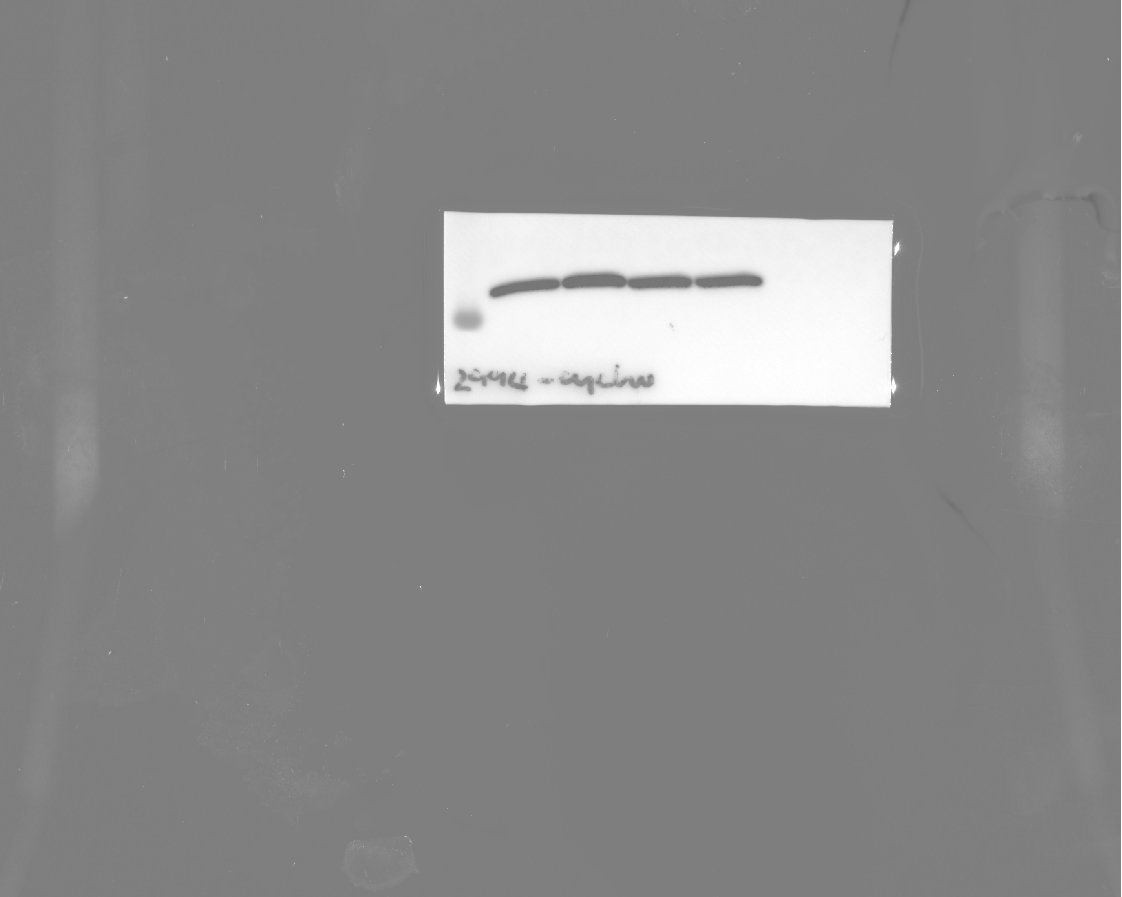

Supplement: Figure 3—source data 8. [file elife-106699-fig3-data8.zip › Figure 3ΓÇösource data 8 Original files for Western blot analysis displayed in Figure 3D./Cyclophilin CCRF-CEM Abd-VHL.tif]

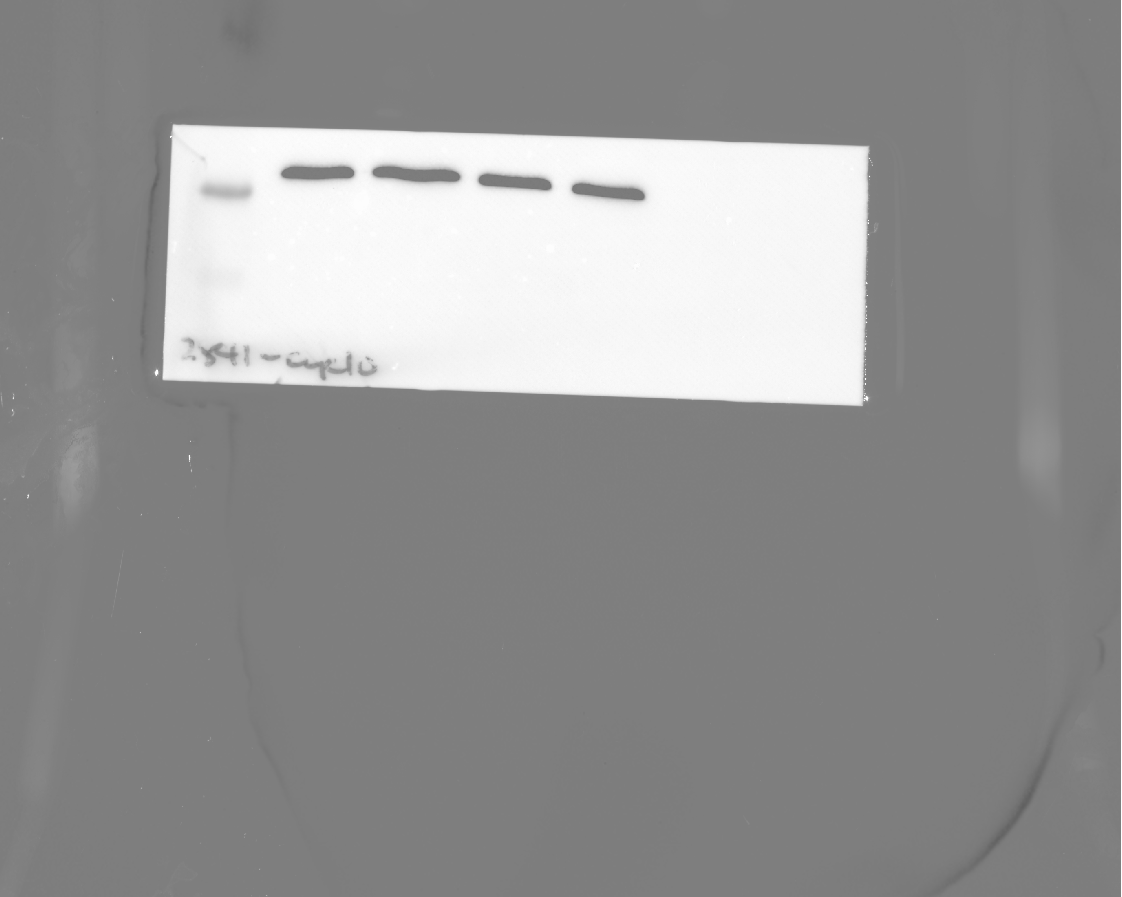

Supplement: Figure 3—source data 8. [file elife-106699-fig3-data8.zip › Figure 3ΓÇösource data 8 Original files for Western blot analysis displayed in Figure 3D./Cyclophilin KOPT-K1 Abd-CRBN.tif]
