# Supplementary material for: Degradation of LMO2 in T cell leukaemia results in collateral breakdown of transcription complex partners and causes LMO2-dependent apoptosis
Source: eLife. 2025 Dec 12;14:RP106699. doi: 10.7554/eLife.106699 (PMC12700530; doi:10.7554/eLife.106699)
Supplement: Figure 3—source data 5. [file elife-106699-fig3-data5.zip › Figure 3ΓÇösource data 5 PDF files containing original western blots for Figure 3C, indicating the relevant bands and treatments./Figure3-source data 5.pdf]

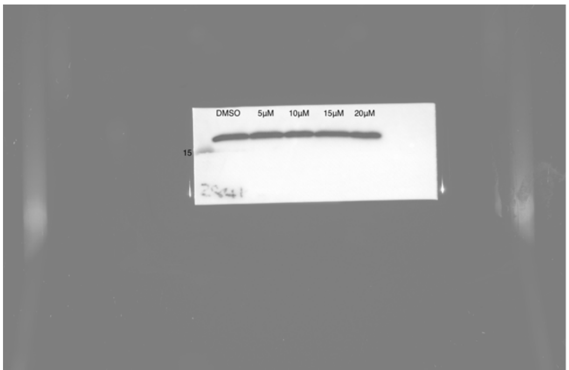

Cyclophilin-b in CCRF-CEM
treated with Abd-CRBN

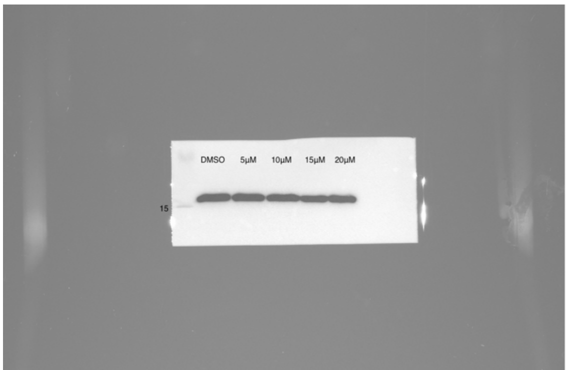

Cyclophilin-b in CCRF-CEM
treated with Abd-VHL

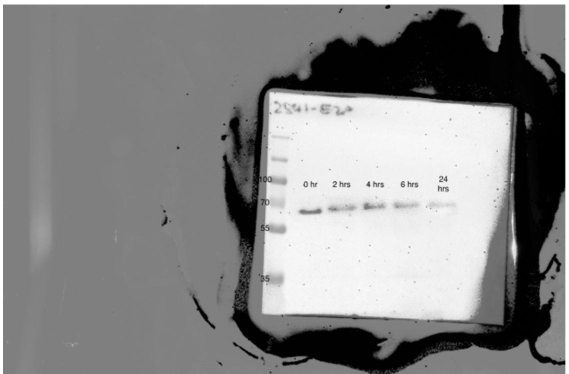

E47 in CCRF-CEM
treated with Abd-CRBN

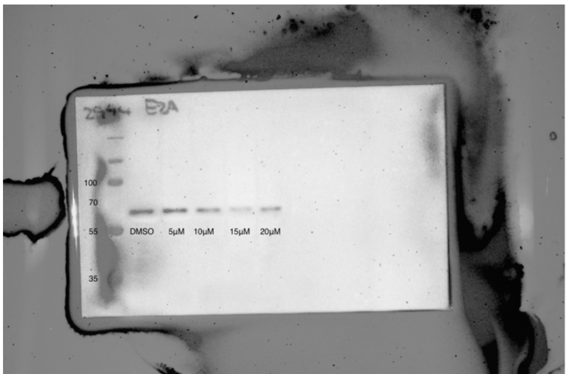

E47 in CCRF-CEM
treated with Abd-VHL

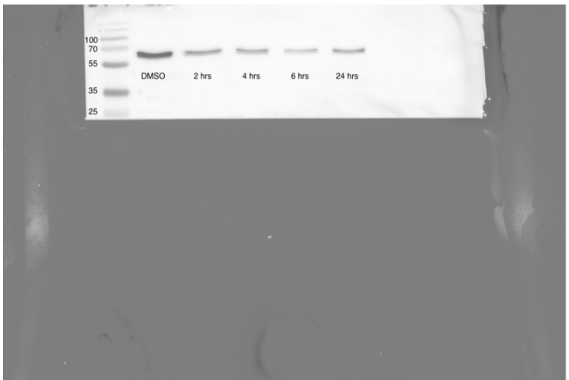

E47 in KOPT-K1
treated with Abd-CRBN

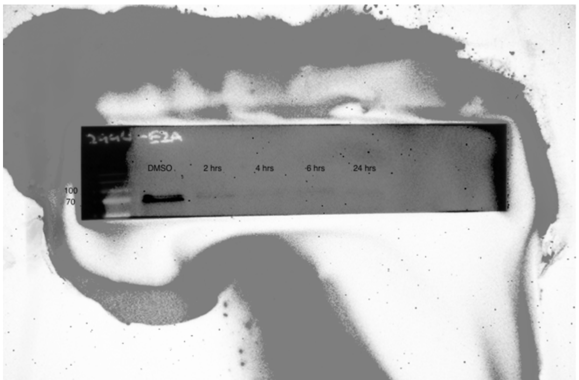

E47 in KOPT-K1
treated with Abd-VHL

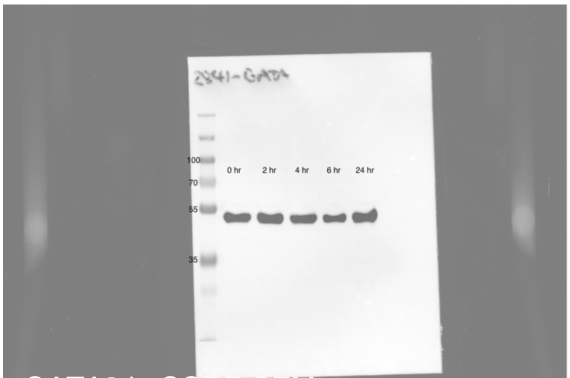

GATA3 in CCRF-CEM
treated with Abd-CRBN

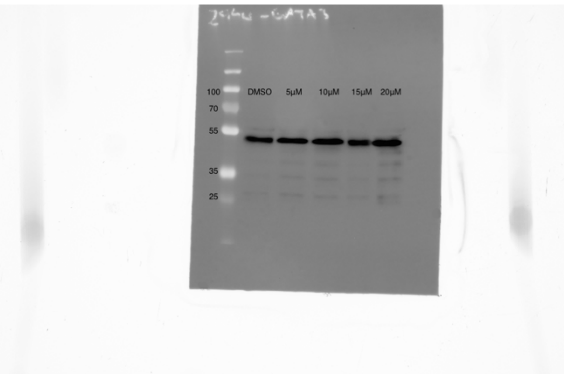

GATA3 in CCRF-CEM
treated with Abd-VHL

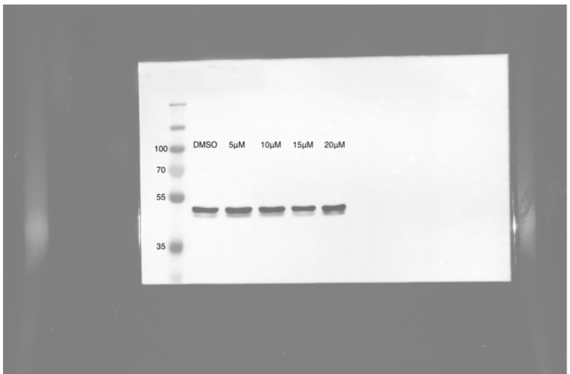

LDB1 in CCRF-CEM
treated with Abd-CRBN

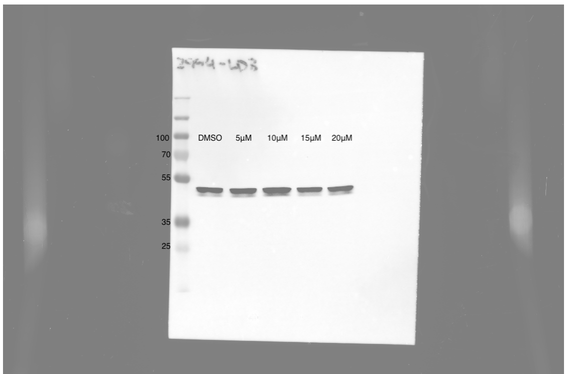

LDB1 in CCRF-CEM
treated with Abd-VHL

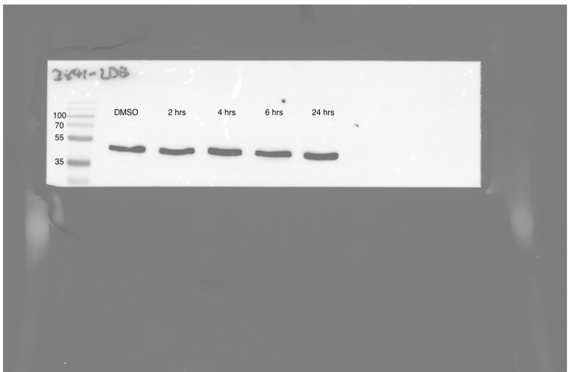

LDB1 in KOPT-K1
treated with Abd-CRBN

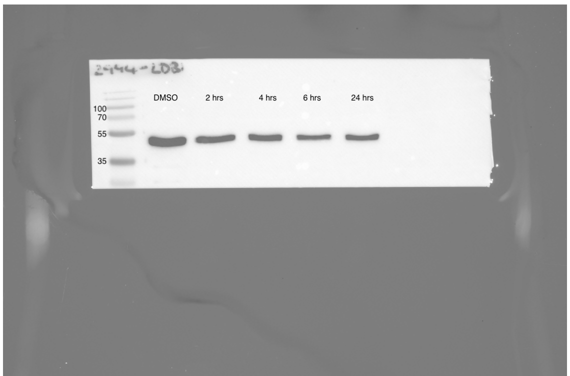

LDB1 in KOPT-K1
treated with Abd-VHL

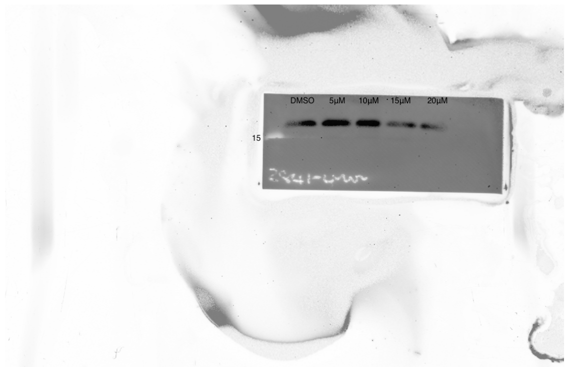

LMO2 in CCRF-CEM
treated with Abd-CRBN

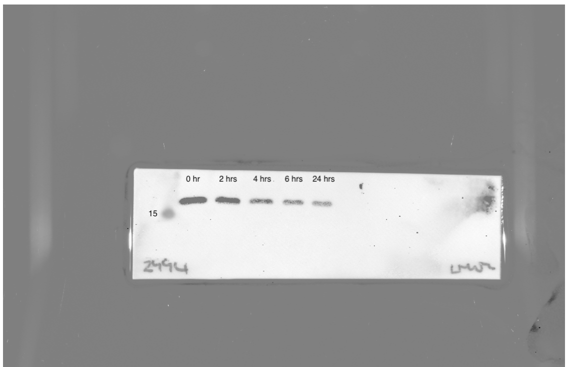

LMO2 in CCRF-CEM
treated with Abd-VHL

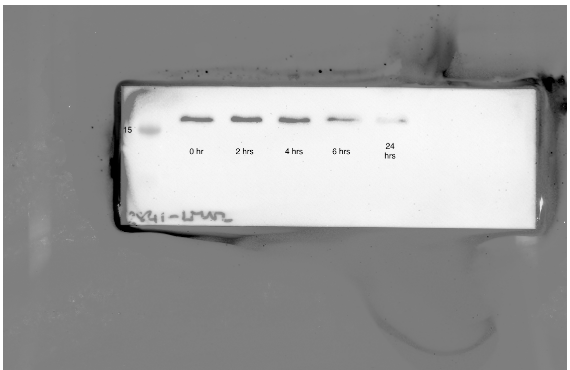

LMO2 in KOPT-K1
treated with Abd-CRBN

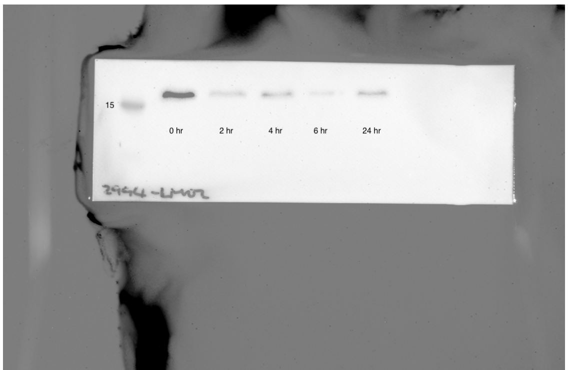

LMO2 in KOPT-K1
treated with Abd-VHL

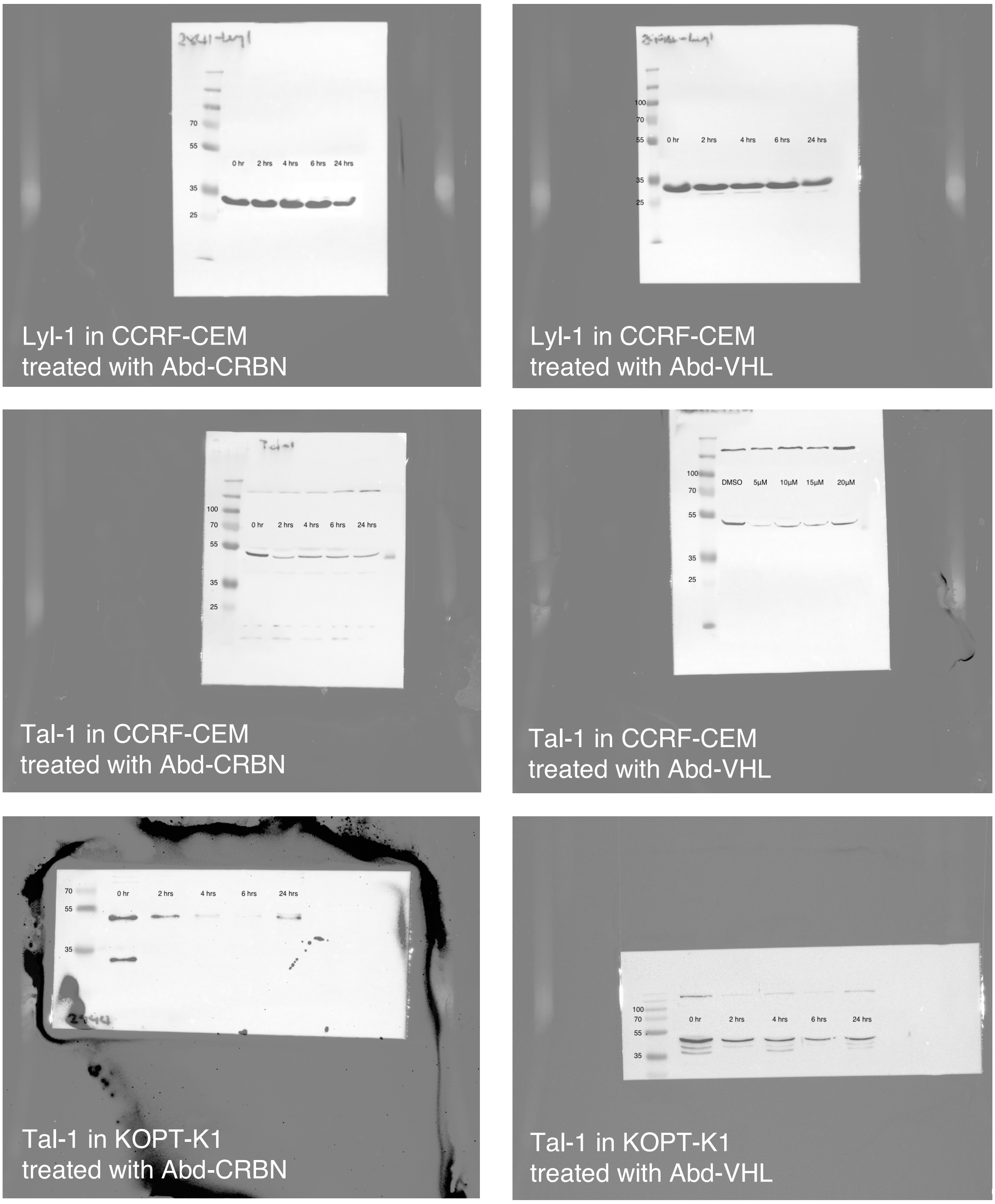

Figure 3, Source Data 5. Original membranes corresponding to Figure 3, panel C.
