# Supplementary figures and images for: Degradation of LMO2 in T cell leukaemia results in collateral breakdown of transcription complex partners and causes LMO2-dependent apoptosis (part 4 of 5)
Source: eLife. 2025 Dec 12;14:RP106699. doi: 10.7554/eLife.106699 (PMC12700530; doi:10.7554/eLife.106699)

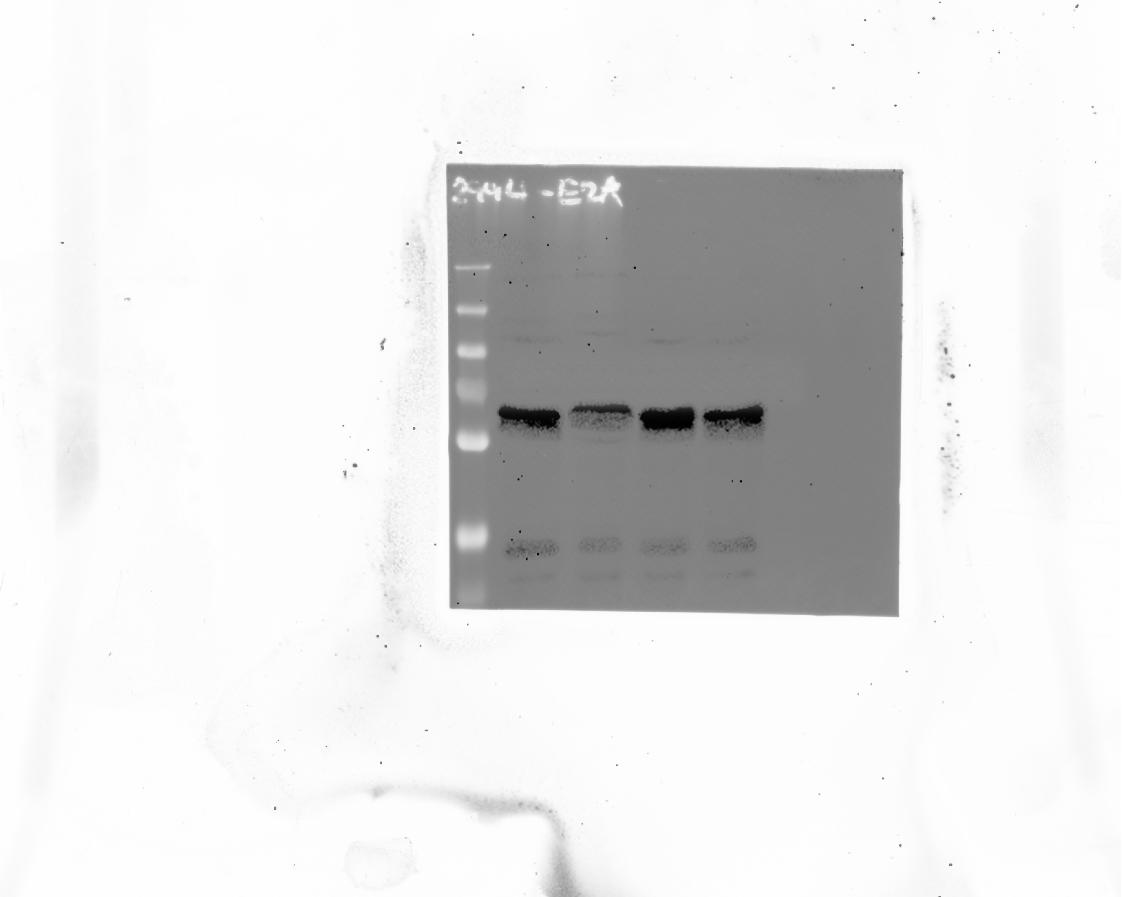

Supplement: Figure 3—source data 8. [file elife-106699-fig3-data8.zip › Figure 3ΓÇösource data 8 Original files for Western blot analysis displayed in Figure 3D./E2A CCRF-CEM Abd-VHL.tif]

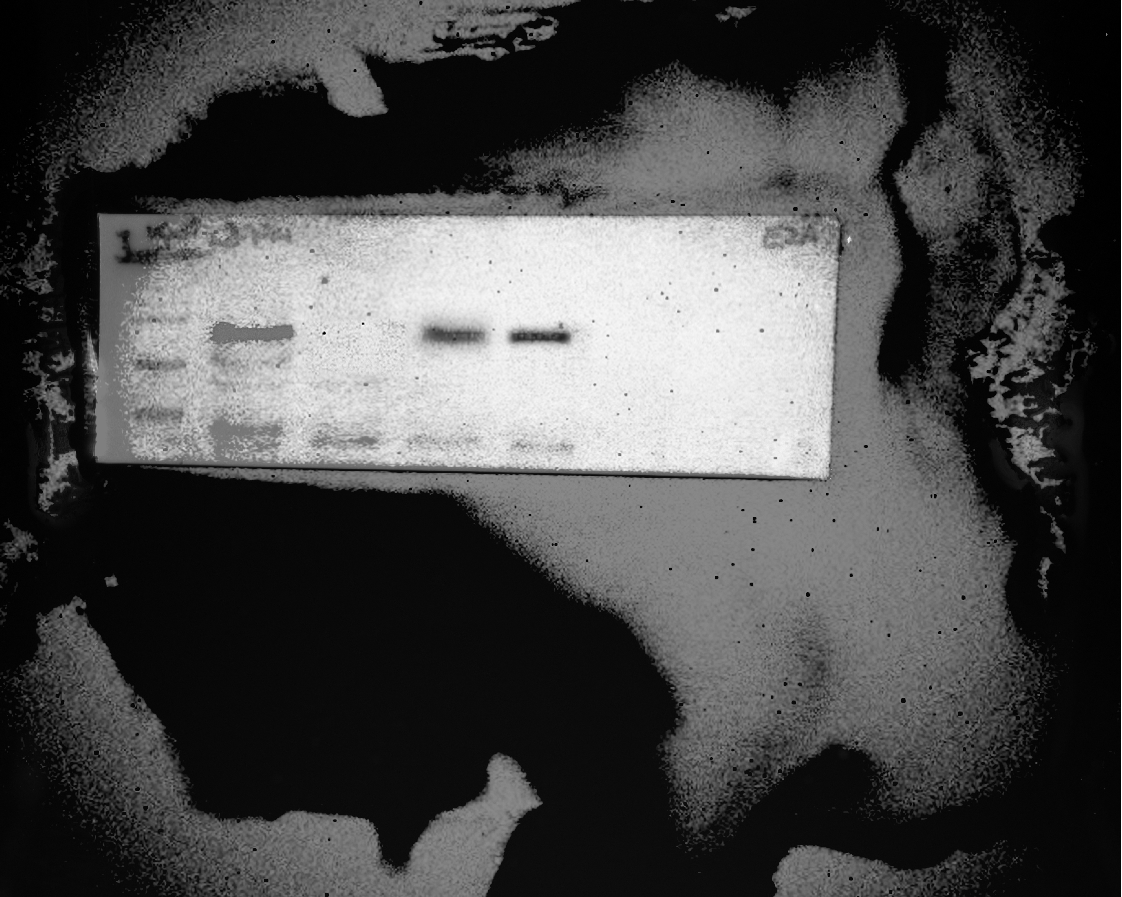

Supplement: Figure 3—source data 8. [file elife-106699-fig3-data8.zip › Figure 3ΓÇösource data 8 Original files for Western blot analysis displayed in Figure 3D./E2A KOPT-K1 Abd-CRBN.tif]

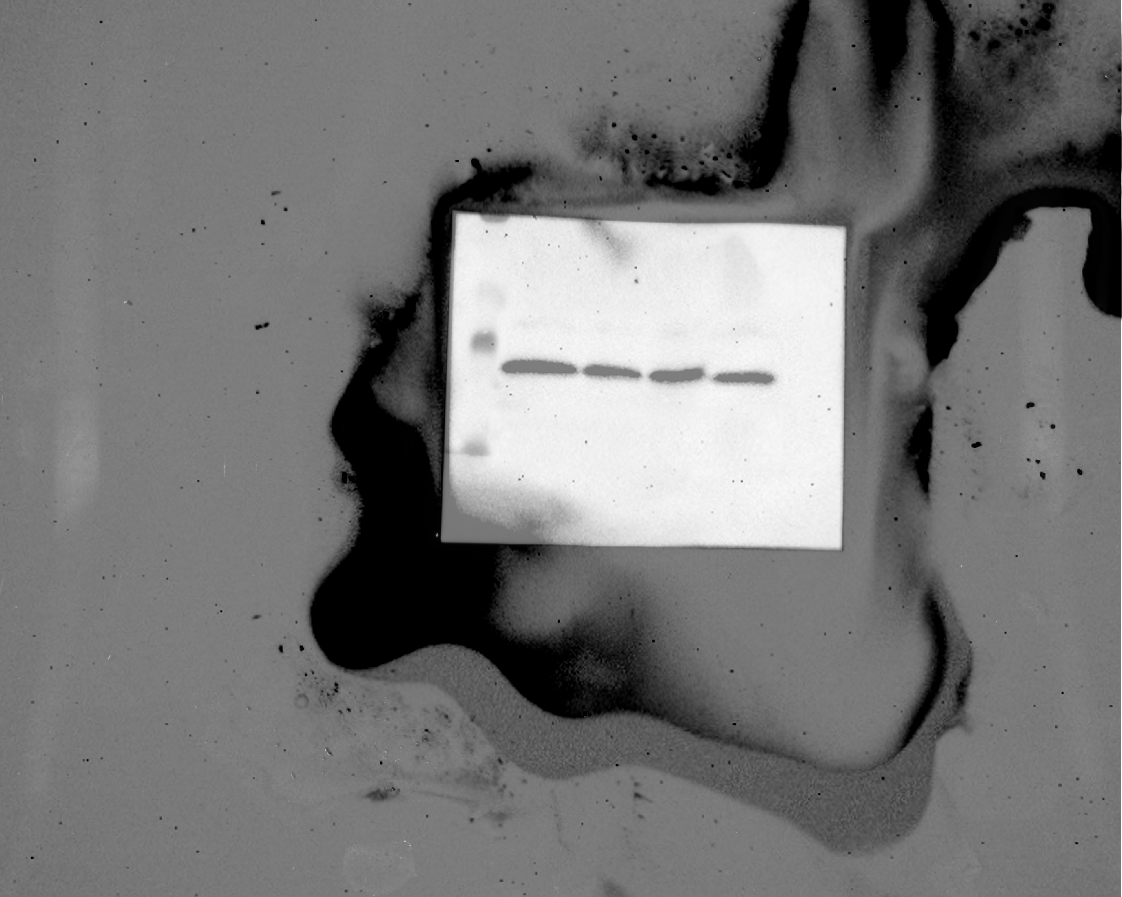

Supplement: Figure 3—source data 8. [file elife-106699-fig3-data8.zip › Figure 3ΓÇösource data 8 Original files for Western blot analysis displayed in Figure 3D./GATA3 CCRF-CEM Abd-CRBN.tif]

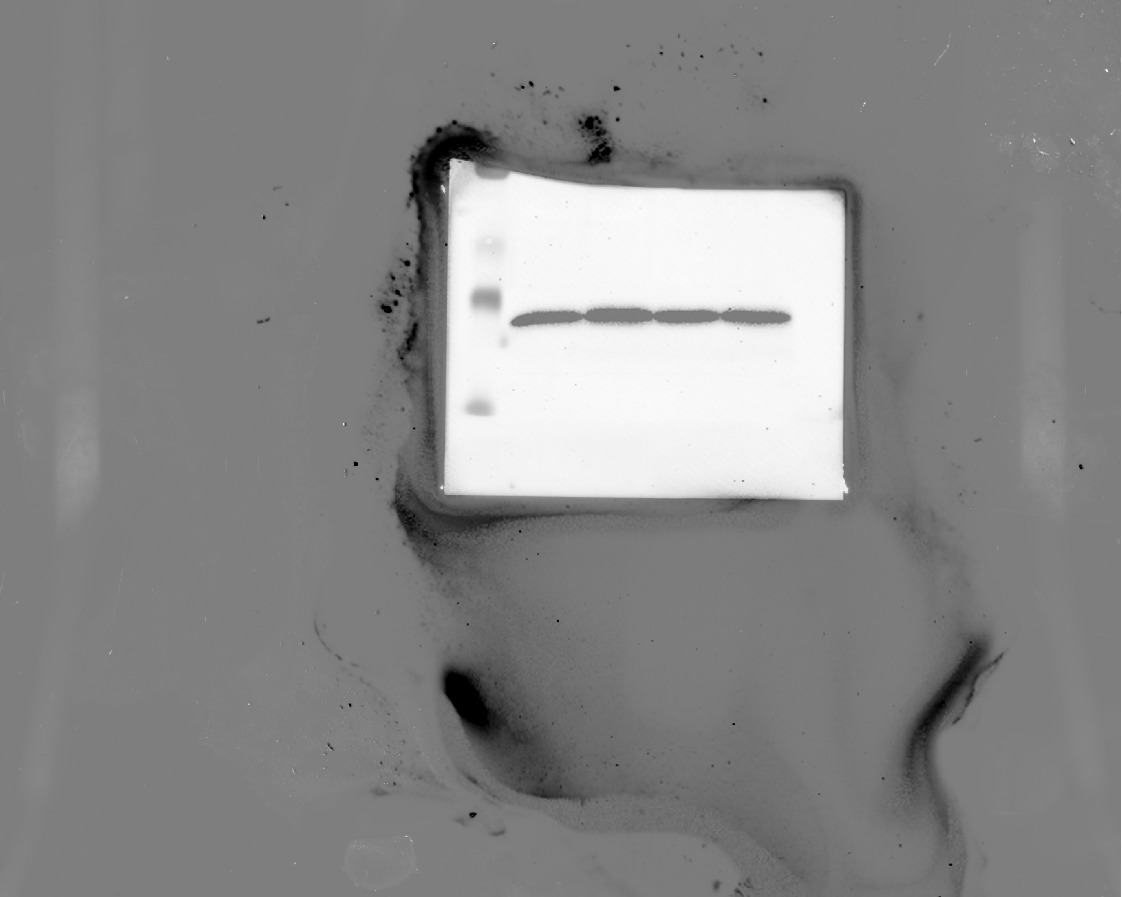

Supplement: Figure 3—source data 8. [file elife-106699-fig3-data8.zip › Figure 3ΓÇösource data 8 Original files for Western blot analysis displayed in Figure 3D./GATA3 CCRF-CEM Abd-VHL.tif]

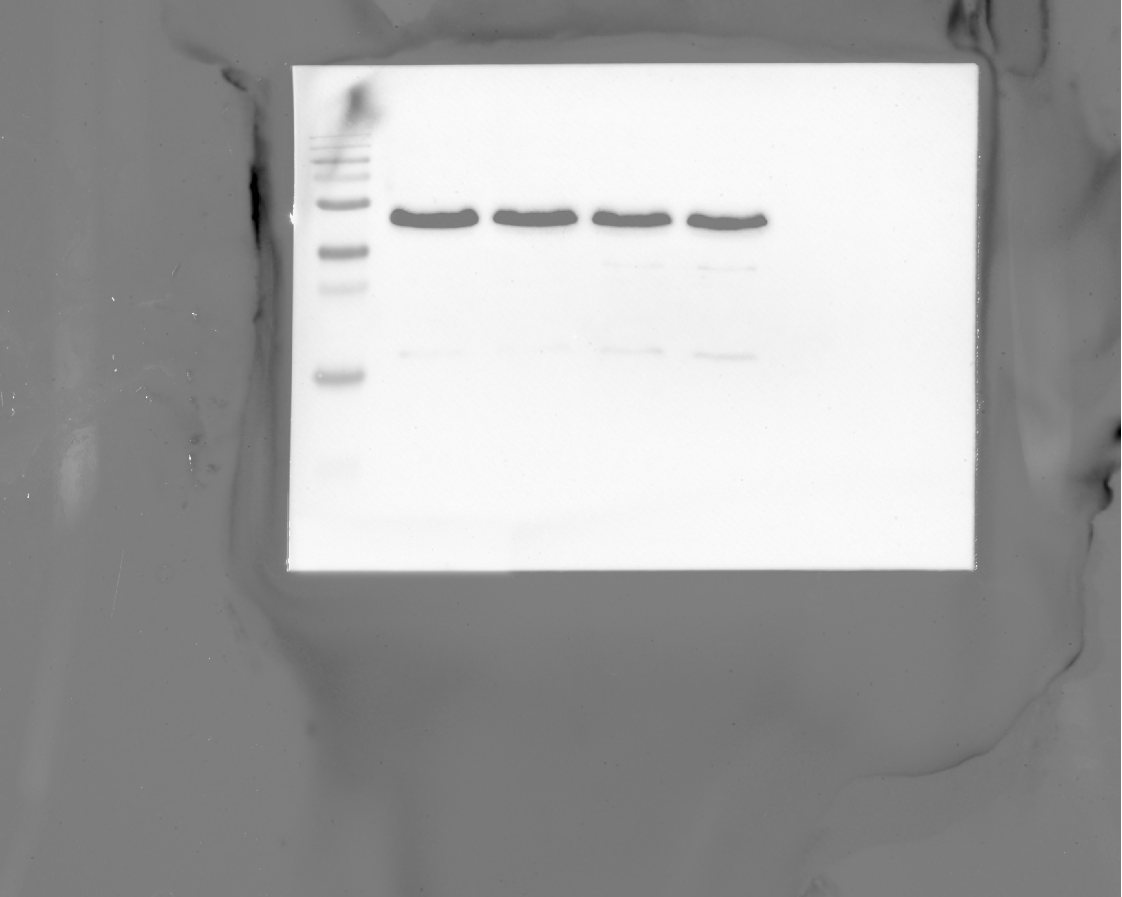

Supplement: Figure 3—source data 8. [file elife-106699-fig3-data8.zip › Figure 3ΓÇösource data 8 Original files for Western blot analysis displayed in Figure 3D./GATA3 KOPT-K1 Abd-CRBN.tif]

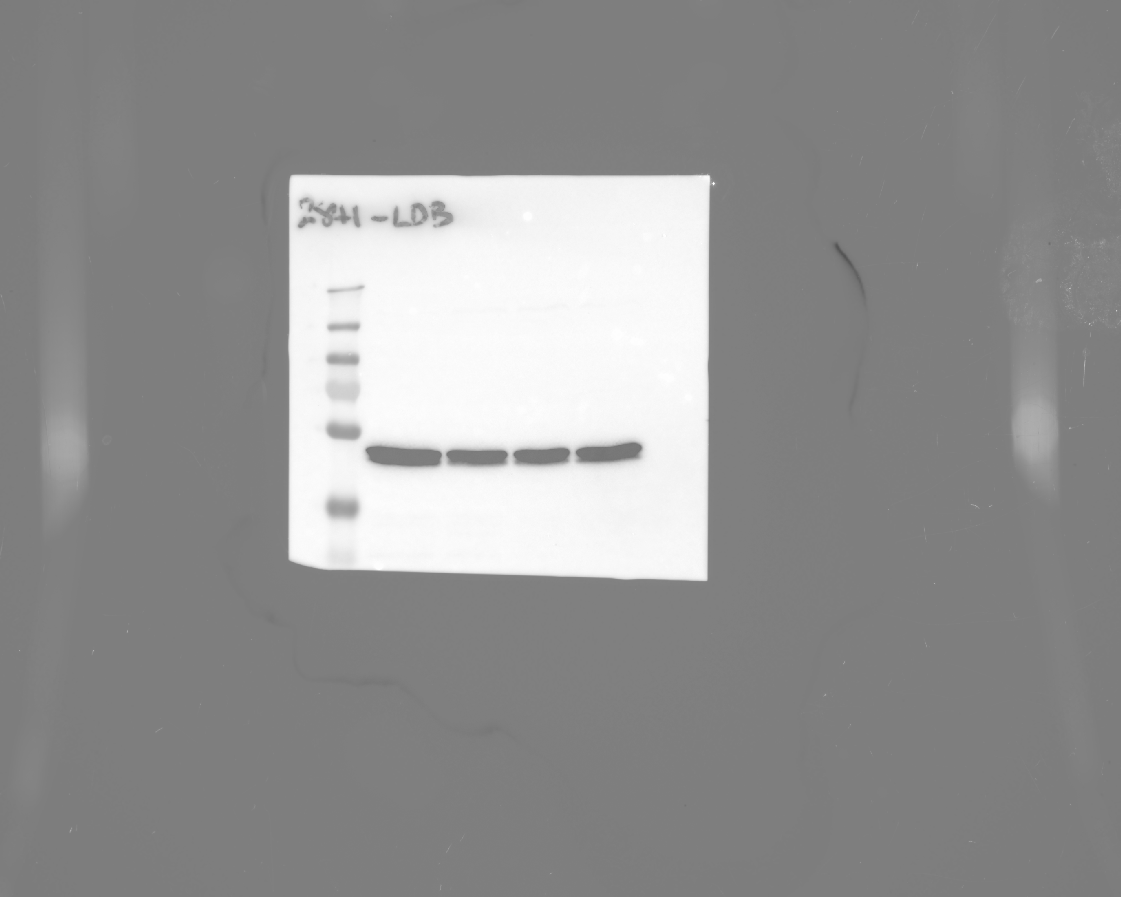

Supplement: Figure 3—source data 8. [file elife-106699-fig3-data8.zip › Figure 3ΓÇösource data 8 Original files for Western blot analysis displayed in Figure 3D./LDB1 CCRF-CEM Abd-CRBN.tif]

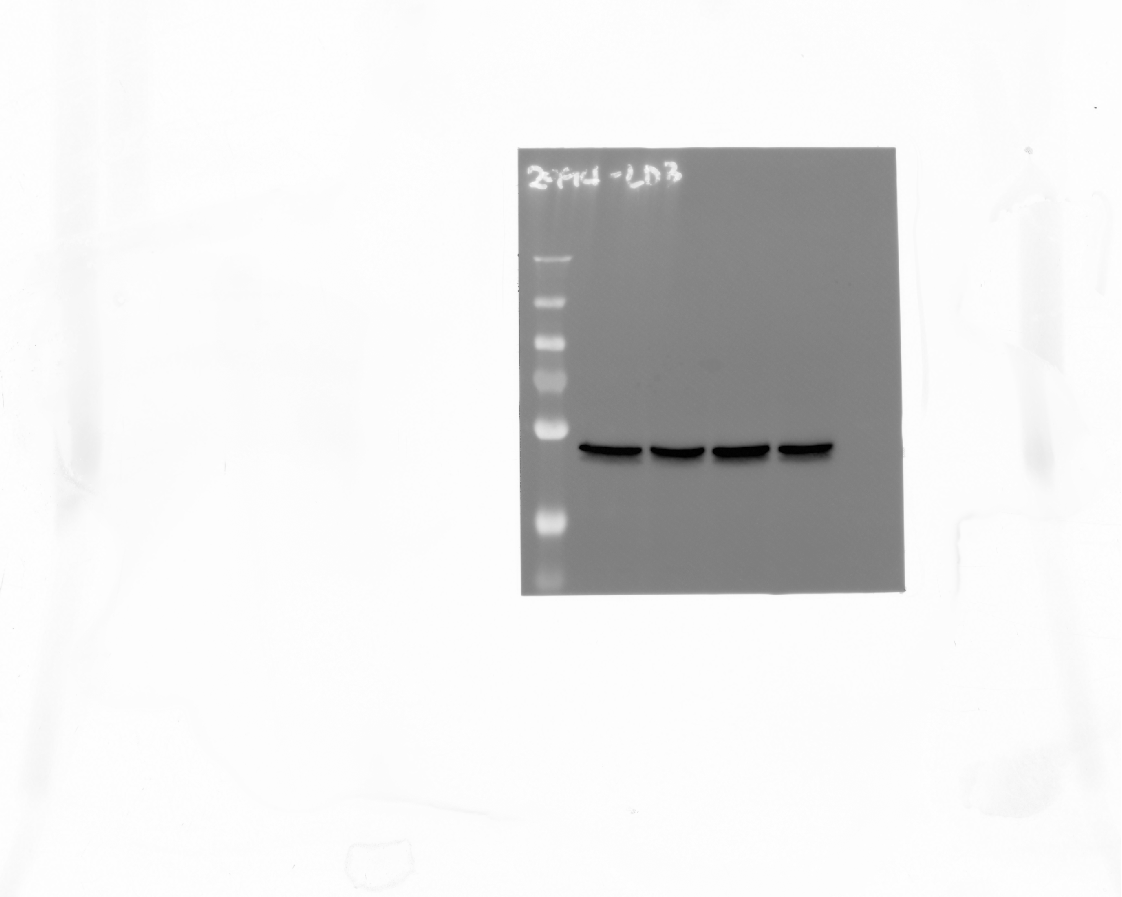

Supplement: Figure 3—source data 8. [file elife-106699-fig3-data8.zip › Figure 3ΓÇösource data 8 Original files for Western blot analysis displayed in Figure 3D./LDB1 CCRF-CEM Abd-VHL.tif]

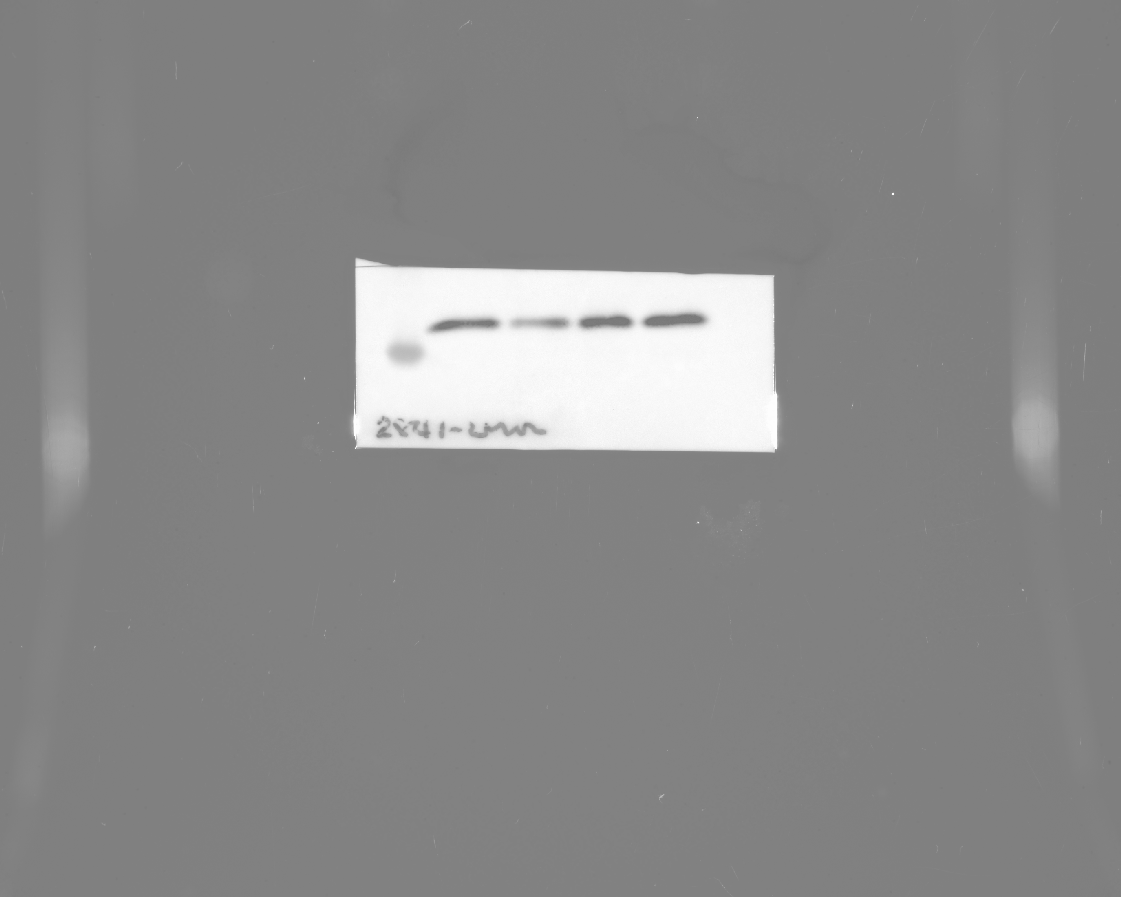

Supplement: Figure 3—source data 8. [file elife-106699-fig3-data8.zip › Figure 3ΓÇösource data 8 Original files for Western blot analysis displayed in Figure 3D./LMO2 CCRF-CEM Abd-CRBN.tif]

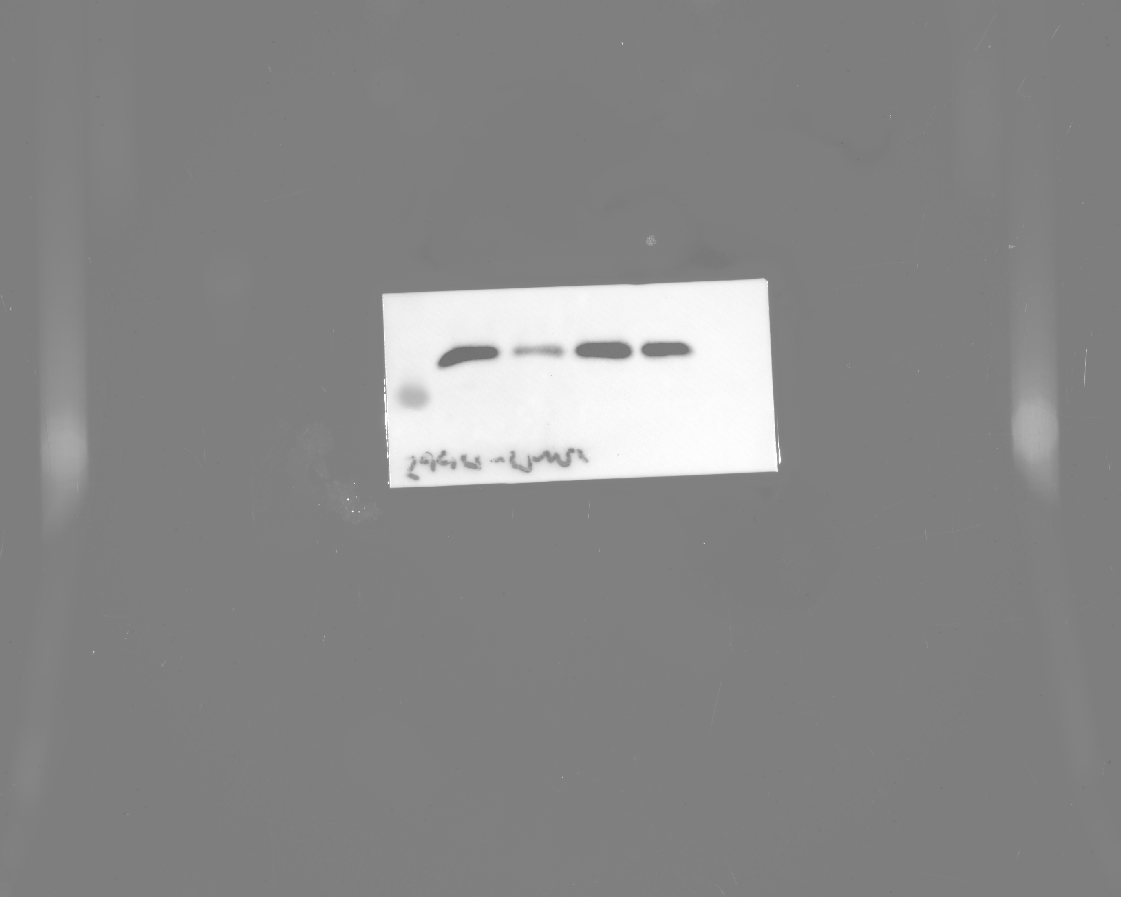

Supplement: Figure 3—source data 8. [file elife-106699-fig3-data8.zip › Figure 3ΓÇösource data 8 Original files for Western blot analysis displayed in Figure 3D./LMO2 CCRF-CEM Abd-VHL.tif]

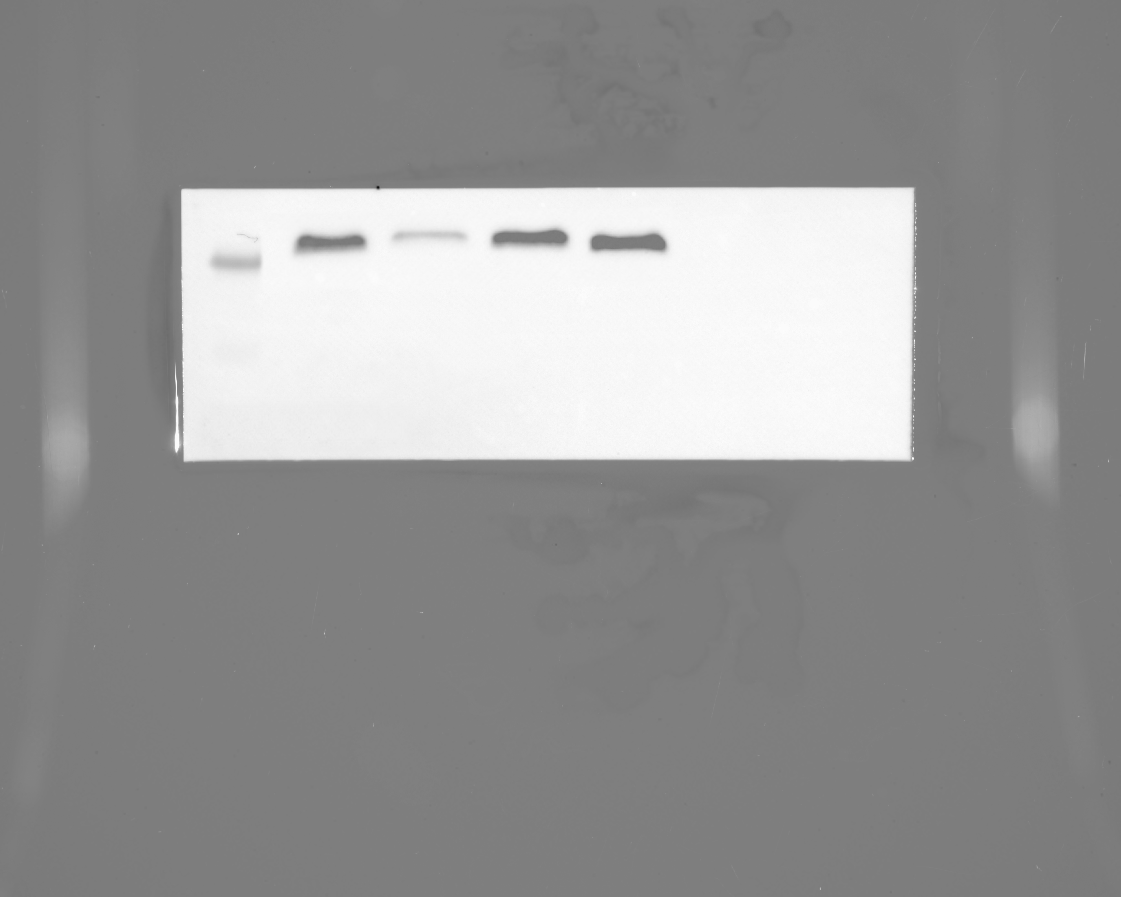

Supplement: Figure 3—source data 8. [file elife-106699-fig3-data8.zip › Figure 3ΓÇösource data 8 Original files for Western blot analysis displayed in Figure 3D./LMO2 KOPT-K1 Abd-CRBN.tif]

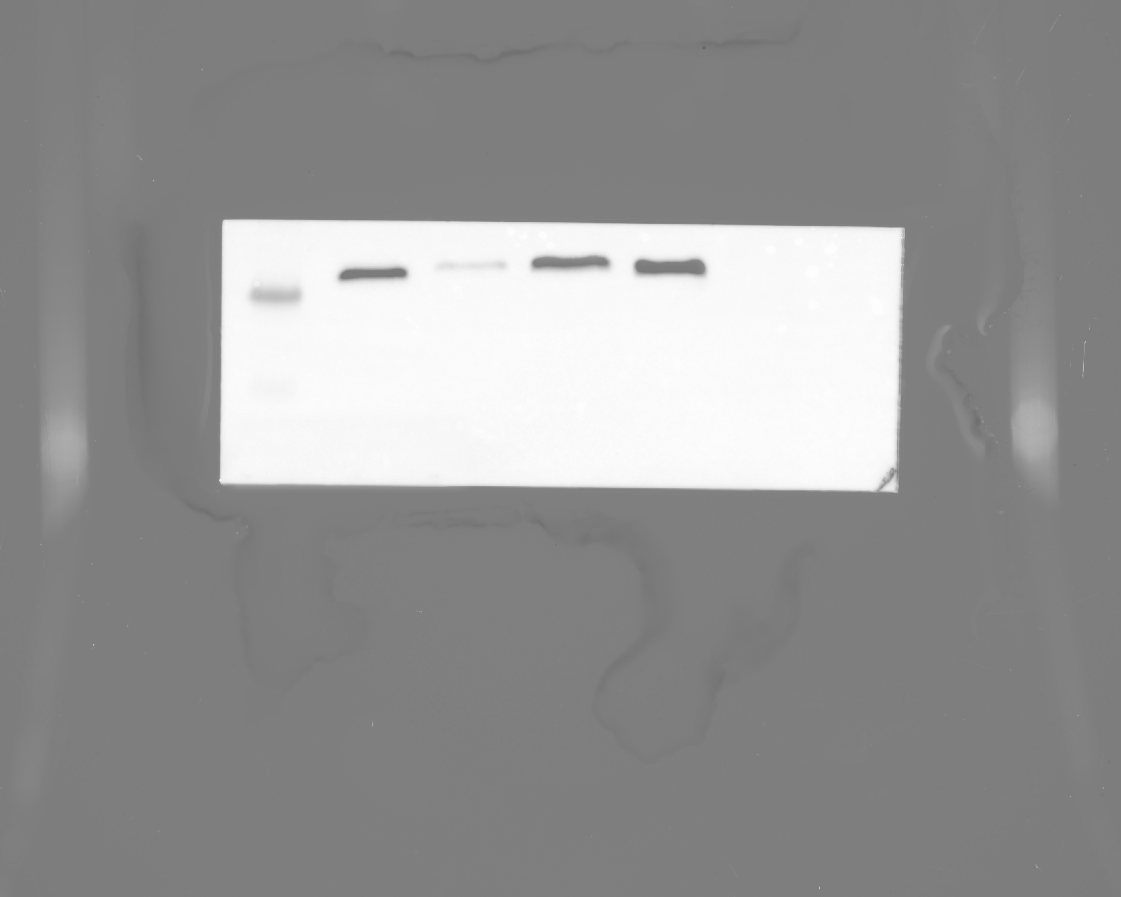

Supplement: Figure 3—source data 8. [file elife-106699-fig3-data8.zip › Figure 3ΓÇösource data 8 Original files for Western blot analysis displayed in Figure 3D./LMO2 KOPT-K1 Abd-VHL.tif]

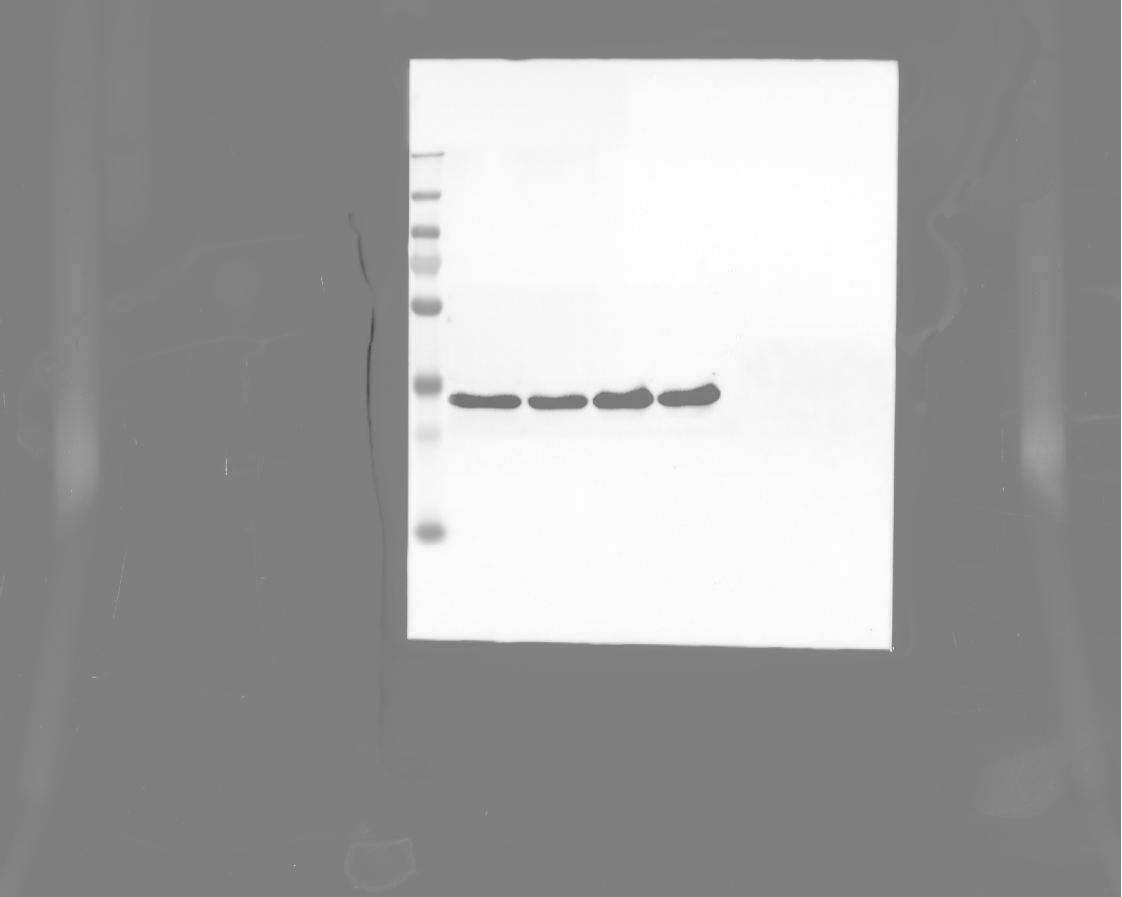

Supplement: Figure 3—source data 8. [file elife-106699-fig3-data8.zip › Figure 3ΓÇösource data 8 Original files for Western blot analysis displayed in Figure 3D./Lyl1 CCRF-CEM Abd-CRBN.tif]

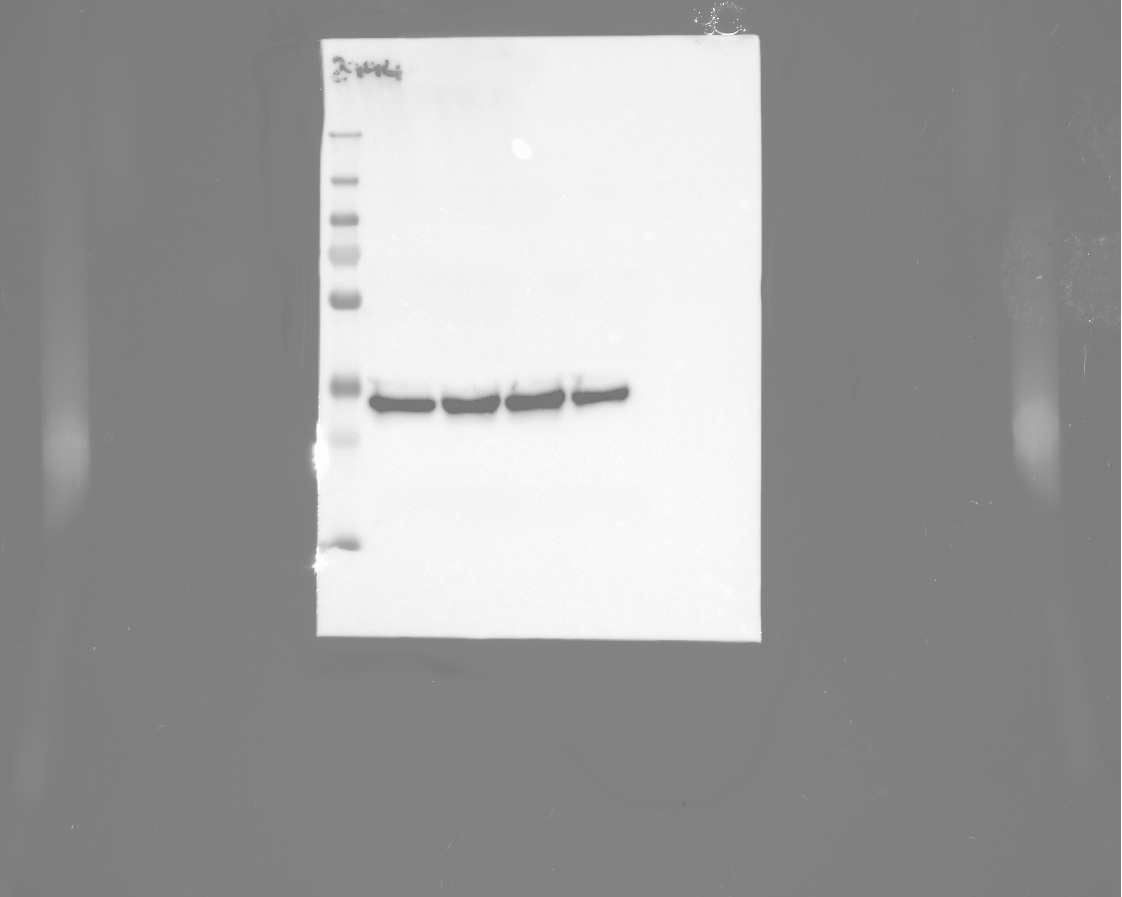

Supplement: Figure 3—source data 8. [file elife-106699-fig3-data8.zip › Figure 3ΓÇösource data 8 Original files for Western blot analysis displayed in Figure 3D./Lyl1 CCRF-CEM Abd-VHL.tif]

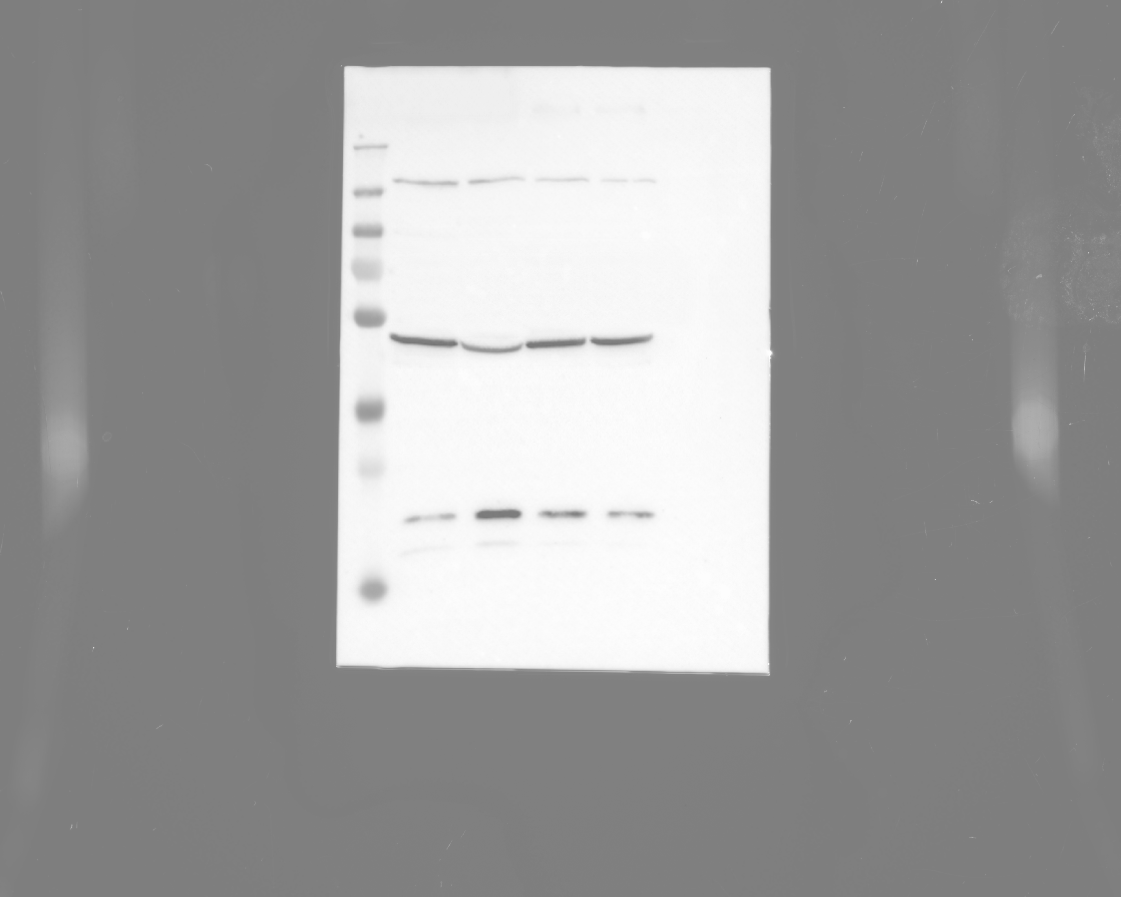

Supplement: Figure 3—source data 8. [file elife-106699-fig3-data8.zip › Figure 3ΓÇösource data 8 Original files for Western blot analysis displayed in Figure 3D./Tal-1 CCRF-CEM Abd-CRBN.tif]

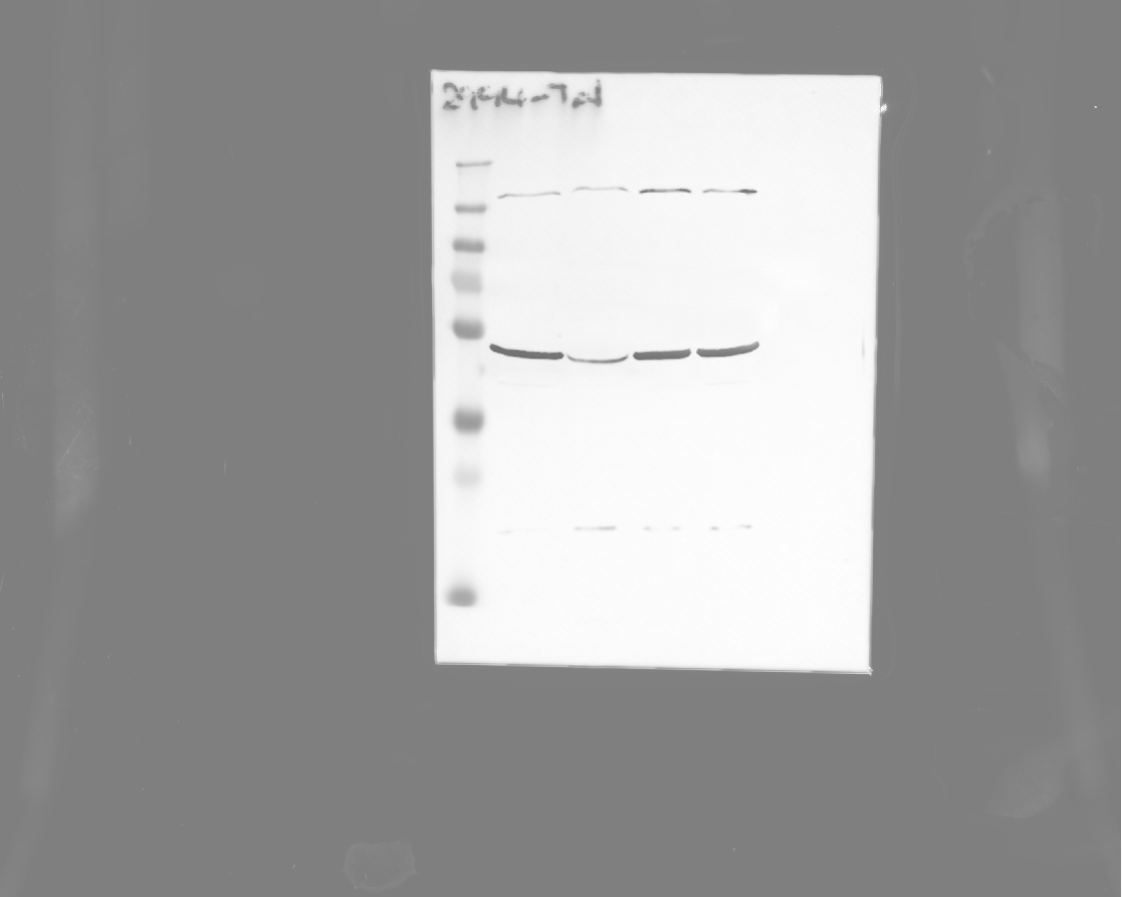

Supplement: Figure 3—source data 8. [file elife-106699-fig3-data8.zip › Figure 3ΓÇösource data 8 Original files for Western blot analysis displayed in Figure 3D./Tal1 CCRF-CEM Abd-VHL.tif]

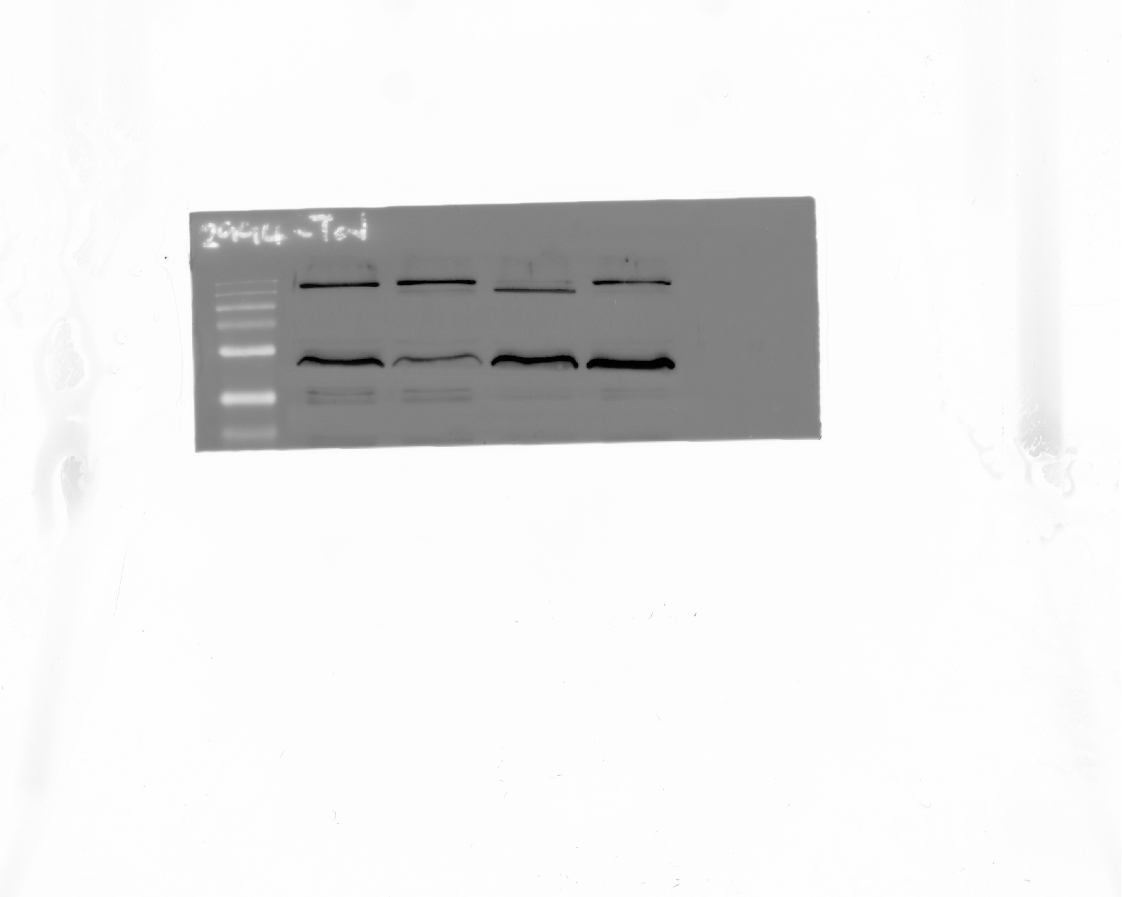

Supplement: Figure 3—source data 8. [file elife-106699-fig3-data8.zip › Figure 3ΓÇösource data 8 Original files for Western blot analysis displayed in Figure 3D./Tal1 KOPT-K1 Abd-VHL.tif]

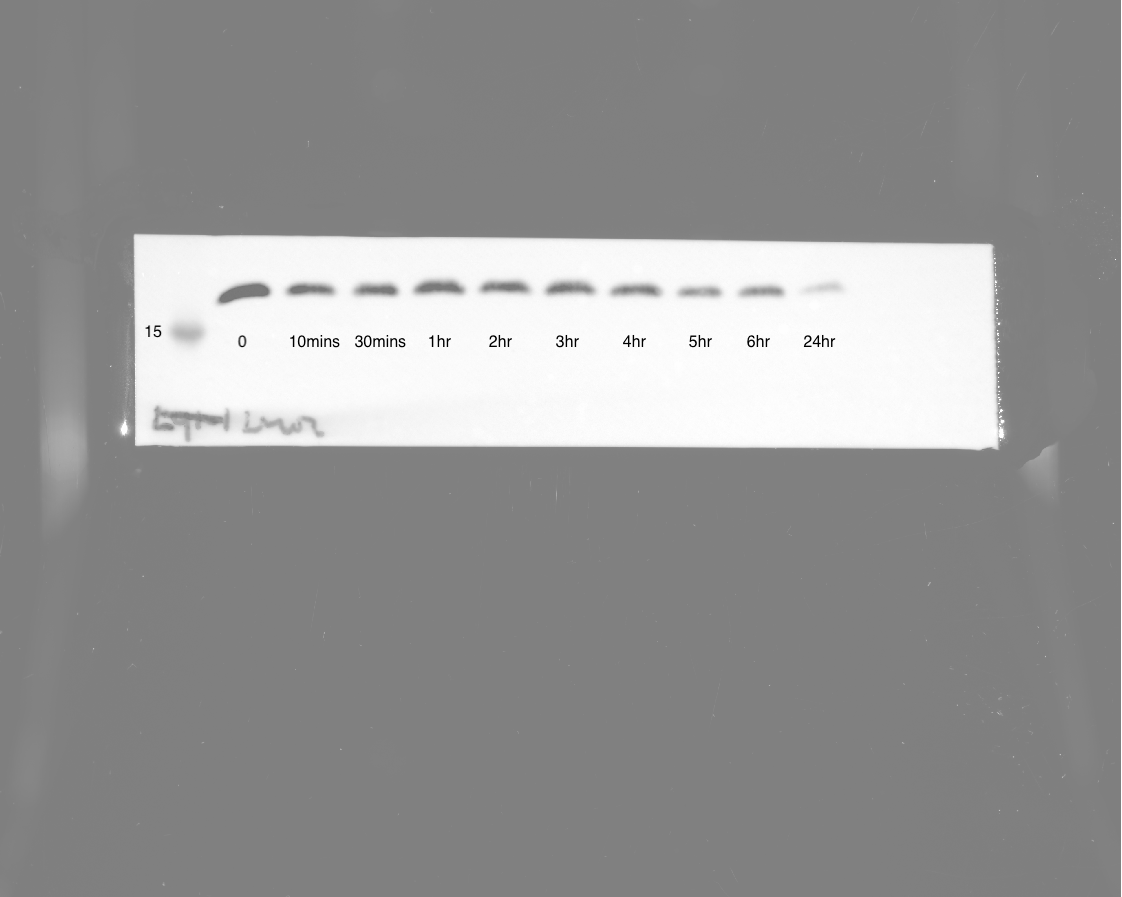

Supplement: Figure 3—figure supplement 1—source data 1. [file elife-106699-fig3-figsupp1-data1.zip › Figure 3ΓÇöfigure supplement 1-source data 1 Western blot data with label shows half-lives of LMO2 and LMO2 protein complex in KOPT-K1./Raw data/LMO2(Composite).tif]

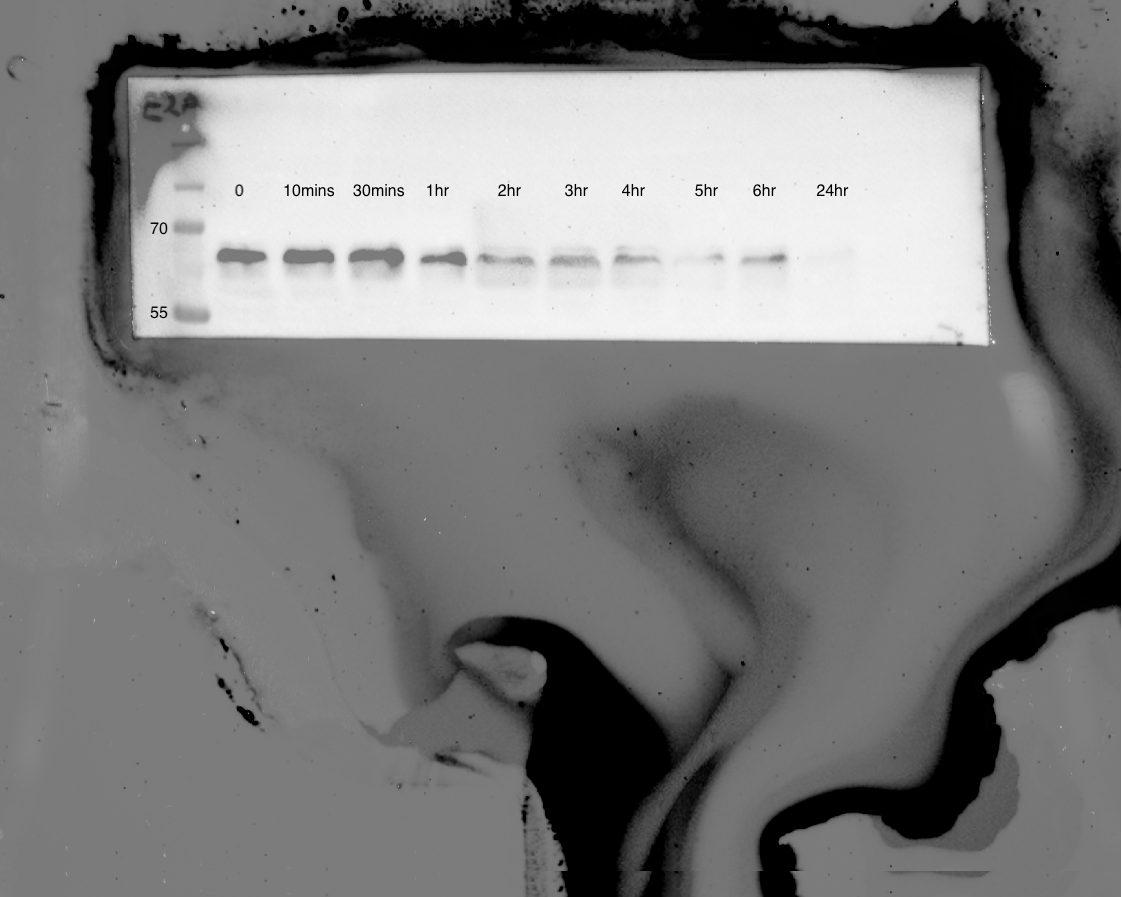

Supplement: Figure 3—figure supplement 1—source data 1. [file elife-106699-fig3-figsupp1-data1.zip › Figure 3ΓÇöfigure supplement 1-source data 1 Western blot data with label shows half-lives of LMO2 and LMO2 protein complex in KOPT-K1./Raw data/E2A(Composite).tif]

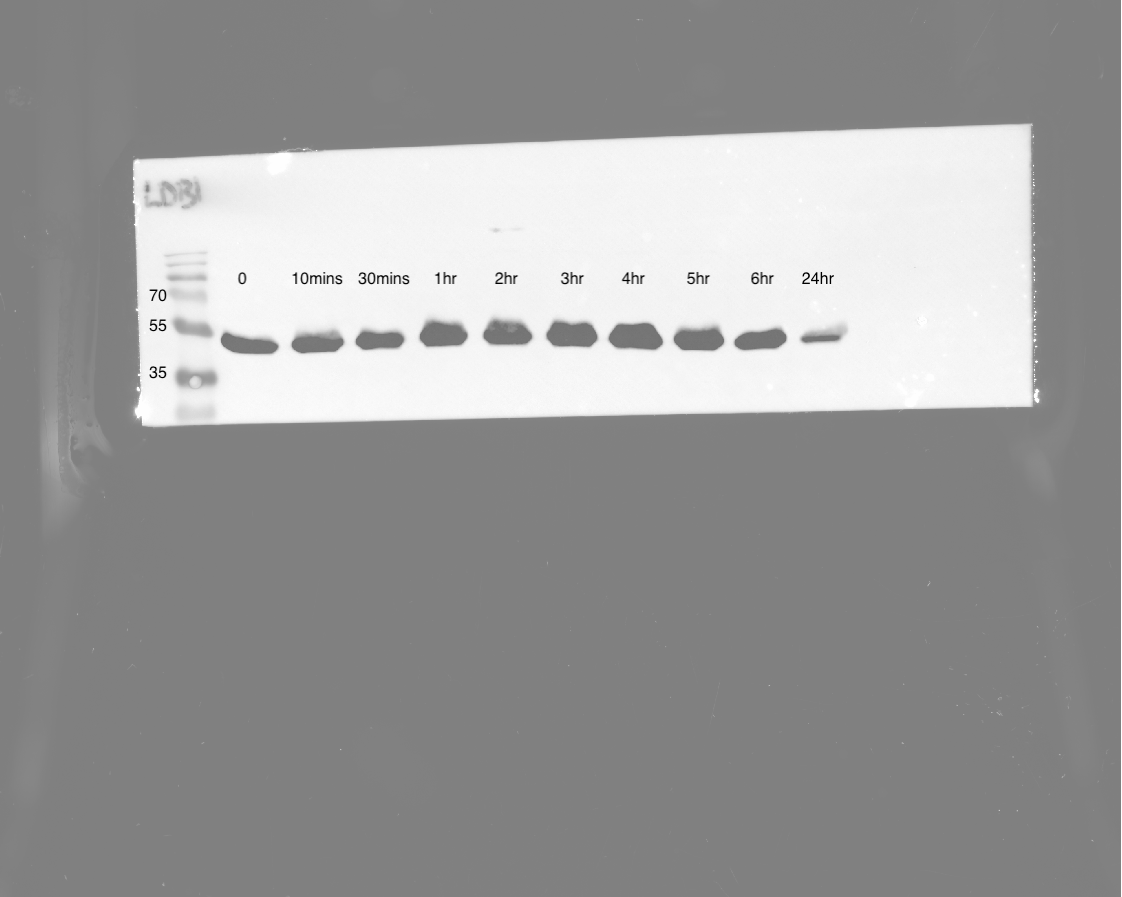

Supplement: Figure 3—figure supplement 1—source data 1. [file elife-106699-fig3-figsupp1-data1.zip › Figure 3ΓÇöfigure supplement 1-source data 1 Western blot data with label shows half-lives of LMO2 and LMO2 protein complex in KOPT-K1./Raw data/LDB1(Composite).tif]

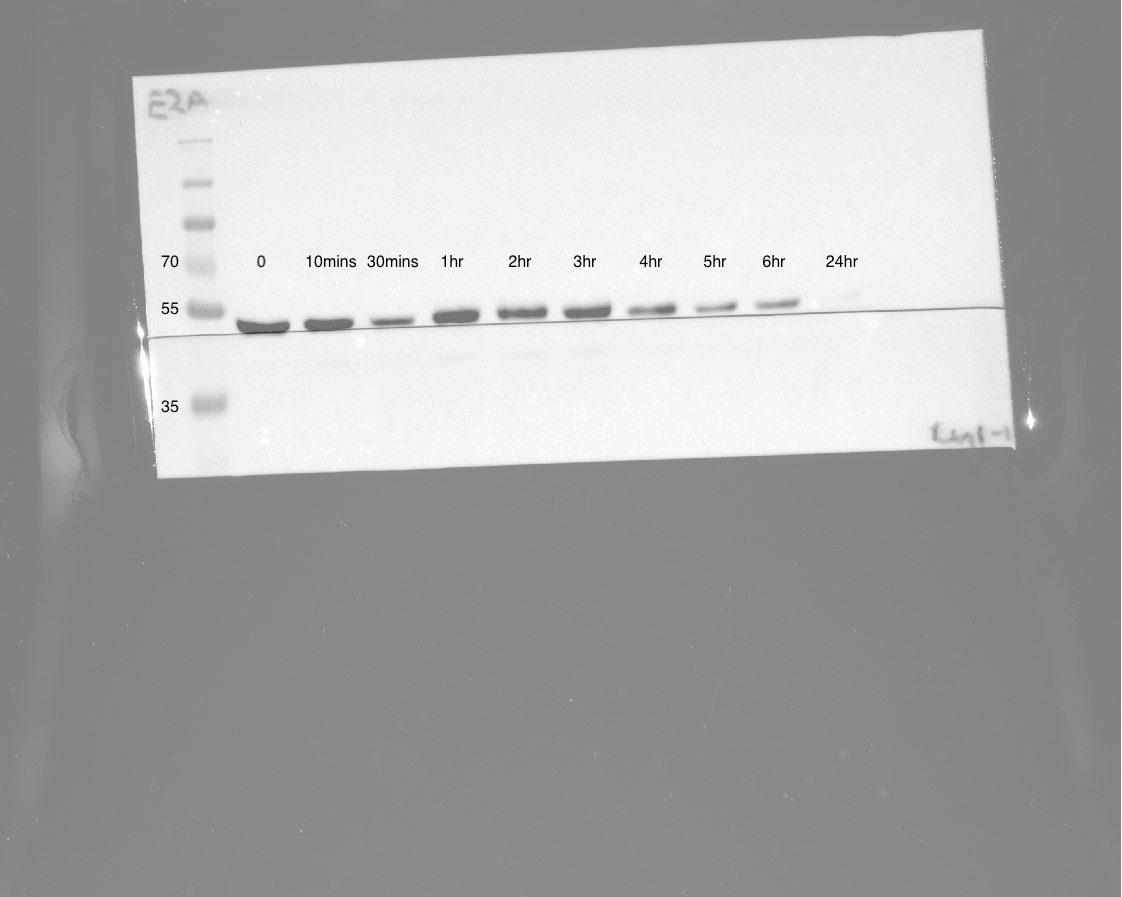

Supplement: Figure 3—figure supplement 1—source data 1. [file elife-106699-fig3-figsupp1-data1.zip › Figure 3ΓÇöfigure supplement 1-source data 1 Western blot data with label shows half-lives of LMO2 and LMO2 protein complex in KOPT-K1./Raw data/GATA3(Composite).tif]

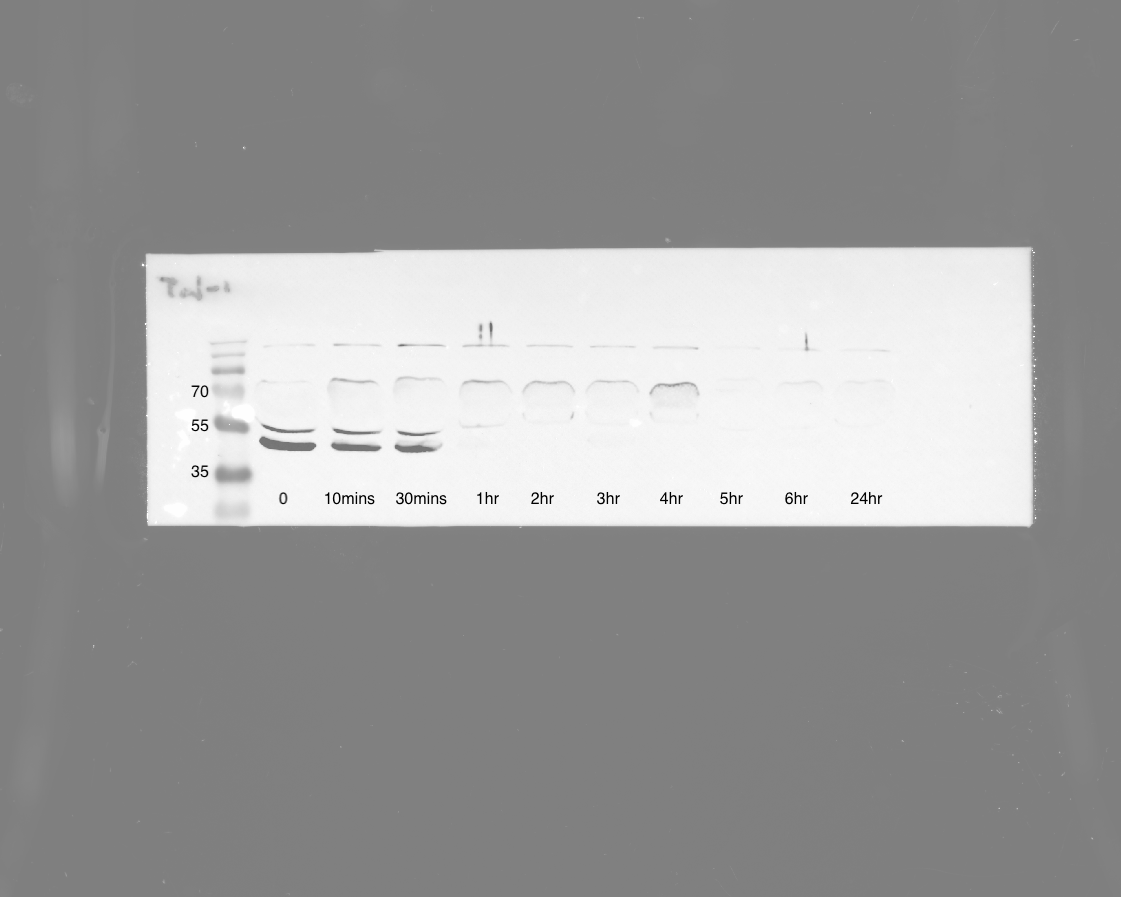

Supplement: Figure 3—figure supplement 1—source data 1. [file elife-106699-fig3-figsupp1-data1.zip › Figure 3ΓÇöfigure supplement 1-source data 1 Western blot data with label shows half-lives of LMO2 and LMO2 protein complex in KOPT-K1./Raw data/Tal1(Composite).tif]

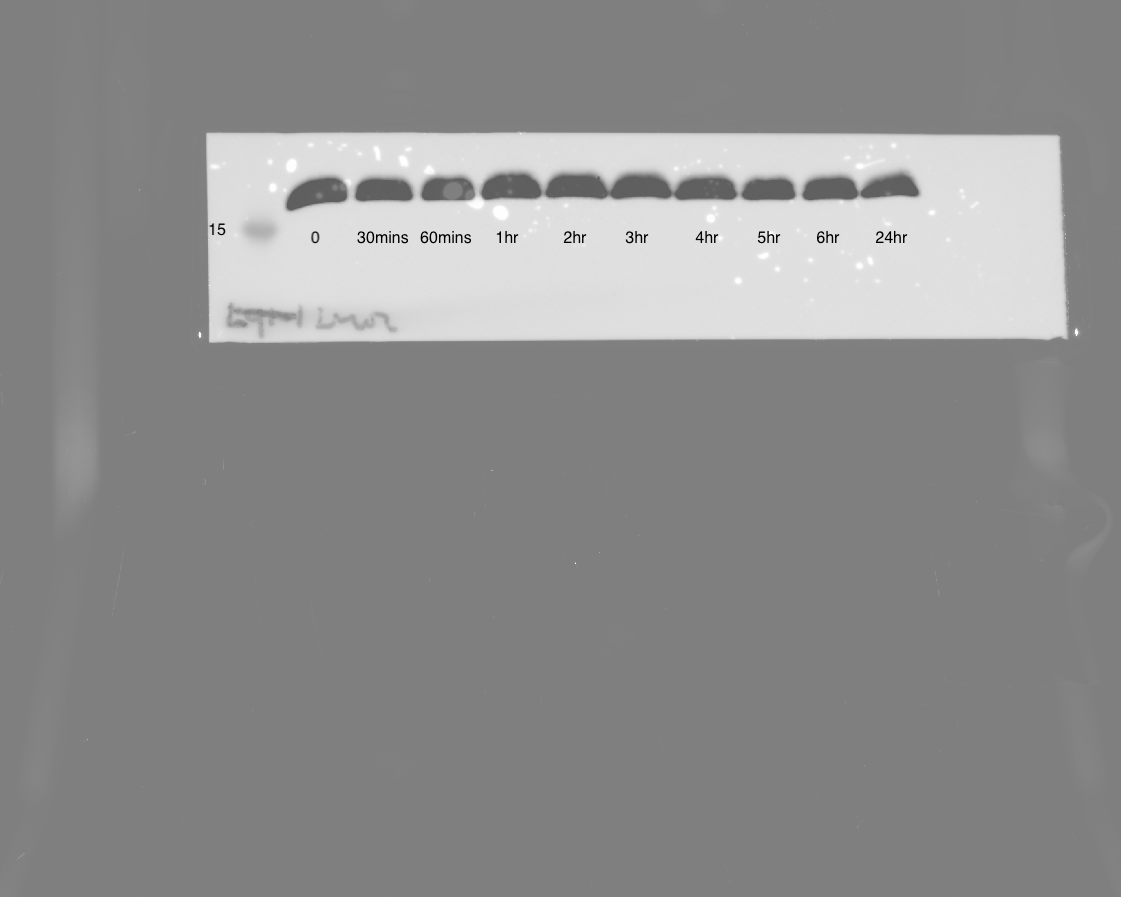

Supplement: Figure 3—figure supplement 1—source data 1. [file elife-106699-fig3-figsupp1-data1.zip › Figure 3ΓÇöfigure supplement 1-source data 1 Western blot data with label shows half-lives of LMO2 and LMO2 protein complex in KOPT-K1./Raw data/Cyclophilin(Composite).tif]

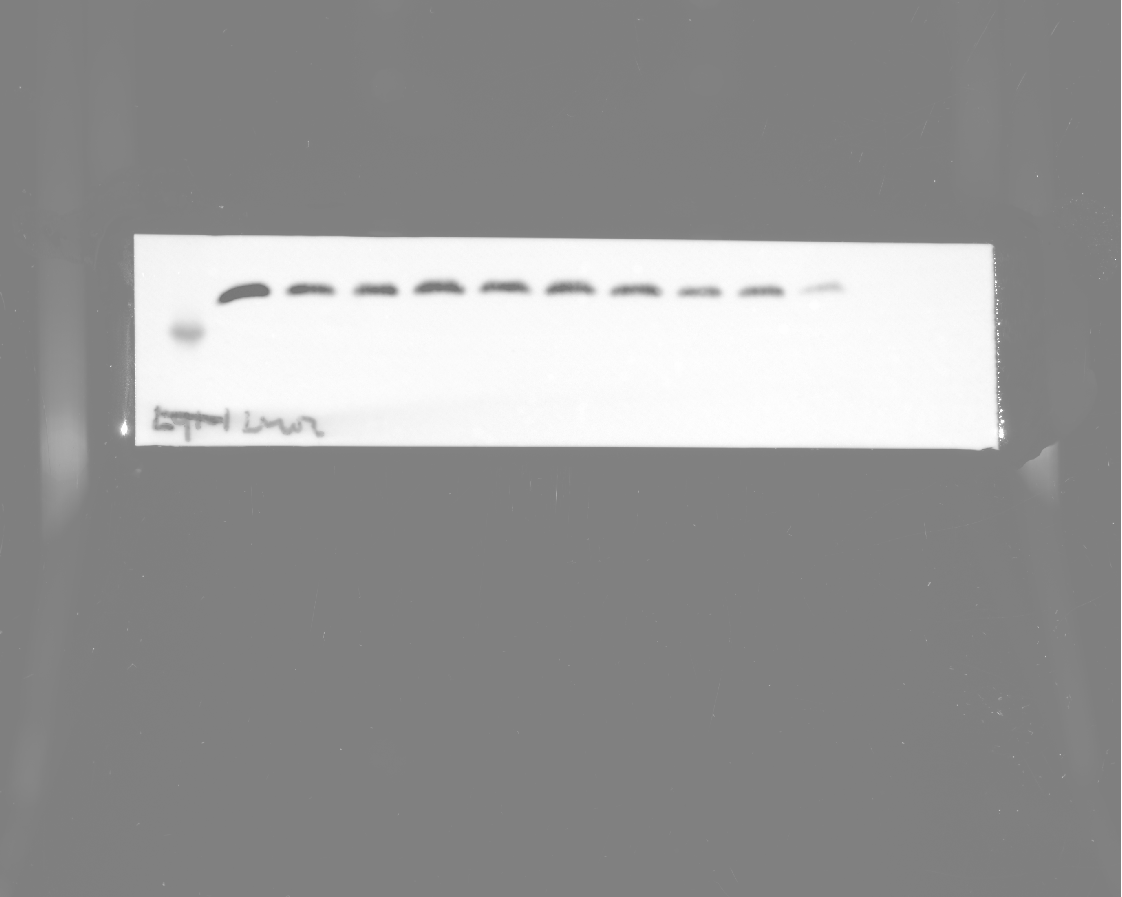

Supplement: Figure 3—figure supplement 1—source data 2. [file elife-106699-fig3-figsupp1-data2.zip › Figure 3ΓÇöfigure supplement 1-source data 2 Western blot raw data shows half-lives of LMO2 and LMO2 protein complex in KOPT-K1./LMO2(Composite).tif]

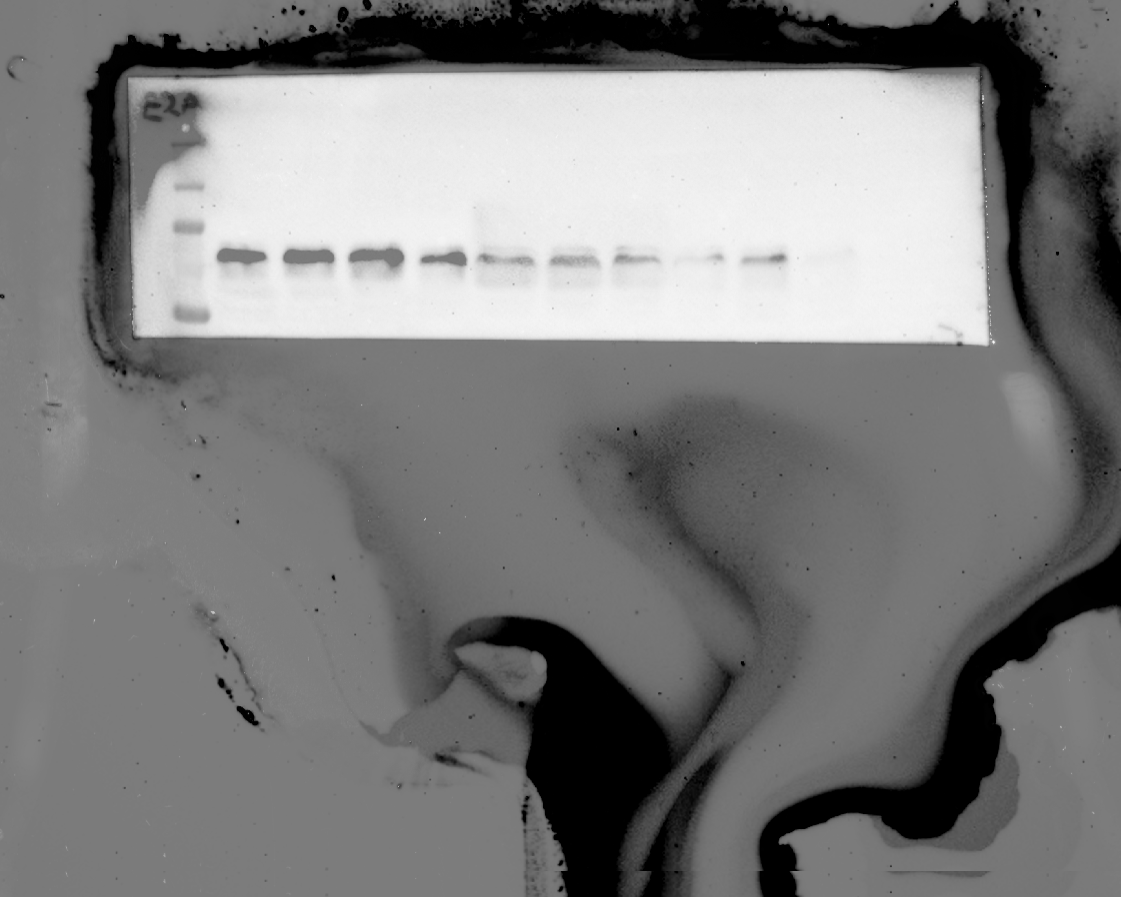

Supplement: Figure 3—figure supplement 1—source data 2. [file elife-106699-fig3-figsupp1-data2.zip › Figure 3ΓÇöfigure supplement 1-source data 2 Western blot raw data shows half-lives of LMO2 and LMO2 protein complex in KOPT-K1./E2A(Composite).tif]

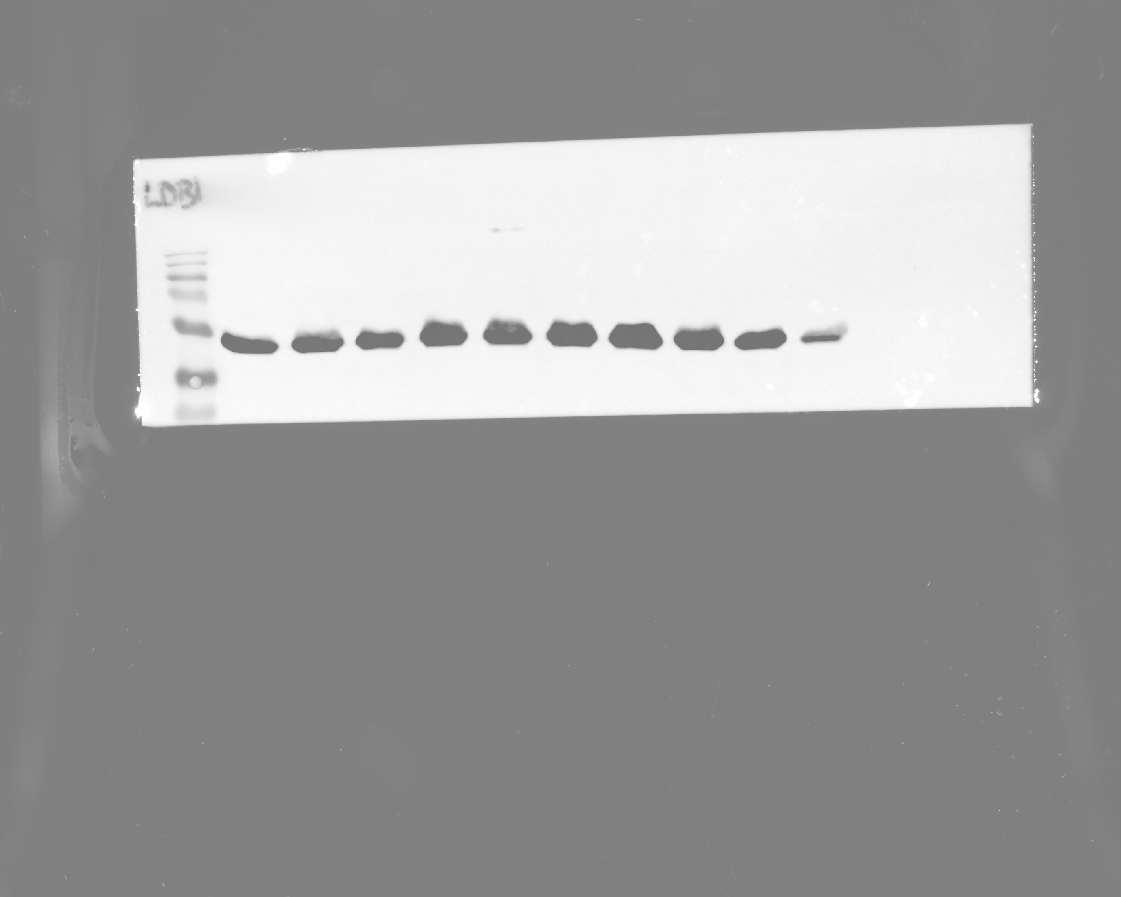

Supplement: Figure 3—figure supplement 1—source data 2. [file elife-106699-fig3-figsupp1-data2.zip › Figure 3ΓÇöfigure supplement 1-source data 2 Western blot raw data shows half-lives of LMO2 and LMO2 protein complex in KOPT-K1./LDB1(Composite).tif]

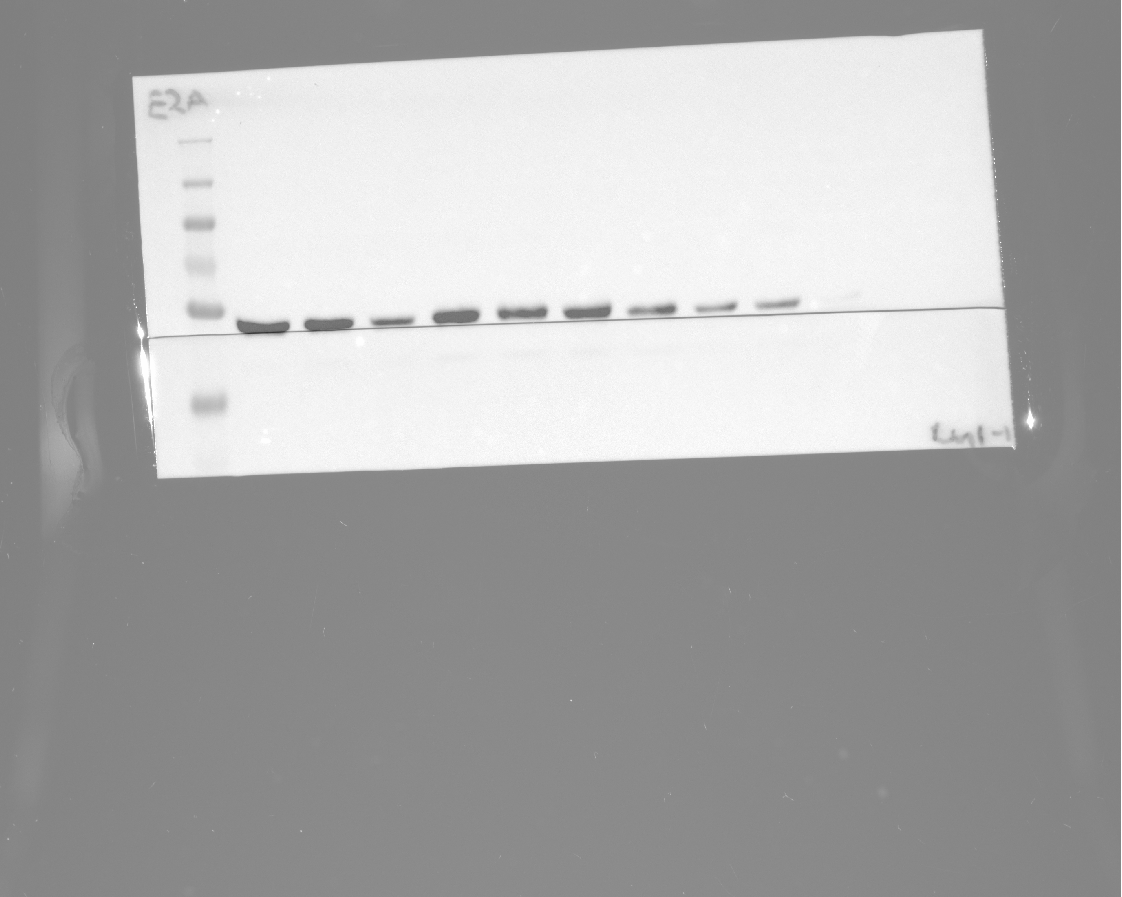

Supplement: Figure 3—figure supplement 1—source data 2. [file elife-106699-fig3-figsupp1-data2.zip › Figure 3ΓÇöfigure supplement 1-source data 2 Western blot raw data shows half-lives of LMO2 and LMO2 protein complex in KOPT-K1./GATA3(Composite).tif]

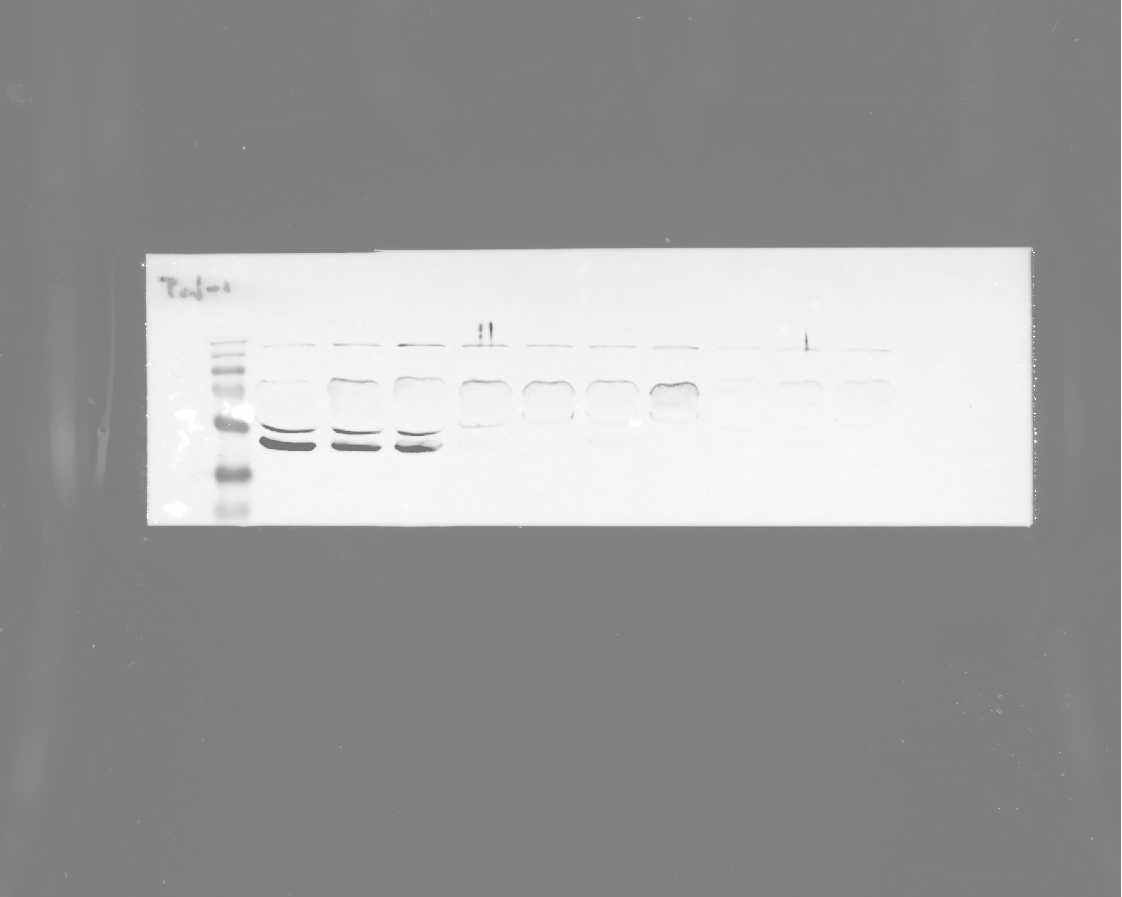

Supplement: Figure 3—figure supplement 1—source data 2. [file elife-106699-fig3-figsupp1-data2.zip › Figure 3ΓÇöfigure supplement 1-source data 2 Western blot raw data shows half-lives of LMO2 and LMO2 protein complex in KOPT-K1./Tal1(Composite).tif]

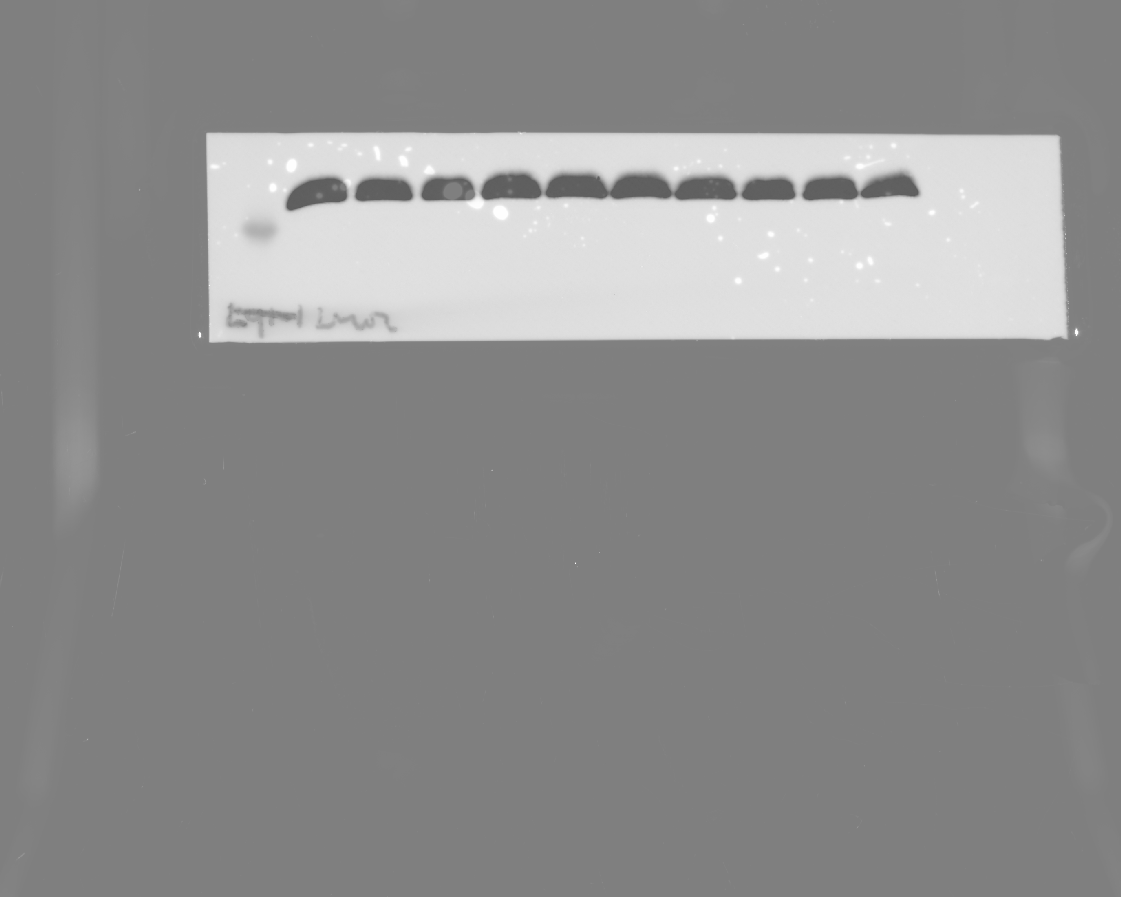

Supplement: Figure 3—figure supplement 1—source data 2. [file elife-106699-fig3-figsupp1-data2.zip › Figure 3ΓÇöfigure supplement 1-source data 2 Western blot raw data shows half-lives of LMO2 and LMO2 protein complex in KOPT-K1./Cyclophilin(Composite).tif]

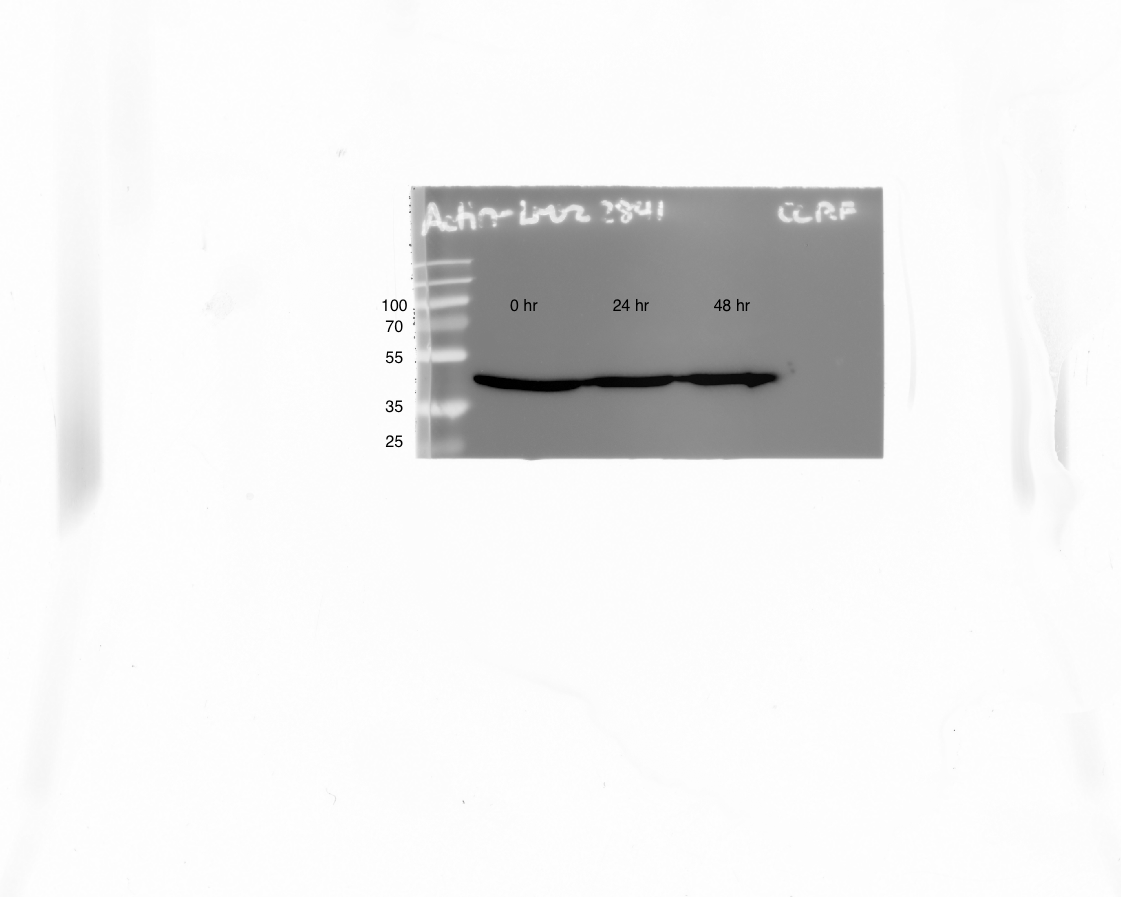

Supplement: Figure 4—source data 1. [file elife-106699-fig4-data1.zip › Figure 4ΓÇösource data 1 PDF files containing original western blots for Figure 4A, indicating the relevant bands and treatments./Raw data/Actin CCRF-CEM Abd-CRBN.tif]

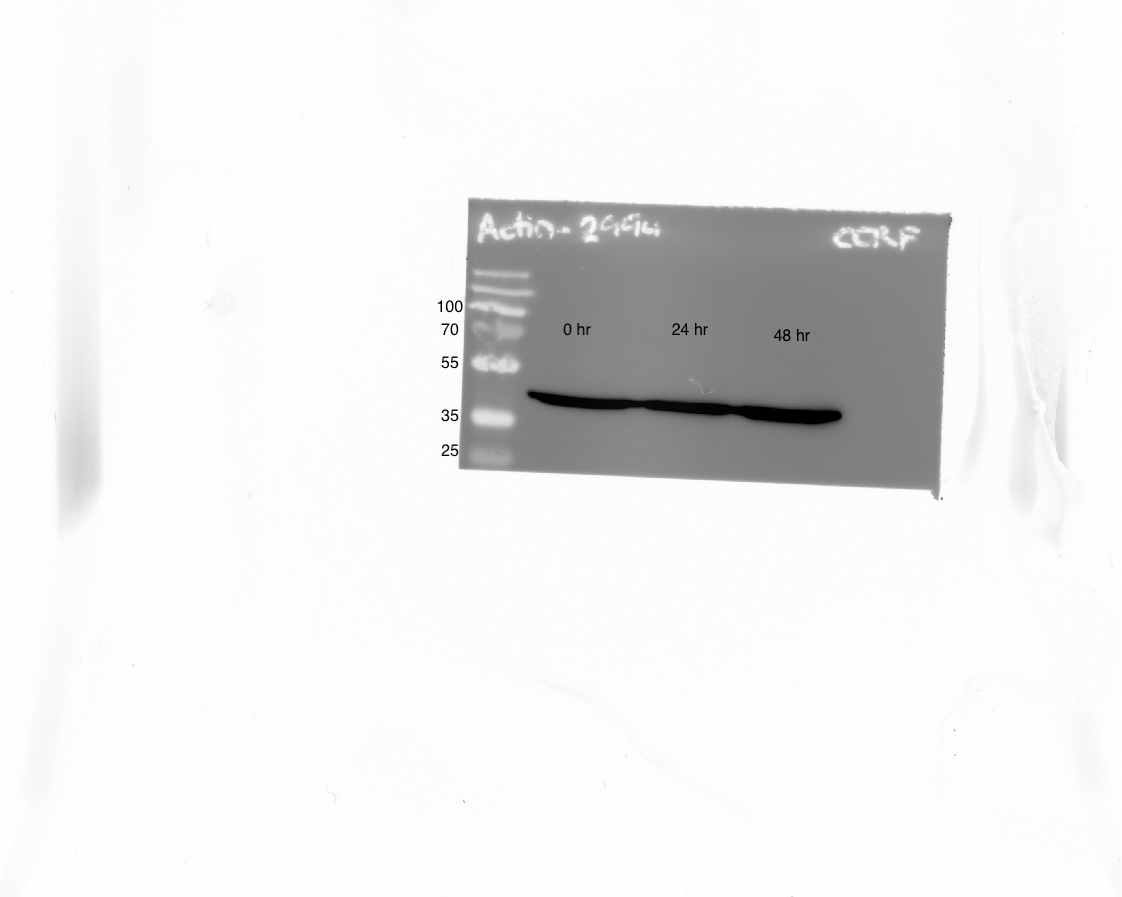

Supplement: Figure 4—source data 1. [file elife-106699-fig4-data1.zip › Figure 4ΓÇösource data 1 PDF files containing original western blots for Figure 4A, indicating the relevant bands and treatments./Raw data/Actin CCRF-CEM Abd-VHL.tif]

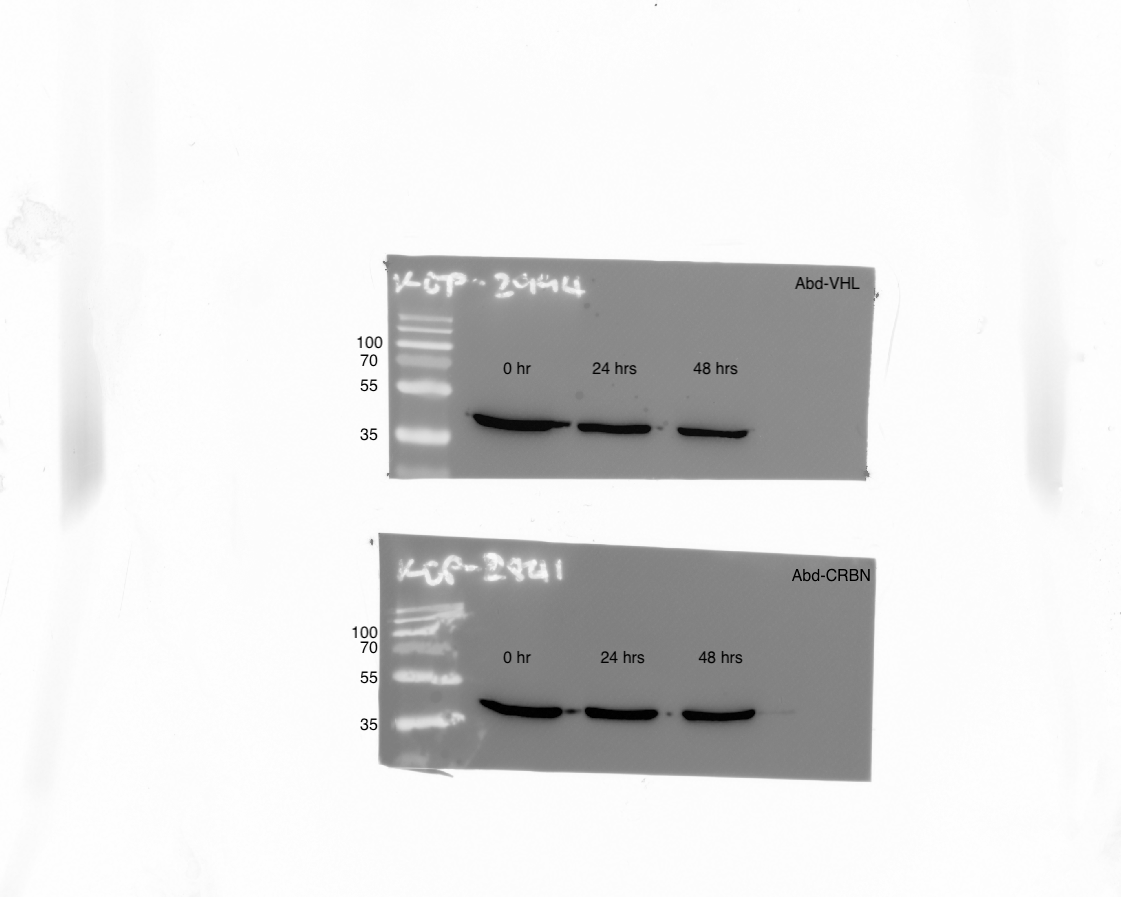

Supplement: Figure 4—source data 1. [file elife-106699-fig4-data1.zip › Figure 4ΓÇösource data 1 PDF files containing original western blots for Figure 4A, indicating the relevant bands and treatments./Raw data/Actin KOPT-K1 Abd-CRBN abd Avd-VHL.tif]

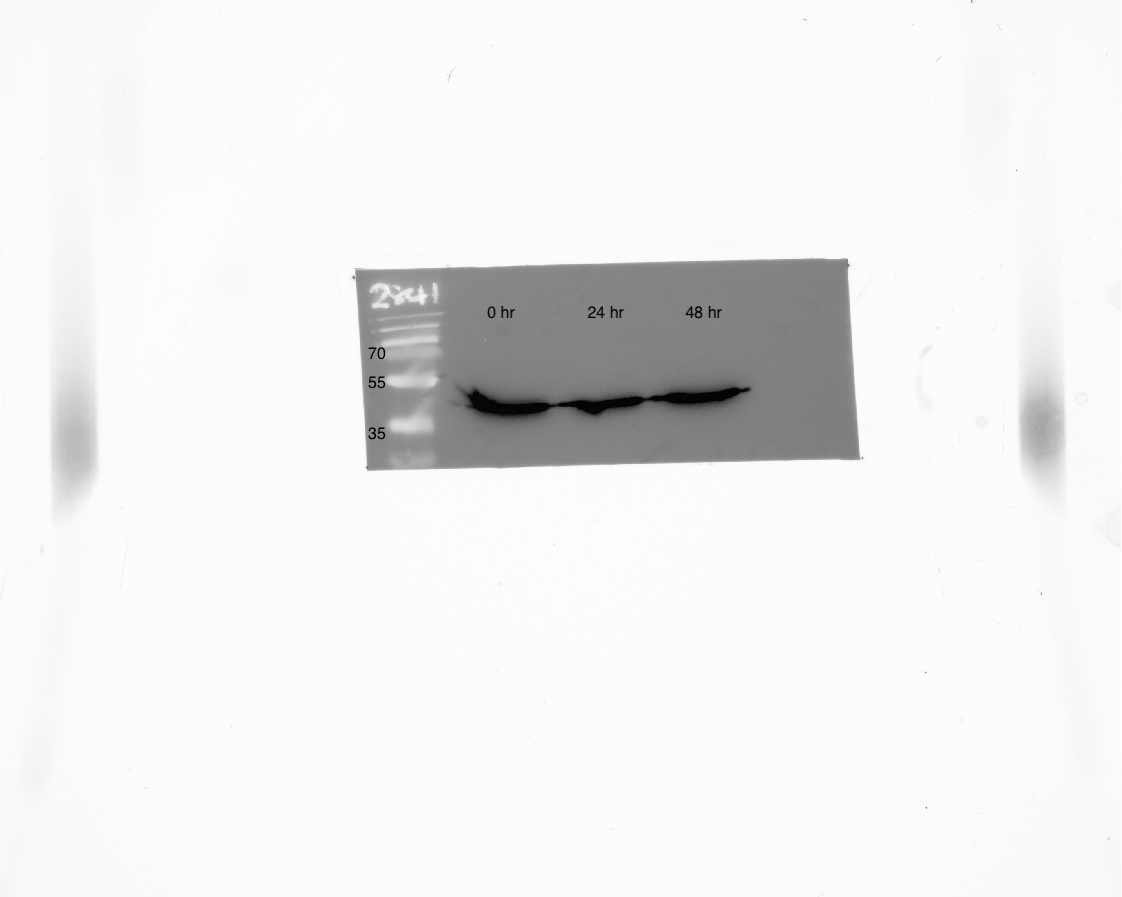

Supplement: Figure 4—source data 1. [file elife-106699-fig4-data1.zip › Figure 4ΓÇösource data 1 PDF files containing original western blots for Figure 4A, indicating the relevant bands and treatments./Raw data/Actin LOUCY Abd-CRBN.tif]

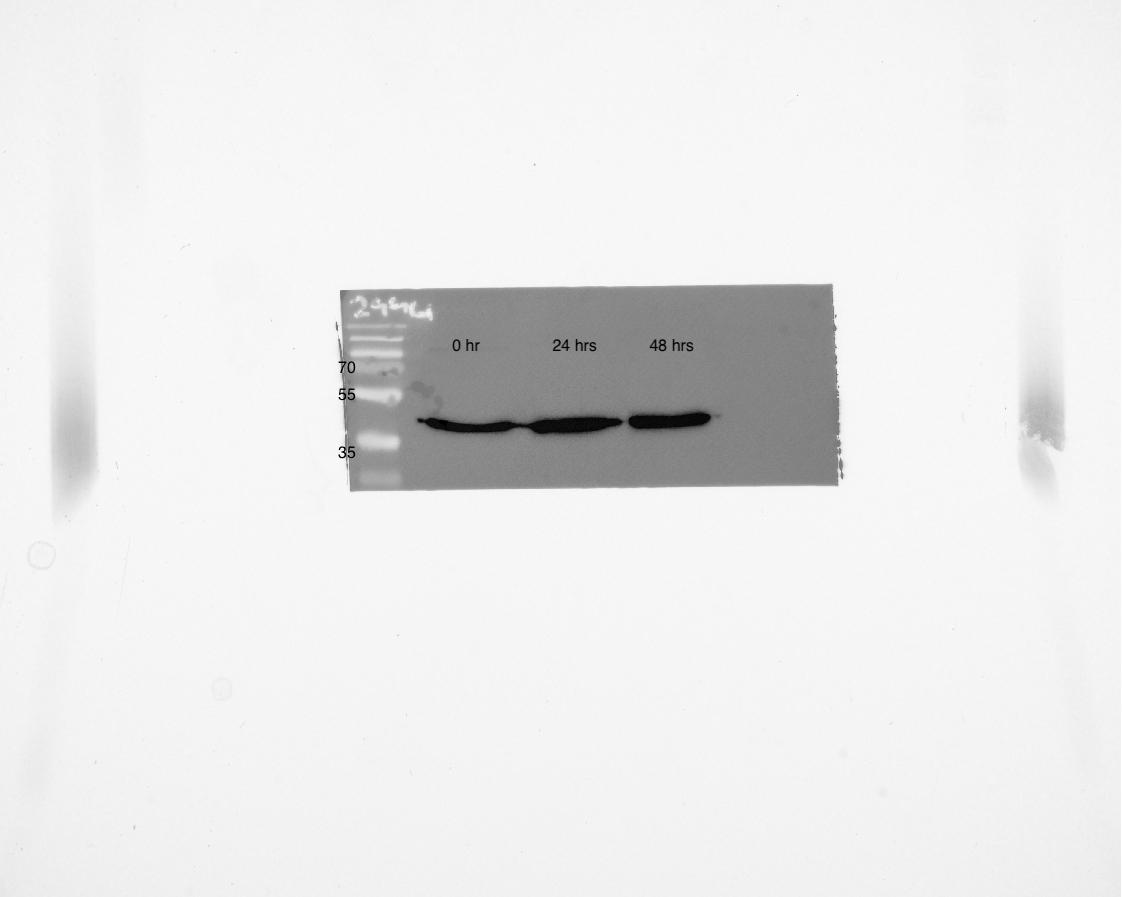

Supplement: Figure 4—source data 1. [file elife-106699-fig4-data1.zip › Figure 4ΓÇösource data 1 PDF files containing original western blots for Figure 4A, indicating the relevant bands and treatments./Raw data/Actin LOUCY Abd-VHL.tif]

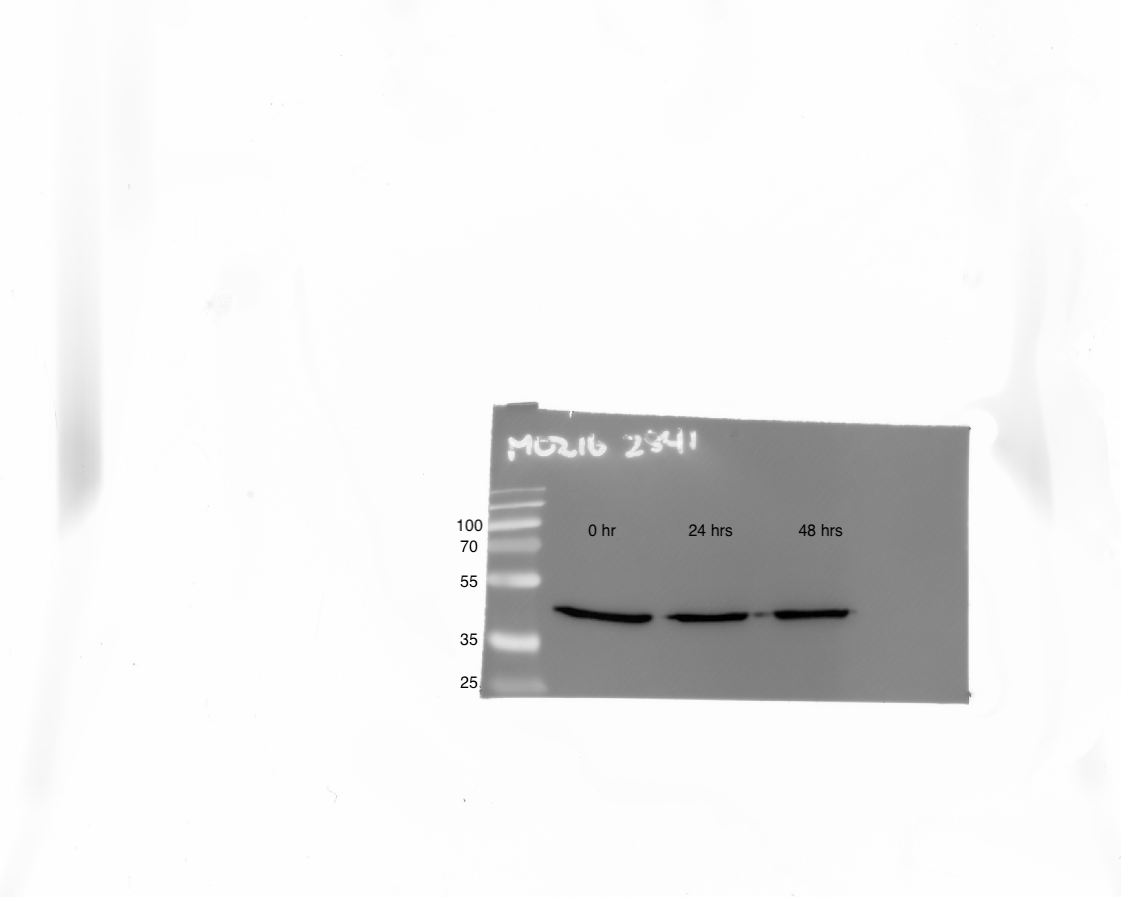

Supplement: Figure 4—source data 1. [file elife-106699-fig4-data1.zip › Figure 4ΓÇösource data 1 PDF files containing original western blots for Figure 4A, indicating the relevant bands and treatments./Raw data/Actin MOLT-16 Abd-CRBN.tif]

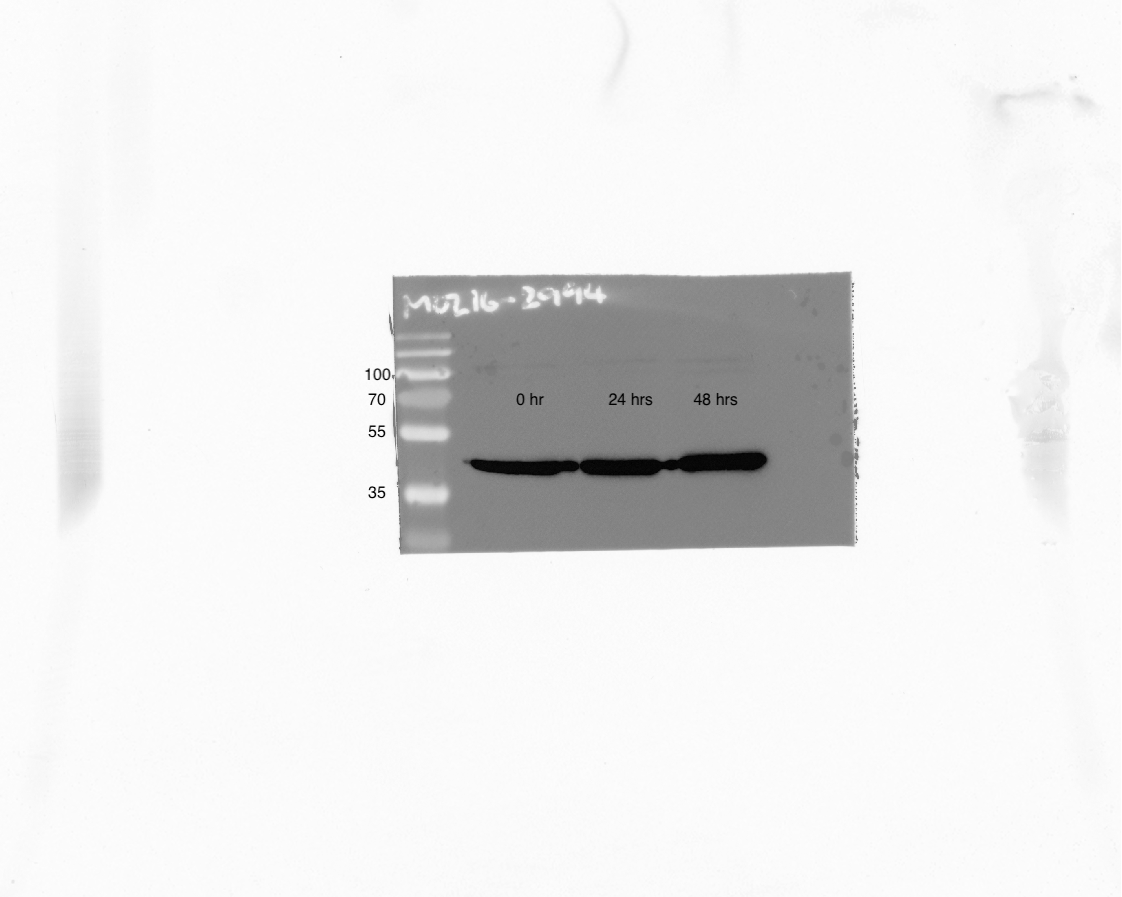

Supplement: Figure 4—source data 1. [file elife-106699-fig4-data1.zip › Figure 4ΓÇösource data 1 PDF files containing original western blots for Figure 4A, indicating the relevant bands and treatments./Raw data/Actin MOLT-16 Abd-VHL.tif]

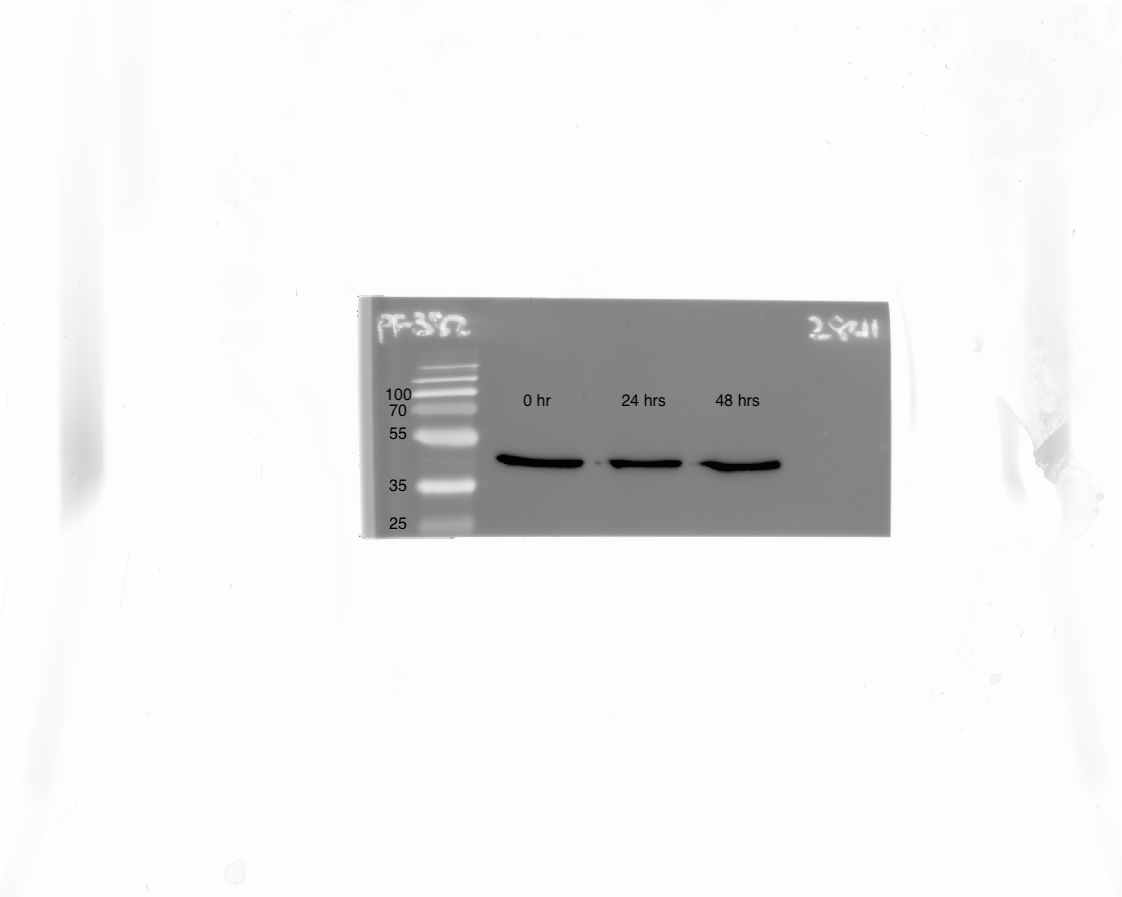

Supplement: Figure 4—source data 1. [file elife-106699-fig4-data1.zip › Figure 4ΓÇösource data 1 PDF files containing original western blots for Figure 4A, indicating the relevant bands and treatments./Raw data/Actin PF-382 Abd-CRBN.tif]

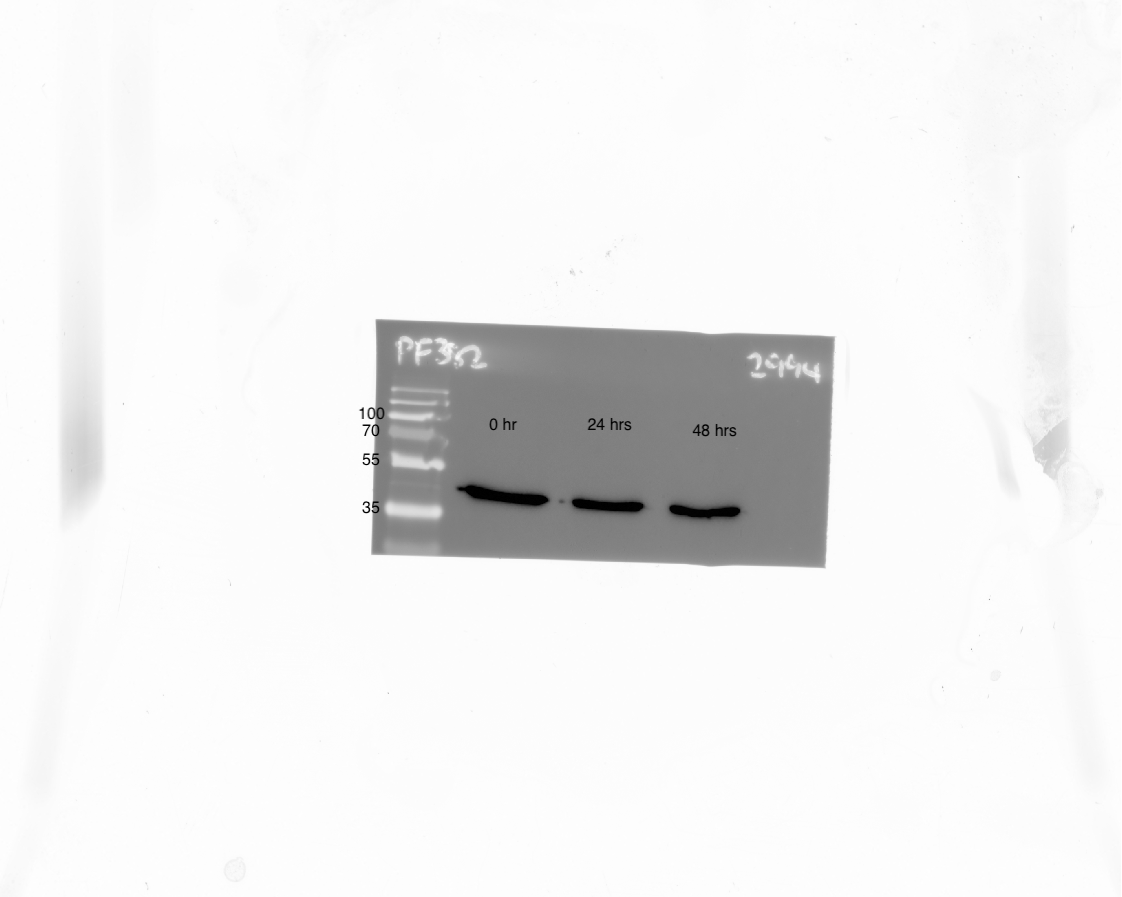

Supplement: Figure 4—source data 1. [file elife-106699-fig4-data1.zip › Figure 4ΓÇösource data 1 PDF files containing original western blots for Figure 4A, indicating the relevant bands and treatments./Raw data/Actin PF-382 Abd-VHL.tif]

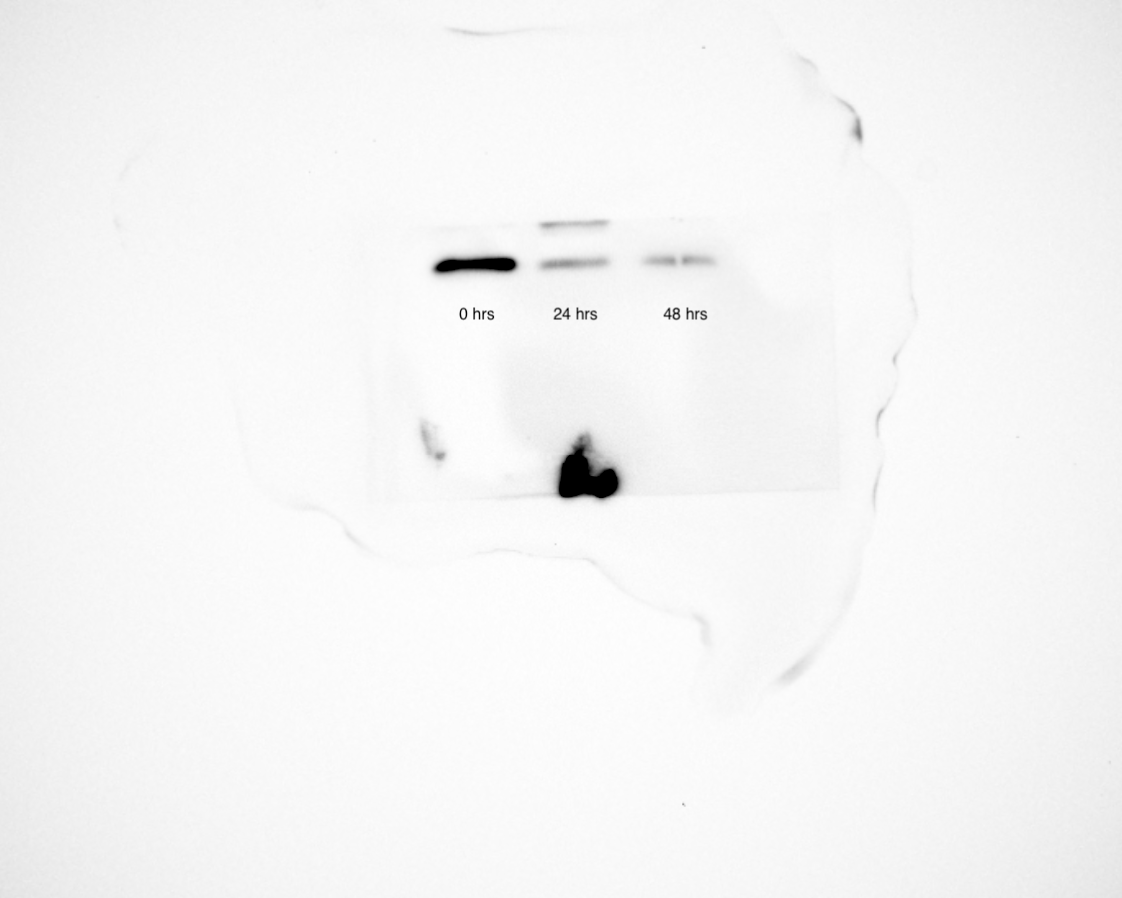

Supplement: Figure 4—source data 1. [file elife-106699-fig4-data1.zip › Figure 4ΓÇösource data 1 PDF files containing original western blots for Figure 4A, indicating the relevant bands and treatments./Raw data/LMO2 CCRF-CEM Abd-CRBN.tif]

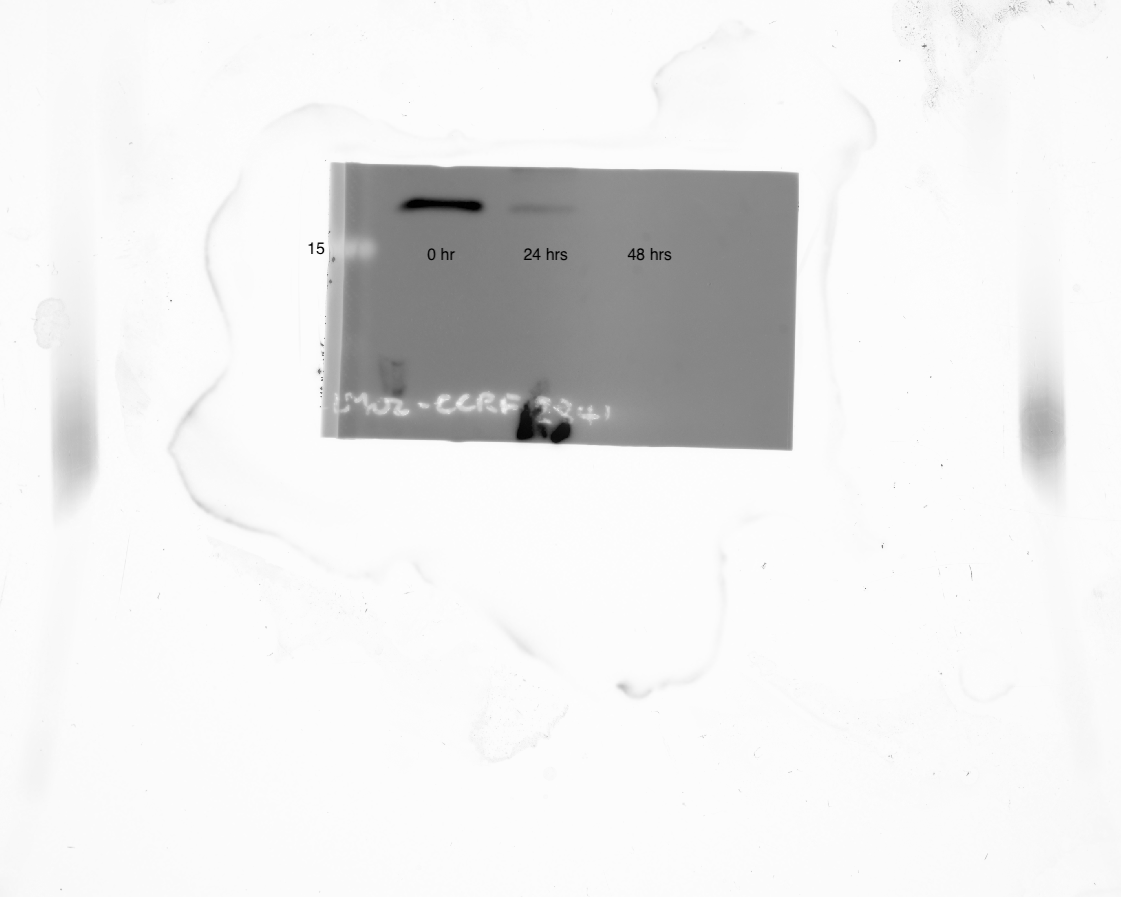

Supplement: Figure 4—source data 1. [file elife-106699-fig4-data1.zip › Figure 4ΓÇösource data 1 PDF files containing original western blots for Figure 4A, indicating the relevant bands and treatments./Raw data/LMO2 CCRF-CEM Abd-VHL.tif]

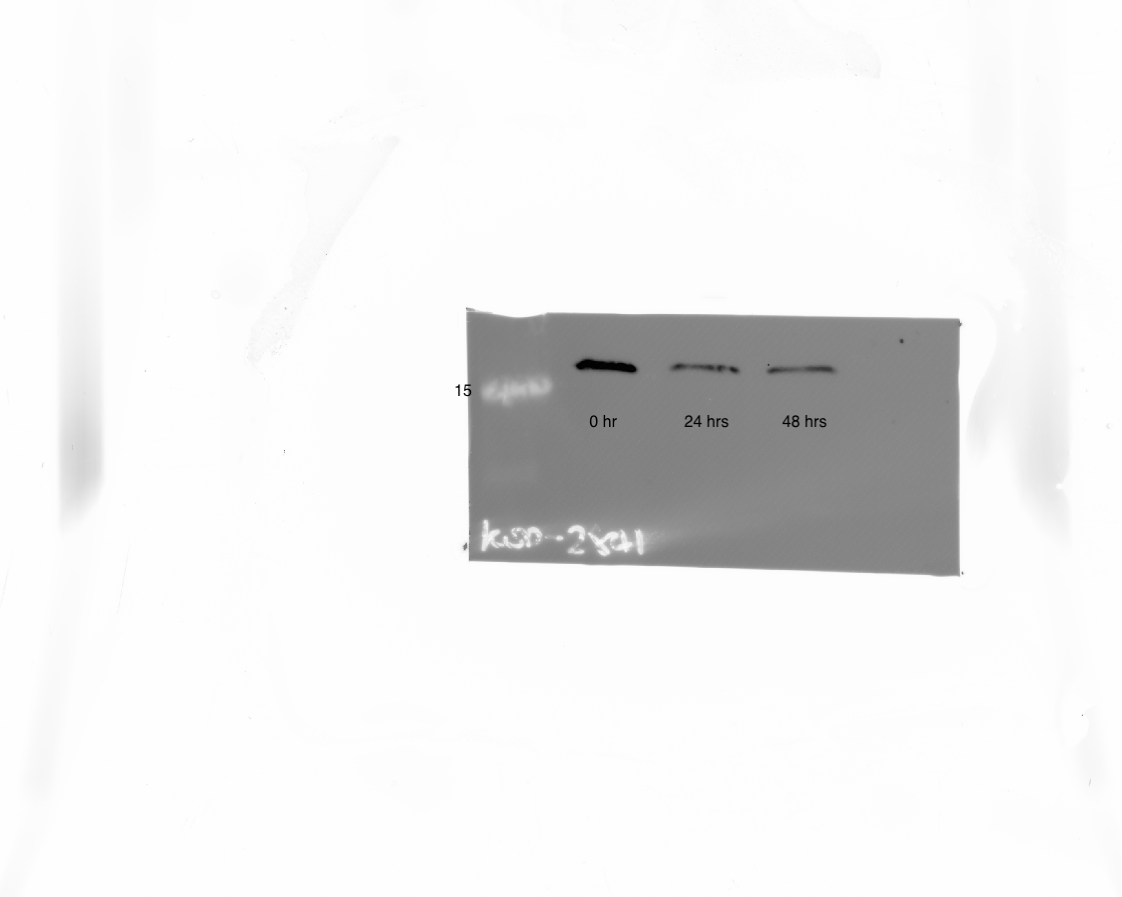

Supplement: Figure 4—source data 1. [file elife-106699-fig4-data1.zip › Figure 4ΓÇösource data 1 PDF files containing original western blots for Figure 4A, indicating the relevant bands and treatments./Raw data/LMO2 KOPT-K1 Abd-CRBN.tif]

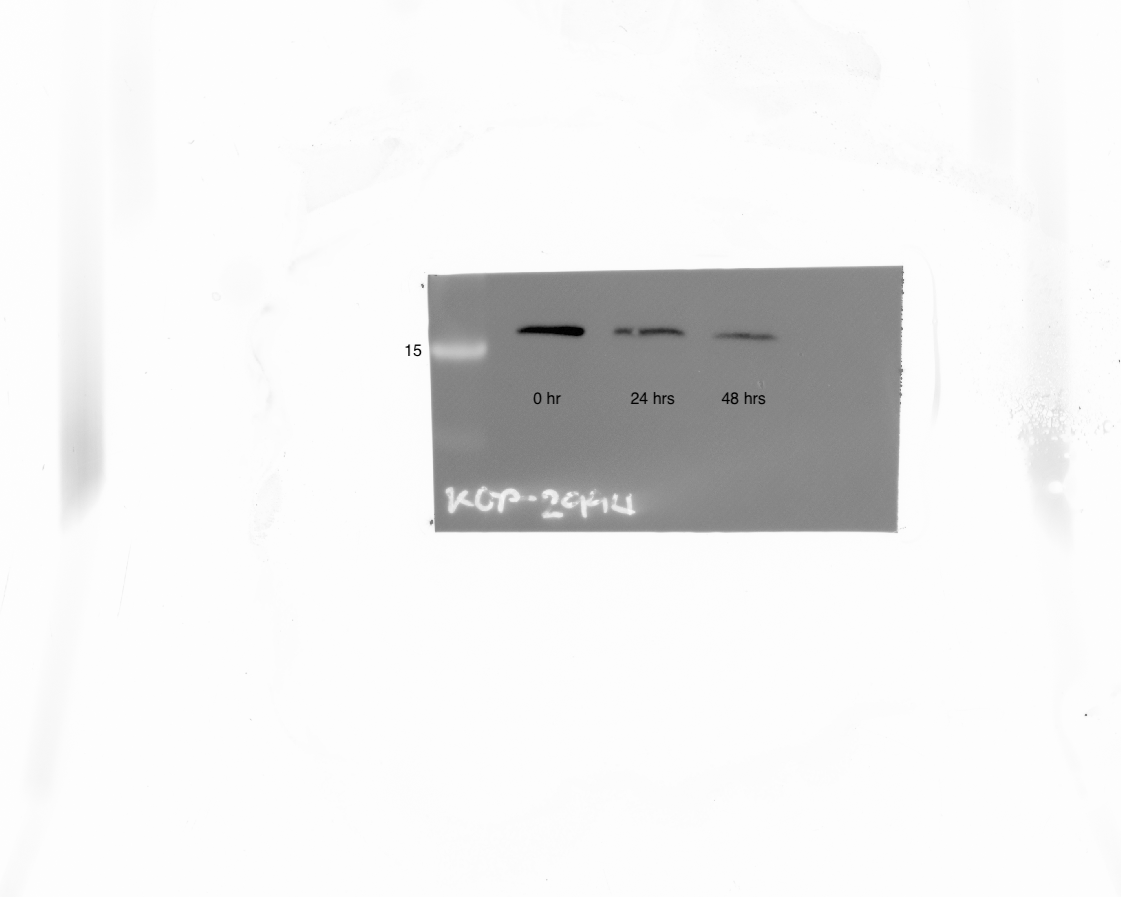

Supplement: Figure 4—source data 1. [file elife-106699-fig4-data1.zip › Figure 4ΓÇösource data 1 PDF files containing original western blots for Figure 4A, indicating the relevant bands and treatments./Raw data/LMO2 KOPT-K1 Abd-VHL.tif]

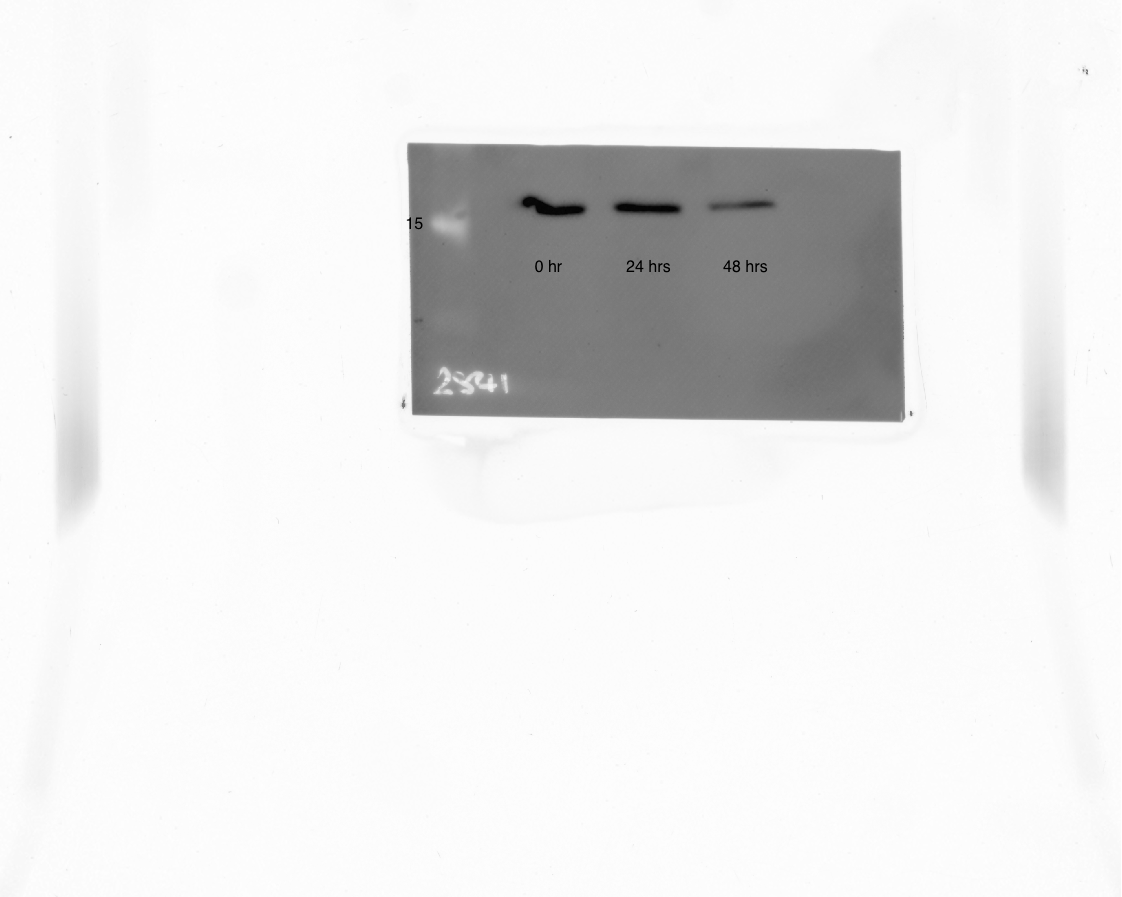

Supplement: Figure 4—source data 1. [file elife-106699-fig4-data1.zip › Figure 4ΓÇösource data 1 PDF files containing original western blots for Figure 4A, indicating the relevant bands and treatments./Raw data/LMO2 LOUCY Abd-CRBN.tif]

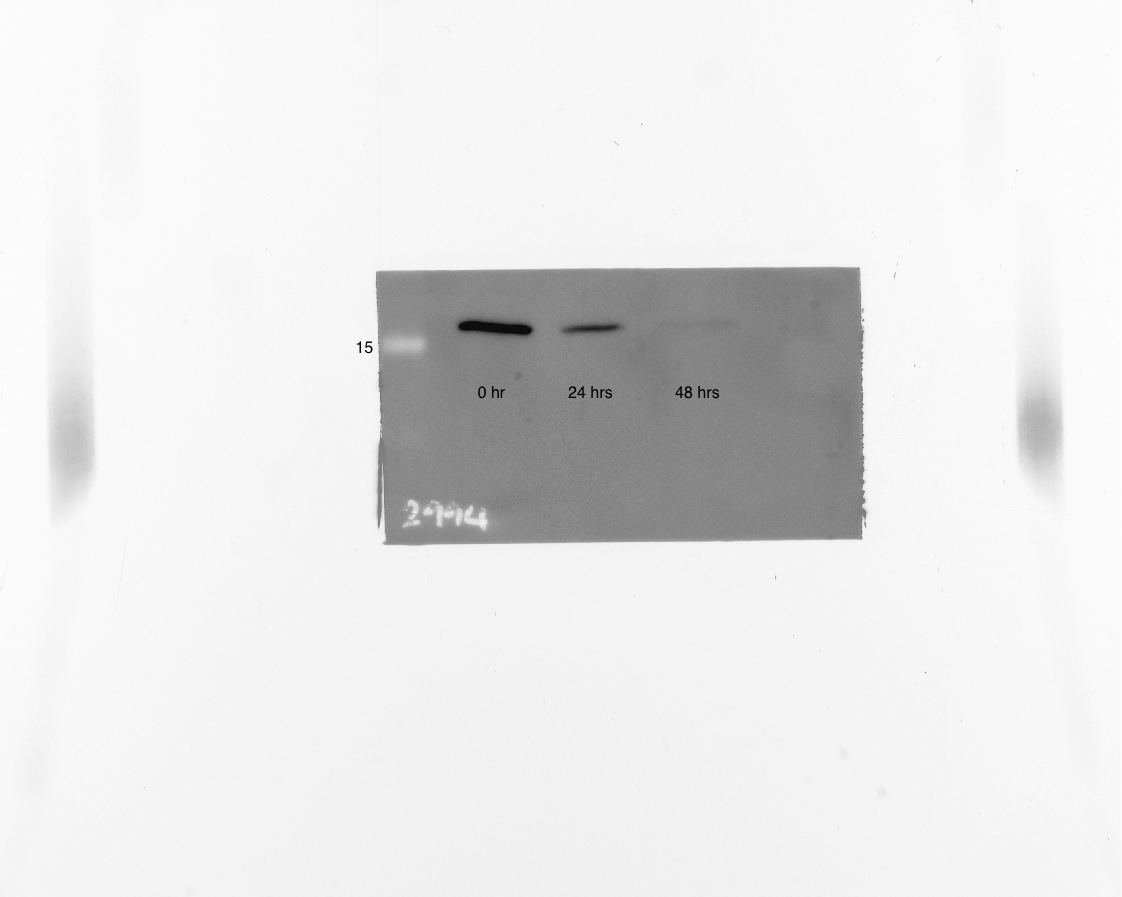

Supplement: Figure 4—source data 1. [file elife-106699-fig4-data1.zip › Figure 4ΓÇösource data 1 PDF files containing original western blots for Figure 4A, indicating the relevant bands and treatments./Raw data/LMO2 LOUCY Abd-VHL.tif]

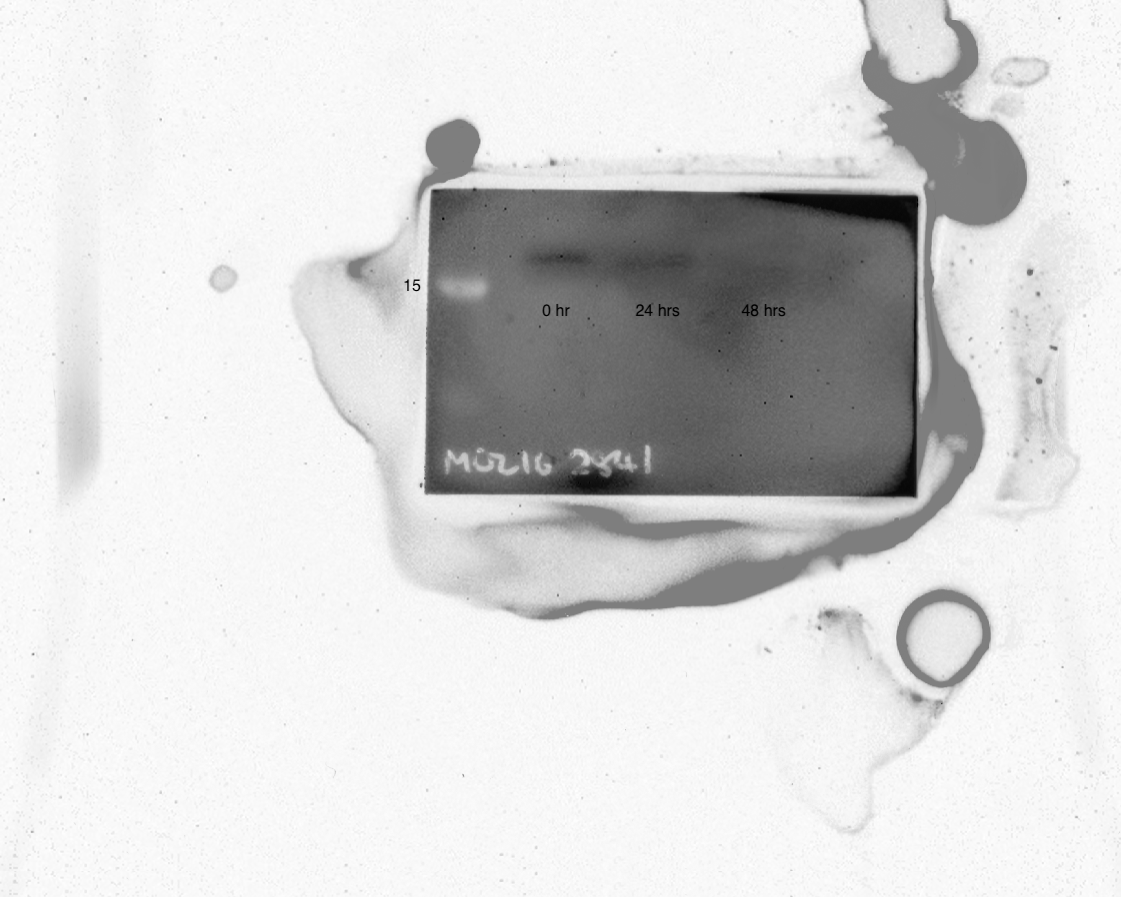

Supplement: Figure 4—source data 1. [file elife-106699-fig4-data1.zip › Figure 4ΓÇösource data 1 PDF files containing original western blots for Figure 4A, indicating the relevant bands and treatments./Raw data/LMO2 MOLT-16 Abd-CRBN.tif]

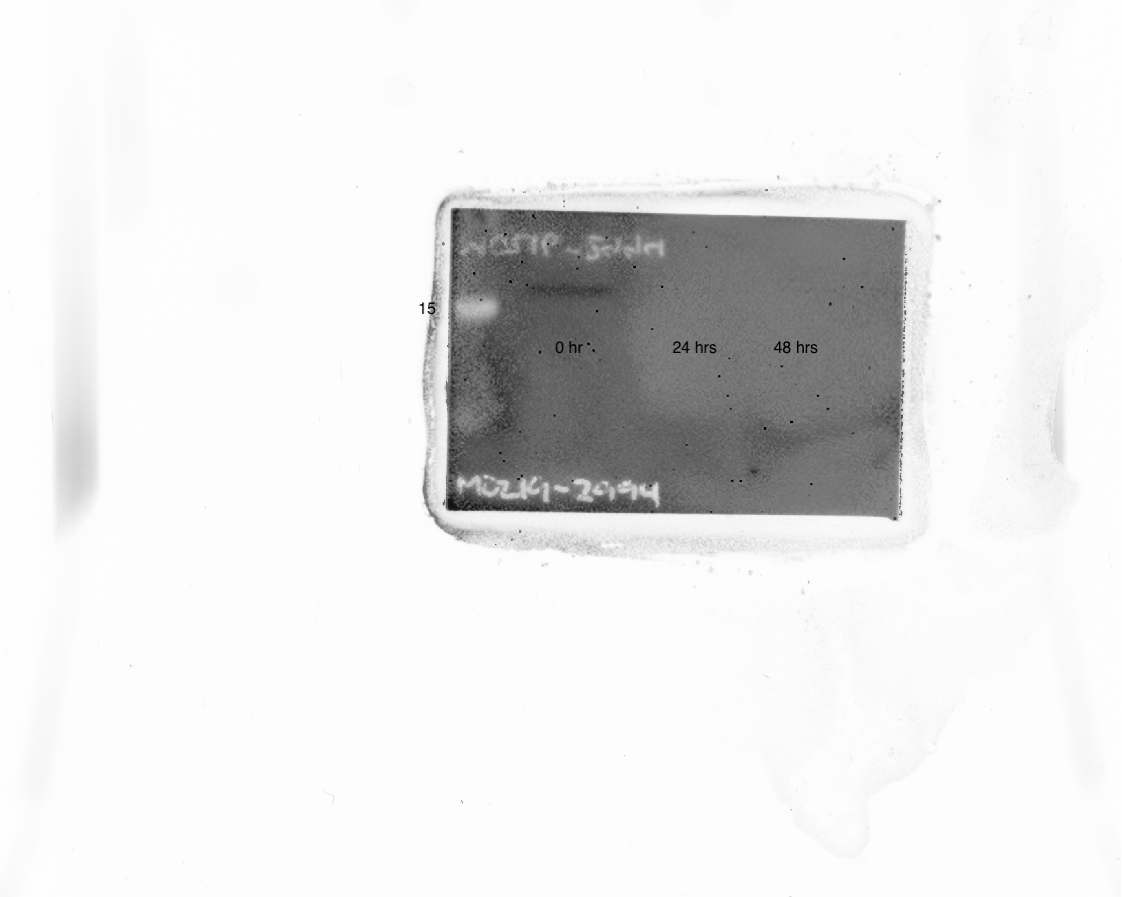

Supplement: Figure 4—source data 1. [file elife-106699-fig4-data1.zip › Figure 4ΓÇösource data 1 PDF files containing original western blots for Figure 4A, indicating the relevant bands and treatments./Raw data/LMO2 MOLT-16 Abd-VHL.tif]

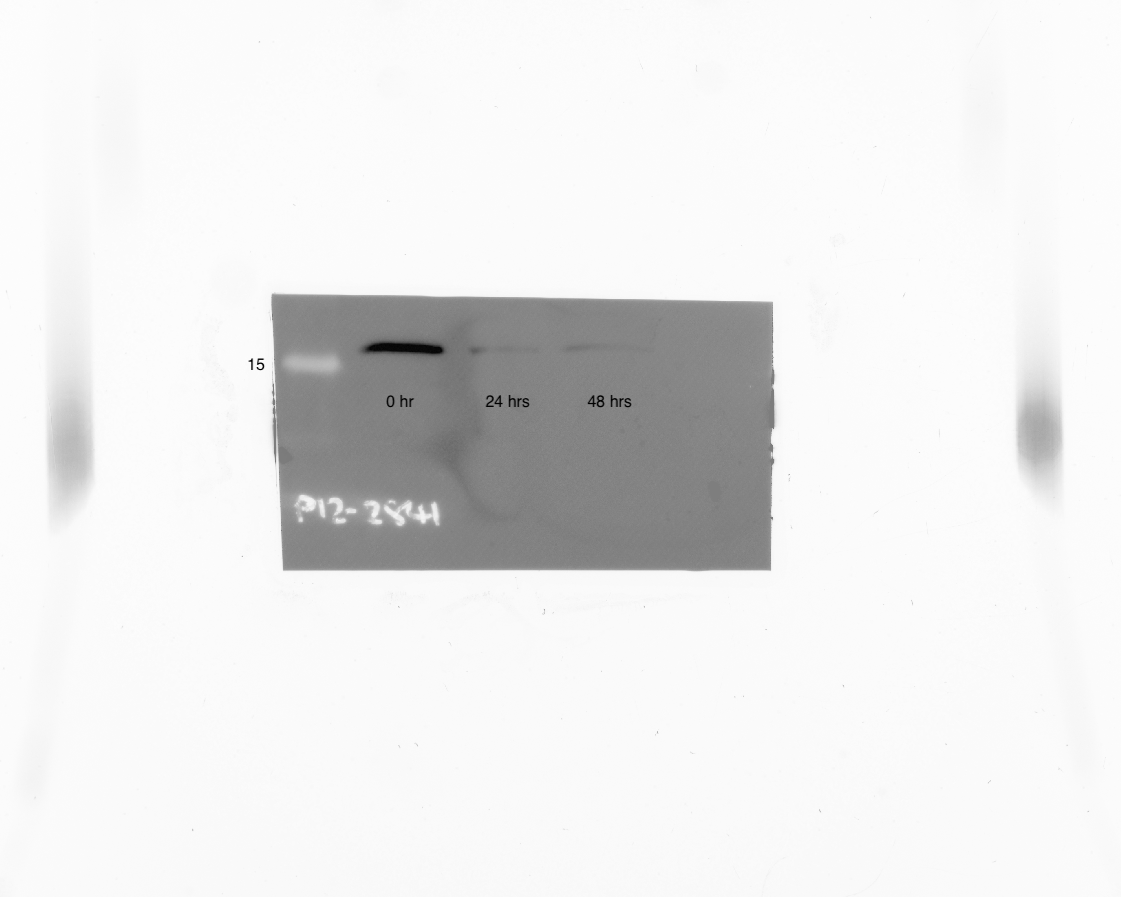

Supplement: Figure 4—source data 1. [file elife-106699-fig4-data1.zip › Figure 4ΓÇösource data 1 PDF files containing original western blots for Figure 4A, indicating the relevant bands and treatments./Raw data/LMO2 P12-Ichikawa Abd-CRBN.tif]

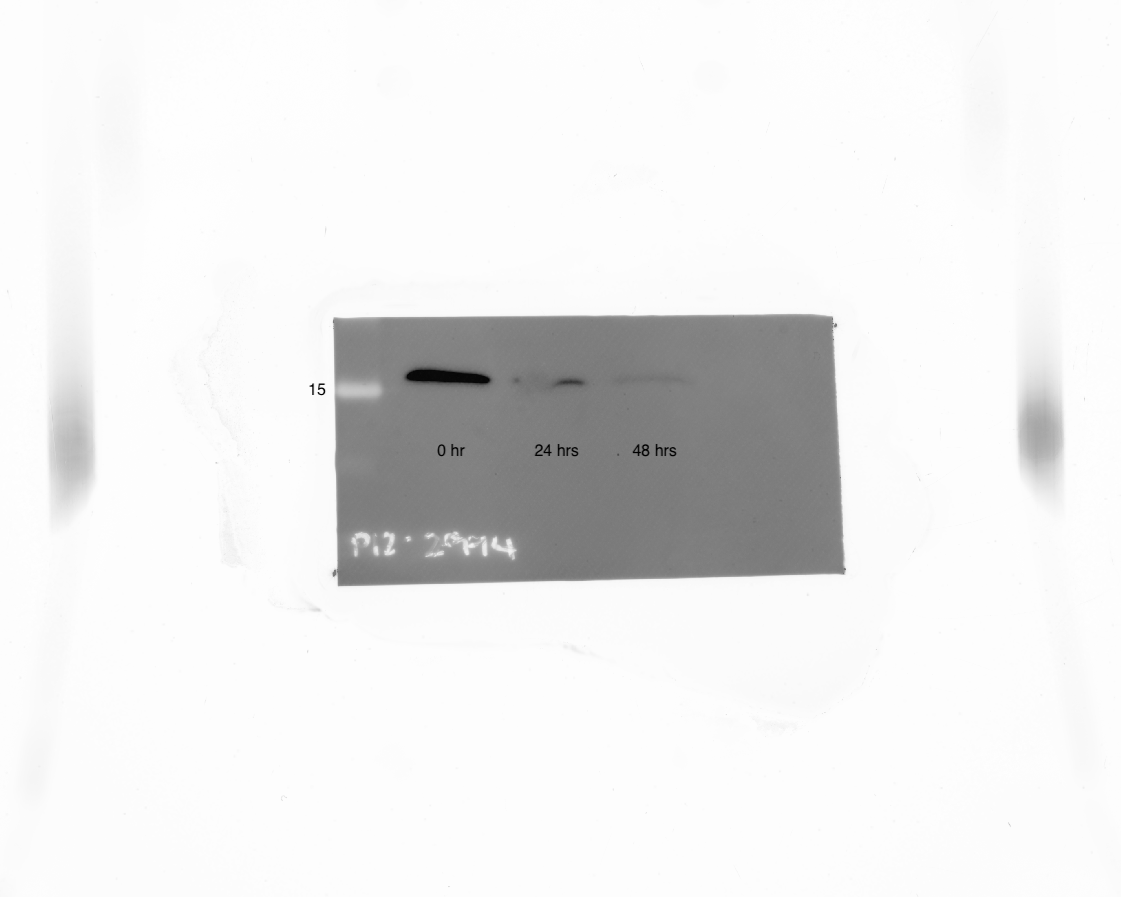

Supplement: Figure 4—source data 1. [file elife-106699-fig4-data1.zip › Figure 4ΓÇösource data 1 PDF files containing original western blots for Figure 4A, indicating the relevant bands and treatments./Raw data/LMO2 P12-Ichikawa Abd-VHL.tif]

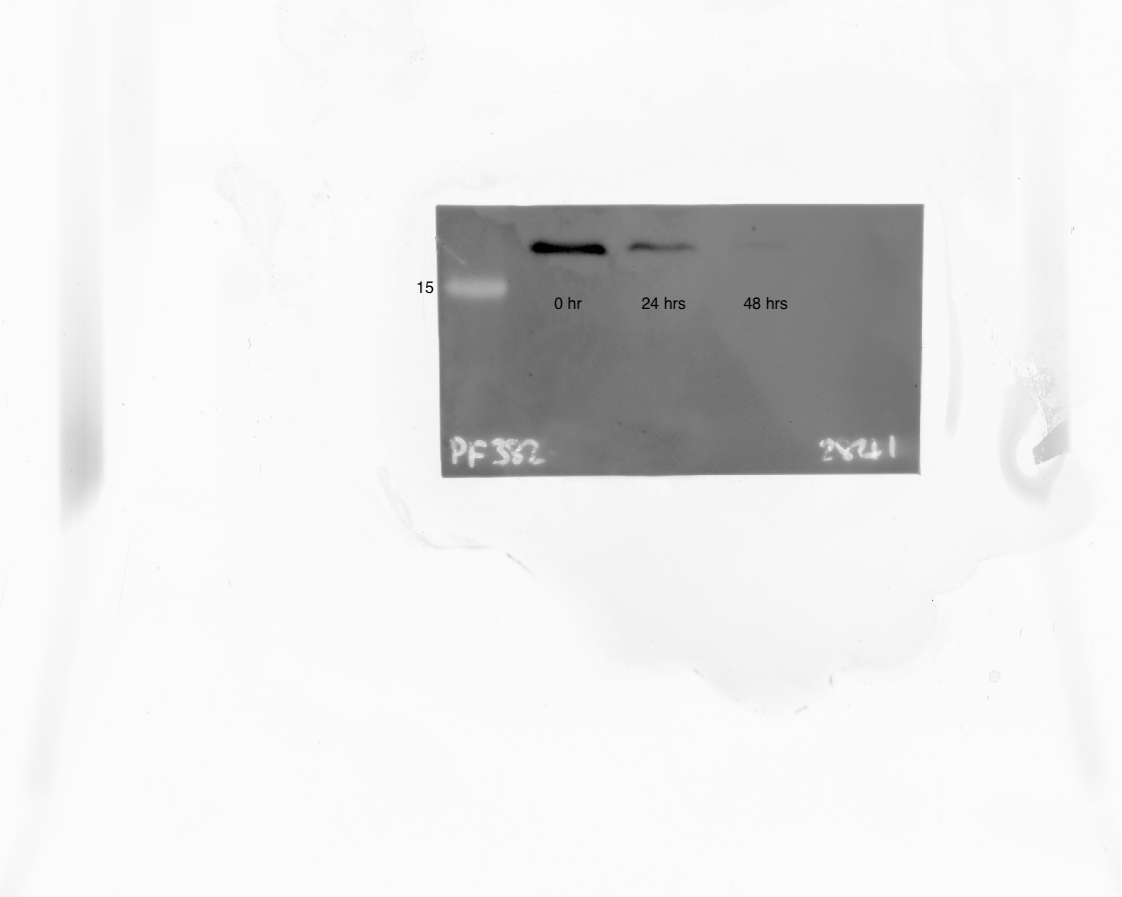

Supplement: Figure 4—source data 1. [file elife-106699-fig4-data1.zip › Figure 4ΓÇösource data 1 PDF files containing original western blots for Figure 4A, indicating the relevant bands and treatments./Raw data/LMO2 PF-382 Abd-CRBN.tif]

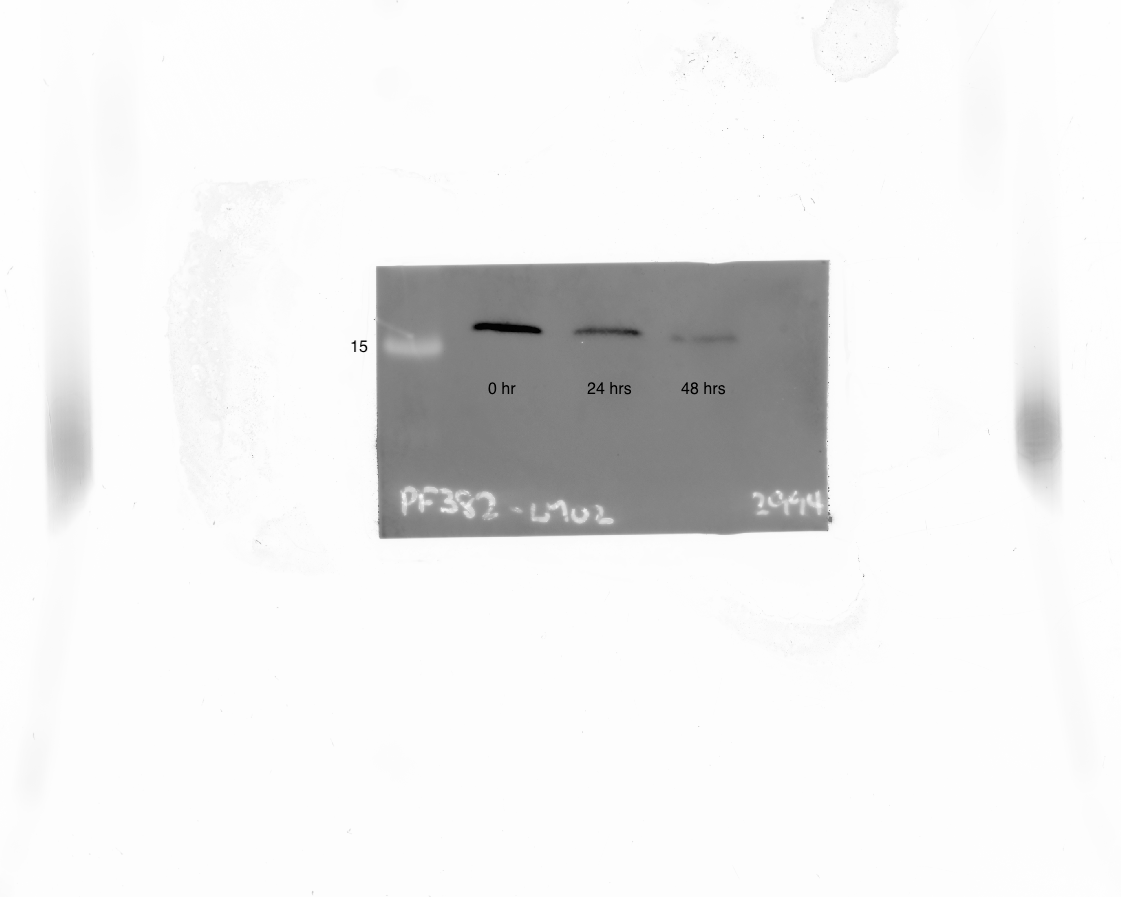

Supplement: Figure 4—source data 1. [file elife-106699-fig4-data1.zip › Figure 4ΓÇösource data 1 PDF files containing original western blots for Figure 4A, indicating the relevant bands and treatments./Raw data/LMO2 PF-382 Abd-VHL.tif]

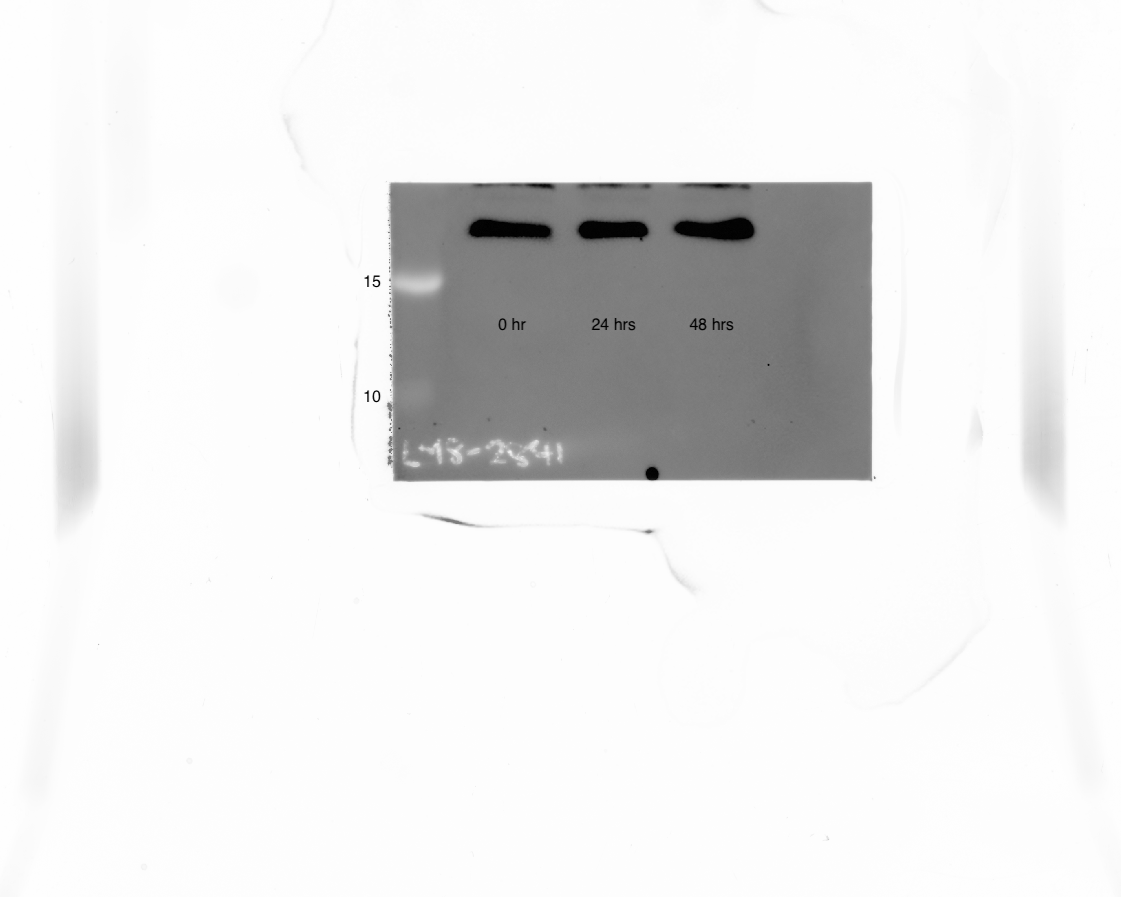

Supplement: Figure 4—source data 1. [file elife-106699-fig4-data1.zip › Figure 4ΓÇösource data 1 PDF files containing original western blots for Figure 4A, indicating the relevant bands and treatments./Raw data/RAS CCRF-CEM Abd-CRBN.tif]

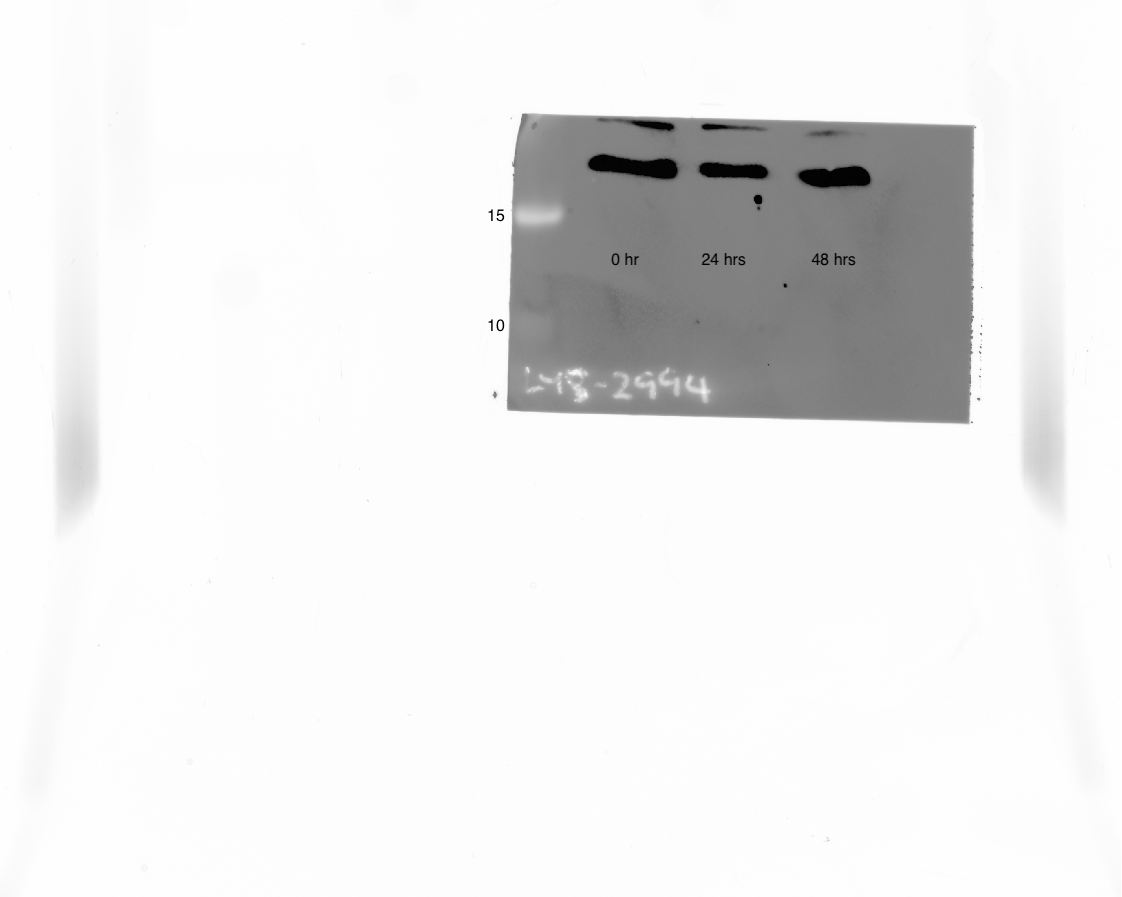

Supplement: Figure 4—source data 1. [file elife-106699-fig4-data1.zip › Figure 4ΓÇösource data 1 PDF files containing original western blots for Figure 4A, indicating the relevant bands and treatments./Raw data/RAS CCRF-CRM Abd-VHL.tif]

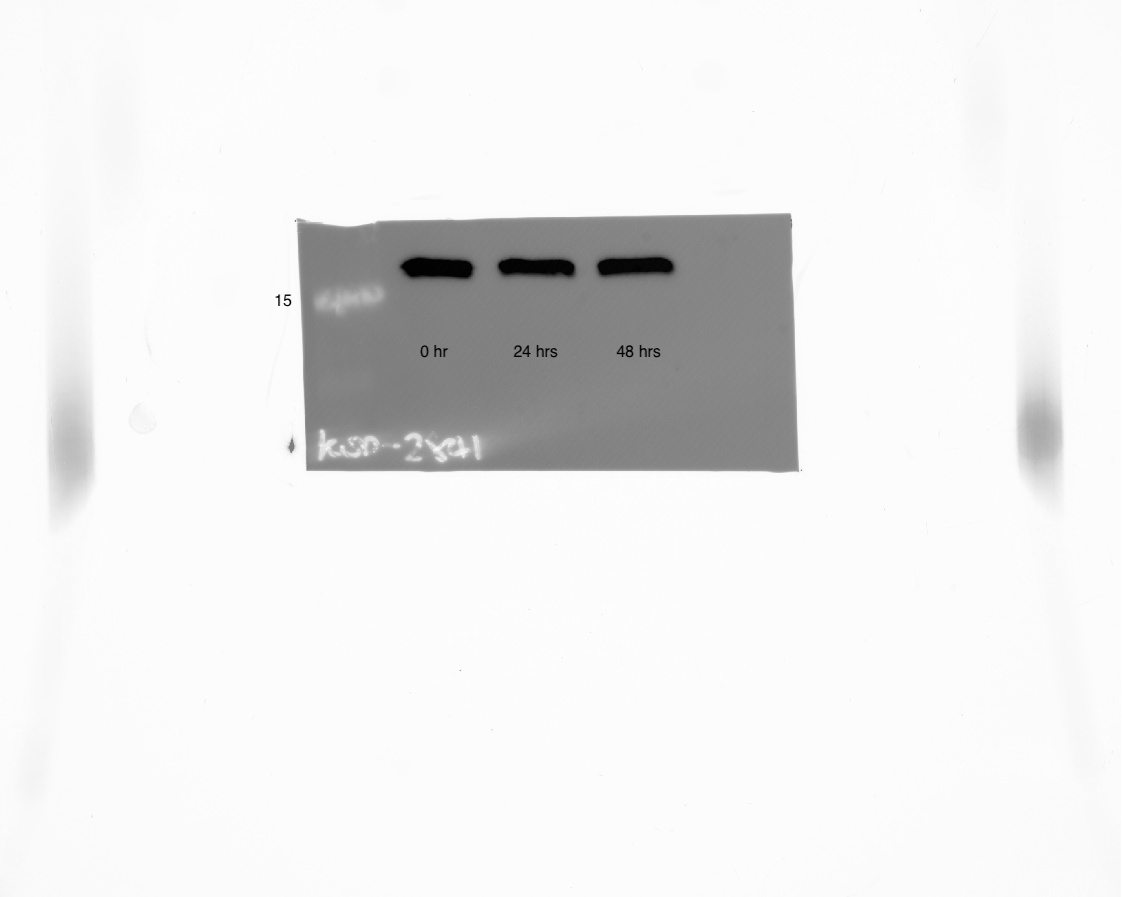

Supplement: Figure 4—source data 1. [file elife-106699-fig4-data1.zip › Figure 4ΓÇösource data 1 PDF files containing original western blots for Figure 4A, indicating the relevant bands and treatments./Raw data/RAS KOPT-K1 Abd-CRBN.tif]

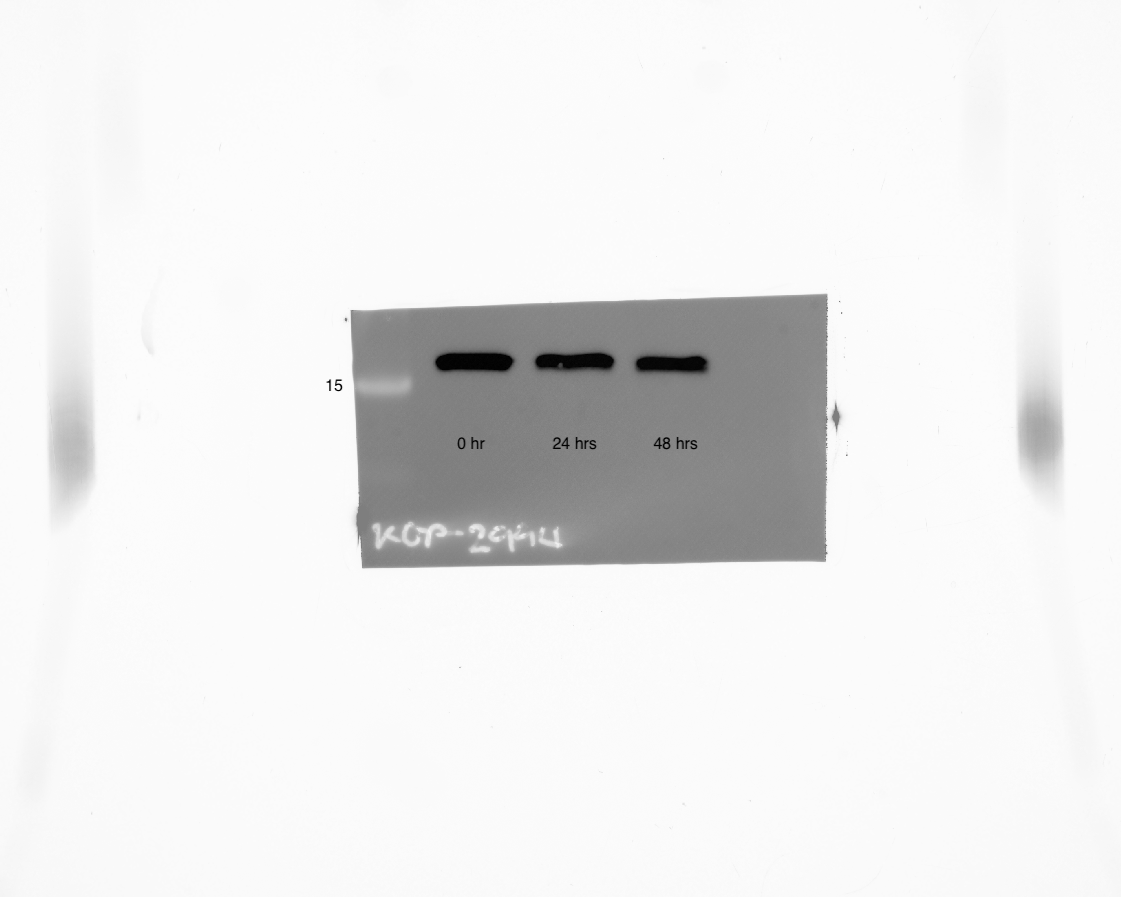

Supplement: Figure 4—source data 1. [file elife-106699-fig4-data1.zip › Figure 4ΓÇösource data 1 PDF files containing original western blots for Figure 4A, indicating the relevant bands and treatments./Raw data/RAS KOPT-K1 Abd-VHL.tif]

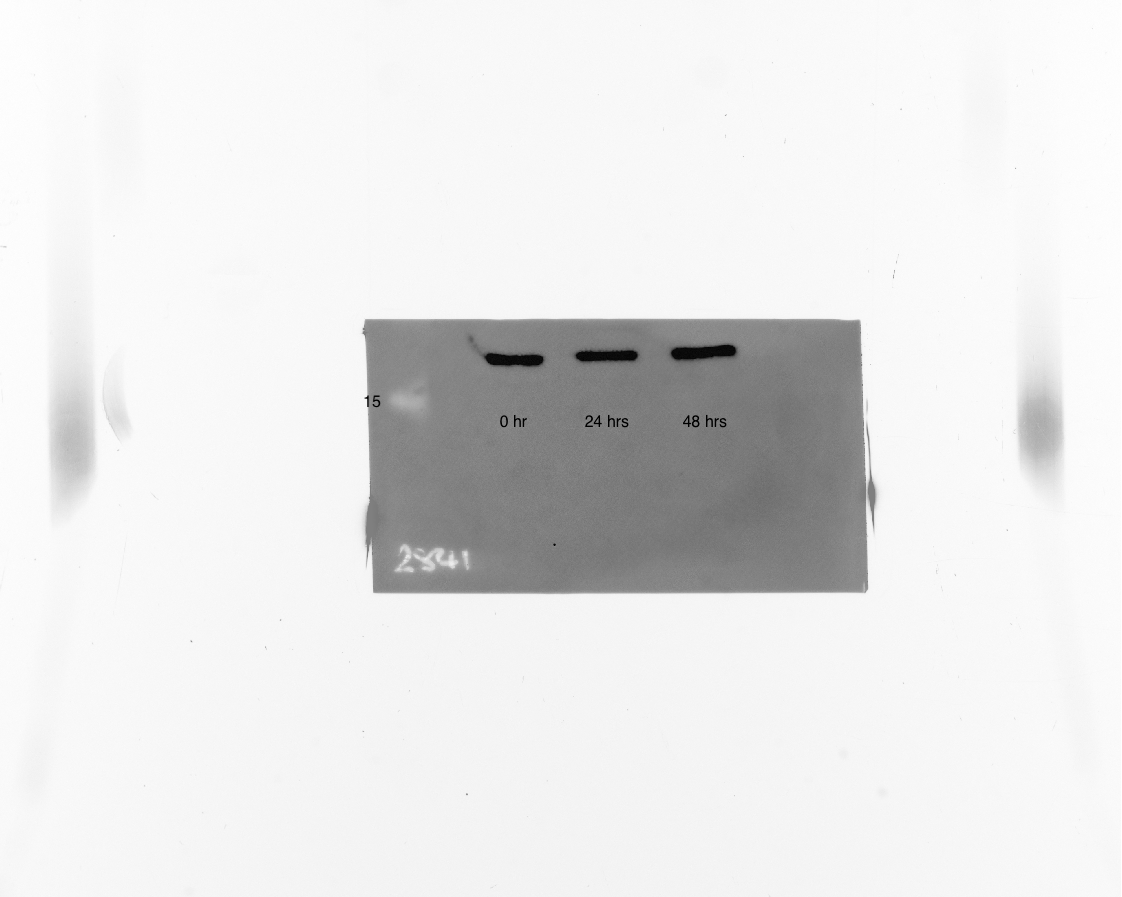

Supplement: Figure 4—source data 1. [file elife-106699-fig4-data1.zip › Figure 4ΓÇösource data 1 PDF files containing original western blots for Figure 4A, indicating the relevant bands and treatments./Raw data/RAS LOUCY Abd-CRBN.tif]

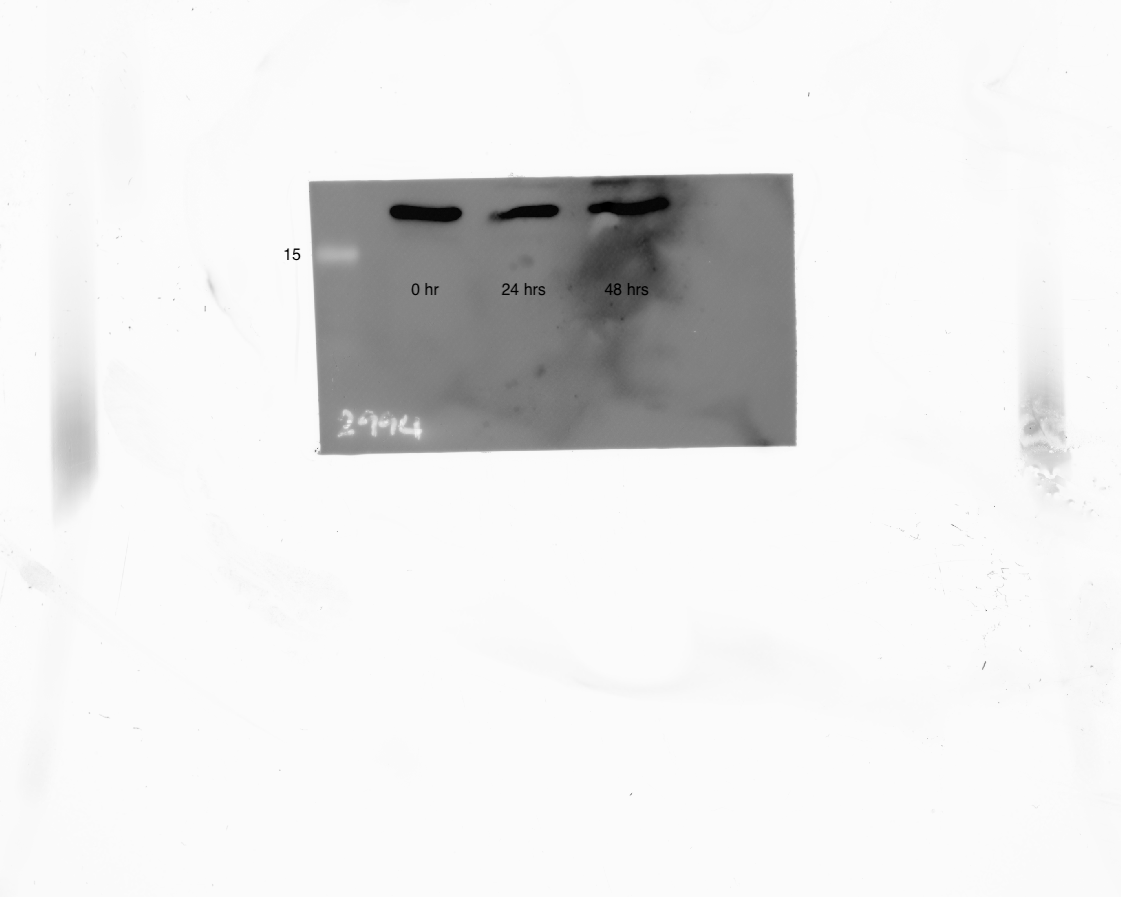

Supplement: Figure 4—source data 1. [file elife-106699-fig4-data1.zip › Figure 4ΓÇösource data 1 PDF files containing original western blots for Figure 4A, indicating the relevant bands and treatments./Raw data/RAS LOUCY Abd-VHL.tif]

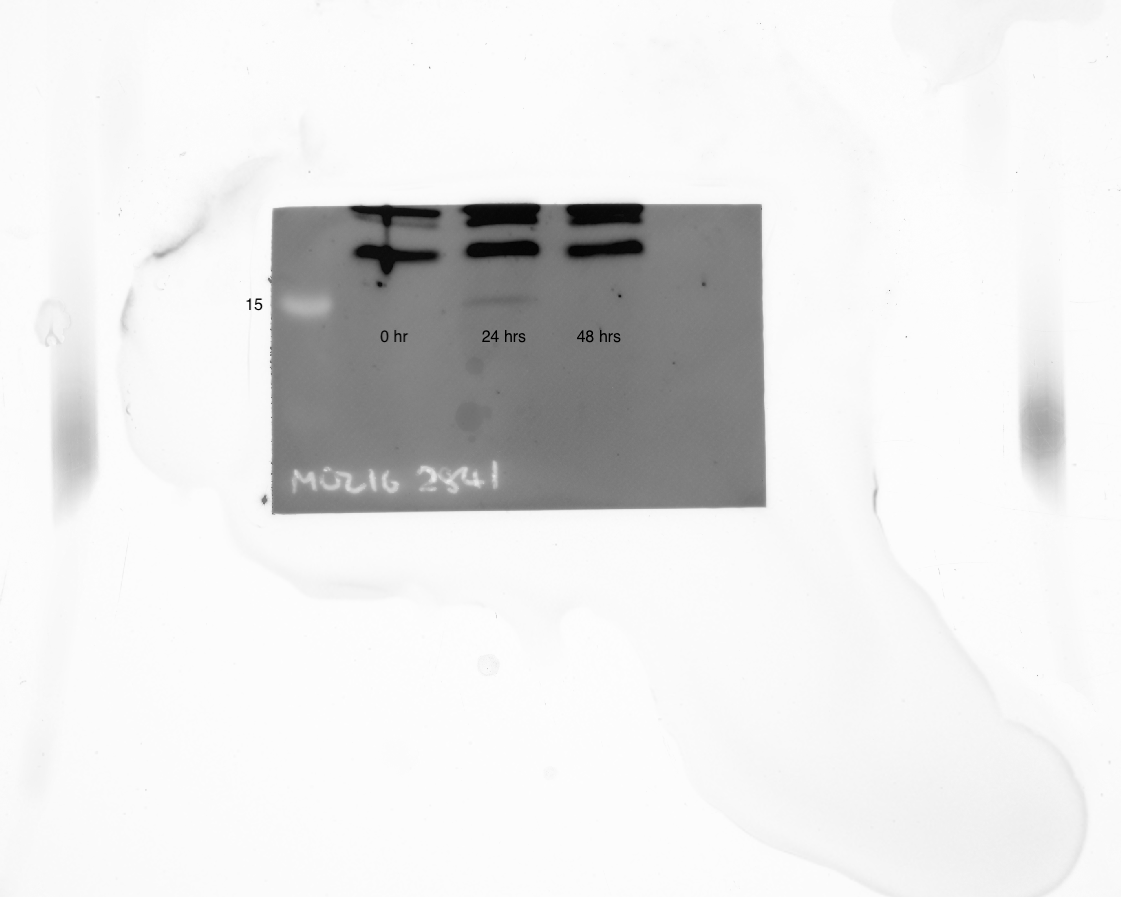

Supplement: Figure 4—source data 1. [file elife-106699-fig4-data1.zip › Figure 4ΓÇösource data 1 PDF files containing original western blots for Figure 4A, indicating the relevant bands and treatments./Raw data/RAS MOLT-16 Abd-CRBN.tif]

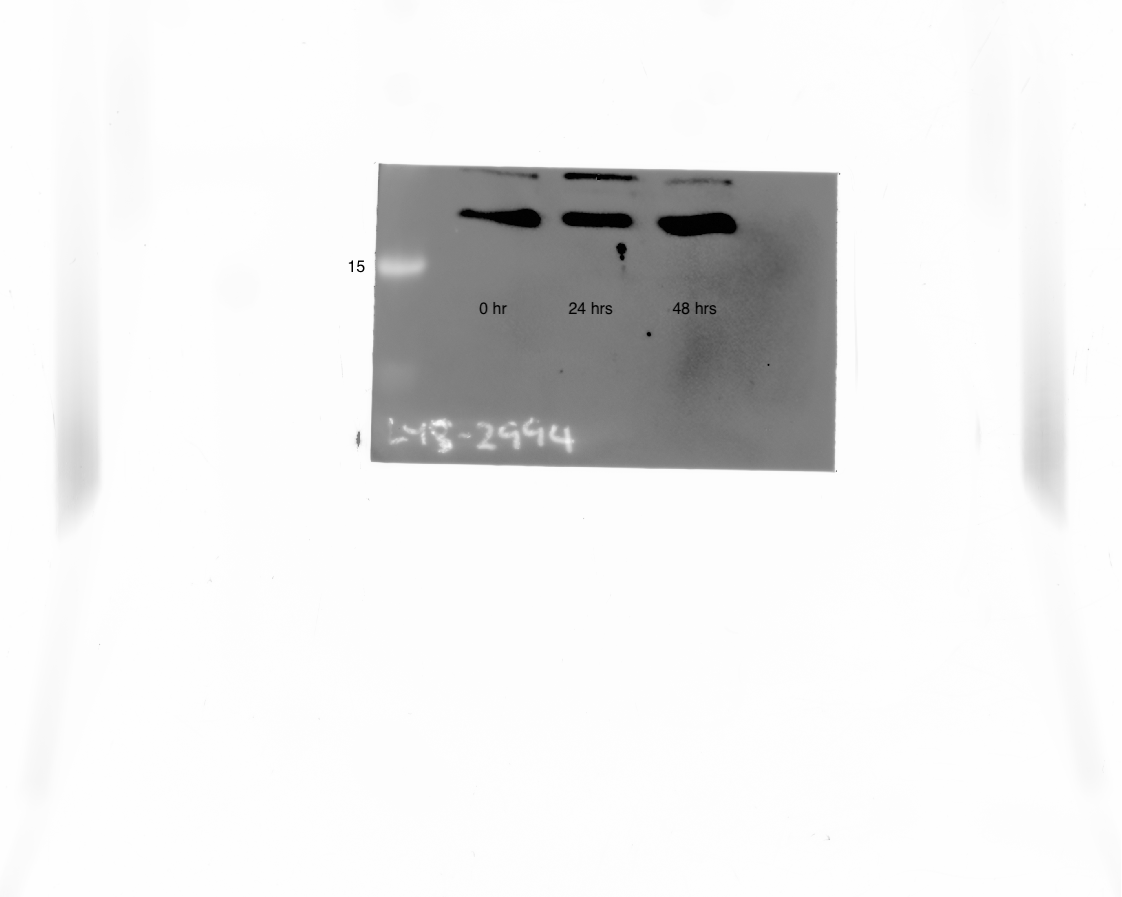

Supplement: Figure 4—source data 1. [file elife-106699-fig4-data1.zip › Figure 4ΓÇösource data 1 PDF files containing original western blots for Figure 4A, indicating the relevant bands and treatments./Raw data/RAS MOLT-16 Abd-VHL.tif]

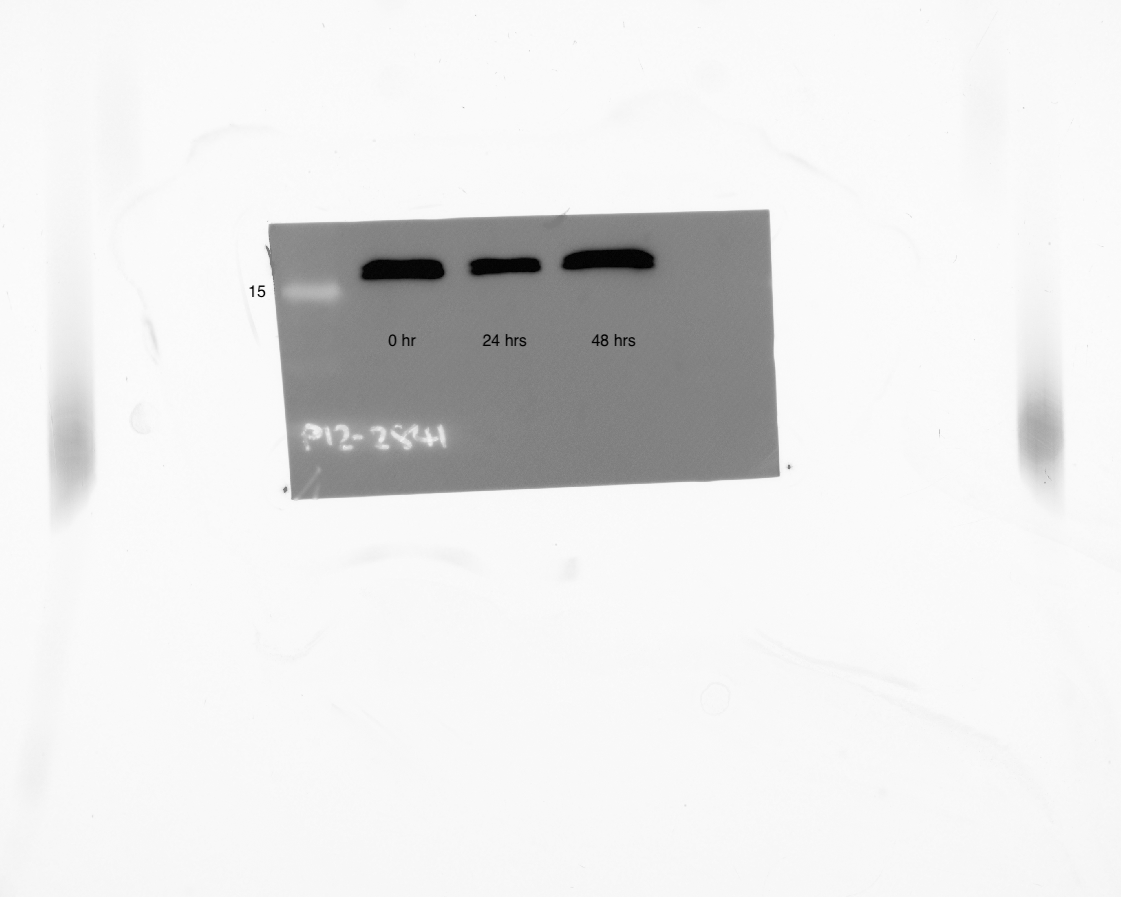

Supplement: Figure 4—source data 1. [file elife-106699-fig4-data1.zip › Figure 4ΓÇösource data 1 PDF files containing original western blots for Figure 4A, indicating the relevant bands and treatments./Raw data/RAS P12-Ichikawa Abd-CRBN.tif]

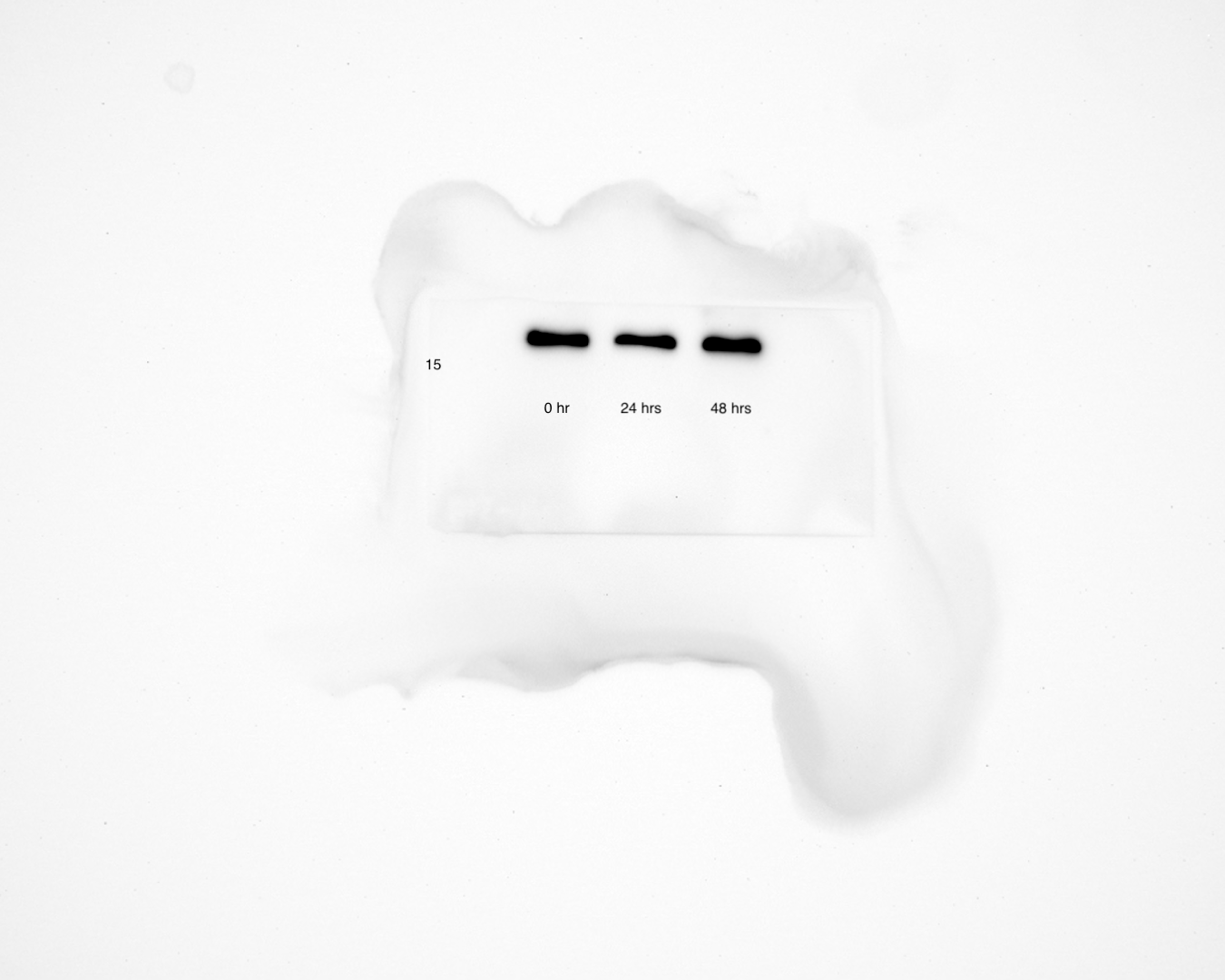

Supplement: Figure 4—source data 1. [file elife-106699-fig4-data1.zip › Figure 4ΓÇösource data 1 PDF files containing original western blots for Figure 4A, indicating the relevant bands and treatments./Raw data/RAS P12-Ichikawa Abd-VHL.tif]

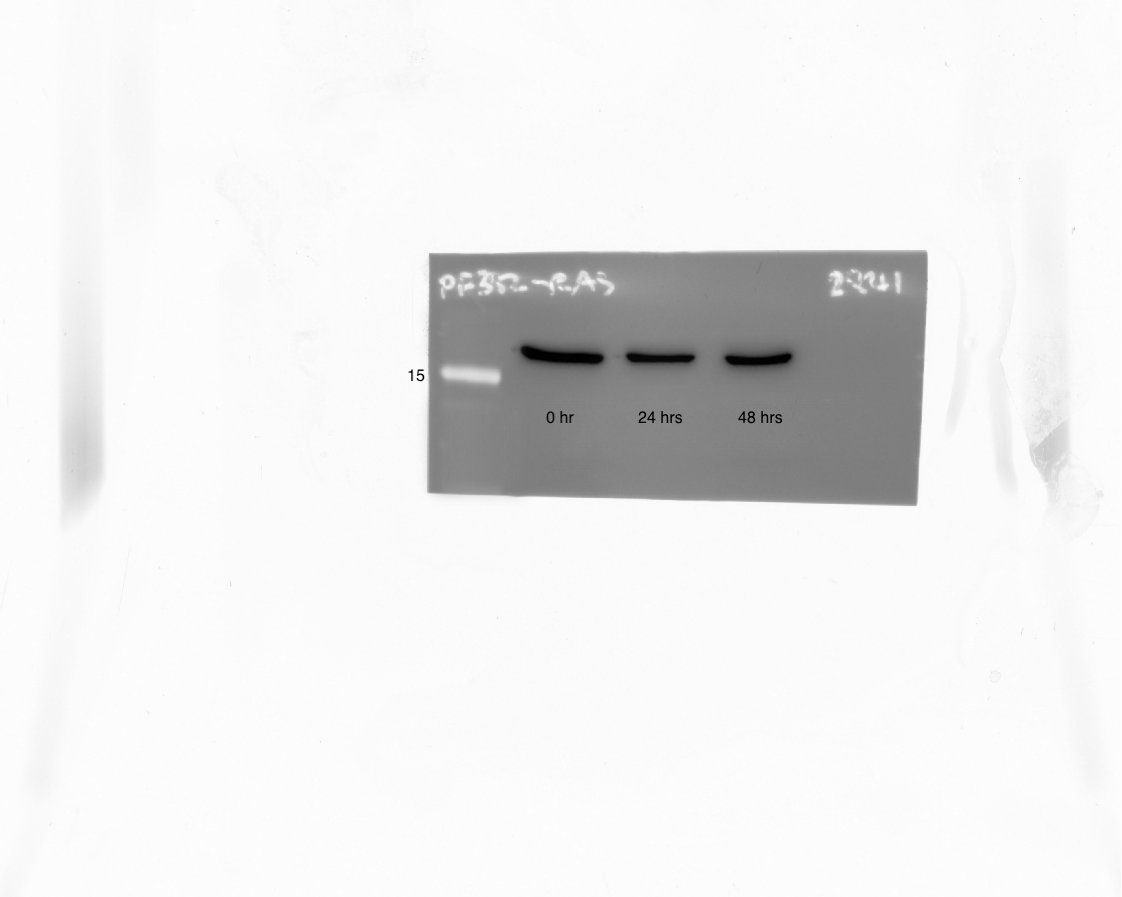

Supplement: Figure 4—source data 1. [file elife-106699-fig4-data1.zip › Figure 4ΓÇösource data 1 PDF files containing original western blots for Figure 4A, indicating the relevant bands and treatments./Raw data/RAS PF-382 Abd-CRBN.tif]

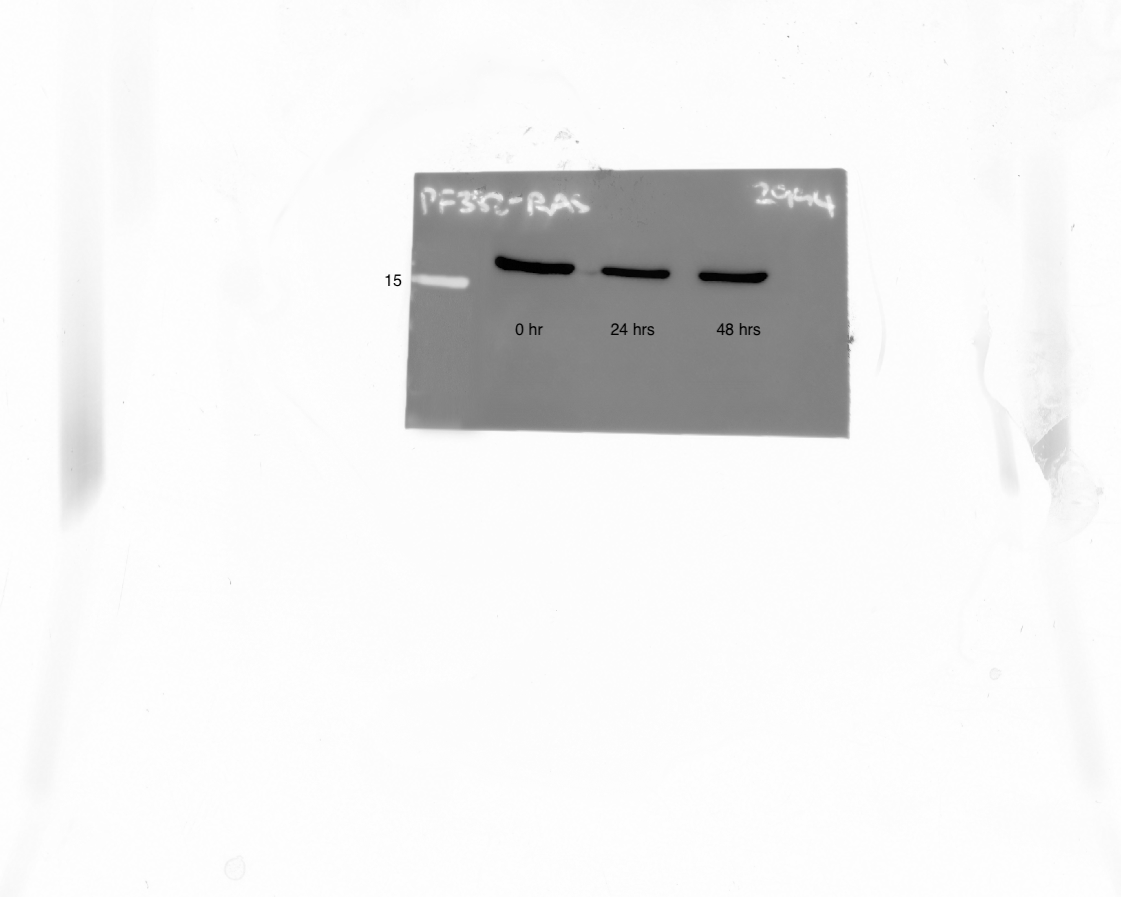

Supplement: Figure 4—source data 1. [file elife-106699-fig4-data1.zip › Figure 4ΓÇösource data 1 PDF files containing original western blots for Figure 4A, indicating the relevant bands and treatments./Raw data/RAS PF-382 Abd-VHL.tif]

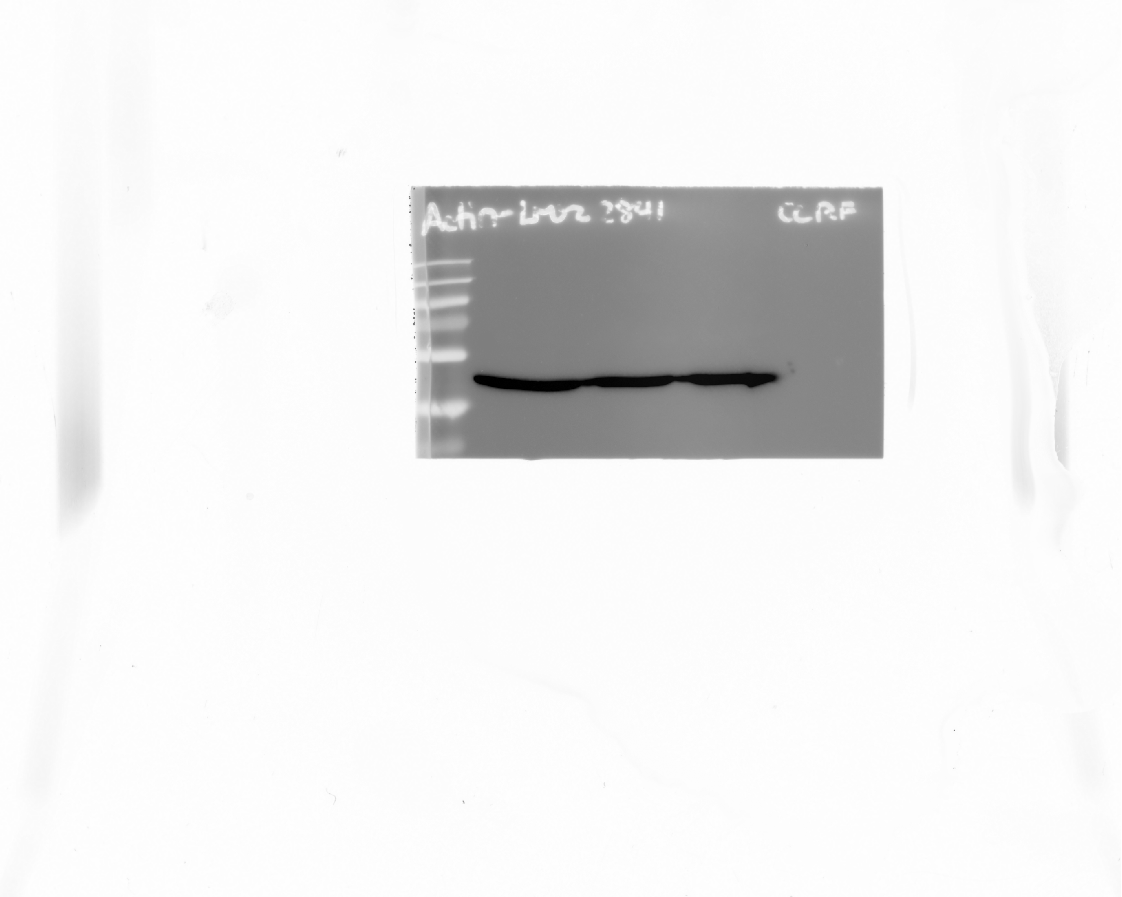

Supplement: Figure 4—source data 2. [file elife-106699-fig4-data2.zip › Figure 4ΓÇösource data 2 Original files for Western blot analysis displayed in Figure 4A./Actin CCRF-CEM Abd-CRBN.tif]

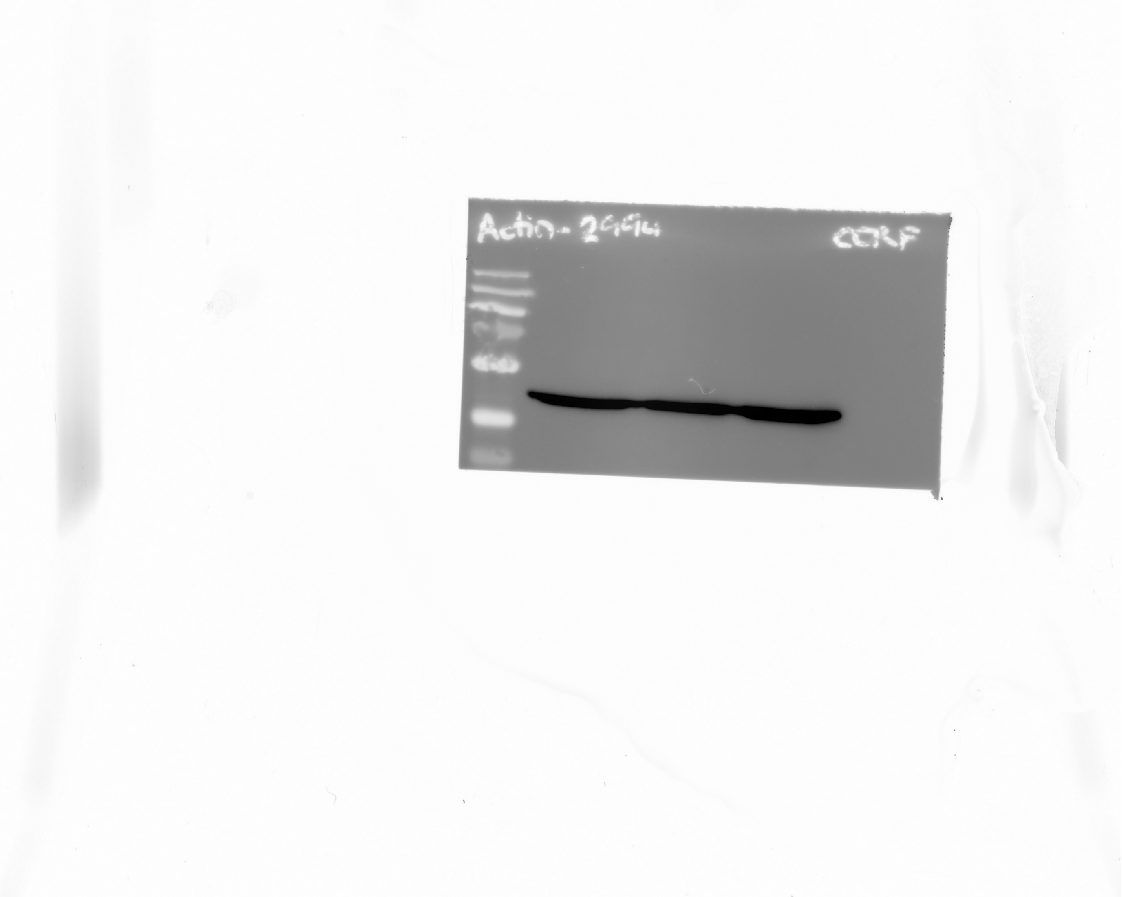

Supplement: Figure 4—source data 2. [file elife-106699-fig4-data2.zip › Figure 4ΓÇösource data 2 Original files for Western blot analysis displayed in Figure 4A./Actin CCRF-CEM Abd-VHL.tif]

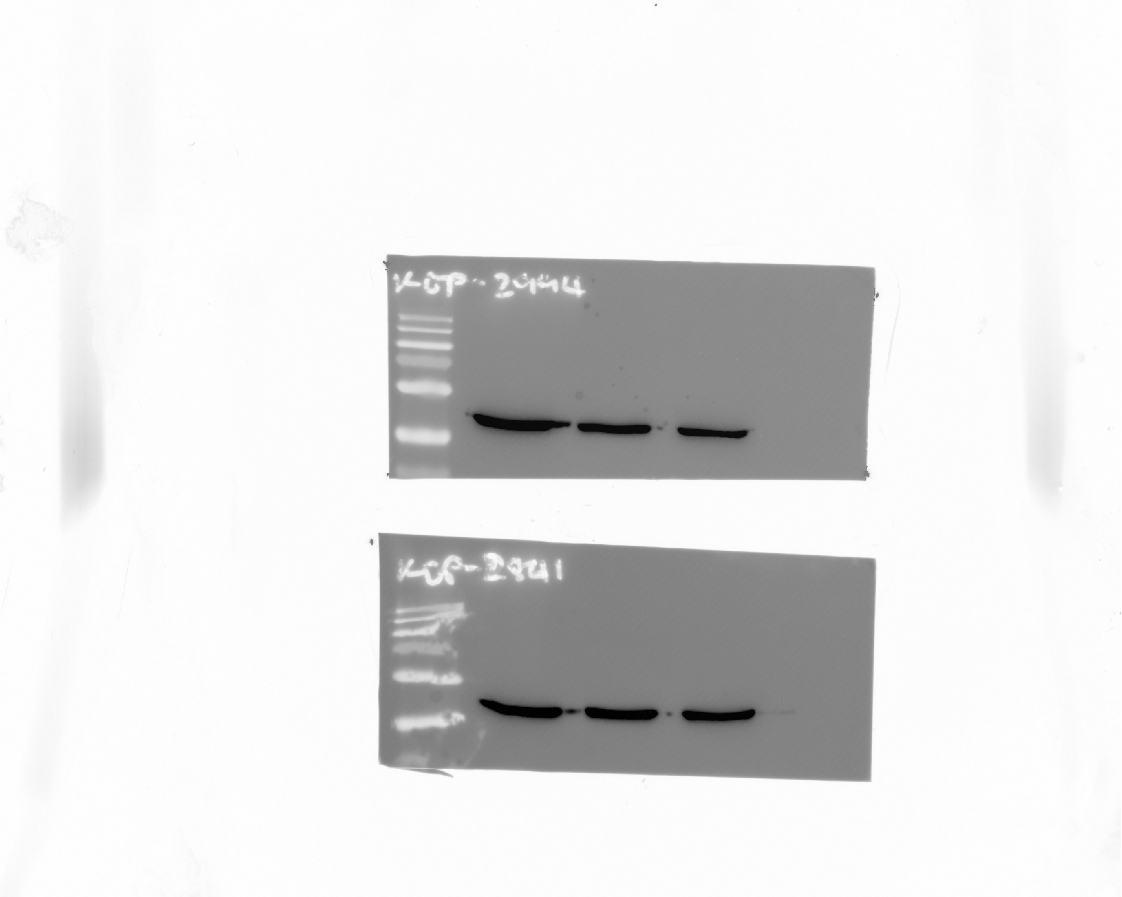

Supplement: Figure 4—source data 2. [file elife-106699-fig4-data2.zip › Figure 4ΓÇösource data 2 Original files for Western blot analysis displayed in Figure 4A./Actin KOPT-K1 Abd-CRBN abd Avd-VHL.tif]

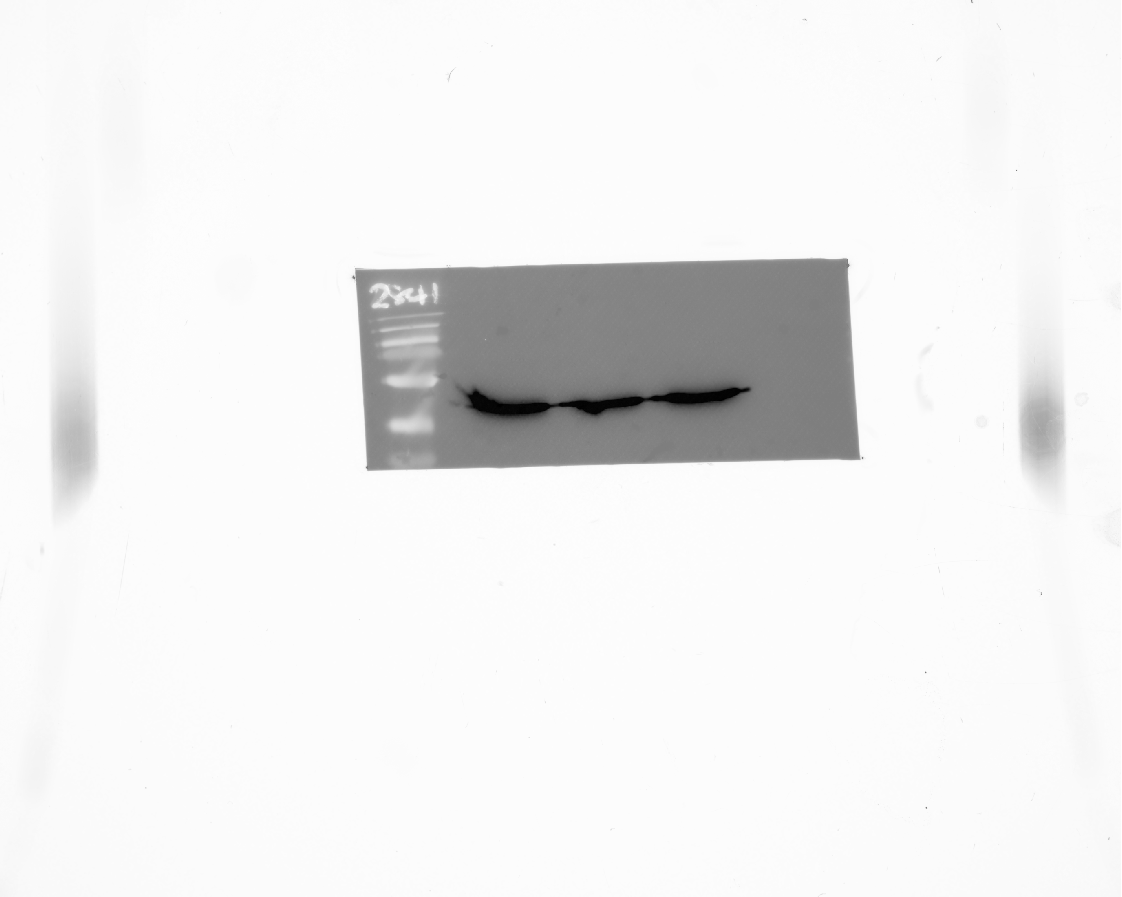

Supplement: Figure 4—source data 2. [file elife-106699-fig4-data2.zip › Figure 4ΓÇösource data 2 Original files for Western blot analysis displayed in Figure 4A./Actin LOUCY Abd-CRBN.tif]

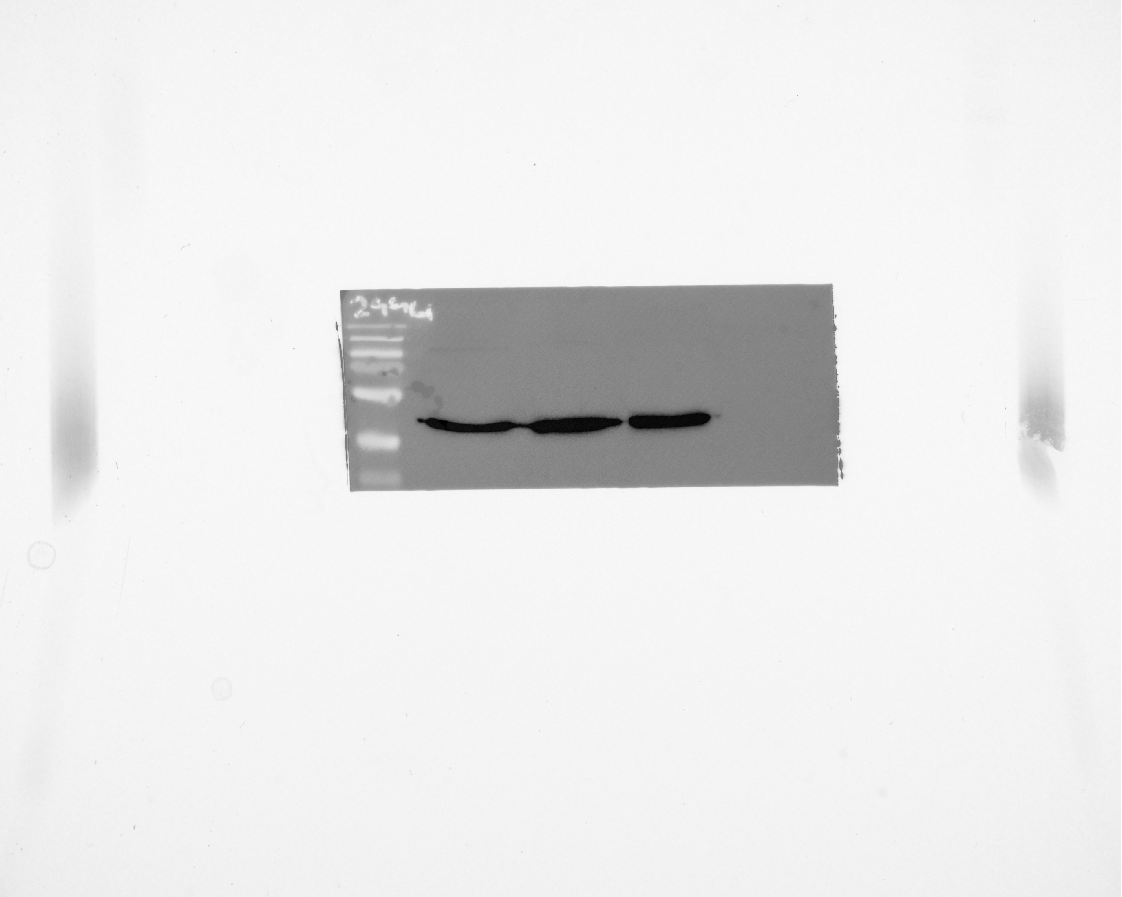

Supplement: Figure 4—source data 2. [file elife-106699-fig4-data2.zip › Figure 4ΓÇösource data 2 Original files for Western blot analysis displayed in Figure 4A./Actin LOUCY Abd-VHL.tif]

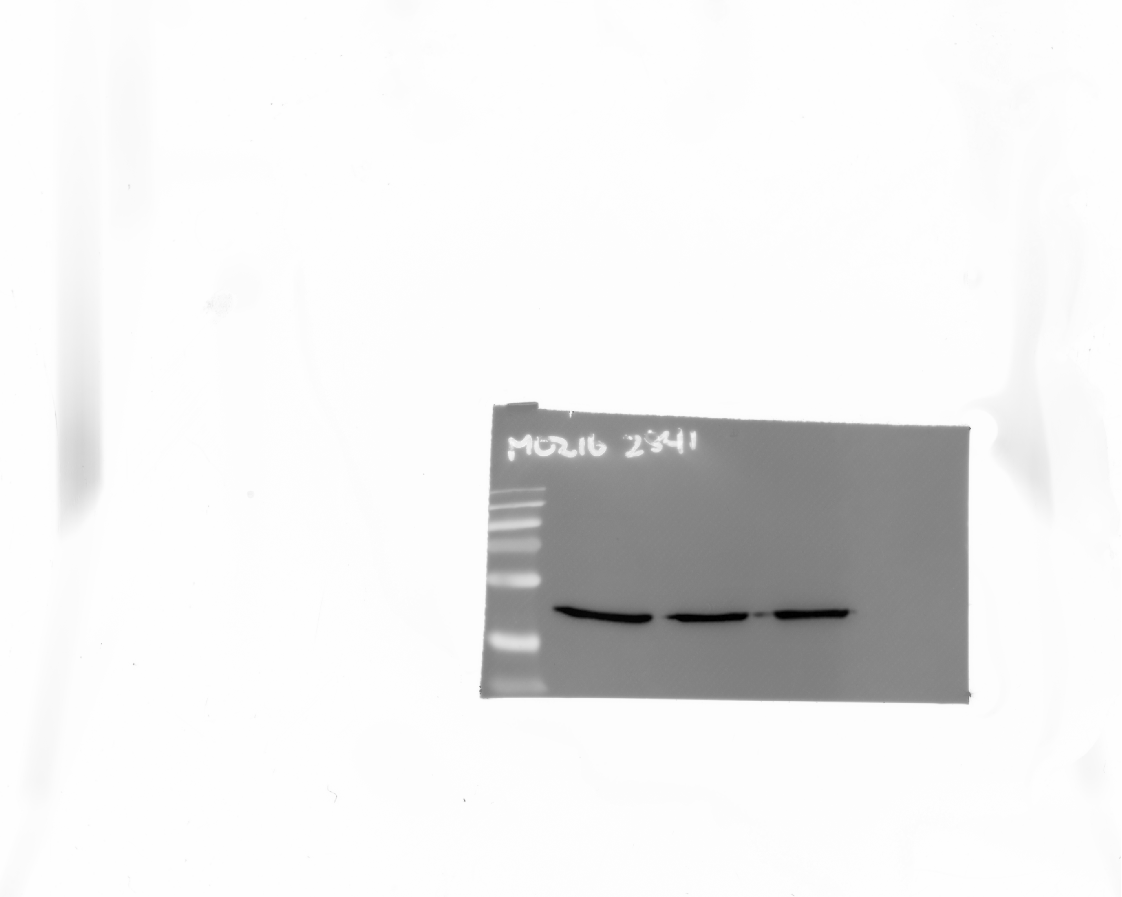

Supplement: Figure 4—source data 2. [file elife-106699-fig4-data2.zip › Figure 4ΓÇösource data 2 Original files for Western blot analysis displayed in Figure 4A./Actin MOLT-16 Abd-CRBN.tif]

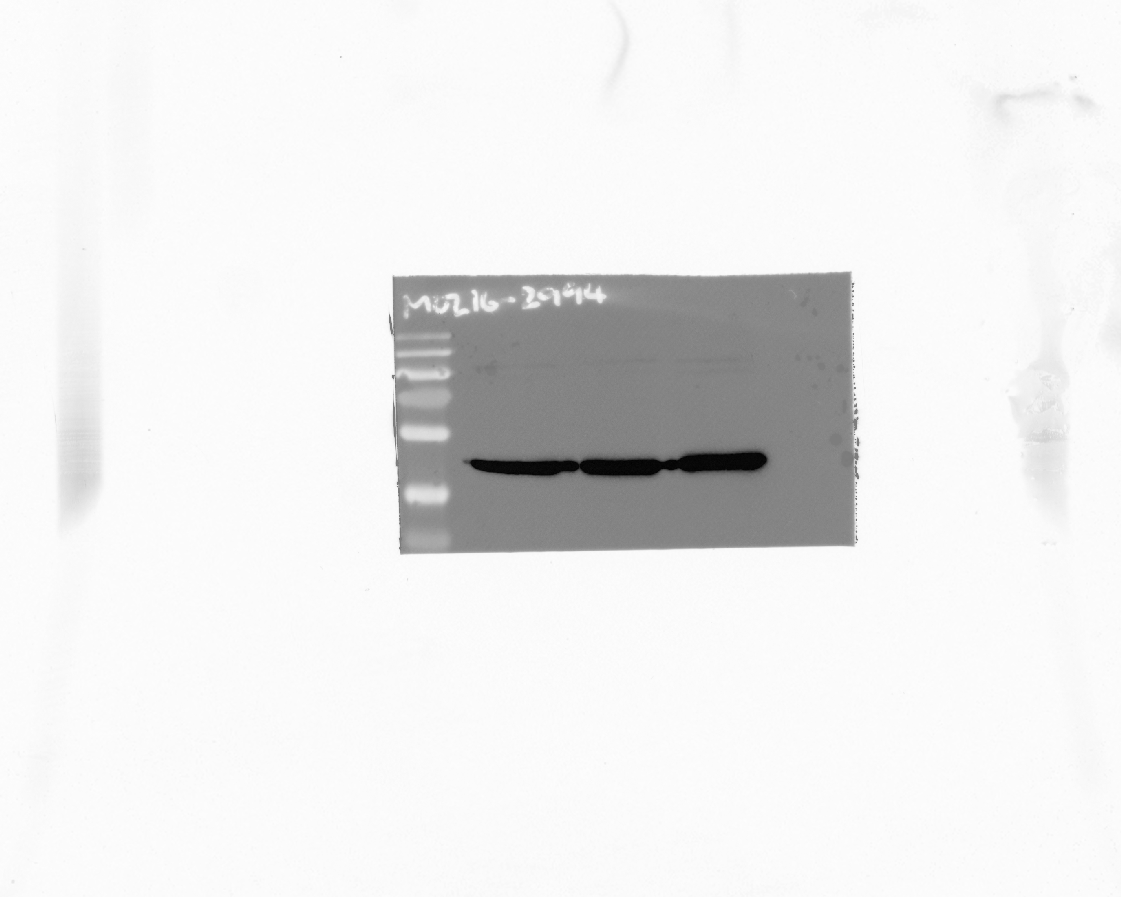

Supplement: Figure 4—source data 2. [file elife-106699-fig4-data2.zip › Figure 4ΓÇösource data 2 Original files for Western blot analysis displayed in Figure 4A./Actin MOLT-16 Abd-VHL.tif]

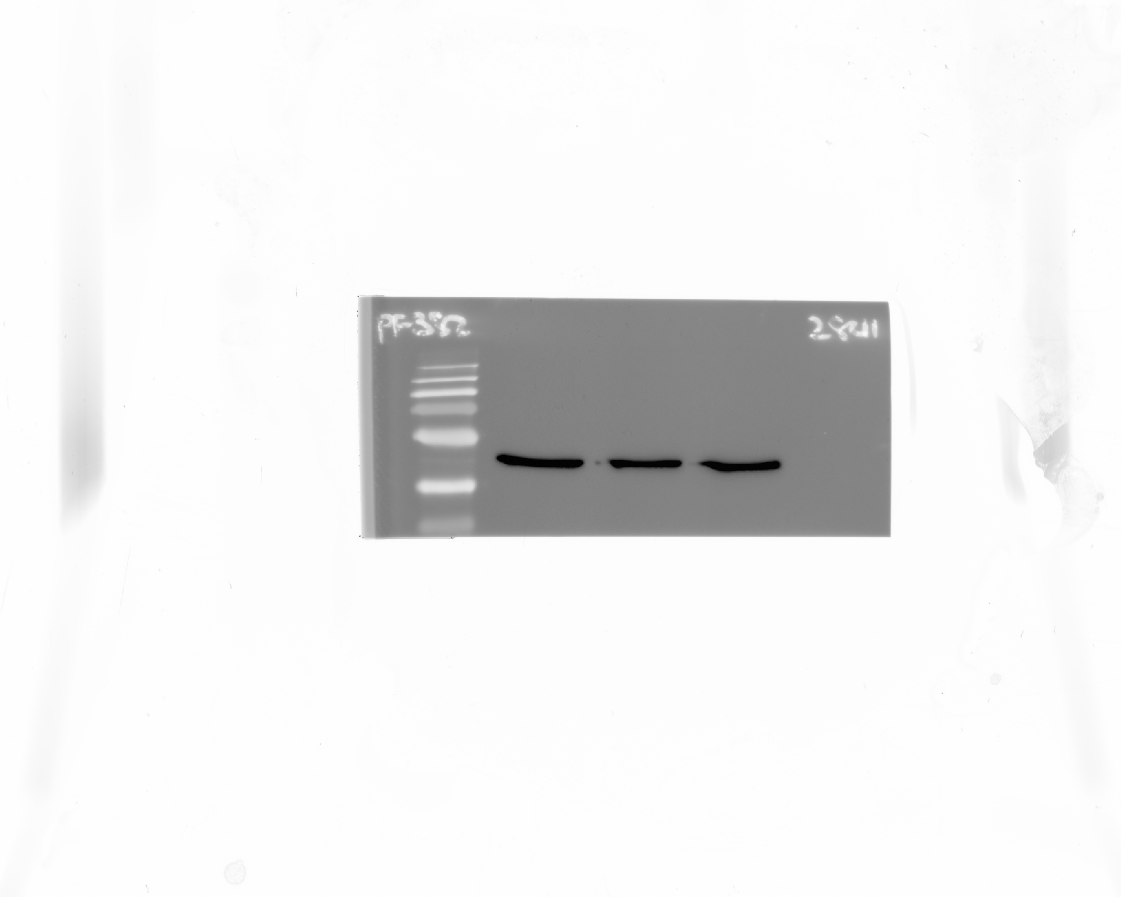

Supplement: Figure 4—source data 2. [file elife-106699-fig4-data2.zip › Figure 4ΓÇösource data 2 Original files for Western blot analysis displayed in Figure 4A./Actin PF-382 Abd-CRBN.tif]

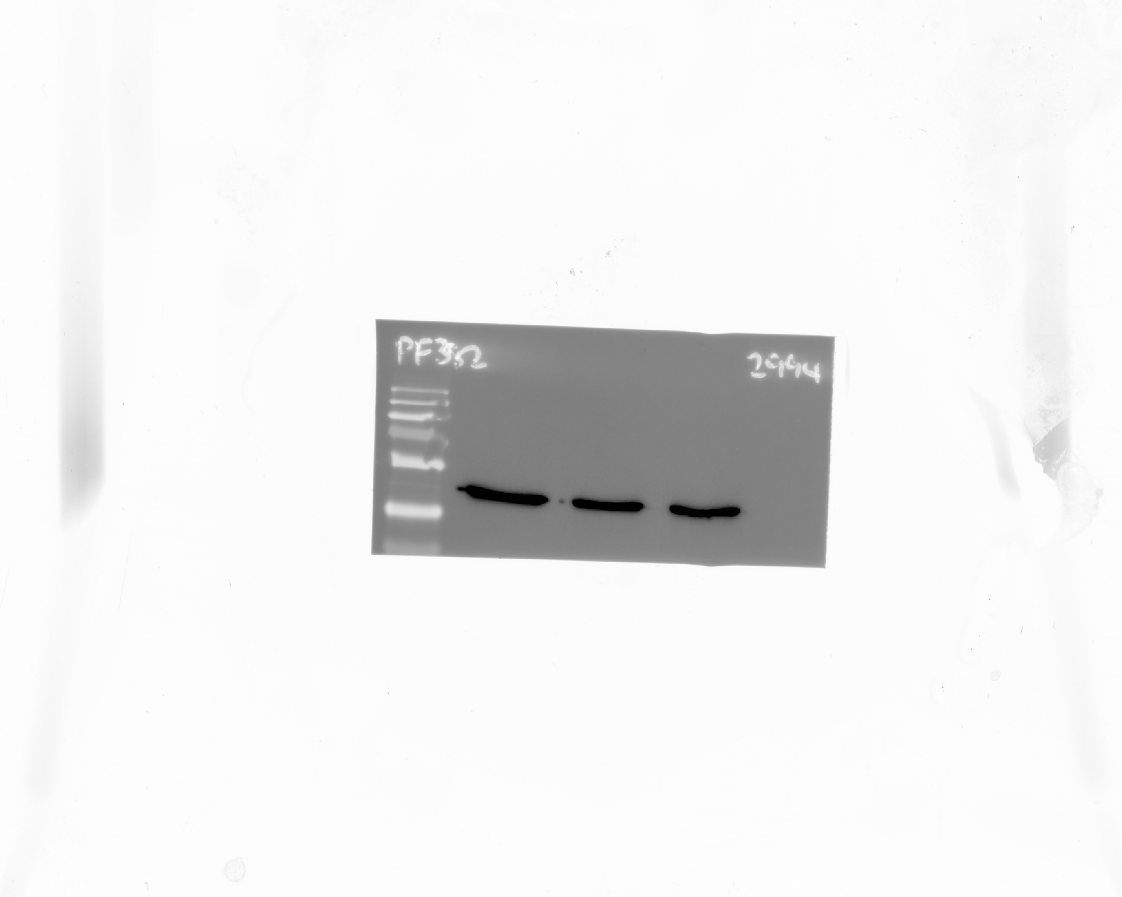

Supplement: Figure 4—source data 2. [file elife-106699-fig4-data2.zip › Figure 4ΓÇösource data 2 Original files for Western blot analysis displayed in Figure 4A./Actin PF-382 Abd-VHL.tif]

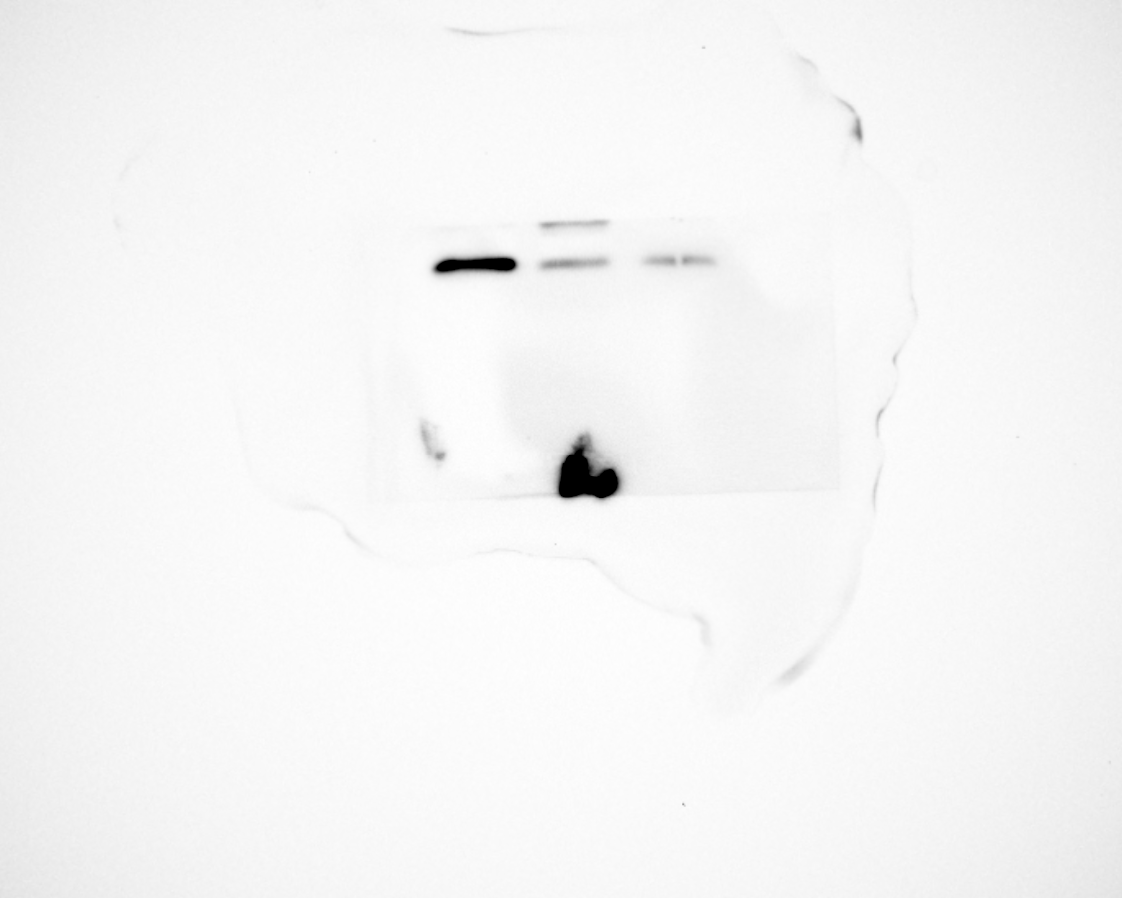

Supplement: Figure 4—source data 2. [file elife-106699-fig4-data2.zip › Figure 4ΓÇösource data 2 Original files for Western blot analysis displayed in Figure 4A./LMO2 CCRF-CEM Abd-CRBN.tif]

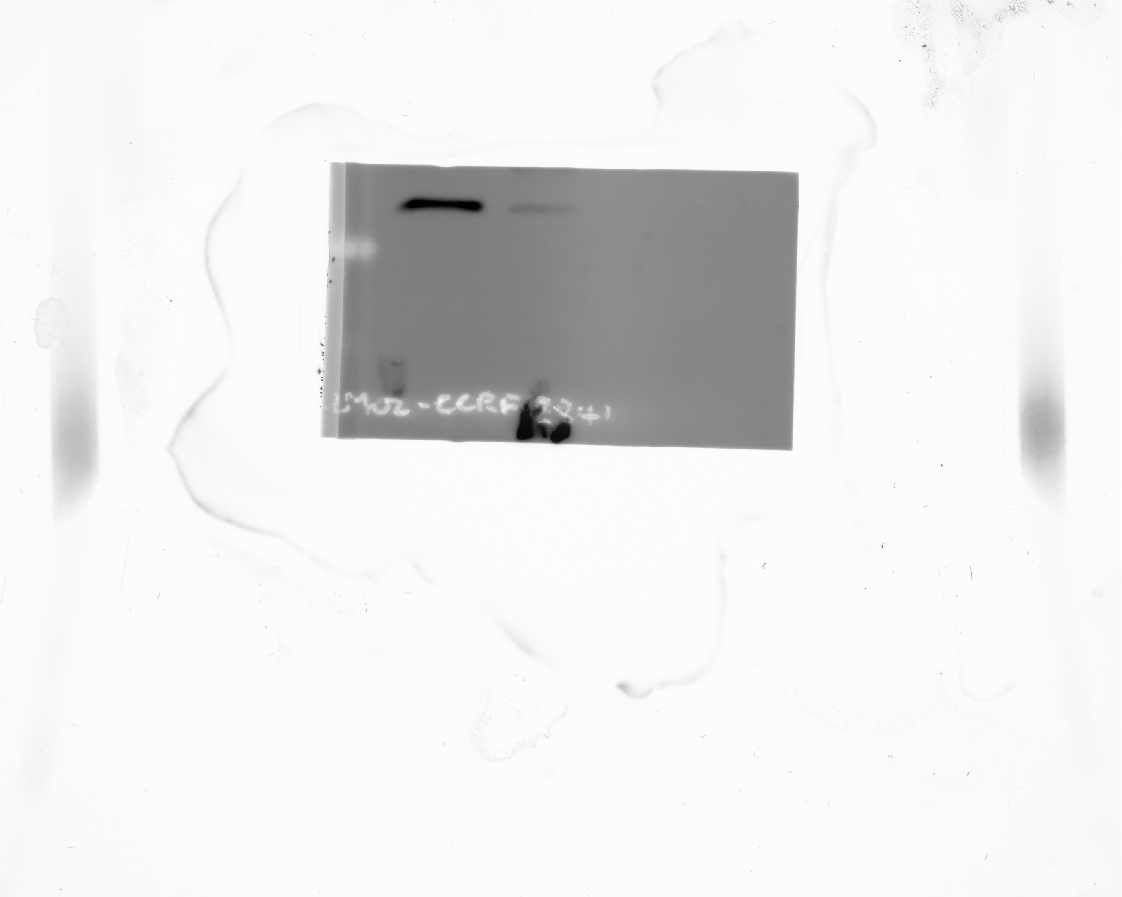

Supplement: Figure 4—source data 2. [file elife-106699-fig4-data2.zip › Figure 4ΓÇösource data 2 Original files for Western blot analysis displayed in Figure 4A./LMO2 CCRF-CEM Abd-VHL.tif]

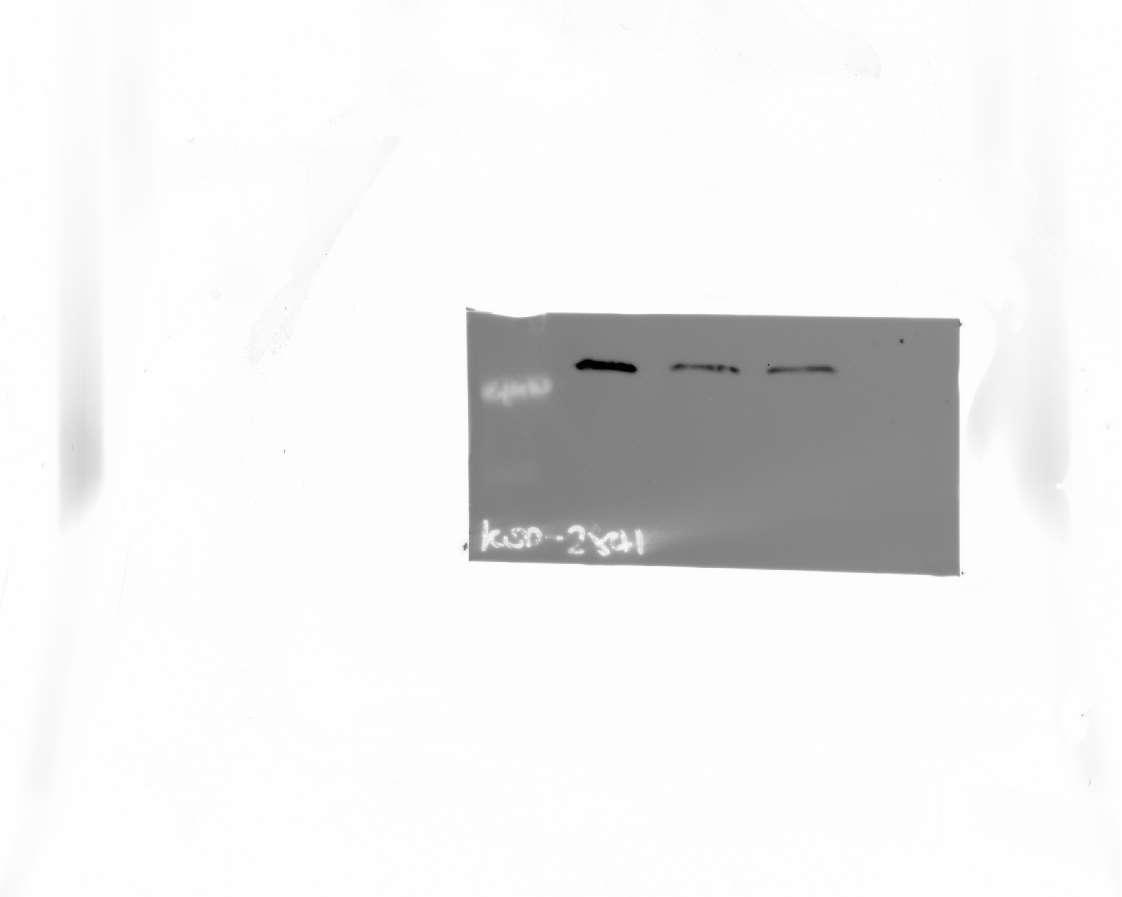

Supplement: Figure 4—source data 2. [file elife-106699-fig4-data2.zip › Figure 4ΓÇösource data 2 Original files for Western blot analysis displayed in Figure 4A./LMO2 KOPT-K1 Abd-CRBN.tif]

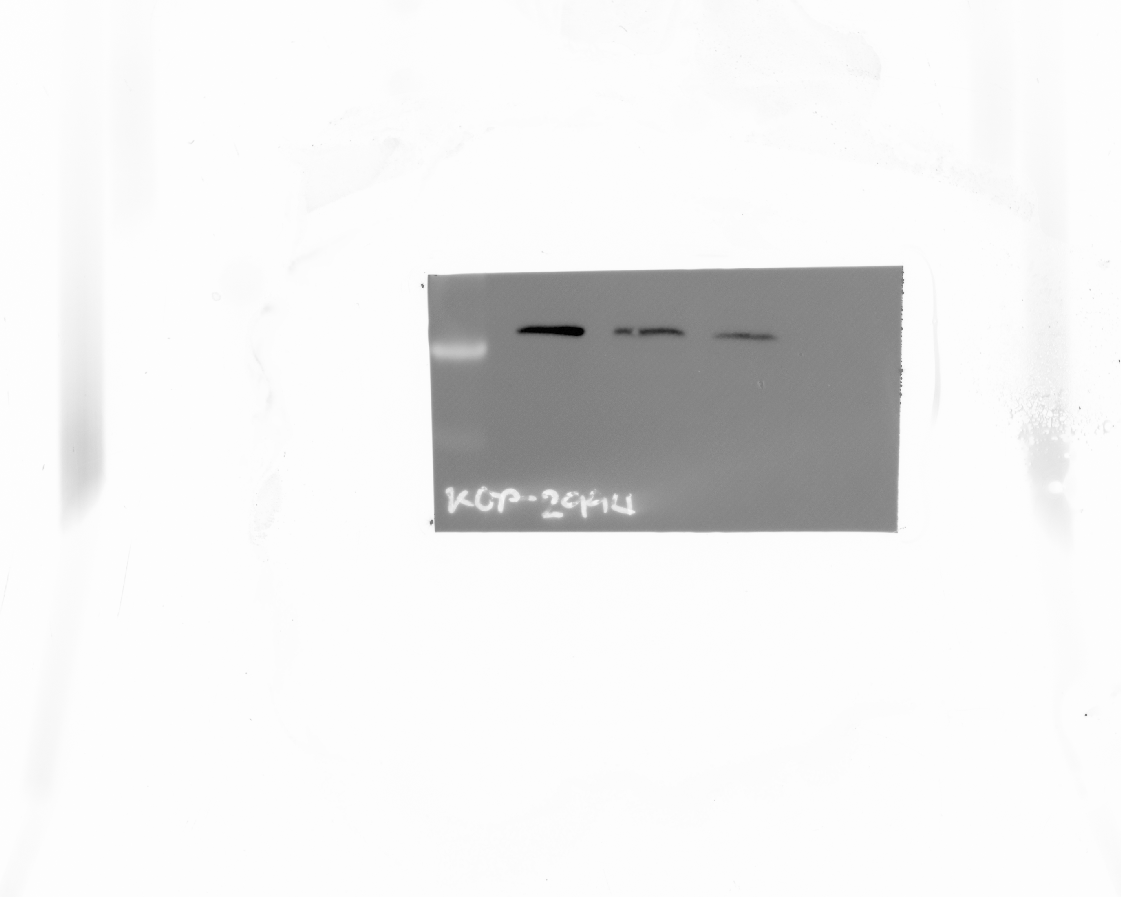

Supplement: Figure 4—source data 2. [file elife-106699-fig4-data2.zip › Figure 4ΓÇösource data 2 Original files for Western blot analysis displayed in Figure 4A./LMO2 KOPT-K1 Abd-VHL.tif]

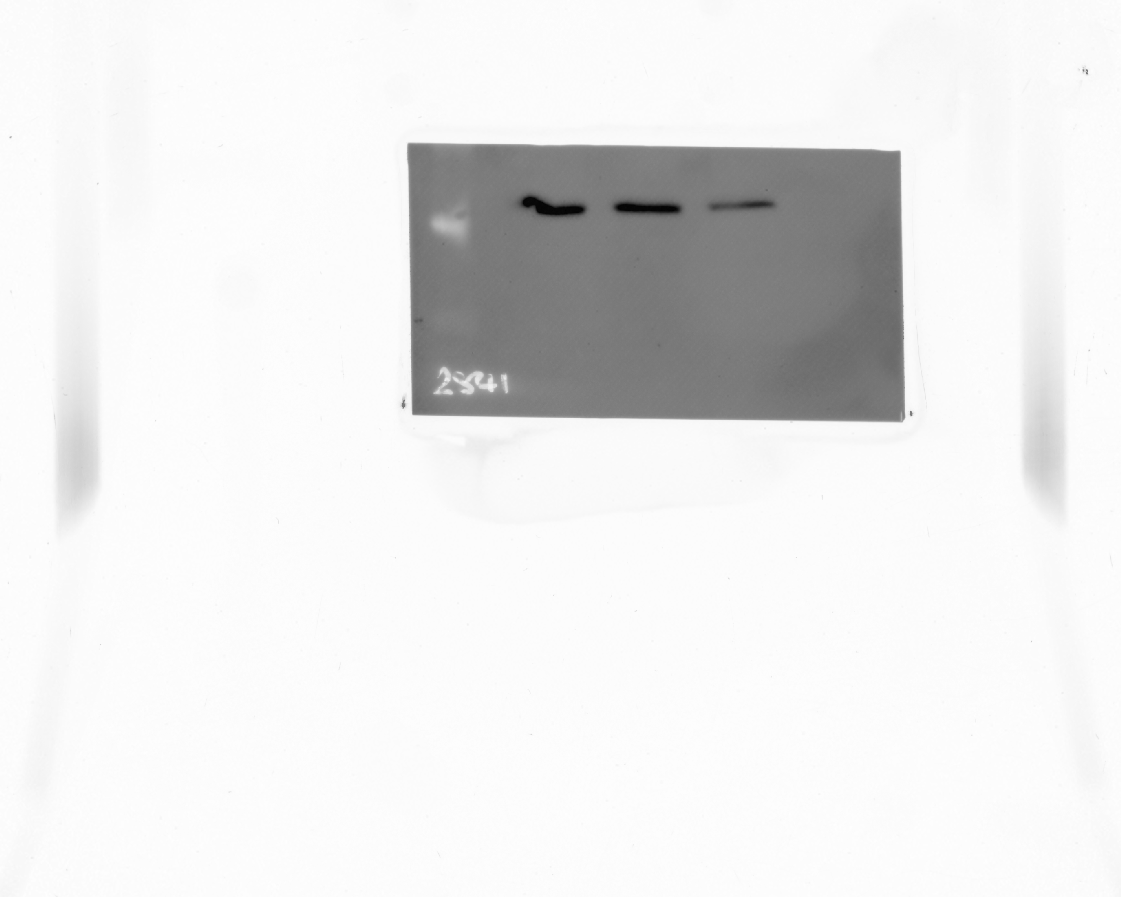

Supplement: Figure 4—source data 2. [file elife-106699-fig4-data2.zip › Figure 4ΓÇösource data 2 Original files for Western blot analysis displayed in Figure 4A./LMO2 LOUCY Abd-CRBN.tif]

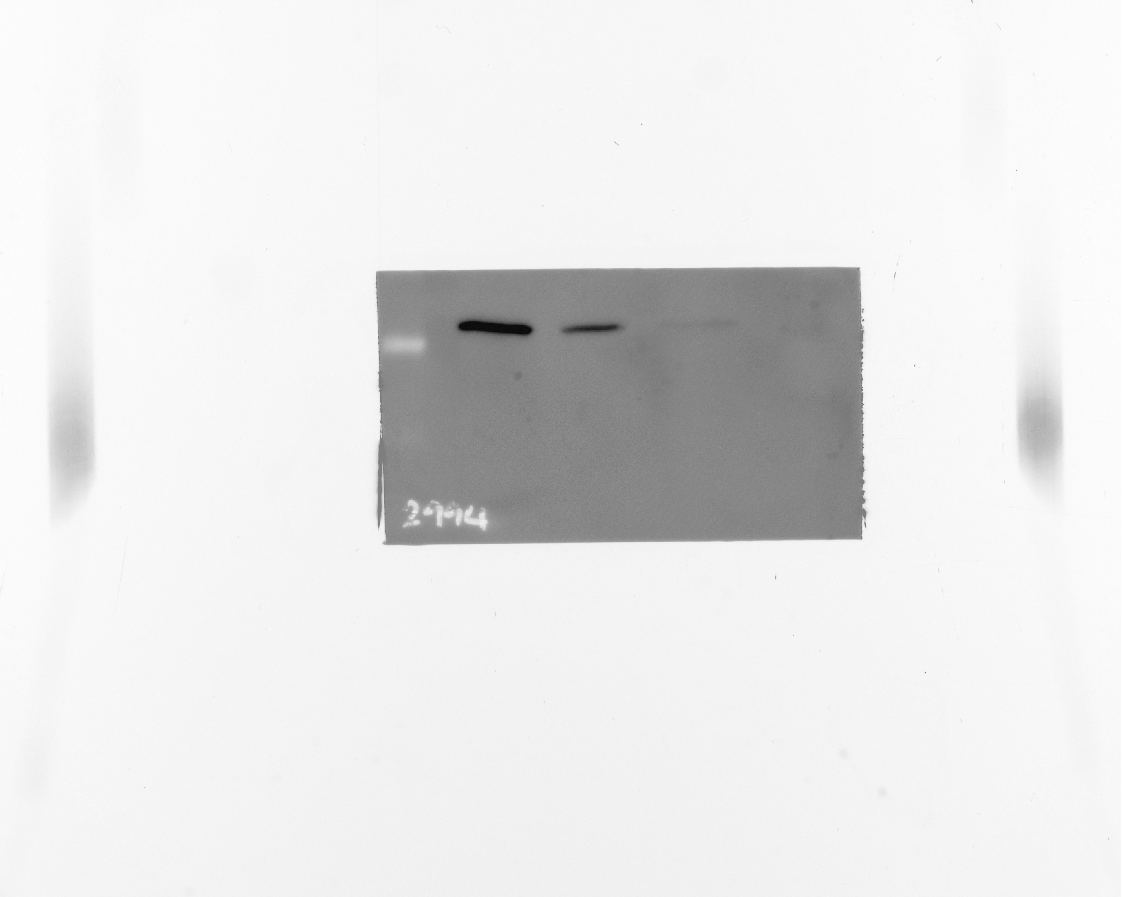

Supplement: Figure 4—source data 2. [file elife-106699-fig4-data2.zip › Figure 4ΓÇösource data 2 Original files for Western blot analysis displayed in Figure 4A./LMO2 LOUCY Abd-VHL.tif]

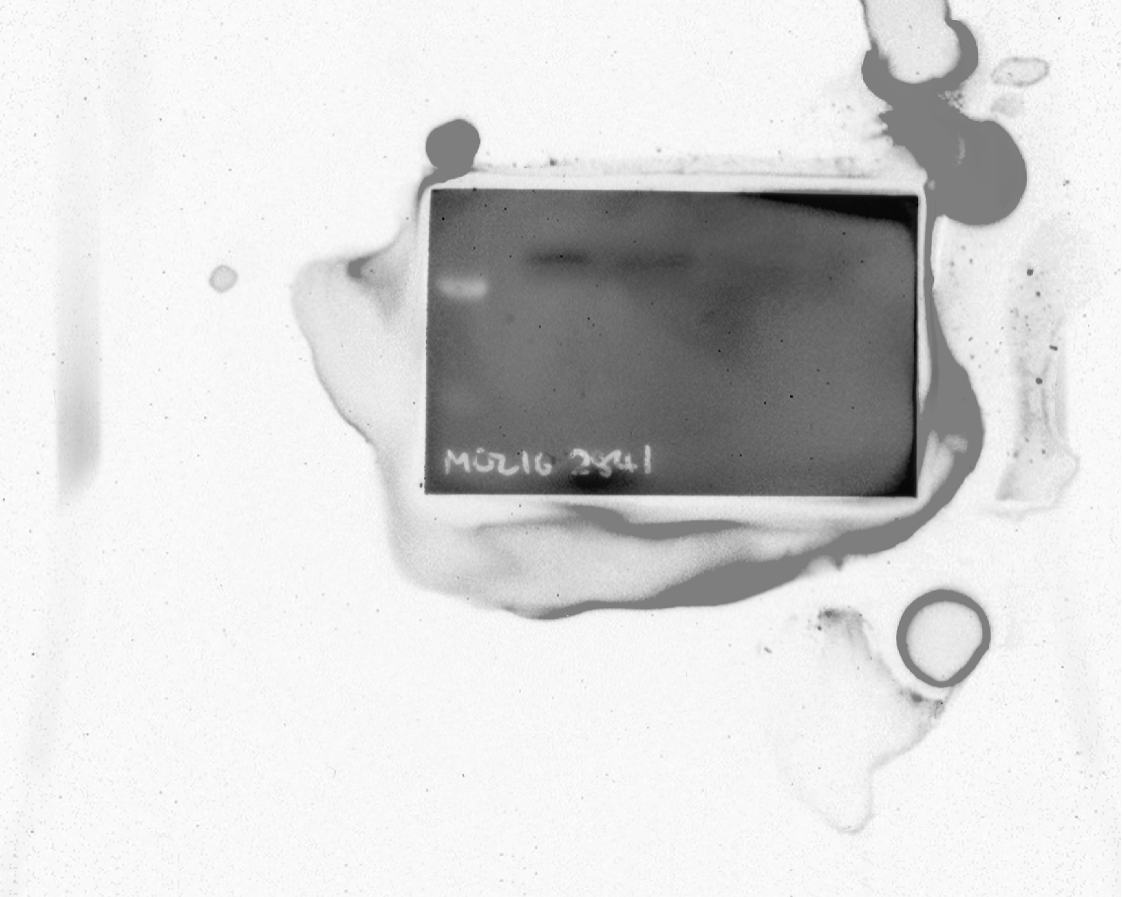

Supplement: Figure 4—source data 2. [file elife-106699-fig4-data2.zip › Figure 4ΓÇösource data 2 Original files for Western blot analysis displayed in Figure 4A./LMO2 MOLT-16 Abd-CRBN.tif]

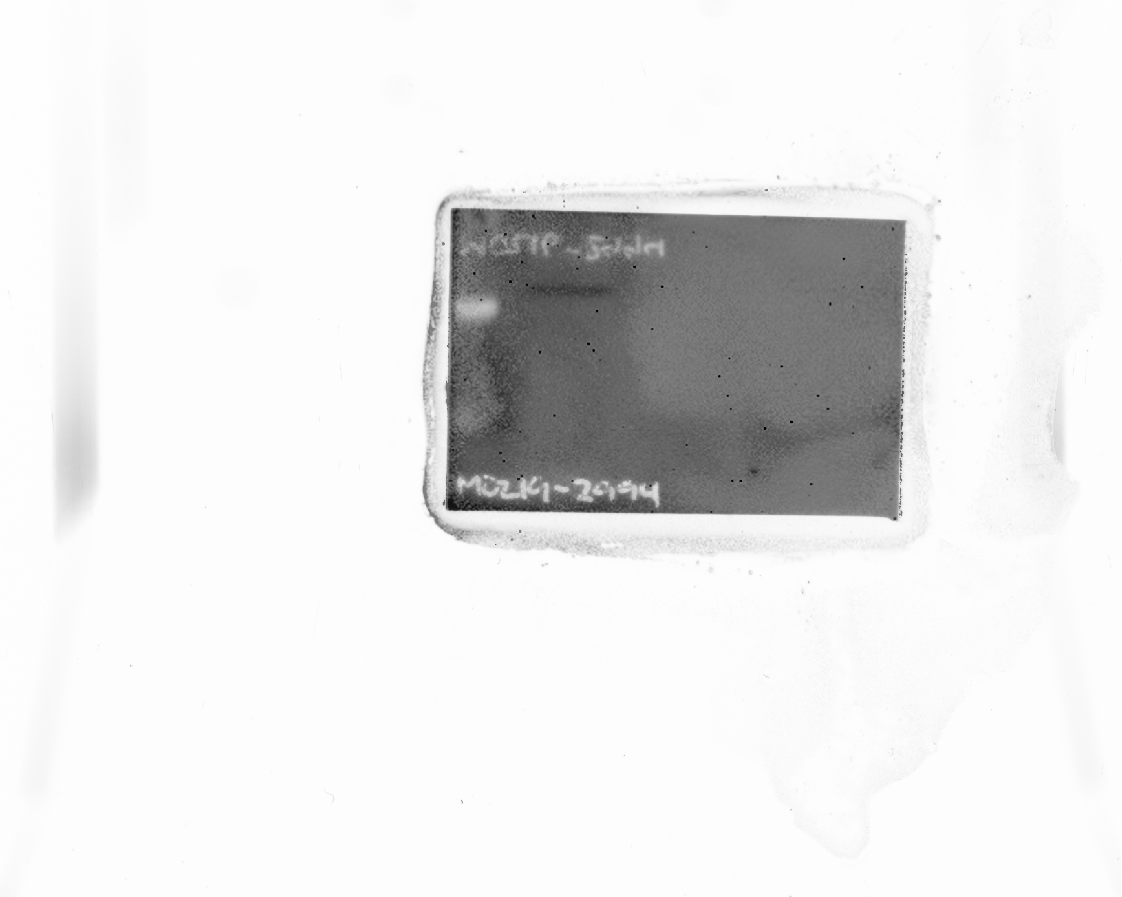

Supplement: Figure 4—source data 2. [file elife-106699-fig4-data2.zip › Figure 4ΓÇösource data 2 Original files for Western blot analysis displayed in Figure 4A./LMO2 MOLT-16 Abd-VHL.tif]

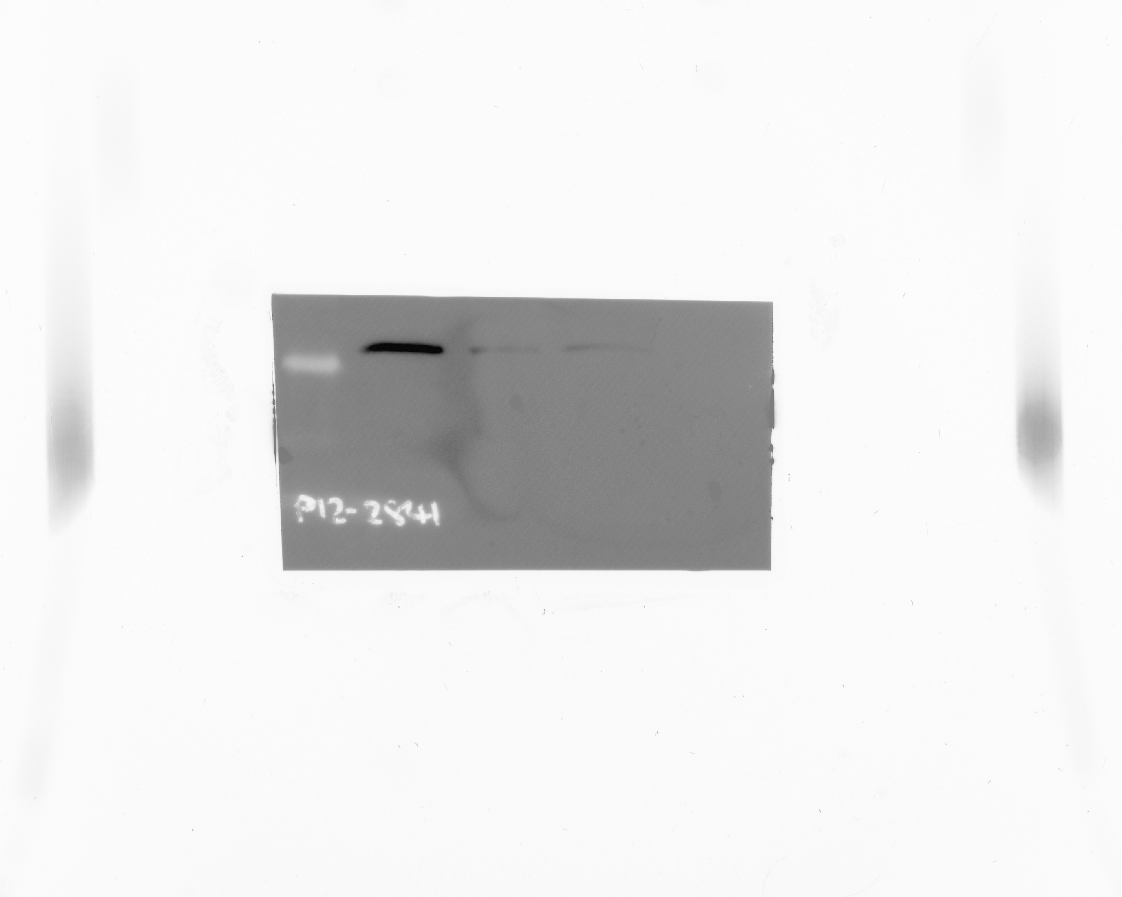

Supplement: Figure 4—source data 2. [file elife-106699-fig4-data2.zip › Figure 4ΓÇösource data 2 Original files for Western blot analysis displayed in Figure 4A./LMO2 P12-Ichikawa Abd-CRBN.tif]

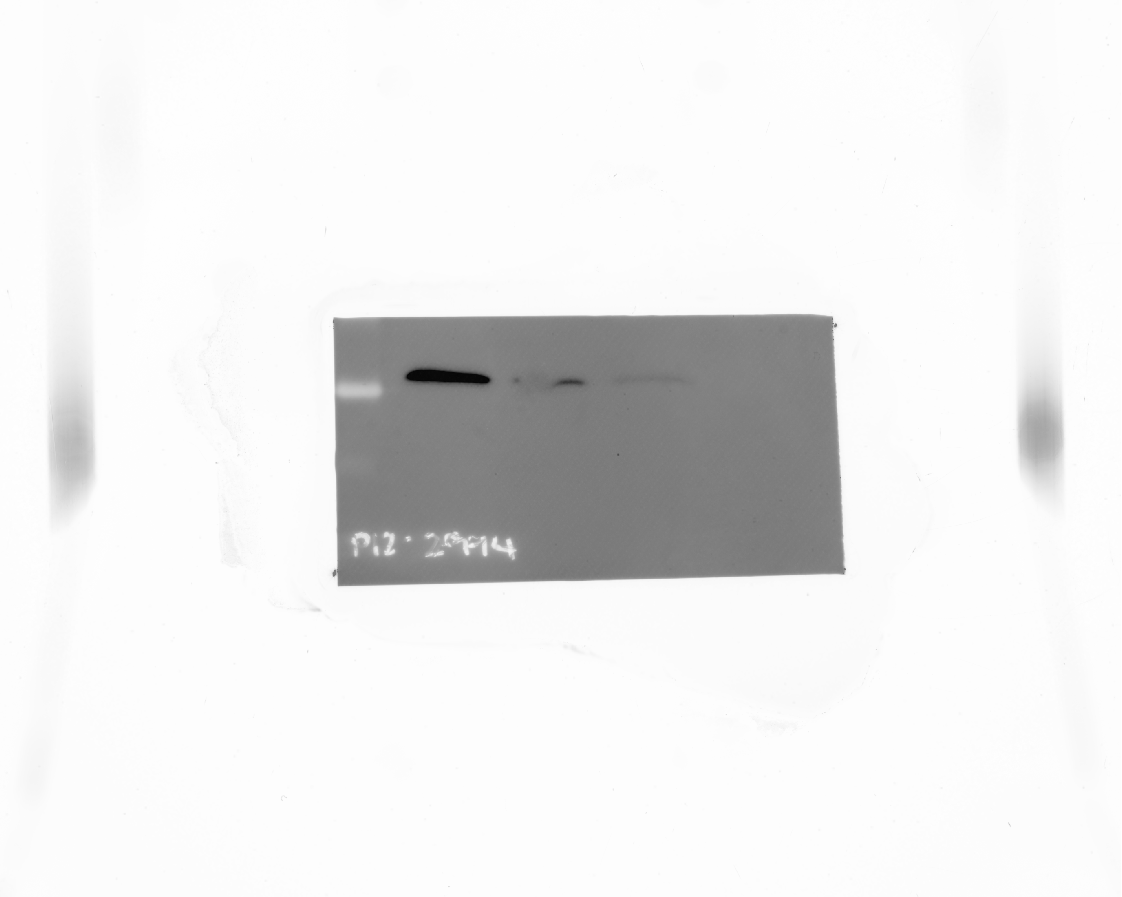

Supplement: Figure 4—source data 2. [file elife-106699-fig4-data2.zip › Figure 4ΓÇösource data 2 Original files for Western blot analysis displayed in Figure 4A./LMO2 P12-Ichikawa Abd-VHL.tif]

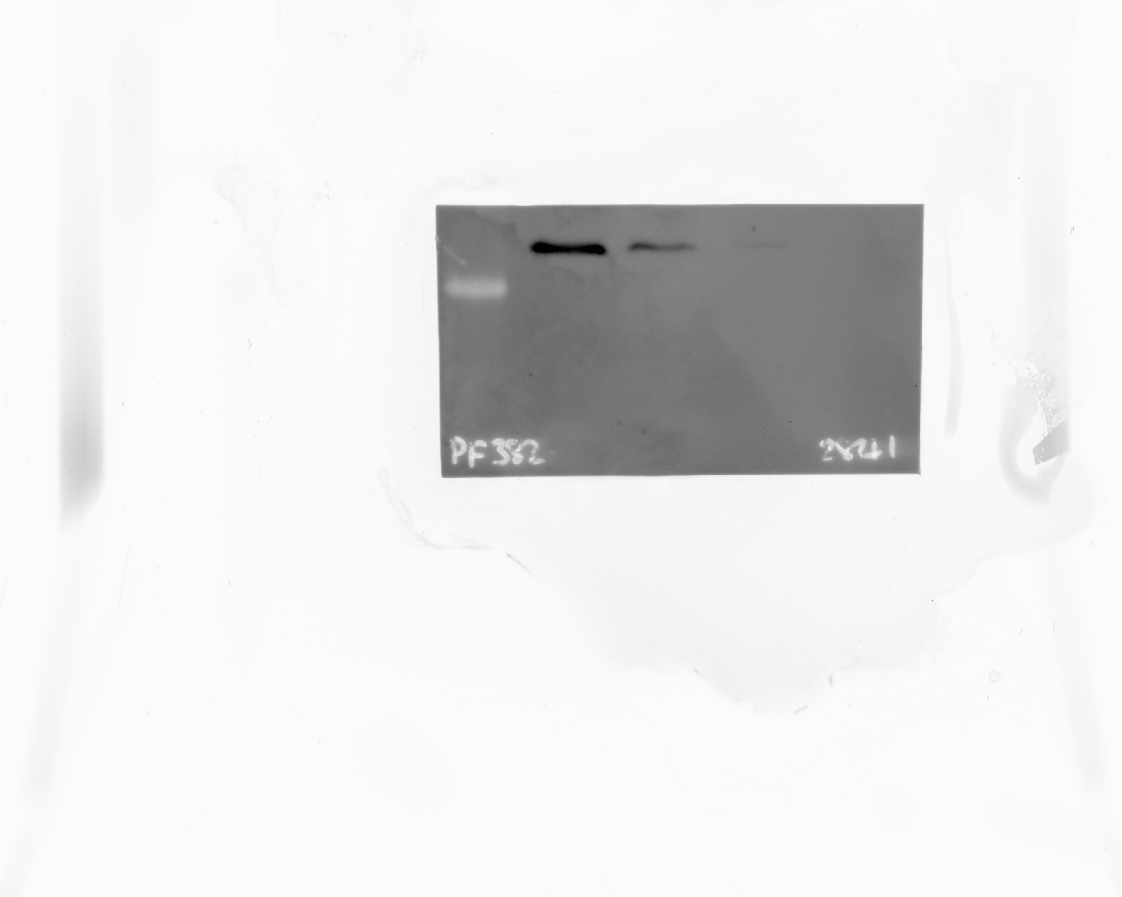

Supplement: Figure 4—source data 2. [file elife-106699-fig4-data2.zip › Figure 4ΓÇösource data 2 Original files for Western blot analysis displayed in Figure 4A./LMO2 PF-382 Abd-CRBN.tif]

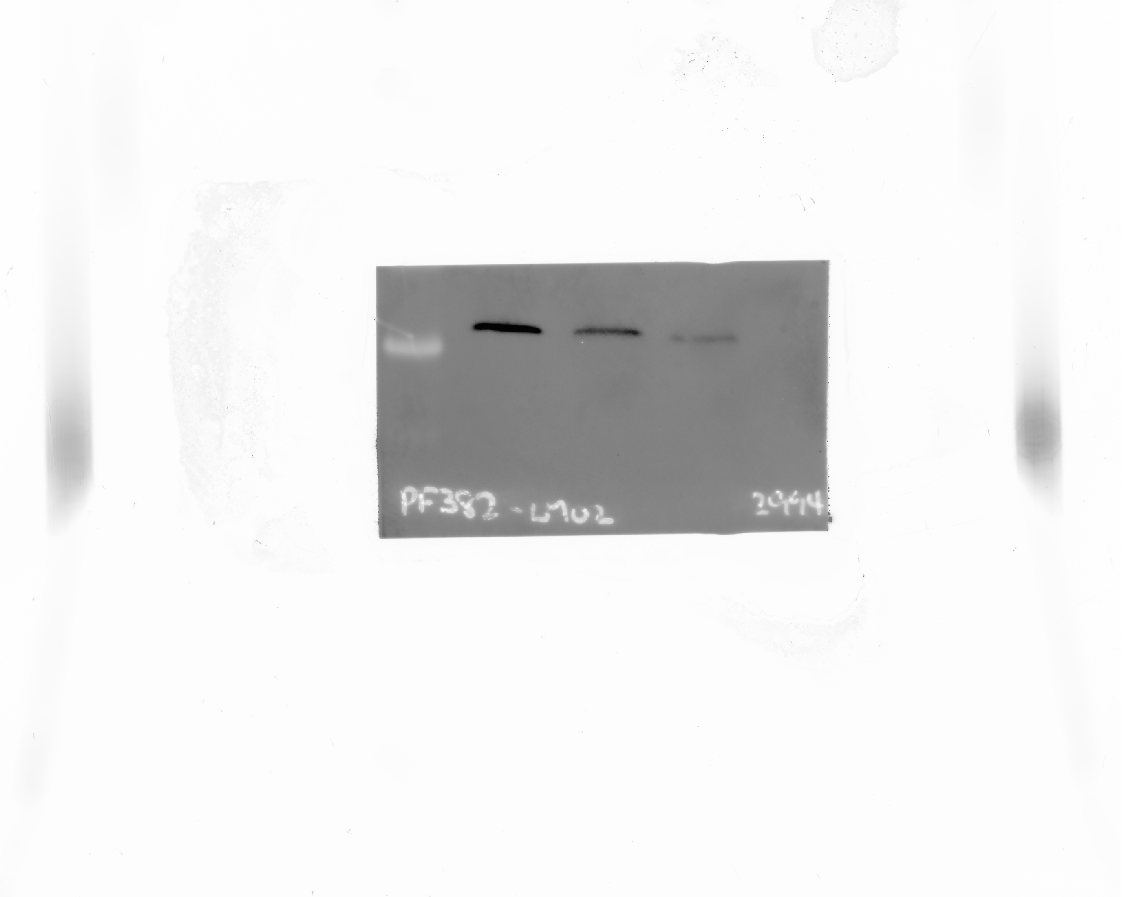

Supplement: Figure 4—source data 2. [file elife-106699-fig4-data2.zip › Figure 4ΓÇösource data 2 Original files for Western blot analysis displayed in Figure 4A./LMO2 PF-382 Abd-VHL.tif]

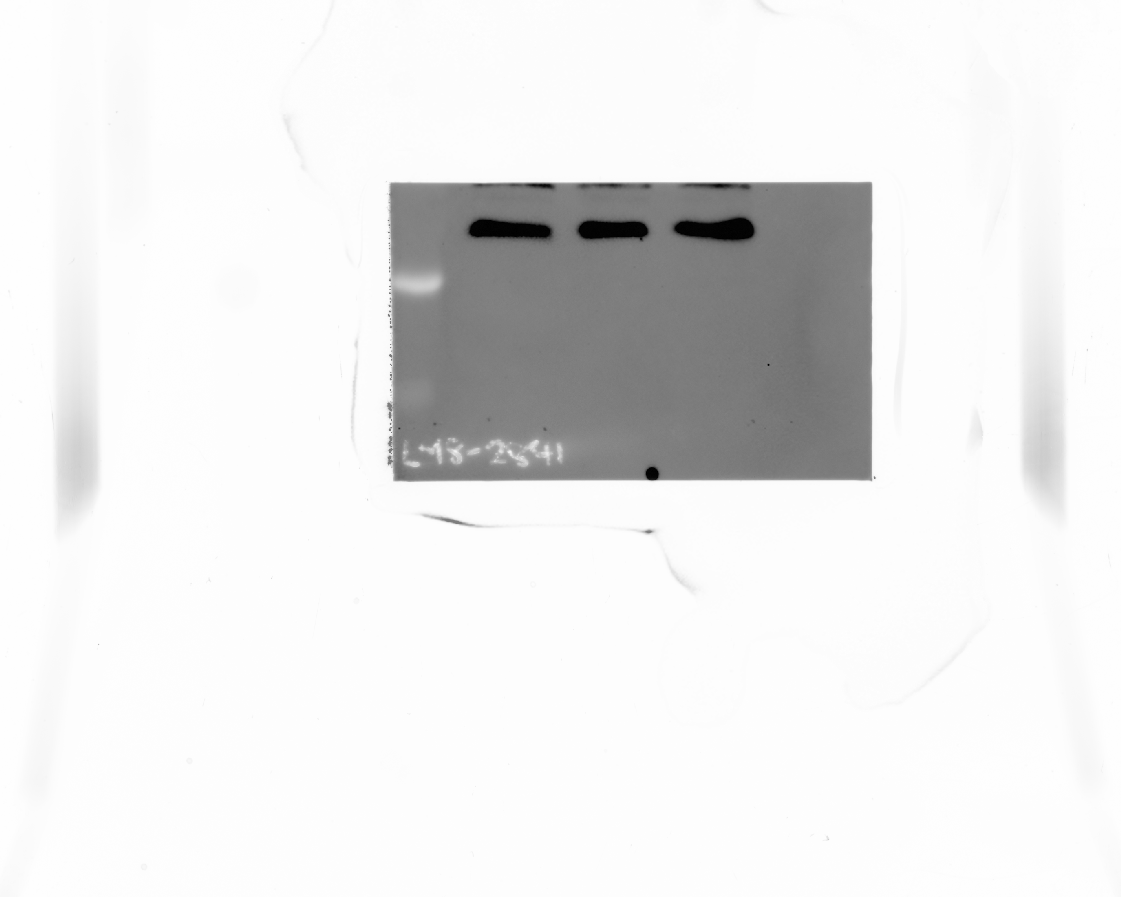

Supplement: Figure 4—source data 2. [file elife-106699-fig4-data2.zip › Figure 4ΓÇösource data 2 Original files for Western blot analysis displayed in Figure 4A./RAS CCRF-CEM Abd-CRBN.tif]

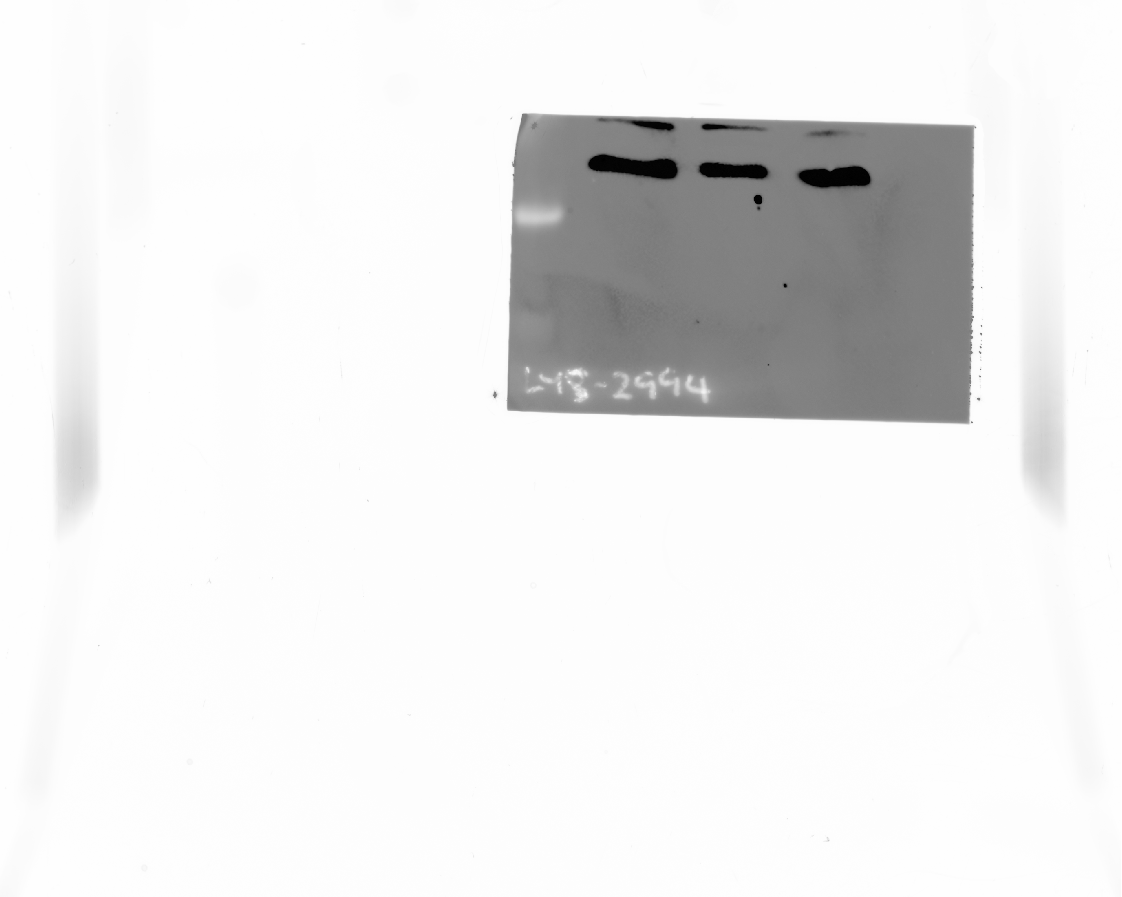

Supplement: Figure 4—source data 2. [file elife-106699-fig4-data2.zip › Figure 4ΓÇösource data 2 Original files for Western blot analysis displayed in Figure 4A./RAS CCRF-CRM Abd-VHL.tif]

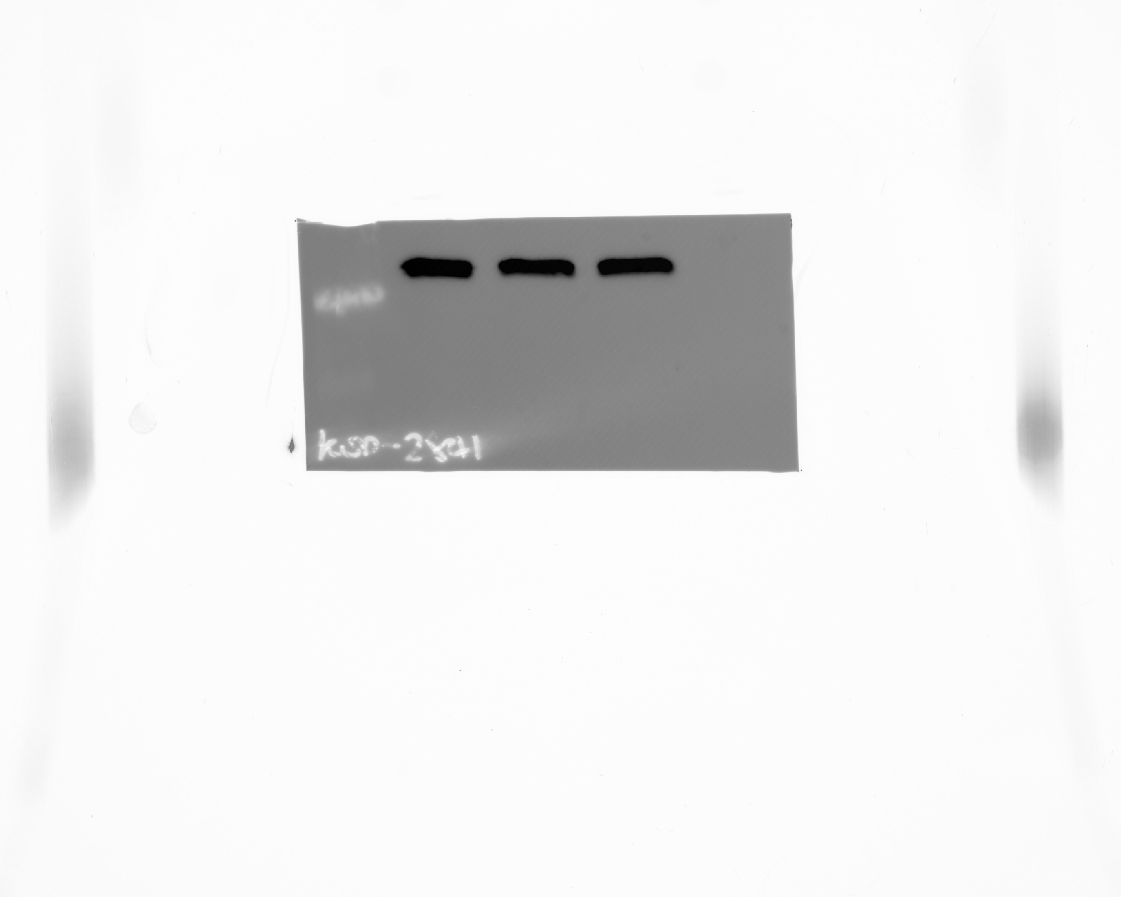

Supplement: Figure 4—source data 2. [file elife-106699-fig4-data2.zip › Figure 4ΓÇösource data 2 Original files for Western blot analysis displayed in Figure 4A./RAS KOPT-K1 Abd-CRBN.tif]

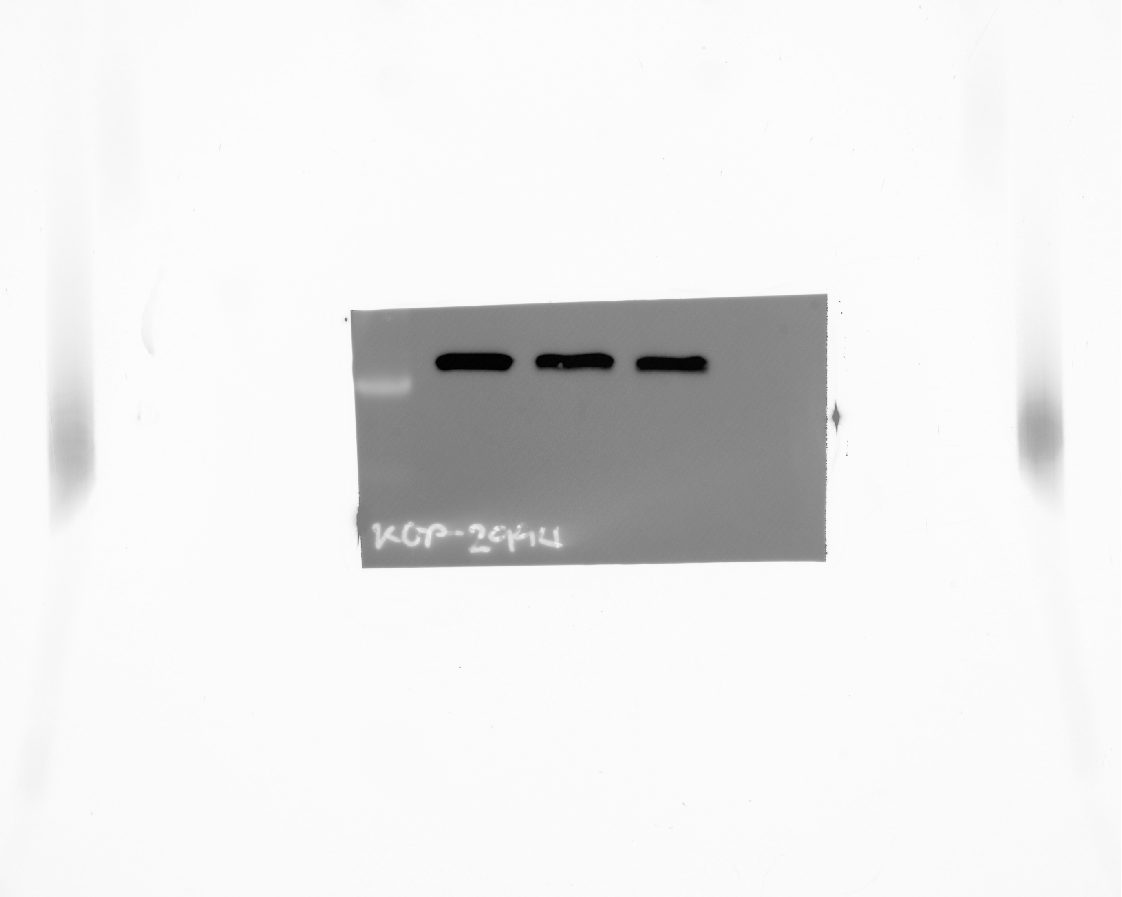

Supplement: Figure 4—source data 2. [file elife-106699-fig4-data2.zip › Figure 4ΓÇösource data 2 Original files for Western blot analysis displayed in Figure 4A./RAS KOPT-K1 Abd-VHL.tif]

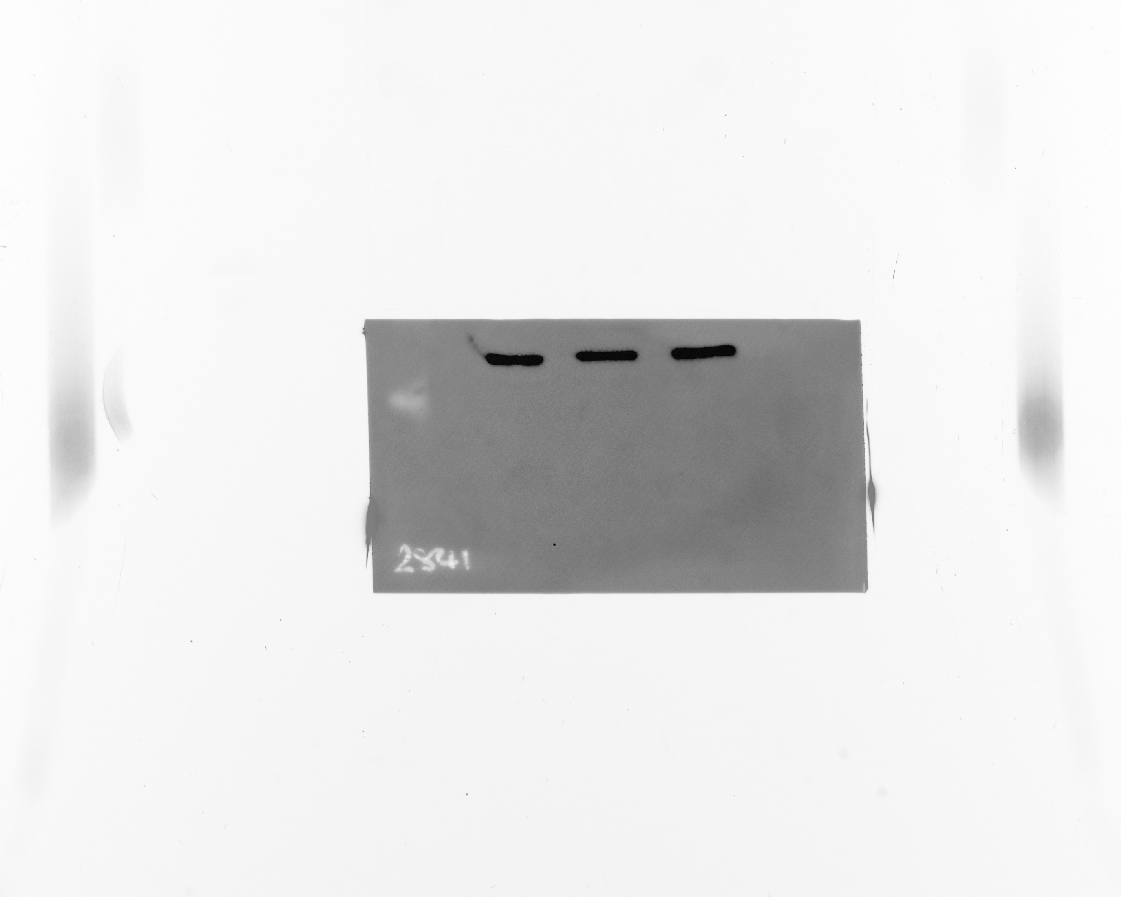

Supplement: Figure 4—source data 2. [file elife-106699-fig4-data2.zip › Figure 4ΓÇösource data 2 Original files for Western blot analysis displayed in Figure 4A./RAS LOUCY Abd-CRBN.tif]

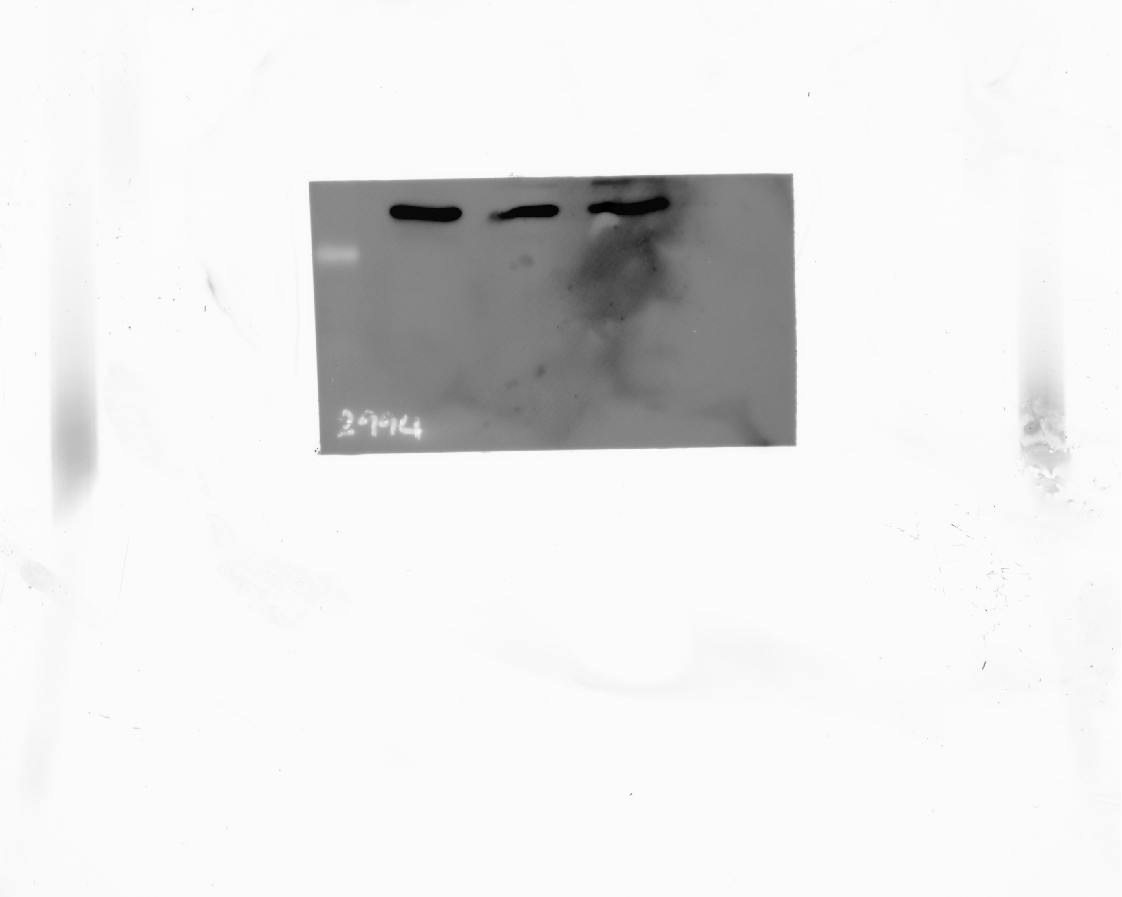

Supplement: Figure 4—source data 2. [file elife-106699-fig4-data2.zip › Figure 4ΓÇösource data 2 Original files for Western blot analysis displayed in Figure 4A./RAS LOUCY Abd-VHL.tif]

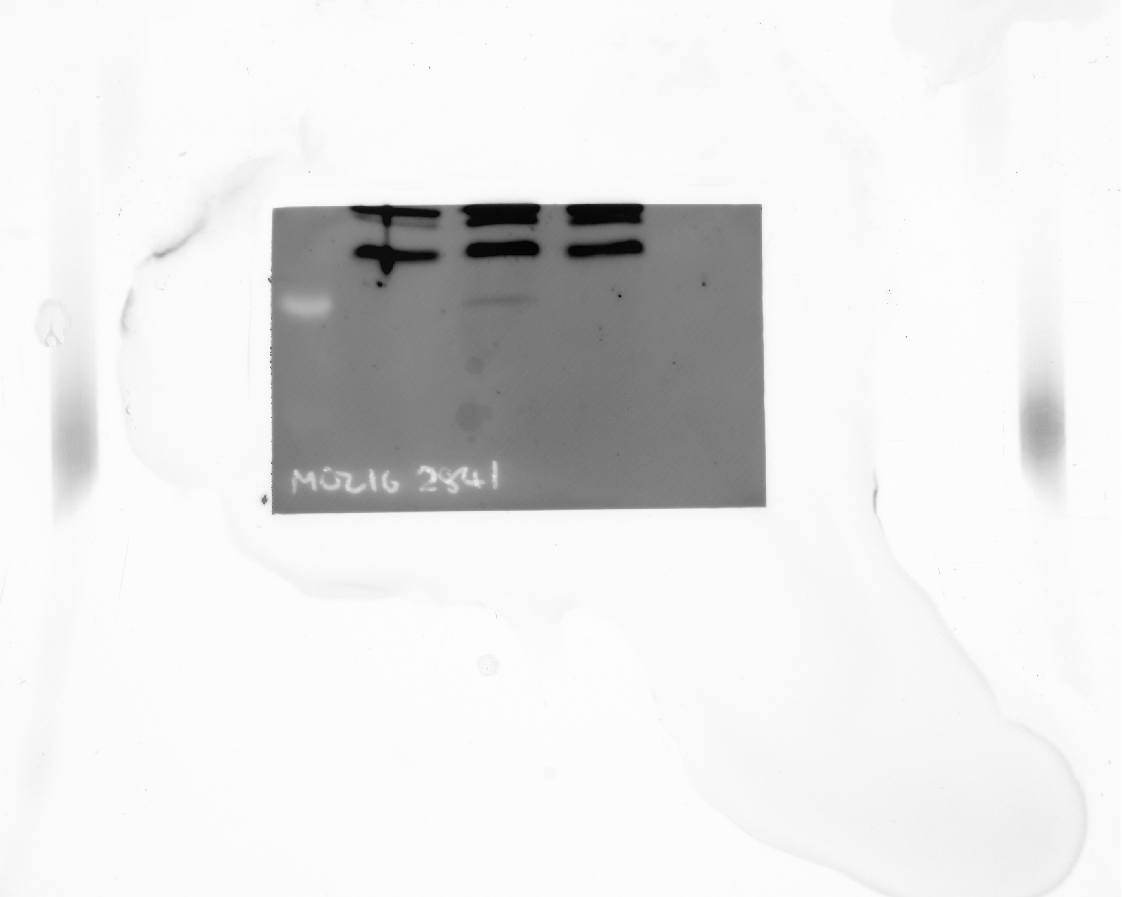

Supplement: Figure 4—source data 2. [file elife-106699-fig4-data2.zip › Figure 4ΓÇösource data 2 Original files for Western blot analysis displayed in Figure 4A./RAS MOLT-16 Abd-CRBN.tif]

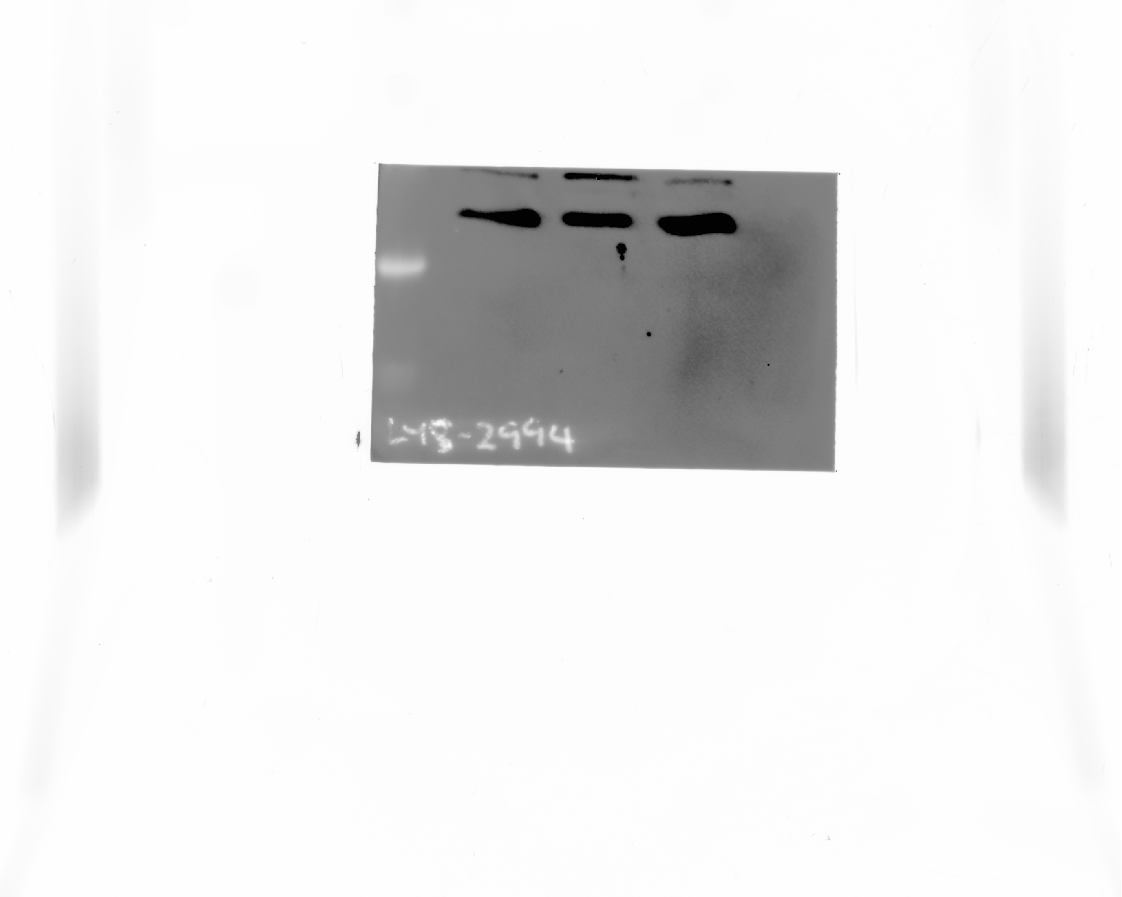

Supplement: Figure 4—source data 2. [file elife-106699-fig4-data2.zip › Figure 4ΓÇösource data 2 Original files for Western blot analysis displayed in Figure 4A./RAS MOLT-16 Abd-VHL.tif]

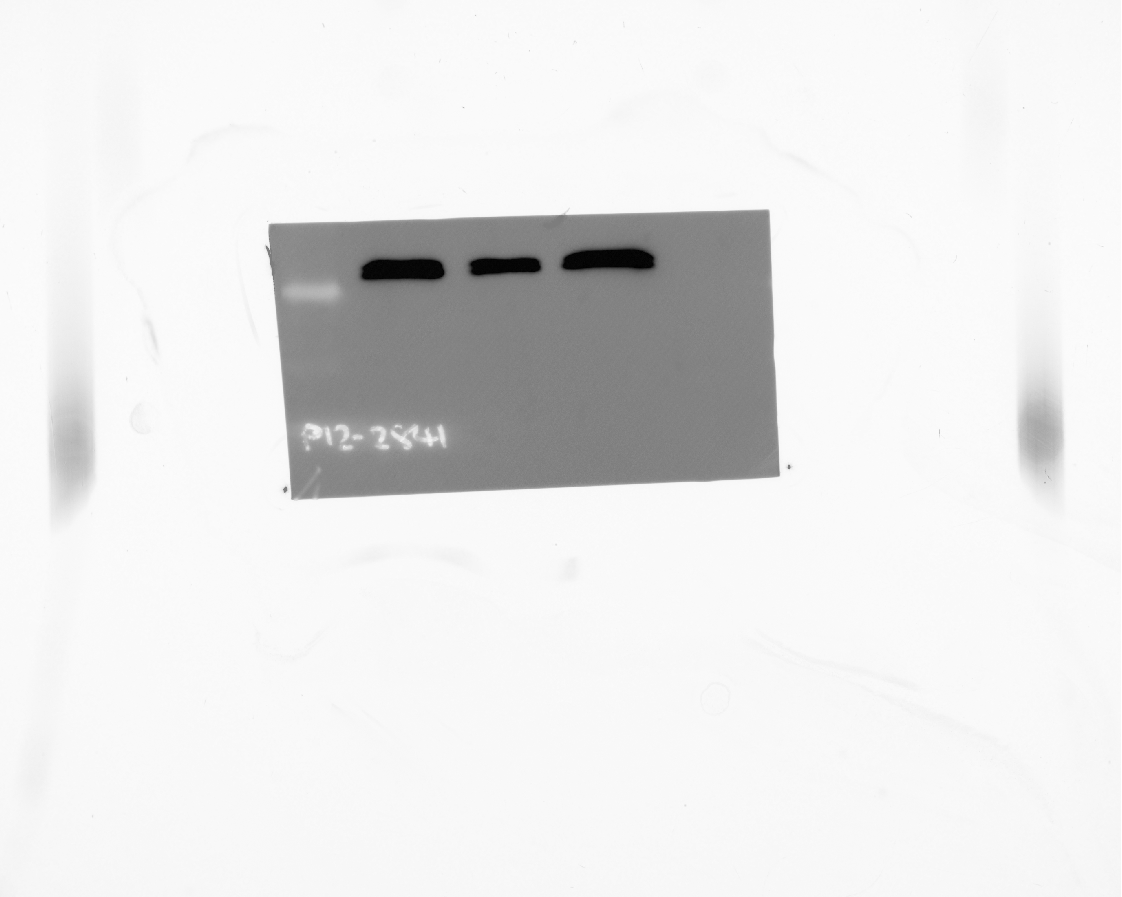

Supplement: Figure 4—source data 2. [file elife-106699-fig4-data2.zip › Figure 4ΓÇösource data 2 Original files for Western blot analysis displayed in Figure 4A./RAS P12-Ichikawa Abd-CRBN.tif]

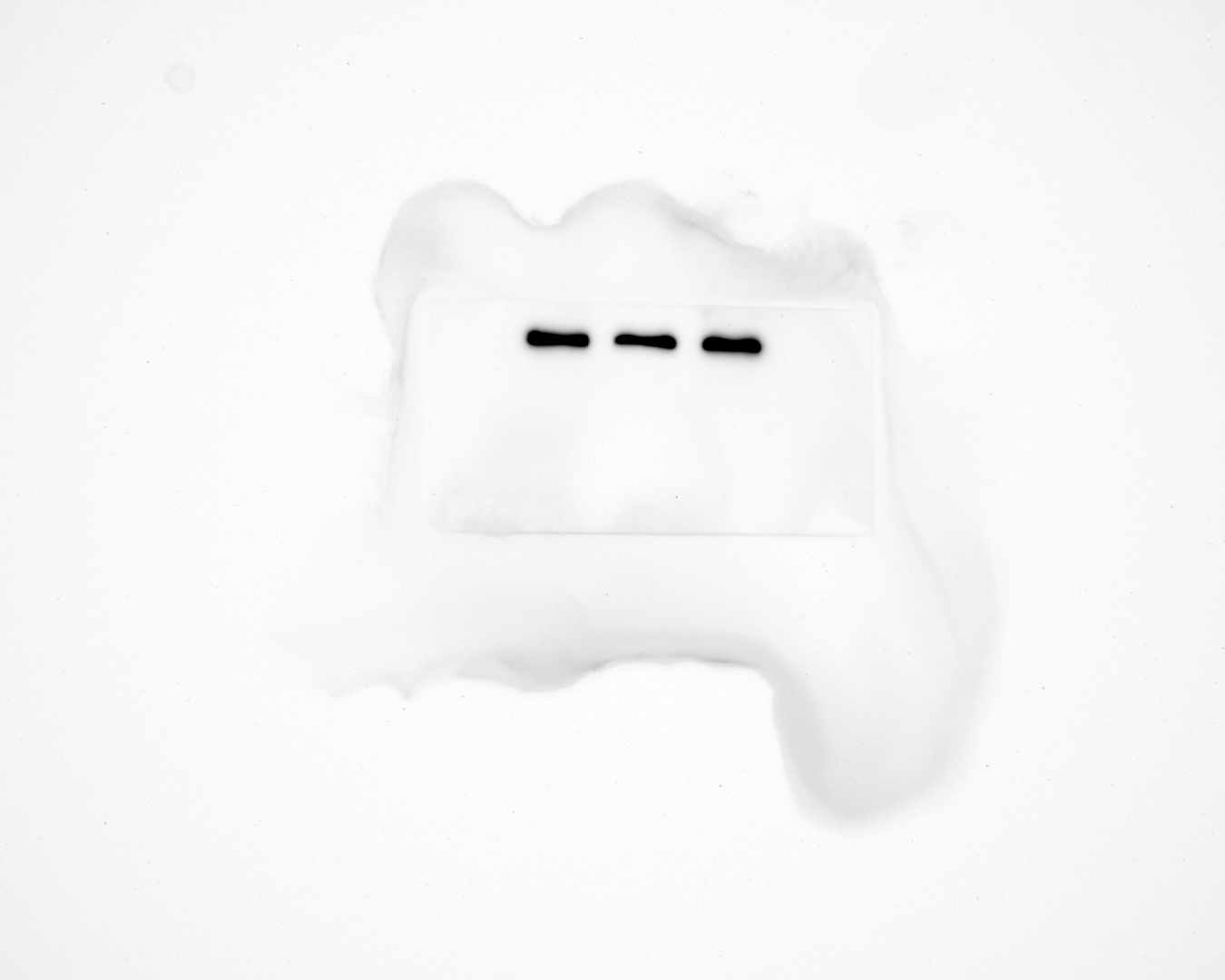

Supplement: Figure 4—source data 2. [file elife-106699-fig4-data2.zip › Figure 4ΓÇösource data 2 Original files for Western blot analysis displayed in Figure 4A./RAS P12-Ichikawa Abd-VHL.tif]

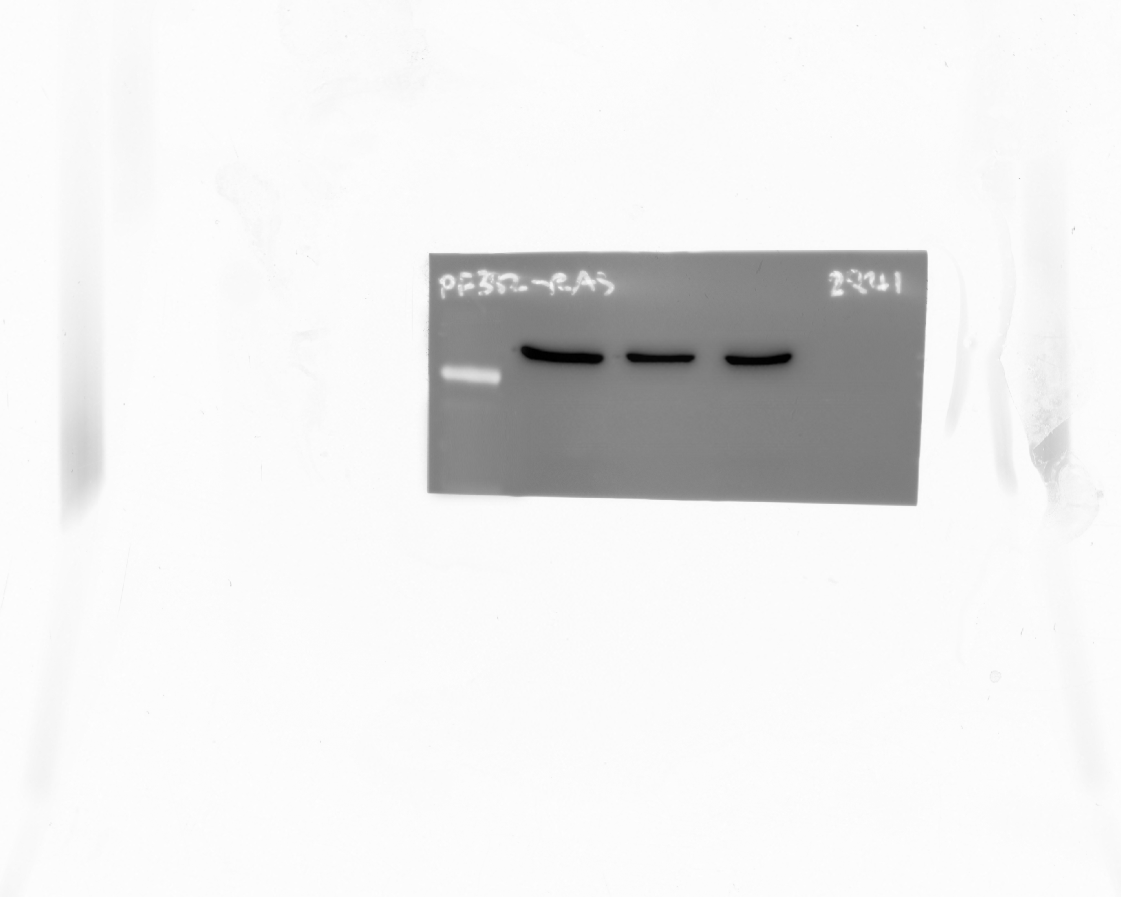

Supplement: Figure 4—source data 2. [file elife-106699-fig4-data2.zip › Figure 4ΓÇösource data 2 Original files for Western blot analysis displayed in Figure 4A./RAS PF-382 Abd-CRBN.tif]

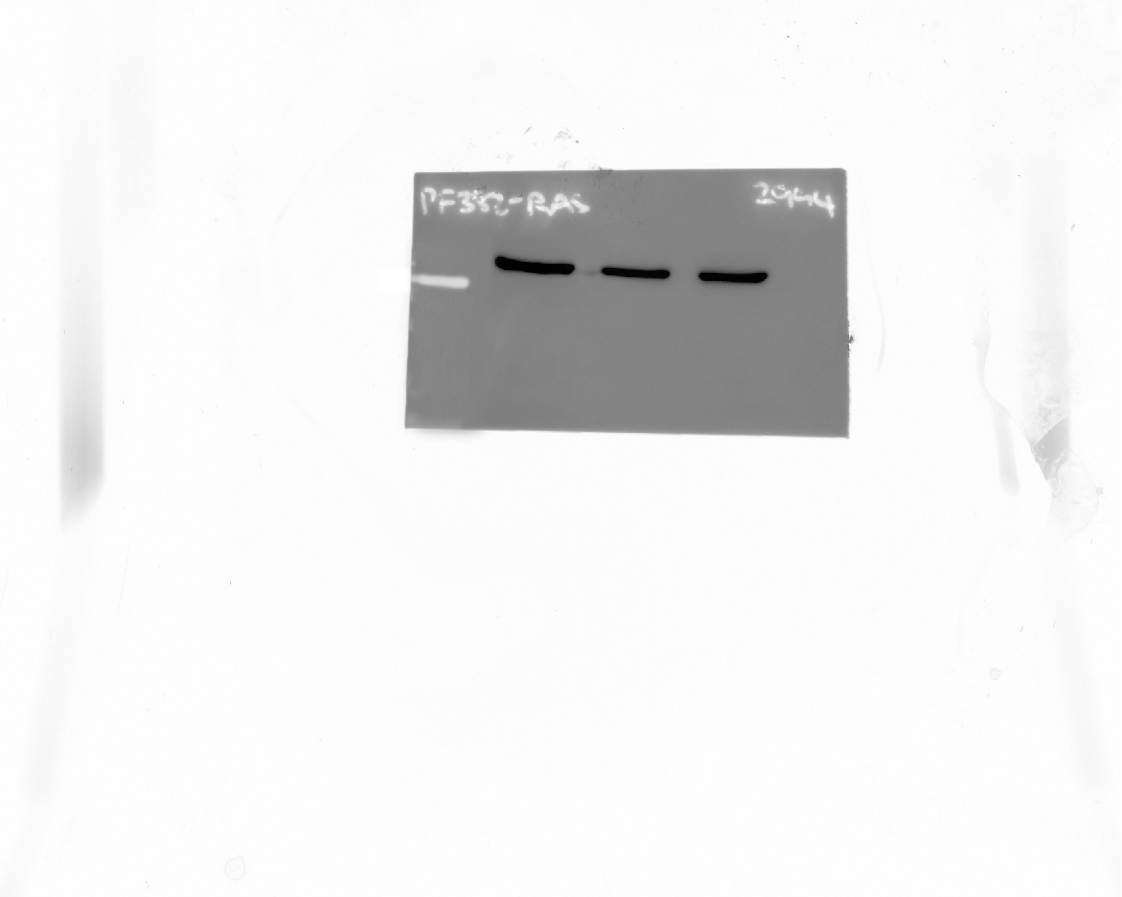

Supplement: Figure 4—source data 2. [file elife-106699-fig4-data2.zip › Figure 4ΓÇösource data 2 Original files for Western blot analysis displayed in Figure 4A./RAS PF-382 Abd-VHL.tif]

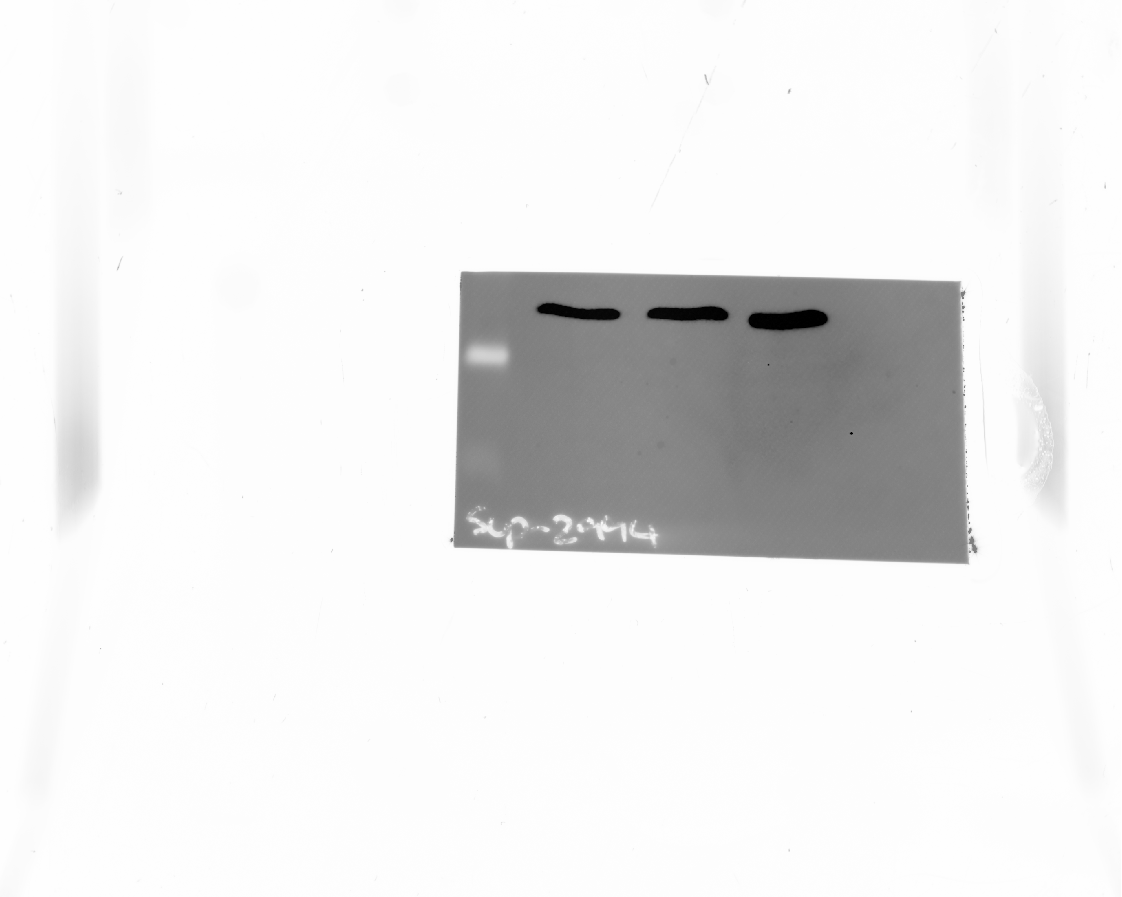

Supplement: Figure 4—figure supplement 1—source data 1. [file elife-106699-fig4-figsupp1-data1.zip › Figure 4ΓÇöfigure supplement 1-source data 2 Western blot raw datashows the verification of LMO2 non-expressing T-cells./pan-RAS SUPT-1 Abd-VHL(Composite).tif]

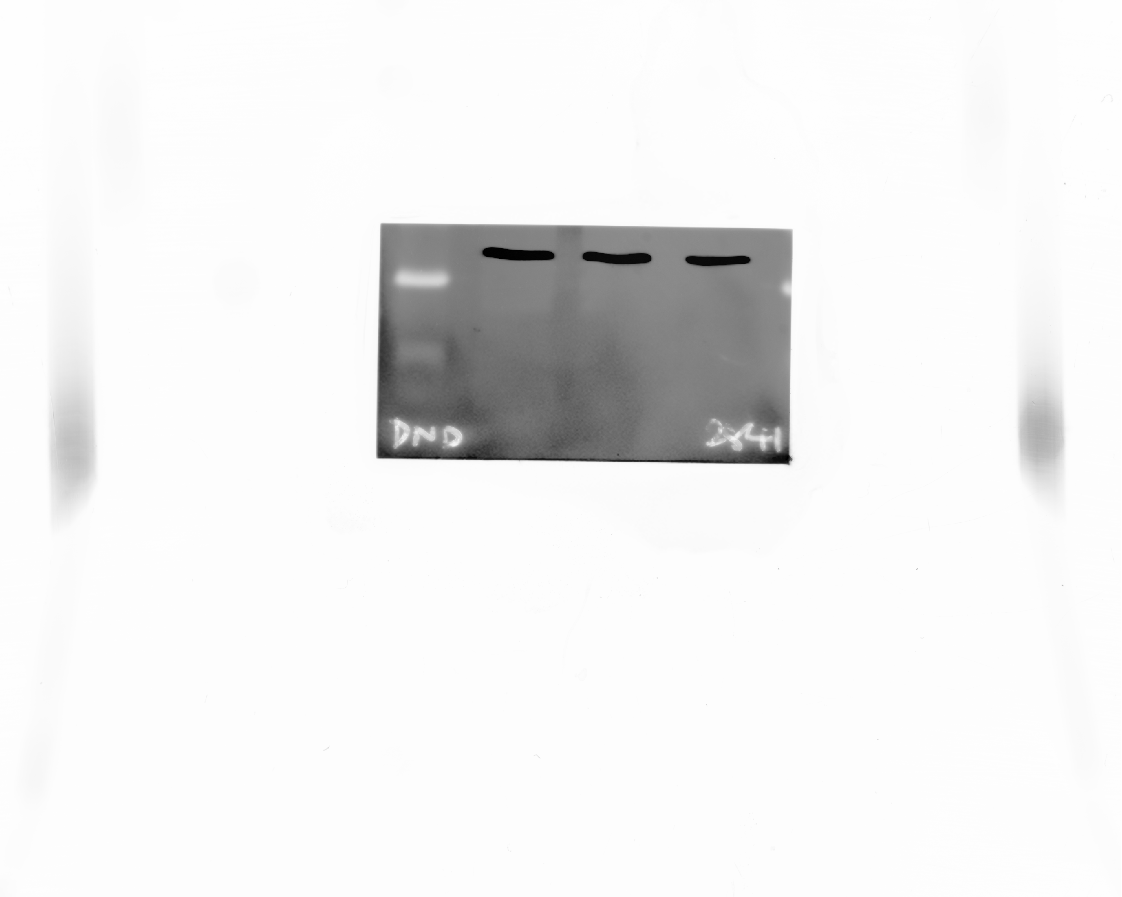

Supplement: Figure 4—figure supplement 1—source data 1. [file elife-106699-fig4-figsupp1-data1.zip › Figure 4ΓÇöfigure supplement 1-source data 2 Western blot raw datashows the verification of LMO2 non-expressing T-cells./pan-RAS DND41 Abd-CRBN (Composite).tif]

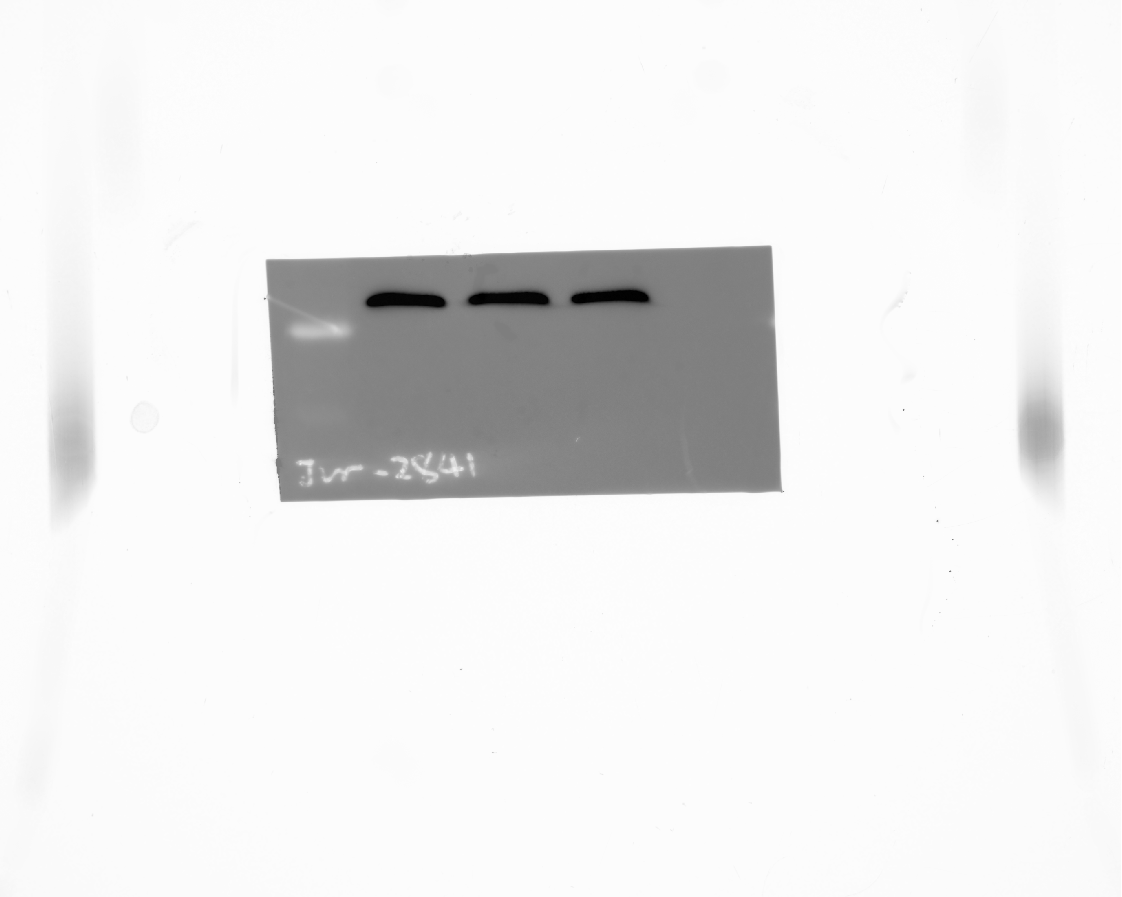

Supplement: Figure 4—figure supplement 1—source data 1. [file elife-106699-fig4-figsupp1-data1.zip › Figure 4ΓÇöfigure supplement 1-source data 2 Western blot raw datashows the verification of LMO2 non-expressing T-cells./pan-RAS Jurkat Abd-CRBN(Composite).tif]

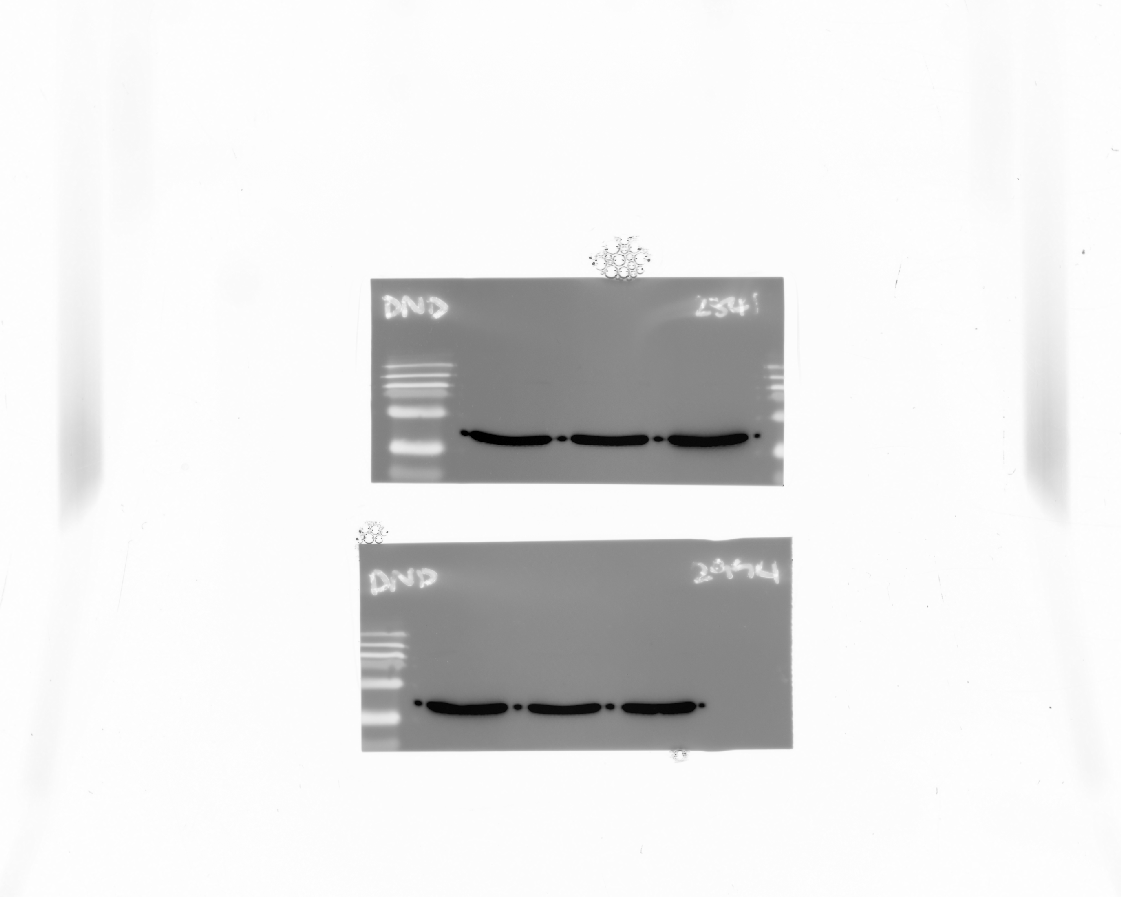

Supplement: Figure 4—figure supplement 1—source data 1. [file elife-106699-fig4-figsupp1-data1.zip › Figure 4ΓÇöfigure supplement 1-source data 2 Western blot raw datashows the verification of LMO2 non-expressing T-cells./Actin DND41(Composite).tif]

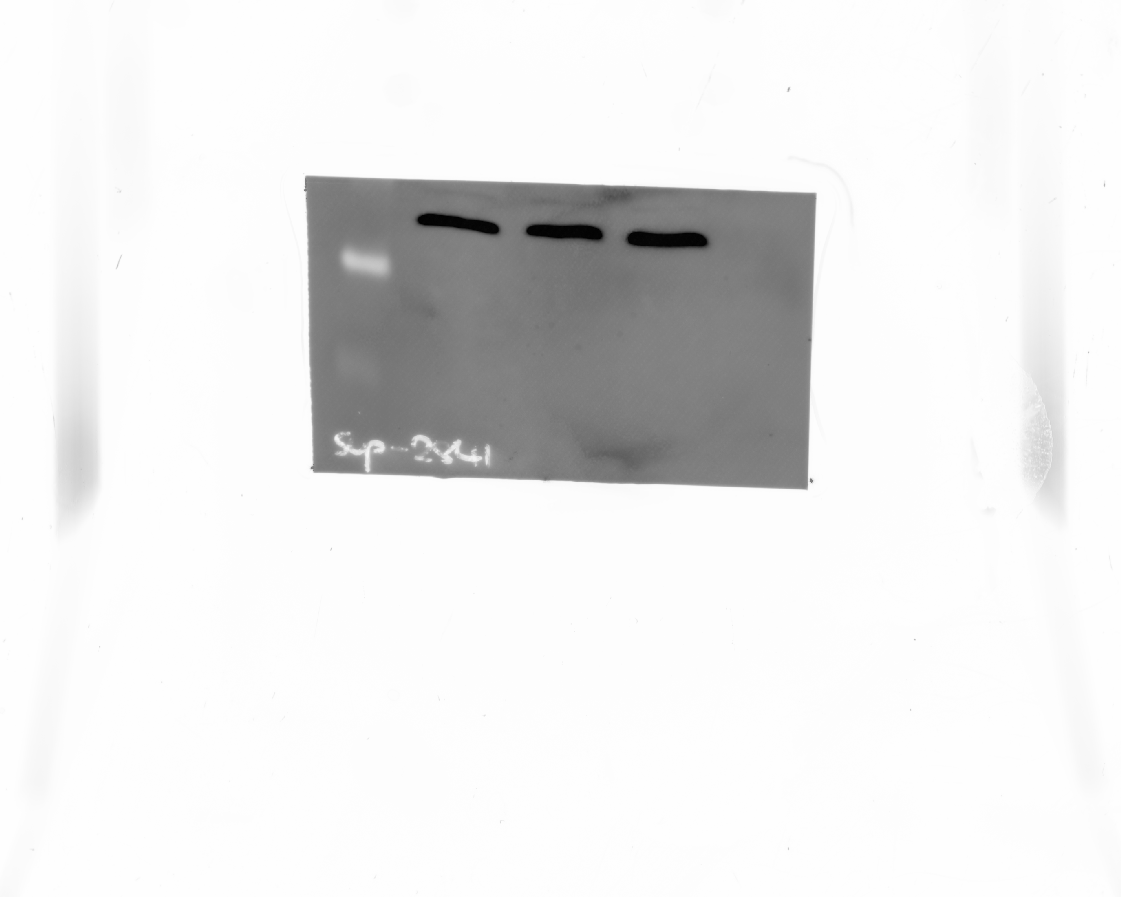

Supplement: Figure 4—figure supplement 1—source data 1. [file elife-106699-fig4-figsupp1-data1.zip › Figure 4ΓÇöfigure supplement 1-source data 2 Western blot raw datashows the verification of LMO2 non-expressing T-cells./pan-RAS SUPT-1 Abd-CRBN(Composite).tif]

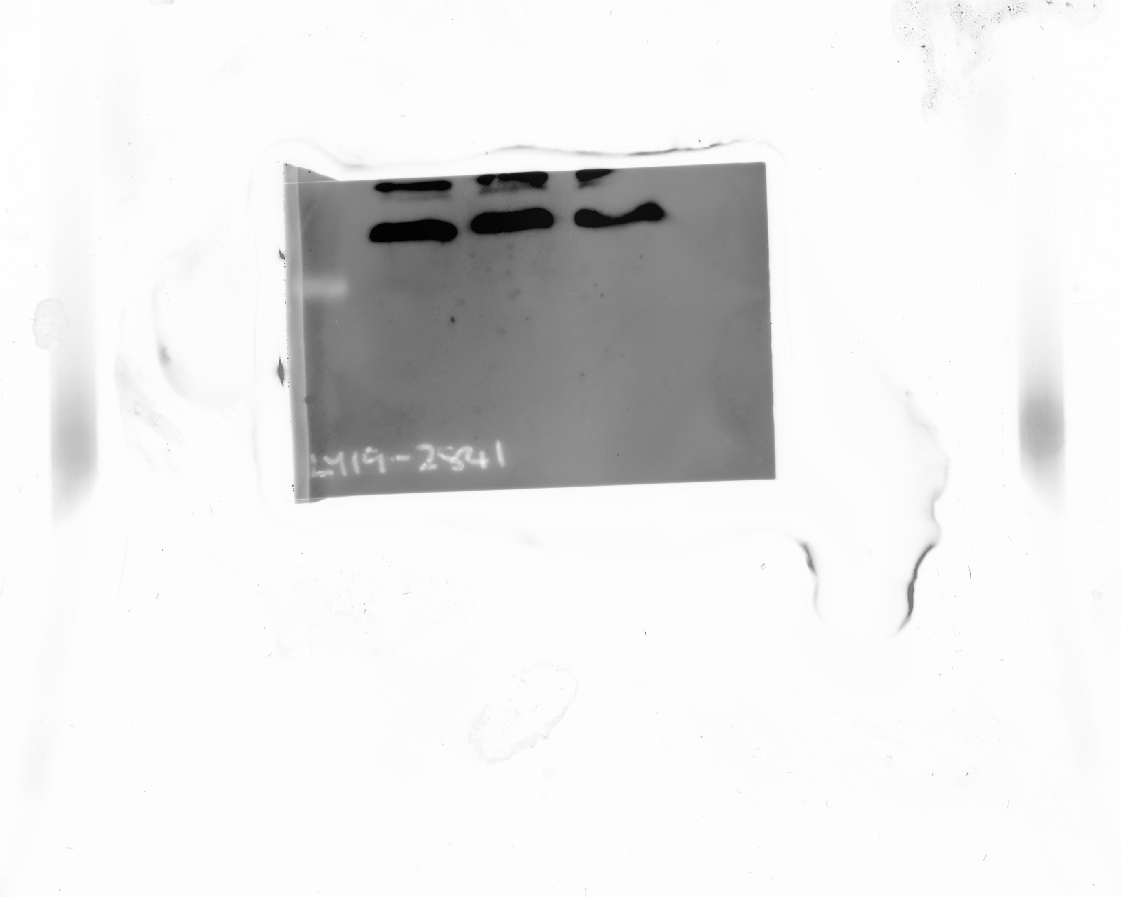

Supplement: Figure 4—figure supplement 1—source data 1. [file elife-106699-fig4-figsupp1-data1.zip › Figure 4ΓÇöfigure supplement 1-source data 2 Western blot raw datashows the verification of LMO2 non-expressing T-cells./pan-RAS RPMI8402 Abd-CRBN(Composite).tif]
