# Supplementary material for: Degradation of LMO2 in T cell leukaemia results in collateral breakdown of transcription complex partners and causes LMO2-dependent apoptosis
Source: eLife. 2025 Dec 12;14:RP106699. doi: 10.7554/eLife.106699 (PMC12700530; doi:10.7554/eLife.106699)
Supplement: Figure 4—source data 1. [file elife-106699-fig4-data1.zip › Figure 4ΓÇösource data 1 PDF files containing original western blots for Figure 4A, indicating the relevant bands and treatments./Figure 4-source data 1.pdf]

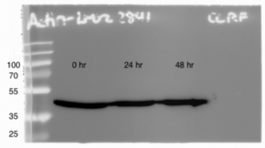

Actin in CCRF-CEM
treated with Abd-CRBN

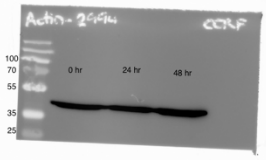

Actin in CCRF-CEM
treated with Abd-VHL

Actin in KOPT-K1
treated with Abd-CRBN and Abd-CRBN

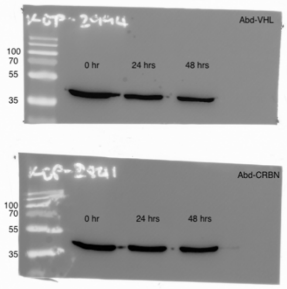

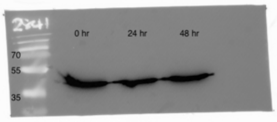

Actin in LOUCY
treated with Abd-CRBN

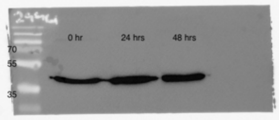

Actin in LOUCY
treated with Abd-VHL

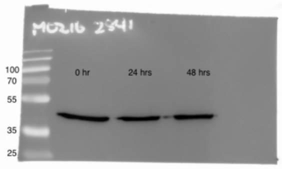

Actin in MOLT-16
treated with Abd-CRBN

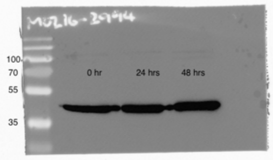

Actin in MOLT-16
treated with Abd-VHL

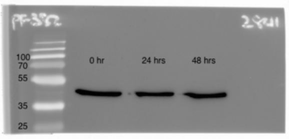

Actin in PF-382
treated with Abd-CRBN

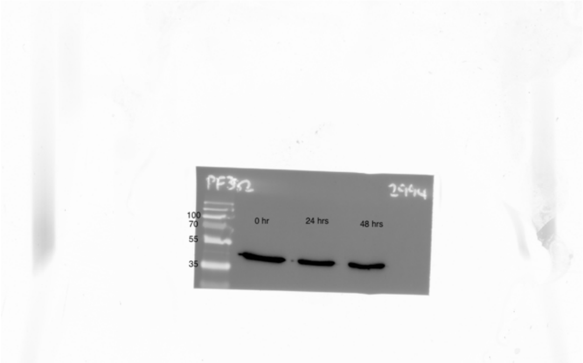

Actin in PF-382
treated with Abd-VHL

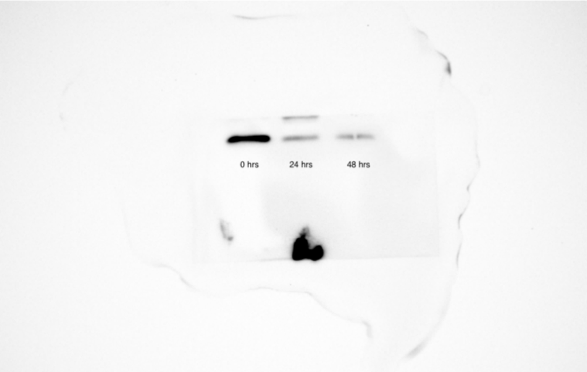

LMO2 in CCRF-CEM
treated with Abd-CRBN

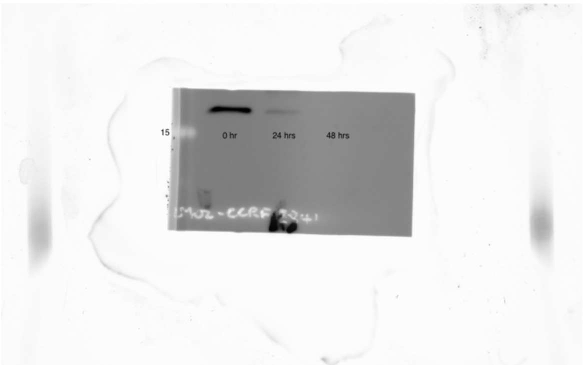

LMO2 in CCRF-CEM
treated with Abd-VHL

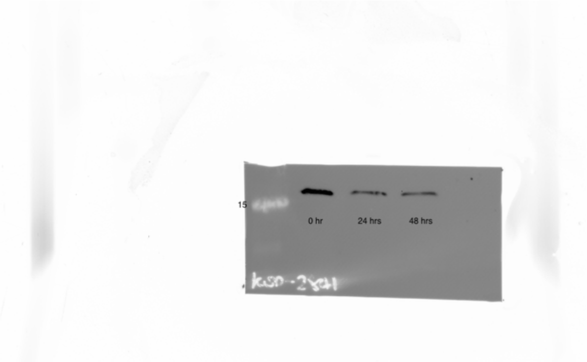

LMO2 in KOPT-K1
treated with Abd-CRBN

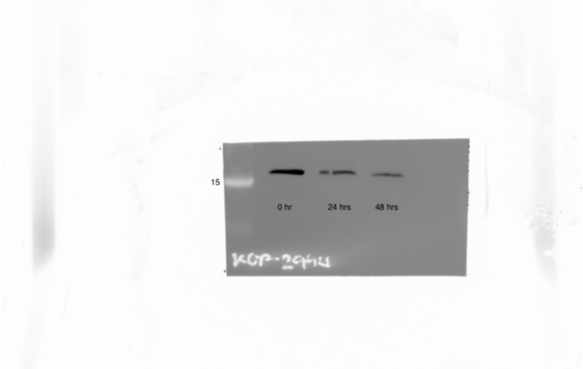

LMO2 in KOPT-K1
treated with Abd-VHL

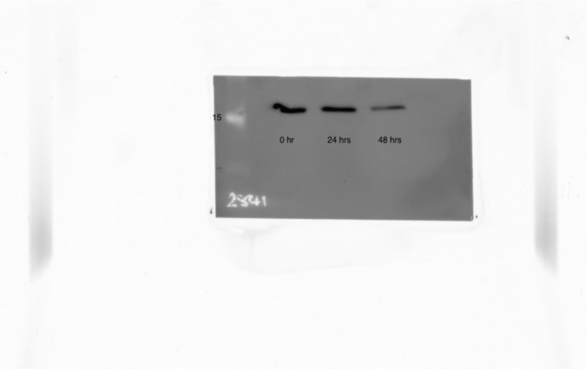

LMO2 in LOUCY
treated with Abd-CRBN

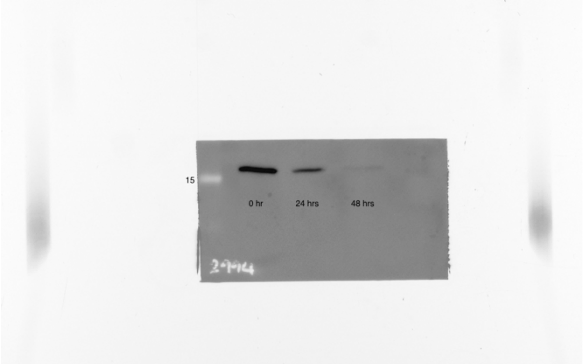

LMO2 in LOUCY
treated with Abd-VHL

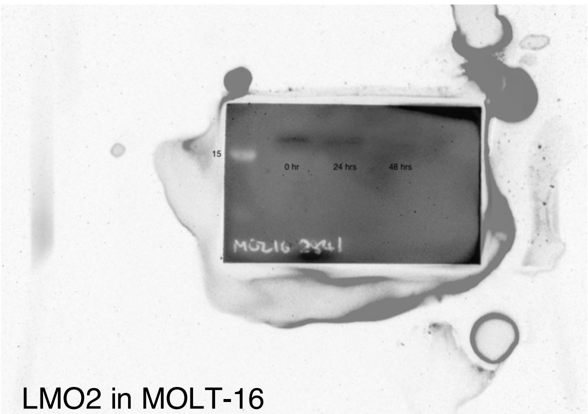

LMO2 in MOLT-16
treated with Abd-CRBN

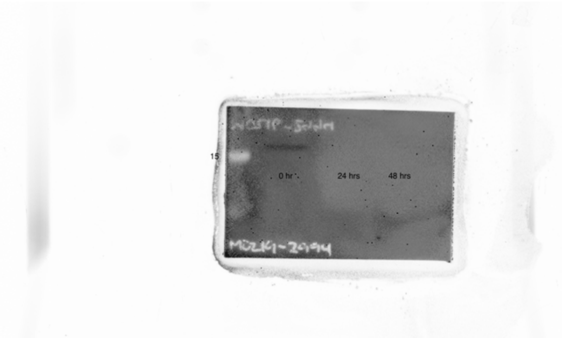

LMO2 in MOLT-16
treated with Abd-VHL

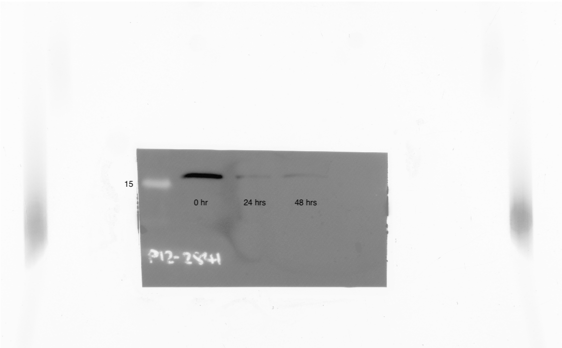

LMO2 in P12-Ichikawa
treated with Abd-CRBN

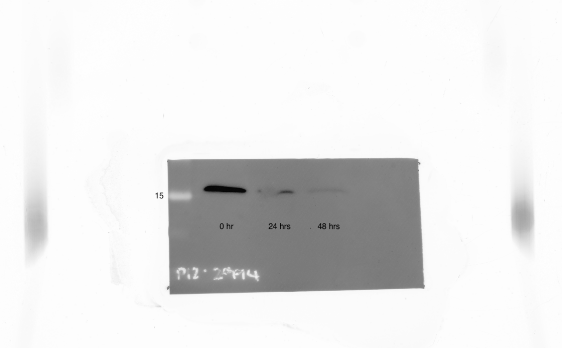

LMO2 in P12-Ichikawa
treated with Abd-VHL

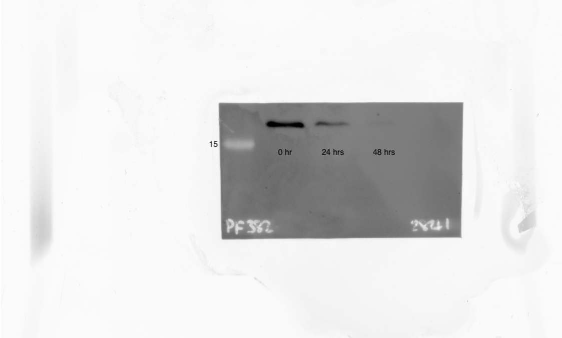

LMO2 in PF-382
treated with Abd-CRBN

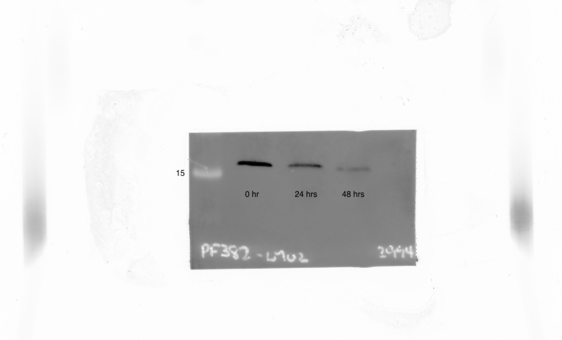

LMO2 in PF-382
treated with Abd-VHL

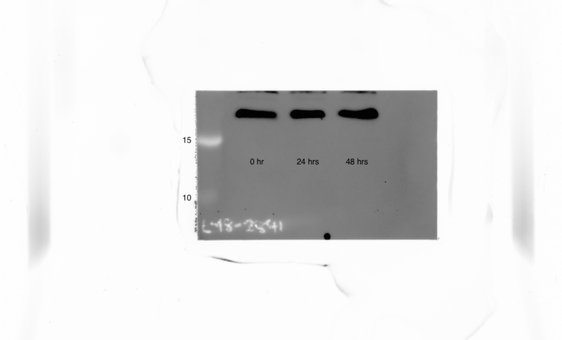

pan-RAS in CCRF-CEM
treated with Abd-CRBN

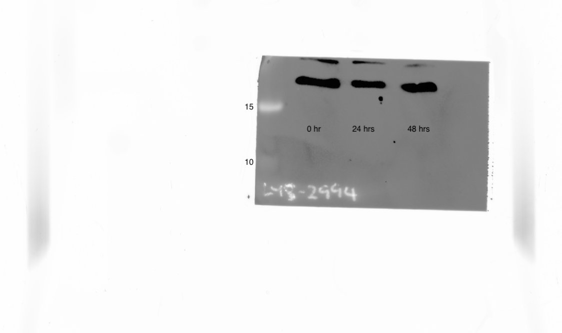

pan-RAS in CCRF-CEM
treated with Abd-VHL

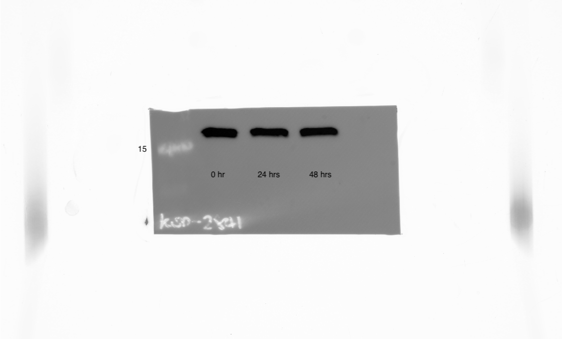

pan-RAS in KOPT-K1
treated with Abd-CRBN

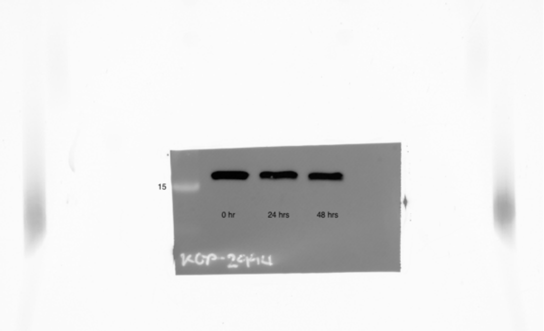

pan-RAS in KOPT-K1
treated with Abd-VHL

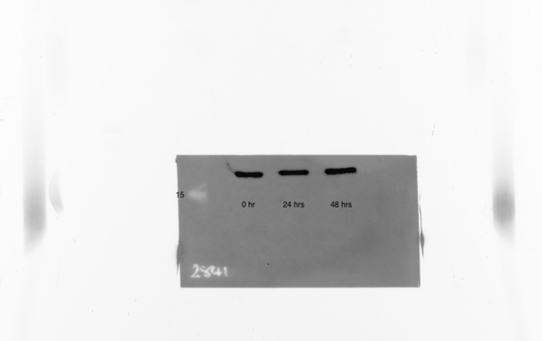

pan-RAS in LOUCY
treated with Abd-CRBN

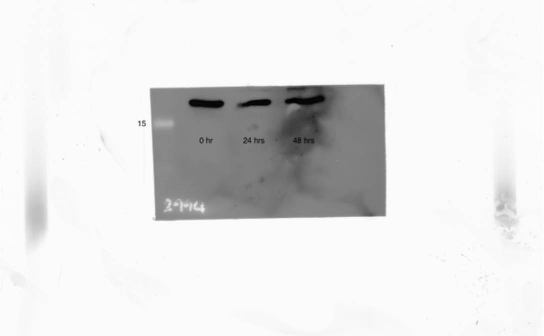

pan-RAS in LOUCY
treated with Abd-VHL

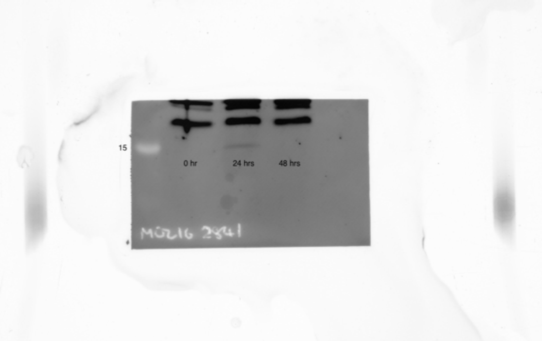

pan-RAS in MOLT-16
treated with Abd-CRBN

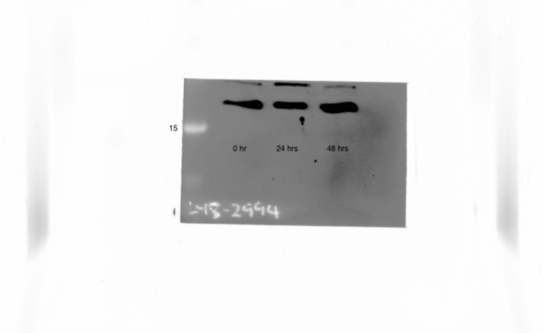

pan-RAS in MOLT-16
treated with Abd-VHL

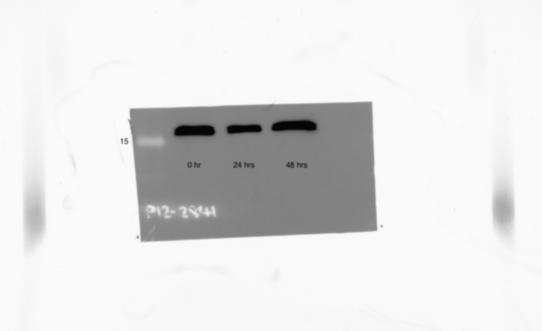

pan-RAS in P12-Ichikawa
treated with Abd-CRBN

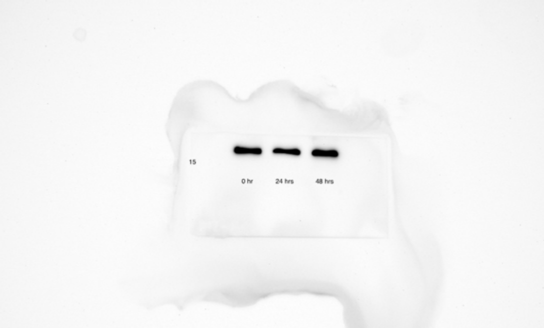

pan-RAS in P12-Ichikawa
treated with Abd-VHL

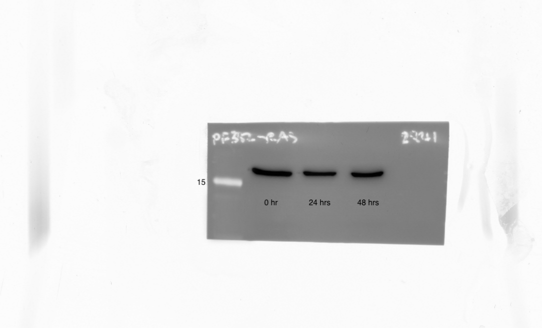

pan-RAS in PF-382
treated with Abd-CRBN

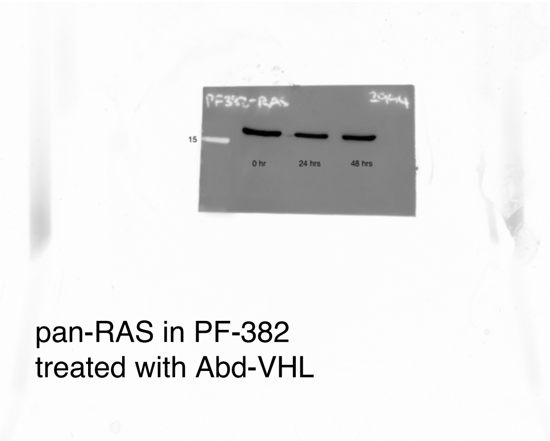

Figure 4, Source Data 1. Original membranes corresponding to Figure 4, panel A.
