# Supplementary figures and images for: Degradation of LMO2 in T cell leukaemia results in collateral breakdown of transcription complex partners and causes LMO2-dependent apoptosis (part 5 of 5)
Source: eLife. 2025 Dec 12;14:RP106699. doi: 10.7554/eLife.106699 (PMC12700530; doi:10.7554/eLife.106699)

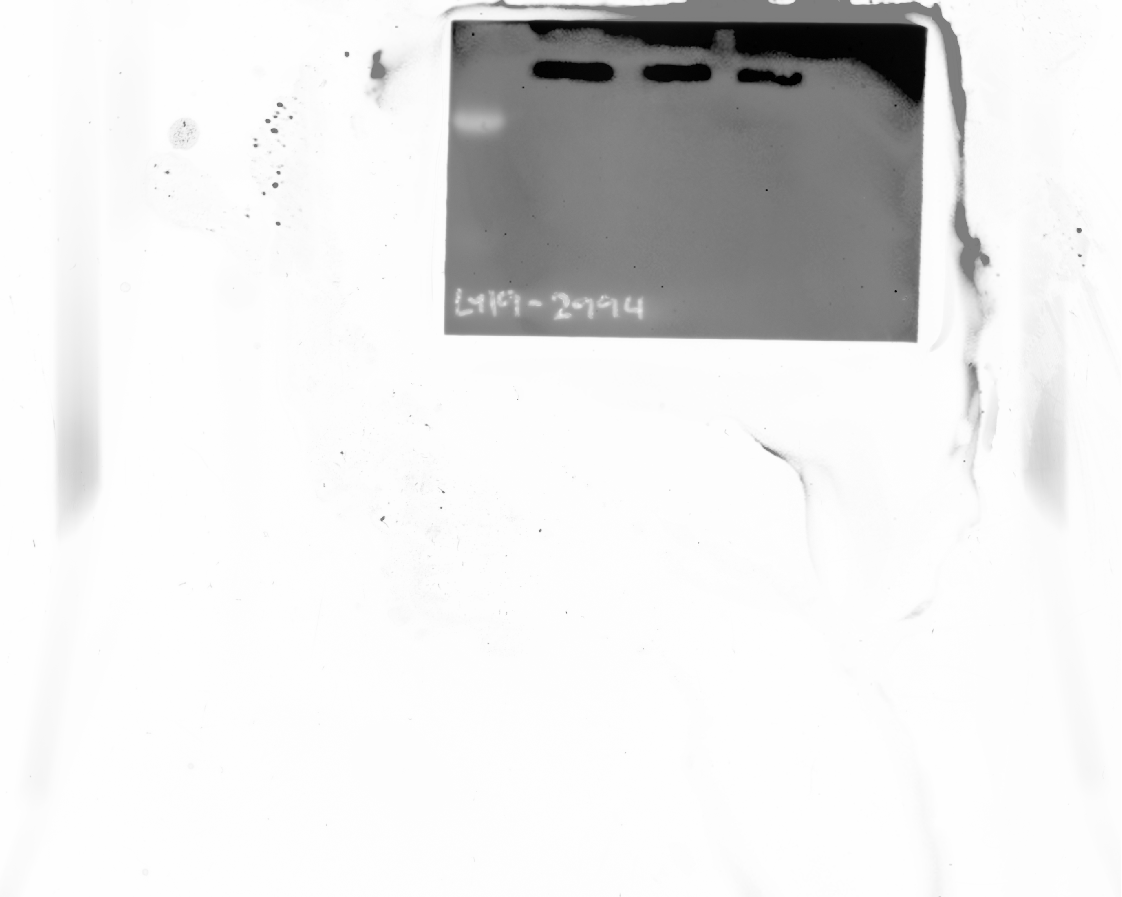

Supplement: Figure 4—figure supplement 1—source data 1. [file elife-106699-fig4-figsupp1-data1.zip › Figure 4ΓÇöfigure supplement 1-source data 2 Western blot raw datashows the verification of LMO2 non-expressing T-cells./pan-Ras RPMI8402 Abd-VHL(Composite).tif]

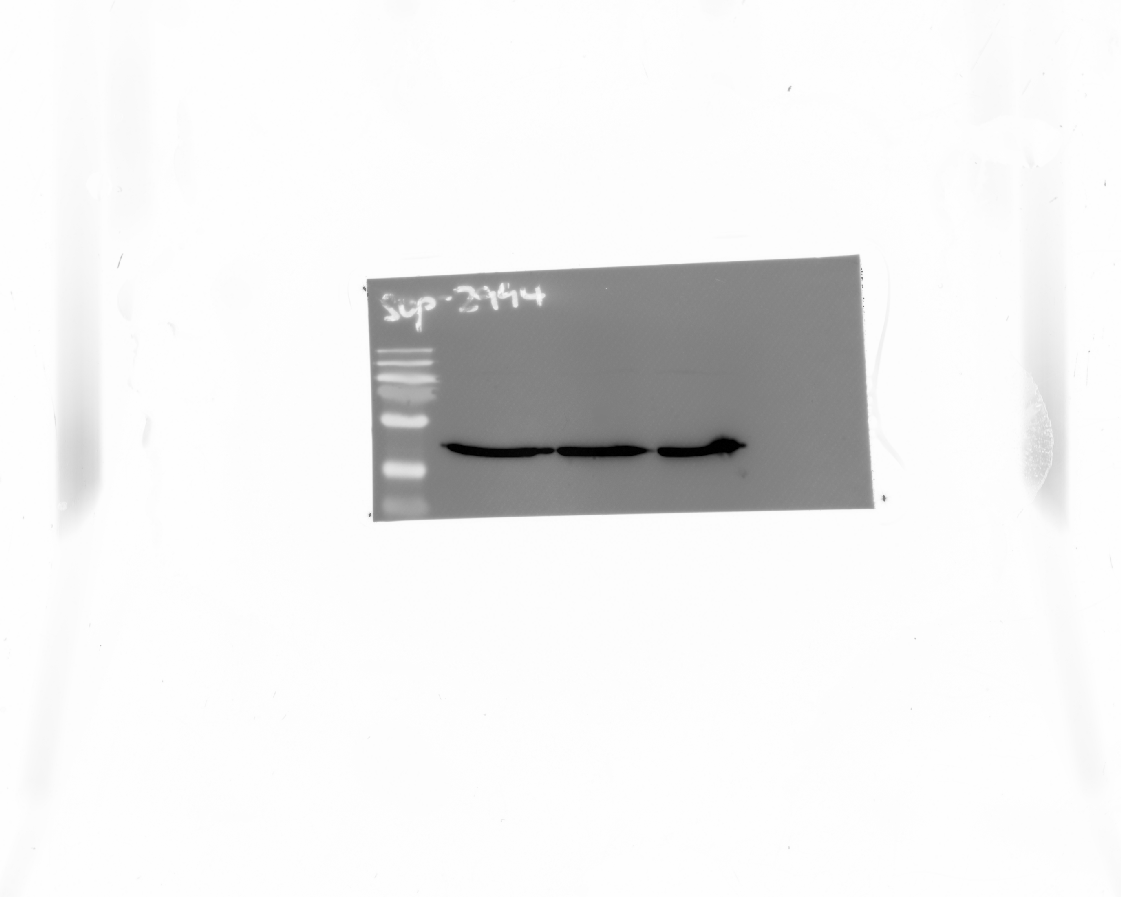

Supplement: Figure 4—figure supplement 1—source data 1. [file elife-106699-fig4-figsupp1-data1.zip › Figure 4ΓÇöfigure supplement 1-source data 2 Western blot raw datashows the verification of LMO2 non-expressing T-cells./Acton SUPT-1 Abd-VHL(Composite).tif]

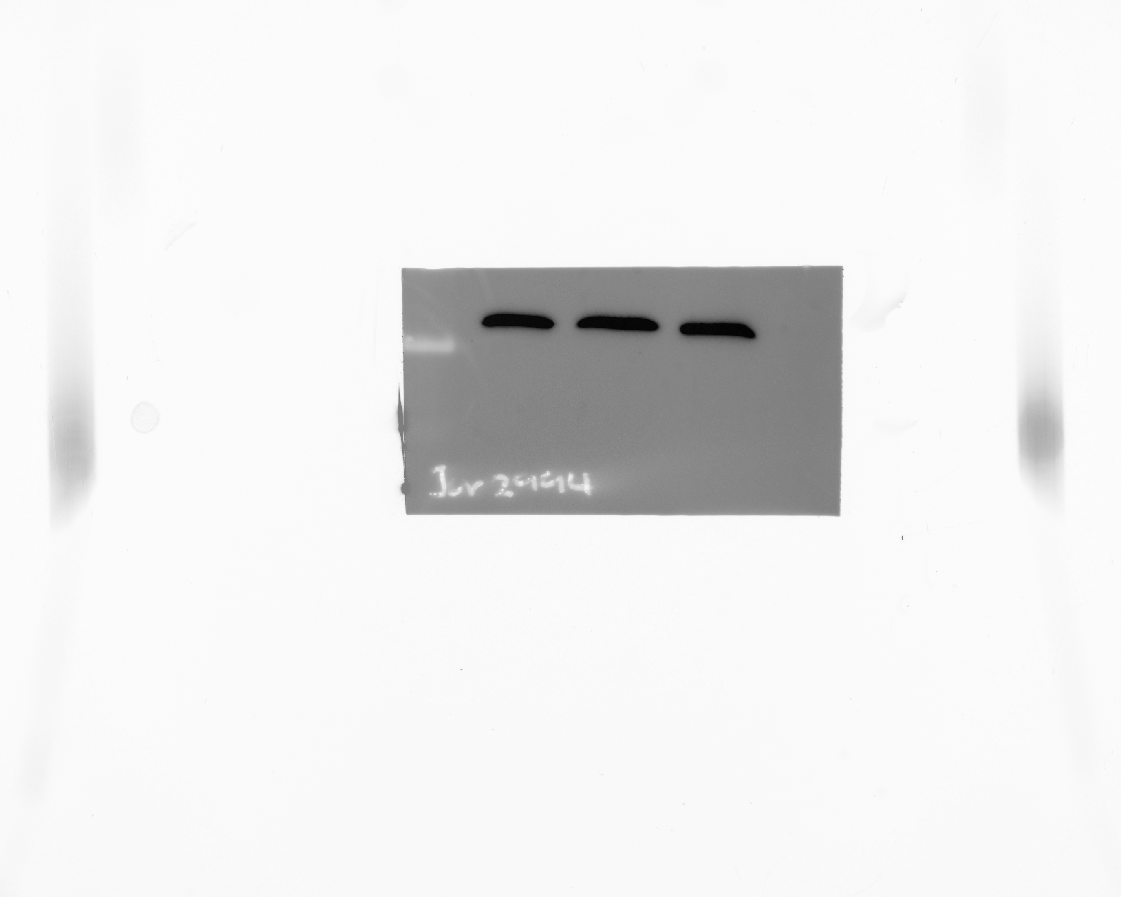

Supplement: Figure 4—figure supplement 1—source data 1. [file elife-106699-fig4-figsupp1-data1.zip › Figure 4ΓÇöfigure supplement 1-source data 2 Western blot raw datashows the verification of LMO2 non-expressing T-cells./pan-RAS Jurkat Abd-VHL(Composite).tif]

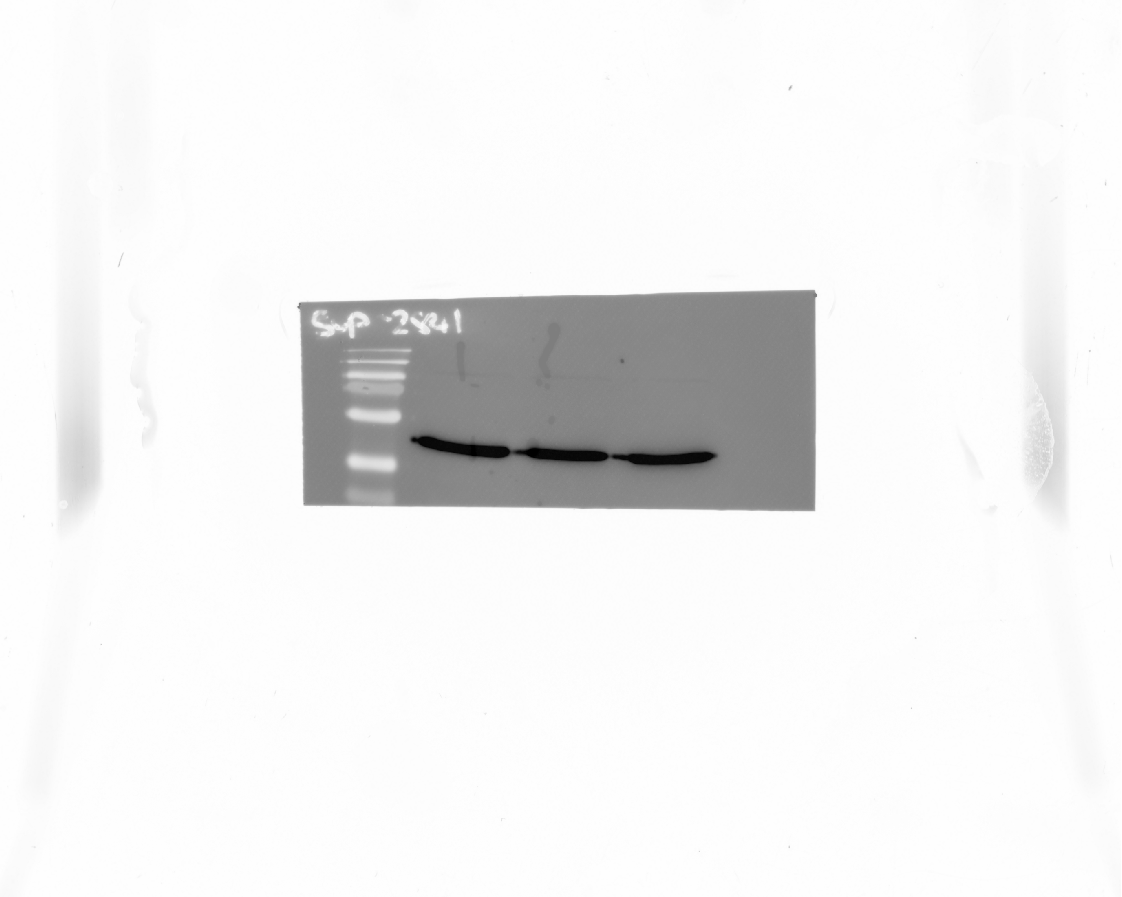

Supplement: Figure 4—figure supplement 1—source data 1. [file elife-106699-fig4-figsupp1-data1.zip › Figure 4ΓÇöfigure supplement 1-source data 2 Western blot raw datashows the verification of LMO2 non-expressing T-cells./Actin SUPT-1 Abd-CRBN(Composite).tif]

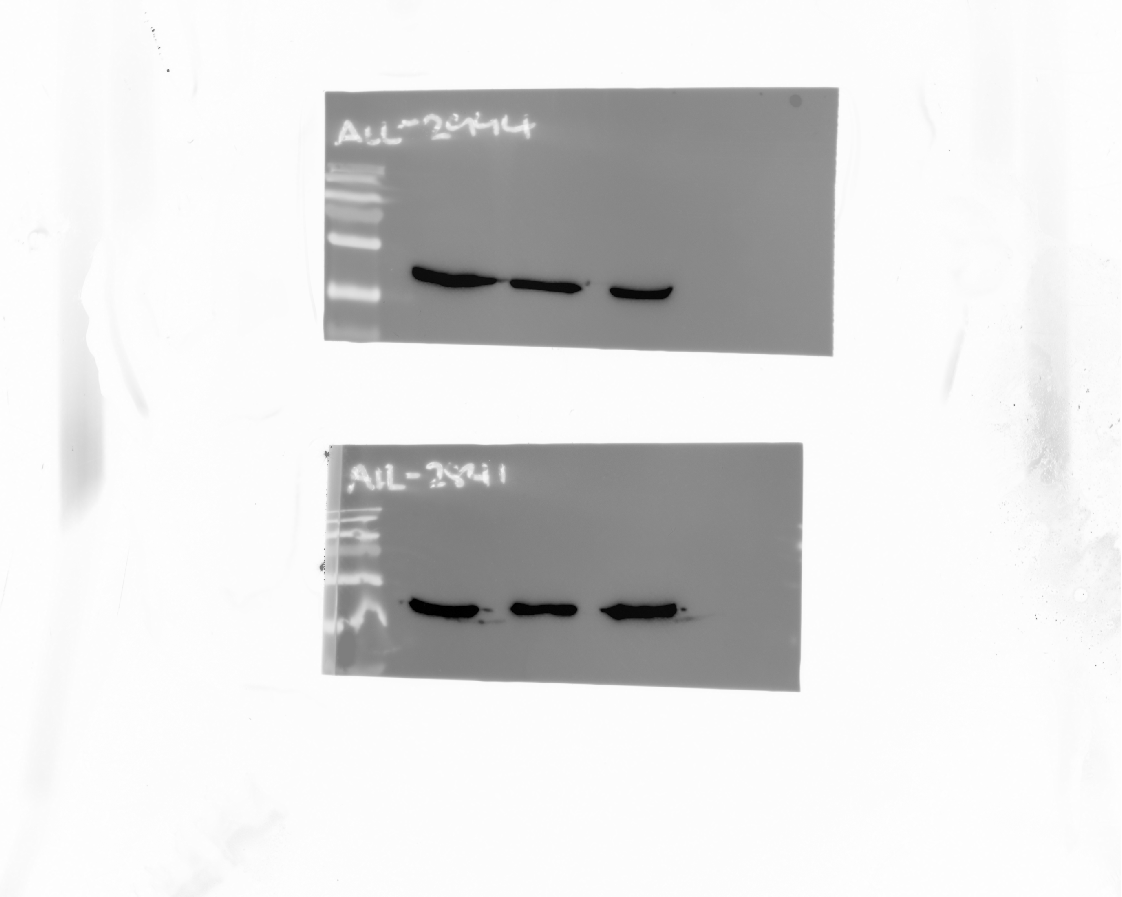

Supplement: Figure 4—figure supplement 1—source data 1. [file elife-106699-fig4-figsupp1-data1.zip › Figure 4ΓÇöfigure supplement 1-source data 2 Western blot raw datashows the verification of LMO2 non-expressing T-cells./Actin ALL-SIL(Composite).tif]

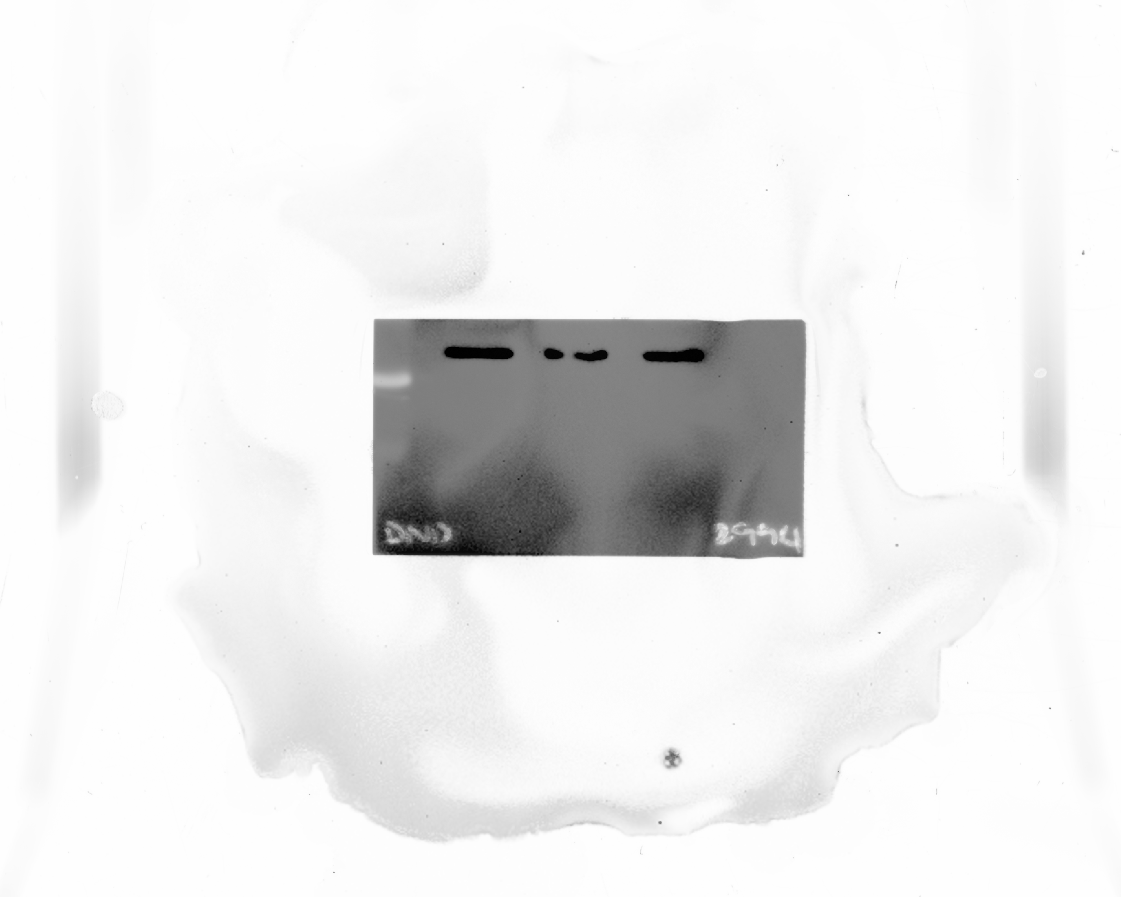

Supplement: Figure 4—figure supplement 1—source data 1. [file elife-106699-fig4-figsupp1-data1.zip › Figure 4ΓÇöfigure supplement 1-source data 2 Western blot raw datashows the verification of LMO2 non-expressing T-cells./pan-RAS DND41 Abd-VHL(Composite).tif]

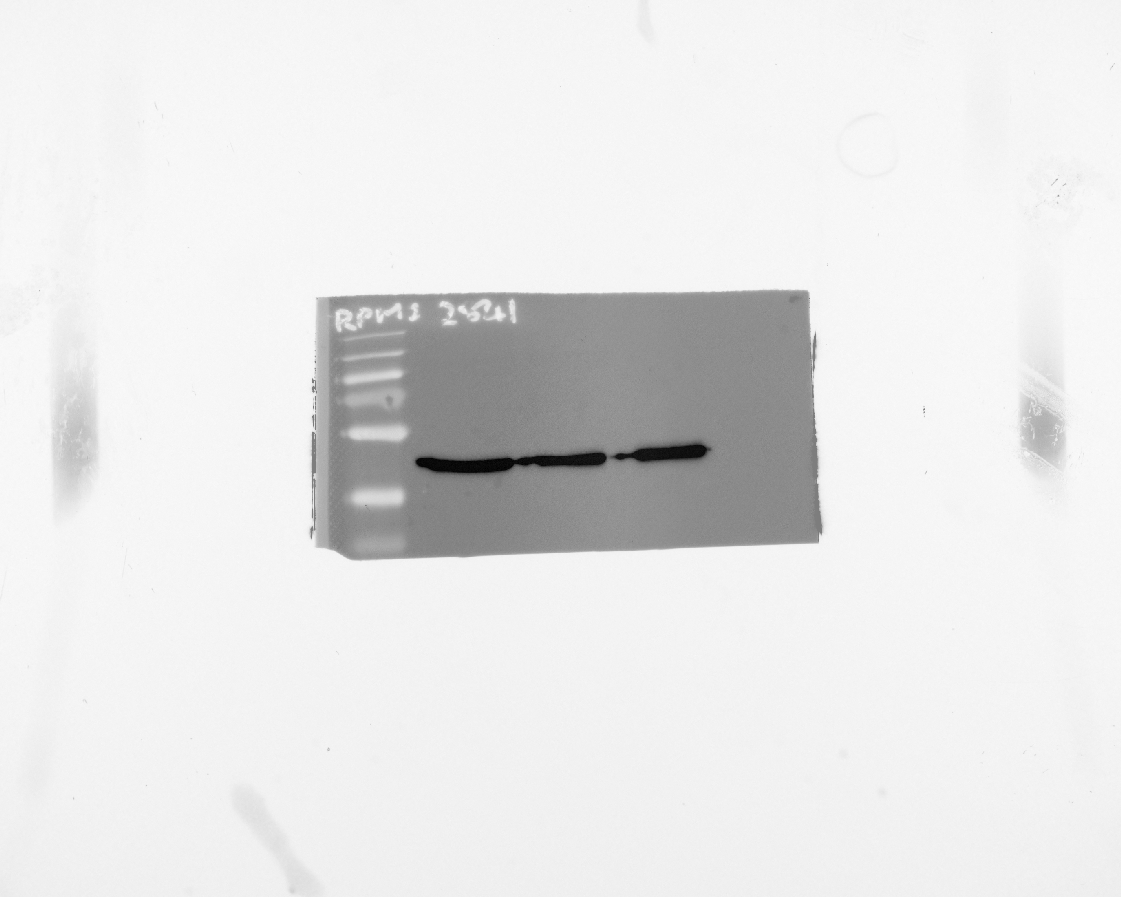

Supplement: Figure 4—figure supplement 1—source data 1. [file elife-106699-fig4-figsupp1-data1.zip › Figure 4ΓÇöfigure supplement 1-source data 2 Western blot raw datashows the verification of LMO2 non-expressing T-cells./Actin RPMI8402 Abd-CRBN(Composite).tif]

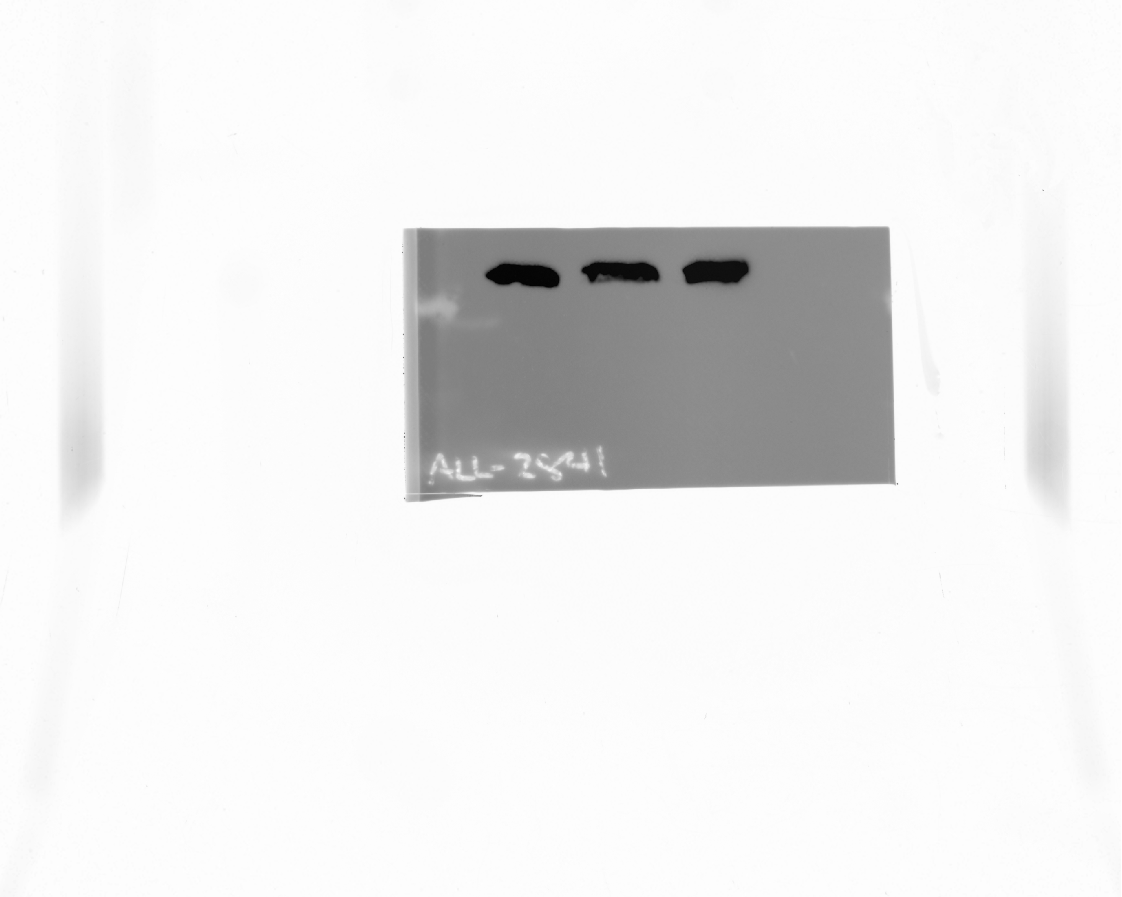

Supplement: Figure 4—figure supplement 1—source data 1. [file elife-106699-fig4-figsupp1-data1.zip › Figure 4ΓÇöfigure supplement 1-source data 2 Western blot raw datashows the verification of LMO2 non-expressing T-cells./pan-RAS ALL-SIL Abd-CRBN(Composite).tif]

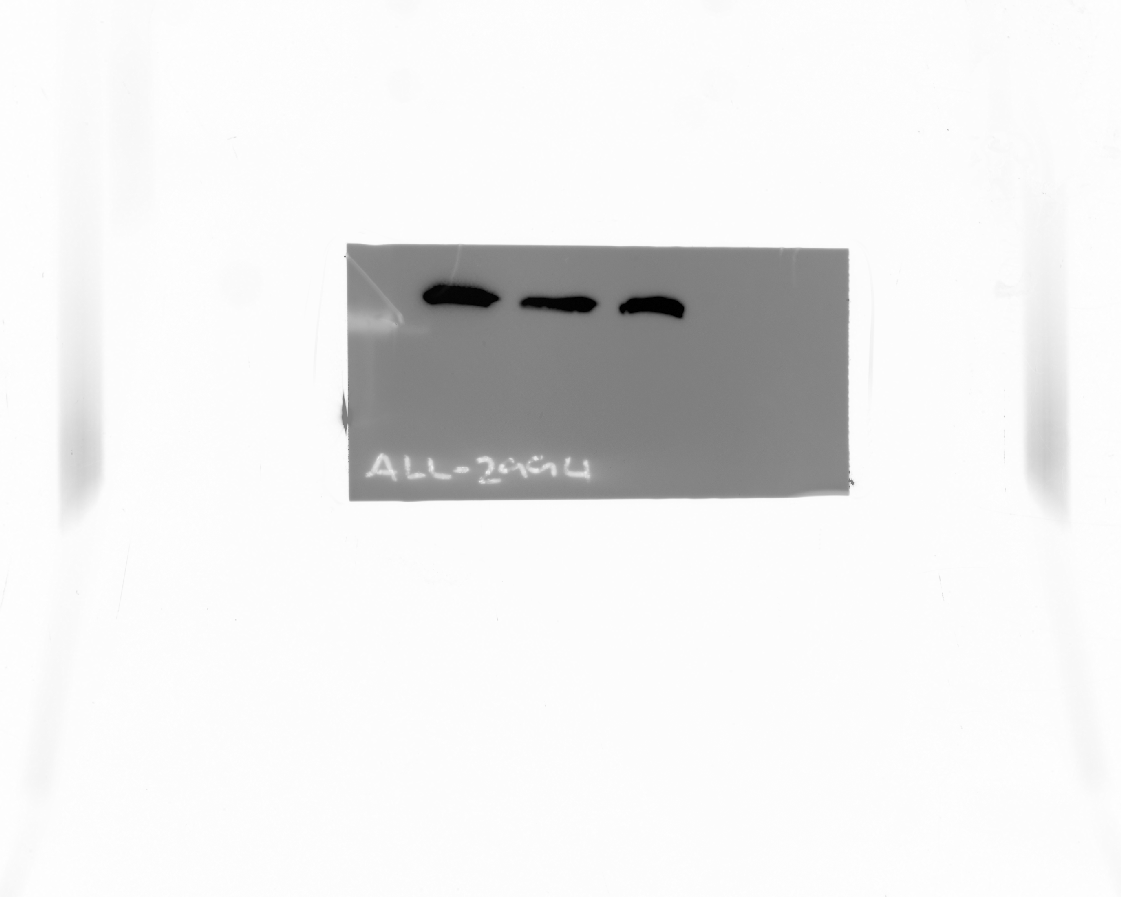

Supplement: Figure 4—figure supplement 1—source data 1. [file elife-106699-fig4-figsupp1-data1.zip › Figure 4ΓÇöfigure supplement 1-source data 2 Western blot raw datashows the verification of LMO2 non-expressing T-cells./pan-RAS ALL-SIL Abd-VHL(Composite).tif]

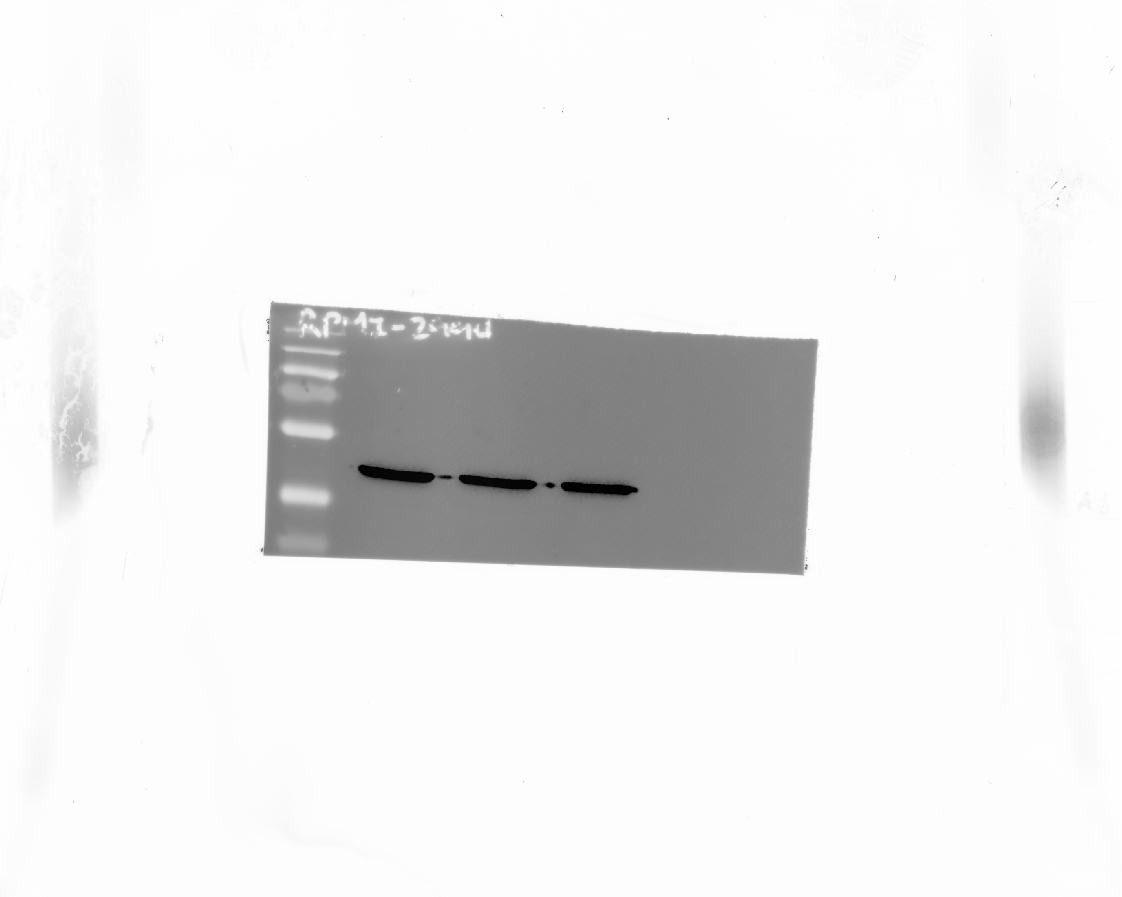

Supplement: Figure 4—figure supplement 1—source data 1. [file elife-106699-fig4-figsupp1-data1.zip › Figure 4ΓÇöfigure supplement 1-source data 2 Western blot raw datashows the verification of LMO2 non-expressing T-cells./Actin RPMI8402 Abd-VHL(Composite).tif]

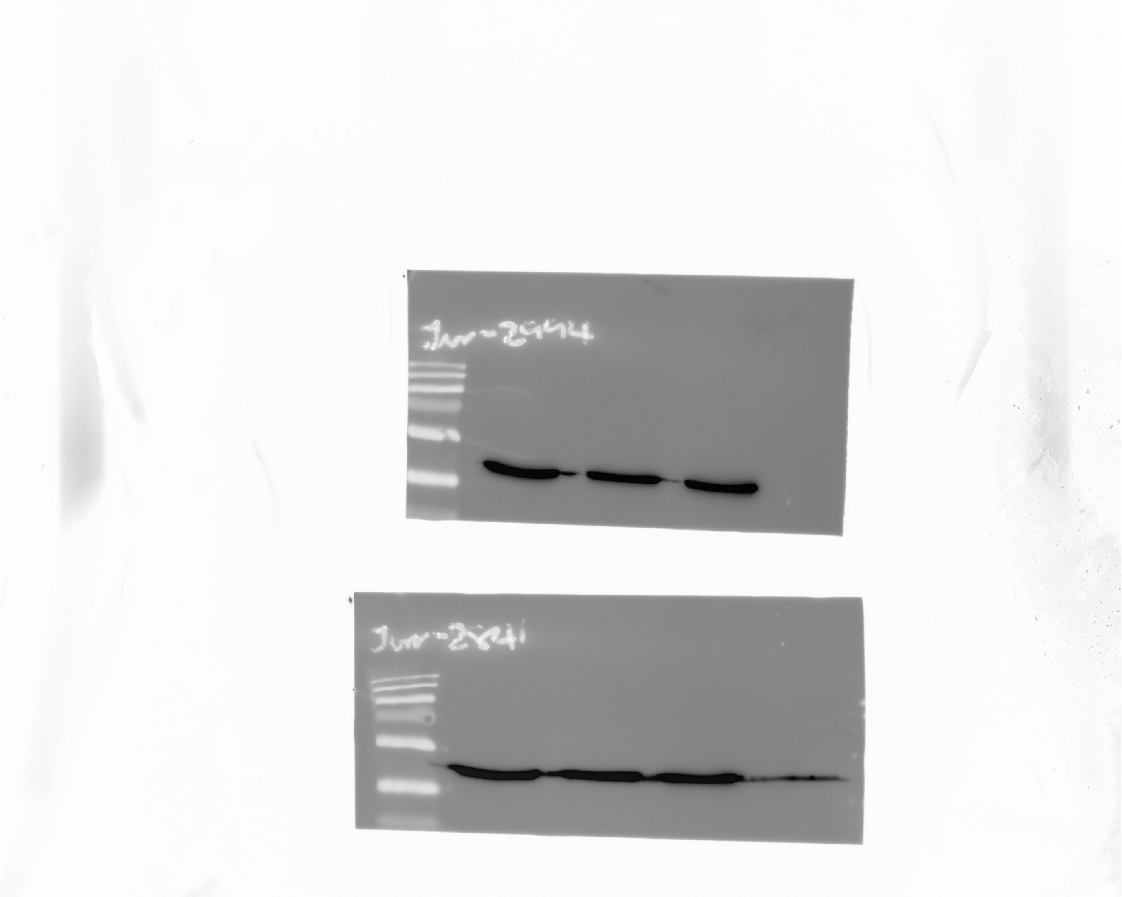

Supplement: Figure 4—figure supplement 1—source data 1. [file elife-106699-fig4-figsupp1-data1.zip › Figure 4ΓÇöfigure supplement 1-source data 2 Western blot raw datashows the verification of LMO2 non-expressing T-cells./Actin Jurkat (Composite).tif]

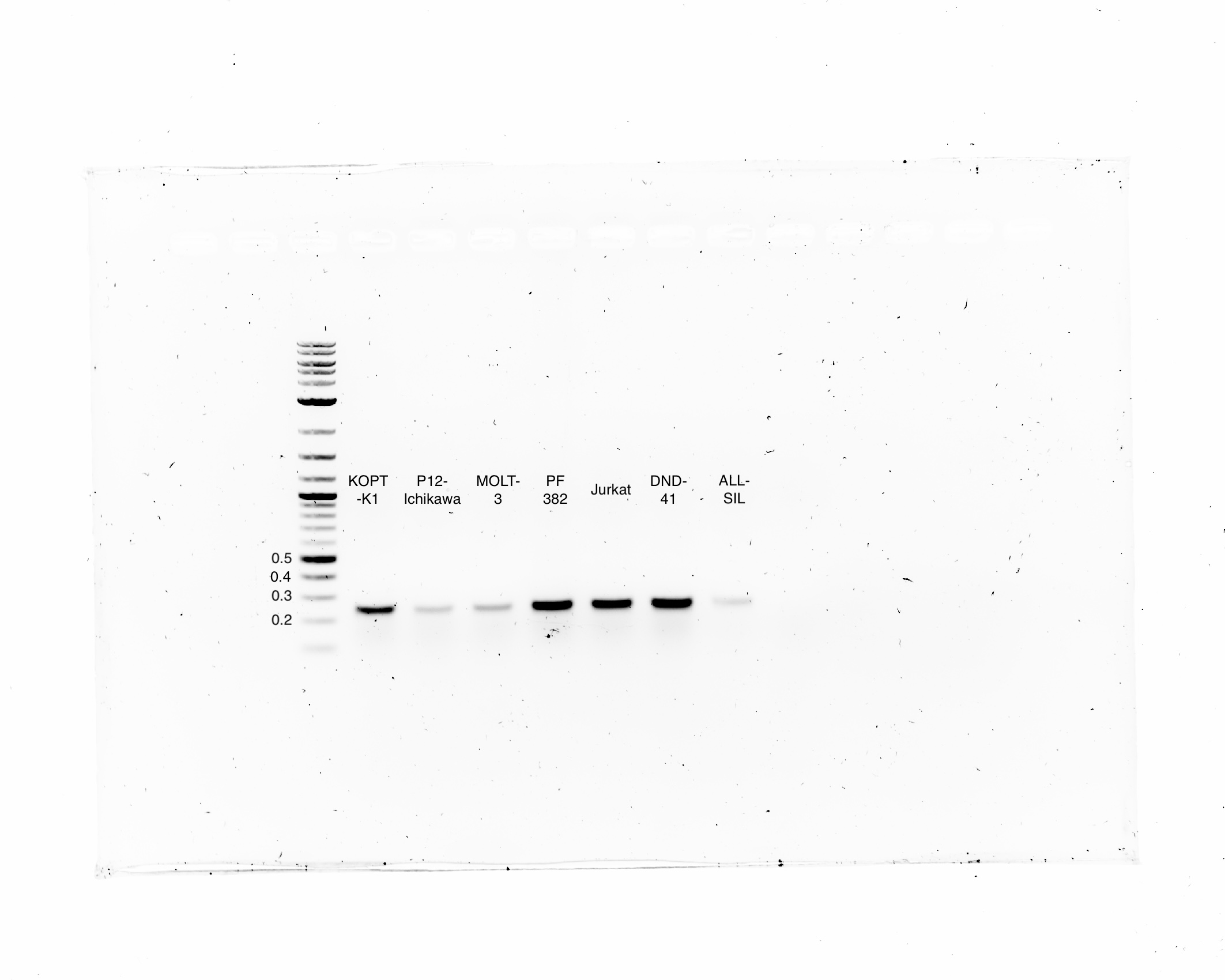

Supplement: Figure 4—figure supplement 2—source data 1. [file elife-106699-fig4-figsupp2-data1.zip › Figure 4ΓÇöfigure supplement 2-source data 1 Agarose gel data with label shows PCR products from RT-PCR analysis of the human T-ALL cells./Raw data/RT-PCR KRAS(SYBR Safe).jpg]

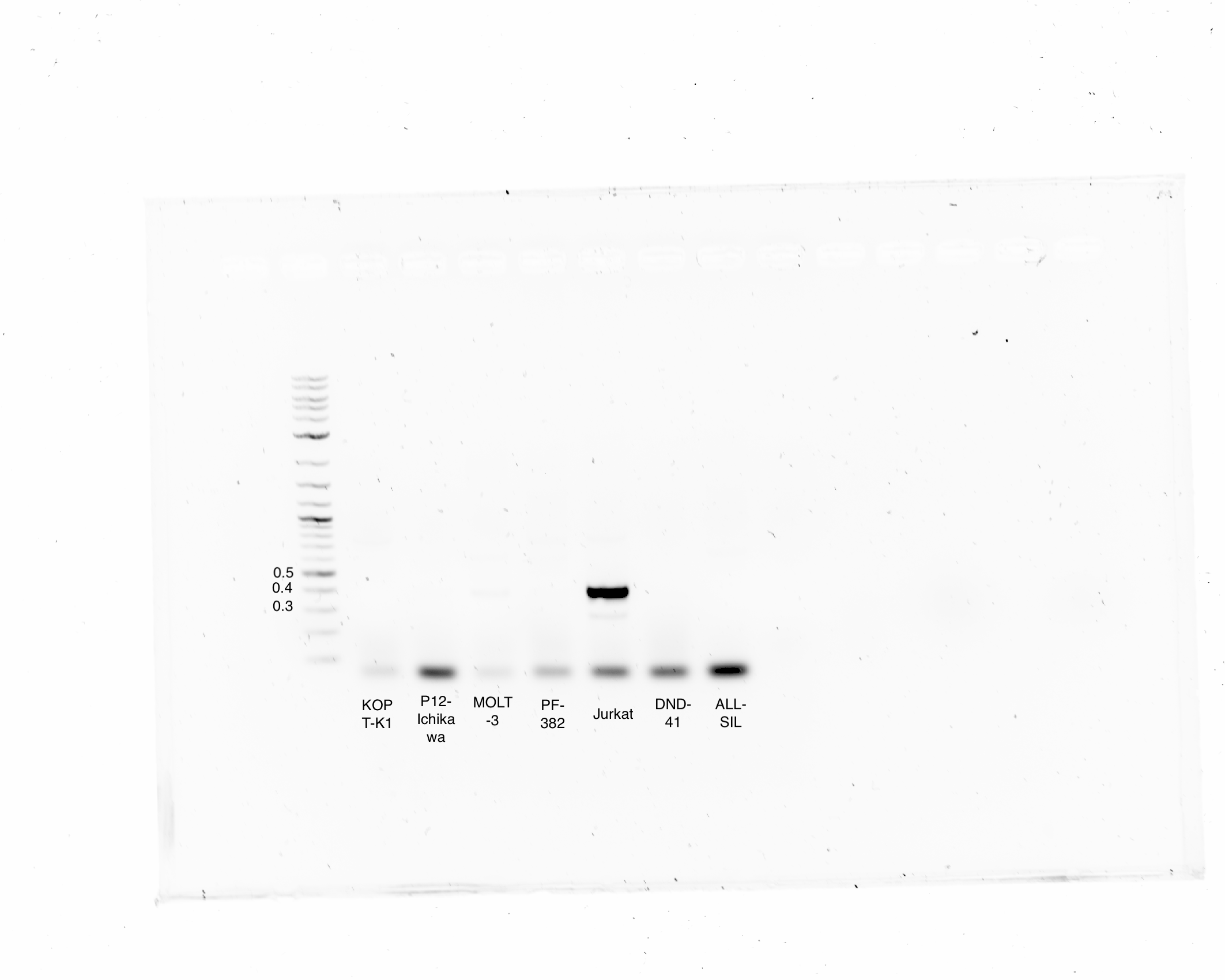

Supplement: Figure 4—figure supplement 2—source data 1. [file elife-106699-fig4-figsupp2-data1.zip › Figure 4ΓÇöfigure supplement 2-source data 1 Agarose gel data with label shows PCR products from RT-PCR analysis of the human T-ALL cells./Raw data/RT-PCR LMO1(SYBR Safe).tif]

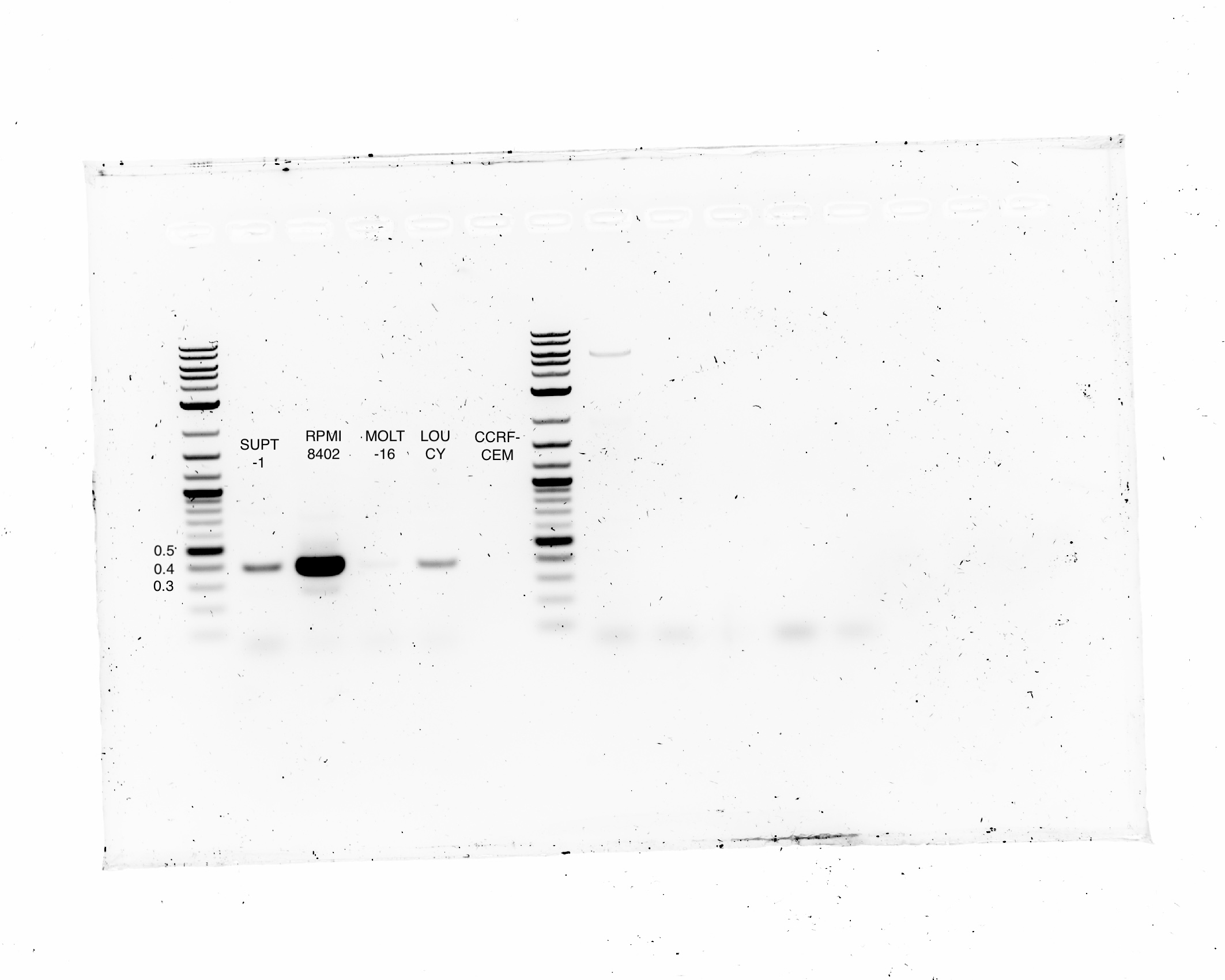

Supplement: Figure 4—figure supplement 2—source data 1. [file elife-106699-fig4-figsupp2-data1.zip › Figure 4ΓÇöfigure supplement 2-source data 1 Agarose gel data with label shows PCR products from RT-PCR analysis of the human T-ALL cells./Raw data/RT-PCR LMO1(SYBR Safe).jpg]

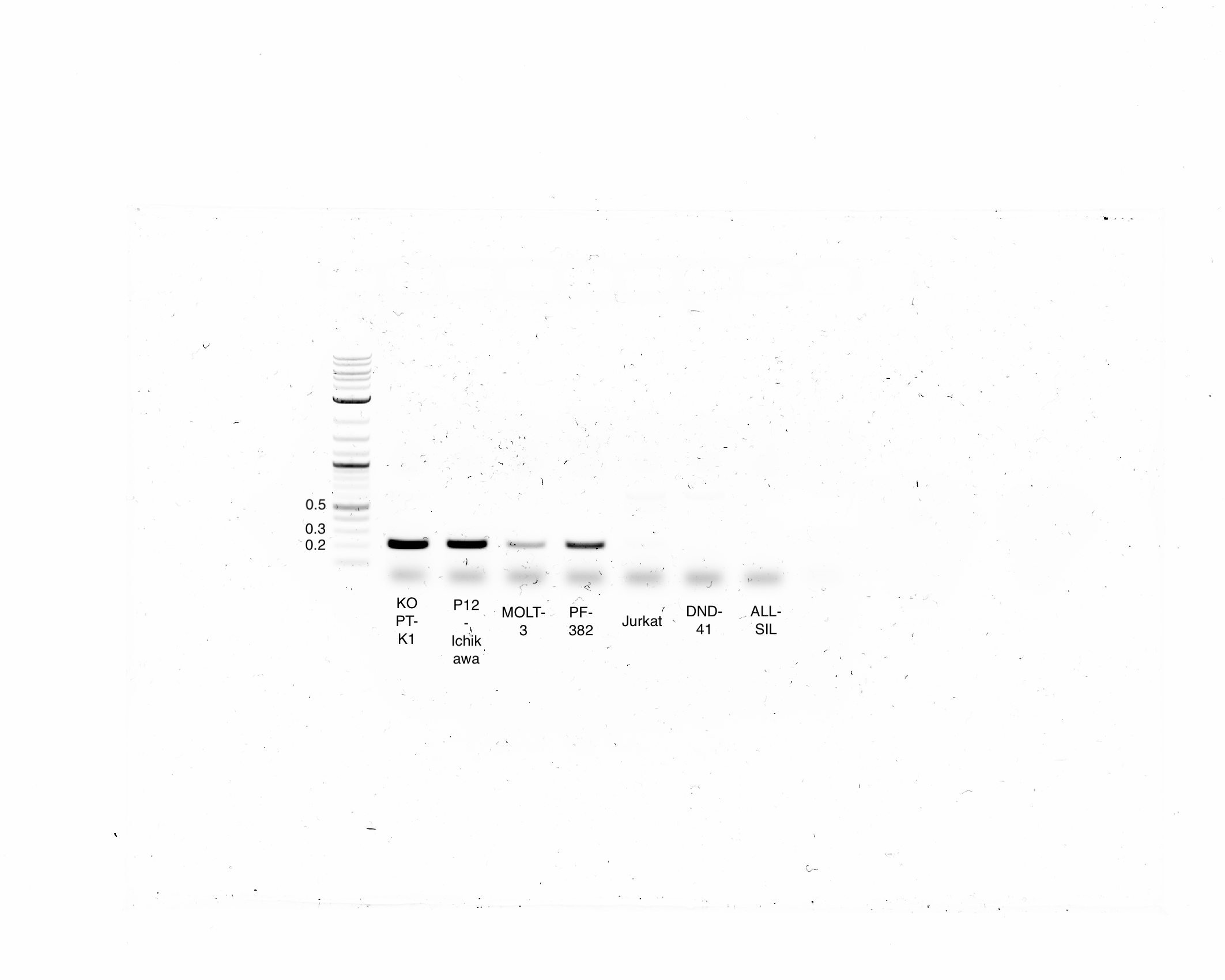

Supplement: Figure 4—figure supplement 2—source data 1. [file elife-106699-fig4-figsupp2-data1.zip › Figure 4ΓÇöfigure supplement 2-source data 1 Agarose gel data with label shows PCR products from RT-PCR analysis of the human T-ALL cells./Raw data/RT-PCR LMO2(SYBR Safe).tif]

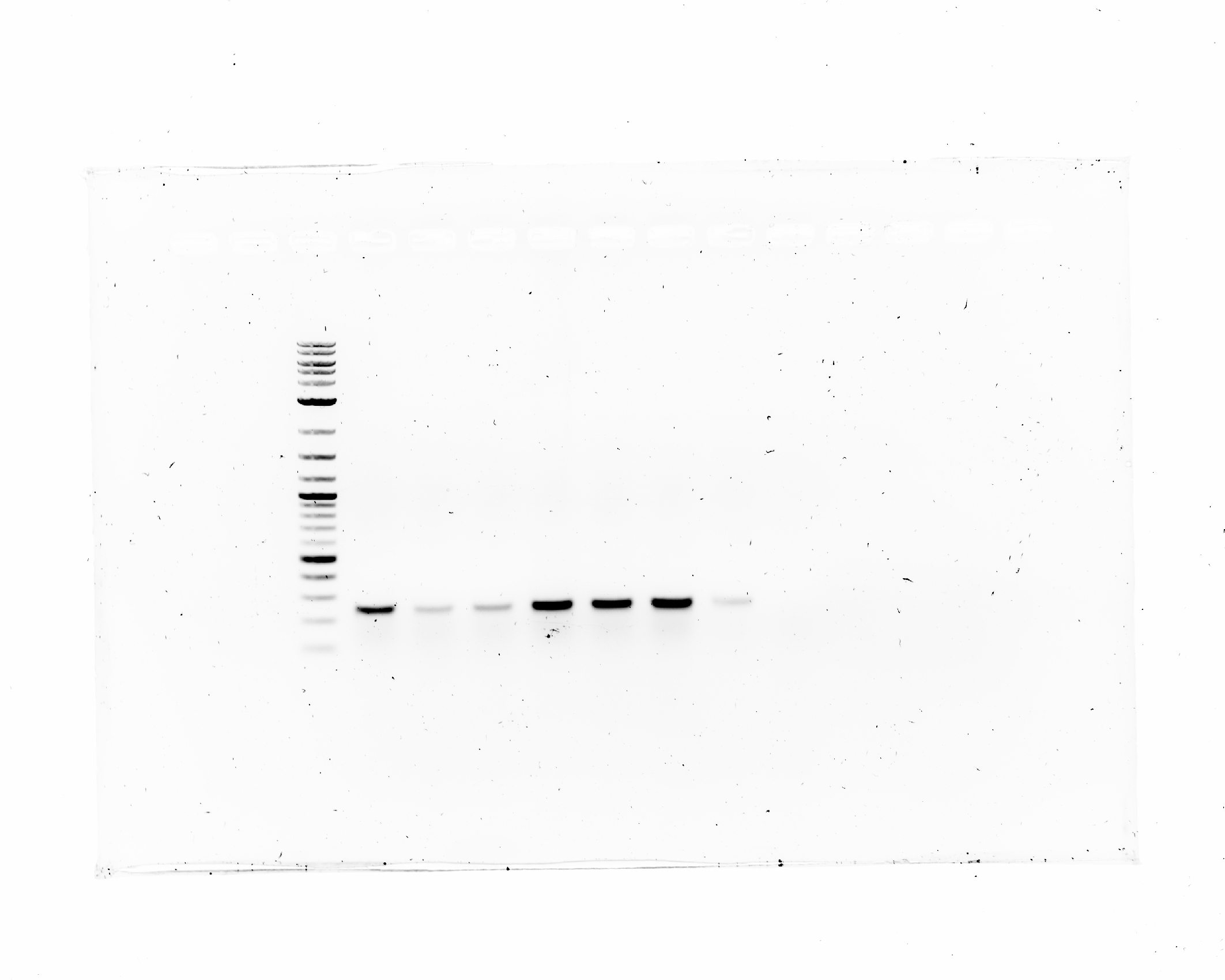

Supplement: Figure 4—figure supplement 2—source data 2. [file elife-106699-fig4-figsupp2-data2.zip › Figure 4ΓÇöfigure supplement 2-source data 2 Agarose gel raw data shows PCR products from RT-PCR analysis of the human T-ALL cells./RT-PCR KRAS(SYBR Safe).jpg]

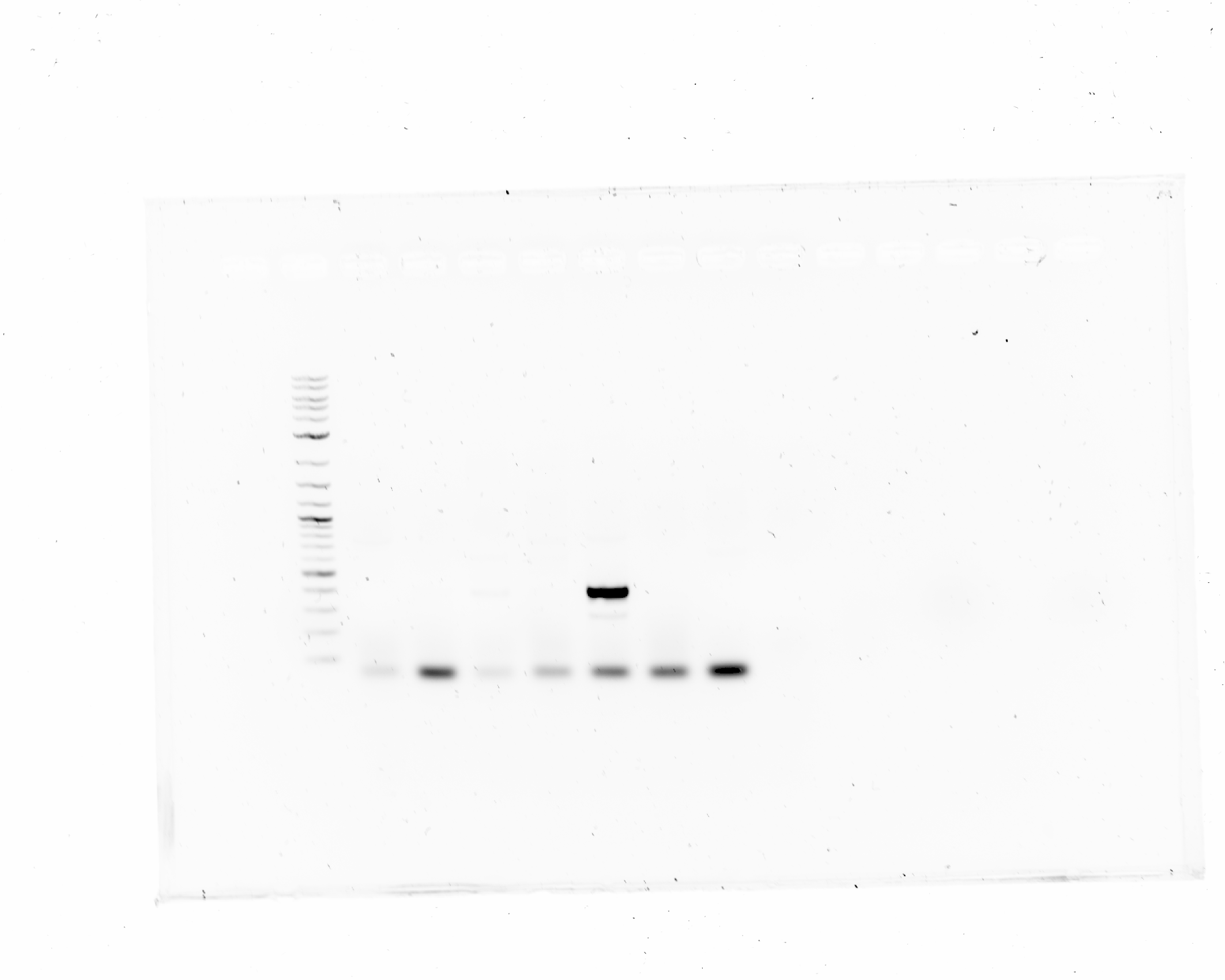

Supplement: Figure 4—figure supplement 2—source data 2. [file elife-106699-fig4-figsupp2-data2.zip › Figure 4ΓÇöfigure supplement 2-source data 2 Agarose gel raw data shows PCR products from RT-PCR analysis of the human T-ALL cells./RT-PCR LMO1(SYBR Safe).tif]

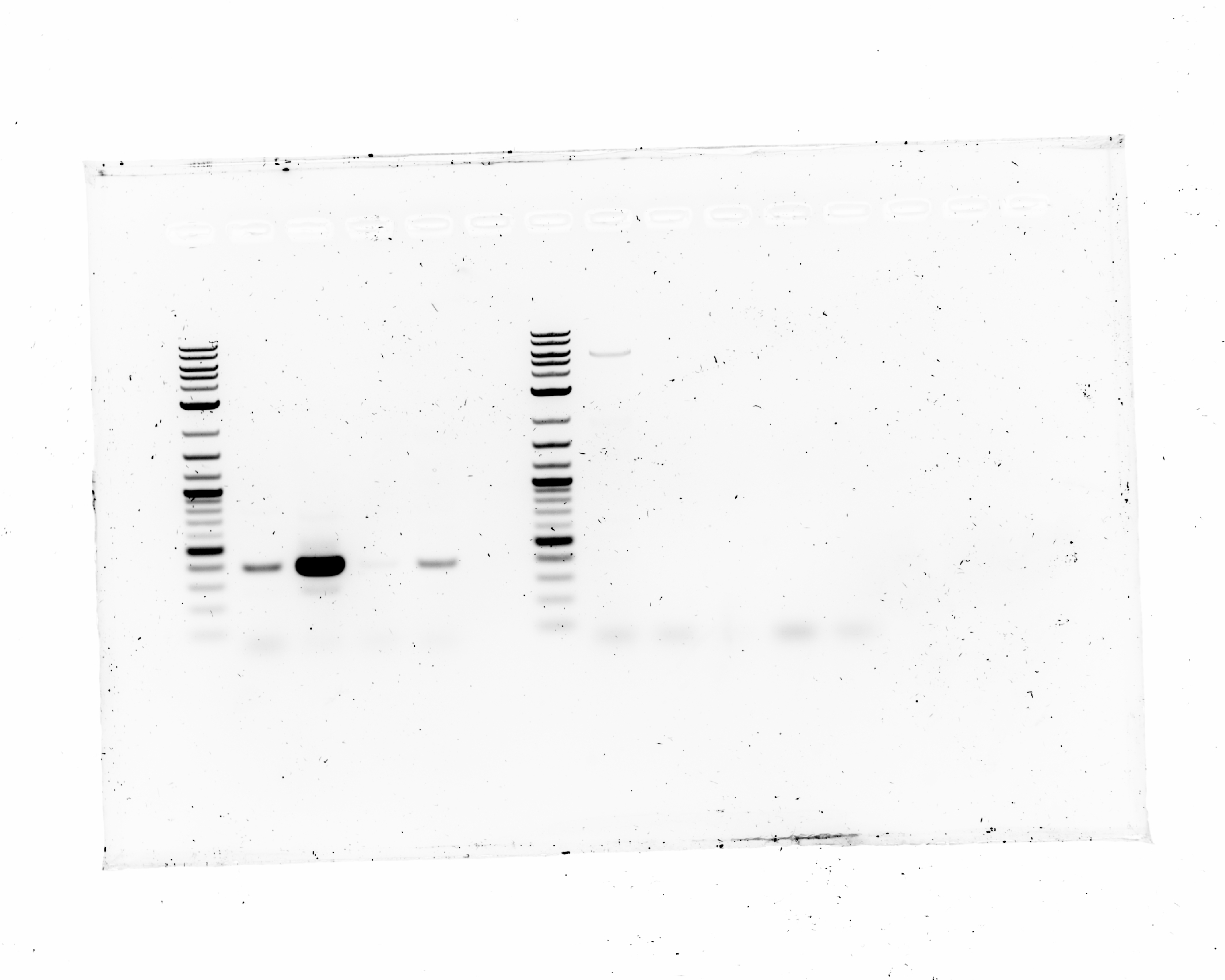

Supplement: Figure 4—figure supplement 2—source data 2. [file elife-106699-fig4-figsupp2-data2.zip › Figure 4ΓÇöfigure supplement 2-source data 2 Agarose gel raw data shows PCR products from RT-PCR analysis of the human T-ALL cells./RT-PCR LMO1(SYBR Safe).jpg]

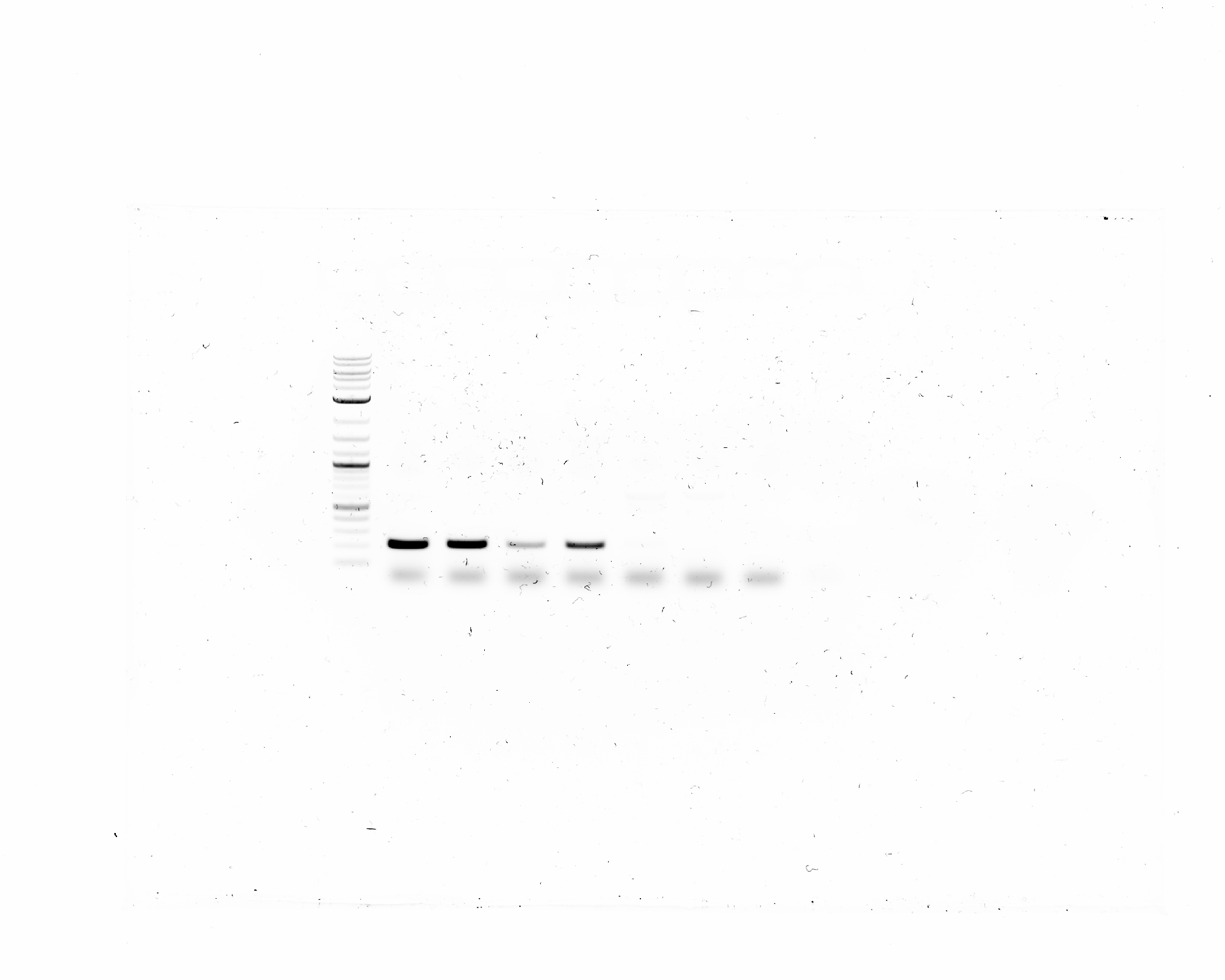

Supplement: Figure 4—figure supplement 2—source data 2. [file elife-106699-fig4-figsupp2-data2.zip › Figure 4ΓÇöfigure supplement 2-source data 2 Agarose gel raw data shows PCR products from RT-PCR analysis of the human T-ALL cells./RT-PCR LMO2(SYBR Safe).tif]

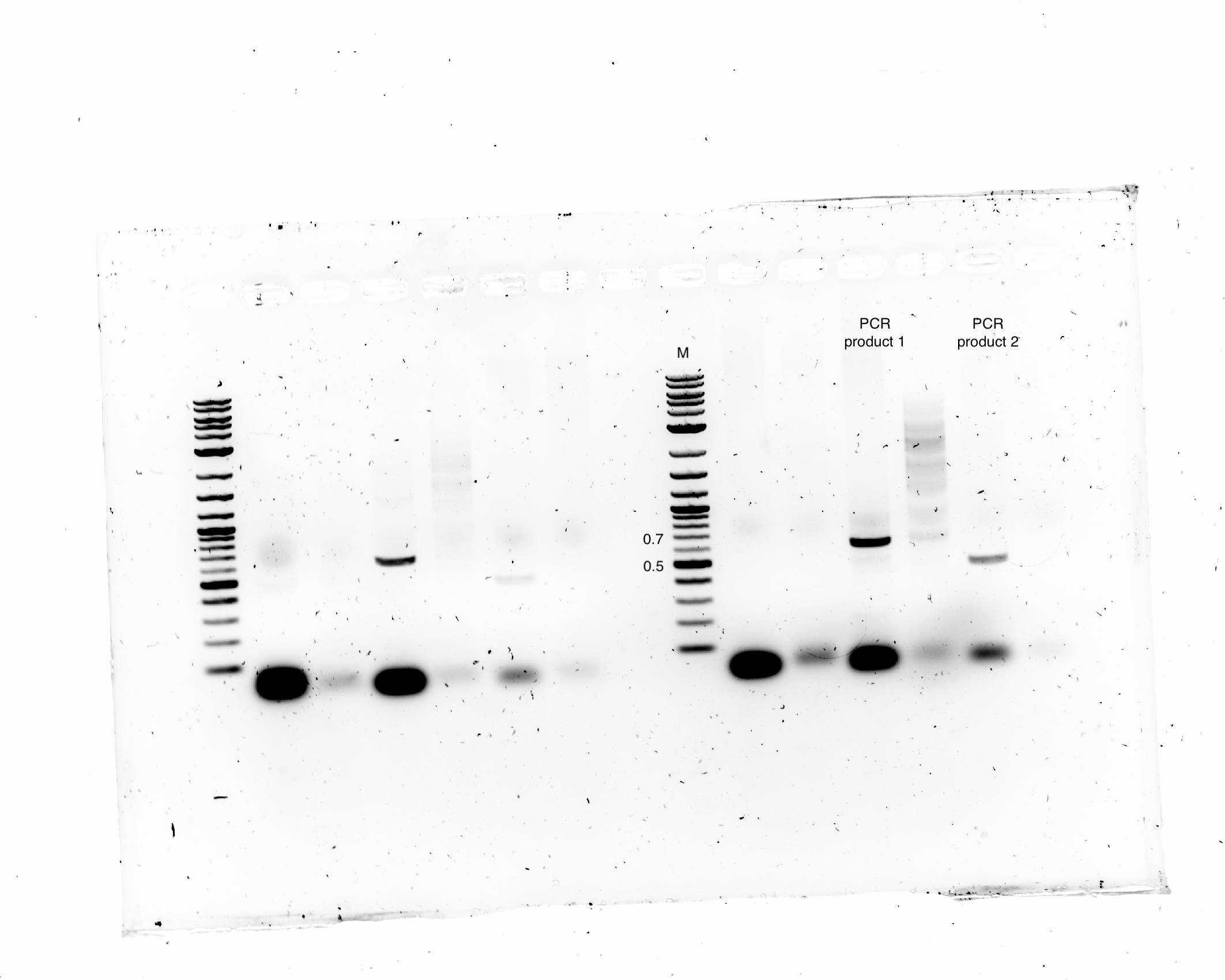

Supplement: Figure 4—figure supplement 3—source data 1. [file elife-106699-fig4-figsupp3-data1.zip › Figure 4ΓÇöfigure supplement 3-source data 1 Agarose gel data with label shows PCR products to confirm the chromosomal translocation in KOPT-K1./gPCR KOPT-K1(SYBR Safe).tif]

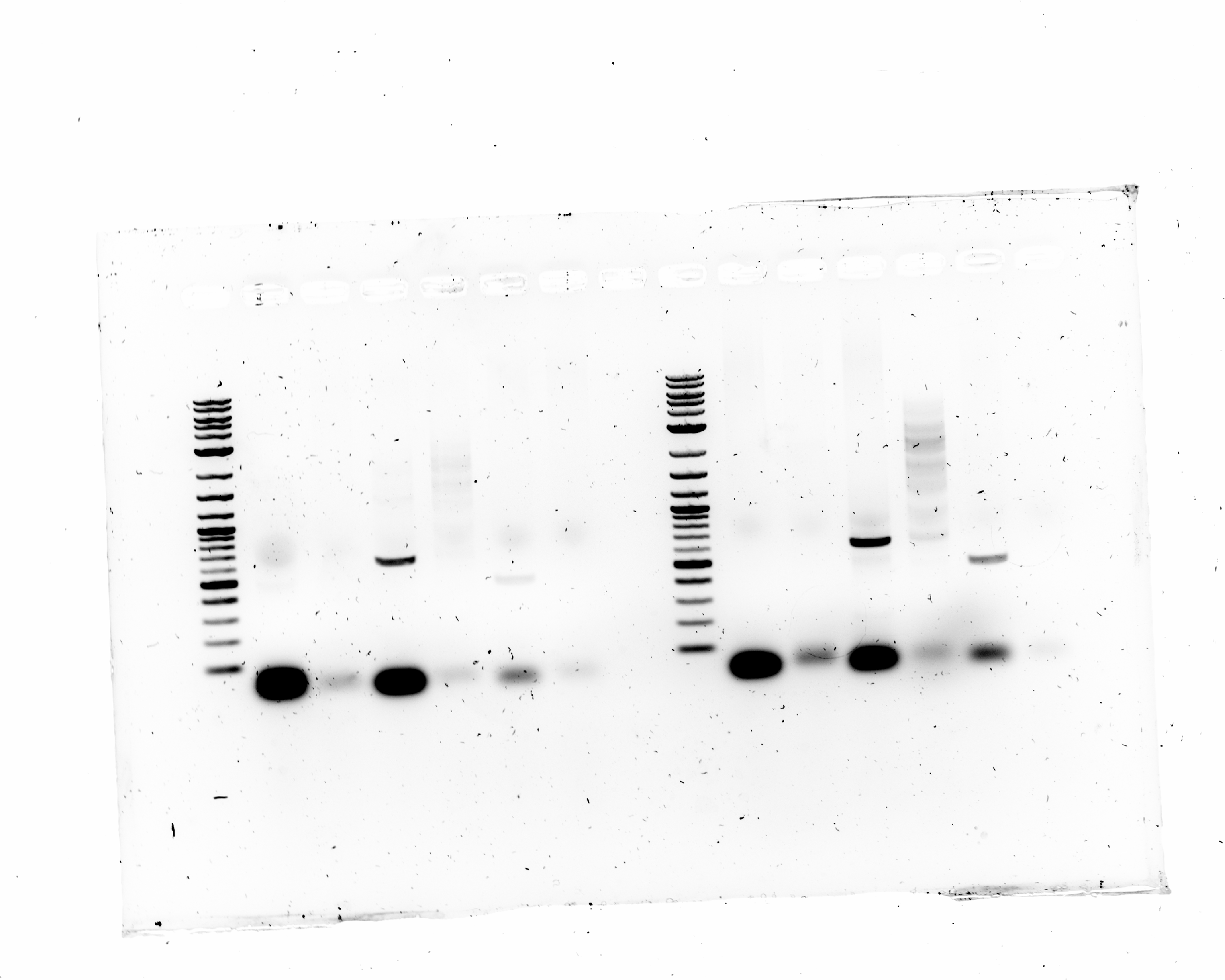

Supplement: Figure 4—figure supplement 3—source data 2. [file elife-106699-fig4-figsupp3-data2.zip › Figure 4ΓÇöfigure supplement 3-source data 2 Agarose gel raw data shows PCR products to confirm the chromosomal translocation in KOPT-K1./gPCR KOPT-K1(SYBR Safe).tif]

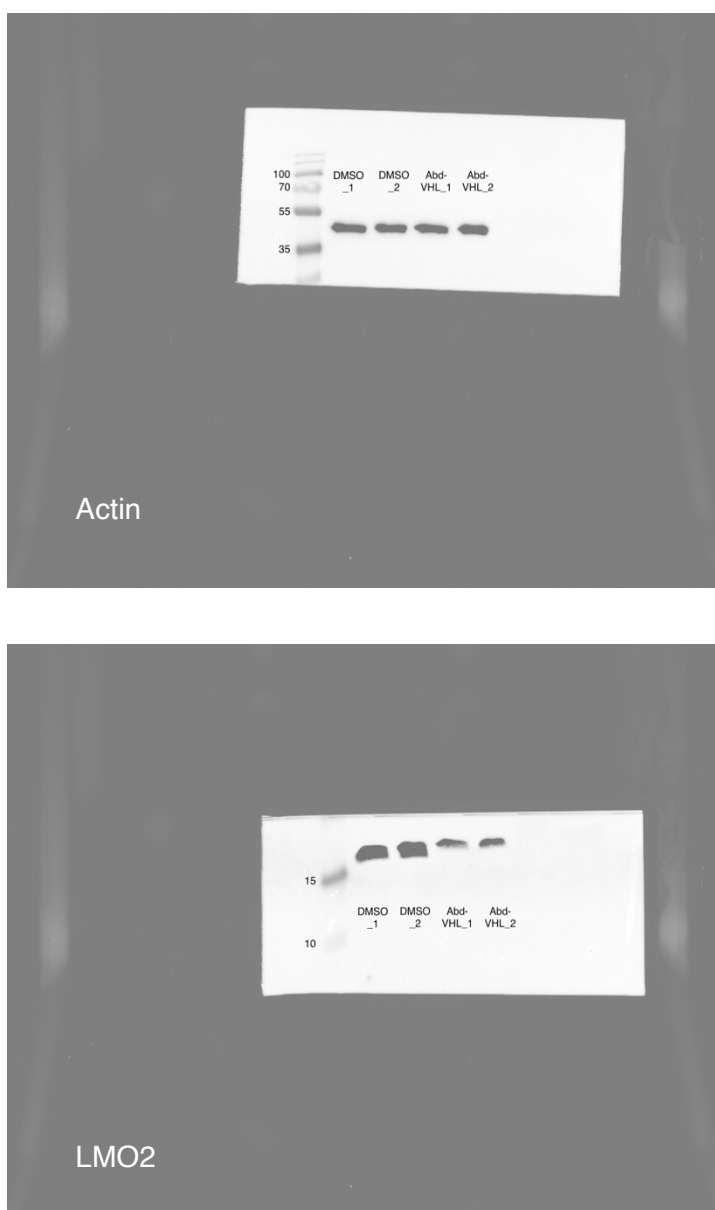

**Figure 5, Source Data 1.** Original membranes corresponding to Figure 5, panel B.

Supplement: Figure 5—source data 1. [file elife-106699-fig5-data1.zip › Figure 5ΓÇösource data 1 PDF files containing original western blots for Figure 5B, indicating the relevant bands and treatments./Figure 5-source data 1.pdf]

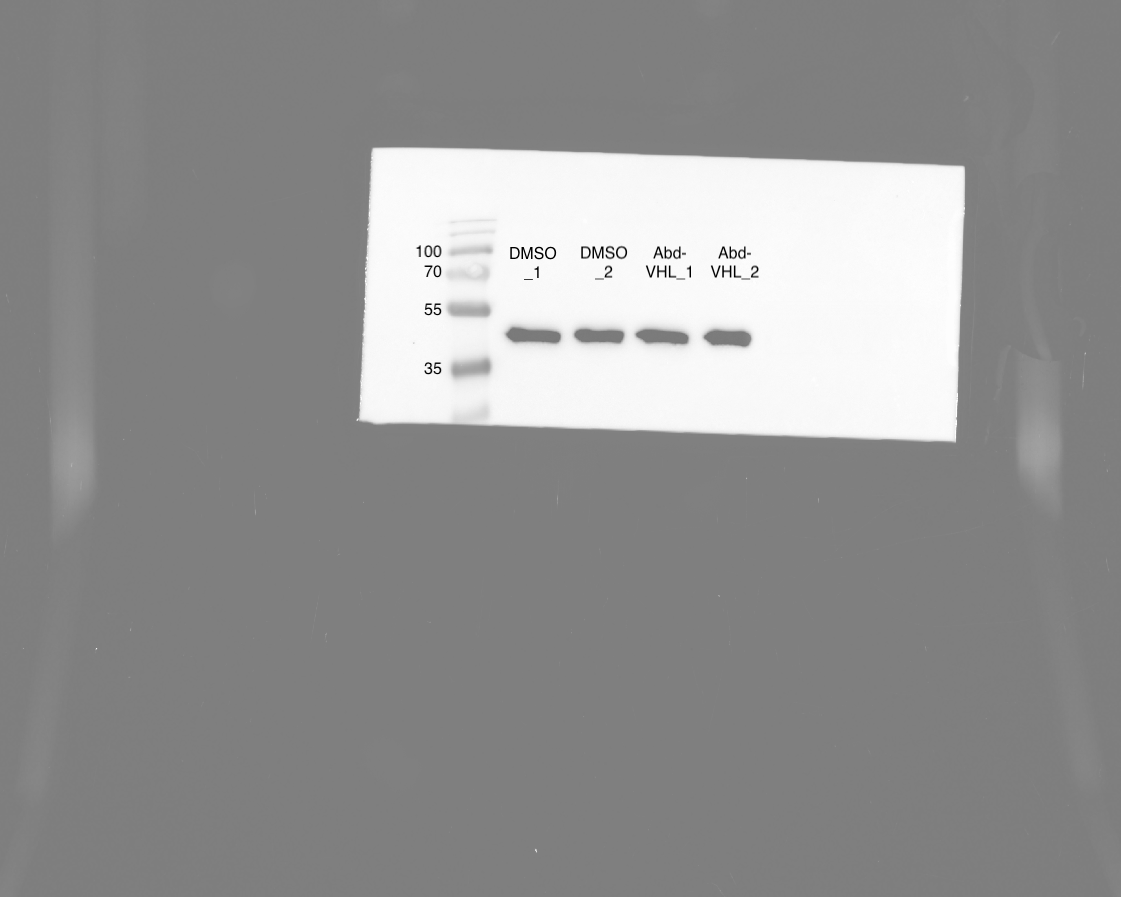

Supplement: Figure 5—source data 1. [file elife-106699-fig5-data1.zip › Figure 5ΓÇösource data 1 PDF files containing original western blots for Figure 5B, indicating the relevant bands and treatments./Raw data/Actin.tif]

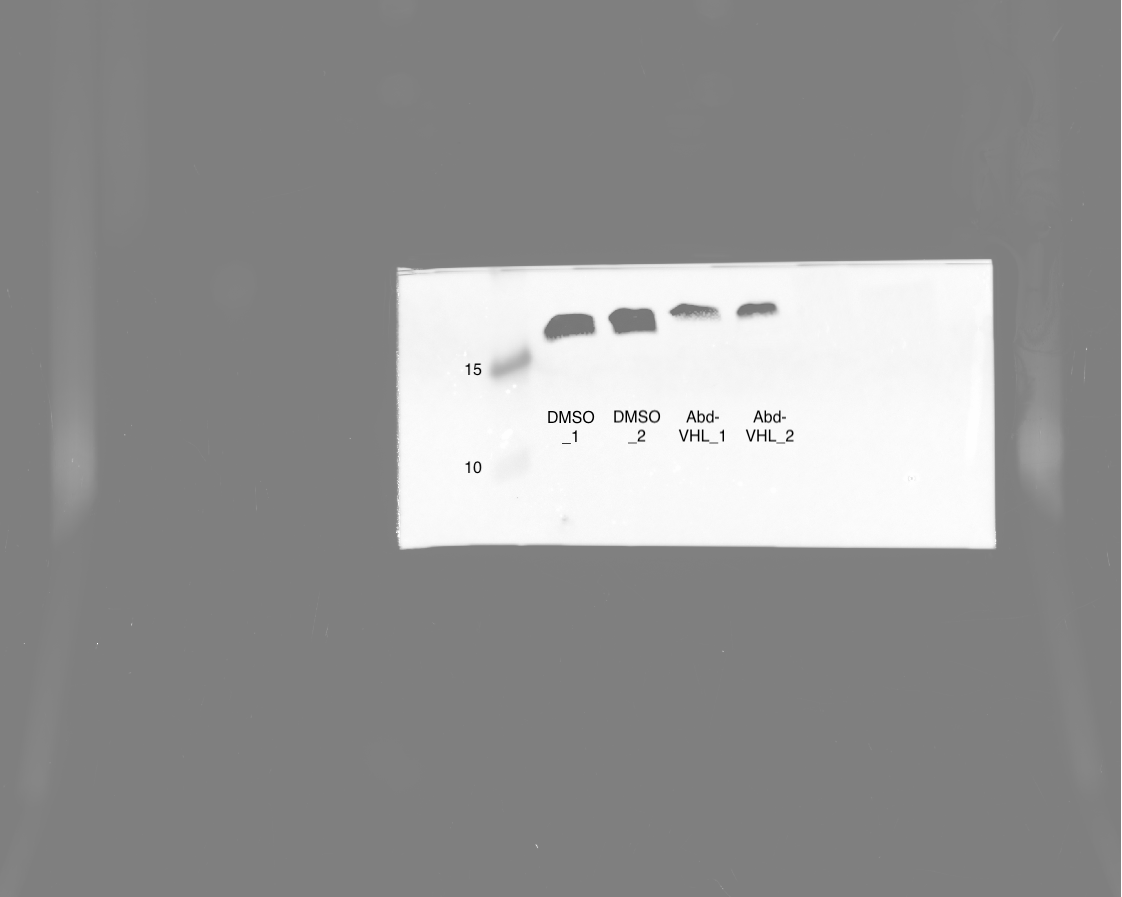

Supplement: Figure 5—source data 1. [file elife-106699-fig5-data1.zip › Figure 5ΓÇösource data 1 PDF files containing original western blots for Figure 5B, indicating the relevant bands and treatments./Raw data/LMO2.tif]

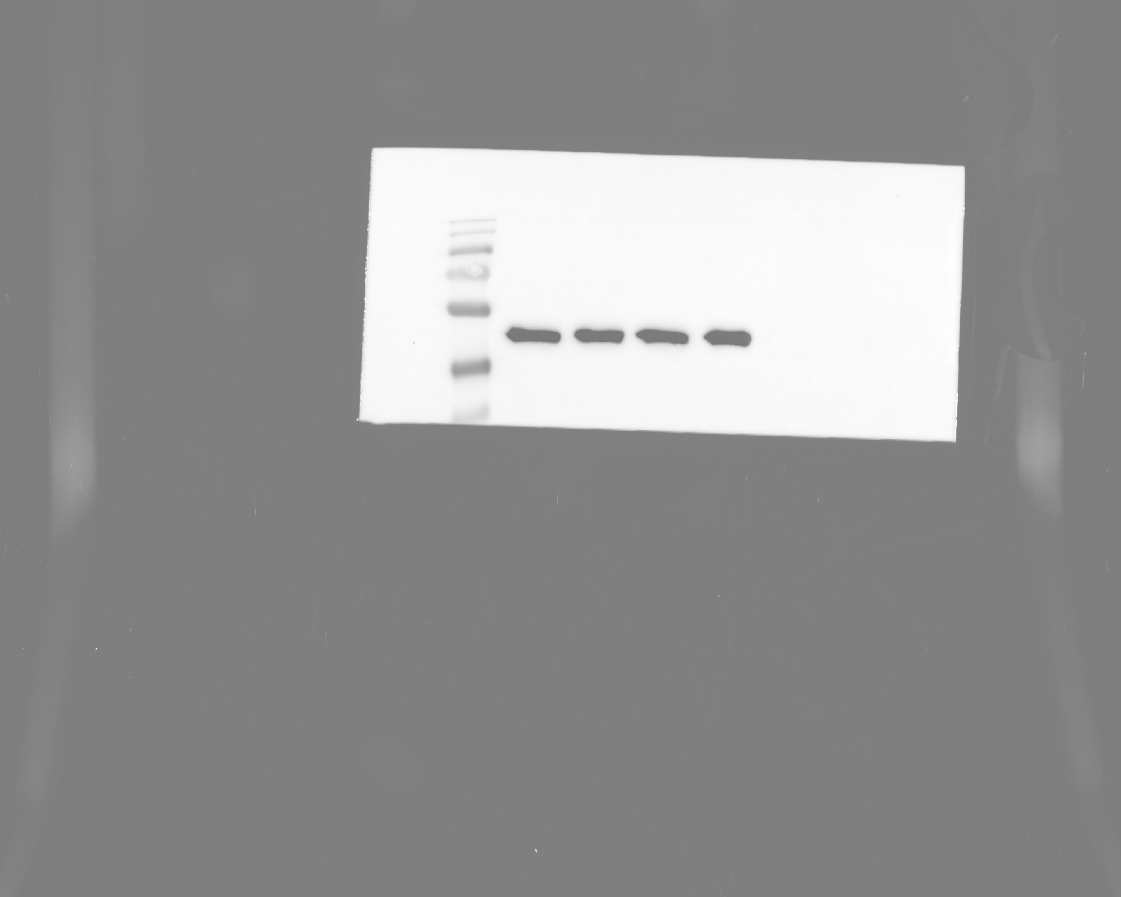

Supplement: Figure 5—source data 2. [file elife-106699-fig5-data2.zip › Figure 5ΓÇösource data 2 Original files for Western blot analysis displayed in Figure 5B./Actin.tif]

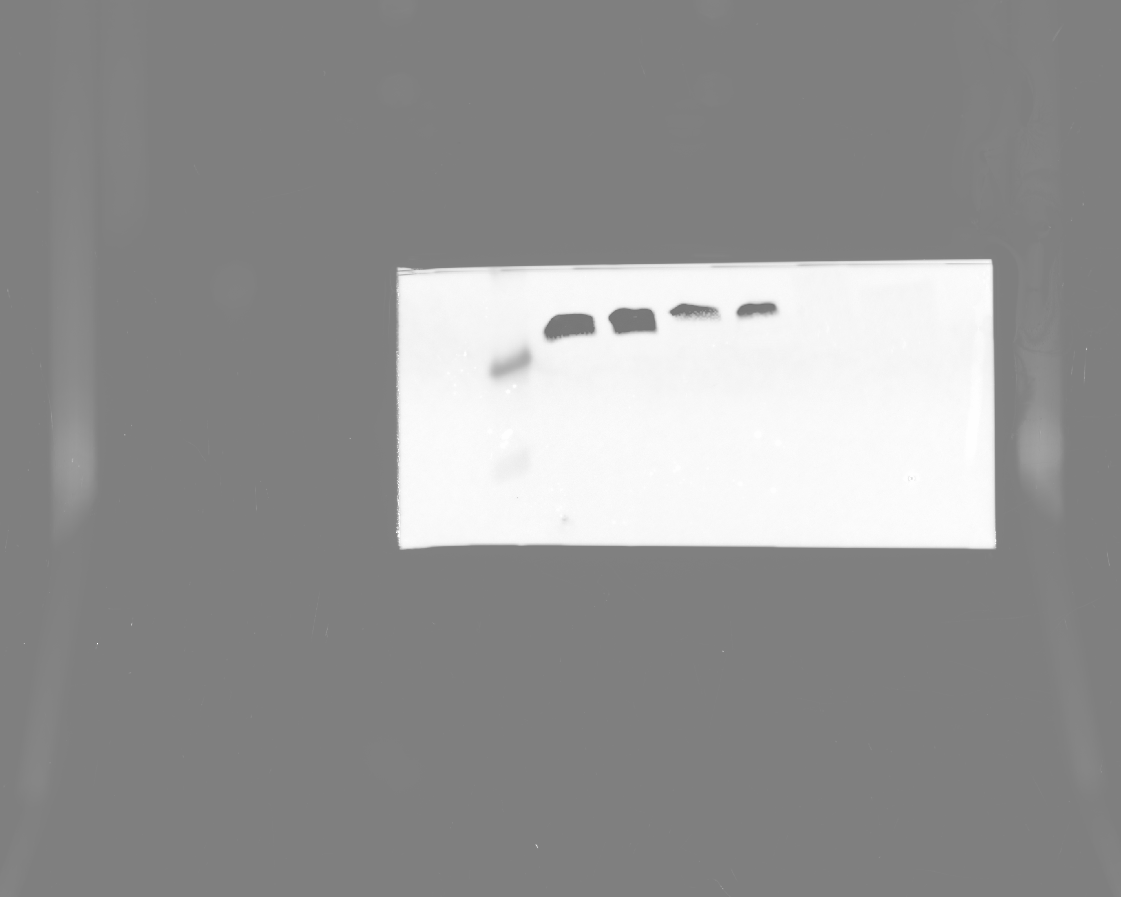

Supplement: Figure 5—source data 2. [file elife-106699-fig5-data2.zip › Figure 5ΓÇösource data 2 Original files for Western blot analysis displayed in Figure 5B./LMO2.tif]

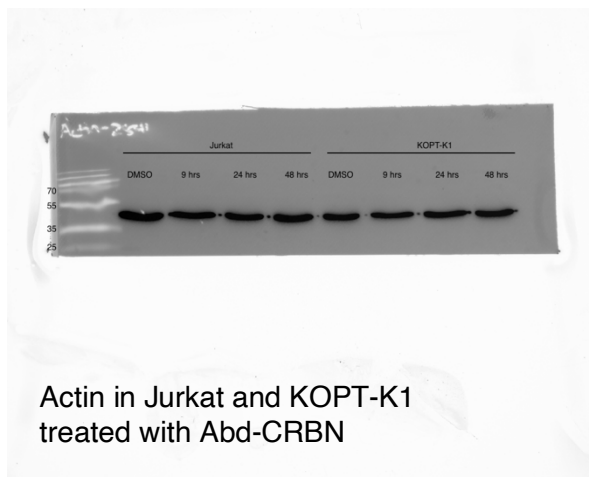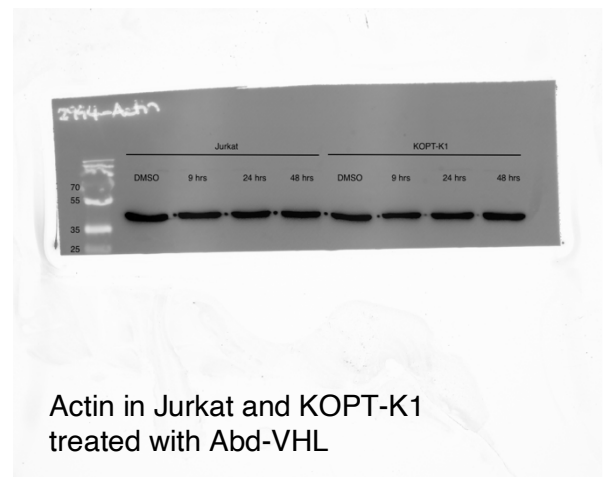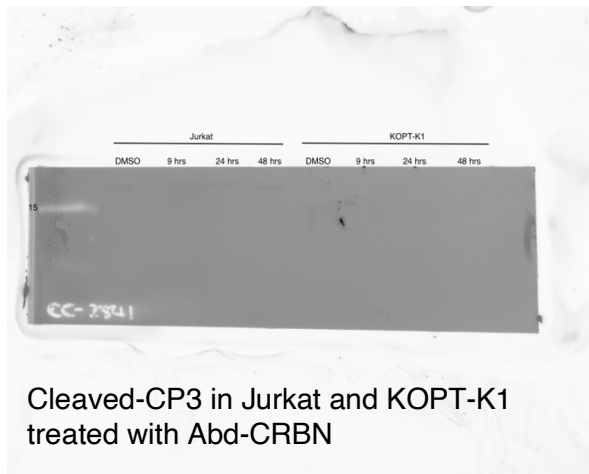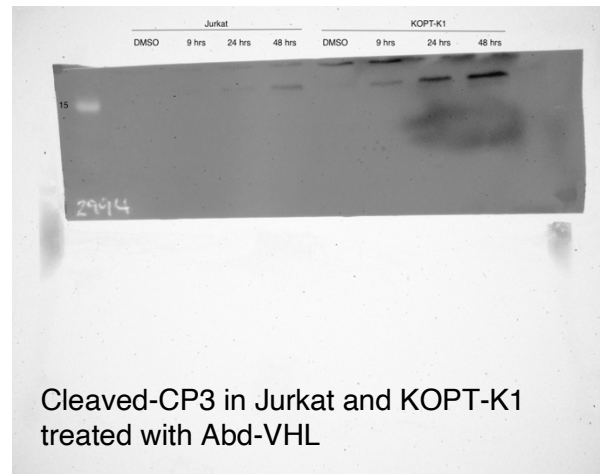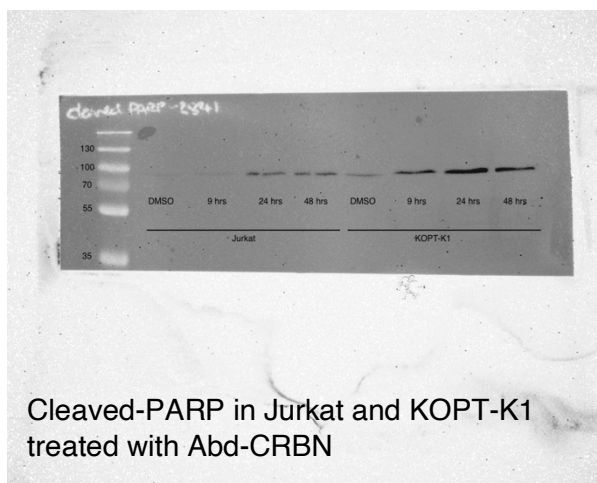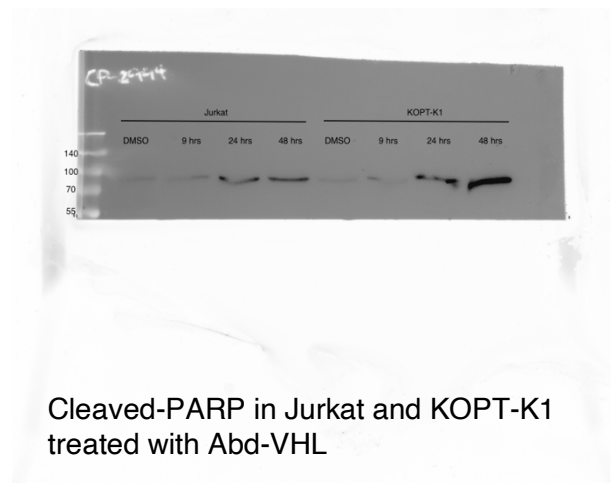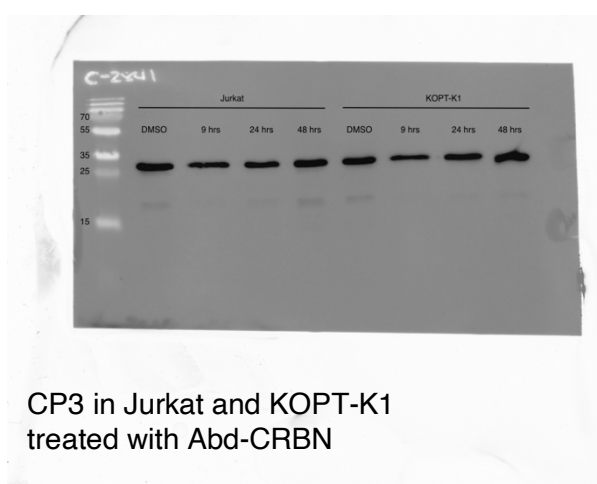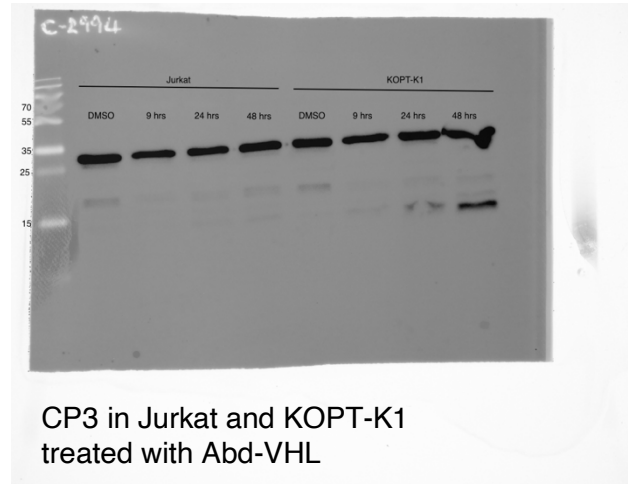

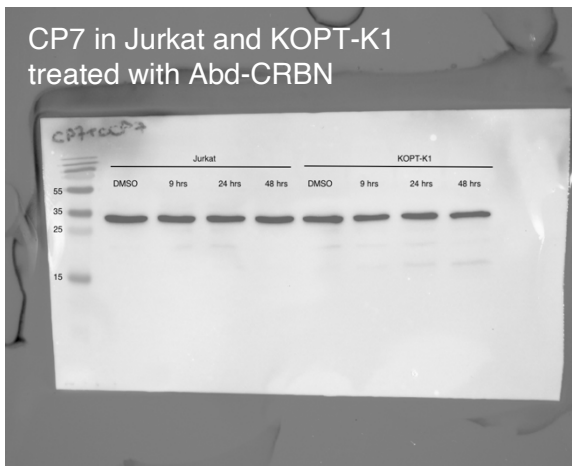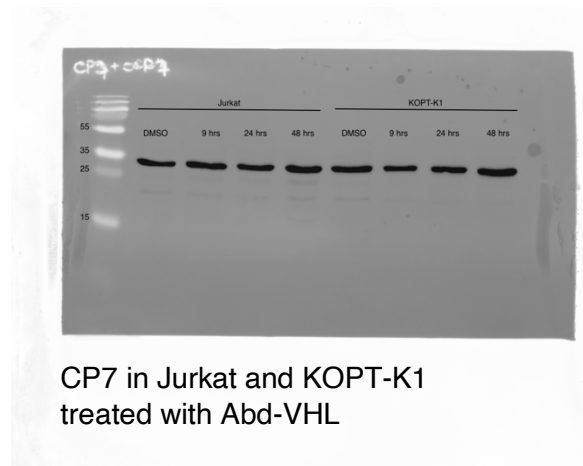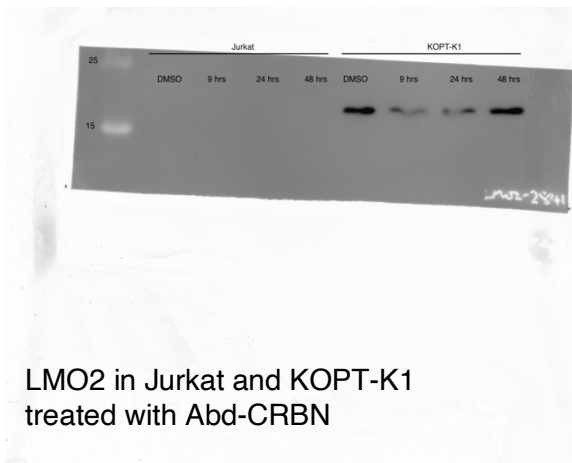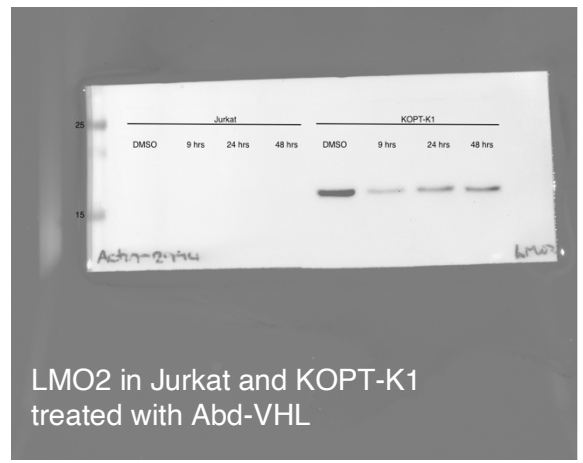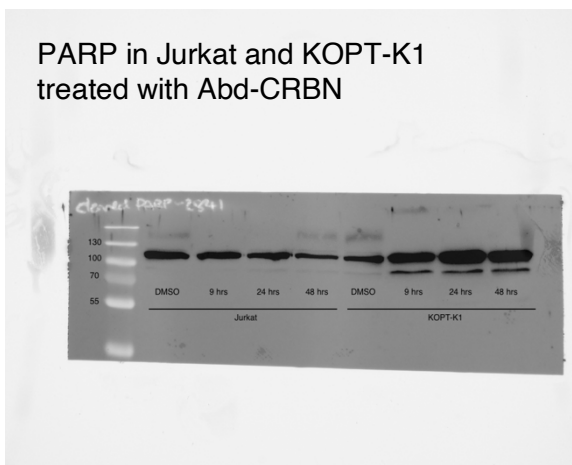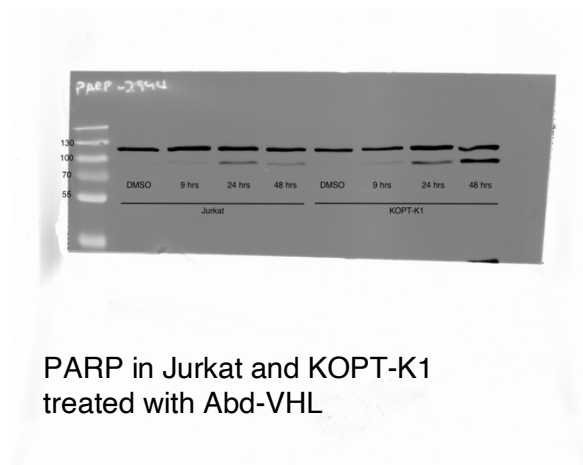

**Figure 6, Source Data 1.** Original membranes corresponding to Figure 6, panel B.

Supplement: Figure 6—source data 1. [file elife-106699-fig6-data1.zip › Figure 6ΓÇösource data 1 PDF files containing original western blots for Figure 6B, indicating the relevant bands and treatments./Figure 6-source data 1.pdf]

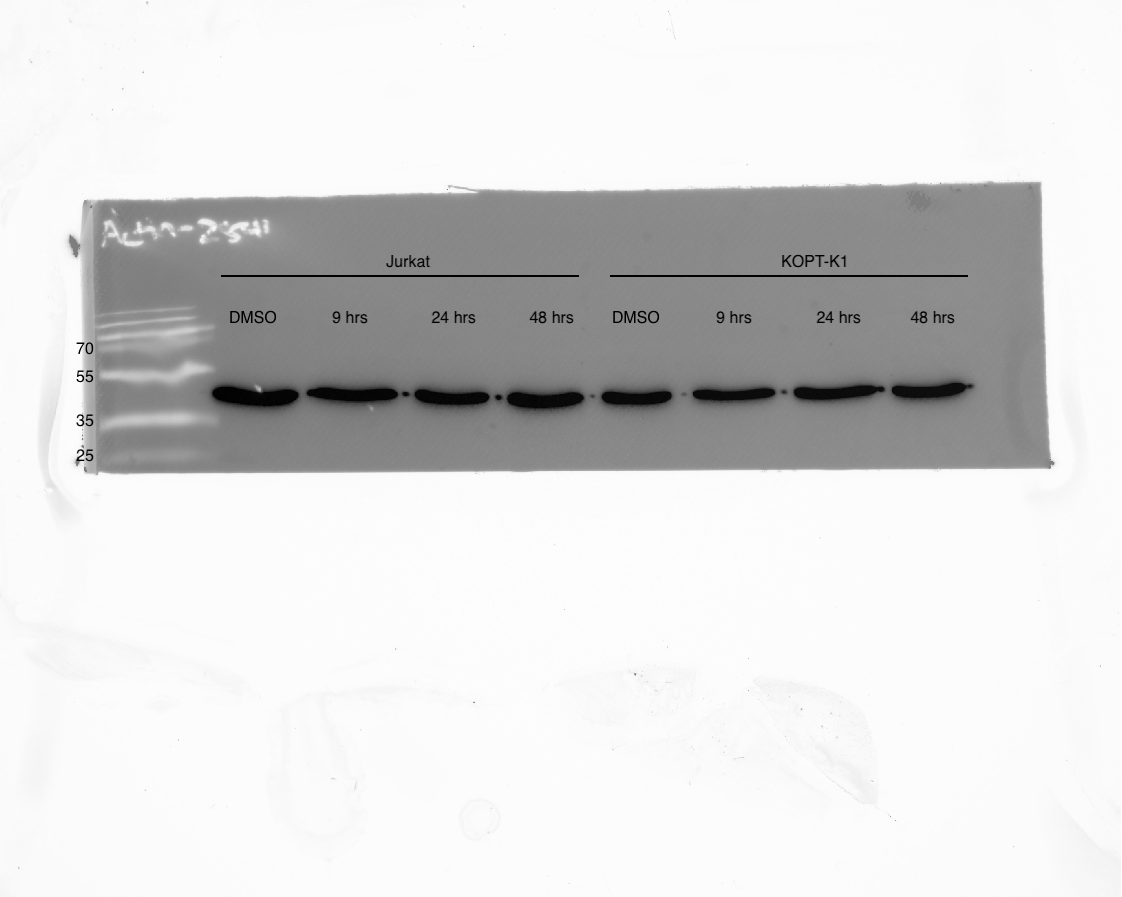

Supplement: Figure 6—source data 1. [file elife-106699-fig6-data1.zip › Figure 6ΓÇösource data 1 PDF files containing original western blots for Figure 6B, indicating the relevant bands and treatments./Raw data/Actin Abd-CRBN.tif]

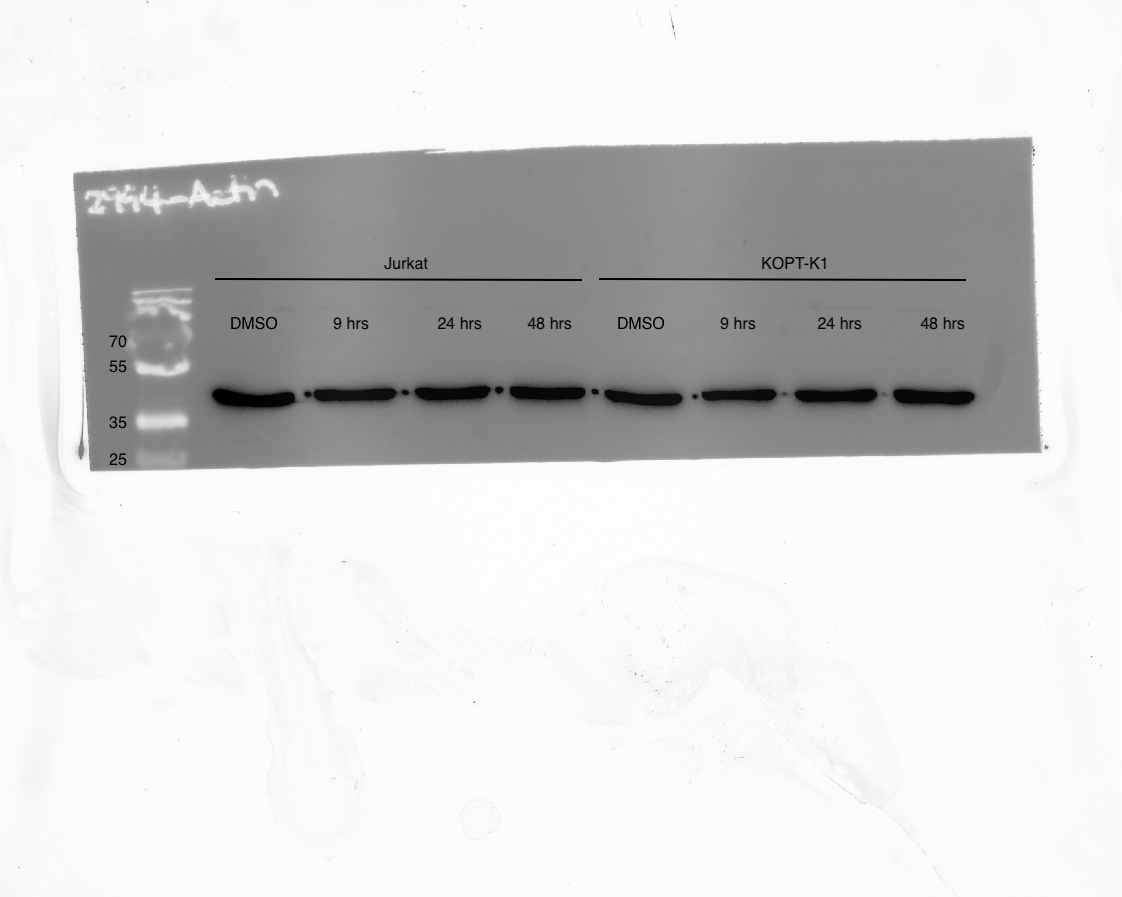

Supplement: Figure 6—source data 1. [file elife-106699-fig6-data1.zip › Figure 6ΓÇösource data 1 PDF files containing original western blots for Figure 6B, indicating the relevant bands and treatments./Raw data/Actin Abd-VHL.tif]

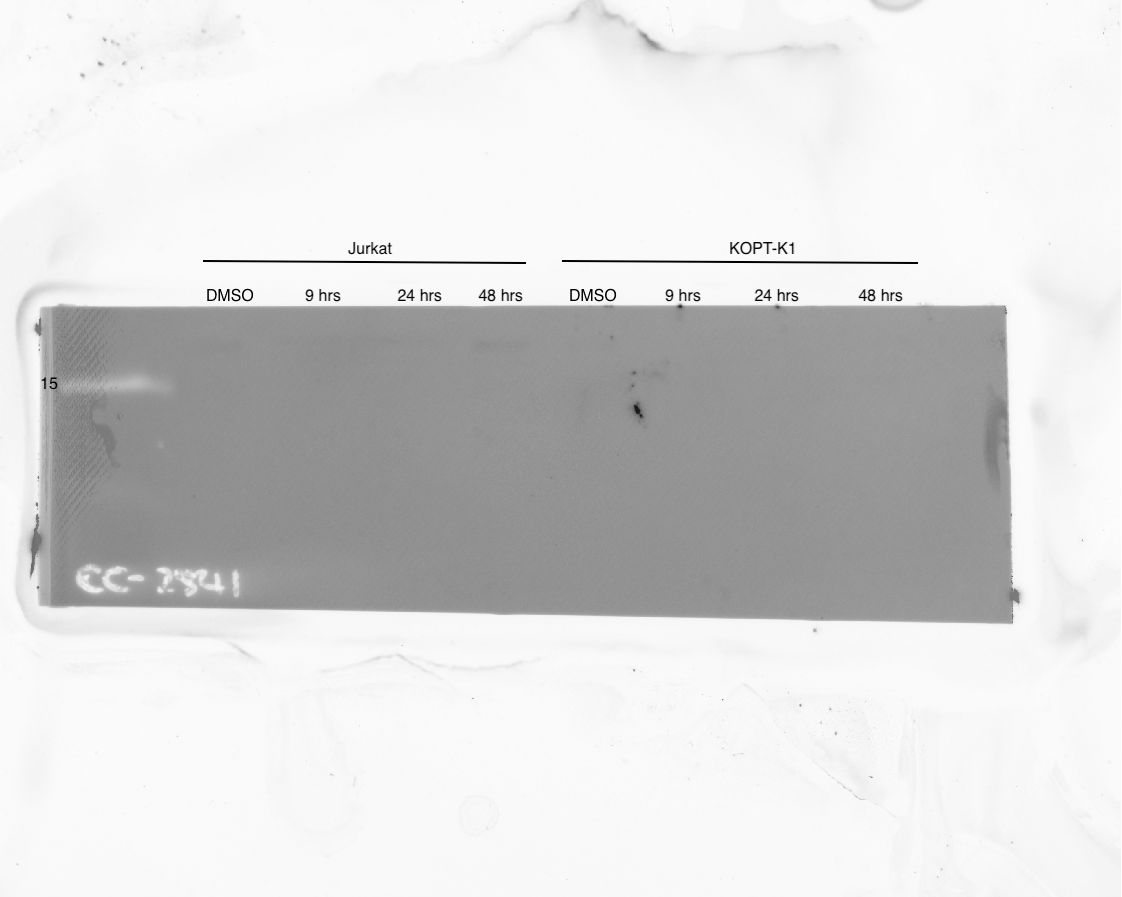

Supplement: Figure 6—source data 1. [file elife-106699-fig6-data1.zip › Figure 6ΓÇösource data 1 PDF files containing original western blots for Figure 6B, indicating the relevant bands and treatments./Raw data/Cleaved CP3 Abd-CRBN.tif]

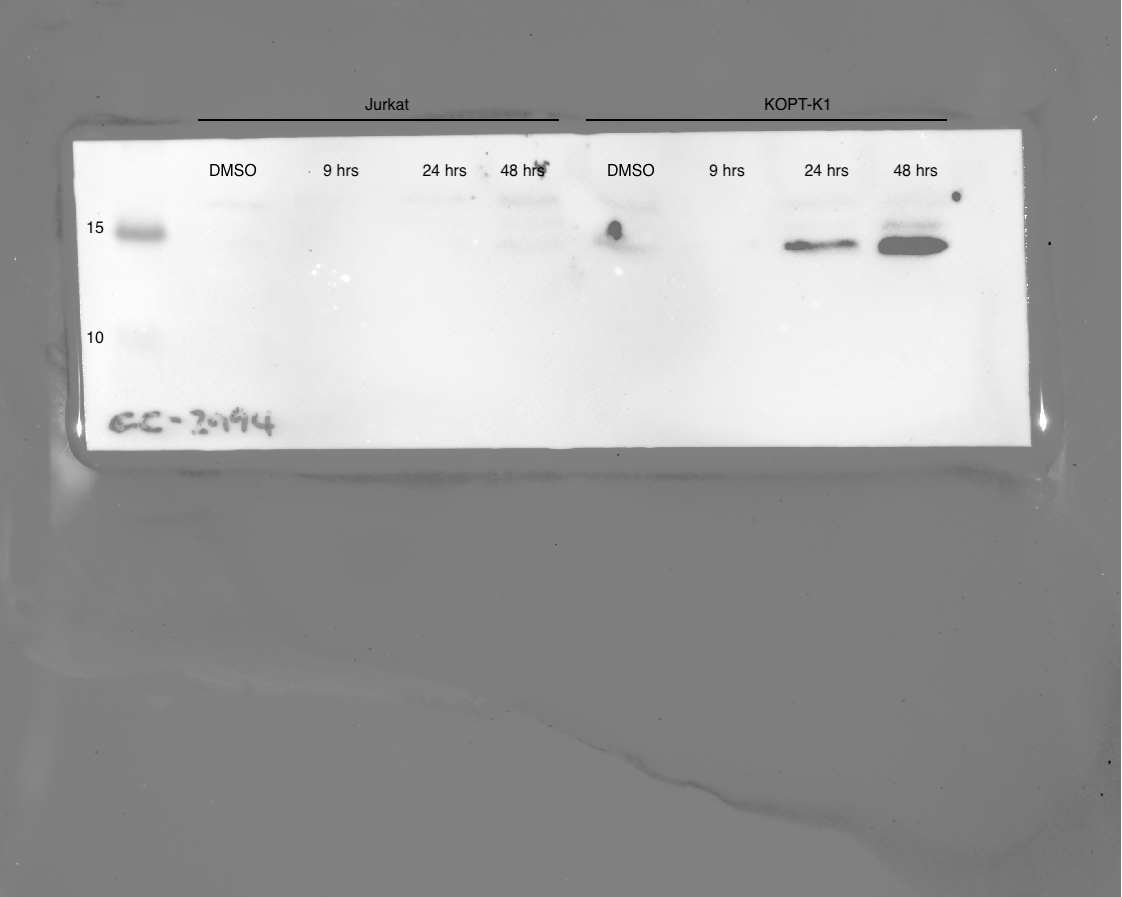

Supplement: Figure 6—source data 1. [file elife-106699-fig6-data1.zip › Figure 6ΓÇösource data 1 PDF files containing original western blots for Figure 6B, indicating the relevant bands and treatments./Raw data/Cleaved CP3 Abd-VHL.tif]

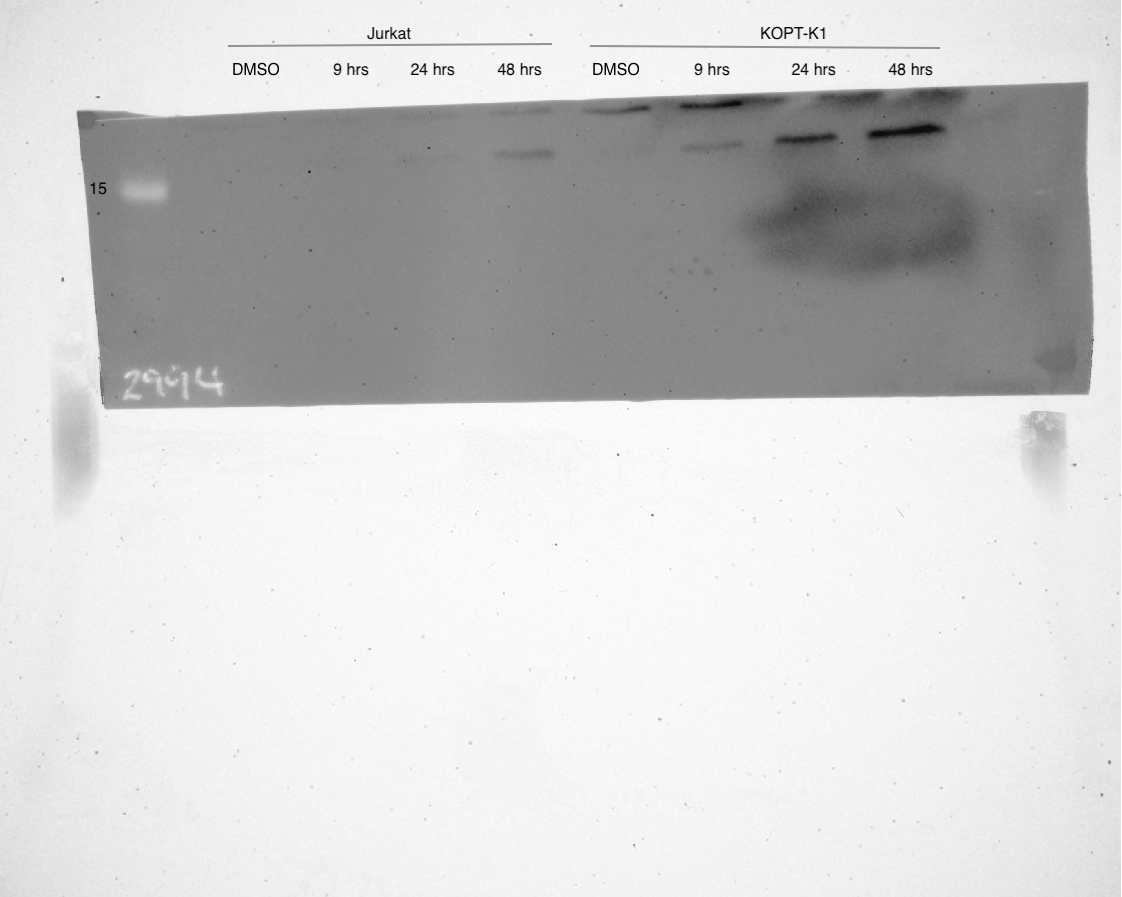

Supplement: Figure 6—source data 1. [file elife-106699-fig6-data1.zip › Figure 6ΓÇösource data 1 PDF files containing original western blots for Figure 6B, indicating the relevant bands and treatments./Raw data/Cleaved CP7 Abd-CRBN.tif]

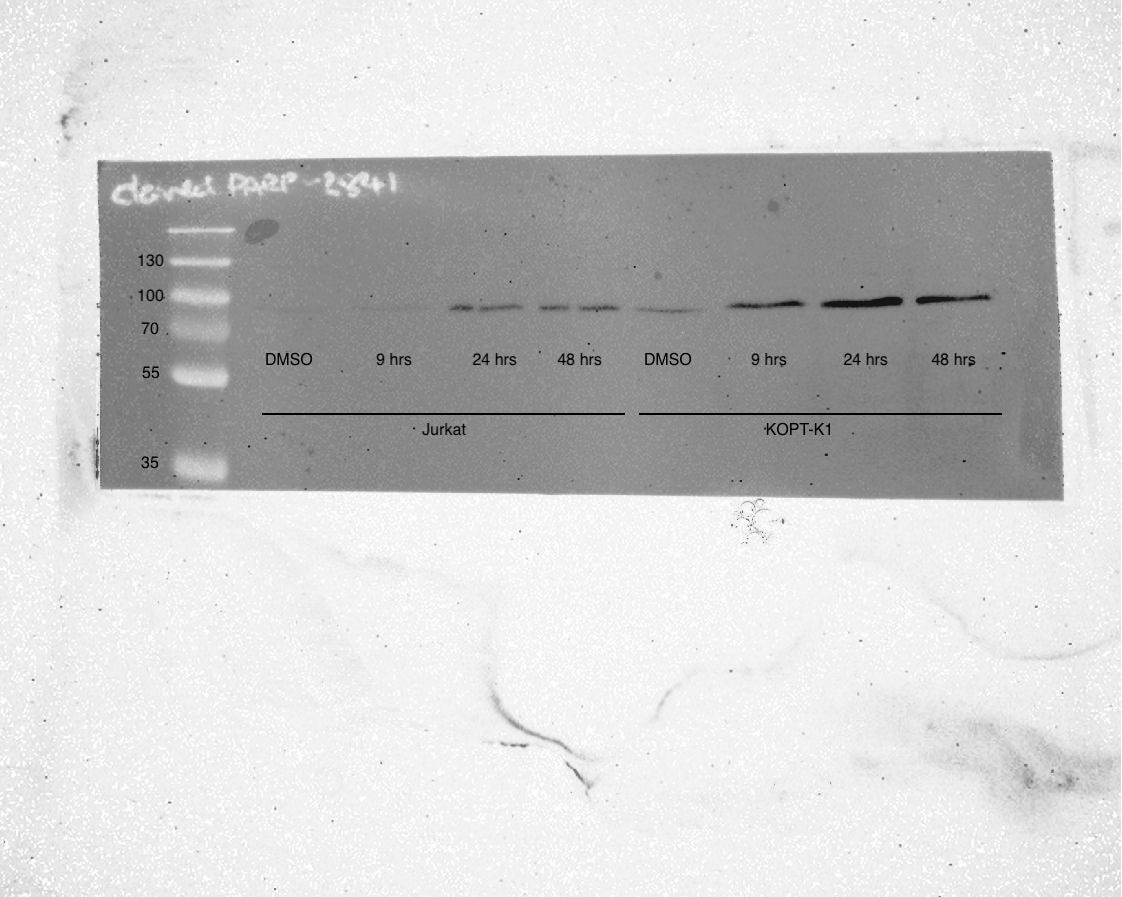

Supplement: Figure 6—source data 1. [file elife-106699-fig6-data1.zip › Figure 6ΓÇösource data 1 PDF files containing original western blots for Figure 6B, indicating the relevant bands and treatments./Raw data/Cleaved PARP Abd-CRBN.tif]

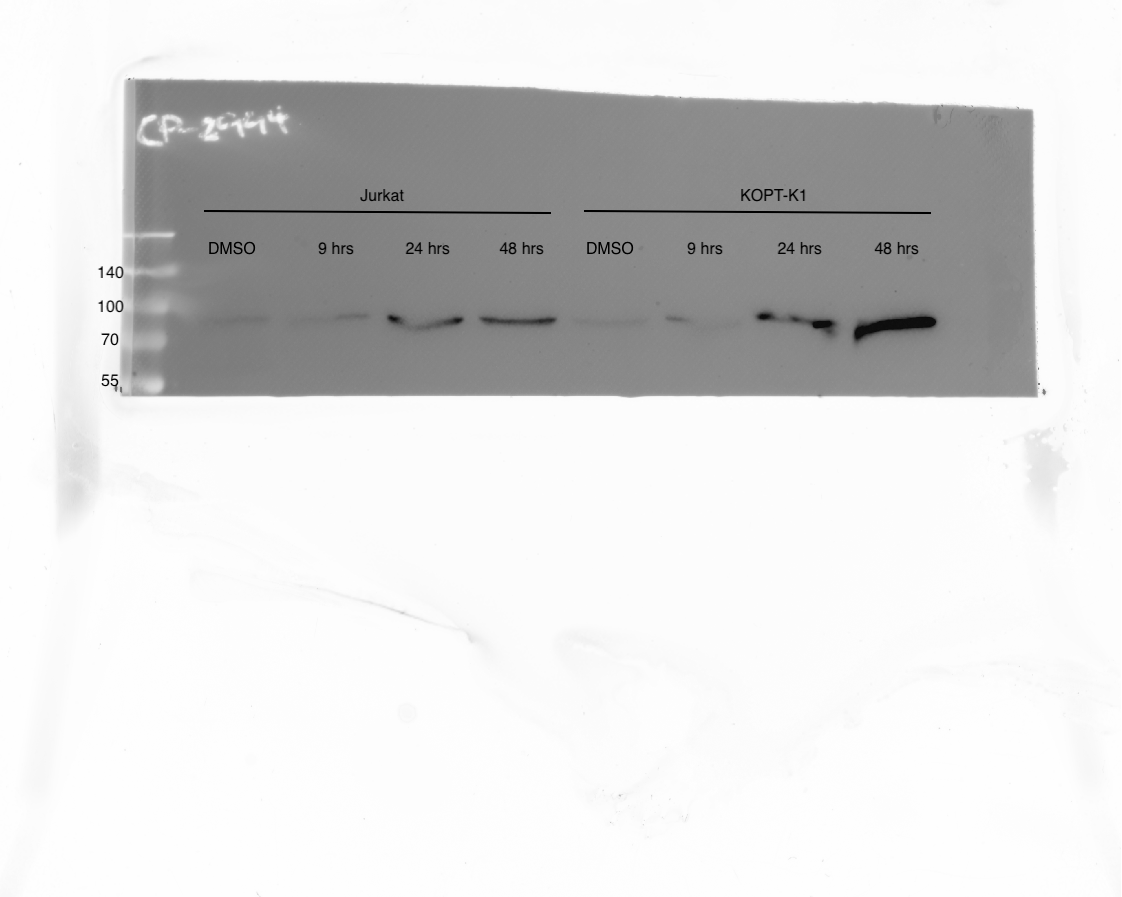

Supplement: Figure 6—source data 1. [file elife-106699-fig6-data1.zip › Figure 6ΓÇösource data 1 PDF files containing original western blots for Figure 6B, indicating the relevant bands and treatments./Raw data/Cleaved PARP Abd-VHL.tif]

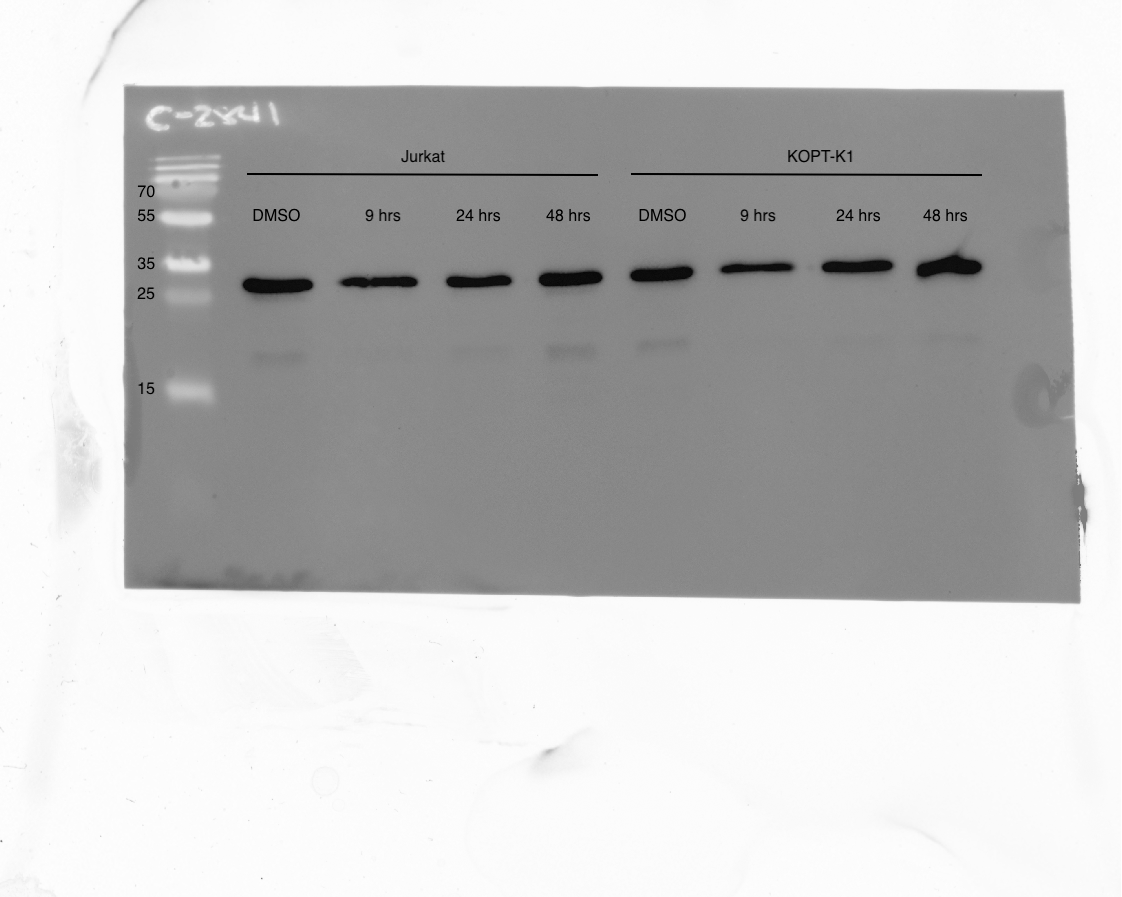

Supplement: Figure 6—source data 1. [file elife-106699-fig6-data1.zip › Figure 6ΓÇösource data 1 PDF files containing original western blots for Figure 6B, indicating the relevant bands and treatments./Raw data/CP3 Abd-CRBN.tif]

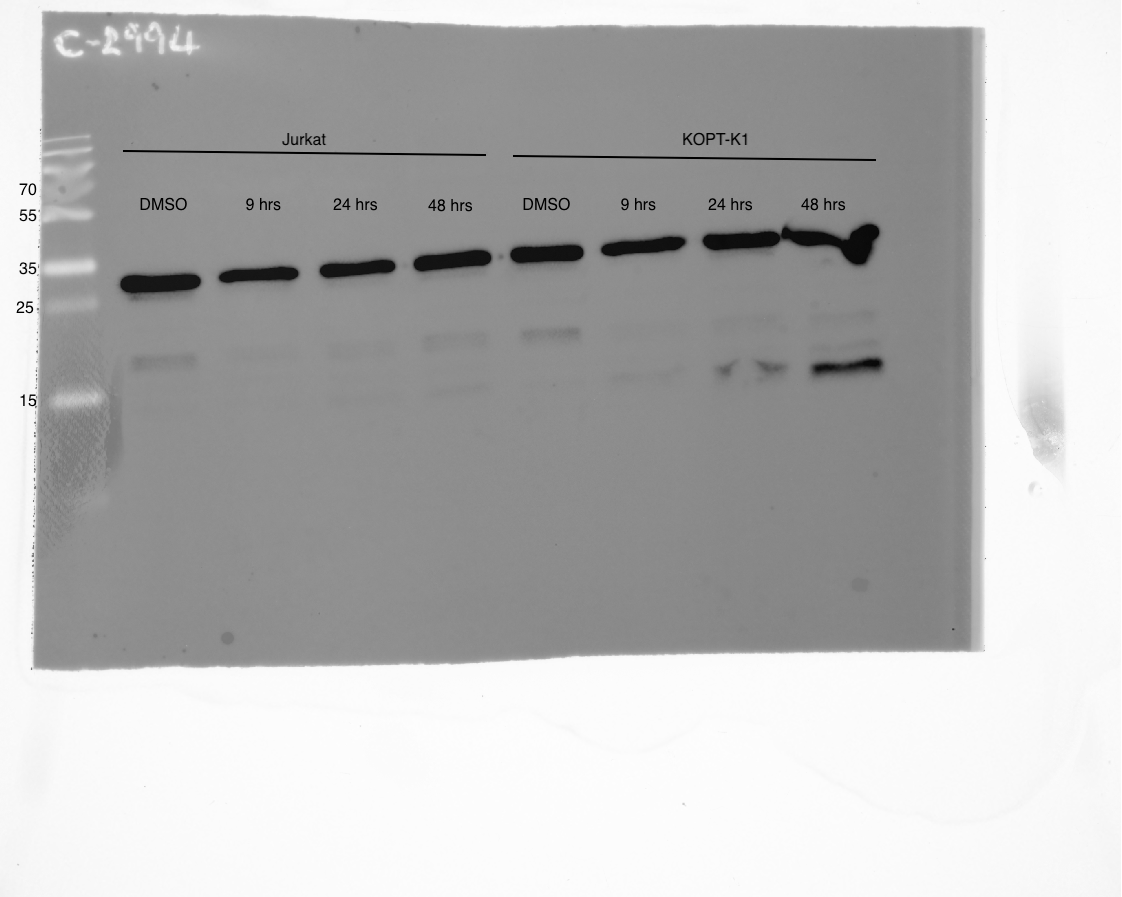

Supplement: Figure 6—source data 1. [file elife-106699-fig6-data1.zip › Figure 6ΓÇösource data 1 PDF files containing original western blots for Figure 6B, indicating the relevant bands and treatments./Raw data/CP3 Abd-VHL.tif]

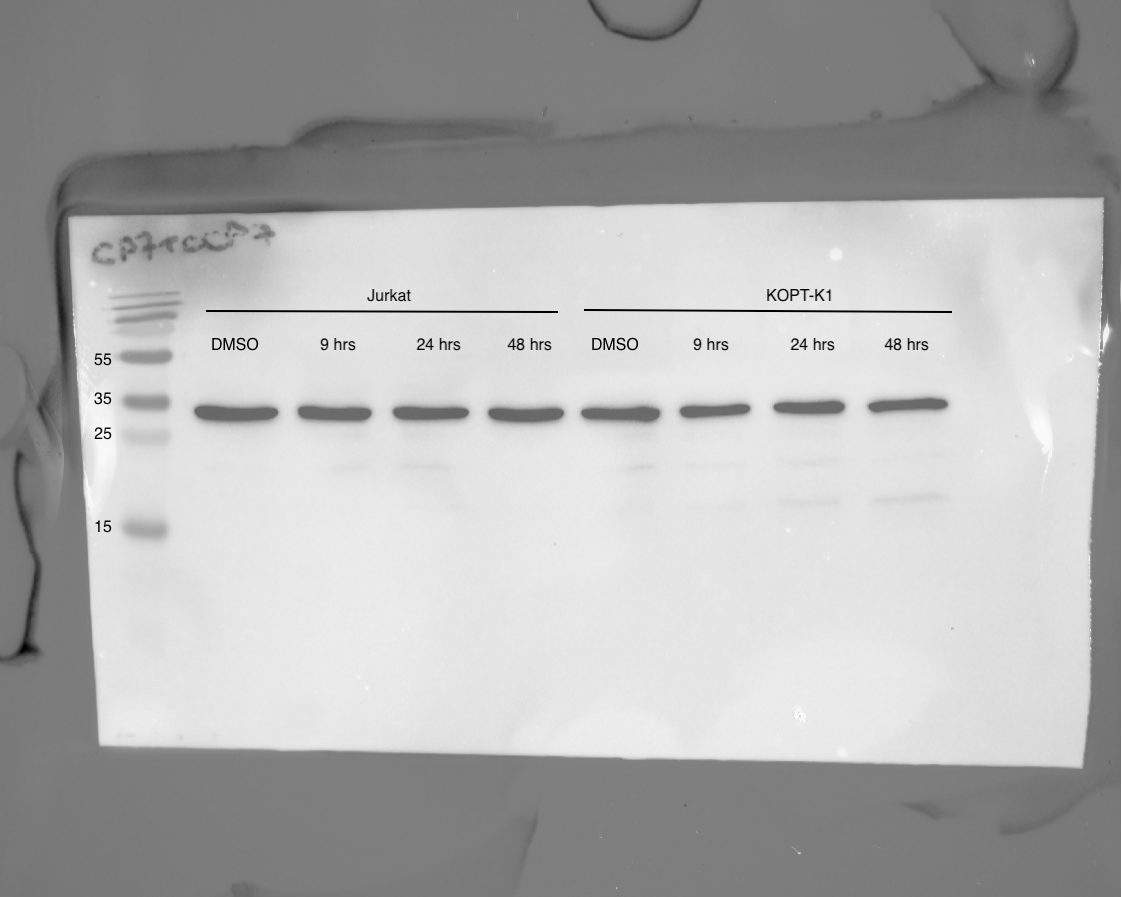

Supplement: Figure 6—source data 1. [file elife-106699-fig6-data1.zip › Figure 6ΓÇösource data 1 PDF files containing original western blots for Figure 6B, indicating the relevant bands and treatments./Raw data/CP7 Abd-CRBN.tif]

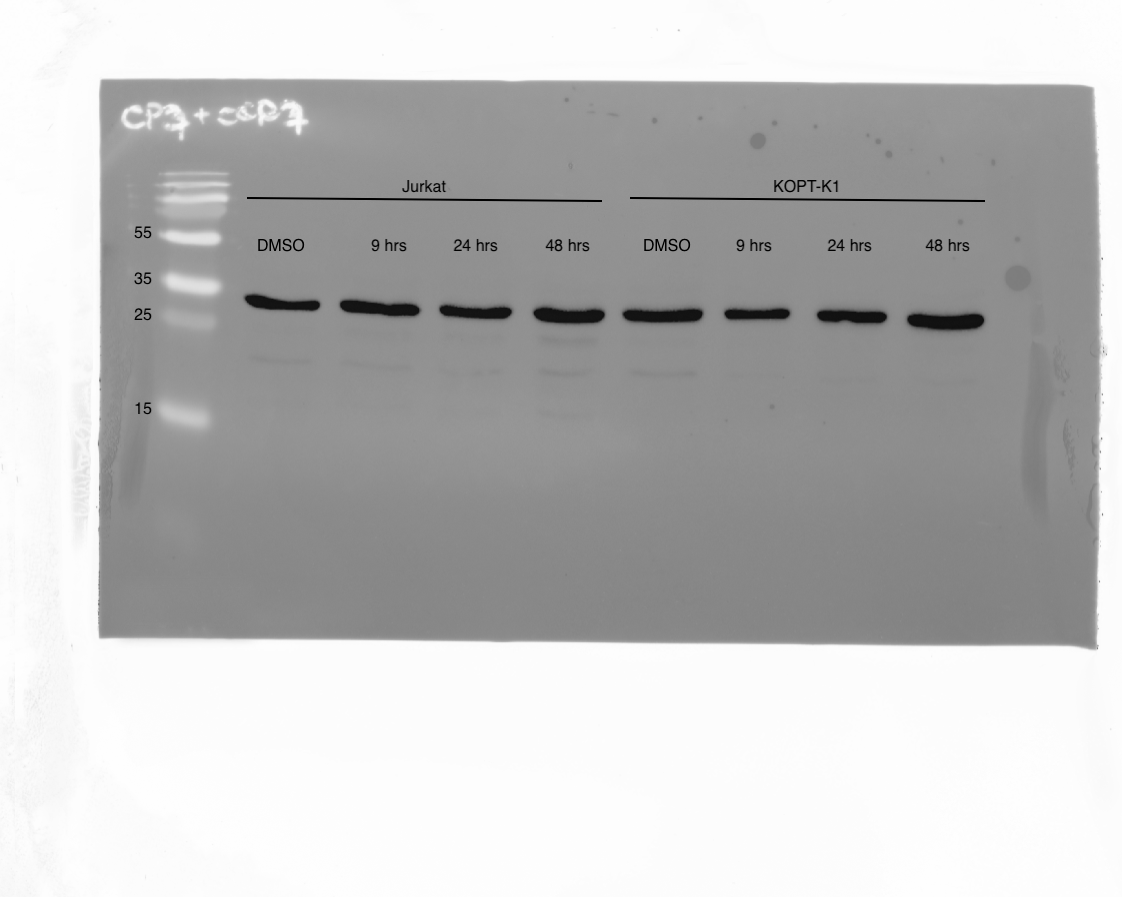

Supplement: Figure 6—source data 1. [file elife-106699-fig6-data1.zip › Figure 6ΓÇösource data 1 PDF files containing original western blots for Figure 6B, indicating the relevant bands and treatments./Raw data/CP7 Abd-VHL.tif]

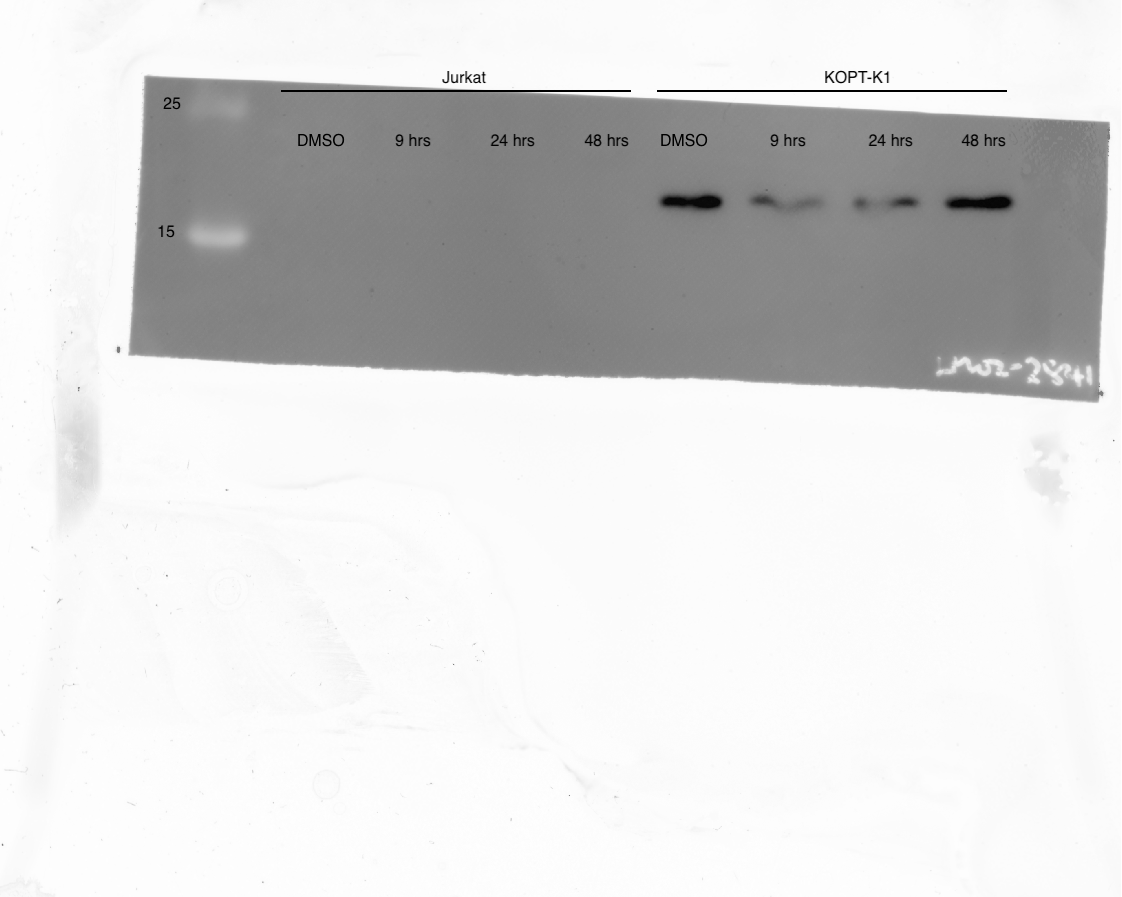

Supplement: Figure 6—source data 1. [file elife-106699-fig6-data1.zip › Figure 6ΓÇösource data 1 PDF files containing original western blots for Figure 6B, indicating the relevant bands and treatments./Raw data/LMO2 Abd-CRBN.tif]

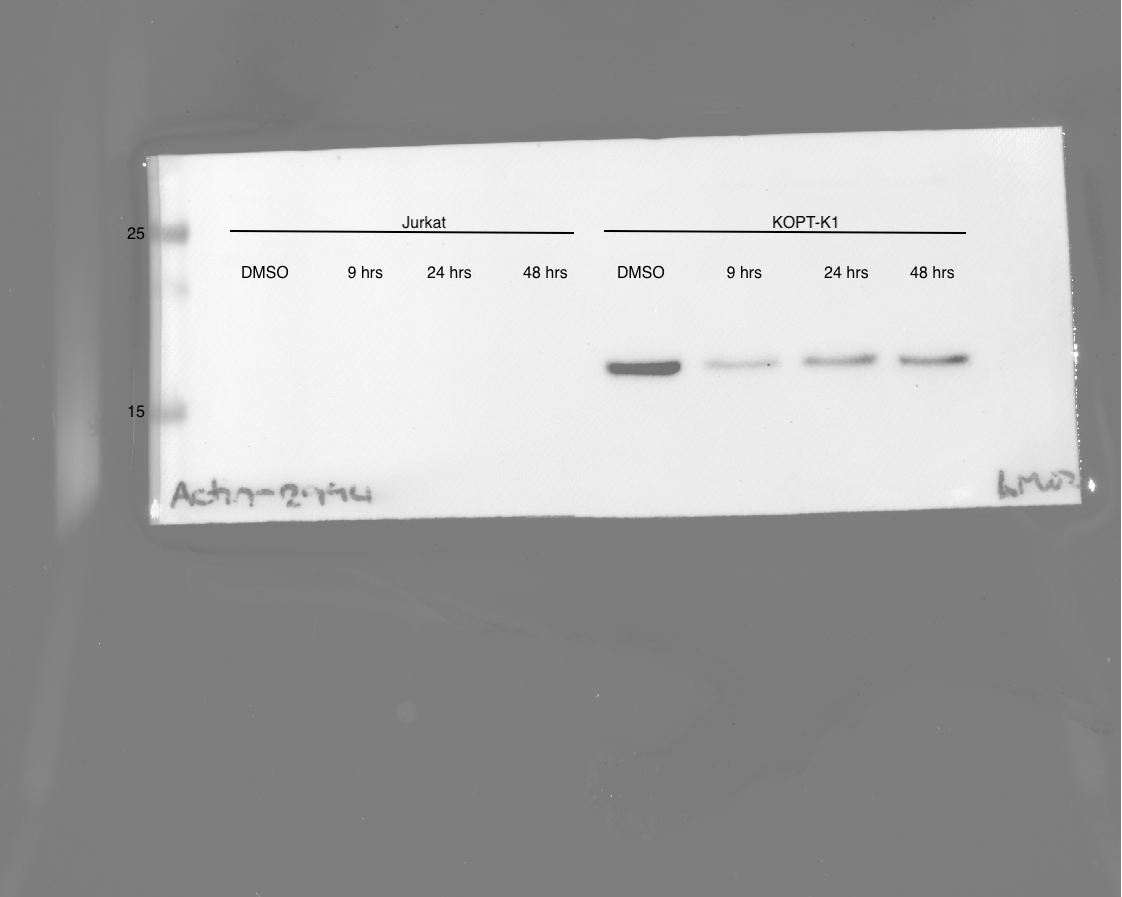

Supplement: Figure 6—source data 1. [file elife-106699-fig6-data1.zip › Figure 6ΓÇösource data 1 PDF files containing original western blots for Figure 6B, indicating the relevant bands and treatments./Raw data/LMO2 Abd-VHL.tif]

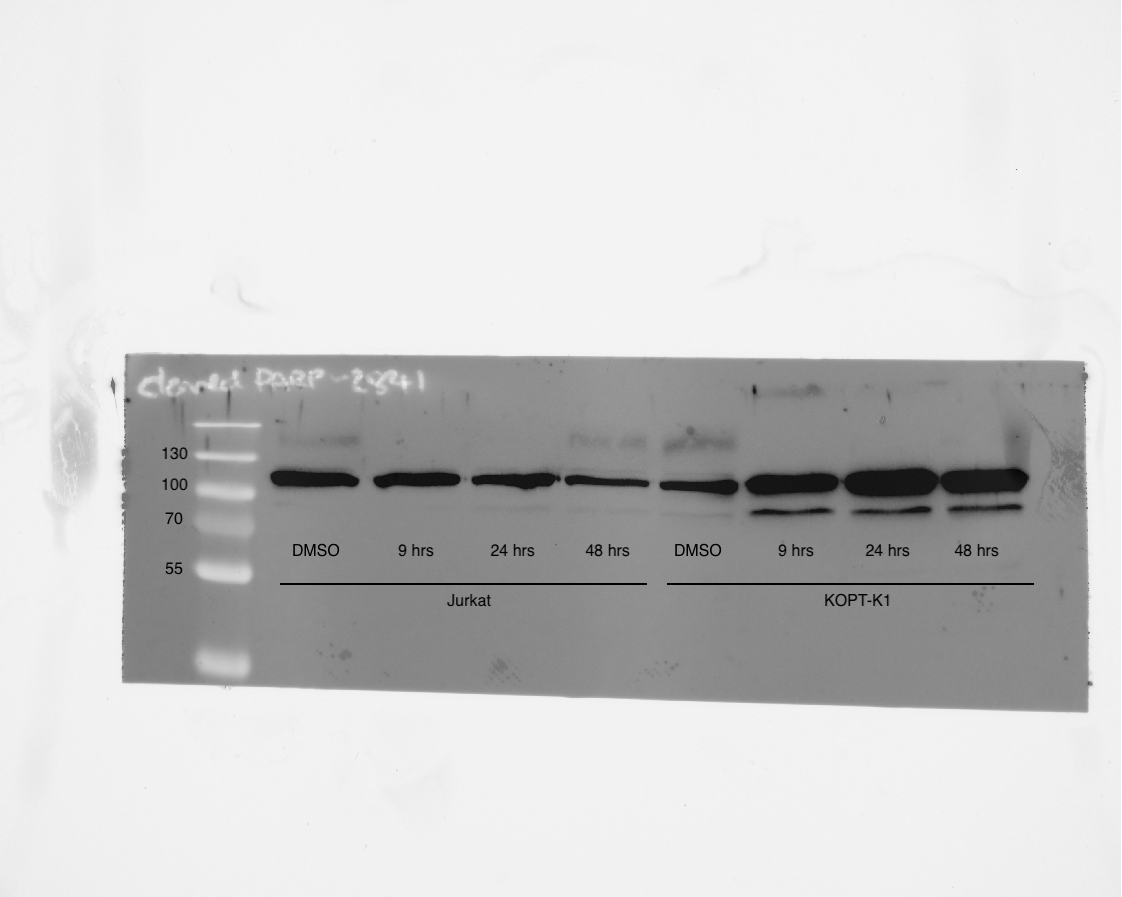

Supplement: Figure 6—source data 1. [file elife-106699-fig6-data1.zip › Figure 6ΓÇösource data 1 PDF files containing original western blots for Figure 6B, indicating the relevant bands and treatments./Raw data/PARP abd-CRBN.tif]

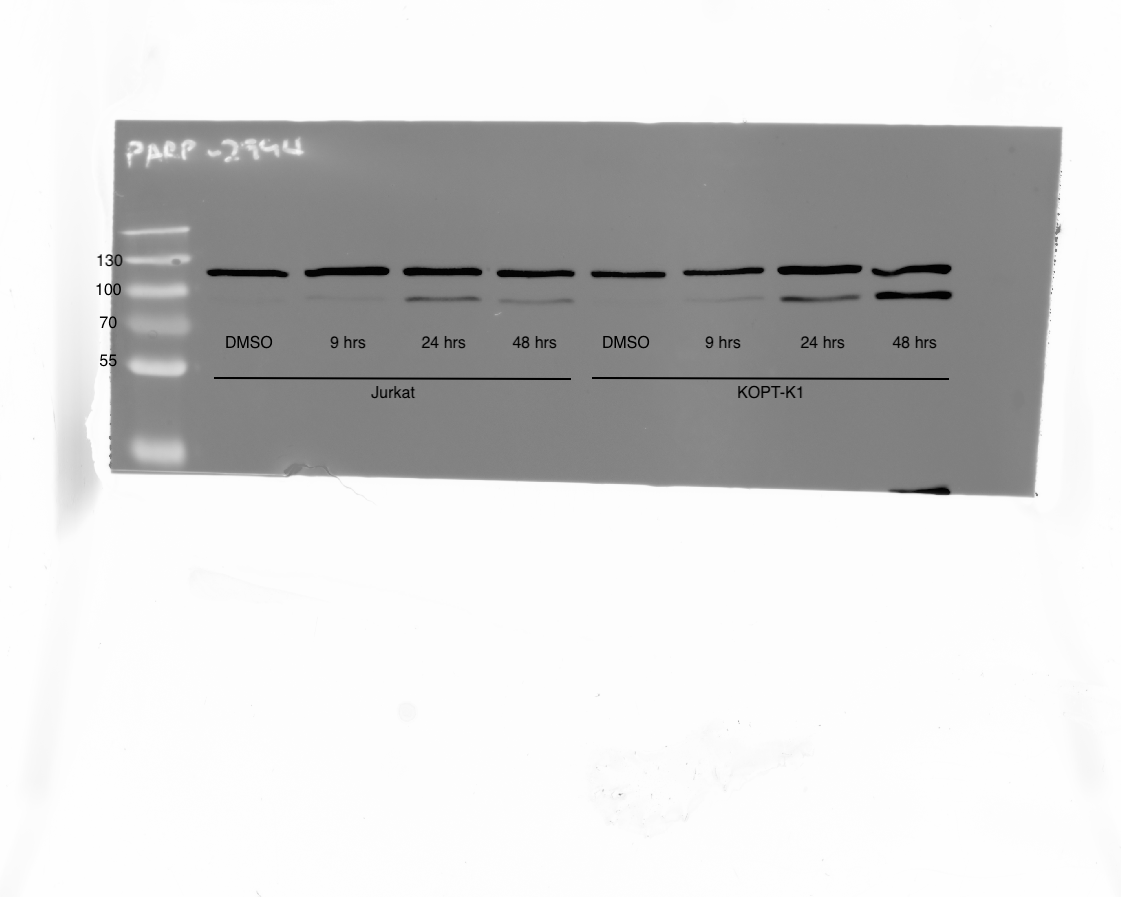

Supplement: Figure 6—source data 1. [file elife-106699-fig6-data1.zip › Figure 6ΓÇösource data 1 PDF files containing original western blots for Figure 6B, indicating the relevant bands and treatments./Raw data/PARP Abd-VHL.tif]

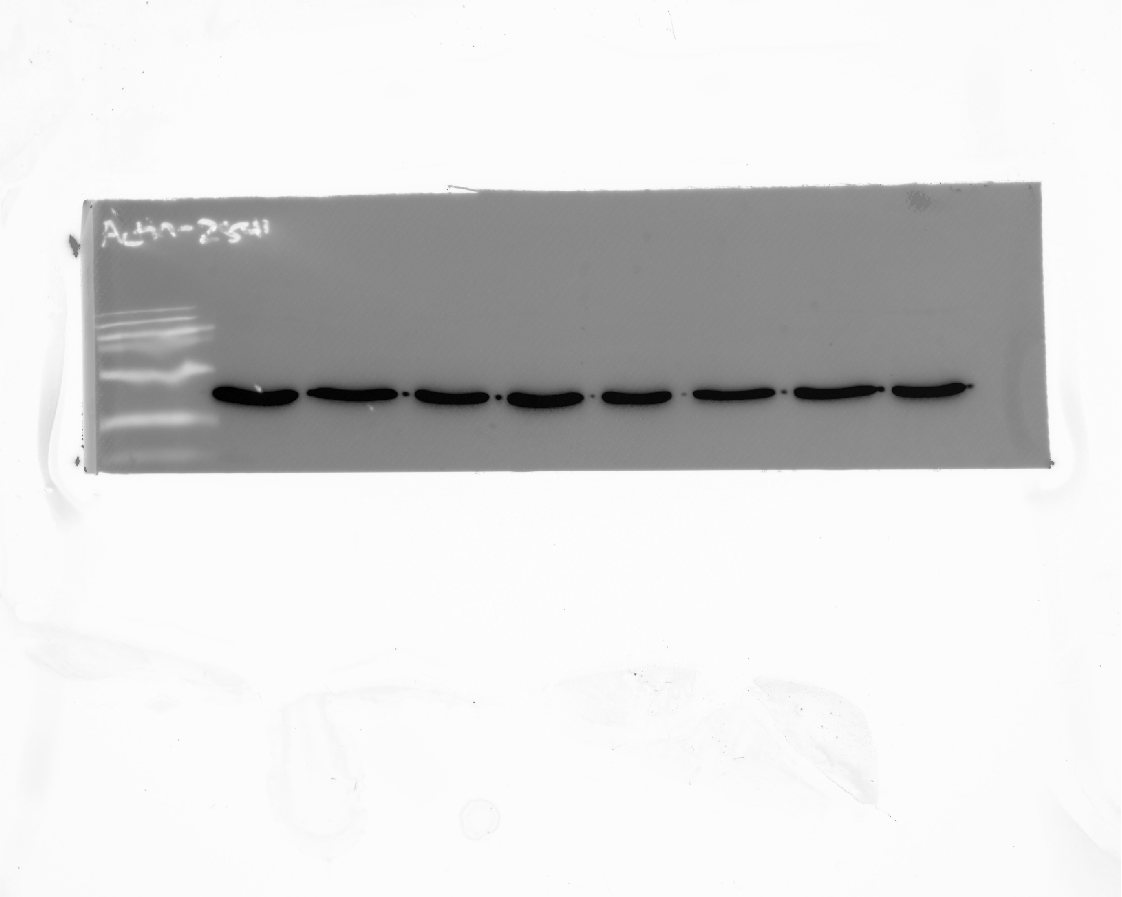

Supplement: Figure 6—source data 2. [file elife-106699-fig6-data2.zip › Figure 6ΓÇösource data 2 Original files for Western blot analysis displayed in Figure 6B./Actin Abd-CRBN.tif]

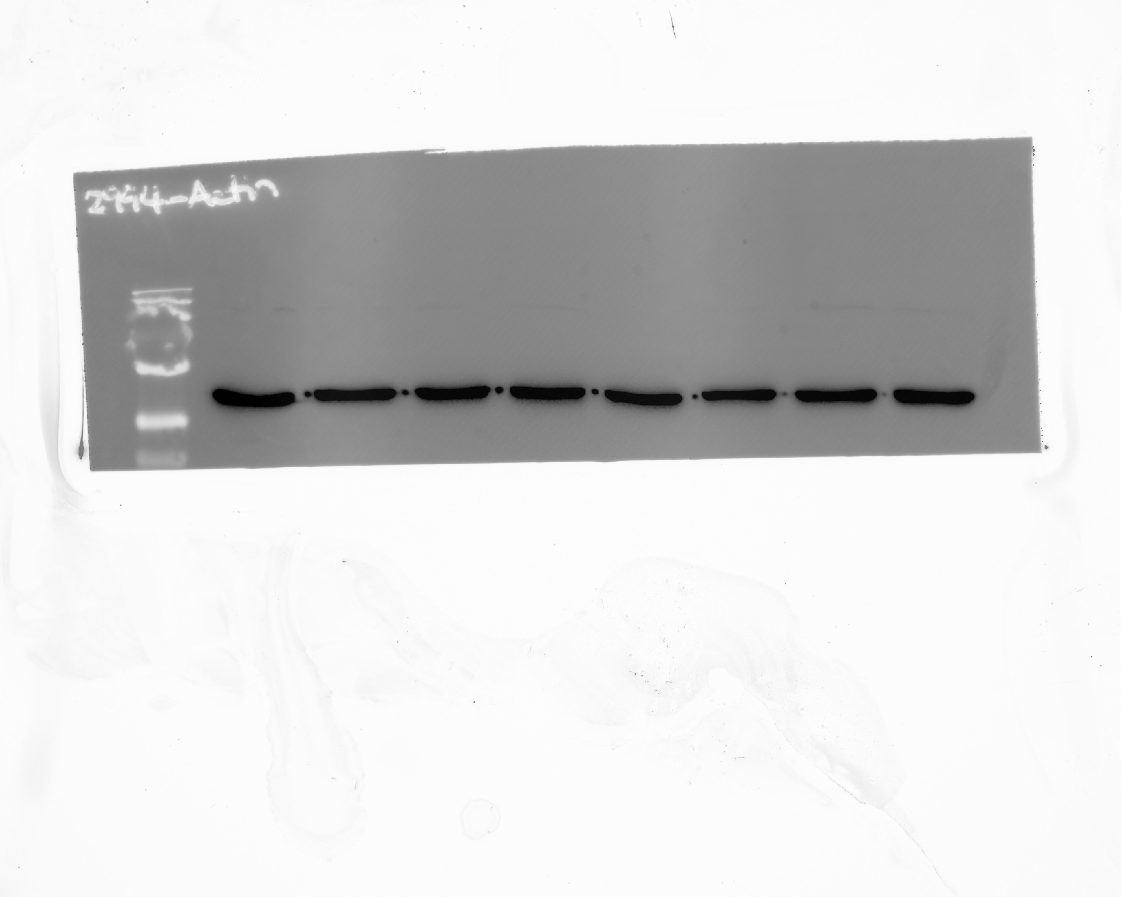

Supplement: Figure 6—source data 2. [file elife-106699-fig6-data2.zip › Figure 6ΓÇösource data 2 Original files for Western blot analysis displayed in Figure 6B./Actin Abd-VHL.tif]

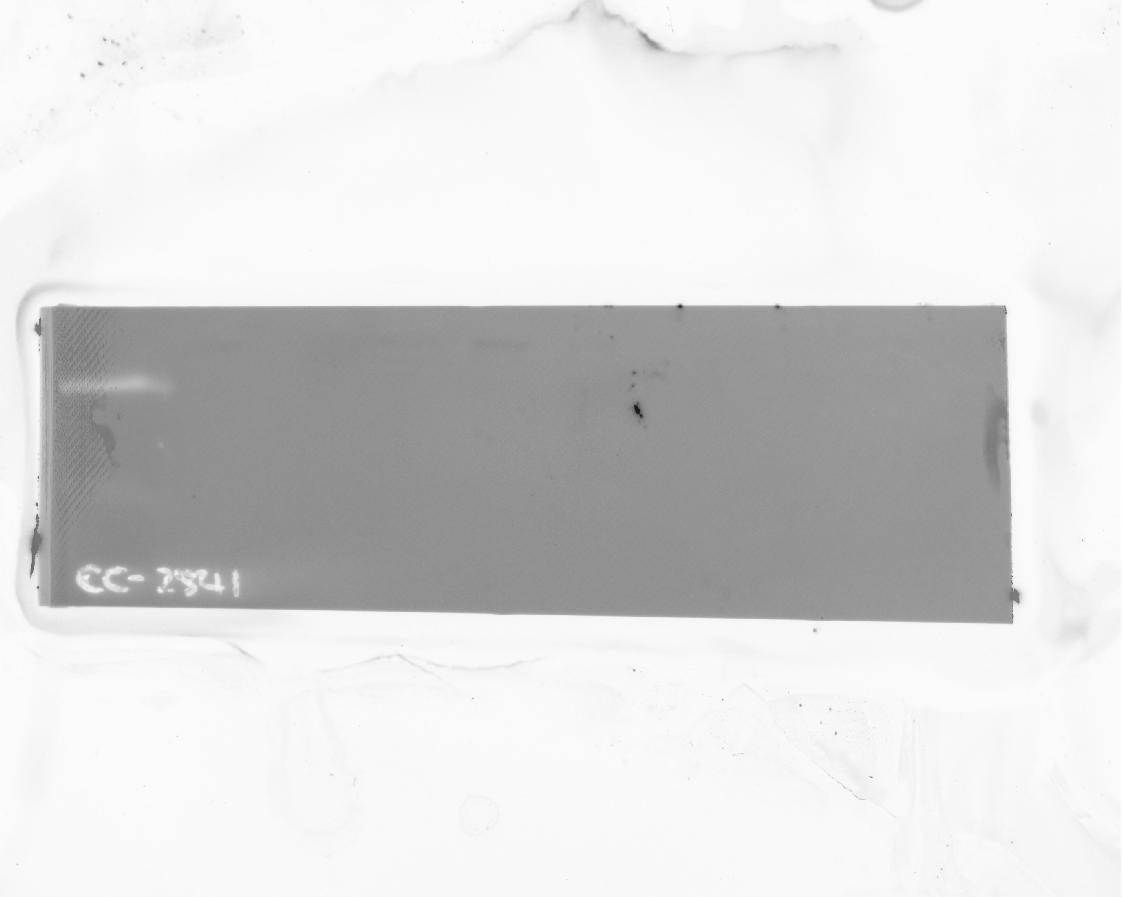

Supplement: Figure 6—source data 2. [file elife-106699-fig6-data2.zip › Figure 6ΓÇösource data 2 Original files for Western blot analysis displayed in Figure 6B./Cleaved CP3 Abd-CRBN.tif]

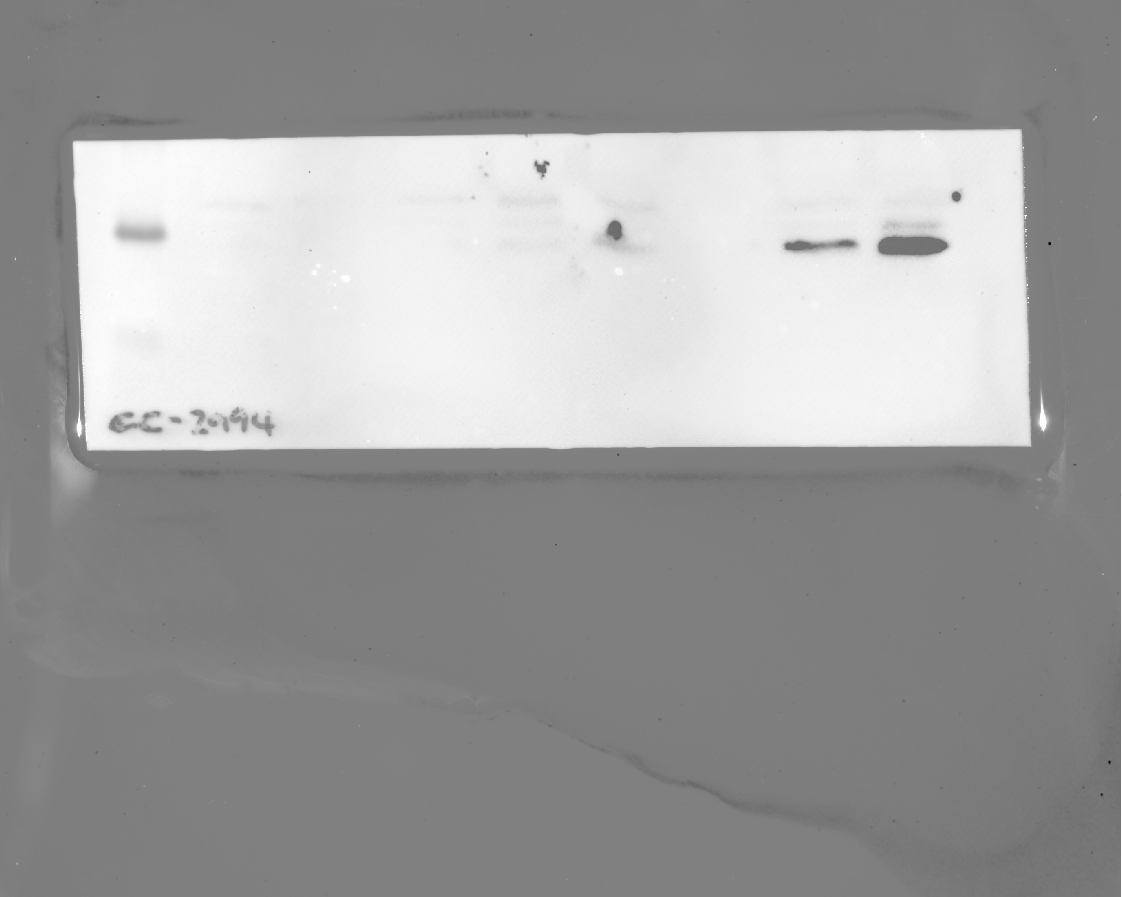

Supplement: Figure 6—source data 2. [file elife-106699-fig6-data2.zip › Figure 6ΓÇösource data 2 Original files for Western blot analysis displayed in Figure 6B./Cleaved CP3 Abd-VHL.tif]

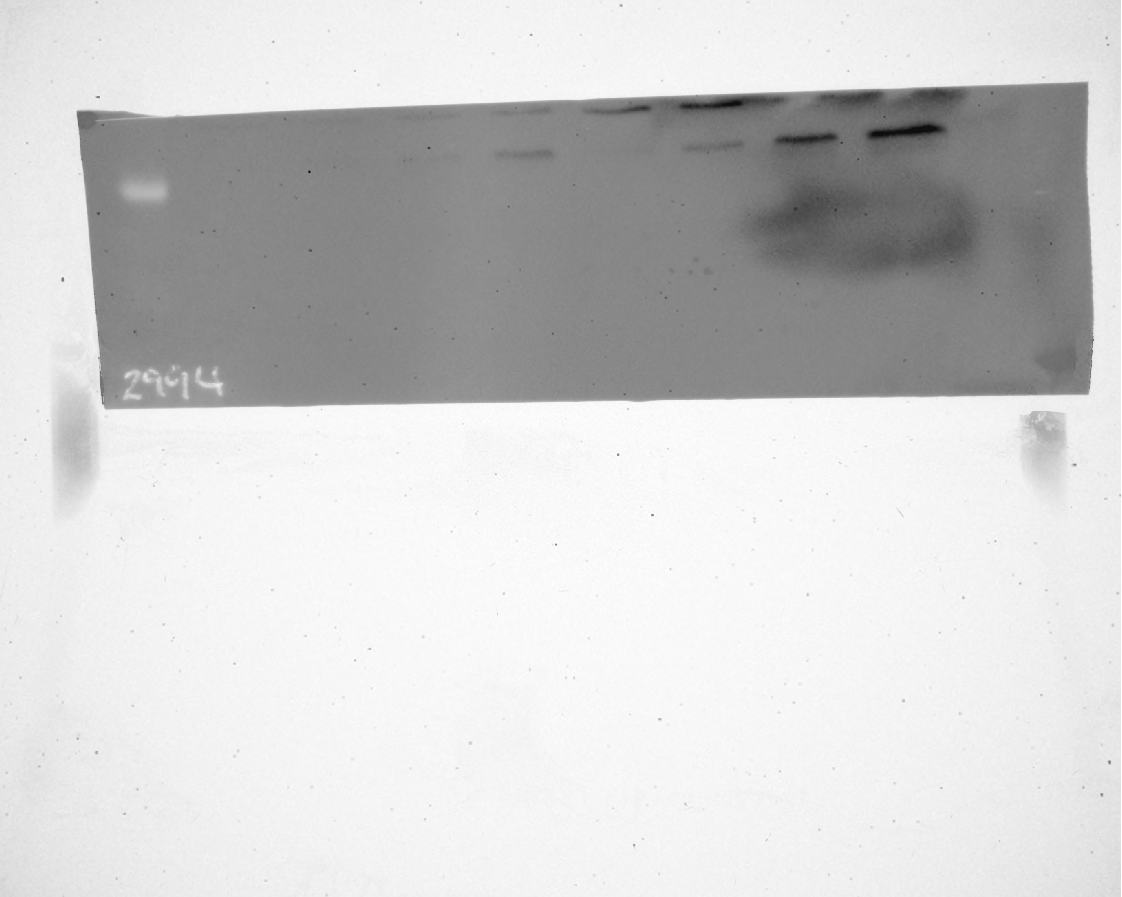

Supplement: Figure 6—source data 2. [file elife-106699-fig6-data2.zip › Figure 6ΓÇösource data 2 Original files for Western blot analysis displayed in Figure 6B./Cleaved CP7 Abd-CRBN.tif]

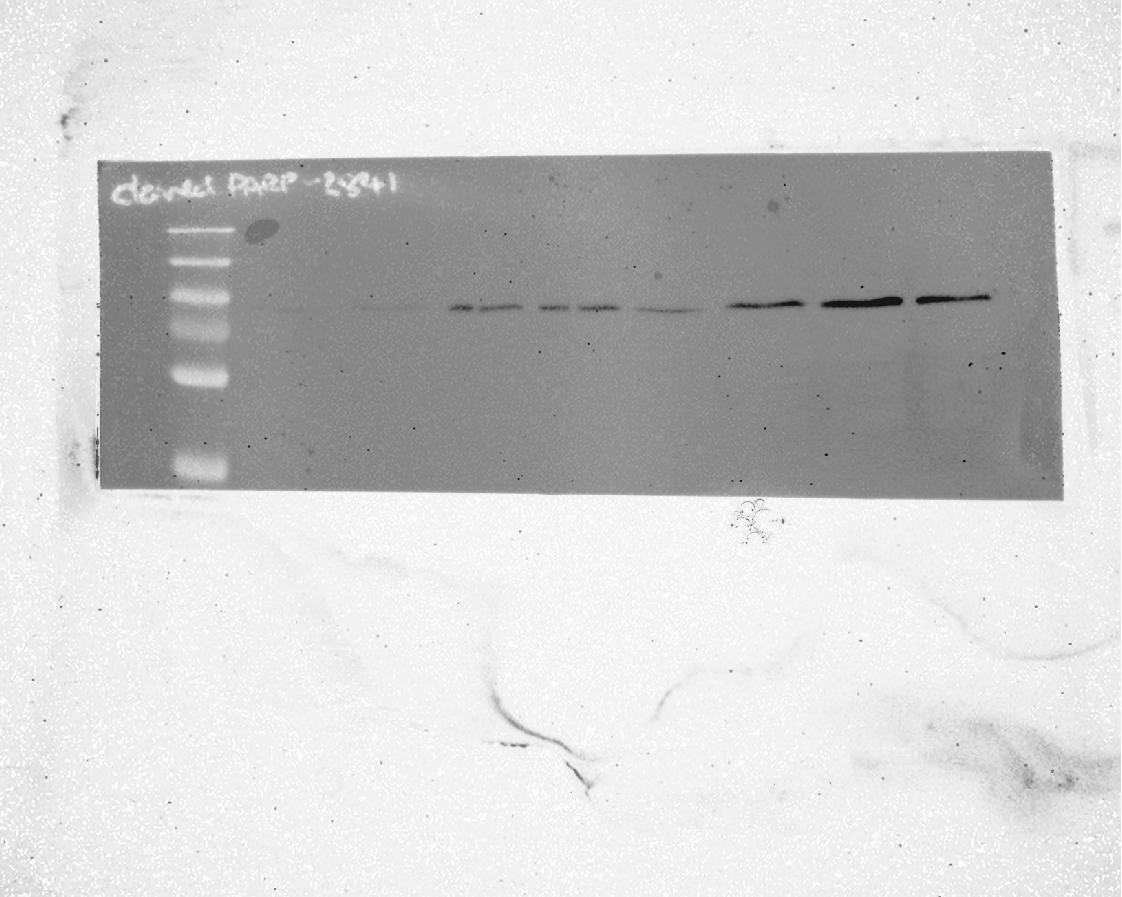

Supplement: Figure 6—source data 2. [file elife-106699-fig6-data2.zip › Figure 6ΓÇösource data 2 Original files for Western blot analysis displayed in Figure 6B./Cleaved PARP Abd-CRBN.tif]

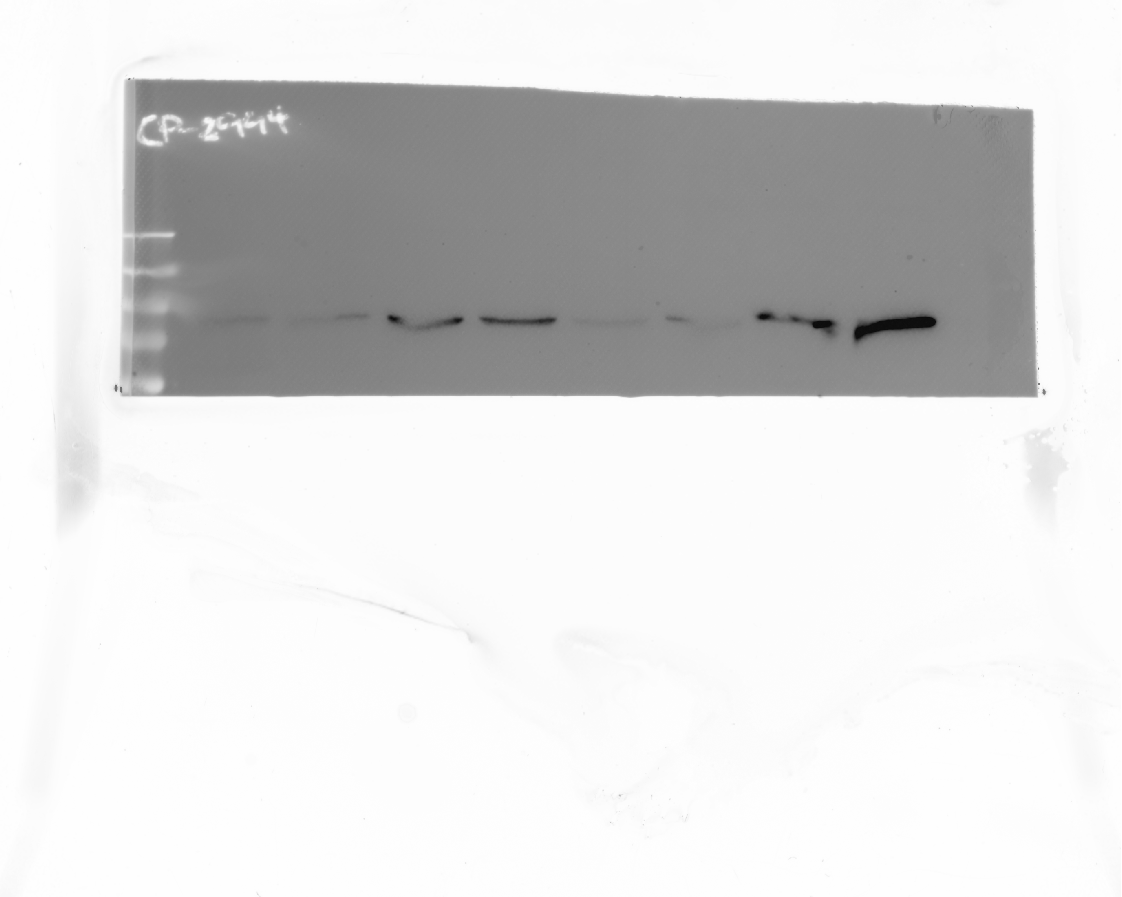

Supplement: Figure 6—source data 2. [file elife-106699-fig6-data2.zip › Figure 6ΓÇösource data 2 Original files for Western blot analysis displayed in Figure 6B./Cleaved PARP Abd-VHL.tif]

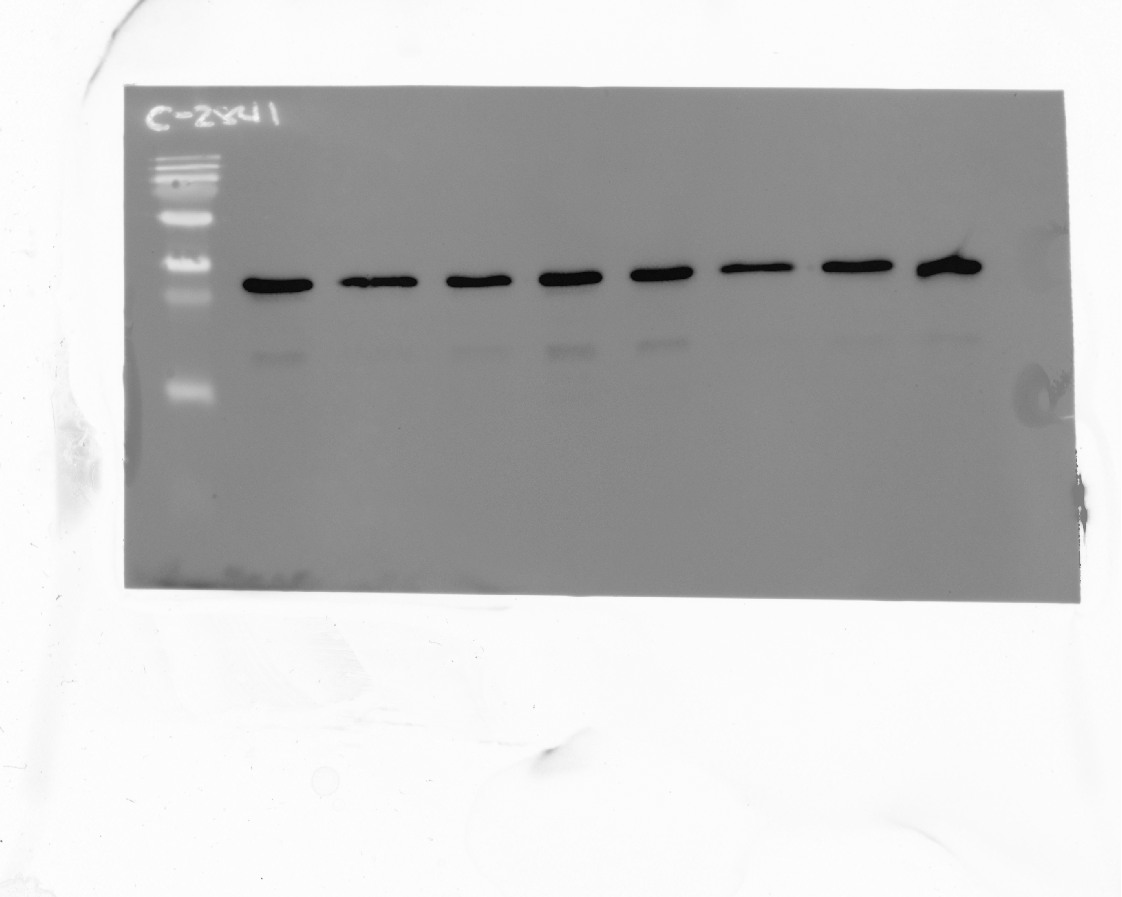

Supplement: Figure 6—source data 2. [file elife-106699-fig6-data2.zip › Figure 6ΓÇösource data 2 Original files for Western blot analysis displayed in Figure 6B./CP3 Abd-CRBN.tif]

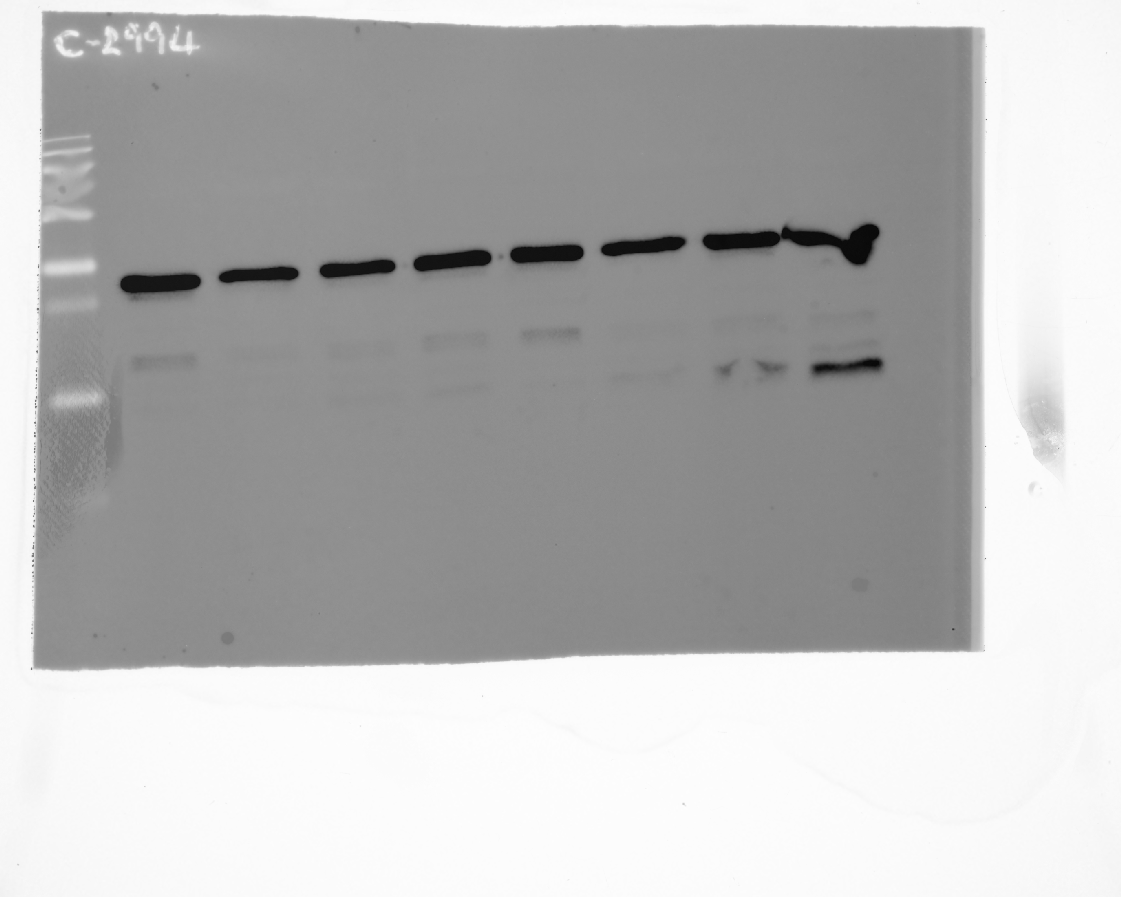

Supplement: Figure 6—source data 2. [file elife-106699-fig6-data2.zip › Figure 6ΓÇösource data 2 Original files for Western blot analysis displayed in Figure 6B./CP3 Abd-VHL.tif]

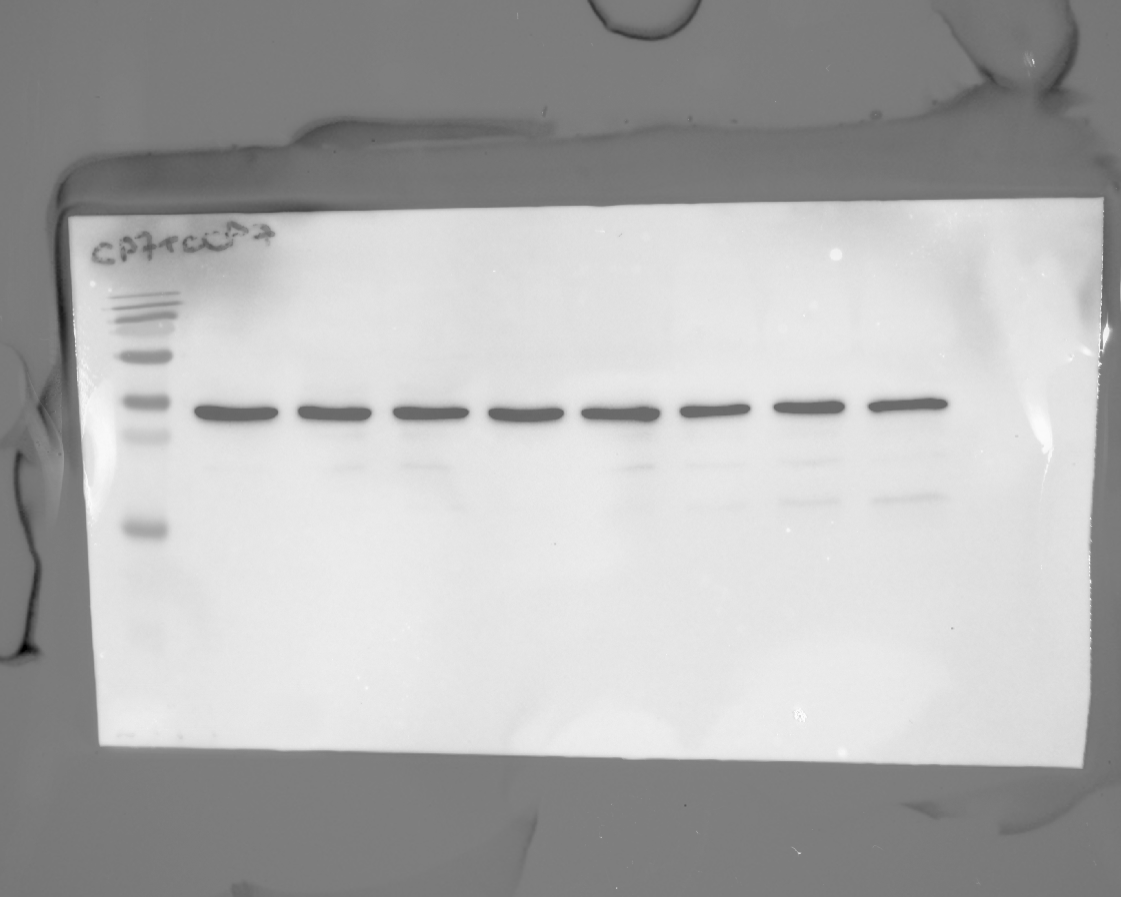

Supplement: Figure 6—source data 2. [file elife-106699-fig6-data2.zip › Figure 6ΓÇösource data 2 Original files for Western blot analysis displayed in Figure 6B./CP7 Abd-CRBN.tif]

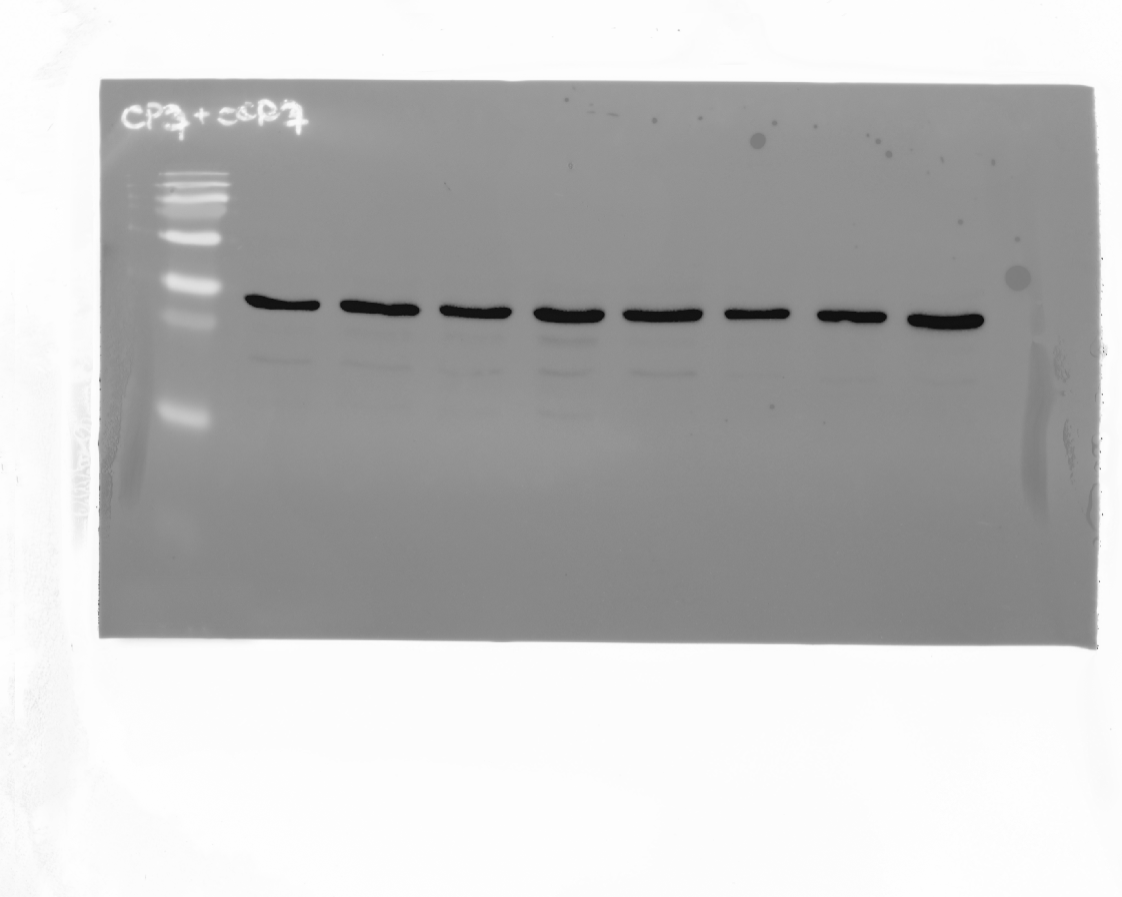

Supplement: Figure 6—source data 2. [file elife-106699-fig6-data2.zip › Figure 6ΓÇösource data 2 Original files for Western blot analysis displayed in Figure 6B./CP7 Abd-VHL.tif]

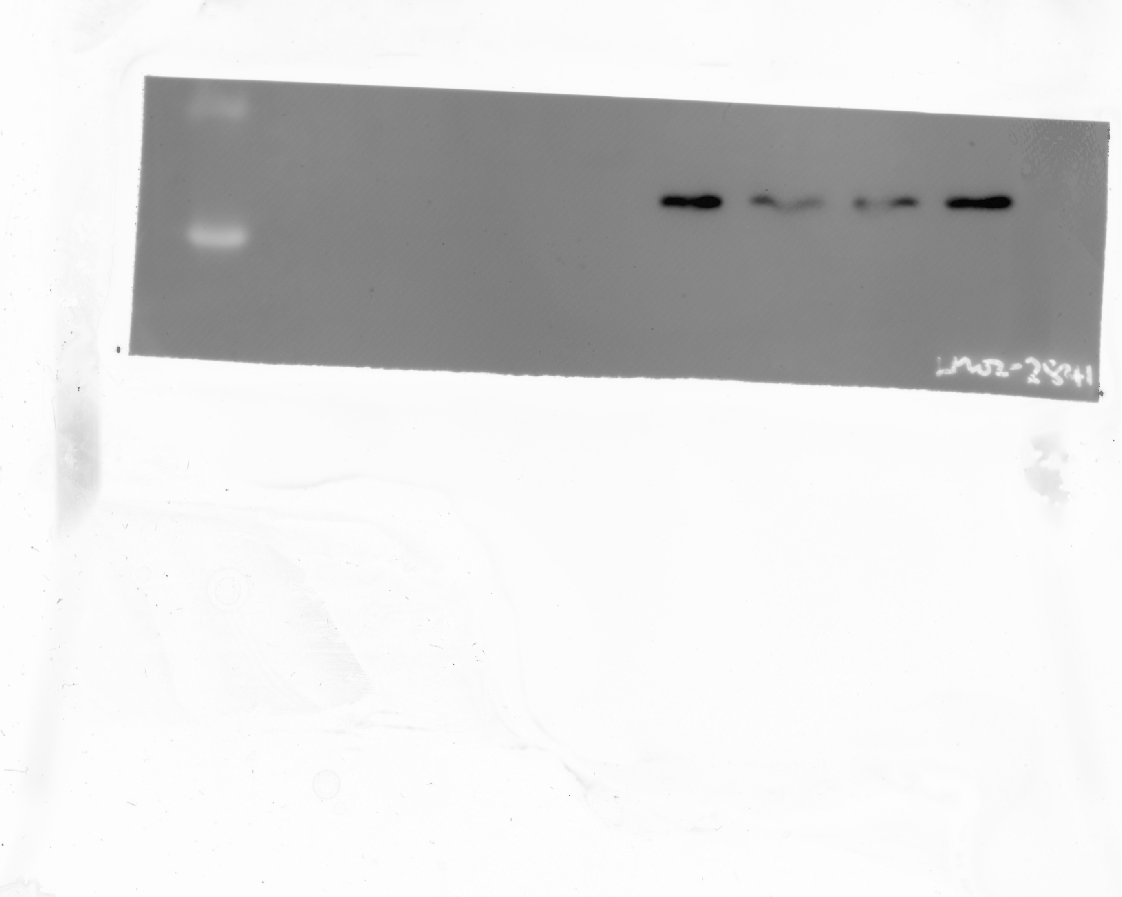

Supplement: Figure 6—source data 2. [file elife-106699-fig6-data2.zip › Figure 6ΓÇösource data 2 Original files for Western blot analysis displayed in Figure 6B./LMO2 Abd-CRBN.tif]

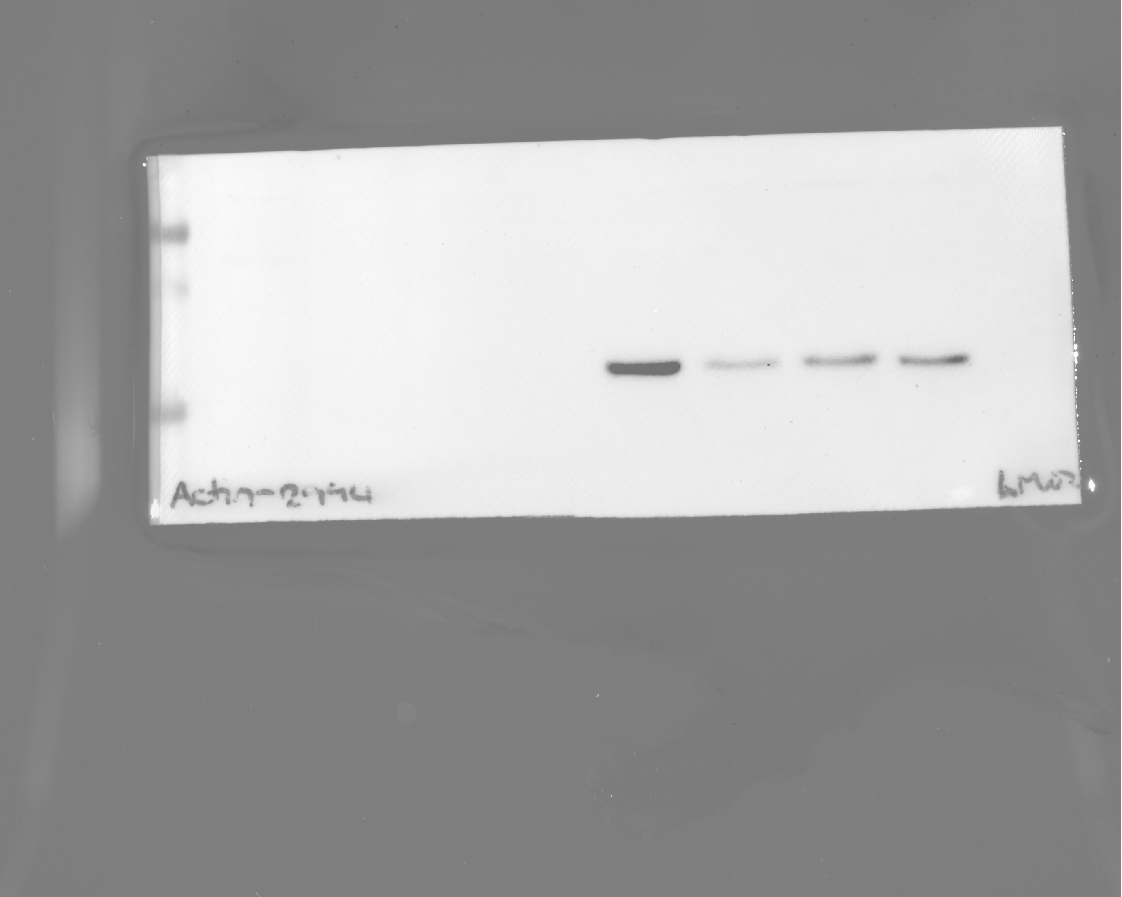

Supplement: Figure 6—source data 2. [file elife-106699-fig6-data2.zip › Figure 6ΓÇösource data 2 Original files for Western blot analysis displayed in Figure 6B./LMO2 Abd-VHL.tif]

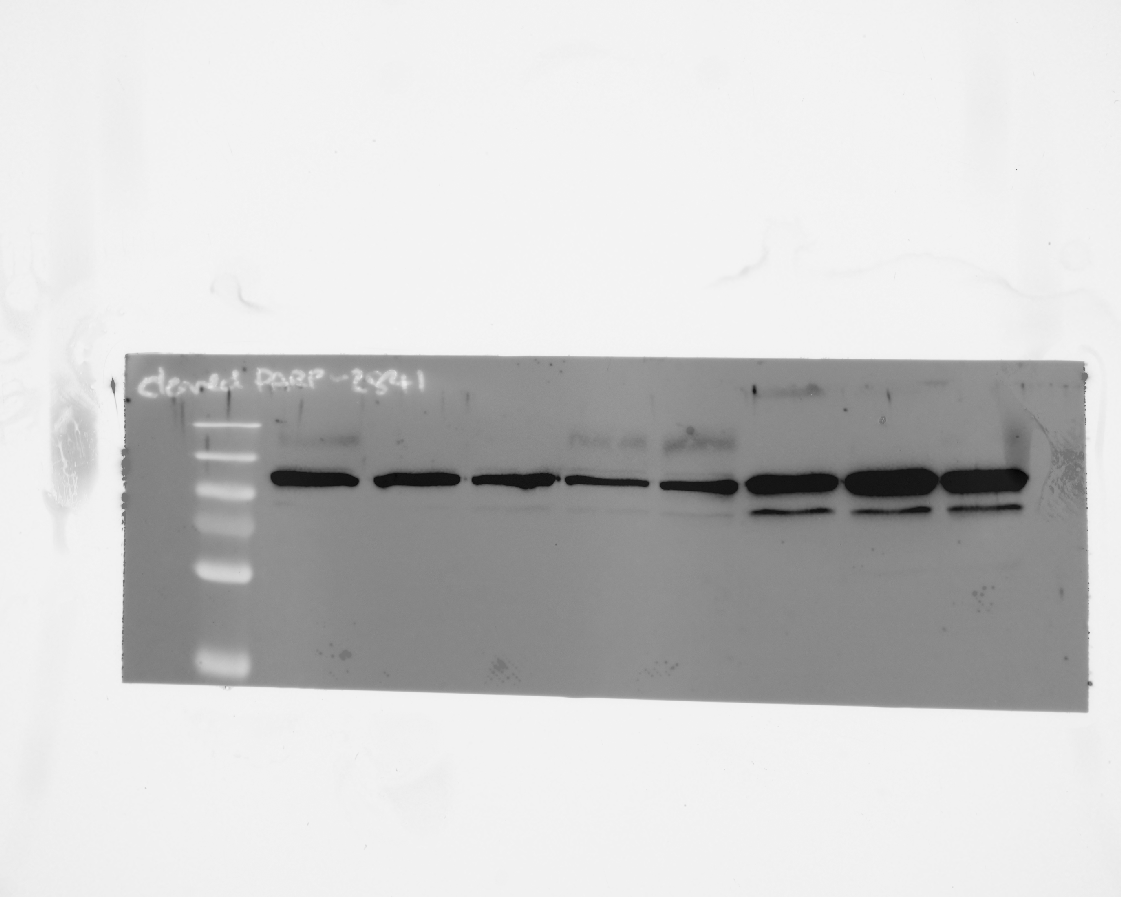

Supplement: Figure 6—source data 2. [file elife-106699-fig6-data2.zip › Figure 6ΓÇösource data 2 Original files for Western blot analysis displayed in Figure 6B./PARP Abd-CRBN.tif]

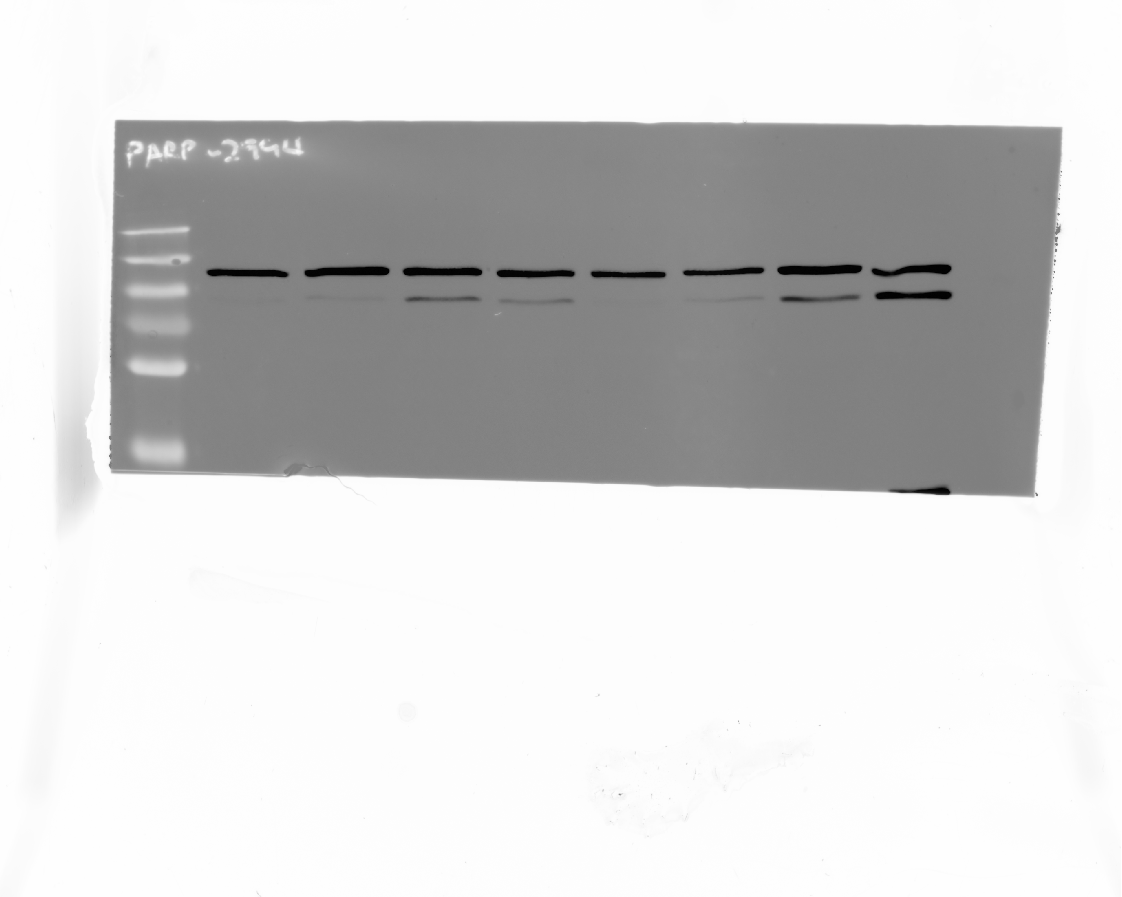

Supplement: Figure 6—source data 2. [file elife-106699-fig6-data2.zip › Figure 6ΓÇösource data 2 Original files for Western blot analysis displayed in Figure 6B./PARP Abd-VHL.tif]

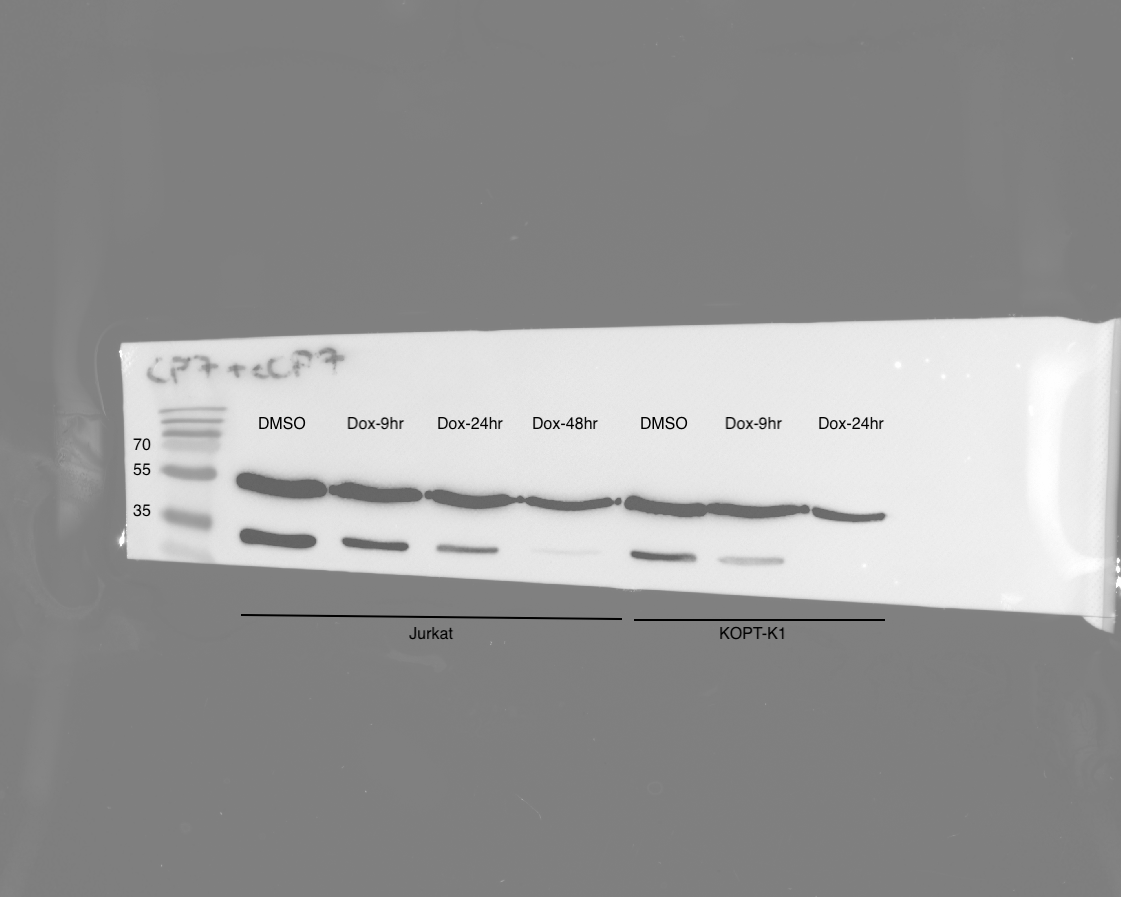

Supplement: Figure 6—figure supplement 1—source data 1. [file elife-106699-fig6-figsupp1-data1.zip › Figure 6ΓÇöfigure supplement 1-source data 1 Western blot data with label shows the expression of caspase 7 and cleaved caspase 7 in Jurkat and KOPT-K1 after the treatment of doxorubicin./Raw data/Tubulin(Composite).tif]

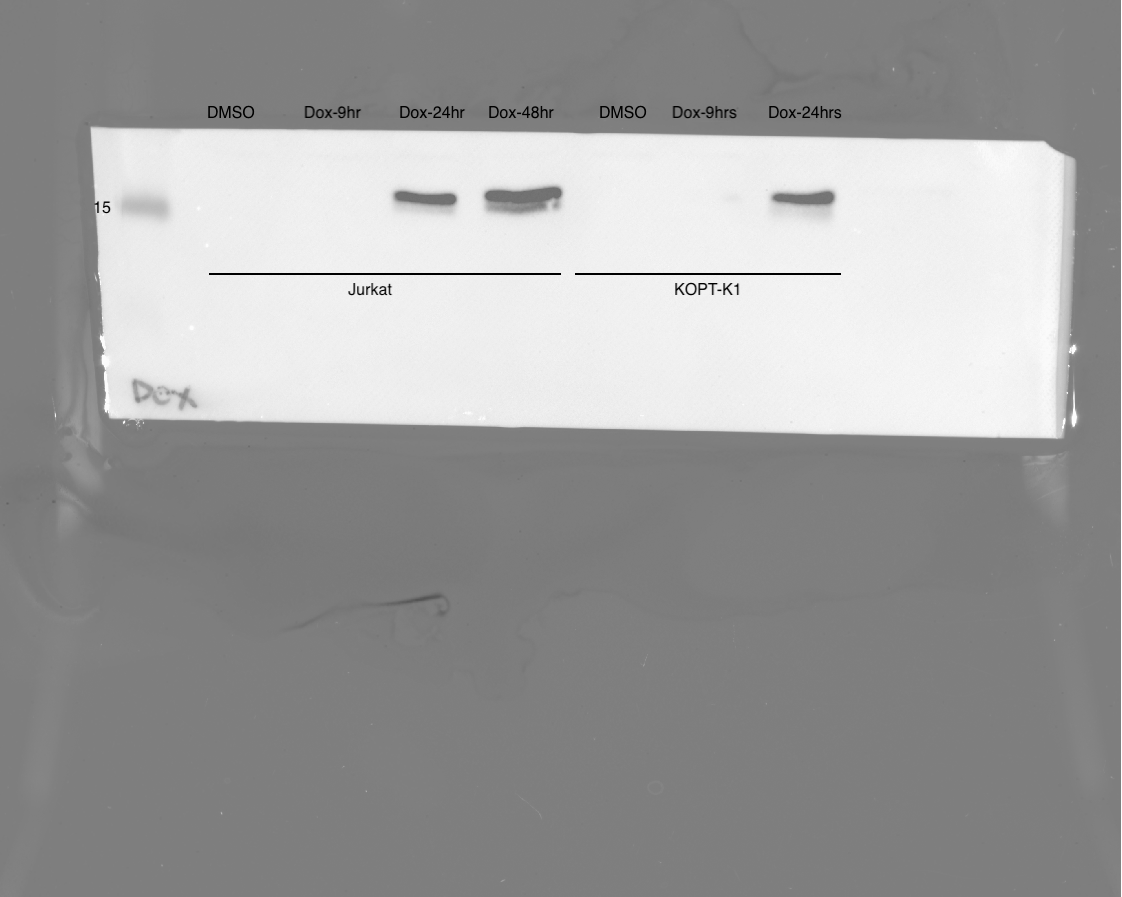

Supplement: Figure 6—figure supplement 1—source data 1. [file elife-106699-fig6-figsupp1-data1.zip › Figure 6ΓÇöfigure supplement 1-source data 1 Western blot data with label shows the expression of caspase 7 and cleaved caspase 7 in Jurkat and KOPT-K1 after the treatment of doxorubicin./Raw data/cleaved CP7(Composite).tif]

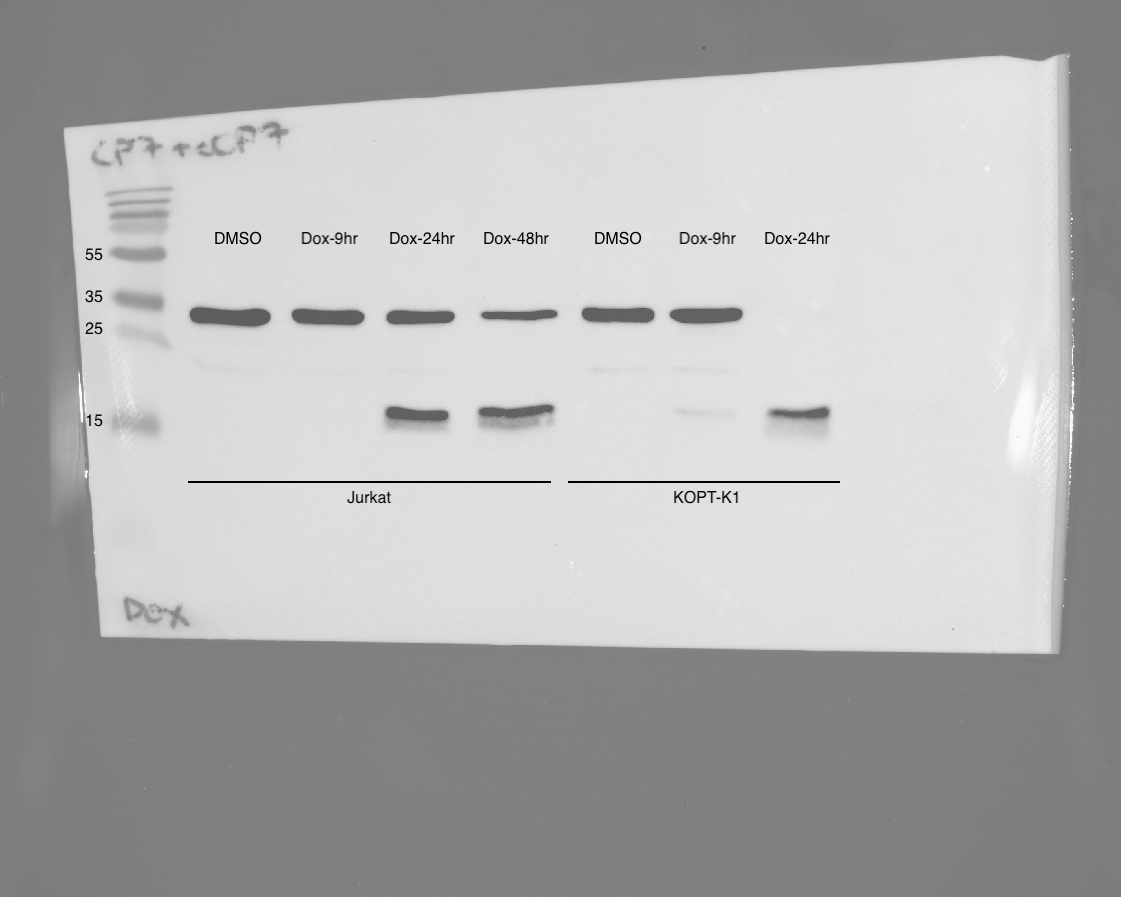

Supplement: Figure 6—figure supplement 1—source data 1. [file elife-106699-fig6-figsupp1-data1.zip › Figure 6ΓÇöfigure supplement 1-source data 1 Western blot data with label shows the expression of caspase 7 and cleaved caspase 7 in Jurkat and KOPT-K1 after the treatment of doxorubicin./Raw data/CP7(Composite).tif]

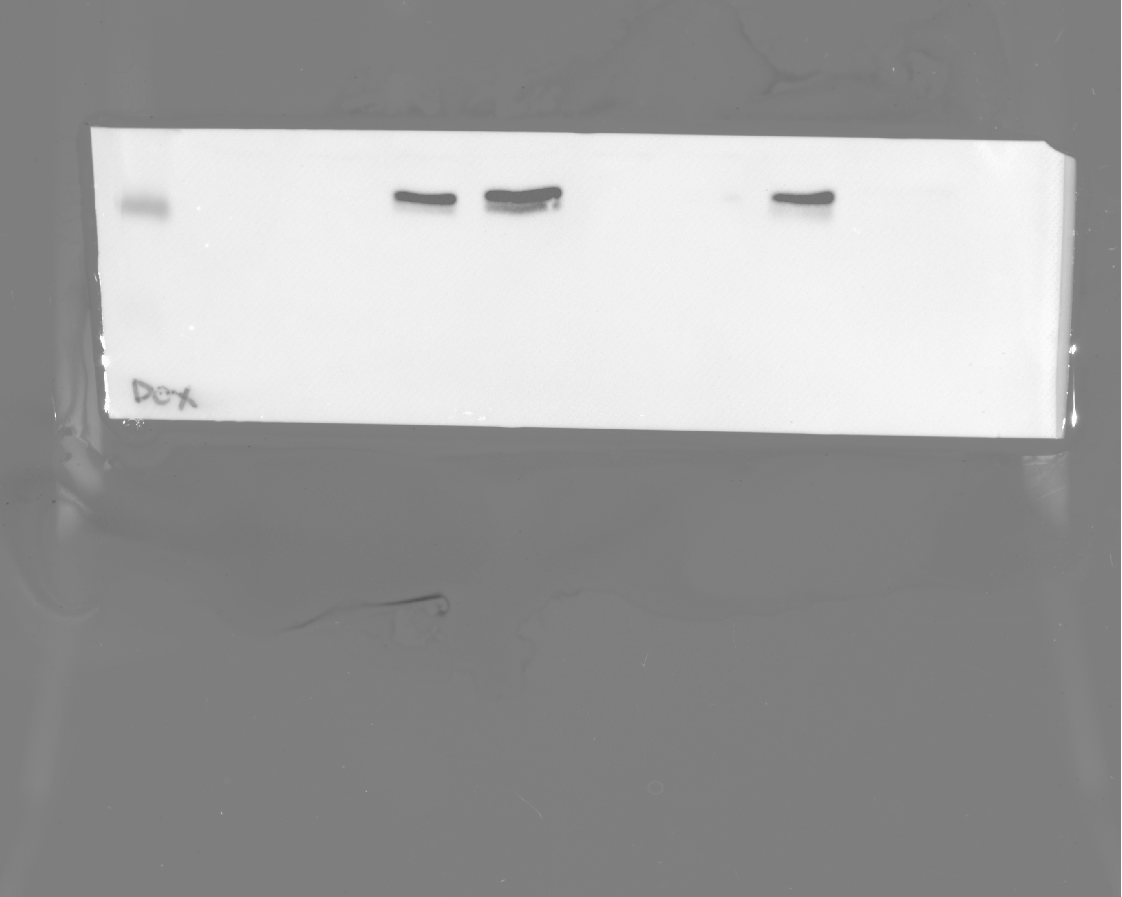

Supplement: Figure 6—figure supplement 1—source data 2. [file elife-106699-fig6-figsupp1-data2.zip › Figure 6ΓÇöfigure supplement 1-source data 2 Western blot raw data shows the expression of caspase 7 and cleaved caspase 7 in Jurkat and KOPT-K1 after the treatment of doxorubicin./cleaved CP7(Composite).tif]

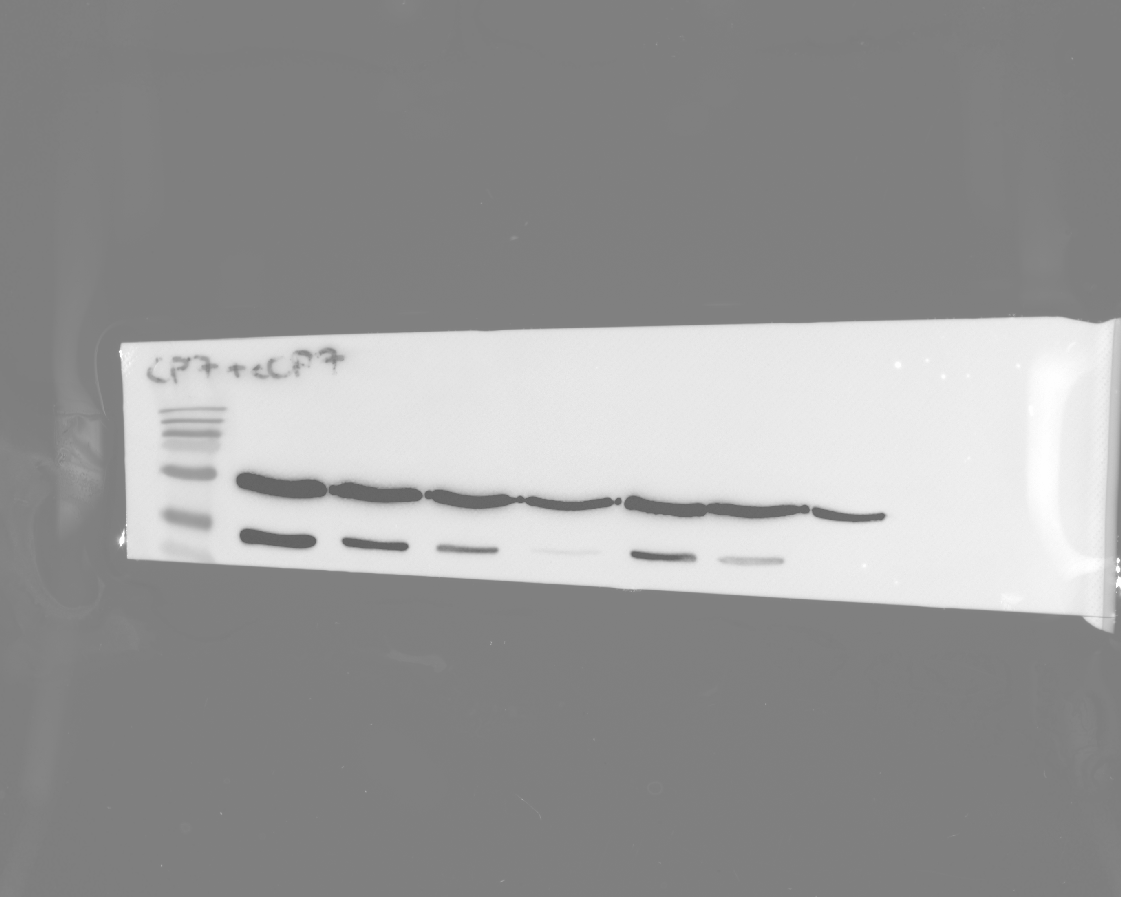

Supplement: Figure 6—figure supplement 1—source data 2. [file elife-106699-fig6-figsupp1-data2.zip › Figure 6ΓÇöfigure supplement 1-source data 2 Western blot raw data shows the expression of caspase 7 and cleaved caspase 7 in Jurkat and KOPT-K1 after the treatment of doxorubicin./Tubulin (Composite).tif]

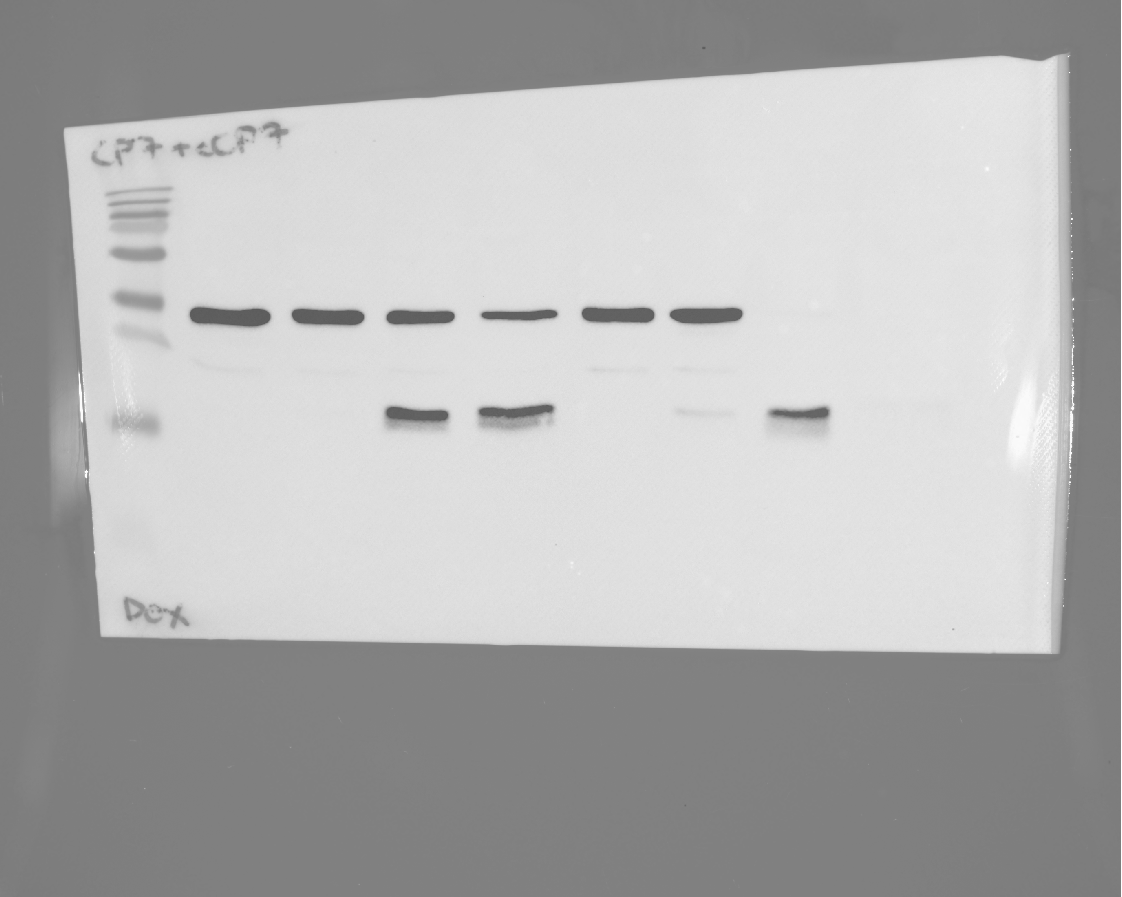

Supplement: Figure 6—figure supplement 1—source data 2. [file elife-106699-fig6-figsupp1-data2.zip › Figure 6ΓÇöfigure supplement 1-source data 2 Western blot raw data shows the expression of caspase 7 and cleaved caspase 7 in Jurkat and KOPT-K1 after the treatment of doxorubicin./CP7(Composite).tif]
