# Supplementary material for: Degradation of LMO2 in T cell leukaemia results in collateral breakdown of transcription complex partners and causes LMO2-dependent apoptosis
Source: eLife. 2025 Dec 12;14:RP106699. doi: 10.7554/eLife.106699 (PMC12700530; doi:10.7554/eLife.106699)
Supplement: Figure 4—figure supplement 2—source data 1. [file elife-106699-fig4-figsupp2-data1.zip › Figure 4ΓÇöfigure supplement 2-source data 1 Agarose gel data with label shows PCR products from RT-PCR analysis of the human T-ALL cells./Figure 4-figure supplement 2-cource data 1.pdf]

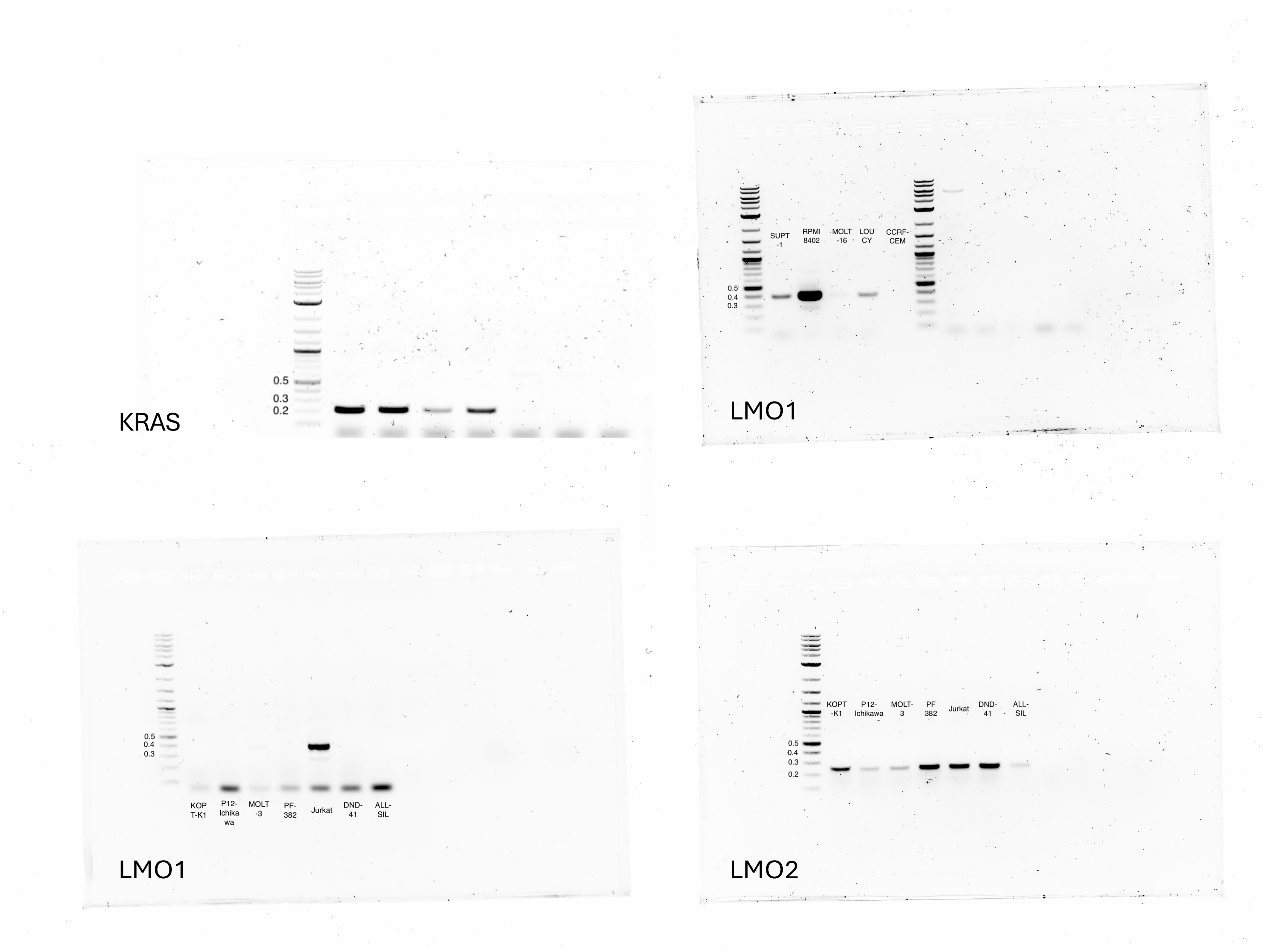

Figure 4-figure supplement 2, Source Data 1. Original membranes corresponding to Figure 4-figure supplement 2, panel A-C.
