# Supplementary material for: Degradation of LMO2 in T cell leukaemia results in collateral breakdown of transcription complex partners and causes LMO2-dependent apoptosis
Source: eLife. 2025 Dec 12;14:RP106699. doi: 10.7554/eLife.106699 (PMC12700530; doi:10.7554/eLife.106699)
Supplement: Figure 5—source data 3. — Proteomic data showed the significant downregulated proteins in KOPT-K1 cells when treated with AbdVHL compared to control treatment (DMSO) and their functions related to cell division. Proteins shown based on the fold change (log2FC) and p-value. [file elife-106699-fig5-data3.pdf]

Proteins	log_2FC	Functions
AURKA	-0.376	Kinase that contributes to the regulation of cell cycle progression
KIF2C	-0.384	Important for anaphase chromosome segregation
HAUS1	-0.394	Maintenance of completion of cytokinesis
CDC25B	-0.428	Activates the cyclin dependent kinase CDC2
CCNA2	-0.429	Cyclin which controls both the G1/S and the G2/M transition
SGO2	-0.466	Part of mitotic cohesion complex
UBE2S	-0.482	E2 enzyme ubiquitinates APC/C ubiquitin ligase
BUB1	-0.488	Member of the mitotic checkpoint complex and activating the spindle checkpoint.
USP37	-0.575	De-ubiquitinate enzyme promotes cell cycle
MASTL	-0.602	MASTL kinase is a master regulator of mitosis
CDCA2	-0.642	Regulates chromosome structure during mitosis
UBE2C	-0.652	E2 enzyme ubiquitinates APC/C
MIS18A	-0.704	Required normal chromosome segregation during mitosis
FAM83D	-0.803	Regulates cell proliferation, growth
CKS2	-1.042	Binds to CDKs for their function
TXNIP	-1.667	Master regulator of cellular oxidation, regulating the expression and function of Thioredoxin

Figure 5, supplement 1: Proteins reduced by treatment of KOPT-K1 cells with Abd-VHL
Proteomic data showed the significant downregulated proteins in KOPT-K1 cells when treated with Abd-VHL compared to control treatment (DMSO) and their functions related to cell division. Proteins shown based on the fold change (log_2FC) and p-value.
