# Supplementary material for: Degradation of LMO2 in T cell leukaemia results in collateral breakdown of transcription complex partners and causes LMO2-dependent apoptosis
Source: eLife. 2025 Dec 12;14:RP106699. doi: 10.7554/eLife.106699 (PMC12700530; doi:10.7554/eLife.106699)
Supplement: Figure 6—figure supplement 1—source data 1. [file elife-106699-fig6-figsupp1-data1.zip › Figure 6ΓÇöfigure supplement 1-source data 1 Western blot data with label shows the expression of caspase 7 and cleaved caspase 7 in Jurkat and KOPT-K1 after the treatment of doxorubicin./Figure 6-figure supplement 1-source data 1.pdf]

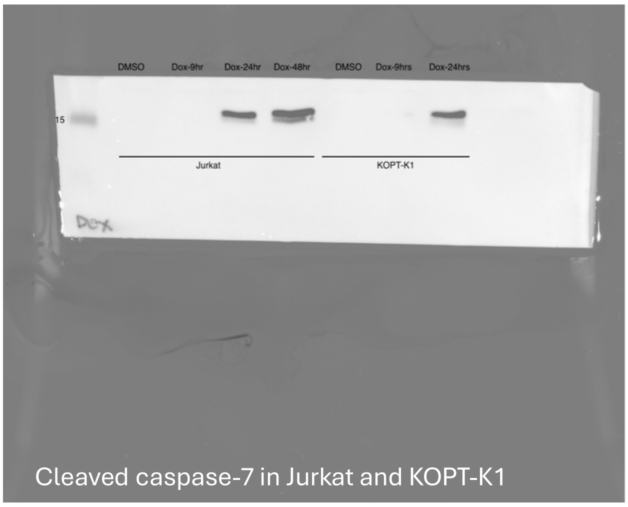

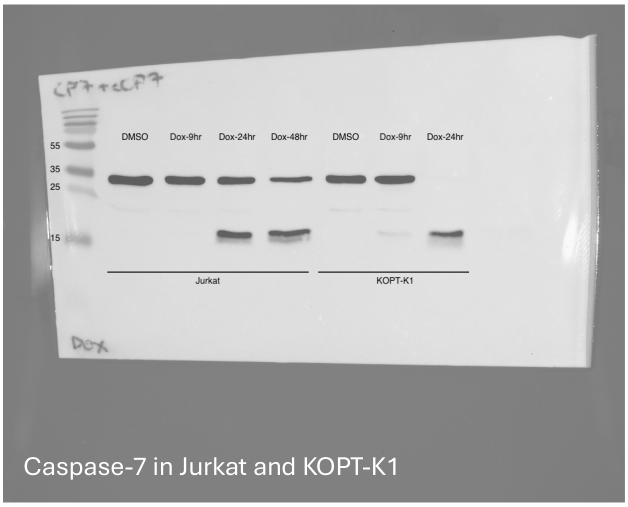

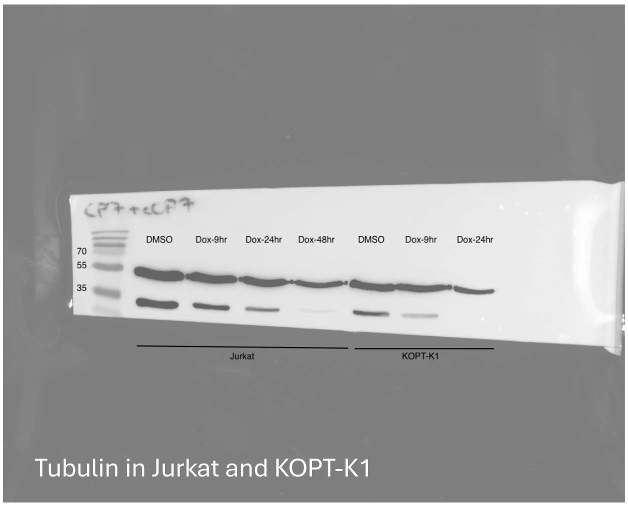

Figure 6-figure supplement 1, Source Data 1. Original membranes corresponding to Figure 6-figure supplement 1, panel A.
